# Highly accurate whole-genome imputation of SARS-CoV-2 from partial or low-quality sequences

**DOI:** 10.1093/gigascience/giab078

**Published:** 2021-12-02

**Authors:** Francisco M Ortuño, Carlos Loucera, Carlos S Casimiro-Soriguer, Jose A Lepe, Pedro Camacho Martinez, Laura Merino Diaz, Adolfo de Salazar, Natalia Chueca, Federico García, Javier Perez-Florido, Joaquin Dopazo

**Affiliations:** Clinical Bioinformatics Area, Fundación Progreso y Salud (FPS), CDCA, Hospital Virgen del Rocio, 41013 Sevilla, Spain; Computational Systems Medicine, Institute of Biomedicine of Seville (IBIS), Hospital Virgen del Rocio, 41013 Sevilla, Spain; Clinical Bioinformatics Area, Fundación Progreso y Salud (FPS), CDCA, Hospital Virgen del Rocio, 41013 Sevilla, Spain; Computational Systems Medicine, Institute of Biomedicine of Seville (IBIS), Hospital Virgen del Rocio, 41013 Sevilla, Spain; Clinical Bioinformatics Area, Fundación Progreso y Salud (FPS), CDCA, Hospital Virgen del Rocio, 41013 Sevilla, Spain; Computational Systems Medicine, Institute of Biomedicine of Seville (IBIS), Hospital Virgen del Rocio, 41013 Sevilla, Spain; Unidad Clínica Enfermedades Infecciosas, Microbiología y Medicina Preventiva, Hospital Universitario Virgen del Rocío, 41013 Sevilla, Spain; Unidad Clínica Enfermedades Infecciosas, Microbiología y Medicina Preventiva, Hospital Universitario Virgen del Rocío, 41013 Sevilla, Spain; Unidad Clínica Enfermedades Infecciosas, Microbiología y Medicina Preventiva, Hospital Universitario Virgen del Rocío, 41013 Sevilla, Spain; Servicio de Microbiología, Hospital Universitario San Cecilio, 18016 Granada, Spain; Servicio de Microbiología, Hospital Universitario San Cecilio, 18016 Granada, Spain; Servicio de Microbiología, Hospital Universitario San Cecilio, 18016 Granada, Spain; Clinical Bioinformatics Area, Fundación Progreso y Salud (FPS), CDCA, Hospital Virgen del Rocio, 41013 Sevilla, Spain; Computational Systems Medicine, Institute of Biomedicine of Seville (IBIS), Hospital Virgen del Rocio, 41013 Sevilla, Spain; Clinical Bioinformatics Area, Fundación Progreso y Salud (FPS), CDCA, Hospital Virgen del Rocio, 41013 Sevilla, Spain; Computational Systems Medicine, Institute of Biomedicine of Seville (IBIS), Hospital Virgen del Rocio, 41013 Sevilla, Spain; FPS/ELIXIR-es, Hospital Virgen del Rocío, Sevilla 42013, Spain; CIBER de Enfermedades Infecciosas (CIBERINFEC), Hospital Universitario San Cecilio, 18016 Granada, Spain

## Abstract

**Background:**

The current SARS-CoV-2 pandemic has emphasized the utility of viral whole-genome sequencing in the surveillance and control of the pathogen. An unprecedented ongoing global initiative is producing hundreds of thousands of sequences worldwide. However, the complex circumstances in which viruses are sequenced, along with the demand of urgent results, causes a high rate of incomplete and, therefore, useless sequences. Viral sequences evolve in the context of a complex phylogeny and different positions along the genome are in linkage disequilibrium. Therefore, an imputation method would be able to predict missing positions from the available sequencing data.

**Results:**

We have developed the impuSARS application, which takes advantage of the enormous number of SARS-CoV-2 genomes available, using a reference panel containing 239,301 sequences, to produce missing data imputation in viral genomes. ImpuSARS was tested in a wide range of conditions (continuous fragments, amplicons or sparse individual positions missing), showing great fidelity when reconstructing the original sequences, recovering the lineage with a 100% precision for almost all the lineages, even in very poorly covered genomes (<20%).

**Conclusions:**

Imputation can improve the pace of SARS-CoV-2 sequencing production by recovering many incomplete or low-quality sequences that would be otherwise discarded. ImpuSARS can be incorporated in any primary data processing pipeline for SARS-CoV-2 whole-genome sequencing.

## Background

SARS-CoV-2 is a 30-kb single-stranded RNA non-fragmented virus. It is classified, together with HCoV-OC43, HCoV-HKU1, SARS-CoV-1, and MERS-CoV, into the β coronaviridae. SARS-CoV-2 was first described in Wuhan, China, in December 2019 and is responsible for COVID-19, which was declared a pandemic by the World Health Organization (WHO) in March 2020 [1]. Whole-genome sequencing (WGS) has been successfully used for classification [2], studying transmission dynamics [3], and evaluating global and regional patterns of pandemic spread [4]. WGS also has the potential to study reinfections, which have been described in a number of patients [5], and has recently gained prominence to characterize viral variants that may escape the neutralizing activity of the antibodies produced by vaccines [6]. Unfortunately, WGS results, especially in complex scenarios like this pandemic, are often imperfect, rendering incomplete viral sequences, with significant regions of the genome poorly covered [7]. In fact, current systems for viral lineage identification, a highly relevant step for the control of potentially harmful strains, fail to provide a lineage assignment if a percentage (typically >50%) of the viral sequences is missing [8]. Given the short response times required in clinics, resequencing low-quality results is frequently not an option. Therefore, alternatives to improve sequencing results, used in other fields, such as genotype imputation, would be extremely useful in this scenario as well. Genotype imputation has traditionally been a crucial component of genome-wide association studies, by increasing the power of the findings, helping in their interpretation, and facilitating further meta-analysis [9]. Genotype imputation relies on the existing correlation between genetic variations or mutations at sites across the genome of an organism [10]. Using this correlation, imputation methods accurately assign genotypes at untyped markers, improving genome coverage [10–14]. The accuracy of this imputation process improves as the number of haplotypes in the reference panel of sequenced genomes increases [15, 16], especially for mutations present at low frequencies (minor allele frequency <0.5%). The accuracy can also be increased with large reference panels. In the case of human genomes, the Haplotype Reference Consortium, composed of ∼32,000 individuals, is considered a large panel, able to reach an accurate imputation for mutations with frequencies of ≤0.1–0.5% [14]. In the case of SARS-CoV-2, the outstanding international effort of sequencing has generated in a short time span a genomic database 10 times larger. In spite of the interest in WGS viral studies and the fact that typically the sequences are imperfect, with positions and regions missing, the imputation, with a few exceptions [17, 18], has scarcely been used in the viral realm, probably because resequencing them resulted in a more practical solution. However, in scenarios in which sampling is logistically complex or takes place under emergency conditions, like the SARS-CoV-2 pandemic, imputation may play a relevant role.

In addition, because WGS may not be routinely available for clinical laboratories, protocols for partial sequencing of the SARS-CoV-2 genome, or even partial sequencing of the spike, where most of the determinants for variant characterization are located, are becoming available [19]. Given the importance of sequencing viral whole genomes for epidemiologic surveillance purposes, as stressed by the WHO [20] and the European Parliament [21], a tool for genotype imputation in SARS-CoV-2 would increase the sequencing throughput by recovering many sequences discarded for low quality that still contain valid information for lineage or clade assignment. Similarly, sequencing kits that only cover some key stretches already miss (or will miss future) relevant mutations. Imputation may predict the existence of these variants of interest (VOI) or variants of concern (VOC) because of their linkage disequilibrium (LD) with resolved parts of the viral genome. Here a fully tested, highly accurate reference panel and tool for the imputation of SARS-CoV-2 whole-genome sequences from incomplete or partial sequences is presented.

## Materials and Methods

### SARS-CoV-2 Imputation

SARS-CoV-2 sequences’ imputation (impuSARS) was performed by using the Minimac software (Minimac, RRID:SCR_009292) [14]. Although Minimac was originally designed for human samples with diploid genotypes, the tool allows imputing haploid genomes as SARS-COV-2 because it supports imputation for non-pseudoautosomal regions at human males’ chromosome X. The reference panel was built with Minimac3 whereas Minimac4 was used for imputation. Minimac4 provides imputation qualities comparable to those of Minimac3, but it reduces memory usage and computational costs. The impuSARS tool accepts either FASTA sequence or variation (VCF) inputs. Note that FASTA sequence can include missing regions (which can be absent or tagged as N), which will be then imputed. FASTA input is aligned to reference with Muscle [22] to retrieve mutation positions. Also, VCF input should include both mutant and reference genotypes when available.

The initial reference panel was created with the available SARS-CoV-2 sequences from Global Initiative on Sharing All Influenza Data (GISAID) [23, 24] (downloaded on 7 January 2021). Only sequences including >29 kb and <1% missing bases were kept (“complete” and “high coverage” tags in GISAID, respectively). Also, sequences were converted to a multi-sample VCF format to only compute mutation positions. As defined by GISAID, the hCoV-19/Wuhan/WIV04/2019 sequence (accession No. EPI_ISL_402 124) was considered the official reference sequence. From this multi-sample VCF, unique mutations, i.e., private mutations for each sequence, were discarded. Therefore, the final reference panel contained 239,301 sequences. The parameter estimation for the reference panel had already been precomputed with Minimac (version 3) to speed up the imputation process (reference panel provided in M3VCF format). This reference panel is periodically updated to allow the collection of novel variants, especially VOIs and VOCs. The last reference panel (v3.0) was generated by July 2021 including >900,000 sequences and expanding it to other mutation types such as small indels.

Once the imputation is performed using the reference panel, impuSARS will retrieve the imputed consensus sequence provided by bcftools consensus v1.11 [25]. Also, the associated lineage for each imputed consensus sequence will be obtained with PANGOLIN v1.10.2 [8]. PANGOLIN assigns a detailed lineage identifier to each sequence on the basis of a multinomial logistic regression model [26]. PANGOLIN classifies sequences along a hierarchical tree reflecting evolutionary events. Each level of the hierarchical tree gathers a group of sequences with common evidence associated with an epidemiological event (usually related to new variations), which could produce an emerging edge of the pandemic [26]. Lineages becoming important in the lowest levels of the phylogeny are retagged with aliases to avoid infinite spread across the hierarchical tree, thus keeping it compacted in 4 levels at most.

Finally, although impuSARS was originally designed for SARS-CoV-2 imputation, note that the tool is adapted to impute any other viral genomes if required. For this purpose, impuSARS includes a complementary tool for users to create their customized reference panel from a set of sequences. Custom reference panels can then be used by impuSARS for other partial genome imputations. In that case, PANGOLIN lineages will be disabled because they are focused on SARS-CoV-2 lineages.

### Code availability

The imputation tool impuSARS has been encapsulated in a Docker container for interoperability and easy distribution purposes [27], and it is freely available on GitHub [28]. Additionally, the impuSARS tool has been registered in bio.tools and SciCrunch repositories under the identifiers biotools:impusars and RRID:SCR_021707, respectively.

### Validation procedure

SARS-CoV-2 imputation was evaluated by using a 10-fold cross-validation process. The dataset was randomly partitioned into 10 test subsets. For each test subset, the imputation panel was computed for the remaining 9 datasets (training subsets). Initially, the loss of genomic regions was simulated by progressively increasing the percentage of the missing genome by 10% intervals. Three different strategies were used to select these missing regions: (i) random selection of only 1 missing region (continuous block), (ii) random selection of mutation positions (missing sites), and (iii) random selection of amplicon regions that are usually independently amplified in SARS-CoV-2 sequencing (missing discontinuous blocks). Amplicon regions were defined by the hCoV-2019/nCoV-2019 v3 Amplicon Set [29] recommended by the ARTIC network [30]. Missing regions for amplicons were simulated as percentages of amplicons completely uncovered. The whole learning-testing procedure was repeated 3 times to reduce bias produced by the random selection. Additionally, imputation was also validated by iteratively removing a sliding window of 3 kb (∼10% of the entire genome) by 1.5-kb steps. This process will allow determination of those hot spot regions in the SARS-CoV-2 genome that are harder to impute if missed.

After validating imputation with several random selections, 2 more real scenarios were considered: (i) imputation from regions covered by the genotyping assay kit DeepChek®-8-plex CoV-2 [31] and (ii) imputation only from mutations belonging to the Spike protein (S) region. As above, a 10-fold cross-validation process was implemented in both cases. The genotyping assay covers several selected regions that represent ∼20% of the entire SARS-COV-2 genome; hence imputation can provide a more comprehensive, improved result. Alternatively, S protein is 1 of the most commonly sequenced regions for SARS-CoV-2 given its crucial role in the docking receptor recognition and cell membrane fusion [32, 33]. Moreover, mutations in Spike have been related to transmissibility or the ability to evade the host immune response [34]. Therefore, studying the ability of imputing the entire SAR-CoV-2 genome from the Spike region can benefit subsequent lineage classification, thus being crucial for epidemiological surveillance.

To facilitate the interpretation of the results the precision, recall, and F1 scores have been computed. Because this is a heavily unbalanced problem (much lower number of mutations against reference positions), the Matthews correlation coefficient (MCC) and balanced accuracy (BACC) scores, which are better suited for handling such scenarios [35–37], have also been provided. For these scores, positions with mutations in each real sequence are considered positive whereas reference positions are negative. Therefore, correctly imputed mutations and reference positions are considered true-positive and true-negative results, respectively. Otherwise, wrongly imputed mutations and reference nucleotides are computed as false-positive and false-negative. Thus, recall determines the true-positive rate whereas precision represents the positive predictive value. The F1-score represents the harmonic mean of the previous 2 metrics. The MCC measures the correlation and agreement between the truth and the predicted labels and varies between −1 and 1, where −1 refers to complete disagreement between the predicted and truth labels; 0, an average random prediction; and 1, a perfect prediction. Finally, the balanced accuracy is the arithmetic mean of sensitivity and specificity.

### Lineage classification

Imputations from simulated genotyping assay and Spike region test subsets were also evaluated in terms of the lineage assigned to the imputed sequences. A standard accuracy metric was calculated to evaluate assigned lineages from imputed sequences against real lineages from original GISAID sequences. Additionally, 2 baseline models were implemented to evaluate the influence of known mutations against missing ones over the assignment of lineages. The first baseline model simply filled missing regions with the SARS-CoV-2 reference sequence. The second model randomly generated the genotype to the missing mutation positions of the entire test subset weighting probabilities by the original genotype frequency in the training datasets. For comparison purposes, lineages were also obtained for the resulting sequences using these 2 baseline models.

### Imputation test with independent datasets

After the entire validation process, the final reference panel including the 239,301 GISAID sequences was built. Several independent datasets were considered for this test phase using the definitive reference panel: (i) new GISAID sequences not included in the reference panel belonging to lineages of interest; (ii) 8 samples sequenced at the Hospital San Cecilio (Granada, Spain) by using both the DeepChek®-8Plex-CoV2 genotyping array [31] and WGS as described below; and (iii) 1 sample, assigned to the B.1.351 (β-variant) [38] by an experimental RT-PCR kit, subjected to WGS that resulted in an incomplete whole-genome sequence, at Hospital Virgen del Rocio (Seville, Spain).

In the first test, new GISAID sequences from highly relevant lineages like B.1.1.7 (α-variant) [39] and B.1.351 (β-variant) [38] were selected: 64,398 and 970 sequences, respectively (sequences downloaded by 23 February 2021). As in the previous validation phase, these sequences were also tested by iteratively removing a 3-kb window sliding by 1.5-kb steps in the entire genome. In this way the importance of specific regions to impute relevant lineages could be evaluated. In the second test the variations obtained by the genotyping array were used to impute the entire genome and the assigned lineages are compared against whole-genome results. Finally, the imputation tool was used in a third test to solve a real case in which an experimental research use only (RUO) test warned of a potential VOC but the confirmatory WGS was of poor quality in a scenario where a quick informed decision was required. Then, the poor-quality sequence was used to impute the whole-genome sequence and lineage. The resolution of this case proves the level of resolution and accuracy of the imputation procedure presented here.

### RT-PCR detection of variants SARS-CoV-2 B.1.1.7, B.1.351, and B.1.1.28.1

An alternative experimental detection of variants SARS-CoV-2 B.1.1.7, B.1.351, and B.1.1.28.1 was performed by RT-PCR using an RUO kit (SARS-CoV-2 variants RT-PCR, Vitro SA, Sevilla, Spain) to detect the presence and/or absence of specific targets in ORF1ab gen (deletion SGF 3675–3677) and Spike gen (deletion HV 69–70).

### Genotyping array and whole-genome sequencing of viral samples

Eight SARS-CoV-2 nasopharyngeal samples were sequenced following the manufacturer DeepChek®-8Plex-CoV2 genotyping array protocol [31]. WGS of the same samples was carried out following the ARTIC protocol [30] with the hCoV-2019/nCoV-2019 v3 Amplicon Set [29]. Whole-genome samples were sequenced in a NextSeq 500 sequencer by Illumina with 150-bp paired-end reads and a total coverage of ∼500,000 reads per sample.

### Sequence data preprocessing

Sequencing data (150 bp ×2) were analyzed using in-house scripts and the nf-core/viralrecon pipeline software [40]. Briefly, after read quality filtering, sequences for each sample were aligned to the SARS-CoV-2 isolate Wuhan-Hu-1 reference genome (MN908947.3) using bowtie 2 algorithm (Bowtie, RRID:SCR_005476) [41], followed by primer sequence removal and duplicate read marking using iVar [42] and Picard (Picard, RRID:SCR_006525) [43] tools, respectively. Genomic mutations are identified through iVar software, using a minimum allele frequency threshold of 0.25 for calling mutations and a filtering step to keep mutations with a minimum allele frequency threshold of 0.75. Using the set of high-confidence mutations and the MN908947.3 genome, a consensus genome per sample is finally built using iVar.

## Results and Discussion

### Imputation of randomly simulated missing regions

**Figure 1:**
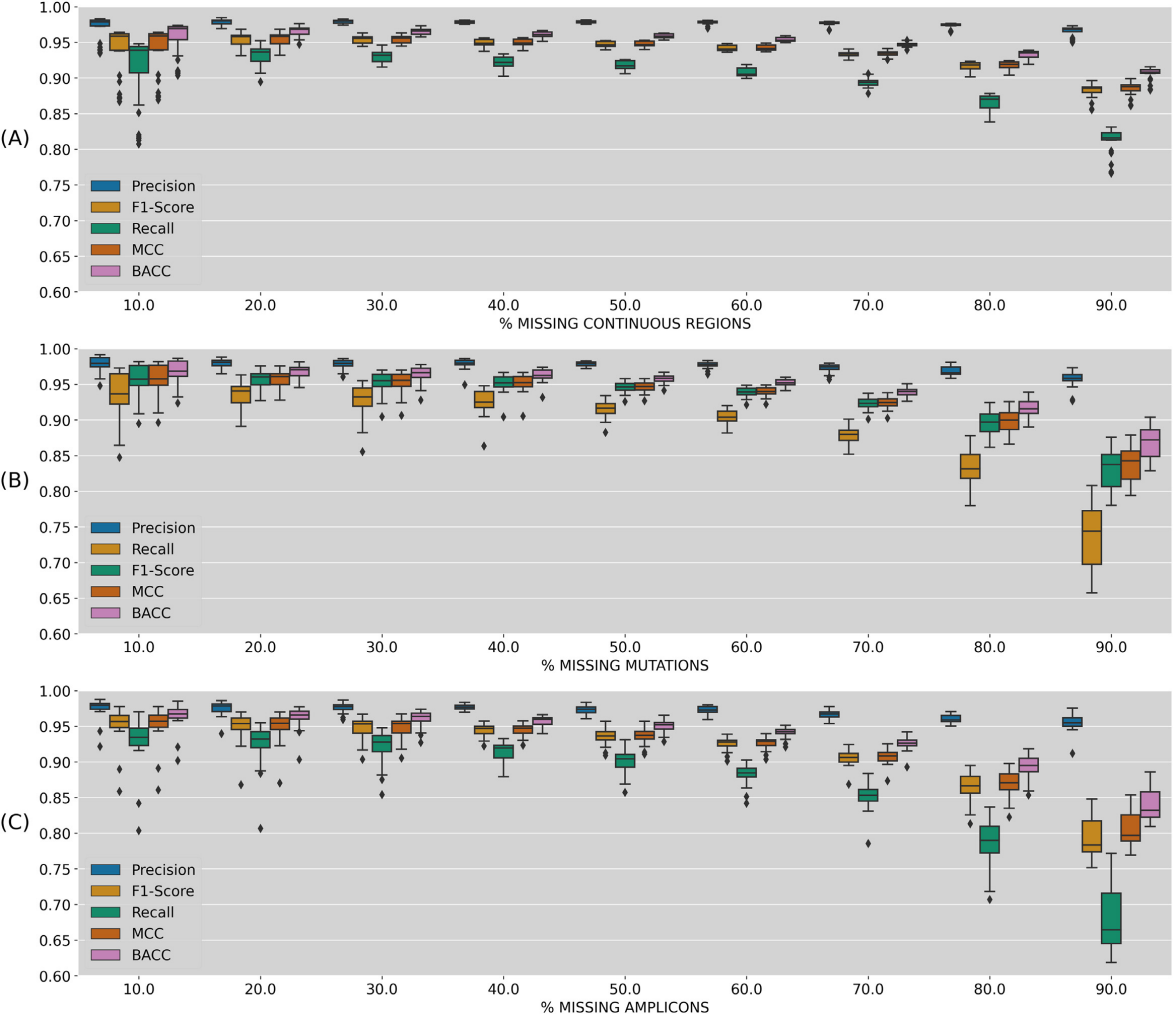
Imputation performance metrics (precision, recall, F1-score, MCC, and BACC) depending on missing genome percentage. (A) One random continuous block of the genome; (B) random selection of missing variants; (C) random selection of missing amplicons. In the Boxplot the box contains the two quartiles around the median, represented by the horizontal line in the box, and the wiskers represent the maximum and minimum value. Dots outside these limits are outlayers.

Each of the 10 test subsets in the 10-fold cross-validation was reduced by randomly simulating missing regions in increasing percentages (10–90%). This process was repeated 3 times for each missing percentage. Classification metrics (MCC, BACC, and F1-score) were obtained for each reduced test dataset as shown in Fig. 1A for 1 random region (missing continuous blocks), Fig. 1B for randomly selected mutations (missing sites), and Fig. 1C for randomly selected amplicons (missing discontinuous blocks). In all cases, imputation performance metrics mean values were >0.65 even for the worst scenario (imputing only from 10% of the genome). Imputation progressively improves when known sequence percentages are increasing, reaching mean values >0.95 for those tests with 90% known genomes. Interestingly, the performance metrics presented a higher dispersion (including some lower outliers) when imputing only 10% of the genome in 1 continuous block (Fig. 1A) whereas this dispersion is more marked at the opposite end of the range of values, for 90% missing regions for missing mutations and discontinuous blocks (Fig. 1B and C). This behavior might be related to the fact that leaving only 1 small random block to impute can involve regions where mutations are rare and harder to impute, even with the remaining 90% known ones. The imputation by missing sliding windows proposed in the next section will help to confirm that hypothesis. Finally, even for extremely high missing percentages like the genotyping assays (∼80%) or only Spike regions used below, the obtained metrics suggest a reasonably accurate imputation.

### Effects of missing specific locations

**Figure 2:**
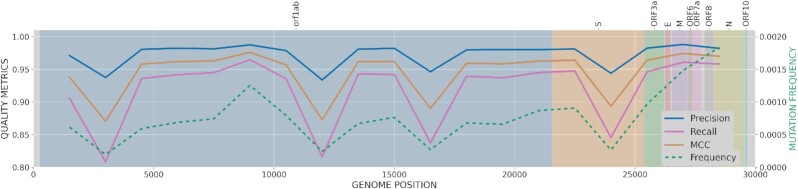
Imputation performance metrics (precision, recall, and MCC) based on the position of a missing 3-kb window along the SARS-CoV-2 genome. Left y-axis values represent variant frequencies (dashed green line). SARS-CoV-2 protein regions are represented by colored background and names specified at the top.

As previously noted, imputation performance is strongly associated with the region missing coverage in the SARS-CoV-2 genome. Therefore, the importance of selecting adequate regions when sequencing SARS-CoV-2 samples and its influence in a subsequent imputation of the remaining regions is analyzed here. For this purpose, a 3-kb window was iteratively removed and imputed from the entire genome, repeating the process by 1.5-kb steps. For the sake of clarity, only key metrics such as precision, recall, and MCC of each imputed window along the entire genome are shown in Fig. 2. Additional metrics BACC and F1-Score are available in Supplementary Fig. S1. Several hot spots (4 regions) have been identified as critical positions where mutations are harder to impute when the block around is missing. More specifically, uncovered regions in positions around 3k, 12k, 16.5k (orf1ab protein, replicase polyprotein 1ab), and 24k (S protein, Spike glycoprotein) would slightly reduce imputation ability. As previously suggested, note that those identified hot spots are strongly associated with regions where mutations are less frequent in the reference panel (dashed green line). Recall values tend to be lower than precision because of the private mutations in the variants, which are virtually impossible to impute because of the lack of information on LD with other mutations. This is not a problem of impuSARS but a general drawback of any imputation method or strategy.

### Imputation from genotyping assay and spike regions

**Figure 3:**
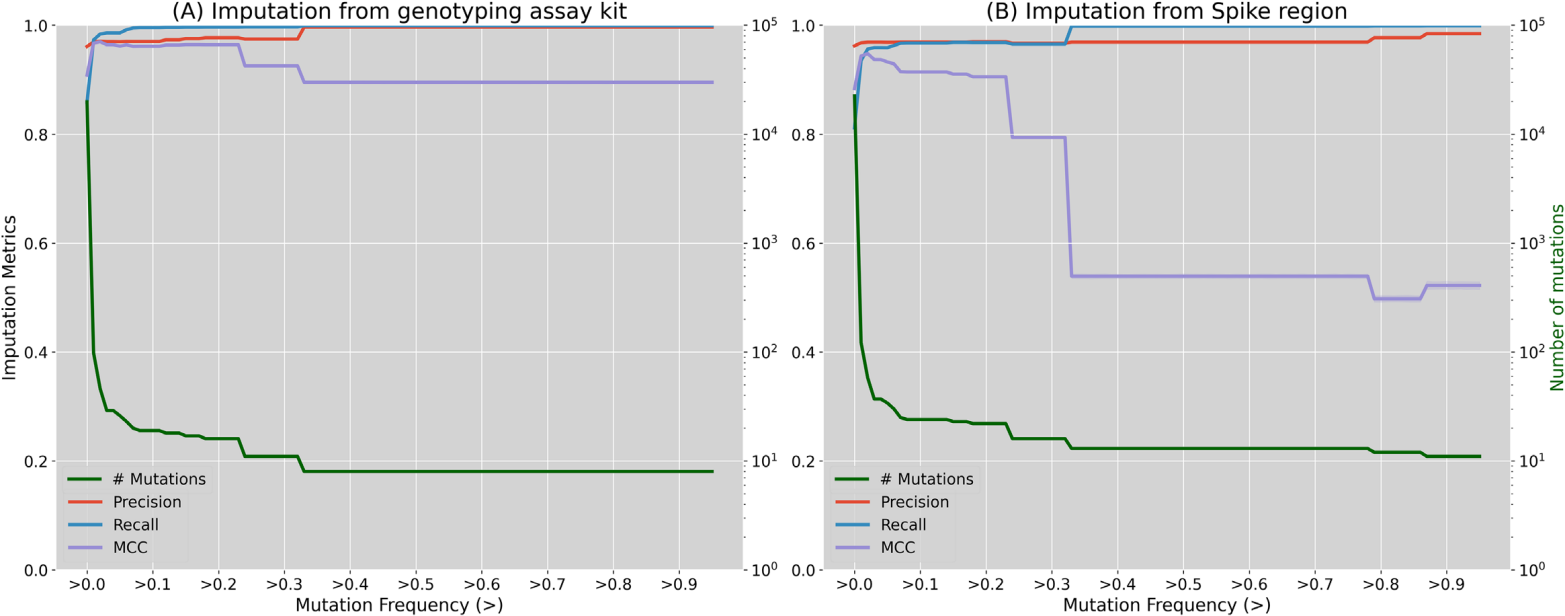
Principal imputation performance metrics (precision, recall, and MCC) calculated depending on imputed variant frequencies. (A) Imputation quality when imputing from the genotyping array positions; (B) imputation quality when imputing from Spike protein positions. Left y-axis (green) represents the number of variants for those frequency thresholds (log scale).

Once the robustness of the imputation in different missing region scenarios has been validated, the focus is set on the validation of the imputation of genomes using only data from the genotyping assay regions previously described or from the Spike protein region. Table 1 shows imputation performance metrics for both cases per test subset. Also, these metrics were calculated against the frequency of imputed mutations in the reference panel (Fig. 3). In both cases, only the representative metrics precision, recall, and MCC were kept. Detailed results for the other mentioned metrics (BACC and F1-score) can be found in Supplementary Table S1 and Supplementary Fig. S2. As shown in Table 1, the imputation performance surpasses 0.81 in the 3 averaged metrics, precision being the highest with >0.96 for both regions while recall remains at 0.86 and 0.81 for genotyping assay and Spike regions, respectively. Regarding Fig. 3, mutation imputation quickly increases to >0.96 in the 3 performance metrics (recall, precision, and MCC) for mutations with frequencies >0.01 and >0.03 for the genotyping array and Spike region imputations, respectively. The imputation from genotyping array sequences reaches its maximum values (>0.996) from frequencies >0.33 for precision and recall metrics, whereas MCC slightly decreases to 0.895 after the same frequency threshold. For imputation from the Spike region, an improvement is also observed from mutation frequencies >0.33 reaching performance values of 0.998 and 0.969 for recall and precision, respectively, but a more drastic decrease is observed in MCC. This MCC decrease is correlated in both cases with the decrease in the number of mutations (green line). When mutation frequency increases, a smaller number of mutations are found but datasets are inversely unbalanced (more mutant than reference positions), which metric-wise is better captured by the MCC. Nevertheless, imputing positive cases (mutations) in those situations is more relevant, so results in recall and precision metrics are more informative.

**Table 1:** Performance metrics (recall, precision, and MCC)

Subset	Imputation from genotyping assay kit			Imputation from Spike region		
	Recall	Precision	MCC	Recall	Precision	MCC
1	0.8595	0.9612	0.9088	0.8129	0.9618	0.8841
2	0.8578	0.9597	0.9072	0.8121	0.9620	0.8838
3	0.8562	0.9614	0.9072	0.8100	0.9625	0.8829
4	0.8609	0.9622	0.9101	0.8106	0.9616	0.8828
5	0.8589	0.9603	0.9081	0.8109	0.9619	0.8831
6	0.8593	0.9602	0.9083	0.8106	0.9608	0.8824
7	0.8586	0.9600	0.9078	0.8126	0.9613	0.8837
8	0.8597	0.9614	0.9091	0.8106	0.9624	0.8831
9	0.8579	0.9605	0.9077	0.8115	0.9622	0.8835
10	0.8574	0.9609	0.9076	0.8121	0.9629	0.8842
Mean ± SD	0.8586 ± 0.0013	0.9608 ± 0.0008	0.9082 ± 0.0009	0.8114 ± 0.0010	0.9619 ± 0.0006	0.8834 ± 0.0006

Metrics obtained for 10-fold cross-validation subsets imputing from the genotyping assay and Spike protein regions. Values are calculated for the entire test subset imputation.

### Lineage classification

**Figure 4:**
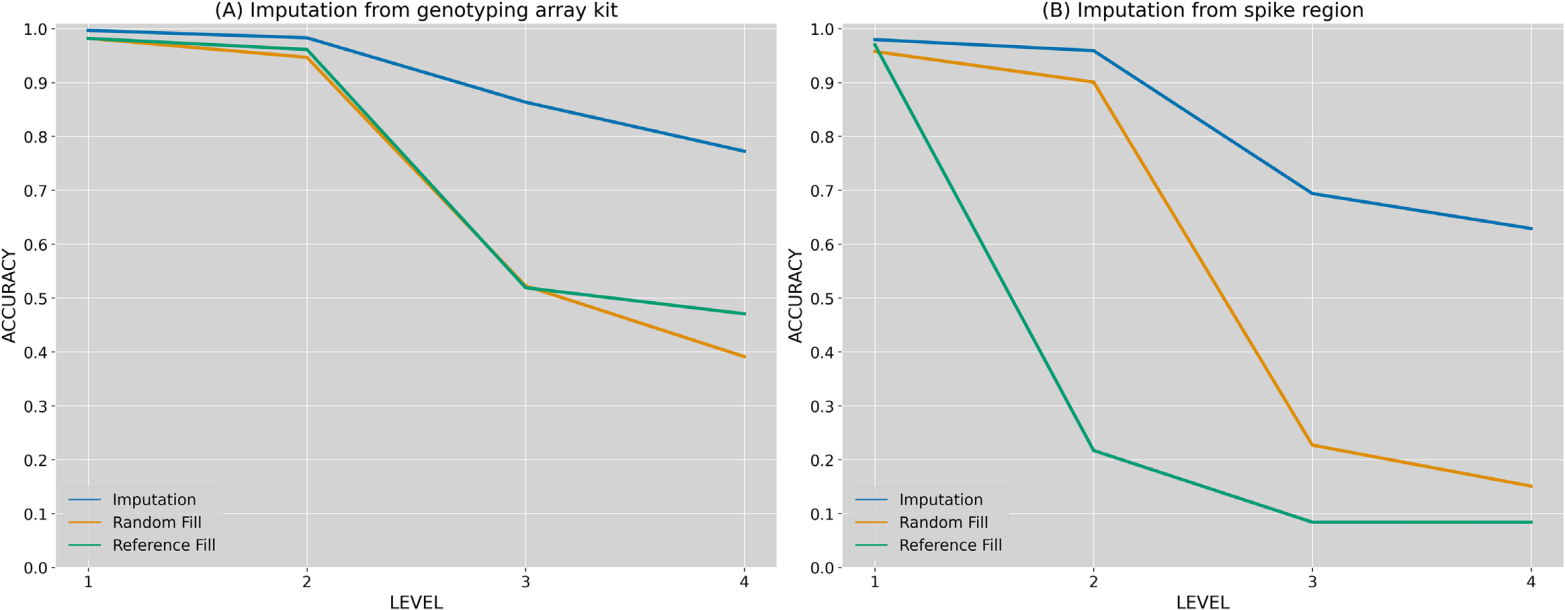
Lineage classification accuracy compared against 2 baseline models. (A) Lineage accuracy when imputing from the genotyping array positions; (B) lineage accuracy when imputing from Spike protein region. Levels represent lineage specification.

The previously imputed mutations for the simulated genotyping arrays and Spike region subsets are used to rebuild the consensus whole-genome sequences and assign their corresponding lineages with PANGOLIN. The quality of the imputed lineage has been measured by the accuracy metric against real lineages and compared to 2 baseline models (Fig. 4). Briefly, these 2 models, respectively, filled missing regions with random mutations assigned by frequency (“Random fill”) or with nucleotides from the reference sequence (“Reference fill”) (see Material and Methods section: for details). Also, accuracy was calculated for the different levels of the hierarchical tree in PANGOLIN lineages. As shown, the first level in the hierarchical classification of lineage was almost always correctly determined (>98%), even for the 2 baseline models. That is, the information provided by the already known regions (genotyping array and Spike protein) was enough to classify this first level. However, the imputed solution becomes more relevant as a lower level has to be determined. Hence, imputation clearly outperformed both baseline methods when lineages were assigned at third and fourth level, achieving 77% and 68% accuracy for genotyping array and spike regions, respectively. As expected, imputation from the genotyping array positions comes up with higher lineage accuracies than the solution with Spike because this kit was specifically designed to capture relevant regions in the SARS-CoV-2 genome. Even so, imputation still produces strong benefits in the lineage assignment for the genotyping array regions, clearly improving lineage assignments with simple baseline models.

**Figure 5:**
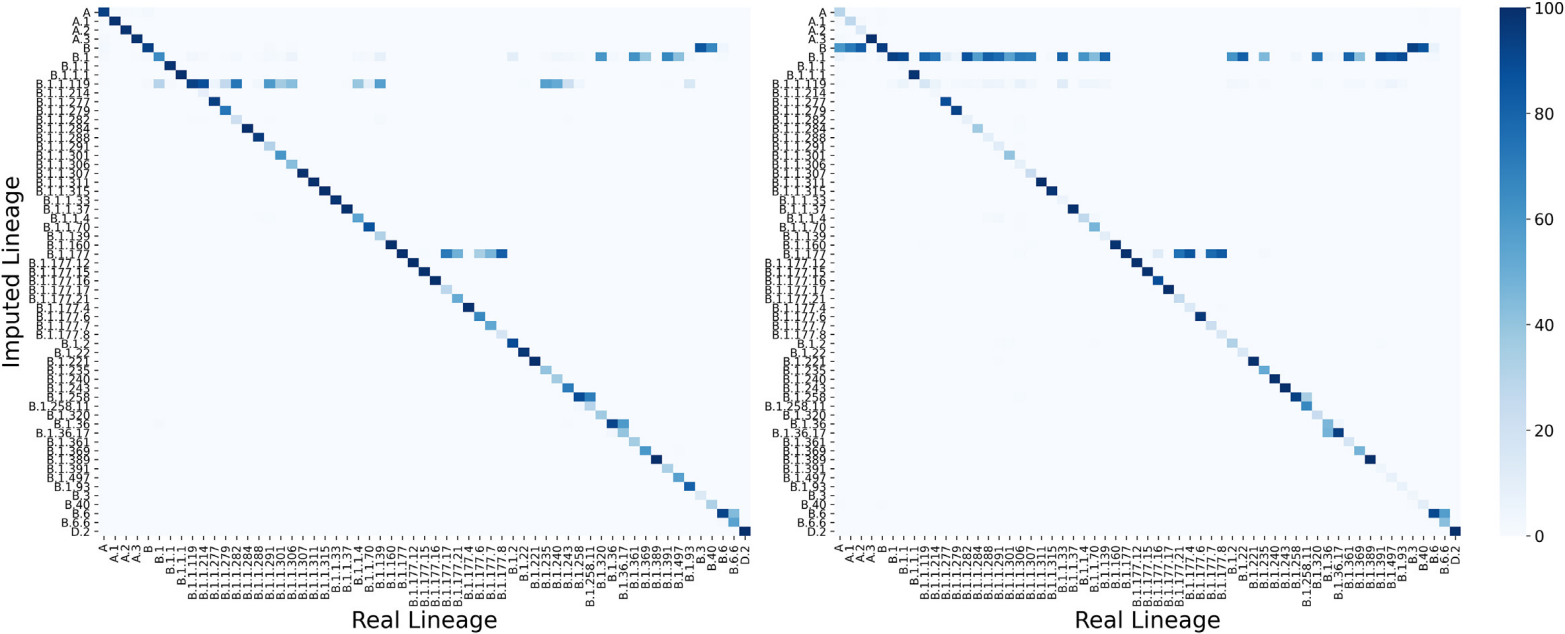
Accuracy obtained for each pair of lineages (real vs imputed) for the top frequent lineages (>500 sequences). Left heat map represents the obtained values for genotyping array imputation whereas right heat map represents accuracies for imputation from Spike protein region. Color represents the percentage of sequences in each real lineage classified by each imputed lineage (the darker, the higher).

Additionally, a detailed view of lineage classification for the top frequent lineages (>500 sequences) is shown in Fig. 5. As noted, there are lineages that are more commonly misclassified. For instance, several sequences are wrongly classified as B.1.1.119 when imputing from the genotyping array regions. Similarly, lineage B.1 is frequently assigned when sequences truly belong to a more specific lineage (lower level in the hierarchical tree) in the imputation from Spike. In the first case, this misclassification is produced by the fact that lineage B.1.1.119 is partially constituted by 3 mutations in positions 28,881–3, which are not captured by the genotyping array that was used. This situation makes sequences from other close lineages like B.1, B.1.1.214, or B.1.1.282 identical to B.1.1.119, from the genotyping array perspective. Consequently, these close lineages are frequently imputed as B.1.1.119 (30%, 88%, and 73%, respectively). Likewise, given the lack of certain regions when imputing from Spike region, several sub-branches like B.1.1.119, B.1.1.214, B.1.1.282, or B.1.1.284 are wrongly classified as the parent node B.1 (80%, 75%, 87%, and 57%, respectively). Although the percentages of misclassification are quite high in these cases, affected lineages are less relevant for prospective imputation purposes because they belong to early phases from the virus evolution, with less informative mutations, not being classified as VOI or VOC, and some of them almost or already extinct. Otherwise, VOCs like α and β were more accurately classified imputing from both the genotyping array (78.3% and 99% accuracy, respectively) and Spike region (78.3% and 98.1%).

### Imputation of new independent datasets

Previous sections have extensively validated the proposed imputation system under several configurations and strategies. This section shows several use cases and test results produced by independent datasets over the final imputation reference panel (239,301 sequences).

**Figure 6:**
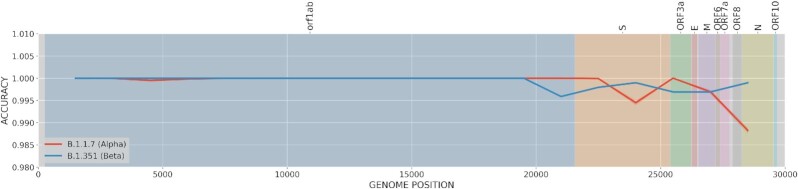
Lineage classification accuracy. Accuracy is estimated for a missed region in sliding windows of 3 kb for the recent α- and β-lineages (B.1.1.7 and B.1.351, respectively)

First, 2 recently emerging lineages, B.1.1.7 (α-variant) and B.1.351 (β-variant), have also been studied in this final testing phase to evaluate the performance of the imputation in new lineages. Sequences recently added to GISAID (not included in our presference panel) under these lineages were selected: 64,398 and 970 sequences, respectively. Their percentage of cosrrectly classified lineages after imputation when missing a 3-kb window (10%) along the entire genome are then calculated (Fig. 6).

As shown in Fig. 6, even when these lineages are underrepresented in the present reference panel (23 and 105 sequences, respectively), the methodology has captured the LD structure at such precision that it can accurately impute the B.1.1.7 and B.1.351 lineages from other sequences. Specifically, both lineages obtained 100% accuracy for almost any missing 3-kb region. The imputation accuracy was slightly reduced in the α-variant (B.1.1.7) when the missing regions are located around the center of S protein (99.5% accuracy) or at ORF8 and N proteins (99% accuracy). This behavior is clearly associated with the loss of constitutive mutations for the α-variant such as N501Y, A570D, or P681H, among others [44]. In the case of the β-variant (B.1.351), performance slightly decreased at the beginning of protein S (99.5%), as well as around E and M proteins (99.8%). Again, these small decreases are associated with important mutations associated with the lineage such as Q57H or P71L [45].

### Imputation for sequencing kits and low-quality sequences

Eight SARS-CoV-2 samples were sequenced using the DeepChek®-8-plex CoV-2 genotyping array (see Table 2). The partial sequences covering ∼20% of the whole viral genome were used to impute the remaining non-covered 80% of the genome with impuSARS. Then, the same samples were subjected to WGS. The imputed whole-genome sequences and lineages were subsequently compared against each other, rendering a highly reliable imputation sequence and 100% successful lineage imputation. FASTQ files as well as consensus whole-genome sequences for both genotyping array and WGS of these 8 samples are available for download at the European Nucleotide Archive (ENA) under the accession ID PRJEB43882. Also, imputation results (both imputed consensus whole-genome sequences and lineages) are provided in the Zenodo repository [46]. Coverage distribution from initial genotyping array results is provided in Supplementary Fig. S3. The 3 main quality metrics and imputed lineages are shown in Table 2. A more detailed table including mutation counts and additional metrics is provided (Supplementary Table S2).

**Table 2:** Variant imputation metrics (precision, recall, and MCC) and lineage classification

Sample	Recall	Precision	MCC	Real lineage	Imputed
AND00023	0.9000	1	0.9486	B.1.1.7	B.1.1.7
AND00040	0.8571	1	0.9258	B.1.1.7	B.1.1.7
AND00065	0.8636	1	0.9293	B.1.1.7	B.1.1.7
AND00073	0.8571	1	0.9258	B.1.1.7	B.1.1.7
AND00123	0.9231	1	0.9607	B.1.1.7	B.1.1.7
AND00128	0.6000	1	0.7745	B.1.1.7	B.1.1.7
AND00132	0.8696	1	0.9324	B.1.1.7	B.1.1.7
AND00139	0.9091	1	0.9534	B.1.1.7	B.1.1.7
Mean ± SD	0.8475 ± 0.103	1.0000 ± 0	0.9188 ± 0.06		100%

Values for 8 independent samples internally sequenced with both the genotyping array and whole-genome sequencing.

To further illustrate the usefulness of the imputation system in a real clinical scenario, a use case of the Hospital Virgen del Rocio is described. In a routine survey a sample was analyzed by RT-PCR using a RUO kit (see Material and Methods section for details), which raised a warning suggesting that it may belong to the emerging β-variant (B.1.351), a VOC. The sample was immediately submitted to confirmatory WGS, which resulted in a poor-quality sequencing, with only 28.91% of SARS-CoV-2 genome covered, having 71 amplicons completely non-covered and 3 covered at low depth (<20×). Lineage assignment with current tools like PANGOLIN is impossible in this low-quality scenario. However, it was urgent to confirm or discard the presence of a VOC for epidemiologic surveillance and medical decision making. Therefore, impuSARS was used on this poor-quality sequence and lineage imputation was carried out with PANGOLIN, producing a B.1.1.7 lineage (α) assignment, also a VOC, but currently more extended in Spain. Detailed analysis of the pattern of available mutations also supported this lineage assignment (see Table 3).

**Table 3:** Study of AND00344 variants

Mutation	Found in variant	Present	Coverage	α	β	γ
L18F	β/γ	No	None		?	?
T20N	γ	No	None			?
P26S	γ	No	None			?
del_21765	α	No	None	?		
D80A	β	No	None		?	
D138Y	γ	No	None			?
del_21991	α	No	None	?		
R190S	γ	No	None			?
D215G	β	No	None		?	
del_22281	β	No	Covered		No	
R246I	β	No	Covered		No	
K417N	β/γ	No	None		?	?
E484K	β/γ	No	Low		No?	No?
N501Y	α/β/γ	Yes	Covered	Yes?	Yes?	Yes?
A570D	α	No	None	?		
D614G	α/β/γ	No	None	?	?	?
H655Y	γ	No	Covered			No
P681H	α	Yes	Covered	Yes	No	No
A701V	β	No	Covered		No	
T716I	α	Yes	Covered	Yes	No	No
S982A	α	No	None	?		
T1027I	γ	No	None			?
D1118H	α	No	None	?		
Q57H	β	No	Covered		No	
P71L	β	No	Covered		No	
Q27stop	α	Yes	Covered	Yes	No	No
T205I	β	No	Low		No?	
Total				Yes (most likely)	No	No (most likely)

Comparison of the available variation in the low-coverage sequence of vial sample AND00344 with respect to the α (B.1.1.7), β (B.1.351), and γ (P.1) VOCs.

### Indels imputation

As shown in previous sections, impuSARS was originally designed and validated for imputation of single-nucleotide polymorphisms (SNPs). In fact, SNPs clearly represent most mutations in SARS-CoV-2 sequences with >33 SNPs per sequence against only 3.12 deletions and almost no insertions (0.4 on average) (see frequencies in Supplementary Fig. S4). However, emerging VOCs are progressively incorporating more indels of interest, mainly short deletions of 1–3 codons (3–12 nucleotides). This is the case, for example, for the 2-codons deletion S:69–70del in α-variant, the 3-codons deletion ORF1a:3675–3677del in β-variant, or, more recently, the deletion S:157–158del in δ-variant. Consequently, impuSARS has been recently updated to accept and also impute short indels by designing a new reference panel (v3.0). Although it is out of the scope of this articlemputation has been successfully validated with the most representative indels like those previously mentioned. In fact, indel imputation has also produced significant improvements in lineage classification.

## Conclusions

Whole-genome sequence imputation from partial sequences from commercial kits or from low-quality WGS has been demonstrated to produce highly reliable results and be an excellent tool for lineage assignment. Given the short response times required for the identification of samples for decision support or for epidemiological surveillance in a clinical context, re-sampling and/or re-sequencing are not realistic options. Therefore, imputation constitutes an accurate and useful tool to complement and improve SARS-CoV-2 WGS pipelines in clinics.

## Availability of Source Code and Requirements

Project name: impuSARS (SARS-CoV-2 imputation)

Project home page: https://github.com/babelomics/impuSARS

Operating system(s): Platform independent (Docker container or, alternatively, conda environment)

Programming language: Python, Bash

Other requirements: Docker, Conda

License: MIT License

Any restrictions to use by non-academics: none

biotools:impusars

RRID:SCR_021707

## Data Availability

The SARS-CoV-2 sequences used to train the impuSARS tool were taken from GISAID [47].

The hCoV-19/Wuhan/WIV04/2019 sequence (EPI_ISL_402 124) was taken from GISAID [48].

The imputation results (both imputed WGS and lineages) are provided in the Zenodo repository [46]. Tabular data and a snapshot of the code are also available in the GigaDB repository [49].

The 8 SARS-CoV-2 whole-genome sequences generated in this study are available at the European Nucleotide Archive [50].

## Additional Files

**Supplementary Table S1:** Supplementary imputation performance metrics (BACC and F1)

**Supplementary Table S2:** Mutation counts and additional metrics

**Supplementary Table S3**: List of the originating laboratories responsible for obtaining the specimens and the submitting laboratories whare the genomes were generated amd shared via GISAID according to the GISAID policy of acknowledgements.

**Supplementary Figure S1:** More imputation performance metrics (F1 and BACC) based on the position of a missing 3-kb window along the SARS-CoV-2 genome. Left y-axis values represent mutation frequencies (dashed green line). SARS-CoV-2 protein regions are represented by colored background and names specified at the top.

**Supplementary Figure S2:** Supplementary imputation performance metrics (BACC and F1) calculated depending on imputed mutation frequencies. (A) Imputation quality when imputing from the genotyping array positions; (B) imputation quality when imputing from spike protein positions. Left y-axis (green) represents the number of mutations for those frequency thresholds (log scale).

**Supplementary Figure S3:** Coverage distribution from genotyping array in the 8 samples studied.

**Supplementary Figure S4:** Frequencies of the different types of mutations (single-nucleotide variants, insertions, and deletions) per SARS-CoV-2 genome.

## Abbreviations

BACC: balanced accuracy; bp: base pairs; GISAID: Global Initiative on Sharing All Influenza Data; kb: kilobase pairs; LD: linkage disequilibrium; MCC: Matthews correlation coefficient; RT-PCR: real time polymerase chain reaction; RUO: research use only; SNP: single-nucleotide polymorphism; VCF: Variant Calling Format; VOC: variant of concern; VOI: variant of interest; WGS: whole-genome sequencing; WHO: World Health Organization.

## Competing Interests

The authors declare that they have no competing interests.

## Funding

This work is supported by grant PT17/0009/0006 from the Spanish Ministry of Economy and Competitiveness, COVID-0012–2020 from Consejería de Salud y Familias, Junta de Andalucía, and postdoctoral contract PAIDI2020- DOC_00350 for C.L., from Junta de Andalucía, co-funded by the European Social Fund (FSE) 2014–2020.

## Authors’ Contributions

F.M.O. performed most of the analysis and wrote the draft of the manuscript; C.L. carried out the statistics part of the work; C.S.C.S. and J.P.F. contributed to the analysis of the samples; J.A.L., P.C.M., and L.M.D. carried out the use case of the RUO kit; A.S., N.C., and F.G. carried out the commercial kit use case; and J.D. conceived the work and wrote the manuscript. All authors reviewed and declare that they have no conflict of interest.

## Supplementary Material

giab078_GIGA-D-21-00168_Original_Submission

giab078_GIGA-D-21-00168_Revision_1

giab078_GIGA-D-21-00168_Revision_2

giab078_Response_to_Reviewer_Comments_Original_Submission

giab078_Response_to_Reviewer_Comments_Revision_1

giab078_Reviewer_1_Report_Original_Submission Siyang Liu -- 8/4/2021 Reviewed

giab078_Reviewer_2_Report_Original_Submission Stephen Nayfach -- 9/1/2021 Reviewed

giab078_Reviewer_2_Report_Revision_1 Stephen Nayfach -- 10/29/2021 Reviewed

giab078_Supplemental_Files
