# Supplementary material for: Highly accurate whole-genome imputation of SARS-CoV-2 from partial or low-quality sequences
Source: Gigascience. 2021 Dec 2;10(12):giab078. doi: 10.1093/gigascience/giab078 (PMC8643610; doi:10.1093/gigascience/giab078)
Supplement: giab078_Supplemental_Files [file giab078_supplemental_files.zip › AdditionalFile1.pdf]

Imputation of SARS-CoV-2 whole genome sequences from incomplete or partial sequences

Francisco M Ortuño[1,2], Carlos Loucera[1,2], Carlos S. Casimiro-Soriguer[1,2], Jose A. Lepe[3], Pedro Camacho Martinez[3], Laura Merino Diaz[3], Adolfo de Salazar[4], Natalia Chueca[4], Federico García[4], Javier Perez-Florido[1,2], Joaquin Dopazo[1,2,5*]

1 Clinical Bioinformatics Area. Fundación Progreso y Salud (FPS). CDCA, Hospital Virgen del Rocio. 41013. Sevilla. Spain;
2 Computational Systems Medicine, Institute of Biomedicine of Seville (IBIS), Hospital Virgen del Rocio. 41013. Sevilla. Spain;
3 Unidad Clínica Enfermedades Infecciosas, Microbiología y Medicina Preventiva. Hospital Universitario Virgen del Rocío. 41013. Sevilla. España.
4 Servicio de Microbiología. Hospital Universitario San Cecilio. 18016. Granada. Spain
5 FPS/ELIXIR-es, Hospital Virgen del Rocío, Sevilla, 42013, Spain.

Supplementary Tables:

Table S1. Supplementary imputation performance metrics (BACC and F1)

Table S2. Mutation counts and additional metrics

Supplementary Figures:

Fig. S1. More imputation performance metrics (F1 and BACC) based on the position of a missing 3kb window along the SARS-CoV-2 genome.
Left y-axis values represent mutation frequencies (dashed green line). SARS-CoV-2 protein regions are represented by colored background and names specified at the top.

Fig. S2. Supplementary imputation performance metrics (BACC and F1) calculated depending on imputed mutation frequencies. (A) Imputation quality when imputing from the genotyping array positions; (B) Imputation quality when imputing from spike protein positions. Left y-axis (green) represents the number of mutations for those frequency threshold (log scale)

Fig S3. Coverage distribution from genotyping array in the eight samples studied.

Fig. S4. Frequencies of the different types of mutations (SNVs, insertions and deletions per SARS-CoV-2 genome.

Table S1. Supplementary imputation performance metrics (BACC and F1)

Subset	Imputation from genotyping assay kit		Imputation from Spike region	
	BACC	F1-Score	BACC	F1-Score
1	0,9297	0,9075	0,9064	0,8811
2	0,9289	0,9059	0,9060	0,8807
3	0,9281	0,9058	0,9050	0,8797
4	0,9305	0,9088	0,9053	0,8797
5	0,9294	0,9068	0,9054	0,8800
6	0,9296	0,9069	0,9053	0,8793
7	0,9293	0,9065	0,9063	0,8807
8	0,9298	0,9077	0,9053	0,8800
9	0,9289	0,9063	0,9057	0,8804
10	0,9287	0,9062	0,9060	0,8811
**Average**	0,9293	0,9068	0,9293	0,9068
**Std Dev**	0,0007	0,0009	0,0007	0,0009

Table S2. Mutation counts and additional metrics

Sample	Number of Ref Positions		Number of Variants				Recall	Precision	MCC	BACC	F1-Score
	In kit	From WGS not in kit (N)	In kit	From WGS not in kit (P)	Imputed (TP + FP)	Correctly Imputed (TP)					
AND00023	7005	22762	11	20	18	18	0,9000	1	0,9486	0,9500	0,9474
AND00040	7313	22455	20	14	12	12	0,8571	1	0,9258	0,9286	0,9231
AND00065	6509	23257	12	22	19	19	0,8636	1	0,9293	0,9318	0,9268
AND00073	7297	22466	23	14	12	12	0,8571	1	0,9258	0,9286	0,9231
AND00123	7379	22388	20	13	12	12	0,9231	1	0,9607	0,9615	0,9600
AND00128	7448	22319	11	20	12	12	0,6000	1	0,7745	0,8000	0,7500
AND00132	6159	23601	19	23	20	20	0,8696	1	0,9324	0,9348	0,9302
AND00139	6425	23338	12	22	20	20	0,9091	1	0,9534	0,9545	0,9524
						**Avg**	0,8475	1,0000	0,9188	0,9237	0,9141
						**Std Dev**	0,1031	0,0000	0,0599	0,0516	0,0678

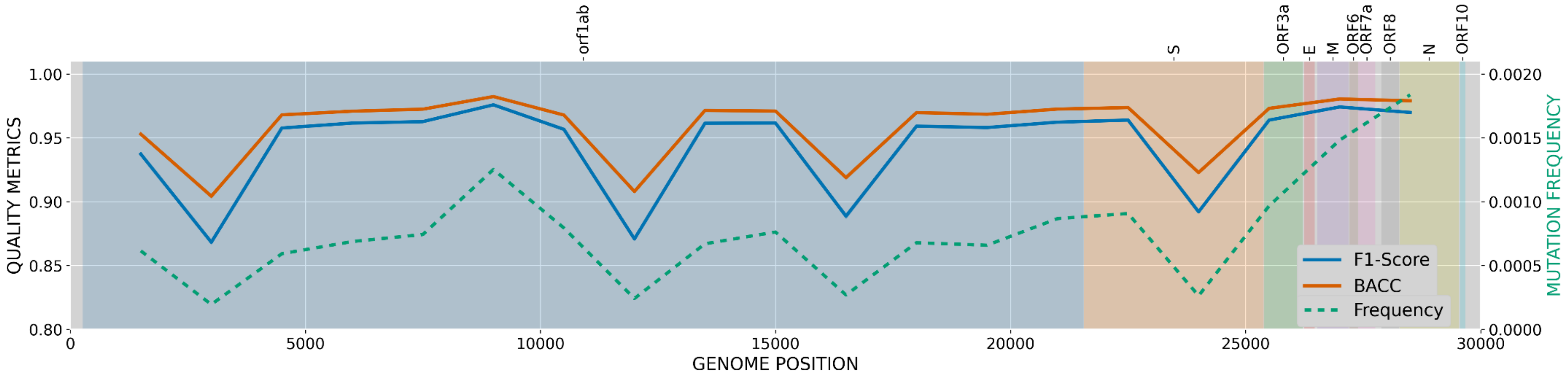

Fig. S1. More imputation performance metrics (F1 and BACC) based on the position of a missing 3kb window along the SARS-CoV-2 genome.

Left y-axis values represent mutation frequencies (dashed green line). SARS-CoV-2 protein regions are represented by colored background and names specified at the top.

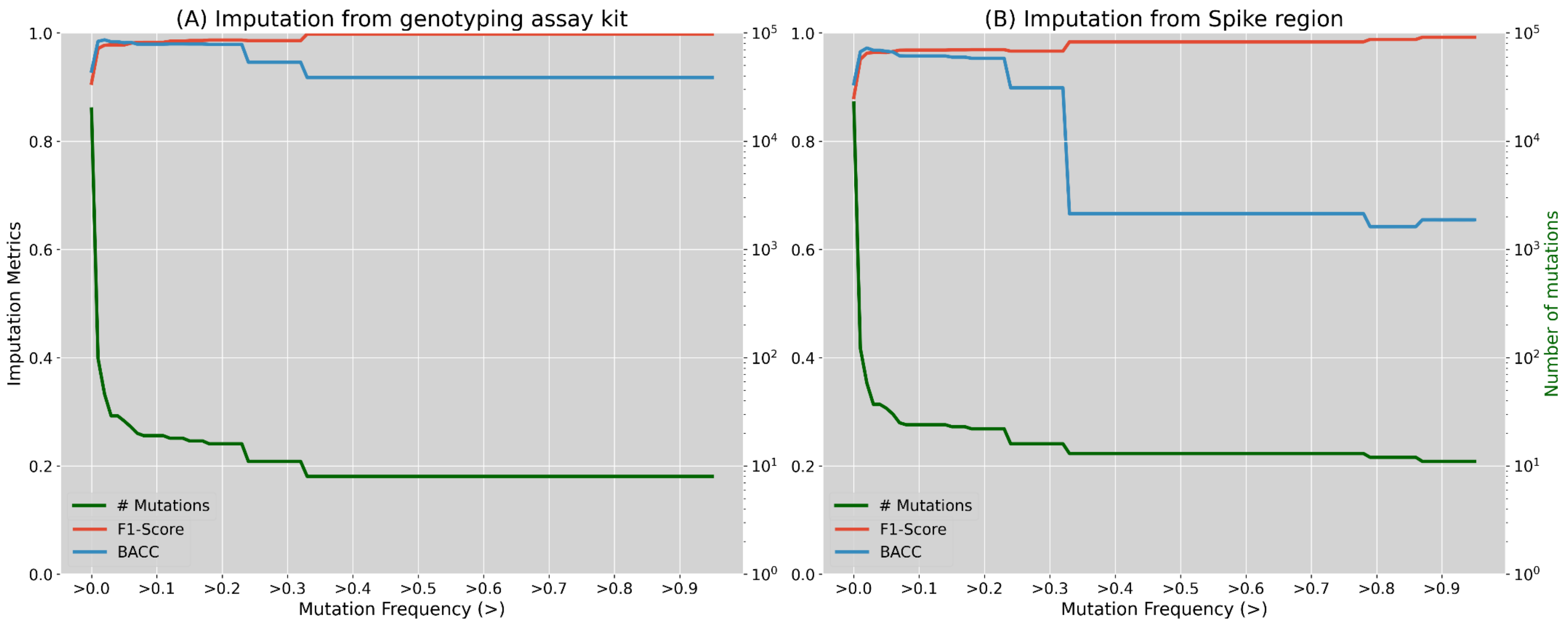

Fig. S2. Supplementary imputation performance metrics (BACC and F1) calculated depending on imputed variant frequencies. (A) Imputation quality when imputing from the genotyping array positions; (B) Imputation quality when imputing from spike protein positions. Left Y axis (green) represents the number of mutations for those frequency threshold (log scale)

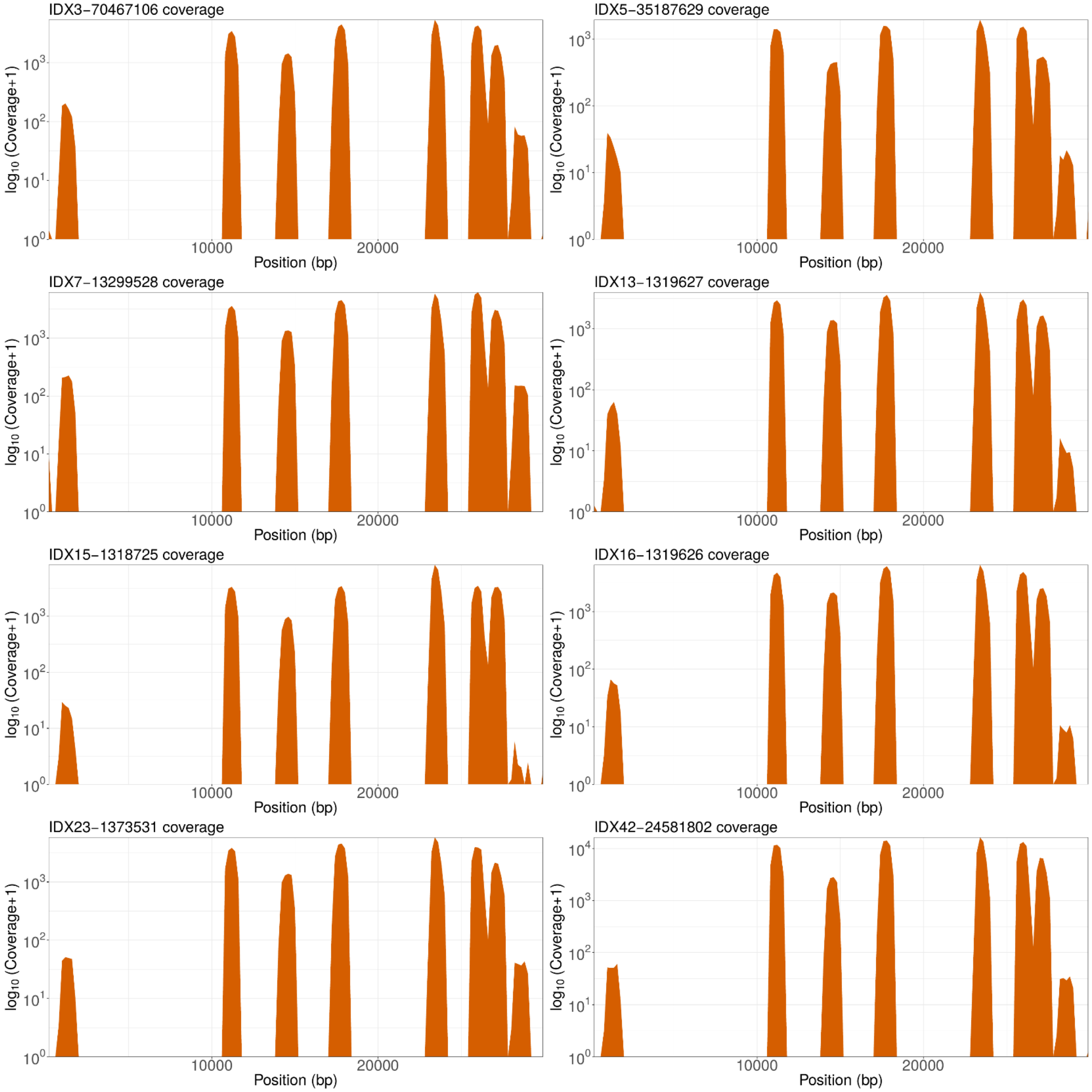

Fig S3. Coverage distribution from genotyping array in the eight samples studied.

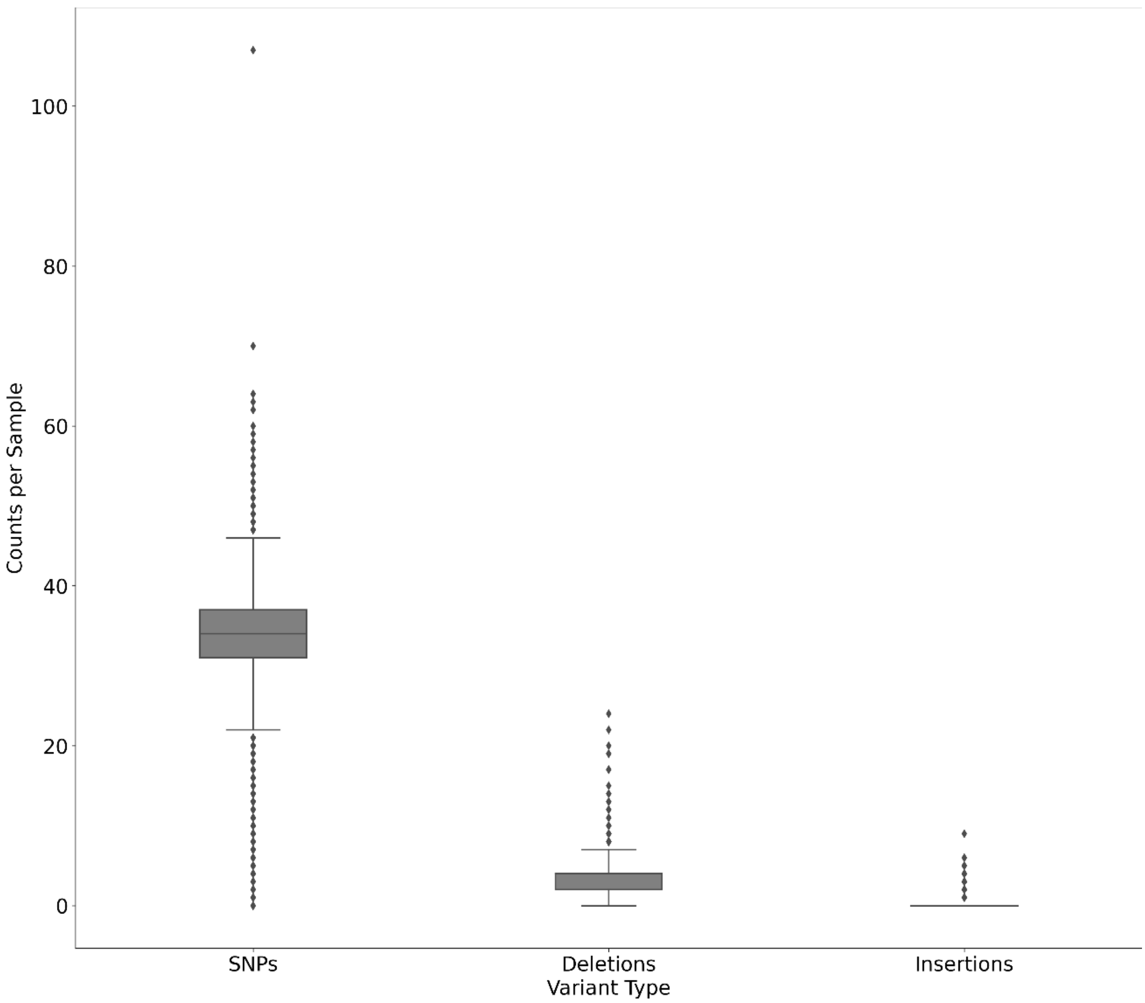

Fig. S4. Frequencies of the different types of mutations (SNVs, insertions and deletions per SARS-CoV-2 genome.
