# Supplementary material for: Highly accurate whole-genome imputation of SARS-CoV-2 from partial or low-quality sequences
Source: Gigascience. 2021 Dec 2;10(12):giab078. doi: 10.1093/gigascience/giab078 (PMC8643610; doi:10.1093/gigascience/giab078)
Supplement: giab078_Supplemental_Files [file giab078_supplemental_files.zip › gisaid_hcov-19_acknowledgement_table_2021_04_27_10.pdf]

We gratefully acknowledge the following Authors from the Originating laboratories responsible for obtaining the specimens, as well as the Submitting laboratories where the genome data were generated and shared via GISAID, on which this research is based.

All Submitters of data may be contacted directly via www.gisaid.org

Authors are sorted alphabetically.

Accession ID	Originating Laboratory	Submitting Laboratory	Authors
EPI_ISL_632310	1-Laboratory of Microbiology, National Reference Lab, Charles Nicolle Hospital; 2-University of Tunis ElManar, Faculty of Medicine of Tunis, LR99ES09, Tunis, Tunisia	1-Clinical and Experimental Pharmacology Lab, LR16SP02, National Center of Pharmacovigilance, University of Tunis El Manar, Tunis, Tunisia. 2-Neurodegenerative diseases and psychiatric troubles, LR18SP03, Razi Hospital, University of Tunis El Manar, Tunis, Tunisia. 3- Ministry of Health, National Observatory of New and Emerging Diseases, 1006, Tunis, Tunisia	Alia Ben Kahla; Gaies Emna; Ilhem Boutiba-Ben Boubaker; Imen Kacem; Imen Mkada; Jalila Ben Khelil; Maher Kharrat; Mouna Ben Sassi; Mouna Safer; Nissaf Ben Alaya; Riadh Daghfous; Riadh Gouider.; Salma Abid; Sameh Trabelsi; Sana Ferjani; Soumaya Rammeh
EPI_ISL_634977, EPI_ISL_635061, EPI_ISL_683329, EPI_ISL_699655 to 699657, EPI_ISL_707697 to 707700, EPI_ISL_707791 to 707793, EPI_ISL_733499 to 733500, EPI_ISL_763065, EPI_ISL_763067			
see above	1-Laboratory of Microbiology, National Reference Lab, Charles Nicolle Hospital; 2-University of Tunis ElManar, Faculty of Medicine of Tunis, LR99ES09, Tunis, Tunisia	1-Clinical and Experimental Pharmacology Lab, LR16SP02, National Center of Pharmacovigilance, University of Tunis El Manar, Tunis, Tunisia. 2-Neurodegenerative diseases and psychiatric troubles, LR18SP03, Razi Hospital, University of Tunis El Manar, Tunis, Tunisia. 3- Ministry of Health, National Observatory of New and Emerging Diseases, 1006, Tunis, Tunisia	Alia Ben Kahla; Asma Ferjani; Awatef El MOussi; Awatef El Moussi; Gaies Emna; Guedi Ali Barreh; Guedi Berrabeh; Habiba Ben Romdhane; Hanen El Jebari; Hanen ElJebari; Ilhem Boutiba-Ben Boubaker; Imen Kacem; Imen Mkada; Ines Mdini; Jalila Ben Khelil; Maher Kharrat; Mouna Ben Sassi; Mouna Safer; Nissaf Ben Alaya; Riadh Daghfous; Riadh Gouider.; Rouaa Ben Othman; Salma Abid; Salwa Mrabet; Sameh Trabelsi; Sana Ferjani; Sarra Chamman; Souissi Amira; Soumaya Rammeh; Zaineb Hamzaoui
EPI_ISL_451971 to 451987	1. ViroGenetics - BSL3 Laboratory of Virology, Maopolska Centre of Biotechnology, Jagiellonian University; 2. II Department of Internal Medicine, Faculty of Medicine, Jagiellonian University Medical College; 3. DIAGNOSTYKA Ltd.	1. ViroGenetics - BSL3 Laboratory of Virology, Maopolska Centre of Biotechnology, Jagiellonian University; 2. II Department of Internal Medicine, Faculty of Medicine, Jagiellonian University Medical College.	Jakub Swadba; Krzysztof Pyr; Marcin Surmiak; Marek Sanak; Marta Rogalska-Kupiec; Monika Gsecka-Czapla; Pawe P abaj; Wojciech Branicki
EPI_ISL_455440 to 455453, EPI_ISL_492066 to 492073	1. ViroGenetics - BSL3 Laboratory of Virology, Maopolska Centre of Biotechnology, Jagiellonian University; 2. II Department of Internal Medicine, Faculty of Medicine, Jagiellonian University Medical College; 3. Narodowy Instytut Zdrowia Publicznego - Pastwowy Zakad Higieny (NIZP-PZH)	1. ViroGenetics - BSL3 Laboratory of Virology, Maopolska Centre of Biotechnology, Jagiellonian University; 2. II Department of Internal Medicine, Faculty of Medicine, Jagiellonian University Medical College; 3. Narodowy Instytut Zdrowia Publicznego - Pastwowy Zakad Higieny (NIZP-PZH).	Agnieszka Koakowska-Kulesza; Aleksandra A. Zasada; Aleksandra Milewska; Ewelina Hallman-Szeliska; Katarzyna Owczarek; Katarzyna Pancer; Katarzyna Zacharczuk; Krzysztof Pyr; Magdalena Rzeczkowska; Marek Sanak; Natalia Wolaniuk; Pawe P abaj; Tomasz Wokowicz; Wojciech Branicki
EPI_ISL_640078, EPI_ISL_640107, EPI_ISL_640115, EPI_ISL_640127, EPI_ISL_700446, EPI_ISL_700555, EPI_ISL_1040736 to 1040737, EPI_ISL_1040741, EPI_ISL_1040743 to 1040744			
see above	2 Military Hospital wc MAA	NHLS/UCT	Arash Iranzadeh; Bruna Galvao; Carolyn Williamson; Deelan Doolabh; Diana Hardie; Innocent Mudau; Kruger Marais; Lynn Tyers; Marvin Hsiao; Stephen Korsman
EPI_ISL_450444	20 Dongda Street, Fengtai District, Beijing, Beijing 100071, China	Dept. OPA, Beijing Institute of Microbiology and Epidemiology	Fan, H.; Fang; Gong; J.H.; L.Q. and Liu, W.; Qi; R.Z.; X.A.; Zhang; Zheng, K.; Zheng, W.
EPI_ISL_513348 to 513349, EPI_ISL_513402, EPI_ISL_513413, EPI_ISL_526186, EPI_ISL_593646 to 593648, EPI_ISL_767931	4Cyte Pathology	NSW Health Pathology - Institute of Clinical Pathology and Medical Research; Westmead Hospital; University of Sydney	CIDM-PH et al.
EPI_ISL_729416 to 729459, EPI_ISL_729461 to 729463, EPI_ISL_729465, EPI_ISL_729498, EPI_ISL_729500 to 729513, EPI_ISL_729515 to 729528, EPI_ISL_729535 to 729547, EPI_ISL_729549 to 729552, EPI_ISL_729562 to 729595, EPI_ISL_729608 to 729734, EPI_ISL_849819, EPI_ISL_869086 to 869117, EPI_ISL_909745 to 909746, EPI_ISL_909748 to 909749			
see above	A. Krumbholz, Labor Dr. Krause und Kollegen MVZ GmbH, Kiel	Charité Universitätsmedizin Berlin, Institut für Virologie	Barbara Mühlemann; Christian Drosten; Julia Schneider; Julia Tesch; Jörn Beheim-Schwarzbach; Talitha Veith; Terry Jones; Tobias Bleicker; Victor M Corman
EPI_ISL_766695, EPI_ISL_766698 to 766699, EPI_ISL_766708, EPI_ISL_766715 to 766723	A05 Biomedicum	The Public Health Agency of Sweden	Department of Microbiology; The Public Health Agency of Sweden
EPI_ISL_1008401 to 1008406, EPI_ISL_1008409 to 1008411	A05 Diagnostics	The Public Health Agency of Sweden	Anna Risberg; Anna-Malin Linde; Carlo Berg; Karin Tegmark-Wisell; Maria Lind Karlberg; Mattias Haukland; Mia Brytting; Noura Walai; Oskar Karlsson Lindsjo; Petra Edquist; Petra Holmstrom; Reza Advani; Sofia Stamouli
EPI_ISL_1008534 to 1008536, EPI_ISL_1008539	A05 diagnostics	The Public Health Agency of Sweden	Anna Risberg; Anna-Malin Linde; Carlo Berg; Karin Tegmark-Wisell; Maria Lind Karlberg; Mattias Haukland; Mia Brytting; Noura Walai; Oskar Karlsson Lindsjo; Petra Edquist; Petra Holmstrom; Reza Advani; Sofia Stamouli
EPI_ISL_1008597	A23 Lab AB	The Public Health Agency of Sweden	Anna Risberg; Anna-Malin Linde; Carlo Berg; Karin Tegmark-Wisell; Maria Lind Karlberg; Mattias Haukland; Mia Brytting; Noura Walai; Oskar Karlsson Lindsjo; Petra Edquist; Petra Holmstrom; Reza Advani; Sofia Stamouli
EPI_ISL_775557	ABC	The Public Health Agency of Sweden	Department of Microbiology; The Public Health Agency of Sweden
EPI_ISL_455042 to 455043, EPI_ISL_455050, EPI_ISL_455074	ACT Pathology	NSW Health Pathology - Institute of Clinical Pathology and Medical Research; Westmead Hospital; University of Sydney	CIDM-PH et al.
EPI_ISL_498468, EPI_ISL_498472 to 498473, EPI_ISL_498477 to 498478, EPI_ISL_498480, EPI_ISL_498483, EPI_ISL_498485 to 498486, EPI_ISL_498489 to 498503, EPI_ISL_498505 to 498506, EPI_ISL_498510 to 498515, EPI_ISL_498518, EPI_ISL_498520, EPI_ISL_498523, EPI_ISL_498525 to 498526, EPI_ISL_498529, EPI_ISL_498531, EPI_ISL_498537, EPI_ISL_498540, EPI_ISL_498542 to 498548, EPI_ISL_735503			
see above	ACT Pathology	Schwessinger Lab	Ashley Jones; Benjamin Schwessinger; Craig Kennedy; Karina Kennedy; Kevin Murray; Megan McDonald; Ming-Dao Chia; Robert Lanfear; Robyn N Hall
EPI_ISL_427711, EPI_ISL_427717 to 427719, EPI_ISL_427721	ACT Pathology, The Canberra Hospital	NSW Health Pathology - Institute of Clinical Pathology and Medical Research; Westmead Hospital; University of Sydney	Arnott A; Bachmann N; Basile K; Byun R; Carter I; Chang S; Chen SC; Draper J; Dwyer DE for the 2019-nCoV Study Group; Eden JS; Gall M; Gray K; Holmes EC; Kok J; Lam C; Maddocks S; O'Sullivan MV; Propenko M; Rockett R; Sadsad R; Sim E; Sintchenko V; Sorrell T; Timms V
EPI_ISL_451540, EPI_ISL_451588, EPI_ISL_451590 to 451593, EPI_ISL_451595 to 451596, EPI_ISL_451598, EPI_ISL_451612	ACT pathology	NSW Health Pathology - Institute of Clinical Pathology and Medical Research; Westmead Hospital; University of Sydney	CIDM-PH et al.
EPI_ISL_1007605	ADMED	Laboratory of genomics and metagenomics, Institute of Microbiology, University Hospital Centre and University of Lausanne, Switzerland	Claire Bertelli; Damien Jacot; Gilbert Greub; Sébastien Aeby; Trestan Pillonel
EPI_ISL_934989 to 934991	ADMED Microbiologie	Genomics and Transcriptomics, Philip Morris International	David Bornand; Emmanuel Guedj; Manuel Peitsch; Marie-Lise Tritten; Maxime Berthouzoz; Mehdi Auberson; Nicolas Sierro; Nikolai Ivanov; Reto Lienhard; Rémi Dulize
EPI_ISL_602622, EPI_ISL_602624 to 602630	AHRI-Sigal	KRISP, KZN Research Innovation and Sequencing Platform	Cele S; Gazy I; Giandhari J; Karim F; Pillay S; Sigl A; Sigla; Tegally H; Wilkinson E; de Oliveira T
EPI_ISL_768758 to 768797, EPI_ISL_768833	AIID	Irish Coronavirus Sequencing Consortium - National Virus	Alejandro Abner Garcia Leon; Gabriel Gonzalez; Michael Carr; Patrick Mallon

		Reference Laboratory	
EPI_ISL_778823, EPI_ISL_778841, EPI_ISL_871799 to 871803, EPI_ISL_918436 to 918439, EPI_ISL_960998 to 961000	AIID	Irish Coronavirus Sequencing Consortium-Teagasc Grange	Aljandro Abner Garcia Leon; Calum Walsh; Fiona Crispie; Gabriel Gonzalez; John Kenny; Matthew McCabe; Michael Carr; Patrick Mallon; Paul Cotter
EPI_ISL_751592 to 751593, EPI_ISL_751597 to 751598, EPI_ISL_751694, EPI_ISL_751793	AK State Public Health Lab, State Health Department	Genomics and Discovery, Respiratory Viruses Branch, Division of Viral Diseases, Centers for Disease Control and Prevention	Anna Montmayeur; Anna Uehara; Clinton R. Paden; Haibin Wang; Jing Zhang; Justin Lee; Krista Queen; Mili Sheth; Peter W. Cook; Rachel Marine; Suxiang Tong; Yan Li; Ying Tao
EPI_ISL_751647, EPI_ISL_751668, EPI_ISL_751753, EPI_ISL_751755 to 751756, EPI_ISL_751791 to 751792	AL Dept. of Public Health Bureau of Clinical Laboratories	Genomics and Discovery, Respiratory Viruses Branch, Division of Viral Diseases, Centers for Disease Control and Prevention	Anna Montmayeur; Anna Uehara; Clinton R. Paden; Haibin Wang; Jing Zhang; Justin Lee; Krista Queen; Mili Sheth; Peter W. Cook; Rachel Marine; Suxiang Tong; Yan Li; Ying Tao
EPI_ISL_471546	AMA DR Jose Soares Hungria	Instituto Adolfo Lutz, Interdiciplinary Procedures Center, Strategic Laboratory	Claudia Regina Gonçalves; Claudio Tavares Sacchi; Erica Valessa Ramos Gomes
EPI_ISL_523989	AMA Jardim Joamar	Instituto Adolfo Lutz, Interdiciplinary Procedures Center, Strategic Laboratory	Claudia Regina Gonçalves; Claudio Tavares Sacchi; Erica Valessa Ramos Gomes
EPI_ISL_523990	AMA Jardim Peri	Instituto Adolfo Lutz, Interdiciplinary Procedures Center, Strategic Laboratory	Claudia Regina Gonçalves; Claudio Tavares Sacchi; Erica Valessa Ramos Gomes
EPI_ISL_467432 to 467435, EPI_ISL_467449 to 467451, EPI_ISL_467453 to 467457, EPI_ISL_467460 to 467462, EPI_ISL_467465 to 467474	AMPATH-DBN	KRISP, KZN Research Innovation and Sequencing Platform	Chimukangara B; Giandhari J; Khan S; Lessells R; Mdlalose K; Pillay S; Tegally H; Wilkinson E; York D; de Oliveira T
EPI_ISL_458150	ANOUAL	ANOUAL	Azami Nawfel; Benhida Rachid; Chenaoui Mohamed; El Aliani Aissam; El Ansari Fatima Zahra; Fekkak Jamal; Jouali Farah; Kasmi Yassine; Kitane Driss Lahlou; Loukman Salma; Marchoudi Nabila
EPI_ISL_751574, EPI_ISL_751652, EPI_ISL_751697 to 751699, EPI_ISL_751723, EPI_ISL_751731, EPI_ISL_751760, EPI_ISL_751764 to 751765			
see above	AR Dept. of Health-Public Health Lab	Genomics and Discovery, Respiratory Viruses Branch, Division of Viral Diseases, Centers for Disease Control and Prevention	Anna Montmayeur; Anna Uehara; Clinton R. Paden; Haibin Wang; Jing Zhang; Justin Lee; Krista Queen; Mili Sheth; Peter W. Cook; Rachel Marine; Suxiang Tong; Yan Li; Ying Tao
EPI_ISL_450800, EPI_ISL_509663 to 509668, EPI_ISL_509670 to 509685, EPI_ISL_527632 to 527657, EPI_ISL_535650 to 535661, EPI_ISL_576196 to 576197	AR Dept. of Health-Public Health Lab	Pathogen Discovery, Respiratory Viruses Branch, Division of Viral Diseases, Centers for Disease Control and Prevention	Anna Montmayeur; Anna Uehara; Bettina Bankamp; Brian Lynch; Clinton Paden; Clinton R. Paden; Haibin Wang; Jing Zhang; Krista Queen; Peter Cook; Rachel Marine; Suxiang Tong; Yan Li; Ying Tao; Zachary Weiner
EPI_ISL_682235, EPI_ISL_682258 to 682259	AREA DE SALUD ALAJUELA NORTE - CLINICA DR. MARCIAL RODRIGUEZ	Inciensa, Instituto Costarricense de Investigación y Enseñanza en Nutrición y Salud	Adriana Godinez; Adriana Godinez & Melany Calderon; Claudio Soto-Garita; Estela Cordero; Francisco Duarte; Hebleen Porras; Melany Calderon & Mariel López
EPI_ISL_682260	AREA DE SALUD CATEDRAL NORESTE	Inciensa, Instituto Costarricense de Investigación y Enseñanza en Nutrición y Salud	Adriana Godinez; Claudio Soto-Garita; Estela Cordero; Francisco Duarte; Hebleen Porras; Melany Calderon & Mariel López
EPI_ISL_682247 to 682248	AREA DE SALUD CIUDAD QUESADA	Inciensa, Instituto Costarricense de Investigación y Enseñanza en Nutrición y Salud	Adriana Godinez; Adriana Godinez & Melany Calderon; Claudio Soto-Garita; Estela Cordero; Francisco Duarte; Hebleen Porras; Melany Calderon & Mariel López
EPI_ISL_682237, EPI_ISL_682256	AREA DE SALUD CORREDORES	Inciensa, Instituto Costarricense de Investigación y Enseñanza en Nutrición y Salud	Adriana Godinez; Adriana Godinez & Melany Calderon; Claudio Soto-Garita; Estela Cordero; Francisco Duarte; Hebleen Porras; Melany Calderon & Mariel López
EPI_ISL_682250, EPI_ISL_682252	AREA DE SALUD ESCAZU (COOPESANA)	Inciensa, Instituto Costarricense de Investigación y Enseñanza en Nutrición y Salud	Adriana Godinez & Melany Calderon; Claudio Soto-Garita; Estela Cordero; Francisco Duarte; Hebleen Porras
EPI_ISL_682238, EPI_ISL_682244	AREA DE SALUD LA CRUZ	Inciensa, Instituto Costarricense de Investigación y Enseñanza en Nutrición y Salud	Adriana Godinez; Adriana Godinez & Melany Calderon; Claudio Soto-Garita; Estela Cordero; Francisco Duarte; Hebleen Porras; Melany Calderon & Mariel López
EPI_ISL_682249	AREA DE SALUD LA UNION	Inciensa, Instituto Costarricense de Investigación y Enseñanza en Nutrición y Salud	Adriana Godinez; Claudio Soto-Garita; Estela Cordero; Francisco Duarte; Hebleen Porras; Melany Calderon & Mariel López
EPI_ISL_682236	AREA DE SALUD LOS CHILES	Inciensa, Instituto Costarricense de Investigación y Enseñanza en Nutrición y Salud	Adriana Godinez; Claudio Soto-Garita; Estela Cordero; Francisco Duarte; Hebleen Porras; Melany Calderon & Mariel López
EPI_ISL_418000 to 418001, EPI_ISL_418004	ARS Algarve - Laboratório Laura Ayres	Instituto Nacional de Saude (INSA)	Guiomar et al
EPI_ISL_911548	ARUP laboratories	ARUP Laboratories	Barker AP; Hillyard DR; Hymas W; Mallory MA; Pyne MT; Shakir SM; Simmon KE; Slechta ES
EPI_ISL_445326, EPI_ISL_445367	ASISTENCIA PUBLICA DR.ALEJANDRO DEL RIO	Instituto de Salud Publica de Chile	Alejandra Acevedo; Andrés E Castillo; Bárbara Parra; Carolina Tambley; Gabriel Leal; Jaime Lagos; Jorge Fernandez; Loredana Arata; Patricia Bustos; Paz Tapia; Rodrigo Fasce; Winston Andrade
EPI_ISL_1036198	ASL AVEZZANO-SULMONA-L'AQUILA DIP PREV SERV IGIENE E SAN PUBBLICA	Istituto Zooprofilattico Sperimentale dell'Abruzzo e Molise "G. Caporale"	Ancora M; Calistri P; Cammà C; Curini V; Di Domenico M; Di Pasquale A; Lorusso A; Mangone I; Marcacci M; Puglia I; Rinaldi A; Savini G; Scialabba S
EPI_ISL_542098 to 542226, EPI_ISL_542228 to 542277, EPI_ISL_542400 to 542443	ASST GOM Niguarda	Dep. Of Oncology and Hemato-Oncology University of Milan	Antonio Piralla; Carlo Federico Perno; Chiara Vismara; Claudia Alteri; Elisa Matarazzo; Fausto Baldanti; Federica Giardina; Federica Novazzi; Luna Colagrossi; Maria Antonello; Massimo Puoti; Monica Tallarita; Oscar Massimiliano Epis; Roberto Fumagalli; Silvia Renica; Stefano Gaiarsa; Valentino Costabile; Valeria Cento
EPI_ISL_891249, EPI_ISL_910013	AZ Delta	AZ Delta	Brigitte Maes; Dieter De Smet; Geert Martens
EPI_ISL_420784	AZ Department of Health Services	Pathogen Discovery, Respiratory Viruses Branch, Division of Viral Diseases, Centers for Disease Control and Prevention	Alison S. Laufer Halpin; Anne Uehara; Christopher A. Elkins; Clinton R. Paden; Haibin Wang; Jasmine Padilla; Jing Zhang; Justin Lee; Krista Queen; Mary S. Keckler; Rachel Marine; Suxiang Tong; Yan Li; Ying Tao
EPI_ISL_981378, EPI_ISL_1018110, EPI_ISL_1018112, EPI_ISL_1018114, EPI_ISL_1018116, EPI_ISL_1020127, EPI_ISL_1020129 to 1020130, EPI_ISL_1020136 to 1020137, EPI_ISL_1020142, EPI_ISL_1020144, EPI_ISL_1020147, EPI_ISL_1020150, EPI_ISL_1020153			
see above	AZ Klina	AZ Klina	Dr. C. Vael
EPI_ISL_424848 to 424849, EPI_ISL_452118	AZ SPHL, Arizona Department of Health Services	Pathogen Discovery, Respiratory Viruses Branch, Division of Viral Diseases, Centers for Disease Control and Prevention	Alison S. Laufer Halpin; Anna Montmayeur; Anna Uehara; Christopher A. Elkins; Clinton R. Paden; Haibin Wang; Jing Zhang; Krista Queen; Mary S. Keckler; Rachel Marine; Suxiang Tong; Yan Li; Ying Tao; Zachary Weiner
EPI_ISL_426485, EPI_ISL_426512 to 426519, EPI_ISL_426527 to 426531, EPI_ISL_426537 to 426538, EPI_ISL_426540 to 426555, EPI_ISL_426558 to 426569, EPI_ISL_427271 to 427272, EPI_ISL_694049, EPI_ISL_694053 to 694062, EPI_ISL_694064, EPI_ISL_694066 to 694075, EPI_ISL_694077 to 694079, EPI_ISL_694082 to 694090, EPI_ISL_694093 to 694107, EPI_ISL_694112 to 694113, EPI_ISL_694115, EPI_ISL_694117, EPI_ISL_694120, EPI_ISL_694124 to 694128, EPI_ISL_694132, EPI_ISL_694134, EPI_ISL_694149, EPI_ISL_694151 to 694152, EPI_ISL_694155 to 694157, EPI_ISL_694159, EPI_ISL_694162, EPI_ISL_694166 to 694168, EPI_ISL_694173 to 694174, EPI_ISL_694177, EPI_ISL_694186 to 694187, EPI_ISL_694189, EPI_ISL_694198 to 694200, EPI_ISL_694205, EPI_ISL_694207 to 694208, EPI_ISL_694210 to 694212, EPI_ISL_694218, EPI_ISL_694249 to 694250, EPI_ISL_694252, EPI_ISL_694254 to 694256, EPI_ISL_694258, EPI_ISL_694269 to 694270, EPI_ISL_694272, EPI_ISL_694276 to 694277, EPI_ISL_694280, EPI_ISL_694283 to 694285, EPI_ISL_694289 to 694292, EPI_ISL_694294, EPI_ISL_694297 to 694299, EPI_ISL_694301, EPI_ISL_694303 to 694305, EPI_ISL_694307 to 694309, EPI_ISL_694311 to 694312, EPI_ISL_694314 to 694315, EPI_ISL_694406, EPI_ISL_694422 to 694423, EPI_ISL_694459, EPI_ISL_694472 to 694477, EPI_ISL_694480, EPI_ISL_694484, EPI_ISL_694487 to 694488, EPI_ISL_694490 to 694492, EPI_ISL_694494, EPI_ISL_694497, EPI_ISL_694499, EPI_ISL_694503, EPI_ISL_694509, EPI_ISL_694523, EPI_ISL_694530 to 694531, EPI_ISL_694533, EPI_ISL_694539, EPI_ISL_694541 to 694543, EPI_ISL_694545, EPI_ISL_694547 to 694548, EPI_ISL_694550 to 694554, EPI_ISL_694556, EPI_ISL_694558, EPI_ISL_694560, EPI_ISL_694562, EPI_ISL_694567, EPI_ISL_694569, EPI_ISL_694572, EPI_ISL_694574, EPI_ISL_694576 to 694582, EPI_ISL_694585, EPI_ISL_694588 to 694589, EPI_ISL_694591 to 694592, EPI_ISL_694595 to 694596, EPI_ISL_694599, EPI_ISL_694724, EPI_ISL_694727, EPI_ISL_694732, EPI_ISL_694738, EPI_ISL_694742 to 694744, EPI_ISL_694746 to 694748, EPI_ISL_694750, EPI_ISL_694753, EPI_ISL_694755, EPI_ISL_694758, EPI_ISL_694761, EPI_ISL_694763 to 694765, EPI_ISL_694769, EPI_ISL_694771 to 694773, EPI_ISL_694775, EPI_ISL_694781 to 694782, EPI_ISL_694784, EPI_ISL_694786, EPI_ISL_694788, EPI_ISL_694796, EPI_ISL_694885, EPI_ISL_694887 to 694893, EPI_ISL_694895, EPI_ISL_694897 to 694898, EPI_ISL_694901, EPI_ISL_694906 to 694909, EPI_ISL_694911 to 694912, EPI_ISL_694914, EPI_ISL_694916 to 694917, EPI_ISL_694923 to 694925, EPI_ISL_694928, EPI_ISL_694930, EPI_ISL_694933 to 694934, EPI_ISL_694937, EPI_ISL_694940 to 694942, EPI_ISL_694946 to 694949, EPI_ISL_695103 to 695106, EPI_ISL_695109 to 695114, EPI_ISL_695116 to 695119, EPI_ISL_695121, EPI_ISL_695124, EPI_ISL_695127, EPI_ISL_695129 to 695139, EPI_ISL_695144 to 695145, EPI_ISL_695147 to 695148, EPI_ISL_695150 to 695152, EPI_ISL_695154 to 695155, EPI_ISL_695158 to 695161, EPI_ISL_695163, EPI_ISL_695165 to 695167, EPI_ISL_695169 to 695170, EPI_ISL_695172 to 695173, EPI_ISL_695177, EPI_ISL_695186, EPI_ISL_695205 to 695207, EPI_ISL_695209, EPI_ISL_695220 to 695221, EPI_ISL_695224, EPI_ISL_695328, EPI_ISL_695345, EPI_ISL_695351, EPI_ISL_695354 to 695355, EPI_ISL_695360 to 695362, EPI_ISL_695367 to 695368, EPI_ISL_695370, EPI_ISL_695397 to 695398,			

EPI_ISL_695401, EPI_ISL_695406, EPI_ISL_695408, EPI_ISL_695411 to 695412, EPI_ISL_695415, EPI_ISL_695419, EPI_ISL_695423, EPI_ISL_695426 to 695429, EPI_ISL_695431, EPI_ISL_695433 to 695435, EPI_ISL_695437 to 695438, EPI_ISL_695440, EPI_ISL_695442, EPI_ISL_695446, EPI_ISL_695451 to 695452, EPI_ISL_695454 to 695456, EPI_ISL_695458 to 695459, EPI_ISL_695462 to 695463, EPI_ISL_695465 to 695466, EPI_ISL_695470, EPI_ISL_695472 to 695473, EPI_ISL_695476, EPI_ISL_695478 to 695482, EPI_ISL_695484, EPI_ISL_695486, EPI_ISL_695488 to 695489, EPI_ISL_695491 to 695494, EPI_ISL_695496, EPI_ISL_695498, EPI_ISL_695500 to 695502, EPI_ISL_695506, EPI_ISL_695514 to 695516, EPI_ISL_695568, EPI_ISL_695571, EPI_ISL_695578 to 695579, EPI_ISL_695586, EPI_ISL_695588, EPI_ISL_695595, EPI_ISL_695598, EPI_ISL_695600, EPI_ISL_695608, EPI_ISL_695611 to 695612, EPI_ISL_695665, EPI_ISL_695667, EPI_ISL_695672, EPI_ISL_695685 to 695686, EPI_ISL_695689, EPI_ISL_695693, EPI_ISL_695696, EPI_ISL_695712 to 695715, EPI_ISL_695717, EPI_ISL_695720, EPI_ISL_695727 to 695728, EPI_ISL_695730 to 695731, EPI_ISL_695736, EPI_ISL_695740, EPI_ISL_695742, EPI_ISL_695744 to 695745, EPI_ISL_695749, EPI_ISL_695830			
see above	AZ SPHL, Arizona Department of Health Services	TGen North	Ashlyn Pfeiffer; Chris French; Darrin Lemmer; Dave Engelthaler; Hayley Yaglom; Jolene Bowers; Megan Folkerts; The Arizona COVID Genomics Union (ACGU)
EPI_ISL_911256 to 911257, EPI_ISL_911259, EPI_ISL_911262 to 911263, EPI_ISL_911266 to 911267, EPI_ISL_911270, EPI_ISL_911282 to 911283, EPI_ISL_911388 to 911389, EPI_ISL_911393, EPI_ISL_911430 to 911433, EPI_ISL_913498, EPI_ISL_913509 to 913510, EPI_ISL_913594, EPI_ISL_913596 to 913597, EPI_ISL_913599 to 913601, EPI_ISL_954755 to 954762, EPI_ISL_954771, EPI_ISL_954777, EPI_ISL_954779, EPI_ISL_954815, EPI_ISL_961183 to 961185, EPI_ISL_961189 to 961191, EPI_ISL_961197 to 961200, EPI_ISL_978791, EPI_ISL_978793 to 978794, EPI_ISL_978797 to 978799, EPI_ISL_978801, EPI_ISL_978806 to 978810, EPI_ISL_978813 to 978816, EPI_ISL_978818, EPI_ISL_978820 to 978823, EPI_ISL_978826, EPI_ISL_978829 to 978830, EPI_ISL_978837 to 978841, EPI_ISL_978843, EPI_ISL_978845 to 978848, EPI_ISL_978850, EPI_ISL_978852, EPI_ISL_978854 to 978855, EPI_ISL_978857 to 978858, EPI_ISL_978860 to 978864, EPI_ISL_978866, EPI_ISL_978869 to 978871, EPI_ISL_978873 to 978874, EPI_ISL_978877 to 978878			
see above	AZDelta	AZDelta	Dieter De Smet; Geert Martens
EPI_ISL_735501 to 735502	Abdul Malek Ukil Medical College, Noakhali	Central Biological Research Laboratory and Department of Biochemistry and Molecular Biology	H. M. Abdullah Al Masud; Imam Hossen; Md. Arif Hossain; Md. Imranul Hoq; Md. Khondakar Raziur Rahman; Md. Omer Faruq; Mohammad Omar Faruque; Robiul Hasan Bhuiyan; Sajib Rudra; Shanta Paul
EPI_ISL_517613, EPI_ISL_517615 to 517616, EPI_ISL_517620 to 517631, EPI_ISL_517633, EPI_ISL_517636 to 517641, EPI_ISL_517644 to 517648, EPI_ISL_517650 to 517654, EPI_ISL_517657 to 517659, EPI_ISL_518799, EPI_ISL_518801 to 518810, EPI_ISL_518812, EPI_ISL_518814, EPI_ISL_518816 to 518817			
see above	Academic Hospital Paramaribo	Erasmus Medical Center	Bas Oude Munnink; Dion Gajadin; Ed Ijzerman; Emmanuelle Munger; Gary Gummels; Ingrid Krishnadath; Lycke Woittiez; Marion Koopmans; Mireille Van de Veer; Princes Wongsowidjojo; Radjesh Ori; Rohma Banwari; Stephen Vreden
EPI_ISL_476135	Achima Care Fristadens VC	The Public Health Agency of Sweden	Anna Risberg; Anna-Malin Linde; Karin Tegmark-Wisell; Maria Lind Karlberg; Mattias Haukland; Mia Brytting; Olov Svartstrom; Oskar Karlsson Lindsjo; Petra Edquist; Reza Advani; Sandra Broddesson
EPI_ISL_509416	Acibadem Labcell Cellular Therapy Laboratory	Acibadem Mehmet Ali Aydinlar University School of Medicine, Medical Genetics Department	Bulut Yurtsever; Cihan Tastan; Derya Dilek Kancagi; Ercument Ovali; Gozde Sir Karakus; Gunseli Bayram Akcapinar; Ilayda Sahin; Ozden Hatirnaz Ng; Ozkan Ozdemir; Sezer Akyoney; Ugur Ozbek
EPI_ISL_458083	Adi Husada Undaan Hospital	Institute of Tropical Disease, Universitas Airlangga	Aldise M Nastri; Gatot Soegiarto; Irawati Marga; Jezzy R Dewantari; Kazufumi Shimizu; Krisnoadi Rahardjo; Laksmi Wulandari; Maria I Lusida; Mitsuhiro Nishimura; Resti Yudhawati; Retno A Setyoningrum; Rima R Prasetya; Soetjipto; Yasuko Mori; Yohko K Shimizu
EPI_ISL_514253	Advanced Biotechnology Laboratory	Genomic Research Lab, BCSIR	Abu Sayeed Mohammad Mahmud; Barna Goswami; Eshrar Osman; Eunus Ali; Hossain Uddin Shekhar; Iffat Jahan; M. Aftab Uddin; Md. Ahashan Habib; Md. Bayejid Hosen; Md. Murshed Hasan Sarkar; Md. Saddam Hossain; Md. Salim Khan; Mohammad Samir Uzzaman; Salek Ahmed Sajib; Shahina Akter; Tanjina Akhter Banu; Utpal Chandra Ray
EPI_ISL_528934 to 528949	Agenzia di Tutela della Salute di Bergamo	Istituto Zooprofilattico Sperimentale dell'Abruzzo e Molise "G.Caporale"	Ancora M; Cammà C; Curini V; Di Domenico M; Di Pasquale A; Lorusso A; Mangone I; Marcacci M; Puglia I; Rinaldi A; Savini G.
EPI_ISL_445244	Akbiomed lab	Tejgaon College bmb lab	Md.Abdul kaium; Md.Easin Arafat
EPI_ISL_420136, EPI_ISL_420139, EPI_ISL_420312, EPI_ISL_500779 to 500780, EPI_ISL_500782 to 500783, EPI_ISL_500794 to 500796, EPI_ISL_549083 to 549084, EPI_ISL_549089 to 549091, EPI_ISL_549169 to 549171, EPI_ISL_590889 to 590892, EPI_ISL_590951 to 590952, EPI_ISL_635102, EPI_ISL_635194, EPI_ISL_668393, EPI_ISL_796665 to 796666, EPI_ISL_860210, EPI_ISL_860212, EPI_ISL_860259 to 860260, EPI_ISL_860277, EPI_ISL_906828 to 906829, EPI_ISL_962895, EPI_ISL_964257, EPI_ISL_964297, EPI_ISL_964303, EPI_ISL_964953 to 964954, EPI_ISL_1013472, EPI_ISL_1013513 to 1013514, EPI_ISL_1013516, EPI_ISL_1034311 to 1034312, EPI_ISL_1034314 to 1034315, EPI_ISL_1040146 to 1040147			
see above	Akershus University Hospital, Department for Microbiology and Infectious Disease Control	Norwegian Institute of Public Health, Department of Virology	Atiya R Ali; Engebretsen Serina Beate Atiya R Ali; Garcia Llorente Ignacio; Hilde Elshaug; Hilde Synnøve Vollan; Hilde Vollan; Ignacio Garcia Llorente; Kamilla Heddeland Instefjord; Karoline Bragstad; Kathrine Stene-Johansen; Marie Paulsen Madsen; Olav Hungnes; Rasmus Riis Kopperud; Serina B Engebretsen
EPI_ISL_480204	Akita City Public Health Center	Pathogen Genomics Center, National Institute of Infectious Diseases	Hajime Kamiya; Kentaro Itokawa; Koichi Ito; Makoto Kuroda; Masanori Hashino; Motoi Suzuki; Rina Tanaka; Tsuyoshi Sekizuka
EPI_ISL_747240	Al Islam Hospital	West Java Health Laboratory; School of Life Sciences and Technology, Institut Teknologi Bandung	Azzania Fibriani; Cut Nur Cinthia Alamanda; Ema Rahmawati; Isak Solihin; Karimatu Khoirunnisa; Miftahul Faridl; Rifky Waluyajati Rachman; Rini Robiani; Ryan Bayusantika Ristandi
EPI_ISL_739660 to 739661	Al-Quds Nutrition and Health Research Institute, Al-Quds University	Al-Quds Nutrition and Health Research Institute, Al-Quds University	Al-Jawabreh, A.; Ereqat, S.; Nasereddin, A.; Rishmawi, C.
EPI_ISL_649153, EPI_ISL_661272	Al-Quds Nutrition and Health Research Institute, Al-Quds University	Al-Quds Nutrition and Health Research Institute, Al-Quds University	A. and Al-Jawabreh, A.; Ereqat; Ereqat, S.; Nasereddin; Nasereddin, A.; S. and Al-Jawabreh, A.
EPI_ISL_509688	Alabama Department of Public Health Bureau of Clinical Laboratories	Pathogen Discovery, Respiratory Viruses Branch, Division of Viral Diseases, Centers for Disease Control and Prevention	Anna Uehara; Clinton Paden; Haibin Wang; Jing Zhang; Krista Queen; Suxiang Tong; Yan Li; Ying Tao
EPI_ISL_454607 to 454613, EPI_ISL_468358, EPI_ISL_468360 to 468361, EPI_ISL_468363 to 468365, EPI_ISL_468367 to 468368, EPI_ISL_468370, EPI_ISL_468372 to 468374, EPI_ISL_468377, EPI_ISL_468380, EPI_ISL_468382 to 468383, EPI_ISL_468385, EPI_ISL_468387, EPI_ISL_625500 to 625503, EPI_ISL_625506 to 625512, EPI_ISL_625514 to 625521, EPI_ISL_625523 to 625532, EPI_ISL_625534 to 625546, EPI_ISL_625611 to 625614, EPI_ISL_625616 to 625617, EPI_ISL_625619, EPI_ISL_625621 to 625622, EPI_ISL_672086, EPI_ISL_672113, EPI_ISL_672305 to 672353, EPI_ISL_672439 to 672476, EPI_ISL_738678, EPI_ISL_738711, EPI_ISL_738715, EPI_ISL_738724, EPI_ISL_738743 to 738745, EPI_ISL_738748 to 738749, EPI_ISL_738751, EPI_ISL_738753, EPI_ISL_738760, EPI_ISL_738766 to 738768, EPI_ISL_738773 to 738776, EPI_ISL_738781, EPI_ISL_738783, EPI_ISL_738788, EPI_ISL_738792 to 738793, EPI_ISL_738796 to 738797, EPI_ISL_738800, EPI_ISL_738802, EPI_ISL_738804, EPI_ISL_738813 to 738814, EPI_ISL_738817, EPI_ISL_738819, EPI_ISL_738821 to 738822, EPI_ISL_738825, EPI_ISL_738828, EPI_ISL_738831, EPI_ISL_738833, EPI_ISL_738835, EPI_ISL_738837 to 738838, EPI_ISL_738843, EPI_ISL_738846 to 738848, EPI_ISL_738858, EPI_ISL_738868, EPI_ISL_738876, EPI_ISL_738881, EPI_ISL_738886, EPI_ISL_738891 to 738892, EPI_ISL_738895 to 738896, EPI_ISL_738904, EPI_ISL_738914, EPI_ISL_738919, EPI_ISL_738925, EPI_ISL_738932, EPI_ISL_738934, EPI_ISL_738940, EPI_ISL_738945, EPI_ISL_738950, EPI_ISL_738957, EPI_ISL_738963, EPI_ISL_738974, EPI_ISL_738976, EPI_ISL_738982 to 738983, EPI_ISL_738986, EPI_ISL_738992, EPI_ISL_739003, EPI_ISL_739018, EPI_ISL_739023, EPI_ISL_739029, EPI_ISL_739033, EPI_ISL_739035, EPI_ISL_739046 to 739047, EPI_ISL_739049, EPI_ISL_739054, EPI_ISL_739056 to 739057, EPI_ISL_739059, EPI_ISL_739062, EPI_ISL_739064, EPI_ISL_739073, EPI_ISL_739075, EPI_ISL_739077, EPI_ISL_739082 to 739084, EPI_ISL_739088, EPI_ISL_739090, EPI_ISL_739093, EPI_ISL_739096, EPI_ISL_739100, EPI_ISL_739109 to 739111, EPI_ISL_739113, EPI_ISL_739115, EPI_ISL_739118, EPI_ISL_739120, EPI_ISL_739124, EPI_ISL_739127, EPI_ISL_739134 to 739137, EPI_ISL_739140, EPI_ISL_739144, EPI_ISL_739149 to 739150, EPI_ISL_739158, EPI_ISL_739160 to 739161, EPI_ISL_739163 to 739164, EPI_ISL_739171, EPI_ISL_739173 to 739174, EPI_ISL_739178 to 739179, EPI_ISL_739189 to 739190, EPI_ISL_739192, EPI_ISL_739196, EPI_ISL_739200 to 739202, EPI_ISL_739206 to 739209, EPI_ISL_739212 to 739213, EPI_ISL_739215, EPI_ISL_739217 to 739218, EPI_ISL_739220, EPI_ISL_739226, EPI_ISL_739229, EPI_ISL_739231, EPI_ISL_739233, EPI_ISL_739236, EPI_ISL_739241, EPI_ISL_739243 to 739244, EPI_ISL_739257, EPI_ISL_739261 to 739262, EPI_ISL_739268, EPI_ISL_739270, EPI_ISL_739280, EPI_ISL_739283, EPI_ISL_739285 to 739286, EPI_ISL_739288, EPI_ISL_739290, EPI_ISL_739294, EPI_ISL_739300, EPI_ISL_739302 to 739303, EPI_ISL_739306 to 739309, EPI_ISL_739312, EPI_ISL_739323 to 739324, EPI_ISL_739326, EPI_ISL_739328, EPI_ISL_739333, EPI_ISL_739337 to 739338, EPI_ISL_739340, EPI_ISL_739349, EPI_ISL_739351, EPI_ISL_739356, EPI_ISL_739358, EPI_ISL_739361, EPI_ISL_739368, EPI_ISL_739378, EPI_ISL_739383, EPI_ISL_739385, EPI_ISL_739387, EPI_ISL_739391, EPI_ISL_739395, EPI_ISL_739406 to 739408, EPI_ISL_739422, EPI_ISL_739424, EPI_ISL_739426 to 739427, EPI_ISL_739434 to 739435, EPI_ISL_739439, EPI_ISL_739444, EPI_ISL_739446, EPI_ISL_739448 to 739449, EPI_ISL_739454, EPI_ISL_739459, EPI_ISL_739463, EPI_ISL_739468, EPI_ISL_739470, EPI_ISL_739472 to 739473, EPI_ISL_739483 to 739484, EPI_ISL_739495, EPI_ISL_739503, EPI_ISL_739506 to 739507, EPI_ISL_739511, EPI_ISL_739515 to 739516, EPI_ISL_739518 to 739519, EPI_ISL_739522 to 739523, EPI_ISL_739526, EPI_ISL_739528, EPI_ISL_739535, EPI_ISL_739538, EPI_ISL_739541, EPI_ISL_739544, EPI_ISL_739551 to 739552, EPI_ISL_739555, EPI_ISL_739563, EPI_ISL_739565, EPI_ISL_739569, EPI_ISL_739572, EPI_ISL_739575 to 739576, EPI_ISL_739578, EPI_ISL_739583, EPI_ISL_739587, EPI_ISL_739590, EPI_ISL_739598, EPI_ISL_739600 to 739601, EPI_ISL_739604, EPI_ISL_739606, EPI_ISL_739610, EPI_ISL_739616, EPI_ISL_739619, EPI_ISL_739622 to 739623, EPI_ISL_739631, EPI_ISL_739634, EPI_ISL_739638, EPI_ISL_739640, EPI_ISL_739643 to 739644, EPI_ISL_739648, EPI_ISL_739652, EPI_ISL_739656			
see above	Alameda County Public Health Lab	Chan-Zuckerberg Biohub	CZB Cliahub Consortium
EPI_ISL_872349	Alaska State Virology Lab	Alaska State Virology Lab	Jack Chen; Lisa Smith; Ph.D.; Stephanie DeRonde
EPI_ISL_420303 to 420306, EPI_ISL_424346 to 424347, EPI_ISL_427619 to 427622, EPI_ISL_431013 to 431019, EPI_ISL_435441 to 435444, EPI_ISL_436464, EPI_ISL_437437, EPI_ISL_437513 to 437518, EPI_ISL_437873, EPI_ISL_476898 to 476899, EPI_ISL_492059, EPI_ISL_492063, EPI_ISL_512144, EPI_ISL_512148, EPI_ISL_512156 to 512157, EPI_ISL_522396 to 522405, EPI_ISL_525758 to 525760, EPI_ISL_528486, EPI_ISL_528493 to 528495, EPI_ISL_528498, EPI_ISL_528513, EPI_ISL_528526, EPI_ISL_560554 to 560566, EPI_ISL_576111 to 576112, EPI_ISL_586243 to 586266, EPI_ISL_602559 to 602561			
see above	Alaska State Virology Laboratory	Alaska State Virology Laboratory	Bortz E; Chen; Chen J et al with Pathogenomics group Dagdag R; Chen, J.; DeRonde, S.; Deuling, H.; Drown DM; George W; J; Jack Chen; Kovalenko A; Milton E; Ph.D.; Redlinger M
EPI_ISL_769919 to 769985	Albany Medical Center Hospital Clinical Laboratories	Wadsworth Center, New York State Department.of Health	Alexis Russel; Daryl M. Lamson; Erasmus Schneider; Erica Lasek-Nesselquist; John Kelly; Jonathan Plitnick; Kirsten St. George; Matthew Shudt; Melissa A Leisner; Navjot Singh; Sara Griesemer
EPI_ISL_984290	Algemeen Medisch Laboratorium	UAntwerp, Laboratory of Medical Microbiology	Basil Britto Xavier; Christine Lammens; Herman Goossens; Jasmine Coppens; Marie Le Mercier; Veerle Matheeussen
EPI_ISL_1039210	Algemeen Medisch Laboratorium (AML)	UAntwerp, Laboratory of Medical Microbiology	Basil Britto Xavier; Christine Lammens; Herman Goossens; Jasmine Coppens; Marie Le Mercier; Veerle Matheeussen
EPI_ISL_508156 to 508191, EPI_ISL_508193 to 508206	All india institute of Medical Sciences Rishikesh	National Institute of Biomedical Genomics	Amit Mangla; Arindam Maitra; Deepjyoti Kalita; Ravi Kant; Saumitra Das
EPI_ISL_511908 to 511910, EPI_ISL_511912,	All india institute of Medical Sciences Rishikesh	National Institute of Biomedical Genomics - DBT's PAN-INDIA	Amit Mangla; Arindam Maitra; Deepjyoti Kalita; Ravi Kant; Saumitra Das

EPI_ISL_511921 to 511922		1000 SARS--CoV-2 RNA Genome Sequencing Consortium	
EPI_ISL_514647, EPI_ISL_514649, EPI_ISL_514651, EPI_ISL_644941	Allina Health Laboratory	Minnesota Department of Health, Public Health Laboratory	Alexandra Lorentz; Jacob Garfin; Matt Plumb; and Xiong Wang
EPI_ISL_700475 to 700476, EPI_ISL_700521, EPI_ISL_700548	Alma CDC wc AHC	NHLS/UCT	Arash Iranzadeh; Bruna Galvao; Carolyn Williamson; Deelan Doolabh; Diana Hardie; Houriiyah Tegally; Innocent Mudau; Kruger Marais; Lynn Tyers; Marvin Hsiao; Stephen Korsman
EPI_ISL_528538	Alsafar	Alsafar	Andreas Henschel; Ernesto Damiani; Gihan Elsir Ahmed Daw Elbait; Guan Tay; Habiba Alsafar; Rifat; Samuel Feng
EPI_ISL_528686 to 528721	Alsafar - Khalifa University Abu Dhabi	Alsafar - Khalifa University Abu Dhabi	Andreas Henschel; Ernesto Damiani; Gihan Daw Elbait; Guan Tay; Habiba Alsafar; Rifat Hamoudi; Samuel Feng
EPI_ISL_900492 to 900494, EPI_ISL_1018220, EPI_ISL_1018222 to 1018227	Althaia. Xarxa Assistencial Universitària de Manresa	IrsiCaixa - Can Ruti CovidSeq	2a planta; Badalona Gloria Trujillo; Bonaventura Clotet; Bonaventura Clotet Gloria Trujillo; Carolina Gonzalez Fernandez; Eulalia Grau; Francesc Catala-Moll; Fundació irsiCaixa. Hospital Universitari Germans Trias i Pujol(HUGTiP); Jaume Trape Pujol; Marc Noguera-Julian; Maria Casadellà; Mariona Parera; Pilar Armengol; Rafel Perez Vidal; Roger Paredes; maternal Ctra Canyet s/n
EPI_ISL_962335, EPI_ISL_1015963	Altius Institute for Biomedical Sciences	Seattle Flu Study	Alex Nguyen; Amanda Adler; Andrew Meuser; Barry R. Lutz; Benjamin Pelle; Caitlin R. Wolf; Chris D. Frazar; Clem Green; Daniel Bates; Deborah A. Nickerson; Elisabeth Brandstetter; Erica Ryke; Hannah Petersen; Helen Y. Chu; Jacob Rodriguez; Janet A. Englund; Jay Shendure; Jessica Halow; John Stamatoyannopoulos; Joshua Richards; Jover Lee; Julia Wald; Kairsten Fay; Kirsten Lacombe; Kneshay Harper; Lea M. Starita; Mark J. Rieder; Matt Hartman; Matthew Richardson; Matthew Thompson; Melissa Truong; Michael Boeckh; Michael Famulare; Misja Ilcisin; Muhammad Halimun; Olivia Waltner; Peter D. Han; Rebecca Bruders; Ryan Alexander; Sadie Patraw; Sofia Olsson; Stephanie DeBaun; Thomas R. Sibley; Tobias Ragoczy; Trevor Bedford; Truong Nguyen
EPI_ISL_515544, EPI_ISL_523984, EPI_ISL_523986	Ama Dr Jose Soares Hungria	Instituto Adolfo Lutz, Interdiciplinary Procedures Center, Strategic Laboratory	Claudia Regina Gonçalves; Claudio Tavares Sacchi; Erica Valessa Ramos Gomes
EPI_ISL_569865 to 569886	Amedeo di savoia	Crosetto lab, Karolinska Institutet, SciLifeLab	Anna Sapino; Luuk Harbers; Maria Grazia Milia; Michele Simonetti; Nicola Crosetto; Ning Zhang; Valeria Ghisetti
EPI_ISL_660121 to 660122, EPI_ISL_660124, EPI_ISL_660127	Ampath	National Health Laboratory Service (NHLS), Tygerberg	Bronwyn Kleinhans; Davis M-A; Draper C; Eduan Wilkindon; Gert van Zyl; Houriiyah Tegally; Hsiao M; Kayla Delaney; Siegfried N; Susan Engelbrecht; Tulio de Oliveira; Williamson C; Wolfgang Preiser
EPI_ISL_860553	Ampath-Netcare	KRISP, KZn Research Innovation and Sequencing Platform	Giandhari J; Khan S; Lessells R; Mdlalose K; Pillay S; Tegally H; Wilkinson E; York D; de Oliveira T
EPI_ISL_766587	Analytica Medizinische Laboratorien AG	Institute of Medical Virology, University of Zurich	Alexandra Trkola; Annette Audigé; Cyril Shah; Jon Huder; Jürg Böni; Kevin Steiner; Maria Grünberg; Maryam Zaheri; Michael Huber; Riccarda Capaul; Stefan Schmutz; Verena Kufner
EPI_ISL_414648, EPI_ISL_416457	Andersen Lab, The Scripps Research Institute	Andersen Lab, The Scripps Research Institute	Catie Anderson; Emily Spender; Karthik Gangavarapu; Kristian Andersen; Laura Nicholson; Mark Zeller; Raphaelle Klitting; Refugio Robles-Sikisaka; Sarah Topol
EPI_ISL_429991, EPI_ISL_430016	Andersen lab at Scripps Research	Andersen lab at Scripps Research	SEARCH Alliance San Diego
EPI_ISL_450818	Aneby VC	The Public Health Agency of Sweden	Anna Risberg; Anna-Malin Linde; Karin Tegmark-Wisell; Ken Granath; Maria Lind Karlberg; Mia Brytting; Olov Svartstrom; Oskar Karlsson Lindsjo; Theresa Enkirch
EPI_ISL_717714 to 717717	Animal Health Centre, British Columbia Ministry of Agriculture	National Centre for Foreign Animal Disease, Canadian Food Inspection Agency	Asma Sultana; Brad Pickering; Oliver Lung; Peter Kruczkiewicz; Tomy Joseph
EPI_ISL_582127 to 582132	Antwerp University Hospital	Institute of Tropical Medicine	Colin Anthony; Philippe Selhorst
EPI_ISL_512663 to 512664, EPI_ISL_512669, EPI_ISL_527740, EPI_ISL_527749	Area De Salud Alajuela Norte - Clinica Dr. Marcial Rodriguez	Inciensa, Instituto Costarricense de Investigación y Enseñanza en Nutrición y Salud	Adriana Godinez & Melany Calderon; Claudio Soto-Garita; Estela Cordero; Francisco Duarte; Hebleen Porras
EPI_ISL_527756	Area De Salud Aserri	Inciensa, Instituto Costarricense de Investigación y Enseñanza en Nutrición y Salud	Adriana Godinez & Melany Calderon; Claudio Soto-Garita; Estela Cordero; Francisco Duarte; Hebleen Porras
EPI_ISL_770030	Area De Salud Buenos Aires	Inciensa, Instituto Costarricense de Investigación y Enseñanza en Nutrición y Salud	Adriana Godínez; Claudio Soto-Garita; Estela Cordero; Francisco Duarte; Hebleen Porras; Melany Calderón & Mariel López
EPI_ISL_769994, EPI_ISL_769997 to 769998, EPI_ISL_770021, EPI_ISL_770026	Area De Salud Catedral Noreste	Inciensa, Instituto Costarricense de Investigación y Enseñanza en Nutrición y Salud	Adriana Godínez; Claudio Soto-Garita; Estela Cordero; Francisco Duarte; Hebleen Porras; Melany Calderón & Mariel López
EPI_ISL_770011 to 770012	Area De Salud Coronado	Inciensa, Instituto Costarricense de Investigación y Enseñanza en Nutrición y Salud	Adriana Godínez; Claudio Soto-Garita; Estela Cordero; Francisco Duarte; Hebleen Porras; Melany Calderón & Mariel López
EPI_ISL_512668, EPI_ISL_527748, EPI_ISL_527751, EPI_ISL_770008, EPI_ISL_770029	Area De Salud Corredores	Inciensa, Instituto Costarricense de Investigación y Enseñanza en Nutrición y Salud	Adriana Godinez & Melany Calderon; Adriana Godínez; Claudio Soto-Garita; Estela Cordero; Francisco Duarte; Hebleen Porras; Melany Calderón & Mariel López
EPI_ISL_770014	Area De Salud Curridabat 2	Inciensa, Instituto Costarricense de Investigación y Enseñanza en Nutrición y Salud	Adriana Godínez; Claudio Soto-Garita; Estela Cordero; Francisco Duarte; Hebleen Porras; Melany Calderón & Mariel López
EPI_ISL_512653	Area De Salud Desamparados 1 - Clinica Dr. Marcial Fallas [Grifo Alto/Desampara	Inciensa, Instituto Costarricense de Investigación y Enseñanza en Nutrición y Salud	Adriana Godinez & Melany Calderon; Claudio Soto-Garita; Estela Cordero; Francisco Duarte; Hebleen Porras
EPI_ISL_769993	Area De Salud El Guarco	Inciensa, Instituto Costarricense de Investigación y Enseñanza en Nutrición y Salud	Adriana Godínez; Claudio Soto-Garita; Estela Cordero; Francisco Duarte; Hebleen Porras; Melany Calderón & Mariel López
EPI_ISL_770009 to 770010	Area De Salud Escazu (Coopesana)	Inciensa, Instituto Costarricense de Investigación y Enseñanza en Nutrición y Salud	Adriana Godínez; Adriana Godínez & Melany Calderón; Claudio Soto-Garita; Estela Cordero; Francisco Duarte; Hebleen Porras; Melany Calderón & Mariel López
EPI_ISL_512659	Area De Salud Fortuna	Inciensa, Instituto Costarricense de Investigación y Enseñanza en Nutrición y Salud	Adriana Godinez & Melany Calderon; Claudio Soto-Garita; Estela Cordero; Francisco Duarte; Hebleen Porras
EPI_ISL_527757	Area De Salud Goicoechea 1	Inciensa, Instituto Costarricense de Investigación y Enseñanza en Nutrición y Salud	Adriana Godinez & Melany Calderon; Claudio Soto-Garita; Estela Cordero; Francisco Duarte; Hebleen Porras
EPI_ISL_770016, EPI_ISL_770020	Area De Salud Goicoechea 2 - Clinica Dr. Jimenez Nuñez	Inciensa, Instituto Costarricense de Investigación y Enseñanza en Nutrición y Salud	Adriana Godínez; Claudio Soto-Garita; Estela Cordero; Francisco Duarte; Hebleen Porras; Melany Calderón & Mariel López
EPI_ISL_512662, EPI_ISL_512666 to 512667, EPI_ISL_512671, EPI_ISL_527746 to 527747, EPI_ISL_770000 to 770003, EPI_ISL_770027	Area De Salud La Cruz	Inciensa, Instituto Costarricense de Investigación y Enseñanza en Nutrición y Salud	Adriana Godinez & Melany Calderon; Adriana Godínez; Claudio Soto-Garita; Estela Cordero; Francisco Duarte; Hebleen Porras; Melany Calderón & Mariel López
EPI_ISL_512660 to 512661	Area De Salud Los Chiles	Inciensa, Instituto Costarricense de Investigación y Enseñanza en Nutrición y Salud	Adriana Godinez & Melany Calderon; Claudio Soto-Garita; Estela Cordero; Francisco Duarte; Hebleen Porras
EPI_ISL_770005	Area De Salud Moravia	Inciensa, Instituto Costarricense de Investigación y Enseñanza en Nutrición y Salud	Adriana Godínez; Claudio Soto-Garita; Estela Cordero; Francisco Duarte; Hebleen Porras; Melany Calderón & Mariel López
EPI_ISL_512658	Area De Salud Orotina-San Mateo [Orotina/Alajuela]	Inciensa, Instituto Costarricense de Investigación y Enseñanza en Nutrición y Salud	Adriana Godinez & Melany Calderon; Claudio Soto-Garita; Estela Cordero; Francisco Duarte; Hebleen Porras
EPI_ISL_769999	Area De Salud Paraiso-Cervantes	Inciensa, Instituto Costarricense de Investigación y Enseñanza en Nutrición y Salud	Adriana Godínez; Claudio Soto-Garita; Estela Cordero; Francisco Duarte; Hebleen Porras; Melany Calderón & Mariel López

EPI_ISL_512656	Area De Salud Pavas (Coopesalud) [Pavas/San Jose]	Inciensa, Instituto Costarricense de Investigación y Enseñanza en Nutrición y Salud	Adriana Godinez & Melany Calderon; Claudio Soto-Garita; Estela Cordero; Francisco Duarte; Hebleen Porras
EPI_ISL_770017, EPI_ISL_770022	Area De Salud Perez Zeledon	Inciensa, Instituto Costarricense de Investigación y Enseñanza en Nutrición y Salud	Adriana Godínez; Claudio Soto-Garita; Estela Cordero; Francisco Duarte; Hebleen Porras; Melany Calderón & Mariel López
EPI_ISL_769988 to 769989, EPI_ISL_770015, EPI_ISL_770023	Area De Salud San Francisco-San Antonio (Coopesana)	Inciensa, Instituto Costarricense de Investigación y Enseñanza en Nutrición y Salud	Adriana Godínez; Claudio Soto-Garita; Estela Cordero; Francisco Duarte; Hebleen Porras; Melany Calderón & Mariel López
EPI_ISL_769996, EPI_ISL_770025	Area De Salud San Juan-San Diego-Concepcion 2	Inciensa, Instituto Costarricense de Investigación y Enseñanza en Nutrición y Salud	Adriana Godínez; Claudio Soto-Garita; Estela Cordero; Francisco Duarte; Hebleen Porras; Melany Calderón & Mariel López
EPI_ISL_770007	Area De Salud San Rafael	Inciensa, Instituto Costarricense de Investigación y Enseñanza en Nutrición y Salud	Adriana Godínez; Claudio Soto-Garita; Estela Cordero; Francisco Duarte; Hebleen Porras; Melany Calderón & Mariel López
EPI_ISL_512657	Area De Salud Tibas-Uruca-Merced - Clinica Dr. Clorito Picado [Tibas/San Jose]	Inciensa, Instituto Costarricense de Investigación y Enseñanza en Nutrición y Salud	Adriana Godinez & Melany Calderon; Claudio Soto-Garita; Estela Cordero; Francisco Duarte; Hebleen Porras
EPI_ISL_491446	Area de Salud Alajuela Central	Inciensa, Instituto Costarricense de Investigación y Enseñanza en Nutrición y Salud	Adriana Godinez & Melany Calderon; Claudio Soto-Garita; Estela Cordero; Francisco Duarte; Hebleen Brenes
EPI_ISL_434533, EPI_ISL_434535, EPI_ISL_491449	Area de Salud Alajuela Sur	Inciensa, Instituto Costarricense de Investigación y Enseñanza en Nutrición y Salud	Adriana Godinez & Melany Calderon; Claudio Soto-Garita; Estela Cordero; Francisco Duarte; Hebleen Brenes; Hebleen Porras
EPI_ISL_491437, EPI_ISL_491444	Area de Salud Escazu (Coopesana)	Inciensa, Instituto Costarricense de Investigación y Enseñanza en Nutrición y Salud	Adriana Godinez & Melany Calderon; Claudio Soto-Garita; Estela Cordero; Francisco Duarte; Hebleen Brenes
EPI_ISL_491457	Area de Salud Los Santos	Inciensa, Instituto Costarricense de Investigación y Enseñanza en Nutrición y Salud	Adriana Godinez & Melany Calderon; Claudio Soto-Garita; Estela Cordero; Francisco Duarte; Hebleen Brenes
EPI_ISL_491445	Area de Salud Mata Redonda	Inciensa, Instituto Costarricense de Investigación y Enseñanza en Nutrición y Salud	Adriana Godinez & Melany Calderon; Claudio Soto-Garita; Estela Cordero; Francisco Duarte; Hebleen Brenes
EPI_ISL_434539	Area de Salud Orotina	Inciensa, Instituto Costarricense de Investigación y Enseñanza en Nutrición y Salud	Adriana Godinez & Melany Calderon; Claudio Soto-Garita; Estela Cordero; Francisco Duarte; Hebleen Porras
EPI_ISL_500596 to 500706, EPI_ISL_500717, EPI_ISL_509492 to 509523, EPI_ISL_527008 to 527064, EPI_ISL_527179 to 527180, EPI_ISL_527400, EPI_ISL_530334 to 530340, EPI_ISL_537286 to 537287, EPI_ISL_539327, EPI_ISL_569858, EPI_ISL_577597, EPI_ISL_577604, EPI_ISL_586571, EPI_ISL_710513, EPI_ISL_738137, EPI_ISL_745029 to 745031, EPI_ISL_794671			
see above	Area of Virology, Serology and Virology Division (SAViD), New South Wales Health Pathology Randwick	Area of Virology, Serology and Virology Division (SAViD), New South Wales Health Pathology Randwick	Bull, R.; Deveson, I.; Foster, C.; Rawlinson; Rawlinson, W.; Van Haal, S.; Van Hal, S.; W
EPI_ISL_678293, EPI_ISL_678297, EPI_ISL_678299, EPI_ISL_678306 to 678310, EPI_ISL_678314 to 678316, EPI_ISL_678318, EPI_ISL_678320 to 678323, EPI_ISL_678325 to 678328, EPI_ISL_678330 to 678333, EPI_ISL_678336 to 678337, EPI_ISL_678340 to 678343, EPI_ISL_678345, EPI_ISL_678348, EPI_ISL_678351, EPI_ISL_678354, EPI_ISL_678358 to 678361, EPI_ISL_678363, EPI_ISL_678365 to 678368, EPI_ISL_678370 to 678381, EPI_ISL_678383, EPI_ISL_707891 to 707892, EPI_ISL_707894, EPI_ISL_707896 to 707902, EPI_ISL_717701 to 717708, EPI_ISL_717710, EPI_ISL_717712 to 717713, EPI_ISL_1005536 to 1005538			
see above	Area of Virology, Serology and Virology Division (SAViD), New South Wales Health Pathology Randwick	Virology Research Laboratory; Area of Virology, Serology and Virology Division (SAViD), New South Wales Health Pathology Randwick	Au, J.; Bull, R.; Deveson, I.; Foster, C.; Rawlinson, W.; Ruiz Silva, M.; Van Hal, S.
EPI_ISL_406223	Arizona Department of Health Services	Pathogen Discovery, Respiratory Viruses Branch, Division of Viral Diseases, Centers for Disease Control and Prevention	Anna Uehara; Brett L. Whitaker; Brian Lynch; Clinton R. Paden; Janna' R. Murray; Jing Zhang; Krista Queen; Lijuan Wang; Senthil Kumar K. Sakthivel; Shifaq Kamili; Stephen Lindstrom; Susan I. Gerber; Suxiang Tong; Xiaoyan Lu; Yan Li; Ying Tao
EPI_ISL_967882	Arizona State Public Health Laboratory	Arizona State Public Health Laboratory	Jessica Escobar; Katherine Fullerton; Linda Getsinger; Nobuko Fukushima; Stacy White; Trung Huynh; Victor Waddell
EPI_ISL_903361 to 903363, EPI_ISL_943569	Arizona State University	Arizona State University	Efrem S. Lim; Emily A. Kaelin; Joshua LaBaer; Joy M. Blain; Kristina Buss; LaRinda A. Holland; Nicholas J. Mellor; Peter T. Skidmore; Valerie Harris; Vel Murugan
EPI_ISL_424668 to 424669, EPI_ISL_424671, EPI_ISL_467372 to 467373	Arizona State University Health Services	Arizona State University	Arvind Varsani; Bereket Estifanos; Brenda G. Hogue; Efrem S. Lim; Emily A. Kaelin; Jason Steel; LaRinda A. Holland; Lily I. Wu; Matthew Scotch; Nicholas J. Mellor; Peter T. Skidmore; Rabia Maqsood; Rolf U. Halden
EPI_ISL_496518 to 496520, EPI_ISL_496529, EPI_ISL_496533, EPI_ISL_496537 to 496545, EPI_ISL_496602, EPI_ISL_721629 to 721645, EPI_ISL_722187 to 722200			
see above	Armed Forces Medical College	National Centre For Cell Science	Arvind Sahu; DBT's PAN-INDIA 1000 SARS-CoV2 RNA genome sequencing consortium; Dhiraj Paul; Girdhari Lal; Janesh Kumar; Kavita Bala Anand; Kunal Jani; Maharashtra COVID-19 Study Group; Manoj Kumar Bhat; Murlidhar Tambe; Radha Chauhan; Rajesh Karyakarte; Rajiv Mohan Gupta; Santosh Karade; Shelinder Pal Singh Shergill; Sourav Sen; Suvarna Joshi; Vasudevan Seshadri; Yogesh S Shouche
EPI_ISL_457826	Army Medical Center - Scientific Department	Army Medical and Veterinary Research Center	Anna Anselmo; Antonella Fortunato; Florigio Lista; Francesco Giordani; Giovanni Faggioni; Nino D'Amore; Riccardo De Sanctis; Silvia Fillo; Vanessa Vera Fain
EPI_ISL_457825	Army Medical Research Center - Scientific Department	Army Medical and Veterinary Research Center	Anna Anselmo; Antonella Fortunato; Florigio Lista; Francesco Giordani; Giovanni Faggioni; Nino D'Amore; Riccardo De Sanctis; Silvia Fillo; Vanessa Vera Fain
EPI_ISL_539809 to 539812, EPI_ISL_539821	Asiaworld Expo Command Post	Hong Kong Department of Health	Alan K.L. Tsang; Dominic N.C. Tsang; Edman T.K. Lam; Peter C.W. Yip; Rickjason C.W. Chan
EPI_ISL_900525	Astralab	CNR Virus des Infections Respiratoires - France SUD	Antonin Bal; Bruno Lina; Gregory Destras; Gwendolyne Burfin; Hadrien Règue; Laurence Josset; Martine Valette; Quentin Semanas
EPI_ISL_902969	Atlas Genomics - UW Virology Lab	UW Virology Lab	Alexander Greninger; Hong Xie; Keith R Jerome; Lasata Shrestha; Meei-Li Huang; Michelle Lin; Pavitra Roychoudhury
EPI_ISL_413490, EPI_ISL_416519	Auckland Hospital	Institute of Environmental Science and Research (ESR)	Erasmus Smit; Gary McAuliffe; Joep de Ligt; Lauren Jelly; Matt Storey; Matthew Blakiston; Sally Roberts; Xiaoyun Ren
EPI_ISL_569616	Aurora County Clinic	South Dakota Public Health Laboratory	Jacob Garfin; Matt Plumb; Xiong Wang; and Chris Carlson
EPI_ISL_513340, EPI_ISL_526166 to 526169, EPI_ISL_593649	Austech Medical Laboratories	NSW Health Pathology - Institute of Clinical Pathology and Medical Research; Westmead Hospital; University of Sydney	CIDM-PH et al.
EPI_ISL_745091 to 745092	Australian Clinical Labs	CIDM-PH, Westmead Hospital	CIDM-PH et al.
EPI_ISL_451486, EPI_ISL_451574, EPI_ISL_451599, EPI_ISL_513331, EPI_ISL_544966 to 544967, EPI_ISL_544988, EPI_ISL_545024, EPI_ISL_547597, EPI_ISL_591484, EPI_ISL_767896, EPI_ISL_767902, EPI_ISL_767916 to 767918			
see above	Australian Clinical Labs	NSW Health Pathology - Institute of Clinical Pathology and Medical Research; Westmead Hospital; University of Sydney	CIDM-PH et al.
EPI_ISL_526212, EPI_ISL_767884	Australian Clinical Labs (formerly Healthscope Pathology)	NSW Health Pathology - Institute of Clinical Pathology and Medical Research; Westmead Hospital; University of Sydney	CIDM-PH et al.
EPI_ISL_475830 to 475845, EPI_ISL_475848 to 475855, EPI_ISL_475859 to 475866, EPI_ISL_475869 to 475876, EPI_ISL_475878 to 475880, EPI_ISL_475882 to 475886, EPI_ISL_583577 to 583578, EPI_ISL_583630 to 583631, EPI_ISL_583633 to 583637, EPI_ISL_583639 to 583676, EPI_ISL_583678 to 583690, EPI_ISL_583883 to 583892, EPI_ISL_854176 to 854208, EPI_ISL_934434 to 934435, EPI_ISL_934437 to 934439, EPI_ISL_934441 to 934446, EPI_ISL_934448 to 934452, EPI_ISL_934454, EPI_ISL_934457 to 934459, EPI_ISL_934465 to 934467, EPI_ISL_934469 to 934472, EPI_ISL_934478 to 934479, EPI_ISL_934481 to 934483, EPI_ISL_934485, EPI_ISL_934487, EPI_ISL_934490 to 934492, EPI_ISL_934495, EPI_ISL_934531 to 934534, EPI_ISL_934627 to 934631, EPI_ISL_1008029, EPI_ISL_1008076, EPI_ISL_1008103, EPI_ISL_1008144, EPI_ISL_1008146, EPI_ISL_1008163, EPI_ISL_1008172			
see above	Austrian Agency for Health and Food Safety (AGES)	Bergthaler laboratory, CeMM Research Center for Molecular Medicine of the Austrian Academy of Sciences	Adi Steinrigl; Alexander Lercher; Alexandra Popa; Andreas Bergthaler; Anna Schedl; Bekir Erguner; Benedikt Agerer; Christian Paar; Christoph Bock; Christoph Bock; Daniela Schmid; Dorothee von Laer; Elisabeth Puchhammer-Stoeckl; Franz Allerberger; Gernot Walder; Gregor Hörmann; Guenter Weiss; Gunther Vogl; Henrique Colaco; Jakob-Wendelin Genger; Jan Laine; Judith Aberle; Kinga Rigler-Hohenwarter; Lukas Endler; Maelle Le Moing; Manfred Nairz; Mark Smyth; Martin Senekowitsch; Martin Senekowitsch; Michael Schuster; Michael Schuster; Peter Hufnagl; Peter Obrist; Rainer Gattringer; Sabine Sussitz-Rack; Stephan Aberle; Thomas Penz; Wegene Borena
EPI_ISL_1020299	Austrian Agency for Health and Food Safety (AGES)	Center for Virology	Irene Goerzer; Jeremy V. Camp; Monika Redlberger-Fritz; Stephan W. Aberle

EPI_ISL_569610, EPI_ISL_569624	Avera McKennan Hospital	South Dakota Public Health Laboratory	Jacob Garfin; Matt Plumb; Xiong Wang; and Chris Carlson
EPI_ISL_507963 to 507972, EPI_ISL_514645	Avera Mckennan Laboratory	Minnesota Department of Health, Public Health Laboratory	Jacob Garfin; Matt Plumb; and Xiong Wang
EPI_ISL_882637, EPI_ISL_882639, EPI_ISL_882642	Azerbaijan National Hematology Center Division of Medical Genetics	Azerbaijan National Hematology Center Division of Medical Genetics	Aghayev Agha Rza; Bayraml Ramin
EPI_ISL_902753, EPI_ISL_1039784	Azienda Ospedaliera San Camillo Forlanini	INMI Lazzaro Spallanzani IRCCS	A Di Caro; B Bartolini; C.E.M Gruber; CEM Gruber; D.Gallone; E Giombini; F Messina; G Parisi; M Rueca; ML Guarino; MR Capobianchi; O Butera
EPI_ISL_1013095	Azienda Sanitaria dell'Alto Adige Laboratorio Aziendale di Microbiologia e Virologia	Istituto di Genomica Applicata	Davide Scaglione; Eleonora Paparelli; Elisa Masi; Elisabetta Giacobazzi; Elisabetta Pagani; Irena Jurman; Irene Bianconi; Michele Morgante; Stefanie Wieser; Vera Vendramin
EPI_ISL_1015532 to 1015533, EPI_ISL_1015536 to 1015539, EPI_ISL_1015541, EPI_ISL_1015549, EPI_ISL_1015552, EPI_ISL_1015560	Azienda Sanitaria dell'Alto Adige Laboratorio Aziendale di Microbiologia e Virologia	Istituto di Genomica Applicata	Davide Scaglione; Eleonora Paparelli; Elisa Masi; Elisabetta Giacobazzi; Elisabetta Pagani; Gabriele Magris; Irena Jurman; Irene Bianconi; Michele Morgante; Stefanie Wieser; Vera Vendramin
EPI_ISL_496521 to 496523, EPI_ISL_496530, EPI_ISL_496534, EPI_ISL_496546 to 496554, EPI_ISL_728254 to 728271, EPI_ISL_728325 to 728328	B.J. Govt. Medical College	National Centre For Cell Science	Arvind Sahu; DBT's PAN-INDIA 1000 SARS-CoV2 RNA genome sequencing consortium; Dhiraj Paul; Girdhari Lal; Janesh Kumar; Kavita Bala Anand; Kunal Jani; Maharashtra COVID-19 Study Group; Manoj Kumar Bhat; Murlidhar Tambe; Radha Chauhan; Rajesh Karyakarte; Rajiv Mohan Gupta; Santosh Karade; Shelinder Pal Singh Shergill; Sourav Sen; Suvarna Joshi; Vasudevan Seshadri; Yogesh S Shouche
EPI_ISL_435049 to 435054, EPI_ISL_437445 to 437454, EPI_ISL_444456 to 444481, EPI_ISL_447030 to 447046, EPI_ISL_458086 to 458102, EPI_ISL_461483 to 461506, EPI_ISL_467041 to 467054, EPI_ISL_469024 to 469028, EPI_ISL_495016 to 495019			
see above	B.J. Medical College and Civil hospital	Gujarat Biotechnology Research Centre	; A M Kadri; Afzal Ansari; Akanksha Verma; Amit Kanani; Anjali Rajwar; Ankit Hinsu; Apurvasinh Puvar; Armi Chaudhari; Bhavesh Modi; Bhavya Jindal; Binita Aring; Camellia Chakraborty; Chaitanya Joshi; Dhaval Vaghela; Dinesh Kumar; Dipa Kinariwala; Dipeshwari Shewale; Disha Patel; Fenil Patel; Gaurishankar Shrimali; Geeta Vaghela; Harsh Bakshi; Janvi Raval; Kairavi Joshi; Kamlesh J Upadhyay; Komal Patel; Labdhi Pandya; Madhvi Joshi; Maharshi Pandya; Monika Gandhi; Neelam Nathani; Neeta Khandelwal; Neha Rajpara; Nidhi Patel; Nidhi Sood; Nikha Trivedi; Nirav Mungalpara; Nitin Savaliya; Pinal Trivedi; Pooja P Doshi; Pragya Sharma; Pranay Shah; Pritesh Sabara; Priti Pandita; Priyanka P Vatsa; R D Dixit; Raghawendra Kumar; Ramesh Pandit; Ramesh Patel; Sanjay Kapadia; Sharmistha Majumdar; Siddhant Kumar; Snehal Bagatharia; Sonia Barve; Tejas Shah; Umang Mishra; Vasudha Sharma; Zarna Patel; Zuber Saiyed
EPI_ISL_512058 to 512060, EPI_ISL_512063 to 512065, EPI_ISL_514581 to 514609, EPI_ISL_524713 to 524729, EPI_ISL_525421 to 525422, EPI_ISL_586513 to 586530	B.J. Medical College and Civil hospital, Ahmedabad	Gujarat Biotechnology Research Centre	A M Kadri; Afzal Ansari; Apurvasinh Puvar; Chaitanya Joshi; Dinesh Kumar; Harsh Bakshi; Janvi Raval; Kamlesh J Upadhyay; Komal Patel; Labdhi Pandya; Madhvi Joshi; Maharshi Pandya; Monika Gandhi; Nidhi Patel; Nikha Trivedi; Nitin Savaliya; Pinal Trivedi; Pranay Shah; R D Dixit; Raghawendra Kumar; Sanjay Kapadia; Zarna Patel; Zuber Saiyed
EPI_ISL_995711	BANGALORE MEDICAL COLLEGE AND RESEARCH INSTITUTE	Department of Neurovirology, National Institute of Mental Health and Neurosciences (NIMHANS)	Anita S Desai; Anson Kunjumon George; Chitra Pattabiraman; Darshan Sreenivas; Nakka Vijay Kiran Reddy; Pramada Prasad; Risha Rasheed; V Ravi
EPI_ISL_412965, EPI_ISL_415578, EPI_ISL_415580 to 415586, EPI_ISL_415588 to 415589, EPI_ISL_418816 to 418826, EPI_ISL_418829 to 418858, EPI_ISL_460609 to 460612, EPI_ISL_460614 to 460616, EPI_ISL_462757 to 462761, EPI_ISL_462765 to 462769, EPI_ISL_462771 to 462772, EPI_ISL_462774 to 462784, EPI_ISL_462786 to 462787, EPI_ISL_462789 to 462794, EPI_ISL_462798, EPI_ISL_462802, EPI_ISL_462805 to 462816, EPI_ISL_462818 to 462822, EPI_ISL_462824 to 462831, EPI_ISL_462833 to 462838, EPI_ISL_462840 to 462843, EPI_ISL_463186 to 463187, EPI_ISL_463189, EPI_ISL_463191, EPI_ISL_463193 to 463196, EPI_ISL_463198 to 463217, EPI_ISL_463219 to 463232, EPI_ISL_463235 to 463268, EPI_ISL_463270 to 463276, EPI_ISL_466696 to 466743, EPI_ISL_466745, EPI_ISL_466747 to 466770, EPI_ISL_466772 to 466777, EPI_ISL_466779, EPI_ISL_466781 to 466785, EPI_ISL_466787 to 466789, EPI_ISL_466792 to 466796, EPI_ISL_466798, EPI_ISL_466800, EPI_ISL_466802 to 466803, EPI_ISL_466806 to 466812, EPI_ISL_466814 to 466816, EPI_ISL_466819 to 466822, EPI_ISL_466824, EPI_ISL_466826, EPI_ISL_466829, EPI_ISL_466831 to 466832, EPI_ISL_466834, EPI_ISL_466838, EPI_ISL_467305 to 467306, EPI_ISL_467309 to 467310, EPI_ISL_467312 to 467315, EPI_ISL_467317 to 467318, EPI_ISL_467320 to 467322, EPI_ISL_467324, EPI_ISL_467327, EPI_ISL_467329 to 467343, EPI_ISL_467423 to 467429, EPI_ISL_468658 to 468662, EPI_ISL_468664 to 468666, EPI_ISL_468668 to 468680, EPI_ISL_468683 to 468689, EPI_ISL_468691, EPI_ISL_468693 to 468696, EPI_ISL_468698 to 468700, EPI_ISL_469209 to 469216, EPI_ISL_469218, EPI_ISL_469222 to 469223, EPI_ISL_477020 to 477024, EPI_ISL_477029, EPI_ISL_477031 to 477041, EPI_ISL_477045 to 477052, EPI_ISL_477054, EPI_ISL_477056 to 477076, EPI_ISL_477079 to 477081, EPI_ISL_477085 to 477088, EPI_ISL_477090 to 477096, EPI_ISL_477098 to 477103, EPI_ISL_477105 to 477115, EPI_ISL_477117 to 477119, EPI_ISL_740548, EPI_ISL_740550 to 740551, EPI_ISL_740553 to 740555, EPI_ISL_740557, EPI_ISL_740559 to 740592, EPI_ISL_740595 to 740603, EPI_ISL_740606, EPI_ISL_740608 to 740609, EPI_ISL_740611 to 740612, EPI_ISL_740614 to 740623, EPI_ISL_740625 to 740627, EPI_ISL_740630 to 740638, EPI_ISL_740640 to 740641, EPI_ISL_740643 to 740645, EPI_ISL_740647 to 740649, EPI_ISL_740651 to 740657, EPI_ISL_740659 to 740665, EPI_ISL_740668 to 740670, EPI_ISL_740678, EPI_ISL_740680 to 740687, EPI_ISL_740689 to 740690, EPI_ISL_740693 to 740698, EPI_ISL_740700 to 740711, EPI_ISL_740713, EPI_ISL_740715, EPI_ISL_740717 to 740721, EPI_ISL_740723 to 740724, EPI_ISL_740727 to 740731, EPI_ISL_740735 to 740736, EPI_ISL_740738 to 740740, EPI_ISL_740742 to 740771, EPI_ISL_740773 to 740775, EPI_ISL_740778 to 740779, EPI_ISL_740781 to 740785, EPI_ISL_740788, EPI_ISL_740790 to 740793, EPI_ISL_740795 to 740797, EPI_ISL_740799 to 740831, EPI_ISL_740833 to 740838, EPI_ISL_740840 to 740847, EPI_ISL_740849 to 740851, EPI_ISL_740853, EPI_ISL_740855 to 740862, EPI_ISL_975333, EPI_ISL_976766 to 976767, EPI_ISL_976879			
see above	BCCDC Public Health Laboratory	BCCDC Public Health Laboratory	Ana Pacagnella; Choi; Corrinne Ng; Dan Fornika; Gilmour; Harrigan; Hoang; Hope Lapointe; Inna Sekirov; Jinny Choi; John Tyson; Kamelian; Kim Macdonald; Kimia Kamelian; Krajden; Lapointe; Lee; Levett; Li; Linda Hoang; Loman; Loretta Janz; Mel Krajden; Natalie Prystajecky; Paul Levett; Prystajecky; Prystajecky Natalie; Quick; Richard Harrigan; Robert Azana Terry Snutch; Sekirov; Shannon Russell; Snutch; Terry Snutch; Tyson
EPI_ISL_486395	BIMS	Department of Neurovirology, National Institute of Mental Health and Neuroscience (NIMHANS)	Anita Desai; Chitra Pattabiraman; Harsha PK; Manjunatha Venkataswamy; Ravi Vasanthapuram; Risha Rasheed; Shafeeq S Hameed; Vijayalakshmi Reddy
EPI_ISL_804982 to 804983, EPI_ISL_861177 to 861178, EPI_ISL_896297	BIO-REFERENCE LABORATORIES	Wadsworth Center, New York State Department of Health	Alexis Russel; Daryl M. Lamson; Erasmus Schneider; Erica Lasek-Nesselquist; John Kelly; Jonathan Plitnick; Kirsten St. George; Matthew Shudt; Melissa A Leisner; Navjot Singh
EPI_ISL_767651 to 767653, EPI_ISL_767665 to 767680, EPI_ISL_804946	BIO-REFERENCE LABORATORIES	Wadsworth Center, New York State Department.of Health	Alexis Russel; Daryl M. Lamson; Erasmus Schneider; Erica Lasek-Nesselquist; John Kelly; Jonathan Plitnick; Kirsten St. George; Matthew Shudt; Melissa A Leisner; Navjot Singh; Sara Griesemer
EPI_ISL_830214, EPI_ISL_854446, EPI_ISL_883429 to 883430	BOSTON HEART DIAGNOSTICS CORP	Wadsworth Center, New York State Department of Health	Alexis Russel; Daryl M. Lamson; Erasmus Schneider; Erica Lasek-Nesselquist; John Kelly; Jonathan Plitnick; Kirsten St. George; Matthew Shudt; Melissa A Leisner; Navjot Singh
EPI_ISL_859675, EPI_ISL_859852, EPI_ISL_859878, EPI_ISL_859885, EPI_ISL_859985, EPI_ISL_859995, EPI_ISL_859999, EPI_ISL_860016, EPI_ISL_860028 to 860029, EPI_ISL_860031 to 860032, EPI_ISL_860037 to 860038, EPI_ISL_860042 to 860043, EPI_ISL_860046, EPI_ISL_860049, EPI_ISL_860058, EPI_ISL_860064, EPI_ISL_860067, EPI_ISL_860071 to 860072, EPI_ISL_860076, EPI_ISL_860080, EPI_ISL_860082, EPI_ISL_860084 to 860085, EPI_ISL_860087 to 860089			
see above	BTC, Khalifa University	BTC, Khalifa University	Al Safar et al
EPI_ISL_574619 to 574623	BTKLPP Kelas I Manado	Eijkman Institute for Molecular Biology, Ministry of Research and Technology/National Agency for Research and Innovation	Amin Soebandrio; David H Muljono; Edison Johar; Frilasita A Yudhaputri; Herawati Sudoyo; Hidayat Trimarsanto; Iskandar A Adnan; Khin Saw Myint; Safarina G Malik; Willy Agustine
EPI_ISL_538506 to 538507, EPI_ISL_538512	Balai Penelitian dan Pengembangan Biomedis Papua	National Institute of Health Research and Development	A; AA; HA; HD; HML; Hutapea; Ikawati; KD; KNA; M; Nugraha; Oktavian; Paisal; Pangesti; Pasaribu; Pawestri; Puspa; Setiawaty, V.; Soekarso; Subangkit; T
EPI_ISL_403962 to 403963	Bamrasnaradura Hospital	1. Department of Medical Sciences, Ministry of Public Health, Thailand 2. Thai Red Cross Emerging Infectious Diseases - Health Science Centre 3. Department of Disease Control, Ministry of Public Health, Thailand	Buathong; Chittaganpitch; Malinee; Mekha; Nanthawan; Okada; Parnmen; Phuygun; Pilailuk; Rome; Siripaporn; Sittiporn; Sunthareeya; Supaporn; Thanadachakul; Thanutsapa; Wacharapluesadee; Waicharoen; Warawan; Wongboot
EPI_ISL_434693 to 434694, EPI_ISL_515469	Bamrasnaradura hospital	National Institute of Health. Department of medical Sciences, Ministry of Public Health, Thailand	Chittaganpitch; Malinee; Okada; Parnmen; Phuygun; Pilailuk; Siripaporn; Sittiporn; Sunthareeya; Thanadachakul; Thanutsapa; Waicharoen; Warawan; Wongboot
EPI_ISL_469048, EPI_ISL_475026 to 475029, EPI_ISL_476867 to 476868	Banas Medical College and Research Institute	Gujarat Biotechnology Research Centre	A M Kadri; Afzal Ansari; Ankit Hinsu; Apurvasinh Puvar; Chaitanya Joshi; Dinesh Kumar; Fenil Patel; Harsh Bakshi; Janvi Raval; Komal Patel; Labdhi Pandya; Madhvi Joshi; Maharshi Pandya; Monika Gandhi; Neelam Nathani; Neha Rajpara; Nidhi Patel; Nikha Trivedi; Nitin Savaliya; Pinal Trivedi; Pritesh Sabara; Priti Pandita; R D Dixit; Radhika Khara; Raghawendra Kumar; Snehal Bagatharia; Sunil R Joshi; Tejas Shah; Viren s Doshi; Zarna Patel; Zuber Saiyed
EPI_ISL_860182 to 860183	Bangalore Medical College and Research Institute	Department of Neurovirology, National Institute of Mental Health and Neurosciences (NIMHANS)	Anita S Desai; Anson Kunjumon George; Chitra Pattabiraman; Darshan Sreenivas; Nakka Vijay Kiran Reddy; Pramada Prasad; Risha Rasheed; V Ravi
EPI_ISL_450339, EPI_ISL_450341, EPI_ISL_450343 to 450345	Bangladesh Institute of Tropical & Infectious Diseases, COVID-19 Testing Laboratory	Basic and Applied Research on Jute Project	A S M Anwarul Huq; AMAM Zonaed Siddiki; Eaftekhar Ahmed Rana; Emdadul Mannan Emdad; Goutam Buddha Das; M A Hassan Chowdhury; Md. Monjurul Alam; Md. Nazmul Haq Rony; Md. Sabbir Hossain; Md. Samiul Haque; Md. Shahidul Islam; Md. Shakeel Ahmed; Md. Sharifur Rahman; Paritous Kumar Biswas; Rasel Ahmed; Shah Md Tamim Kabir
EPI_ISL_699484, EPI_ISL_708721 to 708722,	Bangpakok 9 international hospital	National Institute of Health, Department of Medical Sciences,	Malinee Chittaganpitch; Pilailuk Okada; Siripaporn Phuygun; Sittiporn Parnmen; Sunthareeya Waicharoen; Thanutsapa Thanadachakul; Warawan

EPI_ISL_708736		Ministry of Public Health, Thailand	Wongboot
EPI_ISL_539886, EPI_ISL_710613, EPI_ISL_732657, EPI_ISL_766645	Barnakuten	The Public Health Agency of Sweden	Anna Risberg; Anna-Malin Linde; Department of Microbiology; Karin Tegmark-Wisell; Maria Lind Karlberg; Mattias Haukland; Mia Brytting; Olov Svartstrom; Oskar Karlsson Lindsjo; Reza Advani; Sandra Broddesson; The Public Health Agency of Sweden; Theresa Enkirch
EPI_ISL_842882, EPI_ISL_842908, EPI_ISL_842929, EPI_ISL_842931, EPI_ISL_842935 to 842936, EPI_ISL_842938, EPI_ISL_842941, EPI_ISL_842953, EPI_ISL_842957, EPI_ISL_842966, EPI_ISL_842968, EPI_ISL_842975, EPI_ISL_842981, EPI_ISL_842986, EPI_ISL_842996, EPI_ISL_843000, EPI_ISL_843003, EPI_ISL_843008, EPI_ISL_843015, EPI_ISL_843017, EPI_ISL_843019, EPI_ISL_843025, EPI_ISL_843027, EPI_ISL_843034 to 843035, EPI_ISL_843041 to 843043, EPI_ISL_843045 to 843047, EPI_ISL_843049, EPI_ISL_843054 to 843057, EPI_ISL_843059 to 843060, EPI_ISL_843062, EPI_ISL_843064, EPI_ISL_843068, EPI_ISL_843070 to 843075, EPI_ISL_843080 to 843086, EPI_ISL_843088 to 843089, EPI_ISL_843092 to 843094, EPI_ISL_843096 to 843102, EPI_ISL_843104, EPI_ISL_843106 to 843112, EPI_ISL_843114 to 843116, EPI_ISL_843119, EPI_ISL_843123 to 843126, EPI_ISL_843131 to 843132, EPI_ISL_843135, EPI_ISL_843137, EPI_ISL_843139 to 843140, EPI_ISL_843142 to 843143, EPI_ISL_843147, EPI_ISL_843149 to 843150, EPI_ISL_865488 to 865490, EPI_ISL_865493 to 865504, EPI_ISL_865507 to 865509, EPI_ISL_865511 to 865514, EPI_ISL_865517, EPI_ISL_865519 to 865521, EPI_ISL_865524, EPI_ISL_865527, EPI_ISL_865529 to 865532, EPI_ISL_865535 to 865537, EPI_ISL_865541 to 865542, EPI_ISL_865544, EPI_ISL_865546 to 865548, EPI_ISL_865551 to 865553, EPI_ISL_865555, EPI_ISL_865559 to 865560, EPI_ISL_865568 to 865570, EPI_ISL_865572, EPI_ISL_865574 to 865576, EPI_ISL_865578 to 865580, EPI_ISL_865583, EPI_ISL_919783, EPI_ISL_919785 to 919786, EPI_ISL_919793, EPI_ISL_919797 to 919798, EPI_ISL_919801, EPI_ISL_919803 to 919809, EPI_ISL_919811, EPI_ISL_919813, EPI_ISL_919816, EPI_ISL_919818, EPI_ISL_919821 to 919823, EPI_ISL_919825 to 919832, EPI_ISL_919834 to 919835, EPI_ISL_919839 to 919841, EPI_ISL_919844 to 919845, EPI_ISL_919847, EPI_ISL_919850 to 919853, EPI_ISL_919855, EPI_ISL_949748, EPI_ISL_949750 to 949755, EPI_ISL_949761, EPI_ISL_949763, EPI_ISL_949769, EPI_ISL_949771 to 949774, EPI_ISL_949780, EPI_ISL_949782 to 949783, EPI_ISL_997615 to 997617, EPI_ISL_997619 to 997620, EPI_ISL_997623 to 997626, EPI_ISL_997629 to 997630, EPI_ISL_997634, EPI_ISL_997636 to 997642, EPI_ISL_997644 to 997656, EPI_ISL_997658 to 997665, EPI_ISL_997667, EPI_ISL_997670 to 997671, EPI_ISL_997673 to 997674			
see above	Barts Health NHS Trust	COVID-19 Genomics UK (COG-UK) Consortium	BROAD; Beatrix; CUTINO-MOGUEL; Claire; David; Dola; HARRINGTON; KELE; KULASEGARAN-SHYLINI; Maria-Teresa; OWOYEMI; Raghavendran; SHYLINI
EPI_ISL_541752, EPI_ISL_541754 to 541755, EPI_ISL_541757, EPI_ISL_541759 to 541766, EPI_ISL_541768 to 541772, EPI_ISL_541775 to 541782	Barts Health NHS Trust	Wellcome Sanger Institute for the COVID-19 Genomics UK (COG-UK) consortium	Beatrix Kele; Cordelia Langford; David Harrington and Alex Alderton; David K. Jackson; Dominic Kwiatkowski; Ewan Harrison; Ian Johnston; John Sillitoe on behalf of the Wellcome Sanger Institute COVID-19 Surveillance Team; Mark Hopkins; Roberto Amato; Sonia Goncalves; Teresa Cutino-Moguel
EPI_ISL_444022, EPI_ISL_445078 to 445084, EPI_ISL_501167, EPI_ISL_501169, EPI_ISL_501171 to 501173	Baylor College of Medicine	Baylor College of Medicine: HGSC	David Henke; Donna Muzny; Erin Nicholson; George Weissenberger; Ginger Metcalf; Harsha Doddapaneni; Hsu Chao; Hua Shen; Joseph F. Petrosino; Kavya Kottapalli; Kristi L. Hoffman; Matthew C. Ross; Matthew Wong; Pedro Piedra; Qingchang Meng; Richard Sucgang; Sara J.J. Cregeen; Sejal Salvi; Tulin Ayvaz; Vasanthi Avadhanula; Vipin Menon; Yimiti Meiheerguli; Zeineen Momin
EPI_ISL_966310	Baylor Scott & White - Temple	Baylor Scott & White - Temple	Ari Rao; Kimberly Walker; Linden Morales; Marcus Volz; Shelby Hendrickson
EPI_ISL_925208, EPI_ISL_943550	Baylor Scott & White-Temple	Baylor Scott & White-Temple	Ari Rao; Kimberly Walker; Linden Morales; Marcus Volz; Shelby Hendrickson
EPI_ISL_700553	Beaufort West CDC wc BWC	NHLS/UCT	Arash Iranzadeh; Bruna Galvao; Carolyn Williamson; Deelan Doolabh; Diana Hardie; Innocent Mudau; Kruger Marais; Lynn Tyers; Marvin Hsiao; Stephen Korsman
EPI_ISL_640031, EPI_ISL_640047	Beaufort West Hospital wc BWH	NHLS/UCT	Arash Iranzadeh; Bruna Galvao; Carolyn Williamson; Deelan Doolabh; Diana Hardie; Innocent Mudau; Kruger Marais; Lynn Tyers; Marvin Hsiao; Stephen Korsman
EPI_ISL_412386	Beijing Ditan Hospital, Capital Medical University	National Institute for Communicable Disease Control and Prevention, Chinese Center for Disease Control and Prevention	Biao Kan; Chuansheng Li; Fangfang Jin; Haijian Zhou; Haofeng Xiong; Hebing Guo; Huizhu Wang; Jianbo Tan; Jie Gong; Jingjing Hao; Jingyuan Liu; Lili Gao; Lin Pu; Ming Zhang; Pan Xiang; Ruihong Li; Xiaoping Chen; Xin Lu; Xinmin Xu; Yajie Wang; Yanwen Xiong; Yao Sun; Yufeng Liu
EPI_ISL_424355 to 424360, EPI_ISL_529213 to 529217	Beijing Institute of Microbiology and Epidemiology	Beijing Institute of Microbiology and Epidemiology	B. and Cui, Y.; Cui, Y.; Fan; Fan, H.; Guo, Y.; Hang; Hou, J.; Li; Li, B.; Mi, Z.; Mu, J.; Qin, E.; Song; Song, Y.; Teng; Teng, Y.; Wu, Y.; Xu, Z.; Yajun.; Yang, R.; Yong, Y.; Yue; Zhang, X.
EPI_ISL_509711 to 509714	Belize Ministry of Health	Pathogen Discovery, Respiratory Viruses Branch, Division of Viral Diseases, Centers for Disease Control and Prevention	Anna Uehara; Clinton Paden; Haibin Wang; Jing Zhang; Krista Queen; Suxiang Tong; Yan Li; Ying Tao
EPI_ISL_631580, EPI_ISL_631804, EPI_ISL_631838, EPI_ISL_632068 to 632069, EPI_ISL_632098, EPI_ISL_632121, EPI_ISL_632136			
see above	Bellevue Hospital Center	New York City Public Health Laboratory	Jade Wang; et al.
EPI_ISL_569609	Bennet County Hospital	South Dakota Public Health Laboratory	Jacob Garfin; Matt Plumb; Xiong Wang; and Chris Carlson
EPI_ISL_569618 to 569619	Bethel Lutheran Home	South Dakota Public Health Laboratory	Jacob Garfin; Matt Plumb; Xiong Wang; and Chris Carlson
EPI_ISL_457824	Bezmialem Vakif University, Dept Microbiology, Medical School, Fatih, Istanbul, Turkey	Bezmialem Vakif University, Medical School & Beykoz Institute of Life Sciences & Biotechnology	Bilge Sumbul; Elif Karaaslan; Filiz Guney; Mehmet Z. Doymaz; Merve Kalkan; Nesibe Cetin
EPI_ISL_735495	Bhashabir M A Wadud RT-PCR Lab, Chandpur	Central Biological Research Laboratory and Department of Biochemistry and Molecular Biology	H. M. Abdullah Al Masud; Imam Hossen; Md. Arif Hossain; Md. Imranul Hoq; Md. Khondakar Raziur Rahman; Md. Omer Faruq; Mohammad Omar Faruque; Robiul Hasan Bhuiyan; Sajib Rudra; Shanta Paul
EPI_ISL_955232, EPI_ISL_955258 to 955260	BioReference Lab	NJ Public Health and Environmental Laboratories	Byeong Jeong; Dana Woell; Lindsey Bodnar; Shiv Verma
EPI_ISL_861865 to 861866, EPI_ISL_896095	Bioanalytika AG	University Hospital Basel, Clinical Bacteriology	Adrian Egli; Adrian Härri; Alfredo Mari; Hans Hirsch; Helena MB Seth-Smith; Julia Bielicki; Karoline Leuzinger; Madlen Stange; Manuel Battegay; Tim Roloff
EPI_ISL_900515 to 900520	Bioesterel	CNR Virus des Infections Respiratoires - France SUD	Antonin Bal; Bruno Lina; Gregory Destras; Gwendolyne Burfin; Géraldine Gonfrier; Hadrien Règue; Laurence Josset; Martine Valette; Quentin Semanas; Sylvie Larrat; Valérie Giordanengo
EPI_ISL_814417 to 814421, EPI_ISL_814424 to 814438, EPI_ISL_814517 to 814522, EPI_ISL_814525, EPI_ISL_816830, EPI_ISL_816844, EPI_ISL_816846 to 816847, EPI_ISL_816849, EPI_ISL_816851, EPI_ISL_816862, EPI_ISL_816867, EPI_ISL_816871 to 816872, EPI_ISL_816874, EPI_ISL_816877, EPI_ISL_816882 to 816883, EPI_ISL_816886 to 816887, EPI_ISL_816889, EPI_ISL_816898 to 816899, EPI_ISL_816901, EPI_ISL_816903, EPI_ISL_816905, EPI_ISL_816907, EPI_ISL_816912 to 816914, EPI_ISL_816916 to 816917, EPI_ISL_816919 to 816921, EPI_ISL_816923 to 816925, EPI_ISL_816931 to 816932, EPI_ISL_816934, EPI_ISL_816938, EPI_ISL_816940 to 816941, EPI_ISL_816943, EPI_ISL_816946 to 816952, EPI_ISL_816954 to 816955, EPI_ISL_816965 to 816966, EPI_ISL_816968, EPI_ISL_816970, EPI_ISL_816972, EPI_ISL_816974, EPI_ISL_816979 to 816981, EPI_ISL_816983 to 816984, EPI_ISL_816986 to 816988, EPI_ISL_816990 to 816992, EPI_ISL_816998 to 816999, EPI_ISL_817001, EPI_ISL_817005, EPI_ISL_817007 to 817008, EPI_ISL_817010, EPI_ISL_817013 to 817019, EPI_ISL_817021 to 817022, EPI_ISL_817032, EPI_ISL_817035 to 817036, EPI_ISL_817038, EPI_ISL_817040, EPI_ISL_817044, EPI_ISL_817049, EPI_ISL_817051 to 817053, EPI_ISL_817055, EPI_ISL_817057, EPI_ISL_817060 to 817062, EPI_ISL_817065, EPI_ISL_817070 to 817071, EPI_ISL_817073 to 817074, EPI_ISL_817079, EPI_ISL_817082 to 817084, EPI_ISL_817087, EPI_ISL_817089 to 817091, EPI_ISL_817093, EPI_ISL_817095 to 817099, EPI_ISL_817103 to 817105, EPI_ISL_817107, EPI_ISL_817110 to 817111, EPI_ISL_817115, EPI_ISL_817119 to 817121, EPI_ISL_842362 to 842363, EPI_ISL_842365, EPI_ISL_842367, EPI_ISL_842369, EPI_ISL_842371, EPI_ISL_842376 to 842378, EPI_ISL_842380 to 842381, EPI_ISL_842383 to 842385, EPI_ISL_842387 to 842389, EPI_ISL_842395 to 842396, EPI_ISL_842398, EPI_ISL_842402, EPI_ISL_842404 to 842405, EPI_ISL_842407, EPI_ISL_842410 to 842416, EPI_ISL_842418 to 842419, EPI_ISL_842427, EPI_ISL_842429 to 842430, EPI_ISL_842432 to 842433, EPI_ISL_842435 to 842437, EPI_ISL_842439, EPI_ISL_842441 to 842442, EPI_ISL_842450 to 842452, EPI_ISL_842454, EPI_ISL_842456 to 842460, EPI_ISL_842464, EPI_ISL_842467, EPI_ISL_842469, EPI_ISL_842472 to 842474, EPI_ISL_842481, EPI_ISL_842483, EPI_ISL_842485 to 842486, EPI_ISL_842497 to 842498, EPI_ISL_842500, EPI_ISL_842502 to 842503, EPI_ISL_842511, EPI_ISL_842515, EPI_ISL_842518, EPI_ISL_842524, EPI_ISL_842527 to 842528, EPI_ISL_842530 to 842531, EPI_ISL_842535, EPI_ISL_842537, EPI_ISL_842539 to 842540, EPI_ISL_842549, EPI_ISL_842554 to 842562, EPI_ISL_842579, EPI_ISL_842582, EPI_ISL_842595, EPI_ISL_842598, EPI_ISL_868724, EPI_ISL_868730 to 868731, EPI_ISL_868733 to 868734, EPI_ISL_868736, EPI_ISL_868738, EPI_ISL_868740, EPI_ISL_868743, EPI_ISL_868751, EPI_ISL_868783, EPI_ISL_868842, EPI_ISL_868848, EPI_ISL_868850 to 868853, EPI_ISL_868857 to 868861, EPI_ISL_868863, EPI_ISL_868868 to 868869, EPI_ISL_868874, EPI_ISL_868880 to 868882, EPI_ISL_868884, EPI_ISL_868888, EPI_ISL_868890, EPI_ISL_868898, EPI_ISL_868907, EPI_ISL_868913, EPI_ISL_868916, EPI_ISL_868924, EPI_ISL_868927 to 868929, EPI_ISL_868955, EPI_ISL_868960, EPI_ISL_868962 to 868963, EPI_ISL_868966, EPI_ISL_868971, EPI_ISL_868975, EPI_ISL_868981, EPI_ISL_868988, EPI_ISL_868995, EPI_ISL_869005, EPI_ISL_869007, EPI_ISL_869022, EPI_ISL_869025, EPI_ISL_869027, EPI_ISL_869031 to 869035, EPI_ISL_869038 to 869039, EPI_ISL_869041, EPI_ISL_869043 to 869044, EPI_ISL_869049, EPI_ISL_869059, EPI_ISL_869061 to 869062, EPI_ISL_869067 to 869069, EPI_ISL_869071 to 869075, EPI_ISL_869078, EPI_ISL_924438, EPI_ISL_924441, EPI_ISL_924443, EPI_ISL_924447 to 924451, EPI_ISL_924454 to 924455, EPI_ISL_924457, EPI_ISL_924459 to 924460, EPI_ISL_924465, EPI_ISL_924475, EPI_ISL_924477 to 924478, EPI_ISL_924483 to 924485, EPI_ISL_924487 to 924491, EPI_ISL_924494, EPI_ISL_924502 to 924505, EPI_ISL_924507 to 924509, EPI_ISL_924511 to 924515, EPI_ISL_924519 to 924527, EPI_ISL_924532 to 924533, EPI_ISL_924537 to 924540, EPI_ISL_924542, EPI_ISL_924544 to 924545, EPI_ISL_924547 to 924550, EPI_ISL_924552 to 924556, EPI_ISL_924559 to 924560, EPI_ISL_924567 to 924568, EPI_ISL_924570, EPI_ISL_924572, EPI_ISL_924574 to 924575, EPI_ISL_924578 to 924583, EPI_ISL_924586, EPI_ISL_924591, EPI_ISL_924594, EPI_ISL_924596 to 924597, EPI_ISL_924599 to 924600, EPI_ISL_924602, EPI_ISL_924604, EPI_ISL_924632 to 924633, EPI_ISL_924641, EPI_ISL_924645, EPI_ISL_924647 to 924650, EPI_ISL_924659 to 924667, EPI_ISL_924669 to 924670, EPI_ISL_924673, EPI_ISL_924676, EPI_ISL_924680, EPI_ISL_924682, EPI_ISL_924685 to 924687, EPI_ISL_924692, EPI_ISL_924694 to 924698, EPI_ISL_924700 to 924705, EPI_ISL_924707 to 924708, EPI_ISL_924711 to 924717, EPI_ISL_924719 to 924720, EPI_ISL_924722, EPI_ISL_924734, EPI_ISL_924737, EPI_ISL_924741 to 924742, EPI_ISL_924748, EPI_ISL_924750, EPI_ISL_924753 to 924757, EPI_ISL_924766, EPI_ISL_924775, EPI_ISL_924777 to 924778, EPI_ISL_924781 to 924782, EPI_ISL_924790 to 924791, EPI_ISL_924813, EPI_ISL_924817, EPI_ISL_924820, EPI_ISL_924826, EPI_ISL_924835 to 924836, EPI_ISL_924839 to 924840, EPI_ISL_924842 to 924847, EPI_ISL_924849 to 924850, EPI_ISL_924852, EPI_ISL_924854 to 924860, EPI_ISL_924864, EPI_ISL_924867, EPI_ISL_924869 to 924873, EPI_ISL_924875, EPI_ISL_924877 to 924879, EPI_ISL_924881 to 924889, EPI_ISL_924892, EPI_ISL_924900, EPI_ISL_924902, EPI_ISL_924904 to 924906, EPI_ISL_924908, EPI_ISL_924910 to 924917, EPI_ISL_924925 to 924926, EPI_ISL_924929 to 924931, EPI_ISL_924933, EPI_ISL_924936 to 924947, EPI_ISL_924959, EPI_ISL_924961, EPI_ISL_924964, EPI_ISL_924966, EPI_ISL_924968 to 924974, EPI_ISL_924977, EPI_ISL_924979 to 924981, EPI_ISL_924989, EPI_ISL_924995 to 924997, EPI_ISL_925000, EPI_ISL_925003, EPI_ISL_925005 to 925006, EPI_ISL_925009, EPI_ISL_925011 to 925013, EPI_ISL_953047, EPI_ISL_953050, EPI_ISL_953058, EPI_ISL_953090 to 953094, EPI_ISL_953125, EPI_ISL_953128 to 953129, EPI_ISL_953132, EPI_ISL_953135, EPI_ISL_953138, EPI_ISL_953145, EPI_ISL_953150, EPI_ISL_953153, EPI_ISL_953159, EPI_ISL_953174, EPI_ISL_953179 to 953181, EPI_ISL_953184 to 953186, EPI_ISL_953188, EPI_ISL_953190 to 953193, EPI_ISL_953195, EPI_ISL_953197 to 953206, EPI_ISL_953212, EPI_ISL_953215 to 953221, EPI_ISL_953223 to 953231, EPI_ISL_953233, EPI_ISL_953235 to 953239, EPI_ISL_953249, EPI_ISL_953253, EPI_ISL_953255, EPI_ISL_953257, EPI_ISL_953262 to 953266, EPI_ISL_953276, EPI_ISL_953279, EPI_ISL_953282, EPI_ISL_953284 to 953285, EPI_ISL_1000806, EPI_ISL_1000808, EPI_ISL_1000811 to 1000812, EPI_ISL_1000815 to 1000819, EPI_ISL_1000832 to 1000837, EPI_ISL_1000841 to 1000842, EPI_ISL_1000844, EPI_ISL_1000846 to 1000847, EPI_ISL_1000849, EPI_ISL_1000851, EPI_ISL_1000854 to 1000855, EPI_ISL_1000857 to 1000868, EPI_ISL_1000874, EPI_ISL_1000876 to 1000880, EPI_ISL_1000897, EPI_ISL_1000899, EPI_ISL_1000901 to 1000902, EPI_ISL_1000904 to 1000905, EPI_ISL_1000909, EPI_ISL_1000911 to 1000913, EPI_ISL_1000917, EPI_ISL_1000919 to 1000925, EPI_ISL_1000927 to 1000933, EPI_ISL_1000935, EPI_ISL_1000944 to 1000945, EPI_ISL_1000947, EPI_ISL_1000949 to 1000950, EPI_ISL_1000953, EPI_ISL_1000955 to 1000957, EPI_ISL_1000961 to 1000962, EPI_ISL_1000965 to 1000966			
see above	Bioinformatics and Biostatistics Lab, Advanced Sequencing Facility	COVID-19 Genomics UK (COG-UK) Consortium	Aengus Stewart; Chelsea Sawyer; Harshil Patel; Jerome Nicod; Laura Cubitt; Margaret Crawford
EPI_ISL_759866 to 759867	Bioinformatics, National institute of traditional medicine	Bioinformatics, National institute of traditional medicine	Chattopadhyay, D.; D.R.; Harish; Ishwar, S.; Umashankar, V.
EPI_ISL_429992 to 430004, EPI_ISL_430006 to 430007, EPI_ISL_430009, EPI_ISL_430011 to 430012, EPI_ISL_430014 to 430015, EPI_ISL_434516, EPI_ISL_450186 to 450188, EPI_ISL_635777 to 635782, EPI_ISL_636267 to 636360, EPI_ISL_636362 to 636386, EPI_ISL_636388 to 636389, EPI_ISL_636391 to 636458,			

EPI_ISL_730229 to 730261, EPI_ISL_730263 to 730290, EPI_ISL_730372 to 730399, EPI_ISL_730401 to 730429, EPI_ISL_730431 to 730459, EPI_ISL_730461 to 730472, EPI_ISL_730474 to 730542, EPI_ISL_730544, EPI_ISL_730546, EPI_ISL_730548 to 730563, EPI_ISL_755141 to 755145, EPI_ISL_755210 to 755214, EPI_ISL_755232 to 755268, EPI_ISL_878440, EPI_ISL_878443, EPI_ISL_878445, EPI_ISL_878448, EPI_ISL_878450, EPI_ISL_878452, EPI_ISL_878457, EPI_ISL_878460, EPI_ISL_878463, EPI_ISL_878465, EPI_ISL_878470, EPI_ISL_878474, EPI_ISL_878477, EPI_ISL_878480, EPI_ISL_878483, EPI_ISL_878485, EPI_ISL_878488, EPI_ISL_878491 to 878492, EPI_ISL_878500, EPI_ISL_878503, EPI_ISL_878506, EPI_ISL_878509, EPI_ISL_878511, EPI_ISL_878514, EPI_ISL_878516, EPI_ISL_878519, EPI_ISL_878522, EPI_ISL_878524, EPI_ISL_878526, EPI_ISL_878571, EPI_ISL_878574, EPI_ISL_878576, EPI_ISL_878579, EPI_ISL_878581			
see above	Biolab Diagnostic Laboratories	Andersen lab at Scripps Research	Ahmad Tibi; Amid Abdelnour with SEARCH Alliance San Diego; Issa Abu-Dayyeh; Lama Hussein; Lina Mohammad; Zein Naber
EPI_ISL_977537, EPI_ISL_977600	Biolab Diagnostic Laboratories	Biolab Diagnostic Laboratories	Ahmad Tibi; Amid Abdelnour; Badia Saddedin; Issa Abu-Dayyeh; Lama Hussein; Shayma Ali
EPI_ISL_935046	Biolab Diagnostic Laboratories	Princess Haya Biotechnology Center/ Jordan University of Science & Technology	Amid Abdelnour; Areej Alquran; Hazem Haddad; Issa Abu-Dayyeh; Maha Karam; Moh"D Al-Zghoul; Mohammad Alboom; Mustafa Ababneh; Saied Jaradat; Shereen Issa; Suha Hasan
EPI_ISL_900514	Biolittoral	CNR Virus des Infections Respiratoires - France SUD	Antonin Bal; Bruno Lina; Gregory Destras; Gwendolyne Burfin; Hadrien Règue; Laurence Josset; Martine Valette; Quentin Semanas
EPI_ISL_527007	Biological Prevention, Army	Biological Prevention, Army	A.E.; A.F.; A.M.; Ageez; B.E.; Elhoseiny; Gad; Harty; M.D.; M.F.; M.G.; Seadawy; Shabaan; Shamel
EPI_ISL_526975 to 527006	Biological prevention, army	Biological prevention, army	A.E.; A.F.; A.M.; Ageez; B.E.; Elhoseiny; Elhosieny; Gad; Harty; M.D.; M.F.; M.G.; Seadawy; Shabaan; Shamel
EPI_ISL_510526, EPI_ISL_510532	Biological prevention, army	Biological prevention, army	A.A.; A.F.; A.M. and Soliman; Ali; Amer; B.E.; B.S.; ELnabrawy; ElGohary; Elhoseiny; Elhoseny; Elnagdy; Elnakeeb; Gad; H.A.; Harty; Hassan; Kandeil; Karam; M.A.; M.D.; M.F.; M.G.; M.M. and Gad; Raouf; Seadawy; Shamel; T.A.; W.A.; Y.A.; k.E.
EPI_ISL_582030	Biology Department, College of Science, Al-Muthanna University	International Centre for Genetic Engineering and Biotechnology (ICGEB) and ARGO Open Lab Platform	Alessandro Marcello; Danilo Licastro; Nihad Al-Rashedi; Simeone Dal Monego; Sreejith Rajasekharan
EPI_ISL_505003	Biology Dpt	Microbiology and Infections Diseases	Annabelle Garnier; Audrey Ferrier-Rembert; Clarisse Vigne; Emilie Tessier; Emmanuelle Billon-Denis; Flora Nolent; Isabelle Drouet; Jean-Nicolas Tournier; Jessica Denis; Laurence Cheutin; Noémie Verguet; Olivier Ferraris; Olivier Gorgé
EPI_ISL_1015380	Biology Lab, HIA BEGIN	IRBA, 2MI	GORGE O.; MERENS-GONTIER A.; NOLENT F.; SARILAR V.
EPI_ISL_1015382	Biology Lab, HIA CLERMONT-TONNERRE	IRBA, 2MI	DESIDERI C.; GORGE O.; NOLENT F.; SARILAR V.
EPI_ISL_1015378, EPI_ISL_1015383 to 1015387	Biology Lab, HIA PERCY	IRBA, 2MI	FOISSAUD V.; GORGE O.; NOLENT F.; SARILAR V.
EPI_ISL_1015379	Biology Lab, HIA STE-ANNE	IRBA, 2MI	GORGE O.; JANVIER F.; NOLENT F.; SARILAR V.
EPI_ISL_516079 to 516088	Biomedical Sciences and Public Health, Polytechnic University of Marche	Biomedical Sciences and Public Health, Polytechnic University of Marche	Alessandrini, F.; Bagnarelli, P.; Caucci, S.; Di Sante, L.; Melchionda, F.; Menzo, S.; Onofri, V.; Tagliabracci, A.; Turchi, C.
EPI_ISL_985058 to 985104	Biorepository and Clinical Virology Laboratory	Ozer Lab	Adeola A. Fowotade; Babafemi O. Taiwo; Egon A. Ozer; Ewean C. Omoruyi; Johnson A. Adeniji; Judd F. Hultquist; Lacy M. Simons; Olubusuyi M. Adewumi; Ramon Lorenzo-Redondo
EPI_ISL_458287	Biosafety Department PCL3	Biosafety Department PCL3	A. and El Kabbaj, S.; Lemriss, S.; Souiri
EPI_ISL_450813, EPI_ISL_475540	Bla Kustens halsocentral	The Public Health Agency of Sweden	Anna Risberg; Anna-Malin Linde; Karin Tegmark-Wisell; Maria Lind Karlberg; Mattias Haukland; Mia Brytting; Olof Norrby; Olov Svartstrom; Oskar Karlsson Lindsjo; Reza Advani; Sandra Broddesson; Theresa Enkirch
EPI_ISL_747238	Bogor Public Health	West Java Health Laboratory; School of Life Sciences and Technology, Institut Teknologi Bandung	Azzania Fibriani; Cut Nur Cinthia Alamanda; Ema Rahmawati; Isak Solihin; Karimatu Khoirunnisa; Miftahul Faridl; Rifky Waluyajati Rachman; Rini Robiani; Ryan Bayusantika Ristandi
EPI_ISL_700589	Bongolethu Clinic wc BLC	NHLS/UCT	Arash Iranzadeh; Bruna Galvao; Carolyn Williamson; Deelan Doolabh; Diana Hardie; Houriiyah Tegally; Innocent Mudau; Kruger Marais; Lynn Tyers; Marvin Hsiao; Stephen Korsman
EPI_ISL_596453	Booali laboratory, Qom, Iran. Department of Virology, School of Public Health, Tehran University of Medical Sciences, Tehran, Iran.	Genetics Research Center, University of Social Welfare and Rehabilitation Sciences	Ali Jafarpour; Azam Ghaziasadi; Hossein Najmbadi; Khadijeh Jalalvand; Kimia Kahrizi; Marzieh Mohseni; Mohammad Khazeni; Seyed Amir Momeni; Seyed Mohammad Jazayeri; Seyedeh elham Mortazavi; Zohreh Fattahi
EPI_ISL_718167, EPI_ISL_718174, EPI_ISL_718176, EPI_ISL_718178, EPI_ISL_718182, EPI_ISL_718185, EPI_ISL_718191 to 718192, EPI_ISL_718197 to 718198, EPI_ISL_718203 to 718205, EPI_ISL_718216, EPI_ISL_718218, EPI_ISL_718222			
see above	Borneo Medical Centre	Institute of Health and Community Medicine	Chan Chia Jui; Chua Hock Hin; David Perera; Ooi Mong How; Tonnii Sia Loong Loong; Wong Jyn Shan; Wong Kiing Aik
EPI_ISL_640075	Bothasig CDC wc BLD	NHLS/UCT	Arash Iranzadeh; Bruna Galvao; Carolyn Williamson; Deelan Doolabh; Diana Hardie; Innocent Mudau; Kruger Marais; Lynn Tyers; Marvin Hsiao; Stephen Korsman
EPI_ISL_871872	Botswana Harvard HIV Reference Laboratory	Botswana Harvard HIV Reference Laboratory	Boitumelo Zuze; Botshelo Radibe; David Lawrence; Dorcas Maruapula; Joseph; Makhema; Mosepele Mosepele; Roger Shapiro; Shahin Lockman; Sikhulile Moyo; Simani Gaseitsiwe; Wonderful Choga
EPI_ISL_437434	Bozeman Water Reclamation Facility	Wiedenheft lab, Montana State University	Anna Nemudraia; Artem Nemudryi; Blake Wiedenheft; Kevin Surya; Murat Buyukyoruk; Royce Wilkinson; Tanner Wiegand
EPI_ISL_745190	Brackengate Field Hospital COVID-19 wc BRG	National Health Laboratory Service (NHLS), Tygerberg	Bronwyn Kleinhans; Eduan Wilkindon; Gert van Zyl; Houriiyah Tegally; Kayla Delaney; Susan Engelbrecht; Tulio de Oliveira; Wolfgang Preiser
EPI_ISL_479777 to 479778, EPI_ISL_479780 to 479786, EPI_ISL_479788	Breuer Lab, UCL	Breuer Lab, UCL	Breuer Lab
EPI_ISL_420799 to 420801	Brian D. Allgood Army Community Hospital	Pathogen Discovery, Respiratory Viruses Branch, Division of Viral Diseases, Centers for Disease Control and Prevention	Alison S. Laufer Halpin; Anne Uehara; Christopher A. Elkins; Clinton R. Paden; Haibin Wang; Jasmine Padilla; Jing Zhang; Justin Lee; Krista Queen; Mary S. Keckler; Rachel Marine; Suxiang Tong; Yan Li; Ying Tao
EPI_ISL_593478 to 593480, EPI_ISL_593553, EPI_ISL_593556	Brigham and Women's Hospital	Jonathan Li Laboratory	James Regan; Jonathan Z. Li; Manish C. Choudhary
EPI_ISL_632288 to 632309, EPI_ISL_648265, EPI_ISL_648267, EPI_ISL_648270	Brigham and Women's Hospital	Jonathan Li laboratory	Choudhary MC; Douglas S Kwon; Esmaeilzadeh E; Etemad B; George Eng; Jonathan Z. Li; Li JZ; Manish C. Choudhary; Mohammadi A; Regan J; Upasana D. Adhikari
EPI_ISL_765583 to 765586, EPI_ISL_765588 to 765600, EPI_ISL_765602 to 765612	Brigham and Womens Hospital	Infectious Disease Program, Broad Institute of Harvard and MIT	A.E.; Adams, G.; Anahtar, M.; B.L.; B.W.; Bauer, M.; Birren; Branda, J.; Carter, A.; Cerrato, F.; Chaluvadi, S.; Chapman; Cusick, C.; D.J.; DeRuff, K.; E. and Sabeti; Flowers, K.; Gallagher, G.; Gladden-Young, A.; Gnirke, A.; Harris, J.; J.E.; K.J.; LaRocque, R.; Lagerborg, K.; Lemieux; Lin; Loreth, C.; MacInnis; Neumann, A.; Normandin, E.; P.C.; Park; Pierce, V.; Reilly, S.; Rosenberg; Rudy, M.; Ryan, E.; S.B.; Shaw, B.; Siddle; Slater, D.; Smole, S.; Tomkins-Tinch, C.; Turbett, S.
EPI_ISL_1011681, EPI_ISL_1011702	Broad Institute Clinical Research Sequencing Platform	Infectious Disease Program, Broad Institute of Harvard and MIT	Adams, G.; B.L.; B.W.; Bauer, M.; Birren; Carter, A.; Chaluvadi, S.; D.J.; DeRuff, K.; Gallagher, G.; Gladden-Young, A.; J.E.; K.J.; Lagerborg, K.; Lemieux; Loreth, C.; MacInnis; Normandin, E.; P.C.; Park; Reilly, S.; Rudy, M.; Siddle; Smole, S.; Tomkins-Tinch, C.; and Sabeti
EPI_ISL_631553, EPI_ISL_631562, EPI_ISL_631565, EPI_ISL_631573, EPI_ISL_631800, EPI_ISL_631803, EPI_ISL_631805 to 631807, EPI_ISL_631819, EPI_ISL_631829, EPI_ISL_631864, EPI_ISL_631976, EPI_ISL_631978, EPI_ISL_631981, EPI_ISL_631984, EPI_ISL_632077 to 632078, EPI_ISL_632100, EPI_ISL_632114, EPI_ISL_632123, EPI_ISL_632142, EPI_ISL_632155 to 632156			
see above	Brookdale University Hospital Medical Center	New York City Public Health Laboratory	Jade Wang; et al.
EPI_ISL_631508	Brooklyn Hospital Center	New York City Public Health Laboratory	Jade Wang; et al.
EPI_ISL_603248, EPI_ISL_605791	Brotman Baty Institute for Precision Medicine	Brotman Baty Institute for Precision Medicine	Benjamin Pelle; Caitlin R. Wolf; Chris D. Frazar; Christina M. Lockwood; Deborah A. Nickerson; Erica Ryke; Helen Y. Chu; Jay Shendure; Jennifer K. Logue; Jover Lee; Lea M. Starita; Mark J. Rieder; Peter D. Han; Trevor Bedford
EPI_ISL_414520 to 414521, EPI_ISL_732533 to 732564, EPI_ISL_732658, EPI_ISL_755639	Bundeswehr Institute of Microbiology	Bundeswehr Institute of Microbiology	Alexandra Rehn; Christina Bugert; Elham Khatamzas; Enrico Georgi; Joachim Bugert; Malena Bestehorn-Willmann; Markus Antwerpen; Markus H Antwerpen and Roman Wölfel; Mathias C Walter; Mathias Walter; Michael von Bergwelt-Baildon; Roman Wölfel; Sabine Zange
EPI_ISL_996417	Bundeswehrkrankenhaus Hamburg	Bundeswehr Institute of Microbiology	Alexandra Rehn; Enrico Georgi; Malena Bestehorn-Willmann; Markus Antwerpen; Mathias Walter; Mike Pillukat; Roman Wölfel; Sabine Zange
EPI_ISL_915432	Bundeswehrkrankenhaus Westerstede	Bundeswehr Institute of Microbiology	Alexandra Rehn; Enrico Georgi; Klaus Peter Ebert; Malena Bestehorn-Willmann; Markus Antwerpen; Mathias Walter; Roman Wölfel; Sabine Zange
EPI_ISL_450832	Byjorden vardcentral	The Public Health Agency of Sweden	Anna Risberg; Anna-Malin Linde; Karin Tegmark-Wisell; Maria Lind Karlberg; Mia Brytting; Olov Svartstrom; Oskar Karlsson Lindsjo; Pernilla Brunman;

			Theresa Enkirch
EPI_ISL_524476	Bülach Hospital	Institute of Medical Virology, University of Zurich	Alexandra Trkola; Andrea Zbinden; Fiona Steiner; Gabriela Ziltener; Jon Huder; Jürg Böni; Maryam Zaheri; Michael Huber; Patrick Redli; Riccarda Capaul; Stefan Schmutz; Verena Kufner
EPI_ISL_445320	C.C.SALUD FAMILIAR PADRE FELIX DONOSO G.	Instituto de Salud Publica de Chile	Alejandra Acevedo; Andrés E Castillo; Bárbara Parra; Carolina Tambley; Gabriel Leal; Jaime Lagos; Jorge Fernandez; Loredana Arata; Patricia Bustos; Paz Tapia; Rodrigo Fasce; Winston Andrade
EPI_ISL_445318	C.DE SALUD FAMILIAR PABLO NERUDA	Instituto de Salud Publica de Chile	Alejandra Acevedo; Andrés E Castillo; Bárbara Parra; Carolina Tambley; Gabriel Leal; Jaime Lagos; Jorge Fernandez; Loredana Arata; Patricia Bustos; Paz Tapia; Rodrigo Fasce; Winston Andrade
EPI_ISL_912554 to 912556, EPI_ISL_912573	C.H.R. d'Orléans - Hôpital de la Source	National Reference Center for Viruses of Respiratory Infections, Institut Pasteur, Paris	Angela Brisebarre; Camille Capel; Etienne Simon-Lorière; Guinard Jérome; Marion Barbet; Maud Vanpeene; Méline Bizard; Sylvie Behillil; Sylvie van der Werf; Vincent Enouf
EPI_ISL_539531	C.H.U Nuestra Señora de Candelaria	Instituto de Salud Carlos III	A. Monzón; F. Casas; I; I. Jiménez; Iglesias-Caballero; M. Camarero; M. Cuesta; M. González-Esguevillas; M. Molinero Calamita; M. Zaballos; O. Díez; P. Jiménez; S. Juliá; S. Pozo; S. Varona
EPI_ISL_576177	CA, CDPH, Viral and Rickettsial Disease Laboratory	Pathogen Discovery, Respiratory Viruses Branch, Division of Viral Diseases, Centers for Disease Control and Prevention	Anna Uehara; Brian Lynch; Clinton R. Paden; Haibin Wang; Jing Zhang; Krista Queen; Peter Cook; Suxiang Tong; Yan Li; Ying Tao
EPI_ISL_467516	CAPRISA	KRISP, KZN Research Innovation and Sequencing Platform	Chimukangara B; Giandhari J; Khan S; Lessells R; Mdlalose K; Pillay S; Tegally H; Wilkinson E; York D; de Oliveira T
EPI_ISL_586549 to 586550, EPI_ISL_586555 to 586561	CCC,Veraval	Gujarat Biotechnology Research Centre	A M Kadri; Afzal Ansari; Apurvasinh Puvar; Chaitanya Joshi; Dinesh Kumar; Harsh Bakshi; Janvi Raval; Jignesh Parmar; Jitendra Bamrotia; Komal Patel; Labdhi Pandya; Madhvi Joshi; Maharshi Pandya; Monika Gandhi; Nidhi Patel; Nikha Trivedi; Nitin Savaliya; Pinal Trivedi; R D Dixit; Raghawendra Kumar; Zarna Patel; Zuber Saiyed
EPI_ISL_535662 to 535715	CDPH, Microbial Diseases Laboratory	Pathogen Discovery, Respiratory Viruses Branch, Division of Viral Diseases, Centers for Disease Control and Prevention	Anna Uehara; Brian Lynch; Clinton R. Paden; Haibin Wang; Jing Zhang; Krista Queen; Rachel Marine; Suxiang Tong; Yan Li; Ying Tao
EPI_ISL_486852	CDRI/SGPGI	CSIR-CDRI/SGPGI	Dharam Veer Singh; Rahul Vishvkarma; Rajender Singh; Ravishankar Ramachandran; Saumya Sarkar; Tapas Kumar Kundu; Uday Ghoshal; Ujjala Ghoshal
EPI_ISL_605780	CEIRS Data Processing and Coordinating Center, St. Jude Center of Excellence for Influenza Research and Surveillance (CEIRS)	CEIRS Data Processing and Coordinating Center, St. Jude Center of Excellence for Influenza Research and Surveillance (CEIRS)	A.E.; Ali; El-Guindy; El-Sayes, M.; El-Shesheny, R.; El-Taweel, A.; Gomaa, M.; Kamel; Kandeil, A.; Kayali, G.; Kayed; Kutkat, O.; M.A.; M.N.; Mahmoud; Mahrous, N.; Moatasim, Y.; Mostafa, A.; N.M.; Naguib, A.; Roshdy; S.H.; Shehata, M.; Showky, S.; W.H.; Webby, R.
EPI_ISL_445266 to 445267	CENTRO ONCOLOGICO DEL NORTE	Instituto de Salud Publica de Chile	Alejandra Acevedo; Andrés E Castillo; Bárbara Parra; Carolina Tambley; Gabriel Leal; Jaime Lagos; Jorge Fernandez; Loredana Arata; Patricia Bustos; Paz Tapia; Rodrigo Fasce; Winston Andrade
EPI_ISL_644250 to 644254, EPI_ISL_644256, EPI_ISL_644258	CEPHR / Mater Hospital	Irish Coronavirus Sequencing Consortium - National Virus Reference Laboratory	Alejandro Abner Garcia Leon; Gabriel Gonzalez; Michael Carr; Patrick Mallon
EPI_ISL_644247, EPI_ISL_644255, EPI_ISL_644257, EPI_ISL_644259 to 644345	CEPHR / Vincent's Hospital	Irish Coronavirus Sequencing Consortium - National Virus Reference Laboratory	Alejandro Abner Garcia Leon; Gabriel Gonzalez; Michael Carr; Patrick Mallon
EPI_ISL_861715 to 861716, EPI_ISL_861727 to 861728, EPI_ISL_862042 to 862045	CERBA	CERBA LAB	Costa JM; Haïm-Boukobza S.; Hedbaut E; Lecorche E; Malek Ramdane; Olivi M; Roquebert B; Trombert S; Verdurme L
EPI_ISL_445316	CESFAM BALMACEDA DE RENCA	Instituto de Salud Publica de Chile	Alejandra Acevedo; Andrés E Castillo; Bárbara Parra; Carolina Tambley; Gabriel Leal; Jaime Lagos; Jorge Fernandez; Loredana Arata; Patricia Bustos; Paz Tapia; Rodrigo Fasce; Winston Andrade
EPI_ISL_421455, EPI_ISL_421479 to 421480, EPI_ISL_421485 to 421486	CH Barreiro Montijo	Instituto Nacional de Saude (INSA)	Guiomar et al
EPI_ISL_420041, EPI_ISL_420049 to 420050, EPI_ISL_420056 to 420057, EPI_ISL_421500, EPI_ISL_421509 to 421511, EPI_ISL_428353, EPI_ISL_428359 to 428360, EPI_ISL_443309, EPI_ISL_443316			
see above	CH Compiègne Laboratoire de Biologie	National Reference Center for Viruses of Respiratory Infections, Institut Pasteur, Paris	Angela Brisebarre; Etienne Simon-Lorière; Flora Donati; Marion Barbet; Maud Vanpeene; Mélanie Albert; Méline Bizard; Olivia Raulin; Raulin Olivia; Sylvie Behillil; Sylvie van der Werf; Vincent Enouf
EPI_ISL_860848	CH Dunkerque Laboratoire de Biologie	National Reference Center for Viruses of Respiratory Infections, Institut Pasteur, Paris	Angela Brisebarre; Camille Capel; Etienne Simon-Lorière; Joly Isabelle; Marion Barbet; Maud Vanpeene; Méline Bizard; Sylvie Behillil; Sylvie van der Werf; Vincent Enouf
EPI_ISL_416493, EPI_ISL_420044, EPI_ISL_420053, EPI_ISL_428350	CH Jean de Navarre Laboratoire de Biologie	National Reference Center for Viruses of Respiratory Infections, Institut Pasteur, Paris	Angela Brisebarre; Etienne Simon-Lorière; Flora Donati; Marion Barbet; Maud Vanpeene; Mélanie Albert; Méline Bizard; Mélnie Albert; Sylvie Behillil; Sylvie van der Werf; Vincent Enouf
EPI_ISL_428358, EPI_ISL_428366	CH Jeanne de Navarre Laboratoire de Biologie	National Reference Center for Viruses of Respiratory Infections, Institut Pasteur, Paris	Angela Brisebarre; Etienne Simon-Lorière; Flora Donati; Marion Barbet; Maud Vanpeene; Mélanie Albert; Méline Bizard; Sylvie Behillil; Sylvie van der Werf; Vincent Enouf
EPI_ISL_421482 to 421484	CH VN Gaia - Espinho	Instituto Nacional de Saude (INSA)	Guiomar et al
EPI_ISL_1013103, EPI_ISL_1013108, EPI_ISL_1013121, EPI_ISL_1013131 to 1013133, EPI_ISL_1013135, EPI_ISL_1013146 to 1013156, EPI_ISL_1013159, EPI_ISL_1013161 to 1013162, EPI_ISL_1013164, EPI_ISL_1013172 to 1013173, EPI_ISL_1013177, EPI_ISL_1013182 to 1013204, EPI_ISL_1013207, EPI_ISL_1013224 to 1013233, EPI_ISL_1013237 to 1013238, EPI_ISL_1013244 to 1013354, EPI_ISL_1013357 to 1013364			
see above	CH de Mayotte	National Reference Center for Viruses of Respiratory Infections, Institut Pasteur, Paris	Angela Brisebarre; Camille Capel; Combe Patrice; Etienne Simon-Lorière; Marion Barbet; Maud Vanpeene; Méline Bizard; Sylvie Behillil; Sylvie van der Werf; Vincent Enouf
EPI_ISL_894225, EPI_ISL_909687 to 909701	CH de Mayotte - Laboratoire de Biologie	National Reference Center for Viruses of Respiratory Infections, Institut Pasteur, Paris	Angela Brisebarre; Camille Capel; Combe Patrice; Etienne Simon-Lorière; Marion Barbet; Maud Vanpeene; Méline Bizard; Sylvie Behillil; Sylvie van der Werf; Vincent Enouf
EPI_ISL_912681, EPI_ISL_912907, EPI_ISL_981398 to 981400, EPI_ISL_981464, EPI_ISL_981543, EPI_ISL_982147, EPI_ISL_982305 to 982306			
see above	CH.INTERCOMMUNAL DE CRETEIL	Department of Virology, Henri Mondor University Hospital, Assistance Publique Hôpitaux de Paris, Université Paris-Est Créteil, INSERM U955	Alexandre Soulier; Christophe Rodriguez; Elisabeth Trawinski; Guillaume Gricourt; Jean-Michel Pawlotsky; Melissa N'Debi; Slim Fourati; Vanessa Demontant
EPI_ISL_733569	CHAI WAN FAMILIES CLINIC	Hong Kong Department of Health	Alan K.L. Tsang; Dominic N.C. Tsang; Edman T.K. Lam; Peter C.W. Yip; Rickjason C.W. Chan
EPI_ISL_418006	CHBarreiro Montijo	Instituto Nacional de Saude (INSA)	Guiomar et al
EPI_ISL_872051, EPI_ISL_930604	CHC	GIGA Medical Genomics	Bouchra Boujemla; Cécile Meex; Keith Durkin; Maria Artesi; Marie-Pierre Hayette; Pierrette Melin; Raphaël Boreux; Sébastien Bontems; Vincent Bours
EPI_ISL_981455 to 981456, EPI_ISL_981468, EPI_ISL_981475 to 981476	CHI VILLENEUVE ST GEORGES	Department of Virology, Henri Mondor University Hospital, Assistance Publique Hôpitaux de Paris, Université Paris-Est Créteil, INSERM U955	Alexandre Soulier; Christophe Rodriguez; Elisabeth Trawinski; Guillaume Gricourt; Jean-Michel Pawlotsky; Melissa N'Debi; Slim Fourati; Vanessa Demontant
EPI_ISL_418017	CHMT	Instituto Nacional de Saude (INSA)	Guiomar et al
EPI_ISL_872052 to 872056, EPI_ISL_872117 to 872121, EPI_ISL_872123 to 872126	CHR Citadelle	GIGA Medical Genomics	Bouchra Boujemla; Cécile Meex; Keith Durkin; Maria Artesi; Marie-Pierre Hayette; Pierrette Melin; Raphaël Boreux; Sébastien Bontems; Vincent Bours
EPI_ISL_935674 to 935676, EPI_ISL_935678 to 935680, EPI_ISL_935682 to 935695, EPI_ISL_959600 to 959602, EPI_ISL_965415, EPI_ISL_965421 to 965430, EPI_ISL_965432, EPI_ISL_965437 to 965450			
see above	CHR de la Citadelle	GIGA Medical Genomics	Bouchra Boujemla; Cécile Meex; Keith Durkin; Maria Artesi; Marie-Pierre Hayette; Pierrette Melin; Raphaël Boreux; Sébastien Bontems; Vincent Bours
EPI_ISL_418222	CHRU Bretonneau - Serv. Bacterio-Virol.	National Reference Center for Viruses of Respiratory Infections, Institut Pasteur, Paris	Angela Brisebarre; Etienne Simon-Lorière; Fabiana Gambaro; Flora Donati; Julien Marlet; Marion Barbet; Maud Vanpeene; Mélanie Albert; Méline Bizard; Sylvie Behillil; Sylvie van der Werf; Vincent Enouf

EPI_ISL_416502 to 416513, EPI_ISL_443289 to 443294, EPI_ISL_734168 to 734169	CHRU Pontchaillou - Laboratoire de Virologie	National Reference Center for Viruses of Respiratory Infections, Institut Pasteur, Paris	Angela Brisebarre; Camille Capel; Etienne Simon-Lorière; Flora Donati; Gisèle Lagathu; Marion Barbet; Maud Vanpeene; Mélanie Albert; Méline Bizard; Mélnie Albert; Sylvie Behillil; Sylvie van der Werf; Vincent Enouf
EPI_ISL_613545 to 613559, EPI_ISL_614281	CHRU Pontchaillou - Laboratoire de Virologie 2, rue Henri Le Guilloux	National Reference Center for Viruses of Respiratory Infections, Institut Pasteur, Paris	Angela Brisebarre; Camille Capel; Etienne Simon-Lorière; Gisèle Lagathu; Marion Barbet; Maud Vanpeene; Méline Bizard; Sylvie Behillil; Sylvie van der Werf; Vincent Enouf
EPI_ISL_418027, EPI_ISL_421453, EPI_ISL_421464 to 421465	CHTMAD	Instituto Nacional de Saude (INSA)	Guiomar et al
EPI_ISL_418219, EPI_ISL_443265 to 443283, EPI_ISL_754853 to 754856	CHU - Hôpital Cavale Blanche - Labo. de Virologie	National Reference Center for Viruses of Respiratory Infections, Institut Pasteur, Paris	Angela Brisebarre; Camille Capel; Etienne Simon-Lorière; Fabiana Gambaro; Flora Donati; Léa Pilorge; Marion Barbet; Maud Vanpeene; Mélanie Albert; Méline Bizard; Pilorge léa; Sylvie Behillil; Sylvie van der Werf; Vincent Enouf
EPI_ISL_645169 to 645172, EPI_ISL_649954 to 649971, EPI_ISL_660372, EPI_ISL_768823	CHU Bordeaux	CNR Virus des Infections Respiratoires - France SUD	Antonin Bal; Bruno Lina; Camille Ciccone; Gregory Destras; Gwendolyne Burfin; Hadrien Règue; Isabelle Garrigue; Laurence Josset; Marie-Edith Lafon; Martine Valette; Pantxika Bellecave; Pascale Trimoulet; Quentin Semanas
EPI_ISL_641546 to 641550, EPI_ISL_645188 to 645196, EPI_ISL_660353 to 660371, EPI_ISL_745312 to 745315, EPI_ISL_745335 to 745336	CHU Clermont-Ferrand	CNR Virus des Infections Respiratoires - France SUD	Amélie Brebion; Antonin Bal; Audrey Mirand; Bruno Lina; Christel Regagnon; Christine Archimbaud; Cécile Henquell; Gregory Destras; Gwendolyne Burfin; Hadrien Règue; Hélène Chabrolles; Laurence Josset; Martine Chambon; Martine Valette; Maxime Bisseux; Patricia Combes; Quentin Semanas
EPI_ISL_418002	CHU Coimbra	Instituto Nacional de Saude (INSA)	Guiomar et al
EPI_ISL_418005	CHU Coimbra - Pediátrico	Instituto Nacional de Saude (INSA)	Guiomar et al
EPI_ISL_416751 to 416752	CHU Gabriel Montpied	CNR Virus des Infections Respiratoires - France SUD	Alexandre; Antonin; Bal; Bouscambert-Duchamp; Brengel-Pesce; Bruno.; Cheynet; Destras; Florence; Gaymard; Gregory; Josset; Karen; Laurence; Lina; Martine; Maude; Morfin-Sherpa; Valette; Valérie
EPI_ISL_641556, EPI_ISL_644681 to 644696, EPI_ISL_660672 to 660697, EPI_ISL_660700 to 660709	CHU Montpellier	CNR Virus des Infections Respiratoires - France SUD	Antonin Bal; Bruno Lina; Gregory Destras; Gwendolyne Burfin; Hadrien Règue; Laurence Josset; Martine Valette; Michel Segondy; Quentin Semanas; Vincent Foulongne
EPI_ISL_645217 to 645218, EPI_ISL_649173 to 649187, EPI_ISL_663249 to 663275, EPI_ISL_663278 to 663284, EPI_ISL_666721 to 666724, EPI_ISL_700371	CHU Nantes	CNR Virus des Infections Respiratoires - France SUD	Antonin Bal; Bruno Lina; Celine Bressollette; Gregory Destras; Gwendolyne Burfin; Hadrien Règue; Laurence Josset; Louise Castain; Martine Valette; Quentin Semanas; Virginie Ferré
EPI_ISL_644697 to 644702, EPI_ISL_645197 to 645209, EPI_ISL_645215, EPI_ISL_660710 to 660731, EPI_ISL_663243 to 663248	CHU Nîmes	CNR Virus des Infections Respiratoires - France SUD	Antonin Bal; Bruno Lina; Gregory Destras; Gwendolyne Burfin; Hadrien Règue; Jean-Philippe Lavigne; Laurence Josset; Marie-Josée Carles; Martine Valette; Maxence Lotellier; Quentin Semanas; Stephan Robin
EPI_ISL_645216, EPI_ISL_663227 to 663237, EPI_ISL_663239 to 663242, EPI_ISL_707781 to 707782, EPI_ISL_768822	CHU Poitiers	CNR Virus des Infections Respiratoires - France SUD	Agnès Beby-Defaux; Antonin Bal; Bruno Lina; Clément Jousselin; Gregory Destras; Gwendolyne Burfin; Hadrien Règue; Laurence Josset; Magali Garcia; Martine Valette; Nicolas Lévêque; Quentin Semanas
EPI_ISL_591099, EPI_ISL_591541 to 591547, EPI_ISL_593855 to 593901, EPI_ISL_603216 to 603220, EPI_ISL_671941 to 671972, EPI_ISL_671975 to 671977, EPI_ISL_751448, EPI_ISL_751450 to 751458, EPI_ISL_751460 to 751499, EPI_ISL_754136 to 754139, EPI_ISL_864581, EPI_ISL_864583 to 864593, EPI_ISL_875666 to 875667, EPI_ISL_875669 to 875673, EPI_ISL_913102, EPI_ISL_913114			
see above	CHU Purpan - Laboratoire de Virologie - Institut Fédératif de Biologie	CHU Purpan - Laboratoire de Virologie - Institut Fédératif de Biologie	Boyer P.; Carcenac R.; Dubois M.; Harter A.; Izopet J.; Latour J.; Ranger N.; Tremeaux P.
EPI_ISL_424993, EPI_ISL_434616 to 434635, EPI_ISL_482879 to 482889	CHU Purpan - Laboratoire de Virologie - Institut Fédératif de Biologie	Laboratoire de virologie - École Nationale Vétérinaire de Toulouse	Croville, G.; Guerin; Guillaume Croville; J.-L. and Izopet, J.; Jacques Izopet; Jean-Luc Guérin
EPI_ISL_982142 to 982144, EPI_ISL_982146	CHU SUD AMIENS	Department of Virology, Henri Mondor University Hospital, Assistance Publique Hôpitaux de Paris, Université Paris-Est Créteil, INSERM U955	Alexandre Soulier; Christophe Rodriguez; Elisabeth Trawinski; Guillaume Gricourt; Jean-Michel Pawlotsky; Melissa N'Debi; Slim Fourati; Vanessa Demontant
EPI_ISL_535732, EPI_ISL_535801	CHU Sainte-Justine	Laboratoire de santé publique du Québec	Guillaume Bourque; Ioannis Ragoussis; Jesse Shapiro; Mark Lathrop and Michel Roger; Mark Lathrop and Michel Roger on behalf of the CoVSeQ research group; Sandrine Moreira
EPI_ISL_641551 to 641555, EPI_ISL_660373 to 660377, EPI_ISL_660665 to 660668, EPI_ISL_660670 to 660671	CHU Toulouse	CNR Virus des Infections Respiratoires - France SUD	Antonin Bal; Bruno Lina; Gregory Destras; Gwendolyne Burfin; Hadrien Règue; Jean Michel Mansuy; Laurence Josset; Martine Valette; Quentin Semanas
EPI_ISL_443261 to 443264	CHU de Dijon - Laboratoire de Virologie	National Reference Center for Viruses of Respiratory Infections, Institut Pasteur, Paris	Angela Brisebarre; Etienne Simon-Lorière; Flora Donati; Jean-Baptiste Bour; Marion Barbet; Maud Vanpeene; Mélanie Albert; Méline Bizard; Sylvie Behillil; Sylvie van der Werf; Vincent Enouf
EPI_ISL_644673 to 644680, EPI_ISL_645174 to 645183, EPI_ISL_663206 to 663226, EPI_ISL_666711 to 666720	CHU de Limoges	CNR Virus des Infections Respiratoires - France SUD	Antonin Bal; Bruno Lina; Gregory Destras; Gwendolyne Burfin; Hadrien Règue; Laurence Josset; Martine Valette; Quentin Semanas; Sylvie Rogez
EPI_ISL_909702	CHU de Nantes - Hôtel Dieu. Laboratoire de Virologie	National Reference Center for Viruses of Respiratory Infections, Institut Pasteur, Paris	Angela Brisebarre; Bressollette CéLine; Camille Capel; Etienne Simon-Lorière; Marion Barbet; Maud Vanpeene; Méline Bizard; Sylvie Behillil; Sylvie van der Werf; Vincent Enouf
EPI_ISL_645173	CHU de Nice	CNR Virus des Infections Respiratoires - France SUD	Antonin Bal; Bruno Lina; Gregory Destras; Gwendolyne Burfin; Géraldine Gonfrier; Hadrien Règue; Laurence Josset; Martine Valette; Quentin Semanas; Valérie Giordanengo
EPI_ISL_641528	CHU de Nice - Hôpital Archet 10	CNR Virus des Infections Respiratoires - France SUD	Antonin Bal; Bruno Lina; Gregory Destras; Gwendolyne Burfin; Géraldine Gonfrier; Hadrien Règue; Laurence Josset; Martine Valette; Quentin Semanas; Valérie Giordanengo
EPI_ISL_641529	CHU de Nice - Hôpital Archet 11	CNR Virus des Infections Respiratoires - France SUD	Antonin Bal; Bruno Lina; Gregory Destras; Gwendolyne Burfin; Géraldine Gonfrier; Hadrien Règue; Laurence Josset; Martine Valette; Quentin Semanas; Valérie Giordanengo
EPI_ISL_641530	CHU de Nice - Hôpital Archet 12	CNR Virus des Infections Respiratoires - France SUD	Antonin Bal; Bruno Lina; Gregory Destras; Gwendolyne Burfin; Géraldine Gonfrier; Hadrien Règue; Laurence Josset; Martine Valette; Quentin Semanas; Valérie Giordanengo
EPI_ISL_641531	CHU de Nice - Hôpital Archet 13	CNR Virus des Infections Respiratoires - France SUD	Antonin Bal; Bruno Lina; Gregory Destras; Gwendolyne Burfin; Géraldine Gonfrier; Hadrien Règue; Laurence Josset; Martine Valette; Quentin Semanas; Valérie Giordanengo
EPI_ISL_641532	CHU de Nice - Hôpital Archet 14	CNR Virus des Infections Respiratoires - France SUD	Antonin Bal; Bruno Lina; Gregory Destras; Gwendolyne Burfin; Géraldine Gonfrier; Hadrien Règue; Laurence Josset; Martine Valette; Quentin Semanas; Valérie Giordanengo
EPI_ISL_641533	CHU de Nice - Hôpital Archet 15	CNR Virus des Infections Respiratoires - France SUD	Antonin Bal; Bruno Lina; Gregory Destras; Gwendolyne Burfin; Géraldine Gonfrier; Hadrien Règue; Laurence Josset; Martine Valette; Quentin Semanas; Valérie Giordanengo
EPI_ISL_641534	CHU de Nice - Hôpital Archet 16	CNR Virus des Infections Respiratoires - France SUD	Antonin Bal; Bruno Lina; Gregory Destras; Gwendolyne Burfin; Géraldine Gonfrier; Hadrien Règue; Laurence Josset; Martine Valette; Quentin Semanas; Valérie Giordanengo
EPI_ISL_641520, EPI_ISL_693387 to 693390	CHU de Nice - Hôpital Archet 2	CNR Virus des Infections Respiratoires - France SUD	Antonin Bal; Bruno Lina; Gregory Destras; Gwendolyne Burfin; Géraldine Gonfrier; Hadrien Règue; Laurence Josset; Martine Valette; Quentin Semanas; Valérie Giordanengo
EPI_ISL_641522	CHU de Nice - Hôpital Archet 3	CNR Virus des Infections Respiratoires - France SUD	Antonin Bal; Bruno Lina; Gregory Destras; Gwendolyne Burfin; Géraldine Gonfrier; Hadrien Règue; Laurence Josset; Martine Valette; Quentin Semanas; Valérie Giordanengo

EPI_ISL_641523	CHU de Nice - Hôpital Archet 4	CNR Virus des Infections Respiratoires - France SUD	Antonin Bal; Bruno Lina; Gregory Destras; Gwendolyne Burfin; Géraldine Gonfrier; Hadrien Règue; Laurence Josset; Martine Valette; Quentin Semanas; Valérie Giordanengo
EPI_ISL_641524	CHU de Nice - Hôpital Archet 5	CNR Virus des Infections Respiratoires - France SUD	Antonin Bal; Bruno Lina; Gregory Destras; Gwendolyne Burfin; Géraldine Gonfrier; Hadrien Règue; Laurence Josset; Martine Valette; Quentin Semanas; Valérie Giordanengo
EPI_ISL_641525	CHU de Nice - Hôpital Archet 6	CNR Virus des Infections Respiratoires - France SUD	Antonin Bal; Bruno Lina; Gregory Destras; Gwendolyne Burfin; Géraldine Gonfrier; Hadrien Règue; Laurence Josset; Martine Valette; Quentin Semanas; Valérie Giordanengo
EPI_ISL_641526	CHU de Nice - Hôpital Archet 7	CNR Virus des Infections Respiratoires - France SUD	Antonin Bal; Bruno Lina; Gregory Destras; Gwendolyne Burfin; Géraldine Gonfrier; Hadrien Règue; Laurence Josset; Martine Valette; Quentin Semanas; Valérie Giordanengo
EPI_ISL_641527	CHU de Nice - Hôpital Archet 9	CNR Virus des Infections Respiratoires - France SUD	Antonin Bal; Bruno Lina; Gregory Destras; Gwendolyne Burfin; Géraldine Gonfrier; Hadrien Règue; Laurence Josset; Martine Valette; Quentin Semanas; Valérie Giordanengo
EPI_ISL_641535 to 641545, EPI_ISL_645184 to 645187, EPI_ISL_649942 to 649953, EPI_ISL_660326 to 660352, EPI_ISL_660432	CHU de Saint-Étienne Hôpital Nord	CNR Virus des Infections Respiratoires - France SUD	Antonin Bal; Bruno Lina; Bruno Pozzetto; Gregory Destras; Gwendolyne Burfin; Hadrien Règue; Issam Bechri; Laurence Josset; Manon Vogrig; Marine Delorme; Martine Valette; Quentin Semanas; Sylvie Gonzalo; Sylvie Pillet; Thomas Bourlet
EPI_ISL_418024	CHUA - Faro	Instituto Nacional de Saude (INSA)	Guiomar et al
EPI_ISL_535736 to 535737, EPI_ISL_535741, EPI_ISL_535772 to 535775, EPI_ISL_535781 to 535782, EPI_ISL_535784 to 535785, EPI_ISL_535822 to 535825, EPI_ISL_535828			
see above	CHUL-LABO MULTI / MICRO	Laboratoire de santé publique du Québec	Guillaume Bourque; Ioannis Ragoussis; Jesse Shapiro; Mark Lathrop and Michel Roger; Mark Lathrop and Michel Roger on behalf of the CoVSeQ research group; Sandrine Moreira
EPI_ISL_417988, EPI_ISL_417990 to 417991, EPI_ISL_417994 to 417996, EPI_ISL_418010 to 418016	CHULC - H Curry Cabral	Instituto Nacional de Saude (INSA)	Guiomar et al
EPI_ISL_417992 to 417993	CHULC - H D Estefania	Instituto Nacional de Saude (INSA)	Guiomar et al
EPI_ISL_535726, EPI_ISL_535729 to 535730, EPI_ISL_535735, EPI_ISL_535740, EPI_ISL_535753 to 535754, EPI_ISL_535783, EPI_ISL_535789, EPI_ISL_535802, EPI_ISL_535806, EPI_ISL_535826, EPI_ISL_535830, EPI_ISL_535832			
see above	CHUM - Microbiologie - Hôpital Saint-Luc	Laboratoire de santé publique du Québec	Guillaume Bourque; Ioannis Ragoussis; Jesse Shapiro; Mark Lathrop and Michel Roger; Mark Lathrop and Michel Roger on behalf of the CoVSeQ research group; Sandrine Moreira
EPI_ISL_1007586 to 1007587, EPI_ISL_1007591 to 1007594, EPI_ISL_1007599 to 1007600, EPI_ISL_1007603, EPI_ISL_1007611, EPI_ISL_1007620 to 1007622, EPI_ISL_1007639			
see above	CHUV	Laboratory of genomics and metagenomics, Institute of Microbiology, University Hospital Centre and University of Lausanne, Switzerland	Claire Bertelli; Damien Jacot; Gilbert Greub; Sébastien Aeby; Trestan Pillonel
EPI_ISL_683835	CICM	Malaria Research and Training Center (MRTC-Parasito)	Abdoulaye Djimde; Antoine Dara
EPI_ISL_487446 to 487466	CICM-Mali	Bundeswehr Institut of Microbiology	Antwerpen; Bestehorn-Willmann; Dürr; Heitzer; Kouriba; Maiga; Quedraogo; Rehn; Sangaré; Sogodogo; Traoré; Walter; Wölfel; Zimmermann
EPI_ISL_445245, EPI_ISL_445248, EPI_ISL_445250, EPI_ISL_445254 to 445255, EPI_ISL_445258, EPI_ISL_445260	CLINICA ALEMANA DE SANTIAGO S.A.	Instituto de Salud Publica de Chile	Alejandra Acevedo; Andrés E Castillo; Bárbara Parra; Carolina Tambley; Gabriel Leal; Jaime Lagos; Jorge Fernandez; Loredana Arata; Patricia Bustos; Paz Tapia; Rodrigo Fasce; Winston Andrade
EPI_ISL_445272, EPI_ISL_445285, EPI_ISL_445287	CLINICA CIUDAD DEL MAR	Instituto de Salud Publica de Chile	Alejandra Acevedo; Andrés E Castillo; Bárbara Parra; Carolina Tambley; Gabriel Leal; Jaime Lagos; Jorge Fernandez; Loredana Arata; Patricia Bustos; Paz Tapia; Rodrigo Fasce; Winston Andrade
EPI_ISL_445297	CLINICA INTEGRAL S.A.	Instituto de Salud Publica de Chile	Alejandra Acevedo; Andrés E Castillo; Bárbara Parra; Carolina Tambley; Gabriel Leal; Jaime Lagos; Jorge Fernandez; Loredana Arata; Patricia Bustos; Paz Tapia; Rodrigo Fasce; Winston Andrade
EPI_ISL_445259	CLINICA LAS CONDES S.A.	Instituto de Salud Publica de Chile	Alejandra Acevedo; Andrés E Castillo; Bárbara Parra; Carolina Tambley; Gabriel Leal; Jaime Lagos; Jorge Fernandez; Loredana Arata; Patricia Bustos; Paz Tapia; Rodrigo Fasce; Winston Andrade
EPI_ISL_445282 to 445283, EPI_ISL_445290, EPI_ISL_445292	CLINICA MAGALLANES S.A.	Instituto de Salud Publica de Chile	Alejandra Acevedo; Andrés E Castillo; Bárbara Parra; Carolina Tambley; Gabriel Leal; Jaime Lagos; Jorge Fernandez; Loredana Arata; Patricia Bustos; Paz Tapia; Rodrigo Fasce; Winston Andrade
EPI_ISL_445264	CLINICA REDSALUD VITACURA.	Instituto de Salud Publica de Chile	Alejandra Acevedo; Andrés E Castillo; Bárbara Parra; Carolina Tambley; Gabriel Leal; Jaime Lagos; Jorge Fernandez; Loredana Arata; Patricia Bustos; Paz Tapia; Rodrigo Fasce; Winston Andrade
EPI_ISL_445249	CLINICA SANTA MARIA S.A.	Instituto de Salud Publica de Chile	Alejandra Acevedo; Andrés E Castillo; Bárbara Parra; Carolina Tambley; Gabriel Leal; Jaime Lagos; Jorge Fernandez; Loredana Arata; Patricia Bustos; Paz Tapia; Rodrigo Fasce; Winston Andrade
EPI_ISL_445262	CLINICA TABANCURA	Instituto de Salud Publica de Chile	Alejandra Acevedo; Andrés E Castillo; Bárbara Parra; Carolina Tambley; Gabriel Leal; Jaime Lagos; Jorge Fernandez; Loredana Arata; Patricia Bustos; Paz Tapia; Rodrigo Fasce; Winston Andrade
EPI_ISL_445306, EPI_ISL_445312, EPI_ISL_445315, EPI_ISL_445361	CLINICA UC SAN CARLOS DE APOQUINDO	Instituto de Salud Publica de Chile	Alejandra Acevedo; Andrés E Castillo; Bárbara Parra; Carolina Tambley; Gabriel Leal; Jaime Lagos; Jorge Fernandez; Loredana Arata; Patricia Bustos; Paz Tapia; Rodrigo Fasce; Winston Andrade
EPI_ISL_445334	CLINICA UNIVERSITARIA DE PUERTO MONTT S.A.	Instituto de Salud Publica de Chile	Alejandra Acevedo; Andrés E Castillo; Bárbara Parra; Carolina Tambley; Gabriel Leal; Jaime Lagos; Jorge Fernandez; Loredana Arata; Patricia Bustos; Paz Tapia; Rodrigo Fasce; Winston Andrade
EPI_ISL_445330	CLINICA VESPUCIO S. A.	Instituto de Salud Publica de Chile	Alejandra Acevedo; Andrés E Castillo; Bárbara Parra; Carolina Tambley; Gabriel Leal; Jaime Lagos; Jorge Fernandez; Loredana Arata; Patricia Bustos; Paz Tapia; Rodrigo Fasce; Winston Andrade
EPI_ISL_420043, EPI_ISL_420061	CMIP	National Reference Center for Viruses of Respiratory Infections, Institut Pasteur, Paris	Angela Brisebarre; Etienne Simon-Lorière; Flora Donati; Marion Barbet; Maud Vanpeene; Mélanie Albert; Méline Bizard; Sylvie Behillil; Sylvie van der Werf; Vincent Enouf
EPI_ISL_547584, EPI_ISL_560320 to 560321	CMS, Roorkee	CSIR-Institute of Microbial Technology	Amandeep Kaur; Anu Singh; Ashwani Kumar; Debarghya Ghose; Dipak Dutta; Harsh Goar; Kanika Bansal; Navin Baid; Poushali Chakraborty; Prabhu B. Patil; Rajesh Kumar Mishra; Sanjeet Kumar; Sanjeev Khosla
EPI_ISL_410486, EPI_ISL_416745 to 416746, EPI_ISL_508912 to 508918, EPI_ISL_508921 to 508930, EPI_ISL_508933, EPI_ISL_508937, EPI_ISL_508939 to 508940, EPI_ISL_508942, EPI_ISL_508945, EPI_ISL_508948, EPI_ISL_508951, EPI_ISL_508953 to 508957, EPI_ISL_508961 to 508962, EPI_ISL_508964 to 508965, EPI_ISL_508967, EPI_ISL_508969 to 508970, EPI_ISL_508972 to 508974, EPI_ISL_508976 to 508977, EPI_ISL_508979 to 508987, EPI_ISL_508990 to 508991, EPI_ISL_508993 to 508997, EPI_ISL_508999 to 509002, EPI_ISL_525536 to 525537, EPI_ISL_525540 to 525543, EPI_ISL_578176 to 578177, EPI_ISL_582110 to 582112, EPI_ISL_582114 to 582116, EPI_ISL_582118 to 582120, EPI_ISL_582122, EPI_ISL_582508, EPI_ISL_623099 to 623102, EPI_ISL_636476 to 636477, EPI_ISL_636479, EPI_ISL_636484 to 636485, EPI_ISL_636487, EPI_ISL_639974 to 639977, EPI_ISL_639979 to 639981, EPI_ISL_639983, EPI_ISL_639985 to 640014, EPI_ISL_676542 to 676548, EPI_ISL_676550 to 676573, EPI_ISL_678494 to 678508, EPI_ISL_678534 to 678547, EPI_ISL_681261 to 681263, EPI_ISL_683335 to 683345, EPI_ISL_683347 to 683350, EPI_ISL_683352 to 683353, EPI_ISL_683355 to 683359, EPI_ISL_683362 to 683368, EPI_ISL_683370 to 683371, EPI_ISL_683373 to 683390, EPI_ISL_683392 to 683402, EPI_ISL_684107, EPI_ISL_692733 to 692768, EPI_ISL_693490 to 693514, EPI_ISL_700327, EPI_ISL_728304 to 728320, EPI_ISL_728516 to 728527, EPI_ISL_728529 to 728532, EPI_ISL_728534, EPI_ISL_728536 to 728551, EPI_ISL_730640 to 730651, EPI_ISL_732677 to 732703, EPI_ISL_745311, EPI_ISL_745316 to 745317, EPI_ISL_745319, EPI_ISL_745326 to 745334, EPI_ISL_745337 to 745397			
see above	CNR Virus des Infections Respiratoires - France SUD	CNR Virus des Infections Respiratoires - France SUD	Alexandre; Alexandre Gaymard; Antonin; Antonin Bal; Bal; Bouscambert-Duchamp; Brengel-Pesce; Bruno Lina; Bruno.; Carine Moustaud; Cheynet; Claudia Gonzalez; Destras; Emilie Frobert; Florence; Florence Morfin-Sherpa; Gaymard; Gregory; Gregory Destras; Gregory Queromes; Gwendolyne Burfin; Hadrien Règue; Josset; Karen; Laurence; Laurence Josset; Lina; Martine; Martine Valette; Maude; Maude Bouscambert-Duchamp; Morfin-Sherpa; Quentin Semanas; Raphaelle Lamy; Solenne Brun; Valette; Valérie
EPI_ISL_452127 to 452130	CO Department of Public Health and Environment	Pathogen Discovery, Respiratory Viruses Branch, Division of Viral Diseases, Centers for Disease Control and Prevention	Alison S. Laufer Halpin; Anna Montmayeur; Anna Uehara; Christopher A. Elkins; Clinton R. Paden; Haibin Wang; Jing Zhang; Krista Queen; Mary S. Keckler; Rachel Marine; Suxiang Tong; Yan Li; Ying Tao; Zachary Weiner
EPI_ISL_751575, EPI_ISL_751630, EPI_ISL_751701, EPI_ISL_751704 to 751705, EPI_ISL_751728, EPI_ISL_751763, EPI_ISL_751766			
see above	CO Dept. of Public Health and Environment, Lab Services	Genomics and Discovery, Respiratory Viruses Branch,	Anna Montmayeur; Anna Uehara; Clinton R. Paden; Haibin Wang; Jing Zhang; Justin Lee; Krista Queen; Mili Sheth; Peter W. Cook; Rachel Marine;

	Division	Division of Viral Diseases, Centers for Disease Control and Prevention	Suxiang Tong; Yan Li; Ying Tao
EPI_ISL_416994, EPI_ISL_418248 to 418249	COMPLEJO ASISTENCIAL UNIVERSITARIO DE BURGOS	Instituto de Salud Carlos III	A. Monzón; F. Casas; G. Hospital: ------; I. Jiménez; I. Megias-Lobon G.; Iglesias-Caballero; M. Camarero; M. Camarero S. Pozo F. Casas I. Jiménez P. Jiménez M. Zaballos A. Monzón; M. Cuesta; M. González-Esguevillas; M. Molinero Calamita; M. Zaballos; P. Jiménez; S. Juliá; S. Juliá M. Cuesta I. Megias Lobón; S. Pozo; S. Varona
EPI_ISL_434538	COOPESAIN	Inciensa, Instituto Costarricense de Investigación y Enseñanza en Nutrición y Salud	Adriana Godinez & Melany Calderon; Claudio Soto-Garita; Estela Cordero; Francisco Duarte; Hebleen Porras
EPI_ISL_735408	COVID 19 Centro de Combate ao Coronavirus CCC Jandira	Instituto Adolfo Lutz, Interdiciplinary Procedures Center, Strategic Laboratory	Claudia Regina Gonçalves; Claudio Tavares Sacchi; Erica Valessa Ramos Gomes; Karoline Rodrigues Campos
EPI_ISL_450841	COVID-19 Laboratory	DNA Solution Ltd	ABM Khademul Islam; AHM Nurun Nabi; Abu Sufian; Gazi Nurun Nahar; Habibul Bari Shozib; Haseena Khan; Imran Khan; Latiful Bari; M Anwar Hossain.; MA Malek; Mamun Ahmed; Md Imdadul Hoque; Md Ismail Hosen; Md Mizanur Rahman; Mohammad Riazul Islam; Nazmul Ahsan; Richard Malo; Sabita Rezwana Rahman; Sabrina Moriom Elius; Shahryar Nabi; Sharif Akhteruzzaman; Zeba Islam Seraj
EPI_ISL_450843	COVID-19 Laboratory	DNA Solution Ltd.	ABM Khademul Islam; AHM Nurun Nabi; Abu Sufian; Gazi Nurun Nahar; Habibul Bari Shozib; Haseena Khan; Imran Khan; Latiful Bari; M Anwar Hossain.; MA Malek; Mamun Ahmed; Md Imdadul Hoque; Md Ismail Hosen; Md Mizanur Rahman; Mohammad Riazul Islam; Nazmul Ahsan; Richard Malo; Sabita Rezwana Rahman; Sabrina Moriom Elius; Shahryar Nabi; Sharif Akhteruzzaman; Zeba Islam Seraj
EPI_ISL_450840	COVID-19 Laboratory	DNA Solution Ltd. L-5	ABM Khademul Islam; AHM Nurun Nabi; Abu Sufian; Gazi Nurun Nahar; Habibul Bari Shozib; Haseena Khan; Imran Khan; Latiful Bari; M Anwar Hossain.; MA Malek; Mamun Ahmed; Md Imdadul Hoque; Md Ismail Hosen; Md Mizanur Rahman; Mohammad Riazul Islam; Nazmul Ahsan; Richard Malo; Sabita Rezwana Rahman; Sabrina Moriom Elius; Shahryar Nabi; Sharif Akhteruzzaman; Zeba Islam Seraj
EPI_ISL_450839	COVID-19 Laboratory Centre for Advanced Research in Sciences (CARS), University of Dhaka, Dhaka-1000, Bangladesh	DNA Solution Ltd	ABM Khademul Islam; AHM Nurun Nabi; Abu Sufian; Gazi Nurun Nahar; Habibul Bari Shozib; Haseena Khan; Imran Khan; Latiful Bari; M Anwar Hossain.; MA Malek; Mamun Ahmed; Md Imdadul Hoque; Md Ismail Hosen; Md Mizanur Rahman; Mohammad Riazul Islam; Nazmul Ahsan; Richard Malo; Sabita Rezwana Rahman; Sabrina Moriom Elius; Shahryar Nabi; Sharif Akhteruzzaman; Zeba Islam Seraj
EPI_ISL_412981	CR&WISCO GENERAL HOSPITAL	Hubei Provincial Center for Disease Control and Prevention	Bin Fang; Bo Yang; Bo Yu; Faxian Zhan; Guojun Ye; Jing Li; Junqiang Xu; Kun Cai; Linlin Liu; Xiang Li; Xiao Yu; Xixiang Huo; Yongzhong Jiang.
EPI_ISL_574593, EPI_ISL_574596, EPI_ISL_583494	CS II Dr. Antonio Vicoso Moreira de Rezende Sumare	Instituto Adolfo Lutz, Interdiciplinary Procedures Center, Strategic Laboratory	Claudia Regina Gonçalves; Claudio Tavares Sacchi; Erica Valessa Ramos Gomes; Karoline Rodrigues Campos
EPI_ISL_636980	CS Xai Xai	KRISP, KZN Research Innovation and Sequencing Platform	Giandhari J; Ismael N; Nadia Sitoe; Nedio Mabunda; Paulo Arnaldo; Pillay S; Tegally H; Wilkinson E; de Oliveira T
EPI_ISL_486853	CSIR-CDRI/SGPGI	CSIR-CDRI/SGPGI	Dharam Veer Singh; Rahul Vishvkarma; Rajender Singh; Ravishankar Ramachandran; Saumya Sarkar; Tapas Kumar Kundu; Uday Ghoshal; Ujjala Ghoshal
EPI_ISL_489995, EPI_ISL_490106, EPI_ISL_491096, EPI_ISL_491114, EPI_ISL_491479, EPI_ISL_497762 to 497763, EPI_ISL_497765, EPI_ISL_497767			
see above	CSIR-CDRI/SGPGI, Lucknow	CSIR-CDRI/SGPGI, Lucknow	Dharam Veer Singh; Rahul Vishvkarma; Rajender Singh; Ravishankar Ramachandran; Saumya Sarkar; Tapas Kumar Kundu; Uday Ghoshal; Ujjala Ghoshal
EPI_ISL_497766	CSIR-CDRI/SGPGI, Lucknow	CSIR-CDRi/SGPGI, Lucknow	Dharam Veer Singh; Rahul Vishvkarma; Rajender Singh; Ravishankar Ramachandran; Saumya Sarkar; Tapas Kumar Kundu; Uday Ghoshal; Ujjala Ghoshal
EPI_ISL_497758, EPI_ISL_497760	CSIR-CDRI/SGPGI, Lucknow	CSIr-CDRI/SGPGI, Lucknow	Dharam Veer Singh; Rahul Vishvkarma; Rajender Singh; Ravishankar Ramachandran; Saumya Sarkar; Tapas Kumar Kundu; Uday Ghoshal; Ujjala Ghoshal
EPI_ISL_497764	CSIR-CDRi/SGPGI, Lucknow	CSIR-CDRI/SGPGI, Lucknow	Dharam Veer Singh; Rahul Vishvkarma; Rajender Singh; Ravishankar Ramachandran; Saumya Sarkar; Tapas Kumar Kundu; Uday Ghoshal; Ujjala Ghoshal
EPI_ISL_447556 to 447583, EPI_ISL_447847 to 447866, EPI_ISL_450326 to 450332, EPI_ISL_458045 to 458065, EPI_ISL_458070 to 458077, EPI_ISL_458080, EPI_ISL_458298, EPI_ISL_471585 to 471646, EPI_ISL_495161 to 495273, EPI_ISL_528823 to 528869, EPI_ISL_539616 to 539775			
see above	CSIR-Centre for Cellular and Molecular Biology	CSIR-Centre for Cellular and Molecular Biology	Ajay Sarawagi; Amrutha H C; Ananga Ghosh; Annapoorna P Karthyayani; Archana Bharadwaj Siva; Debabrata Jana; Debrya Saha; Deepak Kumar; Devi Prasad Vijayashankar; Devi Prasad Vijayashankara; Dhiviya Vedagiri; Disha Nanda; Divya Das; Divya Gupta; Divya Tej Sowpati; G. Aditya Kumar; Gangumala Srinivas Reddy; Gokulan C G; Gunjan Purohit; Hanuman Tulashiram Kale; Jotin Gogoi; Kakade Aishwarya Arun; Karthik Bharadwaj Tallapaka; Kezia J Ann; Koushick Sivakumar; Krishnan Harinivas Harshan; Lamuk Zaveri; M Soujanya Reddy; M Soujanya Reddy Rakesh K Mishra; Manish Bhattacharjee; Namami Gaur; Nikhil Hajirnis; Onkar Kulkarni; Pankaj Kumar; Payel Mukherjee; Peddapuvala Sai Uday Kiran; Peddapuvala Sai Uday Kiran Rakesh K Mishra; Pooja Ramesh Gupta; Prachand Issarapu; Pratheusa Maccha; Preethi Jampala; Preethi Jampala Rakesh K Mishra; Priya Singh; Priyanka Pant; Purushotham Vodnala; Radhika Khandelwal; Rajan Kumar Jha; Rajkanwar Nathawat; Rakesh K Mishra; Ravi Prasad Mukku; Renu Sudhakar; Roshan Maku Venkata; Sakshi Shambhavi; Santosh Kumar Kuncha; Shagufta Khan; Sharada Ravi Iyer; Shemin Mansuri; Shraddha Vijay Lahoti; Sofia Banu; Somesh Gorde; Sonu Uday; Sudipta Mondal; Sujoy Deb; Sulagana Mukherjee; Swati Bayyana; Swetha Sundar; Tulasi Nagabandi; Umesh Kumar; Unis Ahmad Bhat; Vishal Sah; Zeba Rizvi; Zuberwasim Sayyad
EPI_ISL_911532, EPI_ISL_911535 to 911536	CSIR-IGIB	CSIR-IGIB	Akshay Kanakan; Anil Kumar; Aparna S Murali; Azka Khan; Janani SV; Nisha Rawat; Partha Chattopadhyay; Priti Devi; Priyanka Mehta; Rajesh Pandey; Ranjeet Maurya; Shweta Sahni
EPI_ISL_569859 to 569863, EPI_ISL_578080 to 578081, EPI_ISL_578168, EPI_ISL_578175, EPI_ISL_578178 to 578184, EPI_ISL_581449 to 581451, EPI_ISL_581494 to 581497, EPI_ISL_581499 to 581504, EPI_ISL_582028, EPI_ISL_661304 to 661311			
see above	CSIR-Indian Institute of Chemical Biology, MEDICA Supercpecialty Hospital Kolkata	CSIR-Indian Institute of Chemical Biology, MEDICA Supercpecialty Hospital Kolkata	Abhishake Lahiri; Debaleena Bhowmik; Dr. Partha Chakrabarti; Dr. Arpita Ghosh Mitra; Dr. Aviral Roy; Dr. Aviral Roy; Dr. AviralRoy; Dr. Partha Chakrabarti; Dr. Rajesh Pandey; Dr. Saikat Chakrabarti; Dr. Sandip Paul; Dr. Soumen Saha; Dr. Soumen Saha; Dr.Partha Chakrabarti; Priyanka Mallick; Sujay Krishna Maity
EPI_ISL_450306, EPI_ISL_465685, EPI_ISL_465687, EPI_ISL_465692, EPI_ISL_535799 to 535800, EPI_ISL_535813, EPI_ISL_535815, EPI_ISL_535864, EPI_ISL_535876 to 535877, EPI_ISL_535884, EPI_ISL_535902, EPI_ISL_535910, EPI_ISL_535925, EPI_ISL_535928, EPI_ISL_535946, EPI_ISL_535967, EPI_ISL_535984, EPI_ISL_536028, EPI_ISL_536040, EPI_ISL_536042, EPI_ISL_536044, EPI_ISL_536110, EPI_ISL_536150, EPI_ISL_536158, EPI_ISL_536160, EPI_ISL_536166, EPI_ISL_536181, EPI_ISL_536213, EPI_ISL_536215 to 536216, EPI_ISL_536237, EPI_ISL_536243, EPI_ISL_536272, EPI_ISL_536286, EPI_ISL_536299, EPI_ISL_536301 to 536302, EPI_ISL_536304, EPI_ISL_536344, EPI_ISL_536361, EPI_ISL_536383			
see above	CSSS Haut-Richelieu/Rouville (Hôpital)	Laboratoire de santé publique du Québec	Guillaume Bourque; Ioannis Ragoussis; Jesse Shapiro; Mark Lathrop and Michel Roger; Mark Lathrop and Michel Roger on behalf of the CoVSeQ research group; Sandrine Moreira
EPI_ISL_536109	CSSS de Port-Cartier	Laboratoire de santé publique du Québec	Guillaume Bourque; Ioannis Ragoussis; Jesse Shapiro; Mark Lathrop and Michel Roger on behalf of the CoVSeQ research group; Sandrine Moreira
EPI_ISL_536206, EPI_ISL_536260	CSSS de la Minganie	Laboratoire de santé publique du Québec	Guillaume Bourque; Ioannis Ragoussis; Jesse Shapiro; Mark Lathrop and Michel Roger on behalf of the CoVSeQ research group; Sandrine Moreira
EPI_ISL_751554, EPI_ISL_751624, EPI_ISL_751649 to 751650, EPI_ISL_751706, EPI_ISL_751729, EPI_ISL_751759, EPI_ISL_751762			
see above	CT-Dr. Katherine A. Kelley State Public Health Lab	Genomics and Discovery, Respiratory Viruses Branch, Division of Viral Diseases, Centers for Disease Control and Prevention	Anna Montmayeur; Anna Uehara; Clinton R. Paden; Haibin Wang; Jing Zhang; Justin Lee; Krista Queen; Mili Sheth; Peter W. Cook; Rachel Marine; Suxiang Tong; Yan Li; Ying Tao
EPI_ISL_426416, EPI_ISL_454642, EPI_ISL_454645	CT-Dr. Katherine A. Kelley State Public Health Lab	Pathogen Discovery, Respiratory Viruses Branch, Division of Viral Diseases, Centers for Disease Control and Prevention	Alison S. Laufer Halpin; Anna Uehara; Bettina Bankamp; Christopher A. Elkins; Clinton R. Paden; Haibin Wang; Jing Zhang; Krista Queen; Mary S. Keckler; Rachel Marine; Suxiang Tong; Yan Li; Ying Tao; Zachary Weiner
EPI_ISL_468314, EPI_ISL_583503	CTA Centro de Testagem e Aconselhamento	Instituto Adolfo Lutz, Interdiciplinary Procedures Center, Strategic Laboratory	Claudia Regina Gonçalves; Claudio Tavares Sacchi; Erica Valessa Ramos Gomes; Karoline Rodrigues Campos
EPI_ISL_445303	CTRO.DE SALUD FAMILIAR DR. RAUL YAZIGI	Instituto de Salud Publica de Chile	Alejandra Acevedo; Andrés E Castillo; Bárbara Parra; Carolina Tambley; Gabriel Leal; Jaime Lagos; Jorge Fernandez; Loredana Arata; Patricia Bustos; Paz Tapia; Rodrigo Fasce; Winston Andrade
EPI_ISL_451935, EPI_ISL_452140, EPI_ISL_452142, EPI_ISL_452148 to 452152	CUB Hopital Erasme Laboratoire d'Anatomie Pathologique	CUB Hopital Erasme Laboratoire d'Anatomie Pathologique	Dr Nicky D'Haene; Dr. Nicky D'Haene; Dr.Nikcy D'Haene; Isabelle Salmon; Nicky D'Haene; Nikcy D'Haene; Prof. Isabelle Salmon

EPI_ISL_909949, EPI_ISL_918489	CUSL/UCLouvain COVID testing federal platform	UCLouvain/IREC/MBLG	Benoit Kabamba Mukadi; Jean Ruelle; Lysa Pinsmaye
EPI_ISL_535749, EPI_ISL_535761, EPI_ISL_535764 to 535765, EPI_ISL_535811, EPI_ISL_536337, EPI_ISL_536365 to 536366	CUSM-Site Glen-LAB Microbiologie	Laboratoire de santé publique du Québec	Guillaume Bourque; Ioannis Ragoussis; Jesse Shapiro; Mark Lathrop and Michel Roger; Mark Lathrop and Michel Roger on behalf of the CoVSeQ research group; Sandrine Moreira
EPI_ISL_515938, EPI_ISL_515941 to 515942	CV RAMAN HOSPITAL	Department of Neurovirology, National Institute of Mental Health and Neuroscience (NIMHANS)	Anita Desai; Chitra Pattabiraman; Harsha PK; Manjunatha Venkataswamy; Pramada Prasad; Ravi Vasanthapuram; Risha Rasheed; Shafeeq S Hameed; Vijayalakshmi Reddy
EPI_ISL_486383, EPI_ISL_486881	CV Raman Hospital	Department of Neurovirology, National Institute of Mental Health and Neuroscience (NIMHANS)	Anita Desai; Chitra Pattabiraman; Harsha PK; Manjunatha Venkataswamy; Ravi Vasanthapuram; Risha Rasheed; Shafeeq S Hameed; Vijayalakshmi Reddy
EPI_ISL_428367, EPI_ISL_443300 to 443302, EPI_ISL_443306, EPI_ISL_443311 to 443313, EPI_ISL_443317	Cabinet Médical	National Reference Center for Viruses of Respiratory Infections, Institut Pasteur, Paris	Angela Brisebarre; Etienne Simon-Lorière; Flora Donati; Marion Barbet; Maud Vanpeene; Mélanie Albert; Méline Bizard; Sylvie Behillil; Sylvie van der Werf; Vincent Enouf
EPI_ISL_418235	Cabinet médical	National Reference Center for Viruses of Respiratory Infections, Institut Pasteur, Paris	Angela Brisebarre; Etienne Simon-Lorière; Flora Donati; Marion Barbet; Maud Vanpeene; Mélanie Albert; Méline Bizard; Sylvie Behillil; Sylvie van der Werf; Vincent Enouf
EPI_ISL_418812 to 418813, EPI_ISL_429807, EPI_ISL_429817 to 429820, EPI_ISL_482474 to 482475, EPI_ISL_482478, EPI_ISL_482480 to 482484	Cadham Provincial Laboratory	National Microbiology Laboratory	; Anna Majer; David Alexander; Elsie Grudeski; Gary Van Domselaar; Grace Seo; Jared Bullard; Jennifer Tanner; Kerry Dust; Kristyn Burak; Matthew Gilmour; Morag Graham; Natalie Knox; Nathalie Bastien; Paul Van Caeseele; Philip Mabon; Rhiannon Huzarewich; Russell Mandes; Russell Mandez; Shari Tyson; Timothy Booth; Yan Li
EPI_ISL_582242 to 582249, EPI_ISL_582251 to 582286, EPI_ISL_582288 to 582306, EPI_ISL_582308, EPI_ISL_582311 to 582343, EPI_ISL_582345 to 582347, EPI_ISL_582349 to 582397, EPI_ISL_582399 to 582414, EPI_ISL_582416 to 582420, EPI_ISL_582422 to 582443, EPI_ISL_582445 to 582507			
see above	Cadham Provincial Laboratory	National Microbiology Laboratory (NML)	Anna Majer; Anneliese Landgraff; CanCOGeN's metadata curation team; Darian Hole; David Alexander; Elsie Grudeski; Gary Van Domselaar; Grace Seo; Jared Bullard; Jennifer Tanner; Kerry Dust; Madison Chapel; Morag Graham; Natalie Knox; Nathalie Bastien; Paul Van Caeseele; Philip Mabon; Public Health Agency of Canada CanCOGeN team; Rhiannon Huzarewich; Russell Mandes; Shari Tyson; Timothy Booth; Yan Li
EPI_ISL_632909 to 632933	Cadham Provincial laboratory	Cadham Provincial laboratory	Anna Majer; Anneliese Landgraff; CanCOGeN's metadata curation team; Darian Hole; David Alexander; Elsie Grudeski; Gary Van Domselaar; Grace Seo; Jared Bullard; Jennifer Tanner; Kerry Dust; Madison Chapel; Morag Graham; Natalie Knox; Nathalie Bastien; Paul Van Caeseele; Philip Mabon; Public Health Agency of Canada CanCOGeN team; Rhiannon Huzarewich; Russell Mandes; Shari Tyson; Timothy Booth; Yan Li
EPI_ISL_444794	Cairns Hospital	Public Health Virology Laboratory	Alyssa Pyke; Amanda De Jong; Andrew Van Den Hurk; Bixing Huang; Carmel Taylor; David Warrilow; Doris Genge; Elisabeth Gamez; Glen Hewitson; Ian Maxwell Mackay; Inga Sultana; Jamie McMahon; Jean Barcelon; Judy Northill; Mitchell Finger; Natalie Simpson; Neelima Nair; Peter Burtonclay; Peter Moore; Sarah Wheatley; Sean Moody; Sonja Hall-Mendelin; Timothy Gardam; and Frederick Moore
EPI_ISL_408009 to 408010	California Department of Health	Pathogen Discovery, Respiratory Viruses Branch, Division of Viral Diseases, Centers for Dieases Control and Prevention	Anna Uehara; Brett L. Whitaker; Brian Lynch; Clinton Paden; Janna' R. Murray; Jing Zhang; Krista Queen; Lijuan Wang; Senthil Kumar K. Sakthivel; Shifaq Kamili; Stephen Lindstrom; Susan I. Gerber; Suxiang Tong; Xiaoyan Lu; Yan Li; Ying Tao
EPI_ISL_408008	California Department of Health	Pathogen Discovery, Respiratory Viruses Branch, Division of Viral Diseases, Centers for Disease Control and Prevention	Anna Uehara; Brett L. Whitaker; Brian Lynch; Clinton Paden; Janna' R. Murray; Jing Zhang; Krista Queen; Lijuan Wang; Senthil Kumar K. Sakthivel; Shifaq Kamili; Stephen Lindstrom; Susan I. Gerber; Suxiang Tong; Xiaoyan Lu; Yan Li; Ying Tao
EPI_ISL_515894, EPI_ISL_515896 to 515903, EPI_ISL_515905 to 515911, EPI_ISL_515913, EPI_ISL_515917 to 515920, EPI_ISL_515922 to 515925, EPI_ISL_515928 to 515929, EPI_ISL_755015, EPI_ISL_755030 to 755114			
see above	California Department of Public Health	California Department of Public Health	CDPH IDLB COVIDNet
EPI_ISL_413557 to 413559, EPI_ISL_413561, EPI_ISL_413924 to 413925, EPI_ISL_413928, EPI_ISL_755942	California Department of Public Health	Chiu Laboratory, University of California, San Francisco	Candace Wang; Chao-Yang Pan; Charles Chiu; Debra A. Wadford; Debra Wadford; Hugo Guevara; Jill Hacker; Scot Federman; Wei Gu; Xianding (Wayne) Deng; Xianding Deng; and Charles Y. Chiu
EPI_ISL_406034, EPI_ISL_406036, EPI_ISL_410044, EPI_ISL_411954 to 411955	California Department of Public Health	Pathogen Discovery, Respiratory Viruses Branch, Division of Viral Diseases, Centers for Dieases Control and Prevention	Anna Uehara; Brett L. Whitaker; Brian Lynch; Clinton R. Paden; Haibin Wang; Janna' R. Murray; Jing Zhang; Krista Queen; Lijuan Wang; Senthil Kumar K. Sakthivel; Shifaq Kamili; Stephen Lindstrom; Susan I. Gerber; Suxiang Tong; Xiaoyan Lu; Yan Li; Ying Tao
EPI_ISL_412862, EPI_ISL_419554, EPI_ISL_594458	California Department of Public Health	Pathogen Discovery, Respiratory Viruses Branch, Division of Viral Diseases, Centers for Disease Control and Prevention	Anna Uehara; Brett L. Whitaker; Brian Lynch; Clinton Paden; Clinton R. Paden; Haibin Wang; Janna' R. Murray; Jasmine Padilla; Jing Zhang; Julu Bhatnagar; Justin Lee; Krista Queen; Lijuan Wang; Senthil Kumar K. Sakthivel; Shifaq Kamili; Stephen Lindstrom; Susan I. Gerber; Suxiang Tong; Xiaoyan Lu; Yan Li; Ying Tao
EPI_ISL_447594	Caloundra Hospital	Public Health Virology Laboratory	Alyssa Pyke; Amanda De Jong; Andrew Van Den Hurk; Bixing Huang; Carmel Taylor; David Warrilow; Doris Genge; Elisabeth Gamez; Glen Hewitson; Ian Maxwell Mackay; Inga Sultana; Jamie McMahon; Jean Barcelon; Judy Northill; Mitchell Finger; Natalie Simpson; Neelima Nair; Peter Burtonclay; Peter Moore; Sarah Wheatley; Sean Moody; Sonja Hall-Mendelin; Timothy Gardam; and Frederick Moore
EPI_ISL_576371 to 576373	Cancer Biology Department, National Cancer Institute	Cancer Biology Department, National Cancer Institute	A.N.; Abouelhoda, M.; Ahmed; H.K.; Hafez; Hamdy; M.M.; M.S.; O.S.; Soliman; Zekri
EPI_ISL_447054	Cantacuzino National Military-Medical Institute for Research and Development	Cantacuzino Institute	A.Cretu; L.Ustea; M.Lazar
EPI_ISL_456321 to 456322, EPI_ISL_456348, EPI_ISL_456352 to 456353, EPI_ISL_456355 to 456356, EPI_ISL_456358, EPI_ISL_456360, EPI_ISL_456362 to 456367, EPI_ISL_456369 to 456371, EPI_ISL_456374 to 456375, EPI_ISL_547981 to 547985, EPI_ISL_548129, EPI_ISL_548139 to 548140, EPI_ISL_579060 to 579061, EPI_ISL_579063 to 579075, EPI_ISL_579077 to 579085, EPI_ISL_579090, EPI_ISL_579092, EPI_ISL_579220, EPI_ISL_579223, EPI_ISL_579225 to 579227, EPI_ISL_579408 to 579412, EPI_ISL_579414, EPI_ISL_579426 to 579427, EPI_ISL_579429 to 579448, EPI_ISL_579450 to 579471, EPI_ISL_579474 to 579475, EPI_ISL_579478 to 579489, EPI_ISL_579491 to 579492, EPI_ISL_579494 to 579501, EPI_ISL_579503 to 579506, EPI_ISL_622794 to 622801, EPI_ISL_622808 to 622813, EPI_ISL_622824 to 622830, EPI_ISL_682283, EPI_ISL_682286, EPI_ISL_682295 to 682296, EPI_ISL_732963, EPI_ISL_755622			
see above	Canterbury Health Laboratories	Institute of Environmental Science and Research (ESR)	Anja Werno; Antje van der Linden; Arlo Upton; Chris Mansell; David Hammer; Dragana Drinkovic; Erasmus Smit; Gary McAuliffe; Hana Sofia Andersson; Hermes Perez; James Ussher; Jill Sherwood; Jing Wang; Joep de Ligt; Josh Freeman; Julia Howard; Juliet Elvy; Lauren Jelly; Mary DeAlmeida; Matt Blakiston; Matt Storey; Matthew Rogers; Max Bloomfield; Michael Addidle; Michelle Balm; Muhammad Faisal; Nikki Freed; Olin Silander; Sally Roberts; Sarah Jefferies; Sharmini Muttaiyah; Susan Morpeth; Susan Taylor; Timothy Blackmore; Vani Sathyendran; Veronica Playle; Virginia Hope; Xiaoyun Ren
EPI_ISL_730623	Cantonal Hospital Frauenfeld	Institute of Medical Virology, University of Zurich	Alexandra Trkola; Gabriela Ziltener; Jürg Böni; Maryam Zaheri; Michael Huber; Stefan Schmutz; Verena Kufner
EPI_ISL_524478	Cantonal Hospital Winterthur	Institute of Medical Virology, University of Zurich	Alexandra Trkola; Andrea Zbinden; Fiona Steiner; Gabriela Ziltener; Jon Huder; Jürg Böni; Maryam Zaheri; Michael Huber; Patrick Redli; Riccarda Capaul; Stefan Schmutz; Verena Kufner
EPI_ISL_710612	Capio Bro VC	The Public Health Agency of Sweden	Department of Microbiology; The Public Health Agency of Sweden
EPI_ISL_534230 to 534233, EPI_ISL_560981	Capio S:t Gorans sjukhus	The Public Health Agency of Sweden	Anna Risberg; Anna-Malin Linde; Karin Tegmark-Wisell; Maria Lind Karlberg; Mattias Haukland; Mia Brytting; Olov Svartstrom; Oskar Karlsson Lindsjo; Petra Edquist; Reza Advani; Sandra Broddesson
EPI_ISL_583504 to 583505	Casa de Saude Stella Maris	Instituto Adolfo Lutz, Interdiciplinary Procedures Center, Strategic Laboratory	Claudia Regina Gonçalves; Claudio Tavares Sacchi; Erica Valessa Ramos Gomes; Karoline Rodrigues Campos
EPI_ISL_693235	Casmi Centro Atendimento Saude da Mulher e Infancia	Instituto Adolfo Lutz, Interdiciplinary Procedures Center, Strategic Laboratory	Claudia Regina Gonçalves; Claudio Tavares Sacchi; Erica Valessa Ramos Gomes; Karoline Rodrigues Campos
EPI_ISL_467809, EPI_ISL_475574, EPI_ISL_475576 to 475580, EPI_ISL_475582 to 475583, EPI_ISL_475585, EPI_ISL_475588, EPI_ISL_475590, EPI_ISL_475593 to 475596, EPI_ISL_475598 to 475619, EPI_ISL_475621 to 475622, EPI_ISL_475625, EPI_ISL_475627 to 475641, EPI_ISL_475643 to 475648, EPI_ISL_475650 to 475664, EPI_ISL_475666 to 475671, EPI_ISL_475673 to 475675, EPI_ISL_475677 to 475692, EPI_ISL_475694 to 475695, EPI_ISL_475697 to 475703, EPI_ISL_475705 to 475706, EPI_ISL_475708 to 475716			
see above	Cedars-Sinai Medical Center, Department of Pathology & Laboratory Medicine, Molecular Pathology Laboratory	Cedars-Sinai Medical Center, Molecular Pathology Laboratory of Department of Pathology & Laboratory Medicine and Genomic Core	Brian Davis; Eric Vail; Jasmine T Plummer; Jean Lopategui; Jianbo Song; John Paul Govindavari; Jong Taek Kim; Stephanie Chen; Wenjuan Zhang
EPI_ISL_756293 to 756294	Center for Biotechnology and Cell Therapy, São Rafael Hospital, Salvador, Brazil	Center for Biotechnology and Cell Therapy, São Rafael Hospital, Salvador, Brazil	Ana Verena Almeida Mendes; Bruno Solano de Freitas Souza; Carolina Kymie Vasques Nonaka; Marta Giovanetti; Marília Miranda Franco; Renato Santana de Aguiar; Tiago Gräf
EPI_ISL_450413	Center for Diagnostics, Institute of Medical Microbiology,	University Medical Center Hamburg-Eppendorf	Huang, J.; Pfefferle; S. and Fischer, N.

	Virology and Hygiene		
EPI_ISL_459856 to 459863, EPI_ISL_468752, EPI_ISL_468760	Center for Genome Regulation (CRG)	Center for Mathematical Modeling and Center for Genome Regulation. Santiago, Chile	Allende ML; Gaete A; González M.; Maass A; Palma R; Travisany D; Urra C; Varas M
EPI_ISL_479664 to 479665, EPI_ISL_479667 to 479668, EPI_ISL_479671 to 479672, EPI_ISL_479674, EPI_ISL_481283	Center for Genomics and System Biology, New York University	Center for Genomics and System Biology, New York University	Banakis, S.; Borenstein; Cornelius, A.; D. and Ghedin, E.; E.S.; Fleming, A.; Ghedin, E.; Gresham; Gresham, D.; Herati, R.; Johnson, K.; Khalfan, M.; Kottkamp, A.; M.J.; Mulligan; Raabe, V.; Roder, A.; Samanovic, M.; Ulrich, R.
EPI_ISL_516783 to 516785, EPI_ISL_522440 to 522453, EPI_ISL_522455 to 522456, EPI_ISL_522458 to 522463, EPI_ISL_522467 to 522470, EPI_ISL_522472 to 522473, EPI_ISL_522476, EPI_ISL_522479 to 522483, EPI_ISL_522485, EPI_ISL_522491 to 522493, EPI_ISL_522495 to 522496, EPI_ISL_522502, EPI_ISL_522505, EPI_ISL_522510, EPI_ISL_522515 to 522516, EPI_ISL_522518, EPI_ISL_526730 to 526731, EPI_ISL_526734 to 526746			
see above	Center for Laboratory Control of Infectious Diseases, Korea Centers for Diseases Control and Prevention	Center for Laboratory Control of Infectious Diseases, Korea Centers for Diseases Control and Prevention	Ae Kyung Park; Eunkyung Shin; Heui Man Kim; Jeong-Min Kim; Jin Sun No; Junyoung Kim; Myung Guk Han; Yoon-Seok Chung
EPI_ISL_940771	Center for Laboratory Medicine St. Gallen	Center for Laboratory Medicine St. Gallen	Yannick Gerth
EPI_ISL_493137, EPI_ISL_493139	Center for Research and Innovation, Faculty of Medical Technology, Mahidol University	Center for Research and Innovation, Faculty of Medical Technology, Mahidol University	Anek Mungaomklang; Hatairat Lerdsamran; Jarunee Prasertsopon; Kamolthip Atsawawaranunt; Kantima Sangsiriwut; Nattakan Thinpan; Pilaipan Puthavathana; Prabda Praphasiri; Somrak Sirikhetkon; Tipsuda Chanmanee
EPI_ISL_913073, EPI_ISL_913079, EPI_ISL_913091, EPI_ISL_913093	Center for Virology	Center for Virology	Irene Goerzer; Jeremy V. Camp; Monika Redlberger-Fritz; Stephan W. Aberle
EPI_ISL_419654 to 419662, EPI_ISL_419664 to 419667, EPI_ISL_419669 to 419674, EPI_ISL_437993 to 438128, EPI_ISL_475770 to 475776, EPI_ISL_475779 to 475787, EPI_ISL_475790 to 475795, EPI_ISL_475797 to 475806, EPI_ISL_475808 to 475812, EPI_ISL_583565, EPI_ISL_583569, EPI_ISL_583692 to 583694, EPI_ISL_583696 to 583698, EPI_ISL_583700 to 583707, EPI_ISL_583709 to 583716, EPI_ISL_583718 to 583726, EPI_ISL_583869 to 583881			
see above	Center for Virology, Medical University of Vienna	Bergthaler laboratory, CeMM Research Center for Molecular Medicine of the Austrian Academy of Sciences	Adi Steinrigl; Alexander Lercher; Alexandra Popa; Andreas Bergthaler; Benedikt Agerer; Christian Paar; Christoph Bock; Daniela Schmid; Dorothee von Laer; Elisabeth Puchhammer-Stoeckl; Elisabeth Puchhammer-Stöckl; Franz Allerberger; Gernot Walder; Gregor Hörmann; Guenter Weiss; Gunther Vogl; Henrique Colaco; Jakob-Wendelin Genger; Jan Laine; Judith Aberle; Kinga Rigler-Hohenwarter; Lukas Endler; Manfred Nairz; Mark Smyth; Martin Senekowitsch; Michael Schuster; Peter Hufnagl; Peter Obrist; Rainer Gattringer; Sabine Sussitz-Rack; Stephan Aberle; Thomas Penz; Wegene Borena
EPI_ISL_516414 to 516425, EPI_ISL_677700 to 677702, EPI_ISL_677706, EPI_ISL_677708, EPI_ISL_678248 to 678249	Center for public health - Skopje	Research Center for Genetic Engineering and Biotechnology "Georgi D. Efremov" , Macedonian Academy of Sciences and Arts	RCGEB - MASA
EPI_ISL_755572 to 755574	Center of Advanced Studies and Technology, CAST	Center of Advanced Studies and Technology, CAST	De Fabritiis, S.; Ferrante, R.; Mandatori, D.
EPI_ISL_888676, EPI_ISL_1034748 to 1034749, EPI_ISL_1034751 to 1034752	Center of Advanced Studies and Technology, Molecular Genetics Laboratory	Center of Advanced Studies and Technology, Molecular Genetics Laboratory	Anaclerio Federico; Damiani Verena; De Fabritiis Simone; Ferrante Rossella; Mandatori Domitilla
EPI_ISL_1039069	Center of Hygiene and Epidemiology in Belgorod Region	WHO National Influenza Centre Russian Federation	Andrey Komissarov; Anna Ivanova; Artem Fadeev; Daria Danilenko; Dmitry Bazhenov; Dmitry Lioznov; Elena Nabieva; Georgii Bazykin; Ksenia Safina; Kseniya Komissarova; Lyudmila Berdinskikh; Maria Pisareva; Maria Timofeeva; Tamila Musaeva; Veronika Eder
EPI_ISL_523950	Center of Medical Microbiology, Virology, and Hospital Hygiene, University of Duesseldorf	Center of Medical Microbiology, Virology, and Hospital Hygiene, Heinrich Heine University Düsseldorf	Alexander Dilthey; Andreas Walker; Daniel Strelow; Hendrik Streeck; Jessica Nicolai; Jörg Timm; Klaus Pfeffer; Malte Kohns Vasconcelos; Marek Korencak; Maximilian Damagnez; Tobias Wienemann; Torsten Houwaart
EPI_ISL_413488, EPI_ISL_414497 to 414499, EPI_ISL_414505, EPI_ISL_414508 to 414509, EPI_ISL_417457 to 417468, EPI_ISL_419541 to 419552, EPI_ISL_425121 to 425128, EPI_ISL_425130 to 425132, EPI_ISL_425138 to 425139, EPI_ISL_523927, EPI_ISL_523929, EPI_ISL_523931 to 523935, EPI_ISL_523937 to 523945, EPI_ISL_523947 to 523949, EPI_ISL_602513, EPI_ISL_602517			
see above	Center of Medical Microbiology, Virology, and Hospital Hygiene, University of Duesseldorf	Center of Medical Microbiology, Virology, and Hospital Hygiene, University of Duesseldorf	Alexander Dilthey; Andreas Walker; Bjorn-Erik Jensen; Björn-Erik Jensen; Daniel Strelow; Detlef Kindgen-Milles; Hendrik Streeck; Jessica Nicolai; Jorg Timm; Jörg Timm; Klaus Pfeffer; Lisanna Hülse; Malte Kohns Vasconcelos; Marcel Andree; Marek Korencak; Maximilian Damagnez; Nadine Lübke; Ortwin Adams; Sandra Hauka; Tina Senff; Tobias Wienemann; Torsten Feldt; Torsten Houwaart
EPI_ISL_430820	Center of Scientific Excellence for Influenza Viruses, National Research Centre (NRC), Egypt.	Center of Scientific Excellence for Influenza Viruses, National Research Centre (NRC), Egypt.	Abo Shama; Ahmed E Kayed; Ahmed El-Taweel; Ahmed Kandeil; Ahmed Mostafa; Amal Naguib; M Noura; Mahmoud Shehata; Mina Kamel; Mohamed Ahmed Ali; Mohamed El Sayes; Mokhtar Gomaa; Nancy M. El Guindy; Omnia Kutkat; Rabeh El-Shesheny; Sara Mahmoud; Shymaa Showky Ahmed; Wael Roshdy; Yassmin Moatasim
EPI_ISL_430819	Center of Scientific Excellence for Influenza Viruses,National Research Centre (NRC), Egypt.	Center of Scientific Excellence for Influenza Viruses,National Research Centre (NRC), Egypt.	Abo Shama; Ahmed E Kayed; Ahmed El-Taweel; Ahmed Kandeil; Ahmed Mostafa; Amal Naguib; M Noura; Mahmoud Shehata; Mina Kamel; Mohamed Ahmed Ali; Mohamed El Sayes; Mokhtar Gomaa; Nancy M. El Guindy; Omnia Kutkat; Rabeh El-Shesheny; Sara Mahmoud; Shymaa Showky Ahmed; Wael Roshdy; Yassmin Moatasim
EPI_ISL_429852 to 429854, EPI_ISL_495459	Centers for Disease Control and Prevention of Lishui	Department of InspectionCenters for Disease Control and Prevention of Lishui	Ji Jiansong; Ji Qiaoying; Wang Xiaoguang; Ye Bifeng; Ye Ling
EPI_ISL_539495	Centers for Disease Control and Prevention, Dengue Branch	Centers for Disease Control and Prevention, Dengue Branch	Betzabel Flores; Diego Sainz de la Peña; Gabriela Paz-Bailey; Gilberto A. Santiago; Glenda Gonzalez; Janice Perez; Jorge Bertran; Jorge L. Munoz-Jordan; Keyla Charriez; Vanessa Rivera-Amill
EPI_ISL_406031, EPI_ISL_420082 to 420085, EPI_ISL_421641, EPI_ISL_421651, EPI_ISL_428488 to 428491, EPI_ISL_429882 to 429884	Centers for Disease Control, R.O.C. (Taiwan)	Centers for Disease Control, R.O.C. (Taiwan)	Ji-Rong Yang; Jung-Jung Mu; Ming-Tsan Liu; Ming-Tsan-Liu; Shu-Ying Li; Yu-Chi Lin; Yu-Chi-Lin
EPI_ISL_815264, EPI_ISL_815269, EPI_ISL_815271, EPI_ISL_815274, EPI_ISL_815276, EPI_ISL_815280, EPI_ISL_815282, EPI_ISL_815284, EPI_ISL_815312 to 815313, EPI_ISL_815330, EPI_ISL_815334, EPI_ISL_815366 to 815367			
see above	Centogene	Centogene	Krishna Kumar Kandaswamy; Peter Bauer; Vivi Hue-Trang Lieu
EPI_ISL_457750, EPI_ISL_459962 to 459964	Centogene AG	Centogene AG	Dr. Krishna Kumar Kandaswamy; Prof. Dr. Peter Bauer
EPI_ISL_455583, EPI_ISL_455594	Central Chest Institute of Thailand	National Institute of Health. Department of medical Sciences, Ministry of Public Health, Thailand	Chittaganpitch; Malinee; Okada; Parnmen; Phuygun; Pilailuk; Siripaporn; Sittiporn; Sunthareeya; Thanadachakul; Thanutsapa; Waicharoen; Warawan; Wongboot
EPI_ISL_529032	Central Molecular Microbiology Laboratory and Next Generation Sequencing Reference Laboratory, Clinical and Chemical Pathology Department, Faculty of Medicine, CAIRO UNIVERSITY	Next Generation Sequencing Reference Laboratory, Faculty of Medicine, CAIRO UNIVERSITY and The Center for Genome and Microbiome Research, Faculty of Pharmacy, CAIRO UNIVERSITY	May Abdelfattah; May Sherif Soliman; Ramy Karam Aziz
EPI_ISL_529031	Central Molecular Microbiology Laboratory, Clinical and Chemical Pathology Department, Faculty of Medicine, CAIRO UNIVERSITY	Next Generation Sequencing Reference Laboratory, Faculty of Medicine, Cairo University and The Center for Genome and Microbiome Research, Faculty of Pharmacy, CAIRO UNIVERSITY	May Abdelfattah; May Sherif Soliman; Ramy Karam Aziz
EPI_ISL_693471 to 693483	Central Public Health Laboratory	National Public Health Laboratory, National Centre for Infectious Diseases	Esorom Daoni; Lin Cui; Raymond Tzer Pin Lin; Sophie Octavia; Theresa Palou; Tze Minn Mak; Zhenyang Zhou
EPI_ISL_429667, EPI_ISL_429669, EPI_ISL_429671, EPI_ISL_429674, EPI_ISL_429676, EPI_ISL_429681, EPI_ISL_429687 to 429689, EPI_ISL_429695, EPI_ISL_429702			
see above	Central Public Health Laboratory/Octávio Magalhães Institute (IOM) from the Ezequiel Dias Foundation (FUNED)	Instituto Octávio Magalhães / Fundação Ezequiel Dias (IOM/Funed)	Joilson Xavier; Luiz Carlos Junior Alcantara; Marcos Vinícius Silva; Marluce Aparecida Assunção Oliveira; Marta Giovanetti; Talita Adelino; Vagner Fonseca
EPI_ISL_447251	Central Virology Laboratory	Central Virology Laboratory	Danit Sofer; Efrat Bucris; Ella Mendelson; Michal Mandelboim; Neta Zuckerman; Oran Erster; Orna Mor
EPI_ISL_419211	Central Virology Laboratory	Israel Institute for Biological Research	Adi Beth-Din; Anat Zvi; Boaz Politi; Dana Stein; Einat Vitner; Gadi Segal; Gili Regev-Yochay; Hadas Tamir; Hagit Achdout; Inbar Cohen-Gihon; Lilach Cherry; Michal Mandelboim; Nir Paran; Ofir Israeli; Ohad Shifman; Oran Erster; Orly Laskar; Sharon Melamed; Shay Weiss; Shmuel C. Shapira; Shmuel Yitzhaki; Tomer Israely; Yfat Yahalom Ronen

EPI_ISL_435284, EPI_ISL_435286, EPI_ISL_435289, EPI_ISL_435291 to 435292	Central Virology Laboratory, Israel Ministry of Health	Central Virology Laboratory, Israel Ministry of Health	Danit Sofer; Efrat Bucris; Ella Mendelson; Michal Mandelboim; Neta Zuckerman; Oran Erster; Orna Mor; eta Zuckerman
EPI_ISL_430842	Central chest Institute of Thailand	National Institute of Health. Department of medical Sciences, Ministry of Public Health, Thailand	Chittaganpitch; Malinee; Okada; Parnmen; Phuygun; Pilailuk; Siripaporn; Sittiporn; Sunthareeya; Thanadachakul; Thanutsapa; Waicharoen; Warawan; Wongboot
EPI_ISL_486410, EPI_ISL_486417, EPI_ISL_961812 to 961814, EPI_ISL_961834, EPI_ISL_961838, EPI_ISL_961841, EPI_ISL_961849			
see above	Centrala laboratorija	Latvian Biomedical Research and Study Centre	Davids Fridmanis; Ivars Silamielis; Ivars Silamikelis; Jana Osite; Jana Oste; Janis Pjalkovskis; Janis Klovins; Jurijs Perevoscikovs; Jnis Kloviš; Kaspars Megnis; Kaspars Megnis; Laila Silamikele; Lauma Freimane; Laura Ansone; Liga Birzniece; Marta Priedite; Marta Priedte; Monta Ustinova; Monta Ustinova; Nikita Zrelovs; Stella Lapia; Uga Dumpis; Uga Dumpis; Vita Rovte; Vita Rovite; ikita Zrelovs
EPI_ISL_548256	Centralsjukhuset	The Public Health Agency of Sweden	Anna Risberg; Anna-Malin Linde; Karin Tegmark-Wisell; Maria Lind Karlberg; Mattias Haukland; Mia Brytting; Olov Svartstrom; Oskar Karlsson Lindsjo; Petra Edquist; Reza Advani; Sandra Broddesson
EPI_ISL_458000	Centre For Biotechnology Research and Development	Centre For Biotechnology Research and Development	C.N. and Michuki; D.K.; G.N.; J.O.; Kimotho, J.; Matoke-Muhia; Muuo; Ochwoto, M.; S.L.; S.N.; Symeker; Waruhiu; Zablon
EPI_ISL_414624, EPI_ISL_416494	Centre Hositalier Universitaire de Rouen Laboratoire de Virologie	National Reference Center for Viruses of Respiratory Infections, Institut Pasteur, Paris	Angela Brisebarre; Etienne Simon-Lorière; Flora Donati; Flora Donati Vincent Enouf; Jean-Christophe Plantier; Marion Barbet; Maud Vanpeene; Méline Bizard; Mélnie Albert; Sylvie Behillil; Sylvie van der Werf; Vincent Enouf
EPI_ISL_508958, EPI_ISL_509012	Centre Hospitalier Alpes Leman	CNR Virus des Infections Respiratoires - France SUD	Alexandre Gaymard; Antonin Bal; Bruno Lina; Carine Moustaud; Florence Morfin-Sherpa; Gregory Destras; Gwendolyne Burfin; Laurence Josset; Martine Valette; Maude Bouscambert-Duchamp; Raphaelle Lamy; Solenne Brun
EPI_ISL_414627 to 414630, EPI_ISL_414634 to 414638, EPI_ISL_415654, EPI_ISL_416495 to 416497, EPI_ISL_418218, EPI_ISL_418220 to 418221, EPI_ISL_418223 to 418225, EPI_ISL_418227 to 418228, EPI_ISL_418231, EPI_ISL_418236 to 418239, EPI_ISL_429968			
see above	Centre Hospitalier Compiègne Laboratoire de Biologie	National Reference Center for Viruses of Respiratory Infections, Institut Pasteur, Paris	Angela Brisebarre; Etienne Simon-Lorière; Fabiana Gambaro; Flora Donati; Flora Donati Vincent Enouf; Marion Barbet; Maud Vanpeene; Mélanie Albert; Méline Bizard; Mélnie Albert; Raulin Olivia; Sylvie Behillil; Sylvie van der Werf; Vincent Enouf
EPI_ISL_860847	Centre Hospitalier Compiègne-Noyon - Laboratoire	National Reference Center for Viruses of Respiratory Infections, Institut Pasteur, Paris	Angela Brisebarre; Camille Capel; Etienne Simon-Lorière; Foissaud Vincent; Marion Barbet; Maud Vanpeene; Méline Bizard; Sylvie Behillil; Sylvie van der Werf; Vincent Enouf
EPI_ISL_418428, EPI_ISL_508947	Centre Hospitalier Lucien Hussel	CNR Virus des Infections Respiratoires - France SUD	Alexandre Gaymard; Antonin Bal; Bruno Lina; Carine Moustaud; Florence Morfin-Sherpa; Gregory Destras; Gwendolyne Burfin; Laurence Josset; Martine Valette; Maude Bouscambert-Duchamp; Raphaelle Lamy; Solenne Brun
EPI_ISL_508966, EPI_ISL_508975, EPI_ISL_508998, EPI_ISL_636478, EPI_ISL_636480, EPI_ISL_636482 to 636483, EPI_ISL_636486			
see above	Centre Hospitalier Pierre Oudot	CNR Virus des Infections Respiratoires - France SUD	Alexandre Gaymard; Antonin Bal; Bruno Lina; Carine Moustaud; Florence Morfin-Sherpa; Gregory Destras; Gwendolyne Burfin; Hadrien Règue; Laurence Josset; Martine Valette; Maude Bouscambert-Duchamp; Raphaelle Lamy; Solenne Brun
EPI_ISL_414633	Centre Hospitalier René Dubois Laboratoire de Microbiologie - Bât A	National Reference Center for Viruses of Respiratory Infections, Institut Pasteur, Paris	Angela Brisebarre; Flora Donati Vincent Enouf; Marion Barbet; Maud Vanpeene; Méline Bizard; Mélnie Albert; Pascale Martres; Sylvie Behillil; Sylvie van der Werf
EPI_ISL_414625	Centre Hospitalier Régional Universitaire de Nantes Laboratoire de Virologie	National Reference Center for Viruses of Respiratory Infections, Institut Pasteur, Paris	Angela Brisebarre; Flora Donati Vincent Enouf; Marianne Coste-Burel; Marion Barbet; Maud Vanpeene; Méline Bizard; Mélnie Albert; Sylvie Behillil; Sylvie van der Werf
EPI_ISL_535787 to 535788, EPI_ISL_535790, EPI_ISL_535842, EPI_ISL_535844, EPI_ISL_535871, EPI_ISL_535896, EPI_ISL_535907, EPI_ISL_535931 to 535935, EPI_ISL_535955, EPI_ISL_536002, EPI_ISL_536005 to 536006, EPI_ISL_536009, EPI_ISL_536014 to 536016, EPI_ISL_536047, EPI_ISL_536050, EPI_ISL_536052 to 536054, EPI_ISL_536059 to 536060, EPI_ISL_536063, EPI_ISL_536073 to 536074, EPI_ISL_536076, EPI_ISL_536079, EPI_ISL_536120, EPI_ISL_536126, EPI_ISL_536129 to 536130, EPI_ISL_536134, EPI_ISL_536138, EPI_ISL_536151, EPI_ISL_536153 to 536154, EPI_ISL_536381			
see above	Centre Hospitalier Régional de Lanaudière	Laboratoire de santé publique du Québec	Guillaume Bourque; Ioannis Ragoussis; Jesse Shapiro; Mark Lathrop and Michel Roger; Mark Lathrop and Michel Roger on behalf of the CoVSeQ research group; Sandrine Moreira
EPI_ISL_418418 to 418419, EPI_ISL_420617, EPI_ISL_508879 to 508880, EPI_ISL_508959 to 508960, EPI_ISL_639978	Centre Hospitalier Saint Joseph Saint Luc	CNR Virus des Infections Respiratoires - France SUD	Alexandre Gaymard; Antonin Bal; Bruno Lina; Carine Moustaud; Florence Morfin-Sherpa; Gregory Destras; Gwendolyne Burfin; Hadrien Règue; Laurence Josset; Martine Valette; Maude Bouscambert-Duchamp; Raphaelle Lamy; Solenne Brun
EPI_ISL_416757, EPI_ISL_417340, EPI_ISL_418426, EPI_ISL_419183, EPI_ISL_419185 to 419186, EPI_ISL_420620, EPI_ISL_508938, EPI_ISL_508944, EPI_ISL_508968, EPI_ISL_525539, EPI_ISL_582121, EPI_ISL_636481, EPI_ISL_639984			
see above	Centre Hospitalier de Bourg en Bresse	CNR Virus des Infections Respiratoires - France SUD	Alexandre; Alexandre Gaymard; Antonin; Antonin Bal; Bal; Bouscambert-Duchamp; Brengel-Pesce; Bruno Lina; Bruno.; Carine Moustaud; Cheynet; Destras; Florence; Florence Morfin-Sherpa; Gaymard; Gregory; Gregory Destras; Gwendolyne Burfin; Hadrien Règue; Josset; Karen; Laurence; Laurence Josset; Lina; Martine; Martine Valette; Maude; Maude Bouscambert-Duchamp; Morfin-Sherpa; Raphaelle Lamy; Solenne Brun; Valette; Valérie
EPI_ISL_912557 to 912558	Centre Hospitalier de Gisors Route de Rouen	National Reference Center for Viruses of Respiratory Infections, Institut Pasteur, Paris	Angela Brisebarre; Camille Capel; Etienne Simon-Lorière; Marion Barbet; Maud Vanpeene; Méline Bizard; Sylvie Behillil; Sylvie van der Werf; Vincent Enouf
EPI_ISL_417338, EPI_ISL_418413, EPI_ISL_419174 to 419176, EPI_ISL_419187 to 419188, EPI_ISL_420613 to 420614, EPI_ISL_508875 to 508876, EPI_ISL_508943, EPI_ISL_508946, EPI_ISL_508949 to 508950, EPI_ISL_508952, EPI_ISL_509005			
see above	Centre Hospitalier de Macon	CNR Virus des Infections Respiratoires - France SUD	Alexandre Gaymard; Antonin Bal; Bruno Lina; Carine Moustaud; Florence Morfin-Sherpa; Gregory Destras; Gwendolyne Burfin; Laurence Josset; Martine Valette; Maude Bouscambert-Duchamp; Raphaelle Lamy; Solenne Brun
EPI_ISL_416749, EPI_ISL_418414, EPI_ISL_418417, EPI_ISL_419168, EPI_ISL_508881	Centre Hospitalier de Valence	CNR Virus des Infections Respiratoires - France SUD	Alexandre; Alexandre Gaymard; Antonin; Antonin Bal; Bal; Bouscambert-Duchamp; Brengel-Pesce; Bruno Lina; Bruno.; Carine Moustaud; Cheynet; Destras; Florence; Florence Morfin-Sherpa; Gaymard; Gregory; Gregory Destras; Gwendolyne Burfin; Josset; Karen; Laurence; Laurence Josset; Lina; Martine; Martine Valette; Maude; Maude Bouscambert-Duchamp; Morfin-Sherpa; Raphaelle Lamy; Solenne Brun; Valette; Valérie
EPI_ISL_508932, EPI_ISL_508978, EPI_ISL_509006, EPI_ISL_509015	Centre Hospitalier de Villefranche	CNR Virus des Infections Respiratoires - France SUD	Alexandre Gaymard; Antonin Bal; Bruno Lina; Carine Moustaud; Florence Morfin-Sherpa; Gregory Destras; Gwendolyne Burfin; Laurence Josset; Martine Valette; Maude Bouscambert-Duchamp; Raphaelle Lamy; Solenne Brun
EPI_ISL_418412	Centre Hospitalier des Vals d'Ardeche	CNR Virus des Infections Respiratoires - France SUD	Alexandre Gaymard; Antonin Bal; Bruno Lina; Carine Moustaud; Florence Morfin-Sherpa; Gregory Destras; Gwendolyne Burfin; Laurence Josset; Martine Valette; Maude Bouscambert-Duchamp; Raphaelle Lamy; Solenne Brun
EPI_ISL_509016	Centre Hospitalier du Haut-Bugey	CNR Virus des Infections Respiratoires - France SUD	Alexandre Gaymard; Antonin Bal; Bruno Lina; Carine Moustaud; Florence Morfin-Sherpa; Gregory Destras; Gwendolyne Burfin; Laurence Josset; Martine Valette; Maude Bouscambert-Duchamp; Raphaelle Lamy; Solenne Brun
EPI_ISL_539573 to 539576	Centre de Recherches Medicales de Lambarene (CERMEL)	Department of Emerging Infectious Diseases, Institute of Tropical Medicine, Nagasaki University	Akim A. Adegnika; Bertrand Lell; Haruka Abe; Jiro Yasuda; Rodrigue Bikangui; Yuri Ushijima
EPI_ISL_535940	Centre de SSS D'Arthabaska-et-de-l'érable - Hôtel-Dieu	Laboratoire de santé publique du Québec	Guillaume Bourque; Ioannis Ragoussis; Jesse Shapiro; Mark Lathrop and Michel Roger on behalf of the CoVSeQ research group; Sandrine Moreira
EPI_ISL_535724, EPI_ISL_535742 to 535743	Centre de SSS La Pommeraie	Laboratoire de santé publique du Québec	Guillaume Bourque; Ioannis Ragoussis; Jesse Shapiro; Mark Lathrop and Michel Roger; Mark Lathrop and Michel Roger on behalf of the CoVSeQ research group; Sandrine Moreira
EPI_ISL_535722, EPI_ISL_535748, EPI_ISL_535916, EPI_ISL_535976 to 535977, EPI_ISL_535979, EPI_ISL_536007, EPI_ISL_536031, EPI_ISL_536034 to 536036, EPI_ISL_536038			
see above	Centre de SSS de Trois-Rivières	Laboratoire de santé publique du Québec	Guillaume Bourque; Ioannis Ragoussis; Jesse Shapiro; Mark Lathrop and Michel Roger; Mark Lathrop and Michel Roger on behalf of the CoVSeQ research group; Sandrine Moreira
EPI_ISL_535727, EPI_ISL_535776 to 535780, EPI_ISL_535803 to 535805, EPI_ISL_535816, EPI_ISL_535854	Centre de SSS de la Haute-Yamaska	Laboratoire de santé publique du Québec	Guillaume Bourque; Ioannis Ragoussis; Jesse Shapiro; Mark Lathrop and Michel Roger; Mark Lathrop and Michel Roger on behalf of the CoVSeQ research group; Sandrine Moreira
EPI_ISL_471456 to 471460	Centre de Virologie des Maladies Tropicales	Functional Genomic Platform/Service Analyses Biologique/UATRS/ Centre National Pour la Recherche Scientifique Et Technique (CNRST)	Abdelillah LARAQUI; Abdelkader LAATIRIS; Ahmed REGGAD; Elmostafa EL FAHIME; Farida HILALI; Hicham EL ANNAZ; Idriss-Amine LAHLOU; Khalid ENNIBI; Marouane MELLOUL; Mly Abdelaziz ELALAOUI; Mostafa ELOUENNASS; Nadia TOUIL; Rachid ABI; Rida TAGAJDID; Safae ELKOCHRI; Sanaa ALAOUI-Amine; Tahar BAJJOU; Yassine SEKHSOKH; Youssef AKHOUAD; Zohour KASMY
EPI_ISL_535808	Centre de santé Chibougamau	Laboratoire de santé publique du Québec	Guillaume Bourque; Ioannis Ragoussis; Jesse Shapiro; Mark Lathrop and Michel Roger; Sandrine Moreira

EPI_ISL_443310	Centre de santé Filieris	National Reference Center for Viruses of Respiratory Infections, Institut Pasteur, Paris	Angela Brisebarre; Etienne Simon-Lorière; Flora Donati; Marion Barbet; Maud Vanpeene; Mélanie Albert; Méline Bizard; Sylvie Behillil; Sylvie van der Werf; Vincent Enouf
EPI_ISL_465697, EPI_ISL_536353	Centre de santé Inuulitsivik	Laboratoire de santé publique du Québec	Guillaume Bourque; Ioannis Ragoussis; Jesse Shapiro; Mark Lathrop and Michel Roger on behalf of the CoVSeQ research group; Sandrine Moreira
EPI_ISL_466873	Centre for Clinical Infection and Diagnostics Research and Genomics Innovation Unit	Respiratory Virus Unit, Microbiology Services Colindale, Public Health England	Ali Awan; Chloe Fisher; Gaia Nebbia; Luke Snell; PHE Covid Sequencing Team
EPI_ISL_483923, EPI_ISL_483931, EPI_ISL_483937, EPI_ISL_483945, EPI_ISL_483947 to 483948, EPI_ISL_483952, EPI_ISL_483959, EPI_ISL_483961, EPI_ISL_483964, EPI_ISL_483966 to 483968, EPI_ISL_483971, EPI_ISL_483973, EPI_ISL_483976, EPI_ISL_483980, EPI_ISL_483982, EPI_ISL_483985 to 483987, EPI_ISL_483989, EPI_ISL_483992, EPI_ISL_483995, EPI_ISL_483999, EPI_ISL_484002, EPI_ISL_484005, EPI_ISL_484008, EPI_ISL_484012, EPI_ISL_484019 to 484020, EPI_ISL_484028, EPI_ISL_484034, EPI_ISL_484036, EPI_ISL_484053, EPI_ISL_484056, EPI_ISL_484059, EPI_ISL_484064, EPI_ISL_484070, EPI_ISL_484072, EPI_ISL_484079, EPI_ISL_484082, EPI_ISL_484085, EPI_ISL_484087, EPI_ISL_484091, EPI_ISL_484093, EPI_ISL_484095 to 484096, EPI_ISL_484104, EPI_ISL_484110, EPI_ISL_484115, EPI_ISL_484117, EPI_ISL_484125 to 484126, EPI_ISL_484129, EPI_ISL_484131, EPI_ISL_484134, EPI_ISL_484136, EPI_ISL_484145, EPI_ISL_484150, EPI_ISL_484152, EPI_ISL_484169 to 484170, EPI_ISL_484174, EPI_ISL_484189, EPI_ISL_484191			
see above	Centre for Clinical Infection and Diagnostics Research and Genomics Innovation Unit, Guy's and St. Thomas' NHS Trust	COVID-19 Genomics UK (COG-UK) Consortium	Ali Raza Awan; Chloe Fisher; Jonathan Edgeworth; Luke Snell; Penny Cliff; Rahul Batra
EPI_ISL_560650 to 560652, EPI_ISL_560656, EPI_ISL_560658, EPI_ISL_560660 to 560661, EPI_ISL_560668, EPI_ISL_560671 to 560673, EPI_ISL_560676, EPI_ISL_560678 to 560679, EPI_ISL_560684, EPI_ISL_560686, EPI_ISL_560695 to 560702, EPI_ISL_560705, EPI_ISL_560712, EPI_ISL_560719, EPI_ISL_560722 to 560727, EPI_ISL_560729 to 560731, EPI_ISL_560733 to 560734, EPI_ISL_560736 to 560737, EPI_ISL_560739 to 560740, EPI_ISL_561373			
see above	Centre for Clinical Infection and Diagnostics Research and Genomics Innovation Unit, Guy's and St. Thomas' NHS Trust	Centre for Clinical Infection and Diagnostics Research and Genomics Innovation Unit, Guy's and St. Thomas' NHS Trust	Ali Raza Awan; Chloe Fisher; Jonathan Edgeworth; Luke Snell; Rahul Batra
EPI_ISL_428670 to 428673, EPI_ISL_525476, EPI_ISL_525478 to 525479, EPI_ISL_525481, EPI_ISL_525486, EPI_ISL_525489	Centre for Dengue Research	Centre for Dengue Research	Ananda Wijewickrama; Chandima Jeewandara; Damayanthi Idampitiya; Damayanthi Tdampitiya; Deshni Jayathilaka; Dinuka Ariyaratne; Dinuka Ariyatane; Dinuka Guruge; Diyanath Ranasinghe; Eranga Narangoda; Gathsaurie Neelika Malavige; Laksiri Gomes; Malika Karunaratne; Neelika Malaige; Neelika Malavige; Ruwan Wijayamuni
EPI_ISL_525474	Centre for Dengue Research	Centre for Dengue Research, USJ, SL	Chandima Jeewandara; Deshni Jayathilaka; Dinuka Ariyaratne; Dinuka Guruge; Diyanath Ranasinghe; Gathsaurie Neelika Malavige; Laksiri Gomes; Ruwan Wijayamuni
EPI_ISL_602564	Centre for Dengue Research, Department of Immunology and Molecular Medicine	Centre for Dengue Research	Ananda Wijewickrama; Chandima Jeewandara; Deshni Jayathilaka; Dinuka Ariyaratne; Diyanath Ranasinghe; Gathsaurie Neelika Malavige; Laksiri Gomes; Malika Karunaratne
EPI_ISL_668454	Centre for Dengue Research, Department of Immunology and Molecular Medicine	Centre for Dengue Research, Department of Immunology and Molecular Medicine	Chandima Jeewandara; Deshni Jayathilaka; Dinuka Ariyaratne; Diyanath Ranasinghe; Gathsaurie Neelika Malavige; Laksiri Gomes
EPI_ISL_453670, EPI_ISL_453676 to 453677, EPI_ISL_453679, EPI_ISL_453684 to 453685, EPI_ISL_453687, EPI_ISL_453691, EPI_ISL_461971 to 461977, EPI_ISL_461979, EPI_ISL_461981, EPI_ISL_461983, EPI_ISL_461986 to 461987, EPI_ISL_461989, EPI_ISL_461991, EPI_ISL_461998, EPI_ISL_475240 to 475242, EPI_ISL_475244, EPI_ISL_475247, EPI_ISL_475263, EPI_ISL_475266 to 475269, EPI_ISL_475271 to 475274, EPI_ISL_475276, EPI_ISL_475279 to 475280, EPI_ISL_475282 to 475289, EPI_ISL_475291 to 475293, EPI_ISL_475296 to 475300, EPI_ISL_475302 to 475304, EPI_ISL_475306 to 475307, EPI_ISL_475310 to 475312, EPI_ISL_475315 to 475316, EPI_ISL_475318 to 475323, EPI_ISL_475325 to 475331, EPI_ISL_475333 to 475335, EPI_ISL_475337, EPI_ISL_475339, EPI_ISL_475341, EPI_ISL_479175 to 479194, EPI_ISL_484407, EPI_ISL_484410 to 484413, EPI_ISL_484415 to 484419, EPI_ISL_484421 to 484424, EPI_ISL_484427 to 484430, EPI_ISL_493614 to 493615, EPI_ISL_493617, EPI_ISL_493620 to 493622, EPI_ISL_493624, EPI_ISL_493626, EPI_ISL_493628 to 493629, EPI_ISL_493633 to 493634, EPI_ISL_499272, EPI_ISL_499278, EPI_ISL_499281 to 499283, EPI_ISL_499285, EPI_ISL_499288 to 499289, EPI_ISL_499294 to 499298, EPI_ISL_499300, EPI_ISL_499302 to 499304, EPI_ISL_499306 to 499312, EPI_ISL_499314 to 499316, EPI_ISL_499318, EPI_ISL_499322 to 499325, EPI_ISL_499327 to 499329, EPI_ISL_507131 to 507135, EPI_ISL_507137, EPI_ISL_507139 to 507143, EPI_ISL_507146 to 507149, EPI_ISL_507152 to 507153, EPI_ISL_507155, EPI_ISL_507159, EPI_ISL_512389, EPI_ISL_512395, EPI_ISL_512400, EPI_ISL_512407, EPI_ISL_512409 to 512412, EPI_ISL_512415 to 512417, EPI_ISL_512426, EPI_ISL_512430, EPI_ISL_512432 to 512433, EPI_ISL_514457 to 514463, EPI_ISL_514465 to 514469, EPI_ISL_514472 to 514480, EPI_ISL_514482, EPI_ISL_514484 to 514486, EPI_ISL_514490, EPI_ISL_514505 to 514506, EPI_ISL_514508, EPI_ISL_514510, EPI_ISL_517512 to 517513, EPI_ISL_517517, EPI_ISL_517519, EPI_ISL_517522, EPI_ISL_517526, EPI_ISL_517529, EPI_ISL_517532, EPI_ISL_517535 to 517537, EPI_ISL_517540, EPI_ISL_517542, EPI_ISL_526434 to 526436, EPI_ISL_529668 to 529674, EPI_ISL_535024 to 535025, EPI_ISL_535027 to 535029, EPI_ISL_535031 to 535032, EPI_ISL_535036, EPI_ISL_535038, EPI_ISL_549343, EPI_ISL_549460 to 549476, EPI_ISL_572715 to 572716, EPI_ISL_572721, EPI_ISL_573253 to 573265, EPI_ISL_573870, EPI_ISL_577060 to 577061, EPI_ISL_577299, EPI_ISL_577310, EPI_ISL_577333, EPI_ISL_577335, EPI_ISL_577339 to 577340, EPI_ISL_577382, EPI_ISL_577387, EPI_ISL_595105 to 595106, EPI_ISL_595109 to 595111, EPI_ISL_595114, EPI_ISL_611971, EPI_ISL_613282 to 613306, EPI_ISL_628312 to 628333, EPI_ISL_638050, EPI_ISL_638065, EPI_ISL_638249 to 638257, EPI_ISL_638265 to 638292, EPI_ISL_638454 to 638458, EPI_ISL_638911 to 638913, EPI_ISL_639006 to 639007, EPI_ISL_651099, EPI_ISL_652103 to 652110, EPI_ISL_652620 to 652626, EPI_ISL_664746, EPI_ISL_664763, EPI_ISL_664840 to 664855, EPI_ISL_665652, EPI_ISL_665849, EPI_ISL_665946 to 665955, EPI_ISL_666107 to 666109, EPI_ISL_679981, EPI_ISL_679985, EPI_ISL_680019 to 680020, EPI_ISL_680029, EPI_ISL_680564, EPI_ISL_706864 to 706890, EPI_ISL_723530 to 723531, EPI_ISL_723535 to 723536, EPI_ISL_723540, EPI_ISL_723546, EPI_ISL_723551 to 723552, EPI_ISL_723554 to 723555, EPI_ISL_723557, EPI_ISL_727740, EPI_ISL_727751, EPI_ISL_727755, EPI_ISL_727764 to 727765, EPI_ISL_727767 to 727768, EPI_ISL_727771 to 727774, EPI_ISL_727776, EPI_ISL_727781 to 727782, EPI_ISL_727784, EPI_ISL_727787, EPI_ISL_727791 to 727792, EPI_ISL_727798, EPI_ISL_727801, EPI_ISL_727804 to 727805, EPI_ISL_741348, EPI_ISL_741658 to 741659, EPI_ISL_741672, EPI_ISL_741675, EPI_ISL_741680 to 741684, EPI_ISL_741694, EPI_ISL_741707, EPI_ISL_741711 to 741712, EPI_ISL_741714, EPI_ISL_741717 to 741721, EPI_ISL_741723 to 741724, EPI_ISL_741726, EPI_ISL_741730, EPI_ISL_741732 to 741737, EPI_ISL_741742 to 741744, EPI_ISL_741746 to 741749, EPI_ISL_741751, EPI_ISL_741753, EPI_ISL_741755 to 741756, EPI_ISL_741758 to 741759, EPI_ISL_763828 to 763837, EPI_ISL_763865, EPI_ISL_764006 to 764031, EPI_ISL_764036, EPI_ISL_764081 to 764082, EPI_ISL_764094 to 764098, EPI_ISL_764101 to 764102, EPI_ISL_764118 to 764125, EPI_ISL_765112 to 765148, EPI_ISL_816137 to 816140, EPI_ISL_816142, EPI_ISL_816144, EPI_ISL_816146 to 816147, EPI_ISL_816150, EPI_ISL_816153 to 816155, EPI_ISL_816159, EPI_ISL_816162, EPI_ISL_816165, EPI_ISL_816172 to 816173, EPI_ISL_816175 to 816176, EPI_ISL_816178 to 816180, EPI_ISL_816182, EPI_ISL_816185 to 816187, EPI_ISL_816195, EPI_ISL_819382 to 819383, EPI_ISL_819422 to 819423, EPI_ISL_819426, EPI_ISL_841759, EPI_ISL_841766, EPI_ISL_841772, EPI_ISL_841783, EPI_ISL_841787, EPI_ISL_841872, EPI_ISL_841875 to 841876, EPI_ISL_841878, EPI_ISL_841883, EPI_ISL_841885, EPI_ISL_841888, EPI_ISL_841890, EPI_ISL_841893 to 841894, EPI_ISL_841896 to 841902, EPI_ISL_841907 to 841908, EPI_ISL_841910 to 841911, EPI_ISL_841915 to 841916, EPI_ISL_841918 to 841920, EPI_ISL_841937, EPI_ISL_841939, EPI_ISL_841953, EPI_ISL_841965, EPI_ISL_841967, EPI_ISL_841969, EPI_ISL_841973, EPI_ISL_841996, EPI_ISL_842002 to 842003, EPI_ISL_842008, EPI_ISL_842010, EPI_ISL_842012, EPI_ISL_842014 to 842015, EPI_ISL_842018, EPI_ISL_842021, EPI_ISL_842023, EPI_ISL_842029 to 842030, EPI_ISL_842041, EPI_ISL_842046, EPI_ISL_842060, EPI_ISL_842075, EPI_ISL_867983, EPI_ISL_867985, EPI_ISL_867989 to 867990, EPI_ISL_867996 to 867998, EPI_ISL_868000, EPI_ISL_868008 to 868009, EPI_ISL_868012, EPI_ISL_868020, EPI_ISL_868026 to 868027, EPI_ISL_868029 to 868031, EPI_ISL_868034 to 868036, EPI_ISL_868039, EPI_ISL_868042, EPI_ISL_868051, EPI_ISL_868053 to 868054, EPI_ISL_868057 to 868058, EPI_ISL_868060, EPI_ISL_868063 to 868064, EPI_ISL_868067, EPI_ISL_868072 to 868074, EPI_ISL_868076, EPI_ISL_868078 to 868080, EPI_ISL_868082 to 868087, EPI_ISL_868089 to 868090, EPI_ISL_868093 to 868100, EPI_ISL_868102 to 868104, EPI_ISL_868107 to 868111, EPI_ISL_868113 to 868115, EPI_ISL_868118 to 868119, EPI_ISL_868125 to 868127, EPI_ISL_868130 to 868131, EPI_ISL_868135, EPI_ISL_868155, EPI_ISL_868160, EPI_ISL_868179, EPI_ISL_868183, EPI_ISL_868197 to 868198, EPI_ISL_868201, EPI_ISL_868208, EPI_ISL_868234 to 868237, EPI_ISL_868242 to 868245, EPI_ISL_868249 to 868250, EPI_ISL_868264, EPI_ISL_868266, EPI_ISL_868280, EPI_ISL_868286, EPI_ISL_868295, EPI_ISL_868298 to 868299, EPI_ISL_868304, EPI_ISL_868307 to 868308, EPI_ISL_868310 to 868311, EPI_ISL_868313 to 868314, EPI_ISL_868316 to 868321, EPI_ISL_868323 to 868325, EPI_ISL_868327, EPI_ISL_868329, EPI_ISL_868331, EPI_ISL_868333 to 868337, EPI_ISL_868339 to 868343, EPI_ISL_868346 to 868349, EPI_ISL_868351, EPI_ISL_868355, EPI_ISL_923230, EPI_ISL_923232, EPI_ISL_923242, EPI_ISL_923247, EPI_ISL_923257, EPI_ISL_923280, EPI_ISL_923293 to 923294, EPI_ISL_923303, EPI_ISL_923306, EPI_ISL_923309, EPI_ISL_923312, EPI_ISL_923314, EPI_ISL_923317, EPI_ISL_923320, EPI_ISL_923323, EPI_ISL_923325, EPI_ISL_923331 to 923332, EPI_ISL_923335 to 923337, EPI_ISL_923339, EPI_ISL_923341, EPI_ISL_923344, EPI_ISL_923346, EPI_ISL_923348, EPI_ISL_923354 to 923355, EPI_ISL_923360 to 923361, EPI_ISL_923363 to 923366, EPI_ISL_923369, EPI_ISL_923371 to 923372, EPI_ISL_923376, EPI_ISL_923398, EPI_ISL_923402 to 923404, EPI_ISL_923409 to 923410, EPI_ISL_923413, EPI_ISL_923422 to 923423, EPI_ISL_923425 to 923427, EPI_ISL_923429, EPI_ISL_923432, EPI_ISL_923436, EPI_ISL_923442, EPI_ISL_923446, EPI_ISL_923448 to 923449, EPI_ISL_923452 to 923453, EPI_ISL_923457, EPI_ISL_923564, EPI_ISL_923570 to 923574, EPI_ISL_923576, EPI_ISL_923578, EPI_ISL_923586, EPI_ISL_923589 to 923591, EPI_ISL_923593 to 923598, EPI_ISL_923607, EPI_ISL_923609, EPI_ISL_923624, EPI_ISL_923630 to 923632, EPI_ISL_923634 to 923637, EPI_ISL_923640, EPI_ISL_923643 to 923644, EPI_ISL_923647 to 923649, EPI_ISL_923651 to 923653, EPI_ISL_923655, EPI_ISL_923658, EPI_ISL_923664, EPI_ISL_923666, EPI_ISL_923668 to 923670, EPI_ISL_923672, EPI_ISL_923677 to 923678, EPI_ISL_923681, EPI_ISL_923683, EPI_ISL_952397, EPI_ISL_952405, EPI_ISL_952410, EPI_ISL_952412, EPI_ISL_952414 to 952415, EPI_ISL_952426, EPI_ISL_952429, EPI_ISL_952433, EPI_ISL_952437 to 952438, EPI_ISL_952440, EPI_ISL_952444, EPI_ISL_952448, EPI_ISL_952450, EPI_ISL_952455, EPI_ISL_952460, EPI_ISL_952464 to 952465, EPI_ISL_952467 to 952468, EPI_ISL_952470 to 952473, EPI_ISL_952475 to 952477, EPI_ISL_952480, EPI_ISL_952482, EPI_ISL_952484, EPI_ISL_952488, EPI_ISL_952490, EPI_ISL_952492, EPI_ISL_952494, EPI_ISL_952496 to 952500, EPI_ISL_952502, EPI_ISL_952505 to 952506, EPI_ISL_952508 to 952512, EPI_ISL_952514, EPI_ISL_952516 to 952519, EPI_ISL_952522, EPI_ISL_952524 to 952526, EPI_ISL_952531 to 952532, EPI_ISL_952535 to 952537, EPI_ISL_952539 to 952541, EPI_ISL_952543 to 952544, EPI_ISL_952546 to 952547, EPI_ISL_952550, EPI_ISL_952553, EPI_ISL_952555, EPI_ISL_952558 to 952559, EPI_ISL_952563, EPI_ISL_952565 to 952567, EPI_ISL_952572 to 952573, EPI_ISL_952576, EPI_ISL_952578, EPI_ISL_952580, EPI_ISL_952583, EPI_ISL_952585, EPI_ISL_952587, EPI_ISL_952589, EPI_ISL_952592, EPI_ISL_952595, EPI_ISL_952598 to 952604, EPI_ISL_952607 to 952610, EPI_ISL_952612 to 952618, EPI_ISL_952621 to 952623, EPI_ISL_952630, EPI_ISL_952637 to 952640, EPI_ISL_952644, EPI_ISL_952646, EPI_ISL_952648 to 952649, EPI_ISL_952653 to 952656, EPI_ISL_952658, EPI_ISL_952670, EPI_ISL_952672, EPI_ISL_952676 to 952678, EPI_ISL_952681 to 952684, EPI_ISL_952687, EPI_ISL_952690, EPI_ISL_952692 to 952694, EPI_ISL_952697 to 952699, EPI_ISL_952705 to 952706, EPI_ISL_952712 to 952714, EPI_ISL_952716, EPI_ISL_952718, EPI_ISL_952724, EPI_ISL_952730, EPI_ISL_952733, EPI_ISL_952738, EPI_ISL_952740 to 952741, EPI_ISL_952745 to 952746, EPI_ISL_952768, EPI_ISL_952775, EPI_ISL_952779 to 952780, EPI_ISL_952782 to 952785, EPI_ISL_952800, EPI_ISL_952802 to 952803, EPI_ISL_952806 to 952808, EPI_ISL_952811 to 952813, EPI_ISL_952818 to 952823, EPI_ISL_952826 to 952827, EPI_ISL_952833 to 952836, EPI_ISL_952838, EPI_ISL_952842, EPI_ISL_952846, EPI_ISL_952851 to 952852, EPI_ISL_952854 to 952855, EPI_ISL_952857, EPI_ISL_1000189 to 1000191, EPI_ISL_1000193, EPI_ISL_1000195, EPI_ISL_1000199 to 1000201, EPI_ISL_1000205, EPI_ISL_1000208, EPI_ISL_1000212, EPI_ISL_1000216, EPI_ISL_1000223, EPI_ISL_1000226, EPI_ISL_1000228, EPI_ISL_1000234, EPI_ISL_1000239, EPI_ISL_1000247, EPI_ISL_1000251, EPI_ISL_1000254, EPI_ISL_1000256, EPI_ISL_1000258 to 1000259, EPI_ISL_1000261, EPI_ISL_1000268 to 1000269, EPI_ISL_1000272 to 1000273, EPI_ISL_1000276, EPI_ISL_1000281, EPI_ISL_1000283 to 1000288, EPI_ISL_1000305, EPI_ISL_1000312 to 1000313, EPI_ISL_1000319, EPI_ISL_1000321, EPI_ISL_1000325, EPI_ISL_1000328 to 1000330, EPI_ISL_1000332, EPI_ISL_1000335, EPI_ISL_1000337 to 1000338, EPI_ISL_1000341 to 1000342, EPI_ISL_1000345 to 1000351, EPI_ISL_1000353 to 1000357, EPI_ISL_1000361 to 1000365, EPI_ISL_1000368, EPI_ISL_1000372, EPI_ISL_1000375 to 1000378, EPI_ISL_1000380 to 1000382, EPI_ISL_1000387 to 1000389, EPI_ISL_1000391, EPI_ISL_1000394 to 1000395, EPI_ISL_1000401 to 1000402, EPI_ISL_1000414, EPI_ISL_1000427, EPI_ISL_1000430, EPI_ISL_1000449 to 1000450, EPI_ISL_1000455, EPI_ISL_1000467, EPI_ISL_1000481 to 1000482, EPI_ISL_1000484, EPI_ISL_1000489, EPI_ISL_1000493 to 1000497, EPI_ISL_1000500 to 1000503, EPI_ISL_1000505 to 1000508, EPI_ISL_1000510, EPI_ISL_1000514 to 1000515, EPI_ISL_1000517 to 1000518, EPI_ISL_1000520, EPI_ISL_1000524, EPI_ISL_1000526, EPI_ISL_1000528 to 1000529, EPI_ISL_1000531 to 1000532, EPI_ISL_1000535 to 1000536, EPI_ISL_1000538, EPI_ISL_1000542 to 1000543, EPI_ISL_1000545 to 1000548, EPI_ISL_1000550 to 1000551, EPI_ISL_1000553, EPI_ISL_1000556 to 1000557, EPI_ISL_1000563 to 1000564, EPI_ISL_1000568, EPI_ISL_1000600, EPI_ISL_1000603 to 1000604, EPI_ISL_1000607 to 1000608, EPI_ISL_1000612, EPI_ISL_1000614 to 1000615, EPI_ISL_1000620 to 1000625, EPI_ISL_1000627			
see above	Centre for Enzyme Innovation, University of Portsmouth / Translational Research Laboratory, Portsmouth Hospitals NHS Trust	COVID-19 Genomics UK (COG-UK) Consortium	; Allyson Lloyd; Angela Beckett; Anoop Chauhan; Christopher Fearn; Ethan Butcher; Garry Scarlett; Kate Cook; Katie Loveson; Kelly Bicknell; Robert Impey; Salman Goudarzi; Samuel Robson; Sarah Wyllie; Scott Elliott; Sharon Glaysher; Yann Bourgeois
EPI_ISL_733568	Centre for Health Protection	Hong Kong Department of Health	Alan K.L. Tsang; Dominic N.C. Tsang; Edman T.K. Lam; Peter C.W. Yip; Rickjason C.W. Chan
EPI_ISL_654794	Centre for Human Virology & Genomics, Nigerian Institute of Medical Research	Centre for Human Virology & Genomics, Nigerian Institute of Medical Research	Shaibu, J.
EPI_ISL_413596, EPI_ISL_413598 to 413600	Centre for Infectious Diseases and Microbiology - Public Health	NSW Health Pathology - Institute of Clinical Pathology and Medical Research; Westmead Hospital; University of Sydney	Carter I; Chen SC; Eden J-S; Gall; Gray K; Holmes EC; Kok J and Dwyer DE for the 2019-nCoV Study Group*; Lam C; M; Maddocks S; O'Sullivan MV; Rahman H; Rockett R; Sintchenko V; Timms; V
EPI_ISL_407893, EPI_ISL_412975, EPI_ISL_413213 to 413214, EPI_ISL_413594 to 413595, EPI_ISL_417030	Centre for Infectious Diseases and Microbiology Laboratory Services	NSW Health Pathology - Institute of Clinical Pathology and Medical Research; Westmead Hospital; University of Sydney	; A; Alicia; Carter I; Chen SC; Eden J-S; Gall; Gray K; Holmes EC; Kok J and Dwyer DE for the 2019-nCoV Study Group; Kok J and Dwyer DE for the 2019-nCoV Study Group*; Lam C; M; Maddocks S; O'Sullivan MV; Rahman H; Rockett R; Sintchenko V; Timms; V
EPI_ISL_417383 to 417412, EPI_ISL_427643 to 427710, EPI_ISL_427712 to 427716, EPI_ISL_427720, EPI_ISL_427722 to 427808	Centre for Infectious Diseases and Microbiology Public Health	NSW Health Pathology - Institute of Clinical Pathology and Medical Research; Westmead Hospital; University of Sydney	Arnott A; Arnott A and Sadsad R for the 2019-nCoV Study Group; Bachmann N; Basile K; Byun R; Carter I; Carter I and Rahman H for the 2019-nCoV Study Group; Chang S; Chen SC; Chen SC and Maddocks S for the 2019-nCoV Study Group; Draper J; Dwyer DE; Dwyer DE and Rockett R for the 2019-nCoV Study Group; Dwyer DE for the 2019-nCoV Study Group; Eden J-S; Eden J-S and Lam C for the 2019-nCoV Study Group; Eden JS; Gall M;

			Gall M and Arnott A for the 2019-nCoV Study Group; Gray K; Gray K and Timms V for the 2019-nCoV Study Group; Holmes EC; Holmes EC and O'Sullivan MV for the 2019-nCoV Study Group; Kok J; Kok J and Dwyer DE for the 2019-nCoV Study Group; Lam C; Lam C and Gray K for the 2019-nCoV Study Group; Maddocks S; Maddocks S and Kok J for the 2019-nCoV Study Group; O'Sullivan MV; O'Sullivan MV and Sintchenko V for the 2019-nCoV Study Group; Propenko M; Rahman H; Rahman H and Holmes EC for the 2019-nCoV Study Group; Rockett R; Rockett R and Eden J-S for the 2019-nCoV Study Group; Sadsad R; Sadsad R and Carter I for the 2019-nCoV Study Group; Sim E; Sintchenko V; Sintchenko V and Chen SC for the 2019-nCoV Study Group; Sorrell T; Timms; Timms V; Timms V and Gall M for the 2019-nCoV Study Group; V
EPI_ISL_413597	Centre for Infectious Diseases and Microbiology- Public Health	NSW Health Pathology - Institute of Clinical Pathology and Medical Research; Westmead Hospital; University of Sydney	Carter I; Chen SC; Eden J-S; Gall; Gray K; Holmes EC; Kok J and Dwyer DE for the 2019-nCoV Study Group*; Lam C; M; Maddocks S; O'Sullivan MV; Rahman H; Rockett R; Sintchenko V; Timms; V
EPI_ISL_450302 to 450303, EPI_ISL_465688 to 465690, EPI_ISL_535980 to 535981, EPI_ISL_536000, EPI_ISL_536004, EPI_ISL_536023, EPI_ISL_536045, EPI_ISL_536080, EPI_ISL_536104, EPI_ISL_536141 to 536142, EPI_ISL_536145, EPI_ISL_536156, EPI_ISL_536159, EPI_ISL_536162, EPI_ISL_536182 to 536183, EPI_ISL_536212, EPI_ISL_536218 to 536220, EPI_ISL_536224, EPI_ISL_536232, EPI_ISL_536240 to 536241, EPI_ISL_536283, EPI_ISL_536325, EPI_ISL_536348, EPI_ISL_536369 to 536370, EPI_ISL_536377 to 536378			
see above	Centre hospitalier Anna-Laberge	Laboratoire de santé publique du Québec	Guillaume Bourque; Ioannis Ragoussis; Jesse Shapiro; Mark Lathrop and Michel Roger; Mark Lathrop and Michel Roger on behalf of the CoVSeQ research group; Sandrine Moreira
EPI_ISL_900512	Centre hospitalier Emile Roux	CNR Virus des Infections Respiratoires - France SUD	Antonin Bal; Bruno Lina; Gregory Destras; Gwendolyne Burfin; Hadrien Règue; Laurence Josset; Martine Valette; Quentin Semanas
EPI_ISL_508934 to 508936, EPI_ISL_666683 to 666686, EPI_ISL_693391, EPI_ISL_700372 to 700390, EPI_ISL_700394 to 700399, EPI_ISL_700401 to 700410	Centre hospitalier Métropole Savoie	CNR Virus des Infections Respiratoires - France SUD	Alexandre Gaymard; Antonin Bal; Bruno Lina; Carine Dumollard; Carine Moustaud; Florence Morfin-Sherpa; Gregory Destras; Gwendolyne Burfin; Hadrien Règue; Jérôme Grosjean; Laurence Josset; Martine Valette; Maude Bouscambert-Duchamp; Quentin Semanas; Raphaelle Lamy; Solenne Brun
EPI_ISL_535746, EPI_ISL_535767 to 535768	Centre hospitalier de St-Mary	Laboratoire de santé publique du Québec	Guillaume Bourque; Ioannis Ragoussis; Jesse Shapiro; Mark Lathrop and Michel Roger; Sandrine Moreira
EPI_ISL_535807, EPI_ISL_535959, EPI_ISL_536115, EPI_ISL_536199	Centre hospitalier régional du Grand Portage	Laboratoire de santé publique du Québec	Guillaume Bourque; Ioannis Ragoussis; Jesse Shapiro; Mark Lathrop and Michel Roger; Mark Lathrop and Michel Roger on behalf of the CoVSeQ research group; Sandrine Moreira
EPI_ISL_639739	Centre of Nanotechnologies, INCD IMT-Bucuresti (National Institute for Research and Development in Microtechnologies - Bucharest)	Centre of Nanotechnologies, INCD IMT-Bucuresti (National Institute for Research and Development in Microtechnologies - Bucharest)	Gogianu; L. and Baisan, M.; Salceanu, A.
EPI_ISL_413647, EPI_ISL_417997 to 417999	Centro Hospital do Porto, E.P.E. - H. Geral de Santo Antonio	Instituto Nacional de Saude (INSA)	Guiomar et al; Helena Ramos; Inês Costa; Joana Isidro; Joana Mendonça; João Paulo Gomes; Luís Vieira; Pedro Pechirra; Raquel Guiomar; Vítor Borges
EPI_ISL_417986 to 417987, EPI_ISL_417989	Centro Hospitalar e Universitario de Sao Joao, Porto	Instituto Nacional de Saude (INSA)	Guiomar et al
EPI_ISL_413648	Centro Hospitalar e Universitário de Sao Joao, Porto	Instituto Nacional de Saude (INSA)	Inês Costa; Joana Isidro; Joana Mendonça; João Paulo Gomes; João Tiago Guimarães; Luís Vieira; Pedro Pechirra; Raquel Guiomar; Vítor Borges
EPI_ISL_515547	Centro Medico da Policia Militar do Estado de Sao Paulo	Instituto Adolfo Lutz, Interdiciplinary Procedures Center, Strategic Laboratory	Claudia Regina Gonçalves; Claudio Tavares Sacchi; Erica Valessa Ramos Gomes
EPI_ISL_693248	Centro Municipal de Epidemiologia e Imunizações	Instituto Adolfo Lutz, Interdiciplinary Procedures Center, Strategic Laboratory	Claudia Regina Gonçalves; Claudio Tavares Sacchi; Erica Valessa Ramos Gomes; Karoline Rodrigues Campos
EPI_ISL_693244	Centro Médico da Polícia Militar do Estado de Sao Paulo	Instituto Adolfo Lutz, Interdiciplinary Procedures Center, Strategic Laboratory	Claudia Regina Gonçalves; Claudio Tavares Sacchi; Erica Valessa Ramos Gomes; Karoline Rodrigues Campos
EPI_ISL_512670, EPI_ISL_527742, EPI_ISL_527755	Centro Nacional De Rehabilitacion Humberto Araya Rojas (Cenare)	Inciensa, Instituto Costarricense de Investigación y Enseñanza en Nutrición y Salud	Adriana Godinez & Melany Calderon; Claudio Soto-Garita; Estela Cordero; Francisco Duarte; Hebleen Porras
EPI_ISL_629011 to 629028	Centro de Biotecnología Vegetal, Universidad Andrés Bello, Center for Genome Regulation	Center for Mathematical Modeling and Center for Genome Regulation. Santiago, Chile	Allende ML; Arriagada G; Bastias M; Bustos F; Castro E; González M; M; Maass A; Meneses C.; Montecino; Orellana A; Sanhueza D; Travisany D
EPI_ISL_483065, EPI_ISL_509430 to 509435, EPI_ISL_510536, EPI_ISL_529139	Centro de Desenvolvimento Tecnologico em Saude, Fundacao Oswaldo Cruz	Centro de Desenvolvimento Tecnologico em Saude, Fundacao Oswaldo Cruz	A.D.; Barroso; C.Q.; C.Q. and Medeiros; De Paula; F.B.; Ferreira; Fintelman-Rodrigues, N.; Gregorio; J.S.; M.A.; M.L.; Medeiros; Oliveira; S.P.; Sacramento; Saraiva; Souza; T.M.; Tschoeke, D.
EPI_ISL_510541, EPI_ISL_529140	Centro de Desenvolvimento Tecnologico em Saude, Fundacao Oswaldo Cruz	Centro de Desenvolvimento Tecnologico em Saude, Fundacao Oswaldo Cruz	A.D.; C.Q.; C.Q. and Medeiros; De Paula; F.B.; Ferreira; Fintelman-Rodrigues, N.; M.A.; Medeiros; Sacramento; Saraiva; Souza; T.M.
EPI_ISL_635480 to 635481, EPI_ISL_635483 to 635484, EPI_ISL_635486, EPI_ISL_635489, EPI_ISL_635491 to 635502, EPI_ISL_635504 to 635508, EPI_ISL_635510 to 635513, EPI_ISL_635515 to 635530, EPI_ISL_635532 to 635576			
see above	Centro de Diagnstico COVID-19 UABC Tijuana	Andersen lab at Scripps Research	Germán Ibarra; Jonathan Vincent Baena; Jorge Luis Jiménez Niebla; Manuel Sánchez Alavez; Oscar Efrén Zazueta Fierro; SEARCH Alliance San Diego with Idanya Rubí Serafín Higuera
EPI_ISL_491933 to 491935, EPI_ISL_491941, EPI_ISL_527818 to 527819, EPI_ISL_594118	Centro de Investigaciones, Universidad de Especialidades Espíritu Santo	Institute of Microbiology, Universidad San Francisco de Quito	Belén Prado-Vivar; Bernardo Gutiérrez; Derly Andrade; Edith Lopez; Fernando Espinoza; Gabriel Morey; Gabriel Trueba; Jose Pedro Barberan; Juan Carlos Fernandez; Juan José Guadalupe; Michelle Grunauer; Monica Becerra-Wong; Patricio Rojas-Silva; Paúl Cárdenas; Ruben Armas; Sully Márquez; Verónica Barragán
EPI_ISL_468761, EPI_ISL_468763 to 468764, EPI_ISL_500369, EPI_ISL_500372, EPI_ISL_500375 to 500376, EPI_ISL_500379, EPI_ISL_500386, EPI_ISL_500391, EPI_ISL_500396, EPI_ISL_500398 to 500402, EPI_ISL_500407 to 500413, EPI_ISL_500416 to 500418, EPI_ISL_500421, EPI_ISL_500423, EPI_ISL_500425, EPI_ISL_500427 to 500431, EPI_ISL_500433, EPI_ISL_500439, EPI_ISL_500445, EPI_ISL_500449 to 500451, EPI_ISL_500454 to 500456, EPI_ISL_500458, EPI_ISL_537382, EPI_ISL_537384 to 537385, EPI_ISL_537387, EPI_ISL_537390, EPI_ISL_537394 to 537395, EPI_ISL_537397 to 537399, EPI_ISL_537403 to 537404, EPI_ISL_537406, EPI_ISL_537410 to 537412, EPI_ISL_537414, EPI_ISL_537422, EPI_ISL_537424, EPI_ISL_537426 to 537429, EPI_ISL_537431 to 537432, EPI_ISL_537434, EPI_ISL_537436 to 537440, EPI_ISL_537443, EPI_ISL_537445 to 537447, EPI_ISL_537449, EPI_ISL_537451, EPI_ISL_537453, EPI_ISL_537460, EPI_ISL_537465 to 537466, EPI_ISL_537811, EPI_ISL_537822, EPI_ISL_537828 to 537829, EPI_ISL_537831, EPI_ISL_537836 to 537842, EPI_ISL_537844 to 537845, EPI_ISL_537847, EPI_ISL_537849 to 537850, EPI_ISL_537852 to 537853, EPI_ISL_537855 to 537859, EPI_ISL_537863 to 537866, EPI_ISL_537870, EPI_ISL_537872 to 537873, EPI_ISL_732749 to 732759, EPI_ISL_732762 to 732770, EPI_ISL_732772 to 732774, EPI_ISL_732777 to 732780, EPI_ISL_732783, EPI_ISL_732785 to 732789, EPI_ISL_732792 to 732793, EPI_ISL_732795 to 732819			
see above	Centro de Investigación Biomédica de La Rioja - Hospital San Pedro Logroño	SeqCOVID-SPAIN consortium/IBV(CSIC)	José Manuel Azcona Gutiérrez; María Pilar Bea Escudero; María de Toro; Miriam Blasco Alberdi and SeqCOVID-SPAIN consortium
EPI_ISL_583491	Centro de Saude Esf IV Zona Rual Domingos de SJ Rio Pardo	Instituto Adolfo Lutz, Interdiciplinary Procedures Center, Strategic Laboratory	Claudia Regina Gonçalves; Claudio Tavares Sacchi; Erica Valessa Ramos Gomes; Karoline Rodrigues Campos
EPI_ISL_471543, EPI_ISL_583500	Centro de Saude I Tacito Leite de Carvalho e Silva	Instituto Adolfo Lutz, Interdiciplinary Procedures Center, Strategic Laboratory	Claudia Regina Gonçalves; Claudio Tavares Sacchi; Erica Valessa Ramos Gomes; Karoline Rodrigues Campos
EPI_ISL_735416	Centro de Saude II Dr Jose Paione Mococa	Instituto Adolfo Lutz, Interdiciplinary Procedures Center, Strategic Laboratory	Claudia Regina Gonçalves; Claudio Tavares Sacchi; Erica Valessa Ramos Gomes; Karoline Rodrigues Campos
EPI_ISL_468305, EPI_ISL_468307, EPI_ISL_735411, EPI_ISL_735423 to 735424, EPI_ISL_735426	Centro de Vigilancia a Saude de Diadema	Instituto Adolfo Lutz, Interdiciplinary Procedures Center, Strategic Laboratory	Claudia Regina Gonçalves; Claudio Tavares Sacchi; Erica Valessa Ramos Gomes; Karoline Rodrigues Campos
EPI_ISL_693240, EPI_ISL_693242	Centro de Vigilância a Saude de Diadema	Instituto Adolfo Lutz, Interdiciplinary Procedures Center, Strategic Laboratory	Claudia Regina Gonçalves; Claudio Tavares Sacchi; Erica Valessa Ramos Gomes; Karoline Rodrigues Campos
EPI_ISL_450520 to 450524, EPI_ISL_639632 to 639633, EPI_ISL_639636, EPI_ISL_639640, EPI_ISL_639642, EPI_ISL_639648, EPI_ISL_639650 to 639651, EPI_ISL_639654 to 639657, EPI_ISL_639663, EPI_ISL_639672, EPI_ISL_639676 to 639680, EPI_ISL_639683, EPI_ISL_770038 to 770039, EPI_ISL_770042, EPI_ISL_770045, EPI_ISL_770048 to 770049, EPI_ISL_770052, EPI_ISL_770054, EPI_ISL_770056, EPI_ISL_770058 to 770059, EPI_ISL_770061			
see above	Centrl Laboratorija	Latvian Biomedical Research and Study Centre	Ivars Silamielis; Jana Oste; Jnis Kloviš; Jnis Pjalkovskis; Kaspars Megnis; Marta Priedte; Monta Ustinova; Stella Lapia; Uga Dumpis; Vita Rovte; ikita Zrelovs
EPI_ISL_486390 to 486391, EPI_ISL_486411 to 486416, EPI_ISL_486418 to 486421, EPI_ISL_486437, EPI_ISL_492988 to 492992, EPI_ISL_501286 to 501289, EPI_ISL_501808, EPI_ISL_501817, EPI_ISL_501895 to 501896, EPI_ISL_501915, EPI_ISL_501922, EPI_ISL_515196, EPI_ISL_534200 to 534202, EPI_ISL_534206 to 534208, EPI_ISL_534210 to 534211, EPI_ISL_534220, EPI_ISL_534222 to 534223			
see above	Centrl laboratorija	Latvian Biomedical Research and Study Centre	Ivars Silamielis; Jana Oste; Jnis Kloviš; Jnis Pjalkovskis; Kaspars Megnis; Marta Priedte; Monta Ustinova; Stella Lapia; Uga Dumpis; Vita Rovte; ikita Zrelovs

EPI_ISL_861714	Cerba	CERBA LAB	Costa JM; Haïm-Boukobza S.; Hedbaut E; Lecorche E; Malek Ramdane; Olivi M; Roquebert B; Trombert S; Verdurme L
EPI_ISL_1001461	Cerballiance	Cerba	Haim-Boukobza S; Lecorche E; Meyer S; Roig JC; Roquebert B; Trombert-Paolantoni S
EPI_ISL_861503	Cerballiance Alpes Durance	CERBA LAB	Dyen P; Prots L.
EPI_ISL_1005688	Cerballiance Antony Velpeau	Cerba	Benazra M; Collin T; Delaplace E; Gresset N; Haim-Boukobza S; Lecorche E; Lesenne A; Olivi M; Ricard B; Roquebert B; Trombert-Paolantoni S
EPI_ISL_1005686	Cerballiance Bligny	Cerba	Benazra M; Collin T; Delaplace E; Gresset N; Haim-Boukobza S; Lecorche E; Lesenne A; Olivi M; Ricard B; Roquebert B; Trombert-Paolantoni S
EPI_ISL_1009667	Cerballiance Bourg-la-Reine	Cerba	Benazra M; Collin T; Delaplace E; Gresset N; Haim-Boukobza S; Lecorche E; Lesenne A; Olivi M; Ricard B; Roquebert B; Trombert-Paolantoni S
EPI_ISL_1001465, EPI_ISL_1001467	Cerballiance Carpentras CV	Cerba	Haim-Boukobza S; Lecorche E; Meyer S; Roig JC; Roquebert B; Trombert-Paolantoni S
EPI_ISL_861497, EPI_ISL_861500 to 861502	Cerballiance Centre Est	CERBA LAB	Chanard C; Chanard E; Gazzano V; Munier C; Prots L.
EPI_ISL_1009618	Cerballiance Cerrieres-le-Buisson	Cerba	Benazra M; Collin T; Delaplace E; Gresset N; Haim-Boukobza S; Lecorche E; Lesenne A; Olivi M; Ricard B; Roquebert B; Trombert-Paolantoni S
EPI_ISL_860716, EPI_ISL_861488 to 861495	Cerballiance Côte d'Azur	CERBA LAB	; Aankour N; Adjtoutah Z; Adoul O; Barrieu-Moussat S; Barrieu-Moussat S.; Barsanti M; Boudjahem Z; Chaudet L; Chobert F; De La Chapelle A; Francois J; Gandillet A; Gastaud S; Haïm-Boukobza S.; Jaugin P; Maltese S; Mangiarotti T; Marcadal M; Marius M; Martin L; Morello V; Pichon R; Pichon R. Quintle S; Prots L.; Prots L. Raimondi V.; Prots.L; Roman E; Roquebert B; Sanza O; Sinsoulieu T; Van Der Rhyn I; Viltard B
EPI_ISL_1003769	Cerballiance Endoume	Cerba	Haim-Boukobza S; Lecorche E; Meyer S; Roig JC; Roquebert B; Trombert-Paolantoni S
EPI_ISL_1011752	Cerballiance Grigny	Cerba	Benazra M; Collin T; Delaplace E; Gresset N; Haim-Boukobza S; Lecorche E; Lesenne A; Olivi M; Ricard B; Roquebert B; Trombert-Paolantoni S
EPI_ISL_861508	Cerballiance Haut de France	CERBA LAB	Felloni C; Lahmidi S.; Mainardi A
EPI_ISL_1005687	Cerballiance Lardy	Cerba	Benazra M; Collin T; Delaplace E; Gresset N; Haim-Boukobza S; Lecorche E; Lesenne A; Olivi M; Ricard B; Roquebert B; Trombert-Paolantoni S
EPI_ISL_1001573	Cerballiance Luynes	Cerba	Haim-Boukobza S; Lecorche E; Meyer S; Roig JC; Roquebert B; Trombert-Paolantoni S
EPI_ISL_871789	Cerballiance Normandie	CERBA LAB	BRIAND G; COUDRAY J; COULON C; HAMEL A.; LAFOREST D; LEPIGEON; MARTINS-AMARAL F; THOMAS Johan; VITEL R; technicien V
EPI_ISL_1004174	Cerballiance Port de Bouc	Cerba	Haim-Boukobza S; Lecorche E; Meyer S; Roig JC; Roquebert B; Trombert-Paolantoni S
EPI_ISL_1001463	Cerballiance Port de bouc	Cerba	Haim-Boukobza S; Lecorche E; Meyer S; Roig JC; Roquebert B; Trombert-Paolantoni S
EPI_ISL_861504 to 861506	Cerballiance Provence	CERBA LAB	Delaunay E; Prots L.; Roig JC
EPI_ISL_1000968, EPI_ISL_1001462	Cerballiance Provence	Cerba	Haim-Boukobza S; Lecorche E; Meyer S; Roig JC; Roquebert B; Trombert-Paolantoni S
EPI_ISL_862046 to 862047	Cerballiance Val de Loire	CERBA LAB	Abs G.
EPI_ISL_802994 to 802995	Charite Universitatsmedizin Berlin, Institut fur Virologie, Charitéplatz 1, 10117 Berlin, Germany	Charite Universitatsmedizin Berlin, Institut fur Virologie, Charitéplatz 1, 10117 Berlin, Germany	Barbara Mühlemann; Christian Drosten; Julia Schneider; Julia Tesch; Jörn Beheim-Schwarzbach; Talitha Veith; Terry Jones; Tobias Bleicker; Victor M Corman
EPI_ISL_516629 to 516642, EPI_ISL_516644 to 516645	Charite Universitatsmedizin Berlin, Institut fur Virologie/Labor Berlin	Charite Universitatsmedizin Berlin, Institut fur Virologie/Labor Berlin	Barbara Muhlemann; Christian Drosten; Julia Schneider; Jörn Beheim-Schwarzbach; Talitha Veith; Terry Jones; Victor M Corman
EPI_ISL_862139 to 862142, EPI_ISL_862149	Charité Universitätsmedizin Berlin, Institut fur Virologie/Labor Berlin	Charité Universitätsmedizin Berlin, Institut für Virologie	Barbara Mühlemann; Christian Drosten; Julia Schneider; Julia Tesch; Jörn Beheim-Schwarzbach; Talitha Veith; Terry Jones; Tobias Bleicker; Victor M Corman
EPI_ISL_754175	Charité Universitätsmedizin Berlin, Institut für Virologie	Charité Universitätsmedizin Berlin, Institut für Virologie	Barbara Mühlemann; Christian Drosten; Julia Schneider; Jörn Beheim-Schwarzbach; Talitha Veith; Terry Jones; Victor M Corman
EPI_ISL_729415, EPI_ISL_729473 to 729497, EPI_ISL_729499, EPI_ISL_729514, EPI_ISL_729529 to 729534, EPI_ISL_729548, EPI_ISL_729553 to 729561, EPI_ISL_729596 to 729607, EPI_ISL_753775, EPI_ISL_753777, EPI_ISL_753781 to 753809, EPI_ISL_753811 to 754058, EPI_ISL_754186 to 754194, EPI_ISL_853402 to 853403, EPI_ISL_869085, EPI_ISL_909747, EPI_ISL_909750 to 909752, EPI_ISL_912246, EPI_ISL_912249, EPI_ISL_912260, EPI_ISL_912262 to 912263, EPI_ISL_912266			
see above	Charité Universitätsmedizin Berlin, Institut für Virologie/Labor Berlin	Charité Universitätsmedizin Berlin, Institut für Virologie	Barbara Mühlemann; Christian Drosten; Julia Schneider; Julia Tesch; Jörn Beheim-Schwarzbach; Talitha Veith; Terry Jones; Tobias Bleicker; Victor M Corman
EPI_ISL_856678, EPI_ISL_856680 to 856681, EPI_ISL_860788, EPI_ISL_875344	Charité Universitätsmedizin Berlin, Institute of Virology, Charitéplatz 1, 10117 Berlin, Germany	Charité Universitätsmedizin Berlin, Institute of Virology, Charitéplatz 1, 10117 Berlin, Germany	Barbara Mühlemann; Christian Drosten; Julia Schneider; Julia Tesch; Jörn Beheim-Schwarzbach; Talitha Veith; Terry Jones; Tobias Bleicker; Victor M Corman
EPI_ISL_406862	Charité Universitätsmedizin Berlin, Institute of Virology; Institut für Mikrobiologie der Bundeswehr, Munich	Charité Universitätsmedizin Berlin, Institute of Virology	Barbara Mühlemann; Christian Drosten; Julia Schneider; Markus Antwerpen; Roman Wölfel; Talitha Veith; Victor M Corman
EPI_ISL_735496 to 735497	Chattogram Veterinary and Animal Sciences University	Central Biological Research Laboratory and Department of Biochemistry and Molecular Biology	H. M. Abdullah Al Masud; Imam Hossen; Md. Arif Hossain; Md. Imranul Hoq; Md. Khondakar Raziur Rahman; Md. Omer Faruq; Mohammad Omar Faruque; Robiul Hasan Bhuiyan; Sajib Rudra; Shanta Paul
EPI_ISL_569612 to 569613	Cheyenne River Health Center	South Dakota Public Health Laboratory	Jacob Garfin; Matt Plumb; Xiong Wang; and Chris Carlson
EPI_ISL_419297 to 419298, EPI_ISL_419309 to 419311, EPI_ISL_479809 to 479811, EPI_ISL_480004 to 480014, EPI_ISL_684159 to 684161, EPI_ISL_686659 to 686844, EPI_ISL_691284 to 691288			
see above	Chiba Prefectural Institute of Public Health	Pathogen Genomics Center, National Institute of Infectious Diseases	Hajime Kamiya; Kentaro Itokawa; Makoto Kuroda; Masakatsu Taira; Masanori Hashino; Motoi Suzuki; Rina Tanaka; Tsuyoshi Sekizuka; Yushi Hachisu
EPI_ISL_468070 to 468073, EPI_ISL_468075 to 468078, EPI_ISL_477125 to 477136, EPI_ISL_477138 to 477140, EPI_ISL_492029 to 492030, EPI_ISL_625460 to 625461, EPI_ISL_625465, EPI_ISL_625467, EPI_ISL_625471, EPI_ISL_625473 to 625477, EPI_ISL_700336, EPI_ISL_700339 to 700347, EPI_ISL_768729, EPI_ISL_768732 to 768733, EPI_ISL_768736, EPI_ISL_768741			
see above	Child Health Research Foundation	Child Health Research Foundation	Afroza Akter Tanni; Arif Mohammad Tanmoy; Hafizur Rahman; Maksuda Islam; Md Hafizur Rahman; Md Saiful Islam Sajib; Nikkon Sarkar; Roly Malaker; Samir K Saha; Senjuti Saha; Sharmistha Goswami; Syed Muktadir Al Sium
EPI_ISL_437912	Child Health Research Foundation	Child Health Research Lab	Maksuda Islam; Md Hafizur Rahman; Md Hasanuzzaman; Md Saiful Islam Sajib; Md Shahidul Islam; Roly Malaker; Samir K Saha; Senjuti Saha; Zabed B Ahmed
EPI_ISL_513343 to 513344	Children Westmead Hospital	NSW Health Pathology - Institute of Clinical Pathology and Medical Research; Westmead Hospital; University of Sydney	CIDM-PH et al.
EPI_ISL_507962	Children's Hospitals and Clinics of Minnesota	Minnesota Department of Health, Public Health Laboratory	Jacob Garfin; Matt Plumb; and Xiong Wang
EPI_ISL_451548, EPI_ISL_451594, EPI_ISL_451605, EPI_ISL_455068	Childrens Hospital Westmead	NSW Health Pathology - Institute of Clinical Pathology and Medical Research; Westmead Hospital; University of Sydney	CIDM-PH et al.
EPI_ISL_430722, EPI_ISL_430724 to 430725, EPI_ISL_430728 to 430731, EPI_ISL_430733 to 430738, EPI_ISL_430740 to 430744, EPI_ISL_430746	Chinese PLA Institute for Disease Control and Prevention	Chinese PLA Institute for Disease Control and Prevention	Lizhong Li; Peng LiJinhui Li
EPI_ISL_417330	Chiu Laboratory, University of California, San Francisco	Chiu Laboratory, University of California, San Francisco	Scot Federman; Wei Gu; Xianding Deng; and Charles Y. Chiu
EPI_ISL_443315	Château de la Source	National Reference Center for Viruses of Respiratory Infections, Institut Pasteur, Paris	Angela Brisebarre; Etienne Simon-Lorière; Flora Donati; Marion Barbet; Maud Vanpeene; Mélanie Albert; Méline Bizard; Sylvie Behillil; Sylvie van der Werf; Vincent Enouf
EPI_ISL_491115	Cicin-Sain Lab	Cicin-Sain Lab	Kathrin Eschke; Luka Cicin-Sain; M. Zeeshan Chaudhry; Yeonsu Kim
EPI_ISL_454646	City of El Paso Department of Public Health Laboratory	Pathogen Discovery, Respiratory Viruses Branch, Division of Viral Diseases, Centers for Disease Control and Prevention	Anna Uehara; Clinton R. Paden; Haibin Wang; Jing Zhang; Krista Queen; Michael Bowen; Suxiang Tong; Yan Li; Ying Tao; Zachary Weiner

EPI_ISL_547585 to 547586, EPI_ISL_547588 to 547593, EPI_ISL_560318, EPI_ISL_560322 to 560324, EPI_ISL_561339 to 561341, EPI_ISL_561343	Civil Hospital, Panchkula	CSIR-Institute of Microbial Technology	Amandeep Kaur; Anu Singh; Ashwani Kumar; Debarghya Ghose; Dipak Dutta; Harsh Goar; Kanika Bansal; Navin Baid; Poushali Chakraborty; Prabhu B. Patil; Rajesh Kumar Mishra; Sanjeet Kumar; Sanjeev Khosla
EPI_ISL_539483, EPI_ISL_539486 to 539487, EPI_ISL_561344	Civil Hospital, Rupnagar	CSIR-Institute of Microbial Technology	Amandeep Kaur; Anu Singh; Ashwani Kumar; Debarghya Ghose; Dipak Dutta; Harsh Goar; Kanika Bansal; Navin Baid; Poushali Chakraborty; Prabhu B. Patil; Rajesh Kumar Mishra; Sanjeet Kumar; Sanjeev Khosla
EPI_ISL_700432, EPI_ISL_700519	Clinic-in-Asla	NHLS/UCT	Arash Iranzadeh; Bruna Galvao; Carolyn Williamson; Deelan Doolabh; Diana Hardie; Houriiyah Tegally; Innocent Mudau; Kruger Marais; Lynn Tyers; Marvin Hsiao; Stephen Korsman
EPI_ISL_414579	Clinica Alemana de Santiago, Chile	Instituto de Salud Publica de Chile	Alejandra Acevedo; Andrés E. Castillo; Bárbara Parra; Carolina Tambley; Gabriel Leal; Gisselle Barra; Jaime Lagos; Javier Tognarelli; Jorge Fernández.; Loredana Arata; Patricia Bustos; Paz Tapia; Rodrigo Fasce; Soledad Ulloa; Winston Andrade
EPI_ISL_770004	Clinica Biblica	Inciensa, Instituto Costarricense de Investigación y Enseñanza en Nutrición y Salud	Adriana Godínez; Claudio Soto-Garita; Estela Cordero; Francisco Duarte; Hebleen Porras; Melany Calderón & Karla Gutiérrez-González
EPI_ISL_414580	Clinica Santa Maria, Santiago, Chile	Instituto de Salud Publica de Chile	Alejandra Acevedo; Andrés E. Castillo; Bárbara Parra; Carolina Tambley; Gabriel Leal; Gisselle Barra; Jaime Lagos; Javier Tognarelli; Jorge Fernández.; Loredana Arata; Patricia Bustos; Paz Tapia; Rodrigo Fasce; Soledad Ulloa; Winston Andrade
EPI_ISL_462450 to 462476	Clinical Center, University of Sarajevo	Charite Universitatsmedizin Berlin, Institute of Virology	Almedina Hadzihasanovic-Moro; Amela Dedeic-Ljubovic; Barbara Muehlemann; Christian Drosten; Irma Salimovic-Besic; Jorn Beheim-Schwarzbach; Julia Schneider; Selma Mutevelic; Suzana Arapcic; Talitha Veith; Terry Jones; Victor M Corman
EPI_ISL_421279, EPI_ISL_421281, EPI_ISL_437455 to 437458	Clinical Diagnostics Laboratory, Diagnostic & Experimental Pathology, Lilly Research Laboratories	Clinical Diagnostics Laboratory, Diagnostic & Experimental Pathology, Lilly Research Laboratories	Andrew Schade; Angie Fulford; Erin Wray; Jeff Fill; Joe Oakley; John Calley; John McElwee; Leslie ONeill Reising; Leslie O'Neill Reising; Mayuri Vaidya; Pat Finnegan; Phil Ebert; Rachael Redmond; Sam McNeely; Tim Holzer
EPI_ISL_677715, EPI_ISL_677718	Clinical Hospital - Bitola	Research Center for Genetic Engineering and Biotechnology "Georgi D. Efremov" , Macedonian Academy of Sciences and Arts	RCGEB - MASA
EPI_ISL_516426 to 516431, EPI_ISL_677676 to 677677, EPI_ISL_677716 to 677717, EPI_ISL_677720 to 677721, EPI_ISL_677724, EPI_ISL_678250, EPI_ISL_678259			
see above	Clinical Hospital - Shtip	Research Center for Genetic Engineering and Biotechnology "Georgi D. Efremov" , Macedonian Academy of Sciences and Arts	RCGEB - MASA
EPI_ISL_424352	Clinical Laboratory, Fuyang City Center for Disease Control and Prevention	Clinical Laboratory, Fuyang City Center for Disease Control and Prevention	Ge, B.
EPI_ISL_450506	Clinical Laboratory, Hospital Israelita Albert Einstein	Clinical Laboratory, Hospital Israelita Albert Einstein	Amgarten, D.; Araujo; C.L.P.; D.B.; D.B.L.; Durigon; E.L. and Pinho; J.R.R.; Machado; Malta, F.; Mangueira; R.A.F.; R.R.G.; Santana; de Oliveira
EPI_ISL_416432	Clinical Microbiology Lab	Infectious Disease Research Department, King Abdullah International Medical Research Center (KAIMRC)	Abdulrahman Alswaji; Liliane Okdah; Majed Alghoribi; Michel Doumith; Sadeem Alhayli; Sameera Al Johani
EPI_ISL_1001460	Clinical Microbiology Laboratory, NewYork Presbyterian Hospital/Columbia University Irving Medical Center	Uhlemann Laboratory, Columbia University Irving Medical Center	Anne-Catrin Uhlemann; David Ho; Hiroshi Mori; Medini K. Annavajhala
EPI_ISL_447419 to 447420, EPI_ISL_447422 to 447424, EPI_ISL_447426 to 447430, EPI_ISL_447432, EPI_ISL_447434 to 447436, EPI_ISL_447438, EPI_ISL_447440 to 447450, EPI_ISL_447452 to 447457, EPI_ISL_447460 to 447465, EPI_ISL_447467 to 447469			
see above	Clinical Microbiology Laboratory, Sheba Medical Center	Stern Lab	Stern Lab
EPI_ISL_447384 to 447389, EPI_ISL_447391, EPI_ISL_447393 to 447397, EPI_ISL_447399 to 447404, EPI_ISL_447406, EPI_ISL_447417 to 447418	Clinical Microbiology Laboratory, The Baruch Padeh Medical Center, Poriya	Stern Lab	Stern Lab
EPI_ISL_483570	Clinical Microbiology Laboratory- Basurto University Hospita	Biocruces-Bizkaia	Ana Belén Belén de la Hoz; Estibaliz Ugalde-Zarraga; José Luis Díaz de Tuesta del Arco; Matxalen Vidal-García; Mikel J. Urrutikoetxea-Gutierrez; Mº Carmen Nieto Toboso
EPI_ISL_483566, EPI_ISL_483571 to 483573, EPI_ISL_486876, EPI_ISL_489833 to 489835, EPI_ISL_490202 to 490204, EPI_ISL_490977	Clinical Microbiology Laboratory- Basurto University Hospital	Biocruces-Bizkaia	Ana Belén Belén de la Hoz; Estibaliz Ugalde-Zarraga; José Luis Díaz de Tuesta del Arco; Matxalen Vidal-García; Mikel J. Urrutikoetxea-Gutierrez; Mº Carmen Nieto Toboso
EPI_ISL_914003, EPI_ISL_914006 to 914008, EPI_ISL_955294 to 955297, EPI_ISL_955301, EPI_ISL_955304 to 955306	Clinical Pathology Lab	Pathogen Discovery, Respiratory Viruses Branch, Division of Viral Diseases, Centers for Disease Control and Prevention	Anna Uehara; Clinton R. Paden; Haibin Wang; Jing Zhang; Krista Queen; Peter Cook; Suxiang Tong; Yan Li; Ying Tao
EPI_ISL_778842	Clinical Pathology Labs	Centers for Disease Control and Prevention Division of Viral Diseases, Pathogen Discovery	Ying Tao Yan Li Jing Zhang Krista Queen Anna Uehara Peter Cook Clinton R. Paden Haibin Wang Suxiang Tong
EPI_ISL_605799 to 605816	Clinical Virology Laboratory, Institute of Liver and Biliary Sciences	ILBS - IGIB	Abhishek Padhi; Ekta Gupta; Jaswinder Singh Maras; Reshu Agarwal; Sheetalnath Rooge; Shridhar Sivasubbu; Shvetank Sharma; Vinod Scaria
EPI_ISL_447312 to 447317, EPI_ISL_447319 to 447321, EPI_ISL_447323 to 447324, EPI_ISL_447327 to 447328, EPI_ISL_447330	Clinical Virology Laboratory, Soroka Medical Center and the Faculty of Health Sciences, Ben-Gurion University of the Negev	Stern Lab	Stern Lab
EPI_ISL_447331 to 447332, EPI_ISL_447334, EPI_ISL_447337 to 447353, EPI_ISL_447355 to 447357, EPI_ISL_447359 to 447361, EPI_ISL_447364 to 447367, EPI_ISL_447369 to 447370, EPI_ISL_447372, EPI_ISL_447374 to 447375, EPI_ISL_447379 to 447382, EPI_ISL_447408 to 447412, EPI_ISL_447416			
see above	Clinical Virology Unit, Hadassah Hebrew University Medical Center	Stern Lab	Stern Lab
EPI_ISL_753262 to 753264, EPI_ISL_753266 to 753374, EPI_ISL_753376 to 753388, EPI_ISL_753390 to 753408, EPI_ISL_753410 to 753488, EPI_ISL_753490 to 753496, EPI_ISL_753498 to 753533, EPI_ISL_753535 to 753559, EPI_ISL_753561 to 753679, EPI_ISL_753681 to 753692			
see above	Clinical virology Laboratory, Children's Hospital Los Angeles	Center for Personalized Medicine, Children's Hospital Los Angeles	Gai et al
EPI_ISL_418230	Clinique AVERAY LA BROUSTE, Med. Polyvalente	National Reference Center for Viruses of Respiratory Infections, Institut Pasteur, Paris	Angela Brisebarre; Elsa Ngwem; Etienne Simon-Lorière; Flora Donati; Marion Barbet; Maud Vanpeene; Mélanie Albert; Méline Bizard; Sylvie Behillil; Sylvie van der Werf; Vincent Enouf
EPI_ISL_965393 to 965397, EPI_ISL_965402	Clinique N.D de Grâce Gosselies	GIGA Medical Genomics	Bouchra Boujemla; Cécile Meex; Keith Durkin; Maria Artesi; Marie-Pierre Hayette; Pierrette Melin; Raphaël Boreux; Sébastien Bontems; Vincent Bours
EPI_ISL_949247	Cliniques universitaires Saint-Luc	UCLouvain/IREC/MBLG	Benoit Kabamba Mukadi; Jean Ruelle; Lysa Pinsmaye
EPI_ISL_452473 to 452474, EPI_ISL_452476 to 452477, EPI_ISL_452479 to 452481, EPI_ISL_452483 to 452488, EPI_ISL_452490 to 452493, EPI_ISL_452495 to 452496, EPI_ISL_452498, EPI_ISL_452500, EPI_ISL_452503, EPI_ISL_452505 to 452510, EPI_ISL_452513 to 452514, EPI_ISL_452516 to 452521, EPI_ISL_452523 to 452524, EPI_ISL_452526 to 452528, EPI_ISL_452531 to 452532, EPI_ISL_452534 to 452535, EPI_ISL_452538 to 452541			
see above	Clínica Universidad de Navarra. Servicio de Enfermedades Infecciosas y Microbiología clínica	SeqCOVID-SPAIN consortium/IBV(CSIC)	Jose Luis del Pozo and SeqCOVID-SPAIN consortium; Mirian Fernández-Alonso
EPI_ISL_1015361	Coast Diagnostics	Synergy Laboratories	Megan Cornwell
EPI_ISL_677285 to 677319, EPI_ISL_677638 to 677639, EPI_ISL_677641 to 677645, EPI_ISL_677647 to 677648, EPI_ISL_677652 to 677654, EPI_ISL_710180 to 710244, EPI_ISL_710301 to 710373, EPI_ISL_857044, EPI_ISL_857046, EPI_ISL_954855, EPI_ISL_983721 to 983729, EPI_ISL_1038916			
see above	Colorado Department of Public Health and Environment	Colorado Department of Puplic Health and Environment	Diana Ir; Emily A. Travanty; Laura Bankers; Molly C. Hetherington-Rauth; Molly Hetherington-Rauth; Sarah Elizabeth Totten; Shannon Ely; Shannon R.

			Matzinger
EPI_ISL_765575 to 765578, EPI_ISL_765580, EPI_ISL_765582	Colorado Mesa University	Infectious Disease Program, Broad Institute of Harvard and MIT	A.E.; Adams, G.; Anahtar, M.; B.L.; B.W.; Bauer, M.; Birren; Branda, J.; Carter, A.; Cerrato, F.; Chaluvadi, S.; Chapman; Cusick, C.; D.J.; DeRuff, K.; E. and Sabeti; Flowers, K.; Gallagher, G.; Gladden-Young, A.; Gnirke, A.; Harris, J.; J.E.; K.J.; LaRocque, R.; Lagerborg, K.; Lemieux; Lin; Loreth, C.; MacInnis; Neumann, A.; Normandin, E.; P.C.; Park; Pierce, V.; Reilly, S.; Rosenberg; Rudy, M.; Ryan, E.; S.B.; Shaw, B.; Siddle; Slater, D.; Smole, S.; Tomkins-Tinch, C.; Turbett, S.
EPI_ISL_527401 to 527460, EPI_ISL_527462 to 527466, EPI_ISL_527468 to 527488	Colorado State University - Ebel Lab	Colorado State University - Ebel Lab	Greg Ebel et al.
EPI_ISL_804980	Columbia University Irving Medical Center	Wadsworth Center, New York State Department of Health	Alexis Russel; Daryl M. Lamson; Erasmus Schneider; Erica Lasek-Nesselquist; John Kelly; Jonathan Plitnick; Kirsten St. George; Matthew Shudt; Melissa A Leisner; Navjot Singh
EPI_ISL_539822 to 539823	Communicable Disease Branch	Hong Kong Department of Health	Alan K.L. Tsang; Dominic N.C. Tsang; Edman T.K. Lam; Peter C.W. Yip; Rickjason C.W. Chan
EPI_ISL_632250 to 632282, EPI_ISL_632284 to 632285, EPI_ISL_632899 to 632907, EPI_ISL_678261 to 678272, EPI_ISL_681298 to 681319, EPI_ISL_682299 to 682322, EPI_ISL_684028 to 684036			
see above	Communicable Disease Laboratory, Public Health Directorate	Communicable Disease Laboratory, Public Health Directorate	AlAbbas, Z.; AlHujairi, Z.; AlTaif, Z.; AlWasti, H.; Alhujairi, Z.; Altaif, Z.; Alwasti, H.
EPI_ISL_485401, EPI_ISL_510528	Communicable Disease Laboratory, Public Health Directorate	Communicable Disease Laboratory, Public Health Directorate	Al Wasti; Al-Taif; Al-Wasti, H.; H. and AlTaif, Z.; Z. and Shehab, F.; Zaed, A.
EPI_ISL_862544, EPI_ISL_862553 to 862555	Compejo Hospitalario Xeral-Calde	Instituto de Salud Carlos III	A. Monzón; F. Casas; I. Alonso, P.; I. Jiménez; Iglesias-Caballero; M. Pozo; M. Cuesta; M. González-Esguevillas; M. Zaballos; M.Camarero; P. Jiménez; S. Juliá; S. Molinero Calamita; S. Varona
EPI_ISL_913038	Complejo Asistencial Universitario de Burgos	Instituto de Salud Carlos III	A. Monzón; F. Casas; I. Jiménez; I. Megías, G.; Iglesias-Caballero; M. Camarero; P. Zaballos; S. Cuesta; S. Pozo; S. Sandonís; S. Varona; V. Vázquez
EPI_ISL_954785	Complejo Asistencial Universitario de Salamanca	Instituto de Salud Carlos III	A. Monzón; F. Casas; I. Jiménez; I. Ávila, A.; Iglesias-Caballero; M. Camarero; P. Zaballos; S. Cuesta; S. Pozo; S. Sandonís; S. Varona; V. Vázquez
EPI_ISL_455328 to 455331, EPI_ISL_578194	Complejo Hospitalario Universitario La Coruna	Instituto de Salud Carlos III	A. Monzón; F. Casas; I; I. Jiménez; Iglesias-Caballero; J. López; M. Camarero; M. Cuesta; M. González-Esguevillas; M. Molinero Calamita; M. Zaballos; M.A Canizares; P. Jiménez; S. Juliá; S. Pozo; S. Varona
EPI_ISL_954789, EPI_ISL_954794, EPI_ISL_954800	Complejo Hospitalario Universitario La Coruña	Instituto de Salud Carlos III	A. Monzón; F. Casas; I. Jiménez; I. Peña, F.; Iglesias-Caballero; M. Camarero; P. Zaballos; S. Cuesta; S. Pozo; S. Sandonís; S. Varona; V. Vázquez
EPI_ISL_691684	Complejo Hospitalario Universitario de Albacete	Instituto de Salud Carlos III	A. Monzón; F. Casas; I. Jiménez; I. Martínez, E.; Iglesias-Caballero; M. Camarero; M. Cuesta; M. González-Esguevillas; M. Pozo; M. Zaballos; P. Jiménez; S. Juliá; S. Molinero Calamita; S. Varona
EPI_ISL_474798, EPI_ISL_474801 to 474813, EPI_ISL_474815 to 474816, EPI_ISL_474818 to 474820, EPI_ISL_474822 to 474828, EPI_ISL_474830 to 474831, EPI_ISL_474837 to 474838, EPI_ISL_474847, EPI_ISL_474853, EPI_ISL_474901 to 474904, EPI_ISL_474919, EPI_ISL_474921, EPI_ISL_474933, EPI_ISL_474940, EPI_ISL_474946 to 474947, EPI_ISL_474951 to 474956			
see above	Complejo Hospitalario Universitario de Albacete	SeqCOVID-SPAIN consortium/IBV(CSIC)	Caridad Sainz de Baranda Camino and SeqCOVID-SPAIN consortium; Encarnacion Simarro Córdoba; Julia Lozano Serra; Lorena Robles Fonseca; Monica Parra Grandes
EPI_ISL_455333, EPI_ISL_913024	Complejo Hospitalario Universitario de Santiago	Instituto de Salud Carlos III	A. Monzón; F. Casas; I; I. Jiménez; I. Peña, F.; Iglesias-Caballero; J. Llovo; M. Camarero; M. Cuesta; M. González-Esguevillas; M. Molinero Calamita; M. Zaballos; P. Jiménez; P. Zaballos; S. Cuesta; S. Juliá; S. Pozo; S. Sandonís; S. Varona; V. Vázquez
EPI_ISL_537380 to 537381	Complejo Hospitalario Universitario de Vigo	SeqCOVID-SPAIN consortium/IBV(CSIC)	Benito Regueiro and SeqCOVID-SPAIN consortium
EPI_ISL_913039	Complejo Hospitalario Xeral-Calde	Instituto de Salud Carlos III	A. Monzón; F. Casas; I. Alonso, P.; I. Jiménez; Iglesias-Caballero; M. Camarero; P. Zaballos; S. Cuesta; S. Pozo; S. Sandonís; S. Varona; V. Vázquez
EPI_ISL_539569 to 539572, EPI_ISL_862550, EPI_ISL_862552, EPI_ISL_954784, EPI_ISL_954786, EPI_ISL_954791, EPI_ISL_954802			
see above	Complejo Hospitalario de Navarra	Instituto de Salud Carlos III	A. Monzón; F. Casas; I; I. Ezpeleta, C.; I. Jiménez; Iglesias-Caballero; J. López; M. Pozo; M. Camarero; M. Cuesta; M. González-Esguevillas; M. Molinero Calamita; M. Zaballos; M.Camarero; P. Jiménez; P. Zaballos; S. Cuesta; S. Juliá; S. Molinero Calamita; S. Pozo; S. Sandonís; S. Varona; V. Vázquez
EPI_ISL_455334 to 455335, EPI_ISL_578195	Complejo Hospitalario de Orense	Instituto de Salud Carlos III	A. Monzón; F. Casas; I; I. Jiménez; Iglesias-Caballero; M. Camarero; M. Cuesta; M. García; M. González-Esguevillas; M. Molinero Calamita; M. Paz; M. Zaballos; P. Jiménez; S. Juliá; S. Pozo; S. Varona
EPI_ISL_583497	Complexo Hospitalar Ouro Verde de Campinas	Instituto Adolfo Lutz, Interdiciplinary Procedures Center, Strategic Laboratory	Claudia Regina Gonçalves; Claudio Tavares Sacchi; Erica Valessa Ramos Gomes; Karoline Rodrigues Campos
EPI_ISL_523970	Conjunto Hospitalar do Mandaqui	Instituto Adolfo Lutz, Interdiciplinary Procedures Center, Strategic Laboratory	Claudia Regina Gonçalves; Claudio Tavares Sacchi; Erica Valessa Ramos Gomes
EPI_ISL_435716	Connecticut State Department of Public Health	Grubaugh Lab - Yale School of Public Health	Adam Moore; Akiko Iwasaki; Albert Ko; Alice Lu; Allison Nelson; Anderson Brito; Anne Wyllie; Arnau Casanovas; Catherine Muenker; Chaney Kalinich; Chantal Vogels; Charlese Dela Cruz; Cole Jensen; Isabel Ott; Joseph Fauver; Maria Tokuyama; Mary Petrone; Nathan Grubaugh; Patrick Wong; Peiwen Lu; Richard Martinello; Saad Omer; Shelli Farhadian; Tara Alpert
EPI_ISL_738310, EPI_ISL_738312	Connecticut Veterans' Affairs Hospital	Grubaugh Lab - Yale School of Public Health	Chantal Vogels; Danielle Plank; Ellen Foxman; Isabel Ott; Joseph Fauver; Mary Petrone; Nathan Grubaugh; Shaili Gupta; Tara Alpert
EPI_ISL_536288	Conseil Cri de la SSS de la Baie-James	Laboratoire de santé publique du Québec	Guillaume Bourque; Ioannis Ragoussis; Jesse Shapiro; Mark Lathrop and Michel Roger on behalf of the CoVSeQ research group; Sandrine Moreira
EPI_ISL_455327, EPI_ISL_539526 to 539530, EPI_ISL_691681	Consejeria de Sanidad y Asuntos Sociales	Instituto de Salud Carlos III	A. Monzón; F. Casas; G. Gutiérrez; I; I. Gutiérrez, G.; I. Jiménez; Iglesias-Caballero; M. Camarero; M. Cuesta; M. González-Esguevillas; M. Molinero Calamita; M. Pozo; M. Zaballos; P. Jiménez; S. Juliá; S. Molinero Calamita; S. Pozo; S. Varona
EPI_ISL_862546 to 862547, EPI_ISL_862557 to 862563, EPI_ISL_862566	Consejería de Sanidad y Asuntos Sociales	Instituto de Salud Carlos III	A. Monzón; F. Casas; I. Gutiérrez, G.; I. Jiménez; Iglesias-Caballero; M. Camarero; M. Cuesta; M. González-Esguevillas; M. Pozo; M. Zaballos; P. Jiménez; S. Juliá; S. Molinero Calamita; S. Varona
EPI_ISL_965952, EPI_ISL_965957	Consejería de Sanidad y Asuntos Sociales de Castilla La Mancha	Instituto de Salud Carlos III	A. Monzón; F. Casas; I. Gutiérrez, G.; I. Jiménez; Iglesias-Caballero; M. Sandonís; P. Zaballos; S. Camarero; S. Cuesta; S. Pozo; S. Varona; V. Vázquez
EPI_ISL_672488 to 672489	Contra Costa County Public Health Lab	Chan-Zuckerberg Biohub	CZB Cliahub Consortium
EPI_ISL_468615 to 468618, EPI_ISL_468621 to 468622, EPI_ISL_468624 to 468629, EPI_ISL_468631 to 468634, EPI_ISL_468636 to 468640, EPI_ISL_468642, EPI_ISL_468645 to 468652, EPI_ISL_548491 to 548492, EPI_ISL_548495, EPI_ISL_548499, EPI_ISL_548501, EPI_ISL_548506 to 548508, EPI_ISL_548510, EPI_ISL_548515 to 548516, EPI_ISL_548520, EPI_ISL_548524, EPI_ISL_548527 to 548528, EPI_ISL_548531, EPI_ISL_548534 to 548535, EPI_ISL_548537 to 548540, EPI_ISL_548545, EPI_ISL_548548 to 548549, EPI_ISL_548552, EPI_ISL_548554, EPI_ISL_548556 to 548558, EPI_ISL_548561, EPI_ISL_548564 to 548567, EPI_ISL_548569, EPI_ISL_548572 to 548575, EPI_ISL_548577, EPI_ISL_548579			
see above	Contra Costa Public Health Lab	Chan-Zuckerberg Biohub	CZB Cliahub Consortium
EPI_ISL_640015, EPI_ISL_640118, EPI_ISL_700441, EPI_ISL_700467, EPI_ISL_700488, EPI_ISL_700492, EPI_ISL_700496, EPI_ISL_700504, EPI_ISL_700506, EPI_ISL_700533, EPI_ISL_700536, EPI_ISL_700538 to 700539, EPI_ISL_700554, EPI_ISL_700556 to 700557, EPI_ISL_700563, EPI_ISL_700581, EPI_ISL_700587, EPI_ISL_700590, EPI_ISL_700599			
see above	Conville CDC wc CVC	NHLS/UCT	Arash Iranzadeh; Bruna Galvao; Carolyn Williamson; Deelan Doolabh; Diana Hardie; Houriiyah Tegally; Innocent Mudau; Kruger Marais; Lynn Tyers; Marvin Hsiao; Stephen Korsman
EPI_ISL_960119, EPI_ISL_960134	Conville CDC wc CVC	National Health Laboratory Service/UCT	Arash Iranzadeh; Bruna Galvao; Carolyn Williamson; Deelan Doolabh; Diana Hardie; Innocent Mudau; Kruger Marais; Lynn Tyers; Marvin Hsiao; Stephen Korsman
EPI_ISL_696472, EPI_ISL_696492, EPI_ISL_696508 to 696509, EPI_ISL_696512	Conville CDC wc CVC & NHLS/UCT	KRISP, KZN Research Innovation and Sequencing Platform	Arash Iranzadeh; Bruna Galvao; Carolyn Williamson; Deelan Doolabh; Diana Hardie; Emanuel James San; Houriiyah Tegally; Innocent Mudau; Jennifer Giandhari; Kruger Marais; Lynn Tyers; Marvin Hsiao; Stephen Korsman; Sureshnee Pillay; Tulio de Oliveira
EPI_ISL_514751	CoronaNet Lab- TaskForce Regione Campania, CEINGE Biotecnologie Avanzate, Via G. Salvatore	CoronaNet Lab- TaskForce Regione Campania, CEINGE Biotecnologie Avanzate, Via G. Salvatore	Asadzadeh, F.; Atripaldi, L.; Bianchi, M.; Boccia, A.; Borriello, G.; Brandi, S.; Castaldo, G.; Cerino, R.; Chiariotti, L.; Comegna, M.; Ferrucci, V.; Fusco, G.; H.Y.; J.H.; J.M.; Jung; K.S. and Kim; Kong, Dy.; Lee; Marrone, L.; Paolella, G.; Pascarella, S.; Siciliano, R.; Tiberio, C.; Viscardi, M.; Yun; Zollo, M.
EPI_ISL_454635	County Of San Luis Obispo Public Health Laboratory	Chan-Zuckerberg Biohub	CZB Cliahub Consortium
EPI_ISL_468388 to 468407, EPI_ISL_468409 to 468411, EPI_ISL_468413 to 468415, EPI_ISL_468417 to 468422, EPI_ISL_468424 to 468427, EPI_ISL_468429 to 468433, EPI_ISL_468435 to 468437, EPI_ISL_548265, EPI_ISL_548268 to 548269, EPI_ISL_548280 to 548281, EPI_ISL_548284, EPI_ISL_548286, EPI_ISL_548288,			

EPI_ISL_548292, EPI_ISL_548294 to 548295, EPI_ISL_548304, EPI_ISL_548311, EPI_ISL_548316, EPI_ISL_548322, EPI_ISL_548325, EPI_ISL_548329, EPI_ISL_548336, EPI_ISL_548347, EPI_ISL_548350 to 548351, EPI_ISL_548357, EPI_ISL_548359 to 548361, EPI_ISL_548365 to 548366, EPI_ISL_548369 to 548370, EPI_ISL_548425, EPI_ISL_548428, EPI_ISL_548453, EPI_ISL_548457, EPI_ISL_548493, EPI_ISL_548502, EPI_ISL_548504, EPI_ISL_548517, EPI_ISL_548519, EPI_ISL_548521, EPI_ISL_548532, EPI_ISL_548542, EPI_ISL_548544, EPI_ISL_548560, EPI_ISL_548603, EPI_ISL_548631, EPI_ISL_548644, EPI_ISL_548663, EPI_ISL_548671, EPI_ISL_625548 to 625549, EPI_ISL_625623, EPI_ISL_625625 to 625626, EPI_ISL_672087, EPI_ISL_672354 to 672359, EPI_ISL_738890, EPI_ISL_738990, EPI_ISL_739017, EPI_ISL_739038, EPI_ISL_739095, EPI_ISL_739102, EPI_ISL_739363, EPI_ISL_739399, EPI_ISL_739480, EPI_ISL_739492, EPI_ISL_739588			
see above	County of San Luis Obispo Public Health Laboratory	Chan-Zuckerberg Biohub	CZB Cliahub Consortium
EPI_ISL_437044 to 437051, EPI_ISL_437053 to 437057, EPI_ISL_437059 to 437061, EPI_ISL_437063 to 437068, EPI_ISL_437071 to 437077, EPI_ISL_437079, EPI_ISL_437081 to 437085, EPI_ISL_437087 to 437088, EPI_ISL_444023 to 444026			
see above	County of Santa Clara Public Health	Chan-Zuckerberg Biohub	CZB Cliahub Consortium
EPI_ISL_436641 to 436648, EPI_ISL_436650 to 436667, EPI_ISL_436669 to 436683, EPI_ISL_454654 to 454659, EPI_ISL_454661 to 454668, EPI_ISL_454670, EPI_ISL_454672 to 454689, EPI_ISL_468345 to 468351, EPI_ISL_468353 to 468356, EPI_ISL_486115 to 486118, EPI_ISL_513773 to 513784, EPI_ISL_513786, EPI_ISL_513788, EPI_ISL_548264, EPI_ISL_548266 to 548267, EPI_ISL_548279, EPI_ISL_548282, EPI_ISL_548299, EPI_ISL_548302 to 548303, EPI_ISL_548307, EPI_ISL_548310, EPI_ISL_548314, EPI_ISL_548319, EPI_ISL_548327, EPI_ISL_548337 to 548345, EPI_ISL_548379, EPI_ISL_548394 to 548395, EPI_ISL_548401, EPI_ISL_548417, EPI_ISL_548420, EPI_ISL_548423, EPI_ISL_548426, EPI_ISL_548429, EPI_ISL_548433 to 548434, EPI_ISL_548470, EPI_ISL_548478, EPI_ISL_548482, EPI_ISL_548494, EPI_ISL_548496 to 548498, EPI_ISL_548500, EPI_ISL_548509, EPI_ISL_548511 to 548512, EPI_ISL_548514, EPI_ISL_548518, EPI_ISL_548525, EPI_ISL_548529, EPI_ISL_548533, EPI_ISL_548536, EPI_ISL_548541, EPI_ISL_548543, EPI_ISL_548546 to 548547, EPI_ISL_548550 to 548551, EPI_ISL_548553, EPI_ISL_548559, EPI_ISL_548562 to 548563, EPI_ISL_548568, EPI_ISL_548570, EPI_ISL_548576, EPI_ISL_548578, EPI_ISL_548582 to 548584, EPI_ISL_548588, EPI_ISL_548593, EPI_ISL_548595 to 548598, EPI_ISL_548602, EPI_ISL_548604 to 548607, EPI_ISL_548610, EPI_ISL_548612, EPI_ISL_548616 to 548619, EPI_ISL_548621 to 548622, EPI_ISL_548624, EPI_ISL_548629 to 548630, EPI_ISL_548632 to 548633, EPI_ISL_548636, EPI_ISL_548639, EPI_ISL_548642 to 548643, EPI_ISL_548645, EPI_ISL_548647, EPI_ISL_548649 to 548650, EPI_ISL_548652, EPI_ISL_548655 to 548659, EPI_ISL_548661, EPI_ISL_548664, EPI_ISL_548669 to 548670, EPI_ISL_548672 to 548673, EPI_ISL_548676, EPI_ISL_548678 to 548679, EPI_ISL_582851 to 582897, EPI_ISL_582899 to 582919, EPI_ISL_582921 to 582926, EPI_ISL_582928 to 582942, EPI_ISL_582949 to 582955, EPI_ISL_582957 to 582958			
see above	County of Santa Clara Public Health Department	Chan-Zuckerberg Biohub	CZB Cliahub Consortium
EPI_ISL_735494	Cox's Bazar Medical College	Central Biological Research Laboratory and Department of Biochemistry and Molecular Biology	H. M. Abdullah Al Masud; Imam Hossen; Md. Arif Hossain; Md. Imranul Hoq; Md. Khondakar Raziur Rahman; Md. Omer Faruq; Mohammad Omar Faruque; Robiul Hasan Bhuiyan; Sajib Rudra; Shanta Paul
EPI_ISL_735493	Cox's Bazar Medicila College	Central Biological Research Laboratory and Department of Biochemistry and Molecular Biology Central Biological Research Laboratory and Department of Biochemistry and Molecular Biology	H. M. Abdullah Al Masud; Imam Hossen; Md. Arif Hossain; Md. Imranul Hoq; Md. Khondakar Raziur Rahman; Md. Omer Faruq; Mohammad Omar Faruque; Robiul Hasan Bhuiyan; Sajib Rudra; Shanta Paul
EPI_ISL_700443	Crags Clinic wc CRG	NHLS/UCT	Arash Iranzadeh; Bruna Galvao; Carolyn Williamson; Deelan Doolabh; Diana Hardie; Houriiyah Tegally; Innocent Mudau; Kruger Marais; Lynn Tyers; Marvin Hsiao; Stephen Korsman
EPI_ISL_454602	Croatian Institute of Public Health	University of Zagreb, Centre for research and knowledge transfer in biotechnology	Anamarija Slovic; Irena Tabain; Jelena Ivancic Jelecki; Tatjana Vilibic-Cavlek
EPI_ISL_693207	Cs II Doutor Antonio Vicoso Moreira de Rezende	Instituto Adolfo Lutz, Interdiciplinary Procedures Center, Strategic Laboratory	Claudia Regina Gonçalves; Claudio Tavares Sacchi; Erica Valessa Ramos Gomes; Karoline Rodrigues Campos
EPI_ISL_935155	Cytocheck Laboratory	Kansas Health and Environmental Lab	Ben Olsen; Carissa Robertson; Mike Grose; Paige Drury; and Phil Adam
EPI_ISL_640029, EPI_ISL_640048, EPI_ISL_700427, EPI_ISL_700448, EPI_ISL_700470, EPI_ISL_700564, EPI_ISL_700569			
see above	D'Almeida Clinic wc DAL	NHLS/UCT	Arash Iranzadeh; Bruna Galvao; Carolyn Williamson; Deelan Doolabh; Diana Hardie; Houriiyah Tegally; Innocent Mudau; Kruger Marais; Lynn Tyers; Marvin Hsiao; Stephen Korsman
EPI_ISL_476282, EPI_ISL_476288 to 476289, EPI_ISL_476297	DB Diagnósticos do Brasil	Instituto de Medicina Tropical da Univesidade de São Paulo	Camila Alves Maia da Silva; Darlan da Silva Candido; Erika Regina Manuli; Ester Sabino; Flavia Cristina da Silva Sales; Giula Magalhaes Ferreira; Jaqueline Goes de Jesus; Julien Theze; Mariana Severo Ramundo; Nuno Faria; Samples: Nelson Gaburo Jr; Sequencing: Ingra Morales Claro; Thais de Moura Coletti
EPI_ISL_594460	DC Department of Forensic Sciences	Pathogen Discovery, Respiratory Viruses Branch, Division of Viral Diseases, Centers for Disease Control and Prevention	Anna Uehara; Clinton Paden; Haibin Wang; Jing Zhang; Julu Bhatnagar; Krista Queen; Suxiang Tong; Yan Li; Ying Tao
EPI_ISL_436040 to 436043	DC Public Health Lab Dept of Forensic Science	Pathogen Discovery, Respiratory Viruses Branch, Division of Viral Diseases, Centers for Disease Control and Prevention	Anna Uehara; Bettina Bankamp; Clinton R. Paden; Haibin Wang; Jing Zhang; Krista Queen; Suxiang Tong; Yan Li; Ying Tao; Zachary Weiner
EPI_ISL_424852, EPI_ISL_447840, EPI_ISL_576178	DC Public Health Lab/ Dept. of Forensic Sciences	Pathogen Discovery, Respiratory Viruses Branch, Division of Viral Diseases, Centers for Disease Control and Prevention	Alison S. Laufer Halpin; Anna Uehara; Brian Lynch; Christopher A. Elkins; Clinton R. Paden; Haibin Wang; Jasmine Padilla; Jing Zhang; Justin Lee; Krista Queen; Mary S. Keckler; Peter Cook; Rachel Marine; Suxiang Tong; Yan Li; Ying Tao
EPI_ISL_751602, EPI_ISL_751605, EPI_ISL_751621, EPI_ISL_751623, EPI_ISL_751627, EPI_ISL_751631, EPI_ISL_751726			
see above	DE Public Health Laboratory	Genomics and Discovery, Respiratory Viruses Branch, Division of Viral Diseases, Centers for Disease Control and Prevention	Anna Montmayeur; Anna Uehara; Clinton R. Paden; Haibin Wang; Jing Zhang; Justin Lee; Krista Queen; Mili Sheth; Peter W. Cook; Rachel Marine; Suxiang Tong; Yan Li; Ying Tao
EPI_ISL_486388 to 486389, EPI_ISL_486405, EPI_ISL_486408 to 486409, EPI_ISL_515945, EPI_ISL_515947, EPI_ISL_515954, EPI_ISL_515957 to 515959, EPI_ISL_515961			
see above	DH	Department of Neurovirology, National Institute of Mental Health and Neuroscience (NIMHANS)	Anita Desai; Chitra Pattabiraman; Harsha PK; Manjunatha Venkataswamy; Pramada Prasad; Ravi Vasanthapuram; Risha Rasheed; Shafeeq S Hameed; Vijayalakshmi Reddy
EPI_ISL_961078 to 961080, EPI_ISL_961083, EPI_ISL_961094 to 961095	DIP. PREV. AVEZZANO SERVIZIO DI IGIENE EPIDEMIOLOGIAE SANITA' PUBBLICA	Istituto Zooprofilattico Sperimentale dell'Abruzzo e Molise "G. Caporale"	Ancora M; Calistri P; Cammà C; Curini V; Di Domenico M; Di Pasquale A; Lorusso A; Mangone I; Marcacci M; Puglia I; Rinaldi A; Savini G; Scialabba S
EPI_ISL_849635 to 849636	DIPARTIMENTO PREVENZIONE AVEZZANO-SERVIZIO DI IGIENE EPIDEMIOLOGIA E SANITA' PUBBLICA	Istitituto Zooprofilattico Sperimentale dell'Abruzzo e Molise "G.Caporale"	Ancora M; Cammà C; Curini V; Di Domenico M; Di Pasquale A; Lorusso A; Mangone I; Marcacci M; Puglia I; Rinaldi A; Savini G.
EPI_ISL_747283	DNA SOLUTION LTD.	DNA SOLUTION LTD.	Abdul Khaleque; Abu Sufian; Firoz Kabir; Jannatun Naima; Kazi Nadim Hasan; Md. Imran Khan; Mizanur Rahman; Mohammad Fazle Alam Rabbi; Sharif Akhteruzzaman
EPI_ISL_445214 to 445216	DNA Solution Ltd.	DNA Solution Ltd.	Abdul Khaleque; Abu Sufian; Hasan Ul Haider; Kazi Nadim Hasan; MSM Chowdhury; Mala Khan; Mamudul Hasan Razu; Md. Imran Khan; Mizanur Rahman; Mohammad Fazle Alam Rabbi; Mohammed Nafiz Imtiaz Polol
EPI_ISL_633009 to 633010, EPI_ISL_633037, EPI_ISL_683795, EPI_ISL_683813, EPI_ISL_683829, EPI_ISL_683834, EPI_ISL_683924, EPI_ISL_683927 to 683928, EPI_ISL_683933, EPI_ISL_683937, EPI_ISL_683962, EPI_ISL_683964, EPI_ISL_683968, EPI_ISL_683974, EPI_ISL_683976 to 683977, EPI_ISL_683982 to 683985, EPI_ISL_683991, EPI_ISL_745413, EPI_ISL_745425, EPI_ISL_745451, EPI_ISL_745469, EPI_ISL_857094, EPI_ISL_857200, EPI_ISL_861332, EPI_ISL_883324			
see above	DOHMH Central Harlem	New York City Public Health Laboratory	Jade Wang; et al.
EPI_ISL_633014, EPI_ISL_633016 to 633017, EPI_ISL_633025, EPI_ISL_633031, EPI_ISL_633033, EPI_ISL_633039, EPI_ISL_633076, EPI_ISL_633078, EPI_ISL_671684, EPI_ISL_671693, EPI_ISL_671713, EPI_ISL_671722, EPI_ISL_683765, EPI_ISL_683780, EPI_ISL_683788, EPI_ISL_683792, EPI_ISL_683807 to 683808, EPI_ISL_683828, EPI_ISL_683830, EPI_ISL_683923, EPI_ISL_683931, EPI_ISL_683936, EPI_ISL_683941, EPI_ISL_683957, EPI_ISL_683961, EPI_ISL_683967, EPI_ISL_683971 to 683972, EPI_ISL_683980 to 683981, EPI_ISL_683989, EPI_ISL_745410, EPI_ISL_745421 to 745422, EPI_ISL_745436, EPI_ISL_745453, EPI_ISL_745457			
see above	DOHMH Chelsea	New York City Public Health Laboratory	Jade Wang; et al.
EPI_ISL_632980 to 632982, EPI_ISL_632984 to 632987, EPI_ISL_632989 to 632993, EPI_ISL_633059, EPI_ISL_633074, EPI_ISL_671682 to 671683, EPI_ISL_671685, EPI_ISL_671695, EPI_ISL_671699 to 671700, EPI_ISL_671703, EPI_ISL_671710, EPI_ISL_671719 to 671721, EPI_ISL_671732, EPI_ISL_671746 to 671747, EPI_ISL_683766, EPI_ISL_683773, EPI_ISL_683777, EPI_ISL_683779, EPI_ISL_683785, EPI_ISL_683793 to 683794, EPI_ISL_683796, EPI_ISL_683798 to 683800, EPI_ISL_683802, EPI_ISL_683809, EPI_ISL_683811 to 683812, EPI_ISL_683815, EPI_ISL_683817 to 683818, EPI_ISL_683824, EPI_ISL_683831, EPI_ISL_683833, EPI_ISL_683876, EPI_ISL_683881, EPI_ISL_683893, EPI_ISL_683896 to 683900, EPI_ISL_683908 to 683909, EPI_ISL_683911 to 683912, EPI_ISL_683926, EPI_ISL_683929, EPI_ISL_683932, EPI_ISL_683934, EPI_ISL_683952 to 683953, EPI_ISL_683960, EPI_ISL_683978 to 683979, EPI_ISL_745409, EPI_ISL_745423 to 745424, EPI_ISL_745426 to 745427, EPI_ISL_745434 to 745435, EPI_ISL_745446 to 745447, EPI_ISL_745455, EPI_ISL_745458 to 745462, EPI_ISL_745464, EPI_ISL_745466 to 745467, EPI_ISL_966399			
see above	DOHMH Corona	New York City Public Health Laboratory	Jade Wang; et al.
EPI_ISL_632994 to 632998, EPI_ISL_671690, EPI_ISL_671760, EPI_ISL_683821, EPI_ISL_683827, EPI_ISL_683891, EPI_ISL_683904, EPI_ISL_683910, EPI_ISL_683935, EPI_ISL_683970, EPI_ISL_745419 to 745420, EPI_ISL_745445, EPI_ISL_745448, EPI_ISL_745452, EPI_ISL_745454, EPI_ISL_1018024			
see above	DOHMH Crown Heights	New York City Public Health Laboratory	Jade Wang; et al.
EPI_ISL_633073, EPI_ISL_671738, EPI_ISL_683771, EPI_ISL_683816, EPI_ISL_683832, EPI_ISL_683887 to 683888, EPI_ISL_683906, EPI_ISL_683913, EPI_ISL_683922, EPI_ISL_683963, EPI_ISL_683973, EPI_ISL_683996, EPI_ISL_745463			

see above	DOHMH Fort Greene	New York City Public Health Laboratory	Jade Wang; et al.
EPI_ISL_632999 to 633008, EPI_ISL_633062, EPI_ISL_671692, EPI_ISL_671698, EPI_ISL_671712, EPI_ISL_671723 to 671724, EPI_ISL_671730, EPI_ISL_671735, EPI_ISL_683775 to 683776, EPI_ISL_683781 to 683782, EPI_ISL_683786, EPI_ISL_683791, EPI_ISL_683822, EPI_ISL_683878, EPI_ISL_683885, EPI_ISL_683892, EPI_ISL_683901, EPI_ISL_683903, EPI_ISL_683914 to 683916, EPI_ISL_683920 to 683921, EPI_ISL_683925, EPI_ISL_683930, EPI_ISL_683939, EPI_ISL_683944 to 683945, EPI_ISL_683948, EPI_ISL_683951, EPI_ISL_683956, EPI_ISL_683958, EPI_ISL_683975, EPI_ISL_683986, EPI_ISL_683990, EPI_ISL_745414 to 745418, EPI_ISL_745440, EPI_ISL_745444, EPI_ISL_883318 to 883323, EPI_ISL_966398			
see above	DOHMH Jamaica	New York City Public Health Laboratory	Jade Wang; et al.
EPI_ISL_633015, EPI_ISL_633019, EPI_ISL_633027, EPI_ISL_633029 to 633030, EPI_ISL_633032, EPI_ISL_633036, EPI_ISL_633044, EPI_ISL_633067, EPI_ISL_633075, EPI_ISL_633080 to 633082, EPI_ISL_671687, EPI_ISL_671726 to 671728, EPI_ISL_671736, EPI_ISL_671740, EPI_ISL_671751 to 671754, EPI_ISL_671757 to 671758, EPI_ISL_683772, EPI_ISL_683778, EPI_ISL_683790, EPI_ISL_683801, EPI_ISL_683803 to 683806, EPI_ISL_683814, EPI_ISL_683823, EPI_ISL_683825 to 683826, EPI_ISL_683879 to 683880, EPI_ISL_683884, EPI_ISL_683886, EPI_ISL_683889, EPI_ISL_683905, EPI_ISL_683917 to 683919, EPI_ISL_683938, EPI_ISL_683940, EPI_ISL_683946 to 683947, EPI_ISL_683959, EPI_ISL_683965 to 683966, EPI_ISL_683969, EPI_ISL_683992, EPI_ISL_745412, EPI_ISL_745430 to 745433, EPI_ISL_745441 to 745443, EPI_ISL_745449 to 745450, EPI_ISL_745470, EPI_ISL_857093			
see above	DOHMH Morrisania	New York City Public Health Laboratory	Jade Wang; et al.
EPI_ISL_633011, EPI_ISL_633013, EPI_ISL_633035, EPI_ISL_633038, EPI_ISL_633045, EPI_ISL_633055, EPI_ISL_633071, EPI_ISL_633079, EPI_ISL_671715 to 671716, EPI_ISL_671744, EPI_ISL_683774, EPI_ISL_683783, EPI_ISL_683787, EPI_ISL_683789, EPI_ISL_683797, EPI_ISL_683810, EPI_ISL_683875, EPI_ISL_683890, EPI_ISL_683943, EPI_ISL_683954, EPI_ISL_683987 to 683988, EPI_ISL_683993, EPI_ISL_683995, EPI_ISL_745437 to 745438, EPI_ISL_745456, EPI_ISL_745465, EPI_ISL_937154			
see above	DOHMH PHL	New York City Public Health Laboratory	Jade Wang; et al.
EPI_ISL_633012, EPI_ISL_633034, EPI_ISL_633041 to 633042, EPI_ISL_633072, EPI_ISL_671701, EPI_ISL_671709, EPI_ISL_671725, EPI_ISL_671733, EPI_ISL_671748 to 671750, EPI_ISL_671762, EPI_ISL_683784, EPI_ISL_683819 to 683820, EPI_ISL_683894 to 683895, EPI_ISL_683902, EPI_ISL_683907, EPI_ISL_683942, EPI_ISL_683949 to 683950, EPI_ISL_683955, EPI_ISL_683994, EPI_ISL_745428 to 745429, EPI_ISL_745439, EPI_ISL_745468			
see above	DOHMH Riverside	New York City Public Health Laboratory	Jade Wang; et al.
EPI_ISL_933736 to 933738	DPHL	Delaware Public Health Lab	Gregory Hovan
EPI_ISL_416542 to 416543, EPI_ISL_421652	Dasman Diabetes Institute	Dasman Diabetes Institute	Ebaa Al-Ozairi; Ebaa AlOzairi; Fahd Al-Mulla; Motasem Melhem; Qais Al-Duwairi; Rasheeba Iqbal; Sara Al-Qabandi; Sumi John
EPI_ISL_416541	Dasman Diabetes Institute and Virology Laboratory Ministry of Health	Dasman Diabetes Institute	Ebaa AlOzairi; Fahd Al-Mulla; Motasem Melhem; Qais Al-Duwairi; Rasheeba Iqbal; Sara Al-Qabandi; Sumi John
EPI_ISL_476022	Defence Research & Development Establishment	Defence Research & Development Establishment	Ambuj Shrivastava; Jyoti S Kumar; Paban Kumar Dash; Shashi Sharma; Sushil Kumar Sharma
EPI_ISL_476023, EPI_ISL_476840, EPI_ISL_476842, EPI_ISL_476844, EPI_ISL_476846, EPI_ISL_476848 to 476850, EPI_ISL_476852 to 476854, EPI_ISL_476883 to 476896			
see above	Defence Research & Development Establishment (DRDE)	Defence Research & Development Establishment (DRDE)	Ambuj Shrivastava; Jyoti S. Kumar; Paban Kumar Dash; Shashi Sharma; Sushil Kumar Sharma
EPI_ISL_560406, EPI_ISL_560647, EPI_ISL_560741, EPI_ISL_561335, EPI_ISL_561345 to 561346, EPI_ISL_561351 to 561353, EPI_ISL_561356, EPI_ISL_561358, EPI_ISL_565830, EPI_ISL_565832, EPI_ISL_566084, EPI_ISL_566108, EPI_ISL_574585 to 574586, EPI_ISL_574599, EPI_ISL_576150 to 576158, EPI_ISL_576160 to 576162, EPI_ISL_576164 to 576167, EPI_ISL_576169, EPI_ISL_576223 to 576227, EPI_ISL_583246, EPI_ISL_583248, EPI_ISL_583994, EPI_ISL_584013 to 584014, EPI_ISL_593938, EPI_ISL_593940, EPI_ISL_593945, EPI_ISL_593949 to 593953, EPI_ISL_593955 to 593958, EPI_ISL_593963, EPI_ISL_593965 to 593967, EPI_ISL_593969 to 593971, EPI_ISL_593973 to 593974, EPI_ISL_593976, EPI_ISL_593979 to 593981, EPI_ISL_708433 to 708452			
see above	Delaware Public Health Lab	Delaware Public Health Lab	Gregory Hovan
EPI_ISL_693728 to 693757, EPI_ISL_766989 to 767012	Delaware Public Health Laboratory	Delaware Public Health Laboratory	Gregory Hovan
EPI_ISL_529147 to 529148	Democritus University of Thrace, Department of Medicine	Democritus University of Thrace, Department of Medicine	Bampali, M.; Dovrolis, N.; Froukala, E.; Gatzidou, E.; Karakasiliotis, I.; Kassela, K.; Spanakis, N.; Stavropoulou, A.; Tsakris, A.; Veletza, S.
EPI_ISL_430796 to 430798, EPI_ISL_430802, EPI_ISL_430805, EPI_ISL_430808	Departamento de Biología y genética molecular, IACA Laboratorios.	Área de Secuenciación del Laboratorio de Virología del Hospital de Niños Dr. Ricardo Gutierrez on behalf of 'Proyecto Argentino Interinstitucional de genomica de SARS-CoV-2' (PAIS Consortium)	A; AS; E; Goya; LE; Lusso; MI; MS; Masciovecchio MV; Mistchenko; Nabaes Jodar; Natale; S; Streitenberger ER; Suárez; Tittarelli; Valinotto; Viegas, M.
EPI_ISL_444493	Departamento de Laboratorios de Salud Publica (DLSP, Division Epidemiologia, Ministerio de Salud Publica)	Facultad de Ciencias (Sección Genética Evolutiva, Sección Virología).	Arbiza; Calleros, L.; Chiparelli, H.; Coppola, L.; Delfraro, A.; Frabasile, S.; Fuques, E.; Goni, N.; Grecco, S.; J. and Perez, R.; Panzera, Y.; Ramos, N.; Ramos, V.; Techera, C.
EPI_ISL_508627, EPI_ISL_508639 to 508643, EPI_ISL_508646 to 508647, EPI_ISL_508653, EPI_ISL_508661, EPI_ISL_508663, EPI_ISL_508667, EPI_ISL_508676			
see above	Departamento de Microbiología, CDB, Hospital Clínic, Barcelona	SeqCOVID-SPAIN consortium/IBV(CSIC)	Aida Peiró and SeqCOVID-SPAIN consortium; Andrea Vergara; Elisa Rubio; Jéssica Navero; Mikel Martínez
EPI_ISL_603022, EPI_ISL_603034	Departamento de Vigilância à Saúde	Instituto Adolfo Lutz, Interdiciplinary Procedures Center, Strategic Laboratory	Claudia Regina Gonçalves; Claudio Tavares Sacchi; Erica Valessa Ramos Gomes; Karoline Rodrigues Campos
EPI_ISL_516922 to 516933	Department for Molecular Diagnostics, Centre for Medical Microbiology, Institute of Public Health of Montenegro	Charite Universitatsmedizin Berlin, Institut fur Virologie	Barbara Muehlemann; Christian Drosten; Julia Schneider; Jörn Beheim-Schwarzbach; Marija Govedarica and Danijela Vujoševi; Talitha Veith; Terry Jones; Victor M Corman
EPI_ISL_1013596 to 1013597, EPI_ISL_1013600, EPI_ISL_1013602, EPI_ISL_1013607 to 1013608, EPI_ISL_1013610	Department for Molecular Diagnostics, Centre for Medical Microbiology, Institute of Public Health, Montenegro	Charité Universitätsmedizin Berlin, Institut für Virologie	Barbara Mühlemann; Christian Drosten; Danijela Vujoševi; Julia Schneider; Julia Tesch; Jörn Beheim-Schwarzbach; Marija Govedarica; Talitha Veith; Terry Jones; Tobias Bleicker; Victor M Corman
EPI_ISL_420140, EPI_ISL_420142, EPI_ISL_420144	Department for Virology, Molecular Biology and Genome Research, R. G. Lugar Center for Public Health Research, National Center for Disease Control and Public Health (NCDC) of Georgia.	Department for Virology, Molecular Biology and Genome Research, R. G. Lugar Center for Public Health Research, National Center for Disease Control and Public Health (NCDC) of Georgia.	Adam Kotorashvili; Amiran Gamkrelidze; Amiran Gamkrelidze.; Ana Papkiauri; Ann Machablishvili; Anna Kasradze; Davit Tsaguria; Ekaterine Khmaladze; Ekaterine Zangaladze; Ekaterine Zhghenti; Giorgi Tomashvili; Gvantsa Brachveli; Gvantsa Chanturia; Irma Burjanadze; Ketevan Sidamonidze; Khatuna Zakhashvili; Lela Sabadze; Lela Urushadze; Magda Dgebuadze; Maia Alkhazashvili; Mari Gavashelidze; Mariam Zakalashvili; Marine Murtskhvaladze; Meri Pantsulaia; Nato Kotaria; Nino Berishvili; Paata Imnadze; Roena Sukhiashvili; Tamar Jashiashvili; Tata Imnadze; Tea Tevdoradze
EPI_ISL_447055 to 447056, EPI_ISL_470876 to 470877, EPI_ISL_477169, EPI_ISL_481380, EPI_ISL_481483, EPI_ISL_754180 to 754181, EPI_ISL_763062			
see above	Department for Virology, Molecular Biology and Genome Research, R. G. Lugar Center for Public Health Research, National Center for Disease Control and Public Health (NCDC) of Georgia.	Department for Virology, Molecular Biology and Genome Research, R. G. Lugar Center for Public Health Research, National Center for Disease Control and Public Health (NCDC) of Georgia.	Adam Kotorashvili; Amiran Gamkrelidze.; Ana Papkiauri; Ann Machablishvili; Anna Kasradze; Davit Tsaguria; Ekaterine Khmaladze; Ekaterine Zangaladze; Ekaterine Zhghenti; Giorgi Tomashvili; Gvantsa Brachveli; Gvantsa Chanturia; Irma Burjanadze; Ketevan Sidamonidze; Khatuna Zakhashvili; Lela Sabadze; Lela Urushadze; Magda Dgebuadze; Maia Alkhazashvili; Mari Gavashelidze; Mariam Zakalashvili; Marine Murtskhvaladze; Meri Pantsulaia; Nato Kotaria; Nino Berishvili; Paata Imnadze; Roena Sukhiashvili; Salome Javashvili; Tamar Jashiashvili; Tata Imnadze; Tea Tevdoradze
EPI_ISL_632934	Department of Acute Infectious Diseases Control and Prevention,Yunnan Provincial Center for Disease Control and Prevention	Department of Acute Infectious Diseases Control and Prevention,Yunnan Provincial Center for Disease Control and Prevention	Jienan Zhou; Meiling Zhang; Senquan Jia; Xiaonan Zhao; Xiaoqing Fu
EPI_ISL_515082, EPI_ISL_515084, EPI_ISL_515090, EPI_ISL_515096, EPI_ISL_515098, EPI_ISL_515100 to 515102, EPI_ISL_515107 to 515108, EPI_ISL_515110 to 515111			
see above	Department of Biochemistry, Cell and Molecular Biology	WACCBIP, University of Ghana	A.K.; Adu, B.; Amenga-Etego; Ampofo, W.; Amuzu; Anang; Arjarquah, A.; Asante, I.; Awandare; Bediako, Y.; Boatemaa, L.; Bonney, E.; Bonney, K.; C.M.; D.S.; Eshun, M.; G.A.; G.B.; J.K.; J.M.; Kotey, E.; Kumordjie, S.; Kyei; L.N.; Magnussen, V.; Morang'a; Mutungi; Ngoi; Quashie, P.; Tei-Maya, F.
EPI_ISL_944664, EPI_ISL_944666 to 944677, EPI_ISL_944679 to 944702, EPI_ISL_944706 to 944709, EPI_ISL_944711 to 944716, EPI_ISL_944719, EPI_ISL_944721 to 944726			
see above	Department of Biochemistry, Cell and Molecular Biology, West African Centre for Cell Biology of Infectious Pathogens (WACCBIP), University of Ghana	Department of Biochemistry, Cell and Molecular Biology, West African Centre for Cell Biology of Infectious Pathogens (WACCBIP), University of Ghana	Adusei-Poku, M.; Amenga-Etego; Ampofo; Amuzu; Asante, I.; Awandare; Bediako, Y.; Bonney; Bonney, E.; C.M.; D.S.; E.B.; G.A.; J.H.; J.K.; J.M.; L.N.; Morang'a; N.T.; Ndam; Ngoi; Odoom; Ofori-Boadu, L.; Quansah; Quashie, P.; Saiid, S.; Tei-Maya, F.; W.K.
EPI_ISL_411929	Department of Clinical Diagnostics	Department of Clinical Diagnostics	C.K.; Choe; Choi; G.-W.; J.-S. and Oh; J.Y.; Kang; Kim; Kim, N.; Kwon; Lee; M.-D.; M.-W.; N.-J.; P.G.; Park; S.-J.; Seo; Seong; W.B.; Yun, J.
EPI_ISL_429239	Department of Clinical Laboratory, the First People's Hospital of Yunnan Province	Department of Clinical Laboratory, the First People's Hospital of Yunnan Province	Guiqian Zhang; Xin Fan; Ya Xu; Yi Sun; Yu Zhang; Ziqin Dian
EPI_ISL_416997, EPI_ISL_417004, EPI_ISL_417006, EPI_ISL_417008 to 417009, EPI_ISL_417012 to 417023, EPI_ISL_417025, EPI_ISL_418624 to 418636, EPI_ISL_418638 to 418640, EPI_ISL_418645 to 418646, EPI_ISL_418648 to 418657, EPI_ISL_418659 to 418661, EPI_ISL_418663 to 418664, EPI_ISL_421182 to 421196, EPI_ISL_421198, EPI_ISL_421200 to 421207, EPI_ISL_421210, EPI_ISL_421212 to 421214, EPI_ISL_424629 to 424631, EPI_ISL_424633, EPI_ISL_424636 to 424637, EPI_ISL_424640 to 424647, EPI_ISL_424649 to 424655, EPI_ISL_424657 to 424658, EPI_ISL_424660 to 424662, EPI_ISL_424664, EPI_ISL_427349 to 427352, EPI_ISL_427354, EPI_ISL_427357 to 427359, EPI_ISL_427364, EPI_ISL_427366, EPI_ISL_427370 to 427372, EPI_ISL_427379, EPI_ISL_427385, EPI_ISL_427388, EPI_ISL_427390, EPI_ISL_447120 to 447124, EPI_ISL_447127 to 447130, EPI_ISL_447133 to 447141, EPI_ISL_447148, EPI_ISL_447152 to 447153,			

EPI_ISL_447155, EPI_ISL_455958, EPI_ISL_455961 to 455963, EPI_ISL_455965 to 455970, EPI_ISL_455972 to 455979, EPI_ISL_471427, EPI_ISL_471429 to 471431, EPI_ISL_471435 to 471436, EPI_ISL_484697 to 484700, EPI_ISL_484702 to 484703, EPI_ISL_498127 to 498139, EPI_ISL_498141, EPI_ISL_498143 to 498144, EPI_ISL_498146, EPI_ISL_498149 to 498151, EPI_ISL_498628 to 498629, EPI_ISL_515055 to 515071, EPI_ISL_515073 to 515081, EPI_ISL_540469 to 540482, EPI_ISL_540484, EPI_ISL_540486 to 540508, EPI_ISL_540510 to 540512, EPI_ISL_540514 to 540515, EPI_ISL_540517, EPI_ISL_540523, EPI_ISL_540525, EPI_ISL_540527 to 540528, EPI_ISL_540531, EPI_ISL_540533 to 540535, EPI_ISL_540537, EPI_ISL_540541 to 540544, EPI_ISL_540547 to 540548, EPI_ISL_540550 to 540551, EPI_ISL_540555, EPI_ISL_540558 to 540560, EPI_ISL_540565 to 540566, EPI_ISL_540568 to 540569, EPI_ISL_540573 to 540574, EPI_ISL_540578, EPI_ISL_581586, EPI_ISL_581590 to 581593, EPI_ISL_581595 to 581667, EPI_ISL_582027, EPI_ISL_626231 to 626263, EPI_ISL_626265 to 626291, EPI_ISL_626293 to 626297, EPI_ISL_626299 to 626307, EPI_ISL_626310 to 626312, EPI_ISL_626314 to 626316, EPI_ISL_626318 to 626328, EPI_ISL_626330 to 626339, EPI_ISL_636605 to 636617, EPI_ISL_636619 to 636677, EPI_ISL_636679 to 636682, EPI_ISL_636684 to 636685, EPI_ISL_641557 to 641581, EPI_ISL_641583 to 641596, EPI_ISL_641598 to 641603, EPI_ISL_641605 to 641606, EPI_ISL_644611 to 644617, EPI_ISL_644619 to 644622, EPI_ISL_644624 to 644626, EPI_ISL_661218 to 661251, EPI_ISL_666872 to 666874, EPI_ISL_666877 to 666879, EPI_ISL_666881 to 666887, EPI_ISL_678399 to 678408, EPI_ISL_678411, EPI_ISL_678417 to 678418, EPI_ISL_678422, EPI_ISL_678425 to 678427, EPI_ISL_678430 to 678431, EPI_ISL_678434 to 678437, EPI_ISL_678440, EPI_ISL_678442 to 678449, EPI_ISL_678453, EPI_ISL_678456 to 678461, EPI_ISL_678464 to 678466, EPI_ISL_678468 to 678470, EPI_ISL_678472 to 678480, EPI_ISL_678483 to 678485, EPI_ISL_681264 to 681266, EPI_ISL_707721, EPI_ISL_707723 to 707724, EPI_ISL_707726 to 707770, EPI_ISL_708453 to 708455, EPI_ISL_708458 to 708459, EPI_ISL_722934 to 722966, EPI_ISL_737310 to 737311, EPI_ISL_737313 to 737314, EPI_ISL_737319 to 737320, EPI_ISL_737322, EPI_ISL_737325 to 737326, EPI_ISL_737329 to 737330, EPI_ISL_737333, EPI_ISL_737335, EPI_ISL_737338, EPI_ISL_737343 to 737344, EPI_ISL_737346 to 737347, EPI_ISL_737349 to 737351, EPI_ISL_737353 to 737354, EPI_ISL_737357, EPI_ISL_737359 to 737360, EPI_ISL_737362 to 737373, EPI_ISL_737376 to 737377, EPI_ISL_737379, EPI_ISL_737381, EPI_ISL_737383 to 737387, EPI_ISL_737390 to 737394, EPI_ISL_812122, EPI_ISL_832221, EPI_ISL_872113 to 872116, EPI_ISL_872122, EPI_ISL_872127 to 872128, EPI_ISL_930603, EPI_ISL_930610, EPI_ISL_935657, EPI_ISL_935661, EPI_ISL_935668 to 935669, EPI_ISL_1036603 to 1036604, EPI_ISL_1040438 to 1040439, EPI_ISL_1040442, EPI_ISL_1040447 to 1040448			
see above	Department of Clinical Microbiology	GIGA Medical Genomics	Artesi Maria; Axelle Chaslain; Bontems Sébastien; Boreux Raphaël; Bouchra Boujemla; Bours Vincent.; Cecile Meex; Celine Fombellida-Lopez; Cécile Meex; Céline Fombellida-Lopez; Durkin Keith; Gilles Darcis; Hayette Marie-Pierre; Justine Defêche; Keith Durkin; Maria Artesi; Marie-Pierre Hayette; Meex Cécile; Melin Pierrette; Michel Moutschen; Nathalie Renotte; Pierrette Melin; Raphael Boreux; Raphaël Boreux; Sebastien Bontems; Sébastien Bontems; Vincent Bours; Vincent Bours.
EPI_ISL_429262 to 429285, EPI_ISL_429287, EPI_ISL_429289, EPI_ISL_429293, EPI_ISL_429295, EPI_ISL_429299 to 429312, EPI_ISL_429315 to 429316, EPI_ISL_429318, EPI_ISL_429321 to 429325, EPI_ISL_429327 to 429329, EPI_ISL_429331, EPI_ISL_451988 to 452005, EPI_ISL_452007 to 452010, EPI_ISL_452012 to 452035, EPI_ISL_452037 to 452039, EPI_ISL_452042 to 452045, EPI_ISL_452048 to 452050, EPI_ISL_452052 to 452062, EPI_ISL_452064 to 452070, EPI_ISL_452074 to 452080, EPI_ISL_452082 to 452094, EPI_ISL_452098 to 452100			
see above	Department of Clinical Microbiology, Copenhagen University Hospital, Hvidovre, Kettegaard Alle 30, 2650 Hvidovre.	Albertsen lab, Department of Chemistry and Bioscience, Aalborg University, Denmark	Rasmus Kirkegaard
EPI_ISL_417187 to 417188, EPI_ISL_417193, EPI_ISL_417197, EPI_ISL_418815, EPI_ISL_419214 to 419216, EPI_ISL_419219, EPI_ISL_419224 to 419229, EPI_ISL_419232, EPI_ISL_419245, EPI_ISL_419247, EPI_ISL_419250, EPI_ISL_419252			
see above	Department of Clinical Pathology, Pamela Youde Nethersole Eastern Hospital	Department of Health Technology and Informatics, Faculty of Health and Social Science, The Hong Kong Polytechnic University	Alan Ka-Lun WU; Alex Yat-Man HO; Barry Kin-Chung WONG; David Ho-Keung SHUM; Eugene Yuk-Keung TSO; Gilman Kit-Hang SIU; Hiu-Yin LAO; Kam-Tong YIP; Kam-Tong Yip; Kenneth Siu-Sing LEUNG; Kingsley King-Gee TAM; Kit-Man SIN; Kitty Sau-Chun FUNG; Kwok-Cheung LUNG; Lam-Kwong LEE; Man-Chun CHAN; Ming-Pan CHOI; Miranda Chong-Yee YAU; Raymond Wai-To LIU; Sandy Ka-Yee CHAU; Shea Ping YIP; Tak-Lun QUE; Tak-Lun Que; Timothy Ting-Leung NG; Wai-Shing LEUNG; Wing Cheong YAM; Wing-Kin TO; Yuk-Yung NG
EPI_ISL_419231	Department of Clinical Pathology, Tuen Mun Hospital, 23 Tsing Chung Koon Road, Tuen Mun, N.T.	Department of Health Technology and Informatics, Faculty of Health and Social Science, The Hong Kong Polytechnic University	Alan Ka-Lun WU; Alex Yat-Man HO; Barry Kin-Chung WONG; David Ho-Keung SHUM; Eugene Yuk-Keung TSO; Gilman Kit-Hang SIU; Hiu-Yin LAO; Kam-Tong YIP; Kenneth Siu-Sing LEUNG; Kingsley King-Gee TAM; Kit-Man SIN; Kitty Sau-Chun FUNG; Kwok-Cheung LUNG; Lam-Kwong LEE; Man-Chun CHAN; Ming-Pan CHOI; Miranda Chong-Yee YAU; Raymond Wai-To LIU; Sandy Ka-Yee CHAU; Shea Ping YIP; Tak-Lun QUE; Timothy Ting-Leung NG; Wai-Shing LEUNG; Wing Cheong YAM; Wing-Kin TO; Yuk-Yung NG
EPI_ISL_481251, EPI_ISL_481254 to 481255, EPI_ISL_481257 to 481261, EPI_ISL_481263	Department of Emerging Infectious Diseases, Institute of Tropical Medicine, Nagasaki University	Department of Emerging Infectious Diseases, Institute of Tropical Medicine, Nagasaki University	Haruka Abe; Jiro Yasuda; Rokusuke Yoshikawa; Yuichiro Furusato
EPI_ISL_507206 to 507215, EPI_ISL_605792 to 605796	Department of Experimental Modeling and Pathogenesis of Infectious Diseases	WHO National Influenza Centre Russian Federation	Alekseev A.Yu.; Andrey Komissarov; Anna Ivanova; Artem Fadeev; Chepurnov A.A.; Daria Danilenko; Kononova Yu.V.; Kseniya Komissarova; Mariia Sergeeva; Shestopalov A.M.; Sobolev I.A.
EPI_ISL_610165 to 610175, EPI_ISL_610177, EPI_ISL_610179 to 610192, EPI_ISL_610194, EPI_ISL_610198 to 610199, EPI_ISL_610201 to 610215, EPI_ISL_610217 to 610225			
see above	Department of Health Technology and Informatics, The Hong Kong Polytechnic University	Department of Health Technology and Informatics, The Hong Kong Polytechnic University	A.K.-L.; A.Y.-M.; B.K.-C.; C.T.-M.; Chan; Chau; D.H.-K.; Fung; G.K.-H.; H.-Y.; Ho; J.S.-L.; K.-T.; K.K.-G.; K.S.-C.; K.S.-S.; L.-K.; Lai; Lao; Lee; Leung; Luk, K.; M.C.-Y.; Ng; Que; S.K.-Y.; S.P.; Shum; Siu; T.-L.; T.T.-L.; Tam; To; W.-K.; W.C.; Wong; Wu; Y.W.-M.; Yam; Yau; Yip
EPI_ISL_1019678, EPI_ISL_1020214 to 1020215	Department of Health Technology and Informatics, The Hong Kong Polytechnic University	Department of Health Technology and Informatics, The Hong Kong Polytechnic University	Alan Ka-Lun Wu; Alex Yat-Man Ho; Barry Kin-Chung Wong; Chloe Toi-Mei Chan; David Ho-Keung Shum; Denise Sze-Hang Wong; Gilman Kit-Hang Siu; Hiu-Yin Lao; Jake Siu-Lun Leung; Kam-Tong Yip; Kenneth Siu-Sing Leung; Kingsley King-Gee Tam; Kitty Sau-Chun Fung; Kristine Luk; Lam-Kwong Lee; Miranda Chong-Yee Yau; Sandy Ka-Yee Chau; Shea Ping Yip; Tak-Lun Que; Timothy Ting-Leung Ng; Wing Cheong Yam; Wing-Kin To; Yvette Wai-Man Lai
EPI_ISL_417444	Department of Healthcare Biotechnology, National University of Sciences and Technology (NUST)	Department of Healthcare Biotechnology, National University of Sciences and Technology (NUST)	Corman; Ghani, E.; H.A.; Janjua; Javed, A.; Niazi; S.K.; Saqib, M.; V.M. and Zohaib, A.
EPI_ISL_632964, EPI_ISL_1017989	Department of Homeless Services	New York City Public Health Laboratory	Jade Wang; et al.
EPI_ISL_529151 to 529154, EPI_ISL_529162, EPI_ISL_529164 to 529166	Department of Immunology, The Scripps Research Institute	Andersen lab at Scripps Research	Allison Smither; Antoinette Bell; Arnaud Drouin; Austin; B. with SEARCH Alliance San Diego; Basler, T.; Dahlene Fusco; Gilberto Sabino-Santos; I. with SEARCH Alliance San Diego; Kaylynn Genemaras; Lilia Melnik; Mchardy; Patricia Snarski; Quigley, M.; Robert Garry with SEARCH Alliance San Diego; Shephard, J.; Stefanski, E.
EPI_ISL_524435	Department of Immunology, The Scripps Research Institute	Department of Immunology, The Scripps Research Institute	J.H.; Pride, D.; S.D.; SEARCH Alliance; Shin
EPI_ISL_522407	Department of Infection Prevention and Infectious Diseases, University Hospital Regensburg	Department of Infection Prevention and Infectious Diseases, University Hospital Regensburg	Fritsch, J.; Holzmann, T.; Schneider-Brachert, W.
EPI_ISL_513298 to 513307	Department of Infection Prevention and Infectious Diseases, University Hospital Regensburg	University Hospital Regensburg	Fritsch, J.; Holzmann, T.; Schneider-Brachert, W.
EPI_ISL_605929 to 605930	Department of Infectious Disease Prevention and Control, Henan Provincial Center for Disease Control and Prevention	Department of Infectious Disease Prevention and Control, Henan Provincial Center for Disease Control and Prevention	Guo, W.; Hu, X.; Huang, X.; Li, D.; Li, X.; Lu, S.; Wu, B.; Ye, Y.
EPI_ISL_568556 to 568576, EPI_ISL_636989 to 637016, EPI_ISL_637018 to 637021, EPI_ISL_637075	Department of Infectious Diseases and Immunology, National Hospital Organization Nagoya Medical Center	Clinical Research Center, National Hospital Organization Nagoya Medical Center	Hirotaka Ode; Kazuhiro Matsuoka; Mai Kubota; Masakazu Matsuda; Mayumi Imahashi; Miho Nakasuji; Mikiko Mori; Nakasuji Miho; Yasumasa Iwatani; Yoshihiro Nakata; Yoshiyuki Yokomaku
EPI_ISL_524474, EPI_ISL_524480 to 524481	Department of Infectious Diseases, Cantonal Hospital Baden	Institute of Medical Virology, University of Zurich	Alexandra Trkola; Andrea Zbinden; Fiona Steiner; Gabriela Ziltener; Jon Huder; Jürg Böni; Maryam Zaheri; Michael Huber; Patrick Redli; Riccarda Capaul; Stefan Schmutz; Verena Kufner
EPI_ISL_457699 to 457700, EPI_ISL_457721, EPI_ISL_457724, EPI_ISL_457728, EPI_ISL_457732, EPI_ISL_457736, EPI_ISL_457749			
see above	Department of Infectious Diseases, Istituto Superiore di Sanità, Roma , Italy	Army Medical and Veterinary Research Center	Alessandra Lo Presti; Anna Anselmo; Antonella Fortunato; Antonella Marchi; Concetta Fabiani Silvia Fillo; Concetta Fabiani Silvia Fillo; Eleonora Benedetti; Florigio Lista; Francesco Giordani; Giovanni Faggioni; Nino D'Amore; Paola Stefanelli; Riccardo De Sanctis; Stefano Fiore; Vanessa Vera Fain
EPI_ISL_412973	Department of Infectious Diseases, Istituto Superiore di Sanità, Roma , Italy	Virology Laboratory, Scientific Department, Army Medical Center	Andrea Ciammaruconi; Anna Anselmo; Antonella Fortunato; Antonella Marchi; Concetta Fabiani; Eleonora Benedetti; Florigio Lista; Giovanni Faggioni; Paola Stefanelli; Riccardo De Santis; Silvia Fillo; Stefano Fiore; Stefano Palomba
EPI_ISL_856906	Department of Infectious Diseases, Istituto Superiore di Sanità, Roma, Italy; AO S.M. della Misericordia, S.C. Microbiologia, Perugia, Italy	Virology Laboratory, Scientific Department, Army Medical Center	Alessandra Lo Presti; Angela Di Martino; Anna Anselmo; Antonella Fortunato; Barbara Camilloni; Florigio Lista; Francesco Giordani; Giovanni Faggioni; Nino D'Amore; Paola Stefanelli; Riccardo De Sanctis; Silvia Fillo; Stefano Fiore; Vanessa Vera Fain
EPI_ISL_412974	Department of Infectious Diseases, Istituto Superiore di Sanità, Rome, Italy	Virology Laboratory, Scientific Department, Army Medical Center	Andrea Ciammaruconi; Antonella Fortunato; Antonella Marchi; Concetta Fabiani; Eleonora Benedetti; Filippo Molinari; Florigio Lista; Giancarlo Petralito; Giovanni Faggioni; Paola Stefanelli; Riccardo De Santis; Silvia Fillo; Stefano Fiore
EPI_ISL_1035901, EPI_ISL_1035966	Department of Infectious Diseases, Istituto Superiore di Sanità, Rome, Italy; Università degli Studi di Siena, Siena, Italy	Istituto Superiore di Sanità (ISS)	Alessandra Lo Presti; Angela Di Martino; Claudia Gandolfo; Gabriele Anichini; Gianni Gori Savellini; Manuela Marra; Marco Crescenzi; Maria Carollo; Maria Grazia Cusi; Paola Stefanelli; Stefano Fiore
EPI_ISL_644945 to 644999	Department of Infectious Diseases, Keio University School of Medicine, Tokyo, Japan	Center for Medical Genetics, Keio University School of Medicine, Tokyo, Japan	Haruhiko Siomi; Hirotsugu Ishizu; Kenjiro Kosaki; Kodai Abe; Yuka Iwasaki
EPI_ISL_479821 to 479822, EPI_ISL_479855 to 479861, EPI_ISL_479868, EPI_ISL_479986 to 479989, EPI_ISL_480090 to 480095, EPI_ISL_480097 to 480102, EPI_ISL_480205 to 480207, EPI_ISL_480209 to 480220			
see above	Department of Infectious Diseases, Kobe Institute of Health	Pathogen Genomics Center, National Institute of Infectious Diseases	Hajime Kamiya; Kentaro Itokawa; Makoto Kuroda; Masanori Hashino; Motoi Suzuki; Rina Tanaka; Ryohei Nomoto; Tsuyoshi Sekizuka

EPI_ISL_411218 to 411220	Department of Infectious and Tropical Diseases, Bichat Claude Bernard Hospital, Paris	Laboratoire Virpath, CIRI U111, UCBL1, INSERM, CNRS, ENS Lyon	Alexandre Gaymard; Aurélien Traversier; Bruno Lina; Catherine Legras-Lachuer; Julien Fouret; Manuel Rosa-Calatrava; Olivier Terrier; Xavier Lescure; Yazdan Yazdanpanah
EPI_ISL_406596 to 406597, EPI_ISL_408430, EPI_ISL_410720, EPI_ISL_410984	Department of Infectious and Tropical Diseases, Bichat Claude Bernard Hospital, Paris	National Reference Center for Viruses of Respiratory Infections, Institut Pasteur, Paris	Angela Brisebarre; Flora Donati; Marion Barbet; Maud Vanpeene; Mélanie Albert; Méline Bizard; Sylvie Behillil; Sylvie van der Werf; Vincent Enouf; Xavier Lescure; Xavier Lescure.; Yazdan Yazdanpanah
EPI_ISL_485398	Department of Internal Medicine, College of Medicine, Chosun University	Department of Internal Medicine, College of Medicine, Chosun University	D.-M.; Kim
EPI_ISL_413019 to 413020	Department of Internal Medicine, Triemli Hospital	Institute of Medical Virology, University of Zurich	Alexandra Trkola; Andrea Zbinden; Fiona Steiner; Gerhard Eich; Jon Huder; Jürg Böni; Maryam Zaheri; Michael Huber; Patrick Redli; Riccarda Capaul; Stefan Schmutz; Verena Kufner
EPI_ISL_477172, EPI_ISL_477174 to 477175, EPI_ISL_477177 to 477178, EPI_ISL_477180, EPI_ISL_477182, EPI_ISL_477184, EPI_ISL_477187 to 477192, EPI_ISL_479482 to 479492, EPI_ISL_492978 to 492979			
see above	Department of Laboratory Medicine Tan Tock Seng Hospital	Department of Laboratory Medicine Tan Tock Seng Hospital	Barkham TMS; Chen YYC; Li C; Maurer-Stroh S; Nagarajan N; Sessions OM; Tang WY; Zair X
EPI_ISL_516801 to 516805	Department of Laboratory Medicine, Tan Tock Seng Hospital	Department of Laboratory Medicine, Tan Tock Seng Hospital	Barkham TMS; Chen YYC; Li C; Maurer-Stroh S; Nagarajan N; Sessions OM; Tang WY; Zair X
EPI_ISL_934554 to 934555, EPI_ISL_934568 to 934569, EPI_ISL_934655, EPI_ISL_934657 to 934661, EPI_ISL_934663 to 934666, EPI_ISL_934668 to 934695, EPI_ISL_1008233, EPI_ISL_1008244 to 1008245, EPI_ISL_1008247, EPI_ISL_1008258, EPI_ISL_1008333, EPI_ISL_1008343, EPI_ISL_1008345 to 1008346, EPI_ISL_1008348 to 1008353, EPI_ISL_1008355 to 1008356			
see above	Department of Laboratory Medicine, Division of Clinical Virology, University of Medicine, Vienna	Bergthaler laboratory, CeMM Research Center for Molecular Medicine of the Austrian Academy of Sciences	Andreas Bergthaler; Anna Schedl; Bekir Erguner; Benedikt Agerer; Christoph Bock; Jan Laine; Lukas Endler; Maelle Le Moing; Martin Senekowitsch; Michael Schuster; Thomas Penz
EPI_ISL_408489, EPI_ISL_410218, EPI_ISL_413592, EPI_ISL_422407 to 422422, EPI_ISL_447614 to 447622, EPI_ISL_463007, EPI_ISL_534336, EPI_ISL_693302 to 693306, EPI_ISL_738064 to 738065, EPI_ISL_740547, EPI_ISL_1010728, EPI_ISL_1039160			
see above	Department of Laboratory Medicine, National Taiwan University Hospital	Microbial Genomics Core Lab, National Taiwan University Centers of Genomic and Precision Medicine	Chiao-Ling Li; Pei-Jer Chen; Shan-Chwen Chang; Shiou-Hwei Yeh; Sui-Yuan Chang; Ya-Yun Lai; You-Yu Lin
EPI_ISL_476814, EPI_ISL_476818 to 476819, EPI_ISL_507002 to 507006, EPI_ISL_538435 to 538497, EPI_ISL_648043 to 648121, EPI_ISL_648610 to 648676, EPI_ISL_648678 to 648685, EPI_ISL_648687 to 648820			
see above	Department of Laboratory Medicine, Tan Tock Seng Hospital	Department of Laboratory Medicine, Tan Tock Seng Hospital	Barkham TMS; Chen YYC; Li C; Lim JX; Maurer-Stroh S; Nagarajan N; Sessions OM; Tang WY; Zair X
EPI_ISL_477170	Department of Laboratory, Medicine Tan Tock Seng Hospital	Department of Laboratory Medicine Tan Tock Seng Hospital	Barkham TMS; Chen YYC; Li C; Maurer-Stroh S; Nagarajan N; Sessions OM; Tang WY; Zair X
EPI_ISL_477171	Department of Laboratory, Medicine Tan Tock Seng Hospital	Department of Laboratory, Medicine Tan Tock Seng Hospital	Barkham TMS; Chen YYC; Li C; Maurer-Stroh S; Nagarajan N; Sessions OM; Tang WY; Zair X
EPI_ISL_710483 to 710484	Department of Medical Laboratory Sciences, Arab American University	Department of Medical Laboratory Sciences, Arab American University	Al-Jawabreh, A.; Al-Jawabreh, H.; Dumaidi, K.; Ereqat, S.; Nasereddin, A.
EPI_ISL_591012, EPI_ISL_591018 to 591019	Department of Medical Microbiology - section Molde, Molde Hospital	Norwegian Institute of Public Health, Department of Virology	Hilde Elshaug; Hilde Vollan; Kamilla Heddeland Instefjord; Karoline Bragstad; Kathrine Stene-Johansen; Olav Hungnes; Rasmus Riis Kopperud
EPI_ISL_454416	Department of Medical Microbiology, Leiden University Medical Center	Department of Medical Microbiology, Leiden University Medical Center	Dalebout; E.J.; J.C.; J.J. and Sidorov, I.; N.S.; Ogando; Snijder; T.J.; Zevenhoven; de Vries
EPI_ISL_591007, EPI_ISL_591014, EPI_ISL_635096, EPI_ISL_635100, EPI_ISL_635110 to 635111, EPI_ISL_635126, EPI_ISL_635135 to 635136, EPI_ISL_635165, EPI_ISL_668417 to 668418, EPI_ISL_668420 to 668421, EPI_ISL_668441, EPI_ISL_708042 to 708043, EPI_ISL_708106 to 708109, EPI_ISL_708143 to 708146, EPI_ISL_759969 to 759972, EPI_ISL_1034309			
see above	Department of Medical Microbiology, St. Olavs hospital	Norwegian Institute of Public Health, Department of Virology	Atiya R Ali; Hilde Elshaug; Hilde Vollan; Ignacio Garcia Llorente; Kamilla Heddeland Instefjord; Karoline Bragstad; Kathrine Stene-Johansen; Marie Paulsen Madsen; Olav Hungnes; Rasmus Riis Kopperud; Serina B Engebretsen
EPI_ISL_417917	Department of Medical Microbiology, University Malaya Medical Centre	Department of Medical Microbiology	Adeeba KAMARULZAMAN; Chee Kuan WONG; Cindy Shuan Ju TEH; I-Ching SAM; Sasheela PONNAMPALAVANAR; Sharifah Faridah SYED OMAR; University Malaya Medical Centre COVID Team; Vijayan MUNUSAMY; Yoke Fun Chan; Yoong Min CHONG
EPI_ISL_417918, EPI_ISL_501176 to 501228, EPI_ISL_506996	Department of Medical Microbiology, University Malaya Medical Centre	Department of Medical Microbiology, Faculty of Medicine, University of Malaya	Adeeba KAMARULZAMAN; Chee Kuan WONG; Cindy Shuan Ju TEH; I-Ching SAM; Jennifer Chong; Sasheela PONNAMPALAVANAR; Sharifah Faridah SYED OMAR; University Malaya Medical Centre COVID Team; Vijayan MUNUSAMY; Yoke Fun CHAN; Yoke Fun Chan; Yoong Min CHONG
EPI_ISL_488840, EPI_ISL_488845, EPI_ISL_488854, EPI_ISL_488856, EPI_ISL_488861, EPI_ISL_488863, EPI_ISL_488872, EPI_ISL_492836, EPI_ISL_492838 to 492839, EPI_ISL_492841, EPI_ISL_492848 to 492849, EPI_ISL_492853 to 492854, EPI_ISL_492857, EPI_ISL_492860, EPI_ISL_492866 to 492867, EPI_ISL_492873, EPI_ISL_492881, EPI_ISL_492884, EPI_ISL_492886, EPI_ISL_492893, EPI_ISL_492895 to 492896, EPI_ISL_492898 to 492900, EPI_ISL_492903, EPI_ISL_492909 to 492910, EPI_ISL_492912 to 492913, EPI_ISL_492915, EPI_ISL_495081 to 495082, EPI_ISL_495084, EPI_ISL_495086, EPI_ISL_495090, EPI_ISL_495093, EPI_ISL_495095 to 495096, EPI_ISL_501602, EPI_ISL_501613			
see above	Department of Medical Microbiology, Western Sussex Hospitals NHS Foundation Trust, St Richard's Hospital	Wellcome Sanger Institute for the COVID-19 Genomics UK (COG-UK) consortium	Cordelia Langford; David K. Jackson; Dominic Kwiatkowski; Ewan Harrison; Ian Johnston; John Sillitoe on behalf of the Wellcome Sanger Institute COVID-19 Surveillance Team (http://www.sanger.ac.uk/covid-team); Jonathan Lewis; Manasa Mutingwende; Michelle Erkiert; Olga Podplomyk; Paul Randell and Alex Alderton; Roberto Amato; Sarah Lowdon; Sonia Goncalves
EPI_ISL_512844	Department of Medical Research	DMR_Myanmar	Aung Kyaw Kyaw; Aung Zaw Latt; Hlaing Myat Thu; Hnin Ohnmar Soe; Htin Lin; Kay Thi Aye; Lai Lai San; Myat Htut Nyunt; Nan Aye Thida Oo; Ni Ni Zaw; Phyu Win Ei; Su Mon Win; Theingi Win Myat; Wah Wah Aung; Yi Yi Kyaw; Zaw Than Htun
EPI_ISL_454733	Department of Medical, Biotechnologies University of Siena	Department of Medical, Biotechnologies University of Siena	Anichini, G.; Cusi; G. and Santoro, F.; Gandolfo, C.; M.G.; Pinzauti, D.; Pozzi
EPI_ISL_528809 to 528822	Department of Medicine, Gandhi hospital, Hyderabad	CSIR-Centre for Cellular and Molecular Biology	Ajay Sarawagi; Archana Bharadwaj Siva; Dhiviya Vedagiri; Divya Gupta; Divya Tej Sowpati; G. Aditya Kumar; Gangumala Srinivas Reddy; Karthik Bharadwaj Tallapaka; Koushick Sivakumar; Krishnan Harinivas Harshan; Lamuk Zaveri; M Soujanya Reddy; Namami Gaur; Nikhil Hajirnis; Onkar Kulkarni; Payel Mukherjee; Pooja Ramesh Gupta; Pratheusa Maccha; Priya Singh; Priyanka Pant; Purushotham Vodnala; Rajan Kumar Jha; Rajarao Mesipogu; Rajkanwar Nathawat; Rakesh K Mishra; Renu Sudhakar; Sakshi Shambhavi; Santosh Kumar Kuncha; Shagufta Khan; Shraddha Vijay Lahoti; Sofia Banu; Somesh Gorde; Sujoy Deb; Swati Bayyana; Thrilok Chander Bingi; Tulasi Nagabandi; Umesh Kumar; Unis Ahmad Bhat; Vinayasekhar Aedula; Vishal Sah
EPI_ISL_437438, EPI_ISL_437440 to 437442, EPI_ISL_437444, EPI_ISL_475030 to 475040, EPI_ISL_475043 to 475046, EPI_ISL_476869 to 476882, EPI_ISL_477183, EPI_ISL_495070 to 495080, EPI_ISL_500947 to 500950, EPI_ISL_512070 to 512071			
see above	Department of MicroBiology, Government Medical College, Surat	Gujarat Biotechnology Research Centre	A M Kadri; Afzal Ansari; Akanksha Verma; Amit Kanani; Amit gamit; Anjali Rajwar; Ankit Hinsu; Apurvasinh Puvar; Armi Chaudhari; Bhavesh Modi; Bhavya Jindal; Binita Aring; Chaitanya Joshi; Dinesh Kumar; Dipa Kinariwala; Dipeshwari Shewale; Disha Patel; Fenil Patel; Gaurishankar Shrimali; Geeta Vaghela; Harsh Bakshi; Janvi Raval; Kairavi Joshi; Kamlesh J Upadhyay; Komal Patel; Labdhi Pandya; Madhvi Joshi; Maharshi Pandya; Monika Gandhi; Naresh Chauhan; Neelam Nathani; Neeta Khandelwal; Neha Rajpara; Nidhi Patel; Nidhi Sood; Nikha Trivedi; Nitin Savaliya; Pinal Trivedi; Pooja P Doshi; Pragya Sharma; Pranay Shah; Pritesh Sabara; Priti Pandita; Priyanka P Vatsa; R D Dixit; Raghawendra Kumar; Ramesh Pandit; Snehal Bagatharia; Sonia Barve; Summaiya Mullan; Tejas Shah; Zarna Patel; Zuber Saiyed
EPI_ISL_431102	Department of MicroBiology,Gandhi Medical College and Hospital,Secendrabad,Hyderabad,India	Department of Microbiology, Gandhi Medical College and Hospital, Secendrabad, Hyderabad	Amit A. Upadhyay; Anand Kumar K; Kalyani Putty; Muttineni Radhakrishna; Nagamani K; Pankaj Singh D; Raja Rao M; Rama Amara; Ravikumar P; Steven E. Bosinger; Sunitha P; Thrilok Chander B
EPI_ISL_507039	Department of Microbiology, College of Medicine and Medical Research Institute Chungbuk National University	Department of Microbiology, College of Medicine and Medical Research Institute Chungbuk National University	Eun-Ha Kim; Hye Won Jeong; Mark Anthony B. Casel; Se-Mi Kim; Seong-Gyu Kim; Su-Jin Park; Young Ki Choi; Young-Il Kim
EPI_ISL_416314	Department of Microbiology, Faculty of Medicine, The Chinese University of Hong Kong, Hong Kong SAR, China	Department of Microbiology, Faculty of Medicine, Chinese University of Hong Kong, Hong Kong SAR, China	Paul KS Chan; Zigui Chen
EPI_ISL_431103, EPI_ISL_431117	Department of Microbiology, Gandhi Medical College and Hospital, Secendrabad, Hyderabad, India	Department of Microbiology, Gandhi Medical College and Hospital, Secendrabad, Hyderabad, India	Amit A. Upadhyay; Anand Kumar K; Kalyani Putty; Muttineni Radhakrishna; Nagamani K; Pankaj Singh D; Raja Rao M; Rama Amara; Ravikumar P; Steven E. Bosinger; Steven E.Bosinger; Sunitha P; Thrilok Chander B
EPI_ISL_483850 to 483879	Department of Microbiology, Government Medical College, Surat	Gujarat Biotechnology Research Centre	A M Kadri; Afzal Ansari; Amit gamit; Apurvasinh Puvar; Chaitanya Joshi; Dinesh Kumar; Harsh Bakshi; Janvi Raval; Komal Patel; Labdhi Pandya; Madhvi Joshi; Maharshi Pandya; Monika Gandhi; Naresh Chauhan; Nidhi Patel; Nikha Trivedi; Nitin Savaliya; Pinal Trivedi; R D Dixit; Raghawendra Kumar; Summaiya Mullan; Zarna Patel; Zuber Saiyed
EPI_ISL_413017 to 413018, EPI_ISL_413514, EPI_ISL_413516	Department of Microbiology, Institute for Viral Diseases, College of Medicine, Korea University	Department of Microbiology, Institute for Viral Diseases, College of Medicine, Korea University	Changmin Kang; Cui Chunguang; Dong Min Kim; Gee Eun Lee; Gee eun Lee; Hee Jin Cheong; Heedo Park; Jeonghun Kim; Ji Yun Noh; Jin Gu Yoon; Jin Il Kim; Joon Young Song; Joon-Yong Bae; Jungmin Lee; Juyoung Cho; Kyeong-ryeol Shin; Man-Seong Park; Woo Joo Kim

EPI_ISL_1027645 to 1027646, EPI_ISL_1027649	Department of Microbiology, National Institute for Public Health of Kosova	Charité Universitätsmedizin Berlin, Institut für Virologie	Barbara Mühlemann; Christian Drosten; Donjeta Hajdari; Julia Schneider; Jörn Beheim-Schwarzbach; Talitha Veith; Terry Jones; Victor M Corman; Xhevat Jakupi; Zana Deva
EPI_ISL_420531 to 420534, EPI_ISL_420536 to 420539	Department of Microbiology, PathWest QEII Medical Centre	Department of Microbiology, PathWest QEII Medical Centre	Avram Levy; Chisha Sikazwe; David Speers and David Smith; Jurissa Lang
EPI_ISL_434560, EPI_ISL_497769 to 497771, EPI_ISL_497773, EPI_ISL_497775 to 497778, EPI_ISL_497780, EPI_ISL_497782 to 497786, EPI_ISL_497790 to 497791, EPI_ISL_497793 to 497801, EPI_ISL_497803, EPI_ISL_497806 to 497827, EPI_ISL_497829 to 497833, EPI_ISL_497835 to 497836, EPI_ISL_497840 to 497842, EPI_ISL_497844 to 497851, EPI_ISL_497853 to 497857, EPI_ISL_497860 to 497870, EPI_ISL_498270 to 498271, EPI_ISL_510538 to 510539, EPI_ISL_516798 to 516799			
see above	Department of Microbiology, The University of Hong Kong	Department of Microbiology, The University of Hong Kong	A.C.P.; Chan; Fung; Fung, J.; H.K.H.; He, Z.; K.S.C. and Woo; K.S.M.; Kelvin K.W. To; Kwok-Yung Yuen; Lau; Li; Luk; P.C.Y.; S.K.P.; T.T.Y.; Wong; Zhu, L.
EPI_ISL_693298 to 693299	Department of Microbiology, Yokohama City University School of Medicine	Department of Microbiology, Yokohama City University School of Medicine	Akihide Ryo; Ichiro Takeuchi; Kazuya Sakai; Kei Miyakawa; Mototsugu Nishii; Reo Matsumura; Ryo Saji
EPI_ISL_438138	Department of Microbiology,Gandhi Medical College and Hospital	Department of Microbiology, Gandhi Medical College and Hospital Secendrabad, Hyderbad, India	Amit A. Upadhyay; Anand Kumar K; Kalyani Putty; Muttineni Radhakrishna; Nagamani K; Pankaj Singh D; Raja Rao Mesipogu; Rama Amara; Ravikumar P; Steven Bosinger; Sunitha P; Thrilok Chander B
EPI_ISL_437626	Department of Microbiology,Gandhi Medical College and Hospital	Department of Veterinary Biotechnalogy, College of Veterinary Science, Rajendranagar, PV Narsimha Rao Telengana Veterinary University	Amit A. Upadhyay; Anand Kumar K; Kalyani Putty; Muttineni Radhakrishna; Nagamani K; Pankaj Singh D; Raja Rao M; Rama Amara; Ravikumar P; Steven Bosinger; Sunitha P; Thrilok Chander B
EPI_ISL_431101	Department of Microbiology,Gandhi Medical College and Hospital	Virus Research Laboratory, Department of Zoology, Osmania University,Hyderabad,India	Amit A. Upadhyay Steven E. Bosinger; Anand Kumar K; Kalyani Putty; Muttineni Radhakrishna; Nagamani K; Pankaj Singh D; Raja Rao M; Rama Amara; Ravikumar P; Sunitha P; Thrilok Chander B
EPI_ISL_438139	Department of Microbiology,Gandhi Medical College and Hospital,Hyderabad	Virus Research Laboratory, Department of Zoology, Osmania University, Hyderabad, India	Amit A. Upadhyay; Anand Kumar K; Kalyani Putty; Muttineni Radhakrishna; Nagamani K; Pankaj Singh D; Raja Rao M; Rama Amara; Ravikumar P; Steven Bosinger; Sunitha P; Thrilok Chander B
EPI_ISL_434561 to 434562	Department of Microbiology; Ryota Kumagai Tokyo Metropolitan Institute of Public Health	Department of Microbiology; Ryota Kumagai Tokyo Metropolitan Institute of Public Health	Asakura, H.; Chiba; Kumagai, R.; Nagashima, M.; T. and Sadamasu, K.; Yoshida, I.
EPI_ISL_414511	Department of Microbiology; Ryota Kumagai Tokyo Metropolitan Institute of Public Health	Tokyo Metoropolitan Institute of Public Health	Chiba; Kumagai, R.; Nagashima, M.; T. and Sadamasu, K.; Yoshida, I.
EPI_ISL_463741 to 463748	Department of Molecular Virology, Cyprus Institute of Neurology and Genetics	Department of Molecular Virology, Cyprus Institute of Neurology and Genetics	Christina Christodoulou; Christina Tryfonos; Dana Koptides; George Krashias; Jan Richter; Stavros Bashiardes
EPI_ISL_417176, EPI_ISL_417178, EPI_ISL_419217, EPI_ISL_419222	Department of Pathology, Princess Margaret Hospital	Department of Health Technology and Informatics, Faculty of Health and Social Science, The Hong Kong Polytechnic University	Alan Ka-Lun WU; Alex Yat-Man HO; Barry Kin-Chung WONG; David Ho-Keung SHUM; Eugene Yuk-Keung TSO; Gilman Kit-Hang SIU; Hiu-Yin LAO; Kam-Tong YIP; Kam-Tong Yip; Kenneth Siu-Sing LEUNG; Kingsley King-Gee TAM; Kit-Man SIN; Kitty Sau-Chun FUNG; Kwok-Cheung LUNG; Lam-Kwong LEE; Man-Chun CHAN; Ming-Pan CHOI; Miranda Chong-Yee YAU; Raymond Wai-To LIU; Sandy Ka-Yee CHAU; Shea Ping YIP; Tak-Lun QUE; Tak-Lun Que; Timothy Ting-Leung NG; Wai-Shing LEUNG; Wing Cheong YAM; Wing-Kin TO; Yuk-Yung NG
EPI_ISL_594185 to 594188, EPI_ISL_596451, EPI_ISL_596455	Department of Pathology, School of Medicine, Imam Khomeini Hospital, Tehran University of Medical Sciences	Genetics Research Center, University of Social Welfare and Rehabilitation Sciences	Ali Jafarpour; Alireza Abdollahi; Azam Ghaziasad; Azam Ghaziasadi; Azar Hadadi; Hossein Najmabadi; Khadijeh Jalalvand; Kimia Kahrizi; Marzieh Mohseni; Reza Najafipour; Saber Soltani; Seyed Mohammad Jazayeri; Seyedeh elham Mortazavi; Zohreh Fattahi
EPI_ISL_582033	Department of Pathology, School of Medicine, Imam Khomeini Hospital, Tehran University of Medical Sciences	Genetics Research Center. University Of Social Welfare And Rehabilitation Sciences	Ali Jafarpour; Alireza Abdollahi; Azam Ghaziasad; Azam Ghaziasadi; Azar Hadadi; Hossein Najmabadi; Khadijeh Jalalvand; Kimia Kahrizi; Marzieh Mohseni; Reza Najafipour; Saber Soltani; Seyed Mohammad Jazayeri; Seyedeh elham Mortazavi; Zohreh Fattahi
EPI_ISL_413459	Department of Pathology, Toshima Hospital	Pathogen Genomics Center, National Institute of Infectious Diseases	Haruka Nishioka; Harutaka Katano; Ippei Miyamoto; Ja-Mun Chong; Jun Yamazaki; Kentaro Itokawa; Makoto Kuroda; Masahiro Sano; Minoru Tobiume; Noriko Nakajima; Tadaki Suzuki; Takuya Adachi; Tsuyoshi Sekizuka; Yuko Sato
EPI_ISL_417181, EPI_ISL_417185, EPI_ISL_419221	Department of Pathology, United Christian Hospital	Department of Health Technology and Informatics, Faculty of Health and Social Science, The Hong Kong Polytechnic University	Alan Ka-Lun WU; Alex Yat-Man HO; Barry Kin-Chung WONG; David Ho-Keung SHUM; Eugene Yuk-Keung TSO; Gilman Kit-Hang SIU; Hiu-Yin LAO; Kam-Tong YIP; Kam-Tong Yip; Kenneth Siu-Sing LEUNG; Kingsley King-Gee TAM; Kit-Man SIN; Kitty Sau-Chun FUNG; Kwok-Cheung LUNG; Lam-Kwong LEE; Man-Chun CHAN; Ming-Pan CHOI; Miranda Chong-Yee YAU; Raymond Wai-To LIU; Sandy Ka-Yee CHAU; Shea Ping YIP; Tak-Lun QUE; Tak-Lun Que; Timothy Ting-Leung NG; Wai-Shing LEUNG; Wing Cheong YAM; Wing-Kin TO; Yuk-Yung NG
EPI_ISL_425231 to 425240, EPI_ISL_425242 to 425244, EPI_ISL_425246, EPI_ISL_425248 to 425253, EPI_ISL_425255, EPI_ISL_425257 to 425264, EPI_ISL_425266 to 425270, EPI_ISL_425273, EPI_ISL_425275 to 425278, EPI_ISL_425280 to 425281, EPI_ISL_425283, EPI_ISL_425286 to 425288, EPI_ISL_425290, EPI_ISL_425292 to 425296, EPI_ISL_425298, EPI_ISL_425300 to 425301, EPI_ISL_425303 to 425319, EPI_ISL_425321 to 425327, EPI_ISL_425331 to 425333, EPI_ISL_425335, EPI_ISL_425338 to 425339, EPI_ISL_425342, EPI_ISL_425348, EPI_ISL_425351 to 425352, EPI_ISL_425358 to 425359, EPI_ISL_425361 to 425362, EPI_ISL_425373 to 425374, EPI_ISL_425378, EPI_ISL_425380, EPI_ISL_425382, EPI_ISL_425390, EPI_ISL_425392 to 425393, EPI_ISL_425395, EPI_ISL_425398, EPI_ISL_425401 to 425405, EPI_ISL_425407, EPI_ISL_425409 to 425412, EPI_ISL_425423 to 425425, EPI_ISL_425427 to 425428, EPI_ISL_425430 to 425437, EPI_ISL_425439 to 425441, EPI_ISL_425443, EPI_ISL_425446, EPI_ISL_425449 to 425455, EPI_ISL_425457 to 425458, EPI_ISL_425460, EPI_ISL_433467, EPI_ISL_433469 to 433472, EPI_ISL_433478 to 433497, EPI_ISL_433666 to 433669, EPI_ISL_433671 to 433691, EPI_ISL_433693 to 433695, EPI_ISL_433697 to 433699, EPI_ISL_433701 to 433702, EPI_ISL_433704 to 433707, EPI_ISL_433709 to 433714, EPI_ISL_433716 to 433722, EPI_ISL_433724, EPI_ISL_433728 to 433753, EPI_ISL_433755, EPI_ISL_433757 to 433768, EPI_ISL_433770 to 433772, EPI_ISL_433775 to 433787, EPI_ISL_433789 to 433801, EPI_ISL_433803, EPI_ISL_433805 to 433809, EPI_ISL_433811 to 433821, EPI_ISL_433823 to 433829, EPI_ISL_433833 to 433835, EPI_ISL_433837 to 433840, EPI_ISL_433842 to 433844, EPI_ISL_433846 to 433849, EPI_ISL_433851 to 433852, EPI_ISL_433854 to 433866, EPI_ISL_433868, EPI_ISL_433870 to 433873, EPI_ISL_433875 to 433878, EPI_ISL_433880, EPI_ISL_433882 to 433886, EPI_ISL_433888, EPI_ISL_433890 to 433900, EPI_ISL_433902, EPI_ISL_433904 to 433907, EPI_ISL_433909 to 433911, EPI_ISL_433913 to 433915, EPI_ISL_433918 to 433920, EPI_ISL_433922 to 433923, EPI_ISL_433926 to 433931, EPI_ISL_433933 to 433940, EPI_ISL_433942, EPI_ISL_433944 to 433945, EPI_ISL_433948 to 433959, EPI_ISL_433961 to 433976, EPI_ISL_433982, EPI_ISL_433992, EPI_ISL_433999, EPI_ISL_434001 to 434002, EPI_ISL_434007, EPI_ISL_434009 to 434010, EPI_ISL_434012 to 434015, EPI_ISL_434020 to 434022, EPI_ISL_434024, EPI_ISL_434027, EPI_ISL_434029, EPI_ISL_434032, EPI_ISL_434034 to 434036, EPI_ISL_434038 to 434039, EPI_ISL_434041, EPI_ISL_434045 to 434047, EPI_ISL_434049, EPI_ISL_434051, EPI_ISL_434054, EPI_ISL_438550 to 438551, EPI_ISL_438554, EPI_ISL_438557 to 438568, EPI_ISL_438571 to 438581, EPI_ISL_438583, EPI_ISL_438587 to 438588, EPI_ISL_438590, EPI_ISL_438592 to 438594, EPI_ISL_438597, EPI_ISL_438599 to 438602, EPI_ISL_438604 to 438605, EPI_ISL_438608, EPI_ISL_438610, EPI_ISL_438618 to 438626, EPI_ISL_438628, EPI_ISL_438630 to 438633, EPI_ISL_438644 to 438645, EPI_ISL_438647, EPI_ISL_438649 to 438650, EPI_ISL_438652, EPI_ISL_438656, EPI_ISL_438658, EPI_ISL_438660, EPI_ISL_438662 to 438667, EPI_ISL_438669 to 438672, EPI_ISL_438676 to 438679, EPI_ISL_438681 to 438684, EPI_ISL_438686 to 438693, EPI_ISL_438696 to 438705, EPI_ISL_438707 to 438717, EPI_ISL_438719 to 438722, EPI_ISL_438725 to 438726, EPI_ISL_438729, EPI_ISL_438733, EPI_ISL_438735 to 438737, EPI_ISL_438740, EPI_ISL_438744 to 438748, EPI_ISL_444320, EPI_ISL_444323, EPI_ISL_444328 to 444329, EPI_ISL_444334 to 444340, EPI_ISL_444342 to 444343, EPI_ISL_444345 to 444349, EPI_ISL_444351 to 444366, EPI_ISL_444368, EPI_ISL_444370 to 444372, EPI_ISL_444374 to 444377, EPI_ISL_444384, EPI_ISL_444393 to 444395, EPI_ISL_444397 to 444398, EPI_ISL_444400, EPI_ISL_444402, EPI_ISL_444407, EPI_ISL_444413, EPI_ISL_444415 to 444423, EPI_ISL_444430, EPI_ISL_444432, EPI_ISL_444434 to 444443, EPI_ISL_444445 to 444453, EPI_ISL_447953 to 447956, EPI_ISL_447959 to 447960, EPI_ISL_447963 to 447964, EPI_ISL_447966 to 447967, EPI_ISL_447969 to 447973, EPI_ISL_447976 to 447981, EPI_ISL_447983 to 447990, EPI_ISL_447994 to 447995, EPI_ISL_447997 to 448001, EPI_ISL_448003, EPI_ISL_448007 to 448008, EPI_ISL_448010 to 448019, EPI_ISL_448021 to 448024, EPI_ISL_448028 to 448030, EPI_ISL_448033 to 448044, EPI_ISL_448046 to 448047, EPI_ISL_448049, EPI_ISL_448051, EPI_ISL_448053, EPI_ISL_448056 to 448057, EPI_ISL_448059 to 448060, EPI_ISL_448063 to 448066, EPI_ISL_448068, EPI_ISL_448072 to 448073, EPI_ISL_448077 to 448092, EPI_ISL_448095 to 448098, EPI_ISL_448100 to 448103, EPI_ISL_448105 to 448109, EPI_ISL_448111, EPI_ISL_448114, EPI_ISL_452857 to 452859, EPI_ISL_452862 to 452867, EPI_ISL_452869, EPI_ISL_452871 to 452872, EPI_ISL_452876 to 452882, EPI_ISL_452884 to 452886, EPI_ISL_452888 to 452898, EPI_ISL_452904, EPI_ISL_452906, EPI_ISL_452910, EPI_ISL_452912 to 452918, EPI_ISL_452920, EPI_ISL_452926, EPI_ISL_452928 to 452930, EPI_ISL_452932, EPI_ISL_452934, EPI_ISL_452936 to 452938, EPI_ISL_452940, EPI_ISL_452942, EPI_ISL_452944, EPI_ISL_452946 to 452950, EPI_ISL_452952 to 452963, EPI_ISL_452965, EPI_ISL_452968 to 452969, EPI_ISL_452971 to 452974, EPI_ISL_452977 to 452980, EPI_ISL_452982 to 452986, EPI_ISL_452989, EPI_ISL_452993 to 452994, EPI_ISL_452996, EPI_ISL_452998, EPI_ISL_453000 to 453001, EPI_ISL_453003 to 453005, EPI_ISL_456680, EPI_ISL_456688, EPI_ISL_456697, EPI_ISL_456699, EPI_ISL_456702, EPI_ISL_456704 to 456705, EPI_ISL_456707 to 456709, EPI_ISL_456711, EPI_ISL_456713, EPI_ISL_456716 to 456724, EPI_ISL_456727 to 456734, EPI_ISL_456736, EPI_ISL_456739 to 456740, EPI_ISL_456743, EPI_ISL_456745, EPI_ISL_456747, EPI_ISL_456749 to 456751, EPI_ISL_456754 to 456755, EPI_ISL_461546 to 461548, EPI_ISL_461552 to 461553, EPI_ISL_461561 to 461563, EPI_ISL_461571 to 461575, EPI_ISL_461578 to 461583, EPI_ISL_461586 to 461587, EPI_ISL_473454 to 473455, EPI_ISL_473459, EPI_ISL_473463 to 473464, EPI_ISL_473466, EPI_ISL_473471 to 473472, EPI_ISL_473474, EPI_ISL_473477, EPI_ISL_473479, EPI_ISL_473481 to 473484, EPI_ISL_473487 to 473493, EPI_ISL_473496, EPI_ISL_473498 to 473501, EPI_ISL_473503 to 473504, EPI_ISL_473507, EPI_ISL_477796, EPI_ISL_477798, EPI_ISL_477800, EPI_ISL_477802, EPI_ISL_477805 to 477814, EPI_ISL_483913 to 483915, EPI_ISL_490327 to 490329, EPI_ISL_499968 to 499973, EPI_ISL_512332 to 512333, EPI_ISL_514443, EPI_ISL_514445, EPI_ISL_514448 to 514451, EPI_ISL_516993, EPI_ISL_516995 to 516996, EPI_ISL_516999 to 517000, EPI_ISL_529224 to 529225, EPI_ISL_529236, EPI_ISL_529656 to 529660, EPI_ISL_549389 to 549395, EPI_ISL_572851 to 572852, EPI_ISL_572862 to 572868, EPI_ISL_573132 to 573151, EPI_ISL_573153, EPI_ISL_576897, EPI_ISL_576900 to 576901, EPI_ISL_576903, EPI_ISL_576906, EPI_ISL_576908, EPI_ISL_576910, EPI_ISL_576912, EPI_ISL_576914, EPI_ISL_576918, EPI_ISL_584285, EPI_ISL_584287, EPI_ISL_584293 to 584294, EPI_ISL_584296, EPI_ISL_584298, EPI_ISL_584300, EPI_ISL_584303 to 584305, EPI_ISL_584307, EPI_ISL_584310 to 584317, EPI_ISL_584319 to 584321, EPI_ISL_611964 to 611967, EPI_ISL_612123, EPI_ISL_612186, EPI_ISL_612261 to 612283, EPI_ISL_637856, EPI_ISL_637859 to 637860, EPI_ISL_638038 to 638040, EPI_ISL_638066 to 638072, EPI_ISL_638079 to 638083, EPI_ISL_638165 to 638167, EPI_ISL_638169, EPI_ISL_638174, EPI_ISL_638208 to 638248, EPI_ISL_638258 to 638264, EPI_ISL_638293 to 638448, EPI_ISL_638614 to 638681, EPI_ISL_638731 to 638735, EPI_ISL_638895 to 638910, EPI_ISL_638914 to 638927, EPI_ISL_638936 to 638943, EPI_ISL_639003 to 639005, EPI_ISL_639624 to 639625, EPI_ISL_651423, EPI_ISL_651435 to 651436, EPI_ISL_651542, EPI_ISL_651653 to 651714, EPI_ISL_652157 to 652179, EPI_ISL_652286 to 652288, EPI_ISL_664748 to 664751, EPI_ISL_664765, EPI_ISL_664941 to 665022, EPI_ISL_678834 to 678837, EPI_ISL_678840 to 678845, EPI_ISL_678849, EPI_ISL_678853 to 678854, EPI_ISL_678857 to 678860, EPI_ISL_678862 to 678865, EPI_ISL_678867, EPI_ISL_678869 to 678870, EPI_ISL_678872, EPI_ISL_678875 to 678877, EPI_ISL_678880 to 678882, EPI_ISL_678884 to 678885, EPI_ISL_678887, EPI_ISL_678889 to 678890, EPI_ISL_678892 to 678896, EPI_ISL_678898 to 678899, EPI_ISL_678901 to 678903, EPI_ISL_678905 to 678907, EPI_ISL_678909 to 678913, EPI_ISL_678915, EPI_ISL_679134, EPI_ISL_679137 to 679138, EPI_ISL_679143, EPI_ISL_679146 to 679147, EPI_ISL_679149, EPI_ISL_679152 to 679153, EPI_ISL_679155 to 679158, EPI_ISL_679161 to 679162, EPI_ISL_679164, EPI_ISL_679167 to 679170, EPI_ISL_679174, EPI_ISL_679178 to 679180, EPI_ISL_679182 to 679183, EPI_ISL_679186 to 679189, EPI_ISL_679191, EPI_ISL_679193, EPI_ISL_679196, EPI_ISL_679198, EPI_ISL_679214, EPI_ISL_679224, EPI_ISL_680039, EPI_ISL_680041, EPI_ISL_680043, EPI_ISL_680047 to 680049, EPI_ISL_680051, EPI_ISL_680053 to 680054, EPI_ISL_680056, EPI_ISL_680058, EPI_ISL_680060, EPI_ISL_680064, EPI_ISL_680066 to 680067, EPI_ISL_680070, EPI_ISL_680076, EPI_ISL_680078, EPI_ISL_680080, EPI_ISL_680082, EPI_ISL_680086, EPI_ISL_680093 to 680096, EPI_ISL_680098, EPI_ISL_680100, EPI_ISL_680571 to 680574, EPI_ISL_680576, EPI_ISL_705415, EPI_ISL_705529 to 705566, EPI_ISL_705629 to 705635, EPI_ISL_723853, EPI_ISL_723856 to 723858, EPI_ISL_723860, EPI_ISL_723863 to 723864, EPI_ISL_723870 to 723872, EPI_ISL_723874, EPI_ISL_723876 to 723877, EPI_ISL_723886, EPI_ISL_723888, EPI_ISL_723894 to 723897, EPI_ISL_723900, EPI_ISL_723903 to 723908, EPI_ISL_723910 to 723912, EPI_ISL_723917 to 723918, EPI_ISL_723920 to 723922, EPI_ISL_723924, EPI_ISL_723927 to 723928, EPI_ISL_723930 to 723932, EPI_ISL_723937, EPI_ISL_723939, EPI_ISL_723941, EPI_ISL_723944 to 723945, EPI_ISL_740954 to 740955, EPI_ISL_740957, EPI_ISL_740968 to 740969, EPI_ISL_740972, EPI_ISL_740977 to 740981, EPI_ISL_740984, EPI_ISL_740987 to 740989, EPI_ISL_740999 to 741001, EPI_ISL_741009, EPI_ISL_741011, EPI_ISL_741014, EPI_ISL_763843, EPI_ISL_763847 to 763850, EPI_ISL_763872 to 763891, EPI_ISL_764272, EPI_ISL_764284 to 764355, EPI_ISL_813171, EPI_ISL_813173 to 813175, EPI_ISL_813185, EPI_ISL_813192 to 813193, EPI_ISL_813197 to 813198, EPI_ISL_813200 to 813204, EPI_ISL_813206, EPI_ISL_813208 to 813214, EPI_ISL_813219, EPI_ISL_813221, EPI_ISL_813224 to 813229, EPI_ISL_813232, EPI_ISL_813236, EPI_ISL_813240, EPI_ISL_813242, EPI_ISL_813244 to 813246, EPI_ISL_813248 to 813249, EPI_ISL_813251, EPI_ISL_813254, EPI_ISL_813257, EPI_ISL_813263 to 813264, EPI_ISL_813267, EPI_ISL_813269, EPI_ISL_813271, EPI_ISL_813274, EPI_ISL_813276 to 813279, EPI_ISL_813285 to 813288, EPI_ISL_813290 to 813297, EPI_ISL_813301, EPI_ISL_813307, EPI_ISL_813313, EPI_ISL_813317, EPI_ISL_813319 to 813320, EPI_ISL_813323, EPI_ISL_813326, EPI_ISL_813328, EPI_ISL_813331 to 813332, EPI_ISL_813335, EPI_ISL_813342, EPI_ISL_813345, EPI_ISL_813347, EPI_ISL_813349, EPI_ISL_813353 to 813354, EPI_ISL_837837, EPI_ISL_837839, EPI_ISL_837843, EPI_ISL_837845, EPI_ISL_837847, EPI_ISL_837849 to 837850, EPI_ISL_837853 to 837854, EPI_ISL_837857 to 837858, EPI_ISL_837860 to 837866, EPI_ISL_837868 to 837869, EPI_ISL_837873, EPI_ISL_837876 to 837877, EPI_ISL_837880 to 837883, EPI_ISL_837885 to 837889, EPI_ISL_837892, EPI_ISL_837894 to 837895, EPI_ISL_837902 to 837903, EPI_ISL_837906 to 837907, EPI_ISL_837909 to 837910, EPI_ISL_837912, EPI_ISL_837915 to 837917, EPI_ISL_837919, EPI_ISL_837921 to 837930, EPI_ISL_837933 to 837938, EPI_ISL_837940, EPI_ISL_837942 to 837943, EPI_ISL_837945 to 837946, EPI_ISL_837948 to 837950, EPI_ISL_837953, EPI_ISL_837955, EPI_ISL_837957 to 837961, EPI_ISL_837963 to 837964, EPI_ISL_837967 to 837968, EPI_ISL_837970 to 837972, EPI_ISL_837974 to 837980, EPI_ISL_837982 to 837983, EPI_ISL_837987 to 837988, EPI_ISL_837990 to 837996, EPI_ISL_837998, EPI_ISL_838001, EPI_ISL_838003, EPI_ISL_838005 to 838007, EPI_ISL_838009 to 838013, EPI_ISL_838015 to 838016, EPI_ISL_838018 to 838019, EPI_ISL_838021 to 838024, EPI_ISL_838027 to 838029, EPI_ISL_838032 to 838034, EPI_ISL_838039, EPI_ISL_838044 to 838047,			

EPI_ISL_838049 to 838052, EPI_ISL_838054, EPI_ISL_864738 to 864739, EPI_ISL_864742 to 864746, EPI_ISL_864748, EPI_ISL_864750 to 864751, EPI_ISL_864753 to 864754, EPI_ISL_864756 to 864778, EPI_ISL_864780, EPI_ISL_864785 to 864788, EPI_ISL_864790 to 864794, EPI_ISL_864797, EPI_ISL_864801 to 864803, EPI_ISL_864805 to 864806, EPI_ISL_864808 to 864809, EPI_ISL_864811, EPI_ISL_864814 to 864815, EPI_ISL_864818 to 864825, EPI_ISL_864827 to 864829, EPI_ISL_864831 to 864832, EPI_ISL_864834, EPI_ISL_864837 to 864839, EPI_ISL_864841, EPI_ISL_864845 to 864846, EPI_ISL_864849 to 864850, EPI_ISL_864853 to 864854, EPI_ISL_864856 to 864859, EPI_ISL_864862, EPI_ISL_864865, EPI_ISL_864867, EPI_ISL_864869, EPI_ISL_864871, EPI_ISL_864873, EPI_ISL_864875, EPI_ISL_864877, EPI_ISL_864879, EPI_ISL_864881, EPI_ISL_864885, EPI_ISL_864889 to 864891, EPI_ISL_864895 to 864897, EPI_ISL_864899 to 864902, EPI_ISL_864904, EPI_ISL_864906 to 864907, EPI_ISL_864913, EPI_ISL_864915, EPI_ISL_864917 to 864919, EPI_ISL_864922, EPI_ISL_864925 to 864926, EPI_ISL_864929, EPI_ISL_864932, EPI_ISL_864934 to 864936, EPI_ISL_864939 to 864945, EPI_ISL_864947 to 864951, EPI_ISL_864953, EPI_ISL_864958, EPI_ISL_918984, EPI_ISL_918988 to 918990, EPI_ISL_918993 to 918994, EPI_ISL_918996, EPI_ISL_918998 to 918999, EPI_ISL_919001, EPI_ISL_919004 to 919005, EPI_ISL_919008 to 919010, EPI_ISL_919014 to 919018, EPI_ISL_919020 to 919022, EPI_ISL_919024 to 919035, EPI_ISL_919037, EPI_ISL_919040, EPI_ISL_919042 to 919046, EPI_ISL_919049 to 919061, EPI_ISL_919063, EPI_ISL_919065 to 919074, EPI_ISL_919076 to 919078, EPI_ISL_919080 to 919085, EPI_ISL_919087 to 919099, EPI_ISL_919101 to 919103, EPI_ISL_919105, EPI_ISL_919112, EPI_ISL_919114 to 919115, EPI_ISL_919117 to 919118, EPI_ISL_919120 to 919121, EPI_ISL_919124, EPI_ISL_919126 to 919131, EPI_ISL_919134 to 919135, EPI_ISL_919137, EPI_ISL_919140, EPI_ISL_919142 to 919144, EPI_ISL_919146 to 919151, EPI_ISL_919153 to 919157, EPI_ISL_919159, EPI_ISL_919161 to 919164, EPI_ISL_919166 to 919169, EPI_ISL_919173 to 919175, EPI_ISL_919177, EPI_ISL_919182 to 919184, EPI_ISL_919187 to 919188, EPI_ISL_920895 to 920903, EPI_ISL_920906, EPI_ISL_920908 to 920909, EPI_ISL_920911 to 920917, EPI_ISL_920919 to 920922, EPI_ISL_920925 to 920929, EPI_ISL_920931 to 920933, EPI_ISL_920935, EPI_ISL_920937 to 920940, EPI_ISL_920942, EPI_ISL_920944 to 920946, EPI_ISL_920948, EPI_ISL_920950 to 920953, EPI_ISL_920955 to 920958, EPI_ISL_920960 to 920963, EPI_ISL_920965 to 920967, EPI_ISL_920969, EPI_ISL_920971 to 920974, EPI_ISL_923691 to 923697, EPI_ISL_923699, EPI_ISL_923701 to 923706, EPI_ISL_923708 to 923710, EPI_ISL_923713 to 923715, EPI_ISL_923717, EPI_ISL_923719 to 923723, EPI_ISL_923725 to 923726, EPI_ISL_923728 to 923733, EPI_ISL_923738, EPI_ISL_923741 to 923748, EPI_ISL_923750, EPI_ISL_923752 to 923755, EPI_ISL_923759, EPI_ISL_923761 to 923762, EPI_ISL_923764, EPI_ISL_923766 to 923770, EPI_ISL_923773 to 923774, EPI_ISL_923776, EPI_ISL_923779, EPI_ISL_923782 to 923784, EPI_ISL_923787, EPI_ISL_923789, EPI_ISL_923791 to 923793, EPI_ISL_923797, EPI_ISL_923799 to 923800, EPI_ISL_923802 to 923803, EPI_ISL_923805, EPI_ISL_923807 to 923808, EPI_ISL_923812 to 923816, EPI_ISL_923818, EPI_ISL_923820 to 923822, EPI_ISL_923825 to 923826, EPI_ISL_923828 to 923830, EPI_ISL_923832, EPI_ISL_923834, EPI_ISL_923836 to 923837, EPI_ISL_923841, EPI_ISL_923845, EPI_ISL_923847, EPI_ISL_923849, EPI_ISL_923851, EPI_ISL_923853, EPI_ISL_923856 to 923862, EPI_ISL_923865 to 923866, EPI_ISL_923868 to 923869, EPI_ISL_923873, EPI_ISL_923876, EPI_ISL_923878 to 923880, EPI_ISL_923883 to 923884, EPI_ISL_923886, EPI_ISL_923890 to 923891, EPI_ISL_923893, EPI_ISL_923895 to 923897, EPI_ISL_923901, EPI_ISL_923904, EPI_ISL_923906, EPI_ISL_923908, EPI_ISL_923910 to 923912, EPI_ISL_923914 to 923917, EPI_ISL_923919 to 923920, EPI_ISL_923923 to 923925, EPI_ISL_923928, EPI_ISL_923930, EPI_ISL_923933, EPI_ISL_923935 to 923937, EPI_ISL_923940 to 923943, EPI_ISL_923945 to 923947, EPI_ISL_923952, EPI_ISL_923955 to 923956, EPI_ISL_923958 to 923959, EPI_ISL_923964 to 923967, EPI_ISL_923969 to 923971, EPI_ISL_923973 to 923975, EPI_ISL_923977 to 923980, EPI_ISL_923982, EPI_ISL_923984 to 923985, EPI_ISL_923989, EPI_ISL_923992 to 923994, EPI_ISL_923999, EPI_ISL_924001 to 924002, EPI_ISL_924004, EPI_ISL_924006, EPI_ISL_924009, EPI_ISL_924011 to 924012, EPI_ISL_924014, EPI_ISL_924017 to 924020, EPI_ISL_924022, EPI_ISL_924024 to 924026, EPI_ISL_924029 to 924031, EPI_ISL_924034 to 924036, EPI_ISL_924038, EPI_ISL_924043 to 924045, EPI_ISL_924047 to 924049, EPI_ISL_924052, EPI_ISL_924054 to 924055, EPI_ISL_924057, EPI_ISL_924060, EPI_ISL_924062 to 924067, EPI_ISL_924069 to 924076, EPI_ISL_949258 to 949259, EPI_ISL_949262 to 949263, EPI_ISL_949266 to 949268, EPI_ISL_949272 to 949275, EPI_ISL_949278 to 949280, EPI_ISL_949282, EPI_ISL_949284, EPI_ISL_949287, EPI_ISL_949291, EPI_ISL_949293, EPI_ISL_949295, EPI_ISL_949297 to 949299, EPI_ISL_949301, EPI_ISL_949303 to 949304, EPI_ISL_949306, EPI_ISL_949308 to 949309, EPI_ISL_949313 to 949326, EPI_ISL_949427, EPI_ISL_949429 to 949432, EPI_ISL_949436 to 949439, EPI_ISL_949441 to 949442, EPI_ISL_949444 to 949445, EPI_ISL_949447 to 949448, EPI_ISL_949450 to 949454, EPI_ISL_949457 to 949458, EPI_ISL_949460 to 949464, EPI_ISL_949466 to 949468, EPI_ISL_949470 to 949478, EPI_ISL_949480, EPI_ISL_949483 to 949484, EPI_ISL_949486, EPI_ISL_949489 to 949491, EPI_ISL_949493 to 949499, EPI_ISL_949501 to 949506, EPI_ISL_949509 to 949510, EPI_ISL_949512 to 949515, EPI_ISL_949517 to 949519, EPI_ISL_949521 to 949524, EPI_ISL_949526 to 949527, EPI_ISL_949530 to 949533, EPI_ISL_949535 to 949538, EPI_ISL_949543 to 949548, EPI_ISL_949550 to 949552, EPI_ISL_949554 to 949558, EPI_ISL_949560 to 949563, EPI_ISL_949565 to 949572, EPI_ISL_949574 to 949580, EPI_ISL_949582 to 949590, EPI_ISL_949592 to 949596, EPI_ISL_949599, EPI_ISL_949601 to 949605, EPI_ISL_949607, EPI_ISL_952858, EPI_ISL_952860, EPI_ISL_952862 to 952863, EPI_ISL_952865 to 952866, EPI_ISL_952868, EPI_ISL_952874, EPI_ISL_952876, EPI_ISL_952878 to 952883, EPI_ISL_952886, EPI_ISL_952888 to 952891, EPI_ISL_952893 to 952894, EPI_ISL_952896 to 952897, EPI_ISL_952899, EPI_ISL_952902 to 952906, EPI_ISL_952908, EPI_ISL_952910, EPI_ISL_952914, EPI_ISL_952917, EPI_ISL_952920, EPI_ISL_952923, EPI_ISL_952926, EPI_ISL_952929, EPI_ISL_952934 to 952935, EPI_ISL_952937 to 952938, EPI_ISL_996066, EPI_ISL_996069, EPI_ISL_996073, EPI_ISL_996077, EPI_ISL_996598 to 996612, EPI_ISL_996614 to 996623, EPI_ISL_996626 to 996635, EPI_ISL_996638 to 996645, EPI_ISL_996647 to 996652, EPI_ISL_996655 to 996656, EPI_ISL_996658, EPI_ISL_996661 to 996664, EPI_ISL_996666 to 996667, EPI_ISL_996670, EPI_ISL_996672 to 996681, EPI_ISL_996683, EPI_ISL_996686, EPI_ISL_996689 to 996694, EPI_ISL_996696 to 996698, EPI_ISL_996700 to 996703, EPI_ISL_996705 to 996709, EPI_ISL_996711 to 996720, EPI_ISL_996722 to 996725, EPI_ISL_996727 to 996734, EPI_ISL_996736 to 996739, EPI_ISL_996741 to 996745, EPI_ISL_996747 to 996749, EPI_ISL_996751 to 996758, EPI_ISL_996760, EPI_ISL_996763, EPI_ISL_996765 to 996766, EPI_ISL_996768 to 996769, EPI_ISL_996771 to 996776, EPI_ISL_996778 to 996791, EPI_ISL_996793 to 996795, EPI_ISL_996797 to 996798, EPI_ISL_996801 to 996802, EPI_ISL_996804, EPI_ISL_996806 to 996809, EPI_ISL_996811 to 996820, EPI_ISL_996822 to 996828, EPI_ISL_996831, EPI_ISL_996833, EPI_ISL_996837, EPI_ISL_996839 to 996845, EPI_ISL_996847 to 996850, EPI_ISL_996852 to 996858, EPI_ISL_996861, EPI_ISL_996863, EPI_ISL_996865 to 996869, EPI_ISL_996871 to 996872, EPI_ISL_996874, EPI_ISL_996876 to 996892, EPI_ISL_996894 to 996898, EPI_ISL_996901 to 996914, EPI_ISL_996916 to 996928, EPI_ISL_996930 to 996932, EPI_ISL_996934 to 996950, EPI_ISL_996952, EPI_ISL_996954 to 996980, EPI_ISL_996982 to 997002, EPI_ISL_997004 to 997021, EPI_ISL_997023 to 997049, EPI_ISL_1000633 to 1000634, EPI_ISL_1000636, EPI_ISL_1000639 to 1000640

see above	Department of Pathology, University of Cambridge	COVID-19 Genomics UK (COG-UK) Consortium	Aminu S. Jahun; Anna Yakovleva; Charlotte J. Houldcroft; Fahad A Khokhar; Grant Hall; Ian Goodfellow; Iliana Georgana; Laura G Caller; Luke W Meredith; M. Estee Torok; M. Estée Török; M. Est©e Trk; Malte Pinckert; Martin D. Curran; Myra Hosmillo; Rhys Izu; Rhys Izuagbe; Sarah L. Caddy; Surendra Parmar; Theresa Feltwell; William L. Hamilton; Yasmin Chaudhry

EPI_ISL_438257, EPI_ISL_438259 to 438260, EPI_ISL_438262, EPI_ISL_438265 to 438270, EPI_ISL_438272 to 438274, EPI_ISL_438276 to 438280, EPI_ISL_438282, EPI_ISL_438284, EPI_ISL_438286 to 438297, EPI_ISL_438299 to 438303, EPI_ISL_438305 to 438312, EPI_ISL_438315 to 438316, EPI_ISL_438318 to 438319, EPI_ISL_438321 to 438327, EPI_ISL_438329 to 438330, EPI_ISL_438332 to 438334, EPI_ISL_438337 to 438338, EPI_ISL_438340, EPI_ISL_438342 to 438347, EPI_ISL_438349 to 438352, EPI_ISL_438354 to 438356, EPI_ISL_438358 to 438363, EPI_ISL_438365 to 438372, EPI_ISL_438375 to 438378, EPI_ISL_438380 to 438385, EPI_ISL_438387 to 438392, EPI_ISL_438394, EPI_ISL_438396, EPI_ISL_438398 to 438401, EPI_ISL_438403 to 438404, EPI_ISL_438406 to 438409, EPI_ISL_438411 to 438416, EPI_ISL_438419 to 438431, EPI_ISL_438434, EPI_ISL_438436 to 438437, EPI_ISL_438440 to 438443, EPI_ISL_438446 to 438448, EPI_ISL_438451 to 438460, EPI_ISL_438462 to 438472, EPI_ISL_438474 to 438477, EPI_ISL_438479 to 438482, EPI_ISL_438484 to 438486, EPI_ISL_438488 to 438489, EPI_ISL_438491 to 438493, EPI_ISL_438496 to 438502, EPI_ISL_438504 to 438507, EPI_ISL_438509 to 438521, EPI_ISL_438523 to 438525, EPI_ISL_438527 to 438529, EPI_ISL_438531 to 438532, EPI_ISL_438534 to 438541, EPI_ISL_438543 to 438545, EPI_ISL_439368 to 439369, EPI_ISL_439371 to 439379, EPI_ISL_439381 to 439389, EPI_ISL_439391, EPI_ISL_439393, EPI_ISL_439396 to 439397, EPI_ISL_439399 to 439411, EPI_ISL_439414 to 439424, EPI_ISL_439426, EPI_ISL_439428 to 439434, EPI_ISL_439436 to 439444, EPI_ISL_439446 to 439457, EPI_ISL_439459 to 439461, EPI_ISL_439463 to 439466, EPI_ISL_439468 to 439471, EPI_ISL_439473, EPI_ISL_439475 to 439476, EPI_ISL_439478 to 439479, EPI_ISL_439481 to 439482, EPI_ISL_439484 to 439487, EPI_ISL_439489 to 439492, EPI_ISL_439494 to 439496, EPI_ISL_439498 to 439499, EPI_ISL_439501 to 439505, EPI_ISL_439507 to 439511, EPI_ISL_439514, EPI_ISL_439516 to 439528, EPI_ISL_439532 to 439536, EPI_ISL_439538, EPI_ISL_439540 to 439542, EPI_ISL_439545, EPI_ISL_439547 to 439548, EPI_ISL_439550, EPI_ISL_439555, EPI_ISL_439557, EPI_ISL_439559, EPI_ISL_439562, EPI_ISL_439564 to 439565, EPI_ISL_439567 to 439568, EPI_ISL_439570 to 439572, EPI_ISL_439574 to 439576, EPI_ISL_439582, EPI_ISL_439585, EPI_ISL_439587 to 439592, EPI_ISL_439594, EPI_ISL_439596, EPI_ISL_439598 to 439599, EPI_ISL_439601 to 439602, EPI_ISL_439604, EPI_ISL_439606 to 439615, EPI_ISL_439618 to 439623, EPI_ISL_439625 to 439628, EPI_ISL_439630 to 439631, EPI_ISL_439633 to 439649, EPI_ISL_439652 to 439653, EPI_ISL_439655 to 439657, EPI_ISL_439659 to 439660, EPI_ISL_439662, EPI_ISL_439664, EPI_ISL_439863, EPI_ISL_439865, EPI_ISL_439867 to 439868, EPI_ISL_439872, EPI_ISL_439874 to 439877, EPI_ISL_439880, EPI_ISL_439882 to 439884, EPI_ISL_439886, EPI_ISL_439888 to 439889, EPI_ISL_439891 to 439892, EPI_ISL_439895, EPI_ISL_439898, EPI_ISL_439900, EPI_ISL_439902 to 439905, EPI_ISL_439907, EPI_ISL_439909, EPI_ISL_439911, EPI_ISL_439914, EPI_ISL_439917 to 439921, EPI_ISL_439923, EPI_ISL_439926 to 439927, EPI_ISL_439930 to 439938, EPI_ISL_439941, EPI_ISL_439943, EPI_ISL_439945, EPI_ISL_439947, EPI_ISL_439949, EPI_ISL_439954, EPI_ISL_439956, EPI_ISL_439958, EPI_ISL_439961, EPI_ISL_439963, EPI_ISL_439968, EPI_ISL_439970, EPI_ISL_439975, EPI_ISL_439980, EPI_ISL_439987, EPI_ISL_439989 to 439991, EPI_ISL_439993, EPI_ISL_439997, EPI_ISL_439999, EPI_ISL_440004, EPI_ISL_440007, EPI_ISL_440009 to 440011, EPI_ISL_440014, EPI_ISL_440017, EPI_ISL_440025, EPI_ISL_440030, EPI_ISL_440034, EPI_ISL_440040 to 440041, EPI_ISL_440044 to 440047, EPI_ISL_440053, EPI_ISL_440055, EPI_ISL_440057, EPI_ISL_440059 to 440062, EPI_ISL_440064, EPI_ISL_440066, EPI_ISL_440069, EPI_ISL_440072 to 440077, EPI_ISL_440079 to 440080, EPI_ISL_440085 to 440086, EPI_ISL_440088, EPI_ISL_440090, EPI_ISL_440096 to 440097, EPI_ISL_440099, EPI_ISL_440104, EPI_ISL_440107 to 440112, EPI_ISL_440117, EPI_ISL_440120 to 440122, EPI_ISL_440125, EPI_ISL_440129, EPI_ISL_440132, EPI_ISL_440138, EPI_ISL_440143 to 440144, EPI_ISL_440146 to 440147, EPI_ISL_440150, EPI_ISL_440152, EPI_ISL_440155, EPI_ISL_440159, EPI_ISL_440161, EPI_ISL_440163 to 440164, EPI_ISL_440166, EPI_ISL_440169 to 440170, EPI_ISL_440176 to 440177, EPI_ISL_440179, EPI_ISL_440181 to 440186, EPI_ISL_440188, EPI_ISL_440195, EPI_ISL_440202 to 440203, EPI_ISL_440207 to 440208, EPI_ISL_440213, EPI_ISL_440217, EPI_ISL_440223, EPI_ISL_440226, EPI_ISL_440231, EPI_ISL_440234, EPI_ISL_440237, EPI_ISL_440239, EPI_ISL_440244, EPI_ISL_440246, EPI_ISL_440254, EPI_ISL_440257 to 440261, EPI_ISL_440264 to 440265, EPI_ISL_440267 to 440268, EPI_ISL_440270 to 440271, EPI_ISL_440278 to 440280, EPI_ISL_440282 to 440285, EPI_ISL_440287 to 440290, EPI_ISL_440293, EPI_ISL_440295 to 440297, EPI_ISL_440300 to 440307, EPI_ISL_440309 to 440311, EPI_ISL_440313 to 440315, EPI_ISL_440318 to 440319, EPI_ISL_440321, EPI_ISL_440323, EPI_ISL_440328 to 440330, EPI_ISL_440333, EPI_ISL_440335, EPI_ISL_440337 to 440338, EPI_ISL_440340 to 440345, EPI_ISL_440347 to 440348, EPI_ISL_440351 to 440352, EPI_ISL_440354, EPI_ISL_440356 to 440359, EPI_ISL_440361, EPI_ISL_440364 to 440365, EPI_ISL_440367, EPI_ISL_440369, EPI_ISL_440373 to 440376, EPI_ISL_440378 to 440382, EPI_ISL_440384, EPI_ISL_440386 to 440387, EPI_ISL_440389, EPI_ISL_440391, EPI_ISL_440393, EPI_ISL_440395 to 440397, EPI_ISL_440399 to 440405, EPI_ISL_440407, EPI_ISL_440409, EPI_ISL_440411, EPI_ISL_440413 to 440414, EPI_ISL_440417 to 440422, EPI_ISL_440426, EPI_ISL_440428 to 440434, EPI_ISL_440436 to 440439, EPI_ISL_440445, EPI_ISL_440451, EPI_ISL_440459 to 440460, EPI_ISL_440464, EPI_ISL_440480, EPI_ISL_440483, EPI_ISL_440486 to 440487, EPI_ISL_440489, EPI_ISL_440491 to 440492, EPI_ISL_440494, EPI_ISL_440501, EPI_ISL_440503, EPI_ISL_440506 to 440507, EPI_ISL_440510 to 440511, EPI_ISL_440515 to 440516, EPI_ISL_440518 to 440519, EPI_ISL_440521, EPI_ISL_440525 to 440526, EPI_ISL_440532 to 440534, EPI_ISL_440538 to 440539, EPI_ISL_440541 to 440542, EPI_ISL_440547, EPI_ISL_440551 to 440553, EPI_ISL_440555, EPI_ISL_440557 to 440558, EPI_ISL_440560 to 440571, EPI_ISL_440573 to 440574, EPI_ISL_440576 to 440577, EPI_ISL_440579, EPI_ISL_440581 to 440584, EPI_ISL_440586, EPI_ISL_440589 to 440593, EPI_ISL_440595, EPI_ISL_440597 to 440601, EPI_ISL_440603 to 440608, EPI_ISL_440610 to 440613, EPI_ISL_440615 to 440622, EPI_ISL_440814, EPI_ISL_440832, EPI_ISL_440839, EPI_ISL_441053 to 441055, EPI_ISL_441057 to 441059, EPI_ISL_441062 to 441064, EPI_ISL_441066, EPI_ISL_441068, EPI_ISL_441070, EPI_ISL_441072 to 441077, EPI_ISL_441079, EPI_ISL_441081 to 441086, EPI_ISL_441088 to 441093, EPI_ISL_441095 to 441096, EPI_ISL_441098 to 441104, EPI_ISL_441107 to 441108, EPI_ISL_441110 to 441112, EPI_ISL_441114 to 441115, EPI_ISL_441118 to 441120, EPI_ISL_441122 to 441128, EPI_ISL_441130 to 441132, EPI_ISL_441135 to 441148, EPI_ISL_441150, EPI_ISL_441152 to 441158, EPI_ISL_441160 to 441162, EPI_ISL_441164 to 441167, EPI_ISL_441169 to 441180, EPI_ISL_441183, EPI_ISL_441187 to 441188, EPI_ISL_441191 to 441192, EPI_ISL_441194 to 441196, EPI_ISL_441198, EPI_ISL_441200 to 441211, EPI_ISL_441213 to 441218, EPI_ISL_441220 to 441229, EPI_ISL_441231 to 441232, EPI_ISL_441234, EPI_ISL_441236 to 441237, EPI_ISL_441239 to 441240, EPI_ISL_441242 to 441245, EPI_ISL_441247, EPI_ISL_441249 to 441251, EPI_ISL_441253 to 441256, EPI_ISL_441259 to 441260, EPI_ISL_441262, EPI_ISL_441266, EPI_ISL_441268 to 441272, EPI_ISL_441274, EPI_ISL_441278, EPI_ISL_441280 to 441284, EPI_ISL_441286, EPI_ISL_441288 to 441290, EPI_ISL_441292 to 441305, EPI_ISL_441307 to 441315, EPI_ISL_441317, EPI_ISL_441319 to 441322, EPI_ISL_441325 to 441326, EPI_ISL_441328, EPI_ISL_441331 to 441335, EPI_ISL_441337 to 441342, EPI_ISL_441344 to 441347, EPI_ISL_441349, EPI_ISL_441547, EPI_ISL_441549 to 441552, EPI_ISL_441554 to 441566, EPI_ISL_441569, EPI_ISL_441571 to 441579, EPI_ISL_441582 to 441588, EPI_ISL_441590 to 441591, EPI_ISL_441593 to 441597, EPI_ISL_441599 to 441600, EPI_ISL_441602 to 441607, EPI_ISL_441609 to 441619, EPI_ISL_441621, EPI_ISL_441623 to 441624, EPI_ISL_441626 to 441645, EPI_ISL_441647 to 441651, EPI_ISL_441653 to 441654, EPI_ISL_441656 to 441658, EPI_ISL_441660, EPI_ISL_441664 to 441669, EPI_ISL_441671 to 441674, EPI_ISL_441677 to 441684, EPI_ISL_441686 to 441689, EPI_ISL_441692, EPI_ISL_441695, EPI_ISL_441700 to 441703, EPI_ISL_441707, EPI_ISL_441709, EPI_ISL_441711, EPI_ISL_441713, EPI_ISL_441715, EPI_ISL_441718, EPI_ISL_441720 to 441721, EPI_ISL_441723 to 441729, EPI_ISL_441731 to 441732, EPI_ISL_441734 to 441736, EPI_ISL_441738 to 441746, EPI_ISL_441748 to 441750, EPI_ISL_441752, EPI_ISL_441754 to 441755, EPI_ISL_441758 to 441760, EPI_ISL_441762, EPI_ISL_441764, EPI_ISL_441767 to 441769, EPI_ISL_441772 to 441774, EPI_ISL_441777 to 441780, EPI_ISL_441782, EPI_ISL_441784 to 441793, EPI_ISL_441795, EPI_ISL_441797 to 441798, EPI_ISL_441800 to 441804, EPI_ISL_441806, EPI_ISL_441809, EPI_ISL_441811, EPI_ISL_441813, EPI_ISL_441816 to 441818, EPI_ISL_441820, EPI_ISL_441824, EPI_ISL_441826 to 441827, EPI_ISL_441830 to 441833, EPI_ISL_441835 to 441837, EPI_ISL_441841 to 441845, EPI_ISL_442045 to 442051, EPI_ISL_442053 to 442062, EPI_ISL_442064, EPI_ISL_442066 to 442068, EPI_ISL_442070 to 442071, EPI_ISL_442073 to 442086, EPI_ISL_442088 to 442095, EPI_ISL_442097 to 442098, EPI_ISL_442101 to 442109, EPI_ISL_442111 to 442114, EPI_ISL_442116 to 442117, EPI_ISL_442120 to 442126, EPI_ISL_442129 to 442130, EPI_ISL_442133 to 442134, EPI_ISL_442136, EPI_ISL_442139 to 442140, EPI_ISL_442142 to 442144, EPI_ISL_442146 to 442147, EPI_ISL_442149 to 442150, EPI_ISL_442154, EPI_ISL_442157 to 442158, EPI_ISL_442160 to 442161, EPI_ISL_442163 to 442165, EPI_ISL_442167 to 442172, EPI_ISL_442174 to 442176, EPI_ISL_442179 to 442180, EPI_ISL_442182 to 442184, EPI_ISL_442186 to 442190, EPI_ISL_442192 to 442194, EPI_ISL_442196 to 442204, EPI_ISL_442206 to 442208, EPI_ISL_442210 to 442211, EPI_ISL_442213 to 442217, EPI_ISL_442220 to 442226, EPI_ISL_442229 to 442232, EPI_ISL_442234 to 442237, EPI_ISL_442240 to 442242, EPI_ISL_442244, EPI_ISL_442246 to 442248, EPI_ISL_442250 to 442263, EPI_ISL_442265 to 442266, EPI_ISL_442268 to 442270, EPI_ISL_442272 to 442278, EPI_ISL_442281 to 442289, EPI_ISL_442291 to 442292, EPI_ISL_442294 to 442297, EPI_ISL_442299, EPI_ISL_442301 to 442302, EPI_ISL_442305 to 442310, EPI_ISL_442312 to 442313, EPI_ISL_442315 to 442321, EPI_ISL_442323 to 442327, EPI_ISL_442329 to 442343, EPI_ISL_442524, EPI_ISL_442526 to 442529, EPI_ISL_442531 to 442539, EPI_ISL_442541 to 442543, EPI_ISL_442545 to 442546, EPI_ISL_442548, EPI_ISL_442553, EPI_ISL_442556, EPI_ISL_442558 to 442565, EPI_ISL_442567, EPI_ISL_442569 to 442570, EPI_ISL_442572 to 442575, EPI_ISL_442577 to 442578, EPI_ISL_442581 to 442584, EPI_ISL_442586 to 442589, EPI_ISL_442592 to 442597, EPI_ISL_442599, EPI_ISL_442601 to 442607, EPI_ISL_442610 to 442611, EPI_ISL_442613 to 442616, EPI_ISL_442618, EPI_ISL_442620, EPI_ISL_442622 to 442623, EPI_ISL_442811 to 442820, EPI_ISL_442822 to 442826, EPI_ISL_442828 to 442835, EPI_ISL_442839, EPI_ISL_442841 to 442842, EPI_ISL_442844 to 442854, EPI_ISL_442856 to 442860, EPI_ISL_442862 to 442876, EPI_ISL_442878 to 442886, EPI_ISL_442888 to 442892, EPI_ISL_442894 to 442895, EPI_ISL_442897 to 442901, EPI_ISL_442903 to 442910, EPI_ISL_442912 to 442920, EPI_ISL_442922 to 442923, EPI_ISL_442926 to 442927, EPI_ISL_442930, EPI_ISL_442932, EPI_ISL_442934 to 442940, EPI_ISL_442943 to 442945, EPI_ISL_442947, EPI_ISL_442952 to 442954, EPI_ISL_442956 to 442957, EPI_ISL_442959, EPI_ISL_442961 to 442962, EPI_ISL_442964 to 442970, EPI_ISL_442973 to 442985, EPI_ISL_442987 to 442989, EPI_ISL_442991, EPI_ISL_442993 to 443004, EPI_ISL_443007, EPI_ISL_443009 to 443011, EPI_ISL_443015, EPI_ISL_443017, EPI_ISL_443020 to 443027, EPI_ISL_443029 to 443033, EPI_ISL_443035 to 443039, EPI_ISL_443041 to 443047, EPI_ISL_443049 to 443051, EPI_ISL_443053, EPI_ISL_443055, EPI_ISL_443058 to 443063, EPI_ISL_443065 to 443066, EPI_ISL_443068, EPI_ISL_443070, EPI_ISL_443072, EPI_ISL_443074 to 443078, EPI_ISL_443080 to 443081, EPI_ISL_443083 to 443084, EPI_ISL_443086 to 443087, EPI_ISL_443089 to 443105, EPI_ISL_443111 to 443114, EPI_ISL_443116, EPI_ISL_443319, EPI_ISL_443332, EPI_ISL_443335 to 443336, EPI_ISL_443338, EPI_ISL_443340, EPI_ISL_443342 to 443343, EPI_ISL_443345, EPI_ISL_443349, EPI_ISL_443353, EPI_ISL_443359 to 443360, EPI_ISL_443371 to 443372, EPI_ISL_443375, EPI_ISL_443378, EPI_ISL_443383 to 443396, EPI_ISL_443398 to 443399, EPI_ISL_443402 to 443404, EPI_ISL_443406, EPI_ISL_443408 to 443411, EPI_ISL_443414 to 443417, EPI_ISL_443419, EPI_ISL_443421, EPI_ISL_443425 to 443429, EPI_ISL_443434, EPI_ISL_443436 to 443437, EPI_ISL_443440 to 443441, EPI_ISL_443444 to 443448, EPI_ISL_443450, EPI_ISL_443452 to 443461, EPI_ISL_443463 to 443467, EPI_ISL_443469 to 443470, EPI_ISL_443472 to 443474, EPI_ISL_443476 to 443478, EPI_ISL_443481 to 443483, EPI_ISL_443487 to 443490, EPI_ISL_443493 to 443496, EPI_ISL_443498, EPI_ISL_443500 to 443505, EPI_ISL_443507, EPI_ISL_443509 to 443512, EPI_ISL_443514 to 443515, EPI_ISL_443517, EPI_ISL_443519, EPI_ISL_443522 to 443538, EPI_ISL_443540, EPI_ISL_443545 to 443546, EPI_ISL_443548 to 443549, EPI_ISL_443552, EPI_ISL_443554, EPI_ISL_443557 to 443562, EPI_ISL_443564, EPI_ISL_443566 to 443569, EPI_ISL_443571 to 443572, EPI_ISL_443574 to 443579, EPI_ISL_443581 to 443583, EPI_ISL_443585 to 443595, EPI_ISL_443598 to 443600, EPI_ISL_443602, EPI_ISL_443604 to 443605, EPI_ISL_443607 to 443609, EPI_ISL_443612 to 443614, EPI_ISL_443616 to 443618, EPI_ISL_443621 to 443623, EPI_ISL_443625 to 443626, EPI_ISL_443628 to 443634, EPI_ISL_443636 to 443637, EPI_ISL_443639 to 443640, EPI_ISL_443644, EPI_ISL_443646, EPI_ISL_443648 to 443649, EPI_ISL_443651, EPI_ISL_443653 to 443654, EPI_ISL_443656 to 443658, EPI_ISL_443661 to 443663, EPI_ISL_443667 to 443668, EPI_ISL_443670, EPI_ISL_443672 to 443674, EPI_ISL_443676 to 443677, EPI_ISL_443680 to 443682, EPI_ISL_443685, EPI_ISL_458529, EPI_ISL_458533, EPI_ISL_458552, EPI_ISL_458556, EPI_ISL_458559, EPI_ISL_458576, EPI_ISL_459166 to 459167, EPI_ISL_459169, EPI_ISL_459171, EPI_ISL_459174, EPI_ISL_459178 to 459179, EPI_ISL_459194, EPI_ISL_459204, EPI_ISL_459207, EPI_ISL_459210 to 459211, EPI_ISL_459219, EPI_ISL_459227, EPI_ISL_459235, EPI_ISL_459240, EPI_ISL_459251 to 459252, EPI_ISL_459255, EPI_ISL_459257, EPI_ISL_459259, EPI_ISL_459262 to 459266, EPI_ISL_459269, EPI_ISL_459271, EPI_ISL_459274, EPI_ISL_459284, EPI_ISL_459287, EPI_ISL_459289, EPI_ISL_459292, EPI_ISL_459295 to 459296, EPI_ISL_459309 to 459310, EPI_ISL_459313, EPI_ISL_459315 to 459317, EPI_ISL_459320 to 459321, EPI_ISL_459412 to 459413, EPI_ISL_459416, EPI_ISL_459421, EPI_ISL_459423 to 459425, EPI_ISL_459434 to 459435, EPI_ISL_459437, EPI_ISL_459442, EPI_ISL_459448 to 459449, EPI_ISL_459453, EPI_ISL_459455 to 459456, EPI_ISL_459459, EPI_ISL_459461, EPI_ISL_459463, EPI_ISL_459466 to 459468, EPI_ISL_459476 to 459479, EPI_ISL_459481, EPI_ISL_459493, EPI_ISL_459496, EPI_ISL_459499, EPI_ISL_459501, EPI_ISL_459504, EPI_ISL_469896, EPI_ISL_469899, EPI_ISL_469903, EPI_ISL_469906, EPI_ISL_469911, EPI_ISL_470092, EPI_ISL_470096, EPI_ISL_470098 to 470099, EPI_ISL_470101 to 470105, EPI_ISL_470107 to 470110, EPI_ISL_470114 to 470125, EPI_ISL_470127 to 470128, EPI_ISL_470130 to 470133, EPI_ISL_470136 to 470137, EPI_ISL_470140 to 470144, EPI_ISL_470146 to 470148, EPI_ISL_470150, EPI_ISL_470152 to 470156, EPI_ISL_470158 to 470161, EPI_ISL_470163, EPI_ISL_470166 to 470167, EPI_ISL_470170 to 470174, EPI_ISL_470181 to 470188, EPI_ISL_470190, EPI_ISL_470192, EPI_ISL_470194, EPI_ISL_470197 to 470198, EPI_ISL_470200 to 470206, EPI_ISL_470208 to 470209, EPI_ISL_470212, EPI_ISL_470214 to 470215, EPI_ISL_470217 to 470219, EPI_ISL_470221, EPI_ISL_470223, EPI_ISL_470225, EPI_ISL_470227, EPI_ISL_470229, EPI_ISL_470231 to 470233, EPI_ISL_470235 to 470237, EPI_ISL_470239, EPI_ISL_470241, EPI_ISL_470244 to 470246, EPI_ISL_470248 to 470252, EPI_ISL_470254 to 470256, EPI_ISL_470258 to 470261, EPI_ISL_470265, EPI_ISL_470267 to 470268,

EPI_ISL_470270 to 470271, EPI_ISL_470273 to 470286, EPI_ISL_470289 to 470293, EPI_ISL_470297 to 470300, EPI_ISL_470302 to 470303, EPI_ISL_470307, EPI_ISL_470309 to 470316, EPI_ISL_470319 to 470320, EPI_ISL_470323 to 470325, EPI_ISL_470328 to 470332, EPI_ISL_470334 to 470336, EPI_ISL_470340 to 470345, EPI_ISL_470347 to 470351, EPI_ISL_470353, EPI_ISL_470359 to 470361, EPI_ISL_470363 to 470364, EPI_ISL_470366 to 470368, EPI_ISL_470370, EPI_ISL_470373 to 470375, EPI_ISL_470379 to 470383, EPI_ISL_470385 to 470388, EPI_ISL_470390, EPI_ISL_470392 to 470395, EPI_ISL_470397 to 470398, EPI_ISL_470400 to 470409, EPI_ISL_470411, EPI_ISL_470413, EPI_ISL_470416 to 470419, EPI_ISL_470424 to 470427, EPI_ISL_470430 to 470434, EPI_ISL_470437 to 470443, EPI_ISL_470445 to 470447, EPI_ISL_470449, EPI_ISL_470451 to 470457, EPI_ISL_470459 to 470462, EPI_ISL_470464 to 470466, EPI_ISL_470468, EPI_ISL_470471 to 470474, EPI_ISL_470477 to 470483, EPI_ISL_470485 to 470488, EPI_ISL_470490 to 470491, EPI_ISL_470495 to 470508, EPI_ISL_470510 to 470516, EPI_ISL_470519 to 470524, EPI_ISL_470526 to 470528, EPI_ISL_470531 to 470533, EPI_ISL_470535 to 470537, EPI_ISL_489381 to 489384, EPI_ISL_489386 to 489387, EPI_ISL_489390, EPI_ISL_489394 to 489395, EPI_ISL_489397, EPI_ISL_489409, EPI_ISL_489411, EPI_ISL_489414 to 489416, EPI_ISL_489420, EPI_ISL_489427 to 489428, EPI_ISL_489430 to 489432, EPI_ISL_489435 to 489438, EPI_ISL_489444, EPI_ISL_489447 to 489448, EPI_ISL_489451 to 489454, EPI_ISL_489456 to 489458, EPI_ISL_489461 to 489462, EPI_ISL_489465 to 489469, EPI_ISL_489472 to 489473, EPI_ISL_489475, EPI_ISL_489477 to 489479, EPI_ISL_489481 to 489488, EPI_ISL_489490, EPI_ISL_489492 to 489494, EPI_ISL_489496 to 489500, EPI_ISL_489503 to 489508, EPI_ISL_489510 to 489512, EPI_ISL_489514, EPI_ISL_489517 to 489522, EPI_ISL_489524 to 489527, EPI_ISL_489531, EPI_ISL_489534 to 489535, EPI_ISL_489540 to 489541, EPI_ISL_489544 to 489546, EPI_ISL_489549 to 489550, EPI_ISL_489552 to 489554, EPI_ISL_489557 to 489560, EPI_ISL_489562 to 489565, EPI_ISL_489568 to 489573, EPI_ISL_489575 to 489577, EPI_ISL_492190 to 492194, EPI_ISL_492196, EPI_ISL_492198 to 492201, EPI_ISL_492204 to 492206, EPI_ISL_511970, EPI_ISL_524495, EPI_ISL_524497 to 524498, EPI_ISL_524501, EPI_ISL_524509, EPI_ISL_524530 to 524531, EPI_ISL_524537, EPI_ISL_524551, EPI_ISL_524560, EPI_ISL_524564, EPI_ISL_524568, EPI_ISL_524570, EPI_ISL_524573, EPI_ISL_524575 to 524578, EPI_ISL_524583 to 524584, EPI_ISL_524586 to 524587, EPI_ISL_524589, EPI_ISL_524591, EPI_ISL_524593 to 524594, EPI_ISL_524596 to 524601, EPI_ISL_524603 to 524604, EPI_ISL_524606, EPI_ISL_524608 to 524610, EPI_ISL_524612, EPI_ISL_524615 to 524618			
see above	Department of Pathology, University of Cambridge	Wellcome Sanger Institute for the COVID-19 Genomics UK (COG-UK) consortium	Alex Alderton; Aminu S. Jahun; Anna Yakovleva; Charlotte J. Houldcroft; Cordelia Langford; David K. Jackson; Dominic Kwiatkowski; Ewan Harrison; Fahad A Khokhar; Grant Hall; Ian Goodfellow; Ian Johnston; John Sillitoe on behalf of the Wellcome Sanger Institute COVID-19 Surveillance Team (http://www.sanger.ac.uk/covid-team); Laura G Caller; Luke W Meredith; M. Estée Török; Martin D. Curran; Myra Hosmillo; Roberto Amato; Sarah L. Caddy; Sonia Goncalves; Theresa Feltwell; William L. Hamilton; and Alex Alderton
EPI_ISL_576146 to 576149	Department of Respiratory & Other Viral Infections of L.V. Gromashevsky Institute of Epidemiology & Infectious Diseases NAMS of Ukraine	Department of Respiratory & Other Viral Infections of L.V. Gromashevsky Institute of Epidemiology & Infectious Diseases NAMS of Ukraine, JSC "Farmak"	Alla Mironenko; Andriy Goy; Ihor Kravchuk; Larysa Radchenko; Liudmyla Bolotova; Nataliia Teteriuk
EPI_ISL_582509	Department of Respiratory and other Viral Infections of L.V.Gromashevsky Institute of Epidemiology & Infectious Diseases NAMS of Ukraine	Department of Respiratory and other Viral Infections of L.V.Gromashevsky Institute of Epidemiology & Infectious Diseases NAMS of Ukraine, JSC "Farmak"	Alla Mironenko; Andriy Goy; Ihor Kravchuk; Larysa Radchenko; Ludmyla Bolotova; Nataliia Teteriuk
EPI_ISL_582510	Department of Respiratory and other Viral Infections of L.V.Gromashevsky Institute of Epidemiology & Infectious Diseases NAMS of Ukraine	Department of Respiratory and other Viral Infections of L.V.Gromashevsky Institute of Epidemiology & Infectious Diseases NAMS of Ukrain, JSC "Farmak"	Alla Mironenko; Andriy Goy; Ihor Kravchuk; Larysa Radchenko; Ludmyla Bolotova; Nataliia Teteriuk
EPI_ISL_582511 to 582513, EPI_ISL_654819 to 654820	Department of Respiratory and other Viral Infections of L.V.Gromashevsky Institute of Epidemiology & Infectious Diseases NAMS of Ukrain	Department of Respiratory and other Viral Infections of L.V.Gromashevsky Institute of Epidemiology & Infectious Diseases NAMS of Ukrain, JSC "Farmak"	Alla Mironenko; Andriy Goy; Ihor Kravchuk; Larysa Radchenko; Ludmyla Bolotova; Nataliia Teteriuk
EPI_ISL_654818	Department of Respiratory and other Viral Infections of L.V.Gromashevsky Institute of Epidemiology & Infectious Diseases NAMS of Ukrain	Department of Respiratory and other Viral Infections of L.V.Gromashevsky Institute of Epidemiology & Infectious Diseases NAMS of Ukrain, JSC "Farmak"	Alla Mironenko; Andriy Goy; Ihor Kravchuk; Larysa Radchenko; Ludmyla Bolotova; Nataliia Teteriuk
EPI_ISL_732949, EPI_ISL_732951 to 732959	Department of Tropical Parasitology	Laboratory of Recombinant Vaccines	Boguslaw Szewczyk; Kirsi Aaltonen; Lukasz Rabalski; Maciej Grzybek; Maciej Kosinski; Ravi Kant; Tarja Sironen; Teemu Smura
EPI_ISL_487275	Department of Veterinary Pathology, University of Liege - FARAH	Department of Veterinary Pathology, University of Liege - FARAH	A.-S.; Clercx, C.; Desmecht, D.; Eloit, M.; Escriou, N.; Garigliany, M.; Giet, D.; Huon, C.; Vanlaere; van der Werf, S.
EPI_ISL_450198 to 450211	Department of Virology	Department of Virology	Ackermann, N.; Antwerpen, M.; Bengs, K.; Berger, A.; Boehm, S.; Boehmer; Boender; Buchholz, U.; Cai, W.; Corman; D.V.; Dangel, A.; Drosten, C.; Eberle, U.; Fingerle, V.; Grahl, A.; Haas, W.; Hamouda, O.; Hoch, M.; Hoermansdorfer, S.; Ippisch, S.; Jones; Katz, K.; Konrad, R.; Liebl, B.; M.M.; Marosevic; Muehlemann, B.; Muller, N.; Poertner, K.; Protzer, U.; Reich, A.; Rexroth, U.; Schneider, J.; Sing, A.; T.C.; T.S.; Treis, B.; V.M.; Veith, T.; Walter, M.; Wicklein, B.; Woelfel, R.; Woudenberg, T.; Zapf, A.; Zeitlmann, N.; an der Heiden, M.
EPI_ISL_768526 to 768527, EPI_ISL_804007 to 804008	Department of Virology I, National Institute of Infectious Diseases	Pathogen Genomics Center, National Institute of Infectious Diseases	Kentaro Itokawa; Makoto Kuroda; Masanori Hashino; Rina Tanaka; Shuetsu Fukushi; Souichi Yamada; Tsuyoshi Sekizuka
EPI_ISL_529135	Department of Virology III, National Institute of Infectious Diseases	Pathogen Genomics Center, National Institute of Infectious Diseases	Kentaro Itokawa; Makoto Kuroda; Makoto Takeda; Masanori Hashino; Rina Tanaka; Shutoku Matsuyama; Takaji Wakita; Tsuyoshi Sekizuka
EPI_ISL_413602 to 413604, EPI_ISL_414641 to 414643, EPI_ISL_414646, EPI_ISL_418385 to 418397, EPI_ISL_418399 to 418406, EPI_ISL_418408 to 418411, EPI_ISL_481513 to 481629, EPI_ISL_481631 to 481636, EPI_ISL_481638 to 481678, EPI_ISL_481681 to 481715, EPI_ISL_481717 to 481727, EPI_ISL_481729 to 481740, EPI_ISL_732565 to 732566, EPI_ISL_732568 to 732653, EPI_ISL_737034 to 737080, EPI_ISL_737209 to 737235, EPI_ISL_755943 to 755999, EPI_ISL_756001 to 756005, EPI_ISL_756007 to 756125, EPI_ISL_756165 to 756183, EPI_ISL_756247 to 756252, EPI_ISL_756282 to 756292, EPI_ISL_757288 to 757394, EPI_ISL_759763 to 759841, EPI_ISL_759899 to 759954, EPI_ISL_766016 to 766027, EPI_ISL_833208 to 833211, EPI_ISL_833213 to 833226, EPI_ISL_902858, EPI_ISL_902906, EPI_ISL_995839 to 995871, EPI_ISL_995879, EPI_ISL_995881 to 995882, EPI_ISL_995884, EPI_ISL_995899 to 995904, EPI_ISL_995922 to 995923, EPI_ISL_995931 to 995933, EPI_ISL_995945 to 995946, EPI_ISL_995950 to 995952, EPI_ISL_995955, EPI_ISL_995963, EPI_ISL_995969 to 995970, EPI_ISL_995973			
see above	Department of Virology and Immunology, University of Helsinki and Helsinki University Hospital, Huslab Finland	Department of Virology, Faculty of Medicine, University of Helsinki, Helsinki, Finland	Essi Korhonen; Fathiah Zakham; Hanna Jarva; Hanna Liimatainen; Hannimari Kallio-Kokko; Harri Kangas; Hussein Alburkat; Jenni Virtanen; Maija Lappalainen; Maija Suvanto; Olli Vapalahti; Pekka Ellonen; Phuoc Truong; Ravi Kant; Sari Hannula; Satu Kurkela; Teemu Smura
EPI_ISL_1015238	Department of Virology, Pitié-Salpêtrière hospital	Department of Virology, Pitié-Salpêtrière hospital	Anne-Geneviève Marcelin; Aude Jary; Karen Zafilaza; Stéphane Marot; Valentin Leducq; Vincent Calvez
EPI_ISL_757285 to 757286	Department of Virology, Public Health Laboratories Division	Department of Virology, Public Health Laboratories Division	Aamer Ikram; Massab Umair; Muhammad Salman
EPI_ISL_468161, EPI_ISL_468163	Department of Virology, Public Health Laboratories Division, National Institute of Health	Department of Virology, Public Health Laboratories Division, National Institute of Health	Aamer Ikram; Adnan Khurshid; John Klena; Massab Umair; Muhammad Salman; Nazish Badar; Shannon Whitmer
EPI_ISL_515974	Department of Virus and Microbiological Special Diagnostics, Statens Serum Institut	Albertsen lab, Department of Chemistry and Bioscience, Aalborg University, Denmark	Danish Corona Danica Consortia
EPI_ISL_855619, EPI_ISL_855638, EPI_ISL_855663, EPI_ISL_855673, EPI_ISL_855690, EPI_ISL_855694, EPI_ISL_855722, EPI_ISL_855727, EPI_ISL_855738, EPI_ISL_855760, EPI_ISL_855775, EPI_ISL_855802, EPI_ISL_855807, EPI_ISL_855863, EPI_ISL_855875, EPI_ISL_855893, EPI_ISL_855929, EPI_ISL_855984, EPI_ISL_856006, EPI_ISL_856012, EPI_ISL_856016, EPI_ISL_856045, EPI_ISL_856047, EPI_ISL_856060, EPI_ISL_856077, EPI_ISL_856085, EPI_ISL_856088, EPI_ISL_856092, EPI_ISL_856095, EPI_ISL_856100, EPI_ISL_856115, EPI_ISL_856133, EPI_ISL_856137, EPI_ISL_856142, EPI_ISL_856150, EPI_ISL_856165, EPI_ISL_856167, EPI_ISL_856190, EPI_ISL_856209, EPI_ISL_856238, EPI_ISL_856242, EPI_ISL_856249, EPI_ISL_856255, EPI_ISL_856267, EPI_ISL_856272, EPI_ISL_856278, EPI_ISL_856292, EPI_ISL_856302, EPI_ISL_856334, EPI_ISL_856340, EPI_ISL_856362, EPI_ISL_856373, EPI_ISL_856382, EPI_ISL_856387, EPI_ISL_856413, EPI_ISL_856427 to 856428, EPI_ISL_856461, EPI_ISL_856467, EPI_ISL_856494, EPI_ISL_856534, EPI_ISL_856591, EPI_ISL_869266, EPI_ISL_869298 to 869299, EPI_ISL_869312, EPI_ISL_869338, EPI_ISL_869348, EPI_ISL_869356, EPI_ISL_869359, EPI_ISL_869368 to 869369, EPI_ISL_869373 to 869374, EPI_ISL_869378, EPI_ISL_869386, EPI_ISL_869390, EPI_ISL_869406, EPI_ISL_869409, EPI_ISL_869415, EPI_ISL_869422, EPI_ISL_869451, EPI_ISL_869457, EPI_ISL_869460, EPI_ISL_869462, EPI_ISL_869483, EPI_ISL_869498, EPI_ISL_869504, EPI_ISL_869513, EPI_ISL_869518, EPI_ISL_869533, EPI_ISL_869557, EPI_ISL_869562, EPI_ISL_869599, EPI_ISL_869615, EPI_ISL_869621, EPI_ISL_869635, EPI_ISL_869650, EPI_ISL_869652 to 869653, EPI_ISL_869656 to 869657, EPI_ISL_869686, EPI_ISL_869690, EPI_ISL_869697, EPI_ISL_869706, EPI_ISL_869726 to 869727, EPI_ISL_869760, EPI_ISL_869772, EPI_ISL_869776, EPI_ISL_869793, EPI_ISL_869797, EPI_ISL_869805 to 869806, EPI_ISL_869812, EPI_ISL_869814, EPI_ISL_869821, EPI_ISL_869845, EPI_ISL_869847, EPI_ISL_869855, EPI_ISL_869859, EPI_ISL_869861, EPI_ISL_869891, EPI_ISL_869903 to 869904, EPI_ISL_869913, EPI_ISL_869921, EPI_ISL_869925, EPI_ISL_869927, EPI_ISL_869936, EPI_ISL_869938, EPI_ISL_869946, EPI_ISL_869957, EPI_ISL_869969, EPI_ISL_869985, EPI_ISL_870022, EPI_ISL_870026, EPI_ISL_870034, EPI_ISL_870038, EPI_ISL_870043, EPI_ISL_870045 to 870046, EPI_ISL_870060, EPI_ISL_870062, EPI_ISL_870069, EPI_ISL_870081, EPI_ISL_870094, EPI_ISL_870105, EPI_ISL_870113, EPI_ISL_870125, EPI_ISL_870130, EPI_ISL_870156, EPI_ISL_870189 to 870191, EPI_ISL_870197, EPI_ISL_870212, EPI_ISL_870214, EPI_ISL_870240 to 870241, EPI_ISL_870243, EPI_ISL_870274, EPI_ISL_870276, EPI_ISL_870283 to 870284, EPI_ISL_870333, EPI_ISL_870347, EPI_ISL_870365, EPI_ISL_870385, EPI_ISL_870390, EPI_ISL_870401, EPI_ISL_870408 to 870409, EPI_ISL_870418 to 870419, EPI_ISL_870443, EPI_ISL_870446, EPI_ISL_870465, EPI_ISL_870470, EPI_ISL_870473, EPI_ISL_870479, EPI_ISL_870483, EPI_ISL_870485, EPI_ISL_870492 to 870493, EPI_ISL_870496, EPI_ISL_870502, EPI_ISL_870510, EPI_ISL_870531, EPI_ISL_870546, EPI_ISL_870548, EPI_ISL_870559, EPI_ISL_870569, EPI_ISL_870586, EPI_ISL_870599, EPI_ISL_870602, EPI_ISL_870614, EPI_ISL_870616, EPI_ISL_870633, EPI_ISL_870648, EPI_ISL_870662, EPI_ISL_870670, EPI_ISL_870678, EPI_ISL_870691, EPI_ISL_870707, EPI_ISL_870712, EPI_ISL_870749, EPI_ISL_870755, EPI_ISL_870759, EPI_ISL_870779, EPI_ISL_870784, EPI_ISL_870821, EPI_ISL_870870, EPI_ISL_870874, EPI_ISL_870907, EPI_ISL_870923, EPI_ISL_870927 to 870928, EPI_ISL_870935, EPI_ISL_870940, EPI_ISL_870976, EPI_ISL_870999, EPI_ISL_871034, EPI_ISL_871039, EPI_ISL_871064, EPI_ISL_871068, EPI_ISL_871099, EPI_ISL_871119, EPI_ISL_871137, EPI_ISL_871148, EPI_ISL_871229, EPI_ISL_871240, EPI_ISL_871336, EPI_ISL_871417 to 871418, EPI_ISL_871434, EPI_ISL_871442, EPI_ISL_871466, EPI_ISL_871470, EPI_ISL_871486, EPI_ISL_871492, EPI_ISL_871496, EPI_ISL_871547, EPI_ISL_871569, EPI_ISL_871630, EPI_ISL_871701, EPI_ISL_925918, EPI_ISL_925927, EPI_ISL_925930, EPI_ISL_925933, EPI_ISL_925935 to 925936, EPI_ISL_925940 to 925941, EPI_ISL_925944, EPI_ISL_925947, EPI_ISL_925951, EPI_ISL_925958, EPI_ISL_925963, EPI_ISL_925965, EPI_ISL_925972 to 925973, EPI_ISL_925980 to 925981, EPI_ISL_925985, EPI_ISL_926004, EPI_ISL_926006, EPI_ISL_926013, EPI_ISL_926021, EPI_ISL_926034, EPI_ISL_926040, EPI_ISL_926056, EPI_ISL_926058, EPI_ISL_926067, EPI_ISL_926086, EPI_ISL_926107 to 926109, EPI_ISL_926121, EPI_ISL_926127, EPI_ISL_926141, EPI_ISL_926152, EPI_ISL_926156, EPI_ISL_926160, EPI_ISL_926166, EPI_ISL_926169, EPI_ISL_926174, EPI_ISL_926180, EPI_ISL_926185, EPI_ISL_926199, EPI_ISL_926226, EPI_ISL_926241, EPI_ISL_926244, EPI_ISL_926267, EPI_ISL_926281, EPI_ISL_926316, EPI_ISL_926320, EPI_ISL_926327, EPI_ISL_926337, EPI_ISL_926348, EPI_ISL_926355, EPI_ISL_926374, EPI_ISL_926383, EPI_ISL_926389, EPI_ISL_926400, EPI_ISL_926436, EPI_ISL_926439, EPI_ISL_926450, EPI_ISL_926457, EPI_ISL_926476, EPI_ISL_926516, EPI_ISL_926530 to 926531, EPI_ISL_926549, EPI_ISL_926556, EPI_ISL_926563, EPI_ISL_926567, EPI_ISL_926571, EPI_ISL_926582 to 926583, EPI_ISL_926602, EPI_ISL_926605, EPI_ISL_926608, EPI_ISL_926622, EPI_ISL_926632, EPI_ISL_926640, EPI_ISL_926650, EPI_ISL_926656, EPI_ISL_926663, EPI_ISL_926666 to 926668, EPI_ISL_926674, EPI_ISL_926680, EPI_ISL_926682, EPI_ISL_926691, EPI_ISL_926709, EPI_ISL_926712, EPI_ISL_926722, EPI_ISL_926752, EPI_ISL_926762, EPI_ISL_926779 to 926780, EPI_ISL_926790, EPI_ISL_926797, EPI_ISL_926804, EPI_ISL_926822 to 926823, EPI_ISL_926836 to 926837, EPI_ISL_926840, EPI_ISL_926842, EPI_ISL_926854 to 926855, EPI_ISL_926858, EPI_ISL_926864, EPI_ISL_926869, EPI_ISL_926874, EPI_ISL_926880, EPI_ISL_926884, EPI_ISL_926900, EPI_ISL_926902, EPI_ISL_926907 to 926908, EPI_ISL_926920, EPI_ISL_926923, EPI_ISL_926930, EPI_ISL_926968, EPI_ISL_926975, EPI_ISL_926989, EPI_ISL_926993, EPI_ISL_927004, EPI_ISL_927010, EPI_ISL_927020, EPI_ISL_927065 to 927066, EPI_ISL_927069, EPI_ISL_927071, EPI_ISL_927074, EPI_ISL_927076, EPI_ISL_927080 to 927081, EPI_ISL_927094 to 927095, EPI_ISL_927097, EPI_ISL_927110, EPI_ISL_927113, EPI_ISL_927121, EPI_ISL_927124, EPI_ISL_927129, EPI_ISL_927153, EPI_ISL_927156, EPI_ISL_927158, EPI_ISL_927175, EPI_ISL_927185, EPI_ISL_927188, EPI_ISL_927222, EPI_ISL_927225, EPI_ISL_927229, EPI_ISL_927235, EPI_ISL_927243, EPI_ISL_927245, EPI_ISL_927260, EPI_ISL_927264, EPI_ISL_927278, EPI_ISL_927292, EPI_ISL_927301 to 927302, EPI_ISL_927317 to 927318, EPI_ISL_927329, EPI_ISL_927335, EPI_ISL_927363, EPI_ISL_927372 to 927373, EPI_ISL_927379 to 927380, EPI_ISL_927400, EPI_ISL_927403, EPI_ISL_927437, EPI_ISL_927440, EPI_ISL_927444, EPI_ISL_927454, EPI_ISL_927465, EPI_ISL_927467 to 927468, EPI_ISL_927471, EPI_ISL_927484, EPI_ISL_927495, EPI_ISL_927506, EPI_ISL_927514, EPI_ISL_927520, EPI_ISL_927548, EPI_ISL_927552, EPI_ISL_927576, EPI_ISL_927584, EPI_ISL_927592, EPI_ISL_927619, EPI_ISL_927633 to 927634, EPI_ISL_927641, EPI_ISL_927647, EPI_ISL_927656, EPI_ISL_927661, EPI_ISL_927668, EPI_ISL_927700, EPI_ISL_927702, EPI_ISL_927711, EPI_ISL_927719, EPI_ISL_927738, EPI_ISL_927743, EPI_ISL_927747, EPI_ISL_927750 to 927751, EPI_ISL_927779, EPI_ISL_927822, EPI_ISL_927830 to 927831, EPI_ISL_927846, EPI_ISL_927851, EPI_ISL_927862, EPI_ISL_927878, EPI_ISL_927905, EPI_ISL_927919, EPI_ISL_927926, EPI_ISL_927936, EPI_ISL_927948, EPI_ISL_927952, EPI_ISL_927960, EPI_ISL_927967, EPI_ISL_927979, EPI_ISL_927983, EPI_ISL_927992, EPI_ISL_927994 to 927995, EPI_ISL_928007, EPI_ISL_928013, EPI_ISL_928015, EPI_ISL_928020, EPI_ISL_928030, EPI_ISL_928052, EPI_ISL_928093, EPI_ISL_928099, EPI_ISL_928110,			

EPI_ISL_928119, EPI_ISL_928135, EPI_ISL_928144, EPI_ISL_928147, EPI_ISL_928167, EPI_ISL_928169, EPI_ISL_928174, EPI_ISL_928178, EPI_ISL_928191, EPI_ISL_928194, EPI_ISL_928197 to 928198, EPI_ISL_928207, EPI_ISL_928214 to 928215, EPI_ISL_928217, EPI_ISL_928223, EPI_ISL_928232, EPI_ISL_928235 to 928236, EPI_ISL_928269 to 928271, EPI_ISL_928273, EPI_ISL_928294, EPI_ISL_928301, EPI_ISL_928321, EPI_ISL_928324 to 928325, EPI_ISL_928337, EPI_ISL_928341, EPI_ISL_928351, EPI_ISL_928368, EPI_ISL_928370, EPI_ISL_928372 to 928373, EPI_ISL_928381, EPI_ISL_928393 to 928394, EPI_ISL_928396, EPI_ISL_928411, EPI_ISL_928414, EPI_ISL_928419, EPI_ISL_928421, EPI_ISL_928423, EPI_ISL_928428, EPI_ISL_928435 to 928436, EPI_ISL_928453, EPI_ISL_928463, EPI_ISL_928469 to 928470, EPI_ISL_928479, EPI_ISL_928487, EPI_ISL_928492, EPI_ISL_928512, EPI_ISL_928514, EPI_ISL_928524, EPI_ISL_928572, EPI_ISL_928575 to 928576, EPI_ISL_928583, EPI_ISL_928589 to 928590, EPI_ISL_928598, EPI_ISL_928601, EPI_ISL_928605, EPI_ISL_928623, EPI_ISL_928627, EPI_ISL_928642, EPI_ISL_928644, EPI_ISL_928654, EPI_ISL_928657, EPI_ISL_928659, EPI_ISL_928667, EPI_ISL_928673, EPI_ISL_928688, EPI_ISL_928697 to 928698, EPI_ISL_928711, EPI_ISL_928739, EPI_ISL_928747, EPI_ISL_928755, EPI_ISL_928771, EPI_ISL_928773, EPI_ISL_928781, EPI_ISL_928800, EPI_ISL_928804 to 928805, EPI_ISL_928818 to 928819, EPI_ISL_928823, EPI_ISL_928840, EPI_ISL_928857, EPI_ISL_928859, EPI_ISL_928871 to 928872, EPI_ISL_928880, EPI_ISL_928888, EPI_ISL_928890, EPI_ISL_928892, EPI_ISL_928894, EPI_ISL_928910, EPI_ISL_928913, EPI_ISL_928928, EPI_ISL_928933, EPI_ISL_928938, EPI_ISL_928948, EPI_ISL_928983, EPI_ISL_928989, EPI_ISL_928996, EPI_ISL_928999, EPI_ISL_929007, EPI_ISL_929009, EPI_ISL_929012, EPI_ISL_929014, EPI_ISL_929028, EPI_ISL_929035, EPI_ISL_929039, EPI_ISL_929047, EPI_ISL_929052, EPI_ISL_929054, EPI_ISL_929056 to 929057, EPI_ISL_929077 to 929078, EPI_ISL_929080, EPI_ISL_929083 to 929084, EPI_ISL_929091, EPI_ISL_929102, EPI_ISL_929112 to 929113, EPI_ISL_929123, EPI_ISL_929125, EPI_ISL_929129, EPI_ISL_929162, EPI_ISL_929166, EPI_ISL_929168, EPI_ISL_929178, EPI_ISL_929193 to 929195, EPI_ISL_929200 to 929201, EPI_ISL_929212, EPI_ISL_929220, EPI_ISL_929225, EPI_ISL_929238, EPI_ISL_929240, EPI_ISL_929243, EPI_ISL_929255, EPI_ISL_929278, EPI_ISL_929280, EPI_ISL_929308, EPI_ISL_929310 to 929311, EPI_ISL_929332, EPI_ISL_929336, EPI_ISL_929344, EPI_ISL_929350, EPI_ISL_929367, EPI_ISL_929379, EPI_ISL_929384, EPI_ISL_929401, EPI_ISL_929405 to 929406, EPI_ISL_929426, EPI_ISL_929431, EPI_ISL_929436, EPI_ISL_929448, EPI_ISL_929456, EPI_ISL_929487, EPI_ISL_929499, EPI_ISL_929508, EPI_ISL_929514, EPI_ISL_929538 to 929539, EPI_ISL_929546, EPI_ISL_929550, EPI_ISL_929553 to 929554, EPI_ISL_929559, EPI_ISL_929579, EPI_ISL_929586, EPI_ISL_929592 to 929593, EPI_ISL_929599 to 929601, EPI_ISL_929609, EPI_ISL_929614 to 929615, EPI_ISL_929622, EPI_ISL_929629, EPI_ISL_929631, EPI_ISL_929644, EPI_ISL_929646, EPI_ISL_929650, EPI_ISL_929656 to 929657, EPI_ISL_929677, EPI_ISL_929681, EPI_ISL_929684, EPI_ISL_929701, EPI_ISL_929719, EPI_ISL_929725, EPI_ISL_929742, EPI_ISL_929749, EPI_ISL_929756, EPI_ISL_929766, EPI_ISL_929782, EPI_ISL_929789, EPI_ISL_929792, EPI_ISL_929799, EPI_ISL_929820, EPI_ISL_929824, EPI_ISL_929853, EPI_ISL_929860, EPI_ISL_929873, EPI_ISL_929876, EPI_ISL_929878, EPI_ISL_929901, EPI_ISL_929920, EPI_ISL_929925, EPI_ISL_929929, EPI_ISL_929949, EPI_ISL_929953, EPI_ISL_929956, EPI_ISL_929961, EPI_ISL_929973 to 929974, EPI_ISL_929986, EPI_ISL_929989 to 929990, EPI_ISL_930011, EPI_ISL_930028, EPI_ISL_930074, EPI_ISL_930083, EPI_ISL_930089, EPI_ISL_930097, EPI_ISL_930116, EPI_ISL_930118, EPI_ISL_930137, EPI_ISL_930148 to 930149, EPI_ISL_930156, EPI_ISL_930166, EPI_ISL_930168, EPI_ISL_930173, EPI_ISL_930186, EPI_ISL_930188 to 930189, EPI_ISL_930197, EPI_ISL_930213, EPI_ISL_930221, EPI_ISL_930228, EPI_ISL_930241, EPI_ISL_930249, EPI_ISL_930251, EPI_ISL_930262 to 930264, EPI_ISL_930267, EPI_ISL_930318, EPI_ISL_930328, EPI_ISL_930331, EPI_ISL_930335, EPI_ISL_930337, EPI_ISL_930344, EPI_ISL_930348 to 930349, EPI_ISL_930357, EPI_ISL_930362, EPI_ISL_930384, EPI_ISL_930389, EPI_ISL_930393, EPI_ISL_930401, EPI_ISL_930412 to 930413, EPI_ISL_930415, EPI_ISL_930425, EPI_ISL_930428, EPI_ISL_930431, EPI_ISL_930442, EPI_ISL_930448, EPI_ISL_930451, EPI_ISL_930473, EPI_ISL_930480, EPI_ISL_930487, EPI_ISL_930528, EPI_ISL_930531, EPI_ISL_930533, EPI_ISL_930544, EPI_ISL_930565, EPI_ISL_970810, EPI_ISL_970819, EPI_ISL_970823, EPI_ISL_970825, EPI_ISL_970828, EPI_ISL_970837, EPI_ISL_970850, EPI_ISL_970858, EPI_ISL_970877, EPI_ISL_970881 to 970883, EPI_ISL_970885, EPI_ISL_970888, EPI_ISL_970890, EPI_ISL_970893, EPI_ISL_970903, EPI_ISL_970909, EPI_ISL_970917, EPI_ISL_970927, EPI_ISL_970932, EPI_ISL_970935, EPI_ISL_970951 to 970953, EPI_ISL_970957 to 970958, EPI_ISL_970962, EPI_ISL_970969, EPI_ISL_970990, EPI_ISL_970996, EPI_ISL_971001, EPI_ISL_971009, EPI_ISL_971011, EPI_ISL_971014, EPI_ISL_971016, EPI_ISL_971025, EPI_ISL_971031, EPI_ISL_971033, EPI_ISL_971041, EPI_ISL_971058, EPI_ISL_971067, EPI_ISL_971092, EPI_ISL_971103, EPI_ISL_971106, EPI_ISL_971112, EPI_ISL_971124, EPI_ISL_971133, EPI_ISL_971148, EPI_ISL_971175, EPI_ISL_971198, EPI_ISL_971201, EPI_ISL_971214, EPI_ISL_971250, EPI_ISL_971272, EPI_ISL_971290, EPI_ISL_971293, EPI_ISL_971302, EPI_ISL_971315, EPI_ISL_971318, EPI_ISL_971323, EPI_ISL_971335, EPI_ISL_971338, EPI_ISL_971353, EPI_ISL_971361, EPI_ISL_971363, EPI_ISL_971375, EPI_ISL_971391, EPI_ISL_971401, EPI_ISL_971415, EPI_ISL_971431, EPI_ISL_971462, EPI_ISL_971467, EPI_ISL_971472, EPI_ISL_971474 to 971475, EPI_ISL_971493 to 971494, EPI_ISL_971497, EPI_ISL_971500, EPI_ISL_971508, EPI_ISL_971513, EPI_ISL_971517, EPI_ISL_971521, EPI_ISL_971540, EPI_ISL_971549, EPI_ISL_971555 to 971558, EPI_ISL_971565, EPI_ISL_971575, EPI_ISL_971578, EPI_ISL_971581, EPI_ISL_971585, EPI_ISL_971593, EPI_ISL_971596, EPI_ISL_971613, EPI_ISL_971619 to 971620, EPI_ISL_971629 to 971630, EPI_ISL_971634 to 971636, EPI_ISL_971638 to 971639, EPI_ISL_971654, EPI_ISL_971658, EPI_ISL_971661, EPI_ISL_971666, EPI_ISL_971673 to 971674, EPI_ISL_971676, EPI_ISL_971678 to 971679, EPI_ISL_971682, EPI_ISL_971684 to 971687, EPI_ISL_971693 to 971694, EPI_ISL_971697, EPI_ISL_971700 to 971702, EPI_ISL_971711, EPI_ISL_971721, EPI_ISL_971726, EPI_ISL_971730 to 971732, EPI_ISL_971735, EPI_ISL_971741, EPI_ISL_971746, EPI_ISL_971753, EPI_ISL_971757, EPI_ISL_971761, EPI_ISL_971763, EPI_ISL_971769, EPI_ISL_971776, EPI_ISL_971781, EPI_ISL_971785, EPI_ISL_971789, EPI_ISL_971792, EPI_ISL_971796, EPI_ISL_971809 to 971810, EPI_ISL_971815 to 971816, EPI_ISL_971818, EPI_ISL_971836, EPI_ISL_971838 to 971839, EPI_ISL_971843, EPI_ISL_971858 to 971859, EPI_ISL_971861, EPI_ISL_971864, EPI_ISL_971876, EPI_ISL_971879, EPI_ISL_971884 to 971885, EPI_ISL_971888, EPI_ISL_971894, EPI_ISL_971899, EPI_ISL_971903 to 971904, EPI_ISL_971907, EPI_ISL_971909 to 971910, EPI_ISL_971912, EPI_ISL_971928, EPI_ISL_971931, EPI_ISL_971935, EPI_ISL_971941, EPI_ISL_971946, EPI_ISL_971950, EPI_ISL_971952, EPI_ISL_971957, EPI_ISL_971963, EPI_ISL_971966, EPI_ISL_971969, EPI_ISL_971972, EPI_ISL_971974, EPI_ISL_971980, EPI_ISL_971984, EPI_ISL_972003, EPI_ISL_972008, EPI_ISL_972011, EPI_ISL_972018, EPI_ISL_972021, EPI_ISL_972026, EPI_ISL_972038, EPI_ISL_972046 to 972048, EPI_ISL_972059 to 972060, EPI_ISL_972075, EPI_ISL_972080, EPI_ISL_972086, EPI_ISL_972097, EPI_ISL_972104 to 972105, EPI_ISL_972107, EPI_ISL_972124, EPI_ISL_972132 to 972134, EPI_ISL_972137, EPI_ISL_972139, EPI_ISL_972145 to 972146, EPI_ISL_972148, EPI_ISL_972150, EPI_ISL_972155, EPI_ISL_972166, EPI_ISL_972177, EPI_ISL_972187, EPI_ISL_972198, EPI_ISL_972210 to 972211, EPI_ISL_972220, EPI_ISL_972223, EPI_ISL_972228, EPI_ISL_972234, EPI_ISL_972239, EPI_ISL_972248, EPI_ISL_972250, EPI_ISL_972259, EPI_ISL_972266, EPI_ISL_972271, EPI_ISL_972275, EPI_ISL_972277, EPI_ISL_972289, EPI_ISL_972292, EPI_ISL_972294 to 972295, EPI_ISL_972298, EPI_ISL_972300, EPI_ISL_972308, EPI_ISL_972312, EPI_ISL_972318, EPI_ISL_972321 to 972322, EPI_ISL_972328, EPI_ISL_972331, EPI_ISL_972334, EPI_ISL_972337, EPI_ISL_972343, EPI_ISL_972349, EPI_ISL_972355 to 972356, EPI_ISL_972359, EPI_ISL_972367, EPI_ISL_972372 to 972374, EPI_ISL_972380, EPI_ISL_972402, EPI_ISL_972412, EPI_ISL_972420, EPI_ISL_972425, EPI_ISL_972428, EPI_ISL_972434 to 972436, EPI_ISL_972438, EPI_ISL_972447 to 972448, EPI_ISL_972457, EPI_ISL_972468, EPI_ISL_972471 to 972472, EPI_ISL_972475, EPI_ISL_972481, EPI_ISL_972483, EPI_ISL_972489 to 972490, EPI_ISL_972500, EPI_ISL_972507, EPI_ISL_972510, EPI_ISL_972513 to 972514, EPI_ISL_972518, EPI_ISL_972526 to 972528, EPI_ISL_972530, EPI_ISL_972551, EPI_ISL_972553, EPI_ISL_972562, EPI_ISL_972573, EPI_ISL_972579 to 972580, EPI_ISL_972593, EPI_ISL_972597, EPI_ISL_972602, EPI_ISL_972617 to 972618, EPI_ISL_972639, EPI_ISL_972644, EPI_ISL_972649, EPI_ISL_972652, EPI_ISL_972655, EPI_ISL_972660, EPI_ISL_972662, EPI_ISL_972671 to 972672, EPI_ISL_972681, EPI_ISL_972684, EPI_ISL_972689, EPI_ISL_972692 to 972693, EPI_ISL_972699 to 972700, EPI_ISL_972705, EPI_ISL_972707, EPI_ISL_972720, EPI_ISL_972728, EPI_ISL_972731 to 972732, EPI_ISL_972744 to 972746, EPI_ISL_972757, EPI_ISL_972760, EPI_ISL_972762 to 972763, EPI_ISL_972768, EPI_ISL_972771, EPI_ISL_972776, EPI_ISL_972782, EPI_ISL_972788 to 972789, EPI_ISL_972794, EPI_ISL_972796, EPI_ISL_972799, EPI_ISL_972806, EPI_ISL_972808 to 972809, EPI_ISL_972812, EPI_ISL_972820, EPI_ISL_972822, EPI_ISL_972830, EPI_ISL_972836, EPI_ISL_972839, EPI_ISL_972848 to 972849, EPI_ISL_972856, EPI_ISL_972859, EPI_ISL_972863 to 972864, EPI_ISL_972867, EPI_ISL_972870, EPI_ISL_972872, EPI_ISL_972879, EPI_ISL_972883 to 972884, EPI_ISL_972887, EPI_ISL_972889, EPI_ISL_972896 to 972897, EPI_ISL_972900, EPI_ISL_972903 to 972904, EPI_ISL_972909 to 972910, EPI_ISL_972922, EPI_ISL_972931 to 972932, EPI_ISL_972934, EPI_ISL_972942, EPI_ISL_972945, EPI_ISL_972947 to 972948, EPI_ISL_972956, EPI_ISL_972961, EPI_ISL_972966, EPI_ISL_972976, EPI_ISL_972979, EPI_ISL_972981, EPI_ISL_972995, EPI_ISL_972997, EPI_ISL_973008, EPI_ISL_973015, EPI_ISL_973020, EPI_ISL_973030, EPI_ISL_973034, EPI_ISL_973039, EPI_ISL_973043, EPI_ISL_973052 to 973054, EPI_ISL_973057, EPI_ISL_973060, EPI_ISL_973076, EPI_ISL_973086, EPI_ISL_973104, EPI_ISL_973106, EPI_ISL_973109, EPI_ISL_973111 to 973112, EPI_ISL_973117, EPI_ISL_973120 to 973121, EPI_ISL_973123, EPI_ISL_973133, EPI_ISL_973135 to 973136, EPI_ISL_973143, EPI_ISL_973153 to 973154, EPI_ISL_973159, EPI_ISL_973168, EPI_ISL_973171, EPI_ISL_973174, EPI_ISL_973181 to 973182, EPI_ISL_973189, EPI_ISL_973195, EPI_ISL_973202, EPI_ISL_973205, EPI_ISL_973218 to 973219, EPI_ISL_973221 to 973222, EPI_ISL_973232, EPI_ISL_973235, EPI_ISL_973237, EPI_ISL_973243 to 973244, EPI_ISL_973255, EPI_ISL_973257, EPI_ISL_973260, EPI_ISL_973267 to 973268, EPI_ISL_973274, EPI_ISL_973279, EPI_ISL_973282, EPI_ISL_973285 to 973286, EPI_ISL_973290, EPI_ISL_973294, EPI_ISL_973297, EPI_ISL_973304, EPI_ISL_973311, EPI_ISL_973316, EPI_ISL_973329, EPI_ISL_973331, EPI_ISL_973337 to 973338, EPI_ISL_973349, EPI_ISL_973353, EPI_ISL_973359 to 973360, EPI_ISL_973373, EPI_ISL_973376, EPI_ISL_973382, EPI_ISL_973384 to 973385, EPI_ISL_973394, EPI_ISL_973396, EPI_ISL_973406, EPI_ISL_973408, EPI_ISL_973410 to 973411, EPI_ISL_973419, EPI_ISL_973424, EPI_ISL_973426, EPI_ISL_973430, EPI_ISL_973432, EPI_ISL_973435, EPI_ISL_973453, EPI_ISL_973457, EPI_ISL_973467, EPI_ISL_973471, EPI_ISL_973473 to 973474, EPI_ISL_973478, EPI_ISL_973482, EPI_ISL_973487, EPI_ISL_973499, EPI_ISL_973512, EPI_ISL_973519, EPI_ISL_973523, EPI_ISL_973538, EPI_ISL_973540, EPI_ISL_973553, EPI_ISL_973555, EPI_ISL_973570, EPI_ISL_973578, EPI_ISL_973584, EPI_ISL_973591, EPI_ISL_973593, EPI_ISL_973595, EPI_ISL_973621, EPI_ISL_973624, EPI_ISL_973640, EPI_ISL_973672, EPI_ISL_973677, EPI_ISL_973696, EPI_ISL_973708, EPI_ISL_973734, EPI_ISL_973761 to 973762, EPI_ISL_973768, EPI_ISL_973770, EPI_ISL_973777, EPI_ISL_973782, EPI_ISL_973787, EPI_ISL_973801, EPI_ISL_973803, EPI_ISL_973811 to 973812, EPI_ISL_973817, EPI_ISL_973822, EPI_ISL_973833, EPI_ISL_1022420, EPI_ISL_1022423 to 1022424, EPI_ISL_1022426, EPI_ISL_1022428 to 1022433, EPI_ISL_1022436 to 1022439, EPI_ISL_1022441 to 1022444, EPI_ISL_1022446 to 1022449, EPI_ISL_1022453, EPI_ISL_1022455 to 1022458, EPI_ISL_1022463 to 1022465, EPI_ISL_1022469, EPI_ISL_1022471 to 1022473, EPI_ISL_1022476, EPI_ISL_1022478 to 1022480, EPI_ISL_1022483 to 1022485, EPI_ISL_1022487, EPI_ISL_1022490 to 1022491, EPI_ISL_1022493, EPI_ISL_1022495, EPI_ISL_1022498, EPI_ISL_1022500, EPI_ISL_1022502 to 1022504, EPI_ISL_1022506 to 1022507, EPI_ISL_1022509, EPI_ISL_1022511 to 1022512, EPI_ISL_1022517 to 1022518, EPI_ISL_1022521, EPI_ISL_1022523, EPI_ISL_1022525, EPI_ISL_1022529 to 1022531, EPI_ISL_1022534, EPI_ISL_1022536, EPI_ISL_1022545, EPI_ISL_1022548 to 1022549, EPI_ISL_1022555 to 1022556, EPI_ISL_1022558, EPI_ISL_1022562, EPI_ISL_1022564 to 1022567, EPI_ISL_1022569, EPI_ISL_1022571 to 1022572, EPI_ISL_1022574 to 1022575, EPI_ISL_1022578 to 1022579, EPI_ISL_1022585, EPI_ISL_1022587 to 1022590, EPI_ISL_1022594 to 1022597, EPI_ISL_1022601, EPI_ISL_1022603 to 1022604, EPI_ISL_1022606, EPI_ISL_1022611, EPI_ISL_1022613 to 1022614, EPI_ISL_1022619 to 1022620, EPI_ISL_1022623 to 1022626, EPI_ISL_1022628 to 1022630, EPI_ISL_1022632 to 1022633, EPI_ISL_1022637 to 1022638, EPI_ISL_1022640, EPI_ISL_1022645 to 1022647, EPI_ISL_1022650 to 1022652, EPI_ISL_1022655, EPI_ISL_1022657, EPI_ISL_1022659, EPI_ISL_1022662 to 1022663, EPI_ISL_1022665 to 1022667, EPI_ISL_1022671, EPI_ISL_1022673 to 1022674, EPI_ISL_1022678 to 1022679, EPI_ISL_1022683, EPI_ISL_1022685 to 1022686, EPI_ISL_1022689, EPI_ISL_1022693, EPI_ISL_1022696 to 1022698, EPI_ISL_1022701 to 1022703, EPI_ISL_1022706 to 1022707, EPI_ISL_1022711 to 1022715, EPI_ISL_1022719, EPI_ISL_1022723 to 1022724, EPI_ISL_1022726, EPI_ISL_1022728 to 1022729, EPI_ISL_1022731, EPI_ISL_1022736, EPI_ISL_1022738, EPI_ISL_1022746 to 1022747, EPI_ISL_1022750, EPI_ISL_1022753 to 1022758, EPI_ISL_1022760, EPI_ISL_1022764, EPI_ISL_1022768, EPI_ISL_1022771, EPI_ISL_1022774 to 1022780, EPI_ISL_1022786 to 1022787, EPI_ISL_1022790, EPI_ISL_1022793, EPI_ISL_1022795, EPI_ISL_1022797, EPI_ISL_1022799, EPI_ISL_1022803, EPI_ISL_1022805, EPI_ISL_1022810, EPI_ISL_1022813 to 1022814, EPI_ISL_1022817 to 1022819, EPI_ISL_1022822 to 1022823, EPI_ISL_1022826 to 1022827, EPI_ISL_1022830 to 1022836, EPI_ISL_1022838, EPI_ISL_1022840 to 1022842, EPI_ISL_1022845 to 1022846, EPI_ISL_1022849 to 1022851, EPI_ISL_1022854 to 1022855, EPI_ISL_1022858 to 1022860, EPI_ISL_1022862, EPI_ISL_1022866 to 1022867, EPI_ISL_1022870 to 1022871, EPI_ISL_1022874 to 1022875, EPI_ISL_1022877, EPI_ISL_1022882, EPI_ISL_1022886 to 1022887, EPI_ISL_1022891, EPI_ISL_1022893, EPI_ISL_1022897 to 1022898, EPI_ISL_1022901, EPI_ISL_1022903, EPI_ISL_1022906, EPI_ISL_1022908 to 1022911, EPI_ISL_1022913, EPI_ISL_1022916 to 1022917, EPI_ISL_1022922 to 1022924, EPI_ISL_1022926, EPI_ISL_1022929 to 1022932, EPI_ISL_1022934, EPI_ISL_1022936 to 1022937, EPI_ISL_1022939, EPI_ISL_1022942, EPI_ISL_1022944, EPI_ISL_1022946 to 1022947, EPI_ISL_1022949, EPI_ISL_1022953, EPI_ISL_1022957 to 1022960, EPI_ISL_1022963 to 1022964, EPI_ISL_1022970, EPI_ISL_1022973 to 1022974, EPI_ISL_1022977 to 1022979, EPI_ISL_1022981, EPI_ISL_1022986, EPI_ISL_1022988, EPI_ISL_1022990 to 1022992, EPI_ISL_1022998 to 1022999, EPI_ISL_1023003, EPI_ISL_1023007 to 1023010, EPI_ISL_1023013, EPI_ISL_1023016, EPI_ISL_1023020 to 1023021, EPI_ISL_1023024 to 1023025, EPI_ISL_1023033 to 1023034, EPI_ISL_1023036, EPI_ISL_1023039, EPI_ISL_1023042, EPI_ISL_1023046 to 1023048, EPI_ISL_1023050 to 1023051, EPI_ISL_1023055 to 1023056, EPI_ISL_1023059, EPI_ISL_1023061 to 1023063, EPI_ISL_1023069, EPI_ISL_1023075, EPI_ISL_1023077 to 1023079, EPI_ISL_1023081, EPI_ISL_1023083 to 1023084, EPI_ISL_1023086, EPI_ISL_1023090 to 1023091, EPI_ISL_1023093, EPI_ISL_1023095 to 1023096, EPI_ISL_1023098, EPI_ISL_1023103 to 1023104, EPI_ISL_1023106, EPI_ISL_1023108, EPI_ISL_1023111, EPI_ISL_1023117, EPI_ISL_1023119, EPI_ISL_1023127 to 1023128, EPI_ISL_1023130, EPI_ISL_1023138 to 1023139, EPI_ISL_1023141 to 1023146, EPI_ISL_1023148, EPI_ISL_1023150, EPI_ISL_1023152, EPI_ISL_1023154 to 1023155, EPI_ISL_1023157 to 1023158, EPI_ISL_1023160 to 1023162, EPI_ISL_1023165 to 1023166, EPI_ISL_1023168 to 1023169, EPI_ISL_1023171 to 1023174, EPI_ISL_1023176 to 1023177, EPI_ISL_1023179 to 1023180, EPI_ISL_1023182, EPI_ISL_1023187, EPI_ISL_1023190, EPI_ISL_1023192 to 1023194, EPI_ISL_1023196, EPI_ISL_1023198 to 1023199, EPI_ISL_1023201, EPI_ISL_1023203 to 1023206, EPI_ISL_1023209, EPI_ISL_1023212, EPI_ISL_1023214 to 1023218, EPI_ISL_1023220, EPI_ISL_1023223 to 1023224, EPI_ISL_1023226, EPI_ISL_1023228, EPI_ISL_1023230, EPI_ISL_1023232 to 1023234, EPI_ISL_1023236, EPI_ISL_1023239, EPI_ISL_1023241, EPI_ISL_1023243 to 1023244, EPI_ISL_1023247 to 1023251, EPI_ISL_1023254 to 1023255, EPI_ISL_1023257, EPI_ISL_1023259 to 1023260, EPI_ISL_1023262, EPI_ISL_1023266, EPI_ISL_1023271, EPI_ISL_1023273, EPI_ISL_1023275, EPI_ISL_1023277 to 1023278, EPI_ISL_1023280, EPI_ISL_1023283, EPI_ISL_1023285 to 1023286, EPI_ISL_1023288 to 1023289, EPI_ISL_1023292 to 1023293, EPI_ISL_1023299, EPI_ISL_1023303 to 1023304, EPI_ISL_1023306, EPI_ISL_1023308, EPI_ISL_1023310 to 1023313, EPI_ISL_1023315 to 1023316, EPI_ISL_1023318, EPI_ISL_1023324 to 1023325, EPI_ISL_1023327 to 1023330, EPI_ISL_1023332, EPI_ISL_1023336, EPI_ISL_1023338 to 1023339, EPI_ISL_1023347 to 1023349, EPI_ISL_1023351 to 1023353, EPI_ISL_1023356 to 1023357, EPI_ISL_1023361 to 1023362, EPI_ISL_1023365 to 1023366, EPI_ISL_1023368 to 1023369, EPI_ISL_1023377, EPI_ISL_1023379, EPI_ISL_1023382, EPI_ISL_1023394, EPI_ISL_1023398 to 1023401, EPI_ISL_1023403 to 1023404, EPI_ISL_1023411 to 1023412, EPI_ISL_1023418 to 1023422, EPI_ISL_1023426 to 1023429, EPI_ISL_1023434, EPI_ISL_1023438, EPI_ISL_1023440, EPI_ISL_1023444, EPI_ISL_1023455, EPI_ISL_1023460, EPI_ISL_1023462, EPI_ISL_1023466, EPI_ISL_1023475 to 1023482, EPI_ISL_1023484, EPI_ISL_1023486 to 1023487, EPI_ISL_1023489 to 1023490, EPI_ISL_1023495, EPI_ISL_1023497, EPI_ISL_1023500, EPI_ISL_1023502 to 1023503, EPI_ISL_1023506, EPI_ISL_1023509, EPI_ISL_1023516 to 1023517, EPI_ISL_1023520, EPI_ISL_1023523, EPI_ISL_1023525 to 1023526, EPI_ISL_1023530, EPI_ISL_1023532, EPI_ISL_1023535 to 1023536, EPI_ISL_1023540, EPI_ISL_1023545 to 1023546, EPI_ISL_1023548 to 1023549, EPI_ISL_1023562, EPI_ISL_1023566, EPI_ISL_1023568, EPI_ISL_1023570 to 1023571, EPI_ISL_1023574, EPI_ISL_1023577 to 1023580, EPI_ISL_1023596, EPI_ISL_1023598, EPI_ISL_1023613, EPI_ISL_1023616, EPI_ISL_1023619 to 1023620, EPI_ISL_1023622 to 1023624, EPI_ISL_1023628 to 1023629, EPI_ISL_1023632 to 1023633, EPI_ISL_1023639 to 1023640, EPI_ISL_1023646 to 1023649, EPI_ISL_1023653 to 1023654, EPI_ISL_1023660, EPI_ISL_1023664, EPI_ISL_1023667, EPI_ISL_1023670, EPI_ISL_1023674, EPI_ISL_1023678, EPI_ISL_1023682, EPI_ISL_1023684, EPI_ISL_1023694, EPI_ISL_1023702, EPI_ISL_1023705, EPI_ISL_1023728, EPI_ISL_1023730 to 1023732, EPI_ISL_1023735 to 1023736, EPI_ISL_1023738, EPI_ISL_1023744, EPI_ISL_1023747 to 1023748, EPI_ISL_1023751, EPI_ISL_1023753, EPI_ISL_1023756, EPI_ISL_1023758, EPI_ISL_1023760, EPI_ISL_1023762, EPI_ISL_1023771, EPI_ISL_1023774 to 1023777, EPI_ISL_1023779 to 1023780, EPI_ISL_1023782, EPI_ISL_1023785, EPI_ISL_1023789, EPI_ISL_1023793, EPI_ISL_1023799, EPI_ISL_1023802, EPI_ISL_1023806, EPI_ISL_1023808, EPI_ISL_1023814, EPI_ISL_1023817, EPI_ISL_1023819, EPI_ISL_1023821, EPI_ISL_1023823, EPI_ISL_1023828, EPI_ISL_1023830, EPI_ISL_1023836, EPI_ISL_1023840, EPI_ISL_1023844, EPI_ISL_1023851 to 1023852, EPI_ISL_1023857 to 1023859, EPI_ISL_1023863, EPI_ISL_1023867 to 1023870, EPI_ISL_1023872 to 1023875, EPI_ISL_1023881, EPI_ISL_1023888 to 1023891, EPI_ISL_1023893, EPI_ISL_1023897, EPI_ISL_1023899 to 1023900, EPI_ISL_1023903 to 1023904, EPI_ISL_1023914 to 1023915, EPI_ISL_1023919, EPI_ISL_1023921 to 1023922, EPI_ISL_1023925, EPI_ISL_1023928, EPI_ISL_1023930, EPI_ISL_1023933, EPI_ISL_1023935, EPI_ISL_1023938 to 1023940, EPI_ISL_1023942, EPI_ISL_1023944, EPI_ISL_1023953, EPI_ISL_1023956 to 1023958, EPI_ISL_1023960, EPI_ISL_1023963, EPI_ISL_1023965 to 1023966, EPI_ISL_1023972 to 1023973, EPI_ISL_1023976, EPI_ISL_1023979, EPI_ISL_1023981, EPI_ISL_1023988, EPI_ISL_1023990 to 1023993, EPI_ISL_1024000, EPI_ISL_1024002, EPI_ISL_1024004 to 1024005, EPI_ISL_1024007, EPI_ISL_1024010 to 1024011, EPI_ISL_1024013, EPI_ISL_1024018, EPI_ISL_1024022, EPI_ISL_1024024, EPI_ISL_1024027 to 1024029, EPI_ISL_1024032, EPI_ISL_1024035, EPI_ISL_1024037, EPI_ISL_1024039, EPI_ISL_1024045, EPI_ISL_1024047, EPI_ISL_1024051 to 1024052, EPI_ISL_1024057 to 1024059, EPI_ISL_1024064, EPI_ISL_1024071, EPI_ISL_1024079 to 1024080, EPI_ISL_1024083, EPI_ISL_1024086, EPI_ISL_1024090, EPI_ISL_1024094 to 1024095, EPI_ISL_1024099 to 1024100, EPI_ISL_1024103 to 1024104, EPI_ISL_1024107 to 1024108, EPI_ISL_1024110, EPI_ISL_1024112 to 1024114, EPI_ISL_1024118, EPI_ISL_1024123, EPI_ISL_1024125, EPI_ISL_1024127 to 1024128, EPI_ISL_1024132 to 1024133, EPI_ISL_1024140, EPI_ISL_1024142 to 1024143, EPI_ISL_1024150, EPI_ISL_1024154 to 1024156, EPI_ISL_1024163 to 1024164, EPI_ISL_1024166, EPI_ISL_1024169 to 1024170, EPI_ISL_1024172, EPI_ISL_1024178, EPI_ISL_1024180, EPI_ISL_1024183, EPI_ISL_1024186, EPI_ISL_1024192, EPI_ISL_1024194 to 1024195, EPI_ISL_1024197 to 1024198, EPI_ISL_1024202, EPI_ISL_1024204 to 1024205, EPI_ISL_1024207, EPI_ISL_1024212, EPI_ISL_1024217 to 1024218, EPI_ISL_1024224, EPI_ISL_1024226, EPI_ISL_1024229 to 1024230, EPI_ISL_1024234 to 1024236, EPI_ISL_1024245 to 1024246, EPI_ISL_1024249, EPI_ISL_1024251, EPI_ISL_1024257, EPI_ISL_1024260, EPI_ISL_1024266, EPI_ISL_1024270, EPI_ISL_1024273, EPI_ISL_1024277, EPI_ISL_1024280, EPI_ISL_1024290, EPI_ISL_1024293, EPI_ISL_1024296, EPI_ISL_1024299, EPI_ISL_1024301, EPI_ISL_1024303, EPI_ISL_1024318 to 1024319, EPI_ISL_1024321, EPI_ISL_1024324, EPI_ISL_1024327, EPI_ISL_1024330, EPI_ISL_1024332, EPI_ISL_1024339 to 1024340, EPI_ISL_1024343, EPI_ISL_1024345, EPI_ISL_1024347, EPI_ISL_1024353, EPI_ISL_1024355 to 1024356, EPI_ISL_1024360, EPI_ISL_1024366 to 1024367, EPI_ISL_1024371 to 1024372, EPI_ISL_1024377, EPI_ISL_1024379, EPI_ISL_1024383, EPI_ISL_1024396, EPI_ISL_1024399, EPI_ISL_1024402, EPI_ISL_1024410, EPI_ISL_1024413, EPI_ISL_1024416, EPI_ISL_1024418 to 1024420, EPI_ISL_1024426, EPI_ISL_1024431, EPI_ISL_1024434, EPI_ISL_1024436, EPI_ISL_1024440, EPI_ISL_1024442 to 1024443, EPI_ISL_1024449, EPI_ISL_1024451, EPI_ISL_1024454, EPI_ISL_1024459, EPI_ISL_1024461, EPI_ISL_1024466, EPI_ISL_1024470, EPI_ISL_1024475, EPI_ISL_1024480, EPI_ISL_1024487, EPI_ISL_1024489 to 1024490, EPI_ISL_1024503, EPI_ISL_1024505, EPI_ISL_1024511 to 1024512, EPI_ISL_1024514, EPI_ISL_1024531, EPI_ISL_1024536, EPI_ISL_1024547, EPI_ISL_1024554, EPI_ISL_1024556, EPI_ISL_1024558, EPI_ISL_1024561, EPI_ISL_1024565, EPI_ISL_1024567, EPI_ISL_1024569 to 1024570, EPI_ISL_1024572, EPI_ISL_1024582 to 1024584, EPI_ISL_1024588, EPI_ISL_1024590, EPI_ISL_1024594 to 1024595, EPI_ISL_1024597, EPI_ISL_1024602, EPI_ISL_1024608, EPI_ISL_1024613 to 1024614, EPI_ISL_1024626, EPI_ISL_1024628, EPI_ISL_1024630, EPI_ISL_1024639 to 1024640, EPI_ISL_1024642, EPI_ISL_1024650 to 1024651, EPI_ISL_1024653 to 1024654, EPI_ISL_1024658 to 1024660, EPI_ISL_1024662, EPI_ISL_1024666, EPI_ISL_1024678, EPI_ISL_1024683, EPI_ISL_1024692 to 1024694, EPI_ISL_1024698 to 1024699, EPI_ISL_1024702, EPI_ISL_1024706, EPI_ISL_1024710 to 1024712, EPI_ISL_1024718, EPI_ISL_1024720, EPI_ISL_1024725, EPI_ISL_1024728, EPI_ISL_1024730, EPI_ISL_1024733, EPI_ISL_1024737, EPI_ISL_1024745, EPI_ISL_1024755 to 1024756, EPI_ISL_1024759, EPI_ISL_1024761, EPI_ISL_1024766, EPI_ISL_1024774, EPI_ISL_1024788, EPI_ISL_1024792, EPI_ISL_1024795 to 1024796, EPI_ISL_1024799, EPI_ISL_1024802 to 1024803, EPI_ISL_1024806 to 1024807, EPI_ISL_1024817 to 1024818, EPI_ISL_1024821 to 1024822, EPI_ISL_1024832, EPI_ISL_1024834, EPI_ISL_1024841 to 1024843, EPI_ISL_1024846 to 1024847, EPI_ISL_1024853, EPI_ISL_1024855, EPI_ISL_1024862 to 1024863, EPI_ISL_1024869, EPI_ISL_1024875 to 1024876, EPI_ISL_1024879 to 1024880, EPI_ISL_1024882, EPI_ISL_1024888, EPI_ISL_1024891, EPI_ISL_1024893 to 1024894, EPI_ISL_1024898, EPI_ISL_1024900, EPI_ISL_1024902, EPI_ISL_1024911, EPI_ISL_1024917, EPI_ISL_1024919, EPI_ISL_1024923 to 1024926, EPI_ISL_1024931 to 1024932, EPI_ISL_1024935, EPI_ISL_1024939 to 1024941, EPI_ISL_1024943 to 1024944, EPI_ISL_1024949, EPI_ISL_1024955, EPI_ISL_1024960, EPI_ISL_1024963 to 1024964, EPI_ISL_1024970, EPI_ISL_1024982, EPI_ISL_1024984, EPI_ISL_1025018, EPI_ISL_1025021, EPI_ISL_1025034, EPI_ISL_1025063, EPI_ISL_1025067, EPI_ISL_1025075, EPI_ISL_1025193, EPI_ISL_1025200, EPI_ISL_1025208, EPI_ISL_1025228, EPI_ISL_1025247, EPI_ISL_1025289, EPI_ISL_1025302, EPI_ISL_1025306, EPI_ISL_1025311, EPI_ISL_1025337, EPI_ISL_1025528, EPI_ISL_1025587, EPI_ISL_1025716, EPI_ISL_1025735, EPI_ISL_1025795, EPI_ISL_1025892, EPI_ISL_1025938

see above	Department of Virus and Microbiological Special Diagnostics, Statens Serum Institut, Copenhagen, Denmark	Aalborg University	Danish Covid-19 Genome Consortium

EPI_ISL_668601 to 668611, EPI_ISL_668635 to 668641, EPI_ISL_668663 to 668715, EPI_ISL_668718 to 668726, EPI_ISL_668837, EPI_ISL_668853 to 669457, EPI_ISL_669537 to 669546, EPI_ISL_669549 to 669556, EPI_ISL_669596 to 669600, EPI_ISL_669636 to 669730, EPI_ISL_669733 to 669750, EPI_ISL_669754 to 669755, EPI_ISL_669817, EPI_ISL_669834 to 670457, EPI_ISL_670523 to 670527, EPI_ISL_670529 to 670531, EPI_ISL_670552 to 670557, EPI_ISL_670576 to 670665, EPI_ISL_670667 to 670674, EPI_ISL_670680 to 670681, EPI_ISL_670746 to 670747, EPI_ISL_670772 to 671269, EPI_ISL_682353 to 682354, EPI_ISL_682357 to 682358, EPI_ISL_682361 to 682367, EPI_ISL_682369 to 682372, EPI_ISL_682374 to 682375, EPI_ISL_682378 to 682387, EPI_ISL_682389, EPI_ISL_682391, EPI_ISL_682395, EPI_ISL_682397 to 682398, EPI_ISL_682401, EPI_ISL_682404, EPI_ISL_682406, EPI_ISL_682409 to 682410, EPI_ISL_682412 to 682413, EPI_ISL_682416, EPI_ISL_682418, EPI_ISL_682420 to 682425, EPI_ISL_682427 to 682429, EPI_ISL_682431 to 682438, EPI_ISL_682440 to 682441, EPI_ISL_682445 to 682452, EPI_ISL_682455 to 682464, EPI_ISL_682468, EPI_ISL_682470, EPI_ISL_682472, EPI_ISL_682474 to 682479, EPI_ISL_682481 to 682482, EPI_ISL_682486 to 682489, EPI_ISL_682491 to 682492, EPI_ISL_682494 to 682496, EPI_ISL_682500 to 682505, EPI_ISL_682510, EPI_ISL_682512, EPI_ISL_682514 to 682520, EPI_ISL_682522 to 682535, EPI_ISL_682537 to 682541, EPI_ISL_682543, EPI_ISL_682545 to 682548, EPI_ISL_682552 to 682557, EPI_ISL_682562, EPI_ISL_682564, EPI_ISL_682566 to 682568, EPI_ISL_682571, EPI_ISL_682576 to 682578, EPI_ISL_682580 to 682581, EPI_ISL_682583 to 682587, EPI_ISL_682589, EPI_ISL_682591 to 682601, EPI_ISL_682604 to 682610, EPI_ISL_682613, EPI_ISL_682615 to 682618, EPI_ISL_682620 to 682625, EPI_ISL_682627 to 682629, EPI_ISL_682632, EPI_ISL_682634 to 682635, EPI_ISL_682637 to 682638, EPI_ISL_682641 to 682642, EPI_ISL_682644, EPI_ISL_682646 to 682649, EPI_ISL_682651, EPI_ISL_682654 to 682657, EPI_ISL_682661 to 682664, EPI_ISL_682666 to 682667, EPI_ISL_682670, EPI_ISL_682672 to 682674, EPI_ISL_682679 to 682683, EPI_ISL_682685, EPI_ISL_682687, EPI_ISL_682689 to 682692, EPI_ISL_682694, EPI_ISL_682696 to 682699, EPI_ISL_682702 to 682703, EPI_ISL_682705 to 682713, EPI_ISL_682717 to 682724, EPI_ISL_682726 to 682728, EPI_ISL_682730 to 682734, EPI_ISL_682736, EPI_ISL_682741 to 682742, EPI_ISL_682744, EPI_ISL_682746 to 682754, EPI_ISL_682757 to 682759, EPI_ISL_682761, EPI_ISL_682763 to 682764, EPI_ISL_682766, EPI_ISL_682768 to 682769, EPI_ISL_682772 to 682775, EPI_ISL_682782, EPI_ISL_682784 to 682789, EPI_ISL_682792 to 682793, EPI_ISL_682795 to 682796, EPI_ISL_682798, EPI_ISL_682800 to 682806, EPI_ISL_682809 to 682813, EPI_ISL_682815, EPI_ISL_682817, EPI_ISL_682819 to 682820, EPI_ISL_682822 to 682824, EPI_ISL_682827 to 682828, EPI_ISL_682831, EPI_ISL_682833, EPI_ISL_682835 to 682838, EPI_ISL_682840 to 682841, EPI_ISL_682844 to 682846, EPI_ISL_682848 to 682851, EPI_ISL_682854 to 682859, EPI_ISL_682862, EPI_ISL_682864, EPI_ISL_682866 to 682871, EPI_ISL_682874, EPI_ISL_682876, EPI_ISL_682878, EPI_ISL_682880 to 682881, EPI_ISL_682883, EPI_ISL_682885 to 682886, EPI_ISL_682892 to 682895, EPI_ISL_682897 to 682905, EPI_ISL_682907 to 682908, EPI_ISL_682910, EPI_ISL_682913 to 682914, EPI_ISL_682916, EPI_ISL_682919 to 682922, EPI_ISL_682925, EPI_ISL_682927 to 682928, EPI_ISL_682933 to 682936, EPI_ISL_682939 to 682942, EPI_ISL_682944 to 682948, EPI_ISL_682951 to 682954, EPI_ISL_682958, EPI_ISL_682961 to 682976, EPI_ISL_682979, EPI_ISL_682981, EPI_ISL_682984 to 682988, EPI_ISL_682990, EPI_ISL_682993 to 682995, EPI_ISL_682997 to 682998, EPI_ISL_683000, EPI_ISL_683003 to 683005, EPI_ISL_683009 to 683010, EPI_ISL_683023 to 683025, EPI_ISL_683041, EPI_ISL_683085, EPI_ISL_683087, EPI_ISL_683090, EPI_ISL_683119, EPI_ISL_683125, EPI_ISL_683138, EPI_ISL_683172, EPI_ISL_683175 to 683177, EPI_ISL_683188 to 683189, EPI_ISL_683215 to 683218, EPI_ISL_683243, EPI_ISL_683246 to 683248, EPI_ISL_683270 to 683273, EPI_ISL_683280, EPI_ISL_683298 to 683299, EPI_ISL_683306 to 683307, EPI_ISL_683309, EPI_ISL_683319 to 683321, EPI_ISL_711233 to 711272, EPI_ISL_711286 to 711298, EPI_ISL_711301 to 711383, EPI_ISL_711388 to 711390, EPI_ISL_711394, EPI_ISL_711445 to 712059, EPI_ISL_712249 to 712304, EPI_ISL_712321 to 712337, EPI_ISL_712340 to 712416, EPI_ISL_712420 to 712426, EPI_ISL_712455 to 712567, EPI_ISL_712569 to 713097, EPI_ISL_713270 to 713289, EPI_ISL_713291 to 713294, EPI_ISL_713315 to 713328, EPI_ISL_713331 to 713423, EPI_ISL_713425 to 713429, EPI_ISL_713513 to 714097, EPI_ISL_714259 to 714271, EPI_ISL_714289 to 714298, EPI_ISL_714300 to 714364, EPI_ISL_714366 to 714367, EPI_ISL_714372 to 714377, EPI_ISL_714379 to 714380, EPI_ISL_714478 to 714937, EPI_ISL_747612 to 747614, EPI_ISL_747616 to 747635, EPI_ISL_747661 to 747720, EPI_ISL_747728 to 747730, EPI_ISL_747756 to 748137, EPI_ISL_748281 to 748324, EPI_ISL_748359 to 748471, EPI_ISL_748484 to 748492, EPI_ISL_748507, EPI_ISL_748532 to 748666, EPI_ISL_748668 to 749035, EPI_ISL_749037 to 749147, EPI_ISL_749296 to 749357, EPI_ISL_749379 to 749473, EPI_ISL_749475 to 749483, EPI_ISL_749493 to 749496, EPI_ISL_749522, EPI_ISL_749542 to 749705, EPI_ISL_749707 to 749905, EPI_ISL_749907 to 750107, EPI_ISL_750109 to 750160, EPI_ISL_750297 to 750368, EPI_ISL_750371 to 750372, EPI_ISL_750393 to 750429, EPI_ISL_750431 to 750485, EPI_ISL_750494 to 750497, EPI_ISL_750503, EPI_ISL_750512, EPI_ISL_750537 to 750819, EPI_ISL_750821 to 751010, EPI_ISL_751012 to 751183, EPI_ISL_757451 to 757462, EPI_ISL_757465 to 757469, EPI_ISL_757471 to 757491, EPI_ISL_757494 to 757495, EPI_ISL_757510 to 757700, EPI_ISL_757877 to 757893, EPI_ISL_757896 to 757944, EPI_ISL_757960 to 757973, EPI_ISL_757975 to 758065, EPI_ISL_758073 to 758082, EPI_ISL_758090 to 758093, EPI_ISL_758103 to 758104, EPI_ISL_758128 to 758700, EPI_ISL_758869 to 758944, EPI_ISL_758970 to 759040, EPI_ISL_759046 to 759059, EPI_ISL_759065, EPI_ISL_759075 to 759076, EPI_ISL_759104 to 759700, EPI_ISL_792764 to 792765, EPI_ISL_792797, EPI_ISL_792900, EPI_ISL_792913, EPI_ISL_792919, EPI_ISL_792976, EPI_ISL_792982, EPI_ISL_792986, EPI_ISL_793179 to 793201, EPI_ISL_793300, EPI_ISL_793302, EPI_ISL_793306, EPI_ISL_793399, EPI_ISL_793423, EPI_ISL_793548, EPI_ISL_793576, EPI_ISL_793693, EPI_ISL_793718, EPI_ISL_793783, EPI_ISL_793869, EPI_ISL_793897, EPI_ISL_793927, EPI_ISL_793970, EPI_ISL_794501, EPI_ISL_794545, EPI_ISL_794549, EPI_ISL_794930, EPI_ISL_794958, EPI_ISL_794964, EPI_ISL_794967, EPI_ISL_794983, EPI_ISL_795004, EPI_ISL_795010, EPI_ISL_795189 to 795207, EPI_ISL_795599, EPI_ISL_795710, EPI_ISL_795727, EPI_ISL_795814, EPI_ISL_795837, EPI_ISL_795859, EPI_ISL_795866, EPI_ISL_795881, EPI_ISL_795889, EPI_ISL_795997, EPI_ISL_815492 to 815500, EPI_ISL_815554 to 815555, EPI_ISL_815635, EPI_ISL_815816, EPI_ISL_815901, EPI_ISL_817315 to 817328, EPI_ISL_817330 to 817332, EPI_ISL_817584, EPI_ISL_817587, EPI_ISL_817645, EPI_ISL_817653, EPI_ISL_817656, EPI_ISL_817745, EPI_ISL_817755, EPI_ISL_817886, EPI_ISL_817888, EPI_ISL_817890, EPI_ISL_817916, EPI_ISL_818266 to 818300, EPI_ISL_818715, EPI_ISL_818857, EPI_ISL_818888, EPI_ISL_818892, EPI_ISL_818912, EPI_ISL_818930, EPI_ISL_818943, EPI_ISL_819043, EPI_ISL_819080, EPI_ISL_819119, EPI_ISL_843203, EPI_ISL_843226, EPI_ISL_843254, EPI_ISL_843275, EPI_ISL_843306, EPI_ISL_843372, EPI_ISL_843385, EPI_ISL_843389, EPI_ISL_843391, EPI_ISL_843422, EPI_ISL_843460, EPI_ISL_843471, EPI_ISL_843485, EPI_ISL_843553, EPI_ISL_843594, EPI_ISL_843613, EPI_ISL_843622, EPI_ISL_843624, EPI_ISL_843643, EPI_ISL_843710, EPI_ISL_843749, EPI_ISL_843790, EPI_ISL_843826, EPI_ISL_843842, EPI_ISL_843849, EPI_ISL_843880, EPI_ISL_843908 to 843909, EPI_ISL_843928, EPI_ISL_843971, EPI_ISL_844074, EPI_ISL_844105, EPI_ISL_844175, EPI_ISL_844328, EPI_ISL_844365, EPI_ISL_844393, EPI_ISL_844399, EPI_ISL_844415, EPI_ISL_844424, EPI_ISL_844431, EPI_ISL_844436, EPI_ISL_844523, EPI_ISL_844716 to 844763, EPI_ISL_844979, EPI_ISL_845183, EPI_ISL_845371, EPI_ISL_845375, EPI_ISL_845498, EPI_ISL_845530, EPI_ISL_847180 to 847219, EPI_ISL_847504

see above	Department of Virus and Microbiological Special Diagnostics, Statens Serum Institut, Copenhagen, Denmark	Albertsen Lab, Department of Chemistry and Bioscience, Aalborg University, Denmark	Danish Covid-19 Genome Consortium
EPI_ISL_641399, EPI_ISL_641414, EPI_ISL_641419 to 641423, EPI_ISL_641516	Department of Virus and Microbiological Special Diagnostics, Statens Serum Institut, Copenhagen, Denmark	Albertsen lab, Department of Chemistry and Bioscience, Aalborg University, Denmark	Jannik Fonager; Morten Rasmussen; Thomas Bruun Rasmussen

EPI_ISL_429335, EPI_ISL_429337, EPI_ISL_429339 to 429346, EPI_ISL_429348 to 429358, EPI_ISL_429360 to 429361, EPI_ISL_429364, EPI_ISL_429368 to 429379, EPI_ISL_429381 to 429387, EPI_ISL_429395 to 429396, EPI_ISL_429399, EPI_ISL_429402, EPI_ISL_429404 to 429410, EPI_ISL_429414, EPI_ISL_429420 to 429421, EPI_ISL_429424 to 429428, EPI_ISL_429431, EPI_ISL_429433 to 429434, EPI_ISL_429439 to 429440, EPI_ISL_429447 to 429453, EPI_ISL_429460 to 429461, EPI_ISL_429463 to 429466, EPI_ISL_429468 to 429469, EPI_ISL_429471 to 429474, EPI_ISL_429476 to 429486, EPI_ISL_429488 to 429490, EPI_ISL_429492 to 429493, EPI_ISL_429495 to 429497, EPI_ISL_429499, EPI_ISL_429501 to 429503, EPI_ISL_429505 to 429514, EPI_ISL_429516 to 429521, EPI_ISL_429524 to 429530, EPI_ISL_429533 to 429540, EPI_ISL_429542 to 429567, EPI_ISL_429569 to 429575, EPI_ISL_429577 to 429590, EPI_ISL_436962 to 436963, EPI_ISL_436965, EPI_ISL_436967, EPI_ISL_436969, EPI_ISL_436973, EPI_ISL_436975, EPI_ISL_436978 to 436982, EPI_ISL_436984 to 436991, EPI_ISL_436993, EPI_ISL_436995 to 437001, EPI_ISL_437003 to 437004, EPI_ISL_437006 to 437007, EPI_ISL_437011, EPI_ISL_437013 to 437021, EPI_ISL_437023 to 437033, EPI_ISL_437035 to 437042, EPI_ISL_437628 to 437637, EPI_ISL_437639 to 437640, EPI_ISL_437642 to 437651, EPI_ISL_437653 to 437661, EPI_ISL_437663 to 437664, EPI_ISL_437667 to 437670, EPI_ISL_437672, EPI_ISL_437674, EPI_ISL_437676 to 437679, EPI_ISL_437683, EPI_ISL_444817 to 444820, EPI_ISL_444822 to 444829, EPI_ISL_444831 to 444841, EPI_ISL_444844, EPI_ISL_444846, EPI_ISL_444848 to 444850, EPI_ISL_444852 to 444857, EPI_ISL_444859 to 444864, EPI_ISL_444866 to 444867, EPI_ISL_444869 to 444870, EPI_ISL_444872 to 444875, EPI_ISL_444877, EPI_ISL_444879 to 444881, EPI_ISL_444883 to 444885, EPI_ISL_444887 to 444888, EPI_ISL_444890 to 444906, EPI_ISL_444908 to 444912, EPI_ISL_444915 to 444918, EPI_ISL_444920 to 444931, EPI_ISL_444933 to 444935, EPI_ISL_444937 to 444942, EPI_ISL_444944 to 444950, EPI_ISL_444952, EPI_ISL_444954 to 444961, EPI_ISL_444963 to 444968, EPI_ISL_452101 to 452102

see above	Department of Virus and Microbiological Special Diagnostics, Statens Serum Institut, Copenhagen, Denmark, Artillerivej 5, 2300 Copenahgen S	Albertsen lab, Department of Chemistry and Bioscience, Aalborg University, Denmark	Rasmus Kirkegaard

EPI_ISL_613541 to 613543, EPI_ISL_614419, EPI_ISL_614427 to 614444, EPI_ISL_614448 to 614762, EPI_ISL_614764 to 614888, EPI_ISL_615166 to 615217, EPI_ISL_615220 to 615239, EPI_ISL_615241 to 615244, EPI_ISL_615246 to 615262, EPI_ISL_615264 to 615270, EPI_ISL_615272 to 615295, EPI_ISL_615297, EPI_ISL_615299, EPI_ISL_615302 to 615313, EPI_ISL_615315 to 615317, EPI_ISL_615319 to 615320, EPI_ISL_615322 to 615334, EPI_ISL_615336 to 615344, EPI_ISL_615346 to 615359, EPI_ISL_615361 to 615392, EPI_ISL_615394 to 615396, EPI_ISL_615398 to 615435, EPI_ISL_615437, EPI_ISL_615439 to 615447, EPI_ISL_615450 to 615455, EPI_ISL_615457 to 615461, EPI_ISL_615463 to 615468, EPI_ISL_615470 to 615475, EPI_ISL_615477 to 615499, EPI_ISL_615501 to 615505, EPI_ISL_615507 to 615528, EPI_ISL_615530 to 615542, EPI_ISL_615544 to 615550, EPI_ISL_615552 to 615558, EPI_ISL_615561 to 615566, EPI_ISL_615569, EPI_ISL_615571 to 615572, EPI_ISL_615574 to 615584, EPI_ISL_615586 to 615592, EPI_ISL_615594 to 615605, EPI_ISL_615608 to 615611, EPI_ISL_615613 to 615618, EPI_ISL_615620 to 615635, EPI_ISL_615637 to 615650, EPI_ISL_615654 to 615656, EPI_ISL_615658 to 615660, EPI_ISL_615662 to 615666, EPI_ISL_615668 to 615672, EPI_ISL_615674 to 615677, EPI_ISL_615679 to 615683, EPI_ISL_615685 to 615687, EPI_ISL_615689 to 615695, EPI_ISL_615699 to 615701, EPI_ISL_615703 to 615708, EPI_ISL_615710 to 615712, EPI_ISL_615714 to 615717, EPI_ISL_615719 to 615725, EPI_ISL_615727 to 615728, EPI_ISL_615730 to 615737, EPI_ISL_615739 to 615740, EPI_ISL_615742, EPI_ISL_615744 to 615752, EPI_ISL_615754 to 615774, EPI_ISL_615776 to 615777, EPI_ISL_615779 to 615806, EPI_ISL_615808 to 615838, EPI_ISL_615840 to 615842, EPI_ISL_615844 to 615858, EPI_ISL_615860 to 615873, EPI_ISL_615875 to 615876, EPI_ISL_615879 to 615905, EPI_ISL_615907 to 615914, EPI_ISL_615917 to 615921, EPI_ISL_615923, EPI_ISL_615925 to 615934, EPI_ISL_615937 to 615957, EPI_ISL_615959 to 615962, EPI_ISL_615965 to 615971, EPI_ISL_615973 to 615979, EPI_ISL_615981 to 615982, EPI_ISL_615984 to 615987, EPI_ISL_615989, EPI_ISL_615991 to 615995, EPI_ISL_615997 to 616010, EPI_ISL_616012 to 616033, EPI_ISL_616035 to 616048, EPI_ISL_616050 to 616064, EPI_ISL_616066, EPI_ISL_616068 to 616097, EPI_ISL_616099 to 616106, EPI_ISL_616108 to 616174, EPI_ISL_616176 to 616180, EPI_ISL_616182 to 616189, EPI_ISL_616192, EPI_ISL_616194 to 616201, EPI_ISL_616203 to 616228, EPI_ISL_616230 to 616237, EPI_ISL_616239 to 616251, EPI_ISL_616253 to 616268, EPI_ISL_616270 to 616280, EPI_ISL_616282 to 616291, EPI_ISL_616293 to 616294, EPI_ISL_616296 to 616301, EPI_ISL_616303 to 616312, EPI_ISL_616314 to 616334, EPI_ISL_616336 to 616343, EPI_ISL_616345 to 616356, EPI_ISL_616358 to 616368, EPI_ISL_616370 to 616401, EPI_ISL_616403 to 616407, EPI_ISL_616409 to 616426, EPI_ISL_616428 to 616430, EPI_ISL_616432, EPI_ISL_616434 to 616441, EPI_ISL_616443 to 616458, EPI_ISL_616460 to 616468, EPI_ISL_616470 to 616475, EPI_ISL_616477 to 616479, EPI_ISL_616481 to 616497, EPI_ISL_616499 to 616500, EPI_ISL_616502 to 616532, EPI_ISL_616534 to 616538, EPI_ISL_616540 to 616543, EPI_ISL_616545 to 616582, EPI_ISL_616585 to 616613, EPI_ISL_616615 to 616636, EPI_ISL_616638 to 616646, EPI_ISL_616649 to 616687, EPI_ISL_616689 to 616692, EPI_ISL_616694, EPI_ISL_616696, EPI_ISL_616698 to 616701, EPI_ISL_616703 to 616713, EPI_ISL_616715 to 616723, EPI_ISL_616725 to 616726, EPI_ISL_616728 to 616729, EPI_ISL_616731 to 616740, EPI_ISL_616742 to 616744, EPI_ISL_616747, EPI_ISL_616750, EPI_ISL_616752 to 616756, EPI_ISL_616758 to 616759, EPI_ISL_616763 to 616770, EPI_ISL_616772 to 616774, EPI_ISL_616777, EPI_ISL_616779, EPI_ISL_616781, EPI_ISL_616783 to 616787, EPI_ISL_616789 to 616799, EPI_ISL_616801, EPI_ISL_616803, EPI_ISL_616805 to 616807, EPI_ISL_616809 to 616814, EPI_ISL_616816 to 616818, EPI_ISL_616820 to 616825, EPI_ISL_616827, EPI_ISL_616829 to 616858, EPI_ISL_616860 to 616861, EPI_ISL_616863, EPI_ISL_616865 to 616873, EPI_ISL_616875 to 616903, EPI_ISL_616905, EPI_ISL_616951 to 616956, EPI_ISL_616976 to 617054, EPI_ISL_617077 to 617078, EPI_ISL_617096 to 617414, EPI_ISL_617416 to 617417, EPI_ISL_617419 to 617420, EPI_ISL_617422, EPI_ISL_617424 to 617441, EPI_ISL_617443, EPI_ISL_617446 to 617456, EPI_ISL_617458 to 617465, EPI_ISL_617467, EPI_ISL_617469 to 617473, EPI_ISL_617475 to 617490, EPI_ISL_617492 to 617496, EPI_ISL_617498 to 617503, EPI_ISL_617505 to 617507, EPI_ISL_617509 to 617522, EPI_ISL_617524 to 617528, EPI_ISL_617530 to 617535, EPI_ISL_617537 to 617539, EPI_ISL_617541 to 617563, EPI_ISL_617565 to 617572, EPI_ISL_617574 to 617575, EPI_ISL_617577, EPI_ISL_617579, EPI_ISL_617581 to 617598, EPI_ISL_617600 to 617617, EPI_ISL_617619 to 617637, EPI_ISL_617639 to 617642, EPI_ISL_617644 to 617651, EPI_ISL_617653 to 617654, EPI_ISL_617656 to 617657, EPI_ISL_617659, EPI_ISL_617661 to 617696, EPI_ISL_617698 to 617720, EPI_ISL_617722 to 617731, EPI_ISL_617733 to 617750, EPI_ISL_617752 to 617789, EPI_ISL_617791 to 617795, EPI_ISL_617797 to 617849, EPI_ISL_617851 to 617853, EPI_ISL_617855 to 617866, EPI_ISL_617868 to 617869, EPI_ISL_617871 to 617877, EPI_ISL_617879, EPI_ISL_617881 to 617906, EPI_ISL_617908 to 617917, EPI_ISL_617920 to 617923, EPI_ISL_617926 to 617931, EPI_ISL_617933, EPI_ISL_617935 to 617939, EPI_ISL_617944 to 617952, EPI_ISL_617954 to 617958, EPI_ISL_617962 to 617964, EPI_ISL_617966 to 617968, EPI_ISL_617970 to 617974, EPI_ISL_617976 to 617977, EPI_ISL_617979, EPI_ISL_617981 to 617987, EPI_ISL_617989 to 618006, EPI_ISL_618008 to 618012, EPI_ISL_618014 to 618017, EPI_ISL_618019 to 618032, EPI_ISL_618036 to 618037, EPI_ISL_618039 to 618041, EPI_ISL_618043, EPI_ISL_618045 to 618059, EPI_ISL_618062 to 618074, EPI_ISL_618076 to 618105, EPI_ISL_618108 to 618118, EPI_ISL_618120 to 618122, EPI_ISL_618124 to 618125, EPI_ISL_618128 to 618130, EPI_ISL_618132 to 618137, EPI_ISL_618139, EPI_ISL_618144 to 618150, EPI_ISL_618154 to 618167, EPI_ISL_618169 to 618171, EPI_ISL_618173 to 618192, EPI_ISL_618194 to 618215, EPI_ISL_618217 to 618222, EPI_ISL_618224 to 618244, EPI_ISL_618246 to 618264, EPI_ISL_618266 to 618268, EPI_ISL_618270 to 618273, EPI_ISL_618275 to 618292, EPI_ISL_618294 to 618295, EPI_ISL_618298 to 618329, EPI_ISL_618331, EPI_ISL_618333 to 618348, EPI_ISL_618350 to 618361, EPI_ISL_618363 to 618372, EPI_ISL_618374 to 618387, EPI_ISL_618389 to 618400, EPI_ISL_618402 to 618408, EPI_ISL_618410 to 618412, EPI_ISL_618414 to 618419, EPI_ISL_618421 to 618422, EPI_ISL_618424 to 618425, EPI_ISL_618427 to 618431, EPI_ISL_618433 to 618440, EPI_ISL_618442 to 618444, EPI_ISL_618446 to 618463, EPI_ISL_618465, EPI_ISL_618467 to 618468, EPI_ISL_618470 to 618471, EPI_ISL_618475 to 618482, EPI_ISL_618485, EPI_ISL_618487, EPI_ISL_618489 to 618493, EPI_ISL_618495 to 618503, EPI_ISL_618505, EPI_ISL_618507 to 618508, EPI_ISL_618510 to 618511, EPI_ISL_618513 to 618518, EPI_ISL_618520, EPI_ISL_618522, EPI_ISL_618524 to 618528, EPI_ISL_618530 to 618532, EPI_ISL_618534, EPI_ISL_618536 to 618540, EPI_ISL_618544 to 618545, EPI_ISL_618547 to 618550, EPI_ISL_618552 to 618553, EPI_ISL_618555 to 618570, EPI_ISL_618573 to 618587, EPI_ISL_618590 to 618598, EPI_ISL_618600 to 618617, EPI_ISL_618619 to 618620, EPI_ISL_618622, EPI_ISL_618625 to 618631, EPI_ISL_618633 to 618635, EPI_ISL_618637 to 618645, EPI_ISL_618647 to 618657, EPI_ISL_618659, EPI_ISL_618661 to 618666, EPI_ISL_618668 to 618669, EPI_ISL_618671, EPI_ISL_618673, EPI_ISL_618675 to 618680, EPI_ISL_618682 to 618686, EPI_ISL_618688 to 618694, EPI_ISL_618696, EPI_ISL_618698, EPI_ISL_618701, EPI_ISL_618703 to 618707, EPI_ISL_618709 to 618712, EPI_ISL_618714 to 618720, EPI_ISL_618722, EPI_ISL_618725 to 618726, EPI_ISL_618728 to 618742, EPI_ISL_618744 to 618750, EPI_ISL_618752, EPI_ISL_618754, EPI_ISL_618756 to 618757, EPI_ISL_618759 to 618763, EPI_ISL_618770, EPI_ISL_618772, EPI_ISL_618774 to 618781, EPI_ISL_618783 to 618784, EPI_ISL_618786 to 618787, EPI_ISL_618791 to 618796, EPI_ISL_618798, EPI_ISL_618801 to 618809, EPI_ISL_618811 to 618815, EPI_ISL_618817 to 618825, EPI_ISL_618827, EPI_ISL_618829 to 618840, EPI_ISL_618842 to 618847, EPI_ISL_618849 to 618882, EPI_ISL_618884 to 618904, EPI_ISL_618957 to 618961, EPI_ISL_618974 to 619051, EPI_ISL_619053 to 619059, EPI_ISL_619083 to 619091, EPI_ISL_619112 to 619414, EPI_ISL_619416, EPI_ISL_619420, EPI_ISL_619422 to 619423, EPI_ISL_619425 to 619438, EPI_ISL_619440, EPI_ISL_619442, EPI_ISL_619444 to 619445, EPI_ISL_619447, EPI_ISL_619450 to 619456, EPI_ISL_619459, EPI_ISL_619461 to 619462, EPI_ISL_619465 to 619466, EPI_ISL_619468, EPI_ISL_619471 to 619472, EPI_ISL_619475 to 619478, EPI_ISL_619484 to 619486, EPI_ISL_619489, EPI_ISL_619491 to 619505, EPI_ISL_619508 to 619510, EPI_ISL_619513, EPI_ISL_619515, EPI_ISL_619517, EPI_ISL_619519 to 619524, EPI_ISL_619526, EPI_ISL_619528 to 619531, EPI_ISL_619533 to 619534, EPI_ISL_619536 to 619556, EPI_ISL_619558 to 619577, EPI_ISL_619579, EPI_ISL_619581 to 619583, EPI_ISL_619585 to 619594, EPI_ISL_619599 to 619600, EPI_ISL_619603 to 619606, EPI_ISL_619610, EPI_ISL_619613 to 619616, EPI_ISL_619619, EPI_ISL_619621 to 619632, EPI_ISL_619634 to 619640, EPI_ISL_619645 to 619650, EPI_ISL_619652, EPI_ISL_619654 to 619655, EPI_ISL_619660, EPI_ISL_619662 to 619668, EPI_ISL_619670, EPI_ISL_619673 to 619680, EPI_ISL_619682, EPI_ISL_619684 to 619686, EPI_ISL_619689 to 619697, EPI_ISL_619700 to 619702, EPI_ISL_619704, EPI_ISL_619706 to 619707, EPI_ISL_619709, EPI_ISL_619712 to 619723, EPI_ISL_619726 to 619735, EPI_ISL_619739 to 619740, EPI_ISL_619742, EPI_ISL_619744 to 619748, EPI_ISL_619750, EPI_ISL_619752 to 619756, EPI_ISL_619758 to 619761, EPI_ISL_619763, EPI_ISL_619765 to 619767, EPI_ISL_619769, EPI_ISL_619771 to 619776, EPI_ISL_619778 to 619787, EPI_ISL_619789, EPI_ISL_619791 to 619796, EPI_ISL_619798 to 619806, EPI_ISL_619808, EPI_ISL_619813 to 619814, EPI_ISL_619816 to 619828, EPI_ISL_619830 to 619831, EPI_ISL_619833 to 619837, EPI_ISL_619839 to 619840, EPI_ISL_619842 to 619851, EPI_ISL_619853 to 619862, EPI_ISL_619864 to 619866, EPI_ISL_619868 to 619873, EPI_ISL_619877, EPI_ISL_619879 to 619882, EPI_ISL_619885 to 619887, EPI_ISL_619889, EPI_ISL_619891, EPI_ISL_619893 to 619895, EPI_ISL_619897 to 619899, EPI_ISL_619902 to 619903, EPI_ISL_619905 to 619912, EPI_ISL_619914 to 619941, EPI_ISL_619943 to 619946, EPI_ISL_619949 to 619952, EPI_ISL_619954 to 619962, EPI_ISL_619964 to 619965, EPI_ISL_619967 to 619973, EPI_ISL_619975 to 619976, EPI_ISL_619978 to 619983, EPI_ISL_619986, EPI_ISL_619989, EPI_ISL_619991 to 619997, EPI_ISL_619999 to 620001, EPI_ISL_620003 to 620004, EPI_ISL_620006, EPI_ISL_620010 to 620030, EPI_ISL_620032 to 620040, EPI_ISL_620045 to 620049, EPI_ISL_620052, EPI_ISL_620054 to 620056, EPI_ISL_620058 to 620074, EPI_ISL_620076, EPI_ISL_620078 to 620079, EPI_ISL_620081 to 620091, EPI_ISL_620093 to 620094, EPI_ISL_620097 to 620098, EPI_ISL_620100 to 620105, EPI_ISL_620107, EPI_ISL_620109 to 620115, EPI_ISL_620120, EPI_ISL_620123 to 620128, EPI_ISL_620130, EPI_ISL_620132 to 620136, EPI_ISL_620139 to 620142, EPI_ISL_620145 to 620146, EPI_ISL_620148 to 620151, EPI_ISL_620153 to 620163, EPI_ISL_620167, EPI_ISL_620169 to 620174, EPI_ISL_620176 to 620180, EPI_ISL_620183 to 620187, EPI_ISL_620189, EPI_ISL_620192 to 620198, EPI_ISL_620200 to 620211, EPI_ISL_620213 to 620229, EPI_ISL_620231, EPI_ISL_620236 to 620252, EPI_ISL_620254 to 620264, EPI_ISL_620266, EPI_ISL_620268 to 620275, EPI_ISL_620277 to 620284, EPI_ISL_620286 to 620290, EPI_ISL_620292

to 620296, EPI_ISL_620298 to 620301, EPI_ISL_620303, EPI_ISL_620305 to 620326, EPI_ISL_620328 to 620331, EPI_ISL_620333, EPI_ISL_620335 to 620344, EPI_ISL_620348, EPI_ISL_620350 to 620352, EPI_ISL_620354 to 620372, EPI_ISL_620374, EPI_ISL_620376 to 620381, EPI_ISL_620383 to 620386, EPI_ISL_620389, EPI_ISL_620391 to 620393, EPI_ISL_620396 to 620401, EPI_ISL_620403 to 620404, EPI_ISL_620762 to 620778, EPI_ISL_620780 to 620797, EPI_ISL_620800 to 620803, EPI_ISL_620809 to 620843, EPI_ISL_620845 to 620869, EPI_ISL_620877, EPI_ISL_620879 to 620890, EPI_ISL_620892 to 620906, EPI_ISL_620908 to 620912, EPI_ISL_620914 to 620922, EPI_ISL_620924 to 620930, EPI_ISL_620932 to 620952, EPI_ISL_620954 to 620957, EPI_ISL_620959 to 620971, EPI_ISL_620973 to 620974, EPI_ISL_620976 to 620979, EPI_ISL_620981, EPI_ISL_620983 to 620997, EPI_ISL_620999, EPI_ISL_621001 to 621006, EPI_ISL_621009 to 621010, EPI_ISL_621012 to 621016, EPI_ISL_621018 to 621019, EPI_ISL_621021 to 621027, EPI_ISL_621029 to 621038, EPI_ISL_621040 to 621047, EPI_ISL_621049 to 621054, EPI_ISL_621056 to 621083, EPI_ISL_621086 to 621118, EPI_ISL_621120 to 621126, EPI_ISL_621129 to 621131, EPI_ISL_621133 to 621147, EPI_ISL_621150 to 621151, EPI_ISL_621153 to 621187, EPI_ISL_621191 to 621192, EPI_ISL_621194 to 621200, EPI_ISL_621202 to 621284, EPI_ISL_621287 to 621290, EPI_ISL_621292 to 621310, EPI_ISL_621312 to 621348, EPI_ISL_621350 to 621367, EPI_ISL_621369 to 621370, EPI_ISL_621373 to 621381, EPI_ISL_621383 to 621409, EPI_ISL_621411 to 621414, EPI_ISL_621416 to 621420, EPI_ISL_621422 to 621424, EPI_ISL_621426, EPI_ISL_621428 to 621436, EPI_ISL_621438 to 621453, EPI_ISL_621455 to 621466, EPI_ISL_621468 to 621487, EPI_ISL_621489 to 621532, EPI_ISL_621535 to 621543, EPI_ISL_621545 to 621560, EPI_ISL_621562 to 621591, EPI_ISL_621594 to 621595, EPI_ISL_621597 to 621605, EPI_ISL_621608 to 621610, EPI_ISL_621613 to 621614, EPI_ISL_621616 to 621621, EPI_ISL_621623 to 621629, EPI_ISL_621631 to 621636, EPI_ISL_621638 to 621642, EPI_ISL_621644 to 621646, EPI_ISL_621648 to 621659, EPI_ISL_621661 to 621662, EPI_ISL_621664 to 621665, EPI_ISL_621667 to 621675, EPI_ISL_621677 to 621684, EPI_ISL_621686 to 621695, EPI_ISL_621697 to 621707, EPI_ISL_621709 to 621715, EPI_ISL_621719 to 621722, EPI_ISL_621724 to 621735, EPI_ISL_621737 to 621744, EPI_ISL_621747 to 621753, EPI_ISL_621755 to 621812, EPI_ISL_621814, EPI_ISL_621816 to 621830, EPI_ISL_621832 to 621852, EPI_ISL_621855, EPI_ISL_621857 to 621880, EPI_ISL_621882, EPI_ISL_621884 to 621888, EPI_ISL_621890 to 621897, EPI_ISL_621899 to 621916, EPI_ISL_621918 to 621928, EPI_ISL_621932 to 621933, EPI_ISL_621935 to 621936, EPI_ISL_621941 to 621942, EPI_ISL_621944 to 621945, EPI_ISL_621947 to 621956, EPI_ISL_621959 to 621967, EPI_ISL_621969 to 621972, EPI_ISL_621974 to 621980, EPI_ISL_621982 to 621990, EPI_ISL_621992 to 621993, EPI_ISL_621995 to 622001, EPI_ISL_622003 to 622008, EPI_ISL_622010 to 622012, EPI_ISL_622014 to 622016, EPI_ISL_622018 to 622022, EPI_ISL_622024 to 622028, EPI_ISL_622030 to 622056, EPI_ISL_622059 to 622060, EPI_ISL_622062 to 622064, EPI_ISL_622066 to 622068, EPI_ISL_622070 to 622075, EPI_ISL_622077 to 622101, EPI_ISL_622103 to 622127, EPI_ISL_622129 to 622130, EPI_ISL_622132 to 622159, EPI_ISL_622161 to 622174, EPI_ISL_622177 to 622183, EPI_ISL_622185 to 622194, EPI_ISL_622196 to 622202, EPI_ISL_622204 to 622215, EPI_ISL_622217 to 622218, EPI_ISL_622220 to 622226, EPI_ISL_622229, EPI_ISL_622231 to 622255, EPI_ISL_622257 to 622260, EPI_ISL_622262 to 622317, EPI_ISL_622320 to 622341, EPI_ISL_622343 to 622354, EPI_ISL_622356 to 622358, EPI_ISL_622360 to 622362, EPI_ISL_622364, EPI_ISL_622367 to 622371, EPI_ISL_622373 to 622374, EPI_ISL_622376 to 622381, EPI_ISL_622383 to 622396, EPI_ISL_622398, EPI_ISL_622400 to 622403, EPI_ISL_622407 to 622408, EPI_ISL_622410 to 622416, EPI_ISL_622418 to 622442, EPI_ISL_622444 to 622448, EPI_ISL_622450 to 622460, EPI_ISL_622462 to 622465, EPI_ISL_622467 to 622488, EPI_ISL_622491 to 622496, EPI_ISL_622498 to 622514, EPI_ISL_622516 to 622537, EPI_ISL_622539 to 622546, EPI_ISL_622549 to 622581, EPI_ISL_622583 to 622597, EPI_ISL_622599 to 622610, EPI_ISL_622612 to 622631, EPI_ISL_622633 to 622642, EPI_ISL_622644 to 622648, EPI_ISL_622650, EPI_ISL_622652 to 622668, EPI_ISL_622671, EPI_ISL_622673 to 622675, EPI_ISL_622677 to 622693, EPI_ISL_622695 to 622700, EPI_ISL_622702 to 622712, EPI_ISL_622714 to 622719, EPI_ISL_622721 to 622739, EPI_ISL_622741 to 622760, EPI_ISL_625684, EPI_ISL_625697, EPI_ISL_625703, EPI_ISL_625716, EPI_ISL_625732 to 625741, EPI_ISL_625746, EPI_ISL_625752, EPI_ISL_625756 to 625776, EPI_ISL_625778 to 625839, EPI_ISL_625842, EPI_ISL_625844, EPI_ISL_625846 to 625848, EPI_ISL_625857, EPI_ISL_625872 to 625875, EPI_ISL_625877 to 625878, EPI_ISL_625885, EPI_ISL_625889, EPI_ISL_625892 to 625932, EPI_ISL_625934 to 625936, EPI_ISL_625939 to 625942, EPI_ISL_625945 to 625951, EPI_ISL_625953 to 625954, EPI_ISL_625956 to 625959, EPI_ISL_625961 to 625966, EPI_ISL_625969, EPI_ISL_625971, EPI_ISL_625976 to 625978, EPI_ISL_625981 to 625982, EPI_ISL_625985 to 625988, EPI_ISL_625990 to 625997, EPI_ISL_625999 to 626009, EPI_ISL_626011 to 626210			
see above	Department of Virus and Microbiological Special Diagnostics, Statens Serum Institut, Denmark	Albertsen lab, Department of Chemistry and Bioscience, Aalborg University, Denmark	Danish Corona Genome Consortia; Danish Covid-19 Genome Consortia
EPI_ISL_416140 to 416142	Department of Virus and Microbiological Special diagnostics, Statens Serum Institut, Copenhagen, Denmark.	Statens Serum Institute	Anders Fomsgaard; Maiken Worsoe Rosenstierne; Morten Rasmussen
EPI_ISL_415648, EPI_ISL_416143 to 416144, EPI_ISL_416153	Department of Virus and Microbiological Special diagnostics, Statens Serum Institut, Copenhagen, Denmark.	ViFU	Anders Fomsgaard; Maiken Worsoe Rosenstierne; Morten Rasmussen
EPI_ISL_449791 to 449794, EPI_ISL_590877, EPI_ISL_590953 to 590977, EPI_ISL_591021, EPI_ISL_635120, EPI_ISL_635144, EPI_ISL_635146, EPI_ISL_635166, EPI_ISL_635196, EPI_ISL_668408, EPI_ISL_708044 to 708045, EPI_ISL_708081, EPI_ISL_708131 to 708135, EPI_ISL_759988, EPI_ISL_775272, EPI_ISL_796658, EPI_ISL_796663			
see above	Dept. of Medical Microbiology, Stavanger University Hospital, Helse Stavanger HF	Norwegian Institute of Public Health, Department of Virology	Atiya R Ali; Hilde Elshaug; Hilde Vollan; Iren Löhr; Kamilla Heddeland Instefjord; Karoline Bragstad; Kathrine Stene-Johansen; Marie Paulsen Madsen; Olav Hungnes; Rasmus Riis Kopperud
EPI_ISL_447837	Dept. of Medical Microbiology, Stavanger University Hospital, Helse Stavanger HF,	Norwegian Institute of Public Health, Department of Virology	Hilde Elshaug; Kamilla Heddeland Instefjord; Karoline Bragstad; Kathrine Stene-Johansen; Olav Hungnes; Rasmus Riis Kopperud
EPI_ISL_664106, EPI_ISL_665254 to 665255, EPI_ISL_666593 to 666595, EPI_ISL_666598, EPI_ISL_666602, EPI_ISL_666604, EPI_ISL_666606, EPI_ISL_666610, EPI_ISL_666612			
see above	Dept. of Microbiology and Infection Control, Akershus University Hospital HF	Dept. of Microbiology and Infection Control, Akershus University Hospital HF	Alexander Hesselberg Løvestad; Hege Vangstein Aamot; Nina Handal; Ole Herman Ambur; Silje Bakken Jørgensen
EPI_ISL_410531 to 410532	Dept. of Pathology, National Institute of Infectious Diseases	Pathogen Genomics Center, National Institute of Infectious Diseases	Harutaka Katano; Hideki Hasegawa; Kazuya Shirato; Makoto Kuroda; Makoto Takeda; Motoi Suzuki; Naganori Nao; Shutoku Matsuyama; Tadaki Suzuki; Takaji Wakita; Tsuyoshi Sekizuka
EPI_ISL_408665 to 408667, EPI_ISL_408669	Dept. of Virology III, National Institute of Infectious Diseases	Pathogen Genomics Center, National Institute of Infectious Diseases	Kazuya Shirato; Makoto Kuroda; Makoto Takeda; Naganori Nao; Shutoku Matsuyama; Tsuyoshi Sekizuka
EPI_ISL_459911, EPI_ISL_459913 to 459924, EPI_ISL_459926 to 459927, EPI_ISL_459932 to 459943, EPI_ISL_751245 to 751246, EPI_ISL_751277 to 751313, EPI_ISL_751315 to 751317			
see above	Devki Devi Foundation, a unit of Max Healthcare	CSIR-IGIB/Max	Akansha Tyagi; Akshay Kanakan; Anurag Agrawal; Bansidhar Tarai; Bharathram Uppili; Janani Srinivasa Vasudevan; Mitali Mukerji; Mohammed Faruq; Nishu Tyagi; Pooja Sharma; Poonam Das; Rajesh Pandey#; Ranjeet Maurya; Samreen Siddiqui; Saruchi Wadhwa; Sujeet Jha; Uzma Shamim; Vinita Jha; Vivekanand A
EPI_ISL_699506 to 699509	Diagnostic Virology Laboratory, USDA National Veterinary Services Laboratories	Diagnostic Virology Laboratory, USDA National Veterinary Services Laboratories	Akpalu, Y.; Auckland; Barton Behravesh, C.; C.M.; Davila, E.; E.R.; Fischer; Franzen; G.L.; Ghai; Hamer; I.B.; Jenkins-Moore, M.; K.M.; Killian; L.D.; Love; M.L.; Pauvolid-Correa, A.; R.R.; R.S.; Roundy; S.A.; Spengler, J.; Tang, W.; Torchetti, M.; Zecca
EPI_ISL_437197 to 437203, EPI_ISL_437298 to 437303, EPI_ISL_653916	Diagnostic- and Research Institute of Pathology, Medical University of Graz	Diagnostic- and Research Institute of Pathology, Medical University of Graz	Gregor Gorkiewicz; Karl Kashofer; Martin Zacharias; Peter Regitnig
EPI_ISL_763074 to 763075	Diagnosticos da America - DASA	Instituto Adolfo Lutz, Interdiciplinary Procedures Center, Strategic Laboratory	Claudia Regina Gonçalves; Claudio Tavares Sacchi; Erica Valessa Ramos Gomes; Karoline Rodrigues Campos
EPI_ISL_413573	Dienst Gezondheid & Jeugd Zuid-Holland Zuid	Erasmus Medical Center	Anne van der Linden; Annemiek van der Eijk; Aura Timen; Bas Oude Munnink; Claudia Schapendonk; Corien Swaan; Corine GeurtsvanKessel; David Nieuwenhuijse; Irina Chestakova; Jeroen van Kampen; Jolanda Voermans; Madelief Mollers; Manon Haverkate; Marion Koopmans; Mark Pronk; Mart Stein; Pascal Lexmond; Reina Sikkema; Richard Molenkamp; Sandra Kengne Kamga Mobou; on behalf of the Dutch national COVID-19 response team.
EPI_ISL_751194, EPI_ISL_751196 to 751200	Dienststelle Gesundheit und Sport Kanton Luzern	Institute of Medical Virology, University of Zurich	Alexandra Trkola; Claudia Schmutz; Eva Spieler; Gabriela Ziltener; Jürg Böni; Kevin Steiner; Maryam Zaheri; Michael Huber; Stefan Schmutz; Verena Kufner
EPI_ISL_452234, EPI_ISL_475512, EPI_ISL_475562 to 475563	Din Klinik	The Public Health Agency of Sweden	Anna Risberg; Anna-Malin Linde; Helene Warnborg; Karin Tegmark-Wisell; Maria Lind Karlberg; Mattias Haukland; Mia Brytting; Olov Svartstrom; Oskar Karlsson Lindsjo; Reza Advani; Sandra Broddesson; Theresa Enkirch
EPI_ISL_528753	Dinkes Kota Bogor	School of Life Sciences and Technology & School of Pharmacy-Institut Teknologi Bandung; Molecular Genetics Laboratory-Faculty of Medicine-Universitas Padjadjaran; Laboratorium Kesehatan Provinsi Jawa Barat	Agung Eru Wibowo; Azzania Fibriani; Catur Riani; Cut Nur Cinthia Alamanda; Davin H. E. Setiamarga; Ema Rahmawati; Hammam Riza; Hesti Lina Wiraswati; Husna Nugrahapraja; Irvan Faizal; Lia Faridah; Marselina Irasonia Tan; Rifky Waluyajati Rachman; Rizki Mardian; Ryan Bayusantika Ristandi; Savira Ekawardhani; Sony Solistia Wirawan; Tarwadi; Yunia Sribudiani
EPI_ISL_528748	Dinkes Provinsi Jawa Barat	School of Life Sciences and Technology & School of Pharmacy-Institut Teknologi Bandung; Molecular Genetics Laboratory-Faculty of Medicine-Universitas Padjadjaran; Laboratorium Kesehatan Provinsi Jawa Barat	Adelina Khristiani Rahayu; Agung Eru Wibowo; Azzania Fibriani; Catur Riani; Cut Nur Cinthia Alamanda; Ema Rahmawati; Gusti Ayu Prani Pradani; Hammam Riza; Hesti Lina Wiraswati; Husna Nugrahapraja; Irvan Faizal; Lia Faridah; Marselina Irasonia Tan; Rifky Waluyajati Rachman; Ryan Bayusantika Ristandi; Savira Ekawardhani; Sony Solistia Wirawan; Tarwadi; Yunia Sribudiani
EPI_ISL_467374	Dinkes Samarinda	Eijkman Institute for Molecular Biology, Ministry of Research and Technology/National Agency for Research and Innovation	Amin Soebandrio; David H Muljono; Edison Johar; Frilasita A Yudhaputri; Hidayat Trimarsanto; Khin Saw Myint; Safarina G Malik
EPI_ISL_754241	Dinkes Tasikmalaya	"School of Life Sciences and Technology & School of Pharmacy-Institut Teknologi Bandung; Molecular Genetics Laboratory-Faculty of Medicine-Universitas Padjadjaran; Laboratorium Kesehatan Provinsi Jawa Barat"	Agung Eru Wibowo; Azzania Fibriani; Catur Riani; Cut Nur Cinthia Alamanda; Ema Rahmawati; Hammam Riza; Hesti Lina Wiraswati; Husna Nugrahapraja; Irvan Faizal; Karimatu Khoirunnisa; Lia Faridah; Marselina Irasonia Tan; Miftahul Faridl; Rifky Waluyajati Rachman; Ryan Bayusantika Ristandi; Savira Ekawardhani; Soni Solistia Wirawan; Tarwadi; Yunia Sribudiani
EPI_ISL_961730, EPI_ISL_961734, EPI_ISL_961747	Dipartimento Prevenzione Avezzano-Servizio Igiene epidemiologia Sanità Pubblica	Istituto Zooprofilattico Sperimentale dell'Abruzzo e Molise "G. Caporale"	Ancora M; Calistri P; Cammà C; Curini V; Di Domenico M; Di Pasquale A; Lorusso A; Mangone I; Marcacci M; Puglia I; Rinaldi A; Savini G; Scialabba S
EPI_ISL_591326 to 591335	Dipartimento di Biotecnologie Mediche, University of Siena	Dipartimento di Biotecnologie Mediche, University of Siena	Anichini, G.; Cusi; Gandolfo, C.; M.G.; Pinzauti, D.; Pozzi, G.; Santoro, F.
EPI_ISL_722855 to 722858, EPI_ISL_722872, EPI_ISL_722896 to 722897, EPI_ISL_722899, EPI_ISL_722912	Dipartimento di Scienze Biomediche e Oncologia Umana - Azienda Ospedaliero Universitaria Consorziale Policlinico	Istituto Zooprofilattico Sperimentale della Puglia e della Basilicata	Bianco A.; Capozzi L.; Chironna M.; Del Sambro L.; Loconsole D.; Parisi A.

EPI_ISL_424353 to 424354, EPI_ISL_424983, EPI_ISL_424987, EPI_ISL_454412 to 454415, EPI_ISL_454796 to 454829, EPI_ISL_458002 to 458007, EPI_ISL_458291 to 458297, EPI_ISL_524430			
see above	Dirk Dittmer	Dirk Dittmer	A.B.; A.G.; Aubrey; B.G.; Bailey; C.P.; Caro-Vegas; Caro-Vegas, C.; Dittmer, D.; Eason; F. and Vahrson, W.; J.T.; Juarez, A.; L.J.; Landis; M.B.; McNamara; Mcnamara; Miller; Moorad, R.; Pluta; R.P.; Seltzer; T.A.; Thompson, C.; Vahrson; Vahrson, W.; Villamor; Villamor, F.; W. and Villamor, F.
EPI_ISL_504185, EPI_ISL_515466	Discovery DNA	Discovery DNA	Alice Li; Aneal Khan; Desmond Koo; Dustin Hittel; Leo Dimnik; Marina Kerr
EPI_ISL_640044	District 6 CDC wc DSI	NHLS/UCT	Arash Iranzadeh; Bruna Galvao; Carolyn Williamson; Deelan Doolabh; Diana Hardie; Innocent Mudau; Kruger Marais; Lynn Tyers; Marvin Hsiao; Stephen Korsman
EPI_ISL_436157	District Surveillance Unit	Department of Neurovirology, National Institute of Mental Health and Neuroscience (NIMHANS)	Anita Desai; Chitra Pattabiraman; Harsha PK; Manjunatha Venkataswamy; Ravi Vasanthapuram; Risha Rasheed; Shafeeq S Hameed; Vijayalakshmi Reddy
EPI_ISL_428479 to 428484, EPI_ISL_428486 to 428487, EPI_ISL_486382	District Surveillence Unit	Department of Neurovirology, National Institute of Mental Health and Neuroscience (NIMHANS)	Anita Desai; Chitra Pattabiraman; Harsha PK; Manjunatha Venkataswamy; Ravi Vasanthapuram; Risha Rasheed; Shafeeq S Hameed; Vijayalakshmi Reddy
EPI_ISL_534312	Distrito Sanitario Sul	Instituto Adolfo Lutz, Interdiciplinary Procedures Center, Strategic Laboratory	Claudia Regina Gonçalves; Claudio Tavares Sacchi; Erica Valessa Ramos Gomes
EPI_ISL_583499	Distrito Sanitario Sul Campinas	Instituto Adolfo Lutz, Interdiciplinary Procedures Center, Strategic Laboratory	Claudia Regina Gonçalves; Claudio Tavares Sacchi; Erica Valessa Ramos Gomes; Karoline Rodrigues Campos
EPI_ISL_747245 to 747254, EPI_ISL_747256 to 747281, EPI_ISL_747284 to 747287, EPI_ISL_747289 to 747303, EPI_ISL_747306, EPI_ISL_747308 to 747310, EPI_ISL_747312 to 747317, EPI_ISL_747319 to 747322, EPI_ISL_747324 to 747332, EPI_ISL_747336 to 747341, EPI_ISL_747343 to 747347, EPI_ISL_747349 to 747353, EPI_ISL_747355 to 747361, EPI_ISL_747363 to 747375, EPI_ISL_747377 to 747391, EPI_ISL_747393 to 747403, EPI_ISL_747406 to 747414, EPI_ISL_747416 to 747420, EPI_ISL_747425 to 747433, EPI_ISL_747435 to 747456, EPI_ISL_747458, EPI_ISL_759991 to 760007, EPI_ISL_760009 to 760030, EPI_ISL_760060 to 760072, EPI_ISL_760074 to 760108, EPI_ISL_760110 to 760201, EPI_ISL_760203 to 760230, EPI_ISL_760232 to 760245, EPI_ISL_850653 to 850662, EPI_ISL_1007643 to 1007652, EPI_ISL_1007654 to 1007657			
see above	Division of Emerging Infectious Diseases, Bureau of Infectious Diseases Diagnosis Control, Korea Disease Control and Prevention Agency	Division of Emerging Infectious Diseases, Bureau of Infectious Diseases Diagnosis Control, Korea Disease Control and Prevention Agency	Ae Kyung Park; Chae Young Lee; Chaeyoung Lee; Eun-Jin Kim; Heui Man Kim; Il-Hwan Kim; Jeong-Min Kim; Namjoo Lee; Sang Hee Woo
EPI_ISL_508864 to 508870, EPI_ISL_509222	Division of Infectious Diseases and Hospital Epidemiology, University Hospital Zürich	Institute of Medical Virology, University of Zurich	Aline Wolfensberger; Dana Weissberg; Hugo Sax; Irene A. Abela; Jürg Böni; Maryam Zaheri; Michael Huber; Peter W. Schreiber; Silvana K. Rampini; Verena Kufner
EPI_ISL_413513, EPI_ISL_413515	Division of Infectious Diseases, Department of Internal Medicine, Korea University College of Medicine	Department of Microbiology, Institute for Viral Diseases, College of Medicine, Korea University	Changmin Kang; Cui Chunguang; Gee Eun Lee; Hee Jin Cheong; Heedo Park; Jeonghun Kim; Ji Yun Noh; Jin Gu Yoon; Jin Il Kim; Joon Young Song; Joon-Yong Bae; Jungmin Lee; Juyoung Cho; Kyeong-ryeol Shin; Man-Seong Park; Woo Joo Kim
EPI_ISL_660109 to 660110	Division of Infectious Diseases, Department of Internal Medicine, Yonsei University College of Medicine	Department of biotechnology, Yonsei University	Jae sun Yoon; Jin Young Ahn; Jun Yong Choi*; Jung Ho Kim; Minwoo Kim; Youn-Jung Lee; and Jong-Won Oh*
EPI_ISL_413022 to 413024	Division of Infectious Diseases, University Hospital Zurich	Institute of Medical Virology, University of Zurich	Alexandra Trkola; Andrea Zbinden; Fiona Steiner; Gabriela Ziltener; Jon Huder; Jürg Böni; Maryam Zaheri; Michael Huber; Patrick Redli; Riccarda Capaul; Roberto Speck; Stefan Schmutz; Verena Kufner
EPI_ISL_524475, EPI_ISL_524477, EPI_ISL_524483, EPI_ISL_524485	Division of Infectious Diseases, University Hospital Zürich	Institute of Medical Virology, University of Zurich	Alexandra Trkola; Andrea Zbinden; Fiona Steiner; Gabriela Ziltener; Jon Huder; Jürg Böni; Maryam Zaheri; Michael Huber; Patrick Redli; Riccarda Capaul; Stefan Schmutz; Verena Kufner
EPI_ISL_412869 to 412873, EPI_ISL_425117 to 425118, EPI_ISL_426163 to 426164, EPI_ISL_426166 to 426169, EPI_ISL_426171, EPI_ISL_426173, EPI_ISL_426180 to 426183, EPI_ISL_426187, EPI_ISL_427809 to 427813, EPI_ISL_471425 to 471426, EPI_ISL_471438 to 471455, EPI_ISL_481370 to 481379, EPI_ISL_497951, EPI_ISL_497953 to 497969, EPI_ISL_497971 to 497973, EPI_ISL_497975 to 498053, EPI_ISL_506947 to 506995, EPI_ISL_510547 to 510688, EPI_ISL_514756 to 515050, EPI_ISL_515052 to 515054, EPI_ISL_522465 to 522466, EPI_ISL_522474 to 522475, EPI_ISL_522477 to 522478, EPI_ISL_522484, EPI_ISL_522486 to 522490, EPI_ISL_522498 to 522501, EPI_ISL_522503 to 522504, EPI_ISL_522506 to 522507, EPI_ISL_522509, EPI_ISL_522511 to 522514, EPI_ISL_522517, EPI_ISL_522519 to 522544, EPI_ISL_526700 to 526729, EPI_ISL_526732 to 526733			
see above	Division of Viral Diseases, Center for Laboratory Control of Infectious Diseases, Korea Centers for Diseases Control and Prevention	Division of Viral Diseases, Center for Laboratory Control of Infectious Diseases, Korea Centers for Diseases Control and Prevention	Daesang Lee; Dong Hyun Song; Heui Man Kim; Hye-Jun Jo; Jeong-Min Kim; Jun-Sub Kim; Junhyeong Jang; Mi-Seon Kim; Myung Guk Han; Namjoo Lee; Sang Hee Woo; Sehee Park; Seong Tae Jeong; Yoon-Seok Chung
EPI_ISL_722209	Dom Zdravlja Sarajevo	Alea Genetic Center	Konjhodzic R.; Pecar D.; Salihefendic L.
EPI_ISL_513357, EPI_ISL_513389	Douglas Hanly Moir	NSW Health Pathology - Institute of Clinical Pathology and Medical Research; Westmead Hospital; University of Sydney	CIDM-PH et al.
EPI_ISL_451601, EPI_ISL_455051 to 455053, EPI_ISL_455065	Douglas Hanly Moir Pathology	NSW Health Pathology - Institute of Clinical Pathology and Medical Research; Westmead Hospital; University of Sydney	CIDM-PH et al.
EPI_ISL_498773, EPI_ISL_526153, EPI_ISL_526213 to 526214, EPI_ISL_545027, EPI_ISL_593650 to 593651, EPI_ISL_740881 to 740882	Douglass Hanly Moir Pathology	NSW Health Pathology - Institute of Clinical Pathology and Medical Research; Westmead Hospital; University of Sydney	CIDM-PH et al.
EPI_ISL_700513, EPI_ISL_700517, EPI_ISL_700525, EPI_ISL_700547, EPI_ISL_700552, EPI_ISL_700567 to 700568, EPI_ISL_700570, EPI_ISL_700582, EPI_ISL_700591 to 700592			
see above	Dr Abdurahman CDC wc DAC	NHLS/UCT	Arash Iranzadeh; Bruna Galvao; Carolyn Williamson; Deelan Doolabh; Diana Hardie; Innocent Mudau; Kruger Marais; Lynn Tyers; Marvin Hsiao; Stephen Korsman
EPI_ISL_1007612 to 1007613	Dr. Bard	Laboratory of genomics and metagenomics, Institute of Microbiology, University Hospital Centre and University of Lausanne, Switzerland	Claire Bertelli; Damien Jacot; Gilbert Greub; Sébastien Aeby; Trestan Pillonel
EPI_ISL_1007638	Dr. Borel	Laboratory of genomics and metagenomics, Institute of Microbiology, University Hospital Centre and University of Lausanne, Switzerland	Claire Bertelli; Damien Jacot; Gilbert Greub; Sébastien Aeby; Trestan Pillonel
EPI_ISL_766589 to 766590	Dr. Boubaker Karim laboratory	Institute of Medical Virology, University of Zurich	Alexandra Trkola; Annette Audigé; Cyril Shah; Jon Huder; Jürg Böni; Kevin Steiner; Maria Grünberg; Maryam Zaheri; Michael Huber; Riccarda Capaul; Stefan Schmutz; Verena Kufner
EPI_ISL_418811, EPI_ISL_429806, EPI_ISL_429811, EPI_ISL_429816, EPI_ISL_479625 to 479651, EPI_ISL_481742 to 481758, EPI_ISL_482471 to 482473			
see above	Dr. Georges-L.-Dumont University Hospital Centre	National Microbiology Laboratory	Anna Majer; Elsie Grudeski; Gary Van Domselaar; Grace Seo; Guillaume Desnoyers; Jennifer Tanner; Kristyn Burak; Matthew Gilmour; Morag Graham; Natalie Knox; Nathalie Bastien; Philip Mabon; Rhiannon Huzarewich; Richard Garceau; Russell Mandes; Shari Tyson; Timothy Booth; Yan Li
EPI_ISL_583727 to 583733, EPI_ISL_583735 to 583764, EPI_ISL_583766 to 583845, EPI_ISL_583847 to 583852	Dr. Gernot Walder GmbH	Bergthaler laboratory, CeMM Research Center for Molecular Medicine of the Austrian Academy of Sciences	Adi Steinrigl; Alexander Lercher; Alexandra Popa; Andreas Bergthaler; Benedikt Agerer; Christian Paar; Christoph Bock; Daniela Schmid; Dorothee von Laer; Elisabeth Puchhammer-Stoeckl; Franz Allerberger; Gernot Walder; Gregor Hörmann; Guenter Weiss; Gunther Vogl; Henrique Colaco; Jakob-Wendelin Genger; Jan Laine; Judith Aberle; Kinga Rigler-Hohenwarter; Lukas Endler; Manfred Nairz; Mark Smyth; Martin Senekowitsch; Michael Schuster; Peter Hufnagl; Peter Obrist; Rainer Gattringer; Sabine Sussitz-Rack; Stephan Aberle; Thomas Penz; Wegene Borena
EPI_ISL_528752	Dr. H. A. Rotinsulu Lung Hospital	School of Pharmacy & School of Life Sciences and Technology - Institut Teknologi Bandung; Molecular Genetics Laboratory-Faculty of Medicine-Universitas Padjadjaran; Laboratorium Kesehatan Provinsi Jawa Barat	Adelina Khristiani Rahayu; Agung Eru Wibowo; Azzania Fibriani; Catur Riani; Cut Nur Cinthia Alamanda; Ema Rahmawati; Gusti Ayu Prani Pradani; Hammam Riza; Hesti Lina Wiraswati; Husna Nugrahapraja; Irvan Faizal; Lia Faridah; Marselina Irasonia Tan; Rifky Waluyajati Rachman; Ryan Bayusantika Ristandi; Savira Ekawardhani; Sony Solistia Wirawan; Tarwadi; Yunia Sribudiani
EPI_ISL_469043 to 469047, EPI_ISL_475056 to 475057, EPI_ISL_495067 to 495069	Dr. N. D. Desai Medical College & Hospital	Gujarat Biotechnology Research Centre	A M Kadri; Afzal Ansari; Akanksha Verma; Ankit Hinsu; Apurvasinh Puvar; Bhavya Jindal; Chaitanya Joshi; Dinesh Kumar; Harsh Bakshi; J G Buch; Janvi Raval; Jigar Gusani; Komal Patel; Labdhi Pandya; Madhvi Joshi; Maharshi Pandya; Monika Gandhi; Neha Rajpara; Nidhi Patel; Nikha Trivedi; Nitin Savaliya; Pinal Trivedi; Pooja P Doshi; Pritesh Sabara; Priti Pandita; Priyanka P Vatsa; R D Dixit; Raghawendra Kumar; Snehal Bagatharia; Supreet Prabhu; Tejas Shah; Zarna Patel; Zuber Saiyed
EPI_ISL_512075 to 512077, EPI_ISL_514435 to	Dr. RSS Hospital, Modasa	Gujarat Biotechnology Research Centre	A M Kadri; Afzal Ansari; Apurvasinh Puvar; Chaitanya Joshi; Dinesh Kumar; Harsh Bakshi; Harsh Chaudhari; Janvi Raval; Komal Patel; Labdhi Pandya;

514437, EPI_ISL_524732			Madhvi Joshi; Maharshi Pandya; Monika Gandhi; Neena Doshi; Nidhi Patel; Nikha Trivedi; Nitin Savaliya; Pinal Trivedi; R D Dixit; Raghawendra Kumar; Varsha Godbole; Zarna Patel; Zuber Saiyed
EPI_ISL_495012	Dr. Tony Mazzulli Microbiologist-in-Chief	Dr. Jeff Wrana, Senior Investigator	Bryn Hazlett; Christine Bruce; Dan Trcka; Javier Hernandez; Jeff Wrana; Jess Shen; Jessica Bourke; Kin Chan; Laurence Pelletier; Marie-Ming Aynaud; Seda Barutcu; Sue Poutanen; Tony Mazzulli
EPI_ISL_494970, EPI_ISL_495013	Dr. Tony Mazzulli Microbiologist-in-Chief	Dr. Jeff Wrana, Senior Investigator	Bryn Hazlett; Christine Bruce; Dan Trcka; Javier Hernandez; Jeff Wrana; Jess Shen; Jessica Bourke; Kin Chan; Laurence Pelletier; Marie-Ming Aynaud; Seda Barutcu; Sue Poutanen; Tony Mazzulli
EPI_ISL_417211 to 417212	Dunedin Hospital	University of Otago	B. Lawley; J. Grant; J. Ussher; M.E. Quiñones-Mateu; R. Harfoot
EPI_ISL_414423 to 414426, EPI_ISL_414428 to 414430, EPI_ISL_414432 to 414446, EPI_ISL_414448 to 414449, EPI_ISL_414451 to 414452, EPI_ISL_414454, EPI_ISL_414456 to 414458, EPI_ISL_414460 to 414464, EPI_ISL_414467 to 414471, EPI_ISL_414529 to 414532, EPI_ISL_414534 to 414537, EPI_ISL_414539, EPI_ISL_414541 to 414545, EPI_ISL_414548 to 414549, EPI_ISL_414551 to 414552, EPI_ISL_414554 to 414564, EPI_ISL_414566, EPI_ISL_415460 to 415476, EPI_ISL_415478 to 415489, EPI_ISL_415491 to 415515, EPI_ISL_415517 to 415527, EPI_ISL_415529 to 415535, EPI_ISL_422566, EPI_ISL_422568 to 422569, EPI_ISL_422571 to 422572, EPI_ISL_422576 to 422583, EPI_ISL_422585 to 422588, EPI_ISL_422591 to 422612, EPI_ISL_422615 to 422616, EPI_ISL_422620 to 422623, EPI_ISL_422625 to 422626, EPI_ISL_422628 to 422635, EPI_ISL_422638 to 422653, EPI_ISL_422655 to 422657, EPI_ISL_422659 to 422662, EPI_ISL_422664 to 422682, EPI_ISL_422684 to 422709, EPI_ISL_422711 to 422712, EPI_ISL_422714 to 422722, EPI_ISL_422724 to 422730, EPI_ISL_422732 to 422738, EPI_ISL_422741 to 422742, EPI_ISL_422744 to 422756, EPI_ISL_422758 to 422762, EPI_ISL_422764 to 422772, EPI_ISL_422774, EPI_ISL_422776 to 422778, EPI_ISL_422784 to 422785, EPI_ISL_422787 to 422788, EPI_ISL_422790 to 422799, EPI_ISL_422801 to 422805, EPI_ISL_422807 to 422813, EPI_ISL_422815 to 422823, EPI_ISL_422825, EPI_ISL_422827, EPI_ISL_422833 to 422835, EPI_ISL_422837 to 422840, EPI_ISL_422842 to 422843, EPI_ISL_422845, EPI_ISL_422847 to 422895, EPI_ISL_422897 to 422898, EPI_ISL_422901 to 422905, EPI_ISL_422907 to 422920, EPI_ISL_422923 to 422926, EPI_ISL_422929 to 422930, EPI_ISL_422932, EPI_ISL_422934 to 422935, EPI_ISL_422938 to 422940, EPI_ISL_422942 to 422945, EPI_ISL_422947 to 422952, EPI_ISL_422954 to 422957, EPI_ISL_422960, EPI_ISL_455112 to 455123, EPI_ISL_455125 to 455126, EPI_ISL_455129, EPI_ISL_455131 to 455135, EPI_ISL_455141, EPI_ISL_455143 to 455145, EPI_ISL_455147 to 455149, EPI_ISL_455151 to 455152, EPI_ISL_455155 to 455157, EPI_ISL_455159 to 455165, EPI_ISL_455167 to 455181, EPI_ISL_455184 to 455193, EPI_ISL_455195 to 455196, EPI_ISL_455198 to 455203, EPI_ISL_455205 to 455206, EPI_ISL_455208 to 455217, EPI_ISL_455219, EPI_ISL_455221 to 455228, EPI_ISL_455231 to 455233, EPI_ISL_455235 to 455238, EPI_ISL_455241 to 455242, EPI_ISL_455244 to 455246, EPI_ISL_455249, EPI_ISL_455251 to 455263, EPI_ISL_455266, EPI_ISL_455269 to 455270, EPI_ISL_455274 to 455281, EPI_ISL_455283 to 455287, EPI_ISL_455289 to 455293, EPI_ISL_455296 to 455300, EPI_ISL_455302 to 455307, EPI_ISL_460636 to 460644, EPI_ISL_460646 to 460664, EPI_ISL_460666 to 460677, EPI_ISL_460682 to 460688, EPI_ISL_460690 to 460713, EPI_ISL_460716 to 460718, EPI_ISL_460720, EPI_ISL_460722, EPI_ISL_460724 to 460737, EPI_ISL_460739 to 460743, EPI_ISL_460746 to 460750, EPI_ISL_460752 to 460758, EPI_ISL_460760, EPI_ISL_460762 to 460764, EPI_ISL_460766 to 460768, EPI_ISL_460770 to 460782, EPI_ISL_460785 to 460795, EPI_ISL_460797 to 460811, EPI_ISL_460813 to 460845, EPI_ISL_460847, EPI_ISL_460849 to 460856, EPI_ISL_460859 to 460891, EPI_ISL_460903 to 460915, EPI_ISL_460917 to 460924, EPI_ISL_460926 to 460953, EPI_ISL_460955 to 460956, EPI_ISL_460959 to 460987, EPI_ISL_460989 to 460990, EPI_ISL_460992 to 461004, EPI_ISL_461006 to 461013, EPI_ISL_461015 to 461022, EPI_ISL_461025 to 461027, EPI_ISL_461029 to 461048, EPI_ISL_461051 to 461060, EPI_ISL_461063 to 461066, EPI_ISL_461068 to 461071, EPI_ISL_461074 to 461079, EPI_ISL_461082 to 461095, EPI_ISL_461097 to 461098, EPI_ISL_461100 to 461106, EPI_ISL_461108 to 461109, EPI_ISL_461111 to 461116, EPI_ISL_461118, EPI_ISL_461120 to 461123, EPI_ISL_461125 to 461137, EPI_ISL_461139 to 461141, EPI_ISL_461143 to 461153, EPI_ISL_461155 to 461165, EPI_ISL_461167 to 461169, EPI_ISL_461171 to 461186, EPI_ISL_461188 to 461189, EPI_ISL_461191 to 461195, EPI_ISL_461197 to 461202, EPI_ISL_461204, EPI_ISL_461206 to 461219, EPI_ISL_461221 to 461224, EPI_ISL_461226 to 461230, EPI_ISL_461232 to 461247, EPI_ISL_461250 to 461251, EPI_ISL_461254 to 461255, EPI_ISL_461257 to 461261, EPI_ISL_461264 to 461266, EPI_ISL_461268, EPI_ISL_461270 to 461277, EPI_ISL_461280 to 461289, EPI_ISL_461291 to 461300, EPI_ISL_461302 to 461303, EPI_ISL_461305, EPI_ISL_461307 to 461317, EPI_ISL_461319 to 461326, EPI_ISL_461328 to 461329, EPI_ISL_461331 to 461335, EPI_ISL_461337 to 461353, EPI_ISL_461355 to 461361, EPI_ISL_461363 to 461368, EPI_ISL_461370, EPI_ISL_461373 to 461398, EPI_ISL_522987 to 522990, EPI_ISL_522992, EPI_ISL_522994 to 523010, EPI_ISL_523012, EPI_ISL_523014 to 523020, EPI_ISL_523023 to 523028, EPI_ISL_523034 to 523039, EPI_ISL_523041 to 523046, EPI_ISL_523048 to 523067, EPI_ISL_523069 to 523071, EPI_ISL_523074 to 523077, EPI_ISL_523079 to 523087, EPI_ISL_523091 to 523101, EPI_ISL_523103 to 523106, EPI_ISL_523108 to 523114, EPI_ISL_523116 to 523119, EPI_ISL_523121 to 523130, EPI_ISL_523132 to 523134, EPI_ISL_523138 to 523154, EPI_ISL_523156 to 523161, EPI_ISL_523167 to 523168, EPI_ISL_523170 to 523189, EPI_ISL_523191 to 523193, EPI_ISL_523195 to 523197, EPI_ISL_523199 to 523200, EPI_ISL_523202 to 523205, EPI_ISL_523207 to 523211, EPI_ISL_523213 to 523218, EPI_ISL_523220 to 523223, EPI_ISL_523225 to 523231, EPI_ISL_523233, EPI_ISL_523235 to 523236, EPI_ISL_523238 to 523241, EPI_ISL_523245 to 523252, EPI_ISL_523254, EPI_ISL_523257 to 523265, EPI_ISL_523267 to 523270, EPI_ISL_523272 to 523286, EPI_ISL_523288 to 523299, EPI_ISL_523301, EPI_ISL_523303 to 523337, EPI_ISL_523340 to 523343, EPI_ISL_523345, EPI_ISL_523347 to 523359, EPI_ISL_523361, EPI_ISL_523364, EPI_ISL_523366 to 523378, EPI_ISL_523380 to 523386, EPI_ISL_523388 to 523389, EPI_ISL_523394 to 523397, EPI_ISL_523401 to 523403, EPI_ISL_523405, EPI_ISL_523407 to 523410, EPI_ISL_523412 to 523417, EPI_ISL_523420 to 523437, EPI_ISL_523439, EPI_ISL_523441 to 523476, EPI_ISL_523479, EPI_ISL_523483 to 523487, EPI_ISL_523490 to 523491, EPI_ISL_523493 to 523495, EPI_ISL_523497 to 523499, EPI_ISL_523501 to 523509, EPI_ISL_523513 to 523518, EPI_ISL_523520 to 523521, EPI_ISL_523523 to 523534, EPI_ISL_523536 to 523537, EPI_ISL_523539 to 523547, EPI_ISL_523549 to 523560, EPI_ISL_523563 to 523566, EPI_ISL_523568 to 523573, EPI_ISL_523575 to 523580, EPI_ISL_523583 to 523588, EPI_ISL_523591 to 523593, EPI_ISL_523596 to 523614, EPI_ISL_523616, EPI_ISL_523618 to 523623, EPI_ISL_523625 to 523641, EPI_ISL_523643 to 523652, EPI_ISL_523654, EPI_ISL_523657 to 523687, EPI_ISL_523689 to 523690, EPI_ISL_523692 to 523742, EPI_ISL_523744 to 523780, EPI_ISL_523782 to 523800, EPI_ISL_523802 to 523809, EPI_ISL_574779 to 574789, EPI_ISL_577743 to 577744, EPI_ISL_577746 to 577747, EPI_ISL_577749 to 577750, EPI_ISL_577752 to 577756, EPI_ISL_577758, EPI_ISL_577764, EPI_ISL_577767 to 577768, EPI_ISL_577770 to 577776, EPI_ISL_577781, EPI_ISL_577783 to 577787, EPI_ISL_577791, EPI_ISL_577797 to 577803, EPI_ISL_577805, EPI_ISL_577808 to 577810, EPI_ISL_577813, EPI_ISL_577825, EPI_ISL_577829 to 577830, EPI_ISL_577832 to 577835, EPI_ISL_577837 to 577848, EPI_ISL_577850 to 577859, EPI_ISL_577861, EPI_ISL_577863 to 577866, EPI_ISL_577868 to 577871, EPI_ISL_577873, EPI_ISL_577879 to 577880, EPI_ISL_577882, EPI_ISL_577884, EPI_ISL_577886 to 577901, EPI_ISL_577903 to 577906, EPI_ISL_577909 to 577914, EPI_ISL_577916, EPI_ISL_577918 to 577927, EPI_ISL_577929 to 577931, EPI_ISL_577933 to 577935, EPI_ISL_577937 to 577944, EPI_ISL_577946 to 577961, EPI_ISL_577963 to 577964, EPI_ISL_577966 to 577967, EPI_ISL_577969 to 577973, EPI_ISL_577975 to 577979, EPI_ISL_577981 to 577983, EPI_ISL_577985, EPI_ISL_577989, EPI_ISL_577992, EPI_ISL_577994, EPI_ISL_577996 to 578002, EPI_ISL_578004 to 578009, EPI_ISL_578011, EPI_ISL_578013 to 578030, EPI_ISL_578033 to 578048, EPI_ISL_578050 to 578051, EPI_ISL_578053 to 578054, EPI_ISL_578057, EPI_ISL_578059 to 578065, EPI_ISL_578067 to 578074, EPI_ISL_578077 to 578079, EPI_ISL_632389 to 632391, EPI_ISL_632428 to 632445, EPI_ISL_632448, EPI_ISL_632450 to 632492, EPI_ISL_632494 to 632795, EPI_ISL_722370 to 722371, EPI_ISL_722413 to 722434, EPI_ISL_722437 to 722464, EPI_ISL_722466 to 722850, EPI_ISL_763099 to 763103, EPI_ISL_763105, EPI_ISL_763107 to 763116, EPI_ISL_763118 to 763121, EPI_ISL_763124 to 763137, EPI_ISL_763140 to 763145, EPI_ISL_763147 to 763153, EPI_ISL_763155 to 763180, EPI_ISL_763182 to 763187, EPI_ISL_763189 to 763194, EPI_ISL_763198 to 763200, EPI_ISL_763202 to 763206, EPI_ISL_763211, EPI_ISL_763214 to 763221, EPI_ISL_763223 to 763227, EPI_ISL_763229 to 763231, EPI_ISL_763233, EPI_ISL_763235 to 763237, EPI_ISL_763239, EPI_ISL_763241, EPI_ISL_763243 to 763248, EPI_ISL_763250, EPI_ISL_763252, EPI_ISL_763254 to 763257, EPI_ISL_763262 to 763264, EPI_ISL_763266 to 763270, EPI_ISL_763272, EPI_ISL_763274 to 763279, EPI_ISL_763281 to 763282, EPI_ISL_763284 to 763288, EPI_ISL_763291 to 763293, EPI_ISL_763296 to 763301, EPI_ISL_763303 to 763311, EPI_ISL_763313 to 763315, EPI_ISL_763348 to 763349, EPI_ISL_763355 to 763359, EPI_ISL_801421 to 801423, EPI_ISL_802519 to 802542, EPI_ISL_904194 to 904220, EPI_ISL_904255 to 904277, EPI_ISL_904316 to 904318, EPI_ISL_904375 to 904380			
see above	Dutch COVID-19 response team	Erasmus Medical Center	Anne van der Linden; Annemiek van der Eijk; Aura Timen; Bas Oude Munnink; Claudia Schapendonk; Corien Swaan; Corine GeurtsvanKessel; David Nieuwenhuijse; Emmanuelle Munger; Irina Chestakova; Jeroen van Kampen; Jolanda Voermans; Madelief Mollers; Manon Haverkate; Marion Koopmans; Marjan Boter; Mark Pronk; Mart Stein; OH consortium; Pascal Lexmond; Reina Sikkema; Richard Molenkamp; Sandra Kengne Kamga Mobou; Stefan van Nieuwkoop; Theo Bestebroer; on behalf of the Dutch national COVID-19 response team.
EPI_ISL_965583, EPI_ISL_965595 to 965596	Dutch COVID-19 response team	Medical Microbiology, Maastricht University Medical Centre	Brian van der Veer*; Carmen Reumkens; Christian Hoebe; Erik Beuken; Jozef Dingemans*; Lieke van Alphen; Paul Savelkoul
EPI_ISL_454750 to 454794, EPI_ISL_547445 to 547568, EPI_ISL_636491 to 636603, EPI_ISL_723155 to 723191, EPI_ISL_723193 to 723195, EPI_ISL_723202 to 723467, EPI_ISL_728564 to 728733, EPI_ISL_728735 to 728763, EPI_ISL_790571 to 790574, EPI_ISL_790580 to 790591, EPI_ISL_823976 to 823989, EPI_ISL_823992, EPI_ISL_826523 to 826536, EPI_ISL_904659 to 904672, EPI_ISL_904674 to 904720, EPI_ISL_904753 to 904756, EPI_ISL_904962, EPI_ISL_904968, EPI_ISL_904976 to 904977, EPI_ISL_905064, EPI_ISL_905068, EPI_ISL_905071, EPI_ISL_905101, EPI_ISL_905104, EPI_ISL_905114, EPI_ISL_905122, EPI_ISL_905128, EPI_ISL_905134, EPI_ISL_905138, EPI_ISL_905166, EPI_ISL_905169, EPI_ISL_905175 to 905187, EPI_ISL_905190 to 905201, EPI_ISL_905234 to 905238, EPI_ISL_905376, EPI_ISL_905388, EPI_ISL_905397, EPI_ISL_905450 to 905451, EPI_ISL_905456, EPI_ISL_905488, EPI_ISL_905501, EPI_ISL_905510, EPI_ISL_905513, EPI_ISL_905537, EPI_ISL_905551 to 905552, EPI_ISL_905586, EPI_ISL_905590, EPI_ISL_905608, EPI_ISL_905614, EPI_ISL_905660 to 905661, EPI_ISL_905718, EPI_ISL_905721, EPI_ISL_905730, EPI_ISL_942978 to 942995, EPI_ISL_942998, EPI_ISL_943001 to 943043, EPI_ISL_943062 to 943063, EPI_ISL_943065 to 943067, EPI_ISL_943269, EPI_ISL_943272 to 943274, EPI_ISL_943277 to 943278, EPI_ISL_943280 to 943284, EPI_ISL_943286 to 943287, EPI_ISL_943291, EPI_ISL_943293, EPI_ISL_943298 to 943303, EPI_ISL_943308, EPI_ISL_943312 to 943313, EPI_ISL_943317, EPI_ISL_943319, EPI_ISL_943321 to 943322, EPI_ISL_943324 to 943325, EPI_ISL_943328, EPI_ISL_943335, EPI_ISL_943355, EPI_ISL_943357, EPI_ISL_943360 to 943361, EPI_ISL_943364 to 943365, EPI_ISL_943367, EPI_ISL_943369, EPI_ISL_943376, EPI_ISL_943381, EPI_ISL_943391, EPI_ISL_943404 to 943405, EPI_ISL_943409, EPI_ISL_943419, EPI_ISL_943427 to 943430, EPI_ISL_943432, EPI_ISL_943437, EPI_ISL_943440 to 943442, EPI_ISL_943444, EPI_ISL_943449, EPI_ISL_943453, EPI_ISL_943458, EPI_ISL_943461 to 943465, EPI_ISL_943468, EPI_ISL_943477 to 943479, EPI_ISL_943481 to 943484, EPI_ISL_943490 to 943491, EPI_ISL_943493, EPI_ISL_943496 to 943497, EPI_ISL_943504, EPI_ISL_943508, EPI_ISL_943518 to 943519, EPI_ISL_943532 to 943533, EPI_ISL_943539, EPI_ISL_1013619 to 1013681, EPI_ISL_1013684 to 1013689, EPI_ISL_1013695 to 1013770, EPI_ISL_1013787 to 1013793, EPI_ISL_1013795 to 1013801, EPI_ISL_1014241 to 1014283, EPI_ISL_1014285 to 1014328, EPI_ISL_1014344 to 1014348, EPI_ISL_1020302, EPI_ISL_1034924 to 1034925, EPI_ISL_1034927 to 1035061, EPI_ISL_1035068 to 1035082, EPI_ISL_1035093 to 1035097, EPI_ISL_1035100 to 1035197, EPI_ISL_1035224 to 1035234			
see above	Dutch COVID-19 response team	National Institute for Public Health and the Environment (RIVM)	Adam Meijer; AnneMarie van den Brandt; Bas van der Veer; Chantal Reusken; Dennis Schmitz; Dirk Eggink; Florian Zwagemaker; Harry Vennema; Jeroen Cremer; Pieter Overduin; Sharon van den Brink; on behalf of the national COVID-19 response team
EPI_ISL_913275, EPI_ISL_913454, EPI_ISL_913459, EPI_ISL_1008479, EPI_ISL_1008487	Dynamic Code AB	The Public Health Agency of Sweden	Anna Risberg; Anna-Malin Linde; Carlo Berg; Karin Tegmark-Wisell; Maria Lind Karlberg; Mattias Haukland; Mia Brytting; Noura Walai; Oskar Karlsson Lindsjo; Petra Edquist; Petra Holmstrom; Reza Advani; Sofia Stamouli
EPI_ISL_640024, EPI_ISL_700520	Dysselsdorp Clinic wc DDC	NHLS/UCT	Arash Iranzadeh; Bruna Galvao; Carolyn Williamson; Deelan Doolabh; Diana Hardie; Houriiyah Tegally; Innocent Mudau; Kruger Marais; Lynn Tyers; Marvin Hsiao; Stephen Korsman
EPI_ISL_960096	Dysselsdorp Clinic wc DDC	National Health Laboratory Service/UCT	Arash Iranzadeh; Bruna Galvao; Carolyn Williamson; Deelan Doolabh; Diana Hardie; Innocent Mudau; Kruger Marais; Lynn Tyers; Marvin Hsiao; Stephen Korsman
EPI_ISL_419691	E. Gulbja Laboratorija	Charite Universitatsmedizin Berlin, Institute of Virology	Barbara Mühlemann; Christian Drosten; Dmitrijs Perminovs; Dr. Didzis Gavars; Jorn Beheim-Schwarzbach; Julia Schneider; Mikus Gavars; Talitha Veith; Terry Jones; Victor M Corman
EPI_ISL_421653 to 421656, EPI_ISL_426285 to 426289, EPI_ISL_450518 to 450519, EPI_ISL_492993 to 493000, EPI_ISL_501275, EPI_ISL_501284 to 501285, EPI_ISL_501823, EPI_ISL_501829, EPI_ISL_501833, EPI_ISL_501839, EPI_ISL_501849, EPI_ISL_501894, EPI_ISL_501929, EPI_ISL_501936, EPI_ISL_512313 to 512314, EPI_ISL_512645 to 512646, EPI_ISL_515187 to 515195, EPI_ISL_534199, EPI_ISL_534203, EPI_ISL_534212 to 534216, EPI_ISL_534218 to 534219, EPI_ISL_534221, EPI_ISL_639631, EPI_ISL_639635, EPI_ISL_639637 to 639638, EPI_ISL_639641, EPI_ISL_639643 to 639647, EPI_ISL_639649, EPI_ISL_639652 to 639653, EPI_ISL_639658 to 639662, EPI_ISL_639664 to 639671, EPI_ISL_639673 to 639675, EPI_ISL_639681 to 639682, EPI_ISL_639684 to 639694, EPI_ISL_770033 to 770037, EPI_ISL_770040 to 770041, EPI_ISL_770043 to 770044, EPI_ISL_770046, EPI_ISL_770050 to 770051, EPI_ISL_770053, EPI_ISL_770055, EPI_ISL_770057, EPI_ISL_770062			
see above	E. Gulbja Laboratorija	Latvian Biomedical Research and Study Centre	Dmitrijs Perminovs; Ivars Silamielis; Jnis Kloviš; Jnis Pjalkovskis; Kaspars Megnis; Mikus Gavars; Monta Ustinova; Uga Dumpis; Vita Rovte; ikita Zrelovs
EPI_ISL_486438, EPI_ISL_961804, EPI_ISL_961854, EPI_ISL_961881	E. Gulbja laboratorija	Latvian Biomedical Research and Study Centre	Davids Fridmanis; Dmitrijs Perminovs; Dmitrijs Perminovs; Ivars Silamielis; Ivars Silamikelis; Janis Pjalkovskis; Janis Klovins; Jurijs Perevoscikovs; Jnis Kloviš; Kaspars Megnis; Kaspars Megnis; Laila Silamikele; Lauma Freimane; Laura Ansone; Liga Birzniece; Mikus Gavars; Mikus Gavars; Monta Ustinova; Monta Ustinova; Nikita Zrelovs; Uga Dumpis; Uga Dumpis; Vita Rovte; Vita Rovite; ikita Zrelovs
EPI_ISL_1007585	EHC MORGES	Laboratory of genomics and metagenomics, Institute of Microbiology, University Hospital Centre and University of Lausanne, Switzerland	Claire Bertelli; Damien Jacot; Gilbert Greub; Sébastien Aeby; Trestan Pillonel
EPI_ISL_1007598, EPI_ISL_1007602, EPI_ISL_1007626, EPI_ISL_1007628, EPI_ISL_1007631	EHNV	Laboratory of genomics and metagenomics, Institute of Microbiology, University Hospital Centre and University of Lausanne, Switzerland	Claire Bertelli; Damien Jacot; Gilbert Greub; Sébastien Aeby; Trestan Pillonel

EPI_ISL_765478	EHPAD	National Reference Center for Viruses of Respiratory Infections, Institut Pasteur, Paris	Angela Brisebarre; Camille Capel; Etienne Simon-Lorière; Marion Barbet; Maud Vanpeene; Méline Bizard; Sylvie Behillil; Sylvie van der Werf; Vincent Enouf
EPI_ISL_418226	EHPAD - Résidences les Cèdres	National Reference Center for Viruses of Respiratory Infections, Institut Pasteur, Paris	Angela Brisebarre; Etienne Simon-Lorière; Flora Donati; Marion Barbet; Maud Vanpeene; Mélanie Albert; Méline Bizard; Sylvie Behillil; Sylvie van der Werf; Vincent Enouf
EPI_ISL_745198	ESI Hospital	CSIR-Centre for Cellular and Molecular Biology	Archana Bharadwaj Siva; B Himasri; Blessy B John; Divya Tej Sowpati; Dr.P.Shashikala Reddy; Dr.S.Pavani; Dr.Satyaprasad; Dr.V.Sudha Rani; Karthik Bharadwaj Tallapaka; Lamuk Zaveri; M Soujanya Reddy; Namami Gaur; Nikhil Hajirnis; Onkar Kulkarni; Payel Mukherjee; Pratheusa Maccha; Priya Singh; Purushotham Vodnala; Rakesh K Mishra; Sakshi Shambhavi; Shagufta Khan; Sofia Banu; Tulasi Nagabandi; Viswagithe S L
EPI_ISL_593481, EPI_ISL_593487, EPI_ISL_593489 to 593499, EPI_ISL_593501 to 593504, EPI_ISL_593506 to 593509, EPI_ISL_593511 to 593512, EPI_ISL_593514 to 593515, EPI_ISL_593517 to 593518, EPI_ISL_593523 to 593526, EPI_ISL_593528 to 593529, EPI_ISL_593532, EPI_ISL_593536 to 593541, EPI_ISL_593543, EPI_ISL_593545 to 593551			
see above	Eastern Ontario Regional Laboratory Association	McMaster University	Ahmed Draia; Andrew G. McArthur; Emily Panousis; Hooman Derakhshani; Jalees Nasir; Leanne Mortimer; Robert Slinger
EPI_ISL_468044 to 468062, EPI_ISL_475722 to 475724, EPI_ISL_477161, EPI_ISL_478672, EPI_ISL_479686 to 479701, EPI_ISL_479703 to 479708, EPI_ISL_479710 to 479731, EPI_ISL_479733 to 479735, EPI_ISL_524426 to 524427, EPI_ISL_529141 to 529145			
see above	Egyptian National Cancer Institute (ENCI)	Egyptian National Cancer Institute (ENCI)	A.A.; A.N.; Abdel Rahman N; Abdel Rahman N.; Abdelhamid, W.; Abouelhoda; Abouelhoda, M.; Ahmed; Ali; Ali, M.; Amer; Bahnassy; Elkhateeb; Elsissy; Ezzelarab; Gad, A.; H.K.; Hafez; Hamdy; Hassan; Hassan, W.; K.E.; Khattab; M.A.; M.H.; M.M.; M.S.; Mahmoud; Mohamed; O.S.; Raouf, A.; S.M.; Samir, M.; Soliman; W.A.; Zekri
EPI_ISL_469275	Egyptian National Cancer Institute (ENCI)	Human Genome Center	A.A.; Abdel Rahman N; Abdelhamid, W.; Abouelhoda; Ahmed; Ali, M.; Amer; Bahnassy; Elkhateeb; Elsissy; Ezzelarab; Gad, A.; H.K.; Hafez; Hamdy; Hassan, W.; K.E.; M.H.; M.M.; M.S.; Mohamed; O.S.; Raouf, A.; S.M.; Samir, M.; Soliman; Zekri
EPI_ISL_434652, EPI_ISL_710610 to 710611	Ektorps Vardcentral	The Public Health Agency of Sweden	Anna Risberg; Anna-Malin Linde; Department of Microbiology; Eva Espmark; Karin Tegmark-Wisell; Maria Lind Karlberg; Mia Brytting; Olov Svartstrom; Oskar Karlsson Lindsjo; The Public Health Agency of Sweden; Theresa Enkirch
EPI_ISL_486856	Emergency County Hospital	Stefan cel Mare, University Metagenomics lab	Lobiuc Andrei et al.
EPI_ISL_486854	Emergency County Hospital Suceava	Stefan cel Mare, University Metagenomics lab	Lobiuc Andrei et al.
EPI_ISL_708822, EPI_ISL_708824 to 708826	Emergency Operation Center, (EOC)	National Institute of Health, Department of Medical Sciences, Ministry of Public Health, Thailand	Malinee Chittaganpitch; Pakorn Piromtong; Pilailuk Okada; Siripaporn Phuygun; Sittiporn Parnmen; Sunthareeya Waicharoen; Thanutsapa Thanadachakul; Warawan Wongboot
EPI_ISL_454690, EPI_ISL_455355 to 455356, EPI_ISL_455358 to 455361, EPI_ISL_754751 to 754778	Emory Molecular Diagnostics Laboratory, Emory Healthcare	Piantadosi Lab, Emory Department of Pathology	Ahmed Babiker; Anne Piantadosi
EPI_ISL_632976 to 632977, EPI_ISL_632979	Empire City Laboratories	New York City Public Health Laboratory	Jade Wang; et al.
EPI_ISL_424350 to 424351, EPI_ISL_468718	Environmental and Global Health	Environmental and Global Health	Alam, M.; C.J.; Elbadry; Gibson; J.A.; J.C.; J.G.; J.G. Jr. and Lednicky; L.H.; M.A.; Merck; Morris; Stephenson; Subramaniam, K.; T.B.; Waltzek
EPI_ISL_641318 to 641319, EPI_ISL_666617 to 666622	Environmental and Global Health, University of Florida	Environmental and Global Health, University of Florida	Alam; C.-Y.; C.J.; C.T.; Elbadry; Fan; J.A.; J.C.; J.G.; L.O.; Lauzardo, M.; Lednicky; Loeb; M.A.; M.M.; Morris; Nannu-Shankar, S.; Silva; Stephenson; Subramaniam, K.; T.B.; Waltzek; Witanachchi; Wu; Z.H.
EPI_ISL_524433 to 524434	Environmental and Global Health, University of Florida - Gainesville	University of Florida	Alam; C.J.; Elbadry; Gibson; J.A.; J.C.; J.G. Jr.; Lednicky; M.A.; M.M.; Morris; Stephenson; Subramaniam, K.; T.B.; Waltzek
EPI_ISL_632287, EPI_ISL_681322, EPI_ISL_873277	Environmental and Global Health, University of Florida	Environmental and Global Health, University of Florida	Alam; C.J.; Elbadry; Gibson; Iovine; J.A.; J.C.; J.G. and Lednicky; Lauzardo, M.; Lessard, K.; Loeb; M.A.; M.M.; Merck, L.; Morris; N.M.; Reeves, M.; Stephenson; Subramaniam, K.; T.B.; Waltzek; Wilkerson, G.
EPI_ISL_413582	ErasmusMC	Erasmus Medical Center	Anne van der Linden; Annemiek van der Eijk; Aura Timen; Bas Oude Munnink; Claudia Schapendonk; Corien Swaan; Corine GeurtsvanKessel; David Nieuwenhuijse; Irina Chestakova; Jeroen van Kampen; Jolanda Voermans; Madelief Mollers; Manon Haverkate; Marion Koopmans; Mark Pronk; Mart Stein; Pascal Lexmond; Reina Sikkema; Richard Molenkamp; Sandra Kengne Kamga Mobou; on behalf of the Dutch national COVID-19 response team.
EPI_ISL_1009545	Erie County Public Health (ECPHL)	University at Buffalo Genomics and Bioinformatics Core	Alyssa Pohlman; Amanda Boccolucci; Brandon Marzullo; Donald Yergeau; Jennifer Surtees; Jonathan Bard; Natalie Lamb; Norma Nowak
EPI_ISL_648166 to 648168	Eskilstuna	The Public Health Agency of Sweden	Anna Risberg; Anna-Malin Linde; Karin Tegmark-Wisell; Maria Lind Karlberg; Mattias Haukland; Mia Brytting; Olov Svartstrom; Oskar Karlsson Lindsjo; Petra Edquist; Reza Advani; Sandra Broddesson
EPI_ISL_514648	Essentia Health-St. Joseph's Medical Center	Minnesota Department of Health, Public Health Laboratory	Jacob Garfin; Matt Plumb; and Xiong Wang
EPI_ISL_514642, EPI_ISL_603004 to 603005, EPI_ISL_603012 to 603014, EPI_ISL_603016 to 603017, EPI_ISL_644933 to 644935, EPI_ISL_644943, EPI_ISL_654696 to 654697, EPI_ISL_654699, EPI_ISL_683690 to 683694, EPI_ISL_683696 to 683713, EPI_ISL_708484			
see above	Essentia Health-St. Mary's Medical Center	Minnesota Department of Health, Public Health Laboratory	Alexandra Lorentz; Jacob Garfin; Matt Plumb; and Xiong Wang
EPI_ISL_636969 to 636971	Etlik Veterinary Control Central Research Institute	Etlik Veterinary Control Central Research Institute	Ahu Pakdemirli; Cevdet Yarali; Dilek Dulger; Erdem Danyer; Ozcan Yildirim; Sabri Hacioglu; Ummu Sena Sari
EPI_ISL_614265 to 614280	Eurofins	National Reference Center for Viruses of Respiratory Infections, Institut Pasteur, Paris	Angela Brisebarre; Camille Capel; Etienne Simon-Lorière; Marion Barbet; Maud Vanpeene; Méline Bizard; Sylvie Behillil; Sylvie van der Werf; Vincent Enouf
EPI_ISL_450873 to 450874	Evandro Chagas Institute	Evandro Chagas Institute	A.M.; Barbagelata; E.C.; E.M.A.; Ferreira; G.M.R; J.A.; Junior; L.C.; L.S.; M.C.; Martins; P.S.; Santos; Silva; Sousa; Sousa Junior; Viana; W.D.C.; da Silva
EPI_ISL_602282 to 602303	Evangelisches Klinikum Bethel, Institut für Laboratoriumsmedizin, Mikrobiologie und Hygiene	Bielefeld University	Alexander Sczyrba; Christiane Scherer; David Brandt; Jörn Kalinowski; Levin-Joe Klages; Marina Simunovic; Markus Haak; Svenja Vinke; Tobias Busche
EPI_ISL_962442	Evergreen Healthcare	Seattle Flu Study	Amanda Adler; Barry R. Lutz; Benjamin Pelle; Brian Hiatt; Caitlin R. Wolf; Chris D. Frazar; Deborah A. Nickerson; Elisabeth Brandstetter; Erica Ryke; Geoff Melly; Helen Y. Chu; Janet A. Englund; Jay Shendure; Jover Lee; Kairsten Fay; Kirsten Lacombe; Lea M. Starita; Mark J. Rieder; Matthew Richardson; Matthew Thompson; Melissa Truong; Michael Boeckh; Michael Famulare; Misja Ilcisin; Peter D. Han; Philip Dykema; Romesh Gautom; Scott Lindquist; Thomas R. Sibley; Trevor Bedford
EPI_ISL_548966 to 548968, EPI_ISL_548970 to 548971	Expo2020 Emergency Center	Agiomix	Cengiz Yakicier; Cherif Ben Hamada; Rashid Mohammed; Tamer Degheidy; Walaa Allam; Walid Dridi
EPI_ISL_424856 to 424857	FL Bur. of Public Health Laboratories-Jacksonville	Pathogen Discovery, Respiratory Viruses Branch, Division of Viral Diseases, Centers for Disease Control and Prevention	Alison S. Laufer Halpin; Anna Uehara; Christopher A. Elkins; Clinton R. Paden; Haibin Wang; Jing Zhang; Krista Queen; Mary S. Keckler; Rachel Marine; Suxiang Tong; Yan Li; Ying Tao
EPI_ISL_420785	FL Bureau of Health Laboratories Tampa	Pathogen Discovery, Respiratory Viruses Branch, Division of Viral Diseases, Centers for Disease Control and Prevention	Alison S. Laufer Halpin; Anne Uehara; Christopher A. Elkins; Clinton R. Paden; Haibin Wang; Jasmine Padilla; Jing Zhang; Justin Lee; Krista Queen; Mary S. Keckler; Rachel Marine; Suxiang Tong; Yan Li; Ying Tao
EPI_ISL_452110 to 452111, EPI_ISL_452132, EPI_ISL_594465	FL Bureau of Public Health Laboratories	Pathogen Discovery, Respiratory Viruses Branch, Division of Viral Diseases, Centers for Disease Control and Prevention	Alison S. Laufer Halpin; Anna Montmayeur; Anna Uehara; Christopher A. Elkins; Clinton Paden; Clinton R. Paden; Haibin Wang; Jing Zhang; Julu Bhatnagar; Krista Queen; Mary S. Keckler; Rachel Marine; Suxiang Tong; Yan Li; Ying Tao; Zachary Weiner
EPI_ISL_424853 to 424854	FL Bureau of Public Health Laboratories-Miami	Pathogen Discovery, Respiratory Viruses Branch, Division of Viral Diseases, Centers for Disease Control and Prevention	Alison S. Laufer Halpin; Anna Uehara; Christopher A. Elkins; Clinton R. Paden; Haibin Wang; Jing Zhang; Krista Queen; Mary S. Keckler; Rachel Marine; Suxiang Tong; Yan Li; Ying Tao
EPI_ISL_419559 to 419560, EPI_ISL_424855, EPI_ISL_447841	FL Bureau of Public Health Laboratories-Tampa	Pathogen Discovery, Respiratory Viruses Branch, Division of Viral Diseases, Centers for Disease Control and Prevention	Alison S. Laufer Halpin; Anna Uehara; Christopher A. Elkins; Clinton R. Paden; Haibin Wang; Jasmine Padilla; Jing Zhang; Justin Lee; Krista Queen; Mary S. Keckler; Rachel Marine; Suxiang Tong; Yan Li; Ying Tao
EPI_ISL_576173	FL Department of Health-Bureau of Epidemiology	Pathogen Discovery, Respiratory Viruses Branch, Division of Viral Diseases, Centers for Disease Control and Prevention	Anna Uehara; Brian Lynch; Clinton R. Paden; Haibin Wang; Jing Zhang; Krista Queen; Peter Cook; Suxiang Tong; Yan Li; Ying Tao
EPI_ISL_428851 to 428852	FSBSI "Chumakov Federal Scientific Center for Research and Development of Immune-and-Biological Products of Russian Academy of Sciences"	FSBSI "Chumakov Federal Scientific Center for Research and Development of Immune-and-Biological Products of Russian Academy of Sciences" & NRC "Kurchatov institute"	Anastasia Berestovskaya; Anastasia Piniaeva; Anna Shishova; Aydar Ishmukhametov; Denis Protsenko; Egor Prokhorchuk; Georgy Ignatyev; Liubov Kozlovskaya; Mikhail Rychev

EPI_ISL_445277	FUNDACION DE SALUD EL TENIENTE	Instituto de Salud Publica de Chile	Alejandra Acevedo; Andrés E Castillo; Bárbara Parra; Carolina Tambley; Gabriel Leal; Jaime Lagos; Jorge Fernandez; Loredana Arata; Patricia Bustos; Paz Tapia; Rodrigo Fasce; Winston Andrade
EPI_ISL_417010, EPI_ISL_418252	FUNDACION JIMENEZ DIAZ	Instituto de Salud Carlos III	A. Monzón; F. Casas; I. Fernández Roblas, R.; I. Jiménez; Iglesias-Caballero; M. Camarero; M. Cuesta; M. González-Esguevillas; M. Molinero Calamita; M. Zaballos; P. Jiménez; S. Juliá; S. Pozo; S. Varona
EPI_ISL_468747 to 468751	Facultad de Medicina UC	Center for Mathematical Modeling and Center for Genome Regulation. Santiago, Chile	Allende ML; Ferres M.; Gaete A; González M; Maass A; Palma R; Travisany D; Urra C; Varas M
EPI_ISL_752605	Faculty of Medicine, Al-Quds University	Faculty of Medicine, Al-Quds University	A. and Al-Jawabreh, A.; Ereqat, S.; Nasereddin
EPI_ISL_526686 to 526688, EPI_ISL_548243 to 548244	Faith Laboratory, Immunology Institute, Icahn School of Medicine at Mount Sinai	van Bakel Laboratory, Genetics and Genomics Sciences, Icahn School of Medicine at Mount Sinai	Alexandra Livanos; Alice Chen-Liaw; Ana S. Gonzalez-Reiche; Benjamin R. tenOever; Daisy Hoagland; Divya Jha; Fatima Amanat; Florian Krammer; Francesca Cossarini; Gerold Bongers; Graham J. Britton; Gustavo Martinez-Delgado; Harm Van Bakel; Ilaria Mogno; Joseph Eggers; Lauren Tal Grinspan; Marla C. Dubinsky; Matthew P. Spindler; Michael Tankelevich; Miriam Merad; Rebekah E. Dixon; Saurabh Mehandru and Jeremiah J. Faith; Sophia Sui; Tamar Plitt
EPI_ISL_640033, EPI_ISL_640116, EPI_ISL_640121, EPI_ISL_640123 to 640125, EPI_ISL_1040739	False Bay Hospital wc FBH	NHLS/UCT	Arash Iranzadeh; Bruna Galvao; Carolyn Williamson; Deelan Doolabh; Diana Hardie; Innocent Mudau; Kruger Marais; Lynn Tyers; Marvin Hsiao; Stephen Korsman
EPI_ISL_960105, EPI_ISL_960110	False Bay Hospital wc FBH	National Health Laboratory Service/UCT	Arash Iranzadeh; Bruna Galvao; Carolyn Williamson; Deelan Doolabh; Diana Hardie; Innocent Mudau; Kruger Marais; Lynn Tyers; Marvin Hsiao; Stephen Korsman
EPI_ISL_526937 to 526941, EPI_ISL_526943, EPI_ISL_526946, EPI_ISL_614298, EPI_ISL_614304, EPI_ISL_614307 to 614308, EPI_ISL_614310 to 614312			
see above	Faroese National Reference Laboratory for Fish and Animal Diseases	Faroese National Reference Laboratory for Fish and Animal Diseases	Debes Hammershaimb Christiansen; Maria Marjunardóttir Dahl; Petra Elisabeth Petersen
EPI_ISL_451963, EPI_ISL_451966, EPI_ISL_451969 to 451970	Federal Budget Institution of Science, State Research Center for Applied Microbiology & Biotechnology	Federal Budget Institution of Science, State Research Center for Applied Microbiology & Biotechnology	Abaimova A; Bakhteeva I; Blagodatskikh S; Bogun A; Borzilov A; Chekan L; Chernysh S; Denisenko E; Dentovskaya S; Detushev K; Detusheva E; Dyatlov I; Firstova V; Frolov V; Fursov M; Fursova N; Galkina E; Gapelchenkova T; Goncharova J; Gorbatov A; Hlyntseva A; Ivanov S; Kalmantayev T; Kalmantayeva O; Kanashenko M; Kartsev N; Kartseva A; Khomyakov A; Khramov M; Kislichkina A; Kolchanova A; Koroleva-Ushakova A; Kosilova I; Krasilnikova E; Kuzin V; Kuzina E; Makarova M; Marin M; Novikova T; Platonov M; Podkopaev Y; Ryabko A; Shaikhutdinova R; Shemyakin I; Shishkina L; Silkina M; Sizova A; Skryabin Y; Slukin P; Slukina N; Solomentsev V; Solovieva A; Teymurazov M; Timofeev V; Titareva G; Trunyakova A; Tyurin E; Vagayskaya A; Zeninskaya N; Zhumakaev R
EPI_ISL_436512, EPI_ISL_436549, EPI_ISL_445043, EPI_ISL_450849 to 450850, EPI_ISL_480791, EPI_ISL_480796, EPI_ISL_480801 to 480802, EPI_ISL_480804 to 480806, EPI_ISL_480808 to 480812, EPI_ISL_480815 to 480819, EPI_ISL_480821 to 480842, EPI_ISL_480844 to 480847, EPI_ISL_480850 to 480852, EPI_ISL_480855 to 480859, EPI_ISL_480862 to 480863, EPI_ISL_480866, EPI_ISL_480870 to 480880, EPI_ISL_480882 to 480885, EPI_ISL_480888 to 480889, EPI_ISL_480893, EPI_ISL_480896 to 480910, EPI_ISL_480913 to 480914, EPI_ISL_480916 to 480918, EPI_ISL_480921 to 480922, EPI_ISL_480926 to 480928, EPI_ISL_480933 to 480935, EPI_ISL_480942, EPI_ISL_480944 to 480945, EPI_ISL_480947 to 480951, EPI_ISL_489710 to 489712, EPI_ISL_489716 to 489718, EPI_ISL_489720, EPI_ISL_489722 to 489724, EPI_ISL_489727 to 489731, EPI_ISL_489733 to 489734, EPI_ISL_489737, EPI_ISL_489739, EPI_ISL_489741, EPI_ISL_489745, EPI_ISL_489747, EPI_ISL_489752 to 489754, EPI_ISL_489756, EPI_ISL_489758 to 489765, EPI_ISL_489767, EPI_ISL_489769 to 489770, EPI_ISL_489772 to 489773, EPI_ISL_489777 to 489778, EPI_ISL_489780 to 489782, EPI_ISL_489784 to 489797, EPI_ISL_489799, EPI_ISL_489803, EPI_ISL_489810, EPI_ISL_489817, EPI_ISL_489819, EPI_ISL_489823, EPI_ISL_489827 to 489828, EPI_ISL_495332 to 495334, EPI_ISL_495342, EPI_ISL_495344, EPI_ISL_495346 to 495347, EPI_ISL_495349 to 495351, EPI_ISL_495355, EPI_ISL_495357, EPI_ISL_495359 to 495360, EPI_ISL_495363, EPI_ISL_495371, EPI_ISL_495378 to 495379, EPI_ISL_495384, EPI_ISL_495386 to 495390, EPI_ISL_495393 to 495400, EPI_ISL_495402 to 495404, EPI_ISL_495406 to 495407, EPI_ISL_495409, EPI_ISL_508708 to 508709, EPI_ISL_508711 to 508713, EPI_ISL_508716 to 508721, EPI_ISL_508723 to 508727, EPI_ISL_508729 to 508737, EPI_ISL_508743 to 508746, EPI_ISL_508748 to 508750, EPI_ISL_508752 to 508760, EPI_ISL_508762, EPI_ISL_508765 to 508767, EPI_ISL_508769, EPI_ISL_508771 to 508782, EPI_ISL_508784, EPI_ISL_508786 to 508788, EPI_ISL_508790 to 508805, EPI_ISL_508807, EPI_ISL_508821, EPI_ISL_508836 to 508841, EPI_ISL_509716 to 509719, EPI_ISL_509721 to 509743, EPI_ISL_509745 to 509767, EPI_ISL_509769, EPI_ISL_509771 to 509774, EPI_ISL_509776, EPI_ISL_509778, EPI_ISL_509780 to 509783, EPI_ISL_509786 to 509789, EPI_ISL_509791 to 509797, EPI_ISL_512547, EPI_ISL_512549 to 512550, EPI_ISL_512552, EPI_ISL_512554 to 512568, EPI_ISL_512570 to 512573, EPI_ISL_512575, EPI_ISL_512578 to 512579, EPI_ISL_512676, EPI_ISL_514140 to 514148, EPI_ISL_514151 to 514153, EPI_ISL_514155 to 514163, EPI_ISL_514168 to 514169, EPI_ISL_514171, EPI_ISL_514176 to 514178, EPI_ISL_514180, EPI_ISL_514183 to 514184, EPI_ISL_514186 to 514189, EPI_ISL_514197, EPI_ISL_514201 to 514205, EPI_ISL_514207, EPI_ISL_514211 to 514216, EPI_ISL_514219 to 514220, EPI_ISL_514222 to 514225, EPI_ISL_517772, EPI_ISL_517778, EPI_ISL_517790 to 517792, EPI_ISL_517794 to 517795, EPI_ISL_517797 to 517798, EPI_ISL_517800 to 517810, EPI_ISL_517812 to 517817, EPI_ISL_517819, EPI_ISL_517821, EPI_ISL_517823, EPI_ISL_517825, EPI_ISL_517827 to 517836, EPI_ISL_517839 to 517840, EPI_ISL_517842 to 517844, EPI_ISL_517846 to 517847, EPI_ISL_517849 to 517850, EPI_ISL_517852, EPI_ISL_517854, EPI_ISL_517857 to 517871, EPI_ISL_517873, EPI_ISL_517875, EPI_ISL_517877 to 517880, EPI_ISL_517882 to 517883, EPI_ISL_517885, EPI_ISL_517888 to 517890, EPI_ISL_517892 to 517897, EPI_ISL_517899 to 517900, EPI_ISL_517902 to 517912, EPI_ISL_517914 to 517916, EPI_ISL_517918, EPI_ISL_517920 to 517926, EPI_ISL_517928 to 517934, EPI_ISL_517936 to 517937, EPI_ISL_517939 to 517941, EPI_ISL_517944 to 517947, EPI_ISL_517949 to 517954, EPI_ISL_517956, EPI_ISL_526553, EPI_ISL_526555, EPI_ISL_526563, EPI_ISL_526567 to 526573, EPI_ISL_526575, EPI_ISL_526580 to 526581, EPI_ISL_526583, EPI_ISL_526585 to 526586, EPI_ISL_526588 to 526600, EPI_ISL_526602 to 526603, EPI_ISL_526605 to 526608, EPI_ISL_526610 to 526614, EPI_ISL_526616 to 526638, EPI_ISL_526643 to 526646, EPI_ISL_526648 to 526649, EPI_ISL_526651 to 526653, EPI_ISL_526656, EPI_ISL_526659 to 526667, EPI_ISL_526669 to 526682, EPI_ISL_549184, EPI_ISL_549189, EPI_ISL_549193 to 549197, EPI_ISL_549199 to 549208, EPI_ISL_549210 to 549227, EPI_ISL_549231 to 549232, EPI_ISL_549236 to 549241, EPI_ISL_549245, EPI_ISL_549247 to 549251, EPI_ISL_549253, EPI_ISL_549255, EPI_ISL_549258 to 549259, EPI_ISL_549262, EPI_ISL_549264 to 549269, EPI_ISL_568587, EPI_ISL_568590 to 568591, EPI_ISL_568595, EPI_ISL_568597 to 568604, EPI_ISL_568606 to 568608, EPI_ISL_568610, EPI_ISL_568612, EPI_ISL_568616 to 568628, EPI_ISL_568633, EPI_ISL_568637 to 568640, EPI_ISL_568644 to 568645, EPI_ISL_568648 to 568649, EPI_ISL_568651 to 568652, EPI_ISL_568654, EPI_ISL_568656 to 568669, EPI_ISL_568671, EPI_ISL_568675 to 568679, EPI_ISL_568681, EPI_ISL_594279 to 594282, EPI_ISL_594284, EPI_ISL_594288 to 594291, EPI_ISL_594293 to 594297, EPI_ISL_594299 to 594308, EPI_ISL_594310 to 594314, EPI_ISL_594316, EPI_ISL_594324, EPI_ISL_594332, EPI_ISL_594334, EPI_ISL_594336 to 594337, EPI_ISL_594340 to 594347, EPI_ISL_594349 to 594355, EPI_ISL_594357, EPI_ISL_594360 to 594369, EPI_ISL_594371, EPI_ISL_594373 to 594376, EPI_ISL_594378, EPI_ISL_594380, EPI_ISL_594385, EPI_ISL_594388 to 594389, EPI_ISL_594391, EPI_ISL_613769 to 613771, EPI_ISL_613829 to 613834, EPI_ISL_613837, EPI_ISL_613843 to 613950, EPI_ISL_653098, EPI_ISL_653100 to 653115, EPI_ISL_653117 to 653120, EPI_ISL_653122 to 653124, EPI_ISL_653126 to 653128, EPI_ISL_653130, EPI_ISL_653132 to 653133, EPI_ISL_653137, EPI_ISL_653139 to 653141, EPI_ISL_653145 to 653148, EPI_ISL_653153, EPI_ISL_653157, EPI_ISL_653162 to 653164, EPI_ISL_653169 to 653170, EPI_ISL_653181, EPI_ISL_653185, EPI_ISL_653191, EPI_ISL_653193, EPI_ISL_653200, EPI_ISL_653203 to 653204, EPI_ISL_653206 to 653207, EPI_ISL_653211, EPI_ISL_653214, EPI_ISL_653218, EPI_ISL_653220 to 653221, EPI_ISL_653224, EPI_ISL_653226, EPI_ISL_653228, EPI_ISL_653232 to 653233, EPI_ISL_653236, EPI_ISL_653245, EPI_ISL_653253 to 653259, EPI_ISL_653261, EPI_ISL_653263, EPI_ISL_653265 to 653269, EPI_ISL_653272 to 653278, EPI_ISL_653280 to 653281, EPI_ISL_653284, EPI_ISL_653288, EPI_ISL_653295 to 653297, EPI_ISL_653300, EPI_ISL_653302 to 653304, EPI_ISL_653306, EPI_ISL_653317, EPI_ISL_653320 to 653321, EPI_ISL_653323 to 653324, EPI_ISL_653326, EPI_ISL_653329, EPI_ISL_653331, EPI_ISL_653333, EPI_ISL_653339, EPI_ISL_653341, EPI_ISL_666825, EPI_ISL_666833, EPI_ISL_666836 to 666837, EPI_ISL_666840, EPI_ISL_666842, EPI_ISL_666847, EPI_ISL_666849, EPI_ISL_666852, EPI_ISL_666854, EPI_ISL_763342, EPI_ISL_767416, EPI_ISL_802725, EPI_ISL_852861			
see above	Florida Bureau of Public Health Laboratories	Florida Bureau of Public Health Laboratories	Jason Blanton; Sarah Schmedes
EPI_ISL_541144, EPI_ISL_541150, EPI_ISL_541154 to 541155, EPI_ISL_541158, EPI_ISL_541164, EPI_ISL_541168, EPI_ISL_541173 to 541176, EPI_ISL_541178 to 541182, EPI_ISL_541185 to 541189, EPI_ISL_541191 to 541193, EPI_ISL_541195 to 541196, EPI_ISL_541198 to 541201, EPI_ISL_541204 to 541205, EPI_ISL_541209 to 541210, EPI_ISL_541213, EPI_ISL_541215, EPI_ISL_541217, EPI_ISL_541219 to 541222, EPI_ISL_541225 to 541226, EPI_ISL_541231 to 541234, EPI_ISL_541236 to 541237, EPI_ISL_541239 to 541241, EPI_ISL_541244, EPI_ISL_541246, EPI_ISL_541250, EPI_ISL_541253, EPI_ISL_541255 to 541257, EPI_ISL_541259, EPI_ISL_541261 to 541269, EPI_ISL_541271 to 541272, EPI_ISL_541277 to 541279, EPI_ISL_541281 to 541282, EPI_ISL_541285 to 541287, EPI_ISL_541289, EPI_ISL_541291 to 541292, EPI_ISL_541294 to 541295, EPI_ISL_541297, EPI_ISL_541299, EPI_ISL_541301 to 541303, EPI_ISL_541309, EPI_ISL_541315, EPI_ISL_541318 to 541319, EPI_ISL_541323, EPI_ISL_541331			
see above	Florida Bureau of Public Health Laboratories, Florida Department of Health	Florida Bureau of Public Health Laboratories, Florida Department of Health	Blanton, J.; Schmedes, S.
EPI_ISL_631563, EPI_ISL_631579, EPI_ISL_631586, EPI_ISL_631602, EPI_ISL_631606, EPI_ISL_631616, EPI_ISL_631620, EPI_ISL_631701, EPI_ISL_631710, EPI_ISL_631724 to 631725, EPI_ISL_631732, EPI_ISL_631756, EPI_ISL_631766, EPI_ISL_631769 to 631771, EPI_ISL_631773 to 631774, EPI_ISL_631787, EPI_ISL_631826, EPI_ISL_631848, EPI_ISL_631851, EPI_ISL_631855, EPI_ISL_631885, EPI_ISL_631891, EPI_ISL_631893 to 631894, EPI_ISL_631906 to 631907, EPI_ISL_631918, EPI_ISL_631923, EPI_ISL_631929, EPI_ISL_631931, EPI_ISL_631955, EPI_ISL_631959 to 631961, EPI_ISL_631964, EPI_ISL_631968, EPI_ISL_631986, EPI_ISL_631989, EPI_ISL_631991, EPI_ISL_632003, EPI_ISL_632010, EPI_ISL_632018 to 632020, EPI_ISL_632022, EPI_ISL_632024, EPI_ISL_632027, EPI_ISL_632034, EPI_ISL_632097, EPI_ISL_632103 to 632105, EPI_ISL_632119 to 632120, EPI_ISL_632122, EPI_ISL_632124 to 632127, EPI_ISL_632132 to 632134, EPI_ISL_632137 to 632140, EPI_ISL_632144, EPI_ISL_632174, EPI_ISL_632181, EPI_ISL_632185, EPI_ISL_632190, EPI_ISL_632194, EPI_ISL_632196 to 632197, EPI_ISL_632211 to 632213, EPI_ISL_632220, EPI_ISL_632222 to 632226, EPI_ISL_632237, EPI_ISL_632947, EPI_ISL_632951 to 632952, EPI_ISL_632954 to 632956, EPI_ISL_632960, EPI_ISL_632966 to 632968			
see above	Flushing Hospital Medical Center	New York City Public Health Laboratory	Jade Wang; et al.
EPI_ISL_470882, EPI_ISL_500776 to 500778, EPI_ISL_590883 to 590884, EPI_ISL_590899, EPI_ISL_590981, EPI_ISL_590985, EPI_ISL_591008, EPI_ISL_635063 to 635065, EPI_ISL_635098, EPI_ISL_635127, EPI_ISL_635167, EPI_ISL_635170, EPI_ISL_635173, EPI_ISL_635176 to 635177, EPI_ISL_708073 to 708078, EPI_ISL_708176 to 708179			
see above	Foerde Hospital, Department of Microbiology	Norwegian Institute of Public Health, Department of Virology	Hilde Elshaug; Hilde Vollan; Kamilla Heddeland Instefjord; Karoline Bragstad; Kathrine Stene-Johansen; Marie Paulsen Madsen; Olav Hungnes; Rasmus Riis Kopperud
EPI_ISL_455903, EPI_ISL_475152, EPI_ISL_476139	Folkhalsomyndigheten	The Public Health Agency of Sweden	Anna Risberg; Anna-Malin Linde; Karin Tegmark-Wisell; Maria Lind Karlberg; Mattias Haukland; Olov Svartstrom; Oskar Karlsson Lindsjo; Petra Edquist; Reza Advani; Sandra Broddesson; Shamam Muradrasoli
EPI_ISL_434650, EPI_ISL_475536, EPI_ISL_475541	Follinge Halsocentral	The Public Health Agency of Sweden	Anna Risberg; Anna-Malin Linde; Karin Tegmark-Wisell; Kerstin Persson Moberg; Maria Lind Karlberg; Mattias Haukland; Mia Brytting; Olov Svartstrom; Oskar Karlsson Lindsjo; Reza Advani; Sandra Broddesson; Theresa Enkirch
EPI_ISL_581486 to 581493	Fondation Congolaise pour la recherche medicale (FCRM)	NGS Competence Center Tübingen, Institut für Medizinische Mikrobiologie und Hygiene, Universitätsklinikum Tübingen	Angel Angelov
EPI_ISL_902756	Fondazione Policlinico Universitario "A. Gemelli" IRCCS	INMI Lazzaro Spallanzani IRCCS	A Di Caro; B Bartolini; C.E.M Gruber; E Giombini; F Messina; M Rueca; M Sanguinetti; MR Capobianchi; O Butera; P Cattani
EPI_ISL_420145	Forde Hospital Department of Microbiology	Norwegian Institute of Public Health, Department of Virology	Hilde Elshaug; Kamilla Heddeland Instefjord; Karoline Bragstad; Kathrine Stene-Johansen; Olav Hungnes
EPI_ISL_413587	Foundation Elisabeth-Tweesteden Ziekenhuis	Erasmus Medical Center	Anne van der Linden; Annemiek van der Eijk; Aura Timen; Bas Oude Munnink; Claudia Schapendonk; Corien Swaan; Corine GeurtsvanKessel; David Nieuwenhuijse; Irina Chestakova; Jeroen van Kampen; Jolanda Voermans; Madelief Mollers; Manon Haverkate; Marion Koopmans; Mark Pronk; Mart Stein; Pascal Lexmond; Reina Sikkema; Richard Molenkamp; Sandra Kengne Kamga Mobou; on behalf of the Dutch national COVID-19 response team.

EPI_ISL_672423 to 672425	Fresno County Public Health Laboratory	Chan-Zuckerberg Biohub	CZB Cliahub Consortium
EPI_ISL_491111	Friedrich-Loeffler-Institut, Laboratory for NGS and Microarray Diagnostics	Friedrich-Loeffler-Institut, Laboratory for NGS and Microarray Diagnostics	Dirk Höper; Laboratory for NGS and Microarray Diagnostics
EPI_ISL_411060, EPI_ISL_411066, EPI_ISL_431118, EPI_ISL_431180, EPI_ISL_431240, EPI_ISL_431782 to 431785	Fujian Center for Disease Control and Prevention	Fujian Center for Disease Control and Prevention	Chen Wei; He Wenxiang; Huang Zhimiao; Lin Qi; Weng Yuwei; Zhang Yanhua
EPI_ISL_707928	Fujita Health University Hospital	Fujita Health University School of Medicine, Department of Microbiology	Aki Sakurai; Masahiro Suzuki; Yohei Doi
EPI_ISL_728000	Fujita Health University Okazaki Medical Center	Department of Virology and Parasitology, Fujita Health University School of Medicin	Aki Sakurai; Masahiro suzuki; Satoshi Komoto; Takayuki Murata; Takuma Ishihara; Tomihiko Ide; Yohei Doi
EPI_ISL_718273, EPI_ISL_727998 to 727999, EPI_ISL_728153 to 728156, EPI_ISL_728159	Fujita Health University Okazaki Medical Center	Department of Virology and Parasitology, Fujita Health University School of Medicine	Aki Sakurai; Masahiro suzuki; Satoshi Komoto; Takayuki Murata; Takuma Ishihara; Tomihiko Ide; Yohei Doi
EPI_ISL_728206 to 728208	Fujita Health University Okazaki Medical Center	Fujita health University, Department of Microbiology	Aki Sakurai; Masahiro suzuki; Satoshi Komoto; Takayuki Murata; Takuma Ishihara; Tomihiko Ide; Yohei Doi
EPI_ISL_479967 to 479978, EPI_ISL_480120 to 480136, EPI_ISL_480138 to 480168	Fukui Prefectural Institute of Public Health and Environmental Science	Pathogen Genomics Center, National Institute of Infectious Diseases	Hajime Kamiya; Kentaro Itokawa; Makoto Kuroda; Masanori Hashino; Miho Toho; Motoi Suzuki; Rina Tanaka; Tsuyoshi Sekizuka
EPI_ISL_684408 to 684483, EPI_ISL_684634 to 684638, EPI_ISL_684640 to 684692, EPI_ISL_684723 to 684727, EPI_ISL_685918 to 685922, EPI_ISL_685924, EPI_ISL_687533 to 687560, EPI_ISL_688612, EPI_ISL_688619 to 688623, EPI_ISL_688634, EPI_ISL_688897 to 688968, EPI_ISL_689460 to 689496, EPI_ISL_689692 to 689697, EPI_ISL_689759 to 689825, EPI_ISL_690432 to 690526, EPI_ISL_690982 to 690986, EPI_ISL_691550 to 691551, EPI_ISL_691764 to 691765, EPI_ISL_692048 to 692050, EPI_ISL_692219 to 692229, EPI_ISL_692442 to 692497			
see above	Fukuoka Institute of Health and Environmental Sciences	Pathogen Genomics Center, National Institute of Infectious Diseases	Kentaro Itokawa; Makoto Kuroda; Masanori Hashino; Rina Tanaka; Tsuyoshi Sekizuka
EPI_ISL_492074	Functional Genomics Core University of South Carolina / Prisma Health-Midlands	Functional Genomics Core, University of South Carolina,	Alyssa Clay-Glimour; B.Celia Cui; Diego Altomare; Hao Ji; Helmut Albrecht; Mengqian Chen; Michael Shtutman; Michael Wyatt; Phillip Buckhaults
EPI_ISL_491481	Functional Genomics Core University of South Carolina / Prisma Health-Midlands	Functional Genomics Core, Center For Targeted Therapeutics,	Alyssa Clay-Glimour; B.Celia Cui; Diego Altomare; Hao Ji; Helmut Albrecht; Mengqian Chen; Michael Shtutman; Michael Wyatt; Phillip Buckhaults
EPI_ISL_493209 to 493212, EPI_ISL_493427, EPI_ISL_494553 to 494555	Functional Genomics Core University of South Carolina / Prisma Health-Midlands	Functional Genomics Core, University of South Carolina	Alyssa Clay-Glimour; B.Celia Cui; Diego Altomare; Hao Ji; Helmut Albrecht; Mengqian Chen; Michael Shtutman; Michael Wyatt; Phillip Buckhaults
EPI_ISL_493129, EPI_ISL_493426	Functional Genomics Core University of South Carolina / Prisma Health-Midlands	Functional Genomics Core, University of South Carolina,	Alyssa Clay-Glimour; B.Celia Cui; Diego Altomare; Hao Ji; Helmut Albrecht; Mengqian Chen; Michael Shtutman; Michael Wyatt; Phillip Buckhaults
EPI_ISL_419235 to 419237	Fundacion Jimenez Diaz	Instituto de Salud Carlos III	Camarero, S.; Casas, I.; Cuesta, I.; Fernández, R.; González-Esguevillas, M.; Iglesias-Caballero, M.; Jiménez, M.; Jiménez, P.; Juliá, M.; Molinero Calamita, M.; Monzón, S.; Pozo, F.; Varona, S.; Zaballos, A.
EPI_ISL_462447 to 462449	Fundació Lluita contra la SIDA (FLSida)/Hospital Universitari Germans Trias i Pujol	IrsiCaixa AIDS Research Lab	Bonaventura Clotet; Joaquim Segalés; Jorge Carrillo; Julia Blanco; Lidia Ruiz; Marc Corbacho; Marc Noguera-Julian; Maria Pilar Armengol; Maria Ubals; Mariona Parera; Nuria Izquierdo; Oriol Mitjà; Roger Paredes
EPI_ISL_462478	Fundación Jiménez Díaz	Instituto de Salud Carlos III	A. Monzón; F. Casas; I; I. Jiménez; Iglesias-Caballero; M. Camarero; M. Cuesta; M. González-Esguevillas; M. Molinero Calamita; M. Zaballos; P. Jiménez; R. Fernández; S. Juliá; S. Pozo; S. Varona
EPI_ISL_420141, EPI_ISL_420146, EPI_ISL_420313, EPI_ISL_493356 to 493360, EPI_ISL_493362 to 493368, EPI_ISL_493370 to 493373, EPI_ISL_493375 to 493378, EPI_ISL_500769 to 500771, EPI_ISL_500773 to 500774, EPI_ISL_500785 to 500787, EPI_ISL_500789 to 500790, EPI_ISL_549027 to 549028, EPI_ISL_549038, EPI_ISL_549048 to 549049, EPI_ISL_549053 to 549058, EPI_ISL_549060 to 549069, EPI_ISL_549071 to 549080, EPI_ISL_549082, EPI_ISL_549088, EPI_ISL_549112, EPI_ISL_549119 to 549122, EPI_ISL_549124, EPI_ISL_549132, EPI_ISL_549143, EPI_ISL_549154, EPI_ISL_549165, EPI_ISL_549168, EPI_ISL_590881, EPI_ISL_590898, EPI_ISL_590901 to 590903, EPI_ISL_590912, EPI_ISL_635086 to 635087, EPI_ISL_668396 to 668397, EPI_ISL_708035, EPI_ISL_708057 to 708059, EPI_ISL_708126 to 708130, EPI_ISL_759973 to 759974, EPI_ISL_759984 to 759985, EPI_ISL_775273 to 775274, EPI_ISL_860213, EPI_ISL_965001, EPI_ISL_1013468, EPI_ISL_1013518 to 1013519, EPI_ISL_1034313, EPI_ISL_1040145			
see above	Furst Medical Laboratory	Norwegian Institute of Public Health, Department of Virology	Atiya R Ali; Engebretsen Serina Beate Atiya R Ali; Garcia Llorente Ignacio; Hilde Elshaug; Hilde Synnøve Vollan; Hilde Vollan; Ignacio Garcia Llorente; Kamilla Heddeland Instefjord; Karoline Bragstad; Kathrine Stene-Johansen; Marie Paulsen Madsen; Olav Hungnes; Rasmus Riis Kopperud; Serina B Engebretsen
EPI_ISL_522549 to 522550	Félix Guyon Hospital	UMR PIMIT Université de La Réunion	Camille Lebarbenchon; David Wilkinson; Patrick Mavingui
EPI_ISL_912674 to 912676, EPI_ISL_981547, EPI_ISL_982307 to 982310	G.H.E.F.Grand Hôpital EST Francilien	Department of Virology, Henri Mondor University Hospital, Assistance Publique Hôpitaux de Paris, Université Paris-Est Créteil, INSERM U955	Alexandre Soulier; Christophe Rodriguez; Elisabeth Trawinski; Guillaume Gricourt; Jean-Michel Pawlotsky; Melissa N'Debi; Slim Fourati; Vanessa Demontant
EPI_ISL_420786 to 420788	GA Department of Public Health	Pathogen Discovery, Respiratory Viruses Branch, Division of Viral Diseases, Centers for Disease Control and Prevention	Alison S. Laufer Halpin; Anne Uehara; Christopher A. Elkins; Clinton R. Paden; Haibin Wang; Jasmine Padilla; Jing Zhang; Justin Lee; Krista Queen; Mary S. Keckler; Rachel Marine; Suxiang Tong; Yan Li; Ying Tao
EPI_ISL_419556 to 419557, EPI_ISL_424858 to 424864, EPI_ISL_426417 to 426419, EPI_ISL_527658 to 527665, EPI_ISL_566085 to 566107, EPI_ISL_576189 to 576195, EPI_ISL_647987 to 647989, EPI_ISL_648002 to 648003, EPI_ISL_681776 to 681828, EPI_ISL_955302 to 955303			
see above	GA Department of Public Health Laboratory	Pathogen Discovery, Respiratory Viruses Branch, Division of Viral Diseases, Centers for Disease Control and Prevention	Alison S. Laufer Halpin; Anna Montmayeur; Anna Uehara; Brian Lynch; Christopher A. Elkins; Clinton R. Paden; Haibin Wang; Jasmine Padilla; Jing Zhang; Justin Lee; Krista Queen; Mary S. Keckler; Peter Cook; Rachel Marine; Suxiang Tong; Yan Li; Ying Tao
EPI_ISL_475058 to 475059, EPI_ISL_495065 to 495066	GAIMS & G K General Hospital	Gujarat Biotechnology Research Centre	; A M Kadri; Afzal Ansari; Apurvasinh Puvar; Babulal Babhoria; Chaitanya Joshi; Dinesh Kumar; Harsh Bakshi; Hitesh Assudani; Janvi Raval; Komal Patel; Labdhi Pandya; Madhvi Joshi; Maharshi Pandya; Monika Gandhi; Neha Rajpara; Nidhi Patel; Nikha Trivedi; Nitin Savaliya; Pinal Trivedi; Pragya Sharma; Pritesh Sabara; R D Dixit; Raghawendra Kumar; Snehal Bagatharia; Zarna Patel; Zuber Saiyed
EPI_ISL_586569 to 586570	GCRI,Ahmedabad	Gujarat Biotechnology Research Centre	A M Kadri; Afzal Ansari; Apurvasinh Puvar; Chaitanya Joshi; Dinesh Kumar; Harsh Bakshi; Harsha Panchal; Janvi Raval; Komal Patel; Labdhi Pandya; Madhvi Joshi; Maharshi Pandya; Monika Gandhi; Nidhi Patel; Nikha Trivedi; Nitin Savaliya; Pinal Trivedi; R D Dixit; Raghawendra Kumar; Shashank Pandya; Zarna Patel; Zuber Saiyed
EPI_ISL_418416, EPI_ISL_508878, EPI_ISL_509003 to 509004	GH Les Portes du Sud	CNR Virus des Infections Respiratoires - France SUD	Alexandre Gaymard; Antonin Bal; Bruno Lina; Carine Moustaud; Florence Morfin-Sherpa; Gregory Destras; Gwendolyne Burfin; Laurence Josset; Martine Valette; Maude Bouscambert-Duchamp; Raphaelle Lamy; Solenne Brun
EPI_ISL_428351 to 428352, EPI_ISL_428363	GH Nord Essonne Service de Biologie clinique	National Reference Center for Viruses of Respiratory Infections, Institut Pasteur, Paris	Angela Brisebarre; Etienne Simon-Lorière; Flora Donati; Marion Barbet; Maud Vanpeene; Mélanie Albert; Méline Bizard; Sylvie Behillil; Sylvie van der Werf; Vincent Enouf
EPI_ISL_1007633	GHOL	Laboratory of genomics and metagenomics, Institute of Microbiology, University Hospital Centre and University of Lausanne, Switzerland	Claire Bertelli; Damien Jacot; Gilbert Greub; Sébastien Aeby; Trestan Pillonel
EPI_ISL_586562 to 586566	GMERS GMERS Medical College and Hospital, Junagadh	Gujarat Biotechnology Research Centre	A M Kadri; Afzal Ansari; Apurvasinh Puvar; Bhavesh Bagda; Chaitanya Joshi; Dinesh Kumar; Harsh Bakshi; Janvi Raval; Komal Patel; Labdhi Pandya; Madhvi Joshi; Maharshi Pandya; Monika Gandhi; Nidhi Patel; Nikha Trivedi; Nitin Savaliya; Pinal Trivedi; R D Dixit; Raghawendra Kumar; Ravi Dedaniya; Zarna Patel; Zuber Saiyed
EPI_ISL_469037 to 469042, EPI_ISL_475047 to 475055	GMERS Medical College & Hospital	Gujarat Biotechnology Research Centre	A M Kadri; Afzal Ansari; Ankit Hinsu; Apurvasinh Puvar; Armi Chaudhari; Bhavya Jindal; Chaitanya Joshi; Dinesh Kumar; Fenil Patel; Harsh Bakshi; Janvi Raval; Komal Patel; Labdhi Pandya; Madhvi Joshi; Maharshi Pandya; Meenakshi Shah; Monika Gandhi; Neelam Nathani; Neena Doshi; Neha Rajpara; Nidhi Patel; Nitin Savaliya; Pinal Trivedi; Pooja P Doshi; Pragya Sharma; Pritesh Sabara; Priyanka P Vatsa; R D Dixit; Raghawendra Kumar; Snehal Bagatharia; Tejas Shah; Varsha Godbole; Zarna Patel; Zuber Saiyed
EPI_ISL_476855 to 476862, EPI_ISL_495026 to 495062, EPI_ISL_500946, EPI_ISL_514610, EPI_ISL_524730 to 524731	GMERS Medical College & Hospital, Gotri, Vadodara	Gujarat Biotechnology Research Centre	; A M Kadri; Afzal Ansari; Apurvasinh Puvar; Chaitanya Joshi; Dinesh Kumar; Harsh Bakshi; Janvi Raval; Kamlesh J Upadhyay; Komal Patel; Labdhi Pandya; Madhvi Joshi; Maharshi Pandya; Meenakshi Shah; Monika Gandhi; Neena Doshi; Nidhi Patel; Nikha Trivedi; Nitin Savaliya; Pinal Trivedi; Pranay Shah; R D Dixit; Raghawendra Kumar; Sanjay Kapadia; Varsha Godbole; Zarna Patel; Zuber Saiyed

EPI_ISL_495063 to 495064	GMERS Medical College & Hospital, Himmatnagar	Gujarat Biotechnology Research Centre	; A M Kadri; Afzal Ansari; Apurvasinh Puvar; Chaitanya Joshi; Dinesh Kumar; Harsh Bakshi; Himanshu Khatri; Janvi Raval; Komal Patel; Labdhi Pandya; Madhvi Joshi; Maharshi Pandya; Mayur Gandhi; Monika Gandhi; Nidhi Patel; Nikha Trivedi; Nitin Savaliya; Pinal Trivedi; R D Dixit; Raghawendra Kumar; Zarna Patel; Zuber Saiyed
EPI_ISL_483823 to 483828	GMERS Medical College Himmatnagar	Gujarat Biotechnology Research Centre	A M Kadri; Afzal Ansari; Apurvasinh Puvar; Chaitanya Joshi; Dinesh Kumar; Harsh Bakshi; Himanshu Khatri; Janvi Raval; Komal Patel; Labdhi Pandya; Madhvi Joshi; Maharshi Pandya; Mayur Gandhi; Monika Gandhi; Nidhi Patel; Nikha Trivedi; Nitin Savaliya; Pinal Trivedi; R D Dixit; Raghawendra Kumar; Zarna Patel; Zuber Saiyed
EPI_ISL_524733 to 524734, EPI_ISL_524752 to 524766	GMERS Medical College and Hospital, Dharpur, Patan	Gujarat Biotechnology Research Centre	A M Kadri; A N Parmar; Afzal Ansari; Apurvasinh Puvar; Chaitanya Joshi; Dinesh Kumar; Harsh Bakshi; Janvi Raval; Komal Patel; Labdhi Pandya; Madhvi Joshi; Maharshi Pandya; Monika Gandhi; Nidhi Patel; Nikha Trivedi; Nitin Savaliya; Pinal Trivedi; R D Dixit; Raghawendra Kumar; Zarna Patel; Zuber Saiyed
EPI_ISL_447047 to 447053, EPI_ISL_447547 to 447555, EPI_ISL_467029 to 467038, EPI_ISL_476863 to 476866, EPI_ISL_483820, EPI_ISL_514611 to 514612, EPI_ISL_524737 to 524745, EPI_ISL_525419 to 525420			
see above	GMERS Medical College and Hospital, Gandhinagar	Gujarat Biotechnology Research Centre	A M Kadri; Afzal Ansari; Akanksha Verma; Amit Kanani; Anjali Rajwar; Ankit Hinsu; Apurvasinh Puvar; Armi Chaudhari; Bharti Rajani; Bhavesh Modi; Bhavya Jindal; Binita Aring; Chaitanya Joshi; Dinesh Kumar; Dipa Kinariwala; Dipeshwari Shewale; Disha Patel; Fenil Patel; Gaurishankar Shrimali; Geeta Vaghela; Harsh Bakshi; Janvi Raval; Kairavi Joshi; Kamlesh J Upadhyay; Komal Patel; Labdhi Pandya; Madhvi Joshi; Maharshi Pandya; Monika Gandhi; Neelam Nathani; Neeta Khandelwal; Neha Rajpara; Nidhi Patel; Nidhi Sood; Nikha Trivedi; Nitin Savaliya; Pinal Trivedi; Pooja P Doshi; Pragya Sharma; Pranay Shah; Pritesh Sabara; Priti Pandita; Priyanka P Vatsa; R D Dixit; Raghawendra Kumar; Ramesh Pandit; Sanjay Kapadia; Seema Bhatt; Sharmistha Majumdar; Snehal Bagatharia; Sonia Barve; Tejas Shah; Zarna Patel; Zuber Saiyed
EPI_ISL_825054	GMERS Medical College and Hospital, Gotri, Vadodara	Gujarat Biotechnology Research Centre	Afzal Ansari; Apurvasinh Puvar; Bithika Duttaroy; Chaitanya Joshi; Dinesh Kumar; Janvi Raval; Kalpesh Mistry; Madhvi Joshi; Mitesh Kamothi; Nikha Trivedi; Nitin Savaliya; Ramesh Pandit; Zarna Patel; Zuber Saiyed
EPI_ISL_888823, EPI_ISL_888825, EPI_ISL_939654	GZA Sint-Augustinus Hospital	UAntwerp, Laboratory of Medical Microbiology, Campus Drie Eiken S6.26, Universiteitsplein 1, 2610, Wilrijk, Antwerp, Belgium	Basil Britto Xavier; Christine Lammens; Herman Goossens; Jasmine Coppens; Marie Le Mercier; Veerle Matheeussen
EPI_ISL_583426, EPI_ISL_583429, EPI_ISL_583434 to 583436, EPI_ISL_583441 to 583442, EPI_ISL_583458 to 583459, EPI_ISL_583463 to 583464, EPI_ISL_583467, EPI_ISL_583473, EPI_ISL_583477			
see above	Garcia-Sastre Laboratory, Department of Microbiology, Icahn School of Medicine at Mount Sinai	van Bakel Laboratory, Genetics and Genomics Sciences, Icahn School of Medicine at Mount Sinai	Adolfo García-Sastre; Adriana van de Guchte; Ajay Obla; Ana S. Gonzalez-Reiche; Genovefa Papanicolaou; Gunjan Shah; Harm van Bakel; Jayeeta Dutta; Judith Aberg; Kent Sepkowitz; Miguel-Angel Perales; Ngolela Esther Babady; Sadaf Aslam; Teresa Aydillo; Tobias Hohl; Zenab Khan; and Mini Kamboj
EPI_ISL_534249 to 534251	Gavle Sjukhus	The Public Health Agency of Sweden	Anna Risberg; Anna-Malin Linde; Karin Tegmark-Wisell; Maria Lind Karlberg; Mattias Haukland; Mia Brytting; Olov Svartstrom; Oskar Karlsson Lindsjo; Petra Edquist; Reza Advani; Sandra Broddesson
EPI_ISL_475110, EPI_ISL_475115, EPI_ISL_582801, EPI_ISL_582809, EPI_ISL_582834, EPI_ISL_615101 to 615103, EPI_ISL_623095, EPI_ISL_648140 to 648142, EPI_ISL_648144, EPI_ISL_648163, EPI_ISL_661283, EPI_ISL_661286 to 661287, EPI_ISL_710608 to 710609			
see above	Gavle klinisk mikrobiologi	The Public Health Agency of Sweden	Anna Risberg; Anna-Malin Linde; Department of Microbiology; Karin Tegmark-Wisell; Maria Lind Karlberg; Mattias Haukland; Mia Brytting; Olov Svartstrom; Oskar Karlsson Lindsjo; Petra Edquist; Reza Advani; Sandra Broddesson; Shamam Muradrasoli; The Public Health Agency of Sweden
EPI_ISL_676514	Gavleborg	The Public Health Agency of Sweden	Department of Microbiology; The Public Health Agency of Sweden
EPI_ISL_730567 to 730568, EPI_ISL_730570, EPI_ISL_730572 to 730577, EPI_ISL_730620, EPI_ISL_984738	Gazi University Faculty of Medicine, Medical Virology Laboratory	Gazi University Faculty of Medicine, Medical Virology Laboratory	Erdem ahin; Gülendam Bozday; Hager Muftah; Il Fidan; Kayhan Çalar; Murat Dizbay; Selin Yiit; Shaknoza Sarzhanova; Özlem Güzel Tunçcan
EPI_ISL_420855, EPI_ISL_420876 to 420877, EPI_ISL_430064 to 430065	Geelong Centre for Emerging Infectious Diseases	Geelong Centre for Emerging Infectious Diseases	Alexandersen S.; Bhatta T.R.; Chamings A.; Chamings, A.; Raj Bhatta T.
EPI_ISL_467300	General Hospital "Abdulah Nakas"	Alea Genetic Center	Damir Marjanovic; Dino Pecar; Enis Kandic; Lana Salihefendic; Nihad Fejzic; Rijad Konjhodzic; Sead Jazic; Teufik Goletic
EPI_ISL_933566, EPI_ISL_933772, EPI_ISL_933786, EPI_ISL_934418, EPI_ISL_942961, EPI_ISL_942963, EPI_ISL_942970, EPI_ISL_957423, EPI_ISL_958751, EPI_ISL_1011755			
see above	General Hospital - Bitola	Research Center for Genetic Engineering and Biotechnology "Georgi D. Efremov" , Macedonian Academy of Sciences and Arts	Aleksandar J. Dimovski; Aleksandar J.Dimovski; Dijana Plasheska-Karanfilska; Gjorgji Bozinovski; Milena Jakimovska; Predrag Noveski; RCGEB - MASA
EPI_ISL_516413, EPI_ISL_677705, EPI_ISL_677707, EPI_ISL_678252, EPI_ISL_910327, EPI_ISL_933573, EPI_ISL_933776, EPI_ISL_934092, EPI_ISL_942964, EPI_ISL_942966 to 942968, EPI_ISL_956421, EPI_ISL_956668, EPI_ISL_1013451, EPI_ISL_1018105			
see above	General Hospital - Kumanovo	Research Center for Genetic Engineering and Biotechnology "Georgi D. Efremov" , Macedonian Academy of Sciences and Arts	Aleksandar J. Dimovski; Aleksandar J.Dimovski; Dijana Plasheska-Karanfilska; Gjorgji Bozinovski; Milen; Milena Jakimovska; Predrag Noveski; RCGEB - MASA; leksandar J. Dimovski
EPI_ISL_677704, EPI_ISL_677709, EPI_ISL_677711, EPI_ISL_677722, EPI_ISL_677725 to 677726, EPI_ISL_678255 to 678256, EPI_ISL_933775, EPI_ISL_933803, EPI_ISL_959291, EPI_ISL_1014934, EPI_ISL_1018107, EPI_ISL_1018113, EPI_ISL_1018115			
see above	General Hospital - Ohrid	Research Center for Genetic Engineering and Biotechnology "Georgi D. Efremov" , Macedonian Academy of Sciences and Arts	Aleksandar J. Dimovski; Dijana Plasheska-Karanfilska; Gjorgji Bozinovski; Milena Jakimovska; Predrag Noveski; RCGEB - MASA
EPI_ISL_514354, EPI_ISL_677674, EPI_ISL_677703, EPI_ISL_677712 to 677714, EPI_ISL_678258, EPI_ISL_933713, EPI_ISL_944786, EPI_ISL_956382 to 956383, EPI_ISL_956419, EPI_ISL_1013583, EPI_ISL_1014762, EPI_ISL_1014766, EPI_ISL_1014936			
see above	General Hospital - Prilep	Research Center for Genetic Engineering and Biotechnology "Georgi D. Efremov" , Macedonian Academy of Sciences and Arts	Aleksandar J. Dimovski; Dijana Plasheska-Karanfilska; Gjorgji Bozinovski; Milena Jakimovska; Predrag Noveski; RCGEB - MASA
EPI_ISL_933571	General Hospital - Shtip	Research Center for Genetic Engineering and Biotechnology "Georgi D. Efr	RCGEB - MASA
EPI_ISL_909742, EPI_ISL_933602, EPI_ISL_933797, EPI_ISL_959288, EPI_ISL_1018104, EPI_ISL_1018117	General Hospital - Shtip	Research Center for Genetic Engineering and Biotechnology "Georgi D. Efremov" , Macedonian Academy of Sciences and Arts	Aleksandar J. Dimovski; Dijana Plasheska-Karanfilska; Gjorgji Bozinovski; Milena Jakimovska; Predrag Noveski; RCGEB - MASA
EPI_ISL_677719, EPI_ISL_678260, EPI_ISL_944108 to 944109, EPI_ISL_1018106, EPI_ISL_1018218	General Hospital - Struga	Research Center for Genetic Engineering and Biotechnology "Georgi D. Efremov" , Macedonian Academy of Sciences and Arts	Aleksandar J. Dimovski; Dijana Plasheska-Karanfilska; Gjorgji Bozinovski; Milena Jakimovska; Predrag Noveski; RCGEB - MASA
EPI_ISL_943993	General Hospital - Strumica	Research Center for Genetic Engineering and Biotechnology "Georgi D. Efremov" , Macedon	Aleksandar J. Dimovski; Dijana Plasheska-Karanfilska; Gjorgji Bozinovski; Predrag Noveski
EPI_ISL_910326	General Hospital - Strumica	Research Center for Genetic Engineering and Biotechnology "Georgi D. Efremov" , Macedonian Academ	RCGEB - MASA
EPI_ISL_678254, EPI_ISL_933717, EPI_ISL_933726, EPI_ISL_943994, EPI_ISL_944105, EPI_ISL_956404, EPI_ISL_1014763			
see above	General Hospital - Strumica	Research Center for Genetic Engineering and Biotechnology "Georgi D. Efremov" , Macedonian Academy of Sciences and Arts	Aleksandar J. Dimovski; Dijana Plasheska-Karanfilska; Gjorgji Bozinovski; Mi; Milena Jakimovska; Predrag Noveski; RCGEB - MASA
EPI_ISL_933574, EPI_ISL_933720, EPI_ISL_943996 to 943997, EPI_ISL_944107, EPI_ISL_944779, EPI_ISL_1014764	General Hospital - Tetovo	Research Center for Genetic Engineering and Biotechnology "Georgi D. Efremov" , Macedonian Academy of Sciences and Arts	Aleksandar J. Dimovski; Dijana Plasheska-Karanfilska; Gjorgji Bozinovski; Milena Jakimovska; Predrag Noveski; RCGEB - MASA

EPI_ISL_677675, EPI_ISL_677710, EPI_ISL_933578, EPI_ISL_933660, EPI_ISL_934415, EPI_ISL_942965, EPI_ISL_942969, EPI_ISL_956405, EPI_ISL_1011756, EPI_ISL_1013097			
see above	General Hospital - Veles	Research Center for Genetic Engineering and Biotechnology "Georgi D. Efremov" , Macedonian Academy of Sciences and Arts	Aleksandar J. Dimovski; Aleksandar J.Dimovski; Dijana Plasheska-Karanfilska; Gjorgji Bozinovski; Milena Jakimovska; Predrag Noveski; RCGEB - MASA
EPI_ISL_406798, EPI_ISL_406800 to 406801	General Hospital of Central Theater Command of People's Liberation Army of China	BGI & Institute of Microbiology, Chinese Academy of Sciences & Shandong First Medical University & Shandong Academy of Medical Sciences & General Hospital of Central Theater Command of People's Liberation Army of China	Weifeng Shi and Zhenhong Hu; Weijun Chen; Yuhai Bi
EPI_ISL_586531 to 586535	General Hospital,Chhotaudepur	Gujarat Biotechnology Research Centre	A M Kadri; Afzal Ansari; Apurvasinh Puvar; Arti Thakur; Chaitanya Joshi; Dharak Pandya; Dinesh Kumar; Harsh Bakshi; Janvi Raval; Komal Patel; Labdhi Pandya; Madhvi Joshi; Maharshi Pandya; Monika Gandhi; Nidhi Patel; Nikha Trivedi; Nitin Savaliya; Pinal Trivedi; R D Dixit; Raghawendra Kumar; Yogesh Parmar; Zarna Patel; Zuber Saiyed
EPI_ISL_586546 to 586548	General Hospital,Godhra	Gujarat Biotechnology Research Centre	A M Kadri; Afzal Ansari; Apurvasinh Puvar; Chaitanya Joshi; Dinesh Kumar; Harsh Bakshi; Janvi Raval; Komal Patel; Labdhi Pandya; Madhvi Joshi; Maharshi Pandya; Monika Gandhi; Neela Katara; Nidhi Patel; Nikha Trivedi; Nitin Savaliya; Pinal Gandhi; Pinal Trivedi; R D Dixit; Raghawendra Kumar; Sangeeta Kumar; Zarna Patel; Zuber Saiyed
EPI_ISL_614282 to 614293, EPI_ISL_623096 to 623097, EPI_ISL_660324 to 660325, EPI_ISL_693766 to 693774	General practitioner	National Reference Center for Viruses of Respiratory Infections, Institut Pasteur, Paris	Angela Brisebarre; Camille Capel; Etienne Simon-Lorière; Marion Barbet; Maud Vanpeene; Méline Bizard; Sylvie Behillil; Sylvie van der Werf; Vincent Enouf
EPI_ISL_746478 to 746484, EPI_ISL_746486 to 746489, EPI_ISL_746492 to 746493, EPI_ISL_746498 to 746825, EPI_ISL_754391 to 754393	Genetica Molecular and Subdepartamento de Virologia ISP Chile	Instituto de Salud Publica de Chile	Andres Castillo; Barbara Parra; Gisselle Barra; Jaime Lagos; Javier Tognarelli; Jorge Fernandez; Loredana Arata; Patricia Bustos; Rodrigo Fasce
EPI_ISL_475570 to 475571, EPI_ISL_475573, EPI_ISL_605783, EPI_ISL_746324	Genome Center	Genome Center	A. S. M. Rubayet- Ul- Alam; Ashok Kumar; Chakrabortty Atonu; Habiba Ibnat; Hassan M. Al-Emran; M. Rafiul Islam; M. Shaminur Rahman; Md Nur Kabidul Azam; Md. Ali Ahasan Setu; Md. Aminul Islam; Md. Anwar Hossain; Md. Hasan al Pramanik; Md. Iqbal Kabir Jahid; Md. Iqbal Kabir Jahid Md. Anwar Hossain; Md. Muzahidul Islam; Md. Nazmul Hasan; Md. Shazid Hasan; Md. Taijul Islam; Md. Tanvir Islam; Md. Zannat Ali; Mithun Talukder Md. Tawyabur; Najmuj Sakib; Nigar Sultana Meghla; Ovinu Kibria Islam; Pravas Chandra Roy; Proshanto Kumar Das; Provakar Mondol; Ruhul Amin; S. M. Tanjil Shah; Selina Akter; Shireen Nigar; Shohanur Rahaman; Shovon Lal Sarkar; Sourav Dutta; Tanay Chakrovarty
EPI_ISL_427391, EPI_ISL_428346, EPI_ISL_428368, EPI_ISL_480228 to 480230, EPI_ISL_480232 to 480289, EPI_ISL_480291 to 480292, EPI_ISL_735260 to 735286, EPI_ISL_735288 to 735290, EPI_ISL_735292 to 735340, EPI_ISL_735342 to 735371, EPI_ISL_735373, EPI_ISL_833147			
see above	Genomic Laboratory (GLAB) (Conjoint lab of Health Directorate of Istanbul and Istanbul Technical University)	Genomic Laboratory (GLAB), Istanbul Technical University	Arzu Irvem; Ayse Serra Ozel; Betsi Kose; Betsi Köse; Bugra Agaoglu; Elifnaz Çelik; Gizem Alkurt; Gizem Dinler Doganay; Ilker Karacan; Jale Yildiz; Levent Doganay; Mehtap Aydin; Mehtap Aydn; Nihat Bugra Agaoglu; Nilsun Altunal; Nisan Denizce Can; Ozlem Akgun Dogan; Pari Sharifli; Payam Zolfagharian; Tugba Kizilboga Akgun; Yasemin Kendir Demirkol
EPI_ISL_512774	Genomic Research Lab, BCSIR	Bangladesh Council of Scientific and Industrial Research	A. K. M. Shamsuzzaman; Abu Sayeed Mohammad Mahmud; Asish Kumar Ghosh; Barna Goswami; Eshrar Osman; Iffat Jahan; Mahmuda Yeasmin; Md. Ahasan Habib; Md. Maruf Ahmed Molla; Md. Murshed Hasan Sarkar; Md. Saddam Hossain; Md. Salim Khan; Mohammad Samir Uzzaman; Salek Ahmed Sajib; Shahina Akter; Sheikh Md. Selim Al Din; Tanjina Akhter Banu; Tasnim Nafisa; Utpal Chandra Ray
EPI_ISL_483688	Genomic Research Lab, BCSIR	Genomic Research Lab, BCSIR	A. K. M. Shamsuzzaman; Abu Sayeed Mohammad Mahmud; Asish Kumar Ghosh; Barna Goswami; Eshrar Osman; Iffat Jahan; Mahmuda Yeasmin; Md. Ahasan Habib; Md. Maruf Ahmed Molla; Md. Murshed Hasan Sarkar; Md. Saddam Hossain; Md. Salim Khan; Mohammad Samir Uzzaman; Salek Ahmed Sajib; Shahina Akter; Sheikh Md. Selim Al Din; Tanjina Akhter Banu; Tasnim Nafisa; Utpal Chandra Ray
EPI_ISL_632908	Genomic Sciences, Rehman Medical Institute	Genomic Sciences, Rehman Medical Institute	Afridi; Ali, J.; H. and Jehanzeb, V.; Haider; Jan; S.A.; Sabiha, B.; U.K.
EPI_ISL_730197, EPI_ISL_730199 to 730223, EPI_ISL_730225 to 730228	Genomica Lab Molecular, M©xico	Andersen lab at Scripps Research	Jose Horacio Reyna Verdugo; Jose Roman Chavez Mendez; Luis Alberto Rangel Gonzalez; Martin Gonzalez Ibarra; SEARCH Alliance San Diego with Jonathan Gonzalez Garcia
EPI_ISL_436715 to 436717	Genomics and Computational Biology Lab, Scientific Research Institute of Physical-Chemical Medicine, FMBA of Russia	Genomics and Computational Biology Lab, Scientific Research Institute of Physical-Chemical Medicine, FMBA of Russia	A. Manolov; A. Pavlenko; D. Fedorov; K. Klimina; O. Guskova; V. Govorun and E. Ilina; V. Veselovsky
EPI_ISL_640016, EPI_ISL_700497	George Hospital wc GRH	NHLS/UCT	Arash Iranzadeh; Bruna Galvao; Carolyn Williamson; Deelan Doolabh; Diana Hardie; Innocent Mudau; Kruger Marais; Lynn Tyers; Marvin Hsiao; Stephen Korsman
EPI_ISL_696458, EPI_ISL_696471, EPI_ISL_696473, EPI_ISL_696481, EPI_ISL_696503	George Hospital wc GRH & NHLS/UCT	KRISP, KZN Research Innovation and Sequencing Platform	Arash Iranzadeh; Bruna Galvao; Carolyn Williamson; Deelan Doolabh; Diana Hardie; Emanuel James San; Houriiyah Tegally; Innocent Mudau; Jennifer Giandhari; Kruger Marais; Lynn Tyers; Marvin Hsiao; Stephen Korsman; Sureshnee Pillay; Tulio de Oliveira
EPI_ISL_640049	George Road Sat Clinic wc GWM	NHLS/UCT	Arash Iranzadeh; Bruna Galvao; Carolyn Williamson; Deelan Doolabh; Diana Hardie; Innocent Mudau; Kruger Marais; Lynn Tyers; Marvin Hsiao; Stephen Korsman
EPI_ISL_450801, EPI_ISL_452120, EPI_ISL_452125, EPI_ISL_509662	Georgia Department of Health	Pathogen Discovery, Respiratory Viruses Branch, Division of Viral Diseases, Centers for Disease Control and Prevention	Alison S. Laufer Halpin; Anna Montmayeur; Anna Uehara; Bettina Bankamp; Christopher A. Elkins; Clinton R. Paden; Haibin Wang; Jing Zhang; Krista Queen; Mary S. Keckler; Rachel Marine; Suxiang Tong; Yan Li; Ying Tao; Zachary Weiner
EPI_ISL_594456	Georgia Public Health Laboratory	Pathogen Discovery, Respiratory Viruses Branch, Division of Viral Diseases, Centers for Disease Control and Prevention	Anna Uehara; Clinton Paden; Haibin Wang; Jing Zhang; Julu Bhatnagar; Krista Queen; Suxiang Tong; Yan Li; Ying Tao
EPI_ISL_539524, EPI_ISL_862556, EPI_ISL_862564 to 862565	Gerencia de Asistencia Sanitaria de Soria	Instituto de Salud Carlos III	A. Monzón; C. Aldea; F. Casas; I; I. Aldea, C.; I. Jiménez; Iglesias-Caballero; M. Pozo; M. Camarero; M. Cuesta; M. González-Esguevillas; M. Molinero Calamita; M. Zaballos; M.Camarero; P. Jiménez; S. Juliá; S. Molinero Calamita; S. Pozo; S. Varona
EPI_ISL_539557	Gerencia del área de salud de Badajoz, Llerena y Zafra	Instituto de Salud Carlos III	A. Monzón; C. Pazos; F. Casas; I; I. Jiménez; Iglesias-Caballero; M. Camarero; M. Cuesta; M. González-Esguevillas; M. Molinero Calamita; M. Zaballos; P. Jiménez; S. Juliá; S. Pozo; S. Varona
EPI_ISL_960679 to 960682	Germano de sousa	Instituto Gulbenkian de Ciencia	Cathy Paulino; João Costa; João Sobral; Maria Costa; Ricardo Leite; Susana Ladeiro
EPI_ISL_918484	Gerontology Institute - Skopje	Research Center for Genetic Engineering and Biotechnology "Georgi D. Efremov" , Macedonian Academy of Sciences and Arts	RCGEB - MASA
EPI_ISL_480083 to 480089	Gifu Prefectural Institute of Public Health and Environmental Sciences	Pathogen Genomics Center, National Institute of Infectious Diseases	Hajime Kamiya; Kentaro Itokawa; Makoto Kuroda; Masanori Hashino; Motoi Suzuki; Rina Tanaka; Tsuyoshi Sekizuka; Yoshihiko Kameyama
EPI_ISL_745592 to 745598, EPI_ISL_745659 to 745669, EPI_ISL_745671 to 745803, EPI_ISL_745805 to 746293, EPI_ISL_746295 to 746317	Ginkgo Bioworks Clinical Laboratory	Utah Public Health Laboratory	Alex Plocik; Becky Schilling; Birgitte Simen; David R. Hillyard; E. Susan Slechta; Erin L. Young; James McGann; Jeffrey B. Stevenson; Jim Griffin; Keith Robison; Kelly Oakeson; Malaika McKenzie-Bennett; Martha Pierson; Melanie A. Mallory; Michael T. Pyne; Michelle Spencer; Rebecca Littlefield; Salika M. Shakir; Tara Gallagher
EPI_ISL_447608 to 447613, EPI_ISL_452218 to 452223	Goethe University Hospital Frankfurt	Institute for Medical Virology, Goethe University Hospital Frankfurt	Annemarie Berger; Björn Rotter; Denisa Bojkova; Jindrich Cinatl; Klaus Hoffmeier; Sandra Westhaus; Sandra Ciesek; Sebastian Hoehl; Tuna Toptan; and Marek Widera
EPI_ISL_529146	Goethe University Hospital Frankfurt, Institute for Medical Virology	Goethe University Hospital Frankfurt, Institute for Medical Virology	Berger, A.; Bojkova, D.; Ciesek, S.; Cinatl, J.; Grabmair; Hoehl, S.; Hoffmeier, K.; Rotter, B.; T.T.; Westhaus, S.; Widera, M.
EPI_ISL_415152	Gorgas Memorial Institute for Health Studies	Gorgas Memorial Institute for Health Studies	Alexander A. Martinez.; Ambar Moreno; Claudia Gonzalez; Danilo Franco; Elimelec Valdespino; Juan M. Pascale; Leyda Abrego; Oris Chavarria; Sandra Lopez-Verges; Yamilka Diaz

EPI_ISL_524735 to 524736, EPI_ISL_586542 to 586545	Government General Hospital, Jam Khambhaliya, Devbhoomi Dwarka	Gujarat Biotechnology Research Centre	A M Kadri; Afzal Ansari; Apurvasinh Puvar; Chaitanya Joshi; Dinesh Kumar; Harish Matani; Harsh Bakshi; Janvi Raval; Komal Patel; Labdhi Pandya; Madhvi Joshi; Maharshi Pandya; Monika Gandhi; Nidhi Patel; Nikha Trivedi; Nitin Savaliya; Pinal Trivedi; R D Dixit; Raghawendra Kumar; Zarna Patel; Zuber Saiyed
EPI_ISL_586551 to 586554	Government Hospital,Veraval	Gujarat Biotechnology Research Centre	A M Kadri; Afzal Ansari; Apurvasinh Puvar; Chaitanya Joshi; Dinesh Kumar; Harsh Bakshi; Janvi Raval; Jignesh Parmar; Jitendra Bamrotia; Komal Patel; Labdhi Pandya; Madhvi Joshi; Maharshi Pandya; Monika Gandhi; Nidhi Patel; Nikha Trivedi; Nitin Savaliya; Pinal Trivedi; R D Dixit; Raghawendra Kumar; Zarna Patel; Zuber Saiyed
EPI_ISL_508207 to 508286	Government Medical College	National Institute of Biomedical Genomics	Arindam Maitra; Dhaval Khatri; Jyoti Iravane; Maitrik Dave; Saumitra Das
EPI_ISL_511930 to 511935, EPI_ISL_511939	Government Medical College	National Institute of Biomedical Genomics - DBT's PAN-INDIA 1000 SARS--CoV-2 RNA Genome Sequencing Consortium	Arindam Maitra; Dhaval Khatri; Jyoti Iravane; Maitrik Dave; Saumitra Das
EPI_ISL_495020 to 495025	Government Medical College, Bhavnagar	Gujarat Biotechnology Research Centre	A M Kadri; Afzal Ansari; Apurvasinh Puvar; Chaitanya Joshi; Dinesh Kumar; Harsh Bakshi; Janvi Raval; Kairavi Desai; Komal Patel; Labdhi Pandya; Madhvi Joshi; Maharshi Pandya; Monika Gandhi; Nidhi Patel; Nikha Trivedi; Nitin Savaliya; Pinal Trivedi; R D Dixit; Raghawendra Kumar; Saklin Malek; Shirish Patel; Zarna Patel; Zuber Saiyed
EPI_ISL_451154 to 451163, EPI_ISL_455017 to 455027, EPI_ISL_461478 to 461480, EPI_ISL_467039 to 467040, EPI_ISL_469029 to 469036, EPI_ISL_483821 to 483822	Government Medical College, Vadodara	Gujarat Biotechnology Research Centre	; A M Kadri; Afzal Ansari; Akanksha Verma; Amit Kanani; Ankit Hinsu; Apurvasinh Puvar; Armi Chaudhari; Bhavesh Modi; Bhavya Jindal; Camellia Chakraborty; Chaitanya Joshi; Dinesh Kumar; Fenil Patel; Gaurishankar Shrimali; Harsh Bakshi; Janvi Raval; Komal Patel; Labdhi Pandya; Madhvi Joshi; Maharshi Pandya; Manish Pattani; Meenakshi Shah; Monika Gandhi; Neelam Nathani; Neena Doshi; Nidhi Patel; Nikha Trivedi; Nitin Savaliya; Pinal Trivedi; Pooja P Doshi; Pragya Sharma; Pritesh Sabara; Priti Pandita; Priyanka P Vatsa; R D Dixit; R N Daveshwar; Raghawendra Kumar; Ramesh Pandit; Sharmistha Majumdar; Siddhant Kumar; Snehal Bagatharia; Tanuja Javadekar; Tejas Shah; Umang Mishra; Varsha Godbole; Zarna Patel; Zuber Saiyed
EPI_ISL_450781 to 450784	Government Medical College-Bhavnagar	Gujarat Biotechnology Research Centre	A M Kadri; Afzal Ansari; Akanksha Verma; Amit Kanani; Ankit Hinsu; Apurvasinh Puvar; Bhavesh Modi; Chaitanya Joshi; Dinesh Kumar; Gaurishankar Shrimali; Janvi Raval; Kairavi Desai; Komal Patel; Labdhi Pandya; Madhvi Joshi; Maharshi Pandya; Monika Gandhi; Neha Rajpara; Nidhi Patel; Nitin Savaliya; Pinal Trivedi; Pritesh Sabara; Priti Pandita; R D Dixit; Raghawendra Kumar; Ramesh Pandit; Saklain Malek; Shirish Patel; Snehal Bagatharia; Tejas Shah; Zarna Patel; Zuber Saiyed
EPI_ISL_983078, EPI_ISL_983083 to 983084, EPI_ISL_983086 to 983087	Gravity Diagnostics	Kentucky State Public Health Lab	Joshua Tobias; Karim George; Matthew Johnson; Rachel Zinner; Rhonda Lucas; Stephanie Lunn; Vaneet Arora; William Grooms
EPI_ISL_700543, EPI_ISL_700550	Great Brak River Clinic wc GBC	NHLS/UCT	Arash Iranzadeh; Bruna Galvao; Carolyn Williamson; Deelan Doolabh; Diana Hardie; Houriiyah Tegally; Innocent Mudau; Kruger Marais; Lynn Tyers; Marvin Hsiao; Stephen Korsman
EPI_ISL_696466, EPI_ISL_696497, EPI_ISL_696501, EPI_ISL_696507	Great Brak River Clinic wc GBC & NHLS/UCT	KRISP, KZN Research Innovation and Sequencing Platform	Arash Iranzadeh; Bruna Galvao; Carolyn Williamson; Deelan Doolabh; Diana Hardie; Emanuel James San; Houriiyah Tegally; Innocent Mudau; Jennifer Giandhari; Kruger Marais; Lynn Tyers; Marvin Hsiao; Stephen Korsman; Sureshnee Pillay; Tulio de Oliveira
EPI_ISL_794818, EPI_ISL_794820	Greek Genome Center, Biomedical Research Foundation of the Academy of Athens (BRFAA)	Greek Genome Center, Biomedical Research Foundation of the Academy of Athens (BRFAA)	Christina Maria Kravvari; Dimitrios Thanos; Emmanouil Athanasiadis; Ioannis Vatsellas; Katerina Zoi; Thodoris Loupis
EPI_ISL_640040, EPI_ISL_640043, EPI_ISL_640045, EPI_ISL_640067, EPI_ISL_640084, EPI_ISL_640111 to 640112, EPI_ISL_640122, EPI_ISL_640128 to 640135, EPI_ISL_640139, EPI_ISL_700493, EPI_ISL_700507, EPI_ISL_700534, EPI_ISL_700577, EPI_ISL_1040648, EPI_ISL_1040651, EPI_ISL_1040653 to 1040654, EPI_ISL_1040656 to 1040658, EPI_ISL_1040661, EPI_ISL_1040683, EPI_ISL_1040685 to 1040687, EPI_ISL_1040689 to 1040692, EPI_ISL_1040697 to 1040698, EPI_ISL_1040700, EPI_ISL_1040704 to 1040706, EPI_ISL_1040711, EPI_ISL_1040714, EPI_ISL_1040716, EPI_ISL_1040720, EPI_ISL_1040722 to 1040723, EPI_ISL_1040726, EPI_ISL_1040729, EPI_ISL_1040731, EPI_ISL_1040735, EPI_ISL_1040738, EPI_ISL_1040745, EPI_ISL_1040750, EPI_ISL_1040752 to 1040756, EPI_ISL_1040758 to 1040759			
see above	Groote Schuur Hospital wc GSH	NHLS/UCT	Arash Iranzadeh; Bruna Galvao; Carolyn Williamson; Deelan Doolabh; Diana Hardie; Innocent Mudau; Kruger Marais; Lynn Tyers; Marvin Hsiao; Stephen Korsman
EPI_ISL_960102 to 960103, EPI_ISL_960136	Groote Schuur Hospital wc GSH	National Health Laboratory Service/UCT	Arash Iranzadeh; Bruna Galvao; Carolyn Williamson; Deelan Doolabh; Diana Hardie; Innocent Mudau; Kruger Marais; Lynn Tyers; Marvin Hsiao; Stephen Korsman
EPI_ISL_696464	Groote Schuur Hospital wc GSH & NHLS/UCT	KRISP, KZN Research Innovation and Sequencing Platform	Arash Iranzadeh; Bruna Galvao; Carolyn Williamson; Deelan Doolabh; Diana Hardie; Emanuel James San; Houriiyah Tegally; Innocent Mudau; Jennifer Giandhari; Kruger Marais; Lynn Tyers; Marvin Hsiao; Stephen Korsman; Sureshnee Pillay; Tulio de Oliveira
EPI_ISL_698105 to 698121, EPI_ISL_698123 to 698130, EPI_ISL_698132 to 698134, EPI_ISL_698136 to 698169, EPI_ISL_698172 to 698200, EPI_ISL_698202 to 698207, EPI_ISL_698209, EPI_ISL_698213 to 699161			
see above	Group 42 (G42) Healthcare, Abu Dhabi, United Arab Emirates; Department of Health, The United Arab Emirates	G42 Healthcare	Ashish Koshy; Budoor Alqarni; Denghui Liu; Fang Chen; Hanif Khalak; Huanming Yang; Javier Quilez; Jian Wang; Junhua Li; Ke Liang; Long Lin; Mohammed Saifuddin Fasihuddin; Nan Qiao; Nawal Ahmed Mohamed Al Kaabi; Pauline Ogrodzki; Pei Wu; Peng Xiao; Pengjuan Liu; Rong Liu; Sally Mahmoud; Siyang Liu; Stephen S. Francis; Tao Ma; Vinay Kusuma; Walid Abbas Zaher; Weibin Liu; Wenjun He; Xavier Anton; Xin Jin; Xin Meng; Xinyu Huang; Xun Xu; Zhaorong Yuan
EPI_ISL_981401 to 981403, EPI_ISL_981405, EPI_ISL_981409 to 981411, EPI_ISL_981413 to 981424, EPI_ISL_981426 to 981427, EPI_ISL_981429 to 981431, EPI_ISL_981433 to 981434, EPI_ISL_981437 to 981438, EPI_ISL_981441 to 981442, EPI_ISL_981444 to 981446, EPI_ISL_981449 to 981450, EPI_ISL_981489 to 981491, EPI_ISL_981493 to 981495, EPI_ISL_981498 to 981504, EPI_ISL_981506, EPI_ISL_981508 to 981513, EPI_ISL_981517, EPI_ISL_981519, EPI_ISL_981525 to 981531, EPI_ISL_982145			
see above	Groupe LCD	Department of Virology, Henri Mondor University Hospital, Assistance Publique Hôpitaux de Paris, Université Paris-Est Créteil, INSERM U955	Alexandre Soulier; Christophe Rodriguez; Elisabeth Trawinski; Guillaume Gricourt; Jean-Michel Pawlotsky; Melissa N'Debi; Slim Fourati; Vanessa Demontant
EPI_ISL_1039003	Grubaugh Lab - Yale School of Public Health	Grubaugh Lab - Yale School of Public Health	Anderson Brito; Anne Wyllie; Annie Watkins; Chaney Kalinich; Chantal Vogels; Isabel Ott; Joseph Fauver; Mallery Breban; Mary Petrone; Nathan Grubaugh; Tara Alpert
EPI_ISL_447734 to 447736, EPI_ISL_447738 to 447748, EPI_ISL_447750, EPI_ISL_447754	Grupo de Investigaciones Microbiológicas-UR (GIMUR), Departamento de Biología, Facultad de Ciencias Naturales, Universidad del Rosario, Bogotá, Colombia	Grupo de Investigaciones Microbiológicas-UR (GIMUR), Departamento de Biología, Facultad de Ciencias Naturales, Universidad del Rosario, Bogotá, Colombia Instituto Nacional de Salud, Bogotá, Colombia Icahn School of Medicine at Mount Sinai, New York, USA	Adriana Castillo; Alberto Paniz-Mondolfi; Ana S. Gonzalez-Reiche; Angelica Rico; Aníbal A. Teherán; Carolina Florez; Carolina Hernandez; David Martínez; Emilia Mia Sordillo; Esther C. Barros; Harm van Bakel; Jesús E. Jaimes; Juan David Ramírez; Laura Vega; Lisseth Pardo; Marina Muñoz; Martha L. Ospina; Matthew M. Hernandez; Nathalia Ballesteros; Sergio Castañeda; Sergio Gomez; Viviana Simon
EPI_ISL_406531	Guangdong Provincial Center for Diseases Contorl and Prevention; Guangdong Provinical Public Health	Guangdong Provincial Center for Disease Control and Prevention	Baisheng Li; Changwen Ke; Feng Ruan; Guanhao He; Haojie Zhong; Huihong Deng; Jianfeng He; Jianpeng Xiao; Jianxiang Geng; Jianxiong Hu; Jie Wu; Jing Lu; Lifeng Lin; Lijun Liang; Lirong Zou; Min Kang; Qi Zhu; Shujiang Mei; Songjian Xiao; Tao Liu; Tie Song; Weilin Zeng; Wenjun Ma; Xing Li; Xiujuan Tang; Xue Zhuang; Xuguang Chen; Ying Wang; Yingchao Song; Yingtao Zhang; Yuhuang Liao; Zhe Liu
EPI_ISL_406533	Guangdong Provincial Center for Diseases Contorl and Prevention; Guangdong Provinical Public Health	Guangdong Provincial Center for Diseases Control and Prevention	Baisheng Li; Changwen Ke; Feng Ruan; Guanhao He; Haojie Zhong; Huihong Deng; Jianfeng He; Jianpeng Xiao; Jianxiang Geng; Jianxiong Hu; Jie Wu; Jing Lu; Lifeng Lin; Lijun Liang; Lirong Zou; Min Kang; Qi Zhu; Shujiang Mei; Songjian Xiao; Tao Liu; Tie Song; Weilin Zeng; Wenjun Ma; Xing Li; Xiujuan Tang; Xue Zhuang; Xuguang Chen; Ying Wang; Yingchao Song; Yingtao Zhang; Yuhuang Liao; Zhe Liu
EPI_ISL_403932 to 403937	Guangdong Provincial Center for Diseases Control and Prevention; Guangdong Provincial Public Health	Department of Microbiology, Guangdong Provincial Center for Diseases Control and Prevention	Baisheng Li; Changwen Ke; Feng Ruan; Guanhao He; Haojie Zhong; Huihong Deng; Jianfeng He; Jianpeng Xiao; Jianxiang Geng; Jianxiong Hu; Jie Wu; Jing Lu; Lifeng Lin; Lijun Liang; Lirong Zou; Min Kang; Qi Zhu; Shujiang Mei; Songjian Xiao; Tao Liu; Tie Song; Weilin Zeng; Wenjun Ma; Xing Li; Xiujuan Tang; Xue Zhuang; Xuguang Chen; Ying Wang; Yingchao Song; Yingtao Zhang; Yuhuang Liao; Zhe Liu
EPI_ISL_406534 to 406536	Guangdong Provincial Center for Diseases Control and Prevention; Guangdong Provincial Public Health	Guangdong Provincial Center for Diseases Control and Prevention	Baisheng Li; Changwen Ke; Feng Ruan; Guanhao He; Haojie Zhong; Huihong Deng; Jianfeng He; Jianpeng Xiao; Jianxiang Geng; Jianxiong Hu; Jie Wu; Jing Lu; Lifeng Lin; Lijun Liang; Lirong Zou; Min Kang; Qi Zhu; Shujiang Mei; Songjian Xiao; Tao Liu; Tie Song; Weilin Zeng; Wenjun Ma; Xing Li; Xiujuan Tang; Xue Zhuang; Xuguang Chen; Ying Wang; Yingchao Song; Yingtao Zhang; Yuhuang Liao; Zhe Liu
EPI_ISL_406538	Guangdong Provincial Center for Diseases Control and Prevention;Guangdong Provincial Institute of Public Health	Guangdong Provincial Center for Diseases Control and Prevention	Baisheng Li; Changwen Ke; Feng Ruan; Guanhao He; Haojie Zhong; Huihong Deng; Jianfeng He; Jianpeng Xiao; Jianxiang Geng; Jianxiong Hu; Jie Wu; Jing Lu; Lifeng Lin; Lijun Liang; Lirong Zou; Min Kang; Qi Zhu; Shujiang Mei; Songjian Xiao; Tao Liu; Tie Song; Weilin Zeng; Wenjun Ma; Xing Li; Xiujuan Tang; Xue Zhuang; Xuguang Chen; Ying Wang; Yingchao Song; Yingtao Zhang; Yuhuang Liao; Zhe Liu
EPI_ISL_428441 to 428449, EPI_ISL_428452, EPI_ISL_428454 to 428458, EPI_ISL_428460 to 428478	Guangdong Provincial Center for Diseases Control and Prevention;Guangdong Provincial Institute of Public Health	School of Public Health, The University of Hong Kong	Bosheng Li; Hanri Zeng; Haogao Gu; Hui-Ling Yen; Jie Wu; Leo L.M. Poon; Lijun Liang; Tie Song; Yao Hu; Yingchao Song; Zhencui Li
EPI_ISL_413851 to 413858, EPI_ISL_413860 to	Guangdong Provincial Institution of Public Health, Guangdong	Guangdong Provincial Institution of Public Health	Andrew Rambaut; Bo Peng; Changwen Ke; Chuming Liang; Huanying Zheng; Huifang Lin; Jing Lu; Jingju Peng; Jiufeng Sun; Josh Quick; Juan Su; Kang

413864, EPI_ISL_413866 to 413867, EPI_ISL_413875, EPI_ISL_413884	Provinical Center for Disease Control and Prevention		Min; Kuibiao Li; Lilian Zeng; Liu Zhe; Louis du Plessis; Minfeng Liang; Moritz Kraemer; Nick Loman; Nuno Faria; Oliver Pybus; Pingping Zhou; Qianlin Xiong; Ru bai; Ruilin Sun; Runyu Yuan; Sarah François; Shisong Fang; Song Tie; Tao Liu; Verity Hill; Wei Li; Wenjun Ma; Wenzhe Su; Xi Tang
EPI_ISL_444969	Guangzhou Eighth People's Hospital (Jiahe Sector)	Institute of Human Virology, Zhongshan School of Medicine, Sun Yat-sen University	Bingfeng Liu; Fang Li; Fei Yu; Feng Huang; Fengyu Hu; Hui Zhang; Huimin Fan; Jun Liu; Junsong Zhang; Kai Deng; Mang Shi; Ruosu Ying; Ting Pan; Xu Zhang; Yiwen Zhang
EPI_ISL_509695 to 509703, EPI_ISL_509710	Guatemala Ministry of Public Health	Pathogen Discovery, Respiratory Viruses Branch, Division of Viral Diseases, Centers for Disease Control and Prevention	Anna Uehara; Clinton Paden; Haibin Wang; Jing Zhang; Krista Queen; Suxiang Tong; Yan Li; Ying Tao
EPI_ISL_640104, EPI_ISL_700455, EPI_ISL_700481, EPI_ISL_700491, EPI_ISL_700503, EPI_ISL_700512, EPI_ISL_700514, EPI_ISL_700530, EPI_ISL_700576, EPI_ISL_700578, EPI_ISL_1040710, EPI_ISL_1040713, EPI_ISL_1040718, EPI_ISL_1040721, EPI_ISL_1040724, EPI_ISL_1040732, EPI_ISL_1040734			
see above	Guguletu CHC wc GDH	NHLS/UCT	Arash Iranzadeh; Bruna Galvao; Carolyn Williamson; Deelan Doolabh; Diana Hardie; Innocent Mudau; Kruger Marais; Lynn Tyers; Marvin Hsiao; Stephen Korsman
EPI_ISL_960167	Guguletu CHC wc GDH	National Health Laboratory Service/UCT	Arash Iranzadeh; Bruna Galvao; Carolyn Williamson; Deelan Doolabh; Diana Hardie; Innocent Mudau; Kruger Marais; Lynn Tyers; Marvin Hsiao; Stephen Korsman
EPI_ISL_435055 to 435056, EPI_ISL_444482 to 444486, EPI_ISL_447534 to 447546, EPI_ISL_458103 to 458113	Gujarat Biotechnology Research Centre	Gujarat Biotechnology Research Centre	; A M Kadri; Afzal Ansari; Akanksha Verma; Amit Kanani; Anjali Rajwal; Anjali Rajwar; Ankit Hinsu; Apurvasinh Puvar; Armi Chaudhari; Bhavesh Modi; Bhavya Jindal; Binita Aring; Camellia Chakraborty; Chaitanya Joshi; Dinesh Kumar; Dipa Kinariwala; Dipeshwari Shewale; Disha Patel; Gaurishankar Shrimali; Geeta Vaghela; Janvi Raval; Kairavi Joshi; Kamlesh J Upadhyay; Komal Patel; Labdhi Pandya; Madhvi Joshi; Maharshi Pandya; Monika Gandhi; Neelam Nathani; Neeta Khandelwal; Neha Rajpara; Nidhi Patel; Nidhi Sood; Nitin Savaliya; Pinal Trivedi; Pooja P Doshi; Pragya Sharma; Pranay Shah; Pritesh Sabara; Priti Pandita; Priyanka P Vatsa; R D Dixit; Raghawendra Kumar; Ramesh Pandit; Sharmistha Majumdar; Siddhant Kumar; Snehal Bagatharia; Sonia Barve; Tejas Shah; Umang Mishra; Zarna Patel; Zuber Saiyed
EPI_ISL_455573 to 455574, EPI_ISL_455580 to 455582, EPI_ISL_471157, EPI_ISL_489956 to 489958, EPI_ISL_547608 to 547610, EPI_ISL_547666 to 547676, EPI_ISL_547683, EPI_ISL_547705, EPI_ISL_660802 to 660805, EPI_ISL_660869 to 660881, EPI_ISL_660892 to 660893			
see above	Gundersen Clinical Microbiology Laboratory	Kabara Cancer Research Institute	Craig S. Richmond; Paraic A. Kenny
EPI_ISL_418186	Gundersen Molecular Diagnostic Laboratory	Kabara Cancer Research Institute	Craig S. Richmond & Paraic A. Kenny
EPI_ISL_418184, EPI_ISL_418187 to 418188, EPI_ISL_419651 to 419652, EPI_ISL_422453, EPI_ISL_422459, EPI_ISL_422461 to 422463, EPI_ISL_422465, EPI_ISL_426159 to 426161, EPI_ISL_429843 to 429848, EPI_ISL_435394 to 435395, EPI_ISL_435397 to 435402, EPI_ISL_436892 to 436900, EPI_ISL_455568 to 455572, EPI_ISL_455575 to 455576, EPI_ISL_455578, EPI_ISL_471144 to 471156, EPI_ISL_489896 to 489898, EPI_ISL_489900, EPI_ISL_489902 to 489903, EPI_ISL_489905 to 489911, EPI_ISL_489913 to 489923, EPI_ISL_489925 to 489926, EPI_ISL_489928, EPI_ISL_489930 to 489943, EPI_ISL_489945 to 489946, EPI_ISL_489948 to 489955, EPI_ISL_547602 to 547607, EPI_ISL_547611 to 547624, EPI_ISL_547626 to 547662, EPI_ISL_547664 to 547665, EPI_ISL_547678, EPI_ISL_547680 to 547682, EPI_ISL_547685, EPI_ISL_547687 to 547698, EPI_ISL_547701 to 547704, EPI_ISL_547706, EPI_ISL_547708 to 547715, EPI_ISL_547717 to 547736, EPI_ISL_547740 to 547744, EPI_ISL_547746 to 547798, EPI_ISL_547802 to 547829, EPI_ISL_547831, EPI_ISL_547833 to 547851, EPI_ISL_547853, EPI_ISL_547855 to 547869, EPI_ISL_660795 to 660801, EPI_ISL_660806 to 660868, EPI_ISL_660882 to 660891, EPI_ISL_660894 to 661173			
see above	Gundersen Molecular Diagnostics Laboratory	Kabara Cancer Research Institute	Craig S. Richmond; Craig S. Richmond & Paraic A. Kenny; Paraic A. Kenny
EPI_ISL_479850 to 479851, EPI_ISL_479853 to 479854, EPI_ISL_479896 to 479901, EPI_ISL_480015 to 480020, EPI_ISL_480169 to 480177, EPI_ISL_684220 to 684288, EPI_ISL_684325 to 684338, EPI_ISL_684566 to 684576, EPI_ISL_685279 to 685281, EPI_ISL_689047 to 689076, EPI_ISL_689686 to 689691, EPI_ISL_690210 to 690253, EPI_ISL_692047, EPI_ISL_692316 to 692327			
see above	Gunma Prefectural Institute of Public Health and Environmental Sciences	Pathogen Genomics Center, National Institute of Infectious Diseases	Hajime Kamiya; Hiroyuki Tsukagoshi; Kentaro Itokawa; Makoto Kuroda; Masanori Hashino; Motoi Suzuki; Rina Tanaka; Tsuyoshi Sekizuka
EPI_ISL_745201	Guntur General Hospital	CSIR-Centre for Cellular and Molecular Biology	Archana Bharadwaj Siva; B Himasri; Blessy B John; Divya Tej Sowpati; Dr.P.Shashikala Reddy; Dr.S.Pavani; Dr.Satyaprasad; Dr.V.Sudha Rani; Karthik Bharadwaj Tallapaka; Lamuk Zaveri; M Soujanya Reddy; Namami Gaur; Nikhil Hajirnis; Onkar Kulkarni; Payel Mukherjee; Pratheusa Maccha; Priya Singh; Purushotham Vodnala; Rakesh K Mishra; Sakshi Shambhavi; Shagufta Khan; Sofia Banu; Tulasi Nagabandi; Viswagithe S L
EPI_ISL_421454, EPI_ISL_421458, EPI_ISL_421481, EPI_ISL_421487, EPI_ISL_421491	H Beatriz Angelo	Instituto Nacional de Saude (INSA)	Guiomar et al
EPI_ISL_418003, EPI_ISL_418007 to 418008, EPI_ISL_418019 to 418022	H Braga	Instituto Nacional de Saude (INSA)	Guiomar et al
EPI_ISL_421457	H Dr Nelio Mendonca - Funchal	Instituto Nacional de Saude (INSA)	Guiomar et al
EPI_ISL_418026, EPI_ISL_421449, EPI_ISL_421459 to 421461	H Dr. Nelio Mendonca - Funchal	Instituto Nacional de Saude (INSA)	Guiomar et al
EPI_ISL_418023, EPI_ISL_421466 to 421467	H Evora	Instituto Nacional de Saude (INSA)	Guiomar et al
EPI_ISL_418018	H Garcia de Orta	Instituto Nacional de Saude (INSA)	Guiomar et al
EPI_ISL_421446 to 421448	H Guimaraes	Instituto Nacional de Saude (INSA)	Guiomar et al
EPI_ISL_418025, EPI_ISL_421462, EPI_ISL_421468 to 421471, EPI_ISL_421488, EPI_ISL_421490, EPI_ISL_421492, EPI_ISL_421495			
see above	H Santarem	Instituto Nacional de Saude (INSA)	Guiomar et al
EPI_ISL_455587	H.R.H. Maha Chakri Sirindhorn Medical Center	National Institute of Health. Department of medical Sciences, Ministry of Public Health, Thailand	Chittaganpitch; Malinee; Okada; Parnmen; Phuygun; Pilailuk; Siripaporn; Sittiporn; Sunthareeya; Thanadachakul; Thanutsapa; Waicharoen; Warawan; Wongboot
EPI_ISL_596250 to 596260, EPI_ISL_596262 to 596263, EPI_ISL_596268 to 596286, EPI_ISL_596288 to 596289, EPI_ISL_596291 to 596317, EPI_ISL_596357 to 596377	HELIX LCC	WHO National Influenza Centre Russian Federation	Andrey Komissarov; Anna Ivanova; Artem Fadeev; Daria Danilenko; Dmitry Bazhenov; Kseniya Komissarova
EPI_ISL_602329 to 602411, EPI_ISL_602417 to 602461, EPI_ISL_639949, EPI_ISL_639951 to 639959, EPI_ISL_639961, EPI_ISL_639963 to 639964, EPI_ISL_639966 to 639973, EPI_ISL_733024 to 733025, EPI_ISL_733027 to 733028, EPI_ISL_733030, EPI_ISL_733032 to 733034, EPI_ISL_733037, EPI_ISL_733039, EPI_ISL_733041, EPI_ISL_733044, EPI_ISL_733053, EPI_ISL_733055, EPI_ISL_733059 to 733062, EPI_ISL_733065 to 733067, EPI_ISL_733069, EPI_ISL_733071, EPI_ISL_733073 to 733075, EPI_ISL_733077, EPI_ISL_733079 to 733080, EPI_ISL_733082, EPI_ISL_733086 to 733090, EPI_ISL_733092 to 733093, EPI_ISL_733095 to 733097, EPI_ISL_733100, EPI_ISL_733102 to 733103, EPI_ISL_733105 to 733106, EPI_ISL_733113, EPI_ISL_733117, EPI_ISL_733121, EPI_ISL_733126, EPI_ISL_733128 to 733129, EPI_ISL_733132, EPI_ISL_733135, EPI_ISL_733137 to 733138, EPI_ISL_733140, EPI_ISL_733142, EPI_ISL_733144 to 733148, EPI_ISL_733151, EPI_ISL_733153, EPI_ISL_733303 to 733305, EPI_ISL_733308 to 733310, EPI_ISL_733318, EPI_ISL_733321, EPI_ISL_733323 to 733325, EPI_ISL_733331 to 733334, EPI_ISL_733337, EPI_ISL_733341 to 733345, EPI_ISL_733350 to 733351, EPI_ISL_733353 to 733355, EPI_ISL_733361, EPI_ISL_733366, EPI_ISL_733368 to 733369, EPI_ISL_733371, EPI_ISL_733374, EPI_ISL_733376, EPI_ISL_733378 to 733379, EPI_ISL_733381 to 733383, EPI_ISL_733387, EPI_ISL_733393, EPI_ISL_733396, EPI_ISL_733424, EPI_ISL_733436, EPI_ISL_733439, EPI_ISL_733455 to 733456			
see above	HELIX LLC	WHO National Influenza Centre Russian Federation	Andrey Komissarov; Anna Ivanova; Artem Fadeev; Daria Danilenko; Dmitry Bazhenov; Dmitry Lioznov; Elena Nabieva; Georgii Bazykin; Ksenia Safina; Kseniya Komissarova
EPI_ISL_751578, EPI_ISL_751603, EPI_ISL_751651, EPI_ISL_751673 to 751675, EPI_ISL_751758, EPI_ISL_751761, EPI_ISL_751767, EPI_ISL_751772			
see above	HI Dept. of Health, State Laboratories Division	Genomics and Discovery, Respiratory Viruses Branch, Division of Viral Diseases, Centers for Disease Control and Prevention	Anna Montmayeur; Anna Uehara; Clinton R. Paden; Haibin Wang; Jing Zhang; Justin Lee; Krista Queen; Mili Sheth; Peter W. Cook; Rachel Marine; Suxiang Tong; Yan Li; Ying Tao
EPI_ISL_426420 to 426421	HI Dept. of Health, State Laboratories Division	Pathogen Discovery, Respiratory Viruses Branch, Division of Viral Diseases, Centers for Disease Control and Prevention	Alison S. Laufer Halpin; Anna Uehara; Christopher A. Elkins; Clinton R. Paden; Haibin Wang; Jing Zhang; Krista Queen; Mary S. Keckler; Rachel Marine; Suxiang Tong; Yan Li; Ying Tao
EPI_ISL_860849	HIA BÉGIN Service de Biologie	National Reference Center for Viruses of Respiratory Infections, Institut Pasteur, Paris	Angela Brisebarre; Camille Capel; Etienne Simon-Lorière; Marion Barbet; Maud Vanpeene; Merens Audrey; Méline Bizard; Sylvie Behillil; Sylvie van der Werf; Vincent Enouf
EPI_ISL_941270	HIA PERCY - lab biologie	National Reference Center for Viruses of Respiratory Infections, Institut Pasteur, Paris	Angela Brisebarre; Camille Capel; Etienne Simon-Lorière; Marion Barbet; Maud Vanpeene; Méline Bizard; Sylvie Behillil; Sylvie van der Werf; Vincent Enouf; Vincent Foissaud
EPI_ISL_860850 to 860851	HIA PERCY, Biologie médicale	National Reference Center for Viruses of Respiratory Infections, Institut Pasteur, Paris	Angela Brisebarre; Camille Capel; Etienne Simon-Lorière; Foissaud Vincent; Marion Barbet; Maud Vanpeene; Méline Bizard; Sylvie Behillil; Sylvie van der Werf; Vincent Enouf
EPI_ISL_1007590, EPI_ISL_1007641	HIB	Laboratory of genomics and metagenomics, Institute of	Claire Bertelli; Damien Jacot; Gilbert Greub; Sébastien Aeby; Trestan Pillonel

		Microbiology, University Hospital Centre and University of Lausanne, Switzerland	
EPI_ISL_912560 to 912562	HOPITAL SIMONE VEIL	National Reference Center for Viruses of Respiratory Infections, Institut Pasteur, Paris	Angela Brisebarre; Camille Capel; Etienne Simon-Lorière; Marion Barbet; Maud Vanpeene; Moreau Farida; Méline Bizard; Sylvie Behillil; Sylvie van der Werf; Vincent Enouf
EPI_ISL_445323	HOSP.ENFERMEDADES INFECCIOSAS	Instituto de Salud Publica de Chile	Alejandra Acevedo; Andrés E Castillo; Bárbara Parra; Carolina Tambley; Gabriel Leal; Jaime Lagos; Jorge Fernandez; Loredana Arata; Patricia Bustos; Paz Tapia; Rodrigo Fasce; Winston Andrade
EPI_ISL_682239	HOSPITAL CIUDAD NEILY	Inciensa, Instituto Costarricense de Investigación y Enseñanza en Nutrición y Salud	Adriana Godinez & Melany Calderon; Claudio Soto-Garita; Estela Cordero; Francisco Duarte; Hebleen Porras
EPI_ISL_418250, EPI_ISL_419707	HOSPITAL CLINIC	Instituto de Salud Carlos III	A. Monzón; F. Casas; I. Jiménez; I. Marcos; I. Marcos M.A; Iglesias-Caballero; M. Camarero; M. Camarero S. Pozo F. Casas I. Jiménez; M. Cuesta; M. González-Esguevillas; M. Molinero Calamita; M. Zaballos; M.A; P. Jiménez; S. Juliá; S. Pozo; S. Varona
EPI_ISL_445281	HOSPITAL CLINICO DEL SUR	Instituto de Salud Publica de Chile	Alejandra Acevedo; Andrés E Castillo; Bárbara Parra; Carolina Tambley; Gabriel Leal; Jaime Lagos; Jorge Fernandez; Loredana Arata; Patricia Bustos; Paz Tapia; Rodrigo Fasce; Winston Andrade
EPI_ISL_445275	HOSPITAL CLINICO FUSAT	Instituto de Salud Publica de Chile	Alejandra Acevedo; Andrés E Castillo; Bárbara Parra; Carolina Tambley; Gabriel Leal; Jaime Lagos; Jorge Fernandez; Loredana Arata; Patricia Bustos; Paz Tapia; Rodrigo Fasce; Winston Andrade
EPI_ISL_954792, EPI_ISL_954796	HOSPITAL COMARCAL DE MELILLA	Instituto de Salud Carlos III	A. Monzón; F. Casas; I. Illescas, S.; I. Jiménez; I. Roman, S.; Iglesias-Caballero; M. Camarero; P. Zaballos; S. Cuesta; S. Pozo; S. Sandonís; S. Varona; V. Vázquez
EPI_ISL_445378	HOSPITAL DE BULNES	Instituto de Salud Publica de Chile	Alejandra Acevedo; Andrés E Castillo; Bárbara Parra; Carolina Tambley; Gabriel Leal; Jaime Lagos; Jorge Fernandez; Loredana Arata; Patricia Bustos; Paz Tapia; Rodrigo Fasce; Winston Andrade
EPI_ISL_445335	HOSPITAL DE CALBUCO	Instituto de Salud Publica de Chile	Alejandra Acevedo; Andrés E Castillo; Bárbara Parra; Carolina Tambley; Gabriel Leal; Jaime Lagos; Jorge Fernandez; Loredana Arata; Patricia Bustos; Paz Tapia; Rodrigo Fasce; Winston Andrade
EPI_ISL_445251, EPI_ISL_445305	HOSPITAL DE CARABINEROS	Instituto de Salud Publica de Chile	Alejandra Acevedo; Andrés E Castillo; Bárbara Parra; Carolina Tambley; Gabriel Leal; Jaime Lagos; Jorge Fernandez; Loredana Arata; Patricia Bustos; Paz Tapia; Rodrigo Fasce; Winston Andrade
EPI_ISL_419238	HOSPITAL DE CRUCES.	Instituto de Salud Carlos III	A. Monzón; F. Casas; I. Aranzamendi, M.; I. Jiménez; Iglesias-Caballero; M. Camarero; M. Cuesta; M. González-Esguevillas; M. Molinero Calamita; M. Zaballos; P. Jiménez; S. Juliá; S. Pozo; S. Varona
EPI_ISL_445300	HOSPITAL DE RANCAGUA	Instituto de Salud Publica de Chile	Alejandra Acevedo; Andrés E Castillo; Bárbara Parra; Carolina Tambley; Gabriel Leal; Jaime Lagos; Jorge Fernandez; Loredana Arata; Patricia Bustos; Paz Tapia; Rodrigo Fasce; Winston Andrade
EPI_ISL_445352, EPI_ISL_445360, EPI_ISL_445368	HOSPITAL DEL PROFESOR	Instituto de Salud Publica de Chile	Alejandra Acevedo; Andrés E Castillo; Bárbara Parra; Carolina Tambley; Gabriel Leal; Jaime Lagos; Jorge Fernandez; Loredana Arata; Patricia Bustos; Paz Tapia; Rodrigo Fasce; Winston Andrade
EPI_ISL_445270, EPI_ISL_445340, EPI_ISL_445342 to 445348, EPI_ISL_447119	HOSPITAL DR.HERNAN HENRIQUEZ ARAVENA	Instituto de Salud Publica de Chile	Alejandra Acevedo; Andrés E Castillo; Bárbara Parra; Carolina Tambley; Gabriel Leal; Jaime Lagos; Jorge Fernandez; Loredana Arata; Patricia Bustos; Paz Tapia; Rodrigo Fasce; Winston Andrade
EPI_ISL_445310, EPI_ISL_445329, EPI_ISL_445365 to 445366, EPI_ISL_445371	HOSPITAL DR.SOTERO DEL RIO	Instituto de Salud Publica de Chile	Alejandra Acevedo; Andrés E Castillo; Bárbara Parra; Carolina Tambley; Gabriel Leal; Jaime Lagos; Jorge Fernandez; Loredana Arata; Patricia Bustos; Paz Tapia; Rodrigo Fasce; Winston Andrade
EPI_ISL_445313 to 445314, EPI_ISL_445364	HOSPITAL EL CARMEN DR.LUIS VALENTIN F.	Instituto de Salud Publica de Chile	Alejandra Acevedo; Andrés E Castillo; Bárbara Parra; Carolina Tambley; Gabriel Leal; Jaime Lagos; Jorge Fernandez; Loredana Arata; Patricia Bustos; Paz Tapia; Rodrigo Fasce; Winston Andrade
EPI_ISL_445372	HOSPITAL FF.AA. "CIRUJANO C. GUZMAN	Instituto de Salud Publica de Chile	Alejandra Acevedo; Andrés E Castillo; Bárbara Parra; Carolina Tambley; Gabriel Leal; Jaime Lagos; Jorge Fernandez; Loredana Arata; Patricia Bustos; Paz Tapia; Rodrigo Fasce; Winston Andrade
EPI_ISL_418247	HOSPITAL GENERAL DE SEGOVIA	Instituto de Salud Carlos III	A. Monzón; F. Casas; I. Hernando-Real S.; I. Jiménez; Iglesias-Caballero; M. Camarero; M. Cuesta; M. González-Esguevillas; M. Molinero Calamita; M. Zaballos; P. Jiménez; S. Juliá; S. Pozo; S. Varona
EPI_ISL_954795	HOSPITAL GENERAL RIO CARRION	Instituto de Salud Carlos III	A. Monzón; F. Casas; I. García, A.; I. Jiménez; Iglesias-Caballero; M. Camarero; P. Zaballos; S. Cuesta; S. Pozo; S. Sandonís; S. Varona; V. Vázquez
EPI_ISL_445286	HOSPITAL HANGA ROA	Instituto de Salud Publica de Chile	Alejandra Acevedo; Andrés E Castillo; Bárbara Parra; Carolina Tambley; Gabriel Leal; Jaime Lagos; Jorge Fernandez; Loredana Arata; Patricia Bustos; Paz Tapia; Rodrigo Fasce; Winston Andrade
EPI_ISL_445331 to 445332	HOSPITAL HERMINDA MARTIN CHILLAN	Instituto de Salud Publica de Chile	Alejandra Acevedo; Andrés E Castillo; Bárbara Parra; Carolina Tambley; Gabriel Leal; Jaime Lagos; Jorge Fernandez; Loredana Arata; Patricia Bustos; Paz Tapia; Rodrigo Fasce; Winston Andrade
EPI_ISL_445289	HOSPITAL NAVAL PUERTO WILLIAMS	Instituto de Salud Publica de Chile	Alejandra Acevedo; Andrés E Castillo; Bárbara Parra; Carolina Tambley; Gabriel Leal; Jaime Lagos; Jorge Fernandez; Loredana Arata; Patricia Bustos; Paz Tapia; Rodrigo Fasce; Winston Andrade
EPI_ISL_445327, EPI_ISL_445353	HOSPITAL PADRE HURTADO	Instituto de Salud Publica de Chile	Alejandra Acevedo; Andrés E Castillo; Bárbara Parra; Carolina Tambley; Gabriel Leal; Jaime Lagos; Jorge Fernandez; Loredana Arata; Patricia Bustos; Paz Tapia; Rodrigo Fasce; Winston Andrade
EPI_ISL_445268 to 445269, EPI_ISL_445280, EPI_ISL_445284, EPI_ISL_445288, EPI_ISL_445293 to 445294	HOSPITAL REG.LAUTARO NAVARRO AVARIA	Instituto de Salud Publica de Chile	Alejandra Acevedo; Andrés E Castillo; Bárbara Parra; Carolina Tambley; Gabriel Leal; Jaime Lagos; Jorge Fernandez; Loredana Arata; Patricia Bustos; Paz Tapia; Rodrigo Fasce; Winston Andrade
EPI_ISL_593773 to 593775	HOSPITAL REGIONAL LAMBAYEQUE	GENOMA MAYOR	Franklin R. Aguilar-Gamboa; Heber Silva-Díaz; Juan J. Bonifacio-Briceño; Luis M. López-Serquén; Luis Salcedo-Mejía; Marco E. Mechán-Llontop and Juan P. Cárdenas.; Percy O. Tullume-Vergara; Ramsés Salas-Asencios
EPI_ISL_445307	HOSPITAL SAN JOSE DE MAIPO	Instituto de Salud Publica de Chile	Alejandra Acevedo; Andrés E Castillo; Bárbara Parra; Carolina Tambley; Gabriel Leal; Jaime Lagos; Jorge Fernandez; Loredana Arata; Patricia Bustos; Paz Tapia; Rodrigo Fasce; Winston Andrade
EPI_ISL_682241, EPI_ISL_682243, EPI_ISL_682245 to 682246, EPI_ISL_682253 to 682254, EPI_ISL_682257, EPI_ISL_682262 to 682264, EPI_ISL_682266 to 682273			
see above	HOSPITAL SAN JUAN DE DIOS	Inciensa, Instituto Costarricense de Investigación y Enseñanza en Nutrición y Salud	Adriana Godinez & Melany Calderon; Claudio Soto-Garita; Estela Cordero; Francisco Duarte; Hebleen Porras
EPI_ISL_445304, EPI_ISL_445349 to 445351, EPI_ISL_445373, EPI_ISL_445375 to 445377	HOSPITAL SAN JUAN DE DIOS	Instituto de Salud Publica de Chile	Alejandra Acevedo; Andrés E Castillo; Bárbara Parra; Carolina Tambley; Gabriel Leal; Jaime Lagos; Jorge Fernandez; Loredana Arata; Patricia Bustos; Paz Tapia; Rodrigo Fasce; Winston Andrade
EPI_ISL_682242, EPI_ISL_682255	HOSPITAL SAN VICENTE DE PAUL	Inciensa, Instituto Costarricense de Investigación y Enseñanza en Nutrición y Salud	Adriana Godinez; Adriana Godinez & Melany Calderon; Claudio Soto-Garita; Estela Cordero; Francisco Duarte; Hebleen Porras; Melany Calderon & Mariel López
EPI_ISL_417007	HOSPITAL SANTA MARIA NAI	Instituto de Salud Carlos III	A. Monzón; I. García Costa, J.; Iglesias-Caballero; M. Camarero S. Pozo F. Casas I. Jiménez; M. Cuesta; M. González-Esguevillas; M. Molinero Calamita; M. Zaballos; P. Jiménez; S. Juliá; S. Varona
EPI_ISL_418253, EPI_ISL_419240, EPI_ISL_419709	HOSPITAL TXAGORRITXU	Instituto de Salud Carlos III	A. Monzón; C; F. Casas; I. Gomez-Gonzalez C.; I. Gómez; I. Gómez, C.; I. Jiménez; Iglesias-Caballero; M. Camarero; M. Camarero S. Pozo F. Casas I. Jiménez; M. Cuesta; M. González-Esguevillas; M. Molinero Calamita; M. Zaballos; P. Jiménez; S. Juliá; S. Pozo; S. Varona
EPI_ISL_418251	HOSPITAL UNIVERSITARIO LA PAZ	Instituto de Salud Carlos III	A. Monzón; F. Casas; I. Jiménez; I. Romero P.; Iglesias-Caballero; M. Camarero; M. Cuesta; M. González-Esguevillas; M. Molinero Calamita; M. Zaballos; P. Jiménez; S. Juliá; S. Pozo; S. Varona
EPI_ISL_418243 to 418244	HOSPITAL UNIVERSITARIO VIRGEN DE LAS NIEVES	Instituto de Salud Carlos III	A. Monzón; F. Casas; I. Jiménez; I. Sanbonmatsu S.; Iglesias-Caballero; M. Camarero; M. Cuesta; M. González-Esguevillas; M. Molinero Calamita; M. Zaballos; P. Jiménez; S. Juliá; S. Pozo; S. Varona
EPI_ISL_682265	HOSPITAL UPALA	Inciensa, Instituto Costarricense de Investigación y	Adriana Godinez & Melany Calderon; Claudio Soto-Garita; Estela Cordero; Francisco Duarte; Hebleen Porras

		Enseñanza en Nutrición y Salud	
EPI_ISL_636977	HP Pemba	KRISP, KZN Research Innovation and Sequencing Platform	Giandhari J; Ismael N; Nadia Sitoe; Nedio Mabunda; Paulo Arnaldo; Pillay S; Tegally H; Wilkinson E; de Oliveira T
EPI_ISL_430847	HS mikrobiologi virus	The Public Health Agency of Sweden	Anna Risberg; Anna-Malin Linde; Karin Tegmark-Wisell; Maria Lind Karlberg; Olov Svartstrom; Oskar Karlsson Lindsjo; Shaman Muradrasoli; Zhibing Yun
EPI_ISL_418009, EPI_ISL_421463, EPI_ISL_421489, EPI_ISL_421494	HSE Ilha Terceira - Angra do Heroismo	Instituto Nacional de Saude (INSA)	Guiomar et al
EPI_ISL_648151	Halmstad	The Public Health Agency of Sweden	Anna Risberg; Anna-Malin Linde; Karin Tegmark-Wisell; Maria Lind Karlberg; Mattias Haukland; Mia Brytting; Olov Svartstrom; Oskar Karlsson Lindsjo; Petra Edquist; Reza Advani; Sandra Broddesson
EPI_ISL_454436 to 454446, EPI_ISL_455902, EPI_ISL_469073 to 469074, EPI_ISL_475093 to 475094, EPI_ISL_475096 to 475098, EPI_ISL_475100, EPI_ISL_475114, EPI_ISL_475116 to 475117, EPI_ISL_475120 to 475121, EPI_ISL_475123, EPI_ISL_475125, EPI_ISL_475156 to 475159, EPI_ISL_475164, EPI_ISL_475548, EPI_ISL_475553, EPI_ISL_475556 to 475557, EPI_ISL_510831, EPI_ISL_510866, EPI_ISL_548247, EPI_ISL_548249, EPI_ISL_582803 to 582805, EPI_ISL_594148, EPI_ISL_615104 to 615107, EPI_ISL_623086, EPI_ISL_648148			
see above	Halmstad klinisk mikrobiologi	The Public Health Agency of Sweden	Anna Risberg; Anna-Malin Linde; Karin Tegmark-Wisell; Maria Lind Karlberg; Mattias Haukland; Mia Brytting; Olov Svartstrom; Oskar Karlsson Lindsjo; Petra Edquist; Reza Advani; Sandra Broddesson; Shamam Muradrasoli; Shaman Muradrasoli
EPI_ISL_710606 to 710607	Halsomedicinskt Center	The Public Health Agency of Sweden	Department of Microbiology; The Public Health Agency of Sweden
EPI_ISL_660139, EPI_ISL_660141	Hamadi	National Health Laboratory Service (NHLS), Tygerberg	Bronwyn Kleinhans; Davis M-A; Draper C; Eduan Wilkindon; Gert van Zyl; Houriiyah Tegally; Hsiao M; Kayla Delaney; Siegfried N; Susan Engelbrecht; Tulio de Oliveira; Williamson C; Wolfgang Preiser
EPI_ISL_407313	Hangzhou Center for Disease Control and Prevention	Hangzhou Center for Disease Control and Prevention	Haoqiu Wang; Hua Yu; Jun Li; Lingfeng Mao; Qingxin Kong; Shuchang Chen; Xin Qian; Xinfen Yu; Xuchu Wang; Zhou Sun
EPI_ISL_418510	Hangzhou Center for Disease Control and Prevention	Insepction Center of Hangzhou Center for Disease Control and Prevention	Li jun; Pan jingcao; Wang haoqiu; Yu hua; Yu xinfeng
EPI_ISL_418506	Hangzhou Center for Disease Control and Prevention	Inspection Center of Hanghzou Center for Disease Control and Prevention	Li jun; Pan jingcao; Wang haoqiu; Yu hua; Yu xinfeng
EPI_ISL_418441 to 418442, EPI_ISL_418502 to 418504, EPI_ISL_418507 to 418509, EPI_ISL_418511 to 418515	Hangzhou Center for Disease Control and Prevention	Inspection Center of Hangzhou Center for Disease Control and Prevention	Li jun; Pan jingcao; Wang haoqiu; Yu hua; Yu xinfeng
EPI_ISL_406970	Hangzhou Center for Disease and Control Microbiology Lab	Hangzhou Center for Disease and Control Microbiology Lab	Li Jun; Wang Haoqiu; Yu Hua; Yu Xinfeng
EPI_ISL_421221 to 421222, EPI_ISL_421224 to 421236, EPI_ISL_482575 to 482586	Hangzhou Center for Diseases Control and Prevention	Hangzhou Center for Diseases Control and Prevention	Haoqiu Wang; Hua Yu; Jun Li; Junfang Chen; Lingfeng Mao; Shuchang Chen; Xin Qian; Xinfen Yu; Xuchu Wang; Zhou Sun
EPI_ISL_700457, EPI_ISL_700463, EPI_ISL_700477, EPI_ISL_700480, EPI_ISL_700494, EPI_ISL_700515 to 700516, EPI_ISL_700561 to 700562, EPI_ISL_700566, EPI_ISL_700573, EPI_ISL_700585, EPI_ISL_700593, EPI_ISL_1040694 to 1040695, EPI_ISL_1040717, EPI_ISL_1040764, EPI_ISL_1040809			
see above	Hanover Park CHC wc HPH	NHLS/UCT	Arash Iranzadeh; Bruna Galvao; Carolyn Williamson; Deelan Doolabh; Diana Hardie; Innocent Mudau; Kruger Marais; Lynn Tyers; Marvin Hsiao; Stephen Korsman
EPI_ISL_413025	Harborview Medical Center	UW Virology Lab	Alexander Greninger; Arun Nalla; Hong Xie; Keith Jerome; Pavitra Roychoudhury
EPI_ISL_413487	Harborview Medical Center	University of Washington Virology Lab	Alexander Greninger; Arun Nalla; Hong Xie; Keith Jerome; Pavitra Roychoudhury
EPI_ISL_635081, EPI_ISL_635133, EPI_ISL_635184	Haukeland University Hospital, Department of Medical Microbiology	Norwegian Institute of Public Health, Department of Virology	Hilde Elshaug; Hilde Vollan; Kamilla Heddeland Instefjord; Karoline Bragstad; Kathrine Stene-Johansen; Marie Paulsen Madsen; Olav Hungnes; Rasmus Riis Kopperud
EPI_ISL_635128, EPI_ISL_668405, EPI_ISL_759975, EPI_ISL_860208, EPI_ISL_860211, EPI_ISL_860278, EPI_ISL_964951, EPI_ISL_1034310			
see above	Haukeland University Hospital, Dept. of Microbiology	Norwegian Institute of Public Health, Department of Virology	Atiya R Ali; Hilde Elshaug; Hilde Vollan; Ignacio Garcia Llorente; Kamilla Heddeland Instefjord; Karoline Bragstad; Kathrine Stene-Johansen; Marie Paulsen Madsen; Olav Hungnes; Rasmus Riis Kopperud; Serina B Engebretsen
EPI_ISL_450498	Health Board Laboratory of Communicable Diseases	Charite Universitatsmedizin Berlin, Institute of Virology	Barbara Muhlemann; Christian Drosten; Julia Schneider; Jörn Beheim-Schwarzbach; Liidia Dotsenko; Natalja Kuznetsova; Talitha Veith; Terry Jones; Victor M Corman
EPI_ISL_420065 to 420067	Health Board Laboratory of Communicable Diseases	Charite Universitatsmedizin Berlin, Institute of Virology	Barbara Mühlemann; Christian Drosten; Jorn Beheim-Schwarzbach; Julia Schneider; Liidia Dotsenko; Natalja Kuznetsova; Talitha Veith; Terry Jones; Victor M Corman
EPI_ISL_429256	Health Sciences Technology Park, Avicena, 8, 18016 Granada. Spain	Sequencing and Bioinformatics Service FISABIO-Public Health	Almudena Rojas; Joaquín Mendoza; Pablo Mendoza
EPI_ISL_540993 to 541007	Health and Environmental Research Institute of Gwangju Metropolitan city	Health and Environmental Research Institute of Gwangju Metropolitan city	Ji-eun Lee; Min Ji Kim
EPI_ISL_634864 to 634865	HealthPartners Central Lab	Minnesota Department of Health, Public Health Laboratory	Alexandra Lorentz; Jacob Garfin; Matt Plumb; and Xiong Wang
EPI_ISL_640018	Heidelberg Clinic wc HBC	NHLS/UCT	Arash Iranzadeh; Bruna Galvao; Carolyn Williamson; Deelan Doolabh; Diana Hardie; Innocent Mudau; Kruger Marais; Lynn Tyers; Marvin Hsiao; Stephen Korsman
EPI_ISL_700454, EPI_ISL_700459, EPI_ISL_700473, EPI_ISL_700499, EPI_ISL_700508, EPI_ISL_700522, EPI_ISL_700549, EPI_ISL_1040719			
see above	Heideveld CDC wc HVP	NHLS/UCT	Arash Iranzadeh; Bruna Galvao; Carolyn Williamson; Deelan Doolabh; Diana Hardie; Innocent Mudau; Kruger Marais; Lynn Tyers; Marvin Hsiao; Stephen Korsman
EPI_ISL_960173	Heideveld CDC wc HVP	National Health Laboratory Service/UCT	Arash Iranzadeh; Bruna Galvao; Carolyn Williamson; Deelan Doolabh; Diana Hardie; Innocent Mudau; Kruger Marais; Lynn Tyers; Marvin Hsiao; Stephen Korsman
EPI_ISL_640082, EPI_ISL_700445, EPI_ISL_700545, EPI_ISL_700559, EPI_ISL_700574, EPI_ISL_700584, EPI_ISL_700595, EPI_ISL_700598, EPI_ISL_1040662, EPI_ISL_1040688, EPI_ISL_1040757			
see above	Heideveld Emergency Centre	NHLS/UCT	Arash Iranzadeh; Bruna Galvao; Carolyn Williamson; Deelan Doolabh; Diana Hardie; Houriiyah Tegally; Innocent Mudau; Kruger Marais; Lynn Tyers; Marvin Hsiao; Stephen Korsman
EPI_ISL_850690 to 850691, EPI_ISL_850695 to 850700, EPI_ISL_850703, EPI_ISL_850717, EPI_ISL_850734 to 850735, EPI_ISL_850754, EPI_ISL_850764, EPI_ISL_850786 to 850787, EPI_ISL_850790 to 850791, EPI_ISL_850793 to 850795, EPI_ISL_850797, EPI_ISL_850799 to 850806, EPI_ISL_850816, EPI_ISL_850832, EPI_ISL_850854, EPI_ISL_850875, EPI_ISL_850883, EPI_ISL_850889, EPI_ISL_850896, EPI_ISL_850904, EPI_ISL_850939			
see above	Helix / Illumina	Genomics and Discovery, Respiratory Viruses Branch, Division of Viral Diseases, Centers for Disease Control and Prevention	Alexandre Bolze; Ary Ascencio; Ben L. Rambo-Martin Eileen de Feo; Brad Sickler; Charlotte Rivera-Garcia; Christine Tran; Clinton R. Paden; David Becker; Dhwani Batra; Duncan MacCannell; Efren Sandoval; Elizabeth Cirulli; Eric Allen; Geraint Levan; James Lu; Jan Antico; Jason Nguyen; Jimmy Ramirez; Jingtao Liu; Kelly Schiabor Barrett; Kim Gietzen; Magnus Isaksson; Marc Laurent; Matthew Tolentino; Nicole L. Washington; Peter W. Cook; Phil Febbo; Ryan Cho; Shannon Wickline; Sherry Wang; Simon White; Summer Galloway; Suxiang Tong; Tyler Cassens; William Lee
EPI_ISL_755590 to 755595, EPI_ISL_755597 to 755607, EPI_ISL_755609 to 755611, EPI_ISL_755616 to 755617, EPI_ISL_802606 to 802650, EPI_ISL_850521, EPI_ISL_850523 to 850525, EPI_ISL_850527, EPI_ISL_850581, EPI_ISL_850587, EPI_ISL_850602, EPI_ISL_850607, EPI_ISL_850610, EPI_ISL_850612, EPI_ISL_850614, EPI_ISL_850617 to 850618, EPI_ISL_850620, EPI_ISL_850628, EPI_ISL_850631 to 850636, EPI_ISL_850638, EPI_ISL_850641, EPI_ISL_850645, EPI_ISL_850648 to 850649, EPI_ISL_850651, EPI_ISL_850958, EPI_ISL_850960, EPI_ISL_850978, EPI_ISL_851021 to 851025, EPI_ISL_876634 to 876638			
see above	Helix/Illumina	Genomics and Discovery, Respiratory Viruses Branch, Division of Viral Diseases, Centers for Disease Control and Prevention	; Alexandre Bolze; Ary Ascencio; Ben L. Rambo-Martin; Ben L. Rambo-Martin Eileen de Feo; Brad Sickler; Charlotte Rivera-Garcia; Christine Tran; Clinton R. Paden; David Becker; Dhwani Batra; Duncan MacCannell; Efren Sandoval; Eileen de Feo; Elizabeth Cirulli; Eric Allen; Geraint Levan; James Lu; Jan Antico; Jason Nguyen; Jimmy Ramirez; Jingtao Liu; Kelly Schiabor Barrett; Kim Gietzen; Magnus Isaksson; Marc Laurent; Matthew Tolentino; Nicole L. Washington; Peter W. Cook; Phil Febbo; Ryan Cho; Shannon Wickline; Sherry Wang; Simon White; Summer Galloway; Suxiang Tong; Tyler Cassens; William Lee
EPI_ISL_966497 to 966512, EPI_ISL_966514 to 966532, EPI_ISL_966534 to 966538, EPI_ISL_966540 to 966548, EPI_ISL_966560 to 966564, EPI_ISL_966566 to 966590, EPI_ISL_966592 to 966601, EPI_ISL_1016693, EPI_ISL_1016696 to 1016786, EPI_ISL_1016789 to 1016820, EPI_ISL_1016824 to 1016832, EPI_ISL_1016834 to 1016841, EPI_ISL_1036688 to 1036725			

see above	Helix/Illumina	Respiratory Viruses Branch, Division of Viral Diseases, Centers for Disease Control and Prevention	; Alexandre Bolze; Ary Ascencio; Ben L. Rambo-Martin; Brad Sickler; Charlotte Rivera-Garcia; Christine Tran; Clinton R. Paden; Dakota Howard; David Becker; Dhwani Batra; Duncan MacCannell; Efren Sandoval; Eileen de Feo; Elizabeth Cirulli; Eric Allen; Geraint Levan; James Lu; Jan Antico; Jason Nguyen; Jimmy Ramirez; Jingtao Liu; Kelly Schiabor Barrett; Kim Gietzen; Magnus Isaksson; Marc Laurent; Matthew Tolentino; Nicole L. Washington; Peter W. Cook; Phil Febbo; Ryan Cho; Shannon Wickline; Sherry Wang; Simon White; Summer Galloway; Suxiang Tong; Tyler Cassens; William Lee
EPI_ISL_451310 to 451311, EPI_ISL_487377, EPI_ISL_487381, EPI_ISL_501233, EPI_ISL_501236, EPI_ISL_501248 to 501251	Hellenic Pasteur Institute, National Influenza Reference laboratory of Southern Greece & Unit of Bioinformatics and Applied Genomics	Hellenic Pasteur Institute, National Influenza Reference laboratory of Southern Greece & Unit of Bioinformatics and Applied Genomics	Andreas Mentis; Androniki Voulgari-Kokota; Antonios Kalliaropoulos; Aspasia Kontou; Athanasios Kossyvakis; Evangelidou Maria; Horefti Elina; Timokratis Karamitros; Vasiliki Pogka
EPI_ISL_501230	Hellenic Pasteur Institute, Public Health Laboratories	Hellenic Pasteur Institute, National Influenza Reference laboratory of Southern Greece & Unit of Bioinformatics and Applied Genomics	Andreas Mentis; Androniki Voulgari-Kokota; Antonios Kalliaropoulos; Aspasia Kontou; Athanasios Kossyvakis; Evangelidou Maria; Horefti Elina; Timokratis Karamitros; Vasiliki Pogka
EPI_ISL_430469	Hellenic Pasteur Institute, Public Health Laboratories	Hellenic Pasteur Institute, Public Health Laboratories, Unit of Bioinformatics and Applied Genomics	Andreas Mentis; Androniki Voulgari-Kokota; Antonios Kalliaropoulos; Aspasia Kontou; Athanasios Kossyvakis; Evangelidou Maria; Horefti Elina; Timokratis Karamitros; Vasiliki Pogka
EPI_ISL_450525 to 450530, EPI_ISL_512296 to 512303	Hematology Laboratory, Section of Molecular Diagnostics, University Clinical Centre, Medical University of Gdansk	Department of Virology, Faculty of Medicine, University of Helsinki, Helsinki, Finland	Aneta Szulc; Ewa Miosz; Maciej Grzybek; Marlena Robakowska; Olli Vapalahti; Teemu Smura
EPI_ISL_451648 to 451654, EPI_ISL_906738, EPI_ISL_1040939, EPI_ISL_1040951, EPI_ISL_1040959, EPI_ISL_1040963, EPI_ISL_1040965 to 1040966, EPI_ISL_1040974, EPI_ISL_1040996			
see above	Hematology Laboratory, Section of Molecular Diagnostics, University Clinical Centre, Medical University of Gdansk	Laboratory of Recombinant Vaccines	Adam Sodol; Aneta Szulc; Boguslaw Szewczyk; Ewa Milosz; Krystyna Bienkowska-Szewczyk; Krzysztof Lewandowski; Lukasz Rabalski; Maciej Grzybek; Maciej Kosinski; Marlena Robakowska
EPI_ISL_699658 to 699706, EPI_ISL_699708 to 699709, EPI_ISL_699711 to 699721, EPI_ISL_699723 to 699751, EPI_ISL_699754 to 699766, EPI_ISL_699768 to 699809, EPI_ISL_699811 to 699813, EPI_ISL_699815 to 699816, EPI_ISL_699818 to 699819, EPI_ISL_699822 to 699824, EPI_ISL_699826, EPI_ISL_699828 to 699830, EPI_ISL_699832 to 699835, EPI_ISL_699838 to 699841, EPI_ISL_699843 to 699903, EPI_ISL_699905 to 699924, EPI_ISL_699926 to 699939, EPI_ISL_699941 to 700010, EPI_ISL_700012 to 700014, EPI_ISL_700016 to 700017, EPI_ISL_700019 to 700039, EPI_ISL_700042 to 700078, EPI_ISL_700080 to 700129, EPI_ISL_700131 to 700158, EPI_ISL_700160 to 700173, EPI_ISL_700175 to 700212, EPI_ISL_700214 to 700218, EPI_ISL_700220 to 700316, EPI_ISL_700318 to 700325			
see above	Hematopathology Laboratory, ACTREC, TMC	Hematopathology Laboratory, ACTREC, TMC	ACTREC; Hematopathology Laboratory
EPI_ISL_530169 to 530171, EPI_ISL_644942, EPI_ISL_683689	Hennepin County Medical Center	Minnesota Department of Health, Public Health Laboratory	Alexandra Lorentz; Jacob Garfin; Matt Plumb; and Xiong Wang
EPI_ISL_700565, EPI_ISL_700571	Herbertsdale Sat Clinic wc HBD	NHLS/UCT	Arash Iranzadeh; Bruna Galvao; Carolyn Williamson; Deelan Doolabh; Diana Hardie; Innocent Mudau; Kruger Marais; Lynn Tyers; Marvin Hsiao; Stephen Korsman
EPI_ISL_479903 to 479912	Himeji City Institute of Environment and Health	Pathogen Genomics Center, National Institute of Infectious Diseases	Hajime Kamiya; Kentaro Itokawa; Makoto Kuroda; Masanori Hashino; Motoi Suzuki; Rina Tanaka; Tsuyoshi Sekizuka
EPI_ISL_480178 to 480179	Hiroshima City Institute of Public Health	Pathogen Genomics Center, National Institute of Infectious Diseases	Hajime Kamiya; Kentaro Itokawa; Kota Noritsune; Makoto Kuroda; Masanori Hashino; Motoi Suzuki; Rina Tanaka; Tsuyoshi Sekizuka
EPI_ISL_745093 to 745094	Histopath	CIDM-PH, Westmead Hospital	CIDM-PH et al.
EPI_ISL_498774 to 498775, EPI_ISL_513318 to 513320, EPI_ISL_544960, EPI_ISL_544975, EPI_ISL_544987, EPI_ISL_545007, EPI_ISL_545034 to 545036, EPI_ISL_547600, EPI_ISL_591485 to 591486, EPI_ISL_593652, EPI_ISL_740879 to 740880, EPI_ISL_740896, EPI_ISL_767872 to 767875, EPI_ISL_767925 to 767926			
see above	Histopath	NSW Health Pathology - Institute of Clinical Pathology and Medical Research; Westmead Hospital; University of Sydney	CIDM-PH et al.
EPI_ISL_770013	Hle - Asilos De Ancianos	Inciensa, Instituto Costarricense de Investigación y Enseñanza en Nutrición y Salud	Adriana Godínez; Claudio Soto-Garita; Estela Cordero; Francisco Duarte; Hebleen Porras; Melany Calderón & Mariel López
EPI_ISL_770006	Hle - Asociacion Hogar De Ancianos De Palmar Sur De Osa	Inciensa, Instituto Costarricense de Investigación y Enseñanza en Nutrición y Salud	Adriana Godínez; Claudio Soto-Garita; Estela Cordero; Francisco Duarte; Hebleen Porras; Melany Calderón & Mariel López
EPI_ISL_770024	Hle - Asociacion Hogar De Ancianos Santiago Crespo Calvo	Inciensa, Instituto Costarricense de Investigación y Enseñanza en Nutrición y Salud	Adriana Godínez; Claudio Soto-Garita; Estela Cordero; Francisco Duarte; Hebleen Porras; Melany Calderón & Mariel López
EPI_ISL_770031 to 770032	Hle-Asociacion De Atencion Integral Del Anciano San Cayetano	Inciensa, Instituto Costarricense de Investigación y Enseñanza en Nutrición y Salud	Adriana Godínez; Claudio Soto-Garita; Estela Cordero; Francisco Duarte; Hebleen Porras; Melany Calderón & Mariel López
EPI_ISL_479792 to 479795, EPI_ISL_479801, EPI_ISL_479812 to 479820	Hokkaido Institute of Public Health	Pathogen Genomics Center, National Institute of Infectious Diseases	Hajime Kamiya; Kentaro Itokawa; Makoto Kuroda; Masanori Hashino; Motoi Suzuki; Rika Komagome; Rina Tanaka; Tsuyoshi Sekizuka
EPI_ISL_412028, EPI_ISL_412030	Hong Kong Department of Health	School of Public Health, The University of Hon g Kong	Daniel K.W. Chu; Dominic N.C. Tsang; Leo L.M. Poon; Malik Peiris
EPI_ISL_414517, EPI_ISL_414519, EPI_ISL_414527 to 414528, EPI_ISL_414569, EPI_ISL_414571, EPI_ISL_476801 to 476804, EPI_ISL_760031 to 760058			
see above	Hong Kong Department of Health	School of Public Health, The University of Hong Kong	Carrie Wan; Daisy Ng; Daniel Chu; Daniel K.W. Chu; Dominic N.C. Tsang; Gigi Liu; Haogao Gu; Leo L.M. Poon; Leo Poon; Malik Peiris; Pavithra Krishnan
EPI_ISL_412029	Hong Kong Department of Health	The University of Hong Kong	Daniel K.W. Chu; Dominic N.C. Tsang; Leo L.M. Poon; Malik Peiris
EPI_ISL_560581 to 560590, EPI_ISL_846637 to 846640, EPI_ISL_846644, EPI_ISL_872198, EPI_ISL_872206, EPI_ISL_872210 to 872211, EPI_ISL_872213 to 872220, EPI_ISL_872233, EPI_ISL_872236, EPI_ISL_872261, EPI_ISL_872266 to 872269, EPI_ISL_872291 to 872297, EPI_ISL_872307 to 872308, EPI_ISL_872315 to 872316, EPI_ISL_872318 to 872319, EPI_ISL_872325, EPI_ISL_883958, EPI_ISL_883972 to 883980, EPI_ISL_883982 to 883985, EPI_ISL_894219, EPI_ISL_912566 to 912567, EPI_ISL_935540 to 935542, EPI_ISL_935552 to 935557, EPI_ISL_935565, EPI_ISL_935570 to 935575, EPI_ISL_935581 to 935582, EPI_ISL_940995, EPI_ISL_941027, EPI_ISL_953941, EPI_ISL_953959 to 953962, EPI_ISL_953964 to 953967, EPI_ISL_953969 to 953985, EPI_ISL_953987, EPI_ISL_953989 to 953993, EPI_ISL_953995 to 953997, EPI_ISL_954000 to 954003, EPI_ISL_954009 to 954012, EPI_ISL_954014 to 954020, EPI_ISL_955061, EPI_ISL_955064 to 955065, EPI_ISL_955074 to 955075, EPI_ISL_955177, EPI_ISL_964916, EPI_ISL_995449 to 995461, EPI_ISL_995471 to 995472, EPI_ISL_995475, EPI_ISL_995477 to 995478, EPI_ISL_995480, EPI_ISL_995485 to 995486, EPI_ISL_995488, EPI_ISL_1001301 to 1001309, EPI_ISL_1001312 to 1001317, EPI_ISL_1001319 to 1001325, EPI_ISL_1001328, EPI_ISL_1001330, EPI_ISL_1001336 to 1001337, EPI_ISL_1001352, EPI_ISL_1001354 to 1001367, EPI_ISL_1001372 to 1001373, EPI_ISL_1001390			
see above	Hopital	National Reference Center for Viruses of Respiratory Infections, Institut Pasteur, Paris	Angela Brisebarre; Bigaillon Christine; Blazejewski C.; Bressollette (C) CéLine; Bret Laurent; Breuil J.; Brichler SéGolèNe; Brichler Ségolène; Camille Capel; Castelain Sandrine; Delamare Catherine; Ducancelle Alexandra; Eloy Clarence; Etienne Simon-Lorière; Fabiana Gambaro; Farfour Eric; Foissaud Vincent; Fougeaud Jacques; Goudeau Alain; Griscelli Franck; Guigon AuréLie; Guinard JéRome; Guinoiseau Thibault; Lagathu Gisele; Lagathu GisèLe; Laplanche Sophie; Larreche; Lesimple BéAtrice; Louvetl; Maneglie Benjamin; Marion Barbet; Marque Juillet StéPhanie; Martres Pascale; Maud Vanpeene; Merens Audrey; Morvan Odile; Méline Bizard; Pilorge LéA; Ramirez José-Manuel; Schvoerer Evelyne; Smati-Lafarge Mounira; Sylvie Behillil; Sylvie van der Werf; Vincent Enouf
EPI_ISL_912559	Hopital Avicenne Laboratoire central de bactériologie virologie hygiène	National Reference Center for Viruses of Respiratory Infections, Institut Pasteur, Paris	Angela Brisebarre; Brichler Ségolène; Camille Capel; Etienne Simon-Lorière; Marion Barbet; Maud Vanpeene; Méline Bizard; Sylvie Behillil; Sylvie van der Werf; Vincent Enouf
EPI_ISL_909666	Hopital Avicenne. Laboratoire central de bactériologie virologie hygiène	National Reference Center for Viruses of Respiratory Infections, Institut Pasteur, Paris	Angela Brisebarre; Brichler Ségolène; Camille Capel; Etienne Simon-Lorière; Marion Barbet; Maud Vanpeene; Méline Bizard; Sylvie Behillil; Sylvie van der Werf; Vincent Enouf
EPI_ISL_1036398 to 1036402	Hopital CH René Dubos - Laboratoire de Biologie médicale	National Reference Center for Viruses of Respiratory Infections, Institut Pasteur, Paris	Angela Brisebarre; Blanchard GenevièVe; Camille Capel; Etienne Simon-Lorière; Marion Barbet; Maud Vanpeene; Méline Bizard; Sylvie Behillil; Sylvie van der Werf; Vincent Enouf
EPI_ISL_1035904 to 1035916, EPI_ISL_1035924, EPI_ISL_1035927	Hopital CH de Troyes	National Reference Center for Viruses of Respiratory Infections, Institut Pasteur, Paris	Angela Brisebarre; Camille Capel; Etienne Simon-Lorière; Marion Barbet; Maud Vanpeene; Méline Bizard; Stoessel-Thouvenin Patricia; Sylvie Behillil; Sylvie van der Werf; Vincent Enouf
EPI_ISL_1035925 to 1035926	Hopital CHRU Tours	National Reference Center for Viruses of Respiratory Infections, Institut Pasteur, Paris	Angela Brisebarre; Camille Capel; Etienne Simon-Lorière; Guinoiseau Thibault; Marion Barbet; Maud Vanpeene; Méline Bizard; Sylvie Behillil; Sylvie van der Werf; Vincent Enouf
EPI_ISL_1036405 to 1036406	Hopital Groupe Hospitalier Intercommunal Le Raincy Montfermeil	National Reference Center for Viruses of Respiratory Infections, Institut Pasteur, Paris	Angela Brisebarre; Camille Capel; Etienne Simon-Lorière; Luizy Nelly; Marion Barbet; Maud Vanpeene; Méline Bizard; Sylvie Behillil; Sylvie van der Werf; Vincent Enouf
EPI_ISL_1036407	Hopital HIA PERCY	National Reference Center for Viruses of Respiratory	Angela Brisebarre; Camille Capel; Etienne Simon-Lorière; Foissaud Vincent; Marion Barbet; Maud Vanpeene; Méline Bizard; Sylvie Behillil; Sylvie van der

		Infections, Institut Pasteur, Paris	Werf; Vincent Enouf
EPI_ISL_1036427 to 1036428	Hopital Laboratoire de Virologie	National Reference Center for Viruses of Respiratory Infections, Institut Pasteur, Paris	Angela Brisebarre; Camille Capel; Etienne Simon-Lorière; Griscelli Franck; Marion Barbet; Maud Vanpeene; Méline Bizard; Sylvie Behillil; Sylvie van der Werf; Vincent Enouf
EPI_ISL_418427	Hopital Privé de l'Est Lyonnais	CNR Virus des Infections Respiratoires - France SUD	Alexandre Gaymard; Antonin Bal; Bruno Lina; Carine Moustaud; Florence Morfin-Sherpa; Gregory Destras; Gwendolyne Burfin; Laurence Josset; Martine Valette; Maude Bouscambert-Duchamp; Raphaelle Lamy; Solenne Brun
EPI_ISL_418229	Hopital franco britannique - Laboratoire	National Reference Center for Viruses of Respiratory Infections, Institut Pasteur, Paris	Angela Brisebarre; Etienne Simon-Lorière; Flora Donati; Marianne Asso Bonnet; Marion Barbet; Maud Vanpeene; Mélanie Albert; Méline Bizard; Sylvie Behillil; Sylvie van der Werf; Vincent Enouf
EPI_ISL_416501	Hopital franco britannique - Service des Urgences	National Reference Center for Viruses of Respiratory Infections, Institut Pasteur, Paris	Angela Brisebarre; Etienne Simon-Lorière; Flora Donati; Marion Barbet; Maud Vanpeene; Méline Bizard; Mélnie Albert; Sylvie Behillil; Sylvie van der Werf; Vincent Enouf
EPI_ISL_434666	Hornefors Halsocentral	The Public Health Agency of Sweden	Anna Risberg; Anna-Malin Linde; Camilla Eiback; Karin Tegmark-Wisell; Maria Lind Karlberg; Mia Brytting; Olov Svartstrom; Oskar Karlsson Lindsjo; Theresa Enkirch
EPI_ISL_700458, EPI_ISL_700524, EPI_ISL_700558	Hornlee Clinic wc HLC	NHLS/UCT	Arash Iranzadeh; Bruna Galvao; Carolyn Williamson; Deelan Doolabh; Diana Hardie; Houriiyah Tegally; Innocent Mudau; Kruger Marais; Lynn Tyers; Marvin Hsiao; Stephen Korsman
EPI_ISL_696486	Hornlee Clinic wc HLC & NHLS/UCT	KRISP, KZN Research Innovation and Sequencing Platform	Arash Iranzadeh; Bruna Galvao; Carolyn Williamson; Deelan Doolabh; Diana Hardie; Emanuel James San; Houriiyah Tegally; Innocent Mudau; Jennifer Giandhari; Kruger Marais; Lynn Tyers; Marvin Hsiao; Stephen Korsman; Sureshnee Pillay; Tulio de Oliveira
EPI_ISL_471562, EPI_ISL_471581 to 471582, EPI_ISL_515564, EPI_ISL_523977	Hosp. Municipal Prof. Dr. Alípio Corrêa Netto	Instituto Adolfo Lutz, Interdiciplinary Procedures Center, Strategic Laboratory	Claudia Regina Gonçalves; Claudio Tavares Sacchi; Erica Valessa Ramos Gomes
EPI_ISL_560598 to 560618, EPI_ISL_560620 to 560636, EPI_ISL_560643 to 560646, EPI_ISL_660323, EPI_ISL_693758 to 693762, EPI_ISL_693764, EPI_ISL_718226 to 718249, EPI_ISL_832169 to 832170, EPI_ISL_832176 to 832177, EPI_ISL_855353, EPI_ISL_855358 to 855359, EPI_ISL_855369, EPI_ISL_890359			
see above	Hospital	National Reference Center for Viruses of Respiratory Infections, Institut Pasteur, Paris	Alexandra Ducancelle; Angela Brisebarre; Aurélie Guigon; Camille Capel; Clémence Guillaume; Combe Patrice; Etienne Simon-Lorière; Fabiana Gambaro; Fourgeaud Jacques; Gisèle Lagathu; Griscelli Franck; Guigon AuréLie; Marion Barbet; Maud Vanpeene; Méline Bizard; Pilorge Léa; Sylvie Behillil; Sylvie van der Werf; Vincent Enouf
EPI_ISL_693229	Hospital 8 de Maio	Instituto Adolfo Lutz, Interdiciplinary Procedures Center, Strategic Laboratory	Claudia Regina Gonçalves; Claudio Tavares Sacchi; Erica Valessa Ramos Gomes; Karoline Rodrigues Campos
EPI_ISL_434366, EPI_ISL_434368 to 434384, EPI_ISL_450724 to 450732, EPI_ISL_450734, EPI_ISL_450736 to 450737	Hospital AZ Rivierenland	Institute of Tropical Medicine	; Colin Anthony; Philippe Selhorst
EPI_ISL_471554	Hospital Bosque da Saúde	Instituto Adolfo Lutz, Interdiciplinary Procedures Center, Strategic Laboratory	Claudia Regina Gonçalves; Claudio Tavares Sacchi; Erica Valessa Ramos Gomes
EPI_ISL_491439	Hospital Calderon Guardia	Inciensa, Instituto Costarricense de Investigación y Enseñanza en Nutrición y Salud	Adriana Godinez & Melany Calderon; Claudio Soto-Garita; Estela Cordero; Francisco Duarte; Hebleen Brenes
EPI_ISL_539559 to 539560	Hospital Campo Arañuelo	Instituto de Salud Carlos III	A. Monzón; F. Casas; G. Rodríguez; I; I. Jiménez; Iglesias-Caballero; J. López; M. Camarero; M. Cuesta; M. González-Esguevillas; M. Molinero Calamita; M. Zaballos; P. Jiménez; S. Juliá; S. Pozo; S. Varona
EPI_ISL_906078	Hospital Carlos Chagas	Instituto Adolfo Lutz, Interdiciplinary Procedures Center, Strategic Laboratory	Claudia Regina Gonçalves; Claudio Tavares Sacchi; Erica Valessa Ramos Gomes; Karoline Rodrigues Campos
EPI_ISL_574580	Hospital Cidade Tiradentes Carmen Prudente	Instituto Adolfo Lutz, Interdiciplinary Procedures Center, Strategic Laboratory	Claudia Regina Gonçalves; Claudio Tavares Sacchi; Erica Valessa Ramos Gomes; Karoline Rodrigues Campos
EPI_ISL_527743 to 527744	Hospital Cima	Inciensa, Instituto Costarricense de Investigación y Enseñanza en Nutrición y Salud	Adriana Godinez & Melany Calderon; Claudio Soto-Garita; Estela Cordero; Francisco Duarte; Hebleen Porras
EPI_ISL_462479	Hospital Clinic	Instituto de Salud Carlos III	A. Monzón; F. Casas; I; I. Jiménez; Iglesias-Caballero; M. Camarero; M. Cuesta; M. González-Esguevillas; M. Molinero Calamita; M. Zaballos; M.A Marcos; P. Jiménez; S. Juliá; S. Pozo; S. Varona
EPI_ISL_491441 to 491442, EPI_ISL_491455	Hospital Clinica Biblica	Inciensa, Instituto Costarricense de Investigación y Enseñanza en Nutrición y Salud	Adriana Godinez & Melany Calderon; Claudio Soto-Garita; Estela Cordero; Francisco Duarte; Hebleen Brenes
EPI_ISL_539534 to 539556	Hospital Clínic	Instituto de Salud Carlos III	A. Monzón; F. Casas; I; I. Jiménez; Iglesias-Caballero; M. Camarero; M. Cuesta; M. González-Esguevillas; M. Molinero Calamita; M. Zaballos; M.A Marcos; P. Jiménez; S. Juliá; S. Pozo; S. Varona
EPI_ISL_691700 to 691728, EPI_ISL_862548	Hospital Clínico San Carlos	Instituto de Salud Carlos III	A. Monzón; F. Casas; I. Jiménez; I. Rodríguez, I.; Iglesias-Caballero; M. Camarero; M. Cuesta; M. González-Esguevillas; M. Pozo; M. Zaballos; P. Jiménez; S. Juliá; S. Molinero Calamita; S. Varona
EPI_ISL_539500 to 539502, EPI_ISL_539509 to 539512, EPI_ISL_578190 to 578191	Hospital Clínico Universitario Lozano Blesa	Instituto de Salud Carlos III	A. Monzón; F. Casas; I; I. Jiménez; Iglesias-Caballero; M. Camarero; M. Cuesta; M. González-Esguevillas; M. Molinero Calamita; M. Zaballos; P. Jiménez; R. Benito; S. Juliá; S. Pozo; S. Varona
EPI_ISL_671797, EPI_ISL_671799, EPI_ISL_683595, EPI_ISL_1007859	Hospital Clínico Universitario Lozano Blesa de Zaragoza (España)	SeqCOVID-SPAIN consortium/IBV(CSIC)	Jessica Bueno and SeqCOVID-SPAIN consortium; Rafael Benito; Sonia Algarate
EPI_ISL_467262 to 467263, EPI_ISL_467265 to 467267, EPI_ISL_467269, EPI_ISL_467271 to 467281, EPI_ISL_467283 to 467285, EPI_ISL_467288 to 467297, EPI_ISL_510268 to 510269, EPI_ISL_510271 to 510275, EPI_ISL_510277, EPI_ISL_510279 to 510281, EPI_ISL_510283 to 510286, EPI_ISL_510288, EPI_ISL_510290 to 510294, EPI_ISL_510296 to 510298, EPI_ISL_510300 to 510303			
see above	Hospital Clínico Universitario de Santiago de Compostela	SeqCOVID-SPAIN consortium/IBV(CSIC)	Amparo Coira Nieto; Antonio Aguilera Guirao; Gema Barbeito Castiñeiras; José Javier Costa Alcalde; Mª Luisa Pérez del Molino Bernal; Rocio Trastoy Pena and SeqCOVID-SPAIN consortium
EPI_ISL_455344 to 455349, EPI_ISL_539567 to 539568, EPI_ISL_954798, EPI_ISL_965930, EPI_ISL_965936, EPI_ISL_965949	Hospital Comarcal de Melilla	Instituto de Salud Carlos III	A. Monzón; C. Ezpeleta; F. Casas; I; I. Jiménez; I. Pérez; I. Roman, S.; I. Román, S.; Iglesias-Caballero; J. López; M. Camarero; M. Cuesta; M. González-Esguevillas; M. Molinero Calamita; M. Sandonís; M. Zaballos; P. Jiménez; P. Zaballos; S. Camarero; S. Cuesta; S. Iglesias-Caballero; S. Juliá; S. Pozo; S. Sandonís; S. Varona; Sandonís; V. Vázquez
EPI_ISL_512665	Hospital De Las Mujeres Dr. Adolfo Carit	Inciensa, Instituto Costarricense de Investigación y Enseñanza en Nutrición y Salud	Adriana Godinez & Melany Calderon; Claudio Soto-Garita; Estela Cordero; Francisco Duarte; Hebleen Porras
EPI_ISL_512655, EPI_ISL_512672 to 512675, EPI_ISL_527750	Hospital De Niños Dr. Carlos Saenz Herrera [San Jose/San Jose]	Inciensa, Instituto Costarricense de Investigación y Enseñanza en Nutrición y Salud	Adriana Godinez & Melany Calderon; Claudio Soto-Garita; Estela Cordero; Francisco Duarte; Hebleen Porras
EPI_ISL_574591 to 574592, EPI_ISL_603030, EPI_ISL_693218	Hospital Domingos Leonardo Ceravolo Presidente Prudente	Instituto Adolfo Lutz, Interdiciplinary Procedures Center, Strategic Laboratory	Claudia Regina Gonçalves; Claudio Tavares Sacchi; Erica Valessa Ramos Gomes; Karoline Rodrigues Campos
EPI_ISL_547578	Hospital Doutor Domingos Leonardo Cerávolo	Instituto Adolfo Lutz, Interdiciplinary Procedures Center, Strategic Laboratory	Claudia Regina Gonçalves; Claudio Tavares Sacchi; Erica Valessa Ramos Gomes; Karoline Rodrigues Campos
EPI_ISL_527752, EPI_ISL_769990	Hospital Dr. Rafael A. Calderon Guardia	Inciensa, Instituto Costarricense de Investigación y Enseñanza en Nutrición y Salud	Adriana Godinez & Melany Calderon; Adriana Godínez; Claudio Soto-Garita; Estela Cordero; Francisco Duarte; Hebleen Porras; Melany Calderón & Mariel López
EPI_ISL_512654	Hospital Dr. Rafael A. Calderon Guardia [San Jose/San Jose]	Inciensa, Instituto Costarricense de Investigación y Enseñanza en Nutrición y Salud	Adriana Godinez & Melany Calderon; Claudio Soto-Garita; Estela Cordero; Francisco Duarte; Hebleen Porras
EPI_ISL_770028	Hospital Dr. Raul Blanco Cervantes	Inciensa, Instituto Costarricense de Investigación y Enseñanza en Nutrición y Salud	Adriana Godínez; Claudio Soto-Garita; Estela Cordero; Francisco Duarte; Hebleen Porras; Melany Calderón & Mariel López

EPI_ISL_693213	Hospital E Maternidade Municipal Governador Mario Covas	Instituto Adolfo Lutz, Interdiciplinary Procedures Center, Strategic Laboratory	Claudia Regina Gonçalves; Claudio Tavares Sacchi; Erica Valessa Ramos Gomes; Karoline Rodrigues Campos
EPI_ISL_574594	Hospital Escola da Universidade de Taubate	Instituto Adolfo Lutz, Interdiciplinary Procedures Center, Strategic Laboratory	Claudia Regina Gonçalves; Claudio Tavares Sacchi; Erica Valessa Ramos Gomes; Karoline Rodrigues Campos
EPI_ISL_574588, EPI_ISL_583490	Hospital Estadual Sumare	Instituto Adolfo Lutz, Interdiciplinary Procedures Center, Strategic Laboratory	Claudia Regina Gonçalves; Claudio Tavares Sacchi; Erica Valessa Ramos Gomes; Karoline Rodrigues Campos
EPI_ISL_491443, EPI_ISL_491447	Hospital Fernando Escalante Pradilla	Inciensa, Instituto Costarricense de Investigación y Enseñanza en Nutrición y Salud	Adriana Godinez & Melany Calderon; Claudio Soto-Garita; Estela Cordero; Francisco Duarte; Hebleen Brenes
EPI_ISL_476496, EPI_ISL_476561	Hospital Garrahan	Héritas	Andrea Mangano; Cristian Rohr; Dalmacio Pereyra; Fabian Fay; Maria Florencia Fernandez; Martin Vazquez; Mauricio Grisolia; Roberta Crespo
EPI_ISL_578186	Hospital General Juan Ramón Jiménez	Instituto de Salud Carlos III	A. Monzón; F. Casas; I; I. Jiménez; Iglesias-Caballero; J. Saavedra; M. Camarero; M. Cuesta; M. González-Esguevillas; M. Molinero Calamita; M. Zaballos; P. Jiménez; S. Juliá; S. Pozo; S. Varona
EPI_ISL_756273, EPI_ISL_803854 to 803864	Hospital General Universitario Gregorio Marañón	Hospital General Universitario Gregorio Marañón	Darío García de Viedma; Darío García de Viedma.; Laura Pérez-Lago; Patricia Muñoz; Pedro Sola Campoy; Pilar Catalán; Sergio Buenestado Serrano
EPI_ISL_912271, EPI_ISL_912275, EPI_ISL_912282, EPI_ISL_912287, EPI_ISL_912289, EPI_ISL_912291, EPI_ISL_912295 to 912296, EPI_ISL_912300 to 912301, EPI_ISL_912305, EPI_ISL_912307, EPI_ISL_912309 to 912310, EPI_ISL_912312, EPI_ISL_912316, EPI_ISL_912322, EPI_ISL_912326 to 912327, EPI_ISL_912332, EPI_ISL_912337 to 912338, EPI_ISL_912340 to 912341, EPI_ISL_912344 to 912347			
see above	Hospital General Universitario Gregorio Marañón	SeqCOVID-SPAIN consortium / IBV (CSIC)	Andrés Von Wernitz; Arturo fraile Torres; Carmen del Arco; Darío García de Viedma; Darío García de Viedma and SeqCOVID-SPAIN consortium; Julia Suárez; Laura Cardeñoso; Laura Pérez-Lago; Marta Herranz; Patricia Muñoz; Patricia Muñoz and SeqCOVID-SPAIN consortium; Pedro J Sola-Campoy; Pedro J. Sola Campoy; Pilar Catalán; Sergio Buenestado Serrano; Sergio Buenestado-Serrano; Victor Manuel de la Cueva
EPI_ISL_467184 to 467186, EPI_ISL_467188 to 467202, EPI_ISL_467205 to 467209, EPI_ISL_467211 to 467245, EPI_ISL_467247 to 467257, EPI_ISL_467259 to 467261, EPI_ISL_481041, EPI_ISL_481043 to 481045, EPI_ISL_481047 to 481049, EPI_ISL_481051 to 481052, EPI_ISL_481054 to 481060, EPI_ISL_481062 to 481094, EPI_ISL_481096 to 481109, EPI_ISL_510106, EPI_ISL_510110 to 510113, EPI_ISL_510117, EPI_ISL_510122, EPI_ISL_510128 to 510129, EPI_ISL_510131, EPI_ISL_510133, EPI_ISL_510135, EPI_ISL_510145 to 510146, EPI_ISL_510149, EPI_ISL_510165 to 510174, EPI_ISL_510176, EPI_ISL_510178, EPI_ISL_510180, EPI_ISL_510182 to 510183, EPI_ISL_510185 to 510186, EPI_ISL_510190 to 510196, EPI_ISL_510199, EPI_ISL_510201 to 510205, EPI_ISL_510207, EPI_ISL_510211 to 510213, EPI_ISL_510216 to 510218, EPI_ISL_510220 to 510228, EPI_ISL_510231 to 510233, EPI_ISL_510236 to 510238, EPI_ISL_510240, EPI_ISL_510243 to 510244, EPI_ISL_541878, EPI_ISL_541884, EPI_ISL_541886 to 541887, EPI_ISL_541889 to 541890, EPI_ISL_541892, EPI_ISL_541894 to 541900, EPI_ISL_541902 to 541908, EPI_ISL_541911 to 541913, EPI_ISL_541915 to 541922, EPI_ISL_541924 to 541929, EPI_ISL_541933 to 541934, EPI_ISL_541936, EPI_ISL_541938 to 541939, EPI_ISL_541941, EPI_ISL_582814, EPI_ISL_582826 to 582827, EPI_ISL_582843 to 582844, EPI_ISL_654053 to 654090, EPI_ISL_654092, EPI_ISL_654095 to 654106, EPI_ISL_654108 to 654123, EPI_ISL_654125 to 654134, EPI_ISL_654136 to 654139, EPI_ISL_654141 to 654161, EPI_ISL_654186 to 654247, EPI_ISL_654277 to 654327, EPI_ISL_654336 to 654400, EPI_ISL_814027 to 814036, EPI_ISL_853006 to 853029			
see above	Hospital General Universitario Gregorio Marañón	SeqCOVID-SPAIN consortium/IBV(CSIC)	Darío García de Viedma; Darío García de Viedma and SeqCOVID-SPAIN consortium; Jon Sicilia; Julia Suárez; Laura Pérez-Lago; Marta Herranz; Patricia Muñoz; Patricia Muñoz and SeqCOVID-SPAIN consortium; Pedro J Sola-Campoy; Pilar Catalán; Sergio Buenestado-Serrano; Victor Manuel de la Cueva
EPI_ISL_756272	Hospital General Universitario La Princesa	Hospital General Universitario Gregorio Marañón	Andrés Von Wernitz; Arturo fraile Torres; Carmen del Arco; Darío García de Viedma.; Laura Cardeñoso; Patricia Muñoz; Pedro Sola Campoy; Pilar Catalán; Sergio Buenestado Serrano
EPI_ISL_961214 to 961234	Hospital General Universitario de Alicante - Instituto de Investigación Sanitaria y Biomédica de Alicante	SeqCOVID-SPAIN consortium/IBV(CSIC)	Carmen Molina Pardines and SeqCOVID-SPAIN consortium; Maripaz Ventero Martín
EPI_ISL_691682 to 691683, EPI_ISL_954781, EPI_ISL_965914 to 965915, EPI_ISL_965926, EPI_ISL_965934, EPI_ISL_965940, EPI_ISL_965946, EPI_ISL_965959			
see above	Hospital General Universitario de Ciudad Real	Instituto de Salud Carlos III	A. Monzón; F. Casas; I. Illescas, S.; I. Jarilla, M.; I. Jiménez; I. Madrigal, M.; Iglesias-Caballero; M. Camarero; M. Cuesta; M. González-Esguevillas; M. Pozo; M. Sandonís; M. Zaballos; P. Jiménez; P. Zaballos; S. Camarero; S. Cuesta; S. Iglesias-Caballero; S. Juliá; S. Molinero Calamita; S. Pozo; S. Sandonís; S. Varona; Sandonís; V. Camarero; V. Vázquez; Vázquez
EPI_ISL_539523	Hospital General de Segovia	Instituto de Salud Carlos III	A. Monzón; F. Casas; I; I. Jiménez; Iglesias-Caballero; M. Camarero; M. Cuesta; M. González-Esguevillas; M. Molinero Calamita; M. Zaballos; P. Jiménez; S. Hernando; S. Juliá; S. Pozo; S. Varona
EPI_ISL_418245 to 418246	Hospital General y Universitario de Guadalajara	Instituto de Salud Carlos III	A. Monzón; F. Casas; I. Gonzalez-Praetorius A.; I. Jiménez; Iglesias-Caballero; M. Camarero; M. Cuesta; M. González-Esguevillas; M. Molinero Calamita; M. Zaballos; P. Jiménez; S. Juliá; S. Pozo; S. Varona
EPI_ISL_471541, EPI_ISL_523971	Hospital Geral Santa Marcelina	Instituto Adolfo Lutz, Interdiciplinary Procedures Center, Strategic Laboratory	Claudia Regina Gonçalves; Claudio Tavares Sacchi; Erica Valessa Ramos Gomes
EPI_ISL_534317	Hospital Geral de Itapevi	Instituto Adolfo Lutz, Interdiciplinary Procedures Center, Strategic Laboratory	Claudia Regina Gonçalves; Claudio Tavares Sacchi; Erica Valessa Ramos Gomes
EPI_ISL_603037	Hospital Geral de Pedreira	Instituto Adolfo Lutz, Interdiciplinary Procedures Center, Strategic Laboratory	Claudia Regina Gonçalves; Claudio Tavares Sacchi; Erica Valessa Ramos Gomes; Karoline Rodrigues Campos
EPI_ISL_515555	Hospital Geral de Vila Nova Cachoeirinha	Instituto Adolfo Lutz, Interdiciplinary Procedures Center, Strategic Laboratory	Claudia Regina Gonçalves; Claudio Tavares Sacchi; Erica Valessa Ramos Gomes
EPI_ISL_574595	Hospital Geral de Vila Penteado Dr. Jose Pamgella	Instituto Adolfo Lutz, Interdiciplinary Procedures Center, Strategic Laboratory	Claudia Regina Gonçalves; Claudio Tavares Sacchi; Erica Valessa Ramos Gomes; Karoline Rodrigues Campos
EPI_ISL_471267 to 471268, EPI_ISL_481244 to 481248, EPI_ISL_574431	Hospital IESS Babahoyo	Institute of Microbiology, Universidad San Francisco de Quito	Belén Prado-Vivar; Bernardo Gutiérrez; Carla Torres; Fernanda Zurita; Francisco Cordova; Gabriel Trueba; Juan José Guadalupe; Killen Briones-Claudette; Killen Briones-Zamora; Michelle Grunauer; Monica Becerra-Wong; Ninfa Henriquez; Patricio Rojas-Silva; Paúl Cárdenas; Sully Márquez; Verónica Barragán
EPI_ISL_412964	Hospital Israelita Albert Einstein	Instituto Adolfo Lutz Interdisciplinary Procedures Center Strategic Laboratory	Andrew Rambaut; Claudia Regina Gonçalves; Claudio Tavares Sacchi; Daniela Bernardes Borges da Silva; Ester Cerdeira Sabino; Flávia Cristina da Silva Sales; Ingra Morales Claro; Jaqueline Goes de Jesus; Joshua Quick; Maria do Carmo; Nicholas James Loman; Nuno Rodrigues Faria; Sampaio Tavares Timenetsky
EPI_ISL_414014, EPI_ISL_416033 to 416034	Hospital Israelita Albert Einstein	Instituto Adolfo Lutz, Interdiciplinary Procedures Center, Strategic Laboratory	Adriana Bugno; Adriano Abbud; Carlos Henrique Camargo; Claudia Regina Gonçalves; Claudio Tavares Sacchi; Daniela Bernardes Borges da Silva; Erica Valessa Ramos Gomes; Ester Cerdeira Sabino; Fabiana Cristina Pereira dos Santos; Katia Correia dos Santos; Maria do Carmo Sampaio Tavares Timenetsky; Maria do Carmo Sampaito Tavares Timenetsky; Simone Guadagnucci Morillo; Terezinha Maria de Paiva
EPI_ISL_413016	Hospital Israelita Albert Einstein	Instituto Adolfo Lutz, Interdisciplinary Procedures Center, Strategic Laboratory	Andrew Rambaut; Claudia Regina Gonçalves; Claudio Tavares Sacchi; Ester Cerdeira Sabino; Fabiana Cristina Pereira dos Santos; Flávia Cristina da Silva Sales; Ingra Morales Claro; Jaqueline Goes de Jesus; Joshua Quick; Maria do Carmo Sampaio Tavares Timenetsky; Nicholas James Loman; Nuno Rodrigues Faria
EPI_ISL_523957	Hospital Itamaraty	Instituto Adolfo Lutz, Interdiciplinary Procedures Center, Strategic Laboratory	Claudia Regina Gonçalves; Claudio Tavares Sacchi; Erica Valessa Ramos Gomes
EPI_ISL_862551, EPI_ISL_954787, EPI_ISL_954790	Hospital J.M. Morales Meseguer	Instituto de Salud Carlos III	A. Monzón; F. Casas; I. Guerrero, C.; I. Jiménez; I. Navarro, D.; Iglesias-Caballero; M. . Pozo; M. Camarero; M. Cuesta; M. González-Esguevillas; M. Zaballos; P. Jiménez; P. Zaballos; S. Cuesta; S. Juliá; S. Molinero Calamita; S. Pozo; S. Sandonís; S. Varona; V. Vázquez
EPI_ISL_954797, EPI_ISL_954801, EPI_ISL_954803	Hospital Los Arcos	Instituto de Salud Carlos III	A. Monzón; F. Casas; I. Camara, M.; I. Jiménez; Iglesias-Caballero; M. Camarero; P. Zaballos; S. Cuesta; S. Pozo; S. Sandonís; S. Varona; V. Vázquez
EPI_ISL_965911, EPI_ISL_965913, EPI_ISL_965917 to 965919, EPI_ISL_965923, EPI_ISL_965928 to 965929, EPI_ISL_965932, EPI_ISL_965935, EPI_ISL_965939, EPI_ISL_965941, EPI_ISL_965945, EPI_ISL_965947, EPI_ISL_965951, EPI_ISL_965955, EPI_ISL_965958			
see above	Hospital Los Arcos del Mar Menor	Instituto de Salud Carlos III	A. Monzón; F. Casas; I. Cámara, M.; I. Jiménez; Iglesias-Caballero; M. Camarero; M. Sandonís; P. Zaballos; S. Camarero; S. Cuesta; S. Iglesias-Caballero; S. Pozo; S. Varona; Sandonís; V. Camarero; V. Vázquez; Vázquez
EPI_ISL_527741, EPI_ISL_769992, EPI_ISL_769995	Hospital Metropolitano	Inciensa, Instituto Costarricense de Investigación y Enseñanza en Nutrición y Salud	Adriana Godinez & Melany Calderon; Adriana Godínez; Claudio Soto-Garita; Estela Cordero; Francisco Duarte; Hebleen Porras; Melany Calderón & Margarita Lee Lui
EPI_ISL_524462	Hospital Metropolitano	Instituto Adolfo Lutz, Interdiciplinary Procedures Center,	Claudia Regina Gonçalves; Claudio Tavares Sacchi; Erica Valessa Ramos Gomes

		Strategic Laboratory	
EPI_ISL_527739	Hospital Mexico [San Jose/San Jose]	Inciensa, Instituto Costarricense de Investigación y Enseñanza en Nutrición y Salud	Adriana Godinez & Melany Calderon; Claudio Soto-Garita; Estela Cordero; Francisco Duarte; Hebleen Porras
EPI_ISL_515541, EPI_ISL_515561	Hospital Montemagno	Instituto Adolfo Lutz, Interdiciplinary Procedures Center, Strategic Laboratory	Claudia Regina Gonçalves; Claudio Tavares Sacchi; Erica Valessa Ramos Gomes
EPI_ISL_534324	Hospital Mun Ver Jose Storopolli	Instituto Adolfo Lutz, Interdiciplinary Procedures Center, Strategic Laboratory	Claudia Regina Gonçalves; Claudio Tavares Sacchi; Erica Valessa Ramos Gomes
EPI_ISL_534318, EPI_ISL_693208 to 693209	Hospital Municipal Antonio Giglio	Instituto Adolfo Lutz, Interdiciplinary Procedures Center, Strategic Laboratory	Claudia Regina Gonçalves; Claudio Tavares Sacchi; Erica Valessa Ramos Gomes; Karoline Rodrigues Campos
EPI_ISL_547571	Hospital Municipal Antônio Giglio	Instituto Adolfo Lutz, Interdiciplinary Procedures Center, Strategic Laboratory	Claudia Regina Gonçalves; Claudio Tavares Sacchi; Erica Valessa Ramos Gomes; Karoline Rodrigues Campos
EPI_ISL_471549, EPI_ISL_523991 to 523992, EPI_ISL_527869	Hospital Municipal Carmen Prudente	Instituto Adolfo Lutz, Interdiciplinary Procedures Center, Strategic Laboratory	Claudia Regina Gonçalves; Claudio Tavares Sacchi; Erica Valessa Ramos Gomes
EPI_ISL_515562	Hospital Municipal Doutor Alexandre Zaio	Instituto Adolfo Lutz, Interdiciplinary Procedures Center, Strategic Laboratory	Claudia Regina Gonçalves; Claudio Tavares Sacchi; Erica Valessa Ramos Gomes
EPI_ISL_693203	Hospital Municipal Doutor Arthur Ribeiro de Saboya	Instituto Adolfo Lutz, Interdiciplinary Procedures Center, Strategic Laboratory	Claudia Regina Gonçalves; Claudio Tavares Sacchi; Erica Valessa Ramos Gomes; Karoline Rodrigues Campos
EPI_ISL_468311 to 468312	Hospital Municipal Dr Ignacio Proenca de Gouvea	Instituto Adolfo Lutz, Interdiciplinary Procedures Center, Strategic Laboratory	Claudia Regina Gonçalves; Claudio Tavares Sacchi; Erica Valessa Ramos Gomes
EPI_ISL_515521	Hospital Municipal Dr Waldemar Tebaldi	Instituto Adolfo Lutz, Interdiciplinary Procedures Center, Strategic Laboratory	Claudia Regina Gonçalves; Claudio Tavares Sacchi; Erica Valessa Ramos Gomes
EPI_ISL_523985	Hospital Municipal Dr. Benedicto Montenegro	Instituto Adolfo Lutz, Interdiciplinary Procedures Center, Strategic Laboratory	Claudia Regina Gonçalves; Claudio Tavares Sacchi; Erica Valessa Ramos Gomes
EPI_ISL_515553, EPI_ISL_574577, EPI_ISL_574579	Hospital Municipal Dr. Ignacio Proença de Gouvea	Instituto Adolfo Lutz, Interdiciplinary Procedures Center, Strategic Laboratory	Claudia Regina Gonçalves; Claudio Tavares Sacchi; Erica Valessa Ramos Gomes; Karoline Rodrigues Campos
EPI_ISL_515548, EPI_ISL_515563, EPI_ISL_574582, EPI_ISL_574589	Hospital Municipal Dr. Jose Soares Hungria	Instituto Adolfo Lutz, Interdiciplinary Procedures Center, Strategic Laboratory	Claudia Regina Gonçalves; Claudio Tavares Sacchi; Erica Valessa Ramos Gomes; Karoline Rodrigues Campos
EPI_ISL_515557, EPI_ISL_524467	Hospital Municipal Dr. Moysés Deutsch	Instituto Adolfo Lutz, Interdiciplinary Procedures Center, Strategic Laboratory	Claudia Regina Gonçalves; Claudio Tavares Sacchi; Erica Valessa Ramos Gomes
EPI_ISL_583498	Hospital Municipal Dr. Waldemar Tebaldi	Instituto Adolfo Lutz, Interdiciplinary Procedures Center, Strategic Laboratory	Claudia Regina Gonçalves; Claudio Tavares Sacchi; Erica Valessa Ramos Gomes; Karoline Rodrigues Campos
EPI_ISL_693206	Hospital Municipal Mario Gatti	Instituto Adolfo Lutz, Interdiciplinary Procedures Center, Strategic Laboratory	Claudia Regina Gonçalves; Claudio Tavares Sacchi; Erica Valessa Ramos Gomes; Karoline Rodrigues Campos
EPI_ISL_527870, EPI_ISL_574578, EPI_ISL_603029, EPI_ISL_603039	Hospital Municipal Mário Gatti	Instituto Adolfo Lutz, Interdiciplinary Procedures Center, Strategic Laboratory	Claudia Regina Gonçalves; Claudio Tavares Sacchi; Erica Valessa Ramos Gomes; Karoline Rodrigues Campos
EPI_ISL_527856	Hospital Municipal Prof. Waldomiro de Paula	Instituto Adolfo Lutz, Interdiciplinary Procedures Center, Strategic Laboratory	Claudia Regina Gonçalves; Claudio Tavares Sacchi; Erica Valessa Ramos Gomes
EPI_ISL_603028	Hospital Municipal Santa Ana	Instituto Adolfo Lutz, Interdiciplinary Procedures Center, Strategic Laboratory	Claudia Regina Gonçalves; Claudio Tavares Sacchi; Erica Valessa Ramos Gomes; Karoline Rodrigues Campos
EPI_ISL_524468, EPI_ISL_527859	Hospital Municipal Vereador Jose Storopolli	Instituto Adolfo Lutz, Interdiciplinary Procedures Center, Strategic Laboratory	Claudia Regina Gonçalves; Claudio Tavares Sacchi; Erica Valessa Ramos Gomes
EPI_ISL_471647	Hospital Municipal de Barueri Dr. Francisco Moran	Instituto Adolfo Lutz, Interdiciplinary Procedures Center, Strategic Laboratory	Claudia Regina Gonçalves; Claudio Tavares Sacchi; Erica Valessa Ramos Gomes
EPI_ISL_527860	Hospital Municipal de Parelheiros Josanias Castanha Braga	Instituto Adolfo Lutz, Interdiciplinary Procedures Center, Strategic Laboratory	Claudia Regina Gonçalves; Claudio Tavares Sacchi; Erica Valessa Ramos Gomes
EPI_ISL_527862	Hospital Municipal de Urgência	Instituto Adolfo Lutz, Interdiciplinary Procedures Center, Strategic Laboratory	Claudia Regina Gonçalves; Claudio Tavares Sacchi; Erica Valessa Ramos Gomes
EPI_ISL_468308, EPI_ISL_468315, EPI_ISL_515520, EPI_ISL_515526, EPI_ISL_515546, EPI_ISL_515551 to 515552, EPI_ISL_523955, EPI_ISL_523974, EPI_ISL_523976, EPI_ISL_527863			
see above	Hospital Municipal do Tatuape Carmino Caricchio	Instituto Adolfo Lutz, Interdiciplinary Procedures Center, Strategic Laboratory	Claudia Regina Gonçalves; Claudio Tavares Sacchi; Erica Valessa Ramos Gomes
EPI_ISL_735399	Hospital Municiplal Dr Ignacio de gouvea	Instituto Adolfo Lutz, Interdiciplinary Procedures Center, Strategic Laboratory	Claudia Regina Gonçalves; Claudio Tavares Sacchi; Erica Valessa Ramos Gomes; Karoline Rodrigues Campos
EPI_ISL_491451, EPI_ISL_491453	Hospital México	Inciensa, Instituto Costarricense de Investigación y Enseñanza en Nutrición y Salud	Adriana Godinez & Melany Calderon; Claudio Soto-Garita; Estela Cordero; Francisco Duarte; Hebleen Brenes
EPI_ISL_735428 to 735429, EPI_ISL_735431 to 735432	Hospital Nipo Brasileiro	Instituto Adolfo Lutz, Interdiciplinary Procedures Center, Strategic Laboratory	Claudia Regina Gonçalves; Claudio Tavares Sacchi; Erica Valessa Ramos Gomes; Karoline Rodrigues Campos
EPI_ISL_539496	Hospital Nostra Senyora de Meritxell	Instituto de Salud Carlos III	A. Monzón; F. Casas; F. Fernández; I; I. Jiménez; Iglesias-Caballero; M. Camarero; M. Cuesta; M. González-Esguevillas; M. Molinero Calamita; M. Zaballos; P. Jiménez; S. Juliá; S. Pozo; S. Varona
EPI_ISL_539525	Hospital Nuestra Señora de Sonsoles	Instituto de Salud Carlos III	A. Monzón; A. San Pedro; F. Casas; I; I. Jiménez; Iglesias-Caballero; M. Camarero; M. Cuesta; M. González-Esguevillas; M. Molinero Calamita; M. Zaballos; P. Jiménez; S. Juliá; S. Pozo; S. Varona
EPI_ISL_471269 to 471271	Hospital Oncológico Solca Núcleo de Quito	Institute of Microbiology, Universidad San Francisco de Quito	Belén Prado-Vivar; Bernardo Gutiérrez; Gabriel Trueba; Grace Salazar; Juan José Guadalupe; Marcos Di Stefano; Michelle Grunauer; Patricio Rojas-Silva; Paúl Cárdenas; Sully Márquez; Verónica Barragán
EPI_ISL_977502	Hospital Oriente de Asturias	Laboratorio de Virología HUCA	Abreu F; Alvarez-Arguelles ME; Boga JA; Castelló C; Costales I; Coto E; Gómez de Oña J; Martín-Rodríguez G; Melón S; Perez-Martínez Z; Rojo S; Sandoval M
EPI_ISL_906079, EPI_ISL_940610 to 940611	Hospital Paulistano Paulista	Instituto Adolfo Lutz, Interdiciplinary Procedures Center, Strategic Laboratory	Claudia Regina Gonçalves; Claudio Tavares Sacchi; Erica Valessa Ramos Gomes; Karoline Rodrigues Campos
EPI_ISL_419386 to 419387	Hospital Prof. Doutor Fernando Fonseca, EPE	Instituto Gulbenkian de Ciência	Cathy Paulino; Joao Sobral; João Costa; Ricardo Leite; Susana Ladeiro
EPI_ISL_882612 to 882613, EPI_ISL_882615 to 882618, EPI_ISL_882620 to 882621, EPI_ISL_882623, EPI_ISL_882625,	Hospital Ramón y Cajal	Hospital Ramón y Cajal	Concepción Rodríguez; JC Galán; José M Gonzalez-Alba; Laura Martínez; Luz Leticia Olavarrieta; Melanie Abreu; Rafael Cantón; Val F Lanza

EPI_ISL_882628 to 882629			
EPI_ISL_527857	Hospital Regional Vale do Ribeira	Instituto Adolfo Lutz, Interdiciplinary Procedures Center, Strategic Laboratory	Claudia Regina Gonçalves; Claudio Tavares Sacchi; Erica Valessa Ramos Gomes
EPI_ISL_523956	Hospital Regional de Assis	Instituto Adolfo Lutz, Interdiciplinary Procedures Center, Strategic Laboratory	Claudia Regina Gonçalves; Claudio Tavares Sacchi; Erica Valessa Ramos Gomes
EPI_ISL_524463	Hospital Regional de Cotia	Instituto Adolfo Lutz, Interdiciplinary Procedures Center, Strategic Laboratory	Claudia Regina Gonçalves; Claudio Tavares Sacchi; Erica Valessa Ramos Gomes
EPI_ISL_735418	Hospital Regional do Vale do Paraiba	Instituto Adolfo Lutz, Interdiciplinary Procedures Center, Strategic Laboratory	Claudia Regina Gonçalves; Claudio Tavares Sacchi; Erica Valessa Ramos Gomes; Karoline Rodrigues Campos
EPI_ISL_833166	Hospital Samaritano	Instituto Adolfo Lutz, Interdiciplinary Procedures Center, Strategic Laboratory	Claudia Regina Gonçalves; Claudio Tavares Sacchi; Erica Valessa Ramos Gomes; Karoline Rodrigues Campos
EPI_ISL_882670	Hospital Samaritano Paulista	Instituto Adolfo Lutz, Interdiciplinary Procedures Center, Strategic Laboratory	Claudia Regina Gonçalves; Claudio Tavares Sacchi; Erica Valessa Ramos Gomes; Karoline Rodrigues Campos
EPI_ISL_970179	Hospital San Agustin, Avilés	Laboratorio de Virología HUCA	Abreu F; Alvarez-Arguelles ME; Boga JA; Castelló C; Costales I; Coto E; Gómez de Oña J; Martín-Rodríguez G; Melón S; Perez-Martínez Z; Rojo S; Sandoval M
EPI_ISL_1020017, EPI_ISL_1020125	Hospital San Agustín	Laboratorio de Virología HUCA	Abreu F; Alvarez-Arguelles ME; Boga JA; Castelló C; Costales I; Coto E; Gómez de Oña J; Martín-Rodríguez G; Melón S; Perez-Martínez Z; Rojo S; Sandoval M
EPI_ISL_993630	Hospital San Agustín	Laboratorio de Virología Hospital Universitario Central de Asturias (HUCA)	Abreu F; Alvarez-Arguelles ME; Boga JA; Castelló C; Costales I; Coto E; Gómez de Oña J; Martín-Rodríguez G; Melón S; Perez-Martínez Z; Rojo S; Sandoval M
EPI_ISL_491450, EPI_ISL_491454, EPI_ISL_491456	Hospital San Juan de Dios	Inciensa, Instituto Costarricense de Investigación y Enseñanza en Nutrición y Salud	Adriana Godinez & Melany Calderon; Claudio Soto-Garita; Estela Cordero; Francisco Duarte; Hebleen Brenes
EPI_ISL_419234, EPI_ISL_455336 to 455343, EPI_ISL_578196 to 578201	Hospital San Pedro	Instituto de Salud Carlos III	A. Monzón; Alonso, C.; C. Alonso; Camarero, S.; Casas, I.; Cuesta, I.; F. Casas; González-Esguevillas, M.; I; I. Jiménez; Iglesias-Caballero; Iglesias-Caballero, M.; J.M Azcona; Jiménez, M.; Jiménez, P.; Juliá, M.; M. Blasco; M. Camarero; M. Cuesta; M. González-Esguevillas; M. Molinero Calamita; M. Zaballos; Molinero Calamita, M.; Monzón, S.; P. Jiménez; Pozo, F.; S. Juliá; S. Pozo; S. Varona; Varona, S.; Zaballos, A.
EPI_ISL_539558, EPI_ISL_539561 to 539562, EPI_ISL_913021 to 913023, EPI_ISL_913032 to 913035	Hospital San Pedro de Alcántara	Instituto de Salud Carlos III	A. Monzón; E. Cerro; F. Casas; I; I. Jiménez; I. Rodríguez, G.; Iglesias-Caballero; J. López; M. Camarero; M. Cuesta; M. González-Esguevillas; M. Molinero Calamita; M. Zaballos; M.A Cañizares; P. Jiménez; P. Zaballos; S. Cuesta; S. Juliá; S. Pozo; S. Sandonís; S. Varona; V. Vázquez
EPI_ISL_510305 to 510307, EPI_ISL_510309 to 510331	Hospital San Pedro de Alcántara (Cáceres)	SeqCOVID-SPAIN consortium/IBV(CSIC)	Cristina Muñoz Cuevas; Guadalupe Rodriguez Rodriguez and SeqCOVID-SPAIN consortium
EPI_ISL_491438, EPI_ISL_491440, EPI_ISL_491448, EPI_ISL_491452	Hospital San Rafael de Alajuela	Inciensa, Instituto Costarricense de Investigación y Enseñanza en Nutrición y Salud	Adriana Godinez & Melany Calderon; Claudio Soto-Garita; Estela Cordero; Francisco Duarte; Hebleen Brenes
EPI_ISL_527753 to 527754	Hospital San Vicente De Paul	Inciensa, Instituto Costarricense de Investigación y Enseñanza en Nutrición y Salud	Adriana Godinez & Melany Calderon; Claudio Soto-Garita; Estela Cordero; Francisco Duarte; Hebleen Porras
EPI_ISL_471552, EPI_ISL_523967	Hospital Sancta Maggiore	Instituto Adolfo Lutz, Interdiciplinary Procedures Center, Strategic Laboratory	Claudia Regina Gonçalves; Claudio Tavares Sacchi; Erica Valessa Ramos Gomes
EPI_ISL_603036	Hospital Santa Ana	Instituto Adolfo Lutz, Interdiciplinary Procedures Center, Strategic Laboratory	Claudia Regina Gonçalves; Claudio Tavares Sacchi; Erica Valessa Ramos Gomes; Karoline Rodrigues Campos
EPI_ISL_515527, EPI_ISL_693196	Hospital Santa Clara	Instituto Adolfo Lutz, Interdiciplinary Procedures Center, Strategic Laboratory	Claudia Regina Gonçalves; Claudio Tavares Sacchi; Erica Valessa Ramos Gomes; Karoline Rodrigues Campos
EPI_ISL_693233	Hospital Santa Cruz	Instituto Adolfo Lutz, Interdiciplinary Procedures Center, Strategic Laboratory	Claudia Regina Gonçalves; Claudio Tavares Sacchi; Erica Valessa Ramos Gomes; Karoline Rodrigues Campos
EPI_ISL_693236	Hospital Santa Marcelina Sao Paulo	Instituto Adolfo Lutz, Interdiciplinary Procedures Center, Strategic Laboratory	Claudia Regina Gonçalves; Claudio Tavares Sacchi; Erica Valessa Ramos Gomes; Karoline Rodrigues Campos
EPI_ISL_468310	Hospital Sao Paulo de Ensino da UNIFESP	Instituto Adolfo Lutz, Interdiciplinary Procedures Center, Strategic Laboratory	Claudia Regina Gonçalves; Claudio Tavares Sacchi; Erica Valessa Ramos Gomes
EPI_ISL_471545, EPI_ISL_471551, EPI_ISL_515528, EPI_ISL_515545, EPI_ISL_515559 to 515560, EPI_ISL_523969, EPI_ISL_523981, EPI_ISL_523988, EPI_ISL_693201			
see above	Hospital Sao Paulo de Ensino da Unifesp	Instituto Adolfo Lutz, Interdiciplinary Procedures Center, Strategic Laboratory	Claudia Regina Gonçalves; Claudio Tavares Sacchi; Erica Valessa Ramos Gomes; Karoline Rodrigues Campos
EPI_ISL_524484	Hospital Schwyz	Institute of Medical Virology, University of Zurich	Alexandra Trkola; Andrea Zbinden; Fiona Steiner; Gabriela Ziltener; Jon Huder; Jürg Böni; Maryam Zaheri; Michael Huber; Patrick Redli; Riccarda Capaul; Stefan Schmutz; Verena Kufner
EPI_ISL_414015 to 414017	Hospital São Joaquim Beneficencia Portuguesa	Instituto Adolfo Lutz, Interdiciplinary Procedures Center, Strategic Laboratory	Audrey Cilli; Carlos Henrique Camargo; Claudia Regina Gonçalves; Claudio Tavares Sacchi; Daniela Bernardes Borges da Silva; Ester Cerdeira Sabino; Fabiana Cristina Pereira dos Santos; Fabiana Cristina Pereira dos Santos Terezinha Maria de Paiva; Maria do Carmo Sampaito Tavares Timenetsky; SimoneGuadagnucci Morillo; Terezinha Maria de Paiva
EPI_ISL_455350 to 455351	Hospital Txagorritxu	Instituto de Salud Carlos III	A. Monzón; C. Gómez; F. Casas; I; I. Jiménez; Iglesias-Caballero; M. Camarero; M. Cuesta; M. González-Esguevillas; M. Molinero Calamita; M. Zaballos; P. Jiménez; S. Juliá; S. Pozo; S. Varona
EPI_ISL_483059	Hospital Universitari Germans Trias i Pujol	IrsiCaixa AIDS Research Lab	A. Valencia; B. Clotet; C. Avila-Nieto; E. Vidal; G. Cantero; I. Blanco; J. Blanco; J. Carrillo; J. Rodon; J. Segalés; J. Vergara-Alert; M. Noguera-Julián; M. Parera; M. Puig; M.T. Terrón; N. Izquierdo-Useros; S. Cruz; V. Guallar
EPI_ISL_1012925	Hospital Universitari Germans Trias i Pujol(HUGTiP)/Fundació Lluita contra la SIDA (FLSida)	IrsiCaixa - Can Ruti CovidSeq	Bonaventura Clotet; Bonaventura Clotet Marta Massanella; Ester Ballana; Francesc Catala-Moll; Joaquim Segalés; Jorge Carrillo; Julia Blanco; Lidia Ruiz; Marc Noguera-Julian; Maria Casadellà; Mariona Parera; Nuria Izquierdo; Pilar Armengol; Roger Paredes
EPI_ISL_535575, EPI_ISL_538552	Hospital Universitari Germans Trias i Pujol(HUGTiP)/Fundació Lluita contra la SIDA (FLSida)	IrsiCaixa AIDS Research Lab	Bonaventura Clotet; Ester Ballana; Joaquim Segalés; Jorge Carrillo; Julia Blanco; Lidia Ruiz; Marc Noguera-Julian; Maria Pilar Armengol; Mariona Parera; Marta Massanella; Nuria Izquierdo; Roger Paredes
EPI_ISL_471472, EPI_ISL_510689	Hospital Universitari Germans Trias i Pujol(HUGTiP)/Fundació Lluita contra la SIDA (FLSida)/IRTA-CReSA	IrsiCaixa AIDS Research Lab	Albert Bensaid; Bonaventura Clotet; Joaquim Segalés; Jordi Rodón; Jorge Carrillo; Julia Blanco; Julia Vergara; Lidia Ruiz; Marc Noguera-Julian; Nuria Izquierdo; Pilar Armengol; Roger Paredes
EPI_ISL_418860 to 418861, EPI_ISL_444971 to 444990, EPI_ISL_447532 to 447533, EPI_ISL_450337, EPI_ISL_458130 to 458132, EPI_ISL_728361 to 728362, EPI_ISL_728364 to 728365, EPI_ISL_824435, EPI_ISL_850516, EPI_ISL_918292, EPI_ISL_918296, EPI_ISL_918299, EPI_ISL_918301, EPI_ISL_918304, EPI_ISL_954144 to 954177, EPI_ISL_954181 to 954182, EPI_ISL_981297 to 981304, EPI_ISL_981308, EPI_ISL_981318, EPI_ISL_981320, EPI_ISL_981322, EPI_ISL_981328 to 981329, EPI_ISL_981338, EPI_ISL_981345 to 981347, EPI_ISL_981351, EPI_ISL_981353, EPI_ISL_981365 to 981366, EPI_ISL_1020559 to 1020568, EPI_ISL_1020571 to 1020573			
see above	Hospital Universitari Vall d'Hebron - Vall d'Hebron Institut de Recerca	Hospital Universitari Vall d'Hebron - Vall d'Hebron Institut de Recerca	Andrés Antón; Ariadna Rando; Aroa Silgado; Carla Castillo; Cristina Andrés; DAmir Garcia-Cehic; Damir Garcia-Cehic; Dàmir Garcia-Cehic; Elena Sulleiro; Josep F Abril; Josep Gregori; Josep Quer; Juliana Esperalba; Maria Carmen Martin; Maria Carmen Martín; Maria Gema Codina; Maria Piñana; Mercedes Guerrero-Murillo; Tomàs Pumarola
EPI_ISL_850511 to 850513, EPI_ISL_850515, EPI_ISL_981270 to 981272, EPI_ISL_981274 to	Hospital Universitari de Bellvitge	Hospital Universitari Vall d'Hebron - Vall d'Hebron Institut de Recerca	Andrés Antón; Ariadna Rando; Carla Castillo; Cristina Andrés; Damir Garcia-Cehic; Josep F Abril; Josep Quer; Juliana Esperalba; Maria Carmen Martin; Maria Gema Codina; Maria Piñana; Tomàs Pumarola

981291, EPI_ISL_981293 to 981296			
EPI_ISL_538012 to 538028, EPI_ISL_538030, EPI_ISL_538033 to 538039, EPI_ISL_538041 to 538052, EPI_ISL_538054	Hospital Universitari i Politècnic La Fe de València	SeqCOVID-SPAIN consortium/IBV(CSIC)	Ana Gil Brusola; Eva González Barbera; José Luis López Hontangas and SeqCOVID-SPAIN consortium; María Dolores Gómez Ruiz; Salvador Giner Almaraz
EPI_ISL_421171 to 421180, EPI_ISL_428683 to 428684, EPI_ISL_428686 to 428701, EPI_ISL_428703 to 428711, EPI_ISL_430718, EPI_ISL_529972 to 530024, EPI_ISL_534327 to 534329			
see above	Hospital Universitario 12 de Octubre	Hospital Universitario 12 de Octubre	Elias Dahdouh; Esther Viedma; Fernando Lázaro; Jesús Mingorance; Juan Carlos Galán; Julio García; Ma Dolores Folgueira; Mª Dolores Folgueira; Natalia Stella; Rafael Cantón; Rafael Delgado; Raúl Recio; Sara González
EPI_ISL_417954, EPI_ISL_417961, EPI_ISL_417963, EPI_ISL_417967	Hospital Universitario 12 de Octubre	Hospital Universitario La Paz	Elias Dahdouh; Esther Viedma; Fernando Lázaro; Jesús Mingorance; Juan Carlos Galán; Julio García; Mª Dolores Folgueira; Natalia Stella; Rafael Cantón; Rafael Delgado; Sara González
EPI_ISL_467092 to 467101, EPI_ISL_467103 to 467111, EPI_ISL_467113 to 467118, EPI_ISL_467120 to 467132, EPI_ISL_467134, EPI_ISL_467136 to 467183, EPI_ISL_509616 to 509618			
see above	Hospital Universitario Araba. Vitoria-Gasteiz	SeqCOVID-SPAIN consortium/IBV(CSIC)	Amaia Aguirre Quiñonero; Andrés Canut Blasco and SeqCOVID-SPAIN consortium; Andrés Canut Blasco. and SeqCOVID-SPAIN consortium; Carmen Gómez González; Marina Fernández Torres; Mª Concepción Lecaroz Agara; Mª Rosario Almela Ferrer; Silvia Hernáez Crespo
EPI_ISL_452692 to 452696, EPI_ISL_452698 to 452701, EPI_ISL_452703 to 452704, EPI_ISL_452707 to 452733, EPI_ISL_452735 to 452751, EPI_ISL_452753 to 452755, EPI_ISL_452759, EPI_ISL_452761 to 452763, EPI_ISL_452765 to 452775, EPI_ISL_452777, EPI_ISL_452780, EPI_ISL_452782 to 452786			
see above	Hospital Universitario Araba. Vitoria-Gasteiz,	SeqCOVID-SPAIN consortium/IBV(CSIC)	Amaia Aguirre Quiñonero; Andrés Canut Blasco and SeqCOVID-SPAIN consortium; Carmen Gómez González; Maria Concepción Lecaroz Agara; Maria Rosario Almela Ferrer; Marina Fernández Torres; Silvia Hernáez Crespo
EPI_ISL_455326	Hospital Universitario Insular de Gran Canaria	Instituto de Salud Carlos III	A. Hernández; A. Monzón; F. Casas; I; I. Jiménez; Iglesias-Caballero; M. Camarero; M. Cuesta; M. González-Esguevillas; M. Molinero Calamita; M. Zaballos; P. Jiménez; S. Juliá; S. Pozo; S. Varona
EPI_ISL_428674 to 428682, EPI_ISL_430719 to 430721, EPI_ISL_435144	Hospital Universitario La Paz	Hospital Universitario 12 de Octubre	Elias Dahdouh; Esther Viedma; Fernando Lázaro; Jesús Mingorance; Juan Carlos Galán; Julio García; Mª Dolores Folgueira; Natalia Stella; Rafael Cantón; Rafael Delgado; Raúl Recio; Sara González
EPI_ISL_417969, EPI_ISL_417972, EPI_ISL_417975, EPI_ISL_417978, EPI_ISL_530025, EPI_ISL_530027 to 530075, EPI_ISL_530077 to 530093, EPI_ISL_534330 to 534333			
see above	Hospital Universitario La Paz	Hospital Universitario La Paz	Elias Dahdouh; Esther Viedma; Fernando Lázaro; Jesús Mingorance; Juan Carlos Galán; Julio García; Ma Dolores Folgueira; María Rodríguez; Mª Dolores Folgueira; Natalia Stella; Rafael Cantón; Rafael Delgado; Raúl Recio; Sara González
EPI_ISL_831021 to 831027	Hospital Universitario La Paz (Madrid)	SeqCOVID-SPAIN consortium/IBV(CSIC)	Elias Dahdouh; Fernando Lázaro-Perona; Jesús Mingorance and SeqCOVID-SPAIN consortium; María Rodríguez-Tejedor
EPI_ISL_537809 to 537810	Hospital Universitario Marqués de Valdecilla (Santander), Servicio de Microbiología	SeqCOVID-SPAIN consortium/IBV(CSIC)	Daniel Pablo Marcos; Jesús Rodríguez Lozano; Jose Manuel Méndez Legaza; María Eliecer Cano García; María Siller Ruiz and SeqCOVID-SPAIN consortium; Mónica Gozalo Margüello
EPI_ISL_582100 to 582109, EPI_ISL_871894 to 871907, EPI_ISL_1007773 to 1007774, EPI_ISL_1007779, EPI_ISL_1007788, EPI_ISL_1007795, EPI_ISL_1007801 to 1007804, EPI_ISL_1007806 to 1007808, EPI_ISL_1007812, EPI_ISL_1007816 to 1007821, EPI_ISL_1007824 to 1007826, EPI_ISL_1007832, EPI_ISL_1008982, EPI_ISL_1008984 to 1008997, EPI_ISL_1008999 to 1009003, EPI_ISL_1009005 to 1009006, EPI_ISL_1009008 to 1009010			
see above	Hospital Universitario Marqués de Valdecilla - IDIVAL (Santander, Cantabria)	SeqCOVID-SPAIN consortium/IBV(CSIC)	Daniel Pablo Marcos; Jesús Rodríguez Rodríguez; Jose Manuel Méndez Legaza; María Eliecer Cano García; María Siller Ruiz and SeqCOVID-SPAIN consortium; Mónica Gozalo Margüello
EPI_ISL_539503 to 539508, EPI_ISL_539513 to 539519, EPI_ISL_578189, EPI_ISL_578192 to 578193	Hospital Universitario Miguel Servet	Instituto de Salud Carlos III	A. Monzón; A. Rezusta; F. Casas; I; I. Jiménez; Iglesias-Caballero; M. Camarero; M. Cuesta; M. González-Esguevillas; M. Molinero Calamita; M. Zaballos; P. Jiménez; R. Benito; S. Juliá; S. Pozo; S. Varona
EPI_ISL_691731	Hospital Universitario Puerta de Hierro	Instituto de Salud Carlos III	A. Monzón; F. Casas; I. Jiménez; I. Velasco, A.; Iglesias-Caballero; M. Camarero; M. Cuesta; M. González-Esguevillas; M. Pozo; M. Zaballos; P. Jiménez; S. Juliá; S. Molinero Calamita; S. Varona
EPI_ISL_452453 to 452454, EPI_ISL_452457, EPI_ISL_452459 to 452462, EPI_ISL_452464 to 452471, EPI_ISL_467064, EPI_ISL_467066, EPI_ISL_467068 to 467085			
see above	Hospital Universitario Puerta del Mar de Cádiz - INIBICA	SeqCOVID-SPAIN consortium/IBV(CSIC)	Fátima-Galán-Sánchez; Manuel Rodríguez-Iglesias and SeqCOVID-SPAIN consortium; Manuel Rodrí-guez-Iglesias and SeqCOVID-SPAIN consortium; Salud Rodríguez-Pallares
EPI_ISL_530094 to 530099	Hospital Universitario Ramón y Cajal	Hospital Universitario 12 de Octubre	Elias Dahdouh; Esther Viedma; Fernando Lázaro; Jesús Mingorance; Juan Carlos Galán; Julio García; Mª Dolores Folgueira; Natalia Stella; Rafael Cantón; Rafael Delgado; Raúl Recio; Sara González
EPI_ISL_417979 to 417981, EPI_ISL_530100 to 530118, EPI_ISL_534334	Hospital Universitario Ramón y Cajal	Hospital Universitario La Paz	Elias Dahdouh; Esther Viedma; Fernando Lázaro; Jesús Mingorance; Juan Carlos Galán; Julio García; Ma Dolores Folgueira; María Rodríguez; Mª Dolores Folgueira; Natalia Stella; Rafael Cantón; Rafael Delgado; Raúl Recio; Sara González
EPI_ISL_691729 to 691730	Hospital Universitario Severo Ochoa	Instituto de Salud Carlos III	A. Monzón; F. Casas; I. García, M.; I. Jiménez; Iglesias-Caballero; M. Camarero; M. Cuesta; M. González-Esguevillas; M. Pozo; M. Zaballos; P. Jiménez; S. Juliá; S. Molinero Calamita; S. Varona
EPI_ISL_862545, EPI_ISL_862549, EPI_ISL_954782 to 954783, EPI_ISL_954793, EPI_ISL_954799	Hospital Universitario Virgen de la Arrixaca	Instituto de Salud Carlos III	A. Monzón; F. Casas; I; I. Jiménez; I. Moreno, L.; Iglesias-Caballero; M. Camarero; M. Camarero; M. Cuesta; M. González-Esguevillas; M. Pozo; M. Zaballos; Moreno, A.; P. Jiménez; P. Zaballos; S. Cuesta; S. Juliá; S. Molinero Calamita; S. Pozo; S. Sandonís; S. Varona; V. Vázquez
EPI_ISL_419230	Hospital Universitario Virgen de las Nieves	Instituto de Salud Carlos III	Camarero, S.; Casas, I.; Cuesta, I.; González-Esguevillas, M.; Iglesias-Caballero, M.; Jiménez, M.; Jiménez, P.; Juliá, M.; Molinero Calamita, M.; Monzón, S.; Pozo, F.; Sanbonmatsu, S.; Varona, S.; Zaballos, A.
EPI_ISL_467059, EPI_ISL_467061 to 467063, EPI_ISL_474797, EPI_ISL_474832 to 474834, EPI_ISL_474836, EPI_ISL_474841 to 474843, EPI_ISL_474845, EPI_ISL_474850 to 474852, EPI_ISL_474854 to 474857, EPI_ISL_474859 to 474860, EPI_ISL_474862, EPI_ISL_474864, EPI_ISL_474867 to 474876, EPI_ISL_474879 to 474883, EPI_ISL_474886 to 474892, EPI_ISL_474894, EPI_ISL_474897 to 474898, EPI_ISL_474900, EPI_ISL_474908 to 474909, EPI_ISL_474920, EPI_ISL_474922 to 474923, EPI_ISL_474926 to 474928, EPI_ISL_474930, EPI_ISL_474934 to 474936, EPI_ISL_474939, EPI_ISL_474943 to 474944, EPI_ISL_474948 to 474950, EPI_ISL_510424, EPI_ISL_510427 to 510428, EPI_ISL_510431, EPI_ISL_510433 to 510437, EPI_ISL_510439 to 510441, EPI_ISL_510444 to 510452, EPI_ISL_510454 to 510456, EPI_ISL_510458 to 510460, EPI_ISL_1007748			
see above	Hospital Universitario Virgen de las Nieves de Granada-SAS	SeqCOVID-SPAIN consortium/IBV(CSIC)	Irene Pedrosa Corral; José M. Navarro-Marí and SeqCOVID-SPAIN consortium; Mercedes Pérez Ruiz; Sara Sanbonmatsu Gámez
EPI_ISL_468318, EPI_ISL_468321, EPI_ISL_547574	Hospital Universitario da USP	Instituto Adolfo Lutz, Interdiciplinary Procedures Center, Strategic Laboratory	Claudia Regina Gonçalves; Claudio Tavares Sacchi; Erica Valessa Ramos Gomes; Karoline Rodrigues Campos
EPI_ISL_471539	Hospital Universitario da USP Sao Paulo	Instituto Adolfo Lutz, Interdiciplinary Procedures Center, Strategic Laboratory	Claudia Regina Gonçalves; Claudio Tavares Sacchi; Erica Valessa Ramos Gomes
EPI_ISL_534314	Hospital Universitario da USP de SP	Instituto Adolfo Lutz, Interdiciplinary Procedures Center, Strategic Laboratory	Claudia Regina Gonçalves; Claudio Tavares Sacchi; Erica Valessa Ramos Gomes
EPI_ISL_419233, EPI_ISL_455325, EPI_ISL_539532	Hospital Universitario de Canarias	Instituto de Salud Carlos III	A. Monzón; B. Castro; Camarero, S.; Casas, I.; Castro, B.; Cuesta, I.; F. Casas; González-Esguevillas, M.; I; I. Jiménez; Iglesias-Caballero; Iglesias-Caballero, M.; Jiménez, M.; Jiménez, P.; Juliá, M.; M. Camarero; M. Cuesta; M. González-Esguevillas; M. Molinero Calamita; M. Zaballos; Molinero Calamita, M.; Monzón, S.; P. Jiménez; Pozo, F.; S. Juliá; S. Pozo; S. Varona; Varona, S.; Zaballos, A.
EPI_ISL_539520 to 539522, EPI_ISL_691686 to 691699, EPI_ISL_913020	Hospital Universitario de Ceuta	Instituto de Salud Carlos III	A. Monzón; F. Casas; G. Sánchez; I; I. Hijano, S.; I. Jiménez; I. López, J.; Iglesias-Caballero; J. López; M. Camarero; M. Cuesta; M. González-Esguevillas; M. Molinero Calamita; M. Pozo; M. Zaballos; P. Jiménez; P. Zaballos; S. Cuesta; S. Juliá; S. Molinero Calamita; S. Pozo; S. Sandonís; S. Varona; V. Vázquez
EPI_ISL_467086 to 467091, EPI_ISL_474910 to 474917, EPI_ISL_537693, EPI_ISL_537697, EPI_ISL_537700, EPI_ISL_537703, EPI_ISL_537706, EPI_ISL_537708 to 537709, EPI_ISL_537713, EPI_ISL_537715 to 537716, EPI_ISL_537718, EPI_ISL_537720 to 537721, EPI_ISL_537724, EPI_ISL_561370 to 561372, EPI_ISL_962940, EPI_ISL_1000987			
see above	Hospital Universitario de Gran Canaria Dr. Negrín	SeqCOVID-SPAIN consortium/IBV(CSIC)	Ana Bordes Benítez and SeqCOVID-SPAIN consortium; Francisco J. Chamizo López; M. Carmen Pérez González
EPI_ISL_539251 to 539258, EPI_ISL_539281 to 539283	Hospital Universitario de La Ribera (Alzira, València)	SeqCOVID-SPAIN consortium/IBV(CSIC)	Julia González and SeqCOVID-SPAIN consortium; Olalla Martínez Macias
EPI_ISL_977507	Hospital Valle del Nalon	Laboratorio de Virología HUCA	Abreu F; Alvarez-Arguelles ME; Boga JA; Castelló C; Costales I; Coto E; Gómez de Oña J; Martín-Rodríguez G; Melón S; Perez-Martínez Z; Rojo S;

			Sandoval M
EPI_ISL_977509	Hospital Valle del Nalón	Laboratorio de Virología HUCA	Abreu F; Alvarez-Arguelles ME; Boga JA; Castelló C; Costales I; Coto E; Gómez de Oña J; Martín-Rodríguez G; Melón S; Perez-Martínez Z; Rojo S; Sandoval M
EPI_ISL_693630 to 693657	Hospital Vila Franca de Xira	Instituto Nacional de Saude (INSA)	Borges et al
EPI_ISL_906073	Hospital Vila Lobos	Instituto Adolfo Lutz, Interdiciplinary Procedures Center, Strategic Laboratory	Claudia Regina Gonçalves; Claudio Tavares Sacchi; Erica Valessa Ramos Gomes; Karoline Rodrigues Campos
EPI_ISL_455315 to 455322, EPI_ISL_455324, EPI_ISL_539497, EPI_ISL_578187 to 578188	Hospital Virgen de las Nieves	Instituto de Salud Carlos III	A. Monzón; F. Casas; I; I. Jiménez; Iglesias-Caballero; J. Lepe; J.M Navarro; M. Camarero; M. Cuesta; M. González-Esguevillas; M. Molinero Calamita; M. Zaballos; P. Jiménez; S. Juliá; S. Pozo; S. Sanbonmatsu; S. Varona
EPI_ISL_455314	Hospital Virgen del Rocio	Instituto de Salud Carlos III	A. Monzón; F. Casas; I; I. Jiménez; Iglesias-Caballero; J. Lepe; M. Camarero; M. Cuesta; M. González-Esguevillas; M. Molinero Calamita; M. Zaballos; P. Jiménez; S. Juliá; S. Pozo; S. Varona
EPI_ISL_476373, EPI_ISL_476435, EPI_ISL_476439, EPI_ISL_476445 to 476446, EPI_ISL_476469, EPI_ISL_476490	Hospital da Clínicas da Faculdade de Medicina da Universidade de São Paulo	Instituto de Medicina Tropical da Univesidade de São Paulo	Camila Alves Maia da Silva; Carolina S. Lazar; Cecilia Salete Alencar; Darlan da Silva Candido; Erika Regina Manuli; Ester Sabino; Flavia Cristina da Silva Sales; Giula Magalhaes Ferreira; Jaqueline Goes de Jesus; Julien Theze; Mariana Severo Ramundo; Nuno Faria; Samples: Ingra Morales Claro; Sequencing: Ingra Morales Claro; Silvia F. Costa; Thais de Moura Coletti
EPI_ISL_534313	Hospital da Sta Casa de Sto Amaro	Instituto Adolfo Lutz, Interdiciplinary Procedures Center, Strategic Laboratory	Claudia Regina Gonçalves; Claudio Tavares Sacchi; Erica Valessa Ramos Gomes
EPI_ISL_722129	Hospital das Clínicas Universidade de São Paulo Medical School	Laboratório de Parasitologia Médica - Instituto de Medicina Tropical - Universidade de São Paulo	Brazil-UK Centre for Arbovirus Discovery Diagnosis Genomics and Epidemiology (CADDE) Genomic Network - Instituto de Medicina Tropical
EPI_ISL_977417	Hospital de Cabueñes	Laboratorio de Virología / Hospital Universitario Central de Asturias (HUCA)	Abreu F; Alvarez-Arguelles ME; Boga JA; Castelló C; Costales I; Coto E; Gómez de Oña J; Martín-Rodríguez G; Melón S; Perez-Martínez Z; Rojo S; Sandoval M
EPI_ISL_977501, EPI_ISL_1020124, EPI_ISL_1020145	Hospital de Cabueñes	Laboratorio de Virología HUCA	Abreu F; Alvarez-Arguelles ME; Boga JA; Castelló C; Costales I; Coto E; Gómez de Oña J; Martín-Rodríguez G; Melón S; Perez-Martínez Z; Rojo S; Sandoval M
EPI_ISL_693205	Hospital de Campanha Covid-19 Assis	Instituto Adolfo Lutz, Interdiciplinary Procedures Center, Strategic Laboratory	Claudia Regina Gonçalves; Claudio Tavares Sacchi; Erica Valessa Ramos Gomes; Karoline Rodrigues Campos
EPI_ISL_940612	Hospital de Campanha para Enfrentamento do Coronavirus - Goiania	Instituto Adolfo Lutz, Interdiciplinary Procedures Center, Strategic Laboratory	Claudia Regina Gonçalves; Claudio Tavares Sacchi; Erica Valessa Ramos Gomes; Karoline Rodrigues Campos
EPI_ISL_735396	Hospital de Camplanha COVID 19 SER	Instituto Adolfo Lutz, Interdiciplinary Procedures Center, Strategic Laboratory	Claudia Regina Gonçalves; Claudio Tavares Sacchi; Erica Valessa Ramos Gomes; Karoline Rodrigues Campos
EPI_ISL_882667	Hospital de Clinicas Caieiras	Instituto Adolfo Lutz, Interdiciplinary Procedures Center, Strategic Laboratory	Claudia Regina Gonçalves; Claudio Tavares Sacchi; Erica Valessa Ramos Gomes; Karoline Rodrigues Campos
EPI_ISL_455352 to 455354	Hospital de Cruces	Instituto de Salud Carlos III	A. Monzón; F. Casas; I; I. Jiménez; Iglesias-Caballero; M. Aranzamendi; M. Camarero; M. Cuesta; M. González-Esguevillas; M. Molinero Calamita; M. Zaballos; P. Jiménez; S. Juliá; S. Pozo; S. Varona
EPI_ISL_691685	Hospital de Leon	Instituto de Salud Carlos III	A. Monzón; F. Casas; I. Jiménez; I. Vidan, J.; Iglesias-Caballero; M. Camarero; M. Cuesta; M. González-Esguevillas; M. Pozo; M. Zaballos; P. Jiménez; S. Juliá; S. Molinero Calamita; S. Varona
EPI_ISL_691732	Hospital de Madrid	Instituto de Salud Carlos III	A. Monzón; F. Casas; I. Alhambra, A.; I. Jiménez; Iglesias-Caballero; M. Camarero; M. Cuesta; M. González-Esguevillas; M. Pozo; M. Zaballos; P. Jiménez; S. Juliá; S. Molinero Calamita; S. Varona
EPI_ISL_476563, EPI_ISL_476565, EPI_ISL_476567 to 476568, EPI_ISL_476571, EPI_ISL_476573, EPI_ISL_615121	Hospital de Pediatría "Prof. Dr. Juan P Garrahan"	Héritas	Andrea Mangano; Bianca Brun; Cristian Rohr; Dalmacio Pereyra; Fabian Fay; Maria Florencia Fernandez; Martin Vazquez; Mauricio Grisolia; Priscila Aldabe; Roberta Crespo
EPI_ISL_414577 to 414578	Hospital de Talca, Chile	Instituto de Salud Publica de Chile	Alejandra Acevedo; Andrés E. Castillo; Bárbara Parra; Carolina Tambley; Gabriel Leal; Gisselle Barra; Jaime Lagos; Javier Tognarelli; Jorge Fernández.; Loredana Arata; Patricia Bustos; Paz Tapia; Rodrigo Fasce; Soledad Ulloa; Winston Andrade
EPI_ISL_850508	Hospital de Tortosa Verge de la Cinta	Hospital Universitari Vall d'Hebron - Vall d'Hebron Institut de Recerca	Andrés Antón; Ariadna Rando; Carla Castillo; Cristina Andrés; Damir Garcia-Cehic; Josep F Abril; Josep Quer; Juliana Esperalba; Maria Carmen Martin; Maria Gema Codina; Maria Piñana; Tomàs Pumarola
EPI_ISL_510247 to 510253, EPI_ISL_510256 to 510266	Hospital de la Santa Creu i Sant Pau. Servicio de Microbiología	SeqCOVID-SPAIN consortium/IBV(CSIC)	Elisenda Miró and SeqCOVID-SPAIN consortium; Ferran Navarro; Núria Rabella
EPI_ISL_534319 to 534320	Hospital do Serv Pub ESTAFCO Morato de Oliveira	Instituto Adolfo Lutz, Interdiciplinary Procedures Center, Strategic Laboratory	Claudia Regina Gonçalves; Claudio Tavares Sacchi; Erica Valessa Ramos Gomes
EPI_ISL_693199	Hospital do Servidor Publico Estadual Francisco Morato de Oliveira	Instituto Adolfo Lutz, Interdiciplinary Procedures Center, Strategic Laboratory	Claudia Regina Gonçalves; Claudio Tavares Sacchi; Erica Valessa Ramos Gomes; Karoline Rodrigues Campos
EPI_ISL_471548, EPI_ISL_515565, EPI_ISL_523965, EPI_ISL_523972, EPI_ISL_523978, EPI_ISL_523982, EPI_ISL_524470			
see above	Hospital do Servidor Público Estadual Francisco Morato de Oliveira	Instituto Adolfo Lutz, Interdiciplinary Procedures Center, Strategic Laboratory	Claudia Regina Gonçalves; Claudio Tavares Sacchi; Erica Valessa Ramos Gomes
EPI_ISL_527861, EPI_ISL_940609	Hospital e Maternidade Celso Pierro	Instituto Adolfo Lutz, Interdiciplinary Procedures Center, Strategic Laboratory	01246-1301; 355 - Brazil; Av. Dr. Arnaldo; Cerqueira Cesar; Claudia Regina Gonçalves; Claudio Tavares Sacchi; Erica Valessa Ramos Gomes; Karoline Rodrigues Campos; São Paulo - SP
EPI_ISL_693200	Hospital e Maternidade Mairipora	Instituto Adolfo Lutz, Interdiciplinary Procedures Center, Strategic Laboratory	Claudia Regina Gonçalves; Claudio Tavares Sacchi; Erica Valessa Ramos Gomes; Karoline Rodrigues Campos
EPI_ISL_547577	Hospital e Maternidade Nossa Senhora das Graças	Instituto Adolfo Lutz, Interdiciplinary Procedures Center, Strategic Laboratory	Claudia Regina Gonçalves; Claudio Tavares Sacchi; Erica Valessa Ramos Gomes; Karoline Rodrigues Campos
EPI_ISL_693241, EPI_ISL_735425	Hospital e Maternidade Sao Lucas	Instituto Adolfo Lutz, Interdiciplinary Procedures Center, Strategic Laboratory	Claudia Regina Gonçalves; Claudio Tavares Sacchi; Erica Valessa Ramos Gomes; Karoline Rodrigues Campos
EPI_ISL_527865	Hospital e Maternidade São Cristóvão	Instituto Adolfo Lutz, Interdiciplinary Procedures Center, Strategic Laboratory	Claudia Regina Gonçalves; Claudio Tavares Sacchi; Erica Valessa Ramos Gomes
EPI_ISL_527868	Hospital e Maternidade do Braz	Instituto Adolfo Lutz, Interdiciplinary Procedures Center, Strategic Laboratory	Claudia Regina Gonçalves; Claudio Tavares Sacchi; Erica Valessa Ramos Gomes
EPI_ISL_534323	Hospital e Pronto Socorro Comunitario Vila Yolanda	Instituto Adolfo Lutz, Interdiciplinary Procedures Center, Strategic Laboratory	Claudia Regina Gonçalves; Claudio Tavares Sacchi; Erica Valessa Ramos Gomes
EPI_ISL_527864	Hospital e Pronto Socorro Comunitário Vila Iolanda	Instituto Adolfo Lutz, Interdiciplinary Procedures Center, Strategic Laboratory	Claudia Regina Gonçalves; Claudio Tavares Sacchi; Erica Valessa Ramos Gomes
EPI_ISL_693195, EPI_ISL_693230, EPI_ISL_693232, EPI_ISL_735412	Hospital e Pronto Socorro Portinari	Instituto Adolfo Lutz, Interdiciplinary Procedures Center, Strategic Laboratory	Claudia Regina Gonçalves; Claudio Tavares Sacchi; Erica Valessa Ramos Gomes; Karoline Rodrigues Campos
EPI_ISL_458024, EPI_ISL_498257,	Hospital for Tropical Diseases	COVID-19 Network Investigations (CONI) Alliance	Akanitt Jittmittraphap; Angkana Huang; Anthony R. Jones; Arporn Wangwiwatsin; Bhakbhoom Panthan; Chonticha Klungtong; Ekawat Pasomsub;

EPI_ISL_707709 to 707710			Elizabeth Batty; Insee Sensorn; Janjira Thaipadungpanit; Khajohn Joonsalak; Kingkan Rakmanee; Krittikorn Kumpornsin; Namfon Kotanan; Nantarat Chantawat; Pornsawan Leaungwutiwong; Stefan Fernandez; Thanat Chookajorn; Theerarat Kochakarn; Treewat Watthanachockchai; Viravarn Luvira; Wasun Chantratita; Weena Janwitthayanan; Wudtichai Manasatienkij
EPI_ISL_913025 to 913031, EPI_ISL_913036 to 913037	Hospital los Arcos	Instituto de Salud Carlos III	A. Monzón; F. Casas; I. Cámara, M.; I. Jiménez; Iglesias-Caballero; M. Camarero; P. Zaballos; S. Cuesta; S. Pozo; S. Sandonís; S. Varona; V. Vázquez
EPI_ISL_417486 to 417487, EPI_ISL_420147, EPI_ISL_471173, EPI_ISL_471176, EPI_ISL_493384, EPI_ISL_500775, EPI_ISL_500791 to 500792, EPI_ISL_500797, EPI_ISL_500799, EPI_ISL_549036 to 549037, EPI_ISL_549052, EPI_ISL_549167, EPI_ISL_590904 to 590907, EPI_ISL_590915, EPI_ISL_590936 to 590937, EPI_ISL_590984, EPI_ISL_635097, EPI_ISL_635121 to 635123, EPI_ISL_635138, EPI_ISL_635143, EPI_ISL_635161, EPI_ISL_635189, EPI_ISL_668394 to 668395, EPI_ISL_668427 to 668428, EPI_ISL_708055 to 708056, EPI_ISL_708122 to 708125, EPI_ISL_796664, EPI_ISL_813977, EPI_ISL_906831, EPI_ISL_1040152			
see above	Hospital of Southern Norway - Kristiansand, Department of Medical Microbiology	Norwegian Institute of Public Health, Department of Virology	Atiya R Ali; Engebretsen Serina Beate Atiya R Ali; Garcia Llorente Ignacio; Hilde Elshaug; Hilde Synnøve Vollan; Hilde Vollan; Kamilla Heddeland Instefjord; Karoline Bragstad; Kathrine Stene-Johansen; Marie Paulsen Madsen; Olav Hungnes; Rasmus Riis Kopperud
EPI_ISL_593915	Hospital, Amneville	National Reference Center for Viruses of Respiratory Infections, Institut Pasteur, Paris	Etienne Simon-Lorière; Fabiana Gambaro; Maud Vanpeene; Sylvie Behillil; Sylvie van der Werf; Vincent Enouf
EPI_ISL_593903	Hospital, Argenteuil	National Reference Center for Viruses of Respiratory Infections, Institut Pasteur, Paris	Etienne Simon-Lorière; Fabiana Gambaro; Maud Vanpeene; Sylvie Behillil; Sylvie van der Werf; Vincent Enouf
EPI_ISL_593910	Hospital, Boulogne-Billancourt	National Reference Center for Viruses of Respiratory Infections, Institut Pasteur, Paris	Etienne Simon-Lorière; Fabiana Gambaro; Maud Vanpeene; Sylvie Behillil; Sylvie van der Werf; Vincent Enouf
EPI_ISL_593921	Hospital, Fameck	National Reference Center for Viruses of Respiratory Infections, Institut Pasteur, Paris	Etienne Simon-Lorière; Fabiana Gambaro; Maud Vanpeene; Sylvie Behillil; Sylvie van der Werf; Vincent Enouf
EPI_ISL_593918	Hospital, Joeuf	National Reference Center for Viruses of Respiratory Infections, Institut Pasteur, Paris	Etienne Simon-Lorière; Fabiana Gambaro; Maud Vanpeene; Sylvie Behillil; Sylvie van der Werf; Vincent Enouf
EPI_ISL_593911	Hospital, Le Chesnay Cédex	National Reference Center for Viruses of Respiratory Infections, Institut Pasteur, Paris	Etienne Simon-Lorière; Fabiana Gambaro; Maud Vanpeene; Sylvie Behillil; Sylvie van der Werf; Vincent Enouf
EPI_ISL_593922 to 593923	Hospital, Maizières les Metz	National Reference Center for Viruses of Respiratory Infections, Institut Pasteur, Paris	Etienne Simon-Lorière; Fabiana Gambaro; Maud Vanpeene; Sylvie Behillil; Sylvie van der Werf; Vincent Enouf
EPI_ISL_593912	Hospital, Marange Silvange	National Reference Center for Viruses of Respiratory Infections, Institut Pasteur, Paris	Etienne Simon-Lorière; Fabiana Gambaro; Maud Vanpeene; Sylvie Behillil; Sylvie van der Werf; Vincent Enouf
EPI_ISL_593914, EPI_ISL_593920	Hospital, Metz	National Reference Center for Viruses of Respiratory Infections, Institut Pasteur, Paris	Etienne Simon-Lorière; Fabiana Gambaro; Maud Vanpeene; Sylvie Behillil; Sylvie van der Werf; Vincent Enouf
EPI_ISL_593917	Hospital, Montigny les Metz	National Reference Center for Viruses of Respiratory Infections, Institut Pasteur, Paris	Etienne Simon-Lorière; Fabiana Gambaro; Maud Vanpeene; Sylvie Behillil; Sylvie van der Werf; Vincent Enouf
EPI_ISL_593919	Hospital, Ottonville	National Reference Center for Viruses of Respiratory Infections, Institut Pasteur, Paris	Etienne Simon-Lorière; Fabiana Gambaro; Maud Vanpeene; Sylvie Behillil; Sylvie van der Werf; Vincent Enouf
EPI_ISL_593916	Hospital, Sarreguemines	National Reference Center for Viruses of Respiratory Infections, Institut Pasteur, Paris	Etienne Simon-Lorière; Fabiana Gambaro; Maud Vanpeene; Sylvie Behillil; Sylvie van der Werf; Vincent Enouf
EPI_ISL_593913	Hospital, Talange	National Reference Center for Viruses of Respiratory Infections, Institut Pasteur, Paris	Etienne Simon-Lorière; Fabiana Gambaro; Maud Vanpeene; Sylvie Behillil; Sylvie van der Werf; Vincent Enouf
EPI_ISL_979403	Houston Health Dept.	Houston Health Dept.	Adolpho Lara; Pamela Brown; Ryker Penn
EPI_ISL_434712 to 434718, EPI_ISL_434720 to 434722, EPI_ISL_434724 to 434732, EPI_ISL_434735, EPI_ISL_434737, EPI_ISL_434744 to 434746, EPI_ISL_434753, EPI_ISL_434755, EPI_ISL_434759 to 434760, EPI_ISL_434764, EPI_ISL_434766, EPI_ISL_434768, EPI_ISL_434772, EPI_ISL_434776 to 434777, EPI_ISL_434780 to 434781, EPI_ISL_434783 to 434785, EPI_ISL_434787 to 434788, EPI_ISL_434790 to 434792, EPI_ISL_434795, EPI_ISL_434797 to 434803, EPI_ISL_434805, EPI_ISL_434807 to 434808, EPI_ISL_434810 to 434813, EPI_ISL_434815, EPI_ISL_434821 to 434822, EPI_ISL_434824 to 434825, EPI_ISL_434827 to 434829, EPI_ISL_434831 to 434834, EPI_ISL_434837, EPI_ISL_434839 to 434841, EPI_ISL_434843 to 434847, EPI_ISL_434849 to 434861, EPI_ISL_434863 to 434868, EPI_ISL_434870 to 434871, EPI_ISL_434873, EPI_ISL_434875 to 434885, EPI_ISL_434887 to 434928, EPI_ISL_434930 to 434931, EPI_ISL_434933 to 434943, EPI_ISL_434945 to 434967, EPI_ISL_434969 to 435003, EPI_ISL_435005 to 435024, EPI_ISL_435028 to 435031, EPI_ISL_542487 to 542488, EPI_ISL_542491 to 542498, EPI_ISL_542500, EPI_ISL_542502 to 542524, EPI_ISL_542526 to 542553, EPI_ISL_542556 to 542558, EPI_ISL_542560 to 542562, EPI_ISL_542565, EPI_ISL_542567 to 542568, EPI_ISL_542570 to 542607, EPI_ISL_542609 to 542618, EPI_ISL_542620 to 542626, EPI_ISL_542628 to 542631, EPI_ISL_542634 to 542635, EPI_ISL_542637 to 542649, EPI_ISL_542651 to 542655, EPI_ISL_542657 to 542660, EPI_ISL_542663 to 542669, EPI_ISL_542671, EPI_ISL_542673 to 542685, EPI_ISL_542687 to 542696, EPI_ISL_542698 to 542704, EPI_ISL_542706 to 542709, EPI_ISL_542711 to 542731, EPI_ISL_542733 to 542736, EPI_ISL_542738 to 542740, EPI_ISL_542742 to 542744, EPI_ISL_542746, EPI_ISL_542748 to 542749, EPI_ISL_542751 to 542757, EPI_ISL_542759 to 542761, EPI_ISL_542763, EPI_ISL_542765 to 542766, EPI_ISL_542768 to 542769, EPI_ISL_542771 to 542773, EPI_ISL_542775 to 542780, EPI_ISL_542782, EPI_ISL_542784 to 542792, EPI_ISL_542794 to 542806, EPI_ISL_542808 to 542815, EPI_ISL_542817 to 542828, EPI_ISL_542830 to 542836, EPI_ISL_542838 to 542852, EPI_ISL_542854 to 542857, EPI_ISL_542859 to 542860, EPI_ISL_542862 to 542866, EPI_ISL_542868 to 542869, EPI_ISL_542871 to 542873, EPI_ISL_542875 to 542876, EPI_ISL_542878, EPI_ISL_542880 to 542883, EPI_ISL_542885 to 542902, EPI_ISL_542904 to 542911, EPI_ISL_542913 to 542918, EPI_ISL_542920 to 542927, EPI_ISL_542929 to 542931, EPI_ISL_542934, EPI_ISL_543007 to 543009, EPI_ISL_543011 to 543012, EPI_ISL_543014 to 543015, EPI_ISL_543017 to 543020, EPI_ISL_543023 to 543025, EPI_ISL_543027, EPI_ISL_543029 to 543031, EPI_ISL_543033 to 543037, EPI_ISL_543039 to 543045, EPI_ISL_543047, EPI_ISL_543049 to 543050, EPI_ISL_543052 to 543057, EPI_ISL_543060 to 543068, EPI_ISL_543070 to 543074, EPI_ISL_543076 to 543079, EPI_ISL_543081 to 543082, EPI_ISL_543084 to 543095, EPI_ISL_543097, EPI_ISL_543099 to 543102, EPI_ISL_543104, EPI_ISL_543106 to 543112, EPI_ISL_543114, EPI_ISL_543116 to 543120, EPI_ISL_543122 to 543124, EPI_ISL_543126, EPI_ISL_543130 to 543133, EPI_ISL_543135, EPI_ISL_543137 to 543138, EPI_ISL_543140 to 543150, EPI_ISL_543154 to 543161, EPI_ISL_543164 to 543166, EPI_ISL_543169 to 543171, EPI_ISL_543173, EPI_ISL_543176 to 543179, EPI_ISL_543181 to 543191, EPI_ISL_543193 to 543194, EPI_ISL_543196 to 543205, EPI_ISL_543207 to 543227, EPI_ISL_543229, EPI_ISL_543231 to 543235, EPI_ISL_543237, EPI_ISL_543239 to 543247, EPI_ISL_543250 to 543256, EPI_ISL_543259 to 543261, EPI_ISL_543263 to 543264, EPI_ISL_543266 to 543267, EPI_ISL_543269 to 543273, EPI_ISL_543275 to 543278, EPI_ISL_543280 to 543283, EPI_ISL_543285 to 543286, EPI_ISL_543288, EPI_ISL_543290 to 543294, EPI_ISL_543298 to 543301, EPI_ISL_543303 to 543306, EPI_ISL_543308, EPI_ISL_543310 to 543314, EPI_ISL_543316, EPI_ISL_543318 to 543329, EPI_ISL_543331 to 543332, EPI_ISL_543334 to 543336, EPI_ISL_543338, EPI_ISL_543341 to 543343, EPI_ISL_543345 to 543346, EPI_ISL_543348, EPI_ISL_543351, EPI_ISL_543353 to 543354, EPI_ISL_543356 to 543361, EPI_ISL_543363 to 543364, EPI_ISL_543367 to 543369, EPI_ISL_543371 to 543372, EPI_ISL_543374 to 543375, EPI_ISL_543377 to 543378, EPI_ISL_543381 to 543382, EPI_ISL_543386 to 543391, EPI_ISL_543394 to 543401, EPI_ISL_543404 to 543413, EPI_ISL_543415 to 543416, EPI_ISL_543419 to 543423, EPI_ISL_543425, EPI_ISL_543427, EPI_ISL_543429 to 543431, EPI_ISL_543433 to 543444, EPI_ISL_543446 to 543447, EPI_ISL_543450, EPI_ISL_543453 to 543456, EPI_ISL_543458 to 543461, EPI_ISL_543464 to 543467, EPI_ISL_543469 to 543471, EPI_ISL_543473 to 543479, EPI_ISL_543481, EPI_ISL_543483, EPI_ISL_543485 to 543491, EPI_ISL_543493 to 543495, EPI_ISL_543497 to 543498, EPI_ISL_543500, EPI_ISL_543502 to 543504, EPI_ISL_543506 to 543507, EPI_ISL_543509, EPI_ISL_543511 to 543515, EPI_ISL_543518 to 543521, EPI_ISL_543523 to 543525, EPI_ISL_543527 to 543530, EPI_ISL_543532 to 543538, EPI_ISL_543540 to 543541, EPI_ISL_543543 to 543547, EPI_ISL_543549 to 543558, EPI_ISL_543563, EPI_ISL_543565 to 543572, EPI_ISL_543574 to 543577, EPI_ISL_543579 to 543594, EPI_ISL_543596 to 543600, EPI_ISL_543602 to 543609, EPI_ISL_543611 to 543612, EPI_ISL_543615, EPI_ISL_543619 to 543621, EPI_ISL_543623, EPI_ISL_543625 to 543646, EPI_ISL_543650 to 543659, EPI_ISL_543662 to 543663, EPI_ISL_543665 to 543668, EPI_ISL_543670 to 543678, EPI_ISL_543680, EPI_ISL_543682 to 543684, EPI_ISL_543687 to 543689, EPI_ISL_543691 to 543712, EPI_ISL_543715, EPI_ISL_543717, EPI_ISL_543719 to 543740, EPI_ISL_543742 to 543744, EPI_ISL_543746, EPI_ISL_543748, EPI_ISL_543750 to 543751, EPI_ISL_543753, EPI_ISL_543756 to 543765, EPI_ISL_543767 to 543770, EPI_ISL_543772 to 543777, EPI_ISL_543781 to 543783, EPI_ISL_543785 to 543786, EPI_ISL_543788 to 543792, EPI_ISL_543794 to 543799, EPI_ISL_543801 to 543819, EPI_ISL_543821 to 543823, EPI_ISL_543825, EPI_ISL_543827 to 543830, EPI_ISL_543832, EPI_ISL_543834 to 543838, EPI_ISL_543840 to 543862, EPI_ISL_543864 to 543866, EPI_ISL_543868 to 543869, EPI_ISL_543871 to 543873, EPI_ISL_543876, EPI_ISL_543879 to 543883, EPI_ISL_543886, EPI_ISL_543888, EPI_ISL_543892 to 543895, EPI_ISL_543897 to 543899, EPI_ISL_543902 to 543904, EPI_ISL_543906, EPI_ISL_543908 to 543910, EPI_ISL_543914 to 543917, EPI_ISL_543919 to 543921, EPI_ISL_543923, EPI_ISL_543925 to 543927, EPI_ISL_543929 to 543933, EPI_ISL_543935 to 543936, EPI_ISL_543939 to 543940, EPI_ISL_543943 to 543944, EPI_ISL_543946 to 543948, EPI_ISL_543950, EPI_ISL_543952, EPI_ISL_543955 to 543957, EPI_ISL_543959, EPI_ISL_543961 to 543963, EPI_ISL_543966, EPI_ISL_543969 to 543970, EPI_ISL_543972 to 543974, EPI_ISL_543977 to 543979, EPI_ISL_543981 to 543985, EPI_ISL_543987, EPI_ISL_543989 to 543993, EPI_ISL_543997 to 544001, EPI_ISL_544003 to 544012, EPI_ISL_544014, EPI_ISL_544016 to 544024, EPI_ISL_544026 to 544028, EPI_ISL_544030, EPI_ISL_544032 to 544042, EPI_ISL_544044 to 544051, EPI_ISL_544053 to 544057, EPI_ISL_544059 to 544071, EPI_ISL_544074 to 544075, EPI_ISL_544077 to 544098, EPI_ISL_544100 to 544102, EPI_ISL_544105 to 544107, EPI_ISL_544109 to 544110, EPI_ISL_544112 to 544115, EPI_ISL_544117 to 544118, EPI_ISL_544120 to 544124, EPI_ISL_544126 to 544131, EPI_ISL_544133 to 544134, EPI_ISL_544137 to 544138, EPI_ISL_544140, EPI_ISL_544142 to 544148, EPI_ISL_544150 to 544151, EPI_ISL_544153 to 544156, EPI_ISL_544158 to 544167, EPI_ISL_544169 to 544172, EPI_ISL_544174 to 544176, EPI_ISL_544179 to 544180, EPI_ISL_544182, EPI_ISL_544184 to 544186, EPI_ISL_544188, EPI_ISL_544190 to 544192, EPI_ISL_544194 to 544197, EPI_ISL_544199 to 544200, EPI_ISL_544203, EPI_ISL_544206 to 544211, EPI_ISL_544215 to 544216, EPI_ISL_544218 to 544235, EPI_ISL_544238, EPI_ISL_544240, EPI_ISL_544242 to 544243, EPI_ISL_544245 to 544247, EPI_ISL_544249, EPI_ISL_544251, EPI_ISL_544254 to 544255, EPI_ISL_544257, EPI_ISL_544259, EPI_ISL_544261, EPI_ISL_544264 to 544265, EPI_ISL_544267 to 544268, EPI_ISL_544270 to 544271, EPI_ISL_544273, EPI_ISL_544275, EPI_ISL_544277 to 544279, EPI_ISL_544281 to 544286, EPI_ISL_544288, EPI_ISL_544290, EPI_ISL_544294, EPI_ISL_544296 to 544300, EPI_ISL_544302 to 544304, EPI_ISL_544306 to 544312, EPI_ISL_544314 to 544316, EPI_ISL_544318 to 544321, EPI_ISL_544323 to 544326, EPI_ISL_544329 to 544335, EPI_ISL_544337, EPI_ISL_544339, EPI_ISL_544342, EPI_ISL_544344 to 544346, EPI_ISL_544348 to 544349, EPI_ISL_544351 to 544360, EPI_ISL_544362 to 544374, EPI_ISL_544376 to 544377, EPI_ISL_544380 to 544381, EPI_ISL_544384, EPI_ISL_544386 to 544387, EPI_ISL_544389 to 544394, EPI_ISL_544396 to 544400, EPI_ISL_544402 to 544404, EPI_ISL_544406 to 544412, EPI_ISL_544414 to 544416, EPI_ISL_544421 to 544426, EPI_ISL_544428 to 544436, EPI_ISL_544439 to 544441, EPI_ISL_544443, EPI_ISL_544445 to 544447, EPI_ISL_544452, EPI_ISL_544454, EPI_ISL_544456 to 544462, EPI_ISL_544465 to 544471, EPI_ISL_544473 to 544476, EPI_ISL_544478 to 544484, EPI_ISL_544487, EPI_ISL_544489, EPI_ISL_544491 to 544493, EPI_ISL_544495 to 544498, EPI_ISL_544501 to 544502, EPI_ISL_544504 to 544508, EPI_ISL_544511 to 544515, EPI_ISL_544517 to 544524, EPI_ISL_544527 to 544530, EPI_ISL_544532 to 544536, EPI_ISL_544538, EPI_ISL_544541 to 544543, EPI_ISL_544545 to 544546, EPI_ISL_544548 to 544549, EPI_ISL_544551 to 544553, EPI_ISL_544556, EPI_ISL_544558, EPI_ISL_544560 to 544562, EPI_ISL_544565 to 544576, EPI_ISL_544578 to 544581, EPI_ISL_544583 to 544588, EPI_ISL_544593 to 544598, EPI_ISL_544601 to 544605, EPI_ISL_544607, EPI_ISL_544609, EPI_ISL_544611 to 544615, EPI_ISL_544617, EPI_ISL_544619 to 544620, EPI_ISL_544622 to 544624, EPI_ISL_544627, EPI_ISL_544629 to 544630, EPI_ISL_544633 to 544635, EPI_ISL_544639 to 544643, EPI_ISL_544645 to 544649, EPI_ISL_544651 to 544653, EPI_ISL_544656, EPI_ISL_544658 to 544662, EPI_ISL_544664 to 544670, EPI_ISL_544672 to 544673, EPI_ISL_544675 to 544677, EPI_ISL_544679 to 544684, EPI_ISL_544687 to 544688, EPI_ISL_544690 to 544703, EPI_ISL_544705 to 544709, EPI_ISL_544711 to 544728, EPI_ISL_544730, EPI_ISL_544733 to 544734, EPI_ISL_544737 to 544739, EPI_ISL_544741 to 544744, EPI_ISL_544747 to 544748, EPI_ISL_544752, EPI_ISL_544756 to 544757, EPI_ISL_544759 to 544760, EPI_ISL_544762 to 544768, EPI_ISL_544770, EPI_ISL_544772 to 544773, EPI_ISL_544775 to 544784, EPI_ISL_544788, EPI_ISL_544791 to 544792, EPI_ISL_544795 to 544800, EPI_ISL_544802 to 544803, EPI_ISL_544805 to 544807, EPI_ISL_544812 to 544813, EPI_ISL_544815 to 544816, EPI_ISL_544819 to 544826, EPI_ISL_544828, EPI_ISL_544830 to 544835, EPI_ISL_544838 to 544845, EPI_ISL_544847 to 544855, EPI_ISL_544857, EPI_ISL_544859 to 544860, EPI_ISL_544864, EPI_ISL_544866 to 544868, EPI_ISL_544870 to 544876, EPI_ISL_544878 to 544879, EPI_ISL_544882 to 544886, EPI_ISL_544888 to 544891, EPI_ISL_544893, EPI_ISL_544895 to 544896, EPI_ISL_544899 to 544900, EPI_ISL_544902 to 544907, EPI_ISL_544910 to 544911, EPI_ISL_544913, EPI_ISL_544915 to 544917, EPI_ISL_544921 to 544923, EPI_ISL_544925, EPI_ISL_544928 to 544930, EPI_ISL_544932 to 544937, EPI_ISL_544941, EPI_ISL_544944 to 544948, EPI_ISL_544950, EPI_ISL_544953 to 544954, EPI_ISL_545051 to 545053, EPI_ISL_545055 to 545066, EPI_ISL_545068 to 545073, EPI_ISL_545075 to 545076, EPI_ISL_545078 to 545085, EPI_ISL_545087 to 545088, EPI_ISL_545090, EPI_ISL_545093 to 545098, EPI_ISL_545100 to 545103, EPI_ISL_545106, EPI_ISL_545108 to 545123, EPI_ISL_545125 to 545140, EPI_ISL_545142 to 545147, EPI_ISL_545149 to 545150, EPI_ISL_545152 to 545153, EPI_ISL_545155 to 545166, EPI_ISL_545168 to 545173, EPI_ISL_545175 to 545189, EPI_ISL_545191, EPI_ISL_545193 to 545196, EPI_ISL_545198 to 545201, EPI_ISL_545204 to 545208, EPI_ISL_545210, EPI_ISL_545214, EPI_ISL_545216 to 545221, EPI_ISL_545223 to 545231, EPI_ISL_545233 to 545244, EPI_ISL_545246 to 545247, EPI_ISL_545249, EPI_ISL_545251, EPI_ISL_545255 to 545256, EPI_ISL_545258 to 545264, EPI_ISL_545266 to 545267, EPI_ISL_545269 to 545274, EPI_ISL_545276 to 545277, EPI_ISL_545279, EPI_ISL_545281 to 545285, EPI_ISL_545288 to 545291, EPI_ISL_545295 to 545300, EPI_ISL_545302 to 545303, EPI_ISL_545305 to 545308, EPI_ISL_545310 to 545317, EPI_ISL_545319 to 545322, EPI_ISL_545324 to 545340, EPI_ISL_545342 to 545349, EPI_ISL_545351 to 545364, EPI_ISL_545366 to 545369, EPI_ISL_545371 to 545378, EPI_ISL_545381, EPI_ISL_545383 to 545385, EPI_ISL_545387 to 545402, EPI_ISL_545405 to 545407, EPI_ISL_545409, EPI_ISL_545411 to 545414, EPI_ISL_545416, EPI_ISL_545419 to 545427, EPI_ISL_545429 to 545433, EPI_ISL_545435 to 545446,			

EPI_ISL_545448 to 545451, EPI_ISL_545453 to 545458, EPI_ISL_545461 to 545464, EPI_ISL_545466 to 545472, EPI_ISL_545475 to 545480, EPI_ISL_545482 to 545485, EPI_ISL_545487, EPI_ISL_545489 to 545492, EPI_ISL_545495 to 545498, EPI_ISL_545500 to 545501, EPI_ISL_545503 to 545507, EPI_ISL_545509 to 545516, EPI_ISL_545518 to 545524, EPI_ISL_545526 to 545533, EPI_ISL_545535, EPI_ISL_545537 to 545543, EPI_ISL_545545 to 545550, EPI_ISL_545552 to 545553, EPI_ISL_545555 to 545570, EPI_ISL_545573 to 545574, EPI_ISL_545582 to 545584, EPI_ISL_545586 to 545589, EPI_ISL_545591, EPI_ISL_545593, EPI_ISL_545595 to 545597, EPI_ISL_545599 to 545602, EPI_ISL_545606 to 545612, EPI_ISL_545614 to 545615, EPI_ISL_545619, EPI_ISL_545621, EPI_ISL_545624 to 545627, EPI_ISL_545630, EPI_ISL_545633 to 545634, EPI_ISL_545636 to 545640, EPI_ISL_545644 to 545647, EPI_ISL_545650 to 545651, EPI_ISL_545653, EPI_ISL_545656, EPI_ISL_545658 to 545664, EPI_ISL_545666 to 545689, EPI_ISL_545691 to 545698, EPI_ISL_545700 to 545704, EPI_ISL_545706 to 545710, EPI_ISL_545712, EPI_ISL_545714 to 545725, EPI_ISL_545727 to 545728, EPI_ISL_545730 to 545740, EPI_ISL_545744 to 545746, EPI_ISL_545748 to 545749, EPI_ISL_545751 to 545767, EPI_ISL_545769 to 545772, EPI_ISL_545776 to 545780, EPI_ISL_545782 to 545791, EPI_ISL_545793 to 545805, EPI_ISL_545807 to 545812, EPI_ISL_545814 to 545820, EPI_ISL_545822 to 545825, EPI_ISL_545827 to 545844, EPI_ISL_545846 to 545850, EPI_ISL_545852, EPI_ISL_545854 to 545863, EPI_ISL_545865, EPI_ISL_545867 to 545871, EPI_ISL_545873 to 545895, EPI_ISL_545897 to 545903, EPI_ISL_545905 to 545909, EPI_ISL_545911 to 545917, EPI_ISL_545919 to 545924, EPI_ISL_545926 to 545943, EPI_ISL_545945 to 545949, EPI_ISL_545951 to 545953, EPI_ISL_545958, EPI_ISL_545960 to 545961, EPI_ISL_545963 to 545965, EPI_ISL_545967 to 545968, EPI_ISL_545970, EPI_ISL_545973 to 545974, EPI_ISL_545976 to 545977, EPI_ISL_545981 to 545983, EPI_ISL_545985 to 545989, EPI_ISL_545991 to 545992, EPI_ISL_545994 to 545995, EPI_ISL_545998 to 546002, EPI_ISL_546004 to 546011, EPI_ISL_546013 to 546014, EPI_ISL_546017 to 546022, EPI_ISL_546024 to 546029, EPI_ISL_546031 to 546033, EPI_ISL_546035 to 546049, EPI_ISL_546051 to 546053, EPI_ISL_546055 to 546062, EPI_ISL_546064 to 546075, EPI_ISL_546078, EPI_ISL_546080 to 546084, EPI_ISL_546088, EPI_ISL_546090 to 546091, EPI_ISL_546093, EPI_ISL_546095 to 546103, EPI_ISL_546105 to 546106, EPI_ISL_546108 to 546111, EPI_ISL_546113 to 546114, EPI_ISL_546116 to 546117, EPI_ISL_546119 to 546121, EPI_ISL_546123, EPI_ISL_546127 to 546129, EPI_ISL_546135 to 546141, EPI_ISL_546144 to 546151, EPI_ISL_546154 to 546158, EPI_ISL_546160, EPI_ISL_546162, EPI_ISL_546164 to 546165, EPI_ISL_546168, EPI_ISL_546170 to 546173, EPI_ISL_546177 to 546181, EPI_ISL_546183 to 546185, EPI_ISL_546188, EPI_ISL_546190 to 546202, EPI_ISL_546206 to 546214, EPI_ISL_546216 to 546222, EPI_ISL_546224 to 546228, EPI_ISL_546231 to 546237, EPI_ISL_546239 to 546248, EPI_ISL_546250 to 546260, EPI_ISL_546262 to 546265, EPI_ISL_546267, EPI_ISL_546269, EPI_ISL_546272, EPI_ISL_546277 to 546280, EPI_ISL_546282, EPI_ISL_546284, EPI_ISL_546287 to 546288, EPI_ISL_546290 to 546297, EPI_ISL_546299 to 546303, EPI_ISL_546305 to 546306, EPI_ISL_546308 to 546320, EPI_ISL_546322 to 546324, EPI_ISL_546326 to 546329, EPI_ISL_546331 to 546340, EPI_ISL_546342 to 546343, EPI_ISL_546345, EPI_ISL_546348, EPI_ISL_546352 to 546353, EPI_ISL_546355 to 546357, EPI_ISL_546360 to 546363, EPI_ISL_546365 to 546366, EPI_ISL_546368, EPI_ISL_546370, EPI_ISL_546372 to 546373, EPI_ISL_546376, EPI_ISL_546378 to 546380, EPI_ISL_546384 to 546386, EPI_ISL_546390 to 546392, EPI_ISL_546396 to 546397, EPI_ISL_546399 to 546400, EPI_ISL_546403, EPI_ISL_546406, EPI_ISL_546408 to 546411, EPI_ISL_546413 to 546414, EPI_ISL_546419, EPI_ISL_546421, EPI_ISL_546423 to 546427, EPI_ISL_546430, EPI_ISL_546432 to 546434, EPI_ISL_546437 to 546440, EPI_ISL_546443 to 546444, EPI_ISL_546446 to 546451, EPI_ISL_546454, EPI_ISL_546456 to 546457, EPI_ISL_546463 to 546466, EPI_ISL_546468 to 546469, EPI_ISL_546471, EPI_ISL_546473, EPI_ISL_546475 to 546480, EPI_ISL_546482 to 546484, EPI_ISL_546486 to 546490, EPI_ISL_546492, EPI_ISL_546494 to 546495, EPI_ISL_546498 to 546500, EPI_ISL_546502 to 546507, EPI_ISL_546509 to 546512, EPI_ISL_546515, EPI_ISL_546517 to 546527, EPI_ISL_546529, EPI_ISL_546531, EPI_ISL_546533, EPI_ISL_546536 to 546539, EPI_ISL_546541 to 546542, EPI_ISL_546545 to 546547, EPI_ISL_546549 to 546552, EPI_ISL_546554 to 546558, EPI_ISL_546563 to 546572, EPI_ISL_546574 to 546575, EPI_ISL_546579, EPI_ISL_546581 to 546582, EPI_ISL_546584 to 546587, EPI_ISL_546592 to 546594, EPI_ISL_546598 to 546601, EPI_ISL_546603 to 546607, EPI_ISL_546610 to 546615, EPI_ISL_546617 to 546619, EPI_ISL_546621, EPI_ISL_546623 to 546624, EPI_ISL_546626 to 546627, EPI_ISL_546629, EPI_ISL_546631 to 546632, EPI_ISL_546636, EPI_ISL_546638 to 546642, EPI_ISL_546645, EPI_ISL_546647, EPI_ISL_546650 to 546653, EPI_ISL_546656 to 546660, EPI_ISL_546663 to 546664, EPI_ISL_546667 to 546672, EPI_ISL_546674 to 546681, EPI_ISL_546683 to 546684, EPI_ISL_546686 to 546694, EPI_ISL_546697, EPI_ISL_546699 to 546702, EPI_ISL_546704, EPI_ISL_546706 to 546707, EPI_ISL_546709 to 546715, EPI_ISL_546717 to 546719, EPI_ISL_546721 to 546726, EPI_ISL_546728 to 546731, EPI_ISL_546733, EPI_ISL_546736, EPI_ISL_546738 to 546747, EPI_ISL_546749 to 546750, EPI_ISL_546752 to 546760, EPI_ISL_546763 to 546766, EPI_ISL_546769 to 546771, EPI_ISL_546774 to 546783, EPI_ISL_546785 to 546787, EPI_ISL_546789 to 546790, EPI_ISL_546792 to 546795, EPI_ISL_546797, EPI_ISL_546800 to 546805, EPI_ISL_546807 to 546808, EPI_ISL_546810 to 546812, EPI_ISL_546815 to 546816, EPI_ISL_546819 to 546820, EPI_ISL_546824 to 546827, EPI_ISL_546829, EPI_ISL_546831, EPI_ISL_546833 to 546834, EPI_ISL_546837 to 546845, EPI_ISL_546847, EPI_ISL_546850, EPI_ISL_546852 to 546856, EPI_ISL_546860 to 546865, EPI_ISL_546868 to 546871, EPI_ISL_546873, EPI_ISL_546879 to 546882, EPI_ISL_546884 to 546890, EPI_ISL_546892 to 546900, EPI_ISL_546902, EPI_ISL_546904 to 546914, EPI_ISL_546916, EPI_ISL_546918, EPI_ISL_546920 to 546922, EPI_ISL_546925, EPI_ISL_546927, EPI_ISL_546931 to 546933, EPI_ISL_546937 to 546938, EPI_ISL_546940 to 546941, EPI_ISL_546945 to 546946, EPI_ISL_546948 to 546958, EPI_ISL_546961 to 546964, EPI_ISL_546966 to 546968, EPI_ISL_546973 to 546977, EPI_ISL_546979 to 546981, EPI_ISL_546983 to 546985, EPI_ISL_546988 to 546992, EPI_ISL_546995 to 546997, EPI_ISL_546999 to 547004, EPI_ISL_547007 to 547017, EPI_ISL_547020 to 547023, EPI_ISL_547025, EPI_ISL_547027 to 547029, EPI_ISL_547031, EPI_ISL_547034 to 547037, EPI_ISL_547040 to 547042, EPI_ISL_547044 to 547051, EPI_ISL_547054, EPI_ISL_547056 to 547057, EPI_ISL_547060, EPI_ISL_547062, EPI_ISL_547064, EPI_ISL_547066 to 547069, EPI_ISL_547071 to 547074, EPI_ISL_547076 to 547077, EPI_ISL_547079 to 547083, EPI_ISL_547085 to 547088, EPI_ISL_547091 to 547092, EPI_ISL_547094 to 547097, EPI_ISL_547099 to 547102, EPI_ISL_547104 to 547107, EPI_ISL_547109 to 547113, EPI_ISL_547118 to 547119, EPI_ISL_547121 to 547127, EPI_ISL_547129 to 547133, EPI_ISL_547135, EPI_ISL_547138 to 547140, EPI_ISL_547143 to 547144, EPI_ISL_547146, EPI_ISL_547148 to 547149, EPI_ISL_547152, EPI_ISL_547155 to 547156, EPI_ISL_547158 to 547163, EPI_ISL_547165 to 547166, EPI_ISL_547168 to 547178, EPI_ISL_547180 to 547188, EPI_ISL_547190 to 547191, EPI_ISL_547194 to 547195, EPI_ISL_547197, EPI_ISL_547200 to 547219, EPI_ISL_547221 to 547226, EPI_ISL_547228, EPI_ISL_547230, EPI_ISL_547233 to 547240, EPI_ISL_547244 to 547246, EPI_ISL_547248, EPI_ISL_547250 to 547256, EPI_ISL_547259, EPI_ISL_547261 to 547264, EPI_ISL_547266, EPI_ISL_547268 to 547274, EPI_ISL_547276 to 547280, EPI_ISL_547283, EPI_ISL_547286 to 547289, EPI_ISL_547291, EPI_ISL_547293 to 547304, EPI_ISL_547306, EPI_ISL_547309 to 547316, EPI_ISL_547318, EPI_ISL_547320 to 547325, EPI_ISL_547327 to 547328, EPI_ISL_547331, EPI_ISL_547333 to 547337, EPI_ISL_547339 to 547345, EPI_ISL_547347 to 547348, EPI_ISL_547350, EPI_ISL_547352 to 547354, EPI_ISL_547357, EPI_ISL_547359 to 547360, EPI_ISL_547362 to 547363, EPI_ISL_547365, EPI_ISL_547368 to 547369, EPI_ISL_547371 to 547376, EPI_ISL_547378 to 547384, EPI_ISL_547387 to 547388, EPI_ISL_547390, EPI_ISL_547394 to 547397, EPI_ISL_547399, EPI_ISL_547402 to 547405, EPI_ISL_547408 to 547414, EPI_ISL_547416, EPI_ISL_547418 to 547421, EPI_ISL_547423 to 547424, EPI_ISL_547426 to 547427, EPI_ISL_547431 to 547432, EPI_ISL_780858, EPI_ISL_781081, EPI_ISL_784196, EPI_ISL_784259, EPI_ISL_789211, EPI_ISL_789973, EPI_ISL_790302			
see above	Houston Methodist Hospital	Houston Methodist Hospital	Chia-Wei Chou; Concepcion C. Cantu; Daniel Boutz; David W. Bernard; Ghazaleh Eskandari; Hakon Jonsson; Heather Hendrickson; Hoang A. T. Nguyen; Hung-Che Kuo; Ilya J. Finkelstein; J. Hunter Long; James J. Davis; Jason S. McLellan; Jimmy Gollihar; Jule Goike; Kamyah Javanmardi; Kari Stefansson; Layne Pruitt; Marcus Nguyen; Matthew Ojeda Saavedra; Maulik Shukla; Muthiah Kumaraswami; Paul A. Christensen; Prasanti Yerramilli; Randall J. Olsen; S. Wesley Long; Sishir Subedi; and James M. Musser
EPI_ISL_450819, EPI_ISL_469059, EPI_ISL_469068	Hovas Askim Familjelakare och BVC	The Public Health Agency of Sweden	Anna Risberg; Anna Wendel; Anna-Malin Linde; Karin Tegmark-Wisell; Maria Lind Karlberg; Mattias Haukland; Mia Brytting; Olov Svartstrom; Oskar Karlsson Lindsjo; Petra Edquist; Reza Advani; Sandra Broddesson; Shamam Muradrasoli; Theresa Enkirch
EPI_ISL_452237, EPI_ISL_452240, EPI_ISL_469062, EPI_ISL_469064 to 469065, EPI_ISL_475513, EPI_ISL_475522 to 475523, EPI_ISL_475525, EPI_ISL_475567			
see above	Huddinge VC	The Public Health Agency of Sweden	Anders Johansson; Anna Risberg; Anna-Malin Linde; Karin Tegmark-Wisell; Maria Lind Karlberg; Mattias Haukland; Mia Brytting; Olov Svartstrom; Oskar Karlsson Lindsjo; Petra Edquist; Reza Advani; Sandra Broddesson; Shamam Muradrasoli; Theresa Enkirch
EPI_ISL_437512	Human Genetic Research Center, Kawsar Biotech Company	Human Genetic Research Center, Kawsar Biotech Company	Abbasalipour, M.; F. and Haghdoost, Y.; Hosseini; Kehsvar, Y.; Khosravi; M.A.; Sabeghi, S.; Zeinali, S.
EPI_ISL_645000 to 645109	Human Genome Variation Research Group, Malopolska Centre of Biotechnology	Human Genome Variation Research Group, Malopolska Centre of Biotechnology	Botwina, P.; Branicki, W.; Dabrowska, A.; Foremny, J.; Gromowski, T.; Klajmon, A.; Kopera, K.; Kowalski, M.; Labaj; Marszalek, K.; Owczarek, K.; P.P.; Pisarek, A.; Pospiech, E.; Pyrc, K.; Sanak, M.; Swadzba, J.; Szczepanski, A.
EPI_ISL_454636 to 454641, EPI_ISL_468438, EPI_ISL_468440, EPI_ISL_468446 to 468450, EPI_ISL_468452 to 468453, EPI_ISL_468456, EPI_ISL_468458 to 468459, EPI_ISL_468461, EPI_ISL_486281 to 486286, EPI_ISL_513840 to 513848, EPI_ISL_513850, EPI_ISL_513852 to 513855, EPI_ISL_583046 to 583051, EPI_ISL_583053 to 583057, EPI_ISL_583059 to 583066, EPI_ISL_583068 to 583076, EPI_ISL_583078 to 583103, EPI_ISL_583105 to 583129, EPI_ISL_583131 to 583136, EPI_ISL_583138, EPI_ISL_583140 to 583159, EPI_ISL_583161 to 583170, EPI_ISL_738742, EPI_ISL_738756, EPI_ISL_738759, EPI_ISL_738778 to 738779, EPI_ISL_738785 to 738786, EPI_ISL_738795, EPI_ISL_738801, EPI_ISL_738806 to 738807, EPI_ISL_738809 to 738810, EPI_ISL_738827, EPI_ISL_738832, EPI_ISL_738844, EPI_ISL_738849, EPI_ISL_738852, EPI_ISL_738854, EPI_ISL_738865, EPI_ISL_738875, EPI_ISL_738879, EPI_ISL_738902, EPI_ISL_738912 to 738913, EPI_ISL_738917 to 738918, EPI_ISL_738923, EPI_ISL_738929, EPI_ISL_738938, EPI_ISL_738947, EPI_ISL_738951, EPI_ISL_738953, EPI_ISL_738958, EPI_ISL_738961, EPI_ISL_738969, EPI_ISL_738980, EPI_ISL_738985, EPI_ISL_738988, EPI_ISL_738999 to 739001, EPI_ISL_739007, EPI_ISL_739011, EPI_ISL_739032, EPI_ISL_739043, EPI_ISL_739051 to 739052, EPI_ISL_739079, EPI_ISL_739086, EPI_ISL_739106, EPI_ISL_739112, EPI_ISL_739114, EPI_ISL_739123, EPI_ISL_739126, EPI_ISL_739131, EPI_ISL_739148, EPI_ISL_739155, EPI_ISL_739159, EPI_ISL_739165, EPI_ISL_739170, EPI_ISL_739177, EPI_ISL_739183, EPI_ISL_739194, EPI_ISL_739198, EPI_ISL_739210, EPI_ISL_739225, EPI_ISL_739228, EPI_ISL_739235, EPI_ISL_739240, EPI_ISL_739242, EPI_ISL_739250, EPI_ISL_739264, EPI_ISL_739269, EPI_ISL_739272, EPI_ISL_739274, EPI_ISL_739287, EPI_ISL_739292, EPI_ISL_739297, EPI_ISL_739299, EPI_ISL_739311, EPI_ISL_739313, EPI_ISL_739318, EPI_ISL_739330, EPI_ISL_739366 to 739367, EPI_ISL_739389, EPI_ISL_739403, EPI_ISL_739405, EPI_ISL_739411, EPI_ISL_739417 to 739418, EPI_ISL_739425, EPI_ISL_739430, EPI_ISL_739433, EPI_ISL_739436, EPI_ISL_739442, EPI_ISL_739447, EPI_ISL_739452, EPI_ISL_739461, EPI_ISL_739475, EPI_ISL_739489, EPI_ISL_739499, EPI_ISL_739525, EPI_ISL_739527, EPI_ISL_739530 to 739531, EPI_ISL_739533, EPI_ISL_739542, EPI_ISL_739553, EPI_ISL_739558, EPI_ISL_739571, EPI_ISL_739585, EPI_ISL_739608, EPI_ISL_739611, EPI_ISL_739621, EPI_ISL_739651			
see above	Humboldt County Public Health Laboratory	Chan-Zuckerberg Biohub	CZB Cliahub Consortium
EPI_ISL_526215 to 526238	Hungarian Defence Forces Military Medical Centre	National Laboratory of Virology, Szentágothai Research Centre	Balázs Somogyi; Bálint Eszenyi; Endre Gábor Tóth; Ferenc Jakab; Gábor Kemenesi
EPI_ISL_529961, EPI_ISL_759956	Husada Utama Hospital	Institute of Tropical Disease, Universitas Airlangga	Aldise M Nastri; Didi Dewanto; Gatot Soegiarto; Jezzy R Dewantari; Kazufumi Shimizu; Krisnoadi Rahardjo; Laksmi Wulandari; Maria I Lusida; Mitsuhiro Nishimura; Resti Yudhawati; Retno A Setyoningrum; Rima R Prasetya; Soetjipto; Yasuko Mori; Yohko K Shimizu
EPI_ISL_450814, EPI_ISL_452241, EPI_ISL_455103	Huslakarna Varmbadhuset Varberg	The Public Health Agency of Sweden	Anna Risberg; Anna-Malin Linde; Johanna Hilmersson; Karin Tegmark-Wisell; Maria Lind Karlberg; Mia Brytting; Olov Svartstrom; Oskar Karlsson Lindsjo; Theresa Enkirch
EPI_ISL_981533 to 981534	Hôpital Avicenne	Department of Virology, Henri Mondor University Hospital, Assistance Publique Hôpitaux de Paris, Université Paris-Est Créteil, INSERM U955	Alexandre Soulier; Christophe Rodriguez; Elisabeth Trawinski; Guillaume Gricourt; Jean-Michel Pawlotsky; Melissa N'Debi; Slim Fourati; Vanessa Demontant
EPI_ISL_450304 to 450305, EPI_ISL_450308, EPI_ISL_450310, EPI_ISL_450316, EPI_ISL_465681 to 465682, EPI_ISL_465694, EPI_ISL_465698 to 465699, EPI_ISL_535738, EPI_ISL_535796, EPI_ISL_535819, EPI_ISL_535851, EPI_ISL_535866, EPI_ISL_535869, EPI_ISL_535874, EPI_ISL_535888, EPI_ISL_535890 to 535891, EPI_ISL_535920 to 535923, EPI_ISL_535927, EPI_ISL_535944, EPI_ISL_535949 to 535950, EPI_ISL_535952 to 535953, EPI_ISL_535957, EPI_ISL_535962, EPI_ISL_535966, EPI_ISL_535972 to 535973, EPI_ISL_535983, EPI_ISL_536017 to 536018, EPI_ISL_536020, EPI_ISL_536024 to 536025, EPI_ISL_536075, EPI_ISL_536077, EPI_ISL_536082, EPI_ISL_536087 to 536088, EPI_ISL_536090, EPI_ISL_536094, EPI_ISL_536146 to 536148, EPI_ISL_536186 to 536187, EPI_ISL_536223, EPI_ISL_536225, EPI_ISL_536247, EPI_ISL_536268 to 536270, EPI_ISL_536279 to 536282, EPI_ISL_536284 to 536285, EPI_ISL_536290, EPI_ISL_536292 to 536294, EPI_ISL_536296 to 536298, EPI_ISL_536313 to 536314, EPI_ISL_536328, EPI_ISL_536331, EPI_ISL_536336, EPI_ISL_536338 to 536340, EPI_ISL_536342, EPI_ISL_536350, EPI_ISL_536372 to 536373			
see above	Hôpital Charles-LeMoyne	Laboratoire de santé publique du Québec	Guillaume Bourque; Ioannis Ragoussis; Jesse Shapiro; Mark Lathrop and Michel Roger; Mark Lathrop and Michel Roger on behalf of the CoVSeQ research group; Sandrine Moreira
EPI_ISL_535839	Hôpital Cité de la Santé	Laboratoire de santé publique du Québec	Guillaume Bourque; Ioannis Ragoussis; Jesse Shapiro; Mark Lathrop and Michel Roger; Sandrine Moreira
EPI_ISL_710532, EPI_ISL_710534, EPI_ISL_710537, EPI_ISL_710540 to 710541, EPI_ISL_710575, EPI_ISL_711057, EPI_ISL_712060			
see above	Hôpital Fattouma-Bourguiba de Monastir	Laboratoire des Procédés de Criblage Moléculaire et Cellulaire-Centre de Biotechnologie de Sfax	A. and Masmoudi, S.; Abdelmoulah, F.; Abid, N.; Ajili, F.; Aouni, M.; Ben Ayed, I.; Bensaid, M.; Chtourou, A.; Elargoubi, A.; Fki-berrajah, L.; Gaaloul, I.; Gargouri, S.; Hammami, A.; Kamoun, S.; Karray Hakim, H.; Kharat, N.; Mastouri, M.; Mhalla, S.; Nabli, A.; Rebai; Smeti, I.; Souissi, A.; Stambouli, N.; Turki, M.
EPI_ISL_535792	Hôpital Fleurimont	Laboratoire de santé publique du Québec	Guillaume Bourque; Ioannis Ragoussis; Jesse Shapiro; Mark Lathrop and Michel Roger; Sandrine Moreira
EPI_ISL_961580 to 961582, EPI_ISL_961671	Hôpital Georges L. Dumont	National Microbiology Laboratory (NML)	Anna Majer; Anneliese Landgraff; CanCOGeN's metadata curation team; Darian Hole; Elsie Grudeski; Gary Van Domselaar; Grace Seo; Guillaume Desnoyers; Jennifer Tanner; Kirsten Biggar; Madison Chapel; Morag Graham; Natalie Knox; Nathalie Bastien; Philip Mabon; Public Health Agency of Canada CanCOGeN team; Rhiannon Huzarewich; Richard Garceau; Russell Mandes; Shari Tyson; Timothy Booth; Yan Li
EPI_ISL_912669 to 912673, EPI_ISL_912677 to 912680, EPI_ISL_912682 to 912684, EPI_ISL_912830 to 912836, EPI_ISL_912908, EPI_ISL_912921 to 912922, EPI_ISL_982135 to 982141, EPI_ISL_982180			
see above	Hôpital Henri Mondor	Department of Virology, Henri Mondor University Hospital,	Alexandre Soulier; Christophe Rodriguez; Elisabeth Trawinski; Guillaume Gricourt; Jean-Michel Pawlotsky; Melissa N'Debi; Slim Fourati; Vanessa

		Assistance Publique Hôpitaux de Paris, Université Paris-Est Créteil, INSERM U955	Demontant
EPI_ISL_447654 to 447655, EPI_ISL_447657 to 447733	Hôpital Henri-Mondor Ap-Hp	Hôpital Henri-Mondor Ap-Hp	Canoui-Poitrine, F.; Clinical Study Group, C.; De Prost, N.; Demontant, V.; Deveaux, I.; Fourati, S.; Gricourt, G.; J.-M.; Lamoureux, C.; Lepeule, R.; Mekontso-Dessap, A.; N'debi, M.; P.-L.; Pawlotsky; Picard, O.; Rodriguez, C.; Schmitz, D.; Surgers, L.; Trawinski, E.; Woerther
EPI_ISL_535717, EPI_ISL_535797, EPI_ISL_535912, EPI_ISL_535915, EPI_ISL_536013, EPI_ISL_536029, EPI_ISL_536055, EPI_ISL_536180, EPI_ISL_536379			
see above	Hôpital Honoré-Mercier	Laboratoire de santé publique du Québec	Guillaume Bourque; Ioannis Ragoussis; Jesse Shapiro; Mark Lathrop and Michel Roger; Mark Lathrop and Michel Roger on behalf of the CoVSeQ research group; Sandrine Moreira
EPI_ISL_415650	Hôpital Instruction des Armées - BEGIN	National Reference Center for Viruses of Respiratory Infections, Institut Pasteur, Paris	Angela Brisebarre; Christine Bigaillon; Flora Donati Vincent Enouf; Marion Barbet; Maud Vanpeene; Méline Bizard; Mélnie Albert; Sylvie Behillil; Sylvie van der Werf
EPI_ISL_535745, EPI_ISL_535994	Hôpital Laurentien	Laboratoire de santé publique du Québec	Guillaume Bourque; Ioannis Ragoussis; Jesse Shapiro; Mark Lathrop and Michel Roger; Mark Lathrop and Michel Roger on behalf of the CoVSeQ research group; Sandrine Moreira
EPI_ISL_536108	Hôpital Le Royer	Laboratoire de santé publique du Québec	Guillaume Bourque; Ioannis Ragoussis; Jesse Shapiro; Mark Lathrop and Michel Roger on behalf of the CoVSeQ research group; Sandrine Moreira
EPI_ISL_535755, EPI_ISL_535817, EPI_ISL_535831	Hôpital Maisonneuve-Rosemont	Laboratoire de santé publique du Québec	Guillaume Bourque; Ioannis Ragoussis; Jesse Shapiro; Mark Lathrop and Michel Roger; Sandrine Moreira
EPI_ISL_1013210 to 1013213, EPI_ISL_1013235	Hôpital Necker	National Reference Center for Viruses of Respiratory Infections, Institut Pasteur, Paris	Angela Brisebarre; Camille Capel; Etienne Simon-Lorière; Fourgeaud Jacques; Marion Barbet; Maud Vanpeene; Méline Bizard; Sylvie Behillil; Sylvie van der Werf; Vincent Enouf
EPI_ISL_443295 to 443296, EPI_ISL_443298 to 443299	Hôpital Necker - Enfants - Malades Laboratoire de Virologie	National Reference Center for Viruses of Respiratory Infections, Institut Pasteur, Paris	Angela Brisebarre; Etienne Simon-Lorière; Flora Donati; Marianne Leruez-Ville; Marion Barbet; Maud Vanpeene; Mélanie Albert; Méline Bizard; Sylvie Behillil; Sylvie van der Werf; Vincent Enouf
EPI_ISL_535721, EPI_ISL_535795	Hôpital Notre-Dame	Laboratoire de santé publique du Québec	Guillaume Bourque; Ioannis Ragoussis; Jesse Shapiro; Mark Lathrop and Michel Roger; Mark Lathrop and Michel Roger on behalf of the CoVSeQ research group; Sandrine Moreira
EPI_ISL_900535	Hôpital Paris Saint-Joseph	CNR Virus des Infections Respiratoires - France SUD	Antonin Bal; Bruno Lina; Gregory Destras; Gwendolyne Burfin; Hadrien Règue; Laurence Josset; Martine Valette; Quentin Semanas; Sylvie Larrat
EPI_ISL_450309, EPI_ISL_450314 to 450315, EPI_ISL_450317 to 450318, EPI_ISL_465695 to 465696, EPI_ISL_465700, EPI_ISL_465702 to 465703, EPI_ISL_535718, EPI_ISL_535734, EPI_ISL_535763, EPI_ISL_535786, EPI_ISL_535818, EPI_ISL_535840, EPI_ISL_535857, EPI_ISL_535879, EPI_ISL_535909, EPI_ISL_535948, EPI_ISL_536021, EPI_ISL_536039, EPI_ISL_536041, EPI_ISL_536092, EPI_ISL_536096, EPI_ISL_536099, EPI_ISL_536173 to 536174, EPI_ISL_536176, EPI_ISL_536178 to 536179, EPI_ISL_536195 to 536196, EPI_ISL_536229, EPI_ISL_536234, EPI_ISL_536236, EPI_ISL_536276, EPI_ISL_536295, EPI_ISL_536300, EPI_ISL_536303, EPI_ISL_536305 to 536306, EPI_ISL_536308 to 536309, EPI_ISL_536322 to 536323, EPI_ISL_536332, EPI_ISL_536335, EPI_ISL_536347, EPI_ISL_536351 to 536352, EPI_ISL_536360, EPI_ISL_536375, EPI_ISL_536388 to 536389, EPI_ISL_536391, EPI_ISL_536395, EPI_ISL_536397			
see above	Hôpital Pierre-Boucher	Laboratoire de santé publique du Québec	Guillaume Bourque; Ioannis Ragoussis; Jesse Shapiro; Mark Lathrop and Michel Roger; Mark Lathrop and Michel Roger on behalf of the CoVSeQ research group; Sandrine Moreira
EPI_ISL_535731, EPI_ISL_535752, EPI_ISL_535762, EPI_ISL_535766, EPI_ISL_535770 to 535771, EPI_ISL_535850, EPI_ISL_535862 to 535863, EPI_ISL_535881 to 535883, EPI_ISL_535892 to 535893, EPI_ISL_535936, EPI_ISL_535938, EPI_ISL_535954, EPI_ISL_535956, EPI_ISL_536061 to 536062, EPI_ISL_536064 to 536068, EPI_ISL_536070, EPI_ISL_536083 to 536084, EPI_ISL_536086, EPI_ISL_536128, EPI_ISL_536131, EPI_ISL_536137, EPI_ISL_536139, EPI_ISL_536143 to 536144, EPI_ISL_536152, EPI_ISL_536157			
see above	Hôpital Pierre-Le Gardeur	Laboratoire de santé publique du Québec	Guillaume Bourque; Ioannis Ragoussis; Jesse Shapiro; Mark Lathrop and Michel Roger; Mark Lathrop and Michel Roger on behalf of the CoVSeQ research group; Sandrine Moreira
EPI_ISL_912719, EPI_ISL_912909 to 912920, EPI_ISL_912923	Hôpital Pitié-Salpêtrière	Department of Virology, Henri Mondor University Hospital, Assistance Publique Hôpitaux de Paris, Université Paris-Est Créteil, INSERM U955	Alexandre Soulier; Christophe Rodriguez; Elisabeth Trawinski; Guillaume Gricourt; Jean-Michel Pawlotsky; Melissa N'Debi; Slim Fourati; Vanessa Demontant
EPI_ISL_414631 to 414632	Hôpital Robert Debré Laboratoire de Virologie	National Reference Center for Viruses of Respiratory Infections, Institut Pasteur, Paris	Angela Brisebarre; Flora Donati Vincent Enouf; Laurent Andreoletti; Marion Barbet; Maud Vanpeene; Méline Bizard; Mélnie Albert; Sylvie Behillil; Sylvie van der Werf
EPI_ISL_535810, EPI_ISL_535899, EPI_ISL_535901, EPI_ISL_535969, EPI_ISL_536003, EPI_ISL_536010 to 536011, EPI_ISL_536056 to 536057, EPI_ISL_536252			
see above	Hôpital Sainte-Croix	Laboratoire de santé publique du Québec	Guillaume Bourque; Ioannis Ragoussis; Jesse Shapiro; Mark Lathrop and Michel Roger; Mark Lathrop and Michel Roger on behalf of the CoVSeQ research group; Sandrine Moreira
EPI_ISL_535963, EPI_ISL_536046	Hôpital d'Alma	Laboratoire de santé publique du Québec	Guillaume Bourque; Ioannis Ragoussis; Jesse Shapiro; Mark Lathrop and Michel Roger on behalf of the CoVSeQ research group; Sandrine Moreira
EPI_ISL_768829	Hôpital d'Instruction des Armées Sainte-Anne	CNR Virus des Infections Respiratoires - France SUD	Antonin Bal; Bruno Lina; Gregory Destras; Gwendolyne Burfin; Hadrien Règue; Laurence Josset; Martine Valette; Quentin Semanas
EPI_ISL_535829, EPI_ISL_535905, EPI_ISL_535964 to 535965, EPI_ISL_536107	Hôpital de Chicoutimi	Laboratoire de santé publique du Québec	Guillaume Bourque; Ioannis Ragoussis; Jesse Shapiro; Mark Lathrop and Michel Roger; Mark Lathrop and Michel Roger on behalf of the CoVSeQ research group; Sandrine Moreira
EPI_ISL_465691, EPI_ISL_535858, EPI_ISL_536266	Hôpital de Gatineau	Laboratoire de santé publique du Québec	Guillaume Bourque; Ioannis Ragoussis; Jesse Shapiro; Mark Lathrop and Michel Roger on behalf of the CoVSeQ research group; Sandrine Moreira
EPI_ISL_465686, EPI_ISL_535794, EPI_ISL_535809, EPI_ISL_535886 to 535887, EPI_ISL_535911, EPI_ISL_535989, EPI_ISL_535995, EPI_ISL_536051, EPI_ISL_536117, EPI_ISL_536122, EPI_ISL_536124, EPI_ISL_536127, EPI_ISL_536132, EPI_ISL_536168 to 536169, EPI_ISL_536207 to 536209, EPI_ISL_536230, EPI_ISL_536238 to 536239, EPI_ISL_536242, EPI_ISL_536244, EPI_ISL_536291, EPI_ISL_536312, EPI_ISL_536363, EPI_ISL_536367, EPI_ISL_536385 to 536387, EPI_ISL_536392 to 536393			
see above	Hôpital de Hull	Laboratoire de santé publique du Québec	Guillaume Bourque; Ioannis Ragoussis; Jesse Shapiro; Mark Lathrop and Michel Roger; Mark Lathrop and Michel Roger on behalf of the CoVSeQ research group; Sandrine Moreira
EPI_ISL_535744	Hôpital de Lasalle	Laboratoire de santé publique du Québec	Guillaume Bourque; Ioannis Ragoussis; Jesse Shapiro; Mark Lathrop and Michel Roger; Sandrine Moreira
EPI_ISL_465683 to 465684, EPI_ISL_535885, EPI_ISL_536111 to 536112, EPI_ISL_536289	Hôpital de Maria	Laboratoire de santé publique du Québec	Guillaume Bourque; Ioannis Ragoussis; Jesse Shapiro; Mark Lathrop and Michel Roger on behalf of the CoVSeQ research group; Sandrine Moreira
EPI_ISL_535945	Hôpital de Mont-Laurier	Laboratoire de santé publique du Québec	Guillaume Bourque; Ioannis Ragoussis; Jesse Shapiro; Mark Lathrop and Michel Roger; Sandrine Moreira
EPI_ISL_536171	Hôpital de Papineau	Laboratoire de santé publique du Québec	Guillaume Bourque; Ioannis Ragoussis; Jesse Shapiro; Mark Lathrop and Michel Roger on behalf of the CoVSeQ research group; Sandrine Moreira
EPI_ISL_535985 to 535987	Hôpital de Rouyn-Noranda	Laboratoire de santé publique du Québec	Guillaume Bourque; Ioannis Ragoussis; Jesse Shapiro; Mark Lathrop and Michel Roger on behalf of the CoVSeQ research group; Sandrine Moreira
EPI_ISL_535793, EPI_ISL_535865, EPI_ISL_535889, EPI_ISL_535926, EPI_ISL_535929 to 535930, EPI_ISL_535941 to 535943, EPI_ISL_535947, EPI_ISL_535982, EPI_ISL_536008, EPI_ISL_536049, EPI_ISL_536058, EPI_ISL_536069, EPI_ISL_536071 to 536072			
see above	Hôpital de Saint-Eustache	Laboratoire de santé publique du Québec	Guillaume Bourque; Ioannis Ragoussis; Jesse Shapiro; Mark Lathrop and Michel Roger; Mark Lathrop and Michel Roger on behalf of the CoVSeQ research group; Sandrine Moreira
EPI_ISL_535716, EPI_ISL_535723, EPI_ISL_535728	Hôpital de Verdun	Laboratoire de santé publique du Québec	Guillaume Bourque; Ioannis Ragoussis; Jesse Shapiro; Mark Lathrop and Michel Roger; Mark Lathrop and Michel Roger on behalf of the CoVSeQ research group; Sandrine Moreira
EPI_ISL_535739	Hôpital de l'Enfant-Jésus	Laboratoire de santé publique du Québec	Guillaume Bourque; Ioannis Ragoussis; Jesse Shapiro; Mark Lathrop and Michel Roger; Sandrine Moreira
EPI_ISL_535914, EPI_ISL_535937, EPI_ISL_535971, EPI_ISL_535974 to 535975, EPI_ISL_535978, EPI_ISL_535996 to 535999, EPI_ISL_536001, EPI_ISL_536030, EPI_ISL_536032 to 536033, EPI_ISL_536037, EPI_ISL_536246, EPI_ISL_536248 to 536251			
see above	Hôpital du Centre-de-la-Mauricie	Laboratoire de santé publique du Québec	Guillaume Bourque; Ioannis Ragoussis; Jesse Shapiro; Mark Lathrop and Michel Roger; Mark Lathrop and Michel Roger on behalf of the CoVSeQ research group; Sandrine Moreira
EPI_ISL_535812	Hôpital du Sacré-Coeur de Montréal	Laboratoire de santé publique du Québec	Guillaume Bourque; Ioannis Ragoussis; Jesse Shapiro; Mark Lathrop and Michel Roger on behalf of the CoVSeQ research group; Sandrine Moreira
EPI_ISL_450307, EPI_ISL_450311 to 450313, EPI_ISL_465679 to 465680, EPI_ISL_465693, EPI_ISL_465701, EPI_ISL_535868, EPI_ISL_535913, EPI_ISL_535917 to 535919, EPI_ISL_535988, EPI_ISL_536026, EPI_ISL_536095, EPI_ISL_536097 to 536098, EPI_ISL_536102, EPI_ISL_536105, EPI_ISL_536163, EPI_ISL_536198, EPI_ISL_536200 to 536202, EPI_ISL_536228, EPI_ISL_536257, EPI_ISL_536273 to 536274, EPI_ISL_536319, EPI_ISL_536349, EPI_ISL_536354 to 536359, EPI_ISL_536362, EPI_ISL_536390			
see above	Hôpital du Suroît	Laboratoire de santé publique du Québec	Guillaume Bourque; Ioannis Ragoussis; Jesse Shapiro; Mark Lathrop and Michel Roger; Mark Lathrop and Michel Roger on behalf of the CoVSeQ research group; Sandrine Moreira

EPI_ISL_535991, EPI_ISL_536113 to 536114	Hôpital et Centre d'Hébergement de Sept-Iles	Laboratoire de santé publique du Québec	Guillaume Bourque; Ioannis Ragoussis; Jesse Shapiro; Mark Lathrop and Michel Roger on behalf of the CoVSeQ research group; Sandrine Moreira
EPI_ISL_535719 to 535720, EPI_ISL_535756 to 535757, EPI_ISL_535769	Hôpital général Juif	Laboratoire de santé publique du Québec	Guillaume Bourque; Ioannis Ragoussis; Jesse Shapiro; Mark Lathrop and Michel Roger; Mark Lathrop and Michel Roger on behalf of the CoVSeQ research group; Sandrine Moreira
EPI_ISL_535875	Hôpital général du Lakeshore	Laboratoire de santé publique du Québec	Guillaume Bourque; Ioannis Ragoussis; Jesse Shapiro; Mark Lathrop and Michel Roger on behalf of the CoVSeQ research group; Sandrine Moreira
EPI_ISL_536205	Hôpital régional de Rimouski	Laboratoire de santé publique du Québec	Guillaume Bourque; Ioannis Ragoussis; Jesse Shapiro; Mark Lathrop and Michel Roger on behalf of the CoVSeQ research group; Sandrine Moreira
EPI_ISL_535791, EPI_ISL_535798, EPI_ISL_535838, EPI_ISL_536022	Hôpital régional de Saint-Jérôme	Laboratoire de santé publique du Québec	Guillaume Bourque; Ioannis Ragoussis; Jesse Shapiro; Mark Lathrop and Michel Roger; Mark Lathrop and Michel Roger on behalf of the CoVSeQ research group; Sandrine Moreira
EPI_ISL_536101	Hôpital, CLSC et Centre d'Hébergement de Roberval	Laboratoire de santé publique du Québec	Guillaume Bourque; Ioannis Ragoussis; Jesse Shapiro; Mark Lathrop and Michel Roger on behalf of the CoVSeQ research group; Sandrine Moreira
EPI_ISL_535725, EPI_ISL_535750 to 535751, EPI_ISL_535758 to 535759, EPI_ISL_535820 to 535821, EPI_ISL_535833 to 535836	Hôtel-Dieu de Lévis	Laboratoire de santé publique du Québec	Guillaume Bourque; Ioannis Ragoussis; Jesse Shapiro; Mark Lathrop and Michel Roger; Mark Lathrop and Michel Roger on behalf of the CoVSeQ research group; Sandrine Moreira
EPI_ISL_535903 to 535904, EPI_ISL_535990, EPI_ISL_535992, EPI_ISL_536121	Hôtel-Dieu de Sorel	Laboratoire de santé publique du Québec	Guillaume Bourque; Ioannis Ragoussis; Jesse Shapiro; Mark Lathrop and Michel Roger; Mark Lathrop and Michel Roger on behalf of the CoVSeQ research group; Sandrine Moreira
EPI_ISL_649785, EPI_ISL_649938 to 649939, EPI_ISL_653763 to 653783, EPI_ISL_653787 to 653812, EPI_ISL_653814 to 653823, EPI_ISL_722851 to 722854, EPI_ISL_722859 to 722871, EPI_ISL_722895, EPI_ISL_722898, EPI_ISL_722900			
see above	I.R.C.C.S. "S. De Bellis" - Ente Ospedaliero	Istituto Zooprofilattico Sperimentale della Puglia e della Basilicata	Bianco A.; Capozzi L.; Cipolletta D.; Del Sambro L.; Galante D.; Lippolis A.; Manzulli V; Notarnicola M.; Pace L.; Parisi A.; Rondinone V.
EPI_ISL_751565, EPI_ISL_751666, EPI_ISL_751687, EPI_ISL_751692, EPI_ISL_751721, EPI_ISL_751742, EPI_ISL_751779, EPI_ISL_751783			
see above	IA State Hygienic Laboratory	Genomics and Discovery, Respiratory Viruses Branch, Division of Viral Diseases, Centers for Disease Control and Prevention	Anna Montmayeur; Anna Uehara; Clinton R. Paden; Haibin Wang; Jing Zhang; Justin Lee; Krista Queen; Mili Sheth; Peter W. Cook; Rachel Marine; Suxiang Tong; Yan Li; Ying Tao
EPI_ISL_424865, EPI_ISL_424895 to 424900, EPI_ISL_447842, EPI_ISL_647990 to 648001	IA State Hygienic Laboratory	Pathogen Discovery, Respiratory Viruses Branch, Division of Viral Diseases, Centers for Disease Control and Prevention	Alison S. Laufer Halpin; Anna Uehara; Brian Lynch; Christopher A. Elkins; Clinton R. Paden; Haibin Wang; Jasmine Padilla; Jing Zhang; Justin Lee; Krista Queen; Mary S. Keckler; Rachel Marine; Suxiang Tong; Yan Li; Ying Tao
EPI_ISL_452790 to 452795	ICAR-National Institute of High Security Animal Diseases	ICAR-National Institute of High Security Animal Diseases	Anamika Mishra; Ashutosh Aasdev; Ashwin Ashok Raut; Atul Kumar Pateriya; Chakradhar Tosh; Chandan Kumar Dubey; Dipesh Kumar Nayak; Fateh Singh; G Venkatesh; Harshad Murugkar; Katherukamem Rajukumar; Manoj Kumar; Meghna Tripathi; Naveen Kumar; Niranjan Mishra; Pradeep Gandhale; Pushpendra Namdeo; Pushpendra Singh; Richa Sood; Rupal Singh; Sandeep Bhatia; Sandeep Kumar Jhade; Senmannan Kalaiyarasu; Sethil Kumar D; Shanmugasundaram Nagarajan; Shashi Bhushan Sudhakar; Suman Kumari Shah; Suman Mishra; Sushil Tripathi; Vijendra Pal Singh; Vishnupriya Patil
EPI_ISL_1007617, EPI_ISL_1007629 to 1007630	ICH-SION	Laboratory of genomics and metagenomics, Institute of Microbiology, University Hospital Centre and University of Lausanne, Switzerland	Claire Bertelli; Damien Jacot; Gilbert Greub; Sébastien Aeby; Trestan Pillonel
EPI_ISL_430464 to 430468, EPI_ISL_437539, EPI_ISL_455640 to 455641, EPI_ISL_455643 to 455676, EPI_ISL_455678 to 455679, EPI_ISL_508444 to 508454, EPI_ISL_508456 to 508457, EPI_ISL_508460, EPI_ISL_508463 to 508465, EPI_ISL_508468 to 508469, EPI_ISL_508471, EPI_ISL_508475, EPI_ISL_508481 to 508483, EPI_ISL_508487			
see above	ICMR-National Institute of Cholera and Enteric Diseases	National Institute of Biomedical Genomics	Ananya Chatterjee; Arindam Maitra; Hasina Banu; Mamta Chawla Sarkar; Saumitra Das; Shanta Dutta; Sreedhar Chinnaswamy
EPI_ISL_511899	ICMR-National Institute of Cholera and Enteric Diseases	National Institute of Biomedical Genomics - DBT's PAN-INDIA 1000 SARS--CoV-2 RNA Genome Sequencing Consortium	Ananya Chatterjee; Arindam Maitra; Hasina Banu; Mamta Chawla Sarkar; Saumitra Das; Shanta Dutta; Sreedhar Chinnaswamy
EPI_ISL_751567, EPI_ISL_751590, EPI_ISL_751612, EPI_ISL_751614, EPI_ISL_751618, EPI_ISL_751719, EPI_ISL_751748, EPI_ISL_751750, EPI_ISL_751780, EPI_ISL_751782, EPI_ISL_751789			
see above	ID Bureau of Laboratories	Genomics and Discovery, Respiratory Viruses Branch, Division of Viral Diseases, Centers for Disease Control and Prevention	Anna Montmayeur; Anna Uehara; Clinton R. Paden; Haibin Wang; Jing Zhang; Justin Lee; Krista Queen; Mili Sheth; Peter W. Cook; Rachel Marine; Suxiang Tong; Yan Li; Ying Tao
EPI_ISL_539491, EPI_ISL_560319	IDSP unit, Dehradun	CSIR-Institute of Microbial Technology	Amandeep Kaur; Anu Singh; Ashwani Kumar; Debarghya Ghose; Dipak Dutta; Harsh Goar; Kanika Bansal; Navin Baid; Poushali Chakraborty; Prabhu B. Patil; Rajesh Kumar Mishra; Sanjeet Kumar; Sanjeev Khosla
EPI_ISL_569614	IHS Fort Yates Hospital	South Dakota Public Health Laboratory	Jacob Garfin; Matt Plumb; Xiong Wang; and Chris Carlson
EPI_ISL_404253, EPI_ISL_410045	IL Department of Public Health Chicago Laboratory	Pathogen Discovery, Respiratory Viruses Branch, Division of Viral Diseases, Centers for Dieases Control and Prevention	Anna Uehara; Brett L. Whitaker; Brian Lynch; Clinton R. Paden; Janna' R. Murray; Jing Zhang; Krista Queen; Lijuan Wang; Senthil Kumar K. Sakthivel; Shifaq Kamili; Stephen Lindstrom; Susan I. Gerber; Suxiang Tong; Xiaoyan Lu; Yan Li; Ying Tao
EPI_ISL_424850 to 424851, EPI_ISL_452134, EPI_ISL_914025	IL Department of Public Health Chicago Laboratory	Pathogen Discovery, Respiratory Viruses Branch, Division of Viral Diseases, Centers for Disease Control and Prevention	Alison S. Laufer Halpin; Anna Montmayeur; Anna Uehara; Christopher A. Elkins; Clinton R. Paden; Haibin Wang; Jing Zhang; Krista Queen; Mary S. Keckler; Peter Cook; Rachel Marine; Suxiang Tong; Yan Li; Ying Tao; Zachary Weiner
EPI_ISL_914030, EPI_ISL_914033 to 914034	IL Department of Public Health Springfield Laboratory	Pathogen Discovery, Respiratory Viruses Branch, Division of Viral Diseases, Centers for Disease Control and Prevention	Anna Uehara; Clinton R. Paden; Haibin Wang; Jing Zhang; Krista Queen; Peter Cook; Suxiang Tong; Yan Li; Ying Tao
EPI_ISL_913991, EPI_ISL_913996, EPI_ISL_955291, EPI_ISL_955309	IL Dept. of Public Health Springfield Laboratory	Pathogen Discovery, Respiratory Viruses Branch, Division of Viral Diseases, Centers for Disease Control and Prevention	Anna Uehara; Clinton R. Paden; Haibin Wang; Jing Zhang; Krista Queen; Peter Cook; Suxiang Tong; Yan Li; Ying Tao
EPI_ISL_914035	IN State Department of Health Laboratory Service	Pathogen Discovery, Respiratory Viruses Branch, Division of Viral Diseases, Centers for Disease Control and Prevention	Anna Uehara; Clinton R. Paden; Haibin Wang; Jing Zhang; Krista Queen; Peter Cook; Suxiang Tong; Yan Li; Ying Tao
EPI_ISL_751579, EPI_ISL_751599 to 751600, EPI_ISL_751626, EPI_ISL_751632, EPI_ISL_751672, EPI_ISL_751676, EPI_ISL_751727, EPI_ISL_751768, EPI_ISL_751770			
see above	IN State Department of Health Laboratory Services	Genomics and Discovery, Respiratory Viruses Branch, Division of Viral Diseases, Centers for Disease Control and Prevention	Anna Montmayeur; Anna Uehara; Clinton R. Paden; Haibin Wang; Jing Zhang; Justin Lee; Krista Queen; Mili Sheth; Peter W. Cook; Rachel Marine; Suxiang Tong; Yan Li; Ying Tao
EPI_ISL_424866, EPI_ISL_452131, EPI_ISL_527666, EPI_ISL_540431	IN State Department of Health Laboratory Services	Pathogen Discovery, Respiratory Viruses Branch, Division of Viral Diseases, Centers for Disease Control and Prevention	Alison S. Laufer Halpin; Anna Montmayeur; Anna Uehara; Brian Lynch; Christopher A. Elkins; Clinton R. Paden; Haibin Wang; Jing Zhang; Krista Queen; Mary S. Keckler; Rachel Marine; Suxiang Tong; Yan Li; Ying Tao; Zachary Weiner
EPI_ISL_648209 to 648211, EPI_ISL_648213 to 648214, EPI_ISL_648216, EPI_ISL_648218, EPI_ISL_648677	INBIRS-UBA	Laboratorio Mixto de Biotecnología Acuática (LMBA)	Adriana Giri; Agustina Cerri; Ana Cavatorta; Ana Paletta; Diego Chouhy; Elisa Bolatti; Elizabeth Tapia; Federico Remes Lenicov; Flavio Spetale; Gastón Viarengo; Ignacio García Labari; Javier Murillo; Joaquín Ezpeleta; Julian Acosta; Laura Angelone; Leandro Ciappina; María Re; Pablo Casal; Pilar Bulacio; Silvana Spinelli; Silvia Arranz; Sofía Lavista Llanos; Vanina Villanova; Victoria Posner
EPI_ISL_419255, EPI_ISL_493328 to 493331, EPI_ISL_590693 to 590698, EPI_ISL_603137 to 603187, EPI_ISL_609989 to 609999, EPI_ISL_882919 to 882920, EPI_ISL_941338, EPI_ISL_1034280, EPI_ISL_1039782			
see above	INMI Lazzaro Spallanzani IRCCS	INMI Lazzaro Spallanzani IRCCS	A Di Caro; Alessandra D'Abramo; Annunziata Tamburro; Antonino Di Caro; B Bartolini; Barbara Bartolini; Beatrice Valli; C.E.M Gruber; CEM Gruber; Cesare E. M. Gruber; Cesare E.M. Gruber; Concetta Castilletti; Daniele Castiglia; Daniele Lapa; E Giombini; Eleonora Lalle; Emanuela Giombini; Emanuele Nicastri; F Messina; Fabrizio Carletti; Francesca Colavita; Francesco Messina; Francesco Vairo; Fulvia Pimpinelli; Giandomenico Russo; Giuseppe Ippolito.; Giuseppina Cappiello; M Rueca; MR Capobianchi; Marcello Meledandri; Maria Letizia Schiavone; Maria R. Capobianchi; Mariarosa Gaudio; Martina Rueca; O Butera; Ornella Butera; Patrizia Massarelli; Simone Lanini; Stefania Madonna
EPI_ISL_410545 to 410546, EPI_ISL_417921 to 417923, EPI_ISL_419254, EPI_ISL_424342 to 424344	INMI Lazzaro Spallanzani IRCCS	Laboratory of Virology, INMI Lazzaro Spallanzani IRCCS	Antonino Di Caro; Barbara Bartolini; Cesare E. M. Gruber; Cesare Ernesto Maria Gruber; Concetta Castilletti; Daniele Lapa; Eleonora Lalle; Emanuela Giombini; Emanuele Nicastri; Fabrizio Carletti; Francesca Colavita; Francesco Messina; Francesco Vairo; Giulia Matusali; Giuseppe Ippolito; Giuseppe Ippolito.; Licia Bordi; Maria R. Capobianchi; Maria Rosaria Capobianchi; Martina Rueca
EPI_ISL_806544	INSPI Instituto Nacional de Investigación en Salud Pública	Av. Julián Coronel 905 entre Esmeraldas y José Mascote Av. Juan Tanca Marengo No. 100 y Av. de las Américas	Alberto Orlando.; Alfredo Bruno; Andrés Carrazco; Doménica de Mora; Leandro Patiño; Manuel González; Maritza Olmedo; Mary Regato; Melissa Zambrano; Orson Mestanza

EPI_ISL_445302	INSTITUTO MEDICO LEGAL	Instituto de Salud Publica de Chile	Alejandra Acevedo; Andrés E Castillo; Bárbara Parra; Carolina Tambley; Gabriel Leal; Jaime Lagos; Jorge Fernandez; Loredana Arata; Patricia Bustos; Paz Tapia; Rodrigo Fasce; Winston Andrade
EPI_ISL_492184, EPI_ISL_494757, EPI_ISL_494759, EPI_ISL_494761, EPI_ISL_494771	INT Fondazione Pascale	INT Fondazione Pascale	INT Fondazione Pascale; Pascale
EPI_ISL_445261	INTEGRAMEDICA LAB. CLINICO LTDA.	Instituto de Salud Publica de Chile	Alejandra Acevedo; Andrés E Castillo; Bárbara Parra; Carolina Tambley; Gabriel Leal; Jaime Lagos; Jorge Fernandez; Loredana Arata; Patricia Bustos; Paz Tapia; Rodrigo Fasce; Winston Andrade
EPI_ISL_492980 to 492987, EPI_ISL_751320 to 751324, EPI_ISL_751331 to 751447	IRCCS Sacro Cuore Don Calabria Hospital, Department of Infectious, Tropical Diseases & Microbiology	University of Verona, Department of Biotechnology	Antonio Mori; Chiara Degli Esposti; Chiara Piubelli; Cristina Beltrami; Elena Pomari; Emanuela Cosentino; Giulia Lopatriello; Luca Marcolungo; Massimo Delledonne; Michela Deiana
EPI_ISL_480961, EPI_ISL_480975, EPI_ISL_480981, EPI_ISL_480989, EPI_ISL_480995, EPI_ISL_481003, EPI_ISL_481017, EPI_ISL_481025, EPI_ISL_481029, EPI_ISL_481034			
see above	ISGlobal, Institut de Salut Global de Barcelona	SeqCOVID-SPAIN consortium/IBV(CSIC)	Alberto L Garcia-Basteiro; Alfredo Mayor; Carlota Dobaño; Gemma Moncunill; Pau Cisteró and SeqCOVID-SPAIN consortium
EPI_ISL_572320 to 572324, EPI_ISL_584069, EPI_ISL_584071 to 584072	IZSM	IZSM	Giovanna Fusco; Lorena Cardillo; Maurizio Viscardi
EPI_ISL_900587 to 900591	IZSM	TIGEM	Andrea Ballabio; Anna Manfredi; Antonio Grimaldi; Antonio Limone; Biancamaria Pierri; Chiara Colantuono; Davide Cacchiarelli.; Denise Di Concilio; Francesco Panariello; Lucio Di Filippo; Marcello Salvi; Maria Concetta Cuomo; Patrizia Annunziata; Pellegrino Cerino; Valentina Bouche
EPI_ISL_738121 to 738122, EPI_ISL_738124 to 738132	IZSM-U.O.C. Virologia	Istituto Zooprofilattico Sperimentale del Mezzogiorno	Giovanna Fusco; Lorena Cardillo; Maurizio Viscardi
EPI_ISL_479936 to 479943, EPI_ISL_479985, EPI_ISL_480021 to 480029, EPI_ISL_480180 to 480189	Ibaraki Prefectural Institute of Public Health	Pathogen Genomics Center, National Institute of Infectious Diseases	Hajime Kamiya; Keiko Goto; Kentaro Itokawa; Makoto Kuroda; Masanori Hashino; Motoi Suzuki; Rina Tanaka; Tsuyoshi Sekizuka
EPI_ISL_683658 to 683659, EPI_ISL_683663 to 683664, EPI_ISL_683669 to 683670, EPI_ISL_683672 to 683674	IdISSC/Hospital Clínico San Carlos de Madrid	SeqCOVID-SPAIN consortium/IBV(CSIC)	Alberto Delgado-Iribarren; Esther Culebras Lopez; Jorge Matias-Guiu; Luis Ortega Medina; Silvia Sánchez Ramón; Ulises Gómez-Pinedo and SeqCOVID-SPAIN consortium; Vicente Estrada Pérez
EPI_ISL_632847 to 632853, EPI_ISL_632856 to 632859, EPI_ISL_632861, EPI_ISL_632863 to 632865, EPI_ISL_632868, EPI_ISL_632870 to 632873, EPI_ISL_632876, EPI_ISL_632878 to 632883, EPI_ISL_632885 to 632886			
see above	Idaho Bureau of Laboratories	Center for Global Health, University of New Mexico Health Sciences Center	Christopher Ball; Darrell Dinwiddie; Daryl Domman; Kurt Schwalm; Matthew Burns; Robert Voermans
EPI_ISL_1032913	Illinois Department of Public Health	Gagnon Lab, Southern Illinois University	Keith Gagnon
EPI_ISL_420789 to 420790	Illinois Department of Public Health Chicago Laboratory	Pathogen Discovery, Respiratory Viruses Branch, Division of Viral Diseases, Centers for Disease Control and Prevention	Alison S. Laufer Halpin; Anne Uehara; Christopher A. Elkins; Clinton R. Paden; Haibin Wang; Jasmine Padilla; Jing Zhang; Justin Lee; Krista Queen; Mary S. Keckler; Rachel Marine; Suxiang Tong; Yan Li; Ying Tao
EPI_ISL_528746	Immanuel Hospital	Molecular Genetics Laboratory-Faculty of Medicine-Universitas Padjadjaran; School of Life Sciences and Technology & School of Pharmacy-Institut Teknologi Bandung; Laboratorium Kesehatan Provinsi Jawa Barat	Agung Eru Wibowo; Azzania Fibriani; Catur Riani; Cut Nur Cinthia Alamanda; Ema Rahmawati; Fensi Amalina; Hammam Riza; Hesti Lina Wiraswati; Husna Nugrahapraja; Irvan Faizal; Lia Faridah; Marselina Irasonia Tan; Mas Rizky A.A. Syamsunarno; Rifky Waluyajati Rachman; Ryan Bayusantika Ristandi; Savira Ekawardhani; Sony Solistia Wirawan; Tarwadi; Tri Hanggono Achmad; Yunia Sribudiani
EPI_ISL_481110 to 481144, EPI_ISL_481146 to 481187, EPI_ISL_481189 to 481205	Immunogenomics lab, Institute of Life Sciences, Bhubaneswar	Immunogenomics lab, Institute of Life Sciences, Bhubaneswar	Aditi Chatterjee; Ajay Parida; Ajit Singh; Amol Ratnakar Suryawanshi; Ankita Datey; Arup Ghosh; Atimukta Jha; Auromira Khuntia; Bharati Singh; DBT's PAN-INDIA 1000 SARS-CoV2 RNA genome sequencing consortium; Deepika Singh; Dileep Vasudeva; Dileep Vasudevan; Ghulam Hussain Syed; Manasi Priyadarshini; Naga Jogayya Kothakota; Neha Singh; Orissa COVID-19 Study Group; P. Sushree Shyamli; Punit Prasad; Rahul Das; Rajeeb Swain; Rupesh Dash; Shanti Senapati; Sivaram Krishna; Sneha Dutta; Soma Chattopadhyay; Soumyajit Ghosh; Sunil Raghav; Swati Madhulika; Tsheten Sheroa; Tushar K. Beuria; Viplov K. Biswas
EPI_ISL_1018071, EPI_ISL_1018074, EPI_ISL_1018079, EPI_ISL_1018083, EPI_ISL_1018085, EPI_ISL_1018088, EPI_ISL_1018092, EPI_ISL_1018097, EPI_ISL_1018100			
see above	Immunology, Noguchi Memorial Institute for Medical Research	Immunology, Noguchi Memorial Institute for Medical Research	Adu, B.; Adusei-Poku; Agbodzi, B.; Ampofo; Appiah-Kubi, J.; Asare; Bonney; Egyir, B.; J.K.; K.M.; Kumordjie, S.; M.A.; Mohktar, Q.; Odoom; Oteng, F.; Owusu-Nyantakyi, C.; W.K.; Yeboah, C.
EPI_ISL_475572	Imperial College London	Imperial College London	Jie Zhou; Wendy Barclay
EPI_ISL_476702, EPI_ISL_476704	Incubadora Venezolana de Ciencia, Venezuela	Incubadora Venezolana de Ciencia, Venezuela / Instituto Nacional de Salud, Bogotá, Colombia / Grupo de Investigaciones Microbiológicas-UR (GIMUR), Departamento de Biología, Facultad de Ciencias Naturales, Universidad del Rosario, Bogotá, Colombia / Icahn School of Medicine at Mount Sinai, New York, USA	Alberto Paniz-Mondolfi; Ana S. Gonzalez-Reiche; Angelica Rico; Aníbal A. Teherán; Carolina Florez; Carolina Hernández; Emilia Mia Sordillo; Esther C. Barros; Harm van Bakel; Jesús E. Jaimes; Juan David Ramírez; Lisseth Pardo; Lourdes Delgado; Luis Perez-Garcia; Marina Muñoz; Matthew M. Hernandez; Sergio Gomez; Viviana Simon
EPI_ISL_413522	Indian Council of Medical Research - National Institute of Virology	National Influenza Center, Indian Council of Medical Research - National Institute of Virology	Choudhary ML; Potdar V; Shete-Aich A; Yadav PD
EPI_ISL_413523	Indian Council of Medical Research-National Institute of Virology	National Influenza Center, Indian Council of Medical Research-National Institute of Virology	Choudhary ML; Potdar V; Shete-Aich A; Yadav PD
EPI_ISL_420544, EPI_ISL_420546, EPI_ISL_420548, EPI_ISL_420550, EPI_ISL_420552, EPI_ISL_420554, EPI_ISL_420556			
see above	Indian Council of Medical Research-National Institute of Virology, Microbial Containment Complex	Indian Council of Medical Research-National Institute of Virology, Microbial Containment Complex	Amita Jain; Anita M. Shete; Atanu Basu; Bharati Malhotra; Dimpal A. Nyayanit; Gajanan Sapkal; Lalit Dar; M Choudhary; Pragya D. Yadav. Savita Patil; Pranita Gawande; Prasad Sarkale; Priya Abraham; Sarah Cherian; Varsha Potdar
EPI_ISL_825084, EPI_ISL_825086 to 825087	Indian Council of Medical Research-National Institute of Virology,Maximum Containment Laboratory	Indian Council of Medical Research-National Institute of Virology,Maximum Containment Laboratory	Deepak Y Patil; Dimpal A Nyayanit; Jayshri Pethani; Pragya D Yadav; Prasad Sarkale; Rima R Sahay; Savita Patil; Shrikant Baradkar; Varsha Potdar
EPI_ISL_637099 to 637101, EPI_ISL_637103 to 637104	Indian Council of Medical Research-National Institute of Virology, Microbial Containment Complex	Indian Council of Medical Research-National Institute of Virology, Microbial Containment Complex	Anita Shete-Aich; Dimpal A. Nyayanit; Gururaj Rao Deshpande; Padinjaremattathil Thankappan Ullas; Pragya D. Yadav; Prasad Sarkale; Priya Abraham; Varsha Potdar
EPI_ISL_969509	Indian Council of Medical Research-National Institute of Virology, Pune	Indian Council of Medical Research-National Institute of Virology, Maximum Containment Laboratory	Abhinendra Kumar; Anita Shete-Aich; Dimpal A. Nyayanit; Pragya D. Yadav; Savita Patil; Triparna D. Majumdar
EPI_ISL_508287 to 508313, EPI_ISL_508315 to 508337	Indian Institute of Science	National Institute of Biomedical Genomics	Amit Singh; Arindam Maitra; Bharath K Sundararaj; Deepak K Saini; Harsha Raheja; N. Srinivasan; Saumitra Das
EPI_ISL_955218 to 955219	Indiana Animal Disease Diagnostic Laboratory	Carpi Laboratory - Purdue University	Abebe A Fola; G Kenitra Hendrix; Giovanna Carpi; Ilinca I Ciubotariu; Jack Dorman; Lev Gorenstein; Rebecca P Wilkes
EPI_ISL_647986	Indiana State Department of Health	Pathogen Discovery, Respiratory Viruses Branch, Division of Viral Diseases, Centers for Disease Control and Prevention	Anna Montmayeur; Anna Uehara; Brian Lynch; Clinton R. Paden; Haibin Wang; Jing Zhang; Krista Queen; Rachel Marine; Suxiang Tong; Yan Li; Ying Tao
EPI_ISL_755587	Infection and Immunology, Translational Health Science and Technology Institute	Infection and Immunology, Translational Health Science and Technology Institute	A. and Medigeshi; A.K.; Das; G.R.; Kumar, S.; Pandey; Wangchuk, J.
EPI_ISL_413518 to 413521, EPI_ISL_514752	Infectious Disease Control Center, Center for Disease Control and Prevention of PLA	Infectious Disease Control Center, Center for Disease Control and Prevention of PLA	Li; Li, J.; Li, L.; Li, P.; Li, Z.; P. and Li, P.; Qiu, S.; Song, H.
EPI_ISL_596454	Infectious Disease and Tropical Medicine Research Center,	Genetics Research Center, University of Social Welfare and	Ali Jafarpour; Azam Ghaziasadi; Ebrahim Kord; Hossein Najmabadi; Khadijeh Jalalvand; Kimia Kahrizi; Marzieh Mohseni; Seyed Mohammad

	Resistant Tuberculosis Institute, Zahedan University of Medical Sciences, Zahedan, Iran.	Rehabilitation Sciences	Hashemi-Shahri; Seyed Mohammad Jazayeri; Seyedeh elham Mortazavi; Zohreh Fattahi
EPI_ISL_538514 to 538517, EPI_ISL_538519 to 538521	Infectious Diseases, North Carolina State Laboratory of Public Health COVID-19 Response Team	Infectious Diseases, North Carolina State Laboratory of Public Health COVID-19 Response Team	Chase, K.
EPI_ISL_538522 to 538523	Infectious Diseases, North Carolina State Laboratory of Public Health COVID-19 Response Team	North Carolina State Laboratory of Public Health	Chase, K.
EPI_ISL_649152	Infectious diseases, Chan Zuckerberg Biohub	Infectious diseases, Chan Zuckerberg Biohub	Bhatt; K.D.
EPI_ISL_496350, EPI_ISL_496369, EPI_ISL_496374	Infectolab	Andersen lab at Scripps Research	Carlos A. Cota Haros; Octavio Renteria Pacheco; SEARCH Alliance San Diego with Samuel Navarro Alvarez
EPI_ISL_541970	Influenza Centre, University of Bergen	Norwegian Institute of Public Health, Department of Virology	Bjørn Blomberg; Fan Zhou; Hilde Elshaug; Hilde Synnøve Vollan; Kamilla Heddeland Instefjord; Karl A Brokstad; Karoline Bragstad; Kathrine Stene-Johansen; Olav Hungnes; Rasmus Riis Kopperud; Rebecca J Cox
EPI_ISL_470900 to 470904	Influenza etiology and epidemiology laboratory	Pathogenic Microorganisms Variability Laboratory	Alexander Gintsburg; Alexey Shchetinin; Andrey Botikov; Anna Ignatjeva; Daria Grousova; Denis Logunov; Elena Burtseva; Elena Shidlovskaya; Evgeniya Mukasheva; Inna Dolzhikova; Kirill Krasnoslobotsev; Ludmila Kolobukhina; Maria Nikiforova; Nadezhda Kuznetsova; Svetlana Smetanina; Svetlana Trushakova; Vladimir Gushchin
EPI_ISL_469057	Inger Landgren	The Public Health Agency of Sweden	Anna Risberg; Anna-Malin Linde; Karin Tegmark-Wisell; Maria Lind Karlberg; Mattias Haukland; Olov Svartstrom; Oskar Karlsson Lindsjo; Petra Edquist; Reza Advani; Sandra Broddesson; Shamam Muradrasoli
EPI_ISL_635124 to 635125, EPI_ISL_635130 to 635131, EPI_ISL_635183, EPI_ISL_668412 to 668413, EPI_ISL_668415 to 668416, EPI_ISL_708030 to 708031, EPI_ISL_708036 to 708039, EPI_ISL_708151 to 708153			
see above	Innlandet Hospital Trust, Division Lillehammer, Department for Medical Microbiology	Norwegian Institute of Public Health, Department of Virology	Hilde Elshaug; Hilde Vollan; Kamilla Heddeland Instefjord; Karoline Bragstad; Kathrine Stene-Johansen; Marie Paulsen Madsen; Olav Hungnes; Rasmus Riis Kopperud
EPI_ISL_467946, EPI_ISL_467948, EPI_ISL_495122 to 495123, EPI_ISL_495131, EPI_ISL_495136, EPI_ISL_495142, EPI_ISL_495144, EPI_ISL_495149 to 495150, EPI_ISL_511805 to 511810, EPI_ISL_511812 to 511817, EPI_ISL_511819, EPI_ISL_511821 to 511825, EPI_ISL_511828 to 511835, EPI_ISL_511837, EPI_ISL_511839 to 511840, EPI_ISL_511842 to 511843, EPI_ISL_511845, EPI_ISL_511848 to 511851, EPI_ISL_511862, EPI_ISL_569887 to 569896, EPI_ISL_569898 to 569902, EPI_ISL_569904 to 569907, EPI_ISL_569909, EPI_ISL_569911, EPI_ISL_569913, EPI_ISL_569915, EPI_ISL_569932 to 569935, EPI_ISL_569939 to 569943, EPI_ISL_569945, EPI_ISL_569947, EPI_ISL_576519 to 576541, EPI_ISL_576543, EPI_ISL_576550 to 576551, EPI_ISL_677821, EPI_ISL_677824 to 677825, EPI_ISL_677827, EPI_ISL_677832, EPI_ISL_677835, EPI_ISL_677837 to 677862, EPI_ISL_677864 to 677880, EPI_ISL_677883 to 677887, EPI_ISL_677889 to 677894, EPI_ISL_677896 to 677901, EPI_ISL_677904 to 677905, EPI_ISL_677907, EPI_ISL_754864 to 754901, EPI_ISL_756312 to 756318, EPI_ISL_756320, EPI_ISL_756330, EPI_ISL_756332, EPI_ISL_756336, EPI_ISL_756339 to 756343, EPI_ISL_756346 to 756349, EPI_ISL_756351 to 756356, EPI_ISL_918191 to 918192			
see above	Innovative Genomics Institute, UC Berkeley	Innovative Genomics Institute, UC Berkeley	Haridha Shivram; Justin Choi; Liana Lareau; Phil Frankino; Shana McDevitt; Stacia Wyman
EPI_ISL_466648	Innovative Genomics Institute, UCB	Innovative Genomics Institute, UCB	Haridha Shivram; Justin Choi; Liana Lareau; Shana McDevitt; Stacia Wyman
EPI_ISL_418215	Instirut Pasteur Dakar	Institut Pasteur de Dakar	Amadou Alpha Sall; Ndongo Dia; Ousmane Faye
EPI_ISL_416498, EPI_ISL_428355 to 428357	Institut Médico légal- Hop R. Poincaré	National Reference Center for Viruses of Respiratory Infections, Institut Pasteur, Paris	Angela Brisebarre; Etienne Simon-Lorière; Flora Donati; Marion Barbet; Maud Vanpeene; Mélanie Albert; Méline Bizard; Mélnie Albert; Sylvie Behillil; Sylvie van der Werf; Vincent Enouf
EPI_ISL_485712	Institut Pasteur	Institut Pasteur de Dakar	Amadou Alpha Sall.; Mamadou Malado Jallow; Mamadou diop; Marie Henriette Dior Ndione; Moussa Moise Diagne; Ndongo Dia; Ousmane Faye; Safietou Sanke
EPI_ISL_418206 to 418213, EPI_ISL_418216 to 418217, EPI_ISL_420069 to 420070, EPI_ISL_420072 to 420074, EPI_ISL_420076 to 420078, EPI_ISL_476148 to 476149, EPI_ISL_476492 to 476495, EPI_ISL_476514, EPI_ISL_476516, EPI_ISL_476558, EPI_ISL_476562, EPI_ISL_476564, EPI_ISL_476569, EPI_ISL_476572, EPI_ISL_476574, EPI_ISL_477143, EPI_ISL_477150, EPI_ISL_477155 to 477156, EPI_ISL_480554, EPI_ISL_480556, EPI_ISL_480783, EPI_ISL_480787, EPI_ISL_480789, EPI_ISL_481235 to 481237, EPI_ISL_481239 to 481240, EPI_ISL_481243, EPI_ISL_482874 to 482878, EPI_ISL_485635, EPI_ISL_485708, EPI_ISL_485710, EPI_ISL_485713, EPI_ISL_485715 to 485717, EPI_ISL_486859 to 486868, EPI_ISL_486870 to 486873			
see above	Institut Pasteur Dakar	Institut Pasteur de Dakar	Amadou Alpha Sall; Amadou Alpha Sall.; Amadou Alpha sall; Amadou alpha Sall; Mamadou Diop; Mamadou Malado Jallow; Mamadou diop; Mamadou malado Jallow; Marie Henriette Dior Ndione; Moussa Moise; Moussa Moise Diagne; Ndongo Dia; Ousmane Faye; Safietou Sanke; Safietou Sankhe; mamadou Diop
EPI_ISL_498229 to 498230, EPI_ISL_498236, EPI_ISL_498238, EPI_ISL_498242 to 498243, EPI_ISL_498249 to 498250	Institut Pasteur de Dakar	Institut Pasteur de Dakar	Amadou Alpha Sall.; Mamadou Diop; Mamadou Malado Jallow; Marie Henriette Dior Ndione; Moussa Moise Diagne; Ndongo Dia; Ousmane Faye; Safietou Sankhe Mbengue
EPI_ISL_1013214 to 1013216, EPI_ISL_1013236	Institut Pasteur de Guadeloupe	National Reference Center for Viruses of Respiratory Infections, Institut Pasteur, Paris	Angela Brisebarre; Camille Capel; Etienne Simon-Lorière; Marion Barbet; Maud Vanpeene; Méline Bizard; Sylvie Behillil; Sylvie van der Werf; Talarmin Antoine; Vincent Enouf
EPI_ISL_613419 to 613452, EPI_ISL_613455 to 613456	Institut Pasteur de la Guadeloupe	Institut Pasteur de la Guadeloupe	Angela Brisebarre; Antoine Talarmin; Camille Capel; Cherina Fleming; Etienne Simon-Lorière; Marion Barbet; Maud Vanpeene; Méline Bizard; Radjin Steingrover; Stéphanie Guyomard; Sylvie Behillil; Sylvie van der Werf; Sébastien Breurec; Vincent Enouf
EPI_ISL_459965 to 459968, EPI_ISL_459972 to 459984	Institut Pasteur du Maroc	Institut Pasteur du Maroc	Abdellah Faouzi; Anass Abbad; Anderrahmane Maaroufi; Angela Brisebarre; Camille Capel; Etienne Simon-Lorière; Jalal Nourlil; Latifa Anga; Marion Barbet; Maud Vanpeene; Mjid Eloualid; Méline Bizard; Sylvie Behillil; Sylvie van der Werf; Vincent Enouf
EPI_ISL_416747 to 416748, EPI_ISL_416750, EPI_ISL_416753 to 416754, EPI_ISL_416756, EPI_ISL_416758	Institut des Agents Infectieux (IAI) Hospices Civils de Lyon	CNR Virus des Infections Respiratoires - France SUD	Alexandre; Antonin; Bal; Bouscambert-Duchamp; Brengel-Pesce; Bruno.; Cheynet; Destras; Florence; Gaymard; Gregory; Josset; Karen; Laurence; Lina; Martine; Maude; Morfin-Sherpa; Valette; Valérie
EPI_ISL_417333 to 417337, EPI_ISL_417339, EPI_ISL_418420 to 418425, EPI_ISL_418429 to 418432, EPI_ISL_419169 to 419173, EPI_ISL_419177 to 419182, EPI_ISL_419184, EPI_ISL_420604 to 420611, EPI_ISL_420615 to 420616, EPI_ISL_420618 to 420619, EPI_ISL_420621 to 420625, EPI_ISL_508871 to 508874, EPI_ISL_508877, EPI_ISL_508882 to 508911, EPI_ISL_509007 to 509011, EPI_ISL_509013 to 509014			
see above	Institut des Agents Infectieux (IAI), Hospices Civils de Lyon	CNR Virus des Infections Respiratoires - France SUD	Alexandre Gaymard; Antonin Bal; Bruno Lina; Carine Moustaud; Florence Morfin-Sherpa; Gregory Destras; Gwendolyne Burfin; Laurence Josset; Martine Valette; Maude Bouscambert-Duchamp; Raphaelle Lamy; Solenne Brun
EPI_ISL_1008192 to 1008201	Institut für Pathologie, Salzkammergut Klinikum Vöcklabruck	Bergthaler laboratory, CeMM Research Center for Molecular Medicine of the Austrian Academy of Sciences	Andreas Bergthaler; Anna Schedl; Bekir Erguner; Benedikt Agerer; Christoph Bock; Jan Laine; Lukas Endler; Maelle Le Moing; Martin Senekowitsch; Michael Schuster; Thomas Penz
EPI_ISL_437913 to 437932, EPI_ISL_437974 to 437992, EPI_ISL_475769, EPI_ISL_475813 to 475829, EPI_ISL_475919, EPI_ISL_475923 to 475924, EPI_ISL_475926 to 475928, EPI_ISL_583601 to 583603, EPI_ISL_583605 to 583616, EPI_ISL_583691			
see above	Institut für Virologie am Department für Hygiene, Mikrobiologie und Public Health	Bergthaler laboratory, CeMM Research Center for Molecular Medicine of the Austrian Academy of Sciences	Adi Steinrigl; Alexander Lercher; Alexandra Popa; Andreas Bergthaler; Benedikt Agerer; Christian Paar; Christoph Bock; Daniela Schmid; Dorothee von Laer; Elisabeth Puchhammer-Stoeckl; Franz Allerberger; Gernot Walder; Gregor Hörmann; Guenter Weiss; Gunther Vogl; Henrique Colaco; Jakob-Wendelin Genger; Jan Laine; Judith Aberle; Kinga Rigler-Hohenwarter; Lukas Endler; Manfred Nairz; Mark Smyth; Martin Senekowitsch; Michael Schuster; Peter Hufnagl; Peter Obrist; Rainer Gattringer; Sabine Sussitz-Rack; Stephan Aberle; Thomas Penz; Wegene Borena
EPI_ISL_485809 to 485810, EPI_ISL_508688, EPI_ISL_508690 to 508691, EPI_ISL_508693 to 508694, EPI_ISL_508696 to 508697, EPI_ISL_508699 to 508704	Institut für Virologie und Epidemiologie der Viruskrankheiten, Universitätsklinikum Tübingen	NGS Competence Center Tübingen, Institut für Medizinische Mikrobiologie und Hygiene, Universitätsklinikum Tübingen	Angel Angelov; Angelov at al.; Angelov et al.
EPI_ISL_955166	Institute for Biocides and Medical Ecology	Institute of microbiology and Immunology, Faculty of Medicine, University of Belgrade	Jankovic, M.; Jovanovic, T.; Knezevic, A.; Milicevic, O.; Sekler, M.; Tesovic, B.; Vidanovic, D.
EPI_ISL_574790 to 574862	Institute for Infectious Diseases, University of Bern	Institute for Infectious Diseases, University of Bern	Alban Ramette; Christian Baumann; Cora Sägesser; Franziska Suter-Riniker; Michel C Koch; Miguel A Terrazos Miani; Peter Keller; Stephen L Leib
EPI_ISL_860720 to 860723, EPI_ISL_860725, EPI_ISL_913436, EPI_ISL_913442 to 913444	Institute for Infectious Diseases, University of Bern, Switzerland	Institute for Infectious Diseases, University of Bern, Switzerland	Alban Ramette; Christian Baumann; Cora Sägesser; Franziska Suter-Riniker; Michel C Koch; Miguel A Terrazos Miani; Pascal Bittel; Peter Keller; Stephen L Leib
EPI_ISL_583853, EPI_ISL_583855, EPI_ISL_583857 to 583860, EPI_ISL_583862 to 583866, EPI_ISL_583868	Institute for Laboratory Diagnostics and Microbiology, Klinikum Klagenfurt am Worthersee	Bergthaler laboratory, CeMM Research Center for Molecular Medicine of the Austrian Academy of Sciences	Adi Steinrigl; Alexander Lercher; Alexandra Popa; Andreas Bergthaler; Benedikt Agerer; Christian Paar; Christoph Bock; Daniela Schmid; Dorothee von Laer; Elisabeth Puchhammer-Stoeckl; Franz Allerberger; Gernot Walder; Gregor Hörmann; Guenter Weiss; Gunther Vogl; Henrique Colaco; Jakob-Wendelin Genger; Jan Laine; Judith Aberle; Kinga Rigler-Hohenwarter; Lukas Endler; Manfred Nairz; Mark Smyth; Martin Senekowitsch; Michael Schuster; Peter Hufnagl; Peter Obrist; Rainer Gattringer; Sabine Sussitz-Rack; Stephan Aberle; Thomas Penz; Wegene Borena

EPI_ISL_677723, EPI_ISL_678251, EPI_ISL_678253, EPI_ISL_954301, EPI_ISL_1014765	Institute for Lung Diseases in Children - Skopje	Research Center for Genetic Engineering and Biotechnology "Georgi D. Efremov" , Macedonian Academy of Sciences and Arts	Aleksandar J. Dimovski; Dijana Plasheska-Karanfilska; Gjorgji Bozinovski; Milena Jakimovska; Predrag Noveski; RCGEB - MASA
EPI_ISL_455790 to 455793	Institute for Medical Research, Infectious Disease Research Centre, National Institutes of Health, Ministry of Health Malaysia	Malaysia Genome Institute	Azrin Ahmad; Enizza Kasim; Irni Suhayu Sapian; Jeyanthi Suppiah; Mohd Faizal Abu Bakar; Mohd Noor Mat Isa; Norazah Ahmad; Nurhezreen Md Iqbal; Ravindran Thayan; Shahrul Hisham Zainal Ariffin; Shamsidar Sopie; Siti Noraini Othman; Tahir Aris; Yusuf Muhammad Noor; Zarina Mohd Zawawi
EPI_ISL_459957	Institute for Medical Research, Infectious Disease Research Centre, National Institutes of Health, Minis	Institute for Medical Research, Infectious Disease Research Centre, National Institutes of Health, Minis	Ellan K; Kalyanasundram J; Kamel KA; Mohd-Zain R; Mohd-Zawawi Z; Suppiah J; Thayan R
EPI_ISL_430441 to 430442, EPI_ISL_430444, EPI_ISL_459955, EPI_ISL_489992, EPI_ISL_489994, EPI_ISL_490014, EPI_ISL_490016, EPI_ISL_490047 to 490048, EPI_ISL_490089 to 490092, EPI_ISL_490094 to 490096, EPI_ISL_490098 to 490101, EPI_ISL_490103, EPI_ISL_596449, EPI_ISL_644672, EPI_ISL_718267 to 718268, EPI_ISL_718272, EPI_ISL_718274 to 718277, EPI_ISL_718280 to 718285, EPI_ISL_718287, EPI_ISL_718290 to 718294, EPI_ISL_718296 to 718299, EPI_ISL_718301 to 718305, EPI_ISL_718307 to 718308, EPI_ISL_718311 to 718315, EPI_ISL_728160 to 728161, EPI_ISL_728165 to 728167, EPI_ISL_728169 to 728170, EPI_ISL_728178, EPI_ISL_728209 to 728212, EPI_ISL_728242 to 728247, EPI_ISL_728249 to 728252, EPI_ISL_877228, EPI_ISL_934424			
see above	Institute for Medical Research, Infectious Disease Research Centre, National Institutes of Health, Ministry of Health Malaysia	Institute for Medical Research, Infectious Disease Research Centre, National Institutes of Health, Ministry of Health Malaysia	Abdul-Wahid.M-Z; Ahmad.N; Azizan MA; Azizan.M-A; Ellan K; Hisham.H-A; Kalyanasundram J; Kalyanasundram.J; Kamel K; Kamel KA; Mat-Sharani.S; Mohd-Zain R; Mohd-Zain.R; Mohd-Zawawi Z; Mohd-Zawawi.Z; Suppiah J; Suppiah.J; Tan.L-P; Tengku-Rogayah.TAR; Thayan R; Thayan.R
EPI_ISL_583579 to 583598, EPI_ISL_1008213 to 1008214	Institute for Medical and Chemical Laboratory Diagnostics, Kepler Universitätsklinikum	Bergthaler laboratory, CeMM Research Center for Molecular Medicine of the Austrian Academy of Sciences	Adi Steinrigl; Alexander Lercher; Alexandra Popa; Andreas Bergthaler; Anna Schedl; Bekir Erguner; Benedikt Agerer; Christian Paar; Christoph Bock; Daniela Schmid; Dorothee von Laer; Elisabeth Puchhammer-Stoeckl; Franz Allerberger; Gernot Walder; Gregor Hörmann; Guenter Weiss; Gunther Vogl; Henrique Colaco; Jakob-Wendelin Genger; Jan Laine; Judith Aberle; Kinga Rigler-Hohenwarter; Lukas Endler; Maelle Le Moing; Manfred Nairz; Mark Smyth; Martin Senekowitsch; Michael Schuster; Peter Hufnagl; Peter Obrist; Rainer Gattringer; Sabine Sussitz-Rack; Stephan Aberle; Thomas Penz; Wegene Borena
EPI_ISL_429659, EPI_ISL_429663, EPI_ISL_429705, EPI_ISL_429792, EPI_ISL_429802 to 429803, EPI_ISL_429805, EPI_ISL_454605 to 454606, EPI_ISL_455566 to 455567, EPI_ISL_468591, EPI_ISL_468656			
see above	Institute for Public Health	Laboratory for advanced genomics	Filip Roki; Igor Jurak; Lovro Trgovec-Greif; Neven Sui; Oliver Vugrek; Tomislav Rukavina
EPI_ISL_492075 to 492086	Institute for Public Health of the Republic of North Macedonia	Charite Universitatsmedizin Berlin, Institute of Virology	Barbara Muhlemann; Christian Drosten; Elizabeta Jancheska; Golubinka Bosevska; Joern Beheim-Schwarzbach; Julia Schneider; Maja Kuzmanovska; Talitha Veith; Terry Jones; Victor M Corman
EPI_ISL_477205, EPI_ISL_477207, EPI_ISL_477210 to 477211, EPI_ISL_477239, EPI_ISL_477241 to 477242, EPI_ISL_477246, EPI_ISL_477249 to 477250, EPI_ISL_477255, EPI_ISL_477261, EPI_ISL_486669 to 486670, EPI_ISL_486836 to 486841			
see above	Institute for Stem Cell Science and Regenerative Medicine	National Centre for Biological Sciences	Aswin Seshasayee; Awadhesh Pandit; Dasaradhi Palakodeti; Farhan Ali; Mohak Sharda; Shah-e-Jahan Gulzar; Srikar Krishna; Uma Ramakrishnan; Vanessa Molin Paynter; Varadha Sundarmurthy
EPI_ISL_707707 to 707708, EPI_ISL_733528 to 733546, EPI_ISL_733548 to 733550, EPI_ISL_733553 to 733563, EPI_ISL_733565, EPI_ISL_733567	Institute for Urban Disease Control and Prevention	COVID-19 Network Investigations (CONI) Alliance	Amornmas Kongklieng; Anek Mungaomklang; Angkana Huang; Anthony R. Jones; Arporn Wangwiwatsin; Bhakbhoom Panthan; Chonticha Klungtong; Ekawat Pasomsub; Elizabeth Batty; Insee Sensorn; Janjira Thaipadungpanit; Kamolthip Atsawawaranunt; Khajohn Joonsalak; Kingkan Rakmanee; Krittikorn Kumpornsin; Namfon Kotanan; Prayuth Kaewmalang; Pukkaporn Parnwijitkul; Stefan Fernandez; Thanat Chookajorn; Theerarat Kochakarn; Treewat Watthanachockchai; Wasun Chantratita; Wudtichai Manasatienkij
EPI_ISL_572330 to 572331, EPI_ISL_572397, EPI_ISL_573152, EPI_ISL_574259	Institute for Virology, University Hospital Duesseldorf, Medical Faculty, Heinrich-Heine-University Duesseldorf	Institute for Virology, University Hospital Duesseldorf, Medical Faculty, Heinrich-Heine-University Duesseldorf	; Alexander Killer; Andreas Walker; Annemarie Mohring; Anselm Kunstein; Ansgar Schulz; Björn Jensen; Caroline Klindt; Edwin Bölke; Gerald Antoch; Heiner Schaal; Jennifer Neubert; Johannes Bode; Johannes C. Fischer; Jörg Timm; Lisa Müller; Maximilian Damagnez; Nadine Lübke; Ortwin Adams; Philipp Albrecht; Philipp Ostermann; Saskia Elben; Tina Senff; Tom Lüdde; Torsten Feldt; Verena Keitel
EPI_ISL_602463 to 602485, EPI_ISL_602487 to 602509, EPI_ISL_602518 to 602520, EPI_ISL_602522 to 602524, EPI_ISL_602527, EPI_ISL_602529 to 602530, EPI_ISL_626211 to 626212, EPI_ISL_626214, EPI_ISL_626216 to 626217, EPI_ISL_626219 to 626223, EPI_ISL_626230			
see above	Institute for Virology, University Hospital Essen	Center of Medical Microbiology, Virology, and Hospital Hygiene, University of Duesseldorf	Alexander Dilthey; Andreas Walker; Daniel Strelow; Jessica Nicolai; Jörg Timm; Klaus Pfeffer; Lisanna Hülse; Malte Kohns Vasconcelos; Maximilian Damagnez; Nadine Lübke; Olympia E. Anastasiou; Tobias Wienemann; Torsten Houwaart; Ulf Dittmer
EPI_ISL_1008271, EPI_ISL_1008273	Institute for Water Quality and Resource Management, Technical University Vienna	Bergthaler laboratory, CeMM Research Center for Molecular Medicine of the Austrian Academy of Sciences	Andreas Bergthaler; Anna Schedl; Bekir Erguner; Benedikt Agerer; Christoph Bock; Jan Laine; Lukas Endler; Maelle Le Moing; Martin Senekowitsch; Michael Schuster; Thomas Penz
EPI_ISL_956403	Institute for prevention, treatment and rehabilitation of cardiovascular diseases - Ohrid	Research Center for Genetic Engineering and Biotechnology "Georgi D. Efremov" , Macedonian Academy of Sciences and Arts	Aleksandar J. Dimovski; Dijana Plasheska-Karanfilska; Gjorgji Bozinovski; Milena Jakimovska; Predrag Noveski
EPI_ISL_418988	Institute information KU Leuven, Clinical and Epidemiological Virology	Institute information KU Leuven, Clinical and Epidemiological Virology	Bert Vanmechelen; Joan Marti-Carreras; Piet Maes; Tony Wawina
EPI_ISL_891271 to 891272	Institute of Biocides and Medical Ecology, Belgarde, Serbia	Virology department Institute of microbiology and immunology Faculty of Medicine University of Belgrade	Abazovic Dzihan; Banko Ana; Despot Dragana; Loncar Ana; Milicevic Ognjen; Miljanovic Danijela
EPI_ISL_891266	Institute of Biocides and Medical Ecology, Belgrade, Serbia	Virology department Institute of microbiology and immunology Faculty of Medicine University of Belgrade	Abazovic Dzihan; Banko Ana; Despot Dragana; Loncar Ana; Milicevic Ognjen; Miljanovic Danijela
EPI_ISL_525472 to 525473	Institute of Clinical Microbiology and Hygiene, University Hospital Regensburg	Institute of Clinical Microbiology and Hygiene, University Hospital Regensburg	Hiergeist, A.
EPI_ISL_539333 to 539339	Institute of Disease Control and Prevention, People's Liberation Army	Institute of Disease Control and Prevention, People's Liberation Army	Li, P.; Qiu, S.
EPI_ISL_462480, EPI_ISL_485399, EPI_ISL_582031 to 582032	Institute of Human Genetics, Polish Academy of Sciences	Institute of Human Genetics, Polish Academy of Sciences	Adam Ustaszewski; Andrzej Pawski; Emilia Lis; Ewa Zitkiewicz; Marta Kaczmarek-Ry; Micha Witt; Szymon Hryhorowicz
EPI_ISL_485400	Institute of Human Genetics, Polish Academy of Sciences	Institute of Human Genetics, Polish Academy of Sciences,	Adam Ustaszewski; Andrzej Pawski; Emilia Lis; Ewa Zitkiewicz; Marta Kaczmarek-Ry; Micha Witt; Szymon Hryhorowicz
EPI_ISL_450294	Institute of Human Genetics, Polish Academy of Sciences Sanitary and Epidemiological Station in Pozna	Institute of Human Genetics, Polish Academy of Sciences	Adam Ustaszewski; Andrzej Pawski; Emilia Lis; Marta Kaczmarek-Ry; Micha Witt; Szymon Hryhorowicz
EPI_ISL_450295	Institute of Human Genetics, Polish Academy of Sciences; Sanitary and Epidemiological Station in Pozna	Institute of Human Genetics, Polish Academy of Sciences	Adam Ustaszewski; Andrzej Pawski; Emilia Lis; Marta Kaczmarek-Ry; Micha Witt; Szymon Hryhorowicz
EPI_ISL_450338	Institute of Human Genetics, Polish Academy of Sciences; Sanitary and Epidemiological Station in Pozna	Institute of Human Genetics, Polish Academy of Sciences	Adam Ustaszewski; Andrzej Pawski; Emilia Lis; Marta Kaczmarek-Ry; Micha Witt; Szymon Hryhorowicz
EPI_ISL_463010, EPI_ISL_463013 to 463014, EPI_ISL_463017 to 463020, EPI_ISL_463022 to 463027, EPI_ISL_463029, EPI_ISL_463031 to 463034, EPI_ISL_463036 to 463039, EPI_ISL_463041, EPI_ISL_463043, EPI_ISL_463049, EPI_ISL_463051 to 463052, EPI_ISL_463054, EPI_ISL_463056, EPI_ISL_463058, EPI_ISL_463060 to 463061, EPI_ISL_463063 to 463064, EPI_ISL_463067, EPI_ISL_463073 to 463074, EPI_ISL_463076, EPI_ISL_463078, EPI_ISL_463081, EPI_ISL_463085, EPI_ISL_463087 to 463089			
see above	Institute of Life Sciences, Bhubaneswar	Immunogenomics lab, Institute of Life Sciences, Bhubaneswar	Ajay Parida; Aliva Minz; Arup Ghosh; Atimukta Jha; Avula Kiran; DBT's PAN-INDIA 1000 SARS-CoV2 RNA genome sequencing consortium; Debyashrita Barik; Deepak Singh; Eshna Laha; Ghulam Hussain Syed; Hiren G. Dodia; ILS COVID-19 TEAM; Jeky Chawla; Kaushik Sen; Kautilya Kumar Jena; Manasi Priyadarshini; Neha Singh; O. P. Shriwas; Orissa COVID-19 Study Group; Parej Nath; Priyanka Mohapatra; Punit Prasad; Rajeeb Swain; Rina Yadav; Rupesh Dash; Saiket De; Sanchari Chatterjee; Sandhya Suranjika; Satya Ranjan Sahu; Shamima Ansari; Shanti Senapati; Shuchi Smita; Sifu Agarwal; Soma Chattopadhyay; Sunil Raghav; Supriya Suman; Swati Madhulika; Tushar K. Beuria; Viplov K. Biswas
EPI_ISL_723077, EPI_ISL_723079 to 723117	Institute of Medical Genetics and Applied Genomics	Institute of Medical Genetics and Applied Genomics	Angel Angelov; Caspar Gross; Daniela Bezdan; Michael Bitzer; Michael Sonnabend; Michaela Pogoda; Nicolas Casadei; Siri Göpel; Stephan Ossowski; Thomas Iftner; Tina Ganzenmüller
EPI_ISL_960888 to 960890, EPI_ISL_960893, EPI_ISL_1034807 to 1034811, EPI_ISL_1034815 to 1034816	Institute of Medical Microbiology and Hospital Hygiene	Institute of Medical Microbiology and Hospital Hygiene	Aljoscha Tersteegen; Prof. Dr. Achim Kaasch

EPI_ISL_517611	Institute of Medical Microbiology, University Medical Center Goettingen	Institute of Human Genetics, University Medical Center Goettingen	Andreas E Zautner; Antje Dickmanns; Arne Zibat; Bernd Wollnik; Gabriela Salinas-Riester; Maren Sitte; Maren Stegmann; Matthias Dobbelstein; Uwe Groß
EPI_ISL_590823 to 590836	Institute of Medical Virology, University of Zurich	Institute of Medical Virology, University of Zurich	Alexandra Trkola; Benjamin G. Hale; Idoia Busnadiego; Irene Abela; Marie O. Pohl; Maryam Zaheri; Michael Huber; Silke Stertz; Stefan Schmutz; Verena Kufner
EPI_ISL_420294 to 420295, EPI_ISL_420541, EPI_ISL_635201, EPI_ISL_635206 to 635246, EPI_ISL_635248, EPI_ISL_635250 to 635267, EPI_ISL_635269 to 635272, EPI_ISL_635274 to 635278, EPI_ISL_635281 to 635297, EPI_ISL_635299, EPI_ISL_877426, EPI_ISL_877430			
see above	Institute of Microbiology and Immunology, Faculty of Medicine, University of Ljubljana	Institute of Microbiology and Immunology, Faculty of Medicine, University of Ljubljana	Lucijan Skubic; Mario Poljak; Matic Brvar; Miša Korva; Samo Zakotnik; Tatjana Avši - Županc; Tatjana Avši - Županc; Tomaž Mark Zorec
EPI_ISL_548942 to 548946	Institute of Microbiology, University of Veterinary and Animal sciences	Institute of Microbiology, University of Veterinary and Animal sciences	Ali; Altaf, I.; Anwar, M.; Ashraf; Asif, A.; Attique; Awan; Aziz; Bhatti; Cheema; Fazal, S.; Hassan, S.; Khan; Khan, N.; M.A.; M.B.; M.M.; M.S.; M.T.; M.U.; M.W.; M.Z.; Mehmood, A.; Mukhtar, N.; N.A.; Nawaz, M.; Rafique, S.; Rana; Raza, S.; S.Q.; S.Z.; Sardar, N.; Sarwar, H.; Shabbir; Shah; Tahir, Z.; Yaqub, T.; Younis
EPI_ISL_477014 to 477016, EPI_ISL_486842 to 486851, EPI_ISL_491932, EPI_ISL_491936 to 491940, EPI_ISL_516648 to 516652, EPI_ISL_525430 to 525438, EPI_ISL_527809 to 527817, EPI_ISL_539785 to 539793, EPI_ISL_660529 to 660539, EPI_ISL_697783 to 697786, EPI_ISL_697788 to 697790, EPI_ISL_697792 to 697795, EPI_ISL_697798 to 697800, EPI_ISL_728204, EPI_ISL_803098, EPI_ISL_824293 to 824294, EPI_ISL_877561 to 877562			
see above	Institute of Microbiology, Universidad San Francisco de Quito	Institute of Microbiology, Universidad San Francisco de Quito	Adriana Arnao; Alejandra Ramones; Alexandra Noboa; Alexandra Tino; Alvaro Davalos de Castro; Ana Diaz; Andrea Macias; Andres Moreno; Anita Garcia; Belen Prado-Vivar; Belén Prado-Vivar; Bernardo Gutierrez; Bernardo Gutiérrez; Carla Torres; Carlos Guerrero; Carlos Mena; Carlos Tobar; Carolina Pacheco; Cristian Pelaez; Damaris Zandoya; DamarisSandoya; David Acosta-España; Dayron Brossard; Diana Zambrano; Diego Egas; Diego Mora; Diego Morocho; Eddy Chavez; Edgar Espinoza; Edison Chavez; Edison Ligña; Eduardo Males; Edy Quizhpe; Eulalia Pazmiño; Fabricio Picoita; Fausto Maldonado; Fernanda Garcia; Fernando Martinez; Francisco Mora; Francisco Rodriguez; Franklin Espinoza; Freddy Iza; Fredy Loor; Gabriel Trueba; Geovanny Carzola; Hector Moya; Hermelinda Paguay; Jonathan Araujo; Jorge Caamaño; Jorge Luis Velez; Jorge Montaño; Jorge Reyes; Juan Gaviria; Juan Jose Guadalupe; Juan José Guadalupe; Juan Monge; Juan Zuñiga; Karina Barragan; Katalina Pacheco; Khurram Mahbbob; Ligia Briceño; Manuel Jaramillo; Manuel Jibaja; Marco Jimenez; Maribel Cruz; Maureen Mosquera; Michelle Grunauer; Milton Tobar; Monica Becerra-Wong; Nabih Dahik; Nelson Remache; Pablo Flores; Patricio Reyes; Patricio Rojas-Silva; Paul Cardenas; Paúl Cárdenas; Piedad Villacis; Prado-Vivar; Rene Bracho; Rosa Avila; Rosario Erazo; Sayra Caiza; Stalin Castillo; Stephanie Arregui; Sully Marquez; Sully Márquez; Tania Guayasamin; Vanesa Ramirez; Veronica Barragan; Veronica Paredes; Verónica Barragán; Viviana Medina; Yomara Napa
EPI_ISL_634976	Institute of Molecular Biology and Biotechnology, National Institutes of Health	Institute of Molecular Biology and Biotechnology, National Institutes of Health	Arevalo, G.; Dungca; E.M.; N.T. and Salvana; Schwem, B.
EPI_ISL_463008	Institute of Molecular Virology, University Münster	Institute of Molecular Virology, University Münster	Alexander Mellmann; Angeles Mecate Zambrano; Joachim Kühn; Linda Brunotte; Stephan Ludwig
EPI_ISL_402123, EPI_ISL_403928 to 403931	Institute of Pathogen Biology, Chinese Academy of Medical Sciences & Peking Union Medical College	Institute of Pathogen Biology, Chinese Academy of Medical Sciences & Peking Union Medical College	Chao Wu; Jianwei Wang; Lili Ren; Qi Jin; Yiwei Liu; Zhiqiang Wu; Zichun Xiang
EPI_ISL_508338 to 508389, EPI_ISL_508391 to 508414, EPI_ISL_653826 to 653832	Institute of Post Graduate Medical Education & Research	National Institute of Biomedical Genomics	Arindam Maitra; Aritra Biswas; Jayeeta Haldar; Monimoy Banerjee; Raja Ray; Saumitra Das
EPI_ISL_511900 to 511907	Institute of Post Graduate Medical Education & Research	National Institute of Biomedical Genomics - DBT's PAN-INDIA 1000 SARS--CoV-2 RNA Genome Sequencing Consortium	Arindam Maitra; Aritra Biswas; Jayeeta Haldar; Monimoy Banerjee; Raja Ray; Saumitra Das
EPI_ISL_672705, EPI_ISL_672711, EPI_ISL_672719 to 672720, EPI_ISL_672748	Institute of Tropical Medicine at the University of São Paulo (IMT-USP)	Laboratório de Parasitologia Médica - Instituto de Medicina Tropical - Universidade de São Paulo	Brazil-UK Centre for Arbovirus Discovery Diagnosis Genomics and Epidemiology (CADDE) Genomic Network - Instituto de Medicina Tropical
EPI_ISL_408514 to 408515	Institute of Viral Disease Control and Prevention, China CDC	Institute of Viral Disease Control and Prevention, China CDC	Beiwei Ye; Dayan Wang; George F. Gao; Guizhen Wu; Juan Song; Jun Han; Mengjie Yang; Peihua Niu; Peipei Liu; Roujian Lu; Shumei Zou; Wei Zhen; Weifeng Shi; Weimin Zhou; Wenbo Xu; Wenjie Tan; Wenwen Lei; William J. Liu; Xiang Zhao; Yingze Zhao; Yuchao Wu; Zhixiao Chen; Ziqian Xu
EPI_ISL_577734 to 577742, EPI_ISL_583481 to 583489, EPI_ISL_718250 to 718260, EPI_ISL_779651 to 779660, EPI_ISL_831668 to 831669, EPI_ISL_831671 to 831675, EPI_ISL_875519 to 875527, EPI_ISL_903986, EPI_ISL_903989 to 903990, EPI_ISL_904000, EPI_ISL_959604 to 959606, EPI_ISL_959608 to 959618, EPI_ISL_959621 to 959622, EPI_ISL_959627 to 959629, EPI_ISL_959634 to 959635, EPI_ISL_959637 to 959640, EPI_ISL_959645 to 959646			
see above	Institute of Virology, Biomedical Research Center of the Slovak Academy of Sciences, Bratislava	Faculty of Natural Sciences, Comenius University, Bratislava	Andrej Belák; Boris Klempa; Broa Brejová; Dominika Friová; Edita Staroová; Elena Tichá; Jozef Nosek; Juraj Kopáek; Kristína Boršová; Martina Liková; Martina Neboháová; Monika Sláviková; Sabina Fumaová Havlíková; Tomáš Vina; Viktória Hodorová; Viktória abanová; ubomíra Lukáikoá
EPI_ISL_417877 to 417880	Institute of Virology, Biomedical Research Center of the Slovak Academy of Sciences, Bratislava; Public Health Authority of the Slovak Republic, Bratislava	Institute of Virology, Biomedical Research Center of the Slovak Academy of Sciences, Bratislava; Comenius University Science Park, Bratislava	Boris Klempa; Diana Rusáková; Edita Staroová; Elena Tichá; Jaroslav Budiš; Juraj Kopáek; Juraj Koi; Martina Liková; Miroslav Böhmer; Monika Slávikova; Sabina Fumaová Havlíková; Tomáš Szemeš; Werner Krampl
EPI_ISL_796040, EPI_ISL_827042	Institute of Virology, University of Cologne	Institute of Virology, University of Cologne	Alex Thielen; Alexander Dilthey; Andreas Walker; Dominik Aschenmeier; Elena Knops; Eva Heger; Gibran Rubio; Jörg Timm; Martin Däumer; Maximilian Damagnez; Rolf Kaiser; Saleta Sierra; Zevanya Tessalonica
EPI_ISL_1017679, EPI_ISL_1017698	Institute of Virology, Vaccines and Sera "Torlak"	Institute of microbiology and Immunology, Faculty of Medicine, University of Belgrade	Jankovic, M.; Jovanovic, T.; Knezevic, A.; Milicevic, O.; Sekler, M.; Tesovic, B.; Vidanovic, D.
EPI_ISL_455680	Institute of pathogenic microbiology, Jiangsu Provincial Center for Disease Control and Prevention	Institute of pathogenic microbiology, Jiangsu Provincial Center for Disease Control and Prevention	Cui, L.
EPI_ISL_933732	Institution of Nefrology-Struga	Research Center for Genetic Engineering and Biotechnology "Georgi D. Efremov" , Macedonian Academy of Sciences and Arts	RCGEB - MASA
EPI_ISL_755640, EPI_ISL_755642 to 755643, EPI_ISL_755651, EPI_ISL_755653 to 755654	Instituto Adolfo Lutz - Central	Instituto Adolfo Lutz, Interdiciplinary Procedures Center, Strategic Laboratory	Claudia Regina Gonçalves; Claudio Tavares Sacchi; Erica Valessa Ramos Gomes; Karoline Rodrigues Campos
EPI_ISL_755655	Instituto Adolfo Lutz - Regional de Campinas	Instituto Adolfo Lutz, Interdiciplinary Procedures Center, Strategic Laboratory	Claudia Regina Gonçalves; Claudio Tavares Sacchi; Erica Valessa Ramos Gomes; Karoline Rodrigues Campos
EPI_ISL_735401 to 735404, EPI_ISL_735410	Instituto Adolfo Lutz - Regional de Rio Claro	Instituto Adolfo Lutz, Interdiciplinary Procedures Center, Strategic Laboratory	Claudia Regina Gonçalves; Claudio Tavares Sacchi; Erica Valessa Ramos Gomes; Karoline Rodrigues Campos
EPI_ISL_755641, EPI_ISL_755646 to 755647, EPI_ISL_755649	Instituto Adolfo Lutz - Regional de Santo Andre	Instituto Adolfo Lutz, Interdiciplinary Procedures Center, Strategic Laboratory	Claudia Regina Gonçalves; Claudio Tavares Sacchi; Erica Valessa Ramos Gomes; Karoline Rodrigues Campos
EPI_ISL_735400, EPI_ISL_735427, EPI_ISL_735430	Instituto Adolfo Lutz - Regional de Santos	Instituto Adolfo Lutz, Interdiciplinary Procedures Center, Strategic Laboratory	Claudia Regina Gonçalves; Claudio Tavares Sacchi; Erica Valessa Ramos Gomes; Karoline Rodrigues Campos
EPI_ISL_860317, EPI_ISL_860633	Instituto Adolfo Lutz - Regional de Sorocaba	Instituto Adolfo Lutz, Interdiciplinary Procedures Center, Strategic Laboratory	Claudia Regina Gonçalves; Claudio Tavares Sacchi; Erica Valessa Ramos Gomes; Karoline Rodrigues Campos
EPI_ISL_755648, EPI_ISL_755650	Instituto Adolfo Lutz - Regional de Taubate	Instituto Adolfo Lutz, Interdiciplinary Procedures Center, Strategic Laboratory	Claudia Regina Gonçalves; Claudio Tavares Sacchi; Erica Valessa Ramos Gomes; Karoline Rodrigues Campos
EPI_ISL_491175, EPI_ISL_491183 to 491184, EPI_ISL_491187, EPI_ISL_491189, EPI_ISL_491208 to 491211, EPI_ISL_491217, EPI_ISL_491219, EPI_ISL_491222, EPI_ISL_491224 to 491233, EPI_ISL_491235, EPI_ISL_491237 to 491246, EPI_ISL_491248 to 491263, EPI_ISL_491268 to 491271, EPI_ISL_491273 to 491275, EPI_ISL_491289, EPI_ISL_491291 to 491292, EPI_ISL_491297			
see above	Instituto Gulbenkian de Ciência	Instituto Gulbenkian de Ciência	Cathy Paulino; Joao Sobral; João Costa; Ricardo Leite; Susana Ladeiro
EPI_ISL_426364	Instituto Nacional de Ciencias Medicas y Nutricion Salvador Zubiran	Instituto Nacional de Ciencias Medicas y Nutricion	Adnan Araiza Rodríguez; Alejandro Sánchez; Alfredo Ponce de León Garduño; Blanca Taboada; Carlos F. Arias; Carolina González Torres; Celia Boukadida; Cesar Raúl González Bonilla; Concepción Grajales Muñiz; Edgar Mendieta Condado; Eduardo Becerril Vargas; Fabiola Garcés Ayala; Fernando Ledesma Barrientos; Francisco Javier Gaytán Cervantes; Francisco Pulido; Gisela Barrera Badillo; Gloria Vázquez; Guillermo M. Ruiz-Palacios; Irma López Martínez; Joel Armando Vázquez Pérez; José Arturo Martínez Orozco; José Ernesto Ramírez González; José Esteban Muñoz Medina; Lucia

			Hernández Rivas; Luis Alberto García Andrade; Mario Mújica Sánchez; Pavel Isa; Pilar Ramos Cervantes; Ricardo Grande; Santiago Avila Ríos; Victor Hugo Borja Aburto; Violeta Ibarra Gonzalez
EPI_ISL_426361 to 426363, EPI_ISL_426365	Instituto Nacional de Ciencias Medicas y Nutricion Salvador Zubiran	Instituto Nacional de Ciencias Medicas y Nutricion Salvador Zubiran	Adnan Araiza Rodríguez; Alejandro Sánchez; Alfredo Ponce de León Garduño; Blanca Taboada; Carlos F. Arias; Carolina González Torres; Celia Boukadida; Cesar Raúl González Bonilla; Concepción Grajales Muñiz; Edgar Mendieta Condado; Eduardo Becerril Vargas; Fabiola Garcés Ayala; Fernando Ledesma Barrientos; Francisco Javier Gaytán Cervantes; Francisco Pulido; Gisela Barrera Badillo; Gloria Vázquez; Guillermo M. Ruiz-Palacios; Irma López Martínez; Joel Armando Vázquez Pérez; José Arturo Martínez Orozco; José Ernesto Ramírez González; José Esteban Muñoz Medina; Lucia Hernández Rivas; Luis Alberto García Andrade; Mario Mújica Sánchez; Pavel Isa; Pilar Ramos Cervantes; Ricardo Grande; Santiago Avila Ríos; Victor Hugo Borja Aburto; Violeta Ibarra Gonzalez
EPI_ISL_424345, EPI_ISL_424626 to 424627	Instituto Nacional de Enfermedades Respiratorias	Instituto Nacional de Enfermedades Respiratorias	Adnan Araiza Rodríguez; Alejandro Sánchez; Alfredo Ponce de León Garduño; Blanca Taboada; Carlos F. Arias.; Carolina González Torres; Celia Boukadida; Cesar Raúl González Bonilla; Concepción Grajales Muñiz; Edgar Mendieta Condado; Eduardo Becerril Vargas; Fabiola Garcés Ayala; Fernando Ledesma Barrientos; Francisco Javier Gaytán Cervantes; Francisco Pulido; Gisela Barrera Badillo; Gloria Vázquez; Guillermo M. Ruiz-Palacios; Irma López Martínez; Joel Armando Vázquez Pérez; Jorge Salas Hernández; José Arturo Martínez Orozco; José Ernesto Ramírez González; José Esteban Muñoz Medina; Lucia Hernández Rivas; Luis Alberto García Andrade; Mario Mújica Sánchez; Pavel Isa; Pilar Ramos Cervantes; Ricardo Grande; Santiago Avila Ríos; Victor Hugo Borja Aburto; Violeta Ibarra Gonzalez
EPI_ISL_412972	Instituto Nacional de Enfermedades Respiratorias	Instituto de Diagnostico y Referencia Epidemiologicos (INDRE)	Araiza-Rodriguez Adnan; Arias Carlos; Barrera-Badillo Gisela; Boukadida Celia; Garces-Ayala Fabiola; Hernandez-Rivas Lucia; Isa Pavel; Lopez Susana; Lopez-Martinez Irma; Martinez Arturo; Mendieta-Condado Edgar; Munoz-Medina Esteban; Ramirez-Gonzalez Ernesto; Rodriguez-Maldonado Abril; Sanchez Alejandro; Taboada Blanca; Vazquez-Perez Joel; Wong-Arambula Claudia
EPI_ISL_837797	Instituto Nacional de Enfermedades Respiratorias (INER)	Instituto Nacional de Enfermedades Respiratorias (INER)	Alejandra Hernández-Terán; Alma Rincón-Rubio; Celia Boukadida; Edgar Sevilla-Reyes; Eduardo Becerril-Vargas; Fidencio Mejía-Nepomuceno; Hector Esteban Paz-Juárez; Joel Armando Vázquez-Pérez; Jorge Salas-Hernández; José Arturo Martínez-Orozco; Margarita Matías-Florentino; Mario Mújica-Sánchez; Olivia Briceño; Santiago Ávila-Ríos
EPI_ISL_491944 to 491947, EPI_ISL_491949 to 491954	Instituto Nacional de Investigación en Salud Pública - INSPI	INSPI - Charité	Alberto Orlando; Alexandra Usiña; Alfredo Bruno Caicedo; Andres Moreira-Soto; Anna-Lena Sander; Denisses Portugal; Domenica de Mora Coloma; Jan Felix Drexler; Juan Carlos Zeballos; Manuel Gonzalez; Maritza Olmedo; Nina Krause; Silvia Salgado
EPI_ISL_522874, EPI_ISL_522877 to 522878, EPI_ISL_1040562, EPI_ISL_1040569	Instituto Nacional de Medicina Genomica	Instituto Nacional de Medicina Genomica	Alcaraz-Millman M; Cedro-Tanda A; Cisneros-Villanueva M; Herrera-Montalvo LA; Hidalgo-Miranda A; Hurtado-Cordova E; Mendoza-Vargas A; Peñaloza-Figueroa F; Reyes-Grajeda JP
EPI_ISL_522880, EPI_ISL_522940 to 522942, EPI_ISL_522978, EPI_ISL_522980 to 522982, EPI_ISL_522984, EPI_ISL_522986, EPI_ISL_523500			
see above	Instituto Nacional de Medicina Genómica	Instituto Nacional de Medicina Genómica	Cedro-Tanda A; Cisneros-Villanueva M; Herrera-Montalvo LA; Hidalgo-Miranda A; Hurtado-Cordova E; Mendoza-Vargas A; Peñaloza-Figueroa F; Reyes-Grajeda JP
EPI_ISL_418262	Instituto Nacional de Salud	Instituto Nacional de Salud Universidad Cooperativa de Colombia Instituto Alexander von Humboldt Imperial College-London London School of Hygiene & Tropical Medicine	Astrid C. Flórez; Carlos Franco-Muñoz; Christian Julian VillabonaArenas; Diana Marcela Walteros-Acero; Diego A. Álvarez-Díaz; Erika Ospitia; Gloria Puerto; Jose A. Usme-Ciro; Juliana Barbosa; Katherine Laiton-Donato; Liz Villabona-Arenas; Luz Dary Rodriguez; Mailyn A.Gonzalez; Marcela Mercado-Reyes; Martha Lucia Ospina Martinez; Nicolas D. Franco-Sierra; Nuno Rodrigues Faria; Sergio Gomez Rangel; Sussy Echeverria; Zulma M. Cucunubá
EPI_ISL_536477 to 536496, EPI_ISL_536498 to 536500, EPI_ISL_536502 to 536506, EPI_ISL_536508 to 536524, EPI_ISL_536527 to 536529, EPI_ISL_536531, EPI_ISL_536533 to 536534, EPI_ISL_536537 to 536543, EPI_ISL_536545, EPI_ISL_536548 to 536562			
see above	Instituto Nacional de Salud	Laboratorio de Infecciones Respiratorias Agudas	David Tarazona; Dennis Carhuaricra; Eduardo Juscamayta Lopez; Faviola Valdivia Guerrero; Lenin Maturrano Hernandez; Nancy Rojas Serrano; Ronnie Gavilan Chavez
EPI_ISL_456117, EPI_ISL_456119 to 456120, EPI_ISL_456126 to 456127, EPI_ISL_456138, EPI_ISL_456144 to 456155	Instituto Nacional de Salud - Unidad de Secuenciación y Análisis Genómico	Instituto Nacional de Salud, Universidad Cooperativa de Colombia, Instituto Alexander von Humboldt, Imperial College-London, London School of Hygiene & Tropical Medicine	Astrid C. Flórez; Carlos Franco-Muñoz; Christian Julian VillabonaArenas; Diana Marcela Walteros-Acero; Diego A. Álvarez-Díaz; Erika Ospitia; Gloria Puerto; Jose A. Usme-Ciro; Juliana Barbosa; Katherine Laiton-Donato; Liz Villabona-Arenas; Luz Dary Rodriguez; Mailyn A.Gonzalez; Marcela Mercado-Reyes.; Martha Lucia Ospina Martinez; Nicolas D. Franco-Sierra; Sergio Gomez-Rangel; Sussy Echeverria; Zulma M. Cucunubá
EPI_ISL_447755 to 447757, EPI_ISL_447759 to 447763, EPI_ISL_447765 to 447769, EPI_ISL_447771, EPI_ISL_447774 to 447775, EPI_ISL_447777 to 447781, EPI_ISL_447785 to 447786, EPI_ISL_447789 to 447798, EPI_ISL_447800, EPI_ISL_447802 to 447810, EPI_ISL_447812 to 447813, EPI_ISL_447816 to 447817			
see above	Instituto Nacional de Salud, Bogotá, Colombia	Grupo de Investigaciones Microbiológicas-UR (GIMUR), Departamento de Biología, Facultad de Ciencias Naturales, Universidad del Rosario, Bogotá, Colombia Instituto Nacional de Salud, Bogotá, Colombia Icahn School of Medicine at Mount Sinai, New York, USA	Adriana Castillo; Alberto Paniz-Mondolfi; Ana S. Gonzalez-Reiche; Angelica Rico; Aníbal A. Teherán; Carolina Florez; Carolina Hernandez; David Martínez; Emilia Mia Sordillo; Esther C. Barros; Harm van Bakel; Jesús E. Jaimes; Juan David Ramírez; Laura Vega; Lisseth Pardo; Marina Muñoz; Martha L. Ospina; Matthew M. Hernandez; Nathalia Ballesteros; Sergio Castañeda; Sergio Gomez; Viviana Simon
EPI_ISL_497736, EPI_ISL_497738, EPI_ISL_497744 to 497745, EPI_ISL_498152 to 498170, EPI_ISL_526933 to 526934, EPI_ISL_526949 to 526951, EPI_ISL_526955 to 526956, EPI_ISL_526958 to 526959, EPI_ISL_526963 to 526965, EPI_ISL_526967, EPI_ISL_526971, EPI_ISL_653746 to 653747, EPI_ISL_653750 to 653752, EPI_ISL_653754 to 653757, EPI_ISL_739671 to 739673			
see above	Instituto Nacional de Salud, Bogotá, Colombia	Instituto Nacional de Salud, Bogotá, Colombia	Astrid C. Flórez; Carlos Andrés Durán; Carlos Franco-Muñoz; Carolina Ferro; Christian Julian VillabonaArenas; Diana Marcela Walteros-Acero; Diego A. Álvarez-Díaz; Diego Andrés Prada; Edmilson F. de Oliveira-Filho; Felix Betzler; Franklin Prieto; Jeadran Malagón-Rojas; Jonathan Reales; Jose A. Usme-Ciro; Katherine Laiton-Donato; Liz Villabona-Arenas; Magdalena Weisner; Marcela Mercado-Reyes; Martha Lucia Ospina Martinez; Mauricio Pacheco-Montealegre; Nicolas D. Franco-Sierra; Sheryl Corchuelo; Sussy Echeverria; Wendy K. Jo; Zulma M. Cucunubá
EPI_ISL_887439 to 887454, EPI_ISL_964922, EPI_ISL_964924 to 964942	Instituto Nacional de Saude (INS), Mozambique	KRISP, KZN Research Innovation and Sequencing Platform	Emmanuel S; Giandhari J; Nadia Sitoe; Nalia Ismael; Nedio Mabunda; Paulo Arnaldo; Pillay S; Tegally H; Wilkinson E; de Oliveira T
EPI_ISL_421450 to 421452, EPI_ISL_421456, EPI_ISL_421472 to 421478, EPI_ISL_421496 to 421499, EPI_ISL_510889 to 510893, EPI_ISL_510896, EPI_ISL_510898, EPI_ISL_510900 to 510907, EPI_ISL_510909, EPI_ISL_510912, EPI_ISL_510915 to 510916, EPI_ISL_510921, EPI_ISL_510923 to 510929, EPI_ISL_510931 to 510954, EPI_ISL_510956, EPI_ISL_510958 to 510976, EPI_ISL_510978, EPI_ISL_510981 to 510984, EPI_ISL_510987, EPI_ISL_510991 to 510992, EPI_ISL_510994 to 510995, EPI_ISL_510997, EPI_ISL_510999 to 511001, EPI_ISL_511003 to 511004, EPI_ISL_511006 to 511012, EPI_ISL_511014, EPI_ISL_511016 to 511017, EPI_ISL_511019, EPI_ISL_511021 to 511022, EPI_ISL_511025, EPI_ISL_511027 to 511037, EPI_ISL_511039 to 511043, EPI_ISL_511045 to 511047, EPI_ISL_511050, EPI_ISL_511052 to 511074, EPI_ISL_511076 to 511077, EPI_ISL_511079 to 511080, EPI_ISL_511082 to 511083, EPI_ISL_511085 to 511087, EPI_ISL_511089 to 511104, EPI_ISL_511107 to 511141, EPI_ISL_511144 to 511152, EPI_ISL_511154 to 511170, EPI_ISL_511172 to 511177, EPI_ISL_511180 to 511181, EPI_ISL_511316, EPI_ISL_511324, EPI_ISL_511326 to 511328, EPI_ISL_511331 to 511334, EPI_ISL_511336 to 511339, EPI_ISL_511341, EPI_ISL_511343, EPI_ISL_511345 to 511348, EPI_ISL_511350 to 511353, EPI_ISL_511355, EPI_ISL_511357 to 511364, EPI_ISL_511366 to 511379, EPI_ISL_511381 to 511420, EPI_ISL_511422 to 511439, EPI_ISL_511441 to 511442, EPI_ISL_511444, EPI_ISL_511446 to 511453, EPI_ISL_511455 to 511463, EPI_ISL_511465 to 511467, EPI_ISL_511469 to 511474, EPI_ISL_511477, EPI_ISL_511479, EPI_ISL_511481, EPI_ISL_511483, EPI_ISL_511509 to 511510, EPI_ISL_511512 to 511518, EPI_ISL_511520 to 511537, EPI_ISL_511539, EPI_ISL_511541 to 511546, EPI_ISL_511549, EPI_ISL_511551, EPI_ISL_511553, EPI_ISL_511556 to 511561, EPI_ISL_511563, EPI_ISL_511565 to 511567, EPI_ISL_511569 to 511571, EPI_ISL_511576, EPI_ISL_511584, EPI_ISL_511594, EPI_ISL_511598, EPI_ISL_511600, EPI_ISL_511603, EPI_ISL_511611 to 511617, EPI_ISL_511619 to 511640, EPI_ISL_511642 to 511643, EPI_ISL_511645 to 511661, EPI_ISL_511663, EPI_ISL_511668 to 511680, EPI_ISL_511683 to 511685, EPI_ISL_511687 to 511689, EPI_ISL_511691 to 511693, EPI_ISL_511695 to 511696, EPI_ISL_511702, EPI_ISL_511706, EPI_ISL_511708 to 511711, EPI_ISL_511713 to 511717, EPI_ISL_511719 to 511730, EPI_ISL_511733, EPI_ISL_511741, EPI_ISL_511748, EPI_ISL_693529 to 693533, EPI_ISL_693542 to 693629, EPI_ISL_731908 to 731909, EPI_ISL_731918 to 731923, EPI_ISL_731931 to 731939, EPI_ISL_731942 to 732117, EPI_ISL_738112 to 738120, EPI_ISL_759707 to 759709, EPI_ISL_759711, EPI_ISL_759715 to 759716, EPI_ISL_759719, EPI_ISL_759721, EPI_ISL_765210 to 765215, EPI_ISL_765219 to 765221, EPI_ISL_796740 to 796762, EPI_ISL_801517 to 801520, EPI_ISL_861537 to 861563, EPI_ISL_861707 to 861709, EPI_ISL_941352, EPI_ISL_941392 to 941393, EPI_ISL_941413, EPI_ISL_941437, EPI_ISL_941439, EPI_ISL_941467, EPI_ISL_941476, EPI_ISL_941480, EPI_ISL_941485, EPI_ISL_941499 to 941501, EPI_ISL_941505, EPI_ISL_941562, EPI_ISL_941576 to 941577, EPI_ISL_941580, EPI_ISL_941588, EPI_ISL_941594, EPI_ISL_941603 to 941604, EPI_ISL_941607, EPI_ISL_941609 to 941614, EPI_ISL_941616 to 941622, EPI_ISL_941624 to 941632, EPI_ISL_941634 to 941635, EPI_ISL_941637, EPI_ISL_941642, EPI_ISL_1023468			
see above	Instituto Nacional de Saude (INSA)	Instituto Nacional de Saude (INSA)	Borges et al; Guiomar et al
EPI_ISL_511484 to 511497, EPI_ISL_511499 to 511508, EPI_ISL_511752 to 511757, EPI_ISL_511759 to 511771	Instituto Nacional de Saude (INSA)	Instituto Nacional de Saude (INSA) and Instituto Gulbenkian de Ciencia (IGC)	Borges et al
EPI_ISL_511182 to 511188, EPI_ISL_511190 to 511209, EPI_ISL_511211 to 511226, EPI_ISL_511228 to 511256, EPI_ISL_511259, EPI_ISL_511261, EPI_ISL_511264, EPI_ISL_511267 to 511269, EPI_ISL_511271 to 511272, EPI_ISL_511274 to 511277, EPI_ISL_511281, EPI_ISL_511283 to 511290, EPI_ISL_511294 to 511299, EPI_ISL_511301 to 511302, EPI_ISL_511307, EPI_ISL_511310 to 511314, EPI_ISL_731917, EPI_ISL_731924 to 731930, EPI_ISL_731940, EPI_ISL_732118 to 732293, EPI_ISL_765217 to 765218, EPI_ISL_941648, EPI_ISL_941662, EPI_ISL_941669, EPI_ISL_941675, EPI_ISL_941680, EPI_ISL_941692, EPI_ISL_941695, EPI_ISL_941698, EPI_ISL_941703, EPI_ISL_941738, EPI_ISL_941749, EPI_ISL_941752 to 941753, EPI_ISL_941755, EPI_ISL_941760 to 941761, EPI_ISL_941794, EPI_ISL_941812 to 941814, EPI_ISL_941820, EPI_ISL_941826, EPI_ISL_941828, EPI_ISL_941830 to 941833, EPI_ISL_941846, EPI_ISL_941852 to 941853, EPI_ISL_941856 to 941858, EPI_ISL_941868, EPI_ISL_941870, EPI_ISL_941893			
see above	Instituto Nacional de Saude (INSA) and Instituto Gulbenkian de Ciencia (IGC)	Instituto Nacional de Saude (INSA) and Instituto Gulbenkian de Ciencia (IGC)	Borges et al
EPI_ISL_426580	Instituto Sabin	Laboratory of Virology	Bergmann M Ribeiro; Fernando L Melo; Gustavo Barra; Ikaro A Andrade; Pedro G Mesquita; Tatsuya Nagata; Ticiane H Santa-Rita

EPI_ISL_492032 to 492048	Instituto de Biologia do Exército	Laboratório Metabolismo Macromolecular FirminoTorres de Castro, Instituto de Biofísica Carlos Chagas Filho, Universidade Federal do Rio de Janeiro	Aline Rosa Vianna de Souza; Bianca Catarina Azevedo Cabral; Caleb GM Santos; Clarissa Damaso; Elizabeth Valentin; Marcio da Costa Cipitelli; Marcos Dornelas-Ribeiro; Nádia Vaez Gonçalves da Cruz; Rodrigo Soares de Moura Neto; Rosane Silva; Tatiana LS Nogueira; Virginia Sara Grancieri do Amaral
EPI_ISL_424673	Instituto de Diagnostico y Referencia Epidemiologicos	Instituto de Diagnostico y Referencia Epidemiologicos	Adnan Araiza Rodríguez; Alejandro Sánchez; Alfredo Ponce de León Garduño; Blanca Taboada; Carlos F. Arias; Carolina González Torres; Celia Boukadida; Cesar Raúl González Bonilla; Concepción Grajales Muñiz; Edgar Mendieta Condado; Eduardo Becerril Vargas; Fabiola Garcés Ayala; Fernando Ledesma Barrientos; Francisco Javier Gaytán Cervantes; Francisco Pulido; Gisela Barrera Badillo; Gloria Vázquez; Guillermo M. Ruiz-Palacios; Irma López Martínez; Joel Armando Vázquez Pérez; José Arturo Martínez Orozco; José Ernesto Ramírez González; José Esteban Muñoz Medina; Lucia Hernández Rivas; Luis Alberto García Andrade; Mario Mújica Sánchez; Pavel Isa; Pilar Ramos Cervantes; Ricardo Grande; Santiago Avila Ríos; Victor Hugo Borja Aburto; Violeta Ibarra Gonzalez
EPI_ISL_455434 to 455439, EPI_ISL_455455 to 455456, EPI_ISL_493334 to 493336, EPI_ISL_493346, EPI_ISL_493348, EPI_ISL_516609, EPI_ISL_516611, EPI_ISL_516613, EPI_ISL_516618, EPI_ISL_516620, EPI_ISL_516622, EPI_ISL_516624 to 516625, EPI_ISL_576257 to 576279, EPI_ISL_658863, EPI_ISL_658865, EPI_ISL_658868, EPI_ISL_658870, EPI_ISL_658873, EPI_ISL_658875, EPI_ISL_658878, EPI_ISL_658880, EPI_ISL_658883, EPI_ISL_658886, EPI_ISL_658888, EPI_ISL_658891, EPI_ISL_658893, EPI_ISL_658896, EPI_ISL_658901, EPI_ISL_658904			
see above	Instituto de Diagnostico y Referencia Epidemiologicos (INDRE)	Instituto de Diagnostico y Referencia Epidemiologicos (INDRE)	; Abril Rodriguez-Maldonado; Adnan Araiza-Rodriguez; Araiza-Rodriguez Adnan; Barrera-Badillo Gisela; Claudia Wong-Arambula; Dayanira Arellano-Suarez; Edgar Mendieta-Condado; Ernesto Ramirez-Gonzalez; Ernesto Ramirez-Gonzalez.; Fabiola Garces-Ayala; Garces-Ayala Fabiola; Garces-Ayala Fabiola. Ramirez-Gonzalez Ernesto; Garces-Ayala Fabiola. Taboada Ramírez Blanca. Ramirez-Gonzalez Ernesto; Garces-Ayala Fabiola. Taboada Ramirez Blanca. Ramirez-Gonzalez Ernesto; Garces-Ayala Fabiola. Taboada Ramírez Blanca. Ramirez-Gonzalez Ernesto; Gaudalupe Herrera-Ramirez; Gisela Barrera-Badillo; Gisela Barrera-Badillo.; Hernandez-Rivas Lucia; Irma Lopez-Martinez; Jazmin Galicia-Hernandez.; Linda Andarade-Sanchez; Lopez-Martinez Irma; Lopez-Martinez Irma.; Lucia Hernandez-Rivas; Marisol Galindo-Galindo; Mendieta-Condado Edgar; Natividad Cruz-Ortiz; Octavio Ruiz-Muñiz; Pamela Ramirez-Medina; Ramirez-Gonzalez Ernesto.; Rodriguez-Maldonado Abril; Roman Canul-Aguilar; Taboada Ramirez Blanca; Taboada Ramírez Blanca.; Taboada Ramírez Blanca. Ramirez-Gonzalez Ernesto; Tatiana Nunez-Garcia; Wong-Arambula Claudia; Wong-Arambula Claudia.
EPI_ISL_452139	Instituto de Diagnostico y Referencia Epidemiologicos (INDRE)	Instituto de diagnóstico y Referencia Epidemiologicos (INDRE)	Araiza-Rodriguez Adnan; Barrera-Badillo Gisela; Garces-Ayala Fabiola; Hernandez-Rivas Lucia; Lopez-Martinez Irma; Mendieta-Condado Edgar; Ramirez-Gonzalez Ernesto; Rodriguez-Maldonado Abril; Wong-Arambula Claudia
EPI_ISL_913981	Instituto de Diagnostico y Referencia Epidemiologicos INDRE_RNLSP	Instituto de Diagnostico y Referencia Epidemiologicos (INDRE)	Abril Rodriguez-Maldonado; Adnan Araiza-Rodriguez; Claudia Wong-Arambula; David Fragoso-Fonseca; Ernesto Ramirez-Gonzalez.; Fabiola Garces-Ayala; Gisela Barrera-Badillo; Irma Lopez-Martinez; Lucia Hernandez-Rivas; Mayra Jimenez-Morales; Nancy Munoz-Hernandez; Natividad Cruz-Ortiz; Sergio Rangel-Guerrero; Tatiana Nunez-Garcia
EPI_ISL_747615, EPI_ISL_748138 to 748145	Instituto de Investigaciones Biologicas Clemente Estable	Institut Pasteur de Montevideo	Ana Carolina Mendonça; Andres Lizasoain; Camila Simoes; Cecilia Alonso; Cecilia Salazar; Daiana Mir; Fernando Lopez-Tort; Fernando Motta; Gonzalo Bello; Ighor Arantes; Ignacio Ferrés; Jose Sotelo; Leticia Maya; Letícia Garay Martins; Luciana Appolinario; Lucia Spangenberg; Mailen Arleo; Mariana Brandes; Marilda Mendonça Siqueira; Marilda Tereza Mar da Rosa; Maria Jose Benitez-Galeano; Martín Graña; Matias Castells; Matias Victoria; Matías Salvo; Natalia Rego; Natalia Reyes; Pablo Smircich; Paola Cristina Resende; Rodney Colina; Tamara Fernandez-Calero; Tania Possi; Tatiana Schäffer Gregianini; Veronica Noya; Yasser Vega
EPI_ISL_510066 to 510069, EPI_ISL_510072, EPI_ISL_510075, EPI_ISL_510077, EPI_ISL_510463 to 510465	Instituto de Investigaciones Biomédicas de Barcelona (CSIC), Hospital Clinic i Provincial de Barcelona, Instituto de Biomedicina de Valencia (CSIC), Hospital de Sant Pau	SeqCOVID-SPAIN consortium/IBV(CSIC)	Alex Soriano; Andrea Vergara; Anna M. Planas; Israel Fernández Cadenas and SeqCOVID-SPAIN consortium; Jordi Pérez Tur; Miguel J. Martínez; Mª Angeles Marcos
EPI_ISL_729869 to 729871, EPI_ISL_729902 to 729919	Instituto de Medicina Tropical, Universidad Nacional Toribio Rodríguez de Mendoza de Amazonas	Laboratorio de Genómica Microbiana, Universidad Peruana Cayetano Heredia	Alejandra Dávila-Barclay; Brenda Ayzanoa; Carla Montenegro; Cecilia Pajuelo; Janet Huancachoque; Luis González; Pablo Tsukayama; Pedro E. Romero; Pool Marcos; Rafael Tapia; Stella Chenet
EPI_ISL_499083	Instituto de Virologia "Dr. J. M. Vanella", Facultad de Ciencias Medicas, Universidad Nacional de Cordoba. Laboratorio Central de la Provincia de Cordoba, Argentina. Ministerio de Salud de la provincia de Cordoba, Argentina.	Laboratorio de Virología, Hospital de Niños Ricardo Gutiérrez, CABA, Argentina.	Adrian Diaz; Brenda Konigheim; Gabriela Barbas; Gonzalo Castro; Javier Aguilar; Lorena Spinsanti; Mariana Viegas.; Mercedes Nabaes; Monica Natale; Sandra Gallego; Sebastian Blanco; Silvina Lusso; Stephanie Goya
EPI_ISL_745090	Instutite for Medical Microbiology, School of Medicine, University of Goettingen	Institut of Human Genetics, University Medicine Goettingen	Gross U; Wollnik B; Zautner A; Zibat A
EPI_ISL_419299 to 419300, EPI_ISL_479796	Ishikawa Prefectural Institute of Public Health and Environmental Science	Pathogen Genomics Center, National Institute of Infectious Diseases	Eri Nariai; Hajime Kamiya; Kentaro Itokawa; Makoto Kuroda; Masanori Hashino; Motoi Suzuki; Rina Tanaka; Sanae Kuramoto; Tsuyoshi Sekizuka
EPI_ISL_474959 to 474975, EPI_ISL_474977 to 475025, EPI_ISL_483704, EPI_ISL_483708 to 483709, EPI_ISL_483711 to 483713, EPI_ISL_483715, EPI_ISL_483717, EPI_ISL_483725, EPI_ISL_514266, EPI_ISL_514268 to 514272, EPI_ISL_514274, EPI_ISL_514278 to 514281, EPI_ISL_514283 to 514284, EPI_ISL_514286 to 514296, EPI_ISL_514299 to 514303, EPI_ISL_514305 to 514306, EPI_ISL_514308 to 514311, EPI_ISL_514313 to 514316, EPI_ISL_516884 to 516891, EPI_ISL_516893 to 516903, EPI_ISL_516905 to 516906, EPI_ISL_516913, EPI_ISL_516915, EPI_ISL_516917, EPI_ISL_516919 to 516921, EPI_ISL_575332 to 575334, EPI_ISL_649064 to 649107, EPI_ISL_745034, EPI_ISL_745036 to 745046, EPI_ISL_745048 to 745049, EPI_ISL_745051 to 745054, EPI_ISL_745056 to 745058, EPI_ISL_745061 to 745073, EPI_ISL_745075, EPI_ISL_745078 to 745081, EPI_ISL_745083 to 745086, EPI_ISL_776591 to 776600, EPI_ISL_776636, EPI_ISL_776776, EPI_ISL_776780 to 776787			
see above	Israel Central Virology laboratory	Israel Central Virology laboratory	Efrat Dahan Bucris; Ella Mendelson; Michal Mandelboim; Neta Zuckerman; Oran Erster; Orna Mor
EPI_ISL_804059 to 804062, EPI_ISL_804064 to 804083, EPI_ISL_804193 to 804201, EPI_ISL_814097, EPI_ISL_814123, EPI_ISL_814130 to 814131, EPI_ISL_814135, EPI_ISL_814155, EPI_ISL_814157, EPI_ISL_814159, EPI_ISL_814163, EPI_ISL_814175, EPI_ISL_814179, EPI_ISL_814181, EPI_ISL_814185 to 814192, EPI_ISL_889032, EPI_ISL_889041, EPI_ISL_889055, EPI_ISL_889058 to 889059, EPI_ISL_889061, EPI_ISL_889063, EPI_ISL_889070, EPI_ISL_889075, EPI_ISL_889134 to 889135, EPI_ISL_889144, EPI_ISL_889155, EPI_ISL_889170, EPI_ISL_889201, EPI_ISL_944236 to 944247, EPI_ISL_944251 to 944254, EPI_ISL_944256 to 944367, EPI_ISL_944411 to 944412, EPI_ISL_944495, EPI_ISL_944514			
see above	Israel Central Virology laboratory	Israel National Consortium for SARS-CoV-2 sequencing	Assaf Rokney; Dana Bar-Ilan; David A. Zeevi; Efrat Dahan Bucris; Efrat Glick-Saar; Efrat Rorman; Ella Mendelson; Ephraim Fass; Eva Nachum; Gal Zizelski Valenci; Gideon Rechavi; Israel Nissan; Joseph Jaffe; Maya Davidovich Cohen; Michal Mandelboim; Mor Rubinstein; Neta Zuckerman; Omer Murik; Omri Nayshool; Oran Erster; Orna Mor; Tzvia Mann
EPI_ISL_594158 to 594160	Israel Institute for Biological Research	Israel Institute for Biological Research	Adi Beth-Din and Anat Zvi; Dana Stein; Emanuelle Mamroud; Galia Zaide; Inbar Cohen-Gihon; Libby Weiss; Ofir Israeli; Orly Laskar; Shay Weiss; Yoav Gal
EPI_ISL_474958, EPI_ISL_514275, EPI_ISL_516892	Israeli Central Virology laboratory	Israel Central Virology laboratory	Efrat Dahan Bucris; Ella Mendelson; Michal Mandelboim; Neta Zuckerman; Oran Erster
EPI_ISL_477193, EPI_ISL_477203	Istituto Zooprofilattico Sperimentale Puglia e Basilicata;	Beaconlab (Bioinformatics, Evolution and Comparative Genomics lab), Dept of Biosciences, University on Mila	Chiara M.; Manzari C.; Parisi A.; Pesole G.
EPI_ISL_477199 to 477200	Istituto Zooprofilattico Sperimentale Puglia e Basilicata;	Beaconlab (Bioinformatics, Evolution and Comparative Genomics lab), Dept of Biosciences, University on Milan	Chiara M.; Manzari C.; Parisi A.; Pesole G.
EPI_ISL_451961	Istituto Zooprofilattico Sperimentale Puglia e Basilicata; Dipartimento di Bioscienze, Biotecnologie e Biofarmaceutica dell'Università degli Studi di Bari "A.Moro"; Istituto di Biomembrane. Bioenergetica e Biotecnologie Molecolari del Consiglio Nazionale delle Ricerche di Bari	Beaconlab (Bioinformatics Evolution and Comparative Genomics lab), Dept of Biosciences, University of Milan	Chiara M.; Manzari C.; Parisi A.; Pesole G.
EPI_ISL_468914, EPI_ISL_469020, EPI_ISL_469022 to 469023, EPI_ISL_525553, EPI_ISL_525557, EPI_ISL_525560 to 525561, EPI_ISL_525569 to 525570, EPI_ISL_525573 to 525574, EPI_ISL_527380			
see above	Istituto Zooprofilattico Sperimentale Puglia e Basilicata; Dipartimento di Bioscienze, Biotecnologie e Biofarmaceutica dell'Università degli Studi di Bari "A.Moro"; Istituto di Biomembrane. Bioenergetica e Biotecnologie Molecolari del Consiglio Nazionale delle Ricerche di Bari	Beaconlab (Bioinformatics, Evolution and Comparative Genomics lab), Dept of Biosciences, University on Milan	Chiara M; Chiara M.; Manzari C.; Parisi A.; Pesole G.
EPI_ISL_493332 to 493333, EPI_ISL_560407, EPI_ISL_913449	Istituto Zooprofilattico Sperimentale del Mezzogiorno	INMI Lazzaro Spallanzani IRCCS	Antonino Di Caro; Barbara Bartolini; Cesare E.M. Gruber; Cesare EM Gruber; Concetta Castilletti; Emanuela Giombini; Francesca Colavita; Francesco Messina; Giorgia Borriello; Giovanna Fusco; Maria R Capobianchi; Maria R. Capobianchi; Martina Rueca; Maurizio Viscardi; Ornella Butera; Sergio Brandi; Silvia Meschi

EPI_ISL_736789, EPI_ISL_736791 to 736890, EPI_ISL_776924, EPI_ISL_960459 to 960467, EPI_ISL_960470, EPI_ISL_1039262	Istituto Zooprofilattico Sperimentale del Mezzogiorno	TIGEM	Andrea Ballabio; Anna Manfredi; Antonio Grimaldi; Antonio Limone; Biancamaria Pierri; Chiara Colantuono; Davide Cacchiarelli.; Denise Di Concilio; Francesco Panariello; Lucio Di Filippo; Marcello Salvi; Maria Concetta Cuomo; Patrizia Annunziata; Pellegrino Cerino; Valentina Bouche
EPI_ISL_602304	Istituto Zooprofilattico Sperimentale del Mezzogiorno	U.O. Diagnostica Virologica Dip. Sanità Animale IZSM	Lorena Cardillo; Maurizio Viscardi; e Giovanna Fusco
EPI_ISL_763069, EPI_ISL_763071 to 763073, EPI_ISL_763094 to 763098, EPI_ISL_763138, EPI_ISL_763318, EPI_ISL_763320, EPI_ISL_763322 to 763330			
see above	Istituto Zooprofilattico Sperimentale dell' Umbria e delle Marche -Togo Rosati	Istituto Superiore di Sanità	Gabriele Vaccari; Giovanni Ianiro; Ilaria Di Bartolo; Luca De Sabato; Massimo Biagetti; Monica Giammarioli
EPI_ISL_1020311, EPI_ISL_1020313, EPI_ISL_1020328, EPI_ISL_1020330	Istituto Zooprofilattico Sperimentale dell' Umbria e delle Marche -Togo Rosati Università degli Studi di Perugia	Department of Food safety, Nutrition and Veterinary public health - Istituto Superiore di Sanità	Antonella Mencacci; Barbara Camilloni; Gabriele Vaccari; Giovanni Ianiro; Ilaria Di Bartolo; Luca De Sabato; Massimo Biagetti; Monica Giammarioli
EPI_ISL_649189 to 649191, EPI_ISL_649784, EPI_ISL_649940, EPI_ISL_653784 to 653786, EPI_ISL_653813, EPI_ISL_722873 to 722894, EPI_ISL_722901 to 722911, EPI_ISL_722913 to 722925, EPI_ISL_1008665 to 1008677, EPI_ISL_1008679 to 1008699, EPI_ISL_1008701 to 1008702			
see above	Istituto Zooprofilattico Sperimentale della Puglia e della Basilicata	Istituto Zooprofilattico Sperimentale della Puglia e della Basilicata	Bianco A.; Capozzi L.; Chironna M.; Cipolletta D.; Del Sambro L.; Galante D.; Giannico A.; Loconsole D.; Manzulli V; Pace L.; Parisi A.; Ridolfi D.; Rondinone V.; Sallustio A.; Simone D.
EPI_ISL_422426 to 422427	JABER AL AHMAD AL SABAH HOSPITAL - KUWAIT CITY	Dasman Diabetes Institute	Ebaa Al-Ozairi; Fahd Al-Mulla; Qais Al-Duwairi; Rasheeba Iqbal; Sumi John
EPI_ISL_422424	Jaber Al Ahmad Al Sabah Hospital	Dasman diabetes Institute	Ebaa Al-Ozairi; Fahd Al-Mulla; Qais Al-Duwairi; Rasheeba Iqbal; Sumi John
EPI_ISL_631516, EPI_ISL_631524, EPI_ISL_631550 to 631551, EPI_ISL_631554, EPI_ISL_631571, EPI_ISL_631574, EPI_ISL_631578, EPI_ISL_631582, EPI_ISL_631585, EPI_ISL_631590, EPI_ISL_631593, EPI_ISL_631595, EPI_ISL_631597, EPI_ISL_631599, EPI_ISL_631601, EPI_ISL_631604, EPI_ISL_631608 to 631610, EPI_ISL_631612 to 631613, EPI_ISL_631615, EPI_ISL_631621, EPI_ISL_631634, EPI_ISL_631636 to 631637, EPI_ISL_631697 to 631698, EPI_ISL_631702 to 631706, EPI_ISL_631709, EPI_ISL_631711 to 631713, EPI_ISL_631716 to 631722, EPI_ISL_631726, EPI_ISL_631728, EPI_ISL_631730 to 631731, EPI_ISL_631733, EPI_ISL_631735 to 631747, EPI_ISL_631767 to 631768, EPI_ISL_631772, EPI_ISL_631775 to 631777, EPI_ISL_631788 to 631789, EPI_ISL_631809 to 631810, EPI_ISL_631812 to 631813, EPI_ISL_631820, EPI_ISL_631822, EPI_ISL_631825, EPI_ISL_631833, EPI_ISL_631835 to 631836, EPI_ISL_631847, EPI_ISL_631849, EPI_ISL_631852 to 631854, EPI_ISL_631866 to 631867, EPI_ISL_631876, EPI_ISL_631879, EPI_ISL_631882 to 631883, EPI_ISL_631887, EPI_ISL_631889 to 631890, EPI_ISL_631895 to 631896, EPI_ISL_631902, EPI_ISL_631904, EPI_ISL_631909, EPI_ISL_631921 to 631922, EPI_ISL_631925 to 631928, EPI_ISL_631950 to 631951, EPI_ISL_631963, EPI_ISL_631966, EPI_ISL_631969 to 631970, EPI_ISL_631973, EPI_ISL_631979, EPI_ISL_631994, EPI_ISL_631997, EPI_ISL_632005, EPI_ISL_632007 to 632009, EPI_ISL_632011 to 632012, EPI_ISL_632017, EPI_ISL_632021, EPI_ISL_632023, EPI_ISL_632025, EPI_ISL_632028 to 632029, EPI_ISL_632035, EPI_ISL_632037, EPI_ISL_632051, EPI_ISL_632061, EPI_ISL_632065 to 632066, EPI_ISL_632075, EPI_ISL_632093 to 632096, EPI_ISL_632106 to 632110, EPI_ISL_632128, EPI_ISL_632130, EPI_ISL_632143, EPI_ISL_632145, EPI_ISL_632149 to 632152, EPI_ISL_632162, EPI_ISL_632166 to 632172, EPI_ISL_632180, EPI_ISL_632182, EPI_ISL_632184, EPI_ISL_632188 to 632189, EPI_ISL_632191, EPI_ISL_632195, EPI_ISL_632198, EPI_ISL_632204, EPI_ISL_632209 to 632210, EPI_ISL_632214, EPI_ISL_632219, EPI_ISL_632221, EPI_ISL_632227 to 632229, EPI_ISL_632239 to 632241, EPI_ISL_632939, EPI_ISL_632941, EPI_ISL_632944, EPI_ISL_632949 to 632950, EPI_ISL_632953, EPI_ISL_632957 to 632958, EPI_ISL_632961, EPI_ISL_632969 to 632972, EPI_ISL_632974			
see above	Jamaica Hospital Medical Center	New York City Public Health Laboratory	Jade Wang; et al.
EPI_ISL_450792 to 450799	Jamaica Ministry of Health and Wellness	Pathogen Discovery, Respiratory Viruses Branch, Division of Viral Diseases, Centers for Disease Control and Prevention	Anna Montmayeur; Anna Uehara; Bettina Bankamp; Clinton R. Paden; Haibin Wang; Jasmine Padilla; Jing Zhang; Justin Lee; Krista Queen; Rachel Marine; Suxiang Tong; Yan Li; Ying Tao; Zachary Weiner
EPI_ISL_631596, EPI_ISL_631600, EPI_ISL_632179, EPI_ISL_632200	James J. Peters VA Medical Center	New York City Public Health Laboratory	Jade Wang; et al.
EPI_ISL_451958	Jamil-ur-Rahman Center for Genome Research, Dr. Panjwani Center for Molecular Medicine and Drug Research, International Center for Chemical and Biological Sciences, University of Karachi	Jamil-ur-Rahman Center for Genome Research, Dr. Panjwani Center for Molecular Medicine and Drug Research, International Center for Chemical and Biological Sciences, University of Karachi	B.A.; I.A; Khan; Khan, S.; M.A.and Khan; Qureshi; Raza; S.A.; Shakeel, M.; Zahid, M.
EPI_ISL_416565 to 416567, EPI_ISL_416569 to 416587, EPI_ISL_416589 to 416615, EPI_ISL_416617 to 416622, EPI_ISL_416624 to 416634, EPI_ISL_454749	Japanese Quarantine Stations	Pathogen Genomics Center, National Institute of Infectious Diseases	Hajime Kamiya; Hideki Hasegawa; Ikuyo Takayama; Kentaro Itokawa; Makoto Kuroda; Masanori Hashino; Motoi Suzuki; Rina Tanaka; Shinji Saito; Takaji Wakita; Takuri Takahashi; Takuya Yamagishi; Tsutomu Kageyama; Tsuyoshi Sekizuka
EPI_ISL_450821 to 450822	Jarpens HC	The Public Health Agency of Sweden	Anna Risberg; Anna-Malin Linde; Gunilla Johansson; Karin Tegmark-Wisell; Maria Lind Karlberg; Mia Brytting; Olov Svartstrom; Oskar Karlsson Lindsjo; Theresa Enkirch
EPI_ISL_728288	Jena University Hospital, Institute for Infectious Diseases and Infection Control	Institute for infectious medicine & hospital hygiene, CaSe-Group	Brandt; Christian; Marquet; Matthias W.; Mike; Pletz; Riccardo; Spott
EPI_ISL_728292 to 728294, EPI_ISL_728296, EPI_ISL_728298, EPI_ISL_728302 to 728303, EPI_ISL_728323 to 728324, EPI_ISL_728331, EPI_ISL_728333, EPI_ISL_728335, EPI_ISL_728337 to 728338, EPI_ISL_728341, EPI_ISL_763077, EPI_ISL_763086 to 763093, EPI_ISL_768633, EPI_ISL_768725 to 768727			
see above	Jena University Hospital, Institute for Infectious Diseases and Infection Control	Institute of infectious medicine & hospital hygiene, CaSe-Group	Brandt; Christian; Marquet; Matthias W.; Mike; Pletz; Riccardo; Spott
EPI_ISL_940569 to 940570, EPI_ISL_940572, EPI_ISL_940587 to 940588, EPI_ISL_940600, EPI_ISL_940602, EPI_ISL_940604, EPI_ISL_940606, EPI_ISL_949016, EPI_ISL_949074, EPI_ISL_949086 to 949087, EPI_ISL_949108 to 949109, EPI_ISL_949116, EPI_ISL_949123, EPI_ISL_949144 to 949145, EPI_ISL_949151 to 949152, EPI_ISL_949163 to 949167, EPI_ISL_949174, EPI_ISL_949254 to 949255, EPI_ISL_953378, EPI_ISL_953380, EPI_ISL_953428 to 953429, EPI_ISL_953431 to 953435, EPI_ISL_953441, EPI_ISL_953452 to 953454, EPI_ISL_953460 to 953461, EPI_ISL_953465 to 953468, EPI_ISL_953471 to 953473, EPI_ISL_953475 to 953476, EPI_ISL_953481, EPI_ISL_953485 to 953486, EPI_ISL_953488, EPI_ISL_960177, EPI_ISL_960181, EPI_ISL_995495, EPI_ISL_995499 to 995500, EPI_ISL_995504, EPI_ISL_995507 to 995508, EPI_ISL_995513 to 995514, EPI_ISL_995517, EPI_ISL_995522 to 995525, EPI_ISL_995530, EPI_ISL_995534, EPI_ISL_995536, EPI_ISL_995544, EPI_ISL_1014904, EPI_ISL_1014907 to 1014911, EPI_ISL_1014914 to 1014915, EPI_ISL_1014921 to 1014923, EPI_ISL_1014925 to 1014926, EPI_ISL_1014928 to 1014930			
see above	Jessa	Jessa	Jessa_cmdLab
EPI_ISL_421237 to 421254, EPI_ISL_421256 to 421262, EPI_ISL_455460 to 455467	Jiangxi Province Center for Disease Control and Prevention	Jiangxi Province Center for Disease Control and Prevention	DaJin Xiao; Fang Xiao; Gang Xu; JianXiong Li; Jun Zhou; ShiWen Liu; Tian Gong; XiaoQing Liu; Xin Ran; YanNi Zhang; Ying Xiong; Yong Shi
EPI_ISL_417420	Jiangxi province Center for Disease Control and Prevention	Jiangxi province Center for Disease Control and Prevention	Li jian Xiong
EPI_ISL_412459	Jingzhou Center for Disease Control and Prevention	Hubei Provincial Center for Disease Control and Prevention	Bin Fang; Bo Yang; Bo Yu; Chunlin Mao; Faxian Zhan; Guojun Ye; Jie Hu; Jing Li; Junqiang Xu; Kun Cai; Linlin Liu; Maoyi Chen; Xiang Li; Xiao Yu; Xixiang Huo; Yongzhong Jiang.
EPI_ISL_434586 to 434588, EPI_ISL_434638 to 434640, EPI_ISL_434682 to 434691, EPI_ISL_438222 to 438226, EPI_ISL_438228 to 438231, EPI_ISL_438233 to 438235, EPI_ISL_438237, EPI_ISL_438240 to 438247, EPI_ISL_457752 to 457761, EPI_ISL_457764 to 457766, EPI_ISL_457768 to 457769, EPI_ISL_457780 to 457785, EPI_ISL_457787 to 457792, EPI_ISL_457795 to 457814, EPI_ISL_457816 to 457823, EPI_ISL_511869, EPI_ISL_511871, EPI_ISL_511874 to 511875, EPI_ISL_861099, EPI_ISL_887529, EPI_ISL_911874, EPI_ISL_911912 to 911913, EPI_ISL_981114, EPI_ISL_981116 to 981121, EPI_ISL_1036289 to 1036294			
see above	Johns Hopkins Hospital Department of Pathology	Johns Hopkins Hospital Department of Pathology	Adannaya Amadi; Amanda Ernlund; C. Paul Morris; Chun Huai Luo; Craig Howser; Heba H. Mostafa; Heba Mostafa; Kristina Zudock; Mark Hopkins; Matthew Schwartz; Melanie Kirsche; Michael C. Schatz; Michael Schatz; N_di_ Trovao; Nicholas Gallagher; Nidia Trovao; Norah Sadowski; Nídiá Trovao; Oluwaseun Falade-Nwulia; Oluwaseun Nwulia-Falade; Paul Morris; Peter M. Thielen; Shirlee Wohl; Srividya Ramakrishnan; Stuart C. Ray; Thomas Mehoke; Timothy Gilpatrick; Victoria Gniazdowski; Winston Timp; Yunfan Fan; _Amanda Ernlund; _Oluwaseun Falade-Nwulia
EPI_ISL_825013	Johns Hopkins Pathology	Johns Hopkins Hospital Department of Pathology	Adannaya Amadi; C. Paul Morris; Chun Huai Luo; Heba H. Mostafa; Nicholas Gallagher
EPI_ISL_445242	Jokkmokks Halsocentral	The Public Health Agency of Sweden	Anna Risberg; Anna-Malin Linde; Karin Tegmark-Wisell; Maria Lind Karlberg; Markus Beland; Mia Brytting; Olov Svartstrom; Oskar Karlsson Lindsjo; Theresa Enkirch
EPI_ISL_455101, EPI_ISL_469055 to 469056	Jourcentralen	The Public Health Agency of Sweden	Anna Risberg; Anna-Malin Linde; Karin Tegmark-Wisell; Maria Lind Karlberg; Mattias Haukland; Mia Brytting; Olov Svartstrom; Oskar Karlsson Lindsjo; Petra Edquist; Reza Advani; Salvatore Ascione; Sandra Broddesson; Shamam Muradrasoli; Theresa Enkirch
EPI_ISL_486397	KC General Hospital	Department of Neurovirology, National Institute of Mental Health and Neuroscience (NIMHANS)	Anita Desai; Chitra Pattabiraman; Harsha PK; Manjunatha Venkataswamy; Ravi Vasanthapuram; Risha Rasheed; Shafeeq S Hameed; Vijayalakshmi Reddy
EPI_ISL_457854, EPI_ISL_457867 to 457868, EPI_ISL_457875, EPI_ISL_457884, EPI_ISL_457897, EPI_ISL_457906, EPI_ISL_457913, EPI_ISL_457915, EPI_ISL_457920 to 457921, EPI_ISL_457928			
see above	KEMRI-CGMR-C	KEMRI-Wellcome Trust Research Programme/KEMRI-CGMR-C Kilifi	Githinji G. et al 2020
EPI_ISL_568702, EPI_ISL_568725, EPI_ISL_568727, EPI_ISL_568735, EPI_ISL_568764, EPI_ISL_568847, EPI_ISL_568872			
see above	KEMRI-Wellcome Trust Research Programme/KEMRI-CGMR-C Kilifi	KEMRI-Wellcome Trust Research Programme/KEMRI-CGMR-C Kilifi	Githinji et al 2020

EPI_ISL_436684, EPI_ISL_436686, EPI_ISL_455631 to 455633, EPI_ISL_455635 to 455636, EPI_ISL_455639, EPI_ISL_660221, EPI_ISL_660228 to 660233, EPI_ISL_660256 to 660258			
see above	KRISP, KZN Research Innovation and Sequencing Platform	KRISP, KZN Research Innovation and Sequencing Platform	Chimukangara B; Deforche K; Giandhari J; Khan S; Lessells R; Mdlalose K; Pillay S; Tegally H; Wilkinson E; York D; de Oliveira T
EPI_ISL_751572, EPI_ISL_751677, EPI_ISL_751707, EPI_ISL_751724, EPI_ISL_751730	KS Health and Environmental Laboratories	Genomics and Discovery, Respiratory Viruses Branch, Division of Viral Diseases, Centers for Disease Control and Prevention	Anna Montmayeur; Anna Uehara; Clinton R. Paden; Haibin Wang; Jing Zhang; Justin Lee; Krista Queen; Mili Sheth; Peter W. Cook; Rachel Marine; Suxiang Tong; Yan Li; Ying Tao
EPI_ISL_424867, EPI_ISL_452114	KS Health and Environmental Laboratories	Pathogen Discovery, Respiratory Viruses Branch, Division of Viral Diseases, Centers for Disease Control and Prevention	Alison S. Laufer Halpin; Anna Montmayeur; Anna Uehara; Christopher A. Elkins; Clinton R. Paden; Haibin Wang; Jing Zhang; Krista Queen; Mary S. Keckler; Rachel Marine; Suxiang Tong; Yan Li; Ying Tao; Zachary Weiner
EPI_ISL_415154	KU Leuven, Clincal and Epidemiological Virology	KU Leuven, Clincal and Epidemiological Virology	Bert Vanmechelen; Joan Marti-Careras; Marc Van Ranst; Piet Maes.; Tony Wawina
EPI_ISL_407976, EPI_ISL_415153, EPI_ISL_415155 to 415159, EPI_ISL_416467 to 416472, EPI_ISL_416475 to 416476, EPI_ISL_417422, EPI_ISL_417424 to 417430, EPI_ISL_418270, EPI_ISL_418792 to 418798, EPI_ISL_418800, EPI_ISL_418805 to 418806, EPI_ISL_418863, EPI_ISL_418981 to 418987, EPI_ISL_418989, EPI_ISL_420314 to 420454			
see above	KU Leuven, Clinical and Epidemiological Virology	KU Leuven, Clinical and Epidemiological Virology	Annabel Rector; Bert Vanmechelen; Elke Wollants; Els Keyaerts; Joan Marti-Carerras; Joan Marti-Carreras; Lies Laenen; Marc Van Ranst; Piet Maes; Tony Wawina; and Piet Maes
EPI_ISL_458156 to 458235, EPI_ISL_462151 to 462275, EPI_ISL_464065 to 464090, EPI_ISL_476941 to 477007, EPI_ISL_522349 to 522350, EPI_ISL_845566 to 845568, EPI_ISL_845571, EPI_ISL_845578 to 845581, EPI_ISL_845583 to 845584, EPI_ISL_845586, EPI_ISL_845603 to 845605, EPI_ISL_845607 to 845609, EPI_ISL_888743, EPI_ISL_894174 to 894185, EPI_ISL_894187 to 894198, EPI_ISL_894215, EPI_ISL_896447 to 896474, EPI_ISL_897988 to 898005, EPI_ISL_898007, EPI_ISL_912405 to 912422, EPI_ISL_913748 to 913766, EPI_ISL_913772, EPI_ISL_913826 to 913827, EPI_ISL_935187 to 935197, EPI_ISL_935201, EPI_ISL_935205, EPI_ISL_935208, EPI_ISL_935212 to 935214, EPI_ISL_935216 to 935230, EPI_ISL_935238, EPI_ISL_935243 to 935245, EPI_ISL_935247 to 935248, EPI_ISL_935250 to 935257, EPI_ISL_935260 to 935262, EPI_ISL_935284 to 935300, EPI_ISL_935313, EPI_ISL_935316 to 935318, EPI_ISL_935320 to 935324, EPI_ISL_935326 to 935342, EPI_ISL_935346 to 935350, EPI_ISL_935355 to 935358, EPI_ISL_995557, EPI_ISL_995559 to 995562, EPI_ISL_995565, EPI_ISL_995572 to 995574, EPI_ISL_995577, EPI_ISL_995581 to 995583, EPI_ISL_995587 to 995588, EPI_ISL_995590, EPI_ISL_995592, EPI_ISL_995595, EPI_ISL_995599 to 995602, EPI_ISL_995605, EPI_ISL_995607 to 995610, EPI_ISL_995612, EPI_ISL_995614 to 995615, EPI_ISL_995619 to 995622, EPI_ISL_995628, EPI_ISL_995634, EPI_ISL_995646 to 995647, EPI_ISL_995649 to 995650, EPI_ISL_995654, EPI_ISL_995661, EPI_ISL_995663 to 995665, EPI_ISL_995668, EPI_ISL_995678 to 995679, EPI_ISL_995692 to 995693			
see above	KU Leuven, Rega Institute, Clinical and Epidemiological Virology	KU Leuven, Rega Institute, Clinical and Epidemiological Virology	Bert Vanmechelen; Joan Marti-Carerras; Piet Maes; Tony Wawina-Bokalanga
EPI_ISL_539308	KWR Watercycle Research Institute	Erasmus Medical Center	Bas B. Oude Munnink; Claudia M. E. Schapendonk; David Nieuwenhuijse; Frank M. Aarestrup; Gertjan Medema; Goffe Elsinga; Leo Heijnen; Lu Lu; Marion P.G. Koopmans; Matthijs Kon; Miranda de Graaf; Ray Izquierdo-Lara; Samantha Lycett
EPI_ISL_751570, EPI_ISL_751594, EPI_ISL_751596, EPI_ISL_751648, EPI_ISL_751670, EPI_ISL_751754, EPI_ISL_751794			
see above	KY State Public Health Lab	Genomics and Discovery, Respiratory Viruses Branch, Division of Viral Diseases, Centers for Disease Control and Prevention	Anna Montmayeur; Anna Uehara; Clinton R. Paden; Haibin Wang; Jing Zhang; Justin Lee; Krista Queen; Mili Sheth; Peter W. Cook; Rachel Marine; Suxiang Tong; Yan Li; Ying Tao
EPI_ISL_495411 to 495414, EPI_ISL_495416 to 495418, EPI_ISL_495420 to 495423, EPI_ISL_495425 to 495426, EPI_ISL_495428 to 495439, EPI_ISL_495441 to 495445, EPI_ISL_495447 to 495457			
see above	Kafkas University, Faculty of Medicine, Department of Medical Microbiology	Kafkas University, Faculty of Medicine, Department of Medical Microbiology	Didem Ozgur; E. Ediz Tutuncu; Murat Karamese
EPI_ISL_475137 to 475138, EPI_ISL_510825 to 510826, EPI_ISL_510832 to 510833, EPI_ISL_510836 to 510837, EPI_ISL_510859, EPI_ISL_510864 to 510865, EPI_ISL_623091, EPI_ISL_648164, EPI_ISL_676534, EPI_ISL_710605			
see above	Kalmar klinisk mikrobiologi	The Public Health Agency of Sweden	Anna Risberg; Anna-Malin Linde; Department of Microbiology; Karin Tegmark-Wisell; Maria Lind Karlberg; Mattias Haukland; Mia Brytting; Olov Svartstrom; Oskar Karlsson Lindsjo; Petra Edquist; Reza Advani; Sandra Broddesson; Shamam Muradrasoli; The Public Health Agency of Sweden
EPI_ISL_687043 to 687078, EPI_ISL_690818 to 690836	Kanagawa Prefectural Institute of Public Health	Pathogen Genomics Center, National Institute of Infectious Diseases	Kentaro Itokawa; Makoto Kuroda; Masanori Hashino; Rina Tanaka; Tsuyoshi Sekizuka
EPI_ISL_1000971	Kansas Health and Environmental Lab	Kansas Health and Environmental Lab	Ben Olsen; Carissa Robertson; Mike Grose; Paige Drury; and Phil Adam
EPI_ISL_812251 to 812253	Kantonsarztamt Bern	Institute of Medical Virology, University of Zurich	Alexandra Trkola; Annette Audigé; Cyril Shah; Guido Bloemberg; Jon Huder; Jürg Böni; Kevin Steiner; Maria Grünberg; Maryam Zaheri; Michael Huber; Riccarda Capaul; Stefan Schmutz; Verena Kufner
EPI_ISL_896091, EPI_ISL_896101 to 896102, EPI_ISL_896116	Kantonsspital Aarau, Institut für Labormedizin	University Hospital Basel, Clinical Bacteriology	Adrian Egli; Alfredo Mari; Hans Hirsch; Helena MB Seth-Smith; Julia Bielicki; Karoline Leuzinger; Madlen Stange; Manuel Battegay; Michael Oberle; Tim Roloff
EPI_ISL_1039786	Kantonsspital Münsterlingen	Institute of Medical Virology, University of Zurich	Alexandra Trkola; Annette Audigé; Cyril Shah; Guido Bloemberg; Jon Huder; Jürg Böni; Kevin Steiner; Maria Grünberg; Maryam Zaheri; Michael Huber; Riccarda Capaul; Stefan Schmutz; Verena Kufner
EPI_ISL_444488 to 444489, EPI_ISL_444492	Karolinska Universitetslaboratoriet	CTMR, Karolinska Institutet, Stockholm, Sweden	Caroline Bjurnemark; Fredrik Boulund; Jing Wang; Jingkai Ji; Lars Engstrand; Linnéa Påvénius; Marica Hamsten; Shuiqin Li; Stefanie Prast-Nielsen; Vivien Lan Yang Swartz; Yinghua Zha; Yue Hu
EPI_ISL_454447 to 454493, EPI_ISL_454868 to 454902, EPI_ISL_455846 to 455901, EPI_ISL_469075, EPI_ISL_469077 to 469079, EPI_ISL_475086 to 475091, EPI_ISL_475095, EPI_ISL_475136, EPI_ISL_475141 to 475142, EPI_ISL_475149 to 475150, EPI_ISL_475544 to 475547, EPI_ISL_475551 to 475552, EPI_ISL_475558, EPI_ISL_475560 to 475561, EPI_ISL_510834 to 510835, EPI_ISL_510838 to 510840, EPI_ISL_510852, EPI_ISL_510860 to 510863, EPI_ISL_560974, EPI_ISL_615086 to 615089, EPI_ISL_676533			
see above	Karolinska Universitetslaboratoriet	The Public Health Agency of Sweden	Anna Risberg; Anna-Malin Linde; Department of Microbiology; Karin Tegmark-Wisell; Maria Lind Karlberg; Mattias Haukland; Mia Brytting; Olov Svartstrom; Oskar Karlsson Lindsjo; Petra Edquist; Reza Advani; Sandra Broddesson; Shamam Muradrasoli; Shaman Muradrasoli; The Public Health Agency of Sweden
EPI_ISL_534259, EPI_ISL_548257 to 548258, EPI_ISL_560989 to 560990	Karolinska universitetslaboratoriet	The Public Health Agency of Sweden	Anna Risberg; Anna-Malin Linde; Karin Tegmark-Wisell; Maria Lind Karlberg; Mattias Haukland; Mia Brytting; Olov Svartstrom; Oskar Karlsson Lindsjo; Petra Edquist; Reza Advani; Sandra Broddesson
EPI_ISL_534234 to 534236, EPI_ISL_560982 to 560985	Karolinska universitetslaboratoriet SOLNA	The Public Health Agency of Sweden	Anna Risberg; Anna-Malin Linde; Karin Tegmark-Wisell; Maria Lind Karlberg; Mattias Haukland; Mia Brytting; Olov Svartstrom; Oskar Karlsson Lindsjo; Petra Edquist; Reza Advani; Sandra Broddesson
EPI_ISL_442044	Kawsar Human Genetic Research Center	Kawsar Human Genetic Research Center	Fatemeh Hosseini; Maryam Abbasalipour Bashash; Mohammad Ali Khosravi; Sirous Zeinali; Solmaz Sabeghi; Yeganeh Haghdoost; Yeganeh Keshvar
EPI_ISL_469287 to 469296	Keio University Hospital	Keio University Hospital	Kenjiro Kosaki
EPI_ISL_437601, EPI_ISL_438947 to 438949, EPI_ISL_438951 to 438956, EPI_ISL_438958 to 438959, EPI_ISL_438961, EPI_ISL_438965 to 438967, EPI_ISL_438969 to 438972			
see above	Keio University School of Medicine	Keio University School of Medicine	; Haruhiko Siomi; Kenjiro Kosaki; Toshiki Takenouchi; Yuka Iwasaki
EPI_ISL_512811 to 512818, EPI_ISL_512820 to 512821	Kenema Government Hospital, Ministry of Health and Sanitation	Kenema Government Hospital, Ministry of Health and Sanitation	Andersen, K.; Garry, R.; Goba, A.; Grant, D.; Happi, C.; Jalloh, S.; Mehta, S.; Momoh, M.; Olawoye, I.; Oluniyi, P.; Park, D.; Sandi, J.; Siddle, K.; Tomkins-Tinch, C.
EPI_ISL_640093	Kensington CDC wc KSC	NHLS/UCT	Arash Iranzadeh; Bruna Galvao; Carolyn Williamson; Deelan Doolabh; Diana Hardie; Innocent Mudau; Kruger Marais; Lynn Tyers; Marvin Hsiao; Stephen Korsman
EPI_ISL_983085	Kentucky State Public Health Lab	Kentucky State Public Health Lab	Joshua Tobias; Karim George; Matthew Johnson; Rachel Zinner; Rhonda Lucas; Stephanie Lunn; Vaneet Arora; William Grooms
EPI_ISL_813656	Kettering General Hospital	COVID-19 Genomics UK (COG-UK) Consortium	Christopher Moore; Fei Sang; Johnny Debebe; Jonathan Ball; Joseph Chappell; Matthew Carlisle; Matthew Loose; Nadine Holmes; Patrick McClure; Theocharis Tsoleridis; Victoria Wright
EPI_ISL_414510	Key Laboratory of Medical Molecular Virology (MOE/NHC/CAMS), School of Basic Medicine, Shanghai Medical College, Fudan University	Key Laboratory of Medical Molecular Virology (MOE/NHC/CAMS), School of Basic Medicine, Shanghai Medical College, Fudan University	Cai, X.; Chen, H.; Fang, F.; Feng, F.; Fu, C.; Gu, C.; Han, W.; Li, C.; Qu, D.; Song, W.; Sun, Z.; Teng, Z.; Wang, Y.; Xie; Xu, W.; Y. and Yuan, Z.; Ye, R.; Yi, Z.; Zhang, R.; Zhang, X.; Zhou, Y.; Zhu, Y.
EPI_ISL_700423, EPI_ISL_700464, EPI_ISL_700468, EPI_ISL_700485, EPI_ISL_700505, EPI_ISL_700540	Khayelethu Clinic wc KLC	NHLS/UCT	Arash Iranzadeh; Bruna Galvao; Carolyn Williamson; Deelan Doolabh; Diana Hardie; Houriiyah Tegally; Innocent Mudau; Kruger Marais; Lynn Tyers; Marvin Hsiao; Stephen Korsman
EPI_ISL_825146, EPI_ISL_825151,	Kidwai Memorial Institute of Oncology	Department of Neurovirology, National Institute of Mental	Anita S Desai; Chitra Pattabiraman; Darshan Sreenivas; Nakka Vijay Kiran Reddy; Pramada Prasad; Risha Rasheed; V Ravi

EPI_ISL_845881		Health and Neurosciences (NIMHANS)	
EPI_ISL_489996 to 490012	King Fahad Medical City	King Fahad Medical City	Alghoraibi, M.; Alosaimi, B.; Enani, M.; Naeem, A.
EPI_ISL_516940, EPI_ISL_516942 to 516943, EPI_ISL_516946, EPI_ISL_516948 to 516949, EPI_ISL_516969, EPI_ISL_516974, EPI_ISL_516976 to 516977, EPI_ISL_516980 to 516984, EPI_ISL_516986			
see above	King Georges Medical University	CSIR-National Botanical Research Institute	Amita Jain; Babita Singh; Danish Nasar Khan; Hricha Mishra; Kishan Sahu; MLB Bhatt; Mehar H.Asif; Om Prakash; Priti Prasad; SK Barik; Samir V. Sawant; Shantanu Prakash; Sumit Kr. Bag; Suruchi Shukla
EPI_ISL_458030 to 458044, EPI_ISL_471583 to 471584	King Institute of Preventive Medicine & Research	CSIR-Centre for Cellular and Molecular Biology	Archana Bharadwaj Siva; Dhiviya Vedagiri; Divya Gupta; Divya Tej Sowpati; G. Dhinakar Raj; G. Ravikumar; K Thangaraj; K.Kaveri; Karthik Bharadwaj Tallapaka; Krishnan Harinivas Harshan; Lamuk Zaveri; M. Sekar; Namami Gaur; P. Azhahianambi; P.Padmapriya; Payel Mukherjee; Priya Singh; Purushotham Vodnala; R. P. Aravindh Babu; R.Kiruba; Rakesh K Mishra; S.Magesh; S.Sivasubramanian; S.Vennila; Sakshi Shambhavi; Santosh Kumar Kuncha; Shagufta Khan; Sofia Banu; Tulasi Nagabandi; Vishal Sah
EPI_ISL_483542 to 483559, EPI_ISL_483562 to 483565, EPI_ISL_483639	Kingdom of Bahrein Ministry of Health	Erasmus Medical Center	Amjad Ghanem Mohamed; Anne van der Linden; Bas Oude Munnink; Claudia Schapendonk; David Nieuwenhuijse; Ebrahim Shehad; Fatema; Hashmeya Al Wasti; Irina Chestakova; Marion Koopmans; Mark Pronk; Pascal Lexmond; Reina Sikkema; Richard Molenkamp; Stefan van Nieuwkoop; Theo Bestebroer; on behalf of the Dutch national COVID-19 response team.
EPI_ISL_459866 to 459869, EPI_ISL_459871 to 459875, EPI_ISL_459877, EPI_ISL_459879 to 459883, EPI_ISL_459885 to 459892	Kingston Health Sciences Center	Queen's Genomics Lab at Ongwanada (Q-GLO)	Ayub M; Colautti R; Evans GA; Guan H; Huang D; Hudson ML; Perez-Patrigeon S; Rustom N; Sheth P; Sjaarda CP; Soares CN; Wong H
EPI_ISL_538321 to 538333, EPI_ISL_538335 to 538336, EPI_ISL_538338 to 538343, EPI_ISL_538345 to 538351, EPI_ISL_538353, EPI_ISL_538355 to 538356, EPI_ISL_538358 to 538365, EPI_ISL_538367 to 538386			
see above	Kingston Health Sciences Centre / Queen's University	Ontario Institute for Cancer Research	Bernard Lam; Calvin Sjaarda; Ilinca Lungu; Jared T. Simpson; Katya Douchant; Lawrence E Heisler; Michael Laszloffy; Paul Krzyzanowski; Prameet M. Sheth; Richard de Borja; Robert Colautti
EPI_ISL_717727 to 717761	Kingston Health Sciences Centre and Queen's University	Ontario Institute for Cancer Research	Bernard Lam; Calvin Sjaarda; Ilinca Lungu; Jared T. Simpson; Katya Douchant; Lawrence E. Heisler; Michael Laszloffy; Paul Krzyzanowski; Prameet M. Sheth; Richard de Borja; Robert Colautti
EPI_ISL_479990	Kitakyushu City Institute of Health and Environmental Sciences	Pathogen Genomics Center, National Institute of Infectious Diseases	Asuka Kikuchi Kentaro Itokawa; Hajime Kamiya; Katsuya Obata; Makoto Kuroda; Masanori Hashino; Motoi Suzuki; Rina Tanaka; Tsuyoshi Sekizuka
EPI_ISL_413021	Klinik Hirslanden Zurich	Institute of Medical Virology, University of Zurich	Alexandra Trkola; Andrea Zbinden; Christian Ruef; Fiona Steiner; Gabriela Ziltener; Jon Huder; Jürg Böni; Maryam Zaheri; Michael Huber; Patrick Redli; Riccarda Capaul; Stefan Schmutz; Verena Kufner
EPI_ISL_524479	Klinik Hirslanden Zürich	Institute of Medical Virology, University of Zurich	Alexandra Trkola; Andrea Zbinden; Fiona Steiner; Gabriela Ziltener; Jon Huder; Jürg Böni; Maryam Zaheri; Michael Huber; Patrick Redli; Riccarda Capaul; Stefan Schmutz; Verena Kufner
EPI_ISL_475910 to 475915, EPI_ISL_934652 to 934654, EPI_ISL_1008297	Klinikum Wels-Grieskirchen	Bergthaler laboratory, CeMM Research Center for Molecular Medicine of the Austrian Academy of Sciences	Alexander Lercher; Alexandra Popa; Andreas Bergthaler; Anna Schedl; Bekir Erguner; Benedikt Agerer; Christoph Bock; Daniela Schmid; Dorothee von Laer; Elisabeth Puchhammer-Stoeckl; Franz Allerberger; Gregor Hörmann; Guenter Weiss; Henrique Colaco; Jakob-Wendelin Genger; Jan Laine; Judith Aberle; Kinga Rigler-Hohenwarter; Lukas Endler; Maelle Le Moing; Manfred Nairz; Mark Smyth; Martin Senekowitsch; Michael Schuster; Peter Hufnagl; Rainer Gattringer; Stephan Aberle; Thomas Penz; Wegene Borena
EPI_ISL_475101 to 475104, EPI_ISL_476140 to 476142, EPI_ISL_582808, EPI_ISL_615108, EPI_ISL_654954, EPI_ISL_660427 to 660431, EPI_ISL_661301, EPI_ISL_676492, EPI_ISL_676497 to 676501, EPI_ISL_766696, EPI_ISL_766713, EPI_ISL_934393, EPI_ISL_934409, EPI_ISL_1008559 to 1008561, EPI_ISL_1008563, EPI_ISL_1008566			
see above	Klinisk Mikrobiologi	The Public Health Agency of Sweden	Anna Risberg; Anna-Malin Linde; Carlo Berg; Department of Microbiology; Karin Tegmark-Wisell; Maria Lind Karlberg; Mattias Haukland; Mia Brytting; Noura Walai; Olov Svartstrom; Oskar Karlsson Lindsjo; Petra Edquist; Petra Holmstrom; Reza Advani; Sandra Broddesson; Shamam Muradrasoli; Sofia Stamouli; The Public Health Agency of Sweden
EPI_ISL_654892 to 654899, EPI_ISL_654942, EPI_ISL_654944 to 654951, EPI_ISL_660416 to 660421, EPI_ISL_661274 to 661278, EPI_ISL_661280 to 661281, EPI_ISL_661288 to 661289, EPI_ISL_661293, EPI_ISL_661302 to 661303, EPI_ISL_676491, EPI_ISL_676494 to 676496, EPI_ISL_676502 to 676508, EPI_ISL_676520, EPI_ISL_676531 to 676532, EPI_ISL_710595 to 710604, EPI_ISL_732654, EPI_ISL_766592 to 766593, EPI_ISL_766608 to 766610, EPI_ISL_766613, EPI_ISL_766615 to 766629, EPI_ISL_766633 to 766639, EPI_ISL_766694, EPI_ISL_766707, EPI_ISL_766710 to 766712, EPI_ISL_766714, EPI_ISL_766725 to 766730, EPI_ISL_788989 to 788990, EPI_ISL_831941 to 831942, EPI_ISL_913258 to 913262, EPI_ISL_913264 to 913265, EPI_ISL_913268 to 913274, EPI_ISL_913453, EPI_ISL_913457 to 913458, EPI_ISL_913460, EPI_ISL_934359 to 934360, EPI_ISL_934375, EPI_ISL_934378 to 934379, EPI_ISL_934381 to 934382, EPI_ISL_934385 to 934386, EPI_ISL_1008400, EPI_ISL_1008413, EPI_ISL_1008431, EPI_ISL_1008450, EPI_ISL_1008526, EPI_ISL_1008528			
see above	Klinisk mikrobiologi	The Public Health Agency of Sweden	Anna Risberg; Anna-Malin Linde; Carlo Berg; Department of Microbiology; Karin Tegmark-Wisell; Maria Lind Karlberg; Mattias Haukland; Mia Brytting; Noura Walai; Olov Svartstrom; Oskar Karlsson Lindsjo; Petra Edquist; Petra Holmstrom; Reza Advani; Sandra Broddesson; Sofia Stamouli; The Public Health Agency of Sweden
EPI_ISL_615097 to 615100, EPI_ISL_623092 to 623094	Klinisk mikrobiologi Lanssjukhuset Ryhov, Jonkoping	The Public Health Agency of Sweden	Anna Risberg; Anna-Malin Linde; Karin Tegmark-Wisell; Maria Lind Karlberg; Mattias Haukland; Mia Brytting; Olov Svartstrom; Oskar Karlsson Lindsjo; Petra Edquist; Reza Advani; Sandra Broddesson
EPI_ISL_623089	Klinisk mikrobiologi Linkoping	The Public Health Agency of Sweden	Anna Risberg; Anna-Malin Linde; Karin Tegmark-Wisell; Maria Lind Karlberg; Mattias Haukland; Mia Brytting; Olov Svartstrom; Oskar Karlsson Lindsjo; Petra Edquist; Reza Advani; Sandra Broddesson
EPI_ISL_510841 to 510844, EPI_ISL_510868 to 510870, EPI_ISL_548250 to 548251, EPI_ISL_548253 to 548254, EPI_ISL_560973, EPI_ISL_615090 to 615091, EPI_ISL_615109, EPI_ISL_623088, EPI_ISL_648169, EPI_ISL_648173			
see above	Klinisk mikrobiologi NAL Trollhattan	The Public Health Agency of Sweden	Anna Risberg; Anna-Malin Linde; Karin Tegmark-Wisell; Maria Lind Karlberg; Mattias Haukland; Mia Brytting; Olov Svartstrom; Oskar Karlsson Lindsjo; Petra Edquist; Reza Advani; Sandra Broddesson
EPI_ISL_429136 to 429148, EPI_ISL_429150 to 429158, EPI_ISL_429160, EPI_ISL_430860 to 430861, EPI_ISL_430863	Klinisk mikrobiologi Orebro	The Public Health Agency of Sweden	Anna Risberg; Anna-Malin Linde; Karin Tegmark-Wisell; Maria Lind Karlberg; Martin Sundqvist; Olov Svartstrom; Oskar Karlsson Lindsjo; Shaman Muradrasoli
EPI_ISL_582806 to 582807, EPI_ISL_648193	Klinisk mikrobiologi SAS Boras	The Public Health Agency of Sweden	Anna Risberg; Anna-Malin Linde; Karin Tegmark-Wisell; Maria Lind Karlberg; Mattias Haukland; Mia Brytting; Olov Svartstrom; Oskar Karlsson Lindsjo; Petra Edquist; Reza Advani; Sandra Broddesson
EPI_ISL_475144 to 475148, EPI_ISL_475153 to 475155, EPI_ISL_475160 to 475163, EPI_ISL_510818 to 510819, EPI_ISL_510853 to 510858, EPI_ISL_582797 to 582798, EPI_ISL_582800, EPI_ISL_582835 to 582838, EPI_ISL_623090, EPI_ISL_648162, EPI_ISL_654504 to 654505, EPI_ISL_710594			
see above	Klinisk mikrobiologi Vasternorrland	The Public Health Agency of Sweden	Anna Risberg; Anna-Malin Linde; Department of Microbiology; Karin Tegmark-Wisell; Maria Lind Karlberg; Mattias Haukland; Mia Brytting; Olov Svartstrom; Oskar Karlsson Lindsjo; Petra Edquist; Reza Advani; Sandra Broddesson; Shamam Muradrasoli; The Public Health Agency of Sweden
EPI_ISL_510820 to 510824, EPI_ISL_510867, EPI_ISL_548252, EPI_ISL_560975, EPI_ISL_648133	Klinisk mikrobiologi centralsjukhuset Karlstad	The Public Health Agency of Sweden	Anna Risberg; Anna-Malin Linde; Karin Tegmark-Wisell; Maria Lind Karlberg; Mattias Haukland; Mia Brytting; Olov Svartstrom; Oskar Karlsson Lindsjo; Petra Edquist; Reza Advani; Sandra Broddesson
EPI_ISL_429115 to 429125, EPI_ISL_430848 to 430855, EPI_ISL_450834	Klinisk mikrobiologi och vardhygien Halmstad	The Public Health Agency of Sweden	Anna Risberg; Anna-Malin Linde; Arne Kotz; Karin Tegmark-Wisell; Maria Lind Karlberg; Mia Brytting; Olov Svartstrom; Oskar Karlsson Lindsjo; Shaman Muradrasoli; Theresa Enkirch
EPI_ISL_766692 to 766693	Klinisk mikrobiologi, Laboratoriemedicin	The Public Health Agency of Sweden	Department of Microbiology; The Public Health Agency of Sweden
EPI_ISL_534256, EPI_ISL_560988	Klinisk mikrobiologi, Laboratoriemedicin Gavleborg	The Public Health Agency of Sweden	Anna Risberg; Anna-Malin Linde; Karin Tegmark-Wisell; Maria Lind Karlberg; Mattias Haukland; Mia Brytting; Olov Svartstrom; Oskar Karlsson Lindsjo; Petra Edquist; Reza Advani; Sandra Broddesson
EPI_ISL_984717 to 984732, EPI_ISL_984735, EPI_ISL_984952, EPI_ISL_984957 to 984958, EPI_ISL_984960, EPI_ISL_984962, EPI_ISL_984964, EPI_ISL_984966, EPI_ISL_984971 to 984978, EPI_ISL_985322 to 985323, EPI_ISL_985329, EPI_ISL_985332, EPI_ISL_985336 to 985337, EPI_ISL_985347, EPI_ISL_985350 to 985352, EPI_ISL_985354 to 985355, EPI_ISL_985358 to 985361, EPI_ISL_985363, EPI_ISL_985366, EPI_ISL_985368 to 985369, EPI_ISL_985372 to 985373, EPI_ISL_985377, EPI_ISL_985380 to 985381, EPI_ISL_985387 to 985389			
see above	Klinisk mikrobiologi, Region Västerbotten	CBRN Defence and Security, Swedish Defence Research Agency	Andreas Sjödin; Anna-Lena Johansson; Annika Allard; Annika Osterman; Carolin Öhrman; David Sundell; Emelie Näslund Salomonsson; Ingrid Dacklin; Jonas Näslund; Kerstin Myrtennäs; Linda Karlsson; Malin Granberg; Mats Forsman; Stina Bäckman
EPI_ISL_424703, EPI_ISL_428148, EPI_ISL_428201, EPI_ISL_548962 to 548963, EPI_ISL_549024, EPI_ISL_549176	Klinisk mikrobiologi, Region Västerbotten	Unit for Biological Agents, Department for CBRN Defence and Security, Swedish Defence Research Agency	FOI Bioinformatics team; FOI bioinformatics team

EPI_ISL_615080 to 615085, EPI_ISL_623080 to 623085	Klinisk mikrobiologi, Skanes universitetssjukhus, Lund	The Public Health Agency of Sweden	Anna Risberg; Anna-Malin Linde; Karin Tegmark-Wisell; Maria Lind Karlberg; Mattias Haukland; Mia Brytting; Olov Svartstrom; Oskar Karlsson Lindsjo; Petra Edquist; Reza Advani; Sandra Broddesson
EPI_ISL_454903, EPI_ISL_455904 to 455907	Klinisk mikrobiologi, UAS	The Public Health Agency of Sweden	Anna Risberg; Anna-Malin Linde; Karin Tegmark-Wisell; Maria Lind Karlberg; Mattias Haukland; Olov Svartstrom; Oskar Karlsson Lindsjo; Petra Edquist; Reza Advani; Shamam Muradrasoli
EPI_ISL_661290, EPI_ISL_676515, EPI_ISL_766630 to 766632	Klinisk mikrobiologi, Viruslab	The Public Health Agency of Sweden	Department of Microbiology; The Public Health Agency of Sweden
EPI_ISL_766697	Klinisk mikrobiologi, bakteriologi	The Public Health Agency of Sweden	Department of Microbiology; The Public Health Agency of Sweden
EPI_ISL_534245, EPI_ISL_560987	Kliniskt mikrobiologiska laboratoriet	The Public Health Agency of Sweden	Anna Risberg; Anna-Malin Linde; Karin Tegmark-Wisell; Maria Lind Karlberg; Mattias Haukland; Mia Brytting; Olov Svartstrom; Oskar Karlsson Lindsjo; Petra Edquist; Reza Advani; Sandra Broddesson
EPI_ISL_510871 to 510877, EPI_ISL_560976, EPI_ISL_582771 to 582775, EPI_ISL_594149 to 594153, EPI_ISL_615110, EPI_ISL_615117 to 615119, EPI_ISL_654952, EPI_ISL_660384 to 660414, EPI_ISL_661279, EPI_ISL_661284 to 661285, EPI_ISL_676512, EPI_ISL_676519			
see above	Klinisk mikrobiologi Linkoping	The Public Health Agency of Sweden	Anna Risberg; Anna-Malin Linde; Department of Microbiology; Karin Tegmark-Wisell; Maria Lind Karlberg; Mattias Haukland; Mia Brytting; Olov Svartstrom; Oskar Karlsson Lindsjo; Petra Edquist; Reza Advani; Sandra Broddesson; The Public Health Agency of Sweden
EPI_ISL_864550	Knappschaftskrankenhaus Bochum	Bundeswehr Institute of Microbiology	Alexandra Rehn; Antonios Katsounas; Enrico Georgi; Malena Bestehorn-Willmann; Markus Antwerpen; Mathias Walter; Mustafa Özçürümez; Roman Wölfel; Sabine Zange
EPI_ISL_434659, EPI_ISL_450811	Knivsta VC	The Public Health Agency of Sweden	Anna Risberg; Anna-Malin Linde; Johanna Carlson; Karin Tegmark-Wisell; Maria Lind Karlberg; Mia Brytting; Olov Svartstrom; Oskar Karlsson Lindsjo; Theresa Enkirch
EPI_ISL_700433, EPI_ISL_700436, EPI_ISL_700440, EPI_ISL_700469, EPI_ISL_700474, EPI_ISL_700526, EPI_ISL_700529, EPI_ISL_700531, EPI_ISL_700542			
see above	Knysna CDC wc WLC	NHLS/UCT	Arash Iranzadeh; Bruna Galvao; Carolyn Williamson; Deelan Doolabh; Diana Hardie; Houriiyah Tegally; Innocent Mudau; Kruger Marais; Lynn Tyers; Marvin Hsiao; Stephen Korsman
EPI_ISL_696495	Knysna CDC wc WLC & NHLS/UCT	KRISP, KZN Research Innovation and Sequencing Platform	Arash Iranzadeh; Bruna Galvao; Carolyn Williamson; Deelan Doolabh; Diana Hardie; Emanuel James San; Houriiyah Tegally; Innocent Mudau; Jennifer Giandhari; Kruger Marais; Lynn Tyers; Marvin Hsiao; Stephen Korsman; Sureshnee Pillay; Tulio de Oliveira
EPI_ISL_640022, EPI_ISL_640076, EPI_ISL_700422, EPI_ISL_700426, EPI_ISL_700429, EPI_ISL_700456, EPI_ISL_700484, EPI_ISL_700501, EPI_ISL_700509 to 700510, EPI_ISL_700583			
see above	Knysna Hospital wc KNY	NHLS/UCT	Arash Iranzadeh; Bruna Galvao; Carolyn Williamson; Deelan Doolabh; Diana Hardie; Houriiyah Tegally; Innocent Mudau; Kruger Marais; Lynn Tyers; Marvin Hsiao; Stephen Korsman
EPI_ISL_696463, EPI_ISL_696478, EPI_ISL_696491	Knysna Hospital wc KNY & NHLS/UCT	KRISP, KZN Research Innovation and Sequencing Platform	Arash Iranzadeh; Bruna Galvao; Carolyn Williamson; Deelan Doolabh; Diana Hardie; Emanuel James San; Houriiyah Tegally; Innocent Mudau; Jennifer Giandhari; Kruger Marais; Lynn Tyers; Marvin Hsiao; Stephen Korsman; Sureshnee Pillay; Tulio de Oliveira
EPI_ISL_419296, EPI_ISL_479823	Kochi Prefectural Institute of Public Health	Pathogen Genomics Center, National Institute of Infectious Diseases	Akihiko Tokaji; Hajime Kamiya; Kentaro Itokawa; Makoto Kuroda; Masanori Hashino; Motoi Suzuki; Rina Tanaka; Tsuyoshi Sekizuka
EPI_ISL_407193	Korea Centers for Disease Control & Prevention (KCDC) Center for Laboratory Control of Infectious Diseases Division of Viral Diseases	Korea Centers for Disease Control & Prevention (KCDC) Center for Laboratory Control of Infectious Diseases Division of Viral Diseases	Heui Man Kim; Hye-Joon Jo; Jeong-Min Kim; Mi-Seon Kim; Myung Guk Han; Namjoo Lee; SangHee Woo; Sehee Park; Yoon-Seok Chung
EPI_ISL_754847	Korian Villa d'Azon	National Reference Center for Viruses of Respiratory Infections, Institut Pasteur, Paris	Angela Brisebarre; Camille Capel; Etienne Simon-Lorière; Marion Barbet; Maud Vanpeene; Méline Bizard; Sylvie Behillil; Sylvie van der Werf; Vincent Enouf
EPI_ISL_480103 to 480108, EPI_ISL_480221 to 480223	Koshigaya City Public Health Center	Pathogen Genomics Center, National Institute of Infectious Diseases	Aya Tamura; Hajime Kamiya; Kentaro Itokawa; Kyohei Sakata; Makoto Kuroda; Masanori Hashino; Motoi Suzuki; Rina Tanaka; Takumi Daimon; Tsuyoshi Sekizuka; Yoko Togawa; Yoshiko Hamada; Yuka Furui
EPI_ISL_696460	Kranshoek Clinic wc KSH & NHLS/UCT	KRISP, KZN Research Innovation and Sequencing Platform	Arash Iranzadeh; Bruna Galvao; Carolyn Williamson; Deelan Doolabh; Diana Hardie; Emanuel James San; Houriiyah Tegally; Innocent Mudau; Jennifer Giandhari; Kruger Marais; Lynn Tyers; Marvin Hsiao; Stephen Korsman; Sureshnee Pillay; Tulio de Oliveira
EPI_ISL_434657	Kristianstadkliniken	The Public Health Agency of Sweden	Anna Risberg; Anna-Malin Linde; Karin Tegmark-Wisell; Maria Lind Karlberg; Mia Brytting; Mia Settergren Hammer; Olov Svartstrom; Oskar Karlsson Lindsjo; Theresa Enkirch
EPI_ISL_434651	Krokoms Halsocentral	The Public Health Agency of Sweden	Anna Risberg; Anna-Malin Linde; Karin Tegmark-Wisell; Maria Lind Karlberg; Martin Ersson; Mia Brytting; Olov Svartstrom; Oskar Karlsson Lindsjo; Theresa Enkirch
EPI_ISL_479991 to 479996	Kumamoto City Public Health Research Institute	Pathogen Genomics Center, National Institute of Infectious Diseases	Hajime Kamiya; Kaori Tashiro; Kentaro Itokawa; Makoto Kuroda; Masanori Hashino; Motoi Suzuki; Rina Tanaka; Tsuyoshi Sekizuka
EPI_ISL_479824	Kumamoto PrefecturalInstituteof Public-Health and Environmental Science	Pathogen Genomics Center, National Institute of Infectious Diseases	Hajime Kamiya; Kentaro Itokawa; Makoto Kuroda; Masanori Hashino; Motoi Suzuki; Rina Tanaka; Shunsuke Yahiro; Tsuyoshi Sekizuka
EPI_ISL_515181 to 515183	Kumasi Centre for Collaborative Research in Tropical Medicine, Kumasi.	Institute of Virology, Charité - Universitätsmedizin Berlin	Augustina Sylverken; Christian Drosten; Eric Adu; Jesse Addo Asamoah; Julia Schneider; Jörn Beheim-Schwarzbach; Michael Owusu; Philip El-Duah; Richard Phillips.; Richmond Gorman; Richmond Yeboah; Sherihane Aryeetey; Victor Max Corman
EPI_ISL_735492	Kumilla Medical College	Central Biological Research Laboratory and Department of Biochemistry and Molecular Biology	H. M. Abdullah Al Masud; Imam Hossen; Md. Arif Hossain; Md. Imranul Hoq; Md. Khondakar Raziur Rahman; Md. Omer Faruq; Mohammad Omar Faruque; Robiul Hasan Bhuiyan; Sajib Rudra; Shanta Paul
EPI_ISL_539881	Kungsbacka Narakut	The Public Health Agency of Sweden	Anna Risberg; Anna-Malin Linde; Karin Tegmark-Wisell; Maria Lind Karlberg; Mattias Haukland; Mia Brytting; Olov Svartstrom; Oskar Karlsson Lindsjo; Reza Advani; Sandra Broddesson; Theresa Enkirch
EPI_ISL_434644, EPI_ISL_450809, EPI_ISL_469067, EPI_ISL_475542, EPI_ISL_475569	Kungsholmsdoktorn	The Public Health Agency of Sweden	Anna Risberg; Anna-Malin Linde; Karin Tegmark-Wisell; Linus Hammar; Maria Lind Karlberg; Mattias Haukland; Mia Brytting; Olov Svartstrom; Oskar Karlsson Lindsjo; Petra Edquist; Reza Advani; Sandra Broddesson; Shamam Muradrasoli; Theresa Enkirch
EPI_ISL_434668, EPI_ISL_445232, EPI_ISL_455102, EPI_ISL_475529 to 475532, EPI_ISL_475568	Kungsors VC	The Public Health Agency of Sweden	Anna Risberg; Anna-Malin Linde; Jessica Karlsson; Karin Tegmark-Wisell; Maria Lind Karlberg; Mattias Haukland; Mia Brytting; Olov Svartstrom; Oskar Karlsson Lindsjo; Reza Advani; Sandra Broddesson; Theresa Enkirch
EPI_ISL_747239	Kuningan Publlic Health Office	West Java Health Laboratory; School of Life Sciences and Technology, Institut Teknologi Bandung	Azzania Fibriani; Cut Nur Cinthia Alamanda; Ema Rahmawati; Isak Solihin; Karimatu Khoirunnisa; Miftahul Faridl; Rifky Waluyajati Rachman; Rini Robiani; Ryan Bayusantika Ristandi
EPI_ISL_700532	Kwamandlenkosi Clinic wc KMN	NHLS/UCT	Arash Iranzadeh; Bruna Galvao; Carolyn Williamson; Deelan Doolabh; Diana Hardie; Innocent Mudau; Kruger Marais; Lynn Tyers; Marvin Hsiao; Stephen Korsman
EPI_ISL_700437, EPI_ISL_700495, EPI_ISL_700535, EPI_ISL_700537, EPI_ISL_700541, EPI_ISL_700579	Kwanokuthula CDC wc KWA	NHLS/UCT	Arash Iranzadeh; Bruna Galvao; Carolyn Williamson; Deelan Doolabh; Diana Hardie; Houriiyah Tegally; Innocent Mudau; Kruger Marais; Lynn Tyers; Marvin Hsiao; Stephen Korsman
EPI_ISL_960123	Kwanokuthula CDC wc KWA	National Health Laboratory Service/UCT	Arash Iranzadeh; Bruna Galvao; Carolyn Williamson; Deelan Doolabh; Diana Hardie; Innocent Mudau; Kruger Marais; Lynn Tyers; Marvin Hsiao; Stephen Korsman
EPI_ISL_539805	Kwong Wah Hospital	Hong Kong Department of Health	Alan K.L. Tsang; Dominic N.C. Tsang; Edman T.K. Lam; Peter C.W. Yip; Rickjason C.W. Chan
EPI_ISL_420039 to 420040	L'Air du Temps	National Reference Center for Viruses of Respiratory Infections, Institut Pasteur, Paris	Angela Brisebarre; Etienne Simon-Lorière; Flora Donati; Marion Barbet; Maud Vanpeene; Mélanie Albert; Méline Bizard; Sylvie Behillil; Sylvie van der Werf; Vincent Enouf
EPI_ISL_508931	L'Hôpital Nord-Ouest Tarare-Grandris	CNR Virus des Infections Respiratoires - France SUD	Alexandre Gaymard; Antonin Bal; Bruno Lina; Carine Moustaud; Florence Morfin-Sherpa; Gregory Destras; Gwendolyne Burfin; Laurence Josset; Martine

			Valette; Maude Bouscambert-Duchamp; Raphaelle Lamy; Solenne Brun
EPI_ISL_913667	LA Office of Public Health Laboratories	Centers for Disease Control and Prevention Division of Viral Diseases, Pathogen Discovery	Anna Uehara; Clinton R. Paden; Haibin Wang; Jing Zhang; Krista Queen; Peter Cook; Suxiang Tong; Yan Li; Ying Tao
EPI_ISL_751620, EPI_ISL_751669, EPI_ISL_751696, EPI_ISL_751722, EPI_ISL_751752	LA Office of Public Health Laboratories	Genomics and Discovery, Respiratory Viruses Branch, Division of Viral Diseases, Centers for Disease Control and Prevention	Anna Montmayeur; Anna Uehara; Clinton R. Paden; Haibin Wang; Jing Zhang; Justin Lee; Krista Queen; Mili Sheth; Peter W. Cook; Rachel Marine; Suxiang Tong; Yan Li; Ying Tao
EPI_ISL_424868, EPI_ISL_452106 to 452107, EPI_ISL_535305 to 535358, EPI_ISL_913997 to 913998	LA Office of Public Health Laboratories	Pathogen Discovery, Respiratory Viruses Branch, Division of Viral Diseases, Centers for Disease Control and Prevention	Alison S. Laufer Halpin; Anna Montmayeur; Anna Uehara; Christopher A. Elkins; Clinton Paden; Clinton R. Paden; Haibin Wang; Jing Zhang; Krista Queen; Mary S. Keckler; Peter Cook; Rachel Marine; Suxiang Tong; Yan Li; Ying Tao; Zachary Weiner
EPI_ISL_1007632	LA SOURCE	Laboratory of genomics and metagenomics, Institute of Microbiology, University Hospital Centre and University of Lausanne, Switzerland	Claire Bertelli; Damien Jacot; Gilbert Greub; Sébastien Aeby; Trestan Pillonel
EPI_ISL_981497	LABM BIOLAM CLINIQUE ESTREE	Department of Virology, Henri Mondor University Hospital, Assistance Publique Hôpitaux de Paris, Université Paris-Est Créteil, INSERM U955	Alexandre Soulier; Christophe Rodriguez; Elisabeth Trawinski; Guillaume Gricourt; Jean-Michel Pawlotsky; Melissa N'Debi; Slim Fourati; Vanessa Demontant
EPI_ISL_416499 to 416500, EPI_ISL_418234, EPI_ISL_418240	LABM GH nord Essonne	National Reference Center for Viruses of Respiratory Infections, Institut Pasteur, Paris	Angela Brisebarre; Christine Lambert; Etienne Simon-Lorière; Flora Donati; Marion Barbet; Maud Vanpeene; Mélanie Albert; Méline Bizard; Mélnie Albert; Sylvie Behillil; Sylvie van der Werf; Vincent Enouf
EPI_ISL_428354, EPI_ISL_428361, EPI_ISL_428365, EPI_ISL_443260, EPI_ISL_443305, EPI_ISL_443314	LABM GH nord Essonne de Longjumeau - BP 125	National Reference Center for Viruses of Respiratory Infections, Institut Pasteur, Paris	Angela Brisebarre; Etienne Simon-Lorière; Flora Donati; Marion Barbet; Maud Vanpeene; Mélanie Albert; Méline Bizard; Sylvie Behillil; Sylvie van der Werf; Vincent Enouf
EPI_ISL_445271	LABORATORIO CLINICA CHILLAN	Instituto de Salud Publica de Chile	Alejandra Acevedo; Andrés E Castillo; Bárbara Parra; Carolina Tambley; Gabriel Leal; Jaime Lagos; Jorge Fernandez; Loredana Arata; Patricia Bustos; Paz Tapia; Rodrigo Fasce; Winston Andrade
EPI_ISL_445333	LABORATORIO CLINICA UNIVERSITARIA DE CONCEPCION	Instituto de Salud Publica de Chile	Alejandra Acevedo; Andrés E Castillo; Bárbara Parra; Carolina Tambley; Gabriel Leal; Jaime Lagos; Jorge Fernandez; Loredana Arata; Patricia Bustos; Paz Tapia; Rodrigo Fasce; Winston Andrade
EPI_ISL_445279	LABORATORIO INMUNOLAB SPA	Instituto de Salud Publica de Chile	Alejandra Acevedo; Andrés E Castillo; Bárbara Parra; Carolina Tambley; Gabriel Leal; Jaime Lagos; Jorge Fernandez; Loredana Arata; Patricia Bustos; Paz Tapia; Rodrigo Fasce; Winston Andrade
EPI_ISL_445273 to 445274, EPI_ISL_445278	LABORATORIO TORRE MEDICA LTDA.	Instituto de Salud Publica de Chile	Alejandra Acevedo; Andrés E Castillo; Bárbara Parra; Carolina Tambley; Gabriel Leal; Jaime Lagos; Jorge Fernandez; Loredana Arata; Patricia Bustos; Paz Tapia; Rodrigo Fasce; Winston Andrade
EPI_ISL_682275 to 682277	LABORATORIOS LABIN	Inciensa, Instituto Costarricense de Investigación y Enseñanza en Nutrición y Salud	Adriana Godinez; Claudio Soto-Garita; Estela Cordero; Francisco Duarte; Hebleen Porras; Melany Calderon & Pei Ling Chan
EPI_ISL_717809, EPI_ISL_717832 to 717836, EPI_ISL_717841, EPI_ISL_717910 to 717919, EPI_ISL_717958, EPI_ISL_717963 to 717964	LACEN Dr. Francisco Rimolo Neto	Bioinformatics Laboratory / LNCC	Alexandra L Gerber; Amilcar Tanuri; Ana Paula de C Guimarães; Ana Tereza R de Vasconcelos; Andréa Cony Cavalcanti; Carolina M Voloch; Claudia dos Santos Rodrigues; Cynthia C Cardoso; Diana Mariani; Luiz G P de Almeida; Otavio Bustrolini; Ronaldo da Silva F Jr; Terezinha M P P Castiñeira
EPI_ISL_717899 to 717909, EPI_ISL_717962	LACEN RJ - Noel Nutels	Bioinformatics Laboratory / LNCC	Alexandra L Gerber; Amilcar Tanuri; Ana Paula de C Guimarães; Ana Tereza R de Vasconcelos; Andréa Cony Cavalcanti; Carolina M Voloch; Claudia dos Santos Rodrigues; Cynthia C Cardoso; Diana Mariani; Luiz G P de Almeida; Otavio Bustrolini; Ronaldo da Silva F Jr; Terezinha M P P Castiñeira
EPI_ISL_882663 to 882664	LACEN do Distrito Federal	Instituto Adolfo Lutz, Interdiciplinary Procedures Center, Strategic Laboratory	Claudia Regina Gonçalves; Claudio Tavares Sacchi; Erica Valessa Ramos Gomes; Karoline Rodrigues Campos
EPI_ISL_502779, EPI_ISL_502875	LACEN/PE	LABBE, Federal University of Pernambuco	ANTONIO CARLOS DE FREITAS; BRUNO SAMPAIO; HEIDI LACERDA ALVES DA CRUZ; MAIRA GALDINO DA ROCHA PITTA; MARCOS ANTONIO DE MORAIS JUNIOR; MARCOS DA SILVEIRA REGUEIRA NETO; MICHELLY CRISTINY PEREIRA; REGINALDO GONCALVES DE LIMA NETO; SERGIO DE SA LEITAO PAIVA JUNIOR; VALDIR DE QUEIROZ BALBINO.; WILSON JOSE DA SILVA JUNIOR; ZILDENE DE SOUSA SILVEIRA
EPI_ISL_961467 to 961469	LESP Nuevo Leon/Grupo de Diagnostico ARIES	Instituto de Diagnostico y Referencia Epidemiologicos (INDRE)	Abril Rodriguez-Maldonado; Claudia Wong-Arambula; Ernesto Ramirez-Gonzalez.; Fabiola Garces-Ayala; Gisela Barrera-Badillo; Irma Lopez-Martinez; Lucia Hernandez-Rivas; Natividad Cruz-Ortiz; Tatiana Nunez-Garcia
EPI_ISL_1039711, EPI_ISL_1039832	LHUB-ULB	UAntwerp, Laboratory of Medical Microbiology	Basil Britto Xavier; Christine Lammens; Herman Goossens; Jasmine Coppens; Marie Le Mercier; Veerle Matheeussen
EPI_ISL_498226	LIC	LIC	LIC
EPI_ISL_995548 to 995549, EPI_ISL_995551 to 995555	LKOLMC	Jessa	Jessa_cmdLab
EPI_ISL_469017, EPI_ISL_469049, EPI_ISL_469051 to 469054	LNR National Reference Laboratory, Mohammed VI University of Health Sciences	Medical Biotechnology Laboratory, Rabat Medical and Pharmacy School, Mohammed The Vth University in Rabat	Chakib NEJJARI; Houda BENRAHMA; Idrissa Diawara; Imane SMYEJ; Jalil El Atar; Jalila RAHOUI; Lahcen BELYAMANI and Azeddine IBRAHIMI; Laila SBABOU; Loubna ALLAM; M.W. CHEMAO-ELFIHRI; Meriem LAAMARTI; Mouna OUADGHIRI; Rachid EL JAOUDI; Rachid MENTAG; Rokaia LAAMRTI; Saaid AMZAZI; Souad KARTTI
EPI_ISL_435034 to 435044, EPI_ISL_435550 to 435579, EPI_ISL_451203 to 451297, EPI_ISL_455980 to 456002, EPI_ISL_574295 to 574430, EPI_ISL_578698 to 578700, EPI_ISL_578704 to 578815, EPI_ISL_578824 to 579010, EPI_ISL_579012 to 579016, EPI_ISL_579019 to 579057, EPI_ISL_653346 to 653366, EPI_ISL_653368 to 653439, EPI_ISL_653441 to 653464, EPI_ISL_653466 to 653480, EPI_ISL_653482 to 653554, EPI_ISL_653556 to 653744, EPI_ISL_666609, EPI_ISL_666613, EPI_ISL_768325 to 768363, EPI_ISL_768365 to 768393, EPI_ISL_768396 to 768404, EPI_ISL_768406 to 768472, EPI_ISL_768475 to 768490, EPI_ISL_768493 to 768516, EPI_ISL_768518 to 768520, EPI_ISL_768525			
see above	LSUHS Emerging Viral Threat Laboratory	Microbial Genome Sequencing Center	Abida Siddiqa; Adam Greer; Andrew D. Yurochko; Byeong-Jae Lee; Camille F. Abshire; Chan-ki Min; Christopher G. Kevil; Daniel J. Snyder; Edna Ondari; Jason M. Bodily; Jennifer L. Carroll; Jeremy P. Kamil; John A. Vanchiere; Katarzyna Zwolinska; Maarten Van Diest; Malgorzata Bienkowska-Haba; Martin J. Sapp; Md Maksudul Alam; Michelle M. Arnold; Monica Gestal-Cartele; Paul M. Weinberger; Rona S. Scott; Vaughn S. Cooper
EPI_ISL_528637 to 528638	LVM/UFRJ	Bioinformatics Laboratory / LNCC	Amilcar Tanuri; Ana Teresa R. Vasconcelos; Bruno B. Bezerra; Diana Marianni; Elena Cobos; Fabio Limonte; Gustavo D. P. Silva; Isadora A. Correa; Luciana B. Arruda; Luciana J. Costa; Lucio A. Caldas; Luiz Almeida; Luiza Higga; M. Romário M. de Souza; Marcelo Bozza; Orlando Ferreira; Sharton V. A. Coelho; Terezinha M. Castineiras; Wanderley de Souza
EPI_ISL_528539	LVM/UFRJ	LNCC	Amilcar Tanuri; Ana Teresa R. Vasconcelos; Bruno B. Bezerra; Diana Marianni; Elena Cobos; Fabio Limonte; Gustavo M. Romário M. de Souza; Isadora A. Correa; Luciana B. Arruda; Luciana J. Costa.; Lucio A. Caldas; Luiz Almeida; Luiza Higga; Marcelo Bozza; Orlando Ferreira; Sharton V. A. Coelho; Terezinha M. Castineiras; Wanderley de Souza
EPI_ISL_443307	La Villa Papyri	National Reference Center for Viruses of Respiratory Infections, Institut Pasteur, Paris	Angela Brisebarre; Etienne Simon-Lorière; Flora Donati; Marion Barbet; Maud Vanpeene; Mélanie Albert; Méline Bizard; Sylvie Behillil; Sylvie van der Werf; Vincent Enouf
EPI_ISL_755644 to 755645, EPI_ISL_755652	Lab LOC - Itapecerica da Serra	Instituto Adolfo Lutz, Interdiciplinary Procedures Center, Strategic Laboratory	Claudia Regina Gonçalves; Claudio Tavares Sacchi; Erica Valessa Ramos Gomes; Karoline Rodrigues Campos
EPI_ISL_501614 to 501618, EPI_ISL_501620 to 501629	Lab Microbiology, Pathology Department, William Harvey Hospital	Wellcome Sanger Institute for the COVID-19 Genomics UK (COG-UK) consortium	Cordelia Langford; David K. Jackson; Dominic Kwiatkowski; Ewan Harrison; Felicity Ryan and Alex Alderton; Hannah Lowe; Ian Johnston; John Sillitoe on behalf of the Wellcome Sanger Institute COVID-19 Surveillance Team (http://www.sanger.ac.uk/covid-team); Roberto Amato; Samuel Moses; Sonia Goncalves
EPI_ISL_846545 to 846546, EPI_ISL_846553, EPI_ISL_846559, EPI_ISL_846561 to 846568, EPI_ISL_977493, EPI_ISL_982253, EPI_ISL_982255, EPI_ISL_982258, EPI_ISL_982261, EPI_ISL_982263, EPI_ISL_982266 to 982267, EPI_ISL_982273, EPI_ISL_982276, EPI_ISL_982280 to 982281, EPI_ISL_982297			
see above	Lab voor klinische biologie	Lab voor klinische biologie	Bruno Verhasselt; Hannelore Hamerlinck; Marija Janevska
EPI_ISL_419259, EPI_ISL_419264 to 419266, EPI_ISL_425048, EPI_ISL_425050, EPI_ISL_425052 to 425064, EPI_ISL_434347 to 434349, EPI_ISL_434351 to 434352, EPI_ISL_434354 to 434355, EPI_ISL_434357, EPI_ISL_434360 to 434362, EPI_ISL_451164, EPI_ISL_451166 to 451170, EPI_ISL_451173, EPI_ISL_451175, EPI_ISL_451177 to 451181, EPI_ISL_468727 to 468741, EPI_ISL_468743 to 468746, EPI_ISL_475060 to 475082, EPI_ISL_481221 to 481222, EPI_ISL_481224 to 481225, EPI_ISL_481227, EPI_ISL_481229 to 481233, EPI_ISL_634878 to 634895, EPI_ISL_717603 to 717633, EPI_ISL_717991 to 718026, EPI_ISL_745202 to			

745203, EPI_ISL_745205 to 745207, EPI_ISL_745209, EPI_ISL_745211 to 745212, EPI_ISL_745214 to 745219, EPI_ISL_794825, EPI_ISL_855936, EPI_ISL_855938, EPI_ISL_855944 to 855945, EPI_ISL_855947			
see above	Lab voor klinische biologie	Onderzoeksgroep Virologie	Basiel Cole; Bruno Verhasselt; Hans Nauwynck; Jozefien De Clercq; Laurens Lambrechts; Linos Vandekerckhove; Marthe Pauwels; Nick Vereecke; Sebastiaan Theuns
EPI_ISL_456172 to 456180, EPI_ISL_456197 to 456203, EPI_ISL_456205 to 456206, EPI_ISL_456210, EPI_ISL_456212, EPI_ISL_456300, EPI_ISL_456379 to 456384, EPI_ISL_456388, EPI_ISL_456390, EPI_ISL_456393 to 456394, EPI_ISL_547969, EPI_ISL_547976, EPI_ISL_547978 to 547979, EPI_ISL_547996 to 548000, EPI_ISL_548017, EPI_ISL_548021 to 548025, EPI_ISL_548040 to 548041, EPI_ISL_548049 to 548050, EPI_ISL_548069, EPI_ISL_548077, EPI_ISL_548106, EPI_ISL_548108, EPI_ISL_548110 to 548111, EPI_ISL_548120, EPI_ISL_548141 to 548142, EPI_ISL_579105 to 579107, EPI_ISL_579109, EPI_ISL_579111, EPI_ISL_579113, EPI_ISL_579115 to 579116, EPI_ISL_579119, EPI_ISL_579122 to 579123, EPI_ISL_579156, EPI_ISL_579233, EPI_ISL_579236 to 579237, EPI_ISL_579239 to 579240, EPI_ISL_579243, EPI_ISL_579245 to 579249, EPI_ISL_579251 to 579254, EPI_ISL_579256, EPI_ISL_579260 to 579274, EPI_ISL_579277 to 579284, EPI_ISL_579286, EPI_ISL_579288 to 579292, EPI_ISL_579315, EPI_ISL_579320 to 579323, EPI_ISL_579326, EPI_ISL_579328, EPI_ISL_579330, EPI_ISL_579332, EPI_ISL_579334 to 579335, EPI_ISL_579337 to 579338, EPI_ISL_579340 to 579341, EPI_ISL_579344 to 579346, EPI_ISL_579349 to 579352, EPI_ISL_579354, EPI_ISL_579357 to 579367, EPI_ISL_579374 to 579376, EPI_ISL_579378, EPI_ISL_579403, EPI_ISL_579405, EPI_ISL_579415 to 579417, EPI_ISL_579425, EPI_ISL_582017, EPI_ISL_622803, EPI_ISL_622807, EPI_ISL_622814 to 622816, EPI_ISL_622821 to 622823, EPI_ISL_626352 to 626356, EPI_ISL_637093 to 637094, EPI_ISL_661258 to 661259, EPI_ISL_661262, EPI_ISL_661264, EPI_ISL_682291 to 682294, EPI_ISL_707795, EPI_ISL_707797 to 707800, EPI_ISL_707802 to 707805, EPI_ISL_732964 to 732967, EPI_ISL_755629 to 755633, EPI_ISL_755637, EPI_ISL_794610, EPI_ISL_794613, EPI_ISL_794615 to 794619, EPI_ISL_877206 to 877207, EPI_ISL_877209 to 877210, EPI_ISL_877212, EPI_ISL_877225 to 877226, EPI_ISL_1016858 to 1016862, EPI_ISL_1016864, EPI_ISL_1016867, EPI_ISL_1016873, EPI_ISL_1016877			
see above	LabPLUS	Institute of Environmental Science and Research (ESR)	Anja Werno; Antje van der Linden; Arlo Upton; Chris Mansell; David Hammer; Dragana Drinkovic; Erasmus Smit; Gary McAuliffe; Hana Sofia Andersson; Hermes Perez; James Ussher; Jill Sherwood; Jing Wang; Joep de Ligt; Josh Freeman; Julia Howard; Juliet Elvy; Lauren Jelly; Mary DeAlmeida; Matt Blakiston; Matt Storey; Matthew Rogers; Max Bloomfield; Michael Addidle; Michelle Balm; Muhammad Faisal; Nikki Freed; Olin Silander; Sally Roberts; Sarah Jefferies; Sharmini Muttaiyah; Susan Morpeth; Susan Taylor; Timothy Blackmore; Vani Sathyendran; Veronica Playle; Virginia Hope; Xiaoyun Ren
EPI_ISL_547970 to 547975, EPI_ISL_547977, EPI_ISL_547993 to 547995, EPI_ISL_548013 to 548016, EPI_ISL_548046, EPI_ISL_548104, EPI_ISL_579099, EPI_ISL_579421 to 579424, EPI_ISL_637084, EPI_ISL_637096, EPI_ISL_1016856			
see above	LabTests	Institute of Environmental Science and Research (ESR)	Anja Werno; Antje van der Linden; Arlo Upton; Chris Mansell; David Hammer; Dragana Drinkovic; Erasmus Smit; Gary McAuliffe; Hana Sofia Andersson; Hermes Perez; James Ussher; Jill Sherwood; Jing Wang; Joep de Ligt; Josh Freeman; Julia Howard; Juliet Elvy; Lauren Jelly; Mary DeAlmeida; Matt Blakiston; Matt Storey; Matthew Rogers; Max Bloomfield; Michael Addidle; Michelle Balm; Muhammad Faisal; Nikki Freed; Olin Silander; Sally Roberts; Sarah Jefferies; Sharmini Muttaiyah; Susan Morpeth; Susan Taylor; Timothy Blackmore; Vani Sathyendran; Veronica Playle; Virginia Hope; Xiaoyun Ren
EPI_ISL_560637 to 560642, EPI_ISL_846641 to 846643, EPI_ISL_846645 to 846647, EPI_ISL_855381, EPI_ISL_860833 to 860841, EPI_ISL_860846, EPI_ISL_860852 to 860859, EPI_ISL_860889, EPI_ISL_872239, EPI_ISL_872256, EPI_ISL_872260, EPI_ISL_872320 to 872321, EPI_ISL_872323 to 872324, EPI_ISL_883959, EPI_ISL_883962 to 883963, EPI_ISL_883997, EPI_ISL_890354, EPI_ISL_909665, EPI_ISL_909667 to 909668, EPI_ISL_912549 to 912553, EPI_ISL_912569 to 912572, EPI_ISL_912603 to 912605, EPI_ISL_912607 to 912611, EPI_ISL_935527 to 935533, EPI_ISL_935537 to 935538, EPI_ISL_935546, EPI_ISL_935549, EPI_ISL_935560, EPI_ISL_935563, EPI_ISL_935590, EPI_ISL_935640, EPI_ISL_935643, EPI_ISL_935650 to 935651, EPI_ISL_940997 to 940999, EPI_ISL_941004 to 941010, EPI_ISL_941028 to 941031, EPI_ISL_941033 to 941064, EPI_ISL_941066 to 941091, EPI_ISL_941093 to 941103, EPI_ISL_953968, EPI_ISL_953986, EPI_ISL_953988, EPI_ISL_953994, EPI_ISL_953998 to 953999, EPI_ISL_954004 to 954008, EPI_ISL_954022 to 954029, EPI_ISL_954031, EPI_ISL_954036, EPI_ISL_955073, EPI_ISL_955181, EPI_ISL_955183, EPI_ISL_995468 to 995470, EPI_ISL_995491 to 995494, EPI_ISL_1001310, EPI_ISL_1001318, EPI_ISL_1001329, EPI_ISL_1001376 to 1001380			
see above	Labo Analyses Med	National Reference Center for Viruses of Respiratory Infections, Institut Pasteur, Paris	; Amzalag Jonas; Angela Brisebarre; Besson J; Bonnaudet GéRaldine; Cady Anne; Camille Capel; Coignard Catherine; Delamare Catherine; Dynalab; Etienne Simon-Lorière; Fabiana Gambaro; Garand Anne-Laure; Gestin (B) Brieuc; Holstein Anne; Jacques Alexandra; Laboratoire Palissy; Le Berre (D) David; Le Guillou-Guillemette HéLèNe; Le Vicky; Marion Barbet; Maud Vanpeene; Merah Kader; Méline Bizard; Rouah Raquel; Selas Labomaine; Sylvie Behillil; Sylvie van der Werf; Vincent Enouf
EPI_ISL_593924 to 593929, EPI_ISL_593932 to 593933	Labo Analyses Med, Puteaux	National Reference Center for Viruses of Respiratory Infections, Institut Pasteur, Paris	Etienne Simon-Lorière; Fabiana Gambaro; Maud Vanpeene; Sylvie Behillil; Sylvie van der Werf; Vincent Enouf
EPI_ISL_593904 to 593909	Labo Analyses Med, Sarcelles	National Reference Center for Viruses of Respiratory Infections, Institut Pasteur, Paris	Etienne Simon-Lorière; Fabiana Gambaro; Maud Vanpeene; Sylvie Behillil; Sylvie van der Werf; Vincent Enouf
EPI_ISL_860842 to 860845, EPI_ISL_860860 to 860862, EPI_ISL_883965, EPI_ISL_883969, EPI_ISL_883971, EPI_ISL_909656 to 909664, EPI_ISL_909669 to 909673, EPI_ISL_909703 to 909706, EPI_ISL_935587 to 935589, EPI_ISL_935591 to 935595, EPI_ISL_935597 to 935598, EPI_ISL_935602 to 935604, EPI_ISL_935607 to 935608, EPI_ISL_935611 to 935613, EPI_ISL_935615 to 935625, EPI_ISL_935628 to 935631, EPI_ISL_935634 to 935636			
see above	Labo Analyses med	National Reference Center for Viruses of Respiratory Infections, Institut Pasteur, Paris	Amzalag Jonas; Angela Brisebarre; Besson J; Camille Capel; Etienne Simon-Lorière; Marion Barbet; Maud Vanpeene; Méline Bizard; Sylvie Behillil; Sylvie van der Werf; Vincent Enouf
EPI_ISL_420063	Labo BM - Site de Juvisy - Hopital Général	National Reference Center for Viruses of Respiratory Infections, Institut Pasteur, Paris	Angela Brisebarre; Etienne Simon-Lorière; Flora Donati; Marion Barbet; Maud Vanpeene; Mélanie Albert; Méline Bizard; Sylvie Behillil; Sylvie van der Werf; Vincent Enouf
EPI_ISL_930594 to 930595, EPI_ISL_959571 to 959579, EPI_ISL_959581 to 959582, EPI_ISL_965399, EPI_ISL_965406 to 965407, EPI_ISL_965409, EPI_ISL_965411, EPI_ISL_965419 to 965420, EPI_ISL_965469			
see above	Labo Luc Olivier	GIGA Medical Genomics	Bouchra Boujemla; Cécile Meex; Keith Durkin; Maria Artesi; Marie-Pierre Hayette; Pierrette Melin; Raphaël Boreux; Sébastien Bontems; Vincent Bours
EPI_ISL_955062 to 955063, EPI_ISL_955068 to 955072	Labo analyses Med	National Reference Center for Viruses of Respiratory Infections, Institut Pasteur, Paris	Angela Brisebarre; Camille Capel; Etienne Simon-Lorière; Marion Barbet; Maud Vanpeene; Méline Bizard; Potiron GréGoire; Sylvie Behillil; Sylvie van der Werf; Vincent Enouf
EPI_ISL_872212, EPI_ISL_872238, EPI_ISL_872264 to 872265, EPI_ISL_872309 to 872312, EPI_ISL_872314, EPI_ISL_955066 to 955067, EPI_ISL_977661 to 977663, EPI_ISL_1035919 to 1035922, EPI_ISL_1036373 to 1036375, EPI_ISL_1036409 to 1036419			
see above	Labo analyses med	National Reference Center for Viruses of Respiratory Infections, Institut Pasteur, Paris	Amzalag Jonas; Angela Brisebarre; Camille Capel; Etienne Simon-Lorière; Goubard Agathe; Marion Barbet; Maud Vanpeene; Méline Bizard; Sylvie Behillil; Sylvie van der Werf; Vincent Enouf
EPI_ISL_416029	Laboiratório Fleury	Instituto Adolfo Lutz, Interdiciplinary Procedures Center, Strategic Laboratory	Adriana Bugno; Adriano Abbud; Carlos Henrique Camargo; Claudia Regina Gonçalves; Claudio Tavares Sacchi; Daniela Bernardes Borges da Silva; Fabiana Cristina Pereira dos Santos; Maria do Carmo Sampaio Tavares Timenetsky; Simone Guadagnucci Morillo; Terezinha Maria de Paiva
EPI_ISL_831422, EPI_ISL_831425	Labor Dr. Wisplinghoff - Köln	Robert Koch Institute, Influenza and respiratory viruses FG17 & Bioinformatics MF1, Berlin, Germany	Aleksandar Radonic; Dr. R. Grosser; Marianne Wedde; Oliver Drechsel; Ralf Duerrwald; Rene Kmiecinski; Stefan Kroeger; Stephan Fuchs; Thorsten Wolff
EPI_ISL_476705, EPI_ISL_487398 to 487410, EPI_ISL_487412 to 487414, EPI_ISL_487416 to 487427, EPI_ISL_487429 to 487431	Labor Kneißler GmbH & Co. KG	Heinrich Pette Institute, Leibniz Institute for Experimental Virology	Adam Grundhoff; Manja Czech-Sioli; Matthias Ottinger; Melanie M. Brinkmann; Nicole Fischer; Thomas Günther
EPI_ISL_445220	Laborarory for Respiratory Viruses, "Cantacuzino" National Military-Medical Institute for Resararch and Development	Cantacuzino Institute	A.Cretu; L.Ustea; M.Lazar
EPI_ISL_981372	Laborarory for Respiratory Viruses, "Cantacuzino" National Military-Medical Institute for Resararch and Development	Cantacuzino Institute Virology	Catalina Pascu; Luiza Ustea; Mihaela Lazar; Nicoleta Paraschiv
EPI_ISL_768818	Laboratoire 2A2B	CNR Virus des Infections Respiratoires - France SUD	Antonin Bal; Bruno Lina; Gregory Destras; Gwendolyne Burfin; Hadrien Règue; Laurence Josset; Martine Valette; Quentin Semanas
EPI_ISL_768824	Laboratoire BIOLAB33	CNR Virus des Infections Respiratoires - France SUD	Antonin Bal; Bruno Lina; Gregory Destras; Gwendolyne Burfin; Hadrien Règue; Laurence Josset; Martine Valette; Quentin Semanas
EPI_ISL_768830	Laboratoire BIOMED 05	CNR Virus des Infections Respiratoires - France SUD	Antonin Bal; Bruno Lina; Gregory Destras; Gwendolyne Burfin; Hadrien Règue; Laurence Josset; Martine Valette; Quentin Semanas
EPI_ISL_768819 to 768820	Laboratoire BPO-BIOEPINE	CNR Virus des Infections Respiratoires - France SUD	Antonin Bal; Bruno Lina; Gregory Destras; Gwendolyne Burfin; Hadrien Règue; Laurence Josset; Martine Valette; Quentin Semanas
EPI_ISL_723469, EPI_ISL_728219, EPI_ISL_728272 to 728277, EPI_ISL_728289 to 728291, EPI_ISL_728295, EPI_ISL_728297, EPI_ISL_728301, EPI_ISL_728322, EPI_ISL_728332, EPI_ISL_728334, EPI_ISL_728339 to 728340, EPI_ISL_728342, EPI_ISL_728344, EPI_ISL_728347, EPI_ISL_728352 to 728353, EPI_ISL_728355, EPI_ISL_728360, EPI_ISL_728366 to 728367, EPI_ISL_768832, EPI_ISL_768834 to 768840, EPI_ISL_769863			
see above	Laboratoire Biolife	Laboratoire de Biotechnologie	Afaf Alaoui; Amina Benouda; Bouchra Belfquih; Hanae Dakka; Lahcen Belyamani; Lahcen belyamani; Lahecn Belyamani; Mohamed Chenaoui; Mohammed Walid Chemao Elfihri; Mouna Ouadghiri; Otmane Touzani; Saaid Amzazi and Azeddine Ibrahim; Saaid Amzazi and Azeddine Ibrahimi; Tarik Aanniz
EPI_ISL_912563 to 912564, EPI_ISL_912574	Laboratoire Central - American Hospital of Paris	National Reference Center for Viruses of Respiratory Infections, Institut Pasteur, Paris	Angela Brisebarre; Camille Capel; Etienne Simon-Lorière; Marion Barbet; Maud Vanpeene; Méline Bizard; Ramirez José-Manuel; Sylvie Behillil; Sylvie van der Werf; Vincent Enouf
EPI_ISL_1009627	Laboratoire Cerballiance Bretigny	Cerba	Benazra M; Collin T; Delaplace E; Gresset N; Haim-Boukobza S; Lecorche E; Lesenne A; Olivi M; Ricard B; Roquebert B; Trombert-Paolantoni S
EPI_ISL_1001459	Laboratoire Cerballiance Port de Bouc	Cerba	Haim-Boukobza S; Lecorche E; Meyer S; Roig JC; Roquebert B; Trombert-Paolantoni S
EPI_ISL_1001464	Laboratoire Cerballiance du pole santé	Cerba	Haim-Boukobza S; Lecorche E; Meyer S; Roig JC; Roquebert B; Trombert-Paolantoni S
EPI_ISL_768817	Laboratoire Mirialis	CNR Virus des Infections Respiratoires - France SUD	Antonin Bal; Bruno Lina; Gregory Destras; Gwendolyne Burfin; Hadrien Règue; Laurence Josset; Martine Valette; Quentin Semanas

EPI_ISL_421734 to 421735, EPI_ISL_421737 to 421738, EPI_ISL_421740 to 421748, EPI_ISL_421750, EPI_ISL_421752 to 421757, EPI_ISL_421759 to 421762, EPI_ISL_428939 to 428940, EPI_ISL_428943 to 428945, EPI_ISL_428949, EPI_ISL_428954, EPI_ISL_428956, EPI_ISL_428959 to 428962, EPI_ISL_429706 to 429707, EPI_ISL_429710 to 429711, EPI_ISL_429713, EPI_ISL_429715 to 429716, EPI_ISL_429718, EPI_ISL_429722 to 429725, EPI_ISL_429727 to 429730, EPI_ISL_429732 to 429738, EPI_ISL_429740 to 429743, EPI_ISL_429746, EPI_ISL_429748 to 429750, EPI_ISL_429753, EPI_ISL_429755 to 429760, EPI_ISL_429762, EPI_ISL_429764, EPI_ISL_429766 to 429767, EPI_ISL_429769 to 429770, EPI_ISL_429772 to 429782, EPI_ISL_429784, EPI_ISL_429786 to 429787, EPI_ISL_429791, EPI_ISL_429793 to 429794, EPI_ISL_434487 to 434489, EPI_ISL_434491 to 434493, EPI_ISL_434495 to 434498, EPI_ISL_434500, EPI_ISL_434502 to 434507, EPI_ISL_434511 to 434512, EPI_ISL_434514 to 434515, EPI_ISL_445054 to 445058, EPI_ISL_445060 to 445061, EPI_ISL_445064 to 445067, EPI_ISL_445069 to 445072, EPI_ISL_445074 to 445076, EPI_ISL_459893 to 459894, EPI_ISL_459896, EPI_ISL_459898 to 459901, EPI_ISL_459903, EPI_ISL_459905 to 459906			
see above	Laboratoire National de Sante, Microbiology, Virology	Laboratoire National de Sante, Microbiology, Epidemiology and Microbial Genomics	Anke Wienecke-Baldacchino; Ardashel Latsuzbaia; Catherine Ragimbeau; Guillaume Fournier; Jessica Tapp; Joel Mossong; Tamir Abdelrahman; Trung Nguyen Nguyen
EPI_ISL_413593	Laboratoire National de Santé	Erasmus Medical Center	Anne van der Linden; Annemiek van der Eijk; Bas Oude Munnink; Claudia Schapendonk; Corine GeurtsvanKessel; David Nieuwenhuijse; G. Fournier; Irina Chestakova; J. Mossong; Jeroen van Kampen; Jolanda Voermans; Marion Koopmans; Mark Pronk; Pascal Lexmond; Reina Sikkema; Richard Molenkamp; T. Abdelrahman; T. Nguyen; on behalf of the Dutch national COVID-19 response team.
EPI_ISL_419562 to 419564, EPI_ISL_419566, EPI_ISL_419568 to 419570, EPI_ISL_419573, EPI_ISL_419578 to 419580, EPI_ISL_419582 to 419604, EPI_ISL_419606 to 419607			
see above	Laboratoire National de Santé, Microbiology, Virology	Laboratoire National de Santé, Microbiology, Epidemiology and Microbial Genomics	Anke Wienecke-Baldacchino; Ardashel Latsuzbaia; Catherine Ragimbeau; Guillaume Fournier; Jessica Tapp; Joel Mossong; Tamir Abdelrahman; Trung Nguyen Nguyen
EPI_ISL_417526 to 417534	Laboratoire Nationale de Santé, Microbiology, Virology	Laboratoire Nationale de Santé, Microbiology, Epidemiology and Microbial Genomics	Anke Wienecke-Baldacchino; Ardashel Latsuzbaia; Catherine Ragimbeau; Guillaume Fournier; Jessica Tapp; Joel Mossong; Tamir Abdelrahman; Trung Nguyen Nguyen
EPI_ISL_768821	Laboratoire Novabio	CNR Virus des Infections Respiratoires - France SUD	Antonin Bal; Bruno Lina; Gregory Destras; Gwendolyne Burfin; Hadrien Règue; Laurence Josset; Martine Valette; Quentin Semanas
EPI_ISL_754141 to 754145, EPI_ISL_754147 to 754172, EPI_ISL_1013217 to 1013221	Laboratoire de Microbiologie	National Reference Center for Viruses of Respiratory Infections, Institut Pasteur, Paris	Angela Brisebarre; Camille Capel; Etienne Simon-Lorière; Marie-Sarah Fangous; Marion Barbet; Martres Pascale; Maud Vanpeene; Méline Bizard; Sylvie Behillil; Sylvie van der Werf; Vincent Enouf
EPI_ISL_443284 to 443288	Laboratoire de Microbiologie - Bât A - CH René Dubois	National Reference Center for Viruses of Respiratory Infections, Institut Pasteur, Paris	Angela Brisebarre; Etienne Simon-Lorière; Flora Donati; Marion Barbet; Maud Vanpeene; Mélanie Albert; Méline Bizard; Pascale Martres; Sylvie Behillil; Sylvie van der Werf; Vincent Enouf
EPI_ISL_660446 to 660495	Laboratoire de Microbiologie CHU Souro Sanou	Centre Muraz	Abdoul-Salam Ouedraogo; Arsène Zongo; Essia Belarbi; Fabian Leendertz; Grit Schubert; Halidou Tinto; Lassana Sangaré; Soumeya Ouangraoua; Yacouba Sawadogo; Zekiba Tarnagda
EPI_ISL_712062 to 712067, EPI_ISL_712568	Laboratoire de Microbiologie- CHU Habib Bourguiba - Sfax adresse	Laboratoire des Procédés de Criblage Moléculaire et Cellulaire-Centre de Biotechnologie de Sfax	A. and Masmoudi, S.; Abdelmoulah, F.; Abid, N.; Ajili, F.; Aouni, M.; Ben Ayed, I.; Bensaid, M.; Chtourou, A.; Elargoubi, A.; Fki-berrajah, L.; Gaaloul, I.; Gargouri, S.; Hammami, A.; Kamoun, S.; Karray Hakim, H.; Kharat, N.; Mastouri, M.; Mhalla, S.; Nabli, A.; Rebai; Smeti, I.; Souissi, A.; Stambouli, N.; Turki, M.
EPI_ISL_754849 to 754852	Laboratoire de Virologie Hôpital Robert Debré	National Reference Center for Viruses of Respiratory Infections, Institut Pasteur, Paris	Andreoletti Laurent; Angela Brisebarre; Camille Capel; Etienne Simon-Lorière; Marion Barbet; Maud Vanpeene; Méline Bizard; Sylvie Behillil; Sylvie van der Werf; Vincent Enouf
EPI_ISL_414600, EPI_ISL_414623	Laboratoire de Virologie Institut de Virologie - INSERM U 1109 Hôpitaux Universitaires de Strasbourg	National Reference Center for Viruses of Respiratory Infections, Institut Pasteur, Paris	Angela Brisebarre; Flora Donati Vincent Enouf; Marion Barbet; Maud Vanpeene; Méline Bizard; Mélnie Albert; Samira Fafi-Kremer; Sylvie Behillil; Sylvie van der Werf
EPI_ISL_912565, EPI_ISL_912568, EPI_ISL_912575 to 912578, EPI_ISL_912602, EPI_ISL_913608 to 913610	Laboratoire de Virologie du CHU de Lille Bâtiment Paul Boulanger	National Reference Center for Viruses of Respiratory Infections, Institut Pasteur, Paris	Angela Brisebarre; Camille Capel; Etienne Simon-Lorière; Guigon Aurélie; Marion Barbet; Maud Vanpeene; Méline Bizard; Sylvie Behillil; Sylvie van der Werf; Vincent Enouf
EPI_ISL_666870	Laboratoire de Virologie, CHU de Caen, Normandie, France.	GRAM2.0, Université de Caen Normandie	Astrid VABRET; Estelle LEPERCHOIS; Januel VOUTEAU; Juliette ECHE; Meriadeg LE GOUIL
EPI_ISL_413996 to 413997, EPI_ISL_413999, EPI_ISL_414019 to 414023, EPI_ISL_548147 to 548157, EPI_ISL_548159 to 548162, EPI_ISL_548164 to 548168, EPI_ISL_548170, EPI_ISL_548172 to 548199, EPI_ISL_548201 to 548202, EPI_ISL_548204 to 548218, EPI_ISL_548220 to 548235, EPI_ISL_548237 to 548238, EPI_ISL_548240 to 548242			
see above	Laboratoire de Virologie, HUG	Swiss National Reference Centre for Influenza	LAUBSCHER F.; LAUBSCHER Florian et al.
EPI_ISL_765479	Laboratoire de Virologie, Hôpital Robert Debré	National Reference Center for Viruses of Respiratory Infections, Institut Pasteur, Paris	Andreoletti Laurent; Angela Brisebarre; Camille Capel; Etienne Simon-Lorière; Marion Barbet; Maud Vanpeene; Méline Bizard; Sylvie Behillil; Sylvie van der Werf; Vincent Enouf
EPI_ISL_444495, EPI_ISL_444500, EPI_ISL_450639, EPI_ISL_463904 to 463906, EPI_ISL_463908 to 463910, EPI_ISL_463912 to 463914, EPI_ISL_463917 to 463920, EPI_ISL_463922 to 463923, EPI_ISL_463927 to 463931, EPI_ISL_463933 to 463934, EPI_ISL_463936, EPI_ISL_463940 to 463943, EPI_ISL_463946 to 463948, EPI_ISL_463953 to 463954, EPI_ISL_463956 to 463957, EPI_ISL_463961 to 463963, EPI_ISL_463965, EPI_ISL_463968, EPI_ISL_476498 to 476503, EPI_ISL_476505 to 476513, EPI_ISL_515197, EPI_ISL_515202 to 515203, EPI_ISL_515209 to 515212, EPI_ISL_515214, EPI_ISL_515216, EPI_ISL_515220, EPI_ISL_515222, EPI_ISL_515224 to 515231, EPI_ISL_515233 to 515235, EPI_ISL_515237 to 515238, EPI_ISL_515241, EPI_ISL_515243 to 515245			
see above	Laboratoire de microbiologie, Hopital de Verdun	Smith Laboratory, Centre de Recherche CHU Sainte-Justine	Ivan Pavlov; Marieke Rozendaal; Martin Smith
EPI_ISL_889811	Laboratoire de santé publique du Québec	Laboratoire de santé publique du Québec	Guillaume Bourque; Ioannis Ragoussis; Jesse Shapiro; Mark Lathrop and Michel Roger on behalf of the CoVSeQ research group; Sandrine Moreira
EPI_ISL_634818, EPI_ISL_700349	Laboratoire de virologie, CHU de Grenoble - CS 10217 - 38043 Grenoble cedex 10	CNR Virus des Infections Respiratoires - France SUD	Antonin Bal; Bruno Lina; Gregory Destras; Gwendolyne Burfin; Hadrien Règue; Laurence Josset; Martine Valette; Quentin Semanas; Sylvie Larrat
EPI_ISL_634819, EPI_ISL_700350	Laboratoire de virologie, CHU de Grenoble - CS 10217 - 38043 Grenoble cedex 11	CNR Virus des Infections Respiratoires - France SUD	Antonin Bal; Bruno Lina; Gregory Destras; Gwendolyne Burfin; Hadrien Règue; Laurence Josset; Martine Valette; Quentin Semanas; Sylvie Larrat
EPI_ISL_634820, EPI_ISL_700351	Laboratoire de virologie, CHU de Grenoble - CS 10217 - 38043 Grenoble cedex 12	CNR Virus des Infections Respiratoires - France SUD	Antonin Bal; Bruno Lina; Gregory Destras; Gwendolyne Burfin; Hadrien Règue; Laurence Josset; Martine Valette; Quentin Semanas; Sylvie Larrat
EPI_ISL_634821, EPI_ISL_700352	Laboratoire de virologie, CHU de Grenoble - CS 10217 - 38043 Grenoble cedex 13	CNR Virus des Infections Respiratoires - France SUD	Antonin Bal; Bruno Lina; Gregory Destras; Gwendolyne Burfin; Hadrien Règue; Laurence Josset; Martine Valette; Quentin Semanas; Sylvie Larrat
EPI_ISL_634822, EPI_ISL_700353	Laboratoire de virologie, CHU de Grenoble - CS 10217 - 38043 Grenoble cedex 14	CNR Virus des Infections Respiratoires - France SUD	Antonin Bal; Bruno Lina; Gregory Destras; Gwendolyne Burfin; Hadrien Règue; Laurence Josset; Martine Valette; Quentin Semanas; Sylvie Larrat
EPI_ISL_634823, EPI_ISL_700354	Laboratoire de virologie, CHU de Grenoble - CS 10217 - 38043 Grenoble cedex 15	CNR Virus des Infections Respiratoires - France SUD	Antonin Bal; Bruno Lina; Gregory Destras; Gwendolyne Burfin; Hadrien Règue; Laurence Josset; Martine Valette; Quentin Semanas; Sylvie Larrat
EPI_ISL_634824, EPI_ISL_700355	Laboratoire de virologie, CHU de Grenoble - CS 10217 - 38043 Grenoble cedex 16	CNR Virus des Infections Respiratoires - France SUD	Antonin Bal; Bruno Lina; Gregory Destras; Gwendolyne Burfin; Hadrien Règue; Laurence Josset; Martine Valette; Quentin Semanas; Sylvie Larrat
EPI_ISL_634825, EPI_ISL_700356	Laboratoire de virologie, CHU de Grenoble - CS 10217 - 38043 Grenoble cedex 17	CNR Virus des Infections Respiratoires - France SUD	Antonin Bal; Bruno Lina; Gregory Destras; Gwendolyne Burfin; Hadrien Règue; Laurence Josset; Martine Valette; Quentin Semanas; Sylvie Larrat
EPI_ISL_634826, EPI_ISL_700357	Laboratoire de virologie, CHU de Grenoble - CS 10217 - 38043 Grenoble cedex 18	CNR Virus des Infections Respiratoires - France SUD	Antonin Bal; Bruno Lina; Gregory Destras; Gwendolyne Burfin; Hadrien Règue; Laurence Josset; Martine Valette; Quentin Semanas; Sylvie Larrat
EPI_ISL_634827, EPI_ISL_700358	Laboratoire de virologie, CHU de Grenoble - CS 10217 - 38043 Grenoble cedex 19	CNR Virus des Infections Respiratoires - France SUD	Antonin Bal; Bruno Lina; Gregory Destras; Gwendolyne Burfin; Hadrien Règue; Laurence Josset; Martine Valette; Quentin Semanas; Sylvie Larrat
EPI_ISL_634828, EPI_ISL_700359	Laboratoire de virologie, CHU de Grenoble - CS 10217 - 38043 Grenoble cedex 20	CNR Virus des Infections Respiratoires - France SUD	Antonin Bal; Bruno Lina; Gregory Destras; Gwendolyne Burfin; Hadrien Règue; Laurence Josset; Martine Valette; Quentin Semanas; Sylvie Larrat
EPI_ISL_634829, EPI_ISL_700360	Laboratoire de virologie, CHU de Grenoble - CS 10217 - 38043 Grenoble cedex 21	CNR Virus des Infections Respiratoires - France SUD	Antonin Bal; Bruno Lina; Gregory Destras; Gwendolyne Burfin; Hadrien Règue; Laurence Josset; Martine Valette; Quentin Semanas; Sylvie Larrat
EPI_ISL_634830, EPI_ISL_700361	Laboratoire de virologie, CHU de Grenoble - CS 10217 - 38043 Grenoble cedex 22	CNR Virus des Infections Respiratoires - France SUD	Antonin Bal; Bruno Lina; Gregory Destras; Gwendolyne Burfin; Hadrien Règue; Laurence Josset; Martine Valette; Quentin Semanas; Sylvie Larrat
EPI_ISL_634831, EPI_ISL_700362	Laboratoire de virologie, CHU de Grenoble - CS 10217 - 38043 Grenoble cedex 23	CNR Virus des Infections Respiratoires - France SUD	Antonin Bal; Bruno Lina; Gregory Destras; Gwendolyne Burfin; Hadrien Règue; Laurence Josset; Martine Valette; Quentin Semanas; Sylvie Larrat

EPI_ISL_634832, EPI_ISL_700363	Laboratoire de virologie, CHU de Grenoble - CS 10217 - 38043 Grenoble cedex 24	CNR Virus des Infections Respiratoires - France SUD	Antonin Bal; Bruno Lina; Gregory Destras; Gwendolyne Burfin; Hadrien Règue; Laurence Josset; Martine Valette; Quentin Semanas; Sylvie Larrat
EPI_ISL_634833, EPI_ISL_700364	Laboratoire de virologie, CHU de Grenoble - CS 10217 - 38043 Grenoble cedex 25	CNR Virus des Infections Respiratoires - France SUD	Antonin Bal; Bruno Lina; Gregory Destras; Gwendolyne Burfin; Hadrien Règue; Laurence Josset; Martine Valette; Quentin Semanas; Sylvie Larrat
EPI_ISL_634834, EPI_ISL_700365	Laboratoire de virologie, CHU de Grenoble - CS 10217 - 38043 Grenoble cedex 26	CNR Virus des Infections Respiratoires - France SUD	Antonin Bal; Bruno Lina; Gregory Destras; Gwendolyne Burfin; Hadrien Règue; Laurence Josset; Martine Valette; Quentin Semanas; Sylvie Larrat
EPI_ISL_634835, EPI_ISL_700366	Laboratoire de virologie, CHU de Grenoble - CS 10217 - 38043 Grenoble cedex 27	CNR Virus des Infections Respiratoires - France SUD	Antonin Bal; Bruno Lina; Gregory Destras; Gwendolyne Burfin; Hadrien Règue; Laurence Josset; Martine Valette; Quentin Semanas; Sylvie Larrat
EPI_ISL_634836, EPI_ISL_700367	Laboratoire de virologie, CHU de Grenoble - CS 10217 - 38043 Grenoble cedex 28	CNR Virus des Infections Respiratoires - France SUD	Antonin Bal; Bruno Lina; Gregory Destras; Gwendolyne Burfin; Hadrien Règue; Laurence Josset; Martine Valette; Quentin Semanas; Sylvie Larrat
EPI_ISL_634837, EPI_ISL_700368	Laboratoire de virologie, CHU de Grenoble - CS 10217 - 38043 Grenoble cedex 29	CNR Virus des Infections Respiratoires - France SUD	Antonin Bal; Bruno Lina; Gregory Destras; Gwendolyne Burfin; Hadrien Règue; Laurence Josset; Martine Valette; Quentin Semanas; Sylvie Larrat
EPI_ISL_700369	Laboratoire de virologie, CHU de Grenoble - CS 10217 - 38043 Grenoble cedex 30	CNR Virus des Infections Respiratoires - France SUD	Antonin Bal; Bruno Lina; Gregory Destras; Gwendolyne Burfin; Hadrien Règue; Laurence Josset; Martine Valette; Quentin Semanas; Sylvie Larrat
EPI_ISL_700370	Laboratoire de virologie, CHU de Grenoble - CS 10217 - 38043 Grenoble cedex 31	CNR Virus des Infections Respiratoires - France SUD	Antonin Bal; Bruno Lina; Gregory Destras; Gwendolyne Burfin; Hadrien Règue; Laurence Josset; Martine Valette; Quentin Semanas; Sylvie Larrat
EPI_ISL_634817, EPI_ISL_700348, EPI_ISL_900510	Laboratoire de virologie, CHU de Grenoble - CS 10217 - 38043 Grenoble cedex 9	CNR Virus des Infections Respiratoires - France SUD	Antonin Bal; Bruno Lina; Gregory Destras; Gwendolyne Burfin; Hadrien Règue; Laurence Josset; Martine Valette; Quentin Semanas; Sylvie Larrat
EPI_ISL_476822 to 476831, EPI_ISL_476833 to 476834	Laboratoire des Fièvres Hémorragiques Virales du Benin	Charité-Universitätsmedizin Berlin	Anges; Drexler; Jan Felix; Moreira-Soto Andres; Sander Anna-Lena; Yadouleton
EPI_ISL_629081 to 629086, EPI_ISL_629088 to 629099, EPI_ISL_629101 to 629108, EPI_ISL_666654, EPI_ISL_666656 to 666657, EPI_ISL_666659 to 666662, EPI_ISL_666664 to 666665, EPI_ISL_666668 to 666674, EPI_ISL_666676 to 666681, EPI_ISL_683331 to 683332			
see above	Laboratoire du Centre Hospitalier Annecy Genevois	CNR Virus des Infections Respiratoires - France SUD	Antonin Bal; Bruno Chanzy; Bruno Lina; Gregory Destras; Gwendolyne Burfin; Hadrien Règue; Hélène Petitprez; Laurence Josset; Martine Valette; Quentin Semanas
EPI_ISL_910565, EPI_ISL_910631, EPI_ISL_910895, EPI_ISL_910903, EPI_ISL_910910, EPI_ISL_911104, EPI_ISL_911106, EPI_ISL_911113, EPI_ISL_911123, EPI_ISL_911136, EPI_ISL_911138, EPI_ISL_911147, EPI_ISL_911150, EPI_ISL_911156 to 911157, EPI_ISL_911179, EPI_ISL_911185, EPI_ISL_911188, EPI_ISL_911193 to 911194, EPI_ISL_911199, EPI_ISL_911210, EPI_ISL_911216, EPI_ISL_911219 to 911221, EPI_ISL_911233 to 911234			
see above	Laboratoire national de sante, Microbiology, Virology	Laboratoire national de sante, Microbiology, Microbial Genomics Platform	Anke Wienecke-Baldacchino; Catherine Ragimbeau; Fatu Djabi; Jessica Tapp; Lise Pignon; Raoul Salmon; Tamir Abdelrahman
EPI_ISL_739700, EPI_ISL_744295, EPI_ISL_744819	Laboratoire national de santé, Microbiology, Virology	Laboratoire national de santé, Microbiology, Epidemiology and Microbial Genomics	Anke Wienecke-Baldacchino; Catherine Ragimbeau; Fatu Djabi; Jessica Tapp; Tamir Abdelrahman; Trung Nguyen Nguyen
EPI_ISL_739693 to 739699, EPI_ISL_739701 to 739726, EPI_ISL_739728 to 739745, EPI_ISL_739748 to 739757, EPI_ISL_739759 to 739784, EPI_ISL_739786 to 739788, EPI_ISL_739790 to 739795, EPI_ISL_739797 to 739807, EPI_ISL_739809 to 739812, EPI_ISL_739814 to 739837, EPI_ISL_739839 to 739842, EPI_ISL_739844 to 739865, EPI_ISL_739867 to 739876, EPI_ISL_739878 to 739879, EPI_ISL_739881 to 739894, EPI_ISL_739896 to 739899, EPI_ISL_739901 to 739919, EPI_ISL_739921 to 739946, EPI_ISL_739948 to 739950, EPI_ISL_739952 to 739958, EPI_ISL_739960, EPI_ISL_739962 to 739976, EPI_ISL_739978 to 739987, EPI_ISL_739989 to 739991, EPI_ISL_739993, EPI_ISL_739995 to 740015, EPI_ISL_740017 to 740022, EPI_ISL_740024 to 740032, EPI_ISL_740034 to 740037, EPI_ISL_740039, EPI_ISL_740041 to 740048, EPI_ISL_740051 to 740069, EPI_ISL_740071 to 740091, EPI_ISL_740093 to 740097, EPI_ISL_740099 to 740104, EPI_ISL_740106 to 740124, EPI_ISL_740126, EPI_ISL_740128, EPI_ISL_740130 to 740137, EPI_ISL_740139 to 740149, EPI_ISL_740151 to 740156, EPI_ISL_740158 to 740175, EPI_ISL_740177 to 740181, EPI_ISL_740183 to 740201, EPI_ISL_740203, EPI_ISL_740205 to 740218, EPI_ISL_740220 to 740245, EPI_ISL_740247 to 740249, EPI_ISL_740251, EPI_ISL_740253 to 740257, EPI_ISL_740260 to 740277, EPI_ISL_740279 to 740286, EPI_ISL_740288 to 740301, EPI_ISL_740303 to 740305, EPI_ISL_740307, EPI_ISL_740309 to 740312, EPI_ISL_740314 to 740320, EPI_ISL_740322, EPI_ISL_740324 to 740352, EPI_ISL_740354 to 740364, EPI_ISL_740366 to 740367, EPI_ISL_740369 to 740376, EPI_ISL_740378 to 740407, EPI_ISL_740410 to 740420, EPI_ISL_740422 to 740428, EPI_ISL_740430 to 740434, EPI_ISL_740436 to 740438, EPI_ISL_740441 to 740464, EPI_ISL_740467 to 740472, EPI_ISL_740474 to 740486, EPI_ISL_740488 to 740492, EPI_ISL_740494 to 740502, EPI_ISL_740504 to 740546, EPI_ISL_744135, EPI_ISL_744137, EPI_ISL_744140 to 744150, EPI_ISL_744152 to 744172, EPI_ISL_744174 to 744188, EPI_ISL_744190 to 744199, EPI_ISL_744201 to 744210, EPI_ISL_744212 to 744248, EPI_ISL_744250 to 744258, EPI_ISL_744260 to 744268, EPI_ISL_744270 to 744273, EPI_ISL_744275 to 744278, EPI_ISL_744280 to 744290, EPI_ISL_744292 to 744294, EPI_ISL_744296 to 744302, EPI_ISL_744304 to 744306, EPI_ISL_744308 to 744314, EPI_ISL_744316 to 744328, EPI_ISL_744331 to 744335, EPI_ISL_744337 to 744347, EPI_ISL_744349 to 744367, EPI_ISL_744369 to 744370, EPI_ISL_744372 to 744376, EPI_ISL_744378 to 744381, EPI_ISL_744383, EPI_ISL_744385 to 744402, EPI_ISL_744404 to 744418, EPI_ISL_744420, EPI_ISL_744423 to 744463, EPI_ISL_744465 to 744476, EPI_ISL_744478 to 744483, EPI_ISL_744486 to 744487, EPI_ISL_744489 to 744519, EPI_ISL_744521 to 744522, EPI_ISL_744524 to 744530, EPI_ISL_744533 to 744550, EPI_ISL_744552 to 744559, EPI_ISL_744563 to 744584, EPI_ISL_744586 to 744587, EPI_ISL_744589 to 744594, EPI_ISL_744596, EPI_ISL_744599 to 744606, EPI_ISL_744608, EPI_ISL_744611 to 744617, EPI_ISL_744619 to 744637, EPI_ISL_744639 to 744643, EPI_ISL_744645 to 744646, EPI_ISL_744648 to 744650, EPI_ISL_744653 to 744676, EPI_ISL_744678 to 744690, EPI_ISL_744692 to 744708, EPI_ISL_744711 to 744724, EPI_ISL_744726 to 744730, EPI_ISL_744732 to 744735, EPI_ISL_744737 to 744768, EPI_ISL_744770 to 744780, EPI_ISL_744782 to 744785, EPI_ISL_744787 to 744790, EPI_ISL_744792 to 744799, EPI_ISL_744802 to 744818, EPI_ISL_744820 to 744828, EPI_ISL_744831 to 744845, EPI_ISL_744847 to 744862, EPI_ISL_744864 to 744866, EPI_ISL_744868 to 744869, EPI_ISL_744871 to 744893, EPI_ISL_744895 to 744902, EPI_ISL_744904 to 744907, EPI_ISL_744909, EPI_ISL_744911 to 744948, EPI_ISL_744950 to 744957, EPI_ISL_744959 to 744970, EPI_ISL_744972 to 744986, EPI_ISL_744988 to 744992, EPI_ISL_744994 to 744997, EPI_ISL_744999 to 745002, EPI_ISL_745004 to 745014, EPI_ISL_745016 to 745018, EPI_ISL_745020 to 745023, EPI_ISL_745025 to 745027, EPI_ISL_755589, EPI_ISL_770880 to 770881, EPI_ISL_771146			
see above	Laboratoire national de santé, Microbiology, Virology	Laboratoire national de santé, Microbiology, Microbial Genomics Platform	Anke Wienecke-Baldacchino; Catherine Ragimbeau; Fatu Djabi; Jessica Tapp; Lise Pignon; Raoul Salmon; Tamir Abdelrahman
EPI_ISL_429128 to 429135, EPI_ISL_430856 to 430859, EPI_ISL_434641 to 434642, EPI_ISL_450836 to 450837	Laboratoriemedicin	The Public Health Agency of Sweden	Anna Risberg; Anna-Malin Linde; Karin Tegmark-Wisell; Maria Lind Karlberg; Mia Brytting; Olov Svartstrom; Oskar Karlsson Lindsjo; Shaman Muradrasoli; Theresa Enkirch
EPI_ISL_534248, EPI_ISL_534252 to 534255, EPI_ISL_534257 to 534258	Laboratoriemedicin Vasternorrland	The Public Health Agency of Sweden	Anna Risberg; Anna-Malin Linde; Karin Tegmark-Wisell; Maria Lind Karlberg; Mattias Haukland; Mia Brytting; Olov Svartstrom; Oskar Karlsson Lindsjo; Petra Edquist; Reza Advani; Sandra Broddesson
EPI_ISL_661299 to 661300, EPI_ISL_676535 to 676540, EPI_ISL_766640 to 766641	Laboratoriemedicin, Klinisk mikrobiologi	The Public Health Agency of Sweden	Department of Microbiology; The Public Health Agency of Sweden
EPI_ISL_458085	Laboratorio Biologia Molecolare Sars Cov2 - UOC Laboratorio Analisi - Servizio Medicina di Laboratorio , Ospedale "San Francesco" - ATS- ASSL Nuoro	Laboratorio specialistico UOC Ematologia - Ospedale "San Francesco" - ATS-ASSL Nuoro	Asproni Rosanna; Casu Gavino; Fancello Tatiana; Fiamma Maura; Floris Anna Rita; Lo Maglio Iana; Mameli Giuseppe.; Monne Maria Itria; Palmas Angelo Domenico; Piras Giovanna; Sanna Filomena; Sulis Vincenzo; Toja Alessandro
EPI_ISL_458084	Laboratorio Biologia Molecolare Sars Cov2 - UOC Laboratorio Analisi - Servizio Medicina di Laboratorio, Ospedale "San Francesco" - ATS-ASSL Nuoro	Laboratorio specialistico UOC Ematologia - Ospedale "San Francesco" - ATS-ASSL Nuoro	Asproni Rosanna; Casu Gavino; Fancello Tatiana; Fiamma Maura; Floris Anna Rita; Lo Maglio Iana; Mameli Giuseppe.; Monne Maria Itria; Palmas Angelo Domenico; Piras Giovanna; Sanna Filomena; Sulis Vincenzo; Toja Alessandro
EPI_ISL_613706, EPI_ISL_613953, EPI_ISL_613955, EPI_ISL_614396 to 614398, EPI_ISL_614889, EPI_ISL_637108 to 637109, EPI_ISL_1034916			
see above	Laboratorio Biologia Molecolare Sars Cov2 - UOC Laboratorio Analisi - Servizio Medicina di Laboratorio, Ospedale "San Francesco" - ATS-ASSL Nuoro	Laboratorio specialistico UOC Ematologia - Ospedale "San Francesco" - ATS-ASSL Nuoro	Asproni Rosanna; Casu Gavino; Fancello Tatiana; Fiamma Maura; Floris Anna Rita; Lo Maglio Iana; Malune Paolo; Mameli Giuseppe; Monne Maria Itria; Palmas Angelo Domenico; Piras Giovanna; Sanna Filomena; Sulis Vincenzo; Toja Alessandro
EPI_ISL_613560	Laboratorio Biologia Molecolare Sars Cov2 - UOC Laboratorio Analisi - Servizio Medicina di Laboratorio, Ospedale "San Francesco" - ATS-ASSL Nuoro Via Mannironi 1, 08100 Nuoro	Laboratorio specialistico UOC Ematologia - Ospedale "San Francesco" - ATS-ASSL Nuoro Nuoro	Asproni Rosanna; Casu Gavino; Fancello Tatiana; Fiamma Maura; Floris Anna Rita; Lo Maglio Iana; Mameli Giuseppe; Monne Maria Itria; Palmas Angelo Domenico; Piras Giovanna; Sanna Filomena; Sulis Vincenzo; Toja Alessandro
EPI_ISL_770018 to 770019	Laboratorio Cenahce	Inciensa, Instituto Costarricense de Investigación y Enseñanza en Nutrición y Salud	Adriana Godínez; Claudio Soto-Garita; Estela Cordero; Francisco Duarte; Hebleen Porras; Melany Calderón & Andrés Feoli-Grant
EPI_ISL_769987, EPI_ISL_769991	Laboratorio Clinico Labin	Inciensa, Instituto Costarricense de Investigación y Enseñanza en Nutrición y Salud	Adriana Godinez; Adriana Godínez; Claudio Soto-Garita; Estela Cordero; Francisco Duarte; Hebleen Porras; Melany Calderon & Pei Chan Ma; Melany Calderón & Mariel López
EPI_ISL_424667	Laboratorio Estatal de Salud Publica del Estado de México	Instituto de Diagnóstico y Referencia Epidemiológicos	Adnan Araiza Rodríguez; Alejandro Sánchez; Alfredo Ponce de León Garduño; Blanca Taboada; Carlos F. Arias.; Carolina González Torres; Celia Boukadida; Cesar Raúl González Bonilla; Concepción Grajales Muñiz; Edgar Mendieta Condado; Eduardo Becerril Vargas; Fabiola Garcés Ayala; Fernando Ledesma Barrientos; Francisco Javier Gaytán Cervantes; Francisco Pulido; Gisela Barrera Badillo; Gloria Vázquez; Guillermo M. Ruiz-Palacios;

			Irma López Martínez; Joel Armando Vázquez Pérez; José Arturo Martínez Orozco; José Ernesto Ramírez González; José Esteban Muñoz Medina; Lucia Hernández Rivas; Luis Alberto García Andrade; Mario Railway Mújica Sánchez; Pavel Isa; Pilar Ramos Cervantes; Ricardo Grande; Santiago Avila Ríos; Victor Hugo Borja Aburto; Violeta Ibarra Gonzalez
EPI_ISL_424672	Laboratorio Estatal de Salud Publica del Estado de Puebla	Instituto de Diagnostico y Referencia Epidemiologicos	Adnan Araiza Rodríguez; Alejandro Sánchez; Alfredo Ponce de León Garduño; Blanca Taboada; Carlos F. Arias; Carolina González Torres; Celia Boukadida; Cesar Raúl González Bonilla; Concepción Grajales Muñiz; Edgar Mendieta Condado; Eduardo Becerril Vargas; Fabiola Garcés Ayala; Fernando Ledesma Barrientos; Francisco Javier Gaytán Cervantes; Francisco Pulido; Gisela Barrera Badillo; Gloria Vázquez; Guillermo M. Ruiz-Palacios; Irma López Martínez; Joel Armando Vázquez Pérez; José Arturo Martínez Orozco; José Ernesto Ramírez González; José Esteban Muñoz Medina; Lucia Hernández Rivas; Luis Alberto García Andrade; Mario Mújica Sánchez; Pavel Isa; Pilar Ramos Cervantes; Ricardo Grande; Santiago Avila Ríos; Victor Hugo Borja Aburto; Violeta Ibarra Gonzalez
EPI_ISL_424670	Laboratorio Estatal de Salud Publica del Estado de Queretaro	Instituto de Diagnóstico y Referencia Epidemiologicos	Adnan Araiza Rodríguez; Alejandro Sánchez; Alfredo Ponce de León Garduño; Blanca Taboada; Carlos F. Arias; Carolina González Torres; Celia Boukadida; Cesar Raúl González Bonilla; Concepción Grajales Muñiz; Edgar Mendieta Condado; Eduardo Becerril Vargas; Fabiola Garcés Ayala; Fernando Ledesma Barrientos; Francisco Javier Gaytán Cervantes; Francisco Pulido; Gisela Barrera Badillo; Gloria Vázquez; Guillermo M. Ruiz-Palacios; Irma López Martínez; Joel Armando Vázquez Pérez; José Arturo Martínez Orozco; José Ernesto Ramírez González; José Esteban Muñoz Medina; Lucia Hernández Rivas; Luis Alberto García Andrade; Mario Mújica Sánchez; Pavel Isa; Pilar Ramos Cervantes; Ricardo Grande; Santiago Avila Ríos; Victor Hugo Borja Aburto; Violeta Ibarra Gonzalez
EPI_ISL_794592	Laboratorio Estatal de Salud Pública de Tamaulipas	Instituto de diagnóstico y Referencia Epidemiologicos (INDRE)	Abril Rodriguez-Maldonado; Ana Maria Cortez-Calderon; Bernardita Reyes-Berrones; Celia Alpuche-Aranda; Claudia Wong-Arambula; Ernesto Ramirez-Gonzalez.; Fabiola Garces-Ayala; Gisela Barrera-Badillo; Gloria Molina-Gamboa; Hilda del Carmen-Selvera; Hugo Lopez Gatell-Ramirez; Irma Lopez-Martinez; Jose Luis Alomia-Zegarra; Lucia Hernandez-Rivas
EPI_ISL_735398	Laboratorio Fleury	Instituto Adolfo Lutz, Interdiciplinary Procedures Center, Strategic Laboratory	Claudia Regina Gonçalves; Claudio Tavares Sacchi; Erica Valessa Ramos Gomes; Karoline Rodrigues Campos
EPI_ISL_969131, EPI_ISL_969227, EPI_ISL_969884, EPI_ISL_970647, EPI_ISL_974745, EPI_ISL_977598, EPI_ISL_977601, EPI_ISL_977603, EPI_ISL_977651			
see above	Laboratorio Microbiologia e Virologia P.O. Cotugno A.O. dei Colli	Laboratorio Microbiologia e Virologia P.O. Cotugno A.O. dei Colli	; Anna Perfetti; Claudia Tiberio; Luigi Atripaldi
EPI_ISL_693246	Laboratorio Municipal de Rio Grande da Serra	Instituto Adolfo Lutz, Interdiciplinary Procedures Center, Strategic Laboratory	Claudia Regina Gonçalves; Claudio Tavares Sacchi; Erica Valessa Ramos Gomes; Karoline Rodrigues Campos
EPI_ISL_837585	Laboratorio Nacional de Salud	Laboratory of Respiratory Viruses and Measles, Oswaldo Cruz Institute, FIOCRUZ	Ana Carolina Mendonca; Anna Carolina Paixao; Cesar Roberto Conde Pereira; Claudia Estrada; Fernando Motta; Luciana Appolinario; Marilda Siqueira on behalf of the Fiocruz COVID-19 Genomic Surveillance Network; Paola Resende
EPI_ISL_755302 to 755303	Laboratorio de Bioingeniería, Instituto de Ciencias de la Ingeniería, Universidad de O´Higgins	Center for Mathematical Modeling and Center for Genome Regulation. Santiago, Chile	Allende ML; Arriagada G; Bastias M; Bustos F; Castro E; G. Galvez; González M; J. Ortega; M; M. Latorre; Maass A; Meneses C.; Montecino; Orellana A; Sanhueza D; Travisany D
EPI_ISL_468757 to 468759	Laboratorio de Biologia Molecular, Facultad de Medicina, Universidad de Atacama	Center for Mathematical Modeling and Center for Genome Regulation. Santiago, Chile	Allende ML; C Echeverria; Gaete A; González M; Maass A; Palma R; Travisany D; Urra C; Varas M
EPI_ISL_457946, EPI_ISL_457948, EPI_ISL_457952 to 457953, EPI_ISL_457956 to 457959, EPI_ISL_457961, EPI_ISL_457963, EPI_ISL_457965, EPI_ISL_457972			
see above	Laboratorio de Biología Molecular Asociación Española Primera en Salud	Departments of Pathology and Medicine, New York University School of Medicine	Adriana Heguy; Christian Marier; Gael Westby; Gonzalo Manrique; Maria Noel Zubillaga; Maria Victoria Elizondo; Matthew T Maurano; Paul Zappile
EPI_ISL_842652	Laboratorio de Biología Molecular Hospital Pedro de Elizalde	Grupo de Genómica y Bioinformática del Instituto de Investigación de la Cadena Láctea CONICET-INTA on behalf of 'Proyecto Argentino Interinstitucional de genomica de SARS-CoV-2' (PAIS Consortium)	A; AF; Alegre; Alexay; Amadio; Aulicino; B; Bressan; C; Chamorro; Claps; D; Diaz; E; Eberhardt; F; FJ; G; Gondolessi; Goya; Gómez; Indart; Irazoqui; J; König; L; Lorenzo; Lusso; M; ME; MF; ML; MS; Marchetti; Martín; Montoto Piazza; Morandi; N; Nabaes Jodar; Natale; Osaba; P; Paez; Rocovich; Rosales; S; Sanchez; Sueiro; Torres; Valinotto; Viegas, M.; Wenk; Zamora
EPI_ISL_626549 to 626565	Laboratorio de Biología Molecular, Facultad de Medicina, Universidad de Atacama, Copiapo, Chile/ FONDAP CRG, Universidad Andrés Bello, Santiago, Chile	Center for Mathematical Modeling and Center for Genome Regulation. Santiago, Chile	Allende ML; Bastias M; Castro E; Echeverría C; González M; M; Maass A; Manríquez R; Meneses C.; Montecino; Orellana A; Sanhueza D; Travisany D
EPI_ISL_417034	Laboratorio de Ecologia de Doencas Transmissiveis na Amazonia, Instituto Leonidas e Maria Deane - Fiocruz Amazonia	Laboratorio de Ecologia de Doencas Transmissiveis na Amazonia, Instituto Leonidas e Maria Deane - Fiocruz Amazonia	André Corado; Debora Duarte; Felipe Naveca on behalf of the Fiocruz COVID-19 Genomic Surveillance Network; Fernanda Nascimento; Luciana Gonçalves; Michele Jesus; Sérgio Luz; Valdinete Nascimento; Ágatha Costa
EPI_ISL_545955	Laboratorio de Infecciones Respiratorias Agudas. Centro Nacional de Salud Publica,Instituto Nacional de Salud	Laboratorio de Infecciones Respiratorias Agudas. Centro Nacional de Salud Publica,Instituto Nacional de Salud	Juscamayta, E.
EPI_ISL_591531 to 591534	Laboratorio de Infectologia y virologia molecular	Center for Mathematical Modeling and Center for Genome Regulation. Santiago, Chile	Allende ML; Ferres M.; Gaete A; González M; Maass A; Palma R; Travisany D; Urra C; Valiente F; Varas M
EPI_ISL_648595, EPI_ISL_648602 to 648603	Laboratorio de Infectología Servicio de Infectología Hospital Universitario Dr. José Eleuterio González - Universidad Autónoma de Nuevo León	Laboratorio de Infectología Molecular Departamento de Bioquímica y Medicina Molecular Facultad de Medicina - Universidad Autónoma de Nuevo León	Adrian Camacho-Ortiz; Ana M. Rivas-Estilla; Consuelo Treviño-Garza; Daniel Arellanos-Soto; Eduardo Perez-Alba; Elvira Garza-González; Kame A. Galán-Huerta; Laura Nuzzolo-Shihadeh; Manuel E. de-la-O-Cavazos; María F. Herrera-Saldivar; Natalia Martínez-Acuña; Paola Bocanegra-Ibarias; Roberto Montes-de-Oca; Samantha M. Flores-Treviño; Sonia A. Lozano-Sepúlveda
EPI_ISL_648607	Laboratorio de Infectología, Servicio de Infectología, Hospital Universitario Dr. José Eleuterio González - Universidad Autónoma de Nuevo León	Laboratorio de Infectología Molecular, Departamento de Bioquímica y Medicina Molecular,Facultad de Medicina - Universidad Autónoma de Nuevo León	Adrian Camacho-Ortiz; Ana M. Rivas-Estilla; Consuelo Treviño-Garza; Daniel Arellanos-Soto; Eduardo Perez-Alba; Elvira Garza-González; Kame A. Galán-Huerta; Laura Nuzzolo-Shihadeh; Manuel E. de-la-O-Cavazos; María F. Herrera-Saldivar; Natalia Martínez-Acuña; Paola Bocanegra-Ibarias; Roberto Montes-de-Oca; Samantha M. Flores-Treviño; Sonia A. Lozano-Sepúlveda
EPI_ISL_648305, EPI_ISL_648307 to 648308, EPI_ISL_648310, EPI_ISL_648312 to 648313, EPI_ISL_648315, EPI_ISL_648319 to 648322, EPI_ISL_648324 to 648330, EPI_ISL_648334, EPI_ISL_648336, EPI_ISL_648338 to 648341, EPI_ISL_648343 to 648345, EPI_ISL_648347 to 648348, EPI_ISL_648350, EPI_ISL_648352 to 648355, EPI_ISL_648367, EPI_ISL_648373 to 648374, EPI_ISL_648379, EPI_ISL_649156, EPI_ISL_649158, EPI_ISL_649164, EPI_ISL_649166, EPI_ISL_649170 to 649172			
see above	Laboratorio de Investigaciones de Baney	University Hospital Basel, Clinical Bacteriology	Adrian Egli; Alfredo Mari; Bonifacio Manguire Nlavo; Carlos Cortes; Claudia Daubenberger; Diosdado Odjama Nseng Ada; Elizabeth Nyakarungu; Guillermo Garcia; Helena Seth-Smith; Madlen Stange; Maximilian Mpina; Mitoha Ondo O Ayekaba; Philip Wonder Phiri; Salome Hosch; Tim Roloff; Tobias Schindler
EPI_ISL_941225	Laboratorio de Microbiología. Hospital General Universitario de Elda, Alicante	SeqCOVID-SPAIN consortium/IBV(CSIC)	Cristina Torregrosa Hetland; Eva Pastor Boix; Mª Isabel Gascón Ros; Paloma Cascales Ramos and SeqCOVID-SPAIN consortium
EPI_ISL_517770, EPI_ISL_517959	Laboratorio de Referencia Nacional de Virus Respiratorio. Centro Nacional de Salud Publica. Instituto Nacional de Salud Peru	Laboratorio de Referencia Nacional de Biotecnología y Biología Molecular. Centro Nacional de Salud Publica. Instituto Nacional de Salud Peru.	Carlos Padilla Rojas; Henri Bailon Calderon; Johanna Balbuena Torres; Karolyn Vega Chozo; Marco Galarza Perez; Maribel Huaringa Nuñez; Nancy Rojas Serrano.; Omar Caceres Rey; Priscila Lope Pari
EPI_ISL_516626, EPI_ISL_516628, EPI_ISL_516646 to 516647, EPI_ISL_516653 to 516654, EPI_ISL_516685 to 516686, EPI_ISL_516721 to 516722, EPI_ISL_516987, EPI_ISL_517531, EPI_ISL_517686 to 517687, EPI_ISL_517713, EPI_ISL_517958, EPI_ISL_523810, EPI_ISL_523954, EPI_ISL_523979, EPI_ISL_523994, EPI_ISL_524471, EPI_ISL_524473, EPI_ISL_525206, EPI_ISL_527787, EPI_ISL_527789			
see above	Laboratorio de Referencia Nacional de Virus Respiratorio. Centro Nacional de Salud Publica. Instituto Nacional de Salud Peru.	Laboratorio de Referencia Nacional de Biotecnología y Biología Molecular. Centro Nacional de Salud Publica. Instituto Nacional de Salud Peru.	Carlos Padilla Rojas; Henri Bailon Calderon; Johanna Balbuena Torres; Karolyn Vega Chozo; Marco Galarza Perez; Maribel Huaringa Nuñez; Nancy Rojas Serrano.; Omar Caceres Rey; Priscila Lope Pari
EPI_ISL_489836 to 489839, EPI_ISL_489987 to 489990, EPI_ISL_490209, EPI_ISL_490315 to 490316, EPI_ISL_490975 to 490976, EPI_ISL_491427 to 491430, EPI_ISL_491433 to 491436, EPI_ISL_491458 to 491464, EPI_ISL_514226, EPI_ISL_514317, EPI_ISL_514338 to 514342, EPI_ISL_833038			
see above	Laboratorio de Referencia Nacional de Virus Respiratorio. Instituto Nacional de Salud Perú	Laboratorio de Referencia Nacional de Biotecnología y Biología Molecular. Instituto Nacional de Salud Perú	Carlos Padilla Rojas; Henri Bailon Calderon; Johanna Balbuena Torres; Johanna Balbuena Torrez; Karolyn Vega Chozo; Karolyn Chozo Vega; Karolyn Vega Chozo; Luis Barcena; Marco Galarza Perez; Maribel Huaringa Nuñez; Nancy Rojas Serrano; Nancy Rojas Serrano.; Omar Caceres Rey; Priscila Lope Pari

EPI_ISL_491172	Laboratorio de Referencia Nacional de Virus Respiratorio. Instituto Nacional de Salud Perú	Laboratorio de Referencia Nacional de Biotecnología y Biología Molecular. Instituto Nacional de Salud Perú.	Carlos Padilla Rojas; Henri Bailon Calderon; Johanna Balbuena Torres; Karolyn Chozo Vega; Marco Galarza Perez; Maribel Huaringa Nuñez; Nancy Rojas Serrano; Omar Caceres Rey; Priscila Lope Pari
EPI_ISL_487269	Laboratorio de Referencia Nacional de Virus Respiratorio. Instituto Nacional de Salud.	Laboratorio de Referencia Nacional de Biotecnología y Biología Molecular. Instituto Nacional de Salud.	Carlos Padilla Rojas; Henri Bailon Calderon; Johanna Balbuena Torrez; Karolyn Chozo Vega; Marco Galarza Perez; Maribel Huaringa Nuñez; Nancy Rojas Serrano; Omar Caceres Rey; Priscila Lope Pari
EPI_ISL_415787, EPI_ISL_514227	Laboratorio de Referencia Nacional de Virus Respiratorio. Instituto Nacional de Salud. Peru	Laboratorio de Referencia Nacional de Biotecnologia y Biologia Molecular.Instituto Nacional de Salud.Peru	Carlos Padilla Rojas; Hemri Bailon Calderon; Henri Bailon Calderon; Johanna Balbuena Torres; Johanna Balbuena Torrez; Karolyn Vega Chozo; Marco Galarza Perez; Maribel Huaringa Nuñez; Nancy Rojas Serrano; Omar Caceres Rey; Priscila Lope Pari
EPI_ISL_514264 to 514265	Laboratorio de Referencia Nacional de Virus Respiratorio. Instituto Nacional de Salud. Perú	Laboratorio de Referencia Nacional de Biotecnología y Biologia Molecular.Instituto Nacional de Salud.Perú	Carlos Padilla Rojas; Henri Bailon Calderon; Johanna Balbuena Torrez; Karolyn Vega Chozo; Marco Galarza Perez; Maribel Huaringa Nuñez; Nancy Rojas Serrano; Omar Caceres Rey; Priscila Lope Pari
EPI_ISL_491431	Laboratorio de Referencia Nacional de Virus Respiratorio. Instituto Nacional de Salud. Perú	Laboratorio de Referencia Nacional de Biotecnología y Biología Molecular. Instituto Nacional de Salud.Perú	Carlos Padilla Rojas; Henri Bailon Calderon; Johanna Balbuena Torres; Karolyn Vega Chozo; Marco Galarza Perez; Maribel Huaringa Nuñez; Nancy Rojas Serrano.; Omar Caceres Rey; Priscila Lope Pari
EPI_ISL_491432	Laboratorio de Referencia Nacional de Virus Respiratorio. Instituto Nacional de Salud.Perú	Laboratorio de Referencia Nacional de Biotecnología y Biología Molecular. Instituto Nacional de Salud.Perú	Carlos Padilla Rojas; Henri Bailon Calderon; Johanna Balbuena Torres; Karolyn Vega Chozo; Marco Galarza Perez; Maribel Huaringa Nuñez; Nancy Rojas Serrano.; Omar Caceres Rey; Priscila Lope Pari
EPI_ISL_529065 to 529079, EPI_ISL_540923 to 540992, EPI_ISL_568510 to 568555, EPI_ISL_729862 to 729868, EPI_ISL_729872 to 729901, EPI_ISL_812492 to 812493, EPI_ISL_812504			
see above	Laboratorio de Referencia Nacional de Virus Respiratorios, Instituto Nacional de Salud Peru	Laboratorio de Genómica Microbiana, Universidad Peruana Cayetano Heredia	Alejandra Dávila-Barclay; Brenda Ayzanoa; Camila Castillo-Vilcahuaman; Camila Castillo-Vilcahuamán; Guillermo Salvatierra; Janet Huancachoque; Luis González; Marco Galarza; Maribel Huaringa; Nancy Rojas; Oscar Escalante; Pablo Tsukayama; Pedro E. Romero; Pool Marcos; Priscila Lope
EPI_ISL_482468	Laboratorio de Referencia Nacional de Virus Respiratorios. Instituto Nacional de Salud Peru	Laboratorio de Referencia Nacional de Biotecnologia y Biologia Molecular. Instituto Nacional de Salud Peru	Carlos Padilla Rojas; Henri Bailon Calderon; Johanna Balbuena Torres; Karolyn Vega Chozo; Maribel Huaringa Nuñez; Nancy Rojas Serrano; Omar Caceres Rey; Priscila Lope Pari
EPI_ISL_965029	Laboratorio de Virologia HUCA	Laboratorio de Virologia HUCA	Abreu F; Alvarez-Arguelles ME; Boga JA; Castelló C; Costales I; Coto E; Gómez de Oña J; Martín-Rodríguez G; Melón S; Perez-Martínez Z; Rojo S; Sandoval M
EPI_ISL_623108, EPI_ISL_623110, EPI_ISL_623112, EPI_ISL_623114, EPI_ISL_623116, EPI_ISL_623118 to 623120, EPI_ISL_623124, EPI_ISL_623126, EPI_ISL_623129 to 623130, EPI_ISL_623132, EPI_ISL_623134, EPI_ISL_623136, EPI_ISL_623138, EPI_ISL_623140, EPI_ISL_623142 to 623149, EPI_ISL_623152, EPI_ISL_623154, EPI_ISL_623157 to 623158, EPI_ISL_623160 to 623162, EPI_ISL_623164 to 623166, EPI_ISL_623168, EPI_ISL_717806 to 717808, EPI_ISL_717810 to 717831, EPI_ISL_717837 to 717840, EPI_ISL_717842 to 717898, EPI_ISL_717920 to 717957, EPI_ISL_717959 to 717961			
see above	Laboratorio de Virologia Molecular / UFRJ	Bioinformatics Laboratory / LNCC	Alexandra L Gerber; Amilcar Tanuri; Ana Paula de C Guimarães; Ana Tereza R de Vasconcelos; Andréa Cony Cavalcanti; Carolina M Voloch; Claudia dos Santos Rodrigues; Covid19-UFRJ Workgroup; Cynthia C Cardoso; Diana Mariani; Luiz G P de Almeida; Luís Cristóvão Pôrto; Orlando C. Ferreira; Otavio Bustrolini; Otavio J. Brustolini; Renato S Aguiar; Ronaldo S Francisco Jr; Ronaldo da Silva F Jr; Terezinha M P P Castiñeira; Terezinha M P P Castiñeiras
EPI_ISL_964949	Laboratorio de Virologia del HUCA	Laboratorio de Virologia del HUCA	Abreu F; Alvarez-Arguelles ME; Boga JA; Castelló C; Costales I; Coto E; Gómez de Oña J; Martín-Rodriguez G; Melon S; Perez-Martinez Z; Rojo S; Sandoval M
EPI_ISL_974853	Laboratorio de Virología / Hospital Universitario Central de Asturias (HUCA)	Laboratorio de Virología / Hospital Universitario Central de Asturias (HUCA)	Abreu F; Alvarez-Arguelles ME; Boga JA; Castelló C; Costales I; Coto E; Gómez de Oña J; Martín-Rodríguez G; Melón S; Perez-Martínez Z; Rojo S; Sandoval M
EPI_ISL_965033, EPI_ISL_977505 to 977506, EPI_ISL_977511 to 977512, EPI_ISL_984678, EPI_ISL_984683, EPI_ISL_1020121 to 1020123, EPI_ISL_1020126, EPI_ISL_1020132, EPI_ISL_1020135, EPI_ISL_1020151, EPI_ISL_1020154, EPI_ISL_1020305, EPI_ISL_1033159, EPI_ISL_1033162, EPI_ISL_1034147 to 1034148, EPI_ISL_1034150, EPI_ISL_1034209 to 1034211, EPI_ISL_1034227 to 1034230, EPI_ISL_1034303			
see above	Laboratorio de Virología HUCA	Laboratorio de Virología HUCA	Abreu F; Alvarez-Arguelles ME; Boga JA; Castelló C; Costales I; Coto E; Gómez de Oña J; Martín-Rodríguez G; Melón S; Perez-Martínez Z; Rojo S; Sandoval M
EPI_ISL_992956	Laboratorio de Virología HUCA	Laboratorio de Virología Hospital Universitario Central de Asturias (HUCA)	Abreu F; Alvarez-Arguelles ME; Boga JA; Castelló C; Costales I; Coto E; Gómez de Oña J; Martín-Rodríguez G; Melón S; Perez-Martínez Z; Rojo S; Sandoval M
EPI_ISL_430799 to 430801, EPI_ISL_430803 to 430804, EPI_ISL_430807, EPI_ISL_430809 to 430818	Laboratorio de Virología del Hospital de Niños Dr. Ricardo Gutierrez	Área de Secuenciación del Laboratorio de Virología del Hospital de Niños Dr. Ricardo Gutierrez on behalf of 'Proyecto Argentino Interinstitucional de genomica de SARS-CoV-2' (PAIS Consortium)	AS; E; Goya; Gravis; LE; Lusso; MI; MS; Mistchenko; Nabaes Jodar; Natale; S; Valinotto; Viegas, M.
EPI_ISL_671978	Laboratorio de Virología y Microbiología Molecular, Depto. de Microbiología, Facultad de Medicina, Universidad de El Salvador/INS-laboratorio de Ref. Ministerio de Salud	Laboratorio de Virología y Microbiología Molecular, Depto. de Microbiología, Facultad de Medicina, Universidad de El Salvador/INS-laboratorio de Ref. Ministerio de Salud	Rivera NR et al
EPI_ISL_672573	Laboratorio de Virología y Microbiología Molecular, Depto. de Microbiología, Facultad de Medicina, Universidad de El Salvador/INS-laboratorio de Ref. Ministerio de Salud. 1*: Dr. Noé Rigoberto Rivera, profesor del Departamento de Bioquímica e Investigador adjunto de la Sección de Virología y Microbiología Molecular;director de la Unidad de Investigaciones Científicas (UNICA), Facultad de Medicina Universidad de El Salvador,El Salvador C.A 1**: Dr. Carlos Alexander Ortega Pérez, Profesor del Departamento de Microbiología;investigador y Jefe de la Sección de Virología, Director Metodológico de la Unidad de Investigaciones Científicas(UNICA), Facultad de Medicina, Universidad de El Salvador, El Salvador C.A 2*: Dra. Xochitl Sandoval López:Directora e investigadora del Instituto Nacional de Salud (INS) El Salvador C.A 2** Dr. Hernandez Avila CarlosE Director de Gobernanza e investigador del Instituto Nacional de Salud (INS) El Salvador C.A	Laboratorio de Virología y Microbiología Molecular, Depto. de Microbiología, Facultad de Medicina, Universidad de El Salvador/INS-laboratorio de Ref. Ministerio de Salud	Rivera NR et al
EPI_ISL_672572	Laboratorio de Virología y Microbiología Molecular, Depto. de Microbiología, Facultad de Medicina, Universidad de El Salvador/INS-laboratorio de Ref. Ministerio de Salud. 1*: Dr. Noé Rigoberto Rivera, profesor del Departamento de Bioquímica e Investigador adjunto de la Sección de Virología y Microbiología Molecular; director de la Unidad de Investigaciones Científicas (UNICA), Facultad de Medicina Universidad de El Salvador, El Salvador C.A 1**: Dr. Carlos Alexander Ortega Pérez, Profesor del Departamento de Microbiología; investigador y Jefe de la Sección de Virología, Director Metodológico de la Unidad de Investigaciones Científicas (UNICA), Facultad de Medicina, Universidad de El Salvador, El Salvador C.A 2*: Dra. Xochitl Sandoval López: Directora e investigadora del Instituto Nacional de Salud (INS) El Salvador C.A 2** Dr. Hernandez Avila Carlos E Director de Gobernanza e investigador del Instituto Nacional de Salud (INS) El Salvador C.A	Laboratorio de Virología y Microbiología Molecular, Depto. de Microbiología, Facultad de Medicina, Universidad de El Salvador/INS-laboratorio de Ref. Ministerio de Salud.	Rivera NR et al
EPI_ISL_671974, EPI_ISL_672570	Laboratorio de Virología y Microbiología Molecular, Depto. de Microbiología, Facultad de Medicina, Universidad de El	Laboratorio de Virología y Microbiología Molecular, Depto. de Microbiología, Facultad de Medicina, Universidad de El	Carlos Hernández Ávila; Ortega Pérez CA et al; Ortega-Pérez C A; Rivera NR; Xochitl Sandoval López

	Salvador/INS-laboratorio de Ref. Ministerio de Salud	Salvador/INS-laboratorio de Ref. Ministerio de Salud	
EPI_ISL_672012	Laboratorio de Virología y Microbiología Molecular, Depto. de Microbiología, Facultad de Medicina, Universidad de El Salvador/INS-laboratorio de Ref. Ministerio de Salud. 1*: Dr. Noé Rigoberto Rivera, profesor del Departamento de Bioquímica e Investigador adjunto de la Sección de Virología y Microbiología Molecular; director de la Unidad de Investigaciones Científicas (UNICA), Facultad de Medicina Universidad de El Salvador, El Salvador C.A 1**: Dr. Carlos Alexander Ortega Pérez, Profesor del Departamento de Microbiología; investigador y Jefe de la Sección de Virología, Director Metodológico de la Unidad de Investigaciones Científicas (UNICA), Facultad de Medicina, Universidad de El Salvador, El Salvador C.A 2*: Dra. Xochitl Sandoval López: Directora e investigadora del Instituto Nacional de Salud (INS) El Salvador C.A 2** Dr. Hernandez Avila Carlos E Director de Gobernanza e investigador del Instituto Nacional de Salud (INS) El Salvador C.A	Laboratorio de Virología y Microbiología Molecular, Depto. de Microbiología, Facultad de Medicina, Universidad de El Salvador/INS-laboratorio de Ref. Ministerio de Salud.	Ortega Pérez CA et al
EPI_ISL_755304	Laboratorio de Virología, Centro de Biotecnología Acuícola, Universidad de Santiato de Chile	Center for Mathematical Modeling and Center for Genome Regulation. Santiago, Chile	A. Sandino; Allende ML; Arriagada G; Bastias M; Bustos F; Castro E; González M; M; Maass A; Meneses C.; Montecino; Orellana A; Reyes-López F.; Sanhueza D; Travisany D
EPI_ISL_413489	Laboratorio di Microbiologia e Virologia, Università Vita-Salute San Raffaele, Milano	Laboratorio di Microbiologia e Virologia, Università Vita-Salute San Raffaele, Milano	C. Di Resta; E. Boeri; E. Criscuolo; G. Lo Raso; I. Negri; M. Castelli; M. Clementi; M. Sampaolo; N. Mancini & N. Clementi; R. Burioni; R. Ferrarese; R.A Diotti; V. Amato; V. Caputo
EPI_ISL_985027 to 985031	Laboratorio di Patologia Clinica, Ospedale San Paolo in Valloria, ASL 2 Liguria	U.O. Igiene, Ospedale Policlinico San Martino	Bruzzone Bianca; Caligiuri Patrizia; De Pace Vanessa; Domnich Alexander; Icardi Giancarlo; Lillo Flavia; Orsi Andrea; Ricucci Valentina
EPI_ISL_582692 to 582694, EPI_ISL_582767, EPI_ISL_582769, EPI_ISL_582810 to 582811, EPI_ISL_582833, EPI_ISL_582842, EPI_ISL_582845 to 582850			
see above	Laboratorio di Riferimento Regionale della Sicilia Occidentale per l'Emergenza COVID-19	Istituto Zooprofilattico Sperimentale della Sicilia	Lo Presti Alessandra; Maida Carmelo Massimo; Mazzucco Walter; Pulvirenti Claudio; Purpari Giuseppa; Reale Stefano; Rezza Giovanni; Scibetta Silvia; Stefanelli Paola; Tramuto Fabio; Vitale Fabrizio; Vitale Francesco; Zichichi Salvatore
EPI_ISL_1020314	Laboratorio di Riferimento Regionale della Sicilia Occidentale per l'Emergenza COVID-19	Laboratorio di Riferimento Regionale della Sicilia Occidentale per l'Emergenza COVID-19	Carmelo Massimo Maida; Claudio Costantino; Daniela Di Naro; Fabio Tramuto; Francesco Vitale; Giorgio Graziano; Giulia Randazzo; Vincenzo Restivo; Walter Mazzucco
EPI_ISL_710503	Laboratorio specialistico UOC Ematologia - Ospedale "San Francesco" - ATS-ASSL Nuoro	Laboratorio specialistico UOC Ematologia - Ospedale "San Francesco" - ATS-ASSL Nuoro	Giovanna Piras
EPI_ISL_961466	Laboratorios Lister	Instituto de Diagnostico y Referencia Epidemiologicos (INDRE)	Abril Rodriguez-Maldonado; Claudia Wong-Arambula; Ernesto Ramirez-Gonzalez.; Fabiola Garces-Ayala; Gisela Barrera-Badillo; Irma Lopez-Martinez; Lucia Hernandez-Rivas; Natividad Cruz-Ortiz; Tatiana Nunez-Garcia
EPI_ISL_511879	Laboratorium Kesehatan Provinsi Jawa Barat	Molecular Genetics Laboratory-Faculty of Medicine-Universitas Padjadjaran; School of Life Sciences and Technology & School of Pharmacy-Institut Teknologi Bandung; Laboratorium Kesehatan Provinsi Jawa Barat	Agung Eru Wibowo; Azzania Fibriani; Catur Riani; Cut Nur Cinthia Alamanda; Ema Rahmawati; Hammam Riza; Hesti Lina Wiraswati; Husna Nugrahapraja; Lia Faridah; Marselina Irasonia Tan; Rifky Waluyajati Rachman; Ryan Bayusantika Ristandi; Savira Ekawardhani; Soni Solistia Wirawan; Tarwadi; Yunia Sribudiani
EPI_ISL_528745	Laboratorium Kesehatan Provinsi Jawa Barat	School of Life Sciences and Technology & School of Pharmacy-Institut Teknologi Bandung; Molecular Genetics Laboratory-Faculty of Medicine-Universitas Padjadjaran; Laboratorium Kesehatan Provinsi Jawa Barat	Agung Eru Wibowo; Azzania Fibriani; Catur Riani; Cut Nur Cinthia Alamanda; Ema Rahmawati; Fensi Amalina; Hammam Riza; Hesti Lina Wiraswati; Husna Nugrahapraja; Irvan Faizal; Lia Faridah; Marselina Irasonia Tan; Mas Rizky A.A. Syamsunarno; Rifky Waluyajati Rachman; Ryan Bayusantika Ristandi; Savira Ekawardhani; Sony Solistia Wirawan; Tarwadi; Tri Hanggono Achmad; Yunia Sribudiani
EPI_ISL_832194 to 832195, EPI_ISL_832198	Laboratory Analyses Med	National Reference Center for Viruses of Respiratory Infections, Institut Pasteur, Paris	Angela Brisebarre; Brieuc Gestin; Camille Capel; Elise Liscoet; Etienne Simon-Lorière; Marion Barbet; Maud Vanpeene; Méline Bizard; Sylvie Behillil; Sylvie van der Werf; Vincent Enouf
EPI_ISL_514425 to 514431, EPI_ISL_541649 to 541655, EPI_ISL_541657 to 541662, EPI_ISL_582530, EPI_ISL_644565 to 644567	Laboratory Diagnostic, Veterinary Specialized Institute Kraljevo	Laboratory Diagnostic, Veterinary Specialized Institute Kraljevo	Afonso, C.; Banovic Djeri, B.; Jankovic, M.; Jovanovic, T.; Knezevic, A.; Petrovic, T.; Sekler, M.; Tesovic, B.; Vidanovic, D.; Volkening, J.
EPI_ISL_445086 to 445087, EPI_ISL_482744, EPI_ISL_541656	Laboratory Diagnostic, Veterinary Specialized Institute Kraljevo	Laboratory Diagnostic, Veterinary Specialized Institute Kraljevo	Afonso, C.; Banovic Djeri, B.; C.L.; Debeljak, Z.; Dmitric, M.; J. and Alfonso; J. and Alfonso, C.; Jankovic, M.; Jovanovic, T.; Knezevic, A.; Matovic, K.; Petrovic, T.; Sekler, M.; Tesovic, B.; Vaskovic, N.; Vidanovic, D.; Volkening; Volkening, J.
EPI_ISL_754902, EPI_ISL_754904 to 754913	Laboratory Diagnostics and Clinical Immunology of Developmental Age, Medical University of Warsaw	genXone SA, Research & Development Laboratory; The Faculty of Mathematics, Informatics and Mechanics of the University of Warsaw	Anna Gambin; Grzegorz Nowicki; Jakub Grabowski; Maciej Sykulski; Micha Kaszuba; Monika Makowska-Woniak; Natalia Drwska-Matelska; Urszula Demkow; ukasz Krych
EPI_ISL_476221	Laboratory Fleury	Instituto de Medicina Tropical da Univesidade de São Paulo	Camila Alves Maia da Silva; Darlan da Silva Candido; Erika Regina Manuli; Ester Sabino; Flavia Cristina da Silva Sales; Giula Magalhaes Ferreira; Jaqueline Goes de Jesus; Julien Theze; Mariana Severo Ramundo; Nuno Faria; Samples: Celso Granato; Sequencing: Ingra Morales Claro; Thais de Moura Coletti
EPI_ISL_415741 to 415743, EPI_ISL_417519 to 417525, EPI_ISL_424969 to 424975, EPI_ISL_424978, EPI_ISL_444274, EPI_ISL_444276 to 444278, EPI_ISL_452178 to 452179, EPI_ISL_464092 to 464094, EPI_ISL_660543 to 660545, EPI_ISL_667807 to 667809, EPI_ISL_956325			
see above	Laboratory Medicine	Department of Laboratory Medicine, Lin-Kou Chang Gung Memorial Hospital, Taoyuan, Taiwan	Cheng-Hsun Chiu; Cheng-Ta Yang; Chi-Hsien Huang; Chung-Guei Huang; Guang-Wu Chen; Kuang-Tso Le; Kuo-Chien Tsao; Kuo-Ming Lee; Mei-Jen Hsiao; Peng-Nien Huang; Po-Wei Huang; Shin-Ru Shih; Shu-Li Yang; Shu-Min Lin; Yi-Chun Liu; Yu-Nong Gong
EPI_ISL_411915	Laboratory Medicine	Department of Laboratory Medicine, Lin-Kou Chang Gung Memorial Hospital, Taoyuan, Taiwan.	Chung-Guei Huang; Kuo-Chien Tsao; Shin-Ru Shih; Shu-Li Yang; Yhu-Chering Huang; Yi-Chun Li; Yu-Nong Gong
EPI_ISL_529208 to 529212	Laboratory Medicine, University of Washington	University of Washington, Laboratory Medicine	Greninger, A.; Jerome, K.; Roychoudhury, P.
EPI_ISL_445243, EPI_ISL_455468 to 455477	Laboratory for Respiratory Viruses, Cantacuzino National Military-Medical Institute for Research and Development	Cantacuzino Institute	A.Cretu; L.Ustea; M.Lazar; T.Durfee; Tim Durfee
EPI_ISL_754238, EPI_ISL_861710, EPI_ISL_935017, EPI_ISL_939647, EPI_ISL_940144, EPI_ISL_940554, EPI_ISL_981374 to 981375, EPI_ISL_981377			
see above	Laboratory for Respiratory Viruses, Cantacuzino National Military-Medical Institute for Research and Development	Cantacuzino Institute Virology	Catalina Pascu; Luiza Ustea; Mihaela Lazar; Mühlemann Barbara; Nicoleta Paraschiv; Nicoleta Parashiv; Tim Durfee
EPI_ISL_471416 to 471417, EPI_ISL_471419 to 471420, EPI_ISL_471422 to 471424	Laboratory for Respiratory Viruses, National Influenza Centre, Cantacuzino National Military-Medical Institute for Research and Development	Cantacuzino Institute	Luiza Ustea; Mihaela Lazar; Nicoleta Paraschiv; Tim Durfee
EPI_ISL_437689	Laboratory for Urgent Response to Biological Threats	Institut Pasteur CIBU / ERI	A. Kwasiborski; C. Balière; C. Batéjat; J. Vanhomwegen; JC. Manuguerra; V. Caro; V. Hourdel
EPI_ISL_437690	Laboratory for Urgent Response to Biological Threats	Institut Pasteur CIBU /ERI	A. Kwasiborski; C. Balière; C. Batéjat; H. Hourdel; J. Vanhomwegen; JC. Manuguerra; V. Caro
EPI_ISL_435045 to 435048	Laboratory of Applied Genetics	RSE "National Center for Biotechnology"	Alexandr Shevtsov; Asylulan Amirgazin; Ilyas Akhmetollayev; Ruslan Kalendar; Viktoriya Lutsay; Yerlan Ramanculov
EPI_ISL_572398	Laboratory of Biology and Identification of Arboviruses	Pathogenic Microorganisms Variability Laboratory	Alexander Gintsburg; Alexey Prilipov; Alexey Shchetinin; Alina Kozlova; Andrei Siniavin; Maria Nikiforova; Muhammad Saifullin; Victor Larichev; Vladimir Gushchin

EPI_ISL_477160, EPI_ISL_477163	Laboratory of Dr. John Lednicky	University of Florida	Chang-Yu Wu; John A. Lednicky; John Glenn Morris, Jr.; Kuttichantran Subramaniam; Maha A. Elbadry; Thomas B. Waltzek; and John Glenn Morris, Jr.
EPI_ISL_437625	Laboratory of Genomics & Bioinformatics, Institute of Immunology and Experimental Therapy, Polish Academy of Sciences Oddzial Mikrobiologii Wojewodzkiej Stacji Sanitarno-Epidemiologicznej.	Laboratory of Genomics & Bioinformatics, Institute of Immunology and Experimental Therapy, Polish Academy of Sciences	Aleksandra Herud; Dariusz Martynowski; Dorota Kujawa; Grazyna Zalewska; Joanna Sikorska; Krzysztof Jakub Pawlik; Oskar Karpinski and Lukasz Laczmanski; Paulina Zebrowska
EPI_ISL_536399	Laboratory of Immunovirology. Universidad de Antioquia	Instituto Nacional de Salud - Unidad de Secuenciación y Genómica	Carlos Franco-Muñoz; Diego Álvarez-Diaz and Marcela Mercado-Reyes; Francisco J. Díaz; Katherine Laiton-Donato; Lizdany Flórez; Wbeimar Aguilar-Jimenez
EPI_ISL_452327 to 452364	Laboratory of Infectious Diseases Center of Beijing Ditan Hospital	Laboratory of Infectious Diseases Center of Beijing Ditan Hospital	Chengjie Jie; Fengting Yu; Linghang Wang; Liting Yan; Siyuan Yang; Yunxia Tang
EPI_ISL_417445, EPI_ISL_417447	Laboratory of Infectious Diseases, Department of Biomedical and Clinical Sciences L. Sacco, University of Milan	Laboratory of Infectious Diseases, Department of Biomedical and Clinical Sciences L. Sacco, University of Milan	Agostino Riva; Alessia Lai; Annalisa Bergna; Arianna Gabrieli; Claudia Balotta; Dario Bernacchia; Gianguglielmo Zehender; Giuliano Rizzardini; Luca Meroni; Maciej Tarkowski; Massimo Galli; Spinello Antinori; Stefano Rusconi
EPI_ISL_717979	Laboratory of Microbiology and Infectious Diseases, Faculty of Veterinary Medicine, Aristotle University of Thessaloniki, University Campus, 541 24, Thessaloniki, Greece.	Laboratory of Biology, Department of Medicine, Democritus University of Thrace, Alexandroupolis, Greece	Bampali M.; Chaintoutis S.; Dimitriou M.; Dovas C.; Dovrolis N.; Karakasiliotis I.
EPI_ISL_944785	Laboratory of Microbiology and Virology, Ospedale Amedeo di Savoia, ASL "Città di Torino"	U.O. Genomics, S.S. Genetics and Advanced Omics Techniques, Istituto Zooprofilattico Sperimentale del Piemonte Liguria e Valle d'Aosta	Chiara Beltramo; Elena Bozzetta; Francesco Cerutti; Pier Luigi Acutis; Simone Peletto; Valeria Ghisetti
EPI_ISL_882652	Laboratory of Microbiology and Virology, Ospedale Amedeo di Savoia, ASL "Città di Torino"	U.O. Genomics, S.S. Genetics and Advanced Omics Techniques, Istituto Zooprofilattico Sperimentale del Piemonte, Liguria e Valle d'Aosta	Chiara Beltramo; Elena Bozzetta; Francesco Cerutti; Pier Luigi Acutis; Simone Peletto; Valeria Ghisetti
EPI_ISL_418263 to 418265	Laboratory of Microbiology, Department of Medicine, National and Kapodistrian University of Athens, Greece	Laboratory of Biology, Department of Medicine, Democritus University of Thrace, Greece	Elisavet Gatzidou; Ioannis Karakasiliotis; Maria Bampali; Nikolaos Dovrolis; Nikolaos Spanakis; Stavroula Veletza
EPI_ISL_427043, EPI_ISL_434455 to 434486, EPI_ISL_437874 to 437911	Laboratory of Microbiology, Medical School, National and Kapodistrian University of Athens	Laboratory of Biology, Department of Medicine, Democritus University of Thrace	Bampali, M.; Dovrolis, N.; Froukala, E.; Gatzidou, E.; Kassela K.; N. and Karakasiliotis, I.; Spanakis; Stavropoulou, A.; Tsakris, A.; Veletza, S.
EPI_ISL_654016 to 654020	Laboratory of Microbiology, National Reference Lab, Charles Nicolle Hospital; 2-University of Tunis ElManar, Faculty of Medicine of Tunis, LR99ES09, Tunis, Tunisia	1-Clinical and Experimental Pharmacology Lab, LR16SP02, National Center of Pharmacovigilance, University of Tunis El Manar, Tunis, Tunisia. 2-Neurodegenerative diseases and psychiatric troubles, LR18SP03, Razi Hospital, University of Tunis El Manar, Tunis, Tunisia. 3- Ministry of Health, National Observatory of New and Emerging Diseases, 1006, Tunis, Tunisia	Alia Ben Kahla; Gaies Emna; Ilhem Boutiba-Ben Boubaker; Imen Kacem; Imen Mkada; Jalila Ben Khelil; Maher Kharrat; Mouna Ben Sassi; Mouna Safer; Nissaf Ben Alaya; Riadh Daghfous; Riadh Gouider.; Salma Abid; Sameh Trabelsi; Sana Ferjani; Soumaya Rammeh
EPI_ISL_613563 to 613564, EPI_ISL_613707 to 613708	Laboratory of Molecular Biology, Blood Center of Ribeirão Preto	Laboratory of Molecular Biology, Blood Center of Ribeirão Preto, Faculty of Medicine of Ribeirão Preto, University of São Paulo	Aparecida Y Yamamoto; Diego Villa Clé; Dimas T Covas; Elaine V Santos; Evandra S Rodrigues; Glauco de Carvalho Pereira; Joilson Xavier; Luiz CJ Alcantara; Marta Giovanetti; Rodrigo T Calado; Simone Kashima; Svetoslav N Slavov; Talita Adelino; Vagner Fonseca
EPI_ISL_613709, EPI_ISL_613951	Laboratory of Molecular Biology, Blood Center of Ribeirão Preto, Faculty of Medicine of Ribeirão Preto, University of São Paulo	Laboratory of Molecular Biology, Blood Center of Ribeirão Preto, Faculty of Medicine of Ribeirão Preto, University of São Paulo	Aparecida Y Yamamoto; Diego Villa Clé; Dimas T Covas; Elaine V Santos; Evandra S Rodrigues; Glauco de Carvalho Pereira; Joilson Xavier; Luiz CJ Alcantara; Marta Giovanetti; Rodrigo T Calado; Simone Kashima; Svetoslav N Slavov; Talita Adelino; Vagner Fonseca
EPI_ISL_428209, EPI_ISL_450746, EPI_ISL_451644 to 451647	Laboratory of Molecular Biology, Diagnostyka sp. z o.o.	Laboratory of Recombinant Vaccines	Anna Piotrowska-Mietelska; Boguslaw Szewczyk; Krystyna Bienkowska-Szewczyk; Lukasz Rabalski; Maciej Kosinski
EPI_ISL_852842	Laboratory of Molecular Biology, Diagnostyka sp. z o.o.	genXone SA, Research & Development Laboratory	Grzegorz Nowicki; Jakub Grabowski; Maciej Sykulski; Micha Kaszuba; Natalia Drwska-Matelska; ukasz Krych
EPI_ISL_425228 to 425229, EPI_ISL_426051, EPI_ISL_426356 to 426360	Laboratory of Molecular Genetics, 2nd Faculty of Medicine, Charles University in Prague, Prague, Czech Republic	Laboratory of Molecular Genetics, 2nd Faculty of Medicine, Charles University in Prague, Prague, Czech Republic	Katerina Polackova; Kateina Poláková; Lenka Kramna; Lenka Kramná; Ondrej Cinek; Ondej Cinek
EPI_ISL_625673 to 625682	Laboratory of Molecular Medicine, University of Magallanes	Centro Asistencial Docente y de Investigacion, Universidad de Magallanes	Diego Alvarez; Jacqueline Aldridge; Jorge Gonzalez; Marcelo Navarrete
EPI_ISL_498558 to 498563	Laboratory of Molecular Virology International Center for Genetic Engineering and Biotechnology (ICGEB)	ARGO Open Lab Platform for Genome Sequencing	Dal Monego S; D'Agaro P; Licastro D; Marcello A; Rajasekharan S; Segat L
EPI_ISL_417419, EPI_ISL_417421, EPI_ISL_428854	Laboratory of Molecular Virology International Center for Genetic Engineering and Biotechnology (ICGEB)	ARGO Open Lab Platform for Genome sequencing	D'Agaro P; Dal Monego S; Licastro D; Marcello A; Rajasekharan S; Segat L
EPI_ISL_417418	Laboratory of Molecular Virology International Center fro Genetic Engineering and Biotechnology (ICGEB)	ARGO Open Lab Platform for Genome sequencing	D'Agaro P; Dal Monego S; Licastro D; Marcello A; Rajasekharan S; Segat L
EPI_ISL_479616 to 479619, EPI_ISL_479790 to 479791, EPI_ISL_525495 to 525496	Laboratory of Molecular Virology of the International Centre for Genetic Engineering and Biotechnology (ICGEB)	ARGO Open Lab Platform for Genome Sequencing	Confalonieri M; Confalonieri M Marcello A; Confalonieri P; D; D'Agaro P; Dal Monego S; Licastro; Licastro D; Marcello A; Rajasekharan S; Salton F; Segat L
EPI_ISL_584048 to 584049, EPI_ISL_584052	Laboratory of Molecular Virology, Department of Biomedical, Surgical and Dental Sciences University of Milano	Laboratory of Molecular Virology, Department of Biomedical, Surgical and Dental Sciences University of Milano	B. and Ferrante, P.; Basilico, N.; Bianchi, M.; Binda, S.; C. and Ferrante, P.; D'Alessandro, S.; Delbue, S.; Dolci, M.; Fattori, M.; Galli; Incorvaia; Modenese, A.; Parapini, S.; Pariani, E.; Primache, V.; Signorini, L.; Taramelli, D.
EPI_ISL_415658, EPI_ISL_415660 to 415661	Laboratory of Molecular Virology, Pontificia Universidad Católica de Chile	MSHS Pathogen Surveillance Program	Ajay Obla; Ana Silvia Gonzalez-Reiche; Eileen Serrano; Hala Alshammary; Harm van Bakel; Juan Soto; Matthew Hernandez; Melissa Smith; Mitchell Sullivan; Pablo Vial; Rafael A. Medina; Robert Sebra; Shwetha Sridhar Hara; Tamara Garcia; Viviana Simon; Ying-Chih Wang; Zenab Khan
EPI_ISL_427294 to 427304, EPI_ISL_456071 to 456075, EPI_ISL_456079 to 456081, EPI_ISL_456084 to 456087, EPI_ISL_456089 to 456106, EPI_ISL_467344 to 467371, EPI_ISL_541347 to 541369			
see above	Laboratory of Respiratory Viruses and Measles, Oswaldo Cruz Institute, FIOCRUZ	Laboratory of Respiratory Viruses and Measles, Oswaldo Cruz Institute, FIOCRUZ	Aline Mattos; Ana Carolina Mendonca; Ana Carolina Mendonça; Anna Carolina Paixao; Anna Carolina Paixão; Braulia Caetano; Cinthia Avila; Cristiana Garcia; Fernando Motta; Jonathan Lopes; Luciana Appolinario; Maria Ogrzewalska; Marilda Siqueira on behalf of the Fiocruz COVID-19 Genomic Surveillance Network; Milene Miranda; Paola Resende; Priscila Born; Roxana Loayza; Sunando Roy
EPI_ISL_939628 to 939629, EPI_ISL_939643, EPI_ISL_939649, EPI_ISL_940146, EPI_ISL_940157, EPI_ISL_941163, EPI_ISL_945547, EPI_ISL_946913, EPI_ISL_949177 to 949178, EPI_ISL_949181, EPI_ISL_953426, EPI_ISL_956416, EPI_ISL_962890, EPI_ISL_964881, EPI_ISL_964950, EPI_ISL_965142, EPI_ISL_965145			
see above	Laboratory of Virology and Molecular Diagnostics	Institute of Public Health of Republic of North Macedonia Laboratory of Virology and Molecular Diagnostics	Boshevska G.; Elizabeta Janchevska; G. Boshevska; Golubinka Bosehvska; Golubinka Boshevska; Kuzmanovska M.; M. Kuzmanovska; Maja Kuzmanovska; Maja Kuzmanovska. Golubinka Boshevska
EPI_ISL_451298 to 451304	Laboratory of Virology, INMI Lazzaro Spallanzani IRCCS	Laboratory of Virology, INMI Lazzaro Spallanzani IRCCS	Antonino Di Caro; Barbara Bartolini; Cesare E.M. Gruber; Francesco Messina; Giuseppe Ippolito; Maria R. Capobianchi; Martina Rueca
EPI_ISL_454575	Laboratory of virology, National Center of Expertise	Laboratory of molecular-genetic research, National Center for Expertise, Kazakhstan National Center for Biotechnology, Kazakhstan	; Abdaliyev Askar; Akhmetollayev Ilyas; Amirgazin Asylulan; Aushakhmetova Zabira; Kalendar Ruslan; Lutsay Viktoriya; Rakhmetova Akbota; Ramankulov Yerlan; Shevtsov Alexandr
EPI_ISL_454584 to 454586, EPI_ISL_454589	Laboratory of virology, National Center of Expertise	Laboratory of molecular-genetic research, National Center for Expertise, Kazakhstan National Center for Biotechnology, Kazakhstan	; Abdaliyev Askar; Akhmetollayev Ilyas; Amirgazin Asylulan; Aushakhmetova Zabira; Kalendar Ruslan; Lutsay Viktoriya; Rakhmetova Akbota; Ramankulov Yerlan; Shevtsov Alexandr
EPI_ISL_454596 to 454597, EPI_ISL_454599 to 454600, EPI_ISL_454604	Laboratory of virology, National Center of Expertise	Laboratory of molecular-genetic research, National Center for Expertise, Kazakhstan National Center for Biotechnology, Kazakhstan	; Abdaliyev Askar; Akhmetollayev Ilyas; Amirgazin Asylulan; Aushakhmetova Zabira; Kalendar Ruslan; Lutsay Viktoriya; Rakhmetova Akbota; Ramankulov Yerlan; Shevtsov Alexandr

EPI_ISL_454576 to 454577, EPI_ISL_454579 to 454580, EPI_ISL_454582, EPI_ISL_454587, EPI_ISL_454590 to 454591, EPI_ISL_454593 to 454594, EPI_ISL_454598, EPI_ISL_454601, EPI_ISL_454603			
see above	Laboratory of virology, National Center of Expertise	Laboratory of molecular-genetic research, National Center of Expertise, Kazakhstan National Center for Biotechnology, Kazakhstan	; Abdaliyev Askar; Akhmetollayev Ilyas; Amirgazin Asylulan; Aushakhmetova Zabira; Kalendar Ruslan; Lutsay Viktoriya; Rakhmetova Akbota; Ramankulov Yerlan; Shevtsov Alexandr
EPI_ISL_576117 to 576118, EPI_ISL_576120 to 576121, EPI_ISL_576125	Laboratory, The Bio Arte Limited	Laboratory, The Bio Arte Limited	Biazzo, M.; Madeddu, S.; Pinzauti, D.; Santoro, F.
EPI_ISL_414045, EPI_ISL_456076 to 456077, EPI_ISL_456082 to 456083, EPI_ISL_456088	Laboratório Central de Saúde Pública Noel Nutels (LACEN-RJ)	Laboratory of Respiratory Viruses and Measles, Oswaldo Cruz Institute, FIOCRUZ	Aline Mattos; Alisson Fabri; Braulia Caetano; Cristiana Garcia; Fernando Motta; Joilson Xavier; Jonathan Lopes; Luciana Appolinario; Maria Nóbrega; Maria Ogrzewalska; Marilda Siqueira on behalf of the Fiocruz COVID-19 Genomic Surveillance Network; Marilda SiqueiraFiocruz COVID-19 Genomic Surveillance Network; Milene Miranda; Paola Resende; Sunando Roy
EPI_ISL_415105, EPI_ISL_427293	Laboratório Central de Saúde Pública Professor Gonçalo Moniz (LACEN-BA)	Laboratory of Respiratory Viruses and Measles, Oswaldo Cruz Institute, FIOCRUZ	Aline Mattos; Allison Fabri; Braulia Caetano; Cristiana Garcia; Fernando Motta; Joilson Xavier; Jonathan Lopes; Luciana Appolinario; Maria Nóbrega; Maria Ogrzewalska; Marilda Siqueira Fiocruz COVID-19 Genomic Surveillance Network; Marilda Siqueira on behalf of the Fiocruz COVID-19 Genomic Surveillance Network; Milene Miranda; Paola Resende; Priscila Born; Sunando Roy
EPI_ISL_427292	Laboratório Central de Saúde Pública do Estado de Alagoas (LACEN-AL)	Laboratory of Respiratory Viruses and Measles, Oswaldo Cruz Institute, FIOCRUZ	Aline Mattos; Braulia Caetano; Cristiana Garcia; Fernando Motta; Jonathan Lopes; Luciana Appolinario; Maria Ogrzewalska; Marilda Siqueira on behalf of the Fiocruz COVID-19 Genomic Surveillance Network; Milene Miranda; Paola Resende; Priscila Born; Sunando Roy
EPI_ISL_500460 to 500463, EPI_ISL_500471, EPI_ISL_500473, EPI_ISL_500475, EPI_ISL_500477, EPI_ISL_500480 to 500486, EPI_ISL_500872, EPI_ISL_572334 to 572335, EPI_ISL_572351, EPI_ISL_572353, EPI_ISL_572366, EPI_ISL_572371, EPI_ISL_572386			
see above	Laboratório Central de Saúde Pública do Estado de Pernambuco (LACEN-PE)	WallauLab, Aggeu Magalhaes Institute	Alexandre Freitas da Silva; Antonio Mauro Rezende; Armando de Menezes Neto; Bruna Santos Lima Figueiredo de Sá; Caroline Targino Alves da Silva; Claudio Eduardo Cavalcanti; Constância Flávia Junqueira Ayres; Cássia Docena; Derciliano Lopes da Cruz; Duschinka Ribeiro Duarte Guedes; Elisama Helvecio; Filipe Zimmer Dezordi; Gabriel Luz Wallau on behalf of the Fiocruz COVID-19 Genomic Surveillance Network; Gonzalo Bello; Kamila Gaudêncio da Silva Sales; Larissa Krokovsky; Laís Ceschini Machado; Luciane Caroline Albuquerque Bezerra; Luydson Richardson Silva Vasconcelos; Marcelo Henrique Santos Paiva; Maria Almerice Lopes da Silva; Matheus Filgueira Bezerra; Michelle da Silva Barros; Paola Cristina Resende; Renata Pessôa Germano Mendes; Rodrigo Moraes Loyo Arcoverde; Severino Jefferson Ribeiro da Silva; Sinval Pinto Brandão Filho; Tiago Gräf; Wheverton Ricardo Correia do Nascimento
EPI_ISL_427305 to 427306, EPI_ISL_541370	Laboratório Central de Saúde Pública do Estado de Santa Catarina (LACEN-SC)	Laboratory of Respiratory Viruses and Measles, Oswaldo Cruz Institute, FIOCRUZ	Aline Mattos; Ana Carolina Mendonça; Anna Carolina Paixão; Braulia Caetano; Cristiana Garcia; Fernando Motta; Jonathan Lopes; Luciana Appolinario; Maria Ogrzewalska; Marilda Siqueira on behalf of the Fiocruz COVID-19 Genomic Surveillance Network; Milene Miranda; Paola Resende; Priscila Born; Sandra Bianchini; Sunando Roy
EPI_ISL_541372 to 541391, EPI_ISL_541393 to 541396	Laboratório Central de Saúde Pública do Estado de Sergipe (LACEN-SE)	Laboratory of Respiratory Viruses and Measles, Oswaldo Cruz Institute, FIOCRUZ	Ana Carolina Mendonça; Anna Carolina Paixão; Clioma Santos; Fernando Motta; Jonathan Lopes; Luciana Appolinario; Marilda Siqueira on behalf of the Fiocruz COVID-19 Genomic Surveillance Network; Paola Resende
EPI_ISL_415128	Laboratório Central de Saúde Pública do Estado do Espírito Santo (LACEN-ES)	Laboratory of Respiratory Viruses and Measles, Oswaldo Cruz Institute, FIOCRUZ	Aline Mattos; Allison Fabri; Braulia Caetano; Cristiana Garcia; Fernando Motta; Joilson Xavier; Jonathan Lopes; Luciana Appolinario; Maria Nóbrega; Maria Ogrzewalska; Marilda Siqueira on behalf of the Fiocruz COVID-19 Genomic Surveillance Network; Milene Miranda; Paola Resende; Sunando Roy
EPI_ISL_541340 to 541346	Laboratório Central de Saúde Pública do Estado do Paraná (LACEN-PR)	Laboratory of Respiratory Viruses and Measles, Oswaldo Cruz Institute, FIOCRUZ	Ana Carolina Mendonça; Anna Carolina Paixão; Fernando Motta; Irina Riediger; Jonathan Lopes; Luciana Appolinario; Maria do Carmo Debur; Marilda Siqueira on behalf of the Fiocruz COVID-19 Genomic Surveillance Network; Paola Resende
EPI_ISL_729794, EPI_ISL_729800 to 729861	Laboratório Central de Saúde Pública do Estado do Rio Grande do Sul (LACEN-RS)	Laboratory of Respiratory Viruses and Measles, Oswaldo Cruz Institute, FIOCRUZ	Ana Carolina Mendonça; Anna Carolina Paixão; Fernando Motta; Luciana Appolinario; Marilda Siqueira on behalf of the Fiocruz COVID-19 Genomic Surveillance Network; Marilda Tereza Mar da Rosa; Paola Resende; Tatiana Schaffer Gregianini
EPI_ISL_693220, EPI_ISL_693223 to 693224, EPI_ISL_693243	Laboratório Municipal de Piracicaba	Instituto Adolfo Lutz, Interdiciplinary Procedures Center, Strategic Laboratory	Claudia Regina Gonçalves; Claudio Tavares Sacchi; Erica Valessa Ramos Gomes; Karoline Rodrigues Campos
EPI_ISL_636737, EPI_ISL_636834 to 636835, EPI_ISL_636837	Laboratório de Imunofarmacologia - Instituto Oswaldo Cruz	Laboratório de Imunofarmacologia - Instituto Oswaldo Cruz	A.D.; C.Q.; De Paula; F.B.; Ferreira; Fintelman-Rodrigues, N.; M.A. and Sacramento; Saraiva; Souza; T.M.
EPI_ISL_476341, EPI_ISL_476395, EPI_ISL_476398	Laboratório de Patologia Clínica - UNICAMP	Laboratório de Estudos de Vírus Emergentes - UNICAMP	Angelica Schreiber; Camila Simeoni; Darlan da Silva Candido; Jaqueline Goes Jesus e William Marciel de Souza; José Luiz Proença-Modena; Julia Forato; Julien Theze; Luiz Gonzaga; Magnun Nueldo Nunes dos Santos; Marcilio Jorge Fumagalli; Mariene Ribeiro Amorim; Nuno Rodrigues Faria
EPI_ISL_861857 to 861861, EPI_ISL_861863, EPI_ISL_896073, EPI_ISL_896077, EPI_ISL_896079 to 896084, EPI_ISL_896087 to 896088, EPI_ISL_896090, EPI_ISL_896096 to 896097, EPI_ISL_896099, EPI_ISL_896103 to 896104, EPI_ISL_896106			
see above	Labormedizinisches Zentrum Dr Risch	University Hospital Basel, Clinical Bacteriology	Adrian Egli; Alfredo Mari; Hans Hirsch; Helena MB Seth-Smith; Julia Bielicki; Karoline Leuzinger; Lorenz Risch; Madlen Stange; Manuel Battegay; Martin Risch; Nadia Wohlwend; Tim Roloff
EPI_ISL_900513, EPI_ISL_900524	Labosud	CNR Virus des Infections Respiratoires - France SUD	Antonin Bal; Bruno Lina; Gregory Destras; Gwendolyne Burfin; Hadrien Règue; Laurence Josset; Martine Valette; Quentin Semanas
EPI_ISL_700466	Ladismith (Nissenville) Clinic wc LAF	NHLS/UCT	Arash Iranzadeh; Bruna Galvao; Carolyn Williamson; Deelan Doolabh; Diana Hardie; Innocent Mudau; Kruger Marais; Lynn Tyers; Marvin Hsiao; Stephen Korsman
EPI_ISL_434669, EPI_ISL_475515	Lakargruppen	The Public Health Agency of Sweden	Anna Risberg; Anna-Malin Linde; Boris Klanger; Karin Tegmark-Wisell; Maria Lind Karlberg; Mattias Haukland; Mia Brytting; Olov Svartstrom; Oskar Karlsson Lindsjo; Reza Advani; Sandra Broddesson; Theresa Enkirch
EPI_ISL_623073	Lancet Laboratories	National Institute for Communicable Diseases of the National Health Laboratory Service	Allam M; Bhiman JN; Ismail A; Khumalo Z; Kwenda S; Mnyameni F; Mohale T; Mtshali P; Subramoney K
EPI_ISL_960891 to 960892, EPI_ISL_960894 to 960898, EPI_ISL_1034812 to 1034814, EPI_ISL_1034817	Landesamt für Verbraucherschutz Sachsen Anhalt, Magdeburg	Institute of Medical Microbiology and Hospital Hygiene	Aljoscha Tersteegen; Prof. Dr. Achim Kaasch
EPI_ISL_1038795	Landstuhl Regional Medical Center	US Air Force School of Aerospace Medicine	Amanda Javorina; Anthony Fries; Clarise Starr; Cole Anderson; Elizabeth Macias; Fritz Castillo; Jennifer Meyer; Sarah Purves; William Buggele; William Gruner
EPI_ISL_738326 to 738349	Landstuhl Regional Medical Center	United States Air Force School of Aerospace Medicine	Amanda Javorina; Anthony Fries; Clarise Starr; Cole Anderson; Elizabeth Macias; Fritz Castillo; Jennifer Meyer; Sarah Purves; William Gruner
EPI_ISL_534237 to 534238, EPI_ISL_548255	Lanssjukhuset Kalmar	The Public Health Agency of Sweden	Anna Risberg; Anna-Malin Linde; Karin Tegmark-Wisell; Maria Lind Karlberg; Mattias Haukland; Mia Brytting; Olov Svartstrom; Oskar Karlsson Lindsjo; Petra Edquist; Reza Advani; Sandra Broddesson
EPI_ISL_407079	Lapland Central Hospital	Department of Virology, University of Helsinki and Helsinki University Hospital, Helsinki, Finland	Hannimari Kallio-Kokko; Olli Vapalahti; Suvi Kuivanen; Teemu Smura
EPI_ISL_437089 to 437096, EPI_ISL_486422 to 486426, EPI_ISL_486428, EPI_ISL_486430 to 486436, EPI_ISL_512647 to 512652, EPI_ISL_515185 to 515186, EPI_ISL_534204 to 534205, EPI_ISL_534209, EPI_ISL_639634, EPI_ISL_639639, EPI_ISL_770047, EPI_ISL_770060			
see above	Latvijas Infektoloijas centrs	Latvian Biomedical Research and Study Centre	Ivars Silamielis; Jeena Storoženko; Jnis Kloviš; Jnis Pjalkovskis; Kaspars Megnis; Monta Ustinova; Oksana Savicka; Tatjana Kolupajeva; Uga Dumpis; Vita Rovte; ikita Zrelovs
EPI_ISL_745095	Laverty Pathology	CIDM-PH, Westmead Hospital	CIDM-PH et al.
EPI_ISL_451489, EPI_ISL_451545, EPI_ISL_451572, EPI_ISL_451602, EPI_ISL_451609, EPI_ISL_451611, EPI_ISL_451631, EPI_ISL_451641, EPI_ISL_455041, EPI_ISL_455048, EPI_ISL_455063, EPI_ISL_498769 to 498772, EPI_ISL_513403 to 513408, EPI_ISL_526143, EPI_ISL_526202 to 526204, EPI_ISL_544957, EPI_ISL_544962, EPI_ISL_545040, EPI_ISL_591487 to 591491, EPI_ISL_593653, EPI_ISL_629007 to 629009, EPI_ISL_639731, EPI_ISL_639733, EPI_ISL_740892 to 740893, EPI_ISL_767869, EPI_ISL_767878 to 767880, EPI_ISL_767893, EPI_ISL_767899, EPI_ISL_767903, EPI_ISL_767919, EPI_ISL_767938			
see above	Laverty Pathology	NSW Health Pathology - Institute of Clinical Pathology and Medical Research; Westmead Hospital; University of Sydney	CIDM-PH et al.
EPI_ISL_421507 to 421508	Le Château de Seine-Port	National Reference Center for Viruses of Respiratory Infections, Institut Pasteur, Paris	Angela Brisebarre; Etienne Simon-Lorière; Flora Donati; Marion Barbet; Maud Vanpeene; Mélanie Albert; Méline Bizard; Sylvie Behillil; Sylvie van der Werf; Vincent Enouf
EPI_ISL_498551 to 498552, EPI_ISL_498554	Lebanese American University	Lebanese American University	Abdallah, J.; Abi Habib, W.; El Shesheny, R.; Goldstein; J. and Kayali, G.; Mokhbat, J.; R.J.; Webby

EPI_ISL_447588	Lednicky Lab	Lednicky lab	Alam; C.J.; Elbadry; Gibson; J.A.; J.C.; J.G. Jr. and Lednicky; M.A.; M.M.; Morris; Stephenson; Subramaniam, K.; T.B.; Waltzek
EPI_ISL_434677	Lednicky Laboratory at Emerging Pathogens Institute	Lednicky Laboratory at Emerging Pathogens Institute	C.-Y.; Clugston; Elbadry; J.A.; J.G. Jr. and Lednicky; J.R.; M.A.; Morris; S.N.; Shankar; Wu
EPI_ISL_434636	Lednicky Laboratory, Emerging Pathogens Institute, University of Florida	Lednicky Laboratory at Emerging Pathogens Institute, University of Florida	Alam, M.; C.J.; Elbadry; Gibson; J.A.; J.C.; J.G. Jr. and Lednicky; M.A.; Morris; Stephenson; Subramaniam, K.; T.B.; Waltzek
EPI_ISL_434637	Lednicky Laboratory, Emerging Pathogens Institute, University of Florida.	Lednicky Laboratory, Emerging Pathogens Institute, University of Florida.	C.J.; Elbadry; Gibson; J.A.; J.C.; J.G. Jr. and Lednicky; M.A.; Morris; Stephenson; Subramaniam, K.; T.B.; Waltzek

EPI_ISL_538748 to 538753, EPI_ISL_538755 to 538762, EPI_ISL_538764 to 538770, EPI_ISL_538772 to 538774, EPI_ISL_538776 to 538784, EPI_ISL_538786 to 538800, EPI_ISL_538802 to 538810, EPI_ISL_538812 to 538817, EPI_ISL_538819 to 538840, EPI_ISL_538842 to 538849, EPI_ISL_538852 to 538862, EPI_ISL_538864 to 538865, EPI_ISL_538867 to 538872, EPI_ISL_538874, EPI_ISL_538877 to 538883, EPI_ISL_538885 to 538897, EPI_ISL_538900 to 538916, EPI_ISL_538918 to 538926, EPI_ISL_538928 to 538944, EPI_ISL_538946 to 538963, EPI_ISL_538965 to 538986, EPI_ISL_538988 to 538993, EPI_ISL_538995 to 538996, EPI_ISL_538998 to 539004, EPI_ISL_539007 to 539008, EPI_ISL_539010, EPI_ISL_539013 to 539018, EPI_ISL_539020 to 539024, EPI_ISL_539026 to 539029, EPI_ISL_539031 to 539034, EPI_ISL_539036 to 539041, EPI_ISL_539043 to 539045, EPI_ISL_539047, EPI_ISL_539051, EPI_ISL_539053 to 539055, EPI_ISL_539058, EPI_ISL_539061, EPI_ISL_539063 to 539065, EPI_ISL_539067 to 539069, EPI_ISL_539071 to 539087, EPI_ISL_539089 to 539093, EPI_ISL_539096 to 539097, EPI_ISL_539099 to 539100, EPI_ISL_539102 to 539104, EPI_ISL_539106 to 539111, EPI_ISL_539113 to 539115, EPI_ISL_539118 to 539119, EPI_ISL_539121 to 539124, EPI_ISL_539126 to 539135, EPI_ISL_539137 to 539138, EPI_ISL_539142, EPI_ISL_539144 to 539148, EPI_ISL_539153, EPI_ISL_539156, EPI_ISL_539158 to 539160, EPI_ISL_539162 to 539163, EPI_ISL_539165 to 539167, EPI_ISL_539169 to 539176, EPI_ISL_539178 to 539180, EPI_ISL_539183 to 539184, EPI_ISL_539186 to 539187, EPI_ISL_539193 to 539196, EPI_ISL_539198 to 539200, EPI_ISL_539202 to 539203, EPI_ISL_539205 to 539209, EPI_ISL_539211 to 539212, EPI_ISL_539214 to 539219

see above	Leeds Teaching Hospitals NHS Trust and Public Health England, National Infection Service (Leeds laboratory)	Wellcome Sanger Institute for the COVID-19 Genomics UK (COG-UK) consortium	Antony Hale and Alex Alderton; Cordelia Langford; David K. Jackson; Dominic Kwiatkowski; Ewan Harrison; Holli Carden; Ian Johnston; John Sillitoe on behalf of the Wellcome Sanger Institute COVID-19 Surveillance Team; Katherine L. Harper; Louissa Macfarlane-Smith; Roberto Amato; Sonia Goncalves
EPI_ISL_1040702, EPI_ISL_1040709	Lentegeur Hospital wc LGH	NHLS/UCT	Arash Iranzadeh; Bruna Galvao; Carolyn Williamson; Deelan Doolabh; Diana Hardie; Innocent Mudau; Kruger Marais; Lynn Tyers; Marvin Hsiao; Stephen Korsman

EPI_ISL_644095 to 644097, EPI_ISL_644103 to 644106, EPI_ISL_644108 to 644111, EPI_ISL_644113, EPI_ISL_644118, EPI_ISL_645240, EPI_ISL_645266, EPI_ISL_645297, EPI_ISL_645303, EPI_ISL_645362, EPI_ISL_645368, EPI_ISL_645418, EPI_ISL_645452, EPI_ISL_645459, EPI_ISL_645464, EPI_ISL_645473, EPI_ISL_645499, EPI_ISL_645502 to 645503, EPI_ISL_645508, EPI_ISL_645530 to 645535, EPI_ISL_645537 to 645546, EPI_ISL_645548 to 645553, EPI_ISL_645555 to 645582, EPI_ISL_645584 to 645618, EPI_ISL_645620 to 645621, EPI_ISL_645623 to 645632, EPI_ISL_645635 to 645653, EPI_ISL_645655 to 645658, EPI_ISL_645660 to 645670, EPI_ISL_645673 to 645688, EPI_ISL_645690 to 645698, EPI_ISL_645700 to 645704, EPI_ISL_645706 to 645738, EPI_ISL_645740 to 645762, EPI_ISL_645764 to 645765, EPI_ISL_645767 to 645787, EPI_ISL_645789 to 645797, EPI_ISL_645799 to 645801, EPI_ISL_645803 to 645808, EPI_ISL_645810 to 645825, EPI_ISL_645827, EPI_ISL_645830 to 645831, EPI_ISL_645833 to 645835, EPI_ISL_645837, EPI_ISL_645841 to 645849, EPI_ISL_645853 to 645865, EPI_ISL_645867, EPI_ISL_645870 to 645876, EPI_ISL_645878 to 645879, EPI_ISL_645881, EPI_ISL_645883 to 645884, EPI_ISL_645887 to 645895, EPI_ISL_645898, EPI_ISL_645901, EPI_ISL_645903, EPI_ISL_645906 to 645907, EPI_ISL_645909 to 645910, EPI_ISL_645912 to 645915, EPI_ISL_645919, EPI_ISL_645921 to 645922, EPI_ISL_645924, EPI_ISL_645928 to 645929, EPI_ISL_645931 to 645944, EPI_ISL_645946 to 645948, EPI_ISL_645950 to 645952, EPI_ISL_645954 to 645955, EPI_ISL_645957, EPI_ISL_645960 to 645961, EPI_ISL_645963 to 645967, EPI_ISL_645969, EPI_ISL_645971 to 645977, EPI_ISL_645982, EPI_ISL_645984 to 645986, EPI_ISL_645988, EPI_ISL_645990 to 645991, EPI_ISL_645996 to 645997, EPI_ISL_645999 to 646008, EPI_ISL_646010 to 646013, EPI_ISL_646015 to 646016, EPI_ISL_646019, EPI_ISL_646021, EPI_ISL_646023 to 646024, EPI_ISL_646026, EPI_ISL_646029, EPI_ISL_646033, EPI_ISL_646036 to 646038, EPI_ISL_646040, EPI_ISL_646042 to 646044, EPI_ISL_646048 to 646050, EPI_ISL_646053 to 646055, EPI_ISL_646059 to 646064, EPI_ISL_646066, EPI_ISL_646068 to 646069, EPI_ISL_646071 to 646075, EPI_ISL_646078 to 646082, EPI_ISL_646087 to 646088, EPI_ISL_646090, EPI_ISL_646092 to 646093, EPI_ISL_646095, EPI_ISL_646097 to 646100, EPI_ISL_646103 to 646104, EPI_ISL_646106 to 646114, EPI_ISL_646116 to 646121, EPI_ISL_646123 to 646127, EPI_ISL_646130, EPI_ISL_646132 to 646134, EPI_ISL_646137 to 646138, EPI_ISL_646148, EPI_ISL_646150, EPI_ISL_646155 to 646157, EPI_ISL_646162, EPI_ISL_646166, EPI_ISL_646169 to 646170, EPI_ISL_646173, EPI_ISL_646180, EPI_ISL_646182, EPI_ISL_646185, EPI_ISL_646189, EPI_ISL_646191, EPI_ISL_646193, EPI_ISL_646198 to 646199, EPI_ISL_646204, EPI_ISL_646207 to 646208, EPI_ISL_646212, EPI_ISL_646215, EPI_ISL_646223 to 646224, EPI_ISL_646231, EPI_ISL_646235, EPI_ISL_646242, EPI_ISL_646245, EPI_ISL_646248 to 646249, EPI_ISL_646252, EPI_ISL_646261, EPI_ISL_646268 to 646269, EPI_ISL_646271, EPI_ISL_646276 to 646277, EPI_ISL_646279, EPI_ISL_646283, EPI_ISL_646291, EPI_ISL_646297, EPI_ISL_646299, EPI_ISL_646303, EPI_ISL_646305, EPI_ISL_646307, EPI_ISL_646309, EPI_ISL_646313, EPI_ISL_646318, EPI_ISL_646320, EPI_ISL_646327, EPI_ISL_646334, EPI_ISL_646342 to 646343, EPI_ISL_646345 to 646347, EPI_ISL_646349 to 646350, EPI_ISL_646354, EPI_ISL_646356, EPI_ISL_646362 to 646363, EPI_ISL_646366, EPI_ISL_646369 to 646370, EPI_ISL_646373, EPI_ISL_646375 to 646377, EPI_ISL_646382, EPI_ISL_646385, EPI_ISL_646388, EPI_ISL_646397, EPI_ISL_646402, EPI_ISL_646412, EPI_ISL_646418 to 646420, EPI_ISL_646426, EPI_ISL_646428 to 646431, EPI_ISL_646434, EPI_ISL_646441 to 646443, EPI_ISL_646448, EPI_ISL_646454, EPI_ISL_646463, EPI_ISL_646466 to 646468, EPI_ISL_646476, EPI_ISL_646478 to 646479, EPI_ISL_646485 to 646494, EPI_ISL_646497, EPI_ISL_646500, EPI_ISL_646502, EPI_ISL_646505 to 646506, EPI_ISL_646508 to 646509, EPI_ISL_646511 to 646512, EPI_ISL_646514 to 646516, EPI_ISL_646518, EPI_ISL_646520 to 646522, EPI_ISL_646526, EPI_ISL_646528 to 646529, EPI_ISL_646532 to 646533, EPI_ISL_646535, EPI_ISL_646537 to 646538, EPI_ISL_646540 to 646546, EPI_ISL_646548 to 646549, EPI_ISL_646551, EPI_ISL_646555, EPI_ISL_646557 to 646559, EPI_ISL_646561, EPI_ISL_646563 to 646564, EPI_ISL_646566 to 646569, EPI_ISL_646571 to 646573, EPI_ISL_646576 to 646577, EPI_ISL_646580 to 646581, EPI_ISL_646583, EPI_ISL_646585 to 646586, EPI_ISL_646591 to 646592, EPI_ISL_646594 to 646595, EPI_ISL_646597, EPI_ISL_646599 to 646601, EPI_ISL_646603, EPI_ISL_646605 to 646607, EPI_ISL_646610, EPI_ISL_646612 to 646614, EPI_ISL_646616, EPI_ISL_646618, EPI_ISL_646621, EPI_ISL_646623, EPI_ISL_646625 to 646626, EPI_ISL_646628, EPI_ISL_646631, EPI_ISL_646635, EPI_ISL_646638 to 646640, EPI_ISL_646642, EPI_ISL_646648, EPI_ISL_646651 to 646653, EPI_ISL_646655, EPI_ISL_646658, EPI_ISL_646660, EPI_ISL_646662, EPI_ISL_646664 to 646666, EPI_ISL_646668, EPI_ISL_646671, EPI_ISL_646673, EPI_ISL_646675, EPI_ISL_646683 to 646684, EPI_ISL_646686 to 646687, EPI_ISL_646689, EPI_ISL_646691 to 646693, EPI_ISL_646695 to 646698, EPI_ISL_646702, EPI_ISL_646706 to 646708, EPI_ISL_646710 to 646713, EPI_ISL_646715 to 646716, EPI_ISL_646718 to 646719, EPI_ISL_646721, EPI_ISL_646724, EPI_ISL_646727, EPI_ISL_646731, EPI_ISL_646734, EPI_ISL_646736 to 646741, EPI_ISL_646743 to 646744, EPI_ISL_646746 to 646747, EPI_ISL_646749, EPI_ISL_646751 to 646753, EPI_ISL_646755, EPI_ISL_646757 to 646758, EPI_ISL_646760 to 646763, EPI_ISL_646767 to 646768, EPI_ISL_646771, EPI_ISL_646773, EPI_ISL_646776, EPI_ISL_646782, EPI_ISL_646789 to 646790, EPI_ISL_646793, EPI_ISL_646795 to 646797, EPI_ISL_646799 to 646808, EPI_ISL_646811 to 646815, EPI_ISL_646817, EPI_ISL_646819 to 646820, EPI_ISL_646822 to 646824, EPI_ISL_646827 to 646836, EPI_ISL_646838 to 646839, EPI_ISL_646841 to 646849, EPI_ISL_646851 to 646852, EPI_ISL_646855 to 646867, EPI_ISL_646871, EPI_ISL_646873, EPI_ISL_646875 to 646876, EPI_ISL_646878, EPI_ISL_646881, EPI_ISL_646883 to 646884, EPI_ISL_646886 to 646890, EPI_ISL_646893 to 646901, EPI_ISL_646903 to 646904, EPI_ISL_646906 to 646911, EPI_ISL_646913 to 646914, EPI_ISL_646916 to 646918, EPI_ISL_646920, EPI_ISL_646922, EPI_ISL_646924 to 646928, EPI_ISL_646930, EPI_ISL_646932 to 646935, EPI_ISL_646938, EPI_ISL_646940 to 646945, EPI_ISL_646947, EPI_ISL_646949 to 646954, EPI_ISL_646957 to 646959, EPI_ISL_646961 to 646963, EPI_ISL_646965 to 646966, EPI_ISL_646968 to 646969, EPI_ISL_646971 to 646973, EPI_ISL_646978 to 646980, EPI_ISL_646982, EPI_ISL_646984 to 646987, EPI_ISL_646990 to 646991, EPI_ISL_646993 to 646995, EPI_ISL_646997 to 647000, EPI_ISL_647002, EPI_ISL_647004 to 647005, EPI_ISL_647007 to 647008, EPI_ISL_647010 to 647012, EPI_ISL_647015, EPI_ISL_647017 to 647022, EPI_ISL_647024 to 647030, EPI_ISL_647033 to 647034, EPI_ISL_647037, EPI_ISL_647039 to 647044, EPI_ISL_647046 to 647049, EPI_ISL_647051 to 647052, EPI_ISL_647054, EPI_ISL_647056 to 647058, EPI_ISL_647060, EPI_ISL_647062 to 647066, EPI_ISL_647068 to 647070, EPI_ISL_647072, EPI_ISL_647074 to 647075, EPI_ISL_647077 to 647080, EPI_ISL_647083 to 647086, EPI_ISL_647088 to 647089, EPI_ISL_647091 to 647095, EPI_ISL_647098 to 647101, EPI_ISL_647104, EPI_ISL_647106 to 647108, EPI_ISL_647111 to 647113, EPI_ISL_647116, EPI_ISL_647118 to 647120, EPI_ISL_647122, EPI_ISL_647468, EPI_ISL_647473 to 647474, EPI_ISL_647477, EPI_ISL_647497, EPI_ISL_647499, EPI_ISL_647506, EPI_ISL_647511, EPI_ISL_647513, EPI_ISL_647526, EPI_ISL_647528, EPI_ISL_647535 to 647536, EPI_ISL_647541, EPI_ISL_647546, EPI_ISL_647558, EPI_ISL_647576, EPI_ISL_647579, EPI_ISL_647584, EPI_ISL_647591, EPI_ISL_647594, EPI_ISL_647603, EPI_ISL_647606, EPI_ISL_647608, EPI_ISL_647615, EPI_ISL_647618, EPI_ISL_647624, EPI_ISL_647630, EPI_ISL_647632, EPI_ISL_647634 to 647635, EPI_ISL_647638, EPI_ISL_647643, EPI_ISL_647650, EPI_ISL_647655, EPI_ISL_647659, EPI_ISL_647667 to 647668, EPI_ISL_647672, EPI_ISL_647677, EPI_ISL_647682 to 647683, EPI_ISL_647693, EPI_ISL_647695, EPI_ISL_647698, EPI_ISL_647700, EPI_ISL_647704 to 647705, EPI_ISL_647708, EPI_ISL_647711, EPI_ISL_647733, EPI_ISL_647741, EPI_ISL_647750, EPI_ISL_647752, EPI_ISL_647762, EPI_ISL_647771, EPI_ISL_647776, EPI_ISL_647786 to 647787, EPI_ISL_647792, EPI_ISL_649193 to 649204, EPI_ISL_649206 to 649224, EPI_ISL_649226 to 649235, EPI_ISL_649237 to 649243, EPI_ISL_649246 to 649254, EPI_ISL_649256 to 649257, EPI_ISL_649259 to 649261, EPI_ISL_649263 to 649271, EPI_ISL_649273 to 649282, EPI_ISL_649284 to 649285, EPI_ISL_649287, EPI_ISL_649289 to 649291, EPI_ISL_649293 to 649302, EPI_ISL_649304 to 649309, EPI_ISL_649311 to 649313, EPI_ISL_649315 to 649319, EPI_ISL_649321 to 649333, EPI_ISL_649335, EPI_ISL_649338 to 649339, EPI_ISL_649341 to 649345, EPI_ISL_649347 to 649352, EPI_ISL_649354 to 649355, EPI_ISL_649357 to 649361, EPI_ISL_649363 to 649370, EPI_ISL_649372 to 649379, EPI_ISL_649381 to 649385, EPI_ISL_649387, EPI_ISL_649389 to 649402, EPI_ISL_649404, EPI_ISL_649406 to 649419, EPI_ISL_649421 to 649423, EPI_ISL_649426 to 649433, EPI_ISL_649435 to 649438, EPI_ISL_649440 to 649450, EPI_ISL_649452 to 649457, EPI_ISL_649459 to 649469, EPI_ISL_649471 to 649478, EPI_ISL_649480, EPI_ISL_649484 to 649485, EPI_ISL_649487 to 649490, EPI_ISL_655273 to 655284, EPI_ISL_655286 to 655290, EPI_ISL_655293, EPI_ISL_655295 to 655302, EPI_ISL_655305 to 655312, EPI_ISL_655314 to 655315, EPI_ISL_655317 to 655326, EPI_ISL_655328 to 655331, EPI_ISL_655333 to 655334, EPI_ISL_655336 to 655337, EPI_ISL_655340 to 655344, EPI_ISL_655347 to 655350, EPI_ISL_655352 to 655355, EPI_ISL_655357, EPI_ISL_655359 to 655365, EPI_ISL_655367 to 655378, EPI_ISL_655380 to 655394, EPI_ISL_655396, EPI_ISL_655399 to 655401, EPI_ISL_655404 to 655405, EPI_ISL_655408 to 655412, EPI_ISL_655416 to 655428, EPI_ISL_655430, EPI_ISL_655432 to 655435, EPI_ISL_655438, EPI_ISL_655441, EPI_ISL_655445 to 655446, EPI_ISL_655448 to 655449, EPI_ISL_655451, EPI_ISL_655454, EPI_ISL_655456 to 655457, EPI_ISL_655459 to 655460, EPI_ISL_655462 to 655479, EPI_ISL_655481 to 655482, EPI_ISL_655484 to 655486, EPI_ISL_655488 to 655494, EPI_ISL_655496 to 655497, EPI_ISL_655499, EPI_ISL_655501 to 655503, EPI_ISL_655505 to 655510, EPI_ISL_655512, EPI_ISL_655514 to 655517, EPI_ISL_655519 to 655520, EPI_ISL_655522, EPI_ISL_655524, EPI_ISL_655526 to 655536, EPI_ISL_655538, EPI_ISL_655540 to 655543, EPI_ISL_655545 to 655549, EPI_ISL_655551 to 655559, EPI_ISL_655561 to 655578, EPI_ISL_655580, EPI_ISL_655583 to 655585, EPI_ISL_655587 to 655595, EPI_ISL_655597 to 655599, EPI_ISL_655601 to 655603, EPI_ISL_655605, EPI_ISL_655607 to 655609, EPI_ISL_655612 to 655617, EPI_ISL_655619 to 655620, EPI_ISL_655622 to 655626, EPI_ISL_655628, EPI_ISL_655630 to 655638, EPI_ISL_655640 to 655642, EPI_ISL_655644 to 655646, EPI_ISL_655649 to 655650, EPI_ISL_655652, EPI_ISL_655654 to 655656, EPI_ISL_655658 to 655659, EPI_ISL_655661, EPI_ISL_655663 to 655668, EPI_ISL_655670 to 655671, EPI_ISL_655673 to 655675, EPI_ISL_655678 to 655687, EPI_ISL_655689 to 655693, EPI_ISL_655698, EPI_ISL_655700 to 655710, EPI_ISL_655712 to 655713, EPI_ISL_655716, EPI_ISL_655718, EPI_ISL_655720 to 655722, EPI_ISL_655724 to 655726, EPI_ISL_655728 to 655731, EPI_ISL_655733 to 655734, EPI_ISL_655736, EPI_ISL_655738, EPI_ISL_655741 to 655743, EPI_ISL_655746 to 655755, EPI_ISL_655757 to 655761, EPI_ISL_655763 to 655764, EPI_ISL_655767, EPI_ISL_655771, EPI_ISL_655774 to 655776, EPI_ISL_655778, EPI_ISL_655781, EPI_ISL_655784 to 655787, EPI_ISL_655789 to 655797, EPI_ISL_655799 to 655803, EPI_ISL_655805, EPI_ISL_655808, EPI_ISL_655811 to 655813, EPI_ISL_655815 to 655816, EPI_ISL_655820 to 655821, EPI_ISL_655823 to 655824, EPI_ISL_655827, EPI_ISL_655829 to 655831, EPI_ISL_655835 to 655840, EPI_ISL_655842, EPI_ISL_655844 to 655845, EPI_ISL_655849, EPI_ISL_655851 to 655853, EPI_ISL_655855, EPI_ISL_655857 to 655859, EPI_ISL_655862 to 655863, EPI_ISL_655869 to 655872, EPI_ISL_655875 to 655878, EPI_ISL_655880, EPI_ISL_655884 to 655885, EPI_ISL_655887 to 655890, EPI_ISL_655892 to 655899, EPI_ISL_655901 to 655905, EPI_ISL_655908 to 655910, EPI_ISL_655914, EPI_ISL_655916, EPI_ISL_655919, EPI_ISL_655921, EPI_ISL_655923, EPI_ISL_655926 to 655928, EPI_ISL_655930 to 655932, EPI_ISL_655935 to 655937, EPI_ISL_655940, EPI_ISL_655942 to 655944, EPI_ISL_655946, EPI_ISL_655949 to 655956, EPI_ISL_655958, EPI_ISL_655960 to 655965, EPI_ISL_655968 to 655970, EPI_ISL_655972, EPI_ISL_655974, EPI_ISL_655976 to 655982, EPI_ISL_655984 to 655991, EPI_ISL_655993 to 655994, EPI_ISL_655996 to 656015, EPI_ISL_656017 to 656019, EPI_ISL_656023 to 656028, EPI_ISL_656030 to 656040, EPI_ISL_656042 to 656043, EPI_ISL_656046 to 656053, EPI_ISL_656055 to 656067, EPI_ISL_656069 to 656072, EPI_ISL_656074 to 656082, EPI_ISL_656084 to 656089, EPI_ISL_656091 to 656095, EPI_ISL_656097 to 656141, EPI_ISL_656143 to 656160, EPI_ISL_656162, EPI_ISL_656165 to 656172, EPI_ISL_656175 to 656176, EPI_ISL_656178 to 656179, EPI_ISL_656181 to 656189, EPI_ISL_656191, EPI_ISL_656193 to 656202, EPI_ISL_656204 to 656218, EPI_ISL_656220, EPI_ISL_656222 to 656229, EPI_ISL_656231 to 656232, EPI_ISL_656234 to 656238, EPI_ISL_656240 to 656242, EPI_ISL_656244 to 656254, EPI_ISL_656256, EPI_ISL_656258 to 656274, EPI_ISL_656276 to 656284, EPI_ISL_656286 to 656288, EPI_ISL_656290 to 656291, EPI_ISL_656293, EPI_ISL_656295 to 656316, EPI_ISL_656318 to 656320, EPI_ISL_656322, EPI_ISL_656324 to 656325, EPI_ISL_656327, EPI_ISL_656329 to 656341, EPI_ISL_656343 to 656348, EPI_ISL_656350 to 656352, EPI_ISL_656354 to 656359, EPI_ISL_656362 to 656372, EPI_ISL_656374 to 656380, EPI_ISL_656382 to 656386, EPI_ISL_656388 to 656389, EPI_ISL_656391 to 656397, EPI_ISL_656399 to 656408, EPI_ISL_656410 to 656414, EPI_ISL_656416 to 656420, EPI_ISL_656422 to 656427, EPI_ISL_656430 to 656437, EPI_ISL_656439 to 656440, EPI_ISL_656442 to 656452, EPI_ISL_656455 to 656456, EPI_ISL_656458, EPI_ISL_656460, EPI_ISL_656462 to 656467, EPI_ISL_656469 to 656470, EPI_ISL_656472 to 656484, EPI_ISL_656486 to 656492, EPI_ISL_656494 to 656495, EPI_ISL_656497, EPI_ISL_656499 to 656503, EPI_ISL_656505 to 656508, EPI_ISL_656510 to 656512, EPI_ISL_656514 to 656533, EPI_ISL_656535, EPI_ISL_656538 to 656543, EPI_ISL_656547, EPI_ISL_656549 to 656552, EPI_ISL_656554 to 656577, EPI_ISL_656579, EPI_ISL_656581, EPI_ISL_656583 to 656587, EPI_ISL_656589 to 656593, EPI_ISL_656595 to 656597, EPI_ISL_656599 to 656610, EPI_ISL_656613 to 656625, EPI_ISL_656628 to 656633, EPI_ISL_656635 to 656642, EPI_ISL_656644 to 656658, EPI_ISL_656660 to 656673, EPI_ISL_656675 to 656676, EPI_ISL_656678 to 656682, EPI_ISL_656684, EPI_ISL_661956, EPI_ISL_661958, EPI_ISL_661961, EPI_ISL_661964 to 661966, EPI_ISL_661968, EPI_ISL_661970, EPI_ISL_661974, EPI_ISL_661977 to 661978, EPI_ISL_661980, EPI_ISL_661982 to 661983, EPI_ISL_661985, EPI_ISL_661988 to 661992, EPI_ISL_661995, EPI_ISL_661998 to 662002, EPI_ISL_662004, EPI_ISL_662006, EPI_ISL_662008, EPI_ISL_662010, EPI_ISL_662013 to 662014, EPI_ISL_662018 to 662019, EPI_ISL_662025, EPI_ISL_662029, EPI_ISL_662031 to 662032, EPI_ISL_662036, EPI_ISL_662038, EPI_ISL_662043, EPI_ISL_662045 to 662049, EPI_ISL_662051, EPI_ISL_662053, EPI_ISL_662055, EPI_ISL_662057 to 662058, EPI_ISL_662060 to 662063, EPI_ISL_662065, EPI_ISL_662067 to 662068, EPI_ISL_662071, EPI_ISL_662074, EPI_ISL_662078 to 662079, EPI_ISL_662081, EPI_ISL_662086, EPI_ISL_662093, EPI_ISL_662098, EPI_ISL_662100 to 662101, EPI_ISL_662105 to 662108, EPI_ISL_662114 to 662116, EPI_ISL_662118, EPI_ISL_662120, EPI_ISL_662122 to 662124, EPI_ISL_662129, EPI_ISL_662131 to 662133, EPI_ISL_662135, EPI_ISL_662137 to 662138, EPI_ISL_662140 to 662141, EPI_ISL_662143, EPI_ISL_662145 to 662146, EPI_ISL_662148, EPI_ISL_662150 to 662151, EPI_ISL_662156, EPI_ISL_662159, EPI_ISL_662162 to 662167, EPI_ISL_662169 to 662170, EPI_ISL_662174 to 662179, EPI_ISL_662181, EPI_ISL_662183 to 662184, EPI_ISL_662186 to 662187, EPI_ISL_662191 to 662192, EPI_ISL_662194, EPI_ISL_662196, EPI_ISL_662198, EPI_ISL_662200, EPI_ISL_662202, EPI_ISL_662206, EPI_ISL_662208 to 662209, EPI_ISL_662212, EPI_ISL_662214 to 662215, EPI_ISL_662217 to 662219, EPI_ISL_662223, EPI_ISL_662227 to 662229, EPI_ISL_662231, EPI_ISL_662233, EPI_ISL_662235, EPI_ISL_662237 to 662240, EPI_ISL_662244 to 662245, EPI_ISL_662247 to 662249, EPI_ISL_662252, EPI_ISL_662254 to 662255, EPI_ISL_662258, EPI_ISL_662260, EPI_ISL_662263, EPI_ISL_662265 to 662267, EPI_ISL_662272 to 662276, EPI_ISL_662285, EPI_ISL_662290, EPI_ISL_662292, EPI_ISL_662296, EPI_ISL_662305, EPI_ISL_662309, EPI_ISL_662320, EPI_ISL_662327, EPI_ISL_662334, EPI_ISL_662342 to 662344, EPI_ISL_662354, EPI_ISL_662359, EPI_ISL_662365, EPI_ISL_662369, EPI_ISL_662374 to 662375, EPI_ISL_662381 to 662382, EPI_ISL_662385, EPI_ISL_662389 to 662391, EPI_ISL_662393, EPI_ISL_662405, EPI_ISL_662411, EPI_ISL_662415, EPI_ISL_662419, EPI_ISL_662429, EPI_ISL_662431, EPI_ISL_662433, EPI_ISL_662446, EPI_ISL_662452, EPI_ISL_662457, EPI_ISL_662468, EPI_ISL_662472, EPI_ISL_662474, EPI_ISL_662477 to 662478, EPI_ISL_662480, EPI_ISL_662486 to 662488, EPI_ISL_662492, EPI_ISL_662496, EPI_ISL_662499, EPI_ISL_662502, EPI_ISL_662507 to 662508, EPI_ISL_662514, EPI_ISL_662520, EPI_ISL_662535, EPI_ISL_662549, EPI_ISL_662553, EPI_ISL_662558, EPI_ISL_662564, EPI_ISL_662576, EPI_ISL_662578 to 662579, EPI_ISL_662583, EPI_ISL_662588, EPI_ISL_662593, EPI_ISL_662597, EPI_ISL_662604 to 662605, EPI_ISL_662609 to 662610, EPI_ISL_662614, EPI_ISL_662617, EPI_ISL_662621 to 662624, EPI_ISL_662628 to 662630, EPI_ISL_662647 to 662650, EPI_ISL_674159 to 674162, EPI_ISL_674164 to 674165, EPI_ISL_674168 to 674170, EPI_ISL_674172, EPI_ISL_674174 to 674177, EPI_ISL_674179, EPI_ISL_674181 to 674182, EPI_ISL_674186, EPI_ISL_674188, EPI_ISL_674190, EPI_ISL_674192 to 674193, EPI_ISL_674195, EPI_ISL_674197, EPI_ISL_674199, EPI_ISL_674201, EPI_ISL_674203 to 674204, EPI_ISL_674206 to 674207, EPI_ISL_674211, EPI_ISL_674217, EPI_ISL_674222 to 674224, EPI_ISL_674226, EPI_ISL_674228 to 674229, EPI_ISL_674231, EPI_ISL_674234, EPI_ISL_674241 to 674242, EPI_ISL_674245, EPI_ISL_674249, EPI_ISL_674254 to 674255, EPI_ISL_674260 to 674261, EPI_ISL_674264, EPI_ISL_674267, EPI_ISL_674272, EPI_ISL_674276 to 674277, EPI_ISL_674281, EPI_ISL_674283 to 674287, EPI_ISL_674292, EPI_ISL_674294 to 674297, EPI_ISL_674300 to 674301, EPI_ISL_674305, EPI_ISL_674308, EPI_ISL_674310 to 674311, EPI_ISL_674313, EPI_ISL_674317 to 674319, EPI_ISL_674322 to 674323, EPI_ISL_674328 to 674329, EPI_ISL_674332, EPI_ISL_674337, EPI_ISL_674339 to 674341, EPI_ISL_674343, EPI_ISL_674345 to 674347, EPI_ISL_674351 to 674352, EPI_ISL_674354, EPI_ISL_674357, EPI_ISL_674360 to 674361, EPI_ISL_674366 to 674370, EPI_ISL_674372, EPI_ISL_674374 to 674376, EPI_ISL_674380, EPI_ISL_674383, EPI_ISL_674385, EPI_ISL_674389 to 674392, EPI_ISL_674396 to 674397, EPI_ISL_674399 to 674400, EPI_ISL_674402, EPI_ISL_674404, EPI_ISL_674406, EPI_ISL_674408 to 674409, EPI_ISL_674411 to 674413, EPI_ISL_674416, EPI_ISL_674418, EPI_ISL_674421, EPI_ISL_674424, EPI_ISL_674428 to 674429, EPI_ISL_674432, EPI_ISL_674434 to 674436, EPI_ISL_674438 to 674439, EPI_ISL_674445 to 674446, EPI_ISL_674451, EPI_ISL_674454 to 674456, EPI_ISL_674459, EPI_ISL_674462, EPI_ISL_674465, EPI_ISL_674467, EPI_ISL_674469 to 674471, EPI_ISL_674474, EPI_ISL_674476 to 674478, EPI_ISL_674484 to 674488, EPI_ISL_674490, EPI_ISL_674493, EPI_ISL_674495, EPI_ISL_675130, EPI_ISL_675133, EPI_ISL_675135 to 675144, EPI_ISL_675146 to 675148, EPI_ISL_675150 to 675152, EPI_ISL_675154 to 675159, EPI_ISL_675161, EPI_ISL_675163 to 675164, EPI_ISL_675166 to 675169, EPI_ISL_675171, EPI_ISL_675174 to 675177, EPI_ISL_675181 to 675182, EPI_ISL_675184, EPI_ISL_675187, EPI_ISL_675189 to 675198, EPI_ISL_675200, EPI_ISL_675202 to 675206, EPI_ISL_675208, EPI_ISL_675211, EPI_ISL_675213 to 675215, EPI_ISL_675218, EPI_ISL_675220 to 675223, EPI_ISL_675225 to 675226, EPI_ISL_675230 to 675233, EPI_ISL_675235, EPI_ISL_675237, EPI_ISL_675239 to 675240, EPI_ISL_675245 to

675246, EPI_ISL_675248, EPI_ISL_675250 to 675259, EPI_ISL_675261 to 675263, EPI_ISL_675265 to 675266, EPI_ISL_675268 to 675274, EPI_ISL_675278 to 675281, EPI_ISL_675283, EPI_ISL_675287 to 675288, EPI_ISL_675291, EPI_ISL_675293, EPI_ISL_675296 to 675300, EPI_ISL_675302 to 675303, EPI_ISL_675310, EPI_ISL_675312, EPI_ISL_675314 to 675316, EPI_ISL_675321 to 675325, EPI_ISL_675327 to 675328, EPI_ISL_675330, EPI_ISL_675334 to 675337, EPI_ISL_675339, EPI_ISL_675341, EPI_ISL_675343 to 675352, EPI_ISL_675354 to 675356, EPI_ISL_675358 to 675363, EPI_ISL_675366 to 675367, EPI_ISL_675373 to 675378, EPI_ISL_675380 to 675383, EPI_ISL_675385, EPI_ISL_675387 to 675388, EPI_ISL_675390, EPI_ISL_675394 to 675395, EPI_ISL_675398, EPI_ISL_675400 to 675403, EPI_ISL_675405, EPI_ISL_675407 to 675408, EPI_ISL_675410 to 675412, EPI_ISL_675414 to 675416, EPI_ISL_675418, EPI_ISL_675425 to 675428, EPI_ISL_675430, EPI_ISL_675433 to 675435, EPI_ISL_675437 to 675438, EPI_ISL_675440 to 675442, EPI_ISL_675445, EPI_ISL_675447 to 675450, EPI_ISL_675452, EPI_ISL_675454 to 675455, EPI_ISL_675457 to 675461, EPI_ISL_675464, EPI_ISL_675466, EPI_ISL_675468 to 675470, EPI_ISL_675473 to 675474, EPI_ISL_675476 to 675479, EPI_ISL_675481 to 675484, EPI_ISL_675487 to 675489, EPI_ISL_675491 to 675494, EPI_ISL_675498, EPI_ISL_675501 to 675502, EPI_ISL_675508 to 675512, EPI_ISL_675514, EPI_ISL_675517, EPI_ISL_675519 to 675524, EPI_ISL_675526 to 675527, EPI_ISL_675529, EPI_ISL_675532, EPI_ISL_675534, EPI_ISL_675536 to 675537, EPI_ISL_675539, EPI_ISL_675541, EPI_ISL_675543 to 675546, EPI_ISL_675548, EPI_ISL_675551 to 675554, EPI_ISL_675557, EPI_ISL_675559, EPI_ISL_675561, EPI_ISL_675563, EPI_ISL_675567 to 675568, EPI_ISL_675570 to 675571, EPI_ISL_675573 to 675575, EPI_ISL_675580 to 675582, EPI_ISL_675584 to 675595, EPI_ISL_675597 to 675600, EPI_ISL_675603 to 675605, EPI_ISL_675607 to 675609, EPI_ISL_675611 to 675619, EPI_ISL_675622 to 675626, EPI_ISL_675628 to 675630, EPI_ISL_675632 to 675633, EPI_ISL_675635 to 675643, EPI_ISL_675645, EPI_ISL_675647, EPI_ISL_675649 to 675651, EPI_ISL_675653 to 675659, EPI_ISL_675662, EPI_ISL_675665 to 675666, EPI_ISL_675668 to 675671, EPI_ISL_675673 to 675674, EPI_ISL_675677, EPI_ISL_675680 to 675681, EPI_ISL_675684 to 675689, EPI_ISL_675691 to 675692, EPI_ISL_675696 to 675697, EPI_ISL_675699, EPI_ISL_675701 to 675703, EPI_ISL_675705 to 675708, EPI_ISL_675710, EPI_ISL_675715, EPI_ISL_675720, EPI_ISL_675735, EPI_ISL_675740, EPI_ISL_675746, EPI_ISL_675750 to 675751, EPI_ISL_675756 to 675757, EPI_ISL_675759 to 675760, EPI_ISL_675766, EPI_ISL_675771, EPI_ISL_675774 to 675777, EPI_ISL_675779, EPI_ISL_675785, EPI_ISL_675788, EPI_ISL_675794, EPI_ISL_675798, EPI_ISL_675800, EPI_ISL_675802 to 675803, EPI_ISL_675806, EPI_ISL_675810, EPI_ISL_675812, EPI_ISL_675817 to 675818, EPI_ISL_675820, EPI_ISL_675823, EPI_ISL_675825 to 675826, EPI_ISL_675829, EPI_ISL_675833, EPI_ISL_675839, EPI_ISL_675847, EPI_ISL_675852, EPI_ISL_675855, EPI_ISL_675868, EPI_ISL_675878, EPI_ISL_675882, EPI_ISL_675892, EPI_ISL_675894, EPI_ISL_675897, EPI_ISL_675901 to 675902, EPI_ISL_675908, EPI_ISL_675913, EPI_ISL_675916, EPI_ISL_675920, EPI_ISL_675923 to 675925, EPI_ISL_675929 to 675930, EPI_ISL_675934, EPI_ISL_675937, EPI_ISL_675939, EPI_ISL_675942, EPI_ISL_675944, EPI_ISL_675952, EPI_ISL_675954, EPI_ISL_675962, EPI_ISL_675965, EPI_ISL_675968, EPI_ISL_675974, EPI_ISL_675976, EPI_ISL_675980, EPI_ISL_675982, EPI_ISL_675986 to 675988, EPI_ISL_675993, EPI_ISL_676000, EPI_ISL_676002, EPI_ISL_676006, EPI_ISL_676010, EPI_ISL_681661, EPI_ISL_681665, EPI_ISL_693332, EPI_ISL_701674, EPI_ISL_701676 to 701682, EPI_ISL_701684 to 701687, EPI_ISL_701689 to 701693, EPI_ISL_701695 to 701696, EPI_ISL_701699, EPI_ISL_701701, EPI_ISL_701703 to 701705, EPI_ISL_701707 to 701716, EPI_ISL_701718 to 701719, EPI_ISL_701721, EPI_ISL_701723, EPI_ISL_701725 to 701728, EPI_ISL_701730 to 701734, EPI_ISL_701736, EPI_ISL_701738 to 701742, EPI_ISL_701745 to 701750, EPI_ISL_701752 to 701754, EPI_ISL_701756, EPI_ISL_701758 to 701764, EPI_ISL_701766, EPI_ISL_701768 to 701771, EPI_ISL_701773 to 701774, EPI_ISL_701777 to 701781, EPI_ISL_701783, EPI_ISL_701785 to 701791, EPI_ISL_701793 to 701800, EPI_ISL_701802, EPI_ISL_701804 to 701811, EPI_ISL_701813 to 701815, EPI_ISL_701817 to 701829, EPI_ISL_701831 to 701838, EPI_ISL_701840 to 701841, EPI_ISL_701844 to 701847, EPI_ISL_701849 to 701850, EPI_ISL_701852 to 701856, EPI_ISL_701859 to 701862, EPI_ISL_701865 to 701867, EPI_ISL_701869, EPI_ISL_701871 to 701875, EPI_ISL_701877 to 701880, EPI_ISL_701882 to 701885, EPI_ISL_701887 to 701911, EPI_ISL_701913 to 701920, EPI_ISL_701922 to 701925, EPI_ISL_701927 to 701928, EPI_ISL_701931, EPI_ISL_701933 to 701936, EPI_ISL_701938 to 701940, EPI_ISL_701942 to 701946, EPI_ISL_701948, EPI_ISL_701950, EPI_ISL_701952 to 701958, EPI_ISL_701960, EPI_ISL_701962, EPI_ISL_701965 to 701968, EPI_ISL_701970 to 701972, EPI_ISL_701974 to 701976, EPI_ISL_701978 to 701979, EPI_ISL_701981 to 701992, EPI_ISL_701994 to 702003, EPI_ISL_702006 to 702011, EPI_ISL_702013, EPI_ISL_702015 to 702022, EPI_ISL_702370 to 702375, EPI_ISL_702377 to 702379, EPI_ISL_702382 to 702383, EPI_ISL_702387 to 702390, EPI_ISL_702393 to 702402, EPI_ISL_702404 to 702408, EPI_ISL_702410, EPI_ISL_702413 to 702415, EPI_ISL_702417 to 702419, EPI_ISL_702422 to 702434, EPI_ISL_702436 to 702437, EPI_ISL_702439 to 702445, EPI_ISL_702448 to 702452, EPI_ISL_702454, EPI_ISL_702456 to 702463, EPI_ISL_702465, EPI_ISL_702467 to 702468, EPI_ISL_702472, EPI_ISL_702475, EPI_ISL_702479 to 702480, EPI_ISL_702482, EPI_ISL_702485 to 702486, EPI_ISL_702490 to 702491, EPI_ISL_702493 to 702494, EPI_ISL_702499, EPI_ISL_702501 to 702503, EPI_ISL_702506, EPI_ISL_702509, EPI_ISL_702511, EPI_ISL_702514, EPI_ISL_702516 to 702517, EPI_ISL_702519, EPI_ISL_702521 to 702522, EPI_ISL_702524, EPI_ISL_702526 to 702527, EPI_ISL_702529 to 702530, EPI_ISL_702532, EPI_ISL_702534 to 702535, EPI_ISL_702537 to 702538, EPI_ISL_702542, EPI_ISL_702546, EPI_ISL_702548, EPI_ISL_702555, EPI_ISL_702557, EPI_ISL_702559, EPI_ISL_702563, EPI_ISL_702565, EPI_ISL_702567 to 702568, EPI_ISL_702570, EPI_ISL_702572 to 702573, EPI_ISL_702575 to 702576, EPI_ISL_702578, EPI_ISL_702580 to 702581, EPI_ISL_702583, EPI_ISL_702588 to 702589, EPI_ISL_702592 to 702593, EPI_ISL_702595 to 702596, EPI_ISL_702598 to 702599, EPI_ISL_702601, EPI_ISL_702603 to 702604, EPI_ISL_702606, EPI_ISL_702608 to 702609, EPI_ISL_702611, EPI_ISL_702613 to 702614, EPI_ISL_702616 to 702617, EPI_ISL_702619, EPI_ISL_702621, EPI_ISL_702624, EPI_ISL_702627, EPI_ISL_702629 to 702630, EPI_ISL_702632, EPI_ISL_702634 to 702635, EPI_ISL_702637, EPI_ISL_702641, EPI_ISL_702643, EPI_ISL_702645 to 702646, EPI_ISL_702650 to 702651, EPI_ISL_702653, EPI_ISL_702657 to 702658, EPI_ISL_702660, EPI_ISL_702662 to 702663, EPI_ISL_702665, EPI_ISL_702671, EPI_ISL_702675 to 702676, EPI_ISL_702678, EPI_ISL_702680 to 702681, EPI_ISL_702683, EPI_ISL_702686, EPI_ISL_702688 to 702689, EPI_ISL_702691, EPI_ISL_702693, EPI_ISL_702696, EPI_ISL_702701 to 702702, EPI_ISL_702704, EPI_ISL_702706, EPI_ISL_702709, EPI_ISL_702711, EPI_ISL_702714 to 702715, EPI_ISL_702717, EPI_ISL_702719 to 702720, EPI_ISL_702722, EPI_ISL_702724 to 702725, EPI_ISL_702727, EPI_ISL_702730, EPI_ISL_702733, EPI_ISL_702735, EPI_ISL_702737, EPI_ISL_702740 to 702741, EPI_ISL_702743, EPI_ISL_702745, EPI_ISL_702748, EPI_ISL_702750, EPI_ISL_702752, EPI_ISL_702755, EPI_ISL_702757, EPI_ISL_702759 to 702760, EPI_ISL_702764 to 702765, EPI_ISL_702767 to 702768, EPI_ISL_702772 to 702773, EPI_ISL_702775, EPI_ISL_702777 to 702778, EPI_ISL_702780 to 702781, EPI_ISL_702783, EPI_ISL_702785 to 702786, EPI_ISL_702788, EPI_ISL_702796 to 702797, EPI_ISL_702799 to 702800, EPI_ISL_702802, EPI_ISL_702807 to 702808, EPI_ISL_702810 to 702811, EPI_ISL_702813 to 702814, EPI_ISL_702818, EPI_ISL_702821 to 702822, EPI_ISL_702899, EPI_ISL_702901, EPI_ISL_702903, EPI_ISL_702906, EPI_ISL_702909, EPI_ISL_702912, EPI_ISL_702914, EPI_ISL_702916, EPI_ISL_702919, EPI_ISL_702921, EPI_ISL_702923 to 702924, EPI_ISL_702929, EPI_ISL_702931, EPI_ISL_702933, EPI_ISL_702936, EPI_ISL_702938, EPI_ISL_702940, EPI_ISL_702942 to 702943, EPI_ISL_702945, EPI_ISL_702948, EPI_ISL_702951, EPI_ISL_702954 to 702955, EPI_ISL_702957 to 702958, EPI_ISL_702960, EPI_ISL_702967 to 702968, EPI_ISL_702970, EPI_ISL_702973, EPI_ISL_702975 to 702976, EPI_ISL_702978, EPI_ISL_702981, EPI_ISL_702983 to 702984, EPI_ISL_702986, EPI_ISL_702988 to 702989, EPI_ISL_702991, EPI_ISL_702993, EPI_ISL_702995, EPI_ISL_702997 to 702998, EPI_ISL_703000 to 703001, EPI_ISL_703003 to 703004, EPI_ISL_703006, EPI_ISL_703009, EPI_ISL_703011 to 703012, EPI_ISL_703016 to 703017, EPI_ISL_703019, EPI_ISL_703022, EPI_ISL_703027 to 703028, EPI_ISL_703030 to 703031, EPI_ISL_703035, EPI_ISL_703037, EPI_ISL_703039, EPI_ISL_703041 to 703042, EPI_ISL_703044, EPI_ISL_703047, EPI_ISL_703049 to 703050, EPI_ISL_703052, EPI_ISL_703058, EPI_ISL_703063, EPI_ISL_703065, EPI_ISL_703068, EPI_ISL_703070, EPI_ISL_703075, EPI_ISL_703078 to 703079, EPI_ISL_703083 to 703084, EPI_ISL_703086, EPI_ISL_703091 to 703092, EPI_ISL_703094 to 703095, EPI_ISL_703097 to 703098, EPI_ISL_703100 to 703101, EPI_ISL_703103, EPI_ISL_703106, EPI_ISL_703108 to 703109, EPI_ISL_703111 to 703112, EPI_ISL_703114, EPI_ISL_703116 to 703117, EPI_ISL_703120, EPI_ISL_703122 to 703123, EPI_ISL_703125 to 703126, EPI_ISL_703128, EPI_ISL_703131, EPI_ISL_703134 to 703135, EPI_ISL_703142 to 703143, EPI_ISL_703145 to 703146, EPI_ISL_703149, EPI_ISL_703153, EPI_ISL_703156 to 703157, EPI_ISL_703159 to 703161, EPI_ISL_703163, EPI_ISL_703166, EPI_ISL_703168, EPI_ISL_703171, EPI_ISL_703173, EPI_ISL_703175, EPI_ISL_703183, EPI_ISL_703185 to 703186, EPI_ISL_703188 to 703189, EPI_ISL_703191, EPI_ISL_703193 to 703194, EPI_ISL_703199, EPI_ISL_703201 to 703202, EPI_ISL_703204 to 703205, EPI_ISL_703207, EPI_ISL_703210, EPI_ISL_703212, EPI_ISL_703215, EPI_ISL_703217, EPI_ISL_703220 to 703221, EPI_ISL_703224, EPI_ISL_703226, EPI_ISL_703231 to 703232, EPI_ISL_703236 to 703237, EPI_ISL_703239, EPI_ISL_703242, EPI_ISL_703247 to 703248, EPI_ISL_703250, EPI_ISL_703252 to 703253, EPI_ISL_703255, EPI_ISL_703257, EPI_ISL_703265 to 703266, EPI_ISL_703268, EPI_ISL_703271, EPI_ISL_703274, EPI_ISL_703277, EPI_ISL_703282, EPI_ISL_703293 to 703294, EPI_ISL_703298, EPI_ISL_703301, EPI_ISL_703306 to 703307, EPI_ISL_703311 to 703312, EPI_ISL_703314, EPI_ISL_703317 to 703318, EPI_ISL_703322, EPI_ISL_703326, EPI_ISL_703328 to 703329, EPI_ISL_703333, EPI_ISL_703336, EPI_ISL_703339, EPI_ISL_703341 to 703342, EPI_ISL_703347, EPI_ISL_703365, EPI_ISL_703368, EPI_ISL_703374, EPI_ISL_703376, EPI_ISL_703386, EPI_ISL_703388, EPI_ISL_703394, EPI_ISL_703396 to 703397, EPI_ISL_703402, EPI_ISL_703404 to 703405, EPI_ISL_703413, EPI_ISL_703416, EPI_ISL_703418, EPI_ISL_703420, EPI_ISL_703425, EPI_ISL_703433, EPI_ISL_703436 to 703437, EPI_ISL_703441, EPI_ISL_703448, EPI_ISL_703450, EPI_ISL_703460 to 703461, EPI_ISL_703464, EPI_ISL_703467, EPI_ISL_703475, EPI_ISL_703477 to 703478, EPI_ISL_703483, EPI_ISL_703488, EPI_ISL_703490, EPI_ISL_703493, EPI_ISL_703498, EPI_ISL_703503 to 703504, EPI_ISL_703507, EPI_ISL_703509, EPI_ISL_703518, EPI_ISL_703520, EPI_ISL_703526, EPI_ISL_703528, EPI_ISL_703531 to 703532, EPI_ISL_703536, EPI_ISL_703539, EPI_ISL_703543, EPI_ISL_703547, EPI_ISL_703549, EPI_ISL_703552, EPI_ISL_703555, EPI_ISL_703558, EPI_ISL_703563, EPI_ISL_703565 to 703566, EPI_ISL_703568, EPI_ISL_703571, EPI_ISL_703573, EPI_ISL_703576, EPI_ISL_703580 to 703581, EPI_ISL_703586, EPI_ISL_703588, EPI_ISL_703591 to 703592, EPI_ISL_703594 to 703595, EPI_ISL_703597, EPI_ISL_703602, EPI_ISL_703605, EPI_ISL_703613, EPI_ISL_703615, EPI_ISL_703618, EPI_ISL_703620, EPI_ISL_703623, EPI_ISL_703628, EPI_ISL_703630, EPI_ISL_703646, EPI_ISL_703648, EPI_ISL_703662, EPI_ISL_703664 to 703665, EPI_ISL_703675, EPI_ISL_703678, EPI_ISL_703681, EPI_ISL_703686, EPI_ISL_703688, EPI_ISL_703691 to 703692, EPI_ISL_703696, EPI_ISL_703699, EPI_ISL_703702, EPI_ISL_703705, EPI_ISL_703707, EPI_ISL_703777 to 703778, EPI_ISL_703780 to 703781, EPI_ISL_703783, EPI_ISL_703788, EPI_ISL_703791, EPI_ISL_703793, EPI_ISL_703795 to 703796, EPI_ISL_703798, EPI_ISL_703803 to 703804, EPI_ISL_703809, EPI_ISL_703812, EPI_ISL_703814, EPI_ISL_703817, EPI_ISL_708845 to 708846, EPI_ISL_708851 to 708854, EPI_ISL_708861, EPI_ISL_708864 to 708868, EPI_ISL_708870, EPI_ISL_708872, EPI_ISL_708876 to 708878, EPI_ISL_708882, EPI_ISL_708886 to 708888, EPI_ISL_708891 to 708892, EPI_ISL_708894 to 708895, EPI_ISL_708899, EPI_ISL_708902 to 708903, EPI_ISL_708907, EPI_ISL_708911, EPI_ISL_708914, EPI_ISL_708917, EPI_ISL_708919, EPI_ISL_708923 to 708924, EPI_ISL_708929, EPI_ISL_708931 to 708932, EPI_ISL_708935 to 708937, EPI_ISL_708940, EPI_ISL_708942 to 708943, EPI_ISL_708946 to 708947, EPI_ISL_708949 to 708950, EPI_ISL_708955 to 708956, EPI_ISL_708959 to 708962, EPI_ISL_708967 to 708968, EPI_ISL_708972 to 708974, EPI_ISL_708976 to 708978, EPI_ISL_708980 to 708982, EPI_ISL_708987 to 708988, EPI_ISL_709296, EPI_ISL_709298 to 709300, EPI_ISL_709302, EPI_ISL_709304 to 709305, EPI_ISL_709308, EPI_ISL_709313, EPI_ISL_709315, EPI_ISL_709317, EPI_ISL_709322, EPI_ISL_709325 to 709326, EPI_ISL_709329, EPI_ISL_709337, EPI_ISL_709339 to 709340, EPI_ISL_709347 to 709352, EPI_ISL_709356 to 709357, EPI_ISL_709361 to 709363, EPI_ISL_709369, EPI_ISL_709371 to 709372, EPI_ISL_709374 to 709375, EPI_ISL_709379, EPI_ISL_709382, EPI_ISL_709386, EPI_ISL_709390, EPI_ISL_709393, EPI_ISL_709397, EPI_ISL_709401, EPI_ISL_709410, EPI_ISL_709414 to 709416, EPI_ISL_709418, EPI_ISL_709421, EPI_ISL_709425, EPI_ISL_709427, EPI_ISL_709430, EPI_ISL_709433, EPI_ISL_709437, EPI_ISL_709444 to 709445, EPI_ISL_709447 to 709448, EPI_ISL_709451 to 709452, EPI_ISL_709460 to 709461, EPI_ISL_709468, EPI_ISL_709470 to 709471, EPI_ISL_709475, EPI_ISL_709477 to 709480, EPI_ISL_709484, EPI_ISL_709488, EPI_ISL_709491 to 709492, EPI_ISL_709501, EPI_ISL_709504, EPI_ISL_709507 to 709508, EPI_ISL_709512, EPI_ISL_709514, EPI_ISL_709516, EPI_ISL_709519, EPI_ISL_709521, EPI_ISL_709523, EPI_ISL_709543 to 709546, EPI_ISL_709550, EPI_ISL_709552, EPI_ISL_709554, EPI_ISL_709559 to 709560, EPI_ISL_709563, EPI_ISL_710614, EPI_ISL_710616 to 710617, EPI_ISL_710620, EPI_ISL_710625, EPI_ISL_710628, EPI_ISL_710631, EPI_ISL_710638, EPI_ISL_710645, EPI_ISL_710647, EPI_ISL_710656, EPI_ISL_710661 to 710663, EPI_ISL_710665, EPI_ISL_710671, EPI_ISL_710676, EPI_ISL_710689, EPI_ISL_710693 to 710694, EPI_ISL_710698 to 710704, EPI_ISL_710706 to 710709, EPI_ISL_710714 to 710726, EPI_ISL_710728 to 710742, EPI_ISL_710744 to 710745, EPI_ISL_710747, EPI_ISL_710749 to 710751, EPI_ISL_710837, EPI_ISL_710865 to 710866, EPI_ISL_710869, EPI_ISL_710873 to 710876, EPI_ISL_710887 to 710901, EPI_ISL_710908 to 710938, EPI_ISL_710940 to 710956, EPI_ISL_710958 to 710961, EPI_ISL_710963 to 710975, EPI_ISL_710977, EPI_ISL_710979 to 710988, EPI_ISL_710990 to 710993, EPI_ISL_711008 to 711010, EPI_ISL_711012 to 711013, EPI_ISL_711015 to 711022, EPI_ISL_711027 to 711034, EPI_ISL_711036 to 711037, EPI_ISL_711039 to 711042, EPI_ISL_711045, EPI_ISL_711047, EPI_ISL_718629, EPI_ISL_718631, EPI_ISL_718637, EPI_ISL_718639 to 718640, EPI_ISL_718643 to 718644, EPI_ISL_718647, EPI_ISL_718650, EPI_ISL_718653, EPI_ISL_718657 to 718658, EPI_ISL_718662 to 718664, EPI_ISL_718666, EPI_ISL_718670, EPI_ISL_718672, EPI_ISL_718674, EPI_ISL_718676, EPI_ISL_718678, EPI_ISL_718686 to 718687, EPI_ISL_718691, EPI_ISL_718700, EPI_ISL_718707, EPI_ISL_718711 to 718716, EPI_ISL_718718 to 718719, EPI_ISL_718721, EPI_ISL_718725, EPI_ISL_718728, EPI_ISL_718735, EPI_ISL_718738 to 718740, EPI_ISL_718744 to 718745, EPI_ISL_718747, EPI_ISL_718750, EPI_ISL_718754, EPI_ISL_718759, EPI_ISL_718761 to 718762, EPI_ISL_718764, EPI_ISL_718766 to 718767, EPI_ISL_718769, EPI_ISL_718772, EPI_ISL_718774, EPI_ISL_718777, EPI_ISL_718779, EPI_ISL_718791, EPI_ISL_718795, EPI_ISL_718801, EPI_ISL_718807 to 718808, EPI_ISL_718819, EPI_ISL_718827 to 718833, EPI_ISL_718837, EPI_ISL_718841, EPI_ISL_718847, EPI_ISL_718849 to 718850, EPI_ISL_718853, EPI_ISL_718858, EPI_ISL_718864, EPI_ISL_718866, EPI_ISL_718871 to 718873, EPI_ISL_718877, EPI_ISL_718887 to 718889, EPI_ISL_718891, EPI_ISL_718902, EPI_ISL_718908, EPI_ISL_718911, EPI_ISL_718914 to 718915, EPI_ISL_718922 to 718923, EPI_ISL_718929, EPI_ISL_718932, EPI_ISL_718935 to 718936, EPI_ISL_718943, EPI_ISL_718945, EPI_ISL_718950, EPI_ISL_718952 to 718953, EPI_ISL_718957, EPI_ISL_718962 to 718964, EPI_ISL_718966, EPI_ISL_718970, EPI_ISL_718977 to 718979, EPI_ISL_720688, EPI_ISL_720694, EPI_ISL_720697, EPI_ISL_720709, EPI_ISL_720711, EPI_ISL_720713, EPI_ISL_720722, EPI_ISL_720741, EPI_ISL_720745, EPI_ISL_720750, EPI_ISL_720756, EPI_ISL_720764, EPI_ISL_720770, EPI_ISL_720773, EPI_ISL_720779 to 720780, EPI_ISL_720796, EPI_ISL_720810, EPI_ISL_720816, EPI_ISL_720823, EPI_ISL_720827, EPI_ISL_720836, EPI_ISL_720842, EPI_ISL_720846, EPI_ISL_720848 to 720849, EPI_ISL_720853, EPI_ISL_720857 to 720858, EPI_ISL_720864, EPI_ISL_720869, EPI_ISL_720880, EPI_ISL_720885 to 720886, EPI_ISL_720894, EPI_ISL_720898, EPI_ISL_720907, EPI_ISL_721185, EPI_ISL_721187, EPI_ISL_721190, EPI_ISL_721192, EPI_ISL_721194 to 721212, EPI_ISL_721214 to 721217, EPI_ISL_721219 to 721227, EPI_ISL_721229 to 721231, EPI_ISL_721233 to 721239, EPI_ISL_721241 to 721249, EPI_ISL_721251 to 721253, EPI_ISL_721255 to 721257, EPI_ISL_721259 to 721264, EPI_ISL_721266 to 721271, EPI_ISL_721273 to 721284, EPI_ISL_721286 to 721303, EPI_ISL_721305 to 721314, EPI_ISL_721316 to 721319, EPI_ISL_721322 to 721324, EPI_ISL_721326 to 721329, EPI_ISL_721331 to 721334, EPI_ISL_721336 to 721340, EPI_ISL_721342 to 721344, EPI_ISL_721346 to 721350, EPI_ISL_721352, EPI_ISL_721355, EPI_ISL_721357 to 721361, EPI_ISL_721364 to 721371, EPI_ISL_721373 to 721385, EPI_ISL_721387 to 721388, EPI_ISL_721391 to 721394, EPI_ISL_721396 to 721398, EPI_ISL_721400 to 721407, EPI_ISL_721409 to 721420, EPI_ISL_721423 to 721431, EPI_ISL_721433 to 721446, EPI_ISL_721448 to 721452, EPI_ISL_721454, EPI_ISL_721458 to 721459, EPI_ISL_721461 to 721463, EPI_ISL_721465 to 721472, EPI_ISL_721474 to 721475, EPI_ISL_721477, EPI_ISL_721479 to 721485, EPI_ISL_721488 to 721510, EPI_ISL_721512 to 721515, EPI_ISL_721517 to 721518, EPI_ISL_721520, EPI_ISL_721525, EPI_ISL_730658, EPI_ISL_730660, EPI_ISL_730663, EPI_ISL_730665, EPI_ISL_730667 to 730668, EPI_ISL_730670 to 730672, EPI_ISL_730679, EPI_ISL_730682 to 730683, EPI_ISL_730687 to 730690, EPI_ISL_730692 to 730693, EPI_ISL_730696, EPI_ISL_730698 to 730701, EPI_ISL_730704, EPI_ISL_730707 to 730708, EPI_ISL_730713 to 730714, EPI_ISL_730717, EPI_ISL_730721 to 730722, EPI_ISL_730724 to 730726, EPI_ISL_730729 to 730732, EPI_ISL_730736, EPI_ISL_730738 to 730739, EPI_ISL_730741, EPI_ISL_730743, EPI_ISL_730747 to 730749, EPI_ISL_730751, EPI_ISL_730754 to 730756, EPI_ISL_730758, EPI_ISL_730760 to 730761, EPI_ISL_730763 to 730764, EPI_ISL_730766 to 730767, EPI_ISL_730770, EPI_ISL_730772 to 730776, EPI_ISL_730779, EPI_ISL_730783, EPI_ISL_730788, EPI_ISL_730790 to 730791, EPI_ISL_730797 to 730798, EPI_ISL_730807, EPI_ISL_730810, EPI_ISL_730812, EPI_ISL_730818 to 730819, EPI_ISL_730822 to 730823, EPI_ISL_730825 to 730826, EPI_ISL_730829 to 730830, EPI_ISL_730834 to 730835, EPI_ISL_730839, EPI_ISL_730843, EPI_ISL_730845, EPI_ISL_730848, EPI_ISL_730851, EPI_ISL_730853, EPI_ISL_730857, EPI_ISL_730859, EPI_ISL_730861, EPI_ISL_730865, EPI_ISL_730868, EPI_ISL_730872 to 730874, EPI_ISL_730878, EPI_ISL_730883 to 730886, EPI_ISL_730890, EPI_ISL_730893, EPI_ISL_730895 to 730896, EPI_ISL_730903 to 730904, EPI_ISL_730906, EPI_ISL_730912, EPI_ISL_730914 to 730917, EPI_ISL_730919 to 730920, EPI_ISL_730923, EPI_ISL_730925, EPI_ISL_730927 to 730928, EPI_ISL_730930 to 730931, EPI_ISL_730933 to 730934, EPI_ISL_730940 to 730954, EPI_ISL_730956 to 730957, EPI_ISL_730961, EPI_ISL_730963 to 730966, EPI_ISL_730968, EPI_ISL_730971 to 730972, EPI_ISL_730974 to 730982, EPI_ISL_730984 to 730985, EPI_ISL_730989 to 730992, EPI_ISL_730994 to 731000, EPI_ISL_731002, EPI_ISL_731004, EPI_ISL_731006 to 731007, EPI_ISL_731009, EPI_ISL_731011, EPI_ISL_731013, EPI_ISL_731018 to 731019, EPI_ISL_731021, EPI_ISL_731023, EPI_ISL_731027 to 731033, EPI_ISL_731036 to 731042, EPI_ISL_731044, EPI_ISL_731046, EPI_ISL_731049, EPI_ISL_731051 to 731054, EPI_ISL_731056 to 731057, EPI_ISL_731060 to 731061, EPI_ISL_731065 to 731067, EPI_ISL_731069, EPI_ISL_731071 to 731078, EPI_ISL_731081 to 731083, EPI_ISL_731085 to 731086, EPI_ISL_731089, EPI_ISL_731092 to 731093, EPI_ISL_731095 to 731098, EPI_ISL_731100, EPI_ISL_731102 to 731104, EPI_ISL_731106, EPI_ISL_731108 to 731110, EPI_ISL_731112 to 731115, EPI_ISL_731118, EPI_ISL_731120 to 731121, EPI_ISL_731123 to 731125, EPI_ISL_731131 to 731133, EPI_ISL_731135, EPI_ISL_731138, EPI_ISL_731140 to 731142, EPI_ISL_731144 to 731145, EPI_ISL_731147, EPI_ISL_731149, EPI_ISL_731152, EPI_ISL_731154 to 731157, EPI_ISL_731159 to 731161, EPI_ISL_731163 to 731166, EPI_ISL_731168, EPI_ISL_731170, EPI_ISL_731172 to 731178, EPI_ISL_731181 to 731185, EPI_ISL_731187 to 731188, EPI_ISL_731190 to 731191, EPI_ISL_731193, EPI_ISL_731195, EPI_ISL_731198, EPI_ISL_731200 to 731205, EPI_ISL_731207 to 731209, EPI_ISL_731211 to 731213, EPI_ISL_731216, EPI_ISL_731219 to 731221, EPI_ISL_731224, EPI_ISL_731229 to 731230, EPI_ISL_731233 to 731234, EPI_ISL_731236 to 731237, EPI_ISL_731240 to 731243, EPI_ISL_731245 to 731246, EPI_ISL_731248 to 731249, EPI_ISL_731254, EPI_ISL_731265, EPI_ISL_731268 to 731269, EPI_ISL_731274, EPI_ISL_731278 to 731279, EPI_ISL_731285, EPI_ISL_731288 to 731289, EPI_ISL_731292, EPI_ISL_731298, EPI_ISL_731302, EPI_ISL_731312 to 731313, EPI_ISL_731318, EPI_ISL_731323, EPI_ISL_731325, EPI_ISL_731329, EPI_ISL_731339, EPI_ISL_731342 to 731344, EPI_ISL_731351 to 731352, EPI_ISL_731358, EPI_ISL_731360, EPI_ISL_731363 to 731365, EPI_ISL_731369, EPI_ISL_731371, EPI_ISL_731375, EPI_ISL_731384 to 731385, EPI_ISL_731388 to 731389, EPI_ISL_731391, EPI_ISL_731399, EPI_ISL_731415 to 731416, EPI_ISL_731418 to 731419, EPI_ISL_731427 to 731428, EPI_ISL_731434, EPI_ISL_731439 to 731442, EPI_ISL_731446, EPI_ISL_731448, EPI_ISL_731451, EPI_ISL_731457, EPI_ISL_731460 to 731462, EPI_ISL_731464 to 731465, EPI_ISL_731470, EPI_ISL_731473, EPI_ISL_731481, EPI_ISL_731483, EPI_ISL_731487, EPI_ISL_731489, EPI_ISL_731495, EPI_ISL_731498, EPI_ISL_731500, EPI_ISL_731502, EPI_ISL_731504, EPI_ISL_731506, EPI_ISL_731511 to 731512, EPI_ISL_731514 to 731516, EPI_ISL_731520, EPI_ISL_731524 to 731528, EPI_ISL_731530, EPI_ISL_731532, EPI_ISL_731534, EPI_ISL_731538 to 731540, EPI_ISL_731545, EPI_ISL_731547, EPI_ISL_731549, EPI_ISL_731552 to 731554, EPI_ISL_731558 to 731559, EPI_ISL_731561 to 731562, EPI_ISL_731564, EPI_ISL_731566, EPI_ISL_731568, EPI_ISL_731571 to 731572, EPI_ISL_731574 to 731577, EPI_ISL_731582 to 731583, EPI_ISL_731587, EPI_ISL_731590, EPI_ISL_731592 to 731593, EPI_ISL_731595, EPI_ISL_731598 to 731602, EPI_ISL_731604 to 731608, EPI_ISL_731610 to 731611, EPI_ISL_731613 to 731614, EPI_ISL_731618 to 731619, EPI_ISL_731622 to 731623, EPI_ISL_731625 to 731626, EPI_ISL_731630 to 731634, EPI_ISL_731636, EPI_ISL_731638 to 731646, EPI_ISL_731649 to 731650, EPI_ISL_731652 to 731654, EPI_ISL_731656, EPI_ISL_731659 to 731663, EPI_ISL_731667, EPI_ISL_731669, EPI_ISL_731671 to 731672, EPI_ISL_731675, EPI_ISL_731678 to 731679, EPI_ISL_731681 to 731684, EPI_ISL_731686 to 731687, EPI_ISL_731691 to 731692, EPI_ISL_731694 to 731702, EPI_ISL_731704 to 731714, EPI_ISL_731717 to 731720, EPI_ISL_731724, EPI_ISL_731726 to 731728, EPI_ISL_731731 to 731736, EPI_ISL_731738, EPI_ISL_731740 to 731745, EPI_ISL_731747 to 731749, EPI_ISL_731751 to 731756, EPI_ISL_731758 to 731763, EPI_ISL_731765, EPI_ISL_731767 to 731768, EPI_ISL_731770 to 731772, EPI_ISL_731774, EPI_ISL_731777, EPI_ISL_731779 to 731782, EPI_ISL_731784, EPI_ISL_731786, EPI_ISL_731788 to 731789, EPI_ISL_731793 to 731794, EPI_ISL_731796, EPI_ISL_731798, EPI_ISL_731801 to 731806, EPI_ISL_731809 to 731818, EPI_ISL_731820 to 731821, EPI_ISL_731823, EPI_ISL_731825 to 731829, EPI_ISL_731832 to 731834, EPI_ISL_731836 to 731841, EPI_ISL_731843, EPI_ISL_731845 to 731846, EPI_ISL_731848 to 731849, EPI_ISL_731853, EPI_ISL_731856, EPI_ISL_731858 to 731860, EPI_ISL_731862 to 731864, EPI_ISL_731867 to 731871, EPI_ISL_731873 to 731876, EPI_ISL_731879 to 731884, EPI_ISL_731886 to 731888, EPI_ISL_731890, EPI_ISL_733598, EPI_ISL_733611, EPI_ISL_733613, EPI_ISL_733618, EPI_ISL_733621, EPI_ISL_733623, EPI_ISL_733635, EPI_ISL_733638, EPI_ISL_733641 to 733643, EPI_ISL_733647, EPI_ISL_733659, EPI_ISL_733682 to 733683, EPI_ISL_733685, EPI_ISL_733693, EPI_ISL_733697, EPI_ISL_733699, EPI_ISL_733701, EPI_ISL_733703, EPI_ISL_733710, EPI_ISL_733712, EPI_ISL_733725, EPI_ISL_733731, EPI_ISL_733734, EPI_ISL_733737, EPI_ISL_733742, EPI_ISL_733754 to 733755, EPI_ISL_733759, EPI_ISL_733766, EPI_ISL_733769, EPI_ISL_733777, EPI_ISL_733783, EPI_ISL_733794 to 733795, EPI_ISL_733797, EPI_ISL_733803, EPI_ISL_733806 to 733807, EPI_ISL_733815, EPI_ISL_733818, EPI_ISL_733833, EPI_ISL_733837, EPI_ISL_733844, EPI_ISL_733848, EPI_ISL_733867, EPI_ISL_733877, EPI_ISL_733880, EPI_ISL_733882, EPI_ISL_733897, EPI_ISL_733901, EPI_ISL_733912 to 733913, EPI_ISL_735862 to 735863, EPI_ISL_735868, EPI_ISL_735871 to 735872, EPI_ISL_735875 to 735877,

EPI_ISL_735881 to 735883, EPI_ISL_735887 to 735891, EPI_ISL_735897, EPI_ISL_735899 to 735902, EPI_ISL_735904 to 735905, EPI_ISL_735907 to 735908, EPI_ISL_735910 to 735911, EPI_ISL_735913 to 735914, EPI_ISL_735916, EPI_ISL_735922 to 735923, EPI_ISL_735925 to 735926, EPI_ISL_735928, EPI_ISL_735930, EPI_ISL_735933, EPI_ISL_735936, EPI_ISL_735941, EPI_ISL_735943 to 735944, EPI_ISL_735947, EPI_ISL_735950 to 735951, EPI_ISL_735954, EPI_ISL_735957 to 735959, EPI_ISL_735962 to 735964, EPI_ISL_735970, EPI_ISL_735974 to 735976, EPI_ISL_735979 to 735981, EPI_ISL_735984 to 735986, EPI_ISL_735988 to 735989, EPI_ISL_735997 to 735998, EPI_ISL_736008, EPI_ISL_736010 to 736014, EPI_ISL_736016, EPI_ISL_736020 to 736021, EPI_ISL_736023, EPI_ISL_736028 to 736029, EPI_ISL_736034, EPI_ISL_736039 to 736040, EPI_ISL_736043 to 736048, EPI_ISL_736051, EPI_ISL_736054, EPI_ISL_736056, EPI_ISL_736058, EPI_ISL_736061, EPI_ISL_736063, EPI_ISL_736065, EPI_ISL_736070 to 736071, EPI_ISL_736073 to 736074, EPI_ISL_736076 to 736077, EPI_ISL_736079 to 736081, EPI_ISL_736083 to 736084, EPI_ISL_736087, EPI_ISL_736089, EPI_ISL_736093, EPI_ISL_736095, EPI_ISL_736097, EPI_ISL_736103, EPI_ISL_736106, EPI_ISL_736108, EPI_ISL_736111, EPI_ISL_736117 to 736119, EPI_ISL_736122 to 736123, EPI_ISL_736125, EPI_ISL_736127 to 736128, EPI_ISL_736134, EPI_ISL_736136, EPI_ISL_736140 to 736142, EPI_ISL_736144, EPI_ISL_736148, EPI_ISL_736150, EPI_ISL_736152 to 736153, EPI_ISL_736158, EPI_ISL_736164 to 736165, EPI_ISL_736173, EPI_ISL_736187, EPI_ISL_736189, EPI_ISL_736192 to 736194, EPI_ISL_736196 to 736199, EPI_ISL_736482, EPI_ISL_736484 to 736485, EPI_ISL_736494, EPI_ISL_736496, EPI_ISL_736498, EPI_ISL_736500, EPI_ISL_736506, EPI_ISL_736510 to 736511, EPI_ISL_736513, EPI_ISL_736517 to 736518, EPI_ISL_736520, EPI_ISL_736522, EPI_ISL_736526, EPI_ISL_736537, EPI_ISL_736539 to 736541, EPI_ISL_736543, EPI_ISL_736545, EPI_ISL_736548, EPI_ISL_736557, EPI_ISL_736559 to 736562, EPI_ISL_736569 to 736570, EPI_ISL_736574 to 736575, EPI_ISL_736577, EPI_ISL_736579, EPI_ISL_736583, EPI_ISL_736585, EPI_ISL_736587, EPI_ISL_736599 to 736600, EPI_ISL_736604, EPI_ISL_736607, EPI_ISL_736612 to 736616, EPI_ISL_736621, EPI_ISL_736626, EPI_ISL_736634, EPI_ISL_736639, EPI_ISL_736641 to 736642, EPI_ISL_736644, EPI_ISL_736647, EPI_ISL_736649, EPI_ISL_736652, EPI_ISL_736654 to 736656, EPI_ISL_736661 to 736662, EPI_ISL_736664 to 736667, EPI_ISL_736670, EPI_ISL_736680, EPI_ISL_736687 to 736688, EPI_ISL_736698, EPI_ISL_736701, EPI_ISL_736704 to 736705, EPI_ISL_736707 to 736708, EPI_ISL_736710, EPI_ISL_736717 to 736718, EPI_ISL_736720, EPI_ISL_736726, EPI_ISL_736729, EPI_ISL_736741, EPI_ISL_736748, EPI_ISL_736751, EPI_ISL_736755 to 736756, EPI_ISL_736758, EPI_ISL_736760, EPI_ISL_736762, EPI_ISL_736765 to 736766, EPI_ISL_736768 to 736772, EPI_ISL_756378, EPI_ISL_756381 to 756383, EPI_ISL_756385, EPI_ISL_756390 to 756391, EPI_ISL_756394, EPI_ISL_756396 to 756400, EPI_ISL_756402, EPI_ISL_756404 to 756408, EPI_ISL_756411 to 756412, EPI_ISL_756414, EPI_ISL_756421, EPI_ISL_756423, EPI_ISL_756425, EPI_ISL_756433, EPI_ISL_756435 to 756437, EPI_ISL_756439 to 756440, EPI_ISL_756443, EPI_ISL_756445, EPI_ISL_756447, EPI_ISL_756449 to 756452, EPI_ISL_756455, EPI_ISL_756457 to 756459, EPI_ISL_756461 to 756466, EPI_ISL_756468, EPI_ISL_756470 to 756473, EPI_ISL_756476 to 756477, EPI_ISL_756479, EPI_ISL_756482 to 756483, EPI_ISL_756489, EPI_ISL_756493 to 756495, EPI_ISL_756503, EPI_ISL_756510, EPI_ISL_756514, EPI_ISL_756516, EPI_ISL_756520, EPI_ISL_756524, EPI_ISL_756527, EPI_ISL_756530 to 756533, EPI_ISL_756536 to 756539, EPI_ISL_756541, EPI_ISL_756544, EPI_ISL_756546 to 756547, EPI_ISL_756550 to 756552, EPI_ISL_756555 to 756557, EPI_ISL_756560 to 756563, EPI_ISL_756565 to 756566, EPI_ISL_756569, EPI_ISL_756571, EPI_ISL_756578, EPI_ISL_756580, EPI_ISL_756583, EPI_ISL_756587, EPI_ISL_756592, EPI_ISL_756596, EPI_ISL_756598 to 756600, EPI_ISL_756602 to 756603, EPI_ISL_756609, EPI_ISL_756612, EPI_ISL_756616, EPI_ISL_756618 to 756620, EPI_ISL_756624, EPI_ISL_756626, EPI_ISL_756632, EPI_ISL_756634 to 756636, EPI_ISL_756638 to 756641, EPI_ISL_756644 to 756645, EPI_ISL_756647 to 756648, EPI_ISL_756650 to 756652, EPI_ISL_756654, EPI_ISL_756656 to 756658, EPI_ISL_756661 to 756664, EPI_ISL_756669 to 756670, EPI_ISL_756673, EPI_ISL_756681 to 756686, EPI_ISL_760249, EPI_ISL_760259, EPI_ISL_760261, EPI_ISL_760263, EPI_ISL_760266 to 760268, EPI_ISL_760273, EPI_ISL_760276, EPI_ISL_760278, EPI_ISL_760281, EPI_ISL_760284 to 760286, EPI_ISL_760288, EPI_ISL_760293, EPI_ISL_760296, EPI_ISL_760301, EPI_ISL_760305 to 760306, EPI_ISL_760311, EPI_ISL_760326, EPI_ISL_760333, EPI_ISL_760341, EPI_ISL_760344, EPI_ISL_760346, EPI_ISL_760358 to 760360, EPI_ISL_760364, EPI_ISL_760370 to 760372, EPI_ISL_760381, EPI_ISL_760386 to 760387, EPI_ISL_760392, EPI_ISL_760397, EPI_ISL_760405, EPI_ISL_760408, EPI_ISL_760415, EPI_ISL_760418 to 760419, EPI_ISL_760421, EPI_ISL_760430, EPI_ISL_760432, EPI_ISL_760434, EPI_ISL_760436, EPI_ISL_760441 to 760442, EPI_ISL_760445, EPI_ISL_760448, EPI_ISL_760450, EPI_ISL_760460, EPI_ISL_760471, EPI_ISL_760474 to 760475, EPI_ISL_760485 to 760486, EPI_ISL_760491, EPI_ISL_760495, EPI_ISL_760498, EPI_ISL_760500 to 760501, EPI_ISL_760509, EPI_ISL_760516, EPI_ISL_760521, EPI_ISL_760524, EPI_ISL_760530 to 760531, EPI_ISL_760534 to 760535, EPI_ISL_760538, EPI_ISL_760543 to 760546, EPI_ISL_760549, EPI_ISL_760551 to 760552, EPI_ISL_760555, EPI_ISL_760557 to 760559, EPI_ISL_760561 to 760562, EPI_ISL_760564 to 760565, EPI_ISL_760567, EPI_ISL_760570, EPI_ISL_760572, EPI_ISL_760574, EPI_ISL_760582, EPI_ISL_760586 to 760589, EPI_ISL_760591, EPI_ISL_760593, EPI_ISL_760596 to 760597, EPI_ISL_760599 to 760601, EPI_ISL_760608 to 760609, EPI_ISL_760612 to 760613, EPI_ISL_760615, EPI_ISL_760617, EPI_ISL_760623 to 760625, EPI_ISL_760627 to 760628, EPI_ISL_760633, EPI_ISL_760637, EPI_ISL_760640, EPI_ISL_760644, EPI_ISL_760647, EPI_ISL_760649 to 760650, EPI_ISL_760652, EPI_ISL_760654, EPI_ISL_760656, EPI_ISL_760660 to 760661, EPI_ISL_760663 to 760667, EPI_ISL_760670, EPI_ISL_760677, EPI_ISL_760679 to 760680, EPI_ISL_760684, EPI_ISL_760688, EPI_ISL_760691, EPI_ISL_760693, EPI_ISL_760697, EPI_ISL_760699, EPI_ISL_760701 to 760702, EPI_ISL_760705, EPI_ISL_760708, EPI_ISL_760710 to 760711, EPI_ISL_760715, EPI_ISL_760721 to 760724, EPI_ISL_760726 to 760728, EPI_ISL_760733 to 760734, EPI_ISL_760736 to 760738, EPI_ISL_760745, EPI_ISL_760748, EPI_ISL_760752 to 760753, EPI_ISL_760755 to 760756, EPI_ISL_760759, EPI_ISL_760761 to 760763, EPI_ISL_760769, EPI_ISL_760776, EPI_ISL_760780, EPI_ISL_760783 to 760786, EPI_ISL_760788, EPI_ISL_760790, EPI_ISL_760792, EPI_ISL_760794 to 760795, EPI_ISL_760799, EPI_ISL_760802, EPI_ISL_760804, EPI_ISL_760806, EPI_ISL_760808, EPI_ISL_760812, EPI_ISL_760817, EPI_ISL_760825 to 760826, EPI_ISL_760832, EPI_ISL_760838 to 760839, EPI_ISL_760853, EPI_ISL_760857, EPI_ISL_760861 to 760862, EPI_ISL_760864 to 760865, EPI_ISL_760867 to 760868, EPI_ISL_760884, EPI_ISL_760887, EPI_ISL_760889 to 760892, EPI_ISL_760897, EPI_ISL_760900, EPI_ISL_760906, EPI_ISL_760908, EPI_ISL_760912, EPI_ISL_760915 to 760916, EPI_ISL_760918, EPI_ISL_760921, EPI_ISL_760925, EPI_ISL_760928 to 760929, EPI_ISL_760931, EPI_ISL_760933, EPI_ISL_760937 to 760938, EPI_ISL_760941 to 760942, EPI_ISL_760945, EPI_ISL_760948 to 760950, EPI_ISL_760952, EPI_ISL_760960, EPI_ISL_760962, EPI_ISL_760967 to 760968, EPI_ISL_760971, EPI_ISL_760978 to 760979, EPI_ISL_760982, EPI_ISL_760989, EPI_ISL_760994, EPI_ISL_761000 to 761001, EPI_ISL_761011, EPI_ISL_761013, EPI_ISL_761022, EPI_ISL_761025, EPI_ISL_761027, EPI_ISL_761029, EPI_ISL_761040, EPI_ISL_761042, EPI_ISL_761046, EPI_ISL_761049, EPI_ISL_761054, EPI_ISL_761056, EPI_ISL_761060 to 761061, EPI_ISL_761064 to 761066, EPI_ISL_761080 to 761088, EPI_ISL_761090 to 761093, EPI_ISL_761096 to 761112, EPI_ISL_761115, EPI_ISL_761117, EPI_ISL_761119, EPI_ISL_761124, EPI_ISL_761126 to 761127, EPI_ISL_761129 to 761130, EPI_ISL_761136 to 761138, EPI_ISL_761140 to 761142, EPI_ISL_761144, EPI_ISL_761146 to 761153, EPI_ISL_761155 to 761156, EPI_ISL_761159 to 761161, EPI_ISL_761163 to 761167, EPI_ISL_761169 to 761171, EPI_ISL_761173 to 761175, EPI_ISL_761177 to 761186, EPI_ISL_761188 to 761190, EPI_ISL_761192, EPI_ISL_761195 to 761196, EPI_ISL_761198 to 761202, EPI_ISL_761205 to 761206, EPI_ISL_761208, EPI_ISL_761210 to 761213, EPI_ISL_761215 to 761217, EPI_ISL_761219 to 761220, EPI_ISL_761222 to 761224, EPI_ISL_761230 to 761238, EPI_ISL_761240, EPI_ISL_761242 to 761249, EPI_ISL_761251, EPI_ISL_761253 to 761254, EPI_ISL_761256, EPI_ISL_761260 to 761265, EPI_ISL_761267, EPI_ISL_761269 to 761275, EPI_ISL_761277, EPI_ISL_761279, EPI_ISL_761281 to 761282, EPI_ISL_761284 to 761286, EPI_ISL_761288, EPI_ISL_761290 to 761291, EPI_ISL_761294 to 761295, EPI_ISL_761297, EPI_ISL_761299 to 761301, EPI_ISL_761304 to 761306, EPI_ISL_761308 to 761312, EPI_ISL_761315 to 761325, EPI_ISL_761328 to 761330, EPI_ISL_761332, EPI_ISL_761334 to 761337, EPI_ISL_761340 to 761347, EPI_ISL_761349 to 761350, EPI_ISL_761352, EPI_ISL_761354, EPI_ISL_761356, EPI_ISL_761665 to 761678, EPI_ISL_761680 to 761695, EPI_ISL_761697 to 761705, EPI_ISL_761707 to 761713, EPI_ISL_761715 to 761720, EPI_ISL_761722 to 761732, EPI_ISL_761736 to 761738, EPI_ISL_761740, EPI_ISL_761742 to 761747, EPI_ISL_761750 to 761754, EPI_ISL_761756 to 761760, EPI_ISL_761762 to 761764, EPI_ISL_761766, EPI_ISL_761768 to 761770, EPI_ISL_761772, EPI_ISL_761774 to 761784, EPI_ISL_761786 to 761798, EPI_ISL_761800 to 761803, EPI_ISL_761805 to 761815, EPI_ISL_761817 to 761818, EPI_ISL_761820 to 761846, EPI_ISL_761848 to 761862, EPI_ISL_761864, EPI_ISL_761867 to 761881, EPI_ISL_761884 to 761904, EPI_ISL_761906 to 761912, EPI_ISL_761914 to 761917, EPI_ISL_761919, EPI_ISL_762547, EPI_ISL_762554, EPI_ISL_762557 to 762558, EPI_ISL_762562, EPI_ISL_762564, EPI_ISL_762581, EPI_ISL_762588, EPI_ISL_762599, EPI_ISL_762605 to 762606, EPI_ISL_762610, EPI_ISL_762617, EPI_ISL_762620, EPI_ISL_762625, EPI_ISL_762638, EPI_ISL_762642 to 762643, EPI_ISL_762647, EPI_ISL_762655, EPI_ISL_762659, EPI_ISL_762666, EPI_ISL_762671 to 762672, EPI_ISL_762683, EPI_ISL_762686, EPI_ISL_762691, EPI_ISL_762693, EPI_ISL_762702 to 762703, EPI_ISL_762706, EPI_ISL_762709 to 762710, EPI_ISL_762712, EPI_ISL_762714, EPI_ISL_762720, EPI_ISL_762722, EPI_ISL_762724, EPI_ISL_762730, EPI_ISL_762733, EPI_ISL_762735, EPI_ISL_762750, EPI_ISL_762754, EPI_ISL_762756, EPI_ISL_762763 to 762764, EPI_ISL_762771, EPI_ISL_762777, EPI_ISL_762786, EPI_ISL_762789, EPI_ISL_762791, EPI_ISL_762793, EPI_ISL_762796, EPI_ISL_762798 to 762799, EPI_ISL_762812, EPI_ISL_762815, EPI_ISL_762819, EPI_ISL_762821, EPI_ISL_762823, EPI_ISL_762830, EPI_ISL_762832, EPI_ISL_762839, EPI_ISL_762841, EPI_ISL_762843 to 762845, EPI_ISL_762847, EPI_ISL_762849, EPI_ISL_762869, EPI_ISL_762878 to 762879, EPI_ISL_762883, EPI_ISL_762886, EPI_ISL_762890, EPI_ISL_762894, EPI_ISL_762901 to 762902, EPI_ISL_762906, EPI_ISL_762910 to 762911, EPI_ISL_762913, EPI_ISL_762919, EPI_ISL_762921 to 762922, EPI_ISL_762931, EPI_ISL_762935, EPI_ISL_762939, EPI_ISL_762947, EPI_ISL_762949, EPI_ISL_762951 to 762952, EPI_ISL_762955, EPI_ISL_762957, EPI_ISL_762964, EPI_ISL_762973 to 762975, EPI_ISL_762977, EPI_ISL_762988 to 762989, EPI_ISL_767091, EPI_ISL_767093 to 767095, EPI_ISL_767097, EPI_ISL_767102 to 767106, EPI_ISL_767116, EPI_ISL_767120 to 767124, EPI_ISL_767127, EPI_ISL_767129 to 767133, EPI_ISL_767135 to 767136, EPI_ISL_767140 to 767142, EPI_ISL_767144, EPI_ISL_767146 to 767154, EPI_ISL_767156 to 767201, EPI_ISL_767203 to 767210, EPI_ISL_767212 to 767229, EPI_ISL_767231 to 767236, EPI_ISL_767240 to 767253, EPI_ISL_767255 to 767259, EPI_ISL_767261 to 767265, EPI_ISL_767268 to 767270, EPI_ISL_767272 to 767279, EPI_ISL_767281 to 767282, EPI_ISL_767284 to 767286, EPI_ISL_767288 to 767298, EPI_ISL_767301 to 767303, EPI_ISL_767305 to 767309, EPI_ISL_767311 to 767330, EPI_ISL_767332 to 767333, EPI_ISL_768842, EPI_ISL_768844, EPI_ISL_768899 to 768900, EPI_ISL_777011, EPI_ISL_777014 to 777015, EPI_ISL_777017, EPI_ISL_777025 to 777026, EPI_ISL_777028, EPI_ISL_777030, EPI_ISL_777035, EPI_ISL_777045, EPI_ISL_777050, EPI_ISL_777057, EPI_ISL_777069 to 777070, EPI_ISL_777074 to 777076, EPI_ISL_777087 to 777088, EPI_ISL_777092 to 777094, EPI_ISL_777096, EPI_ISL_777103, EPI_ISL_777106, EPI_ISL_777109, EPI_ISL_777111, EPI_ISL_777114, EPI_ISL_777116, EPI_ISL_777123, EPI_ISL_777129, EPI_ISL_777134, EPI_ISL_777136 to 777139, EPI_ISL_777143, EPI_ISL_777147, EPI_ISL_777150, EPI_ISL_777156, EPI_ISL_777159, EPI_ISL_777172, EPI_ISL_777174 to 777175, EPI_ISL_777178 to 777179, EPI_ISL_777186, EPI_ISL_777188, EPI_ISL_777194 to 777195, EPI_ISL_777197 to 777198, EPI_ISL_777202, EPI_ISL_777204, EPI_ISL_777210, EPI_ISL_777214, EPI_ISL_777216, EPI_ISL_777220 to 777221, EPI_ISL_777231, EPI_ISL_777234, EPI_ISL_777237, EPI_ISL_777239, EPI_ISL_777247 to 777249, EPI_ISL_777254, EPI_ISL_777256 to 777257, EPI_ISL_777259 to 777260, EPI_ISL_777263 to 777265, EPI_ISL_777271 to 777272, EPI_ISL_777274, EPI_ISL_777277, EPI_ISL_777280 to 777281, EPI_ISL_777287 to 777288, EPI_ISL_777292, EPI_ISL_777294, EPI_ISL_777307, EPI_ISL_777309, EPI_ISL_777311 to 777312, EPI_ISL_777315, EPI_ISL_777318, EPI_ISL_777327, EPI_ISL_777336, EPI_ISL_777338, EPI_ISL_777349, EPI_ISL_777386, EPI_ISL_777389, EPI_ISL_777411 to 777412, EPI_ISL_777416, EPI_ISL_777425, EPI_ISL_777435, EPI_ISL_777437, EPI_ISL_777440, EPI_ISL_777451, EPI_ISL_777465, EPI_ISL_777467, EPI_ISL_777477, EPI_ISL_777479, EPI_ISL_777481, EPI_ISL_777489, EPI_ISL_777491, EPI_ISL_777495, EPI_ISL_777524, EPI_ISL_777529 to 777530, EPI_ISL_777534 to 777535, EPI_ISL_777538, EPI_ISL_777548, EPI_ISL_777552, EPI_ISL_777558, EPI_ISL_777563, EPI_ISL_777567 to 777568, EPI_ISL_777572, EPI_ISL_777580, EPI_ISL_777582, EPI_ISL_777589, EPI_ISL_777591 to 777592, EPI_ISL_777595, EPI_ISL_777597, EPI_ISL_777605, EPI_ISL_777621, EPI_ISL_777629, EPI_ISL_777633, EPI_ISL_777638, EPI_ISL_777644, EPI_ISL_777649, EPI_ISL_777651, EPI_ISL_777655, EPI_ISL_777659, EPI_ISL_777669, EPI_ISL_778016, EPI_ISL_778021, EPI_ISL_778029, EPI_ISL_778040, EPI_ISL_778050 to 778052, EPI_ISL_778055, EPI_ISL_778064, EPI_ISL_778069 to 778070, EPI_ISL_778101, EPI_ISL_778108, EPI_ISL_778113, EPI_ISL_778116, EPI_ISL_778121, EPI_ISL_778128, EPI_ISL_778163, EPI_ISL_778165, EPI_ISL_778187, EPI_ISL_778191, EPI_ISL_778196, EPI_ISL_778201, EPI_ISL_778228, EPI_ISL_778230, EPI_ISL_778233, EPI_ISL_778238, EPI_ISL_778240, EPI_ISL_778246, EPI_ISL_778249, EPI_ISL_778259, EPI_ISL_778264, EPI_ISL_778266, EPI_ISL_778282, EPI_ISL_778288, EPI_ISL_778292, EPI_ISL_778295, EPI_ISL_778305, EPI_ISL_778311, EPI_ISL_778327, EPI_ISL_778331, EPI_ISL_778335, EPI_ISL_778346, EPI_ISL_778353, EPI_ISL_778355, EPI_ISL_778358, EPI_ISL_778360, EPI_ISL_778365, EPI_ISL_778368, EPI_ISL_778374, EPI_ISL_778382, EPI_ISL_778385, EPI_ISL_778401, EPI_ISL_778408, EPI_ISL_778415, EPI_ISL_778417, EPI_ISL_778426, EPI_ISL_778444, EPI_ISL_778448, EPI_ISL_778472 to 778473, EPI_ISL_778484, EPI_ISL_778487, EPI_ISL_778490, EPI_ISL_778494, EPI_ISL_778500, EPI_ISL_778512, EPI_ISL_778529, EPI_ISL_778531, EPI_ISL_778539, EPI_ISL_778547, EPI_ISL_778558, EPI_ISL_778562, EPI_ISL_778564, EPI_ISL_778571, EPI_ISL_778601, EPI_ISL_778605, EPI_ISL_778607, EPI_ISL_778616, EPI_ISL_778618, EPI_ISL_778621, EPI_ISL_778623, EPI_ISL_778628, EPI_ISL_781206, EPI_ISL_781234, EPI_ISL_781280, EPI_ISL_781291, EPI_ISL_781301, EPI_ISL_781337, EPI_ISL_781366, EPI_ISL_781374, EPI_ISL_781405 to 781406, EPI_ISL_781435, EPI_ISL_781478, EPI_ISL_781872, EPI_ISL_781874, EPI_ISL_781879, EPI_ISL_781884, EPI_ISL_781889, EPI_ISL_781894, EPI_ISL_781900 to 781902, EPI_ISL_781904 to 781906, EPI_ISL_781911 to 781912, EPI_ISL_781917, EPI_ISL_781923, EPI_ISL_781932, EPI_ISL_781934, EPI_ISL_781936 to 781937, EPI_ISL_781939, EPI_ISL_781949, EPI_ISL_781951, EPI_ISL_781958 to 781959, EPI_ISL_781961 to 781964, EPI_ISL_781966, EPI_ISL_781974, EPI_ISL_781983, EPI_ISL_781985, EPI_ISL_781996, EPI_ISL_782007, EPI_ISL_782012, EPI_ISL_782016, EPI_ISL_782019, EPI_ISL_782022, EPI_ISL_782025, EPI_ISL_782033 to 782034, EPI_ISL_782041, EPI_ISL_782043, EPI_ISL_782045, EPI_ISL_782048, EPI_ISL_782057, EPI_ISL_782060, EPI_ISL_782071 to 782072, EPI_ISL_782078, EPI_ISL_782086, EPI_ISL_782089 to 782091, EPI_ISL_782095 to 782097, EPI_ISL_782100, EPI_ISL_782104, EPI_ISL_782108 to 782109, EPI_ISL_782117, EPI_ISL_782120, EPI_ISL_782123 to 782124, EPI_ISL_782135, EPI_ISL_782140 to 782142, EPI_ISL_782148, EPI_ISL_782224, EPI_ISL_782245, EPI_ISL_782249, EPI_ISL_782282, EPI_ISL_782313, EPI_ISL_782315, EPI_ISL_782345, EPI_ISL_782388, EPI_ISL_782412, EPI_ISL_782420, EPI_ISL_782423, EPI_ISL_782425, EPI_ISL_782451, EPI_ISL_782454, EPI_ISL_782461, EPI_ISL_782748, EPI_ISL_782771, EPI_ISL_782792, EPI_ISL_782806, EPI_ISL_782843, EPI_ISL_782849, EPI_ISL_782855, EPI_ISL_782897, EPI_ISL_782899, EPI_ISL_782904 to 782905, EPI_ISL_782924, EPI_ISL_782926, EPI_ISL_782936, EPI_ISL_782946, EPI_ISL_782970, EPI_ISL_782997, EPI_ISL_783014 to 783015, EPI_ISL_783045, EPI_ISL_783056, EPI_ISL_783068, EPI_ISL_796784 to 796788, EPI_ISL_796790 to 796794, EPI_ISL_796797 to 796804, EPI_ISL_796808, EPI_ISL_796814, EPI_ISL_796816 to 796817, EPI_ISL_796819, EPI_ISL_796821 to 796822, EPI_ISL_796824, EPI_ISL_796826 to 796829, EPI_ISL_796831 to 796833, EPI_ISL_796836 to 796837, EPI_ISL_796839 to 796840, EPI_ISL_796844 to 796845, EPI_ISL_796847 to 796848, EPI_ISL_796852, EPI_ISL_796856, EPI_ISL_796859, EPI_ISL_796867, EPI_ISL_796869, EPI_ISL_796871 to 796873, EPI_ISL_796875 to 796876, EPI_ISL_796878 to 796879, EPI_ISL_796882, EPI_ISL_796884, EPI_ISL_796886, EPI_ISL_796888, EPI_ISL_796890 to 796891, EPI_ISL_796895 to 796896, EPI_ISL_796898, EPI_ISL_796900 to 796905, EPI_ISL_796907 to 796908, EPI_ISL_796910 to 796911, EPI_ISL_796914, EPI_ISL_796916 to 796919, EPI_ISL_796924, EPI_ISL_796926 to 796928, EPI_ISL_796931 to 796934, EPI_ISL_796936 to 796937, EPI_ISL_796941, EPI_ISL_796944 to 796948, EPI_ISL_796950, EPI_ISL_796953 to 796955, EPI_ISL_796957, EPI_ISL_796959, EPI_ISL_796962 to 796964, EPI_ISL_796967, EPI_ISL_796969, EPI_ISL_796973, EPI_ISL_796976 to 796978, EPI_ISL_796980, EPI_ISL_796982, EPI_ISL_796984, EPI_ISL_796986, EPI_ISL_796989, EPI_ISL_796994, EPI_ISL_796996 to 797000, EPI_ISL_797003 to 797004, EPI_ISL_797007, EPI_ISL_797010 to 797014, EPI_ISL_797018, EPI_ISL_797023 to 797024, EPI_ISL_797026, EPI_ISL_797028 to 797029, EPI_ISL_797031 to 797032, EPI_ISL_797037, EPI_ISL_797040, EPI_ISL_797043, EPI_ISL_797045 to 797047, EPI_ISL_797049 to 797051, EPI_ISL_797054, EPI_ISL_797057, EPI_ISL_797061, EPI_ISL_797065 to 797066, EPI_ISL_797069 to 797072, EPI_ISL_797074 to 797075, EPI_ISL_797080 to 797082, EPI_ISL_797085 to 797086, EPI_ISL_797088, EPI_ISL_797091, EPI_ISL_797093 to 797094, EPI_ISL_797096 to 797100, EPI_ISL_797103 to 797105, EPI_ISL_797107 to 797108, EPI_ISL_797110 to 797111, EPI_ISL_797114 to 797116, EPI_ISL_797399, EPI_ISL_797402, EPI_ISL_797409, EPI_ISL_797413, EPI_ISL_797420 to 797421, EPI_ISL_797424, EPI_ISL_797430, EPI_ISL_797435, EPI_ISL_797438, EPI_ISL_797461 to 797462, EPI_ISL_797468, EPI_ISL_797480, EPI_ISL_797489, EPI_ISL_797491, EPI_ISL_797499, EPI_ISL_797504, EPI_ISL_797509, EPI_ISL_797512, EPI_ISL_797533, EPI_ISL_797535, EPI_ISL_797540, EPI_ISL_797543, EPI_ISL_797549, EPI_ISL_797553, EPI_ISL_797559 to 797560, EPI_ISL_797568, EPI_ISL_797576, EPI_ISL_797587, EPI_ISL_797599, EPI_ISL_797605 to 797606, EPI_ISL_797610, EPI_ISL_797613, EPI_ISL_797637, EPI_ISL_797641 to 797642, EPI_ISL_797644, EPI_ISL_797654, EPI_ISL_797660, EPI_ISL_797663, EPI_ISL_797666, EPI_ISL_797686, EPI_ISL_797699, EPI_ISL_797712, EPI_ISL_797715, EPI_ISL_797717, EPI_ISL_798013, EPI_ISL_798015, EPI_ISL_798027, EPI_ISL_798061 to 798062, EPI_ISL_798068, EPI_ISL_798084, EPI_ISL_798103, EPI_ISL_798122, EPI_ISL_798131, EPI_ISL_798133, EPI_ISL_798139, EPI_ISL_798154, EPI_ISL_798170, EPI_ISL_798175, EPI_ISL_798204, EPI_ISL_798221 to 798222, EPI_ISL_798236, EPI_ISL_798241 to 798242, EPI_ISL_798245, EPI_ISL_798248, EPI_ISL_798250, EPI_ISL_798255, EPI_ISL_798268, EPI_ISL_798274, EPI_ISL_798277, EPI_ISL_798280, EPI_ISL_798284 to 798285, EPI_ISL_798299, EPI_ISL_798321, EPI_ISL_798324, EPI_ISL_798328, EPI_ISL_798334, EPI_ISL_798336, EPI_ISL_798341, EPI_ISL_798370, EPI_ISL_798386, EPI_ISL_798413, EPI_ISL_798416, EPI_ISL_798475, EPI_ISL_798493, EPI_ISL_798500, EPI_ISL_798502, EPI_ISL_798544, EPI_ISL_798550, EPI_ISL_798555, EPI_ISL_798591, EPI_ISL_798593, EPI_ISL_798598, EPI_ISL_798615, EPI_ISL_798622, EPI_ISL_798633, EPI_ISL_798639, EPI_ISL_798643, EPI_ISL_798652, EPI_ISL_798659, EPI_ISL_798668, EPI_ISL_798676, EPI_ISL_798688 to 798689, EPI_ISL_798704, EPI_ISL_798722, EPI_ISL_798727, EPI_ISL_798749, EPI_ISL_798768, EPI_ISL_798793, EPI_ISL_798814, EPI_ISL_798819, EPI_ISL_798833, EPI_ISL_798845, EPI_ISL_798850, EPI_ISL_798852, EPI_ISL_798869, EPI_ISL_798872, EPI_ISL_798878, EPI_ISL_798884, EPI_ISL_798891, EPI_ISL_798914, EPI_ISL_798926, EPI_ISL_798934, EPI_ISL_798939, EPI_ISL_798952, EPI_ISL_798962, EPI_ISL_798969, EPI_ISL_798991, EPI_ISL_799000, EPI_ISL_799003, EPI_ISL_799021, EPI_ISL_799025, EPI_ISL_799040, EPI_ISL_799057, EPI_ISL_799085, EPI_ISL_799103, EPI_ISL_799105, EPI_ISL_799122, EPI_ISL_799138 to 799139, EPI_ISL_799161, EPI_ISL_799166, EPI_ISL_799170 to 799171, EPI_ISL_799177 to 799178, EPI_ISL_799187, EPI_ISL_799198, EPI_ISL_799202, EPI_ISL_799212, EPI_ISL_799214, EPI_ISL_799231, EPI_ISL_799248, EPI_ISL_799265, EPI_ISL_799269, EPI_ISL_799271, EPI_ISL_799637, EPI_ISL_799644, EPI_ISL_799652, EPI_ISL_799655, EPI_ISL_799683, EPI_ISL_799687, EPI_ISL_799691, EPI_ISL_799702, EPI_ISL_799706, EPI_ISL_799711, EPI_ISL_799732, EPI_ISL_799749 to 799750, EPI_ISL_799760, EPI_ISL_799767, EPI_ISL_799787, EPI_ISL_799796, EPI_ISL_799809, EPI_ISL_799818, EPI_ISL_799822, EPI_ISL_799859, EPI_ISL_799868, EPI_ISL_799877, EPI_ISL_799891, EPI_ISL_799914, EPI_ISL_799920, EPI_ISL_799922, EPI_ISL_799924, EPI_ISL_799930, EPI_ISL_799938, EPI_ISL_799941 to 799943, EPI_ISL_799961, EPI_ISL_799970, EPI_ISL_799975, EPI_ISL_799982 to 799984, EPI_ISL_799995, EPI_ISL_799998, EPI_ISL_800011, EPI_ISL_800019 to 800020, EPI_ISL_800022, EPI_ISL_800025, EPI_ISL_800031, EPI_ISL_800033, EPI_ISL_800035, EPI_ISL_800039, EPI_ISL_800044, EPI_ISL_800051, EPI_ISL_800053, EPI_ISL_800061, EPI_ISL_800063, EPI_ISL_800067, EPI_ISL_800073, EPI_ISL_800076, EPI_ISL_800080, EPI_ISL_800087, EPI_ISL_800090, EPI_ISL_800097, EPI_ISL_800099, EPI_ISL_800101, EPI_ISL_800105, EPI_ISL_800108, EPI_ISL_800112, EPI_ISL_800126 to 800129, EPI_ISL_800136, EPI_ISL_800140, EPI_ISL_800144, EPI_ISL_800150, EPI_ISL_800162 to 800163, EPI_ISL_800165, EPI_ISL_800179, EPI_ISL_800181, EPI_ISL_800185, EPI_ISL_800193, EPI_ISL_800197, EPI_ISL_800199, EPI_ISL_800202, EPI_ISL_800206 to 800207, EPI_ISL_800212, EPI_ISL_800219, EPI_ISL_800229, EPI_ISL_800232, EPI_ISL_800242 to 800243, EPI_ISL_800246, EPI_ISL_800262, EPI_ISL_800267, EPI_ISL_800274, EPI_ISL_800280 to 800281, EPI_ISL_800299, EPI_ISL_800317, EPI_ISL_800367, EPI_ISL_800376, EPI_ISL_800384, EPI_ISL_800392, EPI_ISL_800395, EPI_ISL_800407, EPI_ISL_800444, EPI_ISL_800452, EPI_ISL_800460, EPI_ISL_800468, EPI_ISL_800495, EPI_ISL_800506, EPI_ISL_800509, EPI_ISL_800524 to 800525, EPI_ISL_800531, EPI_ISL_800542, EPI_ISL_800566, EPI_ISL_800578, EPI_ISL_820129 to 820130, EPI_ISL_820132, EPI_ISL_820137, EPI_ISL_820139 to 820140, EPI_ISL_820145, EPI_ISL_820148, EPI_ISL_820155, EPI_ISL_820157, EPI_ISL_820165 to 820166, EPI_ISL_820170, EPI_ISL_820174, EPI_ISL_820179, EPI_ISL_820181, EPI_ISL_820187, EPI_ISL_820189, EPI_ISL_820192, EPI_ISL_820195, EPI_ISL_820198, EPI_ISL_820201, EPI_ISL_820203, EPI_ISL_820205, EPI_ISL_820207, EPI_ISL_820210, EPI_ISL_820214, EPI_ISL_820217, EPI_ISL_820219, EPI_ISL_820222, EPI_ISL_820228, EPI_ISL_820230, EPI_ISL_820232, EPI_ISL_820235, EPI_ISL_820237, EPI_ISL_820240, EPI_ISL_820247 to 820248, EPI_ISL_820258, EPI_ISL_820275, EPI_ISL_820277 to 820278, EPI_ISL_820285, EPI_ISL_820288, EPI_ISL_820290, EPI_ISL_820293, EPI_ISL_820310, EPI_ISL_820315, EPI_ISL_820317, EPI_ISL_820322, EPI_ISL_820324 to 820325, EPI_ISL_820332, EPI_ISL_820339, EPI_ISL_820341 to 820342, EPI_ISL_820346, EPI_ISL_820351 to 820352, EPI_ISL_820357, EPI_ISL_820359, EPI_ISL_820367, EPI_ISL_820369 to 820370, EPI_ISL_820374 to 820375, EPI_ISL_820379 to 820380, EPI_ISL_820382, EPI_ISL_820389 to 820390, EPI_ISL_820392, EPI_ISL_820400 to 820401, EPI_ISL_820403, EPI_ISL_820414, EPI_ISL_820416, EPI_ISL_820422, EPI_ISL_820424 to 820425, EPI_ISL_820427, EPI_ISL_820429 to 820430, EPI_ISL_820432, EPI_ISL_820434, EPI_ISL_820442, EPI_ISL_820447, EPI_ISL_820452 to 820453, EPI_ISL_820455, EPI_ISL_820463, EPI_ISL_820469, EPI_ISL_820471, EPI_ISL_820473 to 820474, EPI_ISL_820476, EPI_ISL_820482, EPI_ISL_820487, EPI_ISL_820491 to 820492, EPI_ISL_820495, EPI_ISL_820497, EPI_ISL_820500, EPI_ISL_820508, EPI_ISL_820515, EPI_ISL_820517, EPI_ISL_820520, EPI_ISL_820523, EPI_ISL_820525, EPI_ISL_820527, EPI_ISL_820529 to 820530, EPI_ISL_820532, EPI_ISL_820534, EPI_ISL_820537, EPI_ISL_820540, EPI_ISL_820544, EPI_ISL_820550 to 820551, EPI_ISL_820553, EPI_ISL_820555, EPI_ISL_820559, EPI_ISL_820564, EPI_ISL_820566, EPI_ISL_820569,

EPI_ISL_820571 to 820572, EPI_ISL_820576, EPI_ISL_820579, EPI_ISL_820581, EPI_ISL_820584 to 820585, EPI_ISL_820587, EPI_ISL_820592, EPI_ISL_820594, EPI_ISL_820599 to 820600, EPI_ISL_820602, EPI_ISL_820604, EPI_ISL_820606, EPI_ISL_820611 to 820612, EPI_ISL_820615 to 820616, EPI_ISL_820623 to
820625, EPI_ISL_820627, EPI_ISL_820630 to 820631, EPI_ISL_820636, EPI_ISL_820640, EPI_ISL_820642 to 820644, EPI_ISL_821003, EPI_ISL_821005, EPI_ISL_821008, EPI_ISL_821010, EPI_ISL_821012 to 821013, EPI_ISL_821017 to 821018, EPI_ISL_821020 to 821023, EPI_ISL_821026, EPI_ISL_821030,
EPI_ISL_821034 to 821035, EPI_ISL_821039 to 821040, EPI_ISL_821042, EPI_ISL_821044 to 821048, EPI_ISL_821055, EPI_ISL_821058, EPI_ISL_821064, EPI_ISL_821066, EPI_ISL_821068, EPI_ISL_821070 to 821073, EPI_ISL_821076, EPI_ISL_821078, EPI_ISL_821082, EPI_ISL_821085, EPI_ISL_821087 to 821088,
EPI_ISL_821090 to 821094, EPI_ISL_821097, EPI_ISL_821099 to 821100, EPI_ISL_821104, EPI_ISL_821106 to 821107, EPI_ISL_821109, EPI_ISL_821111, EPI_ISL_821113 to 821117, EPI_ISL_821119 to 821121, EPI_ISL_821124 to 821127, EPI_ISL_821130 to 821131, EPI_ISL_821133, EPI_ISL_821137, EPI_ISL_821139,
EPI_ISL_821141, EPI_ISL_821144 to 821146, EPI_ISL_821148 to 821149, EPI_ISL_821151 to 821152, EPI_ISL_821154, EPI_ISL_821156, EPI_ISL_821158 to 821159, EPI_ISL_821162 to 821163, EPI_ISL_821165 to 821166, EPI_ISL_821169, EPI_ISL_821171 to 821175, EPI_ISL_821182 to 821185, EPI_ISL_821187,
EPI_ISL_821189, EPI_ISL_821191 to 821193, EPI_ISL_821197, EPI_ISL_821200 to 821202, EPI_ISL_821206, EPI_ISL_821208, EPI_ISL_821211, EPI_ISL_821215, EPI_ISL_821218 to 821219, EPI_ISL_821222 to 821223, EPI_ISL_821228, EPI_ISL_821231, EPI_ISL_821233, EPI_ISL_821236, EPI_ISL_821238,
EPI_ISL_821243 to 821244, EPI_ISL_821247 to 821248, EPI_ISL_821251, EPI_ISL_821253 to 821255, EPI_ISL_821260 to 821261, EPI_ISL_821263 to 821265, EPI_ISL_821267, EPI_ISL_821270 to 821271, EPI_ISL_821283, EPI_ISL_821285, EPI_ISL_821292, EPI_ISL_821294, EPI_ISL_821298, EPI_ISL_821303,
EPI_ISL_821308, EPI_ISL_821314, EPI_ISL_821318, EPI_ISL_821327, EPI_ISL_821329, EPI_ISL_821347, EPI_ISL_821370 to 821371, EPI_ISL_821382, EPI_ISL_821384, EPI_ISL_821390, EPI_ISL_821401, EPI_ISL_821403, EPI_ISL_821412, EPI_ISL_821414 to 821415, EPI_ISL_821425 to 821426, EPI_ISL_821433,
EPI_ISL_821436 to 821437, EPI_ISL_821446, EPI_ISL_821456, EPI_ISL_821459, EPI_ISL_821476, EPI_ISL_821482, EPI_ISL_821490, EPI_ISL_821493, EPI_ISL_821496, EPI_ISL_821502 to 821503, EPI_ISL_821518, EPI_ISL_821526, EPI_ISL_821528, EPI_ISL_821533 to 821534, EPI_ISL_821550 to 821553,
EPI_ISL_821556, EPI_ISL_821558, EPI_ISL_821584 to 821585, EPI_ISL_821600, EPI_ISL_821606 to 821607, EPI_ISL_821614, EPI_ISL_821971, EPI_ISL_821973, EPI_ISL_821976 to 821980, EPI_ISL_821982 to 821984, EPI_ISL_821986 to 821987, EPI_ISL_821990, EPI_ISL_821997 to 822000, EPI_ISL_822002 to 822003,
EPI_ISL_822007 to 822010, EPI_ISL_822012, EPI_ISL_822014 to 822018, EPI_ISL_822020 to 822021, EPI_ISL_822024, EPI_ISL_822027 to 822028, EPI_ISL_822030 to 822032, EPI_ISL_822036, EPI_ISL_822038 to 822039, EPI_ISL_822045 to 822047, EPI_ISL_822049 to 822050, EPI_ISL_822052, EPI_ISL_822054 to 822057,
EPI_ISL_822060 to 822062, EPI_ISL_822064 to 822065, EPI_ISL_822068, EPI_ISL_822070 to 822071, EPI_ISL_822073 to 822074, EPI_ISL_822076 to 822077, EPI_ISL_822081 to 822082, EPI_ISL_822084 to 822085, EPI_ISL_822088 to 822090, EPI_ISL_822093 to 822094, EPI_ISL_822096 to 822097, EPI_ISL_822100,
EPI_ISL_822102, EPI_ISL_822104, EPI_ISL_822106, EPI_ISL_822108 to 822112, EPI_ISL_822114 to 822116, EPI_ISL_822118, EPI_ISL_822120, EPI_ISL_822123, EPI_ISL_822125, EPI_ISL_822128 to 822129, EPI_ISL_822131, EPI_ISL_822137 to 822139, EPI_ISL_822143 to 822145, EPI_ISL_822147, EPI_ISL_822149 to
822152, EPI_ISL_822154 to 822155, EPI_ISL_822157, EPI_ISL_822160 to 822162, EPI_ISL_822164, EPI_ISL_822166 to 822168, EPI_ISL_822171 to 822175, EPI_ISL_822177, EPI_ISL_822179 to 822181, EPI_ISL_822183, EPI_ISL_822185 to 822190, EPI_ISL_822192, EPI_ISL_822194 to 822198, EPI_ISL_822200,
EPI_ISL_822203 to 822208, EPI_ISL_822210 to 822213, EPI_ISL_822215 to 822220, EPI_ISL_822222, EPI_ISL_822225 to 822229, EPI_ISL_822231, EPI_ISL_822234 to 822235, EPI_ISL_822238 to 822239, EPI_ISL_822241, EPI_ISL_822246, EPI_ISL_822248, EPI_ISL_822251 to 822252, EPI_ISL_822254, EPI_ISL_822257 to
822259, EPI_ISL_822261 to 822263, EPI_ISL_822269 to 822271, EPI_ISL_822273, EPI_ISL_822278 to 822280, EPI_ISL_822283 to 822287, EPI_ISL_822289 to 822290, EPI_ISL_822294, EPI_ISL_822308, EPI_ISL_826467 to 826469, EPI_ISL_826472, EPI_ISL_826474, EPI_ISL_826476, EPI_ISL_833935, EPI_ISL_833937,
EPI_ISL_833939, EPI_ISL_833941 to 833942, EPI_ISL_833945 to 833950, EPI_ISL_833953 to 833954, EPI_ISL_833956 to 833959, EPI_ISL_833961, EPI_ISL_833969, EPI_ISL_833972 to 833974, EPI_ISL_833977, EPI_ISL_833980, EPI_ISL_833984 to 833988, EPI_ISL_833990, EPI_ISL_833992, EPI_ISL_833996,
EPI_ISL_833998 to 834000, EPI_ISL_834002, EPI_ISL_834005 to 834006, EPI_ISL_834010 to 834012, EPI_ISL_834016, EPI_ISL_834018, EPI_ISL_834020, EPI_ISL_834022, EPI_ISL_834025 to 834026, EPI_ISL_834031, EPI_ISL_834033, EPI_ISL_834035 to 834036, EPI_ISL_834038, EPI_ISL_834041, EPI_ISL_834044,
EPI_ISL_834046 to 834049, EPI_ISL_834051 to 834053, EPI_ISL_834058, EPI_ISL_834063 to 834065, EPI_ISL_834069, EPI_ISL_834071, EPI_ISL_834075, EPI_ISL_834077, EPI_ISL_834086, EPI_ISL_834093, EPI_ISL_834097, EPI_ISL_834099, EPI_ISL_834101 to 834103, EPI_ISL_834106 to 834108, EPI_ISL_834110 to
834112, EPI_ISL_834114 to 834115, EPI_ISL_834117 to 834123, EPI_ISL_834125 to 834128, EPI_ISL_834131 to 834132, EPI_ISL_834135, EPI_ISL_834137 to 834138, EPI_ISL_834141 to 834143, EPI_ISL_834146, EPI_ISL_834148 to 834150, EPI_ISL_834152 to 834157, EPI_ISL_834160 to 834162, EPI_ISL_834165 to
834167, EPI_ISL_834169, EPI_ISL_834172 to 834180, EPI_ISL_834182 to 834183, EPI_ISL_834186, EPI_ISL_834188 to 834191, EPI_ISL_834193, EPI_ISL_834195 to 834196, EPI_ISL_834201 to 834204, EPI_ISL_834208 to 834215, EPI_ISL_834218, EPI_ISL_834220, EPI_ISL_834222 to 834228, EPI_ISL_834231,
EPI_ISL_834233, EPI_ISL_834235 to 834237, EPI_ISL_834239 to 834241, EPI_ISL_834243 to 834244, EPI_ISL_834247 to 834249, EPI_ISL_834251 to 834253, EPI_ISL_834256 to 834261, EPI_ISL_834263 to 834264, EPI_ISL_834267 to 834272, EPI_ISL_834274 to 834276, EPI_ISL_834282, EPI_ISL_834284 to 834286,
EPI_ISL_834290 to 834293, EPI_ISL_834296, EPI_ISL_834298 to 834299, EPI_ISL_834301 to 834302, EPI_ISL_834304 to 834305, EPI_ISL_834307, EPI_ISL_834310 to 834314, EPI_ISL_834316 to 834318, EPI_ISL_834322, EPI_ISL_834324, EPI_ISL_834328 to 834329, EPI_ISL_834331 to 834332, EPI_ISL_834336 to 834341,
EPI_ISL_834343 to 834347, EPI_ISL_834350, EPI_ISL_834352 to 834358, EPI_ISL_834360 to 834362, EPI_ISL_834364 to 834365, EPI_ISL_834367 to 834368, EPI_ISL_834370 to 834371, EPI_ISL_834376 to 834380, EPI_ISL_834382 to 834383, EPI_ISL_834385 to 834386, EPI_ISL_834388 to 834391, EPI_ISL_834393,
EPI_ISL_834396 to 834397, EPI_ISL_834401 to 834407, EPI_ISL_834409, EPI_ISL_834411 to 834412, EPI_ISL_834414 to 834417, EPI_ISL_834419 to 834420, EPI_ISL_834422 to 834437, EPI_ISL_834439 to 834441, EPI_ISL_834443 to 834444, EPI_ISL_834446 to 834447, EPI_ISL_834449, EPI_ISL_834452, EPI_ISL_834455
to 834460, EPI_ISL_834463, EPI_ISL_834466 to 834468, EPI_ISL_834474 to 834475, EPI_ISL_834477 to 834478, EPI_ISL_834480, EPI_ISL_834484 to 834496, EPI_ISL_834498, EPI_ISL_834500 to 834508, EPI_ISL_834511 to 834514, EPI_ISL_834516 to 834520, EPI_ISL_834523 to 834532, EPI_ISL_834534, EPI_ISL_834536
to 834541, EPI_ISL_834544 to 834548, EPI_ISL_834550, EPI_ISL_834552, EPI_ISL_834798, EPI_ISL_834800, EPI_ISL_834803 to 834807, EPI_ISL_834810, EPI_ISL_834812 to 834815, EPI_ISL_834819 to 834820, EPI_ISL_834822 to 834827, EPI_ISL_834829, EPI_ISL_834831 to 834840, EPI_ISL_834842 to 834844,
EPI_ISL_834847 to 834850, EPI_ISL_834853, EPI_ISL_834857 to 834858, EPI_ISL_834860, EPI_ISL_834865 to 834876, EPI_ISL_834878, EPI_ISL_834880 to 834886, EPI_ISL_834888 to 834889, EPI_ISL_834891 to 834894, EPI_ISL_834896 to 834900, EPI_ISL_834903 to 834905, EPI_ISL_834908, EPI_ISL_834910,
EPI_ISL_834915 to 834916, EPI_ISL_834920 to 834922, EPI_ISL_834925 to 834927, EPI_ISL_834929 to 834934, EPI_ISL_834936 to 834943, EPI_ISL_834945, EPI_ISL_834947, EPI_ISL_834949, EPI_ISL_834951 to 834952, EPI_ISL_834955 to 834956, EPI_ISL_834958 to 834960, EPI_ISL_834964 to 834965, EPI_ISL_834971,
EPI_ISL_834974 to 834977, EPI_ISL_834979, EPI_ISL_834981 to 834985, EPI_ISL_834987 to 834988, EPI_ISL_834990 to 834994, EPI_ISL_834997, EPI_ISL_835000 to 835003, EPI_ISL_835008, EPI_ISL_835010, EPI_ISL_835013, EPI_ISL_835015, EPI_ISL_835017 to 835019, EPI_ISL_835024 to 835025, EPI_ISL_835031,
EPI_ISL_835033 to 835040, EPI_ISL_835043 to 835044, EPI_ISL_835047 to 835048, EPI_ISL_835050 to 835052, EPI_ISL_835054 to 835057, EPI_ISL_835059, EPI_ISL_835064 to 835065, EPI_ISL_835067, EPI_ISL_835069, EPI_ISL_835071, EPI_ISL_835078, EPI_ISL_835080 to 835081, EPI_ISL_835084, EPI_ISL_835087,
EPI_ISL_835090 to 835092, EPI_ISL_835096, EPI_ISL_835098 to 835099, EPI_ISL_835101 to 835104, EPI_ISL_835107, EPI_ISL_835110, EPI_ISL_835113, EPI_ISL_835115 to 835116, EPI_ISL_835119, EPI_ISL_835123, EPI_ISL_835125, EPI_ISL_835127 to 835128, EPI_ISL_835130, EPI_ISL_835132, EPI_ISL_835135 to
835137, EPI_ISL_835139 to 835140, EPI_ISL_835428 to 835432, EPI_ISL_835434 to 835438, EPI_ISL_835443 to 835446, EPI_ISL_835449 to 835452, EPI_ISL_835455 to 835456, EPI_ISL_835460 to 835463, EPI_ISL_835468 to 835469, EPI_ISL_835473, EPI_ISL_835476 to 835478, EPI_ISL_835480, EPI_ISL_835484,
EPI_ISL_835489, EPI_ISL_835491 to 835492, EPI_ISL_835494 to 835495, EPI_ISL_835501, EPI_ISL_835504, EPI_ISL_835509 to 835512, EPI_ISL_835514, EPI_ISL_835516 to 835517, EPI_ISL_835519 to 835522, EPI_ISL_835524, EPI_ISL_835526, EPI_ISL_835528 to 835529, EPI_ISL_835532, EPI_ISL_835534,
EPI_ISL_835536 to 835538, EPI_ISL_835542 to 835543, EPI_ISL_835546, EPI_ISL_835549 to 835551, EPI_ISL_835554, EPI_ISL_835556 to 835558, EPI_ISL_835561 to 835563, EPI_ISL_835565 to 835567, EPI_ISL_835569, EPI_ISL_835571 to 835572, EPI_ISL_835575, EPI_ISL_835578, EPI_ISL_835580, EPI_ISL_835585 to
835586, EPI_ISL_835588, EPI_ISL_835591, EPI_ISL_835593 to 835595, EPI_ISL_835598 to 835599, EPI_ISL_835601 to 835602, EPI_ISL_835604, EPI_ISL_835607, EPI_ISL_835611 to 835613, EPI_ISL_835615, EPI_ISL_835617, EPI_ISL_835619 to 835620, EPI_ISL_835625 to 835630, EPI_ISL_835632 to 835634,
EPI_ISL_835636 to 835637, EPI_ISL_835639, EPI_ISL_835641 to 835642, EPI_ISL_835644 to 835645, EPI_ISL_835649 to 835650, EPI_ISL_835652, EPI_ISL_835655, EPI_ISL_835657 to 835658, EPI_ISL_835660 to 835663, EPI_ISL_835666, EPI_ISL_835669 to 835670, EPI_ISL_835672, EPI_ISL_835674 to 835675,
EPI_ISL_835679 to 835681, EPI_ISL_835684 to 835685, EPI_ISL_835689 to 835691, EPI_ISL_835693 to 835694, EPI_ISL_835696 to 835698, EPI_ISL_835700, EPI_ISL_835702, EPI_ISL_835704, EPI_ISL_835706, EPI_ISL_835708 to 835709, EPI_ISL_836766, EPI_ISL_836769, EPI_ISL_836781, EPI_ISL_836788,
EPI_ISL_836792, EPI_ISL_836795, EPI_ISL_836797, EPI_ISL_836803, EPI_ISL_836806 to 836807, EPI_ISL_836811, EPI_ISL_836814, EPI_ISL_836817, EPI_ISL_836822, EPI_ISL_836840, EPI_ISL_836851, EPI_ISL_836862, EPI_ISL_836866, EPI_ISL_836876 to 836878, EPI_ISL_836883, EPI_ISL_836887, EPI_ISL_836906,
EPI_ISL_836910, EPI_ISL_836913, EPI_ISL_836920 to 836922, EPI_ISL_836929, EPI_ISL_836936, EPI_ISL_836946 to 836948, EPI_ISL_836955, EPI_ISL_836962, EPI_ISL_846200, EPI_ISL_846204 to 846205, EPI_ISL_846208 to 846210, EPI_ISL_846213, EPI_ISL_846215, EPI_ISL_846218 to 846219, EPI_ISL_846228 to
846229, EPI_ISL_846231, EPI_ISL_846233 to 846235, EPI_ISL_846239 to 846240, EPI_ISL_846245 to 846247, EPI_ISL_846249 to 846250, EPI_ISL_846252 to 846253, EPI_ISL_846257 to 846258, EPI_ISL_846260 to 846261, EPI_ISL_846263 to 846265, EPI_ISL_846267, EPI_ISL_846272, EPI_ISL_846274 to 846278,
EPI_ISL_846288 to 846289, EPI_ISL_846291, EPI_ISL_846295, EPI_ISL_846297 to 846300, EPI_ISL_846302 to 846305, EPI_ISL_846307 to 846308, EPI_ISL_846311, EPI_ISL_846313, EPI_ISL_846315 to 846316, EPI_ISL_846321 to 846322, EPI_ISL_846324, EPI_ISL_846326 to 846327, EPI_ISL_846329, EPI_ISL_846331,
EPI_ISL_846338 to 846339, EPI_ISL_846344 to 846345, EPI_ISL_846348, EPI_ISL_846351 to 846354, EPI_ISL_846356 to 846357, EPI_ISL_846359, EPI_ISL_846365, EPI_ISL_846367 to 846370, EPI_ISL_846373 to 846374, EPI_ISL_846376, EPI_ISL_846378, EPI_ISL_846380, EPI_ISL_846382 to 846384, EPI_ISL_846386 to
846390, EPI_ISL_846394 to 846395, EPI_ISL_846397, EPI_ISL_846399 to 846401, EPI_ISL_846403, EPI_ISL_846408, EPI_ISL_846414 to 846415, EPI_ISL_846419, EPI_ISL_846422 to 846424, EPI_ISL_846426, EPI_ISL_846429, EPI_ISL_846431, EPI_ISL_846434, EPI_ISL_846437, EPI_ISL_846445, EPI_ISL_846447 to
846448, EPI_ISL_846450, EPI_ISL_846455 to 846456, EPI_ISL_846458 to 846460, EPI_ISL_846464 to 846466, EPI_ISL_846469 to 846473, EPI_ISL_846478 to 846479, EPI_ISL_846481 to 846483, EPI_ISL_846485 to 846486, EPI_ISL_846489, EPI_ISL_846495, EPI_ISL_846497, EPI_ISL_846499 to 846503, EPI_ISL_846506 to
846508, EPI_ISL_846512 to 846513, EPI_ISL_846515 to 846517, EPI_ISL_846522, EPI_ISL_846526, EPI_ISL_846536, EPI_ISL_851675, EPI_ISL_851679, EPI_ISL_851682 to 851683, EPI_ISL_851691 to 851692, EPI_ISL_851694, EPI_ISL_851696 to 851698, EPI_ISL_851703, EPI_ISL_851707 to 851711, EPI_ISL_851713,
EPI_ISL_851715 to 851716, EPI_ISL_851721 to 851722, EPI_ISL_851724, EPI_ISL_851730, EPI_ISL_851732 to 851733, EPI_ISL_851735 to 851736, EPI_ISL_851739, EPI_ISL_851741, EPI_ISL_851744, EPI_ISL_851746, EPI_ISL_851748, EPI_ISL_851750 to 851751, EPI_ISL_851756, EPI_ISL_851758 to 851759,
EPI_ISL_851765, EPI_ISL_851768, EPI_ISL_851773, EPI_ISL_851775, EPI_ISL_851778 to 851779, EPI_ISL_851782 to 851784, EPI_ISL_851786 to 851787, EPI_ISL_851790, EPI_ISL_851800 to 851802, EPI_ISL_851808 to 851811, EPI_ISL_851819, EPI_ISL_851821, EPI_ISL_851824 to 851828, EPI_ISL_851830 to 851832,
EPI_ISL_851834 to 851835, EPI_ISL_851841 to 851842, EPI_ISL_851845, EPI_ISL_851848, EPI_ISL_851853 to 851854, EPI_ISL_851858, EPI_ISL_851861, EPI_ISL_851867, EPI_ISL_851869, EPI_ISL_851872 to 851874, EPI_ISL_851878, EPI_ISL_851881 to 851883, EPI_ISL_851886 to 851887, EPI_ISL_851889,
EPI_ISL_851893, EPI_ISL_851896 to 851897, EPI_ISL_851900, EPI_ISL_851908 to 851910, EPI_ISL_851912, EPI_ISL_851919, EPI_ISL_851921, EPI_ISL_851923 to 851924, EPI_ISL_851930, EPI_ISL_851935, EPI_ISL_851938, EPI_ISL_851944 to 851946, EPI_ISL_851951 to 851953, EPI_ISL_852047, EPI_ISL_852083,
EPI_ISL_852214, EPI_ISL_852225, EPI_ISL_857879 to 857880, EPI_ISL_857882, EPI_ISL_857969, EPI_ISL_857974 to 857975, EPI_ISL_857987, EPI_ISL_857991, EPI_ISL_857997 to 857998, EPI_ISL_858004, EPI_ISL_858006 to 858007, EPI_ISL_858067, EPI_ISL_858070, EPI_ISL_858076, EPI_ISL_858080,
EPI_ISL_858082, EPI_ISL_858093, EPI_ISL_858096, EPI_ISL_858098, EPI_ISL_858100, EPI_ISL_858107 to 858108, EPI_ISL_858113, EPI_ISL_858115 to 858116, EPI_ISL_858118 to 858120, EPI_ISL_858129, EPI_ISL_858134, EPI_ISL_858140, EPI_ISL_858212, EPI_ISL_858216, EPI_ISL_858219, EPI_ISL_858357,
EPI_ISL_858368, EPI_ISL_858572, EPI_ISL_858575 to 858576, EPI_ISL_858582, EPI_ISL_858645, EPI_ISL_858663, EPI_ISL_858666 to 858667, EPI_ISL_858676, EPI_ISL_858678, EPI_ISL_858681, EPI_ISL_858688, EPI_ISL_858690, EPI_ISL_858693, EPI_ISL_858697, EPI_ISL_858699, EPI_ISL_858701, EPI_ISL_858704,
EPI_ISL_858714, EPI_ISL_858788 to 858790, EPI_ISL_858819, EPI_ISL_858823, EPI_ISL_858825, EPI_ISL_858840, EPI_ISL_858853, EPI_ISL_858872, EPI_ISL_858875, EPI_ISL_858879, EPI_ISL_858885 to 858888, EPI_ISL_858891, EPI_ISL_858896, EPI_ISL_858898, EPI_ISL_858914, EPI_ISL_858924, EPI_ISL_858966,
EPI_ISL_858968, EPI_ISL_858973, EPI_ISL_858999, EPI_ISL_859002, EPI_ISL_859007, EPI_ISL_859010, EPI_ISL_859016, EPI_ISL_859019, EPI_ISL_859026, EPI_ISL_859082, EPI_ISL_859085, EPI_ISL_859113, EPI_ISL_859117, EPI_ISL_859121, EPI_ISL_859138, EPI_ISL_859144, EPI_ISL_859203, EPI_ISL_859210,
EPI_ISL_859217, EPI_ISL_859224 to 859225, EPI_ISL_859227 to 859228, EPI_ISL_859250, EPI_ISL_859257, EPI_ISL_859261, EPI_ISL_859267, EPI_ISL_859269, EPI_ISL_859310, EPI_ISL_859335, EPI_ISL_859374, EPI_ISL_859376, EPI_ISL_859382, EPI_ISL_859386, EPI_ISL_859388, EPI_ISL_859405, EPI_ISL_859410,
EPI_ISL_859415, EPI_ISL_859421, EPI_ISL_859441, EPI_ISL_859449, EPI_ISL_862886, EPI_ISL_862888 to 862889, EPI_ISL_862892 to 862893, EPI_ISL_862895 to 862899, EPI_ISL_862901 to 862902, EPI_ISL_862905, EPI_ISL_862908, EPI_ISL_862913 to 862916, EPI_ISL_862926, EPI_ISL_862928 to 862929,
EPI_ISL_862931 to 862933, EPI_ISL_862936, EPI_ISL_862938, EPI_ISL_862942 to 862944, EPI_ISL_862948, EPI_ISL_862954, EPI_ISL_862960, EPI_ISL_862965, EPI_ISL_862967, EPI_ISL_862969, EPI_ISL_862972, EPI_ISL_862975, EPI_ISL_862977 to 862979, EPI_ISL_862982, EPI_ISL_862984, EPI_ISL_862986 to
862987, EPI_ISL_862990 to 862991, EPI_ISL_862994 to 862995, EPI_ISL_862997, EPI_ISL_863000, EPI_ISL_863005 to 863006, EPI_ISL_863011 to 863014, EPI_ISL_863017, EPI_ISL_863020 to 863021, EPI_ISL_863023 to 863025, EPI_ISL_863033, EPI_ISL_863039 to 863043, EPI_ISL_863045, EPI_ISL_863047,
EPI_ISL_863054 to 863057, EPI_ISL_863060 to 863064, EPI_ISL_863066 to 863068, EPI_ISL_863070 to 863071, EPI_ISL_863073 to 863074, EPI_ISL_863080, EPI_ISL_863085, EPI_ISL_863087, EPI_ISL_863089, EPI_ISL_863091 to 863093, EPI_ISL_863096 to 863099, EPI_ISL_863101, EPI_ISL_863108 to 863109,
EPI_ISL_863111, EPI_ISL_863113 to 863114, EPI_ISL_863120, EPI_ISL_863122 to 863126, EPI_ISL_863129 to 863133, EPI_ISL_863136 to 863137, EPI_ISL_863140 to 863142, EPI_ISL_863146 to 863147, EPI_ISL_863149 to 863152, EPI_ISL_863154, EPI_ISL_863156, EPI_ISL_863158 to 863159, EPI_ISL_863162 to 863163,
EPI_ISL_863165 to 863171, EPI_ISL_863173, EPI_ISL_863176 to 863177, EPI_ISL_863179, EPI_ISL_863184 to 863185, EPI_ISL_863187 to 863188, EPI_ISL_863190, EPI_ISL_863192, EPI_ISL_863195 to 863197, EPI_ISL_863199 to 863200, EPI_ISL_863208 to 863210, EPI_ISL_863212, EPI_ISL_863216 to 863217,
EPI_ISL_863219 to 863221, EPI_ISL_863223 to 863225, EPI_ISL_863227 to 863229, EPI_ISL_863231 to 863232, EPI_ISL_863236 to 863240, EPI_ISL_863242 to 863244, EPI_ISL_863254, EPI_ISL_863268, EPI_ISL_863270 to 863271, EPI_ISL_863274 to 863275, EPI_ISL_863281, EPI_ISL_863302, EPI_ISL_863306,
EPI_ISL_863310 to 863311, EPI_ISL_863319, EPI_ISL_863326, EPI_ISL_863334, EPI_ISL_863338, EPI_ISL_863342 to 863343, EPI_ISL_863346, EPI_ISL_863357, EPI_ISL_863362 to 863364, EPI_ISL_863377, EPI_ISL_863395 to 863397, EPI_ISL_863401, EPI_ISL_863403 to 863404, EPI_ISL_863406, EPI_ISL_863408,
EPI_ISL_863411, EPI_ISL_863414, EPI_ISL_863426, EPI_ISL_863432, EPI_ISL_863437, EPI_ISL_863446 to 863448, EPI_ISL_863454, EPI_ISL_863461, EPI_ISL_863467, EPI_ISL_863470, EPI_ISL_863479, EPI_ISL_863484, EPI_ISL_863514, EPI_ISL_863516, EPI_ISL_863520, EPI_ISL_863869, EPI_ISL_863872 to 863874,
EPI_ISL_863877 to 863883, EPI_ISL_863886, EPI_ISL_863888 to 863890, EPI_ISL_863895 to 863896, EPI_ISL_863898, EPI_ISL_863904, EPI_ISL_863908 to 863910, EPI_ISL_863914, EPI_ISL_863918, EPI_ISL_863925 to 863926, EPI_ISL_863928 to 863929, EPI_ISL_863932, EPI_ISL_863934, EPI_ISL_863936,
EPI_ISL_863938 to 863940, EPI_ISL_863943, EPI_ISL_863945 to 863947, EPI_ISL_863952 to 863953, EPI_ISL_863957 to 863958, EPI_ISL_863960 to 863961, EPI_ISL_863964 to 863965, EPI_ISL_863968, EPI_ISL_863970, EPI_ISL_863972, EPI_ISL_863974, EPI_ISL_863976 to 863977, EPI_ISL_863980, EPI_ISL_863983,
EPI_ISL_863986 to 863989, EPI_ISL_863991 to 863994, EPI_ISL_863997, EPI_ISL_863999 to 864000, EPI_ISL_864003 to 864004, EPI_ISL_864006, EPI_ISL_864008, EPI_ISL_864011 to 864013, EPI_ISL_864015 to 864018, EPI_ISL_864020, EPI_ISL_864022 to 864023, EPI_ISL_864025, EPI_ISL_864029, EPI_ISL_864033 to
864034, EPI_ISL_864038 to 864039, EPI_ISL_864042, EPI_ISL_864044 to 864049, EPI_ISL_864051, EPI_ISL_864054, EPI_ISL_864056 to 864063, EPI_ISL_864066 to 864068, EPI_ISL_864075, EPI_ISL_864077 to 864078, EPI_ISL_864080 to 864081, EPI_ISL_864083 to 864084, EPI_ISL_864086 to 864088, EPI_ISL_864092 to
864097, EPI_ISL_864100 to 864102, EPI_ISL_864108, EPI_ISL_864111 to 864113, EPI_ISL_864115, EPI_ISL_864117 to 864118, EPI_ISL_864120, EPI_ISL_864122, EPI_ISL_864129 to 864130, EPI_ISL_864132, EPI_ISL_864136, EPI_ISL_864138, EPI_ISL_864140, EPI_ISL_864143 to 864145, EPI_ISL_864147,
EPI_ISL_864150, EPI_ISL_864152 to 864156, EPI_ISL_864159, EPI_ISL_864163, EPI_ISL_864167 to 864168, EPI_ISL_864170, EPI_ISL_864172, EPI_ISL_864177, EPI_ISL_864181 to 864183, EPI_ISL_864185 to 864186, EPI_ISL_864188 to 864190, EPI_ISL_864192, EPI_ISL_864194 to 864197, EPI_ISL_864200,
EPI_ISL_864202, EPI_ISL_864226, EPI_ISL_864267, EPI_ISL_864308, EPI_ISL_873963, EPI_ISL_873966 to 873968, EPI_ISL_873970, EPI_ISL_873972 to 873973, EPI_ISL_873976, EPI_ISL_873978, EPI_ISL_873980 to 873981, EPI_ISL_873985 to 873986, EPI_ISL_873990 to 873991, EPI_ISL_873993 to 873998,
EPI_ISL_874000, EPI_ISL_874003, EPI_ISL_874006, EPI_ISL_874008 to 874009, EPI_ISL_874011 to 874012, EPI_ISL_874014, EPI_ISL_874016 to 874017, EPI_ISL_874019 to 874020, EPI_ISL_874023 to 874026, EPI_ISL_874029, EPI_ISL_874031 to 874036, EPI_ISL_874038, EPI_ISL_874040, EPI_ISL_874042 to 874044,
EPI_ISL_874046 to 874048, EPI_ISL_874050 to 874052, EPI_ISL_874054, EPI_ISL_874063 to 874064, EPI_ISL_874066 to 874070, EPI_ISL_874072 to 874073, EPI_ISL_874075, EPI_ISL_874078 to 874080, EPI_ISL_874082 to 874088, EPI_ISL_874090 to 874093, EPI_ISL_874095 to 874096, EPI_ISL_874098, EPI_ISL_874101
to 874106, EPI_ISL_874109, EPI_ISL_874112 to 874114, EPI_ISL_874117 to 874118, EPI_ISL_874120, EPI_ISL_874122, EPI_ISL_874124 to 874126, EPI_ISL_874129, EPI_ISL_874131 to 874137, EPI_ISL_874139 to 874142, EPI_ISL_874146, EPI_ISL_874149 to 874152, EPI_ISL_874154 to 874156, EPI_ISL_874159 to 874160,
EPI_ISL_874163, EPI_ISL_874167, EPI_ISL_874169, EPI_ISL_874171 to 874172, EPI_ISL_874174, EPI_ISL_874179, EPI_ISL_874181 to 874182, EPI_ISL_874184 to 874185, EPI_ISL_874188, EPI_ISL_874190 to 874192, EPI_ISL_874194 to 874195, EPI_ISL_874198 to 874200, EPI_ISL_874202 to 874203, EPI_ISL_874205,
EPI_ISL_874208 to 874209, EPI_ISL_874214 to 874215, EPI_ISL_874220 to 874221, EPI_ISL_874225, EPI_ISL_874230, EPI_ISL_874232 to 874234, EPI_ISL_874236 to 874237, EPI_ISL_874239 to 874246, EPI_ISL_874248, EPI_ISL_874252 to 874253, EPI_ISL_874255, EPI_ISL_874257 to 874262, EPI_ISL_874267 to 874268,
EPI_ISL_874270, EPI_ISL_874272, EPI_ISL_874274 to 874276, EPI_ISL_874279 to 874280, EPI_ISL_874284 to 874285, EPI_ISL_874287, EPI_ISL_874289, EPI_ISL_874293 to 874295, EPI_ISL_874298, EPI_ISL_874302, EPI_ISL_874304 to 874305, EPI_ISL_874307 to 874310, EPI_ISL_874312, EPI_ISL_874316 to 874319,
EPI_ISL_874321 to 874322, EPI_ISL_874324, EPI_ISL_874327, EPI_ISL_874329, EPI_ISL_874331 to 874335, EPI_ISL_874339 to 874344, EPI_ISL_874346, EPI_ISL_874348, EPI_ISL_874352 to 874354, EPI_ISL_874356, EPI_ISL_874358, EPI_ISL_874360, EPI_ISL_874362 to 874363, EPI_ISL_874365 to 874366,
EPI_ISL_874369 to 874370, EPI_ISL_874372, EPI_ISL_874375, EPI_ISL_874378, EPI_ISL_874380, EPI_ISL_874383, EPI_ISL_874385, EPI_ISL_874388 to 874391, EPI_ISL_874393 to 874394, EPI_ISL_874397 to 874398, EPI_ISL_874404 to 874408, EPI_ISL_874410, EPI_ISL_874413, EPI_ISL_874415, EPI_ISL_874417 to
874420, EPI_ISL_874422, EPI_ISL_874424, EPI_ISL_874427, EPI_ISL_874429 to 874430, EPI_ISL_874434, EPI_ISL_874438, EPI_ISL_874441, EPI_ISL_874443, EPI_ISL_874445, EPI_ISL_874448 to 874449, EPI_ISL_874451, EPI_ISL_874453, EPI_ISL_874456, EPI_ISL_874458, EPI_ISL_874460 to 874461, EPI_ISL_874464
to 874470, EPI_ISL_874472 to 874475, EPI_ISL_874477 to 874478, EPI_ISL_874481, EPI_ISL_874483, EPI_ISL_874490, EPI_ISL_874492, EPI_ISL_874494, EPI_ISL_874497, EPI_ISL_874502, EPI_ISL_874504, EPI_ISL_874506 to 874507, EPI_ISL_874510 to 874511, EPI_ISL_874514 to 874515, EPI_ISL_874517 to 874519,
EPI_ISL_874521, EPI_ISL_874523 to 874524, EPI_ISL_874527 to 874528, EPI_ISL_874532 to 874533, EPI_ISL_874536 to 874537, EPI_ISL_874539, EPI_ISL_874541 to 874544, EPI_ISL_874548, EPI_ISL_874551 to 874553, EPI_ISL_874557, EPI_ISL_874559, EPI_ISL_874562 to 874563, EPI_ISL_874566, EPI_ISL_874570,
EPI_ISL_874572, EPI_ISL_874574, EPI_ISL_874579, EPI_ISL_874581 to 874586, EPI_ISL_874590, EPI_ISL_874592, EPI_ISL_874594 to 874595, EPI_ISL_874604 to 874608, EPI_ISL_874610 to 874611, EPI_ISL_874615 to 874616, EPI_ISL_874618, EPI_ISL_874620 to 874622, EPI_ISL_874624, EPI_ISL_874628,
EPI_ISL_874630, EPI_ISL_874633 to 874635, EPI_ISL_874637 to 874642, EPI_ISL_874647 to 874648, EPI_ISL_874655, EPI_ISL_874657 to 874660, EPI_ISL_874662, EPI_ISL_874664 to 874665, EPI_ISL_874667 to 874669, EPI_ISL_874675, EPI_ISL_874678, EPI_ISL_874681, EPI_ISL_874683 to 874684, EPI_ISL_874686,
EPI_ISL_874688, EPI_ISL_874690 to 874694, EPI_ISL_874696, EPI_ISL_874698, EPI_ISL_874700 to 874702, EPI_ISL_874704 to 874705, EPI_ISL_874707, EPI_ISL_874710 to 874711, EPI_ISL_874713 to 874719, EPI_ISL_874721, EPI_ISL_874727 to 874728, EPI_ISL_874730 to 874732, EPI_ISL_874740 to 874741,
EPI_ISL_874745 to 874746, EPI_ISL_874748 to 874749, EPI_ISL_874751 to 874752, EPI_ISL_874754 to 874755, EPI_ISL_874759 to 874760, EPI_ISL_874762 to 874763, EPI_ISL_874766, EPI_ISL_874771, EPI_ISL_874773, EPI_ISL_874782, EPI_ISL_874784 to 874785, EPI_ISL_874787, EPI_ISL_874789, EPI_ISL_874792 to

874794, EPI_ISL_874796 to 874797, EPI_ISL_874804 to 874805, EPI_ISL_874807, EPI_ISL_874809 to 874812, EPI_ISL_874814, EPI_ISL_874818, EPI_ISL_874821 to 874823, EPI_ISL_874825 to 874826, EPI_ISL_874830, EPI_ISL_874832 to 874834, EPI_ISL_874837 to 874838, EPI_ISL_874841 to 874842, EPI_ISL_874848,
EPI_ISL_874850, EPI_ISL_874852 to 874853, EPI_ISL_874856 to 874857, EPI_ISL_874859 to 874861, EPI_ISL_874864, EPI_ISL_874866 to 874867, EPI_ISL_874870, EPI_ISL_874874 to 874875, EPI_ISL_874880 to 874881, EPI_ISL_874883 to 874885, EPI_ISL_874887 to 874893, EPI_ISL_874899, EPI_ISL_874902,
EPI_ISL_874904, EPI_ISL_874906, EPI_ISL_874910 to 874911, EPI_ISL_874913, EPI_ISL_874915 to 874918, EPI_ISL_874920 to 874921, EPI_ISL_874924, EPI_ISL_874926 to 874927, EPI_ISL_874929 to 874932, EPI_ISL_874934, EPI_ISL_874936, EPI_ISL_874939, EPI_ISL_874946 to 874947, EPI_ISL_874949,
EPI_ISL_874951, EPI_ISL_874957 to 874958, EPI_ISL_874960, EPI_ISL_874964, EPI_ISL_874968 to 874974, EPI_ISL_874976 to 874978, EPI_ISL_874980, EPI_ISL_874982, EPI_ISL_874985 to 874989, EPI_ISL_877770, EPI_ISL_877772 to 877774, EPI_ISL_877776 to 877777, EPI_ISL_877779 to 877783, EPI_ISL_877785,
EPI_ISL_877789, EPI_ISL_877791 to 877795, EPI_ISL_877799 to 877800, EPI_ISL_877803 to 877807, EPI_ISL_877810, EPI_ISL_877812 to 877813, EPI_ISL_877816, EPI_ISL_877820, EPI_ISL_877823 to 877824, EPI_ISL_877828 to 877835, EPI_ISL_877837, EPI_ISL_877839, EPI_ISL_877841, EPI_ISL_877843 to 877846,
EPI_ISL_877849 to 877852, EPI_ISL_877856 to 877858, EPI_ISL_877861 to 877865, EPI_ISL_877867 to 877869, EPI_ISL_877872 to 877874, EPI_ISL_877877 to 877881, EPI_ISL_877883 to 877884, EPI_ISL_877886 to 877887, EPI_ISL_877889 to 877892, EPI_ISL_877894, EPI_ISL_877898 to 877900, EPI_ISL_877902 to
877904, EPI_ISL_877907 to 877910, EPI_ISL_877912 to 877915, EPI_ISL_877917, EPI_ISL_877921, EPI_ISL_877923 to 877926, EPI_ISL_877930 to 877934, EPI_ISL_877936 to 877948, EPI_ISL_877950, EPI_ISL_877953 to 877954, EPI_ISL_877959, EPI_ISL_877962 to 877965, EPI_ISL_877967 to 877968, EPI_ISL_877973 to
877975, EPI_ISL_877978 to 877982, EPI_ISL_877984 to 877985, EPI_ISL_877992 to 877993, EPI_ISL_877996, EPI_ISL_877998, EPI_ISL_878000, EPI_ISL_878002, EPI_ISL_878004 to 878005, EPI_ISL_878007, EPI_ISL_878009, EPI_ISL_878011, EPI_ISL_878013, EPI_ISL_878015, EPI_ISL_878018 to 878025,
EPI_ISL_878028 to 878031, EPI_ISL_878033 to 878035, EPI_ISL_878037 to 878045, EPI_ISL_878048 to 878051, EPI_ISL_878054 to 878057, EPI_ISL_878059, EPI_ISL_878063 to 878065, EPI_ISL_878067 to 878071, EPI_ISL_878073 to 878079, EPI_ISL_878081, EPI_ISL_878083, EPI_ISL_878086 to 878087, EPI_ISL_878090
to 878092, EPI_ISL_878094 to 878099, EPI_ISL_878101 to 878102, EPI_ISL_878106 to 878111, EPI_ISL_878114 to 878115, EPI_ISL_878121 to 878122, EPI_ISL_878126 to 878127, EPI_ISL_878129, EPI_ISL_878134, EPI_ISL_878139 to 878140, EPI_ISL_878142, EPI_ISL_878144 to 878145, EPI_ISL_878148 to 878150,
EPI_ISL_878153, EPI_ISL_878156, EPI_ISL_878158, EPI_ISL_878161, EPI_ISL_878166, EPI_ISL_878171, EPI_ISL_878174, EPI_ISL_878177, EPI_ISL_878179, EPI_ISL_878181, EPI_ISL_878184 to 878185, EPI_ISL_878187 to 878188, EPI_ISL_878195 to 878196, EPI_ISL_878198, EPI_ISL_878200 to 878201,
EPI_ISL_878203 to 878204, EPI_ISL_878206, EPI_ISL_878208, EPI_ISL_878210, EPI_ISL_878213 to 878214, EPI_ISL_878219 to 878220, EPI_ISL_878228, EPI_ISL_878230 to 878231, EPI_ISL_878233, EPI_ISL_878235, EPI_ISL_878244, EPI_ISL_878249, EPI_ISL_878251 to 878252, EPI_ISL_878254, EPI_ISL_878257,
EPI_ISL_878259, EPI_ISL_878261 to 878262, EPI_ISL_878265, EPI_ISL_878269 to 878270, EPI_ISL_878273, EPI_ISL_878275, EPI_ISL_878278, EPI_ISL_878280, EPI_ISL_878283, EPI_ISL_878285 to 878286, EPI_ISL_878288 to 878289, EPI_ISL_878291, EPI_ISL_878296, EPI_ISL_878299, EPI_ISL_878302,
EPI_ISL_878304, EPI_ISL_878307, EPI_ISL_878313, EPI_ISL_878320 to 878321, EPI_ISL_878326, EPI_ISL_878329, EPI_ISL_878331, EPI_ISL_878339, EPI_ISL_878410, EPI_ISL_878415, EPI_ISL_878417, EPI_ISL_878419 to 878420, EPI_ISL_878422, EPI_ISL_878425, EPI_ISL_878427 to 878428, EPI_ISL_878433,
EPI_ISL_878435, EPI_ISL_878438, EPI_ISL_878441, EPI_ISL_878446, EPI_ISL_878451, EPI_ISL_878454, EPI_ISL_878459, EPI_ISL_878461 to 878462, EPI_ISL_878464, EPI_ISL_878466 to 878467, EPI_ISL_878475 to 878476, EPI_ISL_878481 to 878482, EPI_ISL_878486 to 878487, EPI_ISL_878493 to 878494,
EPI_ISL_878497, EPI_ISL_878499, EPI_ISL_878502, EPI_ISL_878504 to 878505, EPI_ISL_878507, EPI_ISL_878510, EPI_ISL_878512, EPI_ISL_878515, EPI_ISL_878517 to 878518, EPI_ISL_878520, EPI_ISL_878523, EPI_ISL_878527 to 878528, EPI_ISL_878530, EPI_ISL_878532, EPI_ISL_878535 to 878536,
EPI_ISL_878538, EPI_ISL_878540, EPI_ISL_878545 to 878546, EPI_ISL_878548, EPI_ISL_878550, EPI_ISL_878553, EPI_ISL_878556, EPI_ISL_878558 to 878559, EPI_ISL_878566, EPI_ISL_878569, EPI_ISL_878572, EPI_ISL_878577 to 878578, EPI_ISL_878580, EPI_ISL_878582, EPI_ISL_878585 to 878586,
EPI_ISL_878590 to 878591, EPI_ISL_878593 to 878594, EPI_ISL_878598 to 878599, EPI_ISL_878601, EPI_ISL_878609 to 878610, EPI_ISL_878612, EPI_ISL_878614 to 878615, EPI_ISL_878620 to 878621, EPI_ISL_878628, EPI_ISL_878630, EPI_ISL_878633 to 878634, EPI_ISL_878641 to 878642, EPI_ISL_878644,
EPI_ISL_878646, EPI_ISL_878650, EPI_ISL_878652, EPI_ISL_878654 to 878655, EPI_ISL_878657, EPI_ISL_878660, EPI_ISL_878662 to 878663, EPI_ISL_878667 to 878668, EPI_ISL_878672 to 878673, EPI_ISL_878675, EPI_ISL_878680 to 878681, EPI_ISL_878683, EPI_ISL_878685 to 878686, EPI_ISL_878688,
EPI_ISL_878696, EPI_ISL_878698, EPI_ISL_878706, EPI_ISL_878709, EPI_ISL_878711 to 878712, EPI_ISL_878715, EPI_ISL_878717, EPI_ISL_878720, EPI_ISL_878722, EPI_ISL_878725, EPI_ISL_878727, EPI_ISL_878730, EPI_ISL_878741, EPI_ISL_878746, EPI_ISL_878777, EPI_ISL_878785, EPI_ISL_878804,
EPI_ISL_878808 to 878809, EPI_ISL_878812, EPI_ISL_878819 to 878820, EPI_ISL_878825, EPI_ISL_878835, EPI_ISL_878840, EPI_ISL_878843 to 878844, EPI_ISL_878853, EPI_ISL_878856, EPI_ISL_878866, EPI_ISL_878868, EPI_ISL_878878, EPI_ISL_878888, EPI_ISL_878909, EPI_ISL_878913 to 878915,
EPI_ISL_878917, EPI_ISL_878926, EPI_ISL_878932, EPI_ISL_878936, EPI_ISL_878946, EPI_ISL_878955, EPI_ISL_878961, EPI_ISL_878965 to 878966, EPI_ISL_878970, EPI_ISL_878979, EPI_ISL_878981, EPI_ISL_878983, EPI_ISL_878993 to 878994, EPI_ISL_879003, EPI_ISL_879007 to 879008, EPI_ISL_879015,
EPI_ISL_879018, EPI_ISL_879026, EPI_ISL_879033, EPI_ISL_879040, EPI_ISL_879061, EPI_ISL_879066, EPI_ISL_879073, EPI_ISL_879083, EPI_ISL_879101, EPI_ISL_879109 to 879110, EPI_ISL_879113, EPI_ISL_879121, EPI_ISL_879125 to 879126, EPI_ISL_879128, EPI_ISL_879130 to 879131, EPI_ISL_879137,
EPI_ISL_879140, EPI_ISL_879144, EPI_ISL_879146, EPI_ISL_879149, EPI_ISL_879151 to 879152, EPI_ISL_879159, EPI_ISL_879161 to 879162, EPI_ISL_879164, EPI_ISL_879167, EPI_ISL_879169, EPI_ISL_879171, EPI_ISL_879187, EPI_ISL_879201, EPI_ISL_879207, EPI_ISL_879212, EPI_ISL_879218 to 879220,
EPI_ISL_879226, EPI_ISL_879229 to 879230, EPI_ISL_879234 to 879235, EPI_ISL_879237, EPI_ISL_879239, EPI_ISL_879241, EPI_ISL_879243, EPI_ISL_879249, EPI_ISL_879252 to 879254, EPI_ISL_879256, EPI_ISL_879258 to 879259, EPI_ISL_879263, EPI_ISL_879268, EPI_ISL_879270 to 879271, EPI_ISL_879274,
EPI_ISL_879277, EPI_ISL_879290, EPI_ISL_879297 to 879300, EPI_ISL_879302, EPI_ISL_879306 to 879307, EPI_ISL_879313, EPI_ISL_879316, EPI_ISL_879329, EPI_ISL_879333, EPI_ISL_879338 to 879342, EPI_ISL_879344 to 879345, EPI_ISL_879348, EPI_ISL_879357 to 879361, EPI_ISL_879367, EPI_ISL_879375,
EPI_ISL_879382, EPI_ISL_879384, EPI_ISL_879386, EPI_ISL_879416, EPI_ISL_879420, EPI_ISL_879433 to 879434, EPI_ISL_879444, EPI_ISL_879452, EPI_ISL_879460, EPI_ISL_879475 to 879476, EPI_ISL_879480, EPI_ISL_879483, EPI_ISL_879486 to 879487, EPI_ISL_879493, EPI_ISL_879497, EPI_ISL_879501,
EPI_ISL_879505, EPI_ISL_879510, EPI_ISL_879515, EPI_ISL_879518, EPI_ISL_879520, EPI_ISL_879534 to 879535, EPI_ISL_879556, EPI_ISL_879563, EPI_ISL_879565, EPI_ISL_879578, EPI_ISL_879589, EPI_ISL_879593, EPI_ISL_879596, EPI_ISL_879602, EPI_ISL_879604, EPI_ISL_879619, EPI_ISL_879627,
EPI_ISL_879629, EPI_ISL_879631, EPI_ISL_879646, EPI_ISL_879649, EPI_ISL_879666, EPI_ISL_879672, EPI_ISL_879682, EPI_ISL_879687, EPI_ISL_879692 to 879693, EPI_ISL_879700, EPI_ISL_879702, EPI_ISL_879737, EPI_ISL_879740, EPI_ISL_879742, EPI_ISL_879750 to 879753, EPI_ISL_879755 to 879756,
EPI_ISL_879758 to 879760, EPI_ISL_879765 to 879766, EPI_ISL_879778, EPI_ISL_879780, EPI_ISL_879785, EPI_ISL_879787, EPI_ISL_879789, EPI_ISL_879793, EPI_ISL_879800, EPI_ISL_879802 to 879803, EPI_ISL_879810, EPI_ISL_879817, EPI_ISL_879819, EPI_ISL_879822, EPI_ISL_879826 to 879827,
EPI_ISL_879829, EPI_ISL_879831, EPI_ISL_879836, EPI_ISL_879838, EPI_ISL_879841 to 879844, EPI_ISL_879846, EPI_ISL_879849, EPI_ISL_879851, EPI_ISL_879854 to 879855, EPI_ISL_879858, EPI_ISL_879863, EPI_ISL_879865 to 879866, EPI_ISL_879868, EPI_ISL_879870, EPI_ISL_879878 to 879879,
EPI_ISL_879882, EPI_ISL_879884, EPI_ISL_879886, EPI_ISL_879888, EPI_ISL_879894 to 879895, EPI_ISL_879897, EPI_ISL_879901, EPI_ISL_879903, EPI_ISL_879907, EPI_ISL_879913 to 879915, EPI_ISL_879918 to 879919, EPI_ISL_879922 to 879923, EPI_ISL_879925, EPI_ISL_879927, EPI_ISL_879933,
EPI_ISL_879948, EPI_ISL_879957, EPI_ISL_879968, EPI_ISL_879970 to 879971, EPI_ISL_879974, EPI_ISL_879976, EPI_ISL_879978 to 879979, EPI_ISL_879981, EPI_ISL_879984, EPI_ISL_879986, EPI_ISL_879988, EPI_ISL_879991, EPI_ISL_879997, EPI_ISL_880007 to 880008, EPI_ISL_880010, EPI_ISL_880016,
EPI_ISL_880018, EPI_ISL_880021, EPI_ISL_880023, EPI_ISL_880028, EPI_ISL_880030 to 880031, EPI_ISL_880036, EPI_ISL_880041, EPI_ISL_880043, EPI_ISL_880055, EPI_ISL_880057, EPI_ISL_880064, EPI_ISL_880067, EPI_ISL_880072, EPI_ISL_880088 to 880089, EPI_ISL_880091, EPI_ISL_880096, EPI_ISL_880099 to
880100, EPI_ISL_880105, EPI_ISL_880108, EPI_ISL_880110, EPI_ISL_880112 to 880113, EPI_ISL_880116, EPI_ISL_880118, EPI_ISL_880124, EPI_ISL_880129, EPI_ISL_880131 to 880132, EPI_ISL_880136 to 880137, EPI_ISL_880139, EPI_ISL_880213 to 880214, EPI_ISL_880216 to 880217, EPI_ISL_880219,
EPI_ISL_880221, EPI_ISL_880230, EPI_ISL_880232, EPI_ISL_880234, EPI_ISL_880237 to 880238, EPI_ISL_880240, EPI_ISL_880242, EPI_ISL_880245, EPI_ISL_880247 to 880248, EPI_ISL_880250, EPI_ISL_880252, EPI_ISL_880257 to 880258, EPI_ISL_880262, EPI_ISL_880265, EPI_ISL_880267, EPI_ISL_880269 to
880270, EPI_ISL_880272 to 880273, EPI_ISL_880275 to 880276, EPI_ISL_880278, EPI_ISL_880281, EPI_ISL_880283, EPI_ISL_880288, EPI_ISL_880291, EPI_ISL_880293, EPI_ISL_880295 to 880296, EPI_ISL_880299, EPI_ISL_880301 to 880302, EPI_ISL_880304, EPI_ISL_880307, EPI_ISL_880309, EPI_ISL_880311 to
880312, EPI_ISL_880314, EPI_ISL_880317, EPI_ISL_880320, EPI_ISL_880322, EPI_ISL_880327, EPI_ISL_880331, EPI_ISL_880335 to 880336, EPI_ISL_880339 to 880340, EPI_ISL_880343, EPI_ISL_880347 to 880348, EPI_ISL_880351, EPI_ISL_880353 to 880358, EPI_ISL_880360 to 880363, EPI_ISL_880366 to 880370,
EPI_ISL_880372 to 880373, EPI_ISL_880376 to 880377, EPI_ISL_880381 to 880383, EPI_ISL_880385, EPI_ISL_880388, EPI_ISL_880391 to 880394, EPI_ISL_880398 to 880400, EPI_ISL_880402 to 880404, EPI_ISL_880406, EPI_ISL_880408 to 880411, EPI_ISL_880413, EPI_ISL_880416, EPI_ISL_880418 to 880419,
EPI_ISL_880422, EPI_ISL_880424, EPI_ISL_880426, EPI_ISL_880430 to 880431, EPI_ISL_880437, EPI_ISL_880441 to 880442, EPI_ISL_880444 to 880447, EPI_ISL_880449 to 880455, EPI_ISL_880457, EPI_ISL_880459, EPI_ISL_880462 to 880465, EPI_ISL_880467, EPI_ISL_880471 to 880472, EPI_ISL_880474 to 880477,
EPI_ISL_880479, EPI_ISL_880481 to 880485, EPI_ISL_880487, EPI_ISL_880489 to 880490, EPI_ISL_880493, EPI_ISL_880495 to 880497, EPI_ISL_880499 to 880502, EPI_ISL_880504, EPI_ISL_880506 to 880507, EPI_ISL_880509 to 880510, EPI_ISL_880512 to 880518, EPI_ISL_880521, EPI_ISL_880525, EPI_ISL_880527 to
880528, EPI_ISL_880532 to 880535, EPI_ISL_880538 to 880539, EPI_ISL_880541 to 880547, EPI_ISL_880550, EPI_ISL_880552 to 880553, EPI_ISL_880555 to 880558, EPI_ISL_880560 to 880562, EPI_ISL_880565 to 880567, EPI_ISL_880570 to 880571, EPI_ISL_880574, EPI_ISL_880578 to 880581, EPI_ISL_880583,
EPI_ISL_880936 to 880940, EPI_ISL_880943, EPI_ISL_880946, EPI_ISL_880948 to 880951, EPI_ISL_880954, EPI_ISL_880956 to 880957, EPI_ISL_880959 to 880961, EPI_ISL_880963 to 880965, EPI_ISL_880967, EPI_ISL_880970 to 880974, EPI_ISL_880976, EPI_ISL_880980 to 880982, EPI_ISL_880984, EPI_ISL_880987 to
880988, EPI_ISL_880991 to 880992, EPI_ISL_880994 to 880996, EPI_ISL_880998, EPI_ISL_881000 to 881001, EPI_ISL_881004, EPI_ISL_881008 to 881013, EPI_ISL_881015 to 881020, EPI_ISL_881022, EPI_ISL_881024 to 881025, EPI_ISL_881028 to 881029, EPI_ISL_881032 to 881033, EPI_ISL_881035 to 881042,
EPI_ISL_881044 to 881046, EPI_ISL_881049 to 881050, EPI_ISL_881055 to 881057, EPI_ISL_881059, EPI_ISL_881061 to 881062, EPI_ISL_881064, EPI_ISL_881067, EPI_ISL_881069 to 881070, EPI_ISL_881072, EPI_ISL_881074 to 881075, EPI_ISL_881077 to 881080, EPI_ISL_881082 to 881083, EPI_ISL_881085 to 881086,
EPI_ISL_881088 to 881103, EPI_ISL_881105, EPI_ISL_881108 to 881109, EPI_ISL_881111 to 881112, EPI_ISL_881116 to 881118, EPI_ISL_881121, EPI_ISL_881123 to 881124, EPI_ISL_881126, EPI_ISL_881128 to 881129, EPI_ISL_881131, EPI_ISL_881133, EPI_ISL_881136, EPI_ISL_881138, EPI_ISL_881140 to 881141,
EPI_ISL_881143, EPI_ISL_881146 to 881148, EPI_ISL_881150 to 881155, EPI_ISL_881160 to 881163, EPI_ISL_881167, EPI_ISL_881169 to 881170, EPI_ISL_881172 to 881176, EPI_ISL_881178 to 881185, EPI_ISL_881187 to 881188, EPI_ISL_881190, EPI_ISL_881193 to 881194, EPI_ISL_881196, EPI_ISL_881201 to 881202,
EPI_ISL_881204 to 881205, EPI_ISL_881208, EPI_ISL_881212 to 881213, EPI_ISL_881215 to 881217, EPI_ISL_881219 to 881220, EPI_ISL_881223 to 881224, EPI_ISL_881226, EPI_ISL_881229 to 881231, EPI_ISL_881233, EPI_ISL_881235, EPI_ISL_881237 to 881240, EPI_ISL_881244 to 881246, EPI_ISL_881250 to 881252,
EPI_ISL_881256 to 881258, EPI_ISL_881262 to 881264, EPI_ISL_881268, EPI_ISL_881271 to 881272, EPI_ISL_881274 to 881276, EPI_ISL_881278, EPI_ISL_881280 to 881285, EPI_ISL_881288 to 881291, EPI_ISL_881293, EPI_ISL_881301 to 881302, EPI_ISL_881304 to 881305, EPI_ISL_881307 to 881311, EPI_ISL_881313
to 881314, EPI_ISL_881317, EPI_ISL_881319 to 881322, EPI_ISL_881324 to 881325, EPI_ISL_881327 to 881329, EPI_ISL_881331 to 881335, EPI_ISL_881337, EPI_ISL_881339 to 881349, EPI_ISL_881351 to 881352, EPI_ISL_881354 to 881357, EPI_ISL_881360 to 881364, EPI_ISL_881366, EPI_ISL_881368 to 881371,
EPI_ISL_881374 to 881376, EPI_ISL_881378 to 881380, EPI_ISL_881382 to 881384, EPI_ISL_881386, EPI_ISL_881388 to 881390, EPI_ISL_881393, EPI_ISL_881396 to 881398, EPI_ISL_881403, EPI_ISL_881405, EPI_ISL_881407, EPI_ISL_881410, EPI_ISL_881412 to 881421, EPI_ISL_881425, EPI_ISL_881428 to 881430,
EPI_ISL_881434, EPI_ISL_881436 to 881437, EPI_ISL_881439, EPI_ISL_881442, EPI_ISL_881444 to 881446, EPI_ISL_881448 to 881450, EPI_ISL_881453, EPI_ISL_881456 to 881459, EPI_ISL_881462, EPI_ISL_881464 to 881465, EPI_ISL_881468 to 881471, EPI_ISL_881473 to 881474, EPI_ISL_881476 to 881477,
EPI_ISL_881481, EPI_ISL_881483 to 881484, EPI_ISL_881486 to 881491, EPI_ISL_881493, EPI_ISL_881495 to 881497, EPI_ISL_881499 to 881500, EPI_ISL_881502 to 881507, EPI_ISL_881509 to 881510, EPI_ISL_881512, EPI_ISL_881518 to 881520, EPI_ISL_881522, EPI_ISL_881524 to 881526, EPI_ISL_881533 to 881537,
EPI_ISL_881539 to 881541, EPI_ISL_881543 to 881547, EPI_ISL_881550 to 881552, EPI_ISL_881554 to 881555, EPI_ISL_881557, EPI_ISL_881559, EPI_ISL_881561, EPI_ISL_881563 to 881566, EPI_ISL_881568, EPI_ISL_881570 to 881571, EPI_ISL_881573 to 881576, EPI_ISL_881578, EPI_ISL_881581, EPI_ISL_881584,
EPI_ISL_881587 to 881588, EPI_ISL_881590, EPI_ISL_881592 to 881595, EPI_ISL_881597 to 881600, EPI_ISL_881602, EPI_ISL_881605 to 881607, EPI_ISL_881610 to 881615, EPI_ISL_881623, EPI_ISL_881626 to 881627, EPI_ISL_881629, EPI_ISL_881631 to 881633, EPI_ISL_881636, EPI_ISL_881638, EPI_ISL_881640,
EPI_ISL_881643, EPI_ISL_881646, EPI_ISL_881648 to 881649, EPI_ISL_881651 to 881654, EPI_ISL_881656 to 881658, EPI_ISL_881660 to 881661, EPI_ISL_881664, EPI_ISL_881667, EPI_ISL_881669, EPI_ISL_881672, EPI_ISL_881676, EPI_ISL_881686 to 881688, EPI_ISL_881690, EPI_ISL_881692, EPI_ISL_881694 to
881697, EPI_ISL_881700 to 881701, EPI_ISL_881704 to 881705, EPI_ISL_881709 to 881710, EPI_ISL_881714 to 881715, EPI_ISL_881717, EPI_ISL_881721, EPI_ISL_881727, EPI_ISL_881729 to 881736, EPI_ISL_881743 to 881744, EPI_ISL_881746, EPI_ISL_881749 to 881755, EPI_ISL_881762 to 881763, EPI_ISL_881765 to
881767, EPI_ISL_881769, EPI_ISL_881771 to 881773, EPI_ISL_881775, EPI_ISL_881777 to 881778, EPI_ISL_881782 to 881783, EPI_ISL_881785 to 881786, EPI_ISL_881790 to 881797, EPI_ISL_881799 to 881801, EPI_ISL_881803 to 881804, EPI_ISL_881806 to 881807, EPI_ISL_881809 to 881810, EPI_ISL_881812 to
881814, EPI_ISL_881818 to 881821, EPI_ISL_881823, EPI_ISL_881825, EPI_ISL_881828 to 881829, EPI_ISL_881832, EPI_ISL_881835, EPI_ISL_881838, EPI_ISL_881841 to 881852, EPI_ISL_881854 to 881856, EPI_ISL_881859, EPI_ISL_881861, EPI_ISL_881863 to 881871, EPI_ISL_881874 to 881876, EPI_ISL_881880 to
881881, EPI_ISL_881884 to 881891, EPI_ISL_881894, EPI_ISL_881898 to 881900, EPI_ISL_881902 to 881909, EPI_ISL_881911, EPI_ISL_881914, EPI_ISL_881918 to 881921, EPI_ISL_881923, EPI_ISL_881926, EPI_ISL_881928, EPI_ISL_881931 to 881935, EPI_ISL_881943 to 881944, EPI_ISL_881948, EPI_ISL_881951,
EPI_ISL_881954 to 881955, EPI_ISL_881958 to 881960, EPI_ISL_881964, EPI_ISL_881966 to 881968, EPI_ISL_881971, EPI_ISL_881976, EPI_ISL_881981 to 881982, EPI_ISL_881985, EPI_ISL_881987 to 881988, EPI_ISL_881990, EPI_ISL_881993, EPI_ISL_881997 to 881998, EPI_ISL_882000 to 882001, EPI_ISL_882004 to
882005, EPI_ISL_882007 to 882011, EPI_ISL_882013, EPI_ISL_882015, EPI_ISL_882020 to 882021, EPI_ISL_882023, EPI_ISL_882025, EPI_ISL_882029, EPI_ISL_882033 to 882035, EPI_ISL_882037, EPI_ISL_882040, EPI_ISL_882046 to 882047, EPI_ISL_882050 to 882051, EPI_ISL_882053, EPI_ISL_882056 to 882057,
EPI_ISL_882059, EPI_ISL_882062, EPI_ISL_882066 to 882067, EPI_ISL_882069, EPI_ISL_882072, EPI_ISL_882074, EPI_ISL_882078, EPI_ISL_882080, EPI_ISL_882084, EPI_ISL_882086, EPI_ISL_882088, EPI_ISL_882091, EPI_ISL_882094 to 882095, EPI_ISL_882097, EPI_ISL_882099, EPI_ISL_882101 to 882104,
EPI_ISL_882106, EPI_ISL_882108 to 882109, EPI_ISL_882114, EPI_ISL_882118, EPI_ISL_882120 to 882121, EPI_ISL_882126 to 882127, EPI_ISL_882129 to 882131, EPI_ISL_882135 to 882136, EPI_ISL_882138 to 882140, EPI_ISL_882144, EPI_ISL_882146 to 882147, EPI_ISL_882149, EPI_ISL_882152 to 882154,
EPI_ISL_882156, EPI_ISL_882158 to 882159, EPI_ISL_882163, EPI_ISL_882167, EPI_ISL_882171, EPI_ISL_882174, EPI_ISL_882177 to 882178, EPI_ISL_882182 to 882183, EPI_ISL_882186 to 882187, EPI_ISL_882190, EPI_ISL_882193 to 882195, EPI_ISL_882200, EPI_ISL_882202 to 882206, EPI_ISL_882208,
EPI_ISL_882210 to 882211, EPI_ISL_882213, EPI_ISL_882216 to 882218, EPI_ISL_882223 to 882224, EPI_ISL_882226 to 882228, EPI_ISL_882230 to 882231, EPI_ISL_882234 to 882235, EPI_ISL_882240, EPI_ISL_882242 to 882243, EPI_ISL_882245, EPI_ISL_882247, EPI_ISL_882250 to 882251, EPI_ISL_882253,
EPI_ISL_882258 to 882259, EPI_ISL_882263, EPI_ISL_882266 to 882268, EPI_ISL_882270 to 882278, EPI_ISL_882280 to 882282, EPI_ISL_882284 to 882287, EPI_ISL_882289 to 882290, EPI_ISL_882293 to 882294, EPI_ISL_882296 to 882297, EPI_ISL_882300, EPI_ISL_882302 to 882307, EPI_ISL_882309, EPI_ISL_882311
to 882314, EPI_ISL_882316 to 882322, EPI_ISL_882324, EPI_ISL_882326 to 882329, EPI_ISL_882331 to 882333, EPI_ISL_882337 to 882341, EPI_ISL_882343 to 882346, EPI_ISL_882348 to 882350, EPI_ISL_882352, EPI_ISL_882356, EPI_ISL_882358 to 882359, EPI_ISL_882361 to 882365, EPI_ISL_882367 to 882371,
EPI_ISL_882373 to 882374, EPI_ISL_882379 to 882381, EPI_ISL_882384 to 882386, EPI_ISL_882388 to 882394, EPI_ISL_882396 to 882404, EPI_ISL_882408 to 882409, EPI_ISL_882411, EPI_ISL_882413, EPI_ISL_882415 to 882418, EPI_ISL_882420 to 882422, EPI_ISL_882427 to 882428, EPI_ISL_882431 to 882432,
EPI_ISL_882435 to 882438, EPI_ISL_882440, EPI_ISL_882442, EPI_ISL_882444 to 882445, EPI_ISL_882447, EPI_ISL_882451 to 882452, EPI_ISL_882454, EPI_ISL_882456 to 882463, EPI_ISL_882465, EPI_ISL_882468 to 882469, EPI_ISL_882471 to 882483, EPI_ISL_882485 to 882489, EPI_ISL_882491, EPI_ISL_882493 to
882504, EPI_ISL_882506, EPI_ISL_882508 to 882510, EPI_ISL_882512 to 882513, EPI_ISL_882515, EPI_ISL_882519 to 882525, EPI_ISL_882527 to 882529, EPI_ISL_882531 to 882539, EPI_ISL_882542, EPI_ISL_882544, EPI_ISL_882546, EPI_ISL_882548 to 882552, EPI_ISL_882554 to 882555, EPI_ISL_882557 to 882562,
EPI_ISL_882565 to 882566, EPI_ISL_882569, EPI_ISL_882571 to 882574, EPI_ISL_882576, EPI_ISL_882578, EPI_ISL_882580 to 882584, EPI_ISL_882586 to 882589, EPI_ISL_882592 to 882598, EPI_ISL_882600 to 882604, EPI_ISL_882608, EPI_ISL_885155, EPI_ISL_885157 to 885158, EPI_ISL_885160 to 885164,
EPI_ISL_885167 to 885168, EPI_ISL_885170 to 885171, EPI_ISL_885175, EPI_ISL_885177 to 885180, EPI_ISL_885183 to 885184, EPI_ISL_885186 to 885187, EPI_ISL_885189 to 885192, EPI_ISL_885194 to 885197, EPI_ISL_885200 to 885203, EPI_ISL_885206 to 885208, EPI_ISL_885210, EPI_ISL_885213 to 885215,
EPI_ISL_885217 to 885218, EPI_ISL_885220 to 885222, EPI_ISL_885224, EPI_ISL_885226 to 885228, EPI_ISL_885230 to 885232, EPI_ISL_885235 to 885236, EPI_ISL_885240 to 885241, EPI_ISL_885243 to 885246, EPI_ISL_885248, EPI_ISL_885251 to 885260, EPI_ISL_885262, EPI_ISL_885265 to 885271, EPI_ISL_885274
to 885276, EPI_ISL_885280 to 885282, EPI_ISL_885287 to 885290, EPI_ISL_885292 to 885297, EPI_ISL_885300 to 885303, EPI_ISL_885305, EPI_ISL_885307 to 885311, EPI_ISL_885313 to 885319, EPI_ISL_885321, EPI_ISL_885324 to 885327, EPI_ISL_885330 to 885331, EPI_ISL_885333, EPI_ISL_885337 to 885340,
EPI_ISL_885343 to 885347, EPI_ISL_885349, EPI_ISL_885351 to 885357, EPI_ISL_885359, EPI_ISL_885361 to 885362, EPI_ISL_885364 to 885366, EPI_ISL_885368 to 885370, EPI_ISL_885372 to 885376, EPI_ISL_885378 to 885380, EPI_ISL_885382, EPI_ISL_885385 to 885387, EPI_ISL_885390 to 885391, EPI_ISL_885393,
EPI_ISL_885395, EPI_ISL_885398, EPI_ISL_885400 to 885401, EPI_ISL_885403, EPI_ISL_885405 to 885407, EPI_ISL_885410 to 885412, EPI_ISL_885416 to 885418, EPI_ISL_885420 to 885423, EPI_ISL_885425 to 885427, EPI_ISL_885429 to 885432, EPI_ISL_885435 to 885439, EPI_ISL_885441 to 885442, EPI_ISL_885444
to 885445, EPI_ISL_885447 to 885448, EPI_ISL_885450 to 885453, EPI_ISL_885456 to 885459, EPI_ISL_885461, EPI_ISL_885463 to 885464, EPI_ISL_885466 to 885467, EPI_ISL_885469, EPI_ISL_885471 to 885472, EPI_ISL_885474 to 885476, EPI_ISL_885478, EPI_ISL_885480, EPI_ISL_885483, EPI_ISL_885485 to 885486,
EPI_ISL_885489 to 885490, EPI_ISL_885492 to 885494, EPI_ISL_885497, EPI_ISL_885499 to 885501, EPI_ISL_885503, EPI_ISL_885508, EPI_ISL_885511, EPI_ISL_885513, EPI_ISL_885515 to 885516, EPI_ISL_885519, EPI_ISL_885521, EPI_ISL_885524 to 885525, EPI_ISL_885529, EPI_ISL_885531 to 885533,
EPI_ISL_885538, EPI_ISL_885541 to 885544, EPI_ISL_885547, EPI_ISL_885549, EPI_ISL_885552 to 885553, EPI_ISL_885555 to 885560, EPI_ISL_885563, EPI_ISL_885565, EPI_ISL_885567, EPI_ISL_885571, EPI_ISL_885573, EPI_ISL_885575 to 885576, EPI_ISL_885581 to 885584, EPI_ISL_885586, EPI_ISL_885588 to
885589, EPI_ISL_885591, EPI_ISL_885593 to 885594, EPI_ISL_885596 to 885597, EPI_ISL_885599 to 885601, EPI_ISL_885604, EPI_ISL_885606 to 885607, EPI_ISL_885609 to 885610, EPI_ISL_885616, EPI_ISL_885618, EPI_ISL_885620 to 885622, EPI_ISL_885625 to 885627, EPI_ISL_885630, EPI_ISL_885636 to 885641,
EPI_ISL_885646 to 885647, EPI_ISL_885649 to 885650, EPI_ISL_885652 to 885654, EPI_ISL_885661 to 885662, EPI_ISL_885664 to 885665, EPI_ISL_885667 to 885668, EPI_ISL_885672 to 885676, EPI_ISL_885679, EPI_ISL_885683 to 885685, EPI_ISL_885689 to 885690, EPI_ISL_885692, EPI_ISL_885695, EPI_ISL_885698
to 885699, EPI_ISL_885701 to 885703, EPI_ISL_885706 to 885707, EPI_ISL_885710 to 885712, EPI_ISL_885714 to 885716, EPI_ISL_885718 to 885724, EPI_ISL_885729, EPI_ISL_885731 to 885737, EPI_ISL_885740, EPI_ISL_885743, EPI_ISL_885746 to 885750, EPI_ISL_885752 to 885753, EPI_ISL_885755 to 885756,
EPI_ISL_885758 to 885759, EPI_ISL_885764, EPI_ISL_885766 to 885768, EPI_ISL_885771 to 885772, EPI_ISL_885774 to 885776, EPI_ISL_885779, EPI_ISL_885781, EPI_ISL_885784, EPI_ISL_885788, EPI_ISL_885790 to 885792, EPI_ISL_885794 to 885797, EPI_ISL_885800, EPI_ISL_885804 to 885806, EPI_ISL_885809 to
885811, EPI_ISL_885813 to 885814, EPI_ISL_885817 to 885820, EPI_ISL_885822, EPI_ISL_885824 to 885826, EPI_ISL_885828 to 885834, EPI_ISL_885836 to 885838, EPI_ISL_885840 to 885844, EPI_ISL_885846, EPI_ISL_885848 to 885852, EPI_ISL_885854 to 885857, EPI_ISL_885859 to 885864, EPI_ISL_885866,
EPI_ISL_885869 to 885872, EPI_ISL_885874, EPI_ISL_885880 to 885884, EPI_ISL_885886 to 885887, EPI_ISL_885891 to 885892, EPI_ISL_885895, EPI_ISL_885900 to 885903, EPI_ISL_885905 to 885906, EPI_ISL_885908, EPI_ISL_885910, EPI_ISL_885912 to 885916, EPI_ISL_885918, EPI_ISL_885920 to 885923,
EPI_ISL_885925, EPI_ISL_885927, EPI_ISL_885930, EPI_ISL_885932, EPI_ISL_885934 to 885937, EPI_ISL_885939 to 885940, EPI_ISL_885943 to 885944, EPI_ISL_885946 to 885948, EPI_ISL_885950 to 885954, EPI_ISL_885956 to 885958, EPI_ISL_885960, EPI_ISL_885962, EPI_ISL_885964 to 885967, EPI_ISL_885969 to

885973, EPI_ISL_885976 to 885977, EPI_ISL_885979 to 885980, EPI_ISL_885982 to 885985, EPI_ISL_885988, EPI_ISL_885991 to 885997, EPI_ISL_886000 to 886004, EPI_ISL_886006, EPI_ISL_886010, EPI_ISL_886013 to 886014, EPI_ISL_886017 to 886018, EPI_ISL_886024, EPI_ISL_886027, EPI_ISL_886030 to 886033, EPI_ISL_886035, EPI_ISL_886037 to 886040, EPI_ISL_886042 to 886043, EPI_ISL_886051 to 886052, EPI_ISL_886054 to 886057, EPI_ISL_886060 to 886064, EPI_ISL_886067 to 886070, EPI_ISL_886073, EPI_ISL_886075 to 886078, EPI_ISL_886081, EPI_ISL_886083 to 886084, EPI_ISL_886088 to 886090, EPI_ISL_886093, EPI_ISL_886095 to 886096, EPI_ISL_886098, EPI_ISL_886101 to 886105, EPI_ISL_886107 to 886109, EPI_ISL_886111, EPI_ISL_886113, EPI_ISL_886115, EPI_ISL_886120 to 886126, EPI_ISL_886128 to 886129, EPI_ISL_886131 to 886133, EPI_ISL_886135 to 886136, EPI_ISL_886138, EPI_ISL_886140, EPI_ISL_886142, EPI_ISL_886145, EPI_ISL_886147, EPI_ISL_886149, EPI_ISL_886152 to 886153, EPI_ISL_886156, EPI_ISL_891278, EPI_ISL_891280, EPI_ISL_891285, EPI_ISL_891295, EPI_ISL_891305, EPI_ISL_891311, EPI_ISL_891317 to 891318, EPI_ISL_891323, EPI_ISL_891326, EPI_ISL_891334, EPI_ISL_891337, EPI_ISL_891340, EPI_ISL_891349, EPI_ISL_891352, EPI_ISL_891357, EPI_ISL_891364, EPI_ISL_891374, EPI_ISL_891376 to 891377, EPI_ISL_891384 to 891386, EPI_ISL_891392, EPI_ISL_891400, EPI_ISL_891411, EPI_ISL_891416 to 891417, EPI_ISL_891421, EPI_ISL_891423, EPI_ISL_891444, EPI_ISL_891448, EPI_ISL_891451, EPI_ISL_891454 to 891455, EPI_ISL_891458 to 891459, EPI_ISL_891484, EPI_ISL_891489, EPI_ISL_891496, EPI_ISL_891499, EPI_ISL_891503, EPI_ISL_891511, EPI_ISL_891514, EPI_ISL_891531, EPI_ISL_891536, EPI_ISL_891544 to 891545, EPI_ISL_891547, EPI_ISL_891555, EPI_ISL_891560, EPI_ISL_891563, EPI_ISL_891569, EPI_ISL_891572, EPI_ISL_891582, EPI_ISL_891586, EPI_ISL_891596, EPI_ISL_891599 to 891600, EPI_ISL_891602, EPI_ISL_891615, EPI_ISL_891623, EPI_ISL_891630, EPI_ISL_891634, EPI_ISL_891640, EPI_ISL_891643, EPI_ISL_891647, EPI_ISL_891650, EPI_ISL_891659, EPI_ISL_891661 to 891662, EPI_ISL_891672, EPI_ISL_891677, EPI_ISL_891681, EPI_ISL_891687, EPI_ISL_891704, EPI_ISL_891710, EPI_ISL_891713 to 891714, EPI_ISL_891719, EPI_ISL_891723 to 891724, EPI_ISL_891726, EPI_ISL_891729, EPI_ISL_891735 to 891736, EPI_ISL_891738, EPI_ISL_891742, EPI_ISL_891753, EPI_ISL_891757, EPI_ISL_891760, EPI_ISL_891762, EPI_ISL_891765, EPI_ISL_891774 to 891777, EPI_ISL_891780, EPI_ISL_891786 to 891789, EPI_ISL_891794, EPI_ISL_891804, EPI_ISL_891811, EPI_ISL_891813, EPI_ISL_891822, EPI_ISL_891824, EPI_ISL_891839, EPI_ISL_891843, EPI_ISL_891847, EPI_ISL_891850, EPI_ISL_891858, EPI_ISL_891860, EPI_ISL_891864, EPI_ISL_891866 to 891867, EPI_ISL_891878, EPI_ISL_891887, EPI_ISL_891895, EPI_ISL_891901, EPI_ISL_891903 to 891904, EPI_ISL_891906, EPI_ISL_891908 to 891909, EPI_ISL_891918 to 891919, EPI_ISL_891921, EPI_ISL_891924 to 891925, EPI_ISL_891928, EPI_ISL_891931, EPI_ISL_891935, EPI_ISL_891943, EPI_ISL_891945, EPI_ISL_891947 to 891948, EPI_ISL_891952 to 891953, EPI_ISL_891961, EPI_ISL_891963, EPI_ISL_891965 to 891966, EPI_ISL_891973, EPI_ISL_891981, EPI_ISL_891985, EPI_ISL_891992 to 891994, EPI_ISL_891999, EPI_ISL_892006 to 892007, EPI_ISL_892016, EPI_ISL_892024, EPI_ISL_892028 to 892029, EPI_ISL_892034 to 892035, EPI_ISL_892037, EPI_ISL_892040, EPI_ISL_892042 to 892043, EPI_ISL_892047 to 892048, EPI_ISL_892051, EPI_ISL_892055, EPI_ISL_892059 to 892060, EPI_ISL_892077, EPI_ISL_892099, EPI_ISL_892104, EPI_ISL_892107, EPI_ISL_892109 to 892110, EPI_ISL_892118, EPI_ISL_892121, EPI_ISL_892126, EPI_ISL_892131, EPI_ISL_892134, EPI_ISL_892139, EPI_ISL_892145, EPI_ISL_892147, EPI_ISL_892149 to 892150, EPI_ISL_892156, EPI_ISL_892164, EPI_ISL_892170, EPI_ISL_892184, EPI_ISL_892190 to 892191, EPI_ISL_892203 to 892204, EPI_ISL_907372 to 907374, EPI_ISL_907377, EPI_ISL_907380, EPI_ISL_907382, EPI_ISL_907387 to 907389, EPI_ISL_907391, EPI_ISL_907399 to 907400, EPI_ISL_907404, EPI_ISL_907406 to 907407, EPI_ISL_907409 to 907413, EPI_ISL_907417, EPI_ISL_907422, EPI_ISL_907427, EPI_ISL_907429, EPI_ISL_907432, EPI_ISL_907437 to 907438, EPI_ISL_907440, EPI_ISL_907444, EPI_ISL_907447, EPI_ISL_907449, EPI_ISL_907452, EPI_ISL_907457 to 907460, EPI_ISL_907463, EPI_ISL_907466 to 907468, EPI_ISL_907471, EPI_ISL_907477, EPI_ISL_907479, EPI_ISL_907482, EPI_ISL_907484 to 907485, EPI_ISL_907489, EPI_ISL_907491, EPI_ISL_907493, EPI_ISL_907496 to 907497, EPI_ISL_907500, EPI_ISL_907503, EPI_ISL_907510 to 907512, EPI_ISL_907516 to 907518, EPI_ISL_907522 to 907523, EPI_ISL_907532 to 907533, EPI_ISL_907537, EPI_ISL_907543, EPI_ISL_907546, EPI_ISL_907551, EPI_ISL_907557, EPI_ISL_907559, EPI_ISL_907561, EPI_ISL_907568, EPI_ISL_907573, EPI_ISL_907576, EPI_ISL_907578, EPI_ISL_907582 to 907584, EPI_ISL_907589 to 907590, EPI_ISL_907594, EPI_ISL_907596, EPI_ISL_907598, EPI_ISL_907601, EPI_ISL_907607, EPI_ISL_907610 to 907612, EPI_ISL_907615 to 907616, EPI_ISL_907618, EPI_ISL_907621 to 907623, EPI_ISL_907626, EPI_ISL_907630 to 907631, EPI_ISL_907640 to 907642, EPI_ISL_907645, EPI_ISL_907647, EPI_ISL_907651 to 907652, EPI_ISL_907654, EPI_ISL_907659, EPI_ISL_907661, EPI_ISL_907664, EPI_ISL_907673, EPI_ISL_907676, EPI_ISL_907684, EPI_ISL_907686, EPI_ISL_907689, EPI_ISL_907693, EPI_ISL_907695 to 907697, EPI_ISL_907701, EPI_ISL_907710 to 907714, EPI_ISL_907717 to 907718, EPI_ISL_907720, EPI_ISL_907724, EPI_ISL_907726 to 907727, EPI_ISL_907729 to 907730, EPI_ISL_907732 to 907734, EPI_ISL_907737, EPI_ISL_907740, EPI_ISL_907743 to 907744, EPI_ISL_907746, EPI_ISL_907748, EPI_ISL_907751 to 907754, EPI_ISL_907759, EPI_ISL_907762, EPI_ISL_907764, EPI_ISL_907768 to 907770, EPI_ISL_907775, EPI_ISL_907779 to 907780, EPI_ISL_907783 to 907785, EPI_ISL_907791, EPI_ISL_907798 to 907799, EPI_ISL_907804, EPI_ISL_907808, EPI_ISL_907810, EPI_ISL_907816, EPI_ISL_907819, EPI_ISL_907822, EPI_ISL_907825, EPI_ISL_907828, EPI_ISL_907830 to 907831, EPI_ISL_907836, EPI_ISL_907841 to 907843, EPI_ISL_907845 to 907846, EPI_ISL_907849, EPI_ISL_907856 to 907857, EPI_ISL_907859, EPI_ISL_907866, EPI_ISL_907869 to 907871, EPI_ISL_907873, EPI_ISL_907876, EPI_ISL_907883 to 907884, EPI_ISL_907888, EPI_ISL_907892, EPI_ISL_907896, EPI_ISL_907901, EPI_ISL_907905 to 907909, EPI_ISL_907913, EPI_ISL_907916, EPI_ISL_907918 to 907919, EPI_ISL_907922, EPI_ISL_907924, EPI_ISL_907927, EPI_ISL_907929 to 907930, EPI_ISL_907932 to 907933, EPI_ISL_907935, EPI_ISL_907937, EPI_ISL_907939, EPI_ISL_907947, EPI_ISL_907950 to 907952, EPI_ISL_907955, EPI_ISL_907957, EPI_ISL_907959, EPI_ISL_907961 to 907965, EPI_ISL_907968, EPI_ISL_907971, EPI_ISL_907978, EPI_ISL_907980, EPI_ISL_907985 to 907986, EPI_ISL_907990 to 907992, EPI_ISL_907996 to 907997, EPI_ISL_907999 to 908000, EPI_ISL_908005 to 908006, EPI_ISL_908012, EPI_ISL_908015, EPI_ISL_908019 to 908020, EPI_ISL_908023, EPI_ISL_908032, EPI_ISL_908268, EPI_ISL_908270 to 908271, EPI_ISL_908273, EPI_ISL_908277 to 908280, EPI_ISL_908285 to 908288, EPI_ISL_908292, EPI_ISL_908294, EPI_ISL_908296 to 908297, EPI_ISL_908304 to 908306, EPI_ISL_908309 to 908312, EPI_ISL_908314, EPI_ISL_908319, EPI_ISL_908322, EPI_ISL_908324 to 908325, EPI_ISL_908327, EPI_ISL_908330, EPI_ISL_908336, EPI_ISL_908339, EPI_ISL_908341, EPI_ISL_908343, EPI_ISL_908345 to 908347, EPI_ISL_908350 to 908352, EPI_ISL_908354, EPI_ISL_908356, EPI_ISL_908358, EPI_ISL_908361 to 908362, EPI_ISL_908364, EPI_ISL_908367, EPI_ISL_908369 to 908373, EPI_ISL_908379, EPI_ISL_908381, EPI_ISL_908385 to 908386, EPI_ISL_908388, EPI_ISL_908391 to 908392, EPI_ISL_908395 to 908401, EPI_ISL_908404, EPI_ISL_908406 to 908407, EPI_ISL_908409 to 908410, EPI_ISL_908414 to 908415, EPI_ISL_908418, EPI_ISL_908420, EPI_ISL_908424, EPI_ISL_908426 to 908427, EPI_ISL_908430, EPI_ISL_908434, EPI_ISL_908437 to 908438, EPI_ISL_908441, EPI_ISL_908445 to 908446, EPI_ISL_908448, EPI_ISL_908456 to 908457, EPI_ISL_908459 to 908461, EPI_ISL_908466 to 908469, EPI_ISL_908472 to 908474, EPI_ISL_908477 to 908478, EPI_ISL_908480, EPI_ISL_908482 to 908485, EPI_ISL_908490, EPI_ISL_908492, EPI_ISL_908497 to 908499, EPI_ISL_908501 to 908503, EPI_ISL_908505 to 908506, EPI_ISL_908511, EPI_ISL_908513, EPI_ISL_908515, EPI_ISL_908517 to 908518, EPI_ISL_908520, EPI_ISL_908524, EPI_ISL_908527 to 908529, EPI_ISL_908531 to 908533, EPI_ISL_908535, EPI_ISL_908539 to 908541, EPI_ISL_908544 to 908546, EPI_ISL_908552, EPI_ISL_908561 to 908563, EPI_ISL_908565 to 908566, EPI_ISL_908569 to 908571, EPI_ISL_908577 to 908578, EPI_ISL_908580, EPI_ISL_908583 to 908587, EPI_ISL_908590, EPI_ISL_908593 to 908594, EPI_ISL_908596, EPI_ISL_909260, EPI_ISL_909285, EPI_ISL_909292, EPI_ISL_909294, EPI_ISL_909297, EPI_ISL_909311 to 909312, EPI_ISL_909318, EPI_ISL_909322, EPI_ISL_909328, EPI_ISL_909334, EPI_ISL_909339, EPI_ISL_909341, EPI_ISL_909343 to 909344, EPI_ISL_909358 to 909359, EPI_ISL_909364, EPI_ISL_909366 to 909368, EPI_ISL_909370, EPI_ISL_909372 to 909373, EPI_ISL_909380, EPI_ISL_909388 to 909389, EPI_ISL_909397, EPI_ISL_909409, EPI_ISL_909414, EPI_ISL_909416, EPI_ISL_909425 to 909426, EPI_ISL_909446 to 909447, EPI_ISL_909453, EPI_ISL_909456 to 909457, EPI_ISL_909471, EPI_ISL_909476, EPI_ISL_909482, EPI_ISL_909487 to 909488, EPI_ISL_909503 to 909504, EPI_ISL_909536, EPI_ISL_909539, EPI_ISL_909546, EPI_ISL_909552 to 909553, EPI_ISL_909564, EPI_ISL_909572 to 909573, EPI_ISL_909575, EPI_ISL_909578, EPI_ISL_909580 to 909581, EPI_ISL_909595, EPI_ISL_909597, EPI_ISL_909599 to 909610, EPI_ISL_909612 to 909613, EPI_ISL_909615 to 909616, EPI_ISL_909618 to 909621, EPI_ISL_909623 to 909626, EPI_ISL_909628 to 909631, EPI_ISL_909633, EPI_ISL_909635 to 909636, EPI_ISL_909638, EPI_ISL_909640, EPI_ISL_909643 to 909645, EPI_ISL_909647 to 909650, EPI_ISL_912215 to 912218, EPI_ISL_912220 to 912221, EPI_ISL_912223, EPI_ISL_912226, EPI_ISL_915537, EPI_ISL_915659, EPI_ISL_915683, EPI_ISL_915746, EPI_ISL_915775, EPI_ISL_915822, EPI_ISL_915858, EPI_ISL_915916, EPI_ISL_915933, EPI_ISL_915976, EPI_ISL_916016, EPI_ISL_916184, EPI_ISL_916186, EPI_ISL_916195, EPI_ISL_916197 to 916199, EPI_ISL_916204, EPI_ISL_916217 to 916221, EPI_ISL_916223 to 916224, EPI_ISL_916230, EPI_ISL_916235, EPI_ISL_916251, EPI_ISL_916253, EPI_ISL_916255, EPI_ISL_916257, EPI_ISL_916265, EPI_ISL_916267, EPI_ISL_916272, EPI_ISL_916282, EPI_ISL_916286, EPI_ISL_916289, EPI_ISL_916293 to 916294, EPI_ISL_916300, EPI_ISL_916302, EPI_ISL_916311, EPI_ISL_916317, EPI_ISL_916319, EPI_ISL_916326, EPI_ISL_916330, EPI_ISL_916353, EPI_ISL_916356, EPI_ISL_916386 to 916387, EPI_ISL_916389, EPI_ISL_916393, EPI_ISL_916395 to 916396, EPI_ISL_916398, EPI_ISL_916402, EPI_ISL_916422 to 916424, EPI_ISL_916429, EPI_ISL_916448, EPI_ISL_916452, EPI_ISL_916465 to 916466, EPI_ISL_916468 to 916469, EPI_ISL_916471, EPI_ISL_916474, EPI_ISL_916483, EPI_ISL_916488, EPI_ISL_916490, EPI_ISL_916498, EPI_ISL_916501, EPI_ISL_916622 to 916624, EPI_ISL_916627, EPI_ISL_916629 to 916631, EPI_ISL_916634, EPI_ISL_916637 to 916638, EPI_ISL_916640 to 916641, EPI_ISL_916643, EPI_ISL_916647, EPI_ISL_916650 to 916654, EPI_ISL_916657, EPI_ISL_916659, EPI_ISL_916663, EPI_ISL_916666, EPI_ISL_916669, EPI_ISL_916671, EPI_ISL_916674, EPI_ISL_916676 to 916677, EPI_ISL_916681, EPI_ISL_916685 to 916687, EPI_ISL_916689 to 916691, EPI_ISL_916693, EPI_ISL_916695, EPI_ISL_916697, EPI_ISL_916699, EPI_ISL_916701, EPI_ISL_916706 to 916708, EPI_ISL_916715, EPI_ISL_916718 to 916720, EPI_ISL_916722, EPI_ISL_916724, EPI_ISL_916731 to 916734, EPI_ISL_916737, EPI_ISL_916739 to 916740, EPI_ISL_916742 to 916743, EPI_ISL_916746 to 916753, EPI_ISL_916756 to 916757, EPI_ISL_916759 to 916760, EPI_ISL_916762, EPI_ISL_916764, EPI_ISL_916766 to 916769, EPI_ISL_916772 to 916773, EPI_ISL_916777 to 916778, EPI_ISL_916780, EPI_ISL_916783, EPI_ISL_916786 to 916788, EPI_ISL_916796 to 916797, EPI_ISL_916799, EPI_ISL_916801, EPI_ISL_916803 to 916806, EPI_ISL_916808, EPI_ISL_916813, EPI_ISL_916816 to 916818, EPI_ISL_916823, EPI_ISL_916826, EPI_ISL_916828 to 916831, EPI_ISL_916836, EPI_ISL_916839, EPI_ISL_916843 to 916844, EPI_ISL_916846, EPI_ISL_916848 to 916849, EPI_ISL_916851 to 916852, EPI_ISL_916856 to 916857, EPI_ISL_916859 to 916860, EPI_ISL_916863, EPI_ISL_916868, EPI_ISL_916870 to 916873, EPI_ISL_916875 to 916876, EPI_ISL_916878, EPI_ISL_916880, EPI_ISL_916885 to 916886, EPI_ISL_916889 to 916890, EPI_ISL_916892 to 916893, EPI_ISL_916901 to 916903, EPI_ISL_916906, EPI_ISL_916910, EPI_ISL_916913 to 916914, EPI_ISL_916919 to 916920, EPI_ISL_916924 to 916927, EPI_ISL_916929 to 916934, EPI_ISL_916936, EPI_ISL_916939, EPI_ISL_916941, EPI_ISL_916944, EPI_ISL_916950, EPI_ISL_916952, EPI_ISL_916955, EPI_ISL_916957 to 916960, EPI_ISL_916962 to 916966, EPI_ISL_916975, EPI_ISL_916982, EPI_ISL_916992 to 916994, EPI_ISL_916996 to 916997, EPI_ISL_917000 to 917001, EPI_ISL_917003, EPI_ISL_917007, EPI_ISL_917010 to 917011, EPI_ISL_917020, EPI_ISL_917023, EPI_ISL_917027 to 917028, EPI_ISL_917035, EPI_ISL_917037, EPI_ISL_917040, EPI_ISL_917043 to 917044, EPI_ISL_917046, EPI_ISL_917048, EPI_ISL_917052, EPI_ISL_917057 to 917058, EPI_ISL_917064 to 917065, EPI_ISL_917068, EPI_ISL_917072 to 917073, EPI_ISL_917087, EPI_ISL_917090, EPI_ISL_917094 to 917095, EPI_ISL_917097 to 917098, EPI_ISL_917101 to 917103, EPI_ISL_917106, EPI_ISL_917113, EPI_ISL_917115, EPI_ISL_917117 to 917118, EPI_ISL_917121 to 917123, EPI_ISL_917130, EPI_ISL_917132, EPI_ISL_917134 to 917135, EPI_ISL_917138, EPI_ISL_917142, EPI_ISL_917147, EPI_ISL_917150, EPI_ISL_917152 to 917154, EPI_ISL_917158, EPI_ISL_917162, EPI_ISL_917164 to 917165, EPI_ISL_917168 to 917175, EPI_ISL_917177, EPI_ISL_917179 to 917185, EPI_ISL_917187 to 917190, EPI_ISL_917192 to 917193, EPI_ISL_917197 to 917198, EPI_ISL_917200, EPI_ISL_917202 to 917205, EPI_ISL_917207 to 917208, EPI_ISL_917212, EPI_ISL_917214, EPI_ISL_917217 to 917222, EPI_ISL_917224, EPI_ISL_917227, EPI_ISL_917230 to 917231, EPI_ISL_917233 to 917234, EPI_ISL_917236 to 917237, EPI_ISL_917239, EPI_ISL_917241 to 917243, EPI_ISL_917246 to 917250, EPI_ISL_917254 to 917255, EPI_ISL_917261 to 917266, EPI_ISL_917268 to 917273, EPI_ISL_917276, EPI_ISL_917279 to 917280, EPI_ISL_917282 to 917287, EPI_ISL_917290, EPI_ISL_917292 to 917299, EPI_ISL_917301, EPI_ISL_917303, EPI_ISL_917305 to 917306, EPI_ISL_917308 to 917309, EPI_ISL_917311 to 917312, EPI_ISL_917315 to 917318, EPI_ISL_917320 to 917321, EPI_ISL_917323, EPI_ISL_917325, EPI_ISL_917327, EPI_ISL_917330, EPI_ISL_917334 to 917335, EPI_ISL_917337 to 917339, EPI_ISL_917343, EPI_ISL_917348 to 917353, EPI_ISL_917356, EPI_ISL_917358 to 917359, EPI_ISL_917362 to 917364, EPI_ISL_917366 to 917368, EPI_ISL_917370, EPI_ISL_917373 to 917378, EPI_ISL_917381, EPI_ISL_917383 to 917389, EPI_ISL_917392, EPI_ISL_917395 to 917398, EPI_ISL_917400, EPI_ISL_917404, EPI_ISL_917406 to 917411, EPI_ISL_917413, EPI_ISL_917417 to 917418, EPI_ISL_917421 to 917426, EPI_ISL_917428, EPI_ISL_917433 to 917434, EPI_ISL_917437 to 917442, EPI_ISL_917444, EPI_ISL_917446 to 917448, EPI_ISL_917450 to 917452, EPI_ISL_917454, EPI_ISL_917456 to 917460, EPI_ISL_917462 to 917465, EPI_ISL_917468 to 917475, EPI_ISL_917478 to 917479, EPI_ISL_917481 to 917482, EPI_ISL_917485 to 917486, EPI_ISL_917488 to 917501, EPI_ISL_917503 to 917505, EPI_ISL_917507 to 917511, EPI_ISL_917513, EPI_ISL_917517 to 917518, EPI_ISL_917521 to 917525, EPI_ISL_917527 to 917534, EPI_ISL_917536 to 917538, EPI_ISL_917540 to 917543, EPI_ISL_917545 to 917546, EPI_ISL_917548, EPI_ISL_917552, EPI_ISL_917556 to 917561, EPI_ISL_917564 to 917566, EPI_ISL_917568, EPI_ISL_917570 to 917574, EPI_ISL_917576 to 917579, EPI_ISL_917581 to 917582, EPI_ISL_917585 to 917586, EPI_ISL_917589 to 917591, EPI_ISL_917593, EPI_ISL_917595, EPI_ISL_917598, EPI_ISL_917600, EPI_ISL_917602 to 917606, EPI_ISL_917608 to 917611, EPI_ISL_917613, EPI_ISL_917617 to 917620, EPI_ISL_917622 to 917623, EPI_ISL_917625 to 917626, EPI_ISL_917628, EPI_ISL_917632 to 917634, EPI_ISL_917636 to 917638, EPI_ISL_917641 to 917650, EPI_ISL_917655, EPI_ISL_917658 to 917660, EPI_ISL_917662 to 917665, EPI_ISL_917667, EPI_ISL_917669, EPI_ISL_917671 to 917683, EPI_ISL_917685 to 917686, EPI_ISL_917688 to 917689, EPI_ISL_917692, EPI_ISL_917694, EPI_ISL_917696 to 917697, EPI_ISL_917699 to 917701, EPI_ISL_917703 to 917710, EPI_ISL_917712 to 917713, EPI_ISL_917716 to 917719, EPI_ISL_917721 to 917724, EPI_ISL_917726 to 917730, EPI_ISL_917733 to 917734, EPI_ISL_917737, EPI_ISL_917739, EPI_ISL_917741, EPI_ISL_917744 to 917750, EPI_ISL_917753 to 917754, EPI_ISL_917756 to 917757, EPI_ISL_917759 to 917760, EPI_ISL_917764 to 917766, EPI_ISL_917771 to 917772, EPI_ISL_917774 to 917778, EPI_ISL_917780, EPI_ISL_917783 to 917786, EPI_ISL_917788 to 917789, EPI_ISL_917792, EPI_ISL_917796 to 917797, EPI_ISL_917801, EPI_ISL_917803 to 917807, EPI_ISL_917810 to 917812, EPI_ISL_917814 to 917816, EPI_ISL_917818 to 917822, EPI_ISL_917825, EPI_ISL_917828 to 917830, EPI_ISL_917833 to 917835, EPI_ISL_917837, EPI_ISL_917840 to 917841, EPI_ISL_917843, EPI_ISL_918060 to 918061, EPI_ISL_931586, EPI_ISL_931588 to 931594, EPI_ISL_931596 to 931599, EPI_ISL_931602 to 931608, EPI_ISL_931611 to 931614, EPI_ISL_931617 to 931618, EPI_ISL_931620 to 931623, EPI_ISL_931625 to 931629, EPI_ISL_931632, EPI_ISL_931634 to 931635, EPI_ISL_931637, EPI_ISL_931639 to 931640, EPI_ISL_931642 to 931652, EPI_ISL_931655, EPI_ISL_931658, EPI_ISL_931662, EPI_ISL_931665, EPI_ISL_931667 to 931668, EPI_ISL_931670, EPI_ISL_931673 to 931678, EPI_ISL_931680 to 931684, EPI_ISL_931687 to 931689, EPI_ISL_931691 to 931698, EPI_ISL_931702 to 931709, EPI_ISL_931711 to 931714, EPI_ISL_931716, EPI_ISL_931718 to 931719, EPI_ISL_931722 to 931728, EPI_ISL_931730 to 931731, EPI_ISL_931733 to 931736, EPI_ISL_931738 to 931740, EPI_ISL_931742 to 931744, EPI_ISL_931746 to 931747, EPI_ISL_931750, EPI_ISL_931752, EPI_ISL_931754 to 931758, EPI_ISL_931760 to 931763, EPI_ISL_931765, EPI_ISL_931767 to 931770, EPI_ISL_931773 to 931776, EPI_ISL_931778 to 931786, EPI_ISL_931788, EPI_ISL_931790 to 931791, EPI_ISL_931793 to 931794, EPI_ISL_931796 to 931798, EPI_ISL_931800 to 931806, EPI_ISL_931808 to 931809, EPI_ISL_931811 to 931815, EPI_ISL_931817, EPI_ISL_931819 to 931820, EPI_ISL_931823 to 931824, EPI_ISL_931828 to 931835, EPI_ISL_931838, EPI_ISL_931840 to 931850, EPI_ISL_931852 to 931854, EPI_ISL_931857, EPI_ISL_931860, EPI_ISL_931862 to 931863, EPI_ISL_931865, EPI_ISL_931867 to 931871, EPI_ISL_931873 to 931874, EPI_ISL_931877, EPI_ISL_931879 to 931881, EPI_ISL_931883 to 931885, EPI_ISL_931887 to 931888, EPI_ISL_931890 to 931893, EPI_ISL_931895, EPI_ISL_931897 to 931898, EPI_ISL_931900 to 931902, EPI_ISL_931905 to 931907, EPI_ISL_931912 to 931913, EPI_ISL_931917 to 931921, EPI_ISL_931923 to 931926, EPI_ISL_931928, EPI_ISL_931931 to 931933, EPI_ISL_931935, EPI_ISL_931938 to 931941, EPI_ISL_931943, EPI_ISL_931945 to 931946, EPI_ISL_931948, EPI_ISL_931950 to 931953, EPI_ISL_931961 to 931969, EPI_ISL_931972 to 931975, EPI_ISL_931977 to 931979, EPI_ISL_931981, EPI_ISL_931984, EPI_ISL_931987 to 931991, EPI_ISL_931993 to 931995, EPI_ISL_931999 to 932001, EPI_ISL_932007 to 932014, EPI_ISL_932016 to 932020, EPI_ISL_932023 to 932025, EPI_ISL_932027, EPI_ISL_932029, EPI_ISL_932031 to 932032, EPI_ISL_932034 to 932035, EPI_ISL_932037 to 932038, EPI_ISL_932040, EPI_ISL_932042 to 932043, EPI_ISL_932046 to 932050, EPI_ISL_932052 to 932053, EPI_ISL_932056 to 932057, EPI_ISL_932060 to 932065, EPI_ISL_932067, EPI_ISL_932070 to 932071, EPI_ISL_932074 to 932079, EPI_ISL_932081, EPI_ISL_932083 to 932085, EPI_ISL_932087, EPI_ISL_932089, EPI_ISL_932092 to 932093, EPI_ISL_932095, EPI_ISL_932097, EPI_ISL_932100 to 932103, EPI_ISL_932105 to 932107, EPI_ISL_932110, EPI_ISL_932112 to 932114, EPI_ISL_932116, EPI_ISL_932118, EPI_ISL_932122, EPI_ISL_932127 to 932134, EPI_ISL_932136, EPI_ISL_932139 to 932143, EPI_ISL_932145 to 932146, EPI_ISL_932148 to 932153, EPI_ISL_932155 to 932157, EPI_ISL_932161 to 932162, EPI_ISL_932164 to 932171, EPI_ISL_932173, EPI_ISL_932175 to 932178, EPI_ISL_932181 to 932182, EPI_ISL_932186, EPI_ISL_932189, EPI_ISL_932191 to 932192, EPI_ISL_932194 to 932198, EPI_ISL_932200 to 932204, EPI_ISL_932206 to 932212, EPI_ISL_932214 to 932218, EPI_ISL_932221 to 932222, EPI_ISL_932225 to 932227, EPI_ISL_932229 to 932231, EPI_ISL_932238 to 932241, EPI_ISL_932244, EPI_ISL_932247, EPI_ISL_932250, EPI_ISL_932261, EPI_ISL_932264, EPI_ISL_932268, EPI_ISL_932273, EPI_ISL_932285, EPI_ISL_932290, EPI_ISL_932297, EPI_ISL_932300, EPI_ISL_932325, EPI_ISL_932345, EPI_ISL_932348, EPI_ISL_932357, EPI_ISL_932371, EPI_ISL_932376, EPI_ISL_932388, EPI_ISL_932425, EPI_ISL_932433, EPI_ISL_932440, EPI_ISL_932444, EPI_ISL_932453, EPI_ISL_932455, EPI_ISL_932457 to 932458, EPI_ISL_932473, EPI_ISL_932475, EPI_ISL_932489, EPI_ISL_932502 to 932503, EPI_ISL_932512, EPI_ISL_932515, EPI_ISL_932517, EPI_ISL_932522, EPI_ISL_932530, EPI_ISL_932543, EPI_ISL_932552, EPI_ISL_932620, EPI_ISL_932640, EPI_ISL_932661, EPI_ISL_932668, EPI_ISL_932670, EPI_ISL_932673, EPI_ISL_932690, EPI_ISL_932725, EPI_ISL_932734, EPI_ISL_932743, EPI_ISL_932749, EPI_ISL_932753, EPI_ISL_932760, EPI_ISL_932791, EPI_ISL_932800, EPI_ISL_932835, EPI_ISL_932840, EPI_ISL_932862, EPI_ISL_932867 to 932868, EPI_ISL_932878, EPI_ISL_932881 to 932882, EPI_ISL_932889 to 932891, EPI_ISL_932900 to 932902, EPI_ISL_932911 to 932912, EPI_ISL_932922 to 932924, EPI_ISL_932927, EPI_ISL_932935, EPI_ISL_932940, EPI_ISL_932942 to 932943, EPI_ISL_932947, EPI_ISL_932952, EPI_ISL_932956, EPI_ISL_932964, EPI_ISL_932973, EPI_ISL_932975, EPI_ISL_932979, EPI_ISL_932984, EPI_ISL_932988, EPI_ISL_932990, EPI_ISL_932993, EPI_ISL_933001, EPI_ISL_933003, EPI_ISL_933013, EPI_ISL_933017, EPI_ISL_933024 to 933026, EPI_ISL_933031, EPI_ISL_933041, EPI_ISL_933049, EPI_ISL_933062, EPI_ISL_933064, EPI_ISL_933066, EPI_ISL_933084, EPI_ISL_933090, EPI_ISL_933092, EPI_ISL_933094, EPI_ISL_933098, EPI_ISL_933102, EPI_ISL_933104, EPI_ISL_933106, EPI_ISL_933112, EPI_ISL_937545, EPI_ISL_937547 to 937554, EPI_ISL_937558, EPI_ISL_937560, EPI_ISL_937562 to 937564, EPI_ISL_937566 to 937571, EPI_ISL_937573, EPI_ISL_937575, EPI_ISL_937577 to 937581, EPI_ISL_937583 to 937584, EPI_ISL_937586 to 937588, EPI_ISL_937591 to 937594, EPI_ISL_937596 to 937597, EPI_ISL_937599 to 937604, EPI_ISL_937606 to 937607, EPI_ISL_937611, EPI_ISL_937616 to 937619, EPI_ISL_937621 to 937624, EPI_ISL_937627, EPI_ISL_937629, EPI_ISL_937631 to 937636, EPI_ISL_937638 to 937639, EPI_ISL_937641, EPI_ISL_937643 to 937647, EPI_ISL_937649 to 937652, EPI_ISL_937657, EPI_ISL_937660, EPI_ISL_937662 to 937665, EPI_ISL_937667 to 937670, EPI_ISL_937674, EPI_ISL_937676, EPI_ISL_937678, EPI_ISL_937680 to 937681, EPI_ISL_937684 to 937686, EPI_ISL_937688 to 937691, EPI_ISL_937693 to 937697, EPI_ISL_937699 to 937704, EPI_ISL_937706 to 937707, EPI_ISL_937709, EPI_ISL_937712 to 937713, EPI_ISL_937716 to 937717, EPI_ISL_937719 to 937720, EPI_ISL_937723 to 937724, EPI_ISL_937726, EPI_ISL_937728 to 937729, EPI_ISL_937732, EPI_ISL_937735 to 937738, EPI_ISL_937740 to 937742, EPI_ISL_937744 to 937748, EPI_ISL_937751 to 937754, EPI_ISL_937756 to 937758, EPI_ISL_937760 to 937765, EPI_ISL_937767 to 937768, EPI_ISL_937772, EPI_ISL_937774 to 937777, EPI_ISL_937779 to 937781, EPI_ISL_937783 to 937789, EPI_ISL_937791 to 937792, EPI_ISL_937794, EPI_ISL_937796, EPI_ISL_937798 to 937805, EPI_ISL_937808 to 937816, EPI_ISL_937818 to 937819, EPI_ISL_937821, EPI_ISL_937824 to 937825, EPI_ISL_937827 to 937829, EPI_ISL_937837 to 937842, EPI_ISL_937844 to 937845, EPI_ISL_937849 to 937850, EPI_ISL_937852 to 937853, EPI_ISL_937855 to 937857, EPI_ISL_937861 to 937864, EPI_ISL_937867, EPI_ISL_937869, EPI_ISL_937871 to 937873, EPI_ISL_937877 to 937883, EPI_ISL_938935 to 938939, EPI_ISL_938943, EPI_ISL_939608, EPI_ISL_945201, EPI_ISL_945306, EPI_ISL_945375 to 945380, EPI_ISL_945382 to 945383, EPI_ISL_945385 to 945389, EPI_ISL_945391 to 945397, EPI_ISL_945399 to 945400, EPI_ISL_945405 to 945407, EPI_ISL_945411, EPI_ISL_945413 to 945414, EPI_ISL_945416, EPI_ISL_945419 to 945423, EPI_ISL_945425 to 945426, EPI_ISL_945428 to 945429, EPI_ISL_945431 to 945435, EPI_ISL_945438 to 945451, EPI_ISL_945453 to 945454, EPI_ISL_945457 to 945460, EPI_ISL_945463 to 945470, EPI_ISL_945472 to 945474, EPI_ISL_945476, EPI_ISL_945478 to 945480, EPI_ISL_945483 to 945487, EPI_ISL_945493 to 945494, EPI_ISL_945496, EPI_ISL_945498 to 945499, EPI_ISL_945503, EPI_ISL_945505, EPI_ISL_945508 to 945509, EPI_ISL_945511, EPI_ISL_945513 to 945514, EPI_ISL_945516, EPI_ISL_945518 to 945519, EPI_ISL_945521 to 945522, EPI_ISL_945524 to 945525, EPI_ISL_945528 to 945529, EPI_ISL_945531 to 945533, EPI_ISL_945535 to 945537, EPI_ISL_945539, EPI_ISL_945541, EPI_ISL_945543 to 945544, EPI_ISL_945549, EPI_ISL_945552, EPI_ISL_945554 to 945555, EPI_ISL_945557 to 945559, EPI_ISL_945562 to 945563, EPI_ISL_945566 to 945575, EPI_ISL_945577 to 945588, EPI_ISL_945591 to 945592, EPI_ISL_945594 to 945595, EPI_ISL_945597 to 945607, EPI_ISL_945609 to 945612, EPI_ISL_945615, EPI_ISL_945617 to 945618, EPI_ISL_945621, EPI_ISL_945623 to 945625, EPI_ISL_945627, EPI_ISL_945629 to 945632, EPI_ISL_945634 to 945636, EPI_ISL_945638 to 945642, EPI_ISL_945645 to 945650, EPI_ISL_945652 to 945655, EPI_ISL_945657 to 945659, EPI_ISL_945661 to 945664, EPI_ISL_945667, EPI_ISL_945670 to 945677, EPI_ISL_945679, EPI_ISL_945681 to 945683, EPI_ISL_945685, EPI_ISL_945687, EPI_ISL_945691, EPI_ISL_945693 to 945698, EPI_ISL_945700, EPI_ISL_945703 to 945704, EPI_ISL_945709 to 945710, EPI_ISL_945712, EPI_ISL_945714, EPI_ISL_946421 to 946424,

EPI_ISL_946426 to 946428, EPI_ISL_946431 to 946433, EPI_ISL_946435 to 946436, EPI_ISL_946438, EPI_ISL_946441 to 946442, EPI_ISL_946445 to 946446, EPI_ISL_946449 to 946450, EPI_ISL_946452 to 946453, EPI_ISL_946455 to 946456, EPI_ISL_946458, EPI_ISL_946460, EPI_ISL_946462 to 946463, EPI_ISL_946465 to 946466, EPI_ISL_946468 to 946469, EPI_ISL_946471, EPI_ISL_946473 to 946474, EPI_ISL_946476, EPI_ISL_946478, EPI_ISL_946480 to 946481, EPI_ISL_946484, EPI_ISL_946487 to 946489, EPI_ISL_946491 to 946492, EPI_ISL_946494, EPI_ISL_946496 to 946498, EPI_ISL_946500, EPI_ISL_946503 to 946505, EPI_ISL_946507 to 946513, EPI_ISL_946516 to 946517, EPI_ISL_946519 to 946521, EPI_ISL_946523 to 946524, EPI_ISL_946526, EPI_ISL_946528, EPI_ISL_946530 to 946531, EPI_ISL_946533 to 946535, EPI_ISL_946537 to 946538, EPI_ISL_946540 to 946541, EPI_ISL_946544, EPI_ISL_946546 to 946558, EPI_ISL_946561 to 946564, EPI_ISL_946566, EPI_ISL_946568 to 946573, EPI_ISL_946575 to 946580, EPI_ISL_946582 to 946584, EPI_ISL_946586 to 946596, EPI_ISL_946598 to 946601, EPI_ISL_946603 to 946605, EPI_ISL_946607, EPI_ISL_946609 to 946614, EPI_ISL_946618 to 946622, EPI_ISL_946624 to 946628, EPI_ISL_946630 to 946631, EPI_ISL_946633, EPI_ISL_946635 to 946637, EPI_ISL_946640, EPI_ISL_946642, EPI_ISL_946644, EPI_ISL_946646, EPI_ISL_946652 to 946655, EPI_ISL_946657, EPI_ISL_946659, EPI_ISL_946661 to 946665, EPI_ISL_946668 to 946669, EPI_ISL_946673 to 946674, EPI_ISL_946677 to 946679, EPI_ISL_946681 to 946697, EPI_ISL_946700, EPI_ISL_946702 to 946703, EPI_ISL_946705 to 946709, EPI_ISL_946711 to 946713, EPI_ISL_946715 to 946719, EPI_ISL_946721 to 946724, EPI_ISL_946726 to 946729, EPI_ISL_946734, EPI_ISL_946736, EPI_ISL_946739 to 946745, EPI_ISL_946747 to 946748, EPI_ISL_946752 to 946753, EPI_ISL_946755, EPI_ISL_946759 to 946760, EPI_ISL_946762 to 946766, EPI_ISL_946768 to 946769, EPI_ISL_946772 to 946779, EPI_ISL_946782 to 946784, EPI_ISL_946786, EPI_ISL_946788 to 946791, EPI_ISL_946794 to 946798, EPI_ISL_946800 to 946801, EPI_ISL_946805 to 946806, EPI_ISL_946808 to 946810, EPI_ISL_946814, EPI_ISL_946818 to 946819, EPI_ISL_946821 to 946823, EPI_ISL_946825 to 946828, EPI_ISL_946831, EPI_ISL_946833, EPI_ISL_946835, EPI_ISL_946837, EPI_ISL_946839 to 946840, EPI_ISL_946842 to 946843, EPI_ISL_946845, EPI_ISL_946847, EPI_ISL_946849, EPI_ISL_946851 to 946859, EPI_ISL_946861, EPI_ISL_946863, EPI_ISL_946865, EPI_ISL_946868 to 946870, EPI_ISL_946872 to 946874, EPI_ISL_946876, EPI_ISL_946878 to 946879, EPI_ISL_946881 to 946882, EPI_ISL_946884 to 946887, EPI_ISL_946891 to 946895, EPI_ISL_946897 to 946899, EPI_ISL_946901 to 946903, EPI_ISL_946906 to 946907, EPI_ISL_946910 to 946912, EPI_ISL_946914 to 946917, EPI_ISL_946921 to 946924, EPI_ISL_946926 to 946927, EPI_ISL_946929 to 946933, EPI_ISL_946935 to 946937, EPI_ISL_946940 to 946943, EPI_ISL_946949 to 946951, EPI_ISL_946953 to 946954, EPI_ISL_946957, EPI_ISL_946959, EPI_ISL_946961 to 946962, EPI_ISL_946964, EPI_ISL_946966 to 946968, EPI_ISL_946970 to 946972, EPI_ISL_946976 to 946978, EPI_ISL_946980 to 946983, EPI_ISL_946985 to 946989, EPI_ISL_946994, EPI_ISL_946996 to 947000, EPI_ISL_947002 to 947003, EPI_ISL_947005, EPI_ISL_947007, EPI_ISL_947009, EPI_ISL_947011 to 947013, EPI_ISL_947015, EPI_ISL_947021 to 947027, EPI_ISL_947030 to 947032, EPI_ISL_947034 to 947038, EPI_ISL_947040, EPI_ISL_947043 to 947044, EPI_ISL_947047 to 947052, EPI_ISL_947054 to 947055, EPI_ISL_947057, EPI_ISL_947060, EPI_ISL_947062, EPI_ISL_947064 to 947067, EPI_ISL_947069, EPI_ISL_947071, EPI_ISL_947073 to 947084, EPI_ISL_947086 to 947089, EPI_ISL_947091 to 947100, EPI_ISL_947102 to 947106, EPI_ISL_947406 to 947410, EPI_ISL_947412 to 947415, EPI_ISL_947417 to 947421, EPI_ISL_947423, EPI_ISL_947425 to 947428, EPI_ISL_947430 to 947437, EPI_ISL_947439 to 947440, EPI_ISL_947446 to 947448, EPI_ISL_947450, EPI_ISL_947456 to 947461, EPI_ISL_947464 to 947466, EPI_ISL_947469 to 947472, EPI_ISL_947474 to 947476, EPI_ISL_947479 to 947482, EPI_ISL_947485 to 947488, EPI_ISL_947490 to 947491, EPI_ISL_947493, EPI_ISL_947496 to 947500, EPI_ISL_947502 to 947504, EPI_ISL_947506 to 947507, EPI_ISL_947510 to 947513, EPI_ISL_947515, EPI_ISL_947517, EPI_ISL_947520 to 947522, EPI_ISL_947524 to 947525, EPI_ISL_947527 to 947528, EPI_ISL_947530 to 947533, EPI_ISL_947535, EPI_ISL_947539 to 947543, EPI_ISL_947545, EPI_ISL_947547 to 947549, EPI_ISL_947551 to 947556, EPI_ISL_947561 to 947562, EPI_ISL_947564, EPI_ISL_947566 to 947567, EPI_ISL_947571 to 947574, EPI_ISL_947576 to 947577, EPI_ISL_947579 to 947580, EPI_ISL_947582, EPI_ISL_947584 to 947586, EPI_ISL_947589 to 947591, EPI_ISL_947593 to 947594, EPI_ISL_947596 to 947599, EPI_ISL_947601 to 947602, EPI_ISL_947604, EPI_ISL_947606 to 947608, EPI_ISL_947610 to 947611, EPI_ISL_947613, EPI_ISL_947615 to 947618, EPI_ISL_947620 to 947621, EPI_ISL_947624 to 947625, EPI_ISL_947627 to 947629, EPI_ISL_947631 to 947632, EPI_ISL_947634, EPI_ISL_947636 to 947638, EPI_ISL_947641 to 947642, EPI_ISL_947645, EPI_ISL_947648, EPI_ISL_947651, EPI_ISL_947653, EPI_ISL_947656, EPI_ISL_947658, EPI_ISL_947660, EPI_ISL_947662, EPI_ISL_947664 to 947667, EPI_ISL_947671, EPI_ISL_947675 to 947677, EPI_ISL_947679, EPI_ISL_947681, EPI_ISL_947684 to 947685, EPI_ISL_947687 to 947689, EPI_ISL_947695 to 947696, EPI_ISL_947699 to 947701, EPI_ISL_947703, EPI_ISL_947705, EPI_ISL_947708 to 947714, EPI_ISL_947718 to 947719, EPI_ISL_947721, EPI_ISL_947723 to 947724, EPI_ISL_947726 to 947728, EPI_ISL_947730, EPI_ISL_947733, EPI_ISL_947735 to 947737, EPI_ISL_947740, EPI_ISL_947742 to 947744, EPI_ISL_947748 to 947750, EPI_ISL_947754, EPI_ISL_947756 to 947758, EPI_ISL_947760 to 947767, EPI_ISL_947770, EPI_ISL_947772 to 947773, EPI_ISL_947777 to 947779, EPI_ISL_947782 to 947783, EPI_ISL_947787 to 947789, EPI_ISL_947791 to 947794, EPI_ISL_947797 to 947799, EPI_ISL_947801 to 947802, EPI_ISL_947804 to 947808, EPI_ISL_947810, EPI_ISL_947812 to 947814, EPI_ISL_947816 to 947818, EPI_ISL_947820 to 947821, EPI_ISL_947827 to 947830, EPI_ISL_947833 to 947835, EPI_ISL_947837 to 947838, EPI_ISL_947840, EPI_ISL_947842, EPI_ISL_947844, EPI_ISL_947846 to 947850, EPI_ISL_947852 to 947855, EPI_ISL_947857, EPI_ISL_947859 to 947862, EPI_ISL_947864 to 947866, EPI_ISL_947868 to 947870, EPI_ISL_947874 to 947878, EPI_ISL_947880, EPI_ISL_947882, EPI_ISL_947886 to 947889, EPI_ISL_947891, EPI_ISL_947893 to 947903, EPI_ISL_947906, EPI_ISL_947908, EPI_ISL_947910 to 947911, EPI_ISL_947913 to 947914, EPI_ISL_947917 to 947918, EPI_ISL_947920 to 947922, EPI_ISL_947925, EPI_ISL_947927, EPI_ISL_947929 to 947932, EPI_ISL_947934 to 947941, EPI_ISL_947944 to 947949, EPI_ISL_947952 to 947953, EPI_ISL_947955, EPI_ISL_947958, EPI_ISL_947960 to 947961, EPI_ISL_947963 to 947966, EPI_ISL_947969 to 947970, EPI_ISL_947972, EPI_ISL_947975, EPI_ISL_947977 to 947982, EPI_ISL_947984 to 947986, EPI_ISL_947989 to 947991, EPI_ISL_947995 to 947999, EPI_ISL_948001 to 948006, EPI_ISL_948011 to 948014, EPI_ISL_948016 to 948020, EPI_ISL_948022 to 948024, EPI_ISL_948026, EPI_ISL_948029 to 948030, EPI_ISL_948032, EPI_ISL_948034 to 948035, EPI_ISL_948037 to 948041, EPI_ISL_948044, EPI_ISL_948046 to 948047, EPI_ISL_948051 to 948052, EPI_ISL_948054, EPI_ISL_948056 to 948058, EPI_ISL_948061 to 948064, EPI_ISL_948066 to 948067, EPI_ISL_948069 to 948070, EPI_ISL_948072, EPI_ISL_948074 to 948078, EPI_ISL_948080, EPI_ISL_948082 to 948084, EPI_ISL_948087 to 948091, EPI_ISL_948093 to 948096, EPI_ISL_948099, EPI_ISL_948449, EPI_ISL_948451, EPI_ISL_948453 to 948459, EPI_ISL_948461 to 948462, EPI_ISL_948467 to 948468, EPI_ISL_948470 to 948471, EPI_ISL_948475 to 948478, EPI_ISL_948480, EPI_ISL_948483 to 948489, EPI_ISL_948491 to 948494, EPI_ISL_948497 to 948499, EPI_ISL_948501 to 948502, EPI_ISL_948505, EPI_ISL_948509, EPI_ISL_948511 to 948515, EPI_ISL_948519 to 948520, EPI_ISL_948522 to 948524, EPI_ISL_948526, EPI_ISL_948529 to 948531, EPI_ISL_948533, EPI_ISL_948535, EPI_ISL_948538 to 948539, EPI_ISL_948542, EPI_ISL_948544 to 948547, EPI_ISL_948549 to 948552, EPI_ISL_948554 to 948557, EPI_ISL_948559 to 948567, EPI_ISL_948570, EPI_ISL_948572 to 948573, EPI_ISL_948575, EPI_ISL_948578 to 948580, EPI_ISL_948582, EPI_ISL_948584 to 948591, EPI_ISL_948593 to 948594, EPI_ISL_948597, EPI_ISL_948599 to 948604, EPI_ISL_948606, EPI_ISL_948608, EPI_ISL_948611 to 948613, EPI_ISL_948617 to 948618, EPI_ISL_948620 to 948623, EPI_ISL_948625 to 948629, EPI_ISL_948631, EPI_ISL_948635, EPI_ISL_948637, EPI_ISL_948639 to 948641, EPI_ISL_948643, EPI_ISL_948645 to 948651, EPI_ISL_948655, EPI_ISL_948658 to 948659, EPI_ISL_948661, EPI_ISL_948663 to 948666, EPI_ISL_948668 to 948669, EPI_ISL_948671 to 948672, EPI_ISL_948674, EPI_ISL_948679 to 948681, EPI_ISL_948684, EPI_ISL_948686 to 948691, EPI_ISL_948694 to 948696, EPI_ISL_948699 to 948700, EPI_ISL_948702, EPI_ISL_948705 to 948707, EPI_ISL_948709, EPI_ISL_948711 to 948715, EPI_ISL_948717, EPI_ISL_948721 to 948724, EPI_ISL_948726, EPI_ISL_948728 to 948729, EPI_ISL_948732, EPI_ISL_948734 to 948739, EPI_ISL_948741 to 948742, EPI_ISL_948744 to 948746, EPI_ISL_948749 to 948755, EPI_ISL_948757, EPI_ISL_948762, EPI_ISL_948766, EPI_ISL_948770 to 948771, EPI_ISL_948774 to 948775, EPI_ISL_948777 to 948778, EPI_ISL_948782 to 948783, EPI_ISL_948785, EPI_ISL_956422, EPI_ISL_956424 to 956426, EPI_ISL_956428 to 956434, EPI_ISL_956437 to 956438, EPI_ISL_956440 to 956441, EPI_ISL_956444 to 956447, EPI_ISL_956451 to 956456, EPI_ISL_956458 to 956460, EPI_ISL_956462 to 956463, EPI_ISL_956466 to 956467, EPI_ISL_956469 to 956476, EPI_ISL_956478 to 956482, EPI_ISL_956485 to 956489, EPI_ISL_956491, EPI_ISL_956493 to 956495, EPI_ISL_956497 to 956504, EPI_ISL_956506, EPI_ISL_956508 to 956513, EPI_ISL_956516 to 956523, EPI_ISL_956525 to 956528, EPI_ISL_956530 to 956531, EPI_ISL_956536 to 956539, EPI_ISL_956541 to 956542, EPI_ISL_956544 to 956546, EPI_ISL_956548, EPI_ISL_956550 to 956562, EPI_ISL_956564, EPI_ISL_956568, EPI_ISL_956570 to 956574, EPI_ISL_956577 to 956581, EPI_ISL_956584 to 956594, EPI_ISL_956596, EPI_ISL_956598 to 956606, EPI_ISL_956608 to 956611, EPI_ISL_956613 to 956617, EPI_ISL_956619 to 956621, EPI_ISL_956625, EPI_ISL_956628, EPI_ISL_956630 to 956632, EPI_ISL_956635 to 956638, EPI_ISL_956640 to 956642, EPI_ISL_956644 to 956645, EPI_ISL_956648 to 956656, EPI_ISL_956658, EPI_ISL_956660 to 956661, EPI_ISL_956664, EPI_ISL_956666 to 956667, EPI_ISL_956669 to 956674, EPI_ISL_956678 to 956683, EPI_ISL_956685 to 956688, EPI_ISL_956690 to 956692, EPI_ISL_956694 to 956695, EPI_ISL_956698, EPI_ISL_956701 to 956702, EPI_ISL_956704, EPI_ISL_956706 to 956710, EPI_ISL_956713 to 956715, EPI_ISL_956717 to 956718, EPI_ISL_956720 to 956723, EPI_ISL_956726, EPI_ISL_956729 to 956736, EPI_ISL_956738 to 956739, EPI_ISL_956741 to 956745, EPI_ISL_956748 to 956750, EPI_ISL_956752 to 956756, EPI_ISL_956758, EPI_ISL_956760 to 956761, EPI_ISL_956763 to 956764, EPI_ISL_958918, EPI_ISL_958920 to 958921, EPI_ISL_958923 to 958925, EPI_ISL_958928 to 958929, EPI_ISL_958931 to 958932, EPI_ISL_958934 to 958941, EPI_ISL_958943 to 958945, EPI_ISL_958948 to 958951, EPI_ISL_958955 to 958956, EPI_ISL_958958, EPI_ISL_958960, EPI_ISL_958963 to 958966, EPI_ISL_958968, EPI_ISL_958972 to 958977, EPI_ISL_958979 to 958990, EPI_ISL_958992, EPI_ISL_958994, EPI_ISL_958997 to 959000, EPI_ISL_959002 to 959004, EPI_ISL_959006, EPI_ISL_959009 to 959011, EPI_ISL_959013, EPI_ISL_959015 to 959023, EPI_ISL_959026, EPI_ISL_959028, EPI_ISL_959030 to 959041, EPI_ISL_959043 to 959052, EPI_ISL_959054 to 959063, EPI_ISL_959066 to 959069, EPI_ISL_959071, EPI_ISL_959073 to 959076, EPI_ISL_959079, EPI_ISL_959081 to 959087, EPI_ISL_959092, EPI_ISL_959094 to 959099, EPI_ISL_959101 to 959105, EPI_ISL_959108 to 959109, EPI_ISL_959112, EPI_ISL_959116 to 959118, EPI_ISL_959120 to 959126, EPI_ISL_959128 to 959129, EPI_ISL_959131, EPI_ISL_959133 to 959138, EPI_ISL_959140, EPI_ISL_959142, EPI_ISL_959144 to 959147, EPI_ISL_959150, EPI_ISL_959152 to 959159, EPI_ISL_959161 to 959163, EPI_ISL_959165 to 959169, EPI_ISL_959171 to 959172, EPI_ISL_959175 to 959176, EPI_ISL_959178, EPI_ISL_959180, EPI_ISL_959183 to 959184, EPI_ISL_959187 to 959201, EPI_ISL_959207, EPI_ISL_959209 to 959211, EPI_ISL_959214 to 959216, EPI_ISL_959219 to 959223, EPI_ISL_959226 to 959227, EPI_ISL_959231 to 959237, EPI_ISL_959239, EPI_ISL_959241 to 959249, EPI_ISL_959251 to 959259, EPI_ISL_959261 to 959262, EPI_ISL_959265, EPI_ISL_962975 to 962976, EPI_ISL_962978 to 962979, EPI_ISL_962981 to 962982, EPI_ISL_962984 to 962987, EPI_ISL_962989 to 962990, EPI_ISL_962992 to 962997, EPI_ISL_962999 to 963000, EPI_ISL_963002, EPI_ISL_963004, EPI_ISL_963006, EPI_ISL_963008 to 963017, EPI_ISL_963019 to 963024, EPI_ISL_963026 to 963031, EPI_ISL_963033 to 963036, EPI_ISL_963038 to 963040, EPI_ISL_963043, EPI_ISL_963045 to 963049, EPI_ISL_963051, EPI_ISL_963054 to 963055, EPI_ISL_963057, EPI_ISL_963059 to 963066, EPI_ISL_963069 to 963073, EPI_ISL_963075 to 963082, EPI_ISL_963084 to 963087, EPI_ISL_963089, EPI_ISL_963091 to 963098, EPI_ISL_963100 to 963101, EPI_ISL_963104, EPI_ISL_963106 to 963111, EPI_ISL_963115 to 963120, EPI_ISL_963122 to 963124, EPI_ISL_963127, EPI_ISL_963130 to 963132, EPI_ISL_963134 to 963142, EPI_ISL_963145 to 963149, EPI_ISL_963151 to 963160, EPI_ISL_963163 to 963165, EPI_ISL_963167 to 963169, EPI_ISL_963171 to 963172, EPI_ISL_963175 to 963178, EPI_ISL_963180 to 963191, EPI_ISL_963194 to 963196, EPI_ISL_963198 to 963202, EPI_ISL_963204, EPI_ISL_963206 to 963208, EPI_ISL_963210 to 963212, EPI_ISL_963215 to 963226, EPI_ISL_963228 to 963231, EPI_ISL_963233 to 963235, EPI_ISL_963237 to 963254, EPI_ISL_963256, EPI_ISL_963258 to 963259, EPI_ISL_963261 to 963264, EPI_ISL_963267 to 963284, EPI_ISL_963287 to 963288, EPI_ISL_963290 to 963294, EPI_ISL_963296 to 963310, EPI_ISL_963312 to 963313, EPI_ISL_963315 to 963323, EPI_ISL_963652, EPI_ISL_963654 to 963655, EPI_ISL_963657, EPI_ISL_963659 to 963665, EPI_ISL_963667 to 963672, EPI_ISL_963674, EPI_ISL_963676 to 963680, EPI_ISL_963685, EPI_ISL_963687, EPI_ISL_963689 to 963692, EPI_ISL_963694, EPI_ISL_963696 to 963698, EPI_ISL_963703, EPI_ISL_963705 to 963706, EPI_ISL_963709 to 963712, EPI_ISL_963714, EPI_ISL_963717 to 963726, EPI_ISL_963730 to 963732, EPI_ISL_963734, EPI_ISL_963736 to 963739, EPI_ISL_963741 to 963743, EPI_ISL_963745 to 963749, EPI_ISL_963751 to 963759, EPI_ISL_963761 to 963764, EPI_ISL_963767 to 963768, EPI_ISL_963770 to 963775, EPI_ISL_963777 to 963782, EPI_ISL_963784, EPI_ISL_963786, EPI_ISL_963788 to 963790, EPI_ISL_963792 to 963794, EPI_ISL_963796 to 963797, EPI_ISL_963799, EPI_ISL_963801, EPI_ISL_963804 to 963809, EPI_ISL_963811, EPI_ISL_963813 to 963814, EPI_ISL_963816 to 963817, EPI_ISL_963820 to 963821, EPI_ISL_963824, EPI_ISL_963826, EPI_ISL_963828 to 963829, EPI_ISL_963831 to 963833, EPI_ISL_963836, EPI_ISL_963838, EPI_ISL_963840 to 963848, EPI_ISL_963850 to 963851, EPI_ISL_963853 to 963854, EPI_ISL_963856 to 963865, EPI_ISL_963867, EPI_ISL_963869 to 963873, EPI_ISL_963875 to 963882, EPI_ISL_963884 to 963885, EPI_ISL_963888 to 963889, EPI_ISL_963891 to 963892, EPI_ISL_963895 to 963903, EPI_ISL_963906, EPI_ISL_963908, EPI_ISL_963910, EPI_ISL_963912 to 963914, EPI_ISL_963916 to 963920, EPI_ISL_963922 to 963923, EPI_ISL_963926 to 963929, EPI_ISL_963932 to 963936, EPI_ISL_963939 to 963942, EPI_ISL_963944 to 963952, EPI_ISL_963955 to 963956, EPI_ISL_963958 to 963959, EPI_ISL_963963 to 963964, EPI_ISL_963966 to 963971, EPI_ISL_963973 to 963974, EPI_ISL_963976, EPI_ISL_963978, EPI_ISL_963980 to 963981, EPI_ISL_963984 to 963986, EPI_ISL_963988 to 963991, EPI_ISL_963993 to 963994, EPI_ISL_963996, EPI_ISL_963998 to 964001, EPI_ISL_964003 to 964007, EPI_ISL_964011 to 964013, EPI_ISL_964016 to 964022, EPI_ISL_964024 to 964025, EPI_ISL_964027 to 964029, EPI_ISL_964031 to 964032, EPI_ISL_964034 to 964040, EPI_ISL_964042, EPI_ISL_964045, EPI_ISL_964047 to 964054, EPI_ISL_964056 to 964061, EPI_ISL_964063 to 964065, EPI_ISL_964067 to 964083, EPI_ISL_964085 to 964087, EPI_ISL_964089 to 964090, EPI_ISL_964092 to 964095, EPI_ISL_964098 to 964099, EPI_ISL_964101 to 964110, EPI_ISL_964112 to 964113, EPI_ISL_964115 to 964126, EPI_ISL_964129, EPI_ISL_964131, EPI_ISL_964133, EPI_ISL_964135, EPI_ISL_964137 to 964141, EPI_ISL_964144 to 964148, EPI_ISL_964150, EPI_ISL_964153 to 964154, EPI_ISL_964156 to 964159, EPI_ISL_964161 to 964163, EPI_ISL_964165 to 964166, EPI_ISL_964168, EPI_ISL_964170, EPI_ISL_964172 to 964174, EPI_ISL_964176 to 964181, EPI_ISL_964183, EPI_ISL_964185 to 964196, EPI_ISL_964198 to 964200, EPI_ISL_964202 to 964203, EPI_ISL_964205, EPI_ISL_964208 to 964210, EPI_ISL_964212 to 964215, EPI_ISL_964217, EPI_ISL_964219, EPI_ISL_964221, EPI_ISL_964223, EPI_ISL_964226 to 964229, EPI_ISL_964231 to 964232, EPI_ISL_964235, EPI_ISL_964238, EPI_ISL_964240, EPI_ISL_964242, EPI_ISL_964244, EPI_ISL_964246 to 964247, EPI_ISL_964249 to 964253, EPI_ISL_964255 to 964256, EPI_ISL_964258, EPI_ISL_964260, EPI_ISL_964264, EPI_ISL_964268 to 964269, EPI_ISL_964276 to 964277, EPI_ISL_964279 to 964280, EPI_ISL_964282, EPI_ISL_964285 to 964286, EPI_ISL_964290, EPI_ISL_964296, EPI_ISL_964298 to 964299, EPI_ISL_964302, EPI_ISL_964304, EPI_ISL_964307, EPI_ISL_964309 to 964314, EPI_ISL_964316 to 964318, EPI_ISL_964321 to 964322, EPI_ISL_964324, EPI_ISL_964326 to 964333, EPI_ISL_964335, EPI_ISL_964337 to 964341, EPI_ISL_964543 to 964546, EPI_ISL_964548 to 964553, EPI_ISL_964555 to 964560, EPI_ISL_964562 to 964566, EPI_ISL_964568 to 964571, EPI_ISL_964573 to 964581, EPI_ISL_964584 to 964586, EPI_ISL_964588, EPI_ISL_964590 to 964592, EPI_ISL_964594 to 964595, EPI_ISL_964598 to 964600, EPI_ISL_964603, EPI_ISL_964606 to 964614, EPI_ISL_964616 to 964624, EPI_ISL_964627 to 964638, EPI_ISL_964640 to 964647, EPI_ISL_964649 to 964669, EPI_ISL_964671 to 964677, EPI_ISL_964679 to 964681, EPI_ISL_964683 to 964684, EPI_ISL_964687 to 964692, EPI_ISL_964694 to 964695, EPI_ISL_964697 to 964699, EPI_ISL_964701 to 964708, EPI_ISL_964710 to 964712, EPI_ISL_964714, EPI_ISL_964716, EPI_ISL_964718 to 964720, EPI_ISL_964723 to 964725, EPI_ISL_964727, EPI_ISL_964730 to 964733, EPI_ISL_964735 to 964738, EPI_ISL_964740, EPI_ISL_964742, EPI_ISL_964744 to 964751, EPI_ISL_964754 to 964758, EPI_ISL_964761 to 964763, EPI_ISL_964765 to 964766, EPI_ISL_964769 to 964771, EPI_ISL_964773, EPI_ISL_964775, EPI_ISL_964777, EPI_ISL_964780, EPI_ISL_964782 to 964787, EPI_ISL_964789, EPI_ISL_964791 to 964792, EPI_ISL_964794 to 964801, EPI_ISL_964804 to 964810, EPI_ISL_964812, EPI_ISL_964814, EPI_ISL_964816, EPI_ISL_964819 to 964822, EPI_ISL_964824 to 964826, EPI_ISL_964828 to 964832, EPI_ISL_964834 to 964839, EPI_ISL_964841 to 964847, EPI_ISL_964850 to 964852, EPI_ISL_964854 to 964855, EPI_ISL_964857 to 964864, EPI_ISL_964866, EPI_ISL_964868 to 964869, EPI_ISL_964871 to 964875, EPI_ISL_964877, EPI_ISL_969457, EPI_ISL_969459 to 969460, EPI_ISL_969462, EPI_ISL_969464 to 969466, EPI_ISL_969468 to 969469, EPI_ISL_969472 to 969475, EPI_ISL_969477 to 969478, EPI_ISL_969480 to 969482, EPI_ISL_969484 to 969486, EPI_ISL_969488 to 969489, EPI_ISL_969491 to 969495, EPI_ISL_969497 to 969498, EPI_ISL_969500 to 969505, EPI_ISL_969507 to 969508, EPI_ISL_969510 to 969514, EPI_ISL_969516 to 969517, EPI_ISL_969519 to 969522, EPI_ISL_969525 to 969526, EPI_ISL_969529, EPI_ISL_969532 to 969534, EPI_ISL_969536 to 969543, EPI_ISL_969545 to 969546, EPI_ISL_969548 to 969549, EPI_ISL_969552 to 969554, EPI_ISL_969556 to 969557, EPI_ISL_969559 to 969567, EPI_ISL_969569, EPI_ISL_969571 to 969572, EPI_ISL_969574 to 969584, EPI_ISL_969587 to 969589, EPI_ISL_969591, EPI_ISL_969593 to 969595, EPI_ISL_969597 to 969600, EPI_ISL_969604 to 969611, EPI_ISL_969615 to 969619, EPI_ISL_969621 to 969625, EPI_ISL_969628, EPI_ISL_969630 to 969631, EPI_ISL_969636 to 969645, EPI_ISL_969647, EPI_ISL_969650 to 969655, EPI_ISL_969657 to 969660, EPI_ISL_969662, EPI_ISL_969664 to 969666, EPI_ISL_969669, EPI_ISL_969673 to 969675, EPI_ISL_969677, EPI_ISL_969682 to 969683, EPI_ISL_969685, EPI_ISL_969688, EPI_ISL_969690 to 969691, EPI_ISL_969695, EPI_ISL_969699, EPI_ISL_969702, EPI_ISL_969704, EPI_ISL_969706, EPI_ISL_969710, EPI_ISL_969712, EPI_ISL_969714, EPI_ISL_969716, EPI_ISL_969718, EPI_ISL_969720, EPI_ISL_969724, EPI_ISL_969726, EPI_ISL_969728, EPI_ISL_969730, EPI_ISL_969732, EPI_ISL_969734, EPI_ISL_969742, EPI_ISL_969746, EPI_ISL_969750, EPI_ISL_969752, EPI_ISL_969756, EPI_ISL_969759 to 969760, EPI_ISL_969762, EPI_ISL_969764, EPI_ISL_969766, EPI_ISL_969768, EPI_ISL_969771, EPI_ISL_969780, EPI_ISL_969782, EPI_ISL_969785 to 969786, EPI_ISL_969789, EPI_ISL_969791, EPI_ISL_969793, EPI_ISL_969795, EPI_ISL_969797, EPI_ISL_969799, EPI_ISL_969801 to 969802, EPI_ISL_969804, EPI_ISL_969806, EPI_ISL_969808, EPI_ISL_969812 to 969813, EPI_ISL_969815, EPI_ISL_969817, EPI_ISL_969823, EPI_ISL_969826, EPI_ISL_969828, EPI_ISL_969831, EPI_ISL_969833 to 969834, EPI_ISL_969836, EPI_ISL_969838, EPI_ISL_969840 to 969841, EPI_ISL_969843, EPI_ISL_969848, EPI_ISL_969856, EPI_ISL_969858 to 969859, EPI_ISL_969861, EPI_ISL_969863, EPI_ISL_969867, EPI_ISL_969875, EPI_ISL_969877, EPI_ISL_969879, EPI_ISL_969883, EPI_ISL_969887, EPI_ISL_969889, EPI_ISL_969891, EPI_ISL_969895, EPI_ISL_969897, EPI_ISL_969899, EPI_ISL_969903, EPI_ISL_969906 to 969907, EPI_ISL_969909, EPI_ISL_969914, EPI_ISL_969919, EPI_ISL_969921, EPI_ISL_969925, EPI_ISL_969928, EPI_ISL_969930, EPI_ISL_969932, EPI_ISL_969934, EPI_ISL_969936 to 969937, EPI_ISL_969939 to 969940, EPI_ISL_969943, EPI_ISL_969945, EPI_ISL_969947 to 969948, EPI_ISL_969952, EPI_ISL_969955 to 969956, EPI_ISL_969958, EPI_ISL_969961, EPI_ISL_969967, EPI_ISL_969969, EPI_ISL_969971, EPI_ISL_969975, EPI_ISL_969977, EPI_ISL_969979, EPI_ISL_969982 to 969983, EPI_ISL_969986, EPI_ISL_969988, EPI_ISL_969990, EPI_ISL_969996, EPI_ISL_969998, EPI_ISL_970002, EPI_ISL_970005, EPI_ISL_970007, EPI_ISL_970009, EPI_ISL_970011, EPI_ISL_970013, EPI_ISL_970017, EPI_ISL_970019, EPI_ISL_970022, EPI_ISL_970024, EPI_ISL_970028, EPI_ISL_970030, EPI_ISL_970033, EPI_ISL_970035, EPI_ISL_970037, EPI_ISL_970039, EPI_ISL_970045, EPI_ISL_970047, EPI_ISL_970049 to 970050, EPI_ISL_970052, EPI_ISL_970057, EPI_ISL_970059, EPI_ISL_970066, EPI_ISL_970070, EPI_ISL_970073, EPI_ISL_970075 to 970076, EPI_ISL_970079 to 970080, EPI_ISL_970082, EPI_ISL_970084, EPI_ISL_970086 to 970087, EPI_ISL_970089, EPI_ISL_970094, EPI_ISL_970096, EPI_ISL_970098 to 970099, EPI_ISL_970101, EPI_ISL_970103, EPI_ISL_970105, EPI_ISL_970109, EPI_ISL_970111, EPI_ISL_970115, EPI_ISL_970117, EPI_ISL_970119, EPI_ISL_970122, EPI_ISL_970124, EPI_ISL_970126, EPI_ISL_970128, EPI_ISL_970132, EPI_ISL_970134, EPI_ISL_970136, EPI_ISL_970138, EPI_ISL_970140, EPI_ISL_970142, EPI_ISL_970145, EPI_ISL_970147, EPI_ISL_970150, EPI_ISL_970153, EPI_ISL_970155, EPI_ISL_970160, EPI_ISL_970163, EPI_ISL_970165 to 970166, EPI_ISL_970168, EPI_ISL_970172, EPI_ISL_970175, EPI_ISL_970177, EPI_ISL_970181, EPI_ISL_970183, EPI_ISL_970186, EPI_ISL_970190, EPI_ISL_970192, EPI_ISL_970195, EPI_ISL_970198, EPI_ISL_970200, EPI_ISL_970202, EPI_ISL_970205, EPI_ISL_970209, EPI_ISL_970211, EPI_ISL_970213, EPI_ISL_970217, EPI_ISL_970221, EPI_ISL_970224, EPI_ISL_970227, EPI_ISL_970229, EPI_ISL_970231, EPI_ISL_970233, EPI_ISL_970235, EPI_ISL_970237, EPI_ISL_970242, EPI_ISL_970249, EPI_ISL_970251 to 970252, EPI_ISL_970254, EPI_ISL_970256, EPI_ISL_970258, EPI_ISL_970260, EPI_ISL_970262, EPI_ISL_970266, EPI_ISL_970270, EPI_ISL_970274, EPI_ISL_970278 to 970279, EPI_ISL_970281, EPI_ISL_970283, EPI_ISL_970285, EPI_ISL_970287, EPI_ISL_970291 to 970292, EPI_ISL_970295, EPI_ISL_970297, EPI_ISL_970301, EPI_ISL_970303, EPI_ISL_970307, EPI_ISL_970312, EPI_ISL_970314, EPI_ISL_970316, EPI_ISL_970319, EPI_ISL_970321, EPI_ISL_970326, EPI_ISL_970328, EPI_ISL_970330, EPI_ISL_970332, EPI_ISL_970334, EPI_ISL_970338, EPI_ISL_970340, EPI_ISL_970342, EPI_ISL_970344, EPI_ISL_970352, EPI_ISL_970356, EPI_ISL_970358, EPI_ISL_970362, EPI_ISL_970364 to 970365, EPI_ISL_970368, EPI_ISL_970370 to 970371, EPI_ISL_970373, EPI_ISL_970376 to 970377, EPI_ISL_970382 to 970383, EPI_ISL_970386 to 970387, EPI_ISL_970390, EPI_ISL_970392, EPI_ISL_970394, EPI_ISL_970396, EPI_ISL_970398, EPI_ISL_970400, EPI_ISL_970402, EPI_ISL_970404, EPI_ISL_970409, EPI_ISL_970412, EPI_ISL_970415 to 970416, EPI_ISL_970419, EPI_ISL_970422, EPI_ISL_970426, EPI_ISL_970428, EPI_ISL_970435, EPI_ISL_970439, EPI_ISL_970441, EPI_ISL_970444, EPI_ISL_970446, EPI_ISL_970448, EPI_ISL_970450, EPI_ISL_970452, EPI_ISL_970454, EPI_ISL_970460, EPI_ISL_970464, EPI_ISL_970466, EPI_ISL_970468, EPI_ISL_970470, EPI_ISL_970475, EPI_ISL_970477 to 970478, EPI_ISL_970481, EPI_ISL_970483, EPI_ISL_970486, EPI_ISL_970488, EPI_ISL_970492 to 970493, EPI_ISL_970495, EPI_ISL_970500, EPI_ISL_970502, EPI_ISL_970504, EPI_ISL_970506, EPI_ISL_970508, EPI_ISL_970510, EPI_ISL_970512 to 970513, EPI_ISL_970515, EPI_ISL_970521, EPI_ISL_970523, EPI_ISL_970525, EPI_ISL_970527, EPI_ISL_970530, EPI_ISL_970533, EPI_ISL_970535, EPI_ISL_970537, EPI_ISL_970540 to 970541, EPI_ISL_970546, EPI_ISL_970548, EPI_ISL_970555, EPI_ISL_970558, EPI_ISL_970560, EPI_ISL_970562, EPI_ISL_970566, EPI_ISL_970568 to 970569, EPI_ISL_970573, EPI_ISL_970575, EPI_ISL_970577, EPI_ISL_970583, EPI_ISL_970585, EPI_ISL_985612 to 985619, EPI_ISL_985621 to 985623, EPI_ISL_985625 to 985629, EPI_ISL_985631 to 985633, EPI_ISL_985635, EPI_ISL_985637 to 985638, EPI_ISL_985640, EPI_ISL_985643 to 985649, EPI_ISL_985651 to 985662, EPI_ISL_985665 to 985667, EPI_ISL_985669 to 985670, EPI_ISL_985672 to 985673,

EPI_ISL_985675 to 985677, EPI_ISL_985679 to 985680, EPI_ISL_985682 to 985683, EPI_ISL_985685 to 985686, EPI_ISL_985688, EPI_ISL_985690 to 985693, EPI_ISL_985695 to 985697, EPI_ISL_985699 to 985700, EPI_ISL_985703, EPI_ISL_985705 to 985706, EPI_ISL_985708 to 985712, EPI_ISL_985714 to 985716, EPI_ISL_985718 to 985725, EPI_ISL_985727 to 985728, EPI_ISL_985730 to 985731, EPI_ISL_985733 to 985734, EPI_ISL_985736 to 985751, EPI_ISL_985754, EPI_ISL_985756 to 985757, EPI_ISL_985759 to 985772, EPI_ISL_985774 to 985792, EPI_ISL_985794 to 985796, EPI_ISL_985799, EPI_ISL_985801 to 985803, EPI_ISL_985806 to 985809, EPI_ISL_985811 to 985819, EPI_ISL_985821, EPI_ISL_985823 to 985824, EPI_ISL_985827 to 985834, EPI_ISL_985836, EPI_ISL_985839 to 985851, EPI_ISL_985853 to 985855, EPI_ISL_985857, EPI_ISL_985859 to 985861, EPI_ISL_985865, EPI_ISL_985867 to 985869, EPI_ISL_985871 to 985876, EPI_ISL_985878 to 985885, EPI_ISL_985887 to 985889, EPI_ISL_985891 to 985893, EPI_ISL_985895 to 985896, EPI_ISL_985898 to 985900, EPI_ISL_985902 to 985911, EPI_ISL_985913 to 985918, EPI_ISL_985920, EPI_ISL_985922 to 985924, EPI_ISL_985927, EPI_ISL_985929 to 985931, EPI_ISL_985936 to 985937, EPI_ISL_985940 to 985945, EPI_ISL_986824 to 986825, EPI_ISL_986827 to 986833, EPI_ISL_986835 to 986841, EPI_ISL_986843 to 986854, EPI_ISL_986856, EPI_ISL_986858 to 986864, EPI_ISL_986866 to 986867, EPI_ISL_986869 to 986870, EPI_ISL_986872 to 986873, EPI_ISL_986875 to 986879, EPI_ISL_986881 to 986884, EPI_ISL_986886 to 986889, EPI_ISL_986891 to 986895, EPI_ISL_986898 to 986904, EPI_ISL_986906 to 986910, EPI_ISL_986912 to 986915, EPI_ISL_986918 to 986925, EPI_ISL_986927 to 986931, EPI_ISL_986933, EPI_ISL_986935 to 986938, EPI_ISL_986940 to 986942, EPI_ISL_986944 to 986946, EPI_ISL_986948, EPI_ISL_986950 to 986955, EPI_ISL_986957, EPI_ISL_986960 to 986966, EPI_ISL_986968, EPI_ISL_986970 to 986977, EPI_ISL_986979 to 986985, EPI_ISL_986988 to 987005, EPI_ISL_987007, EPI_ISL_987009 to 987010, EPI_ISL_987012 to 987014, EPI_ISL_987016 to 987023, EPI_ISL_987025 to 987027, EPI_ISL_987029 to 987030, EPI_ISL_987033 to 987043, EPI_ISL_987045 to 987055, EPI_ISL_987057, EPI_ISL_987059, EPI_ISL_987061 to 987068, EPI_ISL_987070 to 987072, EPI_ISL_987076 to 987079, EPI_ISL_987081 to 987082, EPI_ISL_987085 to 987096, EPI_ISL_987098 to 987100, EPI_ISL_987102 to 987111, EPI_ISL_987114 to 987126, EPI_ISL_987128, EPI_ISL_987130 to 987131, EPI_ISL_987133 to 987134, EPI_ISL_987136 to 987140, EPI_ISL_987142 to 987147, EPI_ISL_987149 to 987154, EPI_ISL_987156 to 987159, EPI_ISL_987333, EPI_ISL_987336, EPI_ISL_987410, EPI_ISL_987448, EPI_ISL_987469, EPI_ISL_987524, EPI_ISL_987584, EPI_ISL_988686 to 988696, EPI_ISL_988699 to 988700, EPI_ISL_988704 to 988706, EPI_ISL_988708 to 988709, EPI_ISL_988711 to 988713, EPI_ISL_988715 to 988720, EPI_ISL_988722, EPI_ISL_988725, EPI_ISL_988727 to 988728, EPI_ISL_988730, EPI_ISL_988732 to 988748, EPI_ISL_988751 to 988752, EPI_ISL_988754 to 988755, EPI_ISL_988757 to 988760, EPI_ISL_988762 to 988763, EPI_ISL_988765 to 988770, EPI_ISL_988772 to 988785, EPI_ISL_988787 to 988790, EPI_ISL_988794, EPI_ISL_988796 to 988803, EPI_ISL_988805 to 988807, EPI_ISL_988809 to 988816, EPI_ISL_988818 to 988824, EPI_ISL_988826, EPI_ISL_988828 to 988830, EPI_ISL_988832 to 988834, EPI_ISL_988836 to 988839, EPI_ISL_988841 to 988854, EPI_ISL_988856 to 988859, EPI_ISL_988861 to 988864, EPI_ISL_988866 to 988867, EPI_ISL_988869, EPI_ISL_988871, EPI_ISL_988873 to 988877, EPI_ISL_988879 to 988880, EPI_ISL_988882, EPI_ISL_988884, EPI_ISL_988886 to 988887, EPI_ISL_988889 to 988892, EPI_ISL_988894, EPI_ISL_988897 to 988914, EPI_ISL_988916, EPI_ISL_988918 to 988926, EPI_ISL_988928 to 988929, EPI_ISL_988931 to 988932, EPI_ISL_988934 to 988936, EPI_ISL_988939 to 988944, EPI_ISL_988946 to 988949, EPI_ISL_988951 to 988969, EPI_ISL_988972 to 988981, EPI_ISL_988984 to 988987, EPI_ISL_988989 to 988992, EPI_ISL_988994, EPI_ISL_988996 to 989000, EPI_ISL_989003, EPI_ISL_989005 to 989008, EPI_ISL_989010, EPI_ISL_989012 to 989019, EPI_ISL_989021, EPI_ISL_989023 to 989029, EPI_ISL_989032, EPI_ISL_989034 to 989036, EPI_ISL_989038 to 989049, EPI_ISL_989051 to 989058, EPI_ISL_989061 to 989065, EPI_ISL_989067 to 989068, EPI_ISL_989072 to 989074, EPI_ISL_989076 to 989081, EPI_ISL_989084 to 989087, EPI_ISL_989090 to 989094, EPI_ISL_989097 to 989099, EPI_ISL_989101 to 989103, EPI_ISL_989106, EPI_ISL_989108 to 989110, EPI_ISL_989112 to 989113, EPI_ISL_989116 to 989117, EPI_ISL_989119 to 989126, EPI_ISL_989128, EPI_ISL_989131 to 989135, EPI_ISL_989137 to 989139, EPI_ISL_989141 to 989142, EPI_ISL_989145 to 989149, EPI_ISL_989151, EPI_ISL_989153, EPI_ISL_989155 to 989156, EPI_ISL_989161, EPI_ISL_989163, EPI_ISL_989165, EPI_ISL_989167 to 989172, EPI_ISL_989176 to 989184, EPI_ISL_989186 to 989188, EPI_ISL_989191 to 989196, EPI_ISL_989198 to 989200, EPI_ISL_989202 to 989204, EPI_ISL_989206 to 989214, EPI_ISL_989216, EPI_ISL_989218, EPI_ISL_989220 to 989225, EPI_ISL_989227 to 989240, EPI_ISL_989242, EPI_ISL_989244 to 989245, EPI_ISL_989247 to 989249, EPI_ISL_989252 to 989263, EPI_ISL_989265 to 989271, EPI_ISL_989273 to 989278, EPI_ISL_989280 to 989281, EPI_ISL_989283 to 989289, EPI_ISL_989292 to 989293, EPI_ISL_989295 to 989296, EPI_ISL_989299, EPI_ISL_989301 to 989305, EPI_ISL_989307 to 989309, EPI_ISL_989311 to 989312, EPI_ISL_989314 to 989320, EPI_ISL_989322 to 989324, EPI_ISL_989326 to 989331, EPI_ISL_989333 to 989335, EPI_ISL_989337 to 989340, EPI_ISL_989342, EPI_ISL_989344 to 989357, EPI_ISL_989360 to 989361, EPI_ISL_989446 to 989447, EPI_ISL_989483, EPI_ISL_989490, EPI_ISL_989492, EPI_ISL_989497 to 989499, EPI_ISL_989503 to 989504, EPI_ISL_989506 to 989507, EPI_ISL_989509 to 989511, EPI_ISL_989513 to 989514, EPI_ISL_989520, EPI_ISL_989522 to 989523, EPI_ISL_989525, EPI_ISL_989527 to 989529, EPI_ISL_989697, EPI_ISL_989699, EPI_ISL_989701, EPI_ISL_989707 to 989709, EPI_ISL_989712, EPI_ISL_989714 to 989717, EPI_ISL_989719 to 989727, EPI_ISL_989729, EPI_ISL_989731, EPI_ISL_989735, EPI_ISL_989741 to 989743, EPI_ISL_989751 to 989752, EPI_ISL_989755, EPI_ISL_989757, EPI_ISL_989759 to 989760, EPI_ISL_989763, EPI_ISL_989768 to 989769, EPI_ISL_989773, EPI_ISL_989778 to 989779, EPI_ISL_989786, EPI_ISL_989788 to 989789, EPI_ISL_989791 to 989792, EPI_ISL_989856 to 989859, EPI_ISL_989866, EPI_ISL_989868, EPI_ISL_989876, EPI_ISL_989881, EPI_ISL_989999, EPI_ISL_990014, EPI_ISL_990019, EPI_ISL_990043, EPI_ISL_990045 to 990046, EPI_ISL_990052 to 990053, EPI_ISL_990055, EPI_ISL_990142, EPI_ISL_990151, EPI_ISL_990157, EPI_ISL_990160 to 990168, EPI_ISL_990170 to 990176, EPI_ISL_990178, EPI_ISL_990182 to 990184, EPI_ISL_990186, EPI_ISL_990190 to 990196, EPI_ISL_990200 to 990205, EPI_ISL_990207, EPI_ISL_990209, EPI_ISL_990211 to 990214, EPI_ISL_990217 to 990222, EPI_ISL_990224, EPI_ISL_990226 to 990228, EPI_ISL_990230 to 990232, EPI_ISL_990236 to 990242, EPI_ISL_990244, EPI_ISL_990246 to 990250, EPI_ISL_990252, EPI_ISL_990254 to 990255, EPI_ISL_990258, EPI_ISL_990260 to 990262, EPI_ISL_990265 to 990268, EPI_ISL_990271 to 990279, EPI_ISL_990281 to 990283, EPI_ISL_990285, EPI_ISL_990287 to 990289, EPI_ISL_990291 to 990305, EPI_ISL_990307 to 990308, EPI_ISL_990311 to 990314, EPI_ISL_990316 to 990317, EPI_ISL_990320 to 990324, EPI_ISL_990326 to 990327, EPI_ISL_990329 to 990333, EPI_ISL_990336 to 990338, EPI_ISL_990340, EPI_ISL_990343 to 990344, EPI_ISL_990346, EPI_ISL_990348 to 990349, EPI_ISL_990353 to 990354, EPI_ISL_990356 to 990357, EPI_ISL_990360 to 990366, EPI_ISL_990370 to 990371, EPI_ISL_990374 to 990376, EPI_ISL_990379 to 990382, EPI_ISL_990385 to 990393, EPI_ISL_990395 to 990396, EPI_ISL_990398 to 990406, EPI_ISL_990409 to 990413, EPI_ISL_990416, EPI_ISL_990418 to 990421, EPI_ISL_990424, EPI_ISL_990426 to 990429, EPI_ISL_990431 to 990432, EPI_ISL_990436 to 990444, EPI_ISL_990447 to 990449, EPI_ISL_990454 to 990461, EPI_ISL_990464 to 990468, EPI_ISL_990470 to 990474, EPI_ISL_990476, EPI_ISL_990479 to 990486, EPI_ISL_990488 to 990495, EPI_ISL_990497 to 990508, EPI_ISL_990510, EPI_ISL_990513 to 990514, EPI_ISL_990516 to 990518, EPI_ISL_990522, EPI_ISL_990524, EPI_ISL_990526 to 990531, EPI_ISL_990535 to 990537, EPI_ISL_990541 to 990543, EPI_ISL_990546 to 990550, EPI_ISL_990552 to 990553, EPI_ISL_990555 to 990556, EPI_ISL_990558 to 990559, EPI_ISL_990561, EPI_ISL_990563, EPI_ISL_990565, EPI_ISL_990567, EPI_ISL_990569 to 990571, EPI_ISL_990574 to 990575, EPI_ISL_990577 to 990579, EPI_ISL_990582 to 990586, EPI_ISL_990588 to 990593, EPI_ISL_990595 to 990596, EPI_ISL_990598 to 990601, EPI_ISL_990605 to 990606, EPI_ISL_990608, EPI_ISL_990610 to 990611, EPI_ISL_990614 to 990615, EPI_ISL_990617 to 990618, EPI_ISL_990621, EPI_ISL_990624 to 990627, EPI_ISL_990629 to 990630, EPI_ISL_990632 to 990633, EPI_ISL_990635 to 990637, EPI_ISL_990639, EPI_ISL_990643 to 990644, EPI_ISL_990646, EPI_ISL_990648 to 990656, EPI_ISL_990659, EPI_ISL_990662 to 990664, EPI_ISL_990666, EPI_ISL_990668, EPI_ISL_990670 to 990681, EPI_ISL_990683, EPI_ISL_990685, EPI_ISL_990691 to 990693, EPI_ISL_990697 to 990698, EPI_ISL_990700, EPI_ISL_990702, EPI_ISL_990705 to 990706, EPI_ISL_990708 to 990709, EPI_ISL_990711, EPI_ISL_990713, EPI_ISL_990716 to 990718, EPI_ISL_990720, EPI_ISL_990723 to 990725, EPI_ISL_990727 to 990735, EPI_ISL_990737, EPI_ISL_990739 to 990742, EPI_ISL_990744 to 990747, EPI_ISL_990749 to 990754, EPI_ISL_990756 to 990757, EPI_ISL_990759, EPI_ISL_990761, EPI_ISL_990764 to 990765, EPI_ISL_990769 to 990771, EPI_ISL_990773, EPI_ISL_990775 to 990779, EPI_ISL_990781, EPI_ISL_990783 to 990785, EPI_ISL_990788, EPI_ISL_990790 to 990808, EPI_ISL_990810 to 990818, EPI_ISL_990820 to 990828, EPI_ISL_990830 to 990841, EPI_ISL_990846, EPI_ISL_990848 to 990856, EPI_ISL_990858 to 990863, EPI_ISL_990866 to 990867, EPI_ISL_990869, EPI_ISL_990871 to 990872, EPI_ISL_990876 to 990878, EPI_ISL_990880 to 990883, EPI_ISL_990885, EPI_ISL_990887 to 990889, EPI_ISL_990892, EPI_ISL_990894 to 990897, EPI_ISL_990899 to 990900, EPI_ISL_990902 to 990905, EPI_ISL_990907, EPI_ISL_990909 to 990911, EPI_ISL_990913 to 990915, EPI_ISL_990918, EPI_ISL_990920 to 990921, EPI_ISL_990923 to 990926, EPI_ISL_990928, EPI_ISL_990931, EPI_ISL_990933 to 990938, EPI_ISL_990940 to 990942, EPI_ISL_990944, EPI_ISL_990946 to 990947, EPI_ISL_990949 to 990952, EPI_ISL_990955 to 990956, EPI_ISL_990958, EPI_ISL_990960 to 990964, EPI_ISL_990968 to 990969, EPI_ISL_990971, EPI_ISL_990975 to 990977, EPI_ISL_990980 to 990983, EPI_ISL_990986 to 990987, EPI_ISL_990989, EPI_ISL_990991, EPI_ISL_990994 to 990996, EPI_ISL_990998, EPI_ISL_991000 to 991002, EPI_ISL_991004 to 991006, EPI_ISL_991008 to 991009, EPI_ISL_991011, EPI_ISL_991014 to 991021, EPI_ISL_991024 to 991026, EPI_ISL_991029 to 991031, EPI_ISL_991034 to 991035, EPI_ISL_991037 to 991040, EPI_ISL_991042, EPI_ISL_991044, EPI_ISL_991047 to 991052, EPI_ISL_991054 to 991056, EPI_ISL_991058 to 991059, EPI_ISL_991062 to 991065, EPI_ISL_991067, EPI_ISL_991069 to 991073, EPI_ISL_991076, EPI_ISL_991081 to 991084, EPI_ISL_991087 to 991088, EPI_ISL_991090 to 991092, EPI_ISL_991094 to 991095, EPI_ISL_991097 to 991098, EPI_ISL_991100, EPI_ISL_991102 to 991104, EPI_ISL_991106 to 991111, EPI_ISL_991113 to 991115, EPI_ISL_991117 to 991120, EPI_ISL_991123, EPI_ISL_991128 to 991130, EPI_ISL_991134, EPI_ISL_991137, EPI_ISL_991139 to 991140, EPI_ISL_991142 to 991143, EPI_ISL_991145 to 991147, EPI_ISL_991149 to 991150, EPI_ISL_991152 to 991155, EPI_ISL_991157 to 991162, EPI_ISL_991164 to 991166, EPI_ISL_991168 to 991171, EPI_ISL_991179, EPI_ISL_991183, EPI_ISL_991187, EPI_ISL_991192, EPI_ISL_991194, EPI_ISL_991203, EPI_ISL_991205 to 991206, EPI_ISL_991208, EPI_ISL_991210, EPI_ISL_991212, EPI_ISL_991215 to 991216, EPI_ISL_991218, EPI_ISL_991227, EPI_ISL_991231, EPI_ISL_991241, EPI_ISL_991243, EPI_ISL_991246, EPI_ISL_991256 to 991257, EPI_ISL_991263 to 991264, EPI_ISL_991268, EPI_ISL_991271 to 991273, EPI_ISL_991277, EPI_ISL_991280, EPI_ISL_991287, EPI_ISL_991290 to 991291, EPI_ISL_991304 to 991306, EPI_ISL_991339, EPI_ISL_991565 to 991568, EPI_ISL_991570 to 991572, EPI_ISL_991574, EPI_ISL_991578 to 991580, EPI_ISL_991586 to 991587, EPI_ISL_991590, EPI_ISL_991592 to 991595, EPI_ISL_991598 to 991599, EPI_ISL_991601, EPI_ISL_991603 to 991613, EPI_ISL_991615, EPI_ISL_991619 to 991622, EPI_ISL_991624, EPI_ISL_991626, EPI_ISL_991629 to 991630, EPI_ISL_991632 to 991633, EPI_ISL_991635, EPI_ISL_991637, EPI_ISL_991639, EPI_ISL_991641, EPI_ISL_991644 to 991650, EPI_ISL_991652 to 991658, EPI_ISL_991660, EPI_ISL_991662, EPI_ISL_991664 to 991665, EPI_ISL_991668, EPI_ISL_991670 to 991672, EPI_ISL_991676 to 991679, EPI_ISL_991681, EPI_ISL_991683, EPI_ISL_991685, EPI_ISL_991687 to 991693, EPI_ISL_991695, EPI_ISL_991697, EPI_ISL_991700, EPI_ISL_991702 to 991704, EPI_ISL_991707 to 991708, EPI_ISL_991710, EPI_ISL_991712 to 991721, EPI_ISL_991724 to 991725, EPI_ISL_991727 to 991731, EPI_ISL_991734 to 991738, EPI_ISL_991741 to 991744, EPI_ISL_991746 to 991749, EPI_ISL_991753 to 991756, EPI_ISL_991758, EPI_ISL_991760, EPI_ISL_991762 to 991765, EPI_ISL_991768 to 991769, EPI_ISL_991772, EPI_ISL_991775, EPI_ISL_991777 to 991778, EPI_ISL_991780 to 991781, EPI_ISL_991784, EPI_ISL_991786, EPI_ISL_991788 to 991790, EPI_ISL_991795 to 991797, EPI_ISL_991799 to 991801, EPI_ISL_991803 to 991804, EPI_ISL_991806 to 991810, EPI_ISL_991812, EPI_ISL_991814 to 991819, EPI_ISL_991821 to 991824, EPI_ISL_991826, EPI_ISL_991828 to 991829, EPI_ISL_991831 to 991832, EPI_ISL_991835 to 991837, EPI_ISL_991840 to 991849, EPI_ISL_991854 to 991860, EPI_ISL_991862 to 991870, EPI_ISL_991873 to 991874, EPI_ISL_991876 to 991879, EPI_ISL_991881 to 991882, EPI_ISL_991885, EPI_ISL_991887 to 991888, EPI_ISL_991890, EPI_ISL_991892 to 991895, EPI_ISL_991897 to 991898, EPI_ISL_991900, EPI_ISL_991908, EPI_ISL_991910, EPI_ISL_991913, EPI_ISL_991916 to 991917, EPI_ISL_991923, EPI_ISL_991928, EPI_ISL_991930, EPI_ISL_991941, EPI_ISL_991953, EPI_ISL_991956, EPI_ISL_991964 to 991965, EPI_ISL_991968, EPI_ISL_991970, EPI_ISL_991975, EPI_ISL_991979, EPI_ISL_991981, EPI_ISL_991984, EPI_ISL_991986 to 991989, EPI_ISL_991991 to 991993, EPI_ISL_991995, EPI_ISL_991998, EPI_ISL_992003, EPI_ISL_992008, EPI_ISL_992015, EPI_ISL_992017, EPI_ISL_992025 to 992026, EPI_ISL_992030, EPI_ISL_992032, EPI_ISL_992038, EPI_ISL_992044, EPI_ISL_992048, EPI_ISL_992057 to 992059, EPI_ISL_992061, EPI_ISL_992063 to 992064, EPI_ISL_992066, EPI_ISL_992069, EPI_ISL_992071, EPI_ISL_992073 to 992074, EPI_ISL_992077, EPI_ISL_992079 to 992083, EPI_ISL_992085, EPI_ISL_992089, EPI_ISL_992091 to 992092, EPI_ISL_992100 to 992101, EPI_ISL_992103, EPI_ISL_992105, EPI_ISL_992109, EPI_ISL_992111, EPI_ISL_992116 to 992119, EPI_ISL_992128, EPI_ISL_992130 to 992131, EPI_ISL_992138, EPI_ISL_992143 to 992144, EPI_ISL_992146 to 992147, EPI_ISL_992149, EPI_ISL_992151 to 992153, EPI_ISL_992155 to 992156, EPI_ISL_992159, EPI_ISL_992166, EPI_ISL_992172 to 992173, EPI_ISL_992177 to 992178, EPI_ISL_992180 to 992181, EPI_ISL_992183, EPI_ISL_992187 to 992188, EPI_ISL_992196 to 992198, EPI_ISL_992204 to 992207, EPI_ISL_992214, EPI_ISL_992218 to 992219, EPI_ISL_992225 to 992226, EPI_ISL_992229, EPI_ISL_992234, EPI_ISL_992236, EPI_ISL_992241, EPI_ISL_992243, EPI_ISL_992246, EPI_ISL_992250, EPI_ISL_992252, EPI_ISL_992258, EPI_ISL_992261 to 992262, EPI_ISL_992265, EPI_ISL_992267, EPI_ISL_992269, EPI_ISL_992271, EPI_ISL_992273, EPI_ISL_992275 to 992276, EPI_ISL_992279, EPI_ISL_992283, EPI_ISL_992286 to 992288, EPI_ISL_992294 to 992295, EPI_ISL_992303, EPI_ISL_992305 to 992306, EPI_ISL_992309, EPI_ISL_992311 to 992312, EPI_ISL_992314, EPI_ISL_992317, EPI_ISL_992321, EPI_ISL_992330 to 992331, EPI_ISL_992335 to 992337, EPI_ISL_992344 to 992345, EPI_ISL_992348 to 992350, EPI_ISL_992353 to 992354, EPI_ISL_992364 to 992367, EPI_ISL_992370 to 992371, EPI_ISL_992373, EPI_ISL_992375 to 992379, EPI_ISL_992382, EPI_ISL_992385, EPI_ISL_992389, EPI_ISL_992391, EPI_ISL_992393 to 992394, EPI_ISL_992396 to 992400, EPI_ISL_992403 to 992404, EPI_ISL_992406 to 992408, EPI_ISL_992412 to 992414, EPI_ISL_992416, EPI_ISL_992418, EPI_ISL_992421 to 992422, EPI_ISL_992425 to 992426, EPI_ISL_992428 to 992433, EPI_ISL_992435 to 992436, EPI_ISL_992440, EPI_ISL_992445 to 992446, EPI_ISL_992448 to 992449, EPI_ISL_992453 to 992455, EPI_ISL_992457, EPI_ISL_992459 to 992460, EPI_ISL_992463 to 992466, EPI_ISL_992469 to 992471, EPI_ISL_992473, EPI_ISL_992475 to 992477, EPI_ISL_992479 to 992480, EPI_ISL_992485 to 992488, EPI_ISL_992490 to 992493, EPI_ISL_992495, EPI_ISL_992498, EPI_ISL_992500, EPI_ISL_992503 to 992504, EPI_ISL_992506 to 992510, EPI_ISL_992514, EPI_ISL_992517, EPI_ISL_992519 to 992520, EPI_ISL_992523, EPI_ISL_992528, EPI_ISL_992531, EPI_ISL_992533, EPI_ISL_992535 to 992536, EPI_ISL_992538, EPI_ISL_992540 to 992543, EPI_ISL_992546 to 992548, EPI_ISL_992550, EPI_ISL_992554 to 992555, EPI_ISL_992557 to 992560, EPI_ISL_992562 to 992565, EPI_ISL_992567 to 992569, EPI_ISL_992571 to 992575, EPI_ISL_992577 to 992578, EPI_ISL_992580, EPI_ISL_992582 to 992585, EPI_ISL_992588, EPI_ISL_992593, EPI_ISL_992595 to 992598, EPI_ISL_992601 to 992604, EPI_ISL_992606 to 992607, EPI_ISL_992609 to 992610, EPI_ISL_992612 to 992615, EPI_ISL_992618, EPI_ISL_992621 to 992624, EPI_ISL_992628 to 992631, EPI_ISL_992633, EPI_ISL_992635 to 992639, EPI_ISL_992641 to 992644, EPI_ISL_992646 to 992649, EPI_ISL_992652 to 992653, EPI_ISL_992656, EPI_ISL_992662 to 992663, EPI_ISL_992665, EPI_ISL_992668, EPI_ISL_992671 to 992674, EPI_ISL_992679, EPI_ISL_992681 to 992683, EPI_ISL_992685 to 992686, EPI_ISL_992688 to 992691, EPI_ISL_992693, EPI_ISL_992697 to 992698, EPI_ISL_992701, EPI_ISL_992703, EPI_ISL_992705, EPI_ISL_992707 to 992708, EPI_ISL_992710, EPI_ISL_992712 to 992714, EPI_ISL_992717 to 992726, EPI_ISL_992728, EPI_ISL_992730, EPI_ISL_992732 to 992738, EPI_ISL_992740 to 992743, EPI_ISL_992746, EPI_ISL_992748, EPI_ISL_992750 to 992751, EPI_ISL_992757, EPI_ISL_992759 to 992766, EPI_ISL_992769 to 992772, EPI_ISL_992774, EPI_ISL_992776, EPI_ISL_992778 to 992779, EPI_ISL_992781 to 992783, EPI_ISL_992785 to 992786, EPI_ISL_992788, EPI_ISL_992790, EPI_ISL_992792 to 992793, EPI_ISL_992795 to 992797, EPI_ISL_992800, EPI_ISL_992802 to 992804, EPI_ISL_992806, EPI_ISL_992808, EPI_ISL_992811 to 992812, EPI_ISL_992814, EPI_ISL_992818 to 992824, EPI_ISL_992826, EPI_ISL_992828, EPI_ISL_992830 to 992832, EPI_ISL_992836 to 992839, EPI_ISL_992842, EPI_ISL_992844, EPI_ISL_992846 to 992849, EPI_ISL_992851, EPI_ISL_992854 to 992857, EPI_ISL_992860, EPI_ISL_992862 to 992865, EPI_ISL_992867 to 992869, EPI_ISL_992871 to 992872, EPI_ISL_992876, EPI_ISL_992879 to 992881, EPI_ISL_992883, EPI_ISL_992885 to 992892, EPI_ISL_992896, EPI_ISL_992899 to 992900, EPI_ISL_992902 to 992904, EPI_ISL_992907 to 992909, EPI_ISL_992911, EPI_ISL_992913 to 992917, EPI_ISL_992919 to 992921, EPI_ISL_992924 to 992925, EPI_ISL_992927 to 992929, EPI_ISL_992934 to 992940, EPI_ISL_992942 to 992943, EPI_ISL_992945 to 992946, EPI_ISL_992952 to 992954, EPI_ISL_992957, EPI_ISL_992959, EPI_ISL_992964, EPI_ISL_992968, EPI_ISL_992970, EPI_ISL_992973 to 992984, EPI_ISL_992990 to 992992, EPI_ISL_992996, EPI_ISL_992998 to 993000, EPI_ISL_993003, EPI_ISL_993005 to 993006, EPI_ISL_993008, EPI_ISL_993011, EPI_ISL_993013 to 993015, EPI_ISL_993017 to 993019, EPI_ISL_993021 to 993022, EPI_ISL_993025, EPI_ISL_993028 to 993031, EPI_ISL_993033, EPI_ISL_993038, EPI_ISL_993042 to 993044, EPI_ISL_993046, EPI_ISL_993048 to 993050, EPI_ISL_993059, EPI_ISL_993062, EPI_ISL_993066, EPI_ISL_993069, EPI_ISL_993071 to 993072, EPI_ISL_993075, EPI_ISL_993078, EPI_ISL_993081 to 993082, EPI_ISL_993105 to 993106, EPI_ISL_993109 to 993111, EPI_ISL_993113, EPI_ISL_993127, EPI_ISL_993133, EPI_ISL_993149, EPI_ISL_993152, EPI_ISL_993154, EPI_ISL_993165, EPI_ISL_993168, EPI_ISL_993171, EPI_ISL_993176, EPI_ISL_993184, EPI_ISL_993187, EPI_ISL_993213, EPI_ISL_993221, EPI_ISL_993225, EPI_ISL_993239, EPI_ISL_993245, EPI_ISL_993266, EPI_ISL_993282, EPI_ISL_993291, EPI_ISL_993306, EPI_ISL_993309, EPI_ISL_993311, EPI_ISL_993316, EPI_ISL_993321, EPI_ISL_993325, EPI_ISL_993327, EPI_ISL_993334, EPI_ISL_993338, EPI_ISL_993341, EPI_ISL_993362, EPI_ISL_993370, EPI_ISL_993383, EPI_ISL_993385, EPI_ISL_993390, EPI_ISL_993393, EPI_ISL_993397, EPI_ISL_993403, EPI_ISL_993406, EPI_ISL_993410 to 993411, EPI_ISL_994293, EPI_ISL_994303, EPI_ISL_994306 to 994308, EPI_ISL_994313, EPI_ISL_994320 to 994321, EPI_ISL_994323 to 994325, EPI_ISL_994329 to 994330, EPI_ISL_994335, EPI_ISL_994339, EPI_ISL_994342, EPI_ISL_994348, EPI_ISL_994351, EPI_ISL_994355, EPI_ISL_994357, EPI_ISL_994361, EPI_ISL_994364, EPI_ISL_994368, EPI_ISL_994375, EPI_ISL_994379, EPI_ISL_994385, EPI_ISL_994392 to 994394, EPI_ISL_994396, EPI_ISL_994400, EPI_ISL_994402, EPI_ISL_994404, EPI_ISL_994411, EPI_ISL_994414, EPI_ISL_994416, EPI_ISL_994419, EPI_ISL_994422 to 994424, EPI_ISL_994428, EPI_ISL_994448, EPI_ISL_994451, EPI_ISL_994455, EPI_ISL_994460 to 994463, EPI_ISL_994467 to 994468, EPI_ISL_994473 to 994474, EPI_ISL_994478, EPI_ISL_994482, EPI_ISL_994484, EPI_ISL_994488 to 994489, EPI_ISL_994492, EPI_ISL_994495, EPI_ISL_994498 to 994499, EPI_ISL_994506 to 994507, EPI_ISL_994513, EPI_ISL_994515, EPI_ISL_994519 to 994520, EPI_ISL_994522, EPI_ISL_994525, EPI_ISL_994527, EPI_ISL_994531, EPI_ISL_994534 to 994535, EPI_ISL_994538 to 994540, EPI_ISL_994545, EPI_ISL_994559, EPI_ISL_994564, EPI_ISL_994566 to 994567, EPI_ISL_994572, EPI_ISL_994574 to 994575, EPI_ISL_994578 to 994580, EPI_ISL_994588, EPI_ISL_994590 to 994591, EPI_ISL_994593 to 994595, EPI_ISL_994599, EPI_ISL_994604, EPI_ISL_994607, EPI_ISL_994622 to 994624, EPI_ISL_994629, EPI_ISL_994633, EPI_ISL_994635 to 994637, EPI_ISL_994647, EPI_ISL_994649, EPI_ISL_1006540, EPI_ISL_1006542, EPI_ISL_1006937 to 1006938, EPI_ISL_1007438 to 1007439, EPI_ISL_1007441, EPI_ISL_1007444, EPI_ISL_1007550 to 1007551, EPI_ISL_1007555 to 1007556, EPI_ISL_1007558, EPI_ISL_1007563 to 1007564, EPI_ISL_1007566, EPI_ISL_1007568 to 1007571, EPI_ISL_1007573, EPI_ISL_1007580 to 1007582, EPI_ISL_1011757 to 1011759, EPI_ISL_1011761 to 1011777, EPI_ISL_1011780 to 1011793, EPI_ISL_1011795, EPI_ISL_1011797 to 1011799, EPI_ISL_1011801 to 1011807, EPI_ISL_1011809 to 1011813, EPI_ISL_1011815, EPI_ISL_1011817 to 1011825, EPI_ISL_1011827 to 1011844, EPI_ISL_1011847 to 1011862, EPI_ISL_1011864 to 1011869, EPI_ISL_1011871 to 1011879, EPI_ISL_1011881 to 1011904, EPI_ISL_1011906 to 1011919, EPI_ISL_1011921 to 1011924, EPI_ISL_1011926 to 1011936, EPI_ISL_1011938 to 1011939, EPI_ISL_1011941 to 1011943, EPI_ISL_1011945 to 1011946, EPI_ISL_1011948 to 1011972, EPI_ISL_1011974 to 1011975, EPI_ISL_1011977 to 1011978, EPI_ISL_1011980 to 1011982, EPI_ISL_1011984 to 1011990, EPI_ISL_1011992 to 1011995, EPI_ISL_1011997 to 1011999, EPI_ISL_1012001 to 1012004, EPI_ISL_1012006 to 1012010, EPI_ISL_1012012 to 1012022, EPI_ISL_1012024 to 1012025, EPI_ISL_1012027 to 1012032, EPI_ISL_1012034 to 1012044, EPI_ISL_1012046, EPI_ISL_1012048 to 1012050, EPI_ISL_1012052 to 1012084, EPI_ISL_1012086 to 1012093, EPI_ISL_1012095 to 1012098, EPI_ISL_1012551 to 1012557, EPI_ISL_1012559 to 1012562, EPI_ISL_1012565 to 1012567, EPI_ISL_1012569, EPI_ISL_1012571 to 1012578, EPI_ISL_1012580 to 1012582, EPI_ISL_1012584 to 1012585, EPI_ISL_1012587 to 1012588, EPI_ISL_1012590, EPI_ISL_1012593, EPI_ISL_1012599, EPI_ISL_1012601, EPI_ISL_1012603 to 1012607, EPI_ISL_1012673 to 1012675, EPI_ISL_1012679 to 1012680, EPI_ISL_1012682 to 1012683, EPI_ISL_1012685, EPI_ISL_1012687 to 1012689, EPI_ISL_1012692 to 1012703, EPI_ISL_1012706 to 1012709, EPI_ISL_1012712 to 1012717, EPI_ISL_1012721, EPI_ISL_1012723 to 1012725, EPI_ISL_1012727 to 1012733, EPI_ISL_1012736, EPI_ISL_1012739 to 1012740, EPI_ISL_1012742 to 1012743, EPI_ISL_1012745 to 1012747, EPI_ISL_1012751 to 1012753, EPI_ISL_1012756, EPI_ISL_1012759, EPI_ISL_1012763 to 1012765, EPI_ISL_1012767 to 1012769, EPI_ISL_1012774 to 1012776, EPI_ISL_1012782 to 1012783, EPI_ISL_1012785 to 1012786, EPI_ISL_1012788 to 1012791, EPI_ISL_1012793, EPI_ISL_1012795, EPI_ISL_1012799, EPI_ISL_1012807, EPI_ISL_1012809, EPI_ISL_1012811 to 1012813, EPI_ISL_1012816, EPI_ISL_1012820 to 1012825, EPI_ISL_1012827 to 1012828, EPI_ISL_1012832, EPI_ISL_1012834 to 1012835, EPI_ISL_1012837 to 1012838, EPI_ISL_1012840, EPI_ISL_1012842 to 1012845, EPI_ISL_1012848, EPI_ISL_1012851 to 1012855, EPI_ISL_1012857, EPI_ISL_1012859 to 1012861, EPI_ISL_1012863, EPI_ISL_1012867, EPI_ISL_1012869, EPI_ISL_1018329 to 1018330, EPI_ISL_1018332 to 1018342, EPI_ISL_1018345 to 1018372, EPI_ISL_1018374, EPI_ISL_1018376 to 1018382, EPI_ISL_1018384 to 1018392, EPI_ISL_1018394 to 1018398, EPI_ISL_1018400 to 1018416, EPI_ISL_1018418 to 1018430, EPI_ISL_1018432 to 1018437, EPI_ISL_1018439 to 1018473, EPI_ISL_1018475 to 1018478, EPI_ISL_1018480 to 1018485, EPI_ISL_1018487 to 1018497, EPI_ISL_1018499 to 1018502, EPI_ISL_1018504 to 1018506, EPI_ISL_1018509 to 1018514, EPI_ISL_1018516 to 1018520, EPI_ISL_1018522 to 1018524, EPI_ISL_1018526 to 1018538, EPI_ISL_1018541 to 1018542, EPI_ISL_1018544 to 1018558, EPI_ISL_1018560 to 1018561, EPI_ISL_1018563 to 1018568, EPI_ISL_1018570 to 1018578, EPI_ISL_1018580 to 1018609, EPI_ISL_1018611 to 1018618, EPI_ISL_1018620 to 1018622, EPI_ISL_1018624 to 1018640, EPI_ISL_1018643 to 1018648, EPI_ISL_1018650 to 1018657, EPI_ISL_1018659 to 1018666, EPI_ISL_1018668, EPI_ISL_1018670 to 1018679, EPI_ISL_1018681 to 1018688, EPI_ISL_1018950, EPI_ISL_1018952, EPI_ISL_1018954 to 1018959,

EPI_ISL_1018962 to 1018968, EPI_ISL_1018970 to 1018973, EPI_ISL_1018975 to 1018981, EPI_ISL_1018983 to 1018993, EPI_ISL_1018995 to 1018998, EPI_ISL_1019000 to 1019003, EPI_ISL_1019005 to 1019019, EPI_ISL_1019021 to 1019031, EPI_ISL_1019033 to 1019037, EPI_ISL_1019039 to 1019044, EPI_ISL_1019046 to 1019049, EPI_ISL_1019051 to 1019053, EPI_ISL_1019055 to 1019063, EPI_ISL_1019065 to 1019079, EPI_ISL_1019082 to 1019093, EPI_ISL_1019096 to 1019100, EPI_ISL_1019102 to 1019104, EPI_ISL_1019106, EPI_ISL_1019109 to 1019117, EPI_ISL_1019119 to 1019131, EPI_ISL_1019133 to 1019153, EPI_ISL_1019155 to 1019164, EPI_ISL_1019166, EPI_ISL_1019169 to 1019170, EPI_ISL_1019172 to 1019175, EPI_ISL_1019178 to 1019179, EPI_ISL_1019182 to 1019185, EPI_ISL_1019187 to 1019188, EPI_ISL_1019190 to 1019215, EPI_ISL_1019217 to 1019219, EPI_ISL_1019221 to 1019222, EPI_ISL_1019224, EPI_ISL_1019226 to 1019235, EPI_ISL_1019237, EPI_ISL_1019239, EPI_ISL_1019241 to 1019243, EPI_ISL_1019245, EPI_ISL_1019247 to 1019251, EPI_ISL_1019253 to 1019264, EPI_ISL_1019266 to 1019271, EPI_ISL_1019273 to 1019276, EPI_ISL_1019278 to 1019285, EPI_ISL_1019482 to 1019483, EPI_ISL_1019485, EPI_ISL_1019487 to 1019491, EPI_ISL_1019493 to 1019497, EPI_ISL_1019499 to 1019500, EPI_ISL_1019502 to 1019503, EPI_ISL_1033537 to 1033540, EPI_ISL_1033543, EPI_ISL_1033546 to 1033548, EPI_ISL_1033551 to 1033555, EPI_ISL_1033557 to 1033562, EPI_ISL_1033564 to 1033565, EPI_ISL_1034141 to 1034142

see above	Lighthouse Lab in Alderley Park	Wellcome Sanger Institute for the COVID-19 Genomics UK (COG-UK) Consortium	Cordelia Langford; David K. Jackson; Dominic Kwiatkowski; Ewan Harrison; Ian Johnston; Jacquelyn Wynn; Jeffrey Barrett; John Sillitoe on behalf of the Wellcome Sanger Institute COVID-19 Surveillance Team; John Sillitoe on behalf of the Wellcome Sanger Institute COVID-19 Surveillance Team (http://www.sanger.ac.uk/covid-team); Mairead Hyland; Roberto Amato; Sonia Goncalves; The Lighthouse Lab in Alderley Park and Alex Alderton

EPI_ISL_551285, EPI_ISL_551289 to 551293, EPI_ISL_551295 to 551296, EPI_ISL_551298 to 551299, EPI_ISL_551301 to 551302, EPI_ISL_551304 to 551309, EPI_ISL_551311, EPI_ISL_551313 to 551320, EPI_ISL_551322 to 551326, EPI_ISL_551328, EPI_ISL_551330, EPI_ISL_551332 to 551335, EPI_ISL_551338 to 551348, EPI_ISL_551350 to 551351, EPI_ISL_551354 to 551357, EPI_ISL_551359 to 551365, EPI_ISL_551367 to 551373, EPI_ISL_551375 to 551379, EPI_ISL_551381 to 551383, EPI_ISL_551385 to 551397, EPI_ISL_551399, EPI_ISL_551401, EPI_ISL_551403, EPI_ISL_551405, EPI_ISL_551407 to 551408, EPI_ISL_551411, EPI_ISL_551413, EPI_ISL_551416 to 551427, EPI_ISL_551429 to 551430, EPI_ISL_551433, EPI_ISL_551435 to 551441, EPI_ISL_551443 to 551452, EPI_ISL_551454 to 551469, EPI_ISL_551471 to 551478, EPI_ISL_551480 to 551482, EPI_ISL_551485 to 551486, EPI_ISL_551488 to 551495, EPI_ISL_551497 to 551499, EPI_ISL_551501, EPI_ISL_551503 to 551510, EPI_ISL_551512, EPI_ISL_551514 to 551516, EPI_ISL_551518, EPI_ISL_551523, EPI_ISL_551525 to 551526, EPI_ISL_551528 to 551529, EPI_ISL_551531 to 551533, EPI_ISL_551535 to 551560, EPI_ISL_551562 to 551565, EPI_ISL_551567 to 551573, EPI_ISL_551735 to 551736, EPI_ISL_551740, EPI_ISL_551742 to 551743, EPI_ISL_551749, EPI_ISL_551755, EPI_ISL_551759 to 551764, EPI_ISL_551772, EPI_ISL_551776, EPI_ISL_551779 to 551780, EPI_ISL_551784 to 551785, EPI_ISL_551789, EPI_ISL_551795 to 551796, EPI_ISL_551798 to 551799, EPI_ISL_551801, EPI_ISL_551803 to 551804, EPI_ISL_551806 to 551808, EPI_ISL_551810 to 551811, EPI_ISL_551815, EPI_ISL_551818 to 551819, EPI_ISL_551825 to 551827, EPI_ISL_551832, EPI_ISL_551834, EPI_ISL_552446, EPI_ISL_552449, EPI_ISL_552463 to 552464, EPI_ISL_552466, EPI_ISL_552468, EPI_ISL_552473, EPI_ISL_552476, EPI_ISL_552479, EPI_ISL_552481, EPI_ISL_552486, EPI_ISL_552488, EPI_ISL_552506 to 552508, EPI_ISL_552512, EPI_ISL_552515 to 552516, EPI_ISL_552523, EPI_ISL_552526 to 552527, EPI_ISL_552530, EPI_ISL_552533, EPI_ISL_552539 to 552540, EPI_ISL_552556, EPI_ISL_552562, EPI_ISL_552565 to 552566, EPI_ISL_552574, EPI_ISL_552577, EPI_ISL_552579 to 552580, EPI_ISL_552583, EPI_ISL_552587, EPI_ISL_552590 to 552591, EPI_ISL_552595 to 552596, EPI_ISL_552598, EPI_ISL_552608, EPI_ISL_552614, EPI_ISL_552617, EPI_ISL_552622, EPI_ISL_552629, EPI_ISL_552639, EPI_ISL_552642, EPI_ISL_552644, EPI_ISL_552646, EPI_ISL_552655, EPI_ISL_552659, EPI_ISL_552665, EPI_ISL_552669, EPI_ISL_552671, EPI_ISL_552679, EPI_ISL_552681, EPI_ISL_552686, EPI_ISL_552689, EPI_ISL_552699, EPI_ISL_552703, EPI_ISL_552706 to 552707, EPI_ISL_552712 to 552713, EPI_ISL_552721, EPI_ISL_552723 to 552724, EPI_ISL_552726, EPI_ISL_552728, EPI_ISL_552731 to 552732, EPI_ISL_552735, EPI_ISL_552741 to 552742, EPI_ISL_552746, EPI_ISL_552760, EPI_ISL_552773 to 552774, EPI_ISL_552776, EPI_ISL_552779 to 552780, EPI_ISL_552782, EPI_ISL_552785, EPI_ISL_552794, EPI_ISL_552796 to 552798, EPI_ISL_552801, EPI_ISL_552803 to 552808, EPI_ISL_552812 to 552813, EPI_ISL_552815, EPI_ISL_552817, EPI_ISL_552821 to 552822, EPI_ISL_552824 to 552825, EPI_ISL_552827 to 552837, EPI_ISL_552839 to 552840, EPI_ISL_552843 to 552851, EPI_ISL_552853, EPI_ISL_552855 to 552858, EPI_ISL_552860 to 552866, EPI_ISL_552868, EPI_ISL_552870 to 552873, EPI_ISL_552878, EPI_ISL_552881 to 552896, EPI_ISL_552899, EPI_ISL_552901 to 552904, EPI_ISL_552906 to 552909, EPI_ISL_552911 to 552914, EPI_ISL_552917 to 552919, EPI_ISL_552921 to 552923, EPI_ISL_552925 to 552931, EPI_ISL_552933 to 552935, EPI_ISL_552937, EPI_ISL_552940 to 552942, EPI_ISL_552944 to 552946, EPI_ISL_552951, EPI_ISL_552954 to 552958, EPI_ISL_552964 to 552966, EPI_ISL_552968 to 552971, EPI_ISL_552974, EPI_ISL_552976 to 552978, EPI_ISL_552980 to 552986, EPI_ISL_552988 to 552989, EPI_ISL_552991 to 552994, EPI_ISL_552996, EPI_ISL_552998 to 553001, EPI_ISL_553003 to 553006, EPI_ISL_553269, EPI_ISL_553271, EPI_ISL_553273 to 553274, EPI_ISL_553277 to 553278, EPI_ISL_553280 to 553292, EPI_ISL_553297 to 553303, EPI_ISL_553305, EPI_ISL_553307 to 553319, EPI_ISL_553321 to 553322, EPI_ISL_553325 to 553326, EPI_ISL_553328 to 553331, EPI_ISL_553333 to 553335, EPI_ISL_553337 to 553346, EPI_ISL_553348, EPI_ISL_553351 to 553355, EPI_ISL_553358, EPI_ISL_553360 to 553374, EPI_ISL_553376 to 553380, EPI_ISL_553382 to 553386, EPI_ISL_553388, EPI_ISL_553390 to 553391, EPI_ISL_553393 to 553397, EPI_ISL_553399 to 553406, EPI_ISL_553408 to 553419, EPI_ISL_553422 to 553426, EPI_ISL_553428 to 553433, EPI_ISL_553435 to 553446, EPI_ISL_553448 to 553452, EPI_ISL_553456 to 553457, EPI_ISL_553459 to 553465, EPI_ISL_553467, EPI_ISL_553469 to 553470, EPI_ISL_553472, EPI_ISL_553474 to 553480, EPI_ISL_553482 to 553484, EPI_ISL_553486 to 553489, EPI_ISL_553491 to 553496, EPI_ISL_553498 to 553509, EPI_ISL_553511 to 553513, EPI_ISL_553515 to 553519, EPI_ISL_553521 to 553526, EPI_ISL_553528 to 553529, EPI_ISL_553531 to 553538, EPI_ISL_553540, EPI_ISL_553543 to 553547, EPI_ISL_553549, EPI_ISL_553551 to 553554, EPI_ISL_553556 to 553564, EPI_ISL_553566, EPI_ISL_553569 to 553573, EPI_ISL_553575 to 553583, EPI_ISL_553585 to 553600, EPI_ISL_553602 to 553625, EPI_ISL_553627, EPI_ISL_553629 to 553639, EPI_ISL_553641 to 553647, EPI_ISL_553651, EPI_ISL_553653, EPI_ISL_553655 to 553657, EPI_ISL_553660 to 553663, EPI_ISL_553666, EPI_ISL_553668 to 553673, EPI_ISL_553675 to 553681, EPI_ISL_553683, EPI_ISL_553685 to 553688, EPI_ISL_553691, EPI_ISL_553693 to 553698, EPI_ISL_553702, EPI_ISL_553705, EPI_ISL_553707 to 553713, EPI_ISL_553717 to 553718, EPI_ISL_553720 to 553721, EPI_ISL_553723 to 553725, EPI_ISL_553729 to 553730, EPI_ISL_553733, EPI_ISL_553735 to 553737, EPI_ISL_553739 to 553740, EPI_ISL_553742, EPI_ISL_553744 to 553745, EPI_ISL_553747 to 553752, EPI_ISL_553755 to 553756, EPI_ISL_553760 to 553773, EPI_ISL_553775 to 553777, EPI_ISL_553779, EPI_ISL_553781 to 553783, EPI_ISL_553785 to 553789, EPI_ISL_553791 to 553793, EPI_ISL_553795 to 553802, EPI_ISL_553804 to 553809, EPI_ISL_553813 to 553816, EPI_ISL_553819 to 553830, EPI_ISL_553832, EPI_ISL_553834, EPI_ISL_553836 to 553838, EPI_ISL_553840, EPI_ISL_553842 to 553845, EPI_ISL_553847 to 553850, EPI_ISL_553852 to 553853, EPI_ISL_553855 to 553860, EPI_ISL_553862 to 553864, EPI_ISL_553867, EPI_ISL_553869 to 553870, EPI_ISL_553872 to 553873, EPI_ISL_553875 to 553878, EPI_ISL_553881 to 553884, EPI_ISL_553887 to 553888, EPI_ISL_553890 to 553892, EPI_ISL_553895, EPI_ISL_553899 to 553900, EPI_ISL_553902, EPI_ISL_553904, EPI_ISL_553906, EPI_ISL_553908 to 553912, EPI_ISL_553915 to 553919, EPI_ISL_553921 to 553923, EPI_ISL_553925 to 553926, EPI_ISL_553928, EPI_ISL_553930 to 553932, EPI_ISL_553935 to 553938, EPI_ISL_553940 to 553945, EPI_ISL_553947 to 553951, EPI_ISL_553953, EPI_ISL_553958 to 553960, EPI_ISL_553962 to 553964, EPI_ISL_553966 to 553967, EPI_ISL_553969, EPI_ISL_553973, EPI_ISL_553975 to 553982, EPI_ISL_553984, EPI_ISL_553986, EPI_ISL_553988, EPI_ISL_553991 to 553992, EPI_ISL_553995 to 553996, EPI_ISL_553998 to 554001, EPI_ISL_554006 to 554008, EPI_ISL_554010, EPI_ISL_554013 to 554014, EPI_ISL_554016 to 554019, EPI_ISL_554021 to 554023, EPI_ISL_554027, EPI_ISL_554029 to 554033, EPI_ISL_554035 to 554041, EPI_ISL_554044 to 554045, EPI_ISL_554047, EPI_ISL_554049 to 554053, EPI_ISL_554056, EPI_ISL_554059 to 554062, EPI_ISL_554064, EPI_ISL_554066, EPI_ISL_554068 to 554070, EPI_ISL_554072 to 554078, EPI_ISL_554081 to 554087, EPI_ISL_554090 to 554096, EPI_ISL_554098, EPI_ISL_554102, EPI_ISL_554104 to 554114, EPI_ISL_554116 to 554117, EPI_ISL_554122, EPI_ISL_554129 to 554130, EPI_ISL_554136, EPI_ISL_554155 to 554158, EPI_ISL_554164 to 554165, EPI_ISL_554170 to 554172, EPI_ISL_554174 to 554175, EPI_ISL_554188 to 554190, EPI_ISL_554192, EPI_ISL_554194, EPI_ISL_554196 to 554199, EPI_ISL_554202 to 554203, EPI_ISL_554207, EPI_ISL_554212, EPI_ISL_554215, EPI_ISL_554219, EPI_ISL_554225, EPI_ISL_554227, EPI_ISL_554229 to 554230, EPI_ISL_554233, EPI_ISL_554235, EPI_ISL_554238, EPI_ISL_554252, EPI_ISL_554257, EPI_ISL_554260, EPI_ISL_554263, EPI_ISL_554265 to 554266, EPI_ISL_554270, EPI_ISL_554275, EPI_ISL_554278 to 554280, EPI_ISL_554282 to 554283, EPI_ISL_554288, EPI_ISL_554292, EPI_ISL_554296, EPI_ISL_554298 to 554299, EPI_ISL_554301, EPI_ISL_554305, EPI_ISL_554309, EPI_ISL_554318, EPI_ISL_554320, EPI_ISL_554322 to 554324, EPI_ISL_554326 to 554330, EPI_ISL_554332, EPI_ISL_554334 to 554336, EPI_ISL_554339, EPI_ISL_554343 to 554345, EPI_ISL_554347 to 554349, EPI_ISL_554351 to 554352, EPI_ISL_554354 to 554357, EPI_ISL_554360 to 554365, EPI_ISL_554368 to 554374, EPI_ISL_554376 to 554377, EPI_ISL_554379 to 554381, EPI_ISL_554383 to 554384, EPI_ISL_554387 to 554389, EPI_ISL_554391 to 554399, EPI_ISL_554401 to 554402, EPI_ISL_554404 to 554408, EPI_ISL_554410 to 554419, EPI_ISL_554421 to 554432, EPI_ISL_554434 to 554440, EPI_ISL_554442 to 554458, EPI_ISL_554460 to 554469, EPI_ISL_554471 to 554474, EPI_ISL_554476 to 554477, EPI_ISL_554480 to 554483, EPI_ISL_554485 to 554486, EPI_ISL_554488 to 554490, EPI_ISL_554493 to 554496, EPI_ISL_554499 to 554505, EPI_ISL_554507 to 554509, EPI_ISL_554511 to 554518, EPI_ISL_554520 to 554525, EPI_ISL_554527, EPI_ISL_554529 to 554536, EPI_ISL_554539 to 554540, EPI_ISL_554542 to 554547, EPI_ISL_554549 to 554558, EPI_ISL_554560 to 554573, EPI_ISL_554575 to 554580, EPI_ISL_554582 to 554583, EPI_ISL_554585 to 554590, EPI_ISL_554592, EPI_ISL_554594 to 554600, EPI_ISL_554602 to 554610, EPI_ISL_554612, EPI_ISL_554614 to 554618, EPI_ISL_554623 to 554626, EPI_ISL_554630, EPI_ISL_554640, EPI_ISL_554651, EPI_ISL_554656 to 554657, EPI_ISL_554664, EPI_ISL_554677, EPI_ISL_554680 to 554681, EPI_ISL_554686, EPI_ISL_554688 to 554689, EPI_ISL_554692, EPI_ISL_554694, EPI_ISL_554696 to 554697, EPI_ISL_554703, EPI_ISL_554708, EPI_ISL_554710, EPI_ISL_554716, EPI_ISL_554725 to 554727, EPI_ISL_554730, EPI_ISL_554735, EPI_ISL_554741, EPI_ISL_554743, EPI_ISL_554754 to 554757, EPI_ISL_554759, EPI_ISL_554767, EPI_ISL_554771 to 554772, EPI_ISL_554776 to 554778, EPI_ISL_554780, EPI_ISL_554782, EPI_ISL_554792, EPI_ISL_554794 to 554795, EPI_ISL_554797, EPI_ISL_554799, EPI_ISL_554802, EPI_ISL_554804, EPI_ISL_554808 to 554813, EPI_ISL_554815 to 554816, EPI_ISL_554820, EPI_ISL_554830, EPI_ISL_554832 to 554834, EPI_ISL_554836 to 554837, EPI_ISL_554841, EPI_ISL_554846, EPI_ISL_554853, EPI_ISL_554861 to 554867, EPI_ISL_554870 to 554881, EPI_ISL_554883 to 554885, EPI_ISL_554888 to 554896, EPI_ISL_554899 to 554905, EPI_ISL_554908 to 554915, EPI_ISL_554917 to 554922, EPI_ISL_554924 to 554928, EPI_ISL_554931 to 554932, EPI_ISL_554934 to 554935, EPI_ISL_554937 to 554948, EPI_ISL_554950 to 554953, EPI_ISL_554955 to 554956, EPI_ISL_554960 to 554961, EPI_ISL_554963, EPI_ISL_554967 to 554968, EPI_ISL_554971 to 554977, EPI_ISL_554981 to 554982, EPI_ISL_554985 to 554986, EPI_ISL_554988 to 554989, EPI_ISL_554991 to 554995, EPI_ISL_554997 to 554999, EPI_ISL_555001 to 555002, EPI_ISL_555004 to 555009, EPI_ISL_555013 to 555015, EPI_ISL_555017, EPI_ISL_555021 to 555022, EPI_ISL_555024 to 555030, EPI_ISL_555032 to 555035, EPI_ISL_555037 to 555040, EPI_ISL_555042 to 555055, EPI_ISL_555057 to 555061, EPI_ISL_555064 to 555067, EPI_ISL_555069, EPI_ISL_555071 to 555072, EPI_ISL_555074 to 555079, EPI_ISL_555081 to 555083, EPI_ISL_555085 to 555090, EPI_ISL_555092 to 555095, EPI_ISL_555098 to 555104, EPI_ISL_555106 to 555108, EPI_ISL_555110 to 555126, EPI_ISL_555130 to 555137, EPI_ISL_555139, EPI_ISL_555141 to 555143, EPI_ISL_555147, EPI_ISL_555149 to 555162, EPI_ISL_555164 to 555172, EPI_ISL_555174 to 555177, EPI_ISL_555179 to 555185, EPI_ISL_555187 to 555189, EPI_ISL_555191 to 555199, EPI_ISL_555201 to 555202, EPI_ISL_555204, EPI_ISL_555207 to 555218, EPI_ISL_555222 to 555236, EPI_ISL_555238 to 555242, EPI_ISL_555244 to 555246, EPI_ISL_555251 to 555258, EPI_ISL_555260 to 555265, EPI_ISL_555267 to 555273, EPI_ISL_555275, EPI_ISL_555277 to 555278, EPI_ISL_555280 to 555287, EPI_ISL_555289 to 555302, EPI_ISL_555305, EPI_ISL_555307 to 555313, EPI_ISL_555317 to 555325, EPI_ISL_555327 to 555330, EPI_ISL_555333 to 555336, EPI_ISL_555339 to 555352, EPI_ISL_555355 to 555356, EPI_ISL_555358, EPI_ISL_555360 to 555361, EPI_ISL_555363 to 555367, EPI_ISL_555370 to 555376, EPI_ISL_555378 to 555386, EPI_ISL_555388 to 555390, EPI_ISL_555392 to 555395, EPI_ISL_555397, EPI_ISL_555401 to 555410, EPI_ISL_555412 to 555418, EPI_ISL_555420 to 555421, EPI_ISL_555423 to 555431, EPI_ISL_555435 to 555436, EPI_ISL_555438 to 555439, EPI_ISL_555441 to 555445, EPI_ISL_555447 to 555450, EPI_ISL_555452 to 555454, EPI_ISL_555456 to 555458, EPI_ISL_555460 to 555463, EPI_ISL_555465, EPI_ISL_555468 to 555470, EPI_ISL_555473, EPI_ISL_555476 to 555492, EPI_ISL_555494 to 555499, EPI_ISL_555501, EPI_ISL_555503 to 555504, EPI_ISL_555506 to 555509, EPI_ISL_555512 to 555518, EPI_ISL_555520, EPI_ISL_555522 to 555535, EPI_ISL_555537 to 555539, EPI_ISL_555541 to 555548, EPI_ISL_555550 to 555553, EPI_ISL_555555 to 555556, EPI_ISL_555559 to 555567, EPI_ISL_555569 to 555570, EPI_ISL_555572, EPI_ISL_555574, EPI_ISL_555576 to 555577, EPI_ISL_555579 to 555583, EPI_ISL_555585 to 555587, EPI_ISL_555590 to 555596, EPI_ISL_555599 to 555605, EPI_ISL_555608, EPI_ISL_555610 to 555612, EPI_ISL_555615, EPI_ISL_555617 to 555630, EPI_ISL_555632 to 555639, EPI_ISL_555642 to 555649, EPI_ISL_555653 to 555659, EPI_ISL_555662, EPI_ISL_555664, EPI_ISL_555666 to 555686, EPI_ISL_555688, EPI_ISL_555690 to 555691, EPI_ISL_555693 to 555699, EPI_ISL_555701 to 555702, EPI_ISL_555704, EPI_ISL_555708 to 555713, EPI_ISL_555715, EPI_ISL_555720 to 555728, EPI_ISL_555730 to 555735, EPI_ISL_555737 to 555741, EPI_ISL_555743 to 555744, EPI_ISL_555747 to 555748, EPI_ISL_555750 to 555755, EPI_ISL_555757 to 555759, EPI_ISL_555761 to 555762, EPI_ISL_555764 to 555768, EPI_ISL_555770 to 555774, EPI_ISL_555776 to 555777, EPI_ISL_555779 to 555780, EPI_ISL_555782, EPI_ISL_555784 to 555793, EPI_ISL_555795 to 555806, EPI_ISL_555808, EPI_ISL_555810 to 555812, EPI_ISL_555814, EPI_ISL_555816 to 555818, EPI_ISL_555820 to 555821, EPI_ISL_555823 to 555825, EPI_ISL_555827, EPI_ISL_555830 to 555832, EPI_ISL_555834, EPI_ISL_555836 to 555842, EPI_ISL_555845 to 555847, EPI_ISL_555849 to 555852, EPI_ISL_555854 to 555863, EPI_ISL_555865 to 555867, EPI_ISL_555869 to 555874, EPI_ISL_555876, EPI_ISL_555878 to 555886, EPI_ISL_555888 to 555904, EPI_ISL_555907 to 555909, EPI_ISL_555911 to 555915, EPI_ISL_555917 to 555921, EPI_ISL_555923 to 555928, EPI_ISL_555930, EPI_ISL_555932 to 555938, EPI_ISL_555941 to 555944, EPI_ISL_555946, EPI_ISL_555949, EPI_ISL_555951 to 555952, EPI_ISL_555954, EPI_ISL_555956 to 555966, EPI_ISL_555968 to 555972, EPI_ISL_555974 to 555975, EPI_ISL_555977 to 555981, EPI_ISL_555983 to 555985, EPI_ISL_555987 to 555989, EPI_ISL_555991, EPI_ISL_555994 to 555996, EPI_ISL_555998 to 555999, EPI_ISL_556002 to 556004, EPI_ISL_556006, EPI_ISL_556008 to 556011, EPI_ISL_556013, EPI_ISL_556015 to 556020, EPI_ISL_556023, EPI_ISL_556025, EPI_ISL_556027 to 556028, EPI_ISL_556030 to 556038, EPI_ISL_556040, EPI_ISL_556042, EPI_ISL_556044 to 556045, EPI_ISL_556048 to 556057, EPI_ISL_556059 to 556062, EPI_ISL_556064 to 556065, EPI_ISL_556067 to 556068, EPI_ISL_556070 to 556074, EPI_ISL_556078, EPI_ISL_556080 to 556082, EPI_ISL_556084 to 556086, EPI_ISL_556088 to 556089, EPI_ISL_556099 to 556101, EPI_ISL_556103, EPI_ISL_556105 to 556108, EPI_ISL_556110 to 556111, EPI_ISL_556114, EPI_ISL_556118 to 556122, EPI_ISL_556125, EPI_ISL_556127 to 556132, EPI_ISL_556136, EPI_ISL_556142 to 556145, EPI_ISL_556147, EPI_ISL_556149, EPI_ISL_556153, EPI_ISL_556155, EPI_ISL_556157, EPI_ISL_556160 to 556162, EPI_ISL_556167 to 556169, EPI_ISL_556171, EPI_ISL_556174 to 556175, EPI_ISL_556179, EPI_ISL_556181, EPI_ISL_556185, EPI_ISL_556190, EPI_ISL_556193 to 556194, EPI_ISL_556196, EPI_ISL_556200, EPI_ISL_556202 to 556203, EPI_ISL_556206, EPI_ISL_556209 to 556210, EPI_ISL_556212, EPI_ISL_556215 to 556217, EPI_ISL_556219 to 556222, EPI_ISL_556225, EPI_ISL_556227 to 556231, EPI_ISL_556234, EPI_ISL_556238 to 556241, EPI_ISL_556243, EPI_ISL_556245 to 556247, EPI_ISL_556249, EPI_ISL_556252, EPI_ISL_556254, EPI_ISL_556256, EPI_ISL_556258, EPI_ISL_556260, EPI_ISL_556262, EPI_ISL_556264 to 556265, EPI_ISL_556272 to 556274, EPI_ISL_556278 to 556281, EPI_ISL_556283, EPI_ISL_556286, EPI_ISL_556288, EPI_ISL_556290, EPI_ISL_556292, EPI_ISL_556295, EPI_ISL_556297, EPI_ISL_556301 to 556303, EPI_ISL_556306 to 556307, EPI_ISL_556310 to 556312, EPI_ISL_556314 to 556315, EPI_ISL_556319 to 556320, EPI_ISL_556323, EPI_ISL_556325 to 556329, EPI_ISL_556331 to 556337, EPI_ISL_556339, EPI_ISL_556341 to 556343, EPI_ISL_556345 to 556347, EPI_ISL_556352, EPI_ISL_556355 to 556356, EPI_ISL_556358 to 556362, EPI_ISL_556365 to 556367, EPI_ISL_556369 to 556370, EPI_ISL_556373, EPI_ISL_556375 to 556377, EPI_ISL_556379 to 556381, EPI_ISL_556384 to 556387, EPI_ISL_556389 to 556400, EPI_ISL_556402, EPI_ISL_556405 to 556408, EPI_ISL_556410, EPI_ISL_556412 to 556422, EPI_ISL_556424, EPI_ISL_556426 to 556427, EPI_ISL_556429 to 556431, EPI_ISL_556436 to 556438, EPI_ISL_556440 to 556441, EPI_ISL_556443 to 556444, EPI_ISL_556448 to 556452, EPI_ISL_556454 to 556457, EPI_ISL_556460 to 556465, EPI_ISL_556467 to 556470, EPI_ISL_556473 to 556481, EPI_ISL_556483 to 556487, EPI_ISL_556489 to 556493, EPI_ISL_556496, EPI_ISL_556498 to 556499, EPI_ISL_556501 to 556504, EPI_ISL_556506 to 556507, EPI_ISL_556513 to 556515, EPI_ISL_556577, EPI_ISL_556580, EPI_ISL_556583, EPI_ISL_556588, EPI_ISL_556591 to 556592, EPI_ISL_556594 to 556595, EPI_ISL_556597 to 556600, EPI_ISL_556602, EPI_ISL_556604 to 556605, EPI_ISL_556609 to 556611, EPI_ISL_556613 to 556614, EPI_ISL_556619 to 556620, EPI_ISL_556623, EPI_ISL_556626, EPI_ISL_556629 to 556631, EPI_ISL_556633, EPI_ISL_556636, EPI_ISL_556985 to 556989, EPI_ISL_556991 to 556998, EPI_ISL_557001, EPI_ISL_557003 to 557010, EPI_ISL_557012 to 557013, EPI_ISL_557015, EPI_ISL_557017 to 557020, EPI_ISL_557022 to 557031, EPI_ISL_557034, EPI_ISL_557036 to 557039, EPI_ISL_557041, EPI_ISL_557044 to 557047, EPI_ISL_557049 to 557070, EPI_ISL_557072 to 557073, EPI_ISL_557075 to 557076, EPI_ISL_557078, EPI_ISL_557080, EPI_ISL_557082 to 557084, EPI_ISL_557086, EPI_ISL_557088 to 557093, EPI_ISL_557095, EPI_ISL_557097 to 557102, EPI_ISL_557104 to 557109, EPI_ISL_557111, EPI_ISL_557113 to 557116, EPI_ISL_557118, EPI_ISL_557120 to 557128, EPI_ISL_557130 to 557131, EPI_ISL_557133, EPI_ISL_557135 to 557139, EPI_ISL_557141, EPI_ISL_557143 to 557145, EPI_ISL_557147 to 557148, EPI_ISL_557150 to 557155, EPI_ISL_557157 to 557160, EPI_ISL_557162 to 557165, EPI_ISL_557351, EPI_ISL_557354, EPI_ISL_557356, EPI_ISL_557359, EPI_ISL_557361, EPI_ISL_557363, EPI_ISL_557373 to 557374, EPI_ISL_557378, EPI_ISL_557380 to 557381, EPI_ISL_557388, EPI_ISL_557390, EPI_ISL_557398 to 557399, EPI_ISL_557404 to 557405, EPI_ISL_557408, EPI_ISL_557411, EPI_ISL_557417, EPI_ISL_557419, EPI_ISL_557423, EPI_ISL_557425, EPI_ISL_557433 to 557434, EPI_ISL_557436, EPI_ISL_557439 to 557440, EPI_ISL_557446 to 557447, EPI_ISL_557449, EPI_ISL_557452 to 557454, EPI_ISL_557466, EPI_ISL_557468 to 557469, EPI_ISL_557471, EPI_ISL_557476, EPI_ISL_557481 to 557482, EPI_ISL_557484 to 557485, EPI_ISL_557487 to 557488, EPI_ISL_557491, EPI_ISL_557497, EPI_ISL_557499, EPI_ISL_557507, EPI_ISL_557511, EPI_ISL_557518, EPI_ISL_557523, EPI_ISL_557526 to 557527, EPI_ISL_557531, EPI_ISL_557533, EPI_ISL_557537, EPI_ISL_557539, EPI_ISL_557541, EPI_ISL_557543 to 557544, EPI_ISL_557551, EPI_ISL_557553 to 557554, EPI_ISL_557557, EPI_ISL_557559 to 557569, EPI_ISL_557571 to 557575, EPI_ISL_557577 to 557584, EPI_ISL_557586, EPI_ISL_557588 to 557594, EPI_ISL_557597 to 557603, EPI_ISL_557605 to 557606, EPI_ISL_557608 to 557611, EPI_ISL_557613 to 557614, EPI_ISL_557616 to 557624, EPI_ISL_557627 to 557628, EPI_ISL_557631 to 557642, EPI_ISL_557644 to 557646, EPI_ISL_557648 to 557654, EPI_ISL_557656 to 557657, EPI_ISL_557659, EPI_ISL_557661 to 557671, EPI_ISL_557673 to 557674, EPI_ISL_557677 to 557679, EPI_ISL_557682 to 557684, EPI_ISL_557686 to 557688, EPI_ISL_557690 to 557691, EPI_ISL_557693, EPI_ISL_557695, EPI_ISL_557697 to 557699, EPI_ISL_557702 to 557703, EPI_ISL_557705 to 557708, EPI_ISL_557710, EPI_ISL_557712, EPI_ISL_557714 to 557715, EPI_ISL_557717 to 557725, EPI_ISL_557727, EPI_ISL_557729, EPI_ISL_557731, EPI_ISL_557733 to 557738, EPI_ISL_557744, EPI_ISL_557747, EPI_ISL_557749, EPI_ISL_557751, EPI_ISL_557754 to 557756, EPI_ISL_557759, EPI_ISL_557761 to 557763, EPI_ISL_557769 to 557770, EPI_ISL_557772 to 557773, EPI_ISL_557776, EPI_ISL_557779 to 557780, EPI_ISL_557785, EPI_ISL_557787, EPI_ISL_557790, EPI_ISL_557794, EPI_ISL_557797 to 557799, EPI_ISL_557803 to 557806, EPI_ISL_557809 to 557810, EPI_ISL_557813 to 557815, EPI_ISL_557818 to 557820, EPI_ISL_557825 to 557826, EPI_ISL_557831, EPI_ISL_557833 to 557837, EPI_ISL_557839, EPI_ISL_557841 to 557842, EPI_ISL_557844, EPI_ISL_557846, EPI_ISL_557850 to 557851, EPI_ISL_557855 to 557857, EPI_ISL_557860 to 557863, EPI_ISL_557872 to 557875, EPI_ISL_557887 to 557888, EPI_ISL_557891 to 557892, EPI_ISL_557897, EPI_ISL_557901, EPI_ISL_557903 to 557904, EPI_ISL_557906, EPI_ISL_557912, EPI_ISL_557914, EPI_ISL_557916, EPI_ISL_557920, EPI_ISL_557922 to 557924, EPI_ISL_557928 to 557932, EPI_ISL_557934 to 557935, EPI_ISL_557939, EPI_ISL_557943, EPI_ISL_557945 to 557946, EPI_ISL_557950 to 557951, EPI_ISL_557955, EPI_ISL_557960 to 557962, EPI_ISL_557968, EPI_ISL_557971, EPI_ISL_557973, EPI_ISL_557975, EPI_ISL_557978, EPI_ISL_557980 to 557983, EPI_ISL_557985 to 557987, EPI_ISL_557990 to 557996, EPI_ISL_557998, EPI_ISL_558002 to 558005, EPI_ISL_558007 to 558015, EPI_ISL_558018 to 558019, EPI_ISL_558022, EPI_ISL_558025, EPI_ISL_558027, EPI_ISL_558029, EPI_ISL_558032 to 558034, EPI_ISL_558036, EPI_ISL_558040, EPI_ISL_558042 to 558051, EPI_ISL_558053 to 558054, EPI_ISL_558057, EPI_ISL_558059 to 558063, EPI_ISL_558066 to 558067, EPI_ISL_558070, EPI_ISL_558072, EPI_ISL_558074 to 558078, EPI_ISL_558080 to 558082, EPI_ISL_558084 to 558086, EPI_ISL_558088, EPI_ISL_558090 to 558095, EPI_ISL_558097 to 558102, EPI_ISL_558105, EPI_ISL_558108, EPI_ISL_558110, EPI_ISL_558112, EPI_ISL_558114 to 558116, EPI_ISL_558118 to 558125, EPI_ISL_558128 to 558130, EPI_ISL_558132 to 558134, EPI_ISL_558136 to 558137, EPI_ISL_558139 to 558140, EPI_ISL_558142, EPI_ISL_558144 to 558146, EPI_ISL_558148, EPI_ISL_558151, EPI_ISL_558153 to 558154, EPI_ISL_558156, EPI_ISL_558158, EPI_ISL_558160, EPI_ISL_558164, EPI_ISL_558166, EPI_ISL_558168 to 558174, EPI_ISL_558176, EPI_ISL_558180 to 558184, EPI_ISL_558186 to 558187, EPI_ISL_558189 to 558193, EPI_ISL_558196 to 558203, EPI_ISL_558205 to 558207, EPI_ISL_558209 to 558210, EPI_ISL_558212 to 558213, EPI_ISL_558216 to 558217, EPI_ISL_558219 to 558221, EPI_ISL_558223, EPI_ISL_558226 to 558228, EPI_ISL_558230 to 558233, EPI_ISL_558235 to 558236, EPI_ISL_558238, EPI_ISL_558240,

EPI_ISL_558244, EPI_ISL_558256, EPI_ISL_558259 to 558260, EPI_ISL_558263, EPI_ISL_558268 to 558270, EPI_ISL_558276 to 558277, EPI_ISL_558288 to 558289, EPI_ISL_558291, EPI_ISL_558307, EPI_ISL_558312, EPI_ISL_558316, EPI_ISL_558321, EPI_ISL_558324, EPI_ISL_558329, EPI_ISL_558332, EPI_ISL_558335, EPI_ISL_558338, EPI_ISL_558343, EPI_ISL_558345, EPI_ISL_558348, EPI_ISL_558350, EPI_ISL_558355, EPI_ISL_558364, EPI_ISL_558379, EPI_ISL_558389, EPI_ISL_558394 to 558396, EPI_ISL_558404, EPI_ISL_558406 to 558407, EPI_ISL_558409 to 558414, EPI_ISL_558416 to 558418, EPI_ISL_558420 to 558421, EPI_ISL_558423 to 558427, EPI_ISL_558429 to 558431, EPI_ISL_558433, EPI_ISL_558435 to 558436, EPI_ISL_558438 to 558439, EPI_ISL_558442 to 558443, EPI_ISL_558445, EPI_ISL_558447 to 558451, EPI_ISL_558453 to 558455, EPI_ISL_558457 to 558465, EPI_ISL_558467 to 558470, EPI_ISL_558472 to 558473, EPI_ISL_558475 to 558480, EPI_ISL_558482 to 558484, EPI_ISL_558486, EPI_ISL_558488 to 558499, EPI_ISL_558501 to 558508, EPI_ISL_558510 to 558523, EPI_ISL_558525 to 558529, EPI_ISL_558531 to 558542, EPI_ISL_558544, EPI_ISL_558546 to 558552, EPI_ISL_558555 to 558557, EPI_ISL_558559 to 558560, EPI_ISL_558562 to 558564, EPI_ISL_558566, EPI_ISL_558568 to 558571, EPI_ISL_558573, EPI_ISL_558575 to 558576, EPI_ISL_558579 to 558585, EPI_ISL_558587, EPI_ISL_558589 to 558591, EPI_ISL_558593, EPI_ISL_558595 to 558600, EPI_ISL_558602 to 558604, EPI_ISL_558606 to 558608, EPI_ISL_558610 to 558616, EPI_ISL_558618 to 558623, EPI_ISL_558625 to 558626, EPI_ISL_558628 to 558629, EPI_ISL_558631 to 558637, EPI_ISL_558639, EPI_ISL_558641, EPI_ISL_558643 to 558644, EPI_ISL_558648 to 558652, EPI_ISL_558654 to 558660, EPI_ISL_558662 to 558665, EPI_ISL_558668 to 558678, EPI_ISL_558680 to 558683, EPI_ISL_558685 to 558694, EPI_ISL_558696 to 558700, EPI_ISL_558703, EPI_ISL_558705 to 558707, EPI_ISL_558709, EPI_ISL_558711, EPI_ISL_558713 to 558714, EPI_ISL_558717 to 558719, EPI_ISL_558721 to 558723, EPI_ISL_558725 to 558729, EPI_ISL_558732 to 558736, EPI_ISL_558738 to 558750, EPI_ISL_558752 to 558754, EPI_ISL_558757 to 558762, EPI_ISL_558764 to 558766, EPI_ISL_558768 to 558774, EPI_ISL_558776 to 558782, EPI_ISL_558786 to 558787, EPI_ISL_558789, EPI_ISL_558791, EPI_ISL_558793, EPI_ISL_558795, EPI_ISL_558797 to 558799, EPI_ISL_558801 to 558811, EPI_ISL_558815 to 558824, EPI_ISL_558826 to 558833, EPI_ISL_558835, EPI_ISL_558837 to 558841, EPI_ISL_558843 to 558845, EPI_ISL_558847 to 558859, EPI_ISL_558862 to 558866, EPI_ISL_558868 to 558871, EPI_ISL_558873 to 558876, EPI_ISL_558878, EPI_ISL_558880 to 558881, EPI_ISL_558884 to 558886, EPI_ISL_558889 to 558890, EPI_ISL_558892 to 558893, EPI_ISL_558895 to 558902, EPI_ISL_558904 to 558912, EPI_ISL_558917 to 558918, EPI_ISL_558922, EPI_ISL_558924, EPI_ISL_558927, EPI_ISL_558929 to 558931, EPI_ISL_558933, EPI_ISL_558936, EPI_ISL_558938 to 558942, EPI_ISL_558944 to 558945, EPI_ISL_558948, EPI_ISL_558952, EPI_ISL_558954, EPI_ISL_558956 to 558958, EPI_ISL_558960 to 558964, EPI_ISL_558966 to 558969, EPI_ISL_558971 to 558972, EPI_ISL_558976, EPI_ISL_558979, EPI_ISL_558982 to 558984, EPI_ISL_558986, EPI_ISL_558988 to 558989, EPI_ISL_558991 to 558992, EPI_ISL_558997 to 558999, EPI_ISL_559001 to 559004, EPI_ISL_559006 to 559008, EPI_ISL_559010, EPI_ISL_559012, EPI_ISL_559014 to 559016, EPI_ISL_559020 to 559021, EPI_ISL_559023, EPI_ISL_559025, EPI_ISL_559027, EPI_ISL_559029 to 559032, EPI_ISL_559035 to 559038, EPI_ISL_559040, EPI_ISL_559044 to 559045, EPI_ISL_559050 to 559056, EPI_ISL_559059, EPI_ISL_559064, EPI_ISL_559066 to 559070, EPI_ISL_559072 to 559076, EPI_ISL_559078 to 559079, EPI_ISL_559085 to 559087, EPI_ISL_559090 to 559091, EPI_ISL_559093 to 559094, EPI_ISL_559096, EPI_ISL_559098 to 559099, EPI_ISL_559101 to 559102, EPI_ISL_559105, EPI_ISL_559107 to 559116, EPI_ISL_559118, EPI_ISL_559121 to 559123, EPI_ISL_559127 to 559128, EPI_ISL_559130, EPI_ISL_559134 to 559135, EPI_ISL_559137 to 559141, EPI_ISL_559144 to 559146, EPI_ISL_559148, EPI_ISL_559150, EPI_ISL_559153, EPI_ISL_559155 to 559156, EPI_ISL_559160 to 559162, EPI_ISL_559165, EPI_ISL_559169 to 559170, EPI_ISL_559175, EPI_ISL_559177 to 559180, EPI_ISL_559182 to 559186, EPI_ISL_559188, EPI_ISL_559191, EPI_ISL_559193, EPI_ISL_559197 to 559200, EPI_ISL_559205, EPI_ISL_559208 to 559212, EPI_ISL_559214, EPI_ISL_559216 to 559217, EPI_ISL_559219 to 559220, EPI_ISL_559225, EPI_ISL_559229, EPI_ISL_559233 to 559234, EPI_ISL_559237 to 559239, EPI_ISL_559242, EPI_ISL_559245, EPI_ISL_559248, EPI_ISL_559250 to 559251, EPI_ISL_559255, EPI_ISL_559257 to 559258, EPI_ISL_559260, EPI_ISL_559262, EPI_ISL_559269 to 559274, EPI_ISL_559276, EPI_ISL_559279 to 559281, EPI_ISL_559285 to 559286, EPI_ISL_559288 to 559289, EPI_ISL_559291 to 559293, EPI_ISL_559295, EPI_ISL_559297 to 559299, EPI_ISL_559301 to 559311, EPI_ISL_559313, EPI_ISL_559315 to 559317, EPI_ISL_559321 to 559325, EPI_ISL_559327 to 559330, EPI_ISL_559332 to 559334, EPI_ISL_559336, EPI_ISL_559338 to 559349, EPI_ISL_559351, EPI_ISL_559356, EPI_ISL_559358 to 559363, EPI_ISL_559365 to 559367, EPI_ISL_559369 to 559372, EPI_ISL_559375 to 559386, EPI_ISL_559388, EPI_ISL_559392 to 559405, EPI_ISL_559407 to 559417, EPI_ISL_559419 to 559426, EPI_ISL_559428 to 559434, EPI_ISL_559436 to 559446, EPI_ISL_559448 to 559455, EPI_ISL_559457 to 559460, EPI_ISL_559462 to 559463, EPI_ISL_559466 to 559480, EPI_ISL_559483 to 559489, EPI_ISL_559491 to 559493, EPI_ISL_559495 to 559502, EPI_ISL_559505 to 559516, EPI_ISL_559518 to 559521, EPI_ISL_559523, EPI_ISL_559525 to 559543, EPI_ISL_559546 to 559548, EPI_ISL_559550 to 559574, EPI_ISL_559577 to 559590, EPI_ISL_559592 to 559603, EPI_ISL_559605 to 559615, EPI_ISL_559617 to 559622, EPI_ISL_559624 to 559627, EPI_ISL_559629 to 559643, EPI_ISL_559645 to 559648, EPI_ISL_559650 to 559661, EPI_ISL_566111, EPI_ISL_566113 to 566149, EPI_ISL_566151, EPI_ISL_566153 to 566157, EPI_ISL_566159 to 566164, EPI_ISL_566166 to 566171, EPI_ISL_566174 to 566177, EPI_ISL_566179 to 566192, EPI_ISL_566194 to 566200, EPI_ISL_566202 to 566207, EPI_ISL_566210 to 566231, EPI_ISL_566234 to 566241, EPI_ISL_566243 to 566253, EPI_ISL_566255 to 566265, EPI_ISL_566267 to 566273, EPI_ISL_566275, EPI_ISL_566277 to 566288, EPI_ISL_566290 to 566292, EPI_ISL_566294 to 566304, EPI_ISL_566306, EPI_ISL_566308 to 566314, EPI_ISL_566316 to 566323, EPI_ISL_566325 to 566331, EPI_ISL_566333 to 566343, EPI_ISL_566345 to 566364, EPI_ISL_566366 to 566370, EPI_ISL_566372 to 566376, EPI_ISL_566378, EPI_ISL_566380 to 566384, EPI_ISL_566386 to 566400, EPI_ISL_566402 to 566405, EPI_ISL_566407 to 566420, EPI_ISL_567533 to 567535, EPI_ISL_567537 to 567587, EPI_ISL_567589 to 567592, EPI_ISL_567595 to 567602, EPI_ISL_567604 to 567606, EPI_ISL_567608 to 567622, EPI_ISL_567624 to 567648, EPI_ISL_567650 to 567651, EPI_ISL_567653 to 567667, EPI_ISL_567669 to 567677, EPI_ISL_567680 to 567698, EPI_ISL_567700 to 567718, EPI_ISL_567721 to 567737, EPI_ISL_567739 to 567751, EPI_ISL_567753 to 567758, EPI_ISL_567760 to 567764, EPI_ISL_567766, EPI_ISL_567768 to 567769, EPI_ISL_567771 to 567802, EPI_ISL_567804 to 567810, EPI_ISL_567813 to 567830, EPI_ISL_567832, EPI_ISL_567834 to 567844, EPI_ISL_567846 to 567858, EPI_ISL_567861, EPI_ISL_567863 to 567886, EPI_ISL_567888 to 567898, EPI_ISL_567901 to 567910, EPI_ISL_567912 to 567913, EPI_ISL_567915 to 567916, EPI_ISL_567918 to 567933, EPI_ISL_567935 to 567940, EPI_ISL_567942 to 567953, EPI_ISL_567955, EPI_ISL_567957, EPI_ISL_567959 to 567969, EPI_ISL_567971, EPI_ISL_567973 to 567983, EPI_ISL_567985 to 567990, EPI_ISL_567992 to 568011, EPI_ISL_568013 to 568014, EPI_ISL_568016 to 568022, EPI_ISL_568024 to 568025, EPI_ISL_568027, EPI_ISL_568030 to 568031, EPI_ISL_568033 to 568036, EPI_ISL_568038 to 568039, EPI_ISL_568041 to 568061, EPI_ISL_568063 to 568067, EPI_ISL_568069 to 568076, EPI_ISL_568079 to 568095, EPI_ISL_568097 to 568103, EPI_ISL_568105 to 568116, EPI_ISL_568118 to 568121, EPI_ISL_568123 to 568144, EPI_ISL_568146 to 568147, EPI_ISL_568149 to 568169, EPI_ISL_568171 to 568181, EPI_ISL_568183 to 568186, EPI_ISL_568188 to 568191, EPI_ISL_568193 to 568203, EPI_ISL_568205 to 568208, EPI_ISL_568211 to 568222, EPI_ISL_568467, EPI_ISL_575399 to 575403, EPI_ISL_575406 to 575419, EPI_ISL_575421 to 575422, EPI_ISL_575424 to 575431, EPI_ISL_575433 to 575439, EPI_ISL_575441 to 575442, EPI_ISL_575444 to 575459, EPI_ISL_575461 to 575474, EPI_ISL_575476 to 575485, EPI_ISL_575488 to 575491, EPI_ISL_575494 to 575498, EPI_ISL_575500 to 575503, EPI_ISL_575505 to 575520, EPI_ISL_575522 to 575535, EPI_ISL_575537 to 575542, EPI_ISL_575544 to 575551, EPI_ISL_575554 to 575560, EPI_ISL_575562 to 575578, EPI_ISL_575580, EPI_ISL_575582, EPI_ISL_575584 to 575597, EPI_ISL_575599 to 575607, EPI_ISL_575609 to 575612, EPI_ISL_575614 to 575658, EPI_ISL_575660 to 575668, EPI_ISL_575671 to 575682, EPI_ISL_575684, EPI_ISL_575686 to 575689, EPI_ISL_575691, EPI_ISL_575693 to 575701, EPI_ISL_575703 to 575713, EPI_ISL_575715 to 575717, EPI_ISL_575719 to 575734, EPI_ISL_575736 to 575741, EPI_ISL_575743 to 575749, EPI_ISL_575751, EPI_ISL_575754, EPI_ISL_575756 to 575758, EPI_ISL_575760, EPI_ISL_575982 to 575983, EPI_ISL_579919, EPI_ISL_579921 to 579925, EPI_ISL_579927 to 579938, EPI_ISL_579940 to 579948, EPI_ISL_579950, EPI_ISL_579952, EPI_ISL_579954 to 579959, EPI_ISL_579961 to 579970, EPI_ISL_579972 to 579977, EPI_ISL_579980, EPI_ISL_579982 to 579985, EPI_ISL_579987, EPI_ISL_579989 to 579997, EPI_ISL_579999 to 580004, EPI_ISL_580006 to 580014, EPI_ISL_580016 to 580026, EPI_ISL_580028 to 580040, EPI_ISL_580042 to 580053, EPI_ISL_580055 to 580061, EPI_ISL_580063 to 580064, EPI_ISL_580066 to 580072, EPI_ISL_580074, EPI_ISL_580076 to 580086, EPI_ISL_580088 to 580096, EPI_ISL_580098 to 580101, EPI_ISL_580103 to 580113, EPI_ISL_580115 to 580116, EPI_ISL_580119 to 580131, EPI_ISL_580133, EPI_ISL_580135 to 580136, EPI_ISL_580139, EPI_ISL_580141 to 580143, EPI_ISL_580145 to 580147, EPI_ISL_580149, EPI_ISL_580151 to 580161, EPI_ISL_580166 to 580167, EPI_ISL_580170 to 580175, EPI_ISL_580177 to 580179, EPI_ISL_580181 to 580186, EPI_ISL_580188 to 580194, EPI_ISL_580197 to 580199, EPI_ISL_580201 to 580204, EPI_ISL_580206 to 580209, EPI_ISL_580212, EPI_ISL_580214 to 580220, EPI_ISL_580222 to 580224, EPI_ISL_580226 to 580230, EPI_ISL_580232 to 580233, EPI_ISL_580235 to 580236, EPI_ISL_580238 to 580247, EPI_ISL_580249, EPI_ISL_580251 to 580255, EPI_ISL_580257 to 580258, EPI_ISL_580260 to 580271, EPI_ISL_580273 to 580280, EPI_ISL_580284, EPI_ISL_580288 to 580289, EPI_ISL_580291 to 580298, EPI_ISL_580302 to 580304, EPI_ISL_580306 to 580311, EPI_ISL_580314 to 580321, EPI_ISL_580323 to 580324, EPI_ISL_580326, EPI_ISL_580330 to 580331, EPI_ISL_580334 to 580335, EPI_ISL_580337, EPI_ISL_580339, EPI_ISL_580342 to 580343, EPI_ISL_580345 to 580352, EPI_ISL_580355 to 580361, EPI_ISL_580363, EPI_ISL_580365 to 580366, EPI_ISL_580370, EPI_ISL_580372, EPI_ISL_580374 to 580384, EPI_ISL_580386 to 580390, EPI_ISL_580392, EPI_ISL_580395, EPI_ISL_580397 to 580399, EPI_ISL_580402 to 580404, EPI_ISL_580406 to 580408, EPI_ISL_580410 to 580413, EPI_ISL_580415 to 580417, EPI_ISL_580419 to 580420, EPI_ISL_580422 to 580434, EPI_ISL_580436 to 580443, EPI_ISL_580445 to 580448, EPI_ISL_580451, EPI_ISL_580454 to 580455, EPI_ISL_580457 to 580458, EPI_ISL_580460 to 580464, EPI_ISL_580466, EPI_ISL_580468 to 580469, EPI_ISL_580472 to 580473, EPI_ISL_580475 to 580477, EPI_ISL_580482 to 580484, EPI_ISL_580487 to 580493, EPI_ISL_580496 to 580497, EPI_ISL_580500 to 580501, EPI_ISL_580503 to 580506, EPI_ISL_580510, EPI_ISL_580512 to 580513, EPI_ISL_580516 to 580531, EPI_ISL_580534 to 580535, EPI_ISL_580537 to 580548, EPI_ISL_580551 to 580553, EPI_ISL_580555, EPI_ISL_580558, EPI_ISL_580561, EPI_ISL_580566, EPI_ISL_580568, EPI_ISL_580570 to 580572, EPI_ISL_580574 to 580575, EPI_ISL_580578, EPI_ISL_580580 to 580581, EPI_ISL_580583 to 580585, EPI_ISL_580588 to 580589, EPI_ISL_580591 to 580592, EPI_ISL_580595 to 580602, EPI_ISL_580604 to 580606, EPI_ISL_580608, EPI_ISL_580611 to 580617, EPI_ISL_580620, EPI_ISL_580622 to 580624, EPI_ISL_580627 to 580630, EPI_ISL_580633, EPI_ISL_580635, EPI_ISL_580640, EPI_ISL_580645, EPI_ISL_580648, EPI_ISL_580650 to 580651, EPI_ISL_580655, EPI_ISL_580660, EPI_ISL_580663, EPI_ISL_580674 to 580675, EPI_ISL_580679 to 580682, EPI_ISL_580689 to 580692, EPI_ISL_580695, EPI_ISL_580697, EPI_ISL_580699, EPI_ISL_580706, EPI_ISL_580710, EPI_ISL_580720, EPI_ISL_580722 to 580723, EPI_ISL_580730, EPI_ISL_580734, EPI_ISL_580737 to 580738, EPI_ISL_580740, EPI_ISL_580754, EPI_ISL_580763 to 580764, EPI_ISL_580769, EPI_ISL_580771, EPI_ISL_580784, EPI_ISL_580787 to 580789, EPI_ISL_580791, EPI_ISL_580821, EPI_ISL_580823, EPI_ISL_580833, EPI_ISL_580835, EPI_ISL_580849, EPI_ISL_580851, EPI_ISL_581367, EPI_ISL_581378 to 581379, EPI_ISL_581409, EPI_ISL_581411 to 581414, EPI_ISL_581416 to 581417, EPI_ISL_581426 to 581427, EPI_ISL_581429 to 581432, EPI_ISL_581435 to 581436, EPI_ISL_581442, EPI_ISL_581444, EPI_ISL_581446, EPI_ISL_581448, EPI_ISL_586574 to 586586, EPI_ISL_586588 to 586593, EPI_ISL_586595 to 586603, EPI_ISL_586605 to 586611, EPI_ISL_586613 to 586616, EPI_ISL_586618 to 586620, EPI_ISL_586622 to 586628, EPI_ISL_586630 to 586657, EPI_ISL_586659 to 586661, EPI_ISL_586663 to 586667, EPI_ISL_586670 to 586707, EPI_ISL_586710 to 586724, EPI_ISL_586726 to 586727, EPI_ISL_586729 to 586752, EPI_ISL_586755 to 586771, EPI_ISL_586773 to 586807, EPI_ISL_586809 to 586846, EPI_ISL_586848 to 586854, EPI_ISL_586856, EPI_ISL_586858 to 586874, EPI_ISL_586876 to 586883, EPI_ISL_586885 to 586886, EPI_ISL_586888 to 586901, EPI_ISL_586903 to 586906, EPI_ISL_586908 to 586991, EPI_ISL_586993 to 587001, EPI_ISL_587003 to 587031, EPI_ISL_587034 to 587045, EPI_ISL_587047 to 587084, EPI_ISL_587086, EPI_ISL_587089 to 587129, EPI_ISL_587131 to 587146, EPI_ISL_587148 to 587162, EPI_ISL_587164 to 587234, EPI_ISL_587236 to 587237, EPI_ISL_587239, EPI_ISL_587241 to 587250, EPI_ISL_587252 to 587264, EPI_ISL_587266 to 587292, EPI_ISL_587294 to 587302, EPI_ISL_587711 to 587716, EPI_ISL_587718 to 587720, EPI_ISL_587722 to 587727, EPI_ISL_587729 to 587750, EPI_ISL_587752 to 587753, EPI_ISL_587756 to 587758, EPI_ISL_587760 to 587777, EPI_ISL_587779 to 587789, EPI_ISL_587791 to 587792, EPI_ISL_587794, EPI_ISL_587798 to 587799, EPI_ISL_587801, EPI_ISL_587804 to 587811, EPI_ISL_587813 to 587815, EPI_ISL_587817, EPI_ISL_587819 to 587822, EPI_ISL_587825 to 587838, EPI_ISL_587840 to 587841, EPI_ISL_587843 to 587849, EPI_ISL_587851 to 587857, EPI_ISL_587859 to 587876, EPI_ISL_587878 to 587879, EPI_ISL_587882, EPI_ISL_587884 to 587888, EPI_ISL_587890 to 587901, EPI_ISL_587903 to 587906, EPI_ISL_587908 to 587910, EPI_ISL_587913 to 587924, EPI_ISL_587926 to 587927, EPI_ISL_587930 to 587946, EPI_ISL_587948 to 587949, EPI_ISL_587951, EPI_ISL_587953 to 587958, EPI_ISL_587961, EPI_ISL_587963 to 587971, EPI_ISL_587975 to 587976, EPI_ISL_587978 to 587984, EPI_ISL_587986 to 587998, EPI_ISL_588000 to 588001, EPI_ISL_588003 to 588006, EPI_ISL_588008 to 588010, EPI_ISL_588012 to 588016, EPI_ISL_588018, EPI_ISL_588020 to 588025, EPI_ISL_588027, EPI_ISL_588029 to 588030, EPI_ISL_588032 to 588034, EPI_ISL_588036 to 588049, EPI_ISL_588051 to 588055, EPI_ISL_588058, EPI_ISL_589268 to 589288, EPI_ISL_589290 to 589292, EPI_ISL_589294 to 589296, EPI_ISL_589298 to 589301, EPI_ISL_589305 to 589324, EPI_ISL_589326 to 589328, EPI_ISL_589330 to 589333, EPI_ISL_589336 to 589341, EPI_ISL_589343, EPI_ISL_589346 to 589347, EPI_ISL_589349 to 589350, EPI_ISL_589352 to 589354, EPI_ISL_589356 to 589375, EPI_ISL_589378 to 589389, EPI_ISL_589391 to 589403, EPI_ISL_589405 to 589406, EPI_ISL_589408 to 589416, EPI_ISL_589418 to 589422, EPI_ISL_589424 to 589429, EPI_ISL_589431 to 589439, EPI_ISL_589441 to 589460, EPI_ISL_589462 to 589466, EPI_ISL_589468 to 589474, EPI_ISL_589476, EPI_ISL_589478 to 589492, EPI_ISL_589494 to 589518, EPI_ISL_589522 to 589525, EPI_ISL_589527 to 589542, EPI_ISL_589545, EPI_ISL_589547 to 589550, EPI_ISL_589552 to 589558, EPI_ISL_589560 to 589563, EPI_ISL_589565, EPI_ISL_589568, EPI_ISL_589570 to 589571, EPI_ISL_589762, EPI_ISL_589765, EPI_ISL_589778 to 589781, EPI_ISL_589783 to 589786, EPI_ISL_589802, EPI_ISL_590181, EPI_ISL_590186, EPI_ISL_590193 to 590194, EPI_ISL_590198, EPI_ISL_590200, EPI_ISL_590206, EPI_ISL_590210, EPI_ISL_590213 to 590215, EPI_ISL_590220, EPI_ISL_590224 to 590226, EPI_ISL_590228, EPI_ISL_590234 to 590235, EPI_ISL_590239, EPI_ISL_590246, EPI_ISL_590250, EPI_ISL_590255, EPI_ISL_590257, EPI_ISL_590260, EPI_ISL_590263, EPI_ISL_590265, EPI_ISL_590272, EPI_ISL_590274 to 590277, EPI_ISL_590282 to 590284, EPI_ISL_590286, EPI_ISL_590295 to 590296, EPI_ISL_590300, EPI_ISL_590306, EPI_ISL_590309, EPI_ISL_590319, EPI_ISL_590321, EPI_ISL_590327 to 590328, EPI_ISL_590335, EPI_ISL_590337, EPI_ISL_590346 to 590347, EPI_ISL_590351 to 590352, EPI_ISL_590355, EPI_ISL_590357, EPI_ISL_590361, EPI_ISL_590364, EPI_ISL_590366 to 590367, EPI_ISL_590369, EPI_ISL_590373, EPI_ISL_590379 to 590380, EPI_ISL_590383, EPI_ISL_590386, EPI_ISL_590388, EPI_ISL_590390, EPI_ISL_590392, EPI_ISL_590397 to 590399, EPI_ISL_590403, EPI_ISL_590405, EPI_ISL_590422, EPI_ISL_590438, EPI_ISL_590446, EPI_ISL_590462, EPI_ISL_597523 to 597527, EPI_ISL_597529 to 597540, EPI_ISL_597542 to 597563, EPI_ISL_597565 to 597570, EPI_ISL_597572 to 597574, EPI_ISL_597576 to 597609, EPI_ISL_597612 to 597620, EPI_ISL_597622 to 597630, EPI_ISL_597632, EPI_ISL_597635 to 597657, EPI_ISL_597659 to 597662, EPI_ISL_597664 to 597694, EPI_ISL_597696 to 597711, EPI_ISL_597713 to 597750, EPI_ISL_597752, EPI_ISL_597756 to 597762, EPI_ISL_597764 to 597767, EPI_ISL_597769 to 597776, EPI_ISL_597778 to 597787, EPI_ISL_597789 to 597810, EPI_ISL_597813 to 597820, EPI_ISL_597822 to 597827, EPI_ISL_597829 to 597839, EPI_ISL_597841 to 597862, EPI_ISL_597864 to 597878, EPI_ISL_597881 to 597921, EPI_ISL_597923 to 597928, EPI_ISL_597930 to 597936, EPI_ISL_597938 to 597948, EPI_ISL_597950 to 597952, EPI_ISL_597954 to 597963, EPI_ISL_597965 to 597976, EPI_ISL_597978 to 597983, EPI_ISL_597985 to 598000, EPI_ISL_598002 to 598015, EPI_ISL_598017, EPI_ISL_598019 to 598049, EPI_ISL_598052 to 598063, EPI_ISL_598065 to 598074, EPI_ISL_598076 to 598077, EPI_ISL_598079 to 598080, EPI_ISL_598082 to 598093, EPI_ISL_598095 to 598099, EPI_ISL_598101 to 598132, EPI_ISL_598134 to 598139, EPI_ISL_598141 to 598144, EPI_ISL_598146 to 598147, EPI_ISL_598149 to 598157, EPI_ISL_598159 to 598162, EPI_ISL_598164 to 598166, EPI_ISL_598168 to 598171, EPI_ISL_598173 to 598177, EPI_ISL_598179 to 598182, EPI_ISL_598184 to 598190, EPI_ISL_598192 to 598212, EPI_ISL_598214 to 598235, EPI_ISL_598237 to 598241, EPI_ISL_598243 to 598248, EPI_ISL_598824 to 598830, EPI_ISL_600521, EPI_ISL_600523, EPI_ISL_600525 to 600526, EPI_ISL_600528 to 600529, EPI_ISL_600531 to 600532, EPI_ISL_600534, EPI_ISL_600536 to 600537, EPI_ISL_602158 to 602159, EPI_ISL_602161, EPI_ISL_605932 to 605934, EPI_ISL_605936 to 605937, EPI_ISL_605939 to 605943, EPI_ISL_605945, EPI_ISL_605947 to 605956, EPI_ISL_605959 to 605962, EPI_ISL_605965 to 605973, EPI_ISL_605975 to 605978, EPI_ISL_605980 to 605987, EPI_ISL_605989, EPI_ISL_605991 to 605995, EPI_ISL_606000, EPI_ISL_606002, EPI_ISL_606004 to 606011, EPI_ISL_606013 to 606015, EPI_ISL_606017 to 606039, EPI_ISL_606041 to 606045, EPI_ISL_606047 to 606054, EPI_ISL_606056 to 606072, EPI_ISL_606074 to 606077, EPI_ISL_606079 to 606084, EPI_ISL_606086 to 606087, EPI_ISL_606089 to 606092, EPI_ISL_606094 to 606104, EPI_ISL_606106 to 606109, EPI_ISL_606111, EPI_ISL_606113 to 606116, EPI_ISL_606118 to 606126, EPI_ISL_606128 to 606136, EPI_ISL_606138, EPI_ISL_606140 to 606141, EPI_ISL_606143 to 606154, EPI_ISL_606156 to 606160, EPI_ISL_606162 to 606178, EPI_ISL_606180, EPI_ISL_606183 to 606184, EPI_ISL_606186, EPI_ISL_606188, EPI_ISL_606192 to 606206, EPI_ISL_606208 to 606214, EPI_ISL_606216 to 606219, EPI_ISL_606222 to 606225, EPI_ISL_606227 to 606241, EPI_ISL_606243 to 606246, EPI_ISL_606248 to 606258, EPI_ISL_606260, EPI_ISL_606263 to 606266, EPI_ISL_606268 to 606285, EPI_ISL_606287 to 606290, EPI_ISL_606292 to 606295, EPI_ISL_606925 to 606934, EPI_ISL_606936 to 606947, EPI_ISL_606949 to 606957, EPI_ISL_606959, EPI_ISL_606961 to 606976, EPI_ISL_606978 to 606984, EPI_ISL_606986, EPI_ISL_606988 to 607026, EPI_ISL_607028 to 607080, EPI_ISL_607082 to 607094, EPI_ISL_607096 to 607115, EPI_ISL_607117 to 607142, EPI_ISL_607144 to 607168, EPI_ISL_607170 to 607188, EPI_ISL_607191, EPI_ISL_607193 to 607199, EPI_ISL_607201 to 607215, EPI_ISL_607217, EPI_ISL_607219 to 607222, EPI_ISL_607224 to 607231, EPI_ISL_607233 to 607234, EPI_ISL_607236 to 607241, EPI_ISL_607243 to 607245, EPI_ISL_607247 to 607256, EPI_ISL_607258 to 607279, EPI_ISL_607281 to 607290, EPI_ISL_607623 to 607626, EPI_ISL_607628 to 607634, EPI_ISL_607636 to 607638, EPI_ISL_607640 to 607642, EPI_ISL_607644 to 607648, EPI_ISL_607650 to 607652, EPI_ISL_607655, EPI_ISL_607657 to 607659, EPI_ISL_607661 to 607667, EPI_ISL_607669 to 607674, EPI_ISL_607676 to 607679, EPI_ISL_607681 to 607689, EPI_ISL_607691 to 607695, EPI_ISL_607697, EPI_ISL_607699 to 607709, EPI_ISL_607711 to 607712, EPI_ISL_607714 to 607715, EPI_ISL_607718 to 607724, EPI_ISL_607726 to 607764, EPI_ISL_607766, EPI_ISL_607768 to 607772, EPI_ISL_607774, EPI_ISL_607776 to 607779, EPI_ISL_607781 to 607805, EPI_ISL_607807 to 607814, EPI_ISL_607816 to 607823, EPI_ISL_607825 to 607827, EPI_ISL_607830 to 607833, EPI_ISL_607835 to 607836, EPI_ISL_607838 to 607841, EPI_ISL_607844, EPI_ISL_607846 to 607856, EPI_ISL_607859, EPI_ISL_607861 to 607865, EPI_ISL_607868 to 607892, EPI_ISL_607895, EPI_ISL_607897, EPI_ISL_607900 to 607905, EPI_ISL_607907 to 607916, EPI_ISL_607918, EPI_ISL_607920 to 607923, EPI_ISL_607925 to 607930, EPI_ISL_607932 to 607936, EPI_ISL_607938 to 607959, EPI_ISL_607961 to 607965, EPI_ISL_607967 to 607977, EPI_ISL_607980 to 607993, EPI_ISL_607995, EPI_ISL_607997 to 608026, EPI_ISL_608028 to 608030, EPI_ISL_608033 to 608045, EPI_ISL_608047 to 608049, EPI_ISL_608051 to 608053, EPI_ISL_608055 to 608068, EPI_ISL_608070 to 608077, EPI_ISL_608079 to 608081, EPI_ISL_608083 to 608088, EPI_ISL_608090 to 608094, EPI_ISL_608096, EPI_ISL_608098 to 608116, EPI_ISL_608118 to 608141, EPI_ISL_608143, EPI_ISL_608145 to 608146, EPI_ISL_608148, EPI_ISL_608150 to 608157, EPI_ISL_608159 to 608188, EPI_ISL_608190, EPI_ISL_608192 to 608195, EPI_ISL_608197 to 608201, EPI_ISL_608203 to 608209, EPI_ISL_608212 to 608213, EPI_ISL_608215, EPI_ISL_608217 to 608225, EPI_ISL_608227 to 608236, EPI_ISL_608238 to 608241, EPI_ISL_608243 to 608250, EPI_ISL_608253 to 608257, EPI_ISL_608259 to 608289, EPI_ISL_608291 to 608293, EPI_ISL_608296, EPI_ISL_608298 to 608309, EPI_ISL_608311 to 608321, EPI_ISL_608323 to 608345, EPI_ISL_608350 to 608352, EPI_ISL_608355 to 608356, EPI_ISL_608358, EPI_ISL_608361 to 608362, EPI_ISL_608364 to 608367, EPI_ISL_608369, EPI_ISL_608371 to 608372, EPI_ISL_608374 to 608378, EPI_ISL_608380, EPI_ISL_608384 to 608387, EPI_ISL_608390, EPI_ISL_608399, EPI_ISL_608401, EPI_ISL_608403, EPI_ISL_608405 to 608406, EPI_ISL_608409, EPI_ISL_608413 to 608415, EPI_ISL_608418 to 608422, EPI_ISL_608425, EPI_ISL_608434 to 608435, EPI_ISL_608438, EPI_ISL_608440 to 608441, EPI_ISL_608443 to 608445, EPI_ISL_608448, EPI_ISL_608452 to 608454, EPI_ISL_608457, EPI_ISL_608461 to 608462, EPI_ISL_608465, EPI_ISL_608468 to 608470, EPI_ISL_608474, EPI_ISL_608481, EPI_ISL_608483, EPI_ISL_608487 to 608489, EPI_ISL_608491, EPI_ISL_608496, EPI_ISL_608499 to 608500, EPI_ISL_608502 to 608503, EPI_ISL_608505, EPI_ISL_608511 to 608513, EPI_ISL_608517 to 608520, EPI_ISL_608526, EPI_ISL_608528, EPI_ISL_608533 to 608534, EPI_ISL_608541 to 608542, EPI_ISL_608548 to 608549, EPI_ISL_608551, EPI_ISL_608553 to 608554, EPI_ISL_608556, EPI_ISL_608563 to 608564, EPI_ISL_608567, EPI_ISL_608569, EPI_ISL_608575, EPI_ISL_608579 to 608584, EPI_ISL_608586 to 608587, EPI_ISL_608589 to 608595, EPI_ISL_608599 to 608601, EPI_ISL_608603, EPI_ISL_608605 to 608606, EPI_ISL_608610, EPI_ISL_608614, EPI_ISL_608617, EPI_ISL_608621 to 608623, EPI_ISL_608625, EPI_ISL_608630, EPI_ISL_608634 to 608635, EPI_ISL_608639 to 608640, EPI_ISL_608646 to 608649, EPI_ISL_608652, EPI_ISL_608655, EPI_ISL_608657 to 608659, EPI_ISL_608661, EPI_ISL_608663, EPI_ISL_608665, EPI_ISL_608667 to 608670, EPI_ISL_608672, EPI_ISL_608674 to 608677, EPI_ISL_608680, EPI_ISL_608684 to 608687, EPI_ISL_608690 to 608692, EPI_ISL_608694, EPI_ISL_608847, EPI_ISL_608849, EPI_ISL_608853, EPI_ISL_608856, EPI_ISL_608858, EPI_ISL_608860, EPI_ISL_608862 to 608866, EPI_ISL_608869, EPI_ISL_608874, EPI_ISL_608877 to 608878, EPI_ISL_608880, EPI_ISL_608883 to 608886, EPI_ISL_608888 to 608891, EPI_ISL_608895, EPI_ISL_608897 to 608900, EPI_ISL_608903 to 608904, EPI_ISL_608909, EPI_ISL_608911, EPI_ISL_608913 to 608917, EPI_ISL_608919 to 608922, EPI_ISL_608925 to 608926, EPI_ISL_608928 to 608934, EPI_ISL_608938, EPI_ISL_608941, EPI_ISL_608944 to 608946, EPI_ISL_608948, EPI_ISL_608950, EPI_ISL_608952, EPI_ISL_608956 to 608957, EPI_ISL_608961, EPI_ISL_608963 to 608964, EPI_ISL_608966, EPI_ISL_608970, EPI_ISL_608972 to 608973, EPI_ISL_608979 to 608981, EPI_ISL_608983 to 608985, EPI_ISL_608988 to 608989, EPI_ISL_608991 to 608992, EPI_ISL_608994, EPI_ISL_608998 to 609000, EPI_ISL_609002, EPI_ISL_609004, EPI_ISL_609007, EPI_ISL_609009 to 609013, EPI_ISL_609016, EPI_ISL_609019 to 609020, EPI_ISL_609022, EPI_ISL_609028, EPI_ISL_609031 to 609032, EPI_ISL_609034 to 609035,

EPI_ISL_609039 to 609041, EPI_ISL_609043, EPI_ISL_609047, EPI_ISL_609051 to 609053, EPI_ISL_609055 to 609057, EPI_ISL_609060, EPI_ISL_609064 to 609065, EPI_ISL_609068, EPI_ISL_609070, EPI_ISL_609074 to 609075, EPI_ISL_609078 to 609080, EPI_ISL_609083, EPI_ISL_609085, EPI_ISL_609087,
EPI_ISL_609089, EPI_ISL_609093, EPI_ISL_609095, EPI_ISL_609098, EPI_ISL_609102 to 609105, EPI_ISL_609107, EPI_ISL_609109, EPI_ISL_609120, EPI_ISL_609124, EPI_ISL_609127, EPI_ISL_609130, EPI_ISL_609132 to 609133, EPI_ISL_609135, EPI_ISL_609141, EPI_ISL_609144, EPI_ISL_609146 to 609148,
EPI_ISL_609150, EPI_ISL_609152 to 609154, EPI_ISL_609157, EPI_ISL_609207 to 609208, EPI_ISL_609210 to 609213, EPI_ISL_609215 to 609218, EPI_ISL_610286 to 610295, EPI_ISL_610297, EPI_ISL_610299 to 610304, EPI_ISL_610306, EPI_ISL_610308, EPI_ISL_610310 to 610311, EPI_ISL_610313 to 610316,
EPI_ISL_610318 to 610331, EPI_ISL_610333, EPI_ISL_610335 to 610343, EPI_ISL_610346 to 610355, EPI_ISL_610357 to 610358, EPI_ISL_610360 to 610361, EPI_ISL_610363 to 610370, EPI_ISL_610372 to 610392, EPI_ISL_610394 to 610395, EPI_ISL_610397, EPI_ISL_610399 to 610400, EPI_ISL_610402 to 610406,
EPI_ISL_610408 to 610409, EPI_ISL_610412, EPI_ISL_610414 to 610415, EPI_ISL_610417 to 610427, EPI_ISL_610429, EPI_ISL_610431 to 610445, EPI_ISL_610447, EPI_ISL_610449 to 610450, EPI_ISL_610452 to 610455, EPI_ISL_610457, EPI_ISL_610459, EPI_ISL_610461 to 610465, EPI_ISL_610467, EPI_ISL_610469 to
610470, EPI_ISL_610472 to 610487, EPI_ISL_610489 to 610493, EPI_ISL_610495 to 610501, EPI_ISL_610504 to 610508, EPI_ISL_610510 to 610511, EPI_ISL_610513 to 610518, EPI_ISL_610520 to 610522, EPI_ISL_610524 to 610529, EPI_ISL_610531 to 610532, EPI_ISL_610534 to 610536, EPI_ISL_610538 to 610555,
EPI_ISL_610558 to 610570, EPI_ISL_610572 to 610573, EPI_ISL_610575 to 610576, EPI_ISL_610578 to 610589, EPI_ISL_610594 to 610601, EPI_ISL_610604 to 610605, EPI_ISL_610608 to 610610, EPI_ISL_610612 to 610614, EPI_ISL_610616 to 610622, EPI_ISL_610624 to 610628, EPI_ISL_610922 to 610927,
EPI_ISL_610931, EPI_ISL_610933 to 610934, EPI_ISL_610936 to 610955, EPI_ISL_610957 to 610958, EPI_ISL_610960 to 610968, EPI_ISL_610972 to 610979, EPI_ISL_610981 to 610983, EPI_ISL_610985 to 610988, EPI_ISL_610990 to 610997, EPI_ISL_611000, EPI_ISL_611002 to 611005, EPI_ISL_611007 to 611013,
EPI_ISL_611016 to 611036, EPI_ISL_611038 to 611039, EPI_ISL_611041 to 611044, EPI_ISL_611046 to 611047, EPI_ISL_611049 to 611055, EPI_ISL_611058 to 611066, EPI_ISL_611068 to 611071, EPI_ISL_611073 to 611082, EPI_ISL_611084 to 611087, EPI_ISL_611089 to 611092, EPI_ISL_611095 to 611097,
EPI_ISL_611100 to 611102, EPI_ISL_611104 to 611110, EPI_ISL_611112 to 611116, EPI_ISL_611118 to 611125, EPI_ISL_611127 to 611136, EPI_ISL_611138 to 611140, EPI_ISL_611142 to 611143, EPI_ISL_611145 to 611151, EPI_ISL_611154 to 611155, EPI_ISL_611157 to 611167, EPI_ISL_611169 to 611179,
EPI_ISL_611181 to 611186, EPI_ISL_611188 to 611189, EPI_ISL_611191 to 611195, EPI_ISL_611198 to 611203, EPI_ISL_611205 to 611214, EPI_ISL_611216 to 611224, EPI_ISL_611226 to 611232, EPI_ISL_611234 to 611236, EPI_ISL_611238, EPI_ISL_611240 to 611241, EPI_ISL_611243 to 611244, EPI_ISL_611246 to
611247, EPI_ISL_611249 to 611253, EPI_ISL_611255 to 611256, EPI_ISL_611258 to 611273, EPI_ISL_611275, EPI_ISL_611277 to 611286, EPI_ISL_623858 to 623860, EPI_ISL_623862, EPI_ISL_623866 to 623867, EPI_ISL_623869 to 623871, EPI_ISL_623875, EPI_ISL_623877, EPI_ISL_623879 to 623882, EPI_ISL_623885,
EPI_ISL_623887 to 623892, EPI_ISL_623894, EPI_ISL_623896 to 623900, EPI_ISL_623902, EPI_ISL_623905 to 623906, EPI_ISL_623909, EPI_ISL_623912 to 623915, EPI_ISL_623918 to 623920, EPI_ISL_623922, EPI_ISL_623925, EPI_ISL_623927 to 623929, EPI_ISL_623931 to 623932, EPI_ISL_623935 to 623938,
EPI_ISL_623940 to 623941, EPI_ISL_623943, EPI_ISL_623945 to 623948, EPI_ISL_623950 to 623953, EPI_ISL_623955, EPI_ISL_623957 to 623958, EPI_ISL_623961 to 623962, EPI_ISL_623964 to 623965, EPI_ISL_623967, EPI_ISL_623969 to 623971, EPI_ISL_623973, EPI_ISL_623978, EPI_ISL_623981 to 623982,
EPI_ISL_623984 to 623985, EPI_ISL_623987 to 623989, EPI_ISL_623992 to 623993, EPI_ISL_623996 to 624000, EPI_ISL_624003 to 624006, EPI_ISL_624008 to 624009, EPI_ISL_624011, EPI_ISL_624013 to 624017, EPI_ISL_624020 to 624021, EPI_ISL_624023, EPI_ISL_624025 to 624026, EPI_ISL_624030 to 624031,
EPI_ISL_624035 to 624041, EPI_ISL_624047 to 624048, EPI_ISL_624050 to 624056, EPI_ISL_624058 to 624060, EPI_ISL_624062 to 624064, EPI_ISL_624066, EPI_ISL_624068 to 624070, EPI_ISL_624072, EPI_ISL_624074 to 624076, EPI_ISL_624080, EPI_ISL_624082 to 624091, EPI_ISL_624093, EPI_ISL_624096 to 624100,
EPI_ISL_624102 to 624104, EPI_ISL_624106 to 624111, EPI_ISL_624113, EPI_ISL_624115 to 624118, EPI_ISL_624121, EPI_ISL_624124 to 624127, EPI_ISL_624129 to 624130, EPI_ISL_624134, EPI_ISL_624136 to 624139, EPI_ISL_624141 to 624144, EPI_ISL_624146, EPI_ISL_624149 to 624157, EPI_ISL_624159 to 624162,
EPI_ISL_624167 to 624172, EPI_ISL_624175, EPI_ISL_624177 to 624179, EPI_ISL_624182 to 624185, EPI_ISL_624190 to 624191, EPI_ISL_624193 to 624195, EPI_ISL_624198, EPI_ISL_624201 to 624211, EPI_ISL_624213 to 624225, EPI_ISL_624227 to 624243, EPI_ISL_624245, EPI_ISL_624247, EPI_ISL_624249 to 624251,
EPI_ISL_624253, EPI_ISL_624255 to 624265, EPI_ISL_624267 to 624281, EPI_ISL_624283 to 624290, EPI_ISL_624292 to 624298, EPI_ISL_624300 to 624302, EPI_ISL_624304 to 624306, EPI_ISL_624308 to 624328, EPI_ISL_624330 to 624334, EPI_ISL_624336 to 624349, EPI_ISL_624351 to 624353, EPI_ISL_624355,
EPI_ISL_624357 to 624371, EPI_ISL_624373 to 624417, EPI_ISL_624419 to 624444, EPI_ISL_624446, EPI_ISL_624448 to 624451, EPI_ISL_624453 to 624483, EPI_ISL_624485 to 624487, EPI_ISL_624489 to 624498, EPI_ISL_624500 to 624502, EPI_ISL_624504 to 624513, EPI_ISL_624515 to 624516, EPI_ISL_624518,
EPI_ISL_624520 to 624529, EPI_ISL_624531 to 624537, EPI_ISL_624539 to 624545, EPI_ISL_624547 to 624557, EPI_ISL_624559 to 624561, EPI_ISL_624563 to 624576, EPI_ISL_624578 to 624592, EPI_ISL_624594 to 624597, EPI_ISL_624599 to 624601, EPI_ISL_624603 to 624612, EPI_ISL_624614 to 624616,
EPI_ISL_624620 to 624628, EPI_ISL_624630 to 624633, EPI_ISL_624635, EPI_ISL_624637 to 624644, EPI_ISL_624647 to 624648, EPI_ISL_624650 to 624656, EPI_ISL_624658 to 624670, EPI_ISL_624672 to 624684, EPI_ISL_624686, EPI_ISL_624688 to 624692, EPI_ISL_624694, EPI_ISL_624696 to 624698, EPI_ISL_624700
to 624713, EPI_ISL_624716 to 624723, EPI_ISL_624725 to 624727, EPI_ISL_624729 to 624739, EPI_ISL_624741, EPI_ISL_624743 to 624745, EPI_ISL_624747 to 624757, EPI_ISL_624759 to 624760, EPI_ISL_624762 to 624769, EPI_ISL_624771 to 624774, EPI_ISL_624776 to 624777, EPI_ISL_624779 to 624787,
EPI_ISL_624789 to 624793, EPI_ISL_624795 to 624797, EPI_ISL_624800 to 624806, EPI_ISL_624808 to 624817, EPI_ISL_624819 to 624821, EPI_ISL_624823 to 624834, EPI_ISL_624836 to 624841, EPI_ISL_624847 to 624849, EPI_ISL_624851 to 624856, EPI_ISL_624858 to 624863, EPI_ISL_624865 to 624870,
EPI_ISL_624872 to 624884, EPI_ISL_624886 to 624896, EPI_ISL_624898 to 624902, EPI_ISL_624904 to 624906, EPI_ISL_624908, EPI_ISL_624910, EPI_ISL_624912 to 624919, EPI_ISL_624921 to 624940, EPI_ISL_624942 to 624947, EPI_ISL_624949 to 624953, EPI_ISL_624955 to 624978, EPI_ISL_624980 to 624986,
EPI_ISL_624988 to 624992, EPI_ISL_624994, EPI_ISL_624996 to 625002, EPI_ISL_625004 to 625005, EPI_ISL_625007 to 625012, EPI_ISL_625014 to 625016, EPI_ISL_625019 to 625025, EPI_ISL_625027 to 625048, EPI_ISL_625050 to 625065, EPI_ISL_625067 to 625088, EPI_ISL_625090 to 625091, EPI_ISL_625093 to
625120, EPI_ISL_625122 to 625127, EPI_ISL_625129 to 625132, EPI_ISL_625134 to 625144, EPI_ISL_625147 to 625150, EPI_ISL_625152 to 625167, EPI_ISL_625169 to 625179, EPI_ISL_625181, EPI_ISL_625183 to 625184, EPI_ISL_625186 to 625194, EPI_ISL_625196 to 625212, EPI_ISL_625214 to 625221, EPI_ISL_625223
to 625224, EPI_ISL_625226 to 625234, EPI_ISL_625236 to 625239, EPI_ISL_625241 to 625246, EPI_ISL_625248 to 625255, EPI_ISL_625257 to 625277, EPI_ISL_625279 to 625280, EPI_ISL_625396 to 625400, EPI_ISL_625403, EPI_ISL_625422 to 625423, EPI_ISL_625425, EPI_ISL_625427, EPI_ISL_625429, EPI_ISL_625431,
EPI_ISL_625433 to 625436, EPI_ISL_630348 to 630396, EPI_ISL_630398 to 630419, EPI_ISL_630421 to 630434, EPI_ISL_630436 to 630442, EPI_ISL_630444 to 630454, EPI_ISL_630456 to 630473, EPI_ISL_630475 to 630482, EPI_ISL_630484 to 630488, EPI_ISL_630490 to 630507, EPI_ISL_630509 to 630512,
EPI_ISL_630514 to 630526, EPI_ISL_630528, EPI_ISL_630530 to 630532, EPI_ISL_630534 to 630535, EPI_ISL_630538 to 630539, EPI_ISL_630541 to 630544, EPI_ISL_630548 to 630564, EPI_ISL_630566 to 630575, EPI_ISL_630577 to 630579, EPI_ISL_630581 to 630599, EPI_ISL_630601 to 630608, EPI_ISL_630610 to
630620, EPI_ISL_630622 to 630631, EPI_ISL_630633, EPI_ISL_630635 to 630649, EPI_ISL_630651 to 630656, EPI_ISL_630658 to 630665, EPI_ISL_630667 to 630674, EPI_ISL_630676, EPI_ISL_630678 to 630684, EPI_ISL_630686 to 630691, EPI_ISL_630693 to 630701, EPI_ISL_630707 to 630709, EPI_ISL_630713 to
630717, EPI_ISL_630723 to 630724, EPI_ISL_630726 to 630729, EPI_ISL_630732, EPI_ISL_630735 to 630737, EPI_ISL_630739 to 630740, EPI_ISL_630743 to 630744, EPI_ISL_630746 to 630748, EPI_ISL_630750 to 630760, EPI_ISL_630763, EPI_ISL_630766 to 630769, EPI_ISL_630772 to 630774, EPI_ISL_630776 to
630777, EPI_ISL_630783 to 630785, EPI_ISL_630788, EPI_ISL_630790, EPI_ISL_630792 to 630793, EPI_ISL_630795 to 630803, EPI_ISL_630805, EPI_ISL_630807 to 630812, EPI_ISL_630814, EPI_ISL_630816 to 630823, EPI_ISL_630825 to 630826, EPI_ISL_630828 to 630832, EPI_ISL_630835 to 630837, EPI_ISL_630840 to
630845, EPI_ISL_630847 to 630848, EPI_ISL_630852, EPI_ISL_630854 to 630858, EPI_ISL_630860, EPI_ISL_630863, EPI_ISL_630865 to 630869, EPI_ISL_630874, EPI_ISL_630877 to 630882, EPI_ISL_630884 to 630887, EPI_ISL_630889 to 630890, EPI_ISL_630892 to 630893, EPI_ISL_630896 to 630899, EPI_ISL_630901 to
630902, EPI_ISL_630904 to 630910, EPI_ISL_630912 to 630913, EPI_ISL_630916 to 630918, EPI_ISL_630920 to 630922, EPI_ISL_630924 to 630925, EPI_ISL_630927 to 630930, EPI_ISL_630932, EPI_ISL_630934 to 630935, EPI_ISL_630938 to 630940, EPI_ISL_630942 to 630947, EPI_ISL_630950, EPI_ISL_630952,
EPI_ISL_630954, EPI_ISL_630960 to 630964, EPI_ISL_630966, EPI_ISL_630968 to 630971, EPI_ISL_630975 to 630976, EPI_ISL_630982 to 630983, EPI_ISL_630985, EPI_ISL_630994, EPI_ISL_630999, EPI_ISL_631012, EPI_ISL_631014, EPI_ISL_631018, EPI_ISL_631027, EPI_ISL_631031, EPI_ISL_631033,
EPI_ISL_631035, EPI_ISL_631041, EPI_ISL_631044, EPI_ISL_631046, EPI_ISL_631050, EPI_ISL_631056 to 631057, EPI_ISL_631059 to 631060, EPI_ISL_631074, EPI_ISL_631084, EPI_ISL_631089, EPI_ISL_631093, EPI_ISL_631096, EPI_ISL_631099, EPI_ISL_631107, EPI_ISL_631111, EPI_ISL_631113, EPI_ISL_631116,
EPI_ISL_631127 to 631128, EPI_ISL_631131, EPI_ISL_631133, EPI_ISL_631150, EPI_ISL_631163, EPI_ISL_631165, EPI_ISL_631173, EPI_ISL_631175, EPI_ISL_631184, EPI_ISL_631189 to 631190, EPI_ISL_631202, EPI_ISL_631212, EPI_ISL_631215, EPI_ISL_631217, EPI_ISL_631222, EPI_ISL_631228, EPI_ISL_631231,
EPI_ISL_631241, EPI_ISL_631250, EPI_ISL_631265, EPI_ISL_631269, EPI_ISL_634754 to 634756, EPI_ISL_634758 to 634764, EPI_ISL_634766 to 634778, EPI_ISL_634781 to 634786, EPI_ISL_634788

see above	Lighthouse Lab in Alderley Park	Wellcome Sanger Institute for the COVID-19 Genomics UK (COG-UK) consortium	Cordelia Langford; David K. Jackson; Dominic Kwiatkowski; Ewan Harrison; Ian Johnston; Jacquelyn Wynn; John Sillitoe on behalf of the Wellcome Sanger Institute COVID-19 Surveillance Team; John Sillitoe on behalf of the Wellcome Sanger Institute COVID-19 Surveillance Team (http://www.sanger.ac.uk/covid-team); Mairead Hyland; Roberto Amato; Sonia Goncalves; The Lighthouse Lab in Alderley Park and Alex Alderton

EPI_ISL_643315 to 643318, EPI_ISL_643321 to 643327, EPI_ISL_643329 to 643337, EPI_ISL_643339 to 643340, EPI_ISL_643342 to 643355, EPI_ISL_643357 to 643374, EPI_ISL_643376 to 643377, EPI_ISL_643379 to 643383, EPI_ISL_643385 to 643389, EPI_ISL_643391 to 643393, EPI_ISL_643395 to 643400,
EPI_ISL_643402 to 643406, EPI_ISL_643408 to 643420, EPI_ISL_643422 to 643436, EPI_ISL_643439 to 643447, EPI_ISL_643449 to 643452, EPI_ISL_643454 to 643457, EPI_ISL_643459 to 643471, EPI_ISL_643473 to 643474, EPI_ISL_643476 to 643486, EPI_ISL_643488 to 643500, EPI_ISL_643502 to 643504,
EPI_ISL_643506 to 643512, EPI_ISL_643514 to 643518, EPI_ISL_643520 to 643523, EPI_ISL_643525 to 643569, EPI_ISL_643571, EPI_ISL_643573 to 643577, EPI_ISL_643579 to 643602, EPI_ISL_643604 to 643623, EPI_ISL_643625, EPI_ISL_643627 to 643633, EPI_ISL_643635 to 643640, EPI_ISL_643642 to 643654,
EPI_ISL_643657 to 643665, EPI_ISL_643667 to 643675, EPI_ISL_643677 to 643681, EPI_ISL_643683, EPI_ISL_643685 to 643696, EPI_ISL_643699 to 643709, EPI_ISL_643711 to 643724, EPI_ISL_643726 to 643740, EPI_ISL_643742 to 643748, EPI_ISL_643751, EPI_ISL_643753 to 643758, EPI_ISL_643760 to 643782,
EPI_ISL_643785 to 643796, EPI_ISL_643798 to 643804, EPI_ISL_643806 to 643813, EPI_ISL_643816 to 643827, EPI_ISL_643830 to 643835, EPI_ISL_643837 to 643840, EPI_ISL_643842 to 643852, EPI_ISL_643854 to 643862, EPI_ISL_643864 to 643876, EPI_ISL_643878 to 643883, EPI_ISL_643885 to 643890,
EPI_ISL_643893 to 643897, EPI_ISL_643899, EPI_ISL_643901, EPI_ISL_643903 to 643906, EPI_ISL_643908 to 643914, EPI_ISL_643916 to 643924, EPI_ISL_643926 to 643930, EPI_ISL_643932 to 643938, EPI_ISL_643940 to 643955, EPI_ISL_643958 to 643966, EPI_ISL_643968, EPI_ISL_643970, EPI_ISL_643972 to 643982,
EPI_ISL_644117, EPI_ISL_645219 to 645220, EPI_ISL_645222 to 645225, EPI_ISL_645228 to 645232, EPI_ISL_645234 to 645239, EPI_ISL_645242, EPI_ISL_645245, EPI_ISL_645247, EPI_ISL_645249, EPI_ISL_645253 to 645259, EPI_ISL_645261 to 645262, EPI_ISL_645265, EPI_ISL_645267 to 645281, EPI_ISL_645283 to
645284, EPI_ISL_645286 to 645295, EPI_ISL_645298, EPI_ISL_645301, EPI_ISL_645304 to 645305, EPI_ISL_645307, EPI_ISL_645309 to 645318, EPI_ISL_645320 to 645323, EPI_ISL_645325 to 645326, EPI_ISL_645328, EPI_ISL_645331 to 645335, EPI_ISL_645337 to 645339, EPI_ISL_645342 to 645344, EPI_ISL_645346 to
645347, EPI_ISL_645349 to 645353, EPI_ISL_645355 to 645359, EPI_ISL_645361, EPI_ISL_645363 to 645364, EPI_ISL_645366, EPI_ISL_645370 to 645373, EPI_ISL_645376, EPI_ISL_645379, EPI_ISL_645381, EPI_ISL_645386 to 645387, EPI_ISL_645389, EPI_ISL_645392, EPI_ISL_645397 to 645398, EPI_ISL_645400 to
645404, EPI_ISL_645407 to 645414, EPI_ISL_645416 to 645417, EPI_ISL_645419 to 645420, EPI_ISL_645422, EPI_ISL_645425 to 645435, EPI_ISL_645437, EPI_ISL_645439, EPI_ISL_645442 to 645446, EPI_ISL_645448 to 645451, EPI_ISL_645454 to 645455, EPI_ISL_645457 to 645458, EPI_ISL_645460 to 645463,
EPI_ISL_645465 to 645470, EPI_ISL_645472, EPI_ISL_645474 to 645476, EPI_ISL_645478 to 645480, EPI_ISL_645482 to 645488, EPI_ISL_645492, EPI_ISL_645495 to 645498, EPI_ISL_645500, EPI_ISL_645505 to 645507, EPI_ISL_645509 to 645512, EPI_ISL_645514 to 645516, EPI_ISL_645519, EPI_ISL_645521 to 645526,
EPI_ISL_645528 to 645529, EPI_ISL_645826, EPI_ISL_645828 to 645829, EPI_ISL_645832, EPI_ISL_645838, EPI_ISL_645840, EPI_ISL_645850 to 645852, EPI_ISL_645866, EPI_ISL_645868 to 645869, EPI_ISL_645877, EPI_ISL_645880, EPI_ISL_645882, EPI_ISL_645885 to 645886, EPI_ISL_645896 to 645897,
EPI_ISL_645899, EPI_ISL_645902, EPI_ISL_645905, EPI_ISL_645908, EPI_ISL_645916 to 645918, EPI_ISL_645923, EPI_ISL_645925 to 645927, EPI_ISL_645930, EPI_ISL_645945, EPI_ISL_645949, EPI_ISL_645958, EPI_ISL_645962, EPI_ISL_645968, EPI_ISL_645970, EPI_ISL_645980 to 645981, EPI_ISL_645983,
EPI_ISL_645987, EPI_ISL_645989, EPI_ISL_645992 to 645995, EPI_ISL_645998, EPI_ISL_646014, EPI_ISL_646017 to 646018, EPI_ISL_646022, EPI_ISL_646027 to 646028, EPI_ISL_646030, EPI_ISL_646032, EPI_ISL_646034 to 646035, EPI_ISL_646039, EPI_ISL_646041, EPI_ISL_646046 to 646047, EPI_ISL_646052,
EPI_ISL_646056, EPI_ISL_646058, EPI_ISL_646067, EPI_ISL_646070, EPI_ISL_646076, EPI_ISL_646083 to 646084, EPI_ISL_646091, EPI_ISL_646101 to 646102, EPI_ISL_646105, EPI_ISL_646115, EPI_ISL_646122, EPI_ISL_646128 to 646129, EPI_ISL_646131, EPI_ISL_646135 to 646136, EPI_ISL_646139,
EPI_ISL_647123 to 647127, EPI_ISL_647129, EPI_ISL_647131, EPI_ISL_647134 to 647139, EPI_ISL_647141 to 647147, EPI_ISL_647149 to 647151, EPI_ISL_647153 to 647161, EPI_ISL_647163 to 647174, EPI_ISL_647176 to 647181, EPI_ISL_647183 to 647187, EPI_ISL_647189, EPI_ISL_647191 to 647195, EPI_ISL_647197,
EPI_ISL_647199 to 647206, EPI_ISL_647208 to 647210, EPI_ISL_647212 to 647213, EPI_ISL_647215 to 647216, EPI_ISL_647218, EPI_ISL_647221 to 647222, EPI_ISL_647224 to 647225, EPI_ISL_647227 to 647228, EPI_ISL_647230 to 647236, EPI_ISL_647238 to 647247, EPI_ISL_647249, EPI_ISL_647251 to 647254,
EPI_ISL_647256 to 647257, EPI_ISL_647259 to 647260, EPI_ISL_647262 to 647263, EPI_ISL_647265 to 647268, EPI_ISL_647270 to 647278, EPI_ISL_647281 to 647283, EPI_ISL_647285 to 647288, EPI_ISL_647290 to 647294, EPI_ISL_647296 to 647299, EPI_ISL_647302, EPI_ISL_647304 to 647308, EPI_ISL_647310 to
647316, EPI_ISL_647319 to 647321, EPI_ISL_647323 to 647325, EPI_ISL_647327 to 647328, EPI_ISL_647330 to 647337, EPI_ISL_647339 to 647340, EPI_ISL_647342 to 647348, EPI_ISL_647350 to 647353, EPI_ISL_647357 to 647358, EPI_ISL_647360 to 647365, EPI_ISL_647367, EPI_ISL_647369, EPI_ISL_647371 to
647372, EPI_ISL_647374 to 647377, EPI_ISL_647379 to 647383, EPI_ISL_647385 to 647388, EPI_ISL_647390, EPI_ISL_647392 to 647396, EPI_ISL_647398, EPI_ISL_647400 to 647411, EPI_ISL_647413 to 647421, EPI_ISL_647425 to 647427, EPI_ISL_647429 to 647444, EPI_ISL_647446 to 647451, EPI_ISL_647453 to
647463, EPI_ISL_647466 to 647467, EPI_ISL_647470 to 647472, EPI_ISL_647476, EPI_ISL_647478 to 647487, EPI_ISL_647489 to 647496, EPI_ISL_647498, EPI_ISL_647501 to 647502, EPI_ISL_647505, EPI_ISL_647508 to 647510, EPI_ISL_647514 to 647515, EPI_ISL_647517, EPI_ISL_647519 to 647525, EPI_ISL_647527,
EPI_ISL_647529 to 647534, EPI_ISL_647537 to 647540, EPI_ISL_647542 to 647545, EPI_ISL_647547 to 647548, EPI_ISL_647552, EPI_ISL_647554 to 647555, EPI_ISL_647557, EPI_ISL_647559, EPI_ISL_647561, EPI_ISL_647563 to 647571, EPI_ISL_647574 to 647575, EPI_ISL_647577, EPI_ISL_647581 to 647583,
EPI_ISL_647585 to 647590, EPI_ISL_647593, EPI_ISL_647595 to 647596, EPI_ISL_647598 to 647601, EPI_ISL_647604 to 647605, EPI_ISL_647607, EPI_ISL_647609 to 647611, EPI_ISL_647613 to 647614, EPI_ISL_647616 to 647617, EPI_ISL_647619 to 647620, EPI_ISL_647622 to 647623, EPI_ISL_647625 to 647628,
EPI_ISL_647631, EPI_ISL_647633, EPI_ISL_647636 to 647637, EPI_ISL_647639 to 647640, EPI_ISL_647642, EPI_ISL_647644 to 647649, EPI_ISL_647651 to 647654, EPI_ISL_647656 to 647658, EPI_ISL_647660 to 647661, EPI_ISL_647664 to 647665, EPI_ISL_647669 to 647671, EPI_ISL_647673 to 647676, EPI_ISL_647679,
EPI_ISL_647681, EPI_ISL_647684 to 647688, EPI_ISL_647690 to 647692, EPI_ISL_647694, EPI_ISL_647696 to 647697, EPI_ISL_647699, EPI_ISL_647701 to 647703, EPI_ISL_647706 to 647707, EPI_ISL_647709 to 647710, EPI_ISL_647713 to 647719, EPI_ISL_647721 to 647722, EPI_ISL_647724 to 647725, EPI_ISL_647727
to 647729, EPI_ISL_647731 to 647732, EPI_ISL_647734 to 647740, EPI_ISL_647742 to 647744, EPI_ISL_647747 to 647748, EPI_ISL_647751, EPI_ISL_647753, EPI_ISL_647755 to 647761, EPI_ISL_647763 to 647767, EPI_ISL_647769 to 647770, EPI_ISL_647772, EPI_ISL_647774 to 647775, EPI_ISL_647777 to 647779,
EPI_ISL_647782 to 647785, EPI_ISL_647788 to 647791, EPI_ISL_647793 to 647796, EPI_ISL_647900 to 647901, EPI_ISL_647906, EPI_ISL_647908 to 647909, EPI_ISL_647913, EPI_ISL_647916, EPI_ISL_649482, EPI_ISL_649492 to 649493, EPI_ISL_649625 to 649628, EPI_ISL_649630 to 649633, EPI_ISL_656686 to 656694,
EPI_ISL_656699, EPI_ISL_656701 to 656702, EPI_ISL_656704, EPI_ISL_656706, EPI_ISL_656708, EPI_ISL_656710 to 656715, EPI_ISL_656718 to 656722, EPI_ISL_656724 to 656727, EPI_ISL_656734 to 656737, EPI_ISL_656739 to 656743, EPI_ISL_656747 to 656748, EPI_ISL_656750 to 656752, EPI_ISL_656754 to 656756,
EPI_ISL_656758, EPI_ISL_656760 to 656762, EPI_ISL_656764, EPI_ISL_656766 to 656769, EPI_ISL_656771 to 656773, EPI_ISL_656775, EPI_ISL_656782, EPI_ISL_656784, EPI_ISL_656787, EPI_ISL_656790, EPI_ISL_656792 to 656795, EPI_ISL_656797, EPI_ISL_656800, EPI_ISL_656802 to 656806, EPI_ISL_656809,
EPI_ISL_656812 to 656814, EPI_ISL_656816 to 656818, EPI_ISL_656820 to 656822, EPI_ISL_656825 to 656826, EPI_ISL_656828, EPI_ISL_656830, EPI_ISL_656833, EPI_ISL_656836 to 656837, EPI_ISL_656839, EPI_ISL_656842 to 656843, EPI_ISL_656847, EPI_ISL_656850 to 656854, EPI_ISL_656856 to 656859,
EPI_ISL_656863 to 656864, EPI_ISL_656866, EPI_ISL_656868, EPI_ISL_656872 to 656876, EPI_ISL_656878, EPI_ISL_656880, EPI_ISL_656882 to 656883, EPI_ISL_656885 to 656886, EPI_ISL_656889 to 656891, EPI_ISL_656896, EPI_ISL_656898 to 656900, EPI_ISL_656902 to 656905, EPI_ISL_656907, EPI_ISL_656909,
EPI_ISL_656911 to 656912, EPI_ISL_656914, EPI_ISL_656916 to 656919, EPI_ISL_656921, EPI_ISL_656923, EPI_ISL_656925 to 656926, EPI_ISL_656928 to 656929, EPI_ISL_656931, EPI_ISL_656933 to 656934, EPI_ISL_656937, EPI_ISL_656940 to 656941, EPI_ISL_656943 to 656944, EPI_ISL_656949, EPI_ISL_656952 to
656954, EPI_ISL_656956, EPI_ISL_656959 to 656960, EPI_ISL_656963 to 656964, EPI_ISL_656967 to 656968, EPI_ISL_656970 to 656972, EPI_ISL_656975, EPI_ISL_656977 to 656978, EPI_ISL_656981, EPI_ISL_656983, EPI_ISL_656986, EPI_ISL_656989 to 656990, EPI_ISL_656992 to 656993, EPI_ISL_656995,
EPI_ISL_656998 to 657002, EPI_ISL_657006 to 657007, EPI_ISL_657009 to 657010, EPI_ISL_657013, EPI_ISL_657015, EPI_ISL_657018 to 657020, EPI_ISL_657022, EPI_ISL_657025, EPI_ISL_657357 to 657358, EPI_ISL_657362, EPI_ISL_657366, EPI_ISL_657368, EPI_ISL_657376, EPI_ISL_657383, EPI_ISL_657387 to
657389, EPI_ISL_657394 to 657395, EPI_ISL_657397, EPI_ISL_657399, EPI_ISL_657402 to 657404, EPI_ISL_657407, EPI_ISL_657412, EPI_ISL_657415, EPI_ISL_657423, EPI_ISL_657427, EPI_ISL_657431 to 657434, EPI_ISL_657436 to 657437, EPI_ISL_657444 to 657445, EPI_ISL_657448 to 657449, EPI_ISL_657454,
EPI_ISL_657456, EPI_ISL_657461, EPI_ISL_657469 to 657471, EPI_ISL_657474 to 657475, EPI_ISL_657477, EPI_ISL_657481 to 657482, EPI_ISL_657489, EPI_ISL_657496, EPI_ISL_657498 to 657499, EPI_ISL_657501, EPI_ISL_657506 to 657508, EPI_ISL_657514, EPI_ISL_657516, EPI_ISL_657520, EPI_ISL_657522 to
657525, EPI_ISL_657528 to 657530, EPI_ISL_657534, EPI_ISL_657537 to 657538, EPI_ISL_657540 to 657541, EPI_ISL_657545 to 657546, EPI_ISL_657552, EPI_ISL_657559, EPI_ISL_657561 to 657562, EPI_ISL_657565 to 657566, EPI_ISL_657569, EPI_ISL_657572 to 657573, EPI_ISL_657576, EPI_ISL_657578,
EPI_ISL_657582, EPI_ISL_657586, EPI_ISL_657588, EPI_ISL_657590, EPI_ISL_657592, EPI_ISL_657595, EPI_ISL_657597 to 657598, EPI_ISL_657600, EPI_ISL_657603 to 657604, EPI_ISL_657612 to 657613, EPI_ISL_657617 to 657621, EPI_ISL_657623, EPI_ISL_657626, EPI_ISL_657628, EPI_ISL_657633 to 657634,
EPI_ISL_657637, EPI_ISL_657645, EPI_ISL_657649, EPI_ISL_657656, EPI_ISL_657658 to 657660, EPI_ISL_657664, EPI_ISL_657668 to 657675, EPI_ISL_657677, EPI_ISL_657679, EPI_ISL_657684 to 657687, EPI_ISL_657689 to 657691, EPI_ISL_657697 to 657699, EPI_ISL_657701, EPI_ISL_657707 to 657708,
EPI_ISL_657721, EPI_ISL_657723 to 657726, EPI_ISL_657728, EPI_ISL_657739, EPI_ISL_657744, EPI_ISL_657746 to 657747, EPI_ISL_657750, EPI_ISL_657754, EPI_ISL_657756, EPI_ISL_657759, EPI_ISL_657762 to 657765, EPI_ISL_657771, EPI_ISL_657780, EPI_ISL_657782, EPI_ISL_657786 to 657787,
EPI_ISL_657790, EPI_ISL_657793, EPI_ISL_657801 to 657807, EPI_ISL_657809 to 657810, EPI_ISL_657813, EPI_ISL_657815 to 657818, EPI_ISL_657824 to 657826, EPI_ISL_657831, EPI_ISL_657833 to 657834, EPI_ISL_657837, EPI_ISL_657839, EPI_ISL_657841, EPI_ISL_657845 to 657846, EPI_ISL_657852 to 657853,
EPI_ISL_657855, EPI_ISL_657857, EPI_ISL_657860, EPI_ISL_657863, EPI_ISL_657866 to 657868, EPI_ISL_657871, EPI_ISL_657875 to 657876, EPI_ISL_657881, EPI_ISL_657883 to 657885, EPI_ISL_657888, EPI_ISL_657890, EPI_ISL_657893, EPI_ISL_657895 to 657897, EPI_ISL_657900, EPI_ISL_657903,
EPI_ISL_657907 to 657909, EPI_ISL_657912, EPI_ISL_657914, EPI_ISL_657917, EPI_ISL_657920, EPI_ISL_657924, EPI_ISL_657926, EPI_ISL_657931, EPI_ISL_657935 to 657936, EPI_ISL_657939, EPI_ISL_657943, EPI_ISL_657945 to 657946, EPI_ISL_657949, EPI_ISL_657951 to 657953, EPI_ISL_657957,
EPI_ISL_657960, EPI_ISL_657964, EPI_ISL_657966 to 657968, EPI_ISL_657974, EPI_ISL_657978, EPI_ISL_657983, EPI_ISL_657987, EPI_ISL_657990, EPI_ISL_657992, EPI_ISL_657994, EPI_ISL_657998, EPI_ISL_658001, EPI_ISL_658007 to 658008, EPI_ISL_658010 to 658013, EPI_ISL_658019 to 658020,

EPI_ISL_658024, EPI_ISL_658028 to 658029, EPI_ISL_658031 to 658034, EPI_ISL_658036 to 658037, EPI_ISL_658041, EPI_ISL_658043, EPI_ISL_658045 to 658046, EPI_ISL_658048, EPI_ISL_658052, EPI_ISL_658055 to 658056, EPI_ISL_658063, EPI_ISL_658071, EPI_ISL_658073 to 658076, EPI_ISL_658080, EPI_ISL_658084, EPI_ISL_658093, EPI_ISL_658095, EPI_ISL_658097, EPI_ISL_658099 to 658101, EPI_ISL_658104, EPI_ISL_658110, EPI_ISL_658119, EPI_ISL_658122, EPI_ISL_658124 to 658125, EPI_ISL_658128, EPI_ISL_658131, EPI_ISL_658134, EPI_ISL_658138, EPI_ISL_658142, EPI_ISL_658148, EPI_ISL_658156 to 658157, EPI_ISL_658159 to 658162, EPI_ISL_658164, EPI_ISL_658166 to 658167, EPI_ISL_658171, EPI_ISL_658177 to 658179, EPI_ISL_658181, EPI_ISL_658186 to 658187, EPI_ISL_658189 to 658192, EPI_ISL_658194, EPI_ISL_658196 to 658199, EPI_ISL_658202 to 658203, EPI_ISL_658206 to 658207, EPI_ISL_658209, EPI_ISL_658212, EPI_ISL_658218, EPI_ISL_658220 to 658221, EPI_ISL_658224, EPI_ISL_658227 to 658228, EPI_ISL_658231, EPI_ISL_658234, EPI_ISL_658236, EPI_ISL_658239, EPI_ISL_658242, EPI_ISL_658246, EPI_ISL_658252, EPI_ISL_658256, EPI_ISL_658258, EPI_ISL_658263, EPI_ISL_658266, EPI_ISL_658270 to 658272, EPI_ISL_658274 to 658276, EPI_ISL_658278 to 658279, EPI_ISL_658285 to 658287, EPI_ISL_658299, EPI_ISL_658304 to 658305, EPI_ISL_658310 to 658311, EPI_ISL_658313, EPI_ISL_658316 to 658317, EPI_ISL_658320 to 658321, EPI_ISL_658323, EPI_ISL_658326 to 658327, EPI_ISL_658330 to 658332, EPI_ISL_658334, EPI_ISL_658337, EPI_ISL_658340, EPI_ISL_658348, EPI_ISL_658352, EPI_ISL_658356, EPI_ISL_658360 to 658362, EPI_ISL_658364, EPI_ISL_658370 to 658371, EPI_ISL_658381, EPI_ISL_658383, EPI_ISL_658387, EPI_ISL_658390, EPI_ISL_658396 to 658397, EPI_ISL_658402, EPI_ISL_658744 to 658745, EPI_ISL_658752 to 658753, EPI_ISL_658756, EPI_ISL_658759 to 658760, EPI_ISL_658767 to 658769, EPI_ISL_658775, EPI_ISL_658781, EPI_ISL_658787 to 658788, EPI_ISL_658816, EPI_ISL_658829, EPI_ISL_658836 to 658838, EPI_ISL_658846, EPI_ISL_658852, EPI_ISL_658864, EPI_ISL_658866, EPI_ISL_658877, EPI_ISL_658881, EPI_ISL_658885, EPI_ISL_658900, EPI_ISL_658905, EPI_ISL_658909, EPI_ISL_658912 to 658914, EPI_ISL_658916, EPI_ISL_658923, EPI_ISL_658925, EPI_ISL_658930 to 658931, EPI_ISL_658933, EPI_ISL_658948, EPI_ISL_658954, EPI_ISL_658959, EPI_ISL_658963, EPI_ISL_658970, EPI_ISL_658976, EPI_ISL_658979, EPI_ISL_658995, EPI_ISL_659000, EPI_ISL_659005, EPI_ISL_659007, EPI_ISL_659011, EPI_ISL_659017 to 659019, EPI_ISL_659028, EPI_ISL_659045, EPI_ISL_659050, EPI_ISL_659053 to 659054, EPI_ISL_659071 to 659073, EPI_ISL_659076 to 659077, EPI_ISL_659080, EPI_ISL_659083 to 659084, EPI_ISL_659090 to 659091, EPI_ISL_659093 to 659094, EPI_ISL_659097 to 659100, EPI_ISL_659102, EPI_ISL_659105, EPI_ISL_659107, EPI_ISL_659109 to 659117, EPI_ISL_659119 to 659122, EPI_ISL_659125 to 659126, EPI_ISL_659130 to 659131, EPI_ISL_659133 to 659138, EPI_ISL_659141 to 659142, EPI_ISL_659144 to 659145, EPI_ISL_659148 to 659151, EPI_ISL_659153, EPI_ISL_659155, EPI_ISL_659159 to 659160, EPI_ISL_659162 to 659166, EPI_ISL_659168 to 659169, EPI_ISL_659171, EPI_ISL_659174 to 659175, EPI_ISL_659177, EPI_ISL_659179 to 659183, EPI_ISL_659185 to 659187, EPI_ISL_659189 to 659190, EPI_ISL_659192 to 659196, EPI_ISL_659198, EPI_ISL_659200 to 659201, EPI_ISL_659203 to 659206, EPI_ISL_659208 to 659209, EPI_ISL_659211 to 659227, EPI_ISL_659229 to 659230, EPI_ISL_659232, EPI_ISL_659234, EPI_ISL_659237, EPI_ISL_659242, EPI_ISL_659244 to 659248, EPI_ISL_659253 to 659255, EPI_ISL_659257, EPI_ISL_659260 to 659269, EPI_ISL_659271 to 659272, EPI_ISL_659274 to 659281, EPI_ISL_659284 to 659291, EPI_ISL_659296 to 659298, EPI_ISL_659301, EPI_ISL_659303 to 659304, EPI_ISL_659306 to 659307, EPI_ISL_659309, EPI_ISL_659311 to 659312, EPI_ISL_659314 to 659317, EPI_ISL_659319, EPI_ISL_659321, EPI_ISL_659323, EPI_ISL_659326 to 659328, EPI_ISL_659331 to 659332, EPI_ISL_659334 to 659340, EPI_ISL_659342, EPI_ISL_659344, EPI_ISL_659346 to 659347, EPI_ISL_659351 to 659355, EPI_ISL_659357 to 659359, EPI_ISL_659361 to 659364, EPI_ISL_659367 to 659371, EPI_ISL_659374 to 659377, EPI_ISL_659379, EPI_ISL_659381 to 659385, EPI_ISL_659387 to 659389, EPI_ISL_659391 to 659394, EPI_ISL_659398 to 659399, EPI_ISL_659401 to 659408, EPI_ISL_659410, EPI_ISL_659413, EPI_ISL_659418, EPI_ISL_659423, EPI_ISL_659425 to 659429, EPI_ISL_659431 to 659432, EPI_ISL_659434, EPI_ISL_659436 to 659437, EPI_ISL_659439, EPI_ISL_659441, EPI_ISL_659443 to 659445, EPI_ISL_659447 to 659448, EPI_ISL_659450, EPI_ISL_659452 to 659457, EPI_ISL_659459 to 659462, EPI_ISL_659465 to 659467, EPI_ISL_659469 to 659470, EPI_ISL_659472 to 659474, EPI_ISL_659476 to 659477, EPI_ISL_659479 to 659480, EPI_ISL_659483, EPI_ISL_659485 to 659486, EPI_ISL_659488 to 659490, EPI_ISL_659492 to 659493, EPI_ISL_659495 to 659496, EPI_ISL_659498, EPI_ISL_659501 to 659505, EPI_ISL_659507 to 659509, EPI_ISL_659511 to 659513, EPI_ISL_659515 to 659520, EPI_ISL_659522 to 659524, EPI_ISL_659526 to 659529, EPI_ISL_659531 to 659541, EPI_ISL_659543 to 659548, EPI_ISL_659550, EPI_ISL_659552 to 659555, EPI_ISL_659557, EPI_ISL_659560 to 659562, EPI_ISL_659564 to 659565, EPI_ISL_659567 to 659568, EPI_ISL_659570, EPI_ISL_659572, EPI_ISL_659574 to 659575, EPI_ISL_659578 to 659584, EPI_ISL_659586, EPI_ISL_659589, EPI_ISL_659591, EPI_ISL_659593 to 659594, EPI_ISL_659596, EPI_ISL_659598, EPI_ISL_659600 to 659602, EPI_ISL_659604 to 659605, EPI_ISL_659608, EPI_ISL_659610 to 659614, EPI_ISL_659617 to 659618, EPI_ISL_659620, EPI_ISL_659622 to 659626, EPI_ISL_659629, EPI_ISL_659631 to 659632, EPI_ISL_659634 to 659635, EPI_ISL_659639 to 659641, EPI_ISL_659643 to 659644, EPI_ISL_659646 to 659654, EPI_ISL_659656 to 659660, EPI_ISL_659663 to 659665, EPI_ISL_659667 to 659673, EPI_ISL_659675 to 659677, EPI_ISL_659680 to 659682, EPI_ISL_659684 to 659686, EPI_ISL_659688, EPI_ISL_659692 to 659695, EPI_ISL_659697, EPI_ISL_659701 to 659702, EPI_ISL_659706 to 659708, EPI_ISL_659713 to 659714, EPI_ISL_659716 to 659723, EPI_ISL_659725 to 659728, EPI_ISL_659731 to 659733, EPI_ISL_659735 to 659736, EPI_ISL_659738, EPI_ISL_659740 to 659741, EPI_ISL_659743, EPI_ISL_661640, EPI_ISL_661647, EPI_ISL_661651, EPI_ISL_661653, EPI_ISL_661664, EPI_ISL_661675, EPI_ISL_661679 to 661680, EPI_ISL_661684, EPI_ISL_661691, EPI_ISL_661693, EPI_ISL_661695 to 661697, EPI_ISL_661710, EPI_ISL_661712, EPI_ISL_661726, EPI_ISL_661734, EPI_ISL_661741 to 661742, EPI_ISL_661749, EPI_ISL_661751, EPI_ISL_661761, EPI_ISL_661775, EPI_ISL_661778, EPI_ISL_661781, EPI_ISL_661783, EPI_ISL_661791, EPI_ISL_661799, EPI_ISL_661801, EPI_ISL_661805, EPI_ISL_661811, EPI_ISL_661817, EPI_ISL_661830, EPI_ISL_661833, EPI_ISL_661836, EPI_ISL_661853, EPI_ISL_661856, EPI_ISL_661863, EPI_ISL_661869, EPI_ISL_661873, EPI_ISL_661881, EPI_ISL_661887, EPI_ISL_661900, EPI_ISL_661903, EPI_ISL_661917, EPI_ISL_661925 to 661926, EPI_ISL_661928, EPI_ISL_661930, EPI_ISL_661941, EPI_ISL_661944, EPI_ISL_661947, EPI_ISL_661951, EPI_ISL_662632, EPI_ISL_662635, EPI_ISL_662651 to 662652, EPI_ISL_667812 to 667813, EPI_ISL_667815, EPI_ISL_667817, EPI_ISL_667819 to 667821, EPI_ISL_667823, EPI_ISL_667826 to 667827, EPI_ISL_667835, EPI_ISL_667840, EPI_ISL_667842, EPI_ISL_667852 to 667853, EPI_ISL_667856, EPI_ISL_667858, EPI_ISL_667862, EPI_ISL_667865, EPI_ISL_667869 to 667871, EPI_ISL_667874 to 667875, EPI_ISL_667877 to 667878, EPI_ISL_667880, EPI_ISL_667886, EPI_ISL_667890, EPI_ISL_667894, EPI_ISL_667896 to 667897, EPI_ISL_667901 to 667902, EPI_ISL_667905, EPI_ISL_667909 to 667911, EPI_ISL_667913, EPI_ISL_667921, EPI_ISL_667923, EPI_ISL_667933, EPI_ISL_667935 to 667937, EPI_ISL_667942, EPI_ISL_667944 to 667946, EPI_ISL_667953 to 667955, EPI_ISL_667965, EPI_ISL_667968, EPI_ISL_667971, EPI_ISL_667979, EPI_ISL_667985, EPI_ISL_667991, EPI_ISL_667993 to 667995, EPI_ISL_668002, EPI_ISL_668009, EPI_ISL_668014 to 668016, EPI_ISL_668019, EPI_ISL_668023, EPI_ISL_668025 to 668027, EPI_ISL_668037, EPI_ISL_668041 to 668043, EPI_ISL_668046, EPI_ISL_668048, EPI_ISL_668058 to 668059, EPI_ISL_668061, EPI_ISL_668065, EPI_ISL_668068 to 668069, EPI_ISL_668071 to 668072, EPI_ISL_668076 to 668077, EPI_ISL_668080, EPI_ISL_668085 to 668087, EPI_ISL_668089 to 668091, EPI_ISL_668097, EPI_ISL_668101, EPI_ISL_668110, EPI_ISL_668115, EPI_ISL_668122, EPI_ISL_668127 to 668128, EPI_ISL_668136 to 668137, EPI_ISL_668148, EPI_ISL_668169, EPI_ISL_668171, EPI_ISL_668180, EPI_ISL_668190, EPI_ISL_668192, EPI_ISL_668200 to 668201, EPI_ISL_668212, EPI_ISL_668222, EPI_ISL_668251, EPI_ISL_668254, EPI_ISL_668265, EPI_ISL_668296, EPI_ISL_668299, EPI_ISL_668316, EPI_ISL_668319, EPI_ISL_668329, EPI_ISL_668336, EPI_ISL_668345, EPI_ISL_668353, EPI_ISL_668355, EPI_ISL_668373 to 668374, EPI_ISL_668388 to 668389, EPI_ISL_672912 to 672923, EPI_ISL_672925 to 672938, EPI_ISL_672941 to 672943, EPI_ISL_672945 to 672948, EPI_ISL_672950, EPI_ISL_672952 to 672953, EPI_ISL_672955 to 672961, EPI_ISL_672963 to 672971, EPI_ISL_672973 to 672976, EPI_ISL_672978, EPI_ISL_672980 to 672986, EPI_ISL_672988 to 672991, EPI_ISL_672993 to 672999, EPI_ISL_673002 to 673017, EPI_ISL_673019 to 673033, EPI_ISL_673035 to 673044, EPI_ISL_673046 to 673048, EPI_ISL_673050 to 673059, EPI_ISL_673062 to 673070, EPI_ISL_673072 to 673074, EPI_ISL_673076 to 673077, EPI_ISL_673079, EPI_ISL_673081 to 673085, EPI_ISL_673087 to 673095, EPI_ISL_673098, EPI_ISL_673100 to 673101, EPI_ISL_673103 to 673113, EPI_ISL_673115 to 673129, EPI_ISL_673131 to 673133, EPI_ISL_673135 to 673144, EPI_ISL_673146, EPI_ISL_673148 to 673173, EPI_ISL_673175 to 673180, EPI_ISL_673182 to 673217, EPI_ISL_673219 to 673225, EPI_ISL_673227 to 673230, EPI_ISL_673232 to 673234, EPI_ISL_673237, EPI_ISL_673239 to 673242, EPI_ISL_673244 to 673253, EPI_ISL_673255 to 673256, EPI_ISL_673258 to 673262, EPI_ISL_673266 to 673274, EPI_ISL_673276 to 673282, EPI_ISL_673286 to 673293, EPI_ISL_673295 to 673302, EPI_ISL_673305 to 673306, EPI_ISL_673308 to 673316, EPI_ISL_673318, EPI_ISL_673320 to 673321, EPI_ISL_673324, EPI_ISL_673326, EPI_ISL_673328 to 673330, EPI_ISL_673332 to 673335, EPI_ISL_673337 to 673338, EPI_ISL_673340 to 673341, EPI_ISL_673344, EPI_ISL_673346 to 673354, EPI_ISL_673356 to 673358, EPI_ISL_673360 to 673365, EPI_ISL_673368, EPI_ISL_673372 to 673381, EPI_ISL_673383 to 673391, EPI_ISL_673394 to 673395, EPI_ISL_673397 to 673398, EPI_ISL_673400, EPI_ISL_673402, EPI_ISL_673404 to 673405, EPI_ISL_673408 to 673413, EPI_ISL_673415 to 673417, EPI_ISL_673420 to 673424, EPI_ISL_673427 to 673431, EPI_ISL_673433, EPI_ISL_673436 to 673442, EPI_ISL_673445 to 673446, EPI_ISL_673448 to 673451, EPI_ISL_673453 to 673465, EPI_ISL_673467 to 673471, EPI_ISL_673473 to 673474, EPI_ISL_673476, EPI_ISL_673478 to 673480, EPI_ISL_673482 to 673483, EPI_ISL_673485 to 673489, EPI_ISL_673491, EPI_ISL_673493 to 673499, EPI_ISL_673502 to 673506, EPI_ISL_673508 to 673512, EPI_ISL_673514 to 673517, EPI_ISL_673519 to 673521, EPI_ISL_673523, EPI_ISL_673527 to 673529, EPI_ISL_673532 to 673533, EPI_ISL_673535 to 673539, EPI_ISL_673541 to 673545, EPI_ISL_673547, EPI_ISL_673550 to 673552, EPI_ISL_673554 to 673557, EPI_ISL_673559, EPI_ISL_673561, EPI_ISL_673563 to 673565, EPI_ISL_673567 to 673571, EPI_ISL_673573 to 673576, EPI_ISL_673579 to 673585, EPI_ISL_673588 to 673590, EPI_ISL_673593, EPI_ISL_673595 to 673597, EPI_ISL_673602, EPI_ISL_673604 to 673606, EPI_ISL_673609 to 673611, EPI_ISL_673613 to 673615, EPI_ISL_673617, EPI_ISL_673619 to 673620, EPI_ISL_673624 to 673626, EPI_ISL_673628, EPI_ISL_673630, EPI_ISL_673632, EPI_ISL_673637, EPI_ISL_673640, EPI_ISL_673643 to 673644, EPI_ISL_673647 to 673649, EPI_ISL_673651, EPI_ISL_673653 to 673654, EPI_ISL_673656, EPI_ISL_673658, EPI_ISL_673660, EPI_ISL_673664 to 673672, EPI_ISL_673674 to 673675, EPI_ISL_673678 to 673680, EPI_ISL_673682 to 673685, EPI_ISL_673687, EPI_ISL_673689 to 673690, EPI_ISL_673693 to 673695, EPI_ISL_673697 to 673699, EPI_ISL_673701, EPI_ISL_673704, EPI_ISL_673706 to 673707, EPI_ISL_673710 to 673711, EPI_ISL_673713 to 673714, EPI_ISL_673716 to 673721, EPI_ISL_673725 to 673729, EPI_ISL_673731 to 673732, EPI_ISL_673734, EPI_ISL_673736, EPI_ISL_673739, EPI_ISL_673741, EPI_ISL_673743 to 673744, EPI_ISL_673747, EPI_ISL_673751, EPI_ISL_673753, EPI_ISL_673755 to 673759, EPI_ISL_673761 to 673763, EPI_ISL_673769, EPI_ISL_673772 to 673777, EPI_ISL_673779, EPI_ISL_673782, EPI_ISL_673784, EPI_ISL_673788 to 673789, EPI_ISL_673791, EPI_ISL_673795, EPI_ISL_673800, EPI_ISL_673802, EPI_ISL_673804 to 673805, EPI_ISL_673808 to 673809, EPI_ISL_673816 to 673819, EPI_ISL_673823, EPI_ISL_673826, EPI_ISL_673830, EPI_ISL_673836 to 673839, EPI_ISL_673841, EPI_ISL_673844 to 673847, EPI_ISL_673849 to 673850, EPI_ISL_673852 to 673855, EPI_ISL_673861 to 673862, EPI_ISL_673864, EPI_ISL_673869 to 673870, EPI_ISL_674826, EPI_ISL_674828, EPI_ISL_674834, EPI_ISL_674837 to 674838, EPI_ISL_674840, EPI_ISL_674845 to 674847, EPI_ISL_674849 to 674851, EPI_ISL_674859, EPI_ISL_674862, EPI_ISL_674866 to 674868, EPI_ISL_674876, EPI_ISL_674883, EPI_ISL_674893, EPI_ISL_674896, EPI_ISL_674906, EPI_ISL_674908 to 674910, EPI_ISL_674917, EPI_ISL_674919, EPI_ISL_674922 to 674924, EPI_ISL_674926, EPI_ISL_674933, EPI_ISL_674940, EPI_ISL_674944, EPI_ISL_674946, EPI_ISL_674950 to 674953, EPI_ISL_674957, EPI_ISL_674959, EPI_ISL_674962, EPI_ISL_674972 to 674973, EPI_ISL_674977, EPI_ISL_674983, EPI_ISL_674992, EPI_ISL_674998, EPI_ISL_675008, EPI_ISL_675018, EPI_ISL_675024, EPI_ISL_675026, EPI_ISL_675033, EPI_ISL_675038 to 675040, EPI_ISL_675042, EPI_ISL_675046, EPI_ISL_675053, EPI_ISL_675064, EPI_ISL_675071, EPI_ISL_675075, EPI_ISL_675082, EPI_ISL_675087, EPI_ISL_675094, EPI_ISL_675104, EPI_ISL_675107, EPI_ISL_675110, EPI_ISL_675117, EPI_ISL_675120, EPI_ISL_675126, EPI_ISL_676383, EPI_ISL_681656, EPI_ISL_681659, EPI_ISL_681671 to 681672, EPI_ISL_702025 to 702032, EPI_ISL_702034, EPI_ISL_702038, EPI_ISL_702040, EPI_ISL_702043 to 702045, EPI_ISL_702051 to 702053, EPI_ISL_702055, EPI_ISL_702058 to 702061, EPI_ISL_702064, EPI_ISL_702067, EPI_ISL_702070 to 702071, EPI_ISL_702076 to 702077, EPI_ISL_702079 to 702080, EPI_ISL_702084, EPI_ISL_702087, EPI_ISL_702089, EPI_ISL_702091, EPI_ISL_702099, EPI_ISL_702101, EPI_ISL_702104, EPI_ISL_702106 to 702108, EPI_ISL_702113 to 702115, EPI_ISL_702118, EPI_ISL_702120 to 702125, EPI_ISL_702127 to 702129, EPI_ISL_702131, EPI_ISL_702133, EPI_ISL_702135 to 702136, EPI_ISL_702139 to 702140, EPI_ISL_702143 to 702145, EPI_ISL_702147 to 702150, EPI_ISL_702152 to 702153, EPI_ISL_702157 to 702161, EPI_ISL_702163 to 702164, EPI_ISL_702166, EPI_ISL_702171 to 702173, EPI_ISL_702179, EPI_ISL_702184, EPI_ISL_702187 to 702189, EPI_ISL_702195 to 702196, EPI_ISL_702198, EPI_ISL_702201 to 702203, EPI_ISL_702206, EPI_ISL_702209 to 702218, EPI_ISL_702223 to 702225, EPI_ISL_702227, EPI_ISL_702234 to 702236, EPI_ISL_702240 to 702242, EPI_ISL_702245, EPI_ISL_702251 to 702252, EPI_ISL_702257, EPI_ISL_702259, EPI_ISL_702261 to 702262, EPI_ISL_702264, EPI_ISL_702266 to 702267, EPI_ISL_702269, EPI_ISL_702271 to 702273, EPI_ISL_702278 to 702280, EPI_ISL_702285 to 702289, EPI_ISL_702292 to 702296, EPI_ISL_702298 to 702299, EPI_ISL_702305 to 702306, EPI_ISL_702311, EPI_ISL_702320, EPI_ISL_702322, EPI_ISL_702324 to 702325, EPI_ISL_702332, EPI_ISL_702334, EPI_ISL_702336, EPI_ISL_702341, EPI_ISL_702346 to 702348, EPI_ISL_702354 to 702358, EPI_ISL_702360, EPI_ISL_702363 to 702364, EPI_ISL_702367 to 702368, EPI_ISL_704688 to 704689, EPI_ISL_704691, EPI_ISL_704694, EPI_ISL_704696 to 704697, EPI_ISL_704700, EPI_ISL_704702, EPI_ISL_704707 to 704708, EPI_ISL_704712 to 704713, EPI_ISL_704717 to 704718, EPI_ISL_704720 to 704721, EPI_ISL_704723 to 704724, EPI_ISL_704726 to 704727, EPI_ISL_704729 to 704730, EPI_ISL_704732, EPI_ISL_704738, EPI_ISL_704742 to 704743, EPI_ISL_704745, EPI_ISL_704751, EPI_ISL_704753, EPI_ISL_704756, EPI_ISL_704758, EPI_ISL_704761, EPI_ISL_704764, EPI_ISL_704766 to 704767, EPI_ISL_704769, EPI_ISL_704774, EPI_ISL_704776, EPI_ISL_704778 to 704779, EPI_ISL_704781 to 704782, EPI_ISL_704785, EPI_ISL_704787, EPI_ISL_704789 to 704790, EPI_ISL_704792 to 704793, EPI_ISL_704795 to 704796, EPI_ISL_704798, EPI_ISL_704800 to 704801, EPI_ISL_704803, EPI_ISL_704805, EPI_ISL_704808, EPI_ISL_704810 to 704811, EPI_ISL_704813 to 704814, EPI_ISL_704819, EPI_ISL_704821, EPI_ISL_704824 to 704825, EPI_ISL_704827, EPI_ISL_704830, EPI_ISL_704833, EPI_ISL_704837, EPI_ISL_704840 to 704841, EPI_ISL_704844, EPI_ISL_704846, EPI_ISL_704848 to 704849, EPI_ISL_704851 to 704852, EPI_ISL_704854, EPI_ISL_704857, EPI_ISL_704859 to 704860, EPI_ISL_704863, EPI_ISL_704865, EPI_ISL_704868, EPI_ISL_704880 to 704881, EPI_ISL_704883, EPI_ISL_704886, EPI_ISL_704888 to 704889, EPI_ISL_704891, EPI_ISL_704893 to 704894, EPI_ISL_704896, EPI_ISL_704902, EPI_ISL_704904, EPI_ISL_704906 to 704907, EPI_ISL_704912, EPI_ISL_704917, EPI_ISL_704922, EPI_ISL_704927 to 704928, EPI_ISL_704935, EPI_ISL_704938, EPI_ISL_704943, EPI_ISL_704946, EPI_ISL_704949, EPI_ISL_704951, EPI_ISL_704953, EPI_ISL_704956, EPI_ISL_704958, EPI_ISL_704961 to 704962, EPI_ISL_704974 to 704975, EPI_ISL_704979 to 704980, EPI_ISL_704984, EPI_ISL_704987 to 704988, EPI_ISL_704992 to 704993, EPI_ISL_705003, EPI_ISL_705006, EPI_ISL_705009, EPI_ISL_705011 to 705012, EPI_ISL_705014, EPI_ISL_705016, EPI_ISL_705025, EPI_ISL_705027 to 705028, EPI_ISL_705031, EPI_ISL_705034, EPI_ISL_705036 to 705037, EPI_ISL_705041 to 705042, EPI_ISL_705045, EPI_ISL_705047, EPI_ISL_705049 to 705050, EPI_ISL_705052 to 705053, EPI_ISL_705058, EPI_ISL_705060, EPI_ISL_705066, EPI_ISL_705068, EPI_ISL_705073, EPI_ISL_705076 to 705077, EPI_ISL_705080, EPI_ISL_705084 to 705085, EPI_ISL_705087, EPI_ISL_705089 to 705090, EPI_ISL_705092 to 705093, EPI_ISL_705095, EPI_ISL_705097 to 705098, EPI_ISL_705100, EPI_ISL_705107, EPI_ISL_705109, EPI_ISL_705111, EPI_ISL_705116 to 705117, EPI_ISL_705122 to 705123, EPI_ISL_705127 to 705128, EPI_ISL_705131, EPI_ISL_705135 to 705136, EPI_ISL_705141 to 705142, EPI_ISL_705144, EPI_ISL_705146 to 705147, EPI_ISL_705152, EPI_ISL_705159, EPI_ISL_705162, EPI_ISL_705165, EPI_ISL_705167, EPI_ISL_705169, EPI_ISL_705172, EPI_ISL_705175, EPI_ISL_705177, EPI_ISL_705180 to 705181, EPI_ISL_705183, EPI_ISL_705185 to 705186, EPI_ISL_705188, EPI_ISL_708992, EPI_ISL_708996, EPI_ISL_709000 to 709001, EPI_ISL_709008, EPI_ISL_709018 to 709019, EPI_ISL_709021, EPI_ISL_709026 to 709027, EPI_ISL_709029 to 709030, EPI_ISL_709037 to 709039, EPI_ISL_709053, EPI_ISL_709057, EPI_ISL_709061 to 709064, EPI_ISL_709070, EPI_ISL_709072, EPI_ISL_709075, EPI_ISL_709081, EPI_ISL_709088, EPI_ISL_709094, EPI_ISL_709096, EPI_ISL_709100 to 709101, EPI_ISL_709116 to 709118, EPI_ISL_709122 to 709124, EPI_ISL_709130, EPI_ISL_709143, EPI_ISL_709152 to 709154, EPI_ISL_709166 to 709167, EPI_ISL_709169, EPI_ISL_709177 to 709178, EPI_ISL_709180, EPI_ISL_709193, EPI_ISL_709196, EPI_ISL_709200, EPI_ISL_709204, EPI_ISL_709206 to 709207, EPI_ISL_709209, EPI_ISL_709220, EPI_ISL_709224, EPI_ISL_709232 to 709233, EPI_ISL_709238, EPI_ISL_709244, EPI_ISL_709246, EPI_ISL_709252, EPI_ISL_709256, EPI_ISL_709259, EPI_ISL_709261, EPI_ISL_709268, EPI_ISL_709275, EPI_ISL_709286 to 709287, EPI_ISL_709293 to 709294, EPI_ISL_709805, EPI_ISL_709807 to 709808, EPI_ISL_709810, EPI_ISL_709813 to 709814, EPI_ISL_709817, EPI_ISL_709822, EPI_ISL_709827, EPI_ISL_709836, EPI_ISL_709843, EPI_ISL_709850, EPI_ISL_709854, EPI_ISL_709864, EPI_ISL_709867, EPI_ISL_709869 to 709870, EPI_ISL_709876 to 709877, EPI_ISL_709880, EPI_ISL_710821, EPI_ISL_710823 to 710829, EPI_ISL_710835 to 710836, EPI_ISL_711024, EPI_ISL_711026, EPI_ISL_711038, EPI_ISL_718331 to 718332, EPI_ISL_718334, EPI_ISL_718338 to 718339, EPI_ISL_718344, EPI_ISL_718351 to 718352, EPI_ISL_718357 to 718358, EPI_ISL_718360 to 718361, EPI_ISL_718365, EPI_ISL_718367 to 718368, EPI_ISL_718376, EPI_ISL_718378 to 718379, EPI_ISL_718382, EPI_ISL_718385 to 718386, EPI_ISL_718389 to 718390, EPI_ISL_718393, EPI_ISL_718397, EPI_ISL_718411, EPI_ISL_718415 to 718417, EPI_ISL_718423 to 718424, EPI_ISL_718434, EPI_ISL_718436, EPI_ISL_718441, EPI_ISL_718448 to 718451, EPI_ISL_718453 to 718454, EPI_ISL_718467 to 718468, EPI_ISL_718472 to 718473, EPI_ISL_718475 to 718478, EPI_ISL_718482, EPI_ISL_718484, EPI_ISL_718492 to 718493, EPI_ISL_718495 to 718497, EPI_ISL_718504, EPI_ISL_718506, EPI_ISL_718509, EPI_ISL_718517, EPI_ISL_718525 to 718527, EPI_ISL_718529, EPI_ISL_718536, EPI_ISL_718542 to 718544, EPI_ISL_718547, EPI_ISL_718553, EPI_ISL_718556, EPI_ISL_718558, EPI_ISL_718561, EPI_ISL_718579 to 718580, EPI_ISL_718583, EPI_ISL_718586 to 718587, EPI_ISL_718592, EPI_ISL_718594 to 718595, EPI_ISL_718600, EPI_ISL_718605, EPI_ISL_718607 to 718609, EPI_ISL_718612, EPI_ISL_718619, EPI_ISL_718624 to 718626, EPI_ISL_718630, EPI_ISL_718638, EPI_ISL_718648, EPI_ISL_718655, EPI_ISL_718667 to 718668, EPI_ISL_718679, EPI_ISL_718681, EPI_ISL_718683 to 718685, EPI_ISL_718688, EPI_ISL_718702 to 718703, EPI_ISL_718706, EPI_ISL_718709, EPI_ISL_718717, EPI_ISL_718722, EPI_ISL_718726 to 718727, EPI_ISL_718756, EPI_ISL_718785, EPI_ISL_718788, EPI_ISL_718805, EPI_ISL_718814 to 718817, EPI_ISL_718820, EPI_ISL_718843, EPI_ISL_718857, EPI_ISL_718860, EPI_ISL_718863, EPI_ISL_718867 to 718868, EPI_ISL_718874 to 718875, EPI_ISL_718879, EPI_ISL_718883, EPI_ISL_718918, EPI_ISL_718924, EPI_ISL_718937 to 718938, EPI_ISL_718940, EPI_ISL_718948, EPI_ISL_718954, EPI_ISL_718958, EPI_ISL_718981, EPI_ISL_718985 to 718986, EPI_ISL_718997 to 718999, EPI_ISL_719001 to 719002, EPI_ISL_719006, EPI_ISL_719008, EPI_ISL_719011 to 719012, EPI_ISL_719019, EPI_ISL_719022, EPI_ISL_719030, EPI_ISL_719032, EPI_ISL_719040, EPI_ISL_719044, EPI_ISL_719048, EPI_ISL_719053 to 719054, EPI_ISL_719078, EPI_ISL_719086, EPI_ISL_719090 to 719091, EPI_ISL_719096, EPI_ISL_719099, EPI_ISL_719103 to 719104, EPI_ISL_719107, EPI_ISL_719113, EPI_ISL_719115, EPI_ISL_719117, EPI_ISL_719122, EPI_ISL_719126, EPI_ISL_719138, EPI_ISL_719143, EPI_ISL_719148, EPI_ISL_719151, EPI_ISL_719154, EPI_ISL_719160, EPI_ISL_719163, EPI_ISL_719165, EPI_ISL_719168, EPI_ISL_719173, EPI_ISL_719176, EPI_ISL_719180, EPI_ISL_719182, EPI_ISL_719188, EPI_ISL_719198, EPI_ISL_719205, EPI_ISL_719218, EPI_ISL_719221, EPI_ISL_719226, EPI_ISL_719228, EPI_ISL_719230, EPI_ISL_719235 to 719236, EPI_ISL_719240, EPI_ISL_719245, EPI_ISL_719248 to 719249, EPI_ISL_719253 to 719254, EPI_ISL_719256, EPI_ISL_719260 to 719261, EPI_ISL_719275 to 719276, EPI_ISL_719281, EPI_ISL_719284 to 719286, EPI_ISL_719294, EPI_ISL_719300 to 719301, EPI_ISL_719305, EPI_ISL_719307 to 719308, EPI_ISL_719315, EPI_ISL_719317, EPI_ISL_719322, EPI_ISL_719326, EPI_ISL_719330, EPI_ISL_719333 to 719334, EPI_ISL_719343 to 719344, EPI_ISL_719348, EPI_ISL_719353, EPI_ISL_719356, EPI_ISL_719360, EPI_ISL_719366, EPI_ISL_719368, EPI_ISL_719370, EPI_ISL_719372, EPI_ISL_719375, EPI_ISL_719377, EPI_ISL_719380 to 719381, EPI_ISL_719383, EPI_ISL_719387 to 719389, EPI_ISL_719395 to 719396, EPI_ISL_719398 to 719399, EPI_ISL_719407, EPI_ISL_719416, EPI_ISL_719422, EPI_ISL_719425, EPI_ISL_719428, EPI_ISL_719445, EPI_ISL_719447, EPI_ISL_719454 to 719455, EPI_ISL_719459 to 719460, EPI_ISL_719463, EPI_ISL_719466, EPI_ISL_719471 to 719473, EPI_ISL_719478, EPI_ISL_719481 to 719482, EPI_ISL_719484 to 719485, EPI_ISL_719490, EPI_ISL_719500, EPI_ISL_719505, EPI_ISL_719509, EPI_ISL_719517 to 719518, EPI_ISL_719523, EPI_ISL_719538, EPI_ISL_719540, EPI_ISL_719545, EPI_ISL_719548, EPI_ISL_719551, EPI_ISL_719564, EPI_ISL_719566, EPI_ISL_719570 to 719571, EPI_ISL_719574, EPI_ISL_719576, EPI_ISL_719578, EPI_ISL_719581, EPI_ISL_719583, EPI_ISL_719596, EPI_ISL_719600, EPI_ISL_719602, EPI_ISL_719604, EPI_ISL_728299, EPI_ISL_733596, EPI_ISL_733604, EPI_ISL_733610, EPI_ISL_733614, EPI_ISL_733626, EPI_ISL_733656, EPI_ISL_733681, EPI_ISL_733694, EPI_ISL_733698, EPI_ISL_733702, EPI_ISL_733715 to 733716, EPI_ISL_733722, EPI_ISL_733726, EPI_ISL_733730, EPI_ISL_733732 to 733733, EPI_ISL_733741, EPI_ISL_733758, EPI_ISL_733765, EPI_ISL_733778, EPI_ISL_733784, EPI_ISL_733786 to 733787, EPI_ISL_733793, EPI_ISL_733804, EPI_ISL_733812, EPI_ISL_733846, EPI_ISL_733849, EPI_ISL_733853 to 733854, EPI_ISL_733860, EPI_ISL_733862, EPI_ISL_733868, EPI_ISL_733886, EPI_ISL_733892, EPI_ISL_733906, EPI_ISL_733909 to 733910, EPI_ISL_756379 to 756380, EPI_ISL_756384, EPI_ISL_756389, EPI_ISL_756392, EPI_ISL_756395, EPI_ISL_756409, EPI_ISL_756413, EPI_ISL_756415 to 756418, EPI_ISL_756420, EPI_ISL_756422, EPI_ISL_756424, EPI_ISL_756426, EPI_ISL_756429, EPI_ISL_756431 to 756432, EPI_ISL_756434, EPI_ISL_756438, EPI_ISL_756442, EPI_ISL_756444, EPI_ISL_756448, EPI_ISL_756453 to 756454, EPI_ISL_756456, EPI_ISL_756460, EPI_ISL_756467, EPI_ISL_756469, EPI_ISL_756474 to 756475, EPI_ISL_756478, EPI_ISL_756480 to 756481, EPI_ISL_756484 to 756486, EPI_ISL_756490 to 756492, EPI_ISL_756496 to 756500, EPI_ISL_756502, EPI_ISL_756505 to 756508, EPI_ISL_756511, EPI_ISL_756513, EPI_ISL_756517, EPI_ISL_756519, EPI_ISL_756521 to 756522, EPI_ISL_756525, EPI_ISL_756528 to 756529, EPI_ISL_756534 to 756535, EPI_ISL_756540, EPI_ISL_756542 to 756543, EPI_ISL_756545, EPI_ISL_756549, EPI_ISL_756553 to 756554, EPI_ISL_756558 to 756559, EPI_ISL_756564, EPI_ISL_756567 to 756568, EPI_ISL_756572, EPI_ISL_756576 to 756577, EPI_ISL_756579, EPI_ISL_756582, EPI_ISL_756585 to 756586, EPI_ISL_756588 to 756591, EPI_ISL_756594,

EPI_ISL_756601, EPI_ISL_756604, EPI_ISL_756607 to 756608, EPI_ISL_756610 to 756611, EPI_ISL_756613 to 756615, EPI_ISL_756617, EPI_ISL_756621 to 756623, EPI_ISL_756625, EPI_ISL_756627 to 756631, EPI_ISL_756637, EPI_ISL_756642 to 756643, EPI_ISL_756649, EPI_ISL_756655, EPI_ISL_756659 to 756660, EPI_ISL_756665 to 756668, EPI_ISL_756671 to 756672, EPI_ISL_756674 to 756680, EPI_ISL_760813, EPI_ISL_760822, EPI_ISL_760833, EPI_ISL_760858 to 760859, EPI_ISL_760894, EPI_ISL_760903, EPI_ISL_760920, EPI_ISL_760922, EPI_ISL_760932, EPI_ISL_760936, EPI_ISL_760939, EPI_ISL_760969, EPI_ISL_760976, EPI_ISL_760988, EPI_ISL_761017, EPI_ISL_761021, EPI_ISL_761057, EPI_ISL_761074 to 761075, EPI_ISL_761922, EPI_ISL_761927, EPI_ISL_761929, EPI_ISL_761935, EPI_ISL_761937, EPI_ISL_761939, EPI_ISL_761946, EPI_ISL_761952 to 761953, EPI_ISL_761956, EPI_ISL_761961, EPI_ISL_761967 to 761968, EPI_ISL_761970, EPI_ISL_761979, EPI_ISL_761981, EPI_ISL_761994 to 761995, EPI_ISL_761997, EPI_ISL_762007 to 762008, EPI_ISL_762014 to 762016, EPI_ISL_762018 to 762019, EPI_ISL_762024 to 762030, EPI_ISL_762036 to 762038, EPI_ISL_762041 to 762043, EPI_ISL_762045, EPI_ISL_762050, EPI_ISL_762057, EPI_ISL_762059 to 762061, EPI_ISL_762063 to 762067, EPI_ISL_762069, EPI_ISL_762072 to 762075, EPI_ISL_762078, EPI_ISL_762085, EPI_ISL_762087, EPI_ISL_762089, EPI_ISL_762091, EPI_ISL_762096 to 762097, EPI_ISL_762103, EPI_ISL_762109, EPI_ISL_762112 to 762114, EPI_ISL_762117 to 762118, EPI_ISL_762122, EPI_ISL_762124 to 762126, EPI_ISL_762133, EPI_ISL_762135 to 762139, EPI_ISL_762142, EPI_ISL_762146 to 762148, EPI_ISL_762152 to 762153, EPI_ISL_762155, EPI_ISL_762163, EPI_ISL_762166, EPI_ISL_762173, EPI_ISL_762175, EPI_ISL_762183, EPI_ISL_762186 to 762187, EPI_ISL_762189, EPI_ISL_762192 to 762193, EPI_ISL_762195, EPI_ISL_762546, EPI_ISL_762549 to 762553, EPI_ISL_762559, EPI_ISL_762563, EPI_ISL_762565 to 762566, EPI_ISL_762569 to 762570, EPI_ISL_762572, EPI_ISL_762575, EPI_ISL_762577 to 762578, EPI_ISL_762580, EPI_ISL_762584 to 762585, EPI_ISL_762587, EPI_ISL_762590 to 762591, EPI_ISL_762593 to 762595, EPI_ISL_762598, EPI_ISL_762600 to 762601, EPI_ISL_762603, EPI_ISL_762607 to 762608, EPI_ISL_762611 to 762613, EPI_ISL_762615 to 762616, EPI_ISL_762619, EPI_ISL_762623 to 762624, EPI_ISL_762626 to 762627, EPI_ISL_762632, EPI_ISL_762635, EPI_ISL_762637, EPI_ISL_762645, EPI_ISL_762649 to 762651, EPI_ISL_762658, EPI_ISL_762660, EPI_ISL_762664 to 762665, EPI_ISL_762667 to 762668, EPI_ISL_762670, EPI_ISL_762673, EPI_ISL_762677, EPI_ISL_762679 to 762682, EPI_ISL_762684 to 762685, EPI_ISL_762687, EPI_ISL_762689, EPI_ISL_762695 to 762697, EPI_ISL_762699 to 762700, EPI_ISL_762705, EPI_ISL_762713, EPI_ISL_762715 to 762719, EPI_ISL_762721, EPI_ISL_762723, EPI_ISL_762725, EPI_ISL_762727, EPI_ISL_762729, EPI_ISL_762736 to 762738, EPI_ISL_762740 to 762742, EPI_ISL_762745, EPI_ISL_762747 to 762748, EPI_ISL_762751 to 762753, EPI_ISL_762757, EPI_ISL_762762, EPI_ISL_762767, EPI_ISL_762769, EPI_ISL_762774, EPI_ISL_762778, EPI_ISL_762780 to 762784, EPI_ISL_762788, EPI_ISL_762790, EPI_ISL_762792, EPI_ISL_762795, EPI_ISL_762797, EPI_ISL_762800, EPI_ISL_762803 to 762808, EPI_ISL_762811, EPI_ISL_762817 to 762818, EPI_ISL_762820, EPI_ISL_762822, EPI_ISL_762825, EPI_ISL_762827, EPI_ISL_762831, EPI_ISL_762833 to 762834, EPI_ISL_762836, EPI_ISL_762838, EPI_ISL_762840, EPI_ISL_762842, EPI_ISL_762848, EPI_ISL_762852, EPI_ISL_762855, EPI_ISL_762858, EPI_ISL_767115, EPI_ISL_767138, EPI_ISL_768845, EPI_ISL_768901 to 768903, EPI_ISL_768905 to 768913, EPI_ISL_768915 to 768923, EPI_ISL_768925 to 768930, EPI_ISL_768932 to 768933, EPI_ISL_768935 to 768940, EPI_ISL_768942 to 768950, EPI_ISL_768952 to 768956, EPI_ISL_768958 to 768959, EPI_ISL_768961 to 768964, EPI_ISL_768966 to 768971, EPI_ISL_768973 to 768987, EPI_ISL_768989 to 768991, EPI_ISL_768993 to 768994, EPI_ISL_768996 to 769000, EPI_ISL_769002 to 769006, EPI_ISL_769008 to 769010, EPI_ISL_769012 to 769028, EPI_ISL_769030 to 769033, EPI_ISL_769035 to 769043, EPI_ISL_769045 to 769047, EPI_ISL_769050 to 769053, EPI_ISL_769055 to 769067, EPI_ISL_769069 to 769080, EPI_ISL_769082 to 769098, EPI_ISL_769100 to 769113, EPI_ISL_769115, EPI_ISL_769117 to 769132, EPI_ISL_769134 to 769136, EPI_ISL_769138 to 769139, EPI_ISL_769141, EPI_ISL_769144 to 769145, EPI_ISL_769147 to 769150, EPI_ISL_769152 to 769160, EPI_ISL_769162 to 769171, EPI_ISL_769173 to 769177, EPI_ISL_769179 to 769185, EPI_ISL_769187 to 769190, EPI_ISL_769192 to 769201, EPI_ISL_769203 to 769219, EPI_ISL_769221 to 769222, EPI_ISL_777289, EPI_ISL_777323, EPI_ISL_777328 to 777331, EPI_ISL_777334, EPI_ISL_777339, EPI_ISL_777342, EPI_ISL_777344 to 777345, EPI_ISL_777347 to 777348, EPI_ISL_777350, EPI_ISL_777352, EPI_ISL_777354 to 777355, EPI_ISL_777357, EPI_ISL_777362, EPI_ISL_777365, EPI_ISL_777368, EPI_ISL_777370 to 777371, EPI_ISL_777378 to 777379, EPI_ISL_777384, EPI_ISL_777388, EPI_ISL_777391, EPI_ISL_777396, EPI_ISL_777398, EPI_ISL_777400 to 777401, EPI_ISL_777403 to 777404, EPI_ISL_777406, EPI_ISL_777408, EPI_ISL_777413 to 777415, EPI_ISL_777419, EPI_ISL_777422, EPI_ISL_777426, EPI_ISL_777429, EPI_ISL_777431, EPI_ISL_777442, EPI_ISL_777446, EPI_ISL_777450, EPI_ISL_777452, EPI_ISL_777456 to 777457, EPI_ISL_777464, EPI_ISL_777469, EPI_ISL_777473, EPI_ISL_777476, EPI_ISL_777483, EPI_ISL_777486, EPI_ISL_777492, EPI_ISL_777494, EPI_ISL_777499 to 777500, EPI_ISL_777503, EPI_ISL_777507 to 777508, EPI_ISL_777512, EPI_ISL_777516 to 777517, EPI_ISL_777519, EPI_ISL_777536, EPI_ISL_777541 to 777542, EPI_ISL_777544 to 777546, EPI_ISL_777549, EPI_ISL_777551, EPI_ISL_777553, EPI_ISL_777559, EPI_ISL_777566, EPI_ISL_777570 to 777571, EPI_ISL_777577, EPI_ISL_777585 to 777586, EPI_ISL_777588, EPI_ISL_777590, EPI_ISL_777594, EPI_ISL_777596, EPI_ISL_777598 to 777599, EPI_ISL_777602 to 777604, EPI_ISL_777606 to 777608, EPI_ISL_777611, EPI_ISL_777617 to 777620, EPI_ISL_777626, EPI_ISL_777631, EPI_ISL_777635 to 777637, EPI_ISL_777640, EPI_ISL_777642 to 777643, EPI_ISL_777645, EPI_ISL_777647 to 777648, EPI_ISL_777652, EPI_ISL_777656, EPI_ISL_777661, EPI_ISL_777664 to 777666, EPI_ISL_777668, EPI_ISL_777671, EPI_ISL_777673 to 777675, EPI_ISL_777677 to 777678, EPI_ISL_777680 to 777688, EPI_ISL_777690 to 777695, EPI_ISL_777698 to 777699, EPI_ISL_777702, EPI_ISL_777704 to 777705, EPI_ISL_777707 to 777714, EPI_ISL_777716 to 777717, EPI_ISL_777720 to 777722, EPI_ISL_777725, EPI_ISL_777727, EPI_ISL_777729 to 777736, EPI_ISL_777738, EPI_ISL_777740 to 777742, EPI_ISL_777745 to 777746, EPI_ISL_777748 to 777749, EPI_ISL_777751 to 777767, EPI_ISL_777769 to 777771, EPI_ISL_777773 to 777775, EPI_ISL_777777, EPI_ISL_777782 to 777788, EPI_ISL_777790 to 777792, EPI_ISL_777795, EPI_ISL_777797 to 777799, EPI_ISL_777802, EPI_ISL_777804 to 777810, EPI_ISL_777812 to 777813, EPI_ISL_777815, EPI_ISL_777817, EPI_ISL_777821 to 777823, EPI_ISL_777825 to 777826, EPI_ISL_777828, EPI_ISL_777830 to 777831, EPI_ISL_777833 to 777835, EPI_ISL_777837 to 777838, EPI_ISL_777840, EPI_ISL_777842, EPI_ISL_777846, EPI_ISL_777849 to 777851, EPI_ISL_777853 to 777854, EPI_ISL_777858 to 777859, EPI_ISL_777863 to 777864, EPI_ISL_777866 to 777868, EPI_ISL_777870 to 777874, EPI_ISL_777877, EPI_ISL_777879, EPI_ISL_777883 to 777886, EPI_ISL_777888 to 777892, EPI_ISL_777894 to 777901, EPI_ISL_777903, EPI_ISL_777907 to 777909, EPI_ISL_777911, EPI_ISL_777913, EPI_ISL_777915, EPI_ISL_777917, EPI_ISL_777920, EPI_ISL_777922, EPI_ISL_777927 to 777934, EPI_ISL_777936 to 777937, EPI_ISL_777939 to 777940, EPI_ISL_777943 to 777958, EPI_ISL_777960, EPI_ISL_777963 to 777965, EPI_ISL_777967 to 777969, EPI_ISL_777971, EPI_ISL_777973 to 777988, EPI_ISL_777990, EPI_ISL_777993 to 777999, EPI_ISL_778002, EPI_ISL_778005 to 778006, EPI_ISL_778010 to 778013, EPI_ISL_778015, EPI_ISL_778019 to 778020, EPI_ISL_778022, EPI_ISL_778027 to 778028, EPI_ISL_778032, EPI_ISL_778038 to 778039, EPI_ISL_778041, EPI_ISL_778054, EPI_ISL_778056 to 778057, EPI_ISL_778067, EPI_ISL_778072, EPI_ISL_778075, EPI_ISL_778078 to 778080, EPI_ISL_778084, EPI_ISL_778086 to 778087, EPI_ISL_778091, EPI_ISL_778094 to 778096, EPI_ISL_778103, EPI_ISL_778109 to 778110, EPI_ISL_778112, EPI_ISL_778114 to 778115, EPI_ISL_778118, EPI_ISL_778120, EPI_ISL_778123, EPI_ISL_778125, EPI_ISL_778130, EPI_ISL_778135, EPI_ISL_778137 to 778139, EPI_ISL_778141 to 778143, EPI_ISL_778148, EPI_ISL_778152, EPI_ISL_778157, EPI_ISL_778160 to 778162, EPI_ISL_778169 to 778170, EPI_ISL_778172 to 778173, EPI_ISL_778175, EPI_ISL_778177 to 778178, EPI_ISL_778180 to 778182, EPI_ISL_778186, EPI_ISL_778188 to 778189, EPI_ISL_778193 to 778194, EPI_ISL_778197 to 778200, EPI_ISL_778204 to 778205, EPI_ISL_778210, EPI_ISL_778213 to 778214, EPI_ISL_778217 to 778218, EPI_ISL_778220, EPI_ISL_778223 to 778224, EPI_ISL_778226 to 778227, EPI_ISL_778232, EPI_ISL_778235, EPI_ISL_778237, EPI_ISL_778241, EPI_ISL_778243, EPI_ISL_778254, EPI_ISL_778262, EPI_ISL_778265, EPI_ISL_778267, EPI_ISL_778271, EPI_ISL_778275, EPI_ISL_778277 to 778278, EPI_ISL_778280, EPI_ISL_778284, EPI_ISL_778293 to 778294, EPI_ISL_778296, EPI_ISL_781183, EPI_ISL_781185, EPI_ISL_781187 to 781188, EPI_ISL_781190, EPI_ISL_781192, EPI_ISL_781199, EPI_ISL_781220, EPI_ISL_781223 to 781224, EPI_ISL_781248 to 781249, EPI_ISL_781251, EPI_ISL_781260, EPI_ISL_781269, EPI_ISL_781281, EPI_ISL_781287, EPI_ISL_781304, EPI_ISL_781306, EPI_ISL_781325, EPI_ISL_781327, EPI_ISL_781333, EPI_ISL_781341, EPI_ISL_781345, EPI_ISL_781354 to 781355, EPI_ISL_781358 to 781359, EPI_ISL_781364, EPI_ISL_781367 to 781368, EPI_ISL_781376, EPI_ISL_781379, EPI_ISL_781385, EPI_ISL_781396, EPI_ISL_781401, EPI_ISL_781407, EPI_ISL_781413, EPI_ISL_781431, EPI_ISL_781434, EPI_ISL_781436, EPI_ISL_781438, EPI_ISL_781440 to 781442, EPI_ISL_781466, EPI_ISL_781480 to 781481, EPI_ISL_781488 to 781490, EPI_ISL_781494, EPI_ISL_781497, EPI_ISL_781506, EPI_ISL_781512 to 781513, EPI_ISL_781517, EPI_ISL_781520 to 781521, EPI_ISL_781523 to 781524, EPI_ISL_781526, EPI_ISL_781528, EPI_ISL_781530, EPI_ISL_781532, EPI_ISL_781534, EPI_ISL_781538, EPI_ISL_781540, EPI_ISL_781542 to 781543, EPI_ISL_781545, EPI_ISL_781549 to 781550, EPI_ISL_781552 to 781553, EPI_ISL_781557, EPI_ISL_781560 to 781563, EPI_ISL_781565 to 781566, EPI_ISL_781568 to 781569, EPI_ISL_781571 to 781572, EPI_ISL_781574, EPI_ISL_781577, EPI_ISL_781580, EPI_ISL_781583 to 781587, EPI_ISL_781590 to 781591, EPI_ISL_781594 to 781595, EPI_ISL_781600 to 781602, EPI_ISL_781606, EPI_ISL_781608, EPI_ISL_781610, EPI_ISL_781612 to 781614, EPI_ISL_781616, EPI_ISL_781622, EPI_ISL_781626, EPI_ISL_781628 to 781631, EPI_ISL_781635, EPI_ISL_781637 to 781640, EPI_ISL_781642 to 781645, EPI_ISL_781650, EPI_ISL_781654, EPI_ISL_781656 to 781660, EPI_ISL_781662, EPI_ISL_781667 to 781668, EPI_ISL_781670, EPI_ISL_781672 to 781673, EPI_ISL_781675 to 781676, EPI_ISL_781679, EPI_ISL_781682, EPI_ISL_781687, EPI_ISL_781690 to 781693, EPI_ISL_781697, EPI_ISL_781700, EPI_ISL_781702 to 781709, EPI_ISL_781712, EPI_ISL_781714 to 781717, EPI_ISL_781719, EPI_ISL_781722 to 781723, EPI_ISL_781727, EPI_ISL_781731, EPI_ISL_781733 to 781734, EPI_ISL_781736, EPI_ISL_781738, EPI_ISL_781740 to 781741, EPI_ISL_781744 to 781745, EPI_ISL_781748, EPI_ISL_781750 to 781752, EPI_ISL_781755, EPI_ISL_781758, EPI_ISL_781760 to 781762, EPI_ISL_781764 to 781766, EPI_ISL_781768, EPI_ISL_781771 to 781772, EPI_ISL_781774 to 781775, EPI_ISL_781779 to 781780, EPI_ISL_781786, EPI_ISL_781790 to 781791, EPI_ISL_781794, EPI_ISL_781796 to 781797, EPI_ISL_781799, EPI_ISL_781802 to 781803, EPI_ISL_781805 to 781806, EPI_ISL_781809 to 781810, EPI_ISL_781812 to 781814, EPI_ISL_781816 to 781819, EPI_ISL_781821 to 781823, EPI_ISL_781825 to 781828, EPI_ISL_781830 to 781831, EPI_ISL_781835 to 781836, EPI_ISL_781843 to 781850, EPI_ISL_781852, EPI_ISL_781855, EPI_ISL_781857, EPI_ISL_781859 to 781860, EPI_ISL_781863, EPI_ISL_781866, EPI_ISL_781868, EPI_ISL_782757, EPI_ISL_782759, EPI_ISL_782761, EPI_ISL_782769, EPI_ISL_782776, EPI_ISL_782788, EPI_ISL_782800, EPI_ISL_782809 to 782810, EPI_ISL_782814, EPI_ISL_782818, EPI_ISL_782822, EPI_ISL_782830, EPI_ISL_782834, EPI_ISL_782842, EPI_ISL_782850, EPI_ISL_782853, EPI_ISL_782860, EPI_ISL_782862, EPI_ISL_782866 to 782868, EPI_ISL_782883, EPI_ISL_782885, EPI_ISL_782887, EPI_ISL_782889 to 782890, EPI_ISL_782908 to 782910, EPI_ISL_782928, EPI_ISL_782930, EPI_ISL_782932, EPI_ISL_782941 to 782942, EPI_ISL_782947, EPI_ISL_782954, EPI_ISL_782960, EPI_ISL_782966, EPI_ISL_782971, EPI_ISL_782974, EPI_ISL_782979, EPI_ISL_782984 to 782985, EPI_ISL_782987, EPI_ISL_783012, EPI_ISL_783031, EPI_ISL_783037 to 783038, EPI_ISL_783057, EPI_ISL_783066, EPI_ISL_783369, EPI_ISL_783375, EPI_ISL_783377, EPI_ISL_783379, EPI_ISL_783381, EPI_ISL_783386, EPI_ISL_783399, EPI_ISL_783404 to 783405, EPI_ISL_783407, EPI_ISL_783416, EPI_ISL_783420, EPI_ISL_783422, EPI_ISL_783424, EPI_ISL_783430, EPI_ISL_783433, EPI_ISL_783436, EPI_ISL_783447, EPI_ISL_783460 to 783461, EPI_ISL_783463, EPI_ISL_783465, EPI_ISL_783467, EPI_ISL_783472, EPI_ISL_783474, EPI_ISL_783476, EPI_ISL_783479 to 783481, EPI_ISL_783484, EPI_ISL_783494, EPI_ISL_783501, EPI_ISL_783506, EPI_ISL_783508, EPI_ISL_783513, EPI_ISL_783516, EPI_ISL_783520, EPI_ISL_783525, EPI_ISL_783529, EPI_ISL_783533 to 783534, EPI_ISL_797397 to 797398, EPI_ISL_797401, EPI_ISL_797403 to 797404, EPI_ISL_797406 to 797407, EPI_ISL_797410, EPI_ISL_797412, EPI_ISL_797414, EPI_ISL_797417 to 797419, EPI_ISL_797422 to 797423, EPI_ISL_797425 to 797427, EPI_ISL_797429, EPI_ISL_797431, EPI_ISL_797433, EPI_ISL_797436 to 797437, EPI_ISL_797440, EPI_ISL_797442 to 797444, EPI_ISL_797446 to 797448, EPI_ISL_797450, EPI_ISL_797452, EPI_ISL_797454, EPI_ISL_797457 to 797458, EPI_ISL_797464 to 797467, EPI_ISL_797469, EPI_ISL_797471, EPI_ISL_797473 to 797474, EPI_ISL_797476 to 797479, EPI_ISL_797481 to 797483, EPI_ISL_797485 to 797487, EPI_ISL_797490, EPI_ISL_797492 to 797497, EPI_ISL_797501 to 797502, EPI_ISL_797505 to 797508, EPI_ISL_797513, EPI_ISL_797515 to 797530, EPI_ISL_797532, EPI_ISL_797534, EPI_ISL_797536 to 797539, EPI_ISL_797542, EPI_ISL_797544 to 797546, EPI_ISL_797550 to 797552, EPI_ISL_797555 to 797558, EPI_ISL_797561 to 797562, EPI_ISL_797565 to 797567, EPI_ISL_797569 to 797572, EPI_ISL_797574, EPI_ISL_797577 to 797581, EPI_ISL_797583, EPI_ISL_797585, EPI_ISL_797588 to 797590, EPI_ISL_797592, EPI_ISL_797594 to 797598, EPI_ISL_797600 to 797601, EPI_ISL_797603, EPI_ISL_797607 to 797609, EPI_ISL_797611 to 797612, EPI_ISL_797614, EPI_ISL_797617 to 797618, EPI_ISL_797626, EPI_ISL_797628 to 797631, EPI_ISL_797633 to 797634, EPI_ISL_797636, EPI_ISL_797638 to 797640, EPI_ISL_797643, EPI_ISL_797645, EPI_ISL_797647 to 797648, EPI_ISL_797651 to 797653, EPI_ISL_797655 to 797659, EPI_ISL_797661 to 797662, EPI_ISL_797664 to 797665, EPI_ISL_797667 to 797672, EPI_ISL_797675 to 797676, EPI_ISL_797679 to 797684, EPI_ISL_797687 to 797692, EPI_ISL_797694 to 797697, EPI_ISL_797700, EPI_ISL_797702 to 797703, EPI_ISL_797706 to 797708, EPI_ISL_797710 to 797711, EPI_ISL_797718, EPI_ISL_798000, EPI_ISL_798003, EPI_ISL_798018, EPI_ISL_798038, EPI_ISL_798040, EPI_ISL_798046, EPI_ISL_798048, EPI_ISL_798053, EPI_ISL_798055, EPI_ISL_798058, EPI_ISL_798060, EPI_ISL_798069, EPI_ISL_798072, EPI_ISL_798076 to 798078, EPI_ISL_798088, EPI_ISL_798093 to 798094, EPI_ISL_798097, EPI_ISL_798111 to 798112, EPI_ISL_798117, EPI_ISL_798121, EPI_ISL_798128, EPI_ISL_798130, EPI_ISL_798140, EPI_ISL_798143, EPI_ISL_798145, EPI_ISL_798148 to 798149, EPI_ISL_798153, EPI_ISL_798160 to 798161, EPI_ISL_798167, EPI_ISL_798183, EPI_ISL_798186, EPI_ISL_798193, EPI_ISL_798199, EPI_ISL_798201, EPI_ISL_798203, EPI_ISL_798211, EPI_ISL_798213 to 798214, EPI_ISL_798216, EPI_ISL_798218, EPI_ISL_798224, EPI_ISL_798230, EPI_ISL_798243 to 798244, EPI_ISL_798251 to 798253, EPI_ISL_798256, EPI_ISL_798258, EPI_ISL_798272 to 798273, EPI_ISL_798281, EPI_ISL_798289, EPI_ISL_798294, EPI_ISL_798298, EPI_ISL_798307, EPI_ISL_798311, EPI_ISL_798315, EPI_ISL_798326 to 798327, EPI_ISL_798664, EPI_ISL_798673, EPI_ISL_798678 to 798679, EPI_ISL_798681 to 798683, EPI_ISL_798685 to 798687, EPI_ISL_798694, EPI_ISL_798698 to 798700, EPI_ISL_798706, EPI_ISL_798708, EPI_ISL_798711, EPI_ISL_798713 to 798714, EPI_ISL_798717, EPI_ISL_798723, EPI_ISL_798726, EPI_ISL_798730, EPI_ISL_798733 to 798734, EPI_ISL_798736 to 798737, EPI_ISL_798743, EPI_ISL_798745 to 798747, EPI_ISL_798753 to 798754, EPI_ISL_798756 to 798763, EPI_ISL_798765 to 798766, EPI_ISL_798775, EPI_ISL_798778, EPI_ISL_798782, EPI_ISL_798785 to 798787, EPI_ISL_798789, EPI_ISL_798792, EPI_ISL_798794, EPI_ISL_798796 to 798797, EPI_ISL_798799 to 798800, EPI_ISL_798802, EPI_ISL_798805 to 798807, EPI_ISL_798810, EPI_ISL_798813, EPI_ISL_798815 to 798816, EPI_ISL_798818, EPI_ISL_798820, EPI_ISL_798825, EPI_ISL_798829, EPI_ISL_798831, EPI_ISL_798834 to 798835, EPI_ISL_798840 to 798841, EPI_ISL_798846 to 798848, EPI_ISL_798854, EPI_ISL_798857 to 798859, EPI_ISL_798861 to 798862, EPI_ISL_798865, EPI_ISL_798867 to 798868, EPI_ISL_798873, EPI_ISL_798877, EPI_ISL_798881, EPI_ISL_798886 to 798887, EPI_ISL_798893 to 798897, EPI_ISL_798901, EPI_ISL_798903, EPI_ISL_798907 to 798909, EPI_ISL_798913, EPI_ISL_798918, EPI_ISL_798920, EPI_ISL_798923, EPI_ISL_798929, EPI_ISL_798931 to 798932, EPI_ISL_798937, EPI_ISL_798940, EPI_ISL_798943, EPI_ISL_798945 to 798947, EPI_ISL_798949, EPI_ISL_798953 to 798954, EPI_ISL_798956, EPI_ISL_798958 to 798960, EPI_ISL_798965, EPI_ISL_798974, EPI_ISL_798976, EPI_ISL_798980, EPI_ISL_798985, EPI_ISL_798990, EPI_ISL_798992, EPI_ISL_798997 to 798999, EPI_ISL_799001, EPI_ISL_799007, EPI_ISL_799017 to 799018, EPI_ISL_799020, EPI_ISL_799031 to 799032, EPI_ISL_799035 to 799037, EPI_ISL_799039, EPI_ISL_799048, EPI_ISL_799058, EPI_ISL_799061, EPI_ISL_799074, EPI_ISL_799076, EPI_ISL_799078, EPI_ISL_799083, EPI_ISL_799087 to 799088, EPI_ISL_799091 to 799095, EPI_ISL_799097, EPI_ISL_799110, EPI_ISL_799116, EPI_ISL_799119, EPI_ISL_799121, EPI_ISL_799128, EPI_ISL_799133, EPI_ISL_799135 to 799136, EPI_ISL_799141 to 799142, EPI_ISL_799146, EPI_ISL_799148, EPI_ISL_799153, EPI_ISL_799159, EPI_ISL_799173 to 799174, EPI_ISL_799181, EPI_ISL_799184 to 799186, EPI_ISL_799195, EPI_ISL_799206, EPI_ISL_799208, EPI_ISL_799210, EPI_ISL_799217, EPI_ISL_799220, EPI_ISL_799225 to 799226, EPI_ISL_799242, EPI_ISL_799247, EPI_ISL_799251, EPI_ISL_799253 to 799254, EPI_ISL_799259, EPI_ISL_799291, EPI_ISL_799935, EPI_ISL_799939, EPI_ISL_799947, EPI_ISL_799950, EPI_ISL_799952 to 799953, EPI_ISL_799958, EPI_ISL_799962 to 799963, EPI_ISL_799972, EPI_ISL_799974, EPI_ISL_799988, EPI_ISL_799996, EPI_ISL_800004, EPI_ISL_800006 to 800008, EPI_ISL_800018, EPI_ISL_800024, EPI_ISL_800028, EPI_ISL_800037, EPI_ISL_800045, EPI_ISL_800054 to 800055, EPI_ISL_800072, EPI_ISL_800077, EPI_ISL_800082, EPI_ISL_800085, EPI_ISL_800092, EPI_ISL_800095 to 800096, EPI_ISL_800098, EPI_ISL_800102 to 800103, EPI_ISL_800114 to 800116, EPI_ISL_800119, EPI_ISL_800121, EPI_ISL_800123, EPI_ISL_800133, EPI_ISL_800137, EPI_ISL_800149, EPI_ISL_800153, EPI_ISL_800156, EPI_ISL_800166 to 800167, EPI_ISL_800173, EPI_ISL_800178, EPI_ISL_800182, EPI_ISL_800192, EPI_ISL_800195 to 800196, EPI_ISL_800211, EPI_ISL_800218, EPI_ISL_800220, EPI_ISL_800228, EPI_ISL_800233, EPI_ISL_800238, EPI_ISL_800244, EPI_ISL_800250, EPI_ISL_800252, EPI_ISL_800255, EPI_ISL_800257, EPI_ISL_800259 to 800261, EPI_ISL_800264 to 800265, EPI_ISL_800268 to 800270, EPI_ISL_800272, EPI_ISL_800275, EPI_ISL_800277 to 800279, EPI_ISL_800283 to 800285, EPI_ISL_800287 to 800290, EPI_ISL_800294 to 800297, EPI_ISL_800300, EPI_ISL_800302, EPI_ISL_800304 to 800307, EPI_ISL_800310, EPI_ISL_800313, EPI_ISL_800315 to 800316, EPI_ISL_800318 to 800321, EPI_ISL_800323 to 800326, EPI_ISL_800328, EPI_ISL_800330 to 800337, EPI_ISL_800343 to 800345, EPI_ISL_800349, EPI_ISL_800352 to 800353, EPI_ISL_800356, EPI_ISL_800358, EPI_ISL_800361, EPI_ISL_800363, EPI_ISL_800366, EPI_ISL_800368 to 800369, EPI_ISL_800371 to 800372, EPI_ISL_800377, EPI_ISL_800379, EPI_ISL_800381, EPI_ISL_800385 to 800386, EPI_ISL_800388 to 800389, EPI_ISL_800393 to 800394, EPI_ISL_800396 to 800398, EPI_ISL_800401 to 800402, EPI_ISL_800404 to 800406, EPI_ISL_800408 to 800409, EPI_ISL_800411, EPI_ISL_800414 to 800416, EPI_ISL_800418, EPI_ISL_800423 to 800424, EPI_ISL_800426, EPI_ISL_800430, EPI_ISL_800432 to 800436, EPI_ISL_800438, EPI_ISL_800442 to 800443, EPI_ISL_800445, EPI_ISL_800447 to 800448, EPI_ISL_800451, EPI_ISL_800461 to 800465, EPI_ISL_800467, EPI_ISL_800469, EPI_ISL_800472 to 800473, EPI_ISL_800475, EPI_ISL_800478 to 800479, EPI_ISL_800484, EPI_ISL_800489 to 800491, EPI_ISL_800493, EPI_ISL_800496 to 800500, EPI_ISL_800503 to 800505, EPI_ISL_800507 to 800508, EPI_ISL_800510, EPI_ISL_800512 to 800515, EPI_ISL_800517, EPI_ISL_800519 to 800521, EPI_ISL_800523, EPI_ISL_800527, EPI_ISL_800529, EPI_ISL_800533, EPI_ISL_800538 to 800540, EPI_ISL_800543, EPI_ISL_800545, EPI_ISL_800548 to 800550, EPI_ISL_800554, EPI_ISL_800556, EPI_ISL_800559 to 800561, EPI_ISL_800563, EPI_ISL_800565, EPI_ISL_800567 to 800568, EPI_ISL_800570, EPI_ISL_800573, EPI_ISL_800586 to 800587, EPI_ISL_800589 to 800590, EPI_ISL_800592, EPI_ISL_800597, EPI_ISL_800600, EPI_ISL_800606, EPI_ISL_800615, EPI_ISL_800637, EPI_ISL_800648 to 800649, EPI_ISL_800658, EPI_ISL_800663, EPI_ISL_800665, EPI_ISL_800672, EPI_ISL_800679, EPI_ISL_800682 to 800683, EPI_ISL_800686 to 800687, EPI_ISL_800690, EPI_ISL_800708 to 800709, EPI_ISL_800712, EPI_ISL_800720, EPI_ISL_800730, EPI_ISL_800734, EPI_ISL_800737, EPI_ISL_800763, EPI_ISL_800770 to 800771, EPI_ISL_800776, EPI_ISL_800784, EPI_ISL_800793, EPI_ISL_800795, EPI_ISL_800798, EPI_ISL_800802 to 800803, EPI_ISL_800808, EPI_ISL_800816 to 800817, EPI_ISL_800819, EPI_ISL_800828, EPI_ISL_800832, EPI_ISL_800839, EPI_ISL_800842, EPI_ISL_800854 to 800855, EPI_ISL_800859, EPI_ISL_800865, EPI_ISL_800872, EPI_ISL_800876, EPI_ISL_800894, EPI_ISL_800897, EPI_ISL_800899, EPI_ISL_800903, EPI_ISL_800908 to 800910, EPI_ISL_800912, EPI_ISL_800915 to 800917, EPI_ISL_800922 to 800923, EPI_ISL_800925, EPI_ISL_800929 to 800930, EPI_ISL_800933 to 800935, EPI_ISL_800938 to 800941, EPI_ISL_800946, EPI_ISL_800951 to 800952, EPI_ISL_800955 to 800956, EPI_ISL_800958, EPI_ISL_800961 to 800963, EPI_ISL_800965 to 800967, EPI_ISL_800976, EPI_ISL_800980, EPI_ISL_800985, EPI_ISL_800990 to 800991, EPI_ISL_800994, EPI_ISL_800997 to 800998, EPI_ISL_801001 to 801002, EPI_ISL_801006 to 801007, EPI_ISL_801010 to 801011, EPI_ISL_801013, EPI_ISL_801017 to 801018, EPI_ISL_801028, EPI_ISL_801033, EPI_ISL_801035, EPI_ISL_801039, EPI_ISL_801045, EPI_ISL_801047, EPI_ISL_801050, EPI_ISL_801054, EPI_ISL_801057, EPI_ISL_801064 to 801069, EPI_ISL_801072 to 801073, EPI_ISL_801077, EPI_ISL_801080, EPI_ISL_801082, EPI_ISL_801084, EPI_ISL_801090 to 801092, EPI_ISL_801095, EPI_ISL_801102, EPI_ISL_801107, EPI_ISL_801113, EPI_ISL_801118, EPI_ISL_801123, EPI_ISL_801126, EPI_ISL_801130, EPI_ISL_801132, EPI_ISL_801139 to 801140, EPI_ISL_801152, EPI_ISL_801154, EPI_ISL_801163, EPI_ISL_801166, EPI_ISL_801169, EPI_ISL_801171, EPI_ISL_801173 to 801174, EPI_ISL_811457 to 811459, EPI_ISL_811461 to 811465, EPI_ISL_811467 to 811468, EPI_ISL_811470 to 811471, EPI_ISL_811473 to 811477, EPI_ISL_811479 to 811481, EPI_ISL_811485 to 811486, EPI_ISL_811488 to 811489, EPI_ISL_811491 to 811493, EPI_ISL_811495 to 811497, EPI_ISL_811501, EPI_ISL_811504 to 811505, EPI_ISL_811507 to 811510, EPI_ISL_811512 to 811515, EPI_ISL_811517, EPI_ISL_811520 to 811522, EPI_ISL_811526 to 811529, EPI_ISL_811531 to 811532, EPI_ISL_811534 to 811537, EPI_ISL_811539 to 811540, EPI_ISL_811542 to 811544, EPI_ISL_811546 to 811547, EPI_ISL_811550 to 811555, EPI_ISL_811558, EPI_ISL_811560, EPI_ISL_811564, EPI_ISL_811567 to 811568, EPI_ISL_811570 to 811571, EPI_ISL_811576 to 811579, EPI_ISL_811581 to 811583, EPI_ISL_811585 to 811590, EPI_ISL_811592 to 811593, EPI_ISL_811595 to 811597, EPI_ISL_811599, EPI_ISL_811601, EPI_ISL_811604, EPI_ISL_811607 to 811608, EPI_ISL_811612 to 811613, EPI_ISL_811615 to 811616, EPI_ISL_811619, EPI_ISL_811623 to 811624, EPI_ISL_811628, EPI_ISL_811630 to 811632, EPI_ISL_811635 to 811637, EPI_ISL_811639 to 811641, EPI_ISL_811646 to 811649, EPI_ISL_811652 to 811656, EPI_ISL_811658 to 811659, EPI_ISL_811662 to 811663, EPI_ISL_811665, EPI_ISL_811667 to 811668, EPI_ISL_811674 to 811676, EPI_ISL_811680 to 811681, EPI_ISL_811683, EPI_ISL_811685 to 811687, EPI_ISL_811690 to 811695, EPI_ISL_811697, EPI_ISL_811699 to 811700, EPI_ISL_811704 to 811707, EPI_ISL_811709 to 811711, EPI_ISL_811713 to 811719, EPI_ISL_811723 to 811725, EPI_ISL_811728 to 811730, EPI_ISL_811732 to 811735, EPI_ISL_811737 to 811739, EPI_ISL_811743, EPI_ISL_811745 to 811746, EPI_ISL_811748 to 811749, EPI_ISL_811752, EPI_ISL_811757 to 811758, EPI_ISL_811760 to 811761, EPI_ISL_811763 to 811766, EPI_ISL_811768 to 811771, EPI_ISL_811777 to 811784, EPI_ISL_811786, EPI_ISL_811790, EPI_ISL_811792 to 811794, EPI_ISL_811796 to 811801, EPI_ISL_811803 to 811806, EPI_ISL_821276, EPI_ISL_821279, EPI_ISL_821284, EPI_ISL_821286, EPI_ISL_821289 to 821290, EPI_ISL_821295, EPI_ISL_821309, EPI_ISL_821311, EPI_ISL_821316, EPI_ISL_821320, EPI_ISL_821333, EPI_ISL_821338 to 821339, EPI_ISL_821342 to 821343, EPI_ISL_821354, EPI_ISL_821360, EPI_ISL_821365, EPI_ISL_821367 to 821368,

EPI_ISL_821374 to 821375, EPI_ISL_821386, EPI_ISL_821392 to 821394, EPI_ISL_821399, EPI_ISL_821408, EPI_ISL_821411, EPI_ISL_821416, EPI_ISL_821421, EPI_ISL_821427, EPI_ISL_821439, EPI_ISL_821441 to 821442, EPI_ISL_821444, EPI_ISL_821463, EPI_ISL_821473, EPI_ISL_821479, EPI_ISL_821484 to
821485, EPI_ISL_821488, EPI_ISL_821492, EPI_ISL_821506, EPI_ISL_821510, EPI_ISL_821513, EPI_ISL_821516, EPI_ISL_821519, EPI_ISL_821523, EPI_ISL_821529, EPI_ISL_821546 to 821547, EPI_ISL_821549, EPI_ISL_821555, EPI_ISL_821563 to 821565, EPI_ISL_821568, EPI_ISL_821570, EPI_ISL_821576 to 821577,
EPI_ISL_821579, EPI_ISL_821583, EPI_ISL_821588, EPI_ISL_821590, EPI_ISL_821592 to 821593, EPI_ISL_821596, EPI_ISL_821604 to 821605, EPI_ISL_821608, EPI_ISL_821613, EPI_ISL_821616, EPI_ISL_821618, EPI_ISL_821620, EPI_ISL_821622 to 821624, EPI_ISL_821662 to 821666, EPI_ISL_821669 to 821670,
EPI_ISL_821672 to 821674, EPI_ISL_821677 to 821682, EPI_ISL_821684 to 821685, EPI_ISL_821689 to 821697, EPI_ISL_821699 to 821701, EPI_ISL_821703 to 821709, EPI_ISL_821711, EPI_ISL_821713 to 821717, EPI_ISL_821719 to 821725, EPI_ISL_821727 to 821728, EPI_ISL_821730 to 821734, EPI_ISL_821736,
EPI_ISL_821738 to 821740, EPI_ISL_821742 to 821745, EPI_ISL_821748 to 821755, EPI_ISL_821757, EPI_ISL_821760 to 821763, EPI_ISL_821765 to 821766, EPI_ISL_821769, EPI_ISL_821771 to 821772, EPI_ISL_821777 to 821780, EPI_ISL_821783 to 821786, EPI_ISL_821788 to 821791, EPI_ISL_821793, EPI_ISL_821796,
EPI_ISL_821799 to 821801, EPI_ISL_821803 to 821809, EPI_ISL_821811 to 821814, EPI_ISL_821816, EPI_ISL_821818 to 821826, EPI_ISL_821830 to 821838, EPI_ISL_821842 to 821846, EPI_ISL_821849 to 821852, EPI_ISL_821854 to 821860, EPI_ISL_821862 to 821864, EPI_ISL_821866, EPI_ISL_821869 to 821873,
EPI_ISL_821876 to 821881, EPI_ISL_821883, EPI_ISL_821885, EPI_ISL_821888 to 821889, EPI_ISL_821891 to 821892, EPI_ISL_821894 to 821903, EPI_ISL_821906 to 821907, EPI_ISL_821909, EPI_ISL_821913 to 821914, EPI_ISL_821916 to 821918, EPI_ISL_821920 to 821924, EPI_ISL_821926 to 821930, EPI_ISL_821932
to 821933, EPI_ISL_821936 to 821950, EPI_ISL_821952 to 821957, EPI_ISL_821959, EPI_ISL_821961 to 821962, EPI_ISL_821965 to 821966, EPI_ISL_821968, EPI_ISL_822299, EPI_ISL_822301, EPI_ISL_826473, EPI_ISL_833585, EPI_ISL_833587 to 833589, EPI_ISL_833591 to 833592, EPI_ISL_833594 to 833595,
EPI_ISL_833598, EPI_ISL_833603, EPI_ISL_833605, EPI_ISL_833608 to 833610, EPI_ISL_833613, EPI_ISL_833616, EPI_ISL_833622 to 833626, EPI_ISL_833631, EPI_ISL_833633, EPI_ISL_833635 to 833636, EPI_ISL_833638 to 833642, EPI_ISL_833644 to 833645, EPI_ISL_833649 to 833651, EPI_ISL_833653 to 833655,
EPI_ISL_833657, EPI_ISL_833660, EPI_ISL_833662 to 833666, EPI_ISL_833669 to 833675, EPI_ISL_833677, EPI_ISL_833679 to 833683, EPI_ISL_833686 to 833692, EPI_ISL_833694, EPI_ISL_833696 to 833697, EPI_ISL_833699, EPI_ISL_833701 to 833711, EPI_ISL_833713 to 833715, EPI_ISL_833717 to 833718,
EPI_ISL_833720, EPI_ISL_833722 to 833723, EPI_ISL_833727 to 833733, EPI_ISL_833737, EPI_ISL_833739 to 833741, EPI_ISL_833744 to 833745, EPI_ISL_833747 to 833753, EPI_ISL_833757 to 833758, EPI_ISL_833760 to 833761, EPI_ISL_833763 to 833765, EPI_ISL_833770 to 833774, EPI_ISL_833776 to 833778,
EPI_ISL_833781, EPI_ISL_833783, EPI_ISL_833785 to 833787, EPI_ISL_833790, EPI_ISL_833792 to 833793, EPI_ISL_833799 to 833800, EPI_ISL_833802, EPI_ISL_833806 to 833808, EPI_ISL_833810, EPI_ISL_833812 to 833813, EPI_ISL_833815 to 833816, EPI_ISL_833819 to 833820, EPI_ISL_833822, EPI_ISL_833824 to
833829, EPI_ISL_833831 to 833833, EPI_ISL_833836 to 833838, EPI_ISL_833840, EPI_ISL_833843 to 833844, EPI_ISL_833847, EPI_ISL_833851 to 833852, EPI_ISL_833854, EPI_ISL_833857, EPI_ISL_833860, EPI_ISL_833862 to 833864, EPI_ISL_833866 to 833867, EPI_ISL_833869, EPI_ISL_833871, EPI_ISL_833873 to
833878, EPI_ISL_833880, EPI_ISL_833882 to 833883, EPI_ISL_833885 to 833888, EPI_ISL_833891, EPI_ISL_833893, EPI_ISL_833897 to 833900, EPI_ISL_833902 to 833907, EPI_ISL_833909, EPI_ISL_833911 to 833915, EPI_ISL_833917 to 833919, EPI_ISL_833921, EPI_ISL_833923, EPI_ISL_833925, EPI_ISL_833927 to
833928, EPI_ISL_833932, EPI_ISL_836421 to 836427, EPI_ISL_836429 to 836431, EPI_ISL_836433, EPI_ISL_836436 to 836442, EPI_ISL_836444, EPI_ISL_836446 to 836458, EPI_ISL_836460 to 836463, EPI_ISL_836465, EPI_ISL_836467 to 836468, EPI_ISL_836470, EPI_ISL_836473 to 836476, EPI_ISL_836478 to 836480,
EPI_ISL_836482, EPI_ISL_836484 to 836486, EPI_ISL_836488 to 836494, EPI_ISL_836496 to 836499, EPI_ISL_836501 to 836505, EPI_ISL_836507 to 836509, EPI_ISL_836511 to 836513, EPI_ISL_836515 to 836517, EPI_ISL_836521 to 836522, EPI_ISL_836524 to 836525, EPI_ISL_836527 to 836530, EPI_ISL_836534,
EPI_ISL_836536 to 836538, EPI_ISL_836540 to 836543, EPI_ISL_836545 to 836548, EPI_ISL_836550 to 836553, EPI_ISL_836556 to 836557, EPI_ISL_836559 to 836560, EPI_ISL_836562 to 836564, EPI_ISL_836566 to 836568, EPI_ISL_836570 to 836573, EPI_ISL_836575 to 836579, EPI_ISL_836581, EPI_ISL_836583 to
836587, EPI_ISL_836589, EPI_ISL_836591 to 836595, EPI_ISL_836597 to 836599, EPI_ISL_836601, EPI_ISL_836604 to 836609, EPI_ISL_836611, EPI_ISL_836613, EPI_ISL_836615, EPI_ISL_836617 to 836624, EPI_ISL_836627, EPI_ISL_836629 to 836630, EPI_ISL_836632 to 836637, EPI_ISL_836639 to 836641,
EPI_ISL_836643 to 836652, EPI_ISL_836654 to 836655, EPI_ISL_836658, EPI_ISL_836660 to 836662, EPI_ISL_836665, EPI_ISL_836667 to 836679, EPI_ISL_836681, EPI_ISL_836684, EPI_ISL_836686 to 836698, EPI_ISL_836701 to 836709, EPI_ISL_836711 to 836724, EPI_ISL_836727 to 836730, EPI_ISL_836732 to 836734,
EPI_ISL_836736 to 836737, EPI_ISL_836739 to 836740, EPI_ISL_836743 to 836744, EPI_ISL_836746, EPI_ISL_845893, EPI_ISL_845895 to 845897, EPI_ISL_845899 to 845902, EPI_ISL_845904 to 845909, EPI_ISL_845912 to 845914, EPI_ISL_845916 to 845920, EPI_ISL_845922 to 845924, EPI_ISL_845927 to 845928,
EPI_ISL_845930 to 845932, EPI_ISL_845935 to 845950, EPI_ISL_845952 to 845953, EPI_ISL_845955 to 845959, EPI_ISL_845961 to 845965, EPI_ISL_845967, EPI_ISL_845969 to 845973, EPI_ISL_845975 to 845978, EPI_ISL_845980 to 845987, EPI_ISL_845989 to 845992, EPI_ISL_845994, EPI_ISL_845996, EPI_ISL_845998
to 846000, EPI_ISL_846002, EPI_ISL_846004 to 846006, EPI_ISL_846008 to 846010, EPI_ISL_846012 to 846013, EPI_ISL_846015 to 846016, EPI_ISL_846020 to 846021, EPI_ISL_846023 to 846024, EPI_ISL_846026 to 846032, EPI_ISL_846034 to 846035, EPI_ISL_846037 to 846039, EPI_ISL_846042 to 846043,
EPI_ISL_846045 to 846053, EPI_ISL_846055, EPI_ISL_846058 to 846062, EPI_ISL_846064, EPI_ISL_846067 to 846073, EPI_ISL_846075 to 846078, EPI_ISL_846080 to 846083, EPI_ISL_846085 to 846087, EPI_ISL_846089 to 846091, EPI_ISL_846093 to 846095, EPI_ISL_846097 to 846100, EPI_ISL_846103 to 846105,
EPI_ISL_846107 to 846112, EPI_ISL_846114, EPI_ISL_846116, EPI_ISL_846118 to 846125, EPI_ISL_846128 to 846129, EPI_ISL_846131, EPI_ISL_846133 to 846141, EPI_ISL_846146 to 846150, EPI_ISL_846152 to 846156, EPI_ISL_846159 to 846168, EPI_ISL_846170, EPI_ISL_846172 to 846174, EPI_ISL_846176 to 846177,
EPI_ISL_846179, EPI_ISL_846182 to 846184, EPI_ISL_846186 to 846197, EPI_ISL_846509, EPI_ISL_851062, EPI_ISL_851064, EPI_ISL_851068 to 851069, EPI_ISL_851071, EPI_ISL_851073 to 851076, EPI_ISL_851079, EPI_ISL_851084, EPI_ISL_851086 to 851088, EPI_ISL_851090 to 851092, EPI_ISL_851097 to 851100,
EPI_ISL_851103, EPI_ISL_851106, EPI_ISL_851108 to 851112, EPI_ISL_851114 to 851115, EPI_ISL_851118, EPI_ISL_851120 to 851123, EPI_ISL_851127 to 851128, EPI_ISL_851138, EPI_ISL_851140, EPI_ISL_851144 to 851145, EPI_ISL_851149 to 851153, EPI_ISL_851155 to 851160, EPI_ISL_851165, EPI_ISL_851169 to
851177, EPI_ISL_851179, EPI_ISL_851183 to 851185, EPI_ISL_851188 to 851189, EPI_ISL_851191 to 851192, EPI_ISL_851194, EPI_ISL_851199, EPI_ISL_851201, EPI_ISL_851209, EPI_ISL_851211 to 851214, EPI_ISL_851216, EPI_ISL_851220 to 851223, EPI_ISL_851226 to 851228, EPI_ISL_851230 to 851231,
EPI_ISL_851234 to 851235, EPI_ISL_851238, EPI_ISL_851240, EPI_ISL_851244 to 851245, EPI_ISL_851248 to 851251, EPI_ISL_851254 to 851256, EPI_ISL_851260 to 851262, EPI_ISL_851265, EPI_ISL_851267 to 851269, EPI_ISL_851271 to 851272, EPI_ISL_851274 to 851275, EPI_ISL_851277, EPI_ISL_851279 to 851284,
EPI_ISL_851287 to 851290, EPI_ISL_851292 to 851295, EPI_ISL_851297 to 851300, EPI_ISL_851304, EPI_ISL_851308, EPI_ISL_851311, EPI_ISL_851315 to 851317, EPI_ISL_851320 to 851324, EPI_ISL_851326, EPI_ISL_851328, EPI_ISL_851330, EPI_ISL_851333 to 851334, EPI_ISL_851336 to 851337, EPI_ISL_851339 to
851340, EPI_ISL_851342 to 851344, EPI_ISL_851348 to 851350, EPI_ISL_851352 to 851357, EPI_ISL_851359 to 851364, EPI_ISL_851366, EPI_ISL_851368 to 851369, EPI_ISL_851372, EPI_ISL_851375 to 851379, EPI_ISL_857543, EPI_ISL_857546 to 857547, EPI_ISL_857549, EPI_ISL_857554 to 857557, EPI_ISL_857560 to
857561, EPI_ISL_857563 to 857564, EPI_ISL_857566 to 857567, EPI_ISL_857569, EPI_ISL_857571 to 857574, EPI_ISL_857576 to 857578, EPI_ISL_857580 to 857583, EPI_ISL_857585 to 857596, EPI_ISL_857598, EPI_ISL_857601 to 857602, EPI_ISL_857606 to 857613, EPI_ISL_857617 to 857619, EPI_ISL_857622 to
857630, EPI_ISL_857632 to 857638, EPI_ISL_857640 to 857641, EPI_ISL_857643, EPI_ISL_857646, EPI_ISL_857649 to 857653, EPI_ISL_857655, EPI_ISL_857657, EPI_ISL_857659 to 857662, EPI_ISL_857664 to 857668, EPI_ISL_857670 to 857671, EPI_ISL_857673 to 857678, EPI_ISL_857681, EPI_ISL_857684 to 857685,
EPI_ISL_857687, EPI_ISL_857689, EPI_ISL_857691, EPI_ISL_857693 to 857695, EPI_ISL_857697 to 857698, EPI_ISL_857703, EPI_ISL_857705 to 857706, EPI_ISL_857708 to 857710, EPI_ISL_857712 to 857713, EPI_ISL_857715 to 857716, EPI_ISL_857718 to 857725, EPI_ISL_857727 to 857729, EPI_ISL_857731,
EPI_ISL_857733 to 857738, EPI_ISL_857740 to 857743, EPI_ISL_857745 to 857749, EPI_ISL_857751 to 857752, EPI_ISL_857756 to 857757, EPI_ISL_857759 to 857763, EPI_ISL_857766, EPI_ISL_857768 to 857770, EPI_ISL_857772 to 857773, EPI_ISL_857775 to 857778, EPI_ISL_857780 to 857783, EPI_ISL_857785 to
857804, EPI_ISL_857806, EPI_ISL_857809 to 857810, EPI_ISL_857812 to 857816, EPI_ISL_857820 to 857829, EPI_ISL_857831, EPI_ISL_857833 to 857837, EPI_ISL_857839 to 857851, EPI_ISL_857853 to 857859, EPI_ISL_857863, EPI_ISL_857866 to 857869, EPI_ISL_873282 to 873283, EPI_ISL_873286, EPI_ISL_873292,
EPI_ISL_873295, EPI_ISL_873297, EPI_ISL_873302, EPI_ISL_873304, EPI_ISL_873306, EPI_ISL_873313, EPI_ISL_873316, EPI_ISL_873318, EPI_ISL_873320, EPI_ISL_873325, EPI_ISL_873334, EPI_ISL_873339, EPI_ISL_873341 to 873343, EPI_ISL_873347 to 873348, EPI_ISL_873350, EPI_ISL_873357, EPI_ISL_873359,
EPI_ISL_873362, EPI_ISL_873364 to 873365, EPI_ISL_873368 to 873371, EPI_ISL_873374, EPI_ISL_873381, EPI_ISL_873388, EPI_ISL_873399, EPI_ISL_873416, EPI_ISL_873418, EPI_ISL_873422, EPI_ISL_873426, EPI_ISL_873428, EPI_ISL_873431 to 873432, EPI_ISL_873443, EPI_ISL_873451, EPI_ISL_873454,
EPI_ISL_873457, EPI_ISL_873459, EPI_ISL_873476, EPI_ISL_873481 to 873482, EPI_ISL_873489, EPI_ISL_873492, EPI_ISL_873494 to 873495, EPI_ISL_873499 to 873501, EPI_ISL_873513, EPI_ISL_873518, EPI_ISL_873532, EPI_ISL_873537, EPI_ISL_873561, EPI_ISL_873566, EPI_ISL_873573 to 873574,
EPI_ISL_873576, EPI_ISL_873605, EPI_ISL_873613 to 873614, EPI_ISL_873621, EPI_ISL_873624 to 873625, EPI_ISL_873630, EPI_ISL_873635 to 873636, EPI_ISL_873639 to 873640, EPI_ISL_873642 to 873644, EPI_ISL_873646 to 873647, EPI_ISL_873650 to 873651, EPI_ISL_873653, EPI_ISL_873655, EPI_ISL_873659,
EPI_ISL_873661 to 873668, EPI_ISL_873671, EPI_ISL_873673 to 873676, EPI_ISL_873678, EPI_ISL_873680 to 873683, EPI_ISL_873685, EPI_ISL_873687 to 873688, EPI_ISL_873690 to 873692, EPI_ISL_873694 to 873696, EPI_ISL_873698 to 873701, EPI_ISL_873705, EPI_ISL_873708 to 873709, EPI_ISL_873711 to 873713,
EPI_ISL_873715, EPI_ISL_873718 to 873722, EPI_ISL_873727, EPI_ISL_873731 to 873733, EPI_ISL_873736 to 873737, EPI_ISL_873739, EPI_ISL_873742, EPI_ISL_873747, EPI_ISL_873750 to 873754, EPI_ISL_873756 to 873757, EPI_ISL_873759, EPI_ISL_873761 to 873762, EPI_ISL_873765 to 873767, EPI_ISL_873770,
EPI_ISL_873773 to 873774, EPI_ISL_873776 to 873782, EPI_ISL_873784, EPI_ISL_873787, EPI_ISL_873791 to 873793, EPI_ISL_873796, EPI_ISL_873798, EPI_ISL_873800 to 873801, EPI_ISL_873804, EPI_ISL_873811, EPI_ISL_873813 to 873816, EPI_ISL_873818 to 873821, EPI_ISL_873826, EPI_ISL_873828,
EPI_ISL_873831 to 873832, EPI_ISL_873836 to 873839, EPI_ISL_873841, EPI_ISL_873843 to 873846, EPI_ISL_873849, EPI_ISL_873852, EPI_ISL_873855, EPI_ISL_873858, EPI_ISL_873861, EPI_ISL_873863, EPI_ISL_873866 to 873869, EPI_ISL_873871 to 873872, EPI_ISL_873876 to 873878, EPI_ISL_873882 to 873883,
EPI_ISL_873889 to 873891, EPI_ISL_873894 to 873898, EPI_ISL_873901 to 873903, EPI_ISL_873907 to 873909, EPI_ISL_873912, EPI_ISL_873915, EPI_ISL_873917 to 873919, EPI_ISL_873922, EPI_ISL_873929, EPI_ISL_873931, EPI_ISL_873936, EPI_ISL_873938 to 873939, EPI_ISL_873944, EPI_ISL_873946,
EPI_ISL_873949, EPI_ISL_873952 to 873953, EPI_ISL_873957 to 873959, EPI_ISL_878728, EPI_ISL_878731, EPI_ISL_878733, EPI_ISL_878735, EPI_ISL_878737 to 878738, EPI_ISL_878740, EPI_ISL_878745, EPI_ISL_878748 to 878749, EPI_ISL_878753, EPI_ISL_878758, EPI_ISL_878761 to 878762, EPI_ISL_878764,
EPI_ISL_878766 to 878767, EPI_ISL_878770, EPI_ISL_878774, EPI_ISL_878783, EPI_ISL_878788, EPI_ISL_878795, EPI_ISL_878799, EPI_ISL_878802, EPI_ISL_878811, EPI_ISL_878813, EPI_ISL_878816, EPI_ISL_878823, EPI_ISL_878826 to 878832, EPI_ISL_878834, EPI_ISL_878838 to 878839, EPI_ISL_878847,
EPI_ISL_878862 to 878863, EPI_ISL_878867, EPI_ISL_878869 to 878873, EPI_ISL_878877, EPI_ISL_878881, EPI_ISL_878885, EPI_ISL_878889, EPI_ISL_878893 to 878895, EPI_ISL_878897 to 878901, EPI_ISL_878903, EPI_ISL_878905, EPI_ISL_878908, EPI_ISL_878910 to 878912, EPI_ISL_878916, EPI_ISL_878921,
EPI_ISL_878923 to 878924, EPI_ISL_878928, EPI_ISL_878930 to 878931, EPI_ISL_878933, EPI_ISL_878935, EPI_ISL_878937, EPI_ISL_878940, EPI_ISL_878948 to 878949, EPI_ISL_878954, EPI_ISL_878957, EPI_ISL_878963 to 878964, EPI_ISL_878968 to 878969, EPI_ISL_878973 to 878978, EPI_ISL_878980,
EPI_ISL_878984, EPI_ISL_878986 to 878989, EPI_ISL_878991, EPI_ISL_878996 to 878998, EPI_ISL_879004 to 879005, EPI_ISL_879010 to 879012, EPI_ISL_879017, EPI_ISL_879020, EPI_ISL_879024, EPI_ISL_879027, EPI_ISL_879029 to 879031, EPI_ISL_879035 to 879036, EPI_ISL_879041, EPI_ISL_879045 to 879046,
EPI_ISL_879048, EPI_ISL_879053 to 879054, EPI_ISL_879059, EPI_ISL_879062, EPI_ISL_879067 to 879069, EPI_ISL_879071, EPI_ISL_879074, EPI_ISL_879076, EPI_ISL_879078, EPI_ISL_879082, EPI_ISL_879095, EPI_ISL_879102, EPI_ISL_879105, EPI_ISL_879107, EPI_ISL_879112, EPI_ISL_879115, EPI_ISL_879122 to
879123, EPI_ISL_879127, EPI_ISL_879133, EPI_ISL_879135, EPI_ISL_879139, EPI_ISL_879141, EPI_ISL_879143, EPI_ISL_879150, EPI_ISL_879153, EPI_ISL_879155 to 879156, EPI_ISL_879158, EPI_ISL_879163, EPI_ISL_879165 to 879166, EPI_ISL_879170, EPI_ISL_879174, EPI_ISL_879176 to 879178, EPI_ISL_879180,
EPI_ISL_879182 to 879184, EPI_ISL_879189 to 879190, EPI_ISL_879196 to 879198, EPI_ISL_879204 to 879205, EPI_ISL_879209 to 879210, EPI_ISL_879213, EPI_ISL_879215 to 879217, EPI_ISL_879221 to 879223, EPI_ISL_879228, EPI_ISL_879232 to 879233, EPI_ISL_879236, EPI_ISL_879240, EPI_ISL_879242,
EPI_ISL_879246, EPI_ISL_879251, EPI_ISL_879257, EPI_ISL_879260 to 879261, EPI_ISL_879264, EPI_ISL_879267, EPI_ISL_879269, EPI_ISL_879282 to 879283, EPI_ISL_879285, EPI_ISL_879287 to 879289, EPI_ISL_879291 to 879295, EPI_ISL_879301, EPI_ISL_879309, EPI_ISL_879311 to 879312, EPI_ISL_879315,
EPI_ISL_879317, EPI_ISL_879319 to 879321, EPI_ISL_879325 to 879326, EPI_ISL_879328, EPI_ISL_879330, EPI_ISL_879332, EPI_ISL_879334 to 879336, EPI_ISL_879349, EPI_ISL_879352 to 879353, EPI_ISL_879364, EPI_ISL_879366, EPI_ISL_879368 to 879370, EPI_ISL_879372, EPI_ISL_879381, EPI_ISL_879383,
EPI_ISL_879385, EPI_ISL_879387, EPI_ISL_879389 to 879391, EPI_ISL_879394, EPI_ISL_879398, EPI_ISL_879400, EPI_ISL_879402, EPI_ISL_879405 to 879408, EPI_ISL_879410 to 879411, EPI_ISL_879414, EPI_ISL_879419, EPI_ISL_879421 to 879422, EPI_ISL_879428, EPI_ISL_879430 to 879432, EPI_ISL_879435 to
879441, EPI_ISL_879443, EPI_ISL_879445, EPI_ISL_879447, EPI_ISL_879454, EPI_ISL_879463, EPI_ISL_879468 to 879469, EPI_ISL_879472, EPI_ISL_879481 to 879482, EPI_ISL_879484 to 879485, EPI_ISL_879488 to 879490, EPI_ISL_879494, EPI_ISL_879498, EPI_ISL_879500, EPI_ISL_879503, EPI_ISL_879506 to
879507, EPI_ISL_879509, EPI_ISL_879511 to 879513, EPI_ISL_879516 to 879517, EPI_ISL_879521, EPI_ISL_879523, EPI_ISL_879525, EPI_ISL_879528 to 879529, EPI_ISL_879531, EPI_ISL_879533, EPI_ISL_879537, EPI_ISL_879541, EPI_ISL_879543 to 879544, EPI_ISL_879551, EPI_ISL_879557 to 879559,
EPI_ISL_879562, EPI_ISL_879567, EPI_ISL_879569 to 879570, EPI_ISL_879579 to 879580, EPI_ISL_879583 to 879584, EPI_ISL_879588, EPI_ISL_879590, EPI_ISL_879595, EPI_ISL_879598, EPI_ISL_879605, EPI_ISL_879607, EPI_ISL_879609, EPI_ISL_879612, EPI_ISL_879622, EPI_ISL_879624 to 879626,
EPI_ISL_879630, EPI_ISL_879632 to 879638, EPI_ISL_879640, EPI_ISL_879648, EPI_ISL_879650 to 879652, EPI_ISL_879658 to 879662, EPI_ISL_879667 to 879668, EPI_ISL_879670 to 879671, EPI_ISL_879673, EPI_ISL_879676, EPI_ISL_879680, EPI_ISL_879684 to 879685, EPI_ISL_879690 to 879691, EPI_ISL_879694,
EPI_ISL_879696 to 879699, EPI_ISL_879701, EPI_ISL_879705 to 879706, EPI_ISL_879708 to 879709, EPI_ISL_879716, EPI_ISL_879720, EPI_ISL_879722 to 879725, EPI_ISL_879727, EPI_ISL_879730 to 879732, EPI_ISL_879734 to 879735, EPI_ISL_879738 to 879739, EPI_ISL_879741, EPI_ISL_879745 to 879746,
EPI_ISL_879749, EPI_ISL_879754, EPI_ISL_879764, EPI_ISL_879769 to 879774, EPI_ISL_879776 to 879777, EPI_ISL_879779, EPI_ISL_879782, EPI_ISL_879786, EPI_ISL_879790, EPI_ISL_879796 to 879798, EPI_ISL_879806 to 879807, EPI_ISL_879812, EPI_ISL_879815, EPI_ISL_879818, EPI_ISL_879820,
EPI_ISL_879823 to 879824, EPI_ISL_879828, EPI_ISL_879830, EPI_ISL_879832 to 879835, EPI_ISL_879839, EPI_ISL_879845, EPI_ISL_879847, EPI_ISL_879850, EPI_ISL_879853, EPI_ISL_879861, EPI_ISL_879864, EPI_ISL_879867, EPI_ISL_879874, EPI_ISL_879876 to 879877, EPI_ISL_879883, EPI_ISL_879889 to
879891, EPI_ISL_879893, EPI_ISL_879896, EPI_ISL_879899 to 879900, EPI_ISL_879902, EPI_ISL_879904, EPI_ISL_879908 to 879909, EPI_ISL_879921, EPI_ISL_879928, EPI_ISL_879930, EPI_ISL_879935, EPI_ISL_879940 to 879941, EPI_ISL_879944, EPI_ISL_879946, EPI_ISL_879951 to 879952, EPI_ISL_879954,
EPI_ISL_879960, EPI_ISL_879963, EPI_ISL_879983, EPI_ISL_879989, EPI_ISL_879992, EPI_ISL_879994, EPI_ISL_879996, EPI_ISL_880001, EPI_ISL_880004, EPI_ISL_880013, EPI_ISL_880015, EPI_ISL_880025, EPI_ISL_880033, EPI_ISL_880035, EPI_ISL_880038 to 880039, EPI_ISL_880044, EPI_ISL_880047,
EPI_ISL_880049, EPI_ISL_880051, EPI_ISL_880054, EPI_ISL_880060, EPI_ISL_880065, EPI_ISL_880068, EPI_ISL_880070, EPI_ISL_880075, EPI_ISL_880078, EPI_ISL_880080 to 880081, EPI_ISL_881619 to 881620, EPI_ISL_881622, EPI_ISL_891274 to 891277, EPI_ISL_891282, EPI_ISL_891287, EPI_ISL_891289 to
891293, EPI_ISL_891296, EPI_ISL_891300, EPI_ISL_891302 to 891303, EPI_ISL_891307 to 891309, EPI_ISL_891321 to 891322, EPI_ISL_891325, EPI_ISL_891328 to 891329, EPI_ISL_891331, EPI_ISL_891333, EPI_ISL_891336, EPI_ISL_891339, EPI_ISL_891341, EPI_ISL_891343, EPI_ISL_891346, EPI_ISL_891348,
EPI_ISL_891350, EPI_ISL_891354, EPI_ISL_891356, EPI_ISL_891359, EPI_ISL_891362 to 891363, EPI_ISL_891367, EPI_ISL_891370, EPI_ISL_891373, EPI_ISL_891381, EPI_ISL_891387 to 891390, EPI_ISL_891394 to 891395, EPI_ISL_891397, EPI_ISL_891407 to 891409, EPI_ISL_891412 to 891414, EPI_ISL_891418,
EPI_ISL_891422, EPI_ISL_891425, EPI_ISL_891427 to 891429, EPI_ISL_891431 to 891432, EPI_ISL_891434, EPI_ISL_891441 to 891443, EPI_ISL_891447, EPI_ISL_891449, EPI_ISL_891452, EPI_ISL_891457, EPI_ISL_891461, EPI_ISL_891466 to 891468, EPI_ISL_891472, EPI_ISL_891475 to 891477, EPI_ISL_891480 to
891483, EPI_ISL_891488, EPI_ISL_891492 to 891493, EPI_ISL_891498, EPI_ISL_891500 to 891501, EPI_ISL_891506, EPI_ISL_891510, EPI_ISL_891513, EPI_ISL_891515, EPI_ISL_891517 to 891518, EPI_ISL_891521, EPI_ISL_891524, EPI_ISL_891526, EPI_ISL_891530, EPI_ISL_891533 to 891535, EPI_ISL_891540 to
891541, EPI_ISL_891543, EPI_ISL_891546, EPI_ISL_891550 to 891554, EPI_ISL_891557, EPI_ISL_891561, EPI_ISL_891565 to 891567, EPI_ISL_891570, EPI_ISL_891573, EPI_ISL_891575 to 891581, EPI_ISL_891583 to 891584, EPI_ISL_891589 to 891595, EPI_ISL_891597 to 891598, EPI_ISL_891601, EPI_ISL_891607,
EPI_ISL_891614, EPI_ISL_891619 to 891621, EPI_ISL_891627, EPI_ISL_891631 to 891632, EPI_ISL_891637 to 891638, EPI_ISL_891641, EPI_ISL_891644, EPI_ISL_891646, EPI_ISL_891649, EPI_ISL_891651, EPI_ISL_891653 to 891655, EPI_ISL_891657, EPI_ISL_891660, EPI_ISL_891663, EPI_ISL_891665 to 891667,
EPI_ISL_891669, EPI_ISL_891671, EPI_ISL_891674, EPI_ISL_891676, EPI_ISL_891679, EPI_ISL_891682, EPI_ISL_891684, EPI_ISL_891686, EPI_ISL_891688, EPI_ISL_891690 to 891693, EPI_ISL_891696, EPI_ISL_891701 to 891703, EPI_ISL_891706 to 891709, EPI_ISL_891712, EPI_ISL_891715 to 891716,
EPI_ISL_891720, EPI_ISL_891728, EPI_ISL_891730, EPI_ISL_891732 to 891734, EPI_ISL_891737, EPI_ISL_891739, EPI_ISL_891744, EPI_ISL_891748, EPI_ISL_891750, EPI_ISL_891752, EPI_ISL_891754 to 891756, EPI_ISL_891759, EPI_ISL_891763 to 891764, EPI_ISL_891767 to 891768, EPI_ISL_891770,
EPI_ISL_891778 to 891779, EPI_ISL_891781 to 891785, EPI_ISL_891792, EPI_ISL_891795, EPI_ISL_891797 to 891798, EPI_ISL_891800 to 891801, EPI_ISL_891803, EPI_ISL_891805 to 891806, EPI_ISL_891808, EPI_ISL_891810, EPI_ISL_891812, EPI_ISL_891814 to 891818, EPI_ISL_891820, EPI_ISL_891825,
EPI_ISL_891829 to 891832, EPI_ISL_891835, EPI_ISL_891837, EPI_ISL_891842, EPI_ISL_891849, EPI_ISL_891854, EPI_ISL_891859, EPI_ISL_891862, EPI_ISL_891868, EPI_ISL_891871 to 891872, EPI_ISL_891876 to 891877, EPI_ISL_891880, EPI_ISL_891882 to 891883, EPI_ISL_891886, EPI_ISL_891888 to 891894,
EPI_ISL_891896 to 891898, EPI_ISL_907119, EPI_ISL_907133, EPI_ISL_907136, EPI_ISL_907139, EPI_ISL_907147 to 907148, EPI_ISL_907152, EPI_ISL_907155, EPI_ISL_907160, EPI_ISL_907164, EPI_ISL_907168, EPI_ISL_907173, EPI_ISL_907179, EPI_ISL_907187, EPI_ISL_907190, EPI_ISL_907192 to 907194,
EPI_ISL_907200, EPI_ISL_907206, EPI_ISL_907214, EPI_ISL_907219, EPI_ISL_907221 to 907222, EPI_ISL_907225, EPI_ISL_907238, EPI_ISL_907241, EPI_ISL_907254, EPI_ISL_907256, EPI_ISL_907260, EPI_ISL_907263 to 907264, EPI_ISL_907269, EPI_ISL_907273, EPI_ISL_907281, EPI_ISL_907284, EPI_ISL_907288,
EPI_ISL_907300, EPI_ISL_907303 to 907304, EPI_ISL_907308, EPI_ISL_907315, EPI_ISL_907318, EPI_ISL_907321 to 907323, EPI_ISL_907325, EPI_ISL_907328, EPI_ISL_907336, EPI_ISL_907341, EPI_ISL_907347 to 907348, EPI_ISL_907350, EPI_ISL_907352 to 907353, EPI_ISL_907355, EPI_ISL_907359,
EPI_ISL_907362 to 907363, EPI_ISL_907367, EPI_ISL_908035, EPI_ISL_908037 to 908041, EPI_ISL_908043 to 908048, EPI_ISL_908050, EPI_ISL_908052, EPI_ISL_908055, EPI_ISL_908057 to 908058, EPI_ISL_908062 to 908063, EPI_ISL_908065 to 908069, EPI_ISL_908071, EPI_ISL_908076 to 908080, EPI_ISL_908082 to
908083, EPI_ISL_908088, EPI_ISL_908091, EPI_ISL_908093, EPI_ISL_908095, EPI_ISL_908098 to 908099, EPI_ISL_908101 to 908103, EPI_ISL_908105 to 908112, EPI_ISL_908115, EPI_ISL_908117, EPI_ISL_908121, EPI_ISL_908123 to 908125, EPI_ISL_908127 to 908128, EPI_ISL_908130, EPI_ISL_908132 to 908135,
EPI_ISL_908137 to 908138, EPI_ISL_908140 to 908150, EPI_ISL_908153 to 908161, EPI_ISL_908163 to 908167, EPI_ISL_908169 to 908170, EPI_ISL_908173, EPI_ISL_908175 to 908176, EPI_ISL_908178, EPI_ISL_908180, EPI_ISL_908182, EPI_ISL_908184 to 908186, EPI_ISL_908188 to 908189, EPI_ISL_908191 to 908193,
EPI_ISL_908195 to 908197, EPI_ISL_908199, EPI_ISL_908201 to 908202, EPI_ISL_908205, EPI_ISL_908209 to 908210, EPI_ISL_908212 to 908213, EPI_ISL_908215 to 908217, EPI_ISL_908219 to 908222, EPI_ISL_908224 to 908228, EPI_ISL_908230, EPI_ISL_908232 to 908234, EPI_ISL_908236, EPI_ISL_908239 to 908240,
EPI_ISL_908244, EPI_ISL_908246 to 908248, EPI_ISL_908250 to 908251, EPI_ISL_908255, EPI_ISL_908258 to 908259, EPI_ISL_908264, EPI_ISL_908597 to 908600, EPI_ISL_908602 to 908609, EPI_ISL_908611 to 908613, EPI_ISL_908617 to 908619, EPI_ISL_908622 to 908626, EPI_ISL_908628 to 908629, EPI_ISL_908631,
EPI_ISL_908633 to 908642, EPI_ISL_908645 to 908647, EPI_ISL_908649 to 908651, EPI_ISL_908653 to 908665, EPI_ISL_908667, EPI_ISL_908669, EPI_ISL_908671, EPI_ISL_908673, EPI_ISL_908677 to 908684, EPI_ISL_908686 to 908687, EPI_ISL_908689 to 908690, EPI_ISL_908692, EPI_ISL_908694 to 908698,
EPI_ISL_908701 to 908705, EPI_ISL_908707 to 908715, EPI_ISL_908717 to 908722, EPI_ISL_908724 to 908728, EPI_ISL_908730 to 908741, EPI_ISL_908743 to 908748, EPI_ISL_908750 to 908752, EPI_ISL_908756, EPI_ISL_908758 to 908773, EPI_ISL_908775 to 908779, EPI_ISL_908785, EPI_ISL_908787 to 908790,
EPI_ISL_908795 to 908797, EPI_ISL_908799 to 908802, EPI_ISL_908804 to 908808, EPI_ISL_908810, EPI_ISL_908813 to 908817, EPI_ISL_908819 to 908825, EPI_ISL_908827 to 908828, EPI_ISL_908830 to 908831, EPI_ISL_908835 to 908838, EPI_ISL_908840 to 908845, EPI_ISL_908848 to 908851, EPI_ISL_908853 to
908856, EPI_ISL_908860 to 908863, EPI_ISL_908865 to 908869, EPI_ISL_908871 to 908872, EPI_ISL_908874 to 908876, EPI_ISL_908878, EPI_ISL_908880, EPI_ISL_908882, EPI_ISL_908884 to 908889, EPI_ISL_908891 to 908895, EPI_ISL_908897 to 908899, EPI_ISL_908901 to 908903, EPI_ISL_908905 to 908908,

EPI_ISL_908910 to 908912, EPI_ISL_908914, EPI_ISL_908916, EPI_ISL_908918 to 908925, EPI_ISL_908927 to 908928, EPI_ISL_908930 to 908931, EPI_ISL_908933, EPI_ISL_908935, EPI_ISL_908942, EPI_ISL_908946, EPI_ISL_908949, EPI_ISL_908951 to 908952, EPI_ISL_908955, EPI_ISL_908957, EPI_ISL_908959, EPI_ISL_908961, EPI_ISL_908963, EPI_ISL_908967 to 908968, EPI_ISL_908977 to 908979, EPI_ISL_908983, EPI_ISL_908985, EPI_ISL_908990 to 908991, EPI_ISL_908998 to 908999, EPI_ISL_909006 to 909007, EPI_ISL_909009, EPI_ISL_909011, EPI_ISL_909015, EPI_ISL_909019, EPI_ISL_909035 to 909036, EPI_ISL_909046 to 909050, EPI_ISL_909052, EPI_ISL_909060, EPI_ISL_909066 to 909067, EPI_ISL_909071 to 909072, EPI_ISL_909075, EPI_ISL_909079, EPI_ISL_909081, EPI_ISL_909088, EPI_ISL_909093, EPI_ISL_909095 to 909096, EPI_ISL_909102 to 909103, EPI_ISL_909105, EPI_ISL_909109, EPI_ISL_909119, EPI_ISL_909128, EPI_ISL_909144 to 909145, EPI_ISL_909148, EPI_ISL_909155, EPI_ISL_909173, EPI_ISL_909180, EPI_ISL_909182, EPI_ISL_909187, EPI_ISL_909189, EPI_ISL_909192 to 909193, EPI_ISL_909202 to 909204, EPI_ISL_909217, EPI_ISL_909220, EPI_ISL_909223, EPI_ISL_909230, EPI_ISL_909234, EPI_ISL_909238, EPI_ISL_909243, EPI_ISL_909251, EPI_ISL_909254, EPI_ISL_912214, EPI_ISL_916180, EPI_ISL_916183, EPI_ISL_916185, EPI_ISL_916187 to 916188, EPI_ISL_916190 to 916194, EPI_ISL_916196, EPI_ISL_916200 to 916202, EPI_ISL_916205, EPI_ISL_916207 to 916213, EPI_ISL_916216, EPI_ISL_916222, EPI_ISL_916225, EPI_ISL_916227 to 916229, EPI_ISL_916231 to 916234, EPI_ISL_916236 to 916238, EPI_ISL_916241 to 916242, EPI_ISL_916244 to 916249, EPI_ISL_916252, EPI_ISL_916254, EPI_ISL_916256, EPI_ISL_916258, EPI_ISL_916261 to 916264, EPI_ISL_916268 to 916270, EPI_ISL_916273 to 916281, EPI_ISL_916283 to 916285, EPI_ISL_916288, EPI_ISL_916290 to 916292, EPI_ISL_916295, EPI_ISL_916297, EPI_ISL_916299, EPI_ISL_916303 to 916305, EPI_ISL_916308, EPI_ISL_916310, EPI_ISL_916312 to 916316, EPI_ISL_916318, EPI_ISL_916320 to 916323, EPI_ISL_916325, EPI_ISL_916327 to 916329, EPI_ISL_916331 to 916333, EPI_ISL_916335 to 916336, EPI_ISL_916338, EPI_ISL_916340 to 916341, EPI_ISL_916343 to 916344, EPI_ISL_916346 to 916350, EPI_ISL_916352, EPI_ISL_916354 to 916355, EPI_ISL_916357 to 916358, EPI_ISL_916360, EPI_ISL_916362 to 916376, EPI_ISL_916378 to 916381, EPI_ISL_916383 to 916384, EPI_ISL_916388, EPI_ISL_916390 to 916392, EPI_ISL_916394, EPI_ISL_916397, EPI_ISL_916400 to 916401, EPI_ISL_916406 to 916410, EPI_ISL_916412 to 916417, EPI_ISL_916419 to 916421, EPI_ISL_916425, EPI_ISL_916427 to 916428, EPI_ISL_916431 to 916437, EPI_ISL_916441 to 916447, EPI_ISL_916450 to 916451, EPI_ISL_916453, EPI_ISL_916455, EPI_ISL_916457 to 916464, EPI_ISL_916467, EPI_ISL_916472 to 916473, EPI_ISL_916475 to 916477, EPI_ISL_916479 to 916481, EPI_ISL_916484 to 916487, EPI_ISL_916489, EPI_ISL_916491 to 916497, EPI_ISL_916499 to 916500, EPI_ISL_916502, EPI_ISL_932576, EPI_ISL_932581, EPI_ISL_932596, EPI_ISL_932598, EPI_ISL_932603, EPI_ISL_932613 to 932614, EPI_ISL_932619, EPI_ISL_932621, EPI_ISL_932646, EPI_ISL_932648, EPI_ISL_932652, EPI_ISL_932655, EPI_ISL_932666, EPI_ISL_932669, EPI_ISL_932671, EPI_ISL_932677, EPI_ISL_932680, EPI_ISL_932686 to 932687, EPI_ISL_932689, EPI_ISL_932691, EPI_ISL_932693, EPI_ISL_932703, EPI_ISL_932706, EPI_ISL_932712, EPI_ISL_932715, EPI_ISL_932718, EPI_ISL_932721 to 932722, EPI_ISL_932739, EPI_ISL_932742, EPI_ISL_932754, EPI_ISL_932770 to 932771, EPI_ISL_932776, EPI_ISL_932778, EPI_ISL_932790, EPI_ISL_932794, EPI_ISL_932805, EPI_ISL_932808, EPI_ISL_932812, EPI_ISL_932816, EPI_ISL_932820, EPI_ISL_932822, EPI_ISL_932827, EPI_ISL_932829 to 932830, EPI_ISL_932838 to 932839, EPI_ISL_932842, EPI_ISL_933114 to 933118, EPI_ISL_933120, EPI_ISL_933122, EPI_ISL_933124 to 933125, EPI_ISL_933130 to 933137, EPI_ISL_933139, EPI_ISL_933142 to 933154, EPI_ISL_933156, EPI_ISL_933160 to 933161, EPI_ISL_933163 to 933170, EPI_ISL_933172 to 933177, EPI_ISL_933180 to 933187, EPI_ISL_933189 to 933195, EPI_ISL_933197, EPI_ISL_933200 to 933208, EPI_ISL_933210, EPI_ISL_933212 to 933214, EPI_ISL_933217, EPI_ISL_933219 to 933223, EPI_ISL_933225 to 933228, EPI_ISL_933230 to 933231, EPI_ISL_933234, EPI_ISL_933237, EPI_ISL_933239 to 933241, EPI_ISL_933243 to 933246, EPI_ISL_933250, EPI_ISL_933252 to 933253, EPI_ISL_933256 to 933265, EPI_ISL_933267 to 933268, EPI_ISL_933271, EPI_ISL_933273, EPI_ISL_933276, EPI_ISL_933278 to 933280, EPI_ISL_933282 to 933283, EPI_ISL_933285 to 933294, EPI_ISL_933296 to 933303, EPI_ISL_933305 to 933308, EPI_ISL_933311 to 933315, EPI_ISL_933317, EPI_ISL_933320, EPI_ISL_933322 to 933323, EPI_ISL_933326 to 933327, EPI_ISL_933329, EPI_ISL_933331 to 933332, EPI_ISL_933334 to 933343, EPI_ISL_933346 to 933347, EPI_ISL_933349, EPI_ISL_933351 to 933352, EPI_ISL_933354 to 933358, EPI_ISL_933360 to 933364, EPI_ISL_933366 to 933376, EPI_ISL_933378 to 933393, EPI_ISL_933395, EPI_ISL_933398 to 933408, EPI_ISL_933410 to 933412, EPI_ISL_933415 to 933422, EPI_ISL_933424, EPI_ISL_938234, EPI_ISL_938236, EPI_ISL_938238 to 938240, EPI_ISL_938242 to 938256, EPI_ISL_938258 to 938260, EPI_ISL_938262 to 938269, EPI_ISL_938271, EPI_ISL_938274 to 938275, EPI_ISL_938277 to 938280, EPI_ISL_938282 to 938286, EPI_ISL_938288 to 938299, EPI_ISL_938301, EPI_ISL_938303, EPI_ISL_938305 to 938308, EPI_ISL_938310 to 938311, EPI_ISL_938313 to 938326, EPI_ISL_938328 to 938352, EPI_ISL_938354 to 938360, EPI_ISL_938362 to 938372, EPI_ISL_938374 to 938387, EPI_ISL_938389 to 938397, EPI_ISL_938399 to 938405, EPI_ISL_938407 to 938408, EPI_ISL_938410 to 938416, EPI_ISL_938418 to 938429, EPI_ISL_938431 to 938432, EPI_ISL_938434 to 938436, EPI_ISL_938439 to 938453, EPI_ISL_938456 to 938474, EPI_ISL_938476 to 938491, EPI_ISL_938493, EPI_ISL_938495, EPI_ISL_938497 to 938498, EPI_ISL_938500 to 938504, EPI_ISL_938506 to 938515, EPI_ISL_938517, EPI_ISL_938519, EPI_ISL_938521 to 938527, EPI_ISL_938529 to 938541, EPI_ISL_938543 to 938550, EPI_ISL_938552 to 938561, EPI_ISL_938563 to 938569, EPI_ISL_938571 to 938576, EPI_ISL_938578, EPI_ISL_945049, EPI_ISL_945051, EPI_ISL_945056, EPI_ISL_945061, EPI_ISL_945067, EPI_ISL_945070, EPI_ISL_945076, EPI_ISL_945083 to 945085, EPI_ISL_945095, EPI_ISL_945100, EPI_ISL_945102, EPI_ISL_945106, EPI_ISL_945114 to 945115, EPI_ISL_945125, EPI_ISL_945130 to 945131, EPI_ISL_945143, EPI_ISL_945147, EPI_ISL_945152, EPI_ISL_945155, EPI_ISL_945163, EPI_ISL_945169, EPI_ISL_945176, EPI_ISL_945180 to 945181, EPI_ISL_945186, EPI_ISL_945188, EPI_ISL_945190, EPI_ISL_945192, EPI_ISL_945197, EPI_ISL_945199, EPI_ISL_945203, EPI_ISL_945207, EPI_ISL_945212, EPI_ISL_945217, EPI_ISL_945221 to 945222, EPI_ISL_945232, EPI_ISL_945236, EPI_ISL_945238, EPI_ISL_945241, EPI_ISL_945244, EPI_ISL_945248, EPI_ISL_945250, EPI_ISL_945254, EPI_ISL_945257, EPI_ISL_945261, EPI_ISL_945271, EPI_ISL_945275, EPI_ISL_945279, EPI_ISL_945285, EPI_ISL_945288, EPI_ISL_945297, EPI_ISL_945305, EPI_ISL_945311, EPI_ISL_945319, EPI_ISL_945324, EPI_ISL_945334, EPI_ISL_945337, EPI_ISL_945341, EPI_ISL_945345 to 945347, EPI_ISL_945350, EPI_ISL_945364, EPI_ISL_945366 to 945367, EPI_ISL_948101 to 948102, EPI_ISL_948104, EPI_ISL_948107, EPI_ISL_948110, EPI_ISL_948114, EPI_ISL_948117 to 948118, EPI_ISL_948122 to 948125, EPI_ISL_948131, EPI_ISL_948133 to 948134, EPI_ISL_948136 to 948137, EPI_ISL_948139, EPI_ISL_948145, EPI_ISL_948147, EPI_ISL_948149 to 948151, EPI_ISL_948153, EPI_ISL_948155, EPI_ISL_948159 to 948160, EPI_ISL_948163 to 948166, EPI_ISL_948172, EPI_ISL_948174, EPI_ISL_948176 to 948177, EPI_ISL_948183, EPI_ISL_948185 to 948187, EPI_ISL_948189, EPI_ISL_948191 to 948193, EPI_ISL_948197 to 948199, EPI_ISL_948201, EPI_ISL_948203 to 948205, EPI_ISL_948207 to 948211, EPI_ISL_948213 to 948214, EPI_ISL_948216, EPI_ISL_948219 to 948221, EPI_ISL_948223, EPI_ISL_948227 to 948228, EPI_ISL_948230, EPI_ISL_948233 to 948234, EPI_ISL_948237, EPI_ISL_948240, EPI_ISL_948247, EPI_ISL_948249, EPI_ISL_948251, EPI_ISL_948253, EPI_ISL_948255, EPI_ISL_948257 to 948259, EPI_ISL_948264 to 948266, EPI_ISL_948269, EPI_ISL_948274, EPI_ISL_948277 to 948284, EPI_ISL_948288, EPI_ISL_948292, EPI_ISL_948294, EPI_ISL_948296, EPI_ISL_948298, EPI_ISL_948300 to 948302, EPI_ISL_948305 to 948306, EPI_ISL_948308, EPI_ISL_948310 to 948311, EPI_ISL_948313 to 948314, EPI_ISL_948321 to 948323, EPI_ISL_948328, EPI_ISL_948331 to 948332, EPI_ISL_948337 to 948338, EPI_ISL_948340 to 948342, EPI_ISL_948344 to 948346, EPI_ISL_948350, EPI_ISL_948359, EPI_ISL_948361, EPI_ISL_948364, EPI_ISL_948366, EPI_ISL_948368, EPI_ISL_948372, EPI_ISL_948374, EPI_ISL_948376, EPI_ISL_948378, EPI_ISL_948380 to 948383, EPI_ISL_948388, EPI_ISL_948391 to 948392, EPI_ISL_948395 to 948396, EPI_ISL_948402, EPI_ISL_948407, EPI_ISL_948412 to 948414, EPI_ISL_948420 to 948424, EPI_ISL_948431, EPI_ISL_948433 to 948435, EPI_ISL_948437, EPI_ISL_948441 to 948442, EPI_ISL_948445, EPI_ISL_957612, EPI_ISL_957615, EPI_ISL_957617 to 957618, EPI_ISL_957622, EPI_ISL_957625 to 957626, EPI_ISL_957634, EPI_ISL_957636, EPI_ISL_957638 to 957640, EPI_ISL_957642, EPI_ISL_957644, EPI_ISL_957646 to 957648, EPI_ISL_957661, EPI_ISL_957665, EPI_ISL_957676, EPI_ISL_957688, EPI_ISL_957696, EPI_ISL_957702 to 957704, EPI_ISL_957706, EPI_ISL_957709, EPI_ISL_957714, EPI_ISL_957716, EPI_ISL_957719, EPI_ISL_957731, EPI_ISL_957733 to 957734, EPI_ISL_957737 to 957739, EPI_ISL_957742, EPI_ISL_957746, EPI_ISL_957752, EPI_ISL_957763 to 957764, EPI_ISL_957779, EPI_ISL_957781 to 957782, EPI_ISL_957784, EPI_ISL_957788 to 957789, EPI_ISL_957798, EPI_ISL_957805, EPI_ISL_957807 to 957809, EPI_ISL_957811 to 957812, EPI_ISL_957818, EPI_ISL_957820, EPI_ISL_957825, EPI_ISL_957831, EPI_ISL_957833 to 957834, EPI_ISL_957840, EPI_ISL_957846, EPI_ISL_957852, EPI_ISL_957855, EPI_ISL_957857, EPI_ISL_957861, EPI_ISL_957869 to 957870, EPI_ISL_957873 to 957874, EPI_ISL_957876, EPI_ISL_957880 to 957883, EPI_ISL_957890 to 957891, EPI_ISL_957896, EPI_ISL_957898, EPI_ISL_957902 to 957903, EPI_ISL_957905 to 957906, EPI_ISL_957908, EPI_ISL_959267, EPI_ISL_971425, EPI_ISL_980235 to 980240, EPI_ISL_980242 to 980243, EPI_ISL_980245 to 980246, EPI_ISL_980249, EPI_ISL_980251, EPI_ISL_980253 to 980257, EPI_ISL_980259 to 980262, EPI_ISL_980264 to 980267, EPI_ISL_980269 to 980277, EPI_ISL_980280 to 980293, EPI_ISL_980297 to 980303, EPI_ISL_980306 to 980318, EPI_ISL_980320 to 980324, EPI_ISL_980326 to 980332, EPI_ISL_980334, EPI_ISL_980336 to 980337, EPI_ISL_980339, EPI_ISL_980342, EPI_ISL_980344 to 980361, EPI_ISL_980363 to 980369, EPI_ISL_980371 to 980373, EPI_ISL_980376, EPI_ISL_980379, EPI_ISL_980382, EPI_ISL_980385 to 980389, EPI_ISL_980391 to 980396, EPI_ISL_980399 to 980405, EPI_ISL_980408, EPI_ISL_980411, EPI_ISL_980413 to 980426, EPI_ISL_980429, EPI_ISL_980431 to 980432, EPI_ISL_980434, EPI_ISL_980436 to 980439, EPI_ISL_980441, EPI_ISL_980443 to 980458, EPI_ISL_980460 to 980462, EPI_ISL_980466 to 980468, EPI_ISL_980470 to 980472, EPI_ISL_980474, EPI_ISL_980477, EPI_ISL_980479 to 980481, EPI_ISL_980483 to 980496, EPI_ISL_980498 to 980499, EPI_ISL_980501 to 980511, EPI_ISL_980513, EPI_ISL_980515 to 980517, EPI_ISL_980522 to 980524, EPI_ISL_980527 to 980534, EPI_ISL_980537 to 980546, EPI_ISL_980548, EPI_ISL_985411 to 985421, EPI_ISL_985423 to 985426, EPI_ISL_985428 to 985432, EPI_ISL_985434 to 985444, EPI_ISL_985446 to 985447, EPI_ISL_985449 to 985458, EPI_ISL_985460 to 985465, EPI_ISL_985467 to 985483, EPI_ISL_985485 to 985491, EPI_ISL_985493 to 985494, EPI_ISL_985496 to 985514, EPI_ISL_985516 to 985543, EPI_ISL_985545, EPI_ISL_985547 to 985551, EPI_ISL_985554 to 985567, EPI_ISL_985569 to 985572, EPI_ISL_985574 to 985576, EPI_ISL_985579, EPI_ISL_985581 to 985611, EPI_ISL_986440, EPI_ISL_986442 to 986453, EPI_ISL_986455 to 986502, EPI_ISL_986504 to 986517, EPI_ISL_986519 to 986527, EPI_ISL_986529 to 986534, EPI_ISL_986536 to 986543, EPI_ISL_986545 to 986559, EPI_ISL_986561 to 986566, EPI_ISL_986568 to 986583, EPI_ISL_986585 to 986593, EPI_ISL_986595, EPI_ISL_986597 to 986606, EPI_ISL_986608 to 986609, EPI_ISL_986611 to 986616, EPI_ISL_986618 to 986631, EPI_ISL_986633 to 986639, EPI_ISL_986641 to 986650, EPI_ISL_986652 to 986657, EPI_ISL_986659 to 986691, EPI_ISL_986693 to 986697, EPI_ISL_986699 to 986702, EPI_ISL_986704 to 986708, EPI_ISL_986710 to 986718, EPI_ISL_986720 to 986725, EPI_ISL_986727 to 986728, EPI_ISL_986730 to 986732, EPI_ISL_986734 to 986763, EPI_ISL_986765 to 986795, EPI_ISL_986797 to 986809, EPI_ISL_986811 to 986812, EPI_ISL_986814 to 986823, EPI_ISL_987161 to 987171, EPI_ISL_987173 to 987176, EPI_ISL_987180 to 987200, EPI_ISL_987202 to 987233, EPI_ISL_987235 to 987242, EPI_ISL_987244 to 987261, EPI_ISL_987263 to 987273, EPI_ISL_987275 to 987281, EPI_ISL_987283, EPI_ISL_987287 to 987293, EPI_ISL_987295, EPI_ISL_987299, EPI_ISL_987302 to 987304, EPI_ISL_987306 to 987307, EPI_ISL_987309 to 987310, EPI_ISL_987312 to 987313, EPI_ISL_987315, EPI_ISL_987318 to 987319, EPI_ISL_987321 to 987322, EPI_ISL_987325 to 987326, EPI_ISL_987328, EPI_ISL_987332, EPI_ISL_987339 to 987340, EPI_ISL_987344, EPI_ISL_987348, EPI_ISL_987357, EPI_ISL_987361, EPI_ISL_987365, EPI_ISL_987367 to 987369, EPI_ISL_987372, EPI_ISL_987378 to 987384, EPI_ISL_987387, EPI_ISL_987389, EPI_ISL_987393, EPI_ISL_987397 to 987399, EPI_ISL_987401 to 987402, EPI_ISL_987406, EPI_ISL_987409, EPI_ISL_987411 to 987414, EPI_ISL_987416 to 987418, EPI_ISL_987421, EPI_ISL_987424 to 987430, EPI_ISL_987432, EPI_ISL_987435, EPI_ISL_987443, EPI_ISL_987446, EPI_ISL_987449, EPI_ISL_987451, EPI_ISL_987453, EPI_ISL_987456, EPI_ISL_987458, EPI_ISL_987462, EPI_ISL_987464 to 987465, EPI_ISL_987467, EPI_ISL_987472, EPI_ISL_987474, EPI_ISL_987476, EPI_ISL_987478 to 987480, EPI_ISL_987482, EPI_ISL_987484, EPI_ISL_987488 to 987490, EPI_ISL_987493, EPI_ISL_987498, EPI_ISL_987505 to 987506, EPI_ISL_987508 to 987510, EPI_ISL_987513 to 987514, EPI_ISL_987518 to 987520, EPI_ISL_987523, EPI_ISL_987525 to 987527, EPI_ISL_987530, EPI_ISL_987540 to 987542, EPI_ISL_987545, EPI_ISL_987547, EPI_ISL_987553 to 987554, EPI_ISL_987557, EPI_ISL_987559 to 987561, EPI_ISL_987566 to 987567, EPI_ISL_987573, EPI_ISL_987575, EPI_ISL_987577 to 987579, EPI_ISL_987581, EPI_ISL_987583, EPI_ISL_987587, EPI_ISL_987591, EPI_ISL_987594 to 987596, EPI_ISL_987598 to 987599, EPI_ISL_987602 to 987607, EPI_ISL_987609 to 987613, EPI_ISL_987615, EPI_ISL_987617, EPI_ISL_987622, EPI_ISL_987624 to 987625, EPI_ISL_987633, EPI_ISL_987637 to 987641, EPI_ISL_987643, EPI_ISL_987645 to 987665, EPI_ISL_987667 to 987676, EPI_ISL_987678 to 987680, EPI_ISL_987682 to 987685, EPI_ISL_987687 to 987708, EPI_ISL_987710 to 987711, EPI_ISL_987713 to 987728, EPI_ISL_987730, EPI_ISL_987732 to 987753, EPI_ISL_987756 to 987762, EPI_ISL_987764 to 987771, EPI_ISL_987774, EPI_ISL_987776, EPI_ISL_987779 to 987780, EPI_ISL_987782 to 987785, EPI_ISL_987787 to 987802, EPI_ISL_987804 to 987819, EPI_ISL_987821 to 987822, EPI_ISL_987824 to 987844, EPI_ISL_987848 to 987856, EPI_ISL_987858 to 987860, EPI_ISL_987862 to 987865, EPI_ISL_987868 to 987870, EPI_ISL_987872 to 987873, EPI_ISL_987875, EPI_ISL_987877 to 987894, EPI_ISL_987896, EPI_ISL_987898 to 987909, EPI_ISL_987911 to 987921, EPI_ISL_987923 to 987970, EPI_ISL_987972 to 987973, EPI_ISL_987975 to 987998, EPI_ISL_988000 to 988003, EPI_ISL_988005 to 988011, EPI_ISL_988013 to 988015, EPI_ISL_988017 to 988058, EPI_ISL_988061 to 988065, EPI_ISL_988067 to 988082, EPI_ISL_988084 to 988124, EPI_ISL_988126 to 988147, EPI_ISL_988149 to 988155, EPI_ISL_988157 to 988159, EPI_ISL_988161 to 988163, EPI_ISL_988165 to 988171, EPI_ISL_988173 to 988175, EPI_ISL_988177 to 988186, EPI_ISL_988188 to 988194, EPI_ISL_988196 to 988198, EPI_ISL_988200 to 988204, EPI_ISL_988208, EPI_ISL_988211 to 988212, EPI_ISL_988214 to 988216, EPI_ISL_988219 to 988220, EPI_ISL_988226 to 988228, EPI_ISL_988230 to 988233, EPI_ISL_988244 to 988247, EPI_ISL_988251 to 988253, EPI_ISL_988255, EPI_ISL_988257 to 988260, EPI_ISL_988264 to 988265, EPI_ISL_988267 to 988269, EPI_ISL_988272 to 988275, EPI_ISL_988277 to 988285, EPI_ISL_988287 to 988289, EPI_ISL_988291 to 988295, EPI_ISL_988297, EPI_ISL_988301, EPI_ISL_988303, EPI_ISL_988311, EPI_ISL_988316, EPI_ISL_988318 to 988320, EPI_ISL_988322 to 988324, EPI_ISL_988326 to 988328, EPI_ISL_988330 to 988331, EPI_ISL_988333 to 988334, EPI_ISL_988336, EPI_ISL_988338 to 988343, EPI_ISL_988345, EPI_ISL_988348 to 988350, EPI_ISL_988353, EPI_ISL_988356, EPI_ISL_988360 to 988361, EPI_ISL_988365, EPI_ISL_988367 to 988369, EPI_ISL_988371 to 988376, EPI_ISL_988379, EPI_ISL_988381 to 988382, EPI_ISL_988386 to 988388, EPI_ISL_988390, EPI_ISL_988392 to 988394, EPI_ISL_988396 to 988397, EPI_ISL_988400 to 988402, EPI_ISL_988405 to 988406, EPI_ISL_988408, EPI_ISL_988410 to 988413, EPI_ISL_988415 to 988416, EPI_ISL_988419 to 988422, EPI_ISL_988424 to 988425, EPI_ISL_988429, EPI_ISL_988431 to 988433, EPI_ISL_988435, EPI_ISL_988438 to 988440, EPI_ISL_988444 to 988445, EPI_ISL_988447, EPI_ISL_988450, EPI_ISL_988454 to 988455, EPI_ISL_988458, EPI_ISL_988461 to 988462, EPI_ISL_988464, EPI_ISL_988466 to 988467, EPI_ISL_988469 to 988479, EPI_ISL_988481, EPI_ISL_989363 to 989373, EPI_ISL_989375 to 989387, EPI_ISL_989389 to 989400, EPI_ISL_989402, EPI_ISL_989404 to 989408, EPI_ISL_989410 to 989427, EPI_ISL_989429 to 989435, EPI_ISL_989437 to 989443, EPI_ISL_993415, EPI_ISL_993418, EPI_ISL_993420, EPI_ISL_993424, EPI_ISL_993430 to 993431, EPI_ISL_993435, EPI_ISL_993437, EPI_ISL_993440, EPI_ISL_993448 to 993449, EPI_ISL_993455, EPI_ISL_993457, EPI_ISL_993477, EPI_ISL_993487, EPI_ISL_993496, EPI_ISL_993504, EPI_ISL_993519, EPI_ISL_993522 to 993523, EPI_ISL_993532, EPI_ISL_993538, EPI_ISL_993542 to 993543, EPI_ISL_993546, EPI_ISL_993549 to 993550, EPI_ISL_993562, EPI_ISL_993571, EPI_ISL_993584, EPI_ISL_993589, EPI_ISL_993593, EPI_ISL_993595, EPI_ISL_993598 to 993599, EPI_ISL_993616, EPI_ISL_993619 to 993621, EPI_ISL_993639 to 993641, EPI_ISL_993656, EPI_ISL_993661, EPI_ISL_993683, EPI_ISL_993690, EPI_ISL_993697, EPI_ISL_993704, EPI_ISL_993706, EPI_ISL_993718, EPI_ISL_993720, EPI_ISL_993722, EPI_ISL_993736, EPI_ISL_993738 to 993741, EPI_ISL_993743 to 993744, EPI_ISL_993746, EPI_ISL_993750, EPI_ISL_993754, EPI_ISL_993756 to 993757, EPI_ISL_993761 to 993762, EPI_ISL_993766, EPI_ISL_993770 to 993771, EPI_ISL_993774, EPI_ISL_993776, EPI_ISL_993778 to 993779, EPI_ISL_993791 to 993793, EPI_ISL_993797 to 993798, EPI_ISL_993803 to 993804, EPI_ISL_993807, EPI_ISL_993809, EPI_ISL_993811, EPI_ISL_993813 to 993814, EPI_ISL_993816 to 993817, EPI_ISL_993819, EPI_ISL_993823 to 993824, EPI_ISL_993827, EPI_ISL_993830, EPI_ISL_993837 to 993838, EPI_ISL_993847, EPI_ISL_993849 to 993850, EPI_ISL_993853 to 993856, EPI_ISL_993859, EPI_ISL_993863 to 993869, EPI_ISL_993871 to 993872, EPI_ISL_993878, EPI_ISL_993881, EPI_ISL_993893 to 993895, EPI_ISL_993900, EPI_ISL_993903, EPI_ISL_993907, EPI_ISL_993909, EPI_ISL_993911 to 993912, EPI_ISL_993915, EPI_ISL_993920 to 993922, EPI_ISL_993926, EPI_ISL_993942, EPI_ISL_993945, EPI_ISL_993949, EPI_ISL_993954, EPI_ISL_993957, EPI_ISL_993960 to 993961, EPI_ISL_993963, EPI_ISL_993966, EPI_ISL_993968, EPI_ISL_993972 to 993973, EPI_ISL_993975 to 993976, EPI_ISL_993979, EPI_ISL_993982, EPI_ISL_993987, EPI_ISL_993992, EPI_ISL_993994, EPI_ISL_993996 to 993997, EPI_ISL_994000 to 994001, EPI_ISL_994003, EPI_ISL_994006 to 994008, EPI_ISL_994010 to 994011, EPI_ISL_994015, EPI_ISL_994018 to 994020, EPI_ISL_994022, EPI_ISL_994024 to 994025, EPI_ISL_994032 to 994033, EPI_ISL_994040, EPI_ISL_994045 to 994046, EPI_ISL_994048, EPI_ISL_994053, EPI_ISL_994058, EPI_ISL_994061, EPI_ISL_994064, EPI_ISL_994066, EPI_ISL_994290, EPI_ISL_994295 to 994296, EPI_ISL_994299 to 994300, EPI_ISL_994341, EPI_ISL_994349 to 994350, EPI_ISL_994359, EPI_ISL_994363, EPI_ISL_994373 to 994374, EPI_ISL_994383, EPI_ISL_994390, EPI_ISL_994397, EPI_ISL_994401, EPI_ISL_994403, EPI_ISL_994408 to 994410, EPI_ISL_994420, EPI_ISL_994427, EPI_ISL_994429, EPI_ISL_994431 to 994432, EPI_ISL_994434 to 994436, EPI_ISL_994440, EPI_ISL_994452, EPI_ISL_994457 to 994459, EPI_ISL_994477, EPI_ISL_994480, EPI_ISL_994497, EPI_ISL_994501, EPI_ISL_994504, EPI_ISL_994508, EPI_ISL_994516, EPI_ISL_994521, EPI_ISL_994524, EPI_ISL_994536, EPI_ISL_994544, EPI_ISL_994610, EPI_ISL_1005700 to 1005717, EPI_ISL_1005719 to 1005726, EPI_ISL_1005728 to 1005730, EPI_ISL_1005732 to 1005740, EPI_ISL_1005744 to 1005745, EPI_ISL_1005749 to 1005752, EPI_ISL_1005754 to 1005759, EPI_ISL_1005762 to 1005764, EPI_ISL_1005767, EPI_ISL_1005769 to 1005770, EPI_ISL_1005772, EPI_ISL_1005775, EPI_ISL_1005777, EPI_ISL_1005779 to 1005781, EPI_ISL_1005783 to 1005784, EPI_ISL_1005786 to 1005798, EPI_ISL_1005800 to 1005803, EPI_ISL_1005805 to 1005806, EPI_ISL_1005808, EPI_ISL_1005810 to 1005813, EPI_ISL_1005815, EPI_ISL_1005818 to 1005823, EPI_ISL_1005825 to 1005826, EPI_ISL_1005828 to 1005830, EPI_ISL_1005832 to 1005835, EPI_ISL_1005837, EPI_ISL_1005839, EPI_ISL_1005842, EPI_ISL_1005844 to 1005856, EPI_ISL_1005858 to 1005861, EPI_ISL_1005863 to 1005867, EPI_ISL_1005869 to 1005872, EPI_ISL_1005875 to 1005882, EPI_ISL_1005884, EPI_ISL_1005886 to 1005888, EPI_ISL_1005890 to 1005891, EPI_ISL_1005893 to 1005894, EPI_ISL_1005897 to 1005899, EPI_ISL_1005904 to 1005907, EPI_ISL_1005910, EPI_ISL_1005913, EPI_ISL_1005915 to 1005916, EPI_ISL_1005918 to 1005921, EPI_ISL_1005926, EPI_ISL_1005928 to 1005932, EPI_ISL_1005936 to 1005938, EPI_ISL_1005941 to 1005950, EPI_ISL_1005953 to 1005956, EPI_ISL_1005959 to 1005960, EPI_ISL_1006186 to 1006188, EPI_ISL_1006190 to 1006201, EPI_ISL_1006203 to 1006213, EPI_ISL_1006215, EPI_ISL_1006217 to 1006225, EPI_ISL_1006227 to 1006231, EPI_ISL_1006233 to 1006249, EPI_ISL_1006251 to 1006253, EPI_ISL_1006256 to 1006259, EPI_ISL_1006261 to 1006262, EPI_ISL_1006264 to 1006268, EPI_ISL_1006270 to 1006271, EPI_ISL_1006273, EPI_ISL_1006276 to 1006277, EPI_ISL_1006279 to 1006280, EPI_ISL_1006282 to 1006284, EPI_ISL_1006286 to 1006288, EPI_ISL_1006291 to 1006296, EPI_ISL_1006299, EPI_ISL_1006301, EPI_ISL_1006303, EPI_ISL_1006305, EPI_ISL_1006307 to 1006309, EPI_ISL_1006312 to 1006314, EPI_ISL_1006316 to 1006317, EPI_ISL_1006319 to 1006326, EPI_ISL_1006328 to 1006336, EPI_ISL_1006338, EPI_ISL_1006340, EPI_ISL_1006548 to 1006549, EPI_ISL_1006552, EPI_ISL_1006554, EPI_ISL_1006561 to 1006562, EPI_ISL_1006564, EPI_ISL_1006567, EPI_ISL_1006569, EPI_ISL_1006577, EPI_ISL_1006581, EPI_ISL_1006585, EPI_ISL_1006587 to 1006588, EPI_ISL_1006592, EPI_ISL_1006596 to 1006598, EPI_ISL_1006603, EPI_ISL_1006608, EPI_ISL_1006610 to 1006612, EPI_ISL_1006618 to 1006619, EPI_ISL_1006625, EPI_ISL_1006628, EPI_ISL_1006631, EPI_ISL_1006633, EPI_ISL_1006635, EPI_ISL_1006638, EPI_ISL_1006640 to 1006641, EPI_ISL_1006643, EPI_ISL_1006652 to 1006655, EPI_ISL_1006865, EPI_ISL_1006867 to 1006870, EPI_ISL_1006872, EPI_ISL_1006875 to 1006879, EPI_ISL_1006882 to 1006886, EPI_ISL_1006888 to 1006889, EPI_ISL_1006892 to 1006893, EPI_ISL_1006895 to 1006897, EPI_ISL_1006902, EPI_ISL_1006904 to 1006905, EPI_ISL_1006907 to 1006908, EPI_ISL_1006910, EPI_ISL_1006914 to 1006917, EPI_ISL_1006921 to 1006927, EPI_ISL_1006929 to 1006931, EPI_ISL_1006933 to 1006934, EPI_ISL_1006936, EPI_ISL_1007181 to 1007184, EPI_ISL_1007186 to 1007187, EPI_ISL_1007189 to 1007191, EPI_ISL_1007194 to 1007202, EPI_ISL_1007206 to 1007208, EPI_ISL_1007210, EPI_ISL_1007212, EPI_ISL_1007217 to 1007220, EPI_ISL_1007222 to 1007223, EPI_ISL_1007225 to 1007230, EPI_ISL_1007234, EPI_ISL_1007236, EPI_ISL_1007238 to 1007244, EPI_ISL_1007247, EPI_ISL_1007249 to 1007254, EPI_ISL_1007256 to 1007260, EPI_ISL_1007262 to 1007264, EPI_ISL_1007266 to 1007275, EPI_ISL_1007278 to 1007312, EPI_ISL_1007314 to 1007319, EPI_ISL_1007321 to 1007329, EPI_ISL_1007331 to 1007348, EPI_ISL_1007350 to 1007379, EPI_ISL_1007381, EPI_ISL_1007383 to 1007413, EPI_ISL_1007415 to 1007416, EPI_ISL_1007418 to 1007433, EPI_ISL_1007435, EPI_ISL_1007445 to 1007446, EPI_ISL_1007448 to 1007453, EPI_ISL_1007455 to 1007462, EPI_ISL_1007464, EPI_ISL_1007466, EPI_ISL_1007469 to 1007470, EPI_ISL_1007472 to 1007475, EPI_ISL_1007477 to 1007482, EPI_ISL_1007484, EPI_ISL_1007486 to 1007487, EPI_ISL_1007489 to 1007491, EPI_ISL_1007493, EPI_ISL_1007495, EPI_ISL_1007497, EPI_ISL_1007500 to 1007506, EPI_ISL_1007509 to 1007516, EPI_ISL_1007518 to 1007522, EPI_ISL_1007525, EPI_ISL_1007529, EPI_ISL_1007531, EPI_ISL_1007534 to 1007539, EPI_ISL_1007541, EPI_ISL_1007544 to 1007547, EPI_ISL_1007583, EPI_ISL_1012099, EPI_ISL_1012101 to 1012104, EPI_ISL_1012106 to 1012111, EPI_ISL_1012113 to 1012116, EPI_ISL_1012118 to 1012119, EPI_ISL_1012121 to 1012143, EPI_ISL_1012146 to 1012179, EPI_ISL_1012181 to 1012187, EPI_ISL_1012189 to 1012204, EPI_ISL_1012208 to 1012218, EPI_ISL_1012220 to 1012230, EPI_ISL_1012232 to 1012245, EPI_ISL_1012247 to 1012251, EPI_ISL_1012253 to 1012262, EPI_ISL_1012264 to 1012270, EPI_ISL_1012272 to 1012306, EPI_ISL_1012308 to 1012320, EPI_ISL_1012322 to 1012327, EPI_ISL_1012329, EPI_ISL_1012331 to 1012365, EPI_ISL_1012367 to 1012388, EPI_ISL_1012390 to 1012402, EPI_ISL_1012407, EPI_ISL_1012409, EPI_ISL_1012414, EPI_ISL_1012416, EPI_ISL_1012428, EPI_ISL_1012431, EPI_ISL_1012438, EPI_ISL_1012458, EPI_ISL_1012482, EPI_ISL_1012493, EPI_ISL_1012521, EPI_ISL_1012531, EPI_ISL_1012541, EPI_ISL_1012549, EPI_ISL_1012610 to 1012611, EPI_ISL_1012613 to 1012619, EPI_ISL_1012628 to 1012629, EPI_ISL_1012631 to 1012634,

EPI_ISL_1012638, EPI_ISL_1012640 to 1012646, EPI_ISL_1012649 to 1012650, EPI_ISL_1012653, EPI_ISL_1012655 to 1012657, EPI_ISL_1012659, EPI_ISL_1012661, EPI_ISL_1012663 to 1012665, EPI_ISL_1018689 to 1018697, EPI_ISL_1018699 to 1018702, EPI_ISL_1018704 to 1018707, EPI_ISL_1018709 to 1018742, EPI_ISL_1018744 to 1018757, EPI_ISL_1018759 to 1018774, EPI_ISL_1018776 to 1018788, EPI_ISL_1018791 to 1018847, EPI_ISL_1018850 to 1018851, EPI_ISL_1018853 to 1018858, EPI_ISL_1018861, EPI_ISL_1018863 to 1018872, EPI_ISL_1018875 to 1018883, EPI_ISL_1018885 to 1018897, EPI_ISL_1018900 to 1018902, EPI_ISL_1018904 to 1018905, EPI_ISL_1018907 to 1018925, EPI_ISL_1018927, EPI_ISL_1018929 to 1018935, EPI_ISL_1018937 to 1018940, EPI_ISL_1018942 to 1018945, EPI_ISL_1018947 to 1018949, EPI_ISL_1019286 to 1019288, EPI_ISL_1019290 to 1019299, EPI_ISL_1019301 to 1019305, EPI_ISL_1019307 to 1019308, EPI_ISL_1019310 to 1019332, EPI_ISL_1019334 to 1019336, EPI_ISL_1019338 to 1019356, EPI_ISL_1019359 to 1019362, EPI_ISL_1019364 to 1019380, EPI_ISL_1019382 to 1019400, EPI_ISL_1019402 to 1019404, EPI_ISL_1019406 to 1019408, EPI_ISL_1019504 to 1019506, EPI_ISL_1033163 to 1033175, EPI_ISL_1033177 to 1033194, EPI_ISL_1033196 to 1033200, EPI_ISL_1033202 to 1033211, EPI_ISL_1033213 to 1033214, EPI_ISL_1033216 to 1033228, EPI_ISL_1033230 to 1033248, EPI_ISL_1033250, EPI_ISL_1033253 to 1033259, EPI_ISL_1033261 to 1033283, EPI_ISL_1033286, EPI_ISL_1033290 to 1033334, EPI_ISL_1033336 to 1033352, EPI_ISL_1033354 to 1033418, EPI_ISL_1033420 to 1033430, EPI_ISL_1033433 to 1033491, EPI_ISL_1033493 to 1033511, EPI_ISL_1033513 to 1033535, EPI_ISL_1033610, EPI_ISL_1033616, EPI_ISL_1033644, EPI_ISL_1033669 to 1033670, EPI_ISL_1033699, EPI_ISL_1033755, EPI_ISL_1033767, EPI_ISL_1033772 to 1033773, EPI_ISL_1033791, EPI_ISL_1033793, EPI_ISL_1033797, EPI_ISL_1033802, EPI_ISL_1033806, EPI_ISL_1033836, EPI_ISL_1033845, EPI_ISL_1033850, EPI_ISL_1033853, EPI_ISL_1033855, EPI_ISL_1033868, EPI_ISL_1033884, EPI_ISL_1033889, EPI_ISL_1033893, EPI_ISL_1033898, EPI_ISL_1033904, EPI_ISL_1033909

see above	Lighthouse Lab in Cambridge	Wellcome Sanger Institute for the COVID-19 Genomics UK (COG-UK) Consortium	Cordelia Langford; David K. Jackson; Dominic Kwiatkowski; Ewan Harrison; Ian Johnston; Jeffrey Barrett; John Sillitoe on behalf of the Wellcome Sanger Institute COVID-19 Surveillance Team; John Sillitoe on behalf of the Wellcome Sanger Institute COVID-19 Surveillance Team (http://www.sanger.ac.uk/covid-team); Rob Howes; Roberto Amato; Sonia Goncalves; The Lighthouse Lab in Cambridge and Alex Alderton

EPI_ISL_551835 to 551837, EPI_ISL_551839 to 551848, EPI_ISL_551851 to 551873, EPI_ISL_551875 to 551876, EPI_ISL_551878 to 551886, EPI_ISL_551889 to 551895, EPI_ISL_551898 to 551900, EPI_ISL_551902 to 551919, EPI_ISL_551925, EPI_ISL_551932, EPI_ISL_551951, EPI_ISL_551953, EPI_ISL_551967, EPI_ISL_551980, EPI_ISL_551983, EPI_ISL_551990, EPI_ISL_552001, EPI_ISL_552005, EPI_ISL_552008 to 552010, EPI_ISL_552017, EPI_ISL_552051, EPI_ISL_552065, EPI_ISL_552073, EPI_ISL_552091, EPI_ISL_552094, EPI_ISL_552098, EPI_ISL_552151, EPI_ISL_552166, EPI_ISL_552170, EPI_ISL_552180, EPI_ISL_552185, EPI_ISL_555864, EPI_ISL_555868, EPI_ISL_555875, EPI_ISL_555910, EPI_ISL_555939, EPI_ISL_555945, EPI_ISL_555948, EPI_ISL_555955, EPI_ISL_555973, EPI_ISL_555976, EPI_ISL_556012, EPI_ISL_556021 to 556022, EPI_ISL_556026, EPI_ISL_556039, EPI_ISL_556047, EPI_ISL_556058, EPI_ISL_556063, EPI_ISL_556309, EPI_ISL_556318, EPI_ISL_556321, EPI_ISL_556338, EPI_ISL_556344, EPI_ISL_556351, EPI_ISL_556357, EPI_ISL_556425, EPI_ISL_556434, EPI_ISL_556446, EPI_ISL_556459, EPI_ISL_556471, EPI_ISL_556482, EPI_ISL_556488, EPI_ISL_556494, EPI_ISL_556508 to 556509, EPI_ISL_556518, EPI_ISL_556520 to 556524, EPI_ISL_556526, EPI_ISL_556528 to 556535, EPI_ISL_556537 to 556538, EPI_ISL_556542 to 556543, EPI_ISL_556546, EPI_ISL_556548 to 556549, EPI_ISL_556551 to 556554, EPI_ISL_556560, EPI_ISL_556562 to 556569, EPI_ISL_556571 to 556572, EPI_ISL_556575, EPI_ISL_556639 to 556643, EPI_ISL_556646, EPI_ISL_556648 to 556651, EPI_ISL_556654 to 556663, EPI_ISL_556665 to 556679, EPI_ISL_556681, EPI_ISL_556683 to 556696, EPI_ISL_556699, EPI_ISL_556701 to 556702, EPI_ISL_556704 to 556718, EPI_ISL_556720, EPI_ISL_556722 to 556724, EPI_ISL_556726 to 556745, EPI_ISL_556747 to 556750, EPI_ISL_556752 to 556758, EPI_ISL_556760, EPI_ISL_556762 to 556769, EPI_ISL_556771 to 556786, EPI_ISL_556788 to 556790, EPI_ISL_556792, EPI_ISL_556794 to 556799, EPI_ISL_556801 to 556802, EPI_ISL_556804 to 556816, EPI_ISL_556818 to 556821, EPI_ISL_556823, EPI_ISL_556825, EPI_ISL_556827, EPI_ISL_556830 to 556832, EPI_ISL_556834 to 556835, EPI_ISL_556837 to 556851, EPI_ISL_556853 to 556856, EPI_ISL_556858 to 556862, EPI_ISL_556864 to 556865, EPI_ISL_556867 to 556872, EPI_ISL_556874 to 556880, EPI_ISL_556882, EPI_ISL_556885 to 556886, EPI_ISL_556888 to 556893, EPI_ISL_556895 to 556896, EPI_ISL_556899 to 556902, EPI_ISL_556905 to 556907, EPI_ISL_556909 to 556913, EPI_ISL_556915 to 556917, EPI_ISL_556919 to 556924, EPI_ISL_556926, EPI_ISL_556928, EPI_ISL_556930 to 556939, EPI_ISL_556941 to 556943, EPI_ISL_556945 to 556946, EPI_ISL_556948 to 556952, EPI_ISL_556955, EPI_ISL_556957 to 556961, EPI_ISL_556964, EPI_ISL_556967 to 556971, EPI_ISL_556973 to 556976, EPI_ISL_556979, EPI_ISL_556981 to 556983, EPI_ISL_566423, EPI_ISL_566429, EPI_ISL_566444 to 566445, EPI_ISL_566453, EPI_ISL_566457, EPI_ISL_566467, EPI_ISL_566493, EPI_ISL_566495, EPI_ISL_566500, EPI_ISL_566508, EPI_ISL_566518 to 566519, EPI_ISL_566524, EPI_ISL_566528, EPI_ISL_566534, EPI_ISL_566543 to 566544, EPI_ISL_566567, EPI_ISL_566576, EPI_ISL_566586, EPI_ISL_566589, EPI_ISL_566610 to 566611, EPI_ISL_566625, EPI_ISL_566638, EPI_ISL_566659, EPI_ISL_566661 to 566662, EPI_ISL_566666, EPI_ISL_566674, EPI_ISL_568385, EPI_ISL_568436, EPI_ISL_568453 to 568454, EPI_ISL_568458, EPI_ISL_568461, EPI_ISL_568473, EPI_ISL_580282 to 580283, EPI_ISL_580286 to 580287, EPI_ISL_580299, EPI_ISL_580301, EPI_ISL_580305, EPI_ISL_580313, EPI_ISL_580322, EPI_ISL_580325, EPI_ISL_580327, EPI_ISL_580338, EPI_ISL_580340 to 580341, EPI_ISL_580353 to 580354, EPI_ISL_580362, EPI_ISL_580364, EPI_ISL_580367 to 580369, EPI_ISL_580371, EPI_ISL_580373, EPI_ISL_580385, EPI_ISL_580391, EPI_ISL_580393 to 580394, EPI_ISL_580396, EPI_ISL_580400, EPI_ISL_580414, EPI_ISL_580418, EPI_ISL_580421, EPI_ISL_580435, EPI_ISL_580444, EPI_ISL_580449, EPI_ISL_580453, EPI_ISL_580459, EPI_ISL_580465, EPI_ISL_580467, EPI_ISL_580471, EPI_ISL_580478 to 580481, EPI_ISL_580485 to 580486, EPI_ISL_580495, EPI_ISL_580499, EPI_ISL_580502, EPI_ISL_580508, EPI_ISL_580511, EPI_ISL_580532 to 580533, EPI_ISL_580536, EPI_ISL_580557, EPI_ISL_580559, EPI_ISL_580564 to 580565, EPI_ISL_580569, EPI_ISL_580573, EPI_ISL_580576 to 580577, EPI_ISL_580579, EPI_ISL_580582, EPI_ISL_580586 to 580587, EPI_ISL_580593, EPI_ISL_580603, EPI_ISL_580607, EPI_ISL_580610, EPI_ISL_580618, EPI_ISL_580621, EPI_ISL_580625 to 580626, EPI_ISL_581394, EPI_ISL_581396, EPI_ISL_590173, EPI_ISL_596928 to 596936, EPI_ISL_596938, EPI_ISL_596940 to 596942, EPI_ISL_596944 to 596947, EPI_ISL_596951 to 596968, EPI_ISL_596970 to 596972, EPI_ISL_596974 to 596975, EPI_ISL_596977 to 596981, EPI_ISL_596983, EPI_ISL_596985 to 596995, EPI_ISL_596997 to 597006, EPI_ISL_597008 to 597016, EPI_ISL_597018 to 597025, EPI_ISL_597027 to 597028, EPI_ISL_597030 to 597037, EPI_ISL_597039 to 597043, EPI_ISL_597045 to 597048, EPI_ISL_597050 to 597054, EPI_ISL_597057 to 597064, EPI_ISL_597066, EPI_ISL_597068 to 597073, EPI_ISL_597076 to 597085, EPI_ISL_597087 to 597098, EPI_ISL_597100 to 597103, EPI_ISL_597105 to 597108, EPI_ISL_597110 to 597114, EPI_ISL_597116 to 597122, EPI_ISL_597124, EPI_ISL_597126 to 597133, EPI_ISL_597135 to 597139, EPI_ISL_597141, EPI_ISL_597143 to 597144, EPI_ISL_597146 to 597157, EPI_ISL_597160, EPI_ISL_597162 to 597188, EPI_ISL_597190, EPI_ISL_597192 to 597193, EPI_ISL_597195 to 597197, EPI_ISL_597199 to 597204, EPI_ISL_597206 to 597222, EPI_ISL_597224 to 597230, EPI_ISL_597232 to 597233, EPI_ISL_597237 to 597239, EPI_ISL_597243, EPI_ISL_597246 to 597248, EPI_ISL_597250, EPI_ISL_597253 to 597259, EPI_ISL_597262, EPI_ISL_597264, EPI_ISL_597266, EPI_ISL_597270, EPI_ISL_597273 to 597274, EPI_ISL_597279, EPI_ISL_597282 to 597287, EPI_ISL_597289, EPI_ISL_597293, EPI_ISL_597295, EPI_ISL_597297, EPI_ISL_597300, EPI_ISL_597304 to 597310, EPI_ISL_597316, EPI_ISL_597319 to 597321, EPI_ISL_597323 to 597325, EPI_ISL_597327, EPI_ISL_597331, EPI_ISL_597335, EPI_ISL_597337, EPI_ISL_597340, EPI_ISL_597342 to 597343, EPI_ISL_597345 to 597346, EPI_ISL_597349 to 597350, EPI_ISL_597354, EPI_ISL_597358, EPI_ISL_597361, EPI_ISL_597366 to 597368, EPI_ISL_597371 to 597375, EPI_ISL_597377, EPI_ISL_597381, EPI_ISL_597388, EPI_ISL_597390, EPI_ISL_597392, EPI_ISL_597396 to 597399, EPI_ISL_597401 to 597402, EPI_ISL_597405, EPI_ISL_597412, EPI_ISL_597414, EPI_ISL_597418, EPI_ISL_597425 to 597427, EPI_ISL_597432, EPI_ISL_597435, EPI_ISL_597437 to 597438, EPI_ISL_597441, EPI_ISL_597445 to 597446, EPI_ISL_597450 to 597451, EPI_ISL_597453, EPI_ISL_597458, EPI_ISL_597460, EPI_ISL_597464, EPI_ISL_597466 to 597470, EPI_ISL_597472 to 597473, EPI_ISL_597476 to 597480, EPI_ISL_597487 to 597490, EPI_ISL_597494, EPI_ISL_597497, EPI_ISL_597502, EPI_ISL_597504 to 597505, EPI_ISL_597507 to 597508, EPI_ISL_597510, EPI_ISL_597512, EPI_ISL_597517, EPI_ISL_597519 to 597520, EPI_ISL_601493, EPI_ISL_601495, EPI_ISL_601499, EPI_ISL_601501, EPI_ISL_601506, EPI_ISL_601515, EPI_ISL_601522, EPI_ISL_601530, EPI_ISL_601541, EPI_ISL_601550, EPI_ISL_601555, EPI_ISL_601567, EPI_ISL_601569, EPI_ISL_601571, EPI_ISL_601578 to 601580, EPI_ISL_601585, EPI_ISL_601588 to 601589, EPI_ISL_601591, EPI_ISL_601594, EPI_ISL_601604, EPI_ISL_601606, EPI_ISL_601611, EPI_ISL_601615, EPI_ISL_601617 to 601619, EPI_ISL_601627 to 601628, EPI_ISL_601634, EPI_ISL_601639, EPI_ISL_601648, EPI_ISL_601657, EPI_ISL_601663 to 601664, EPI_ISL_601666, EPI_ISL_601668, EPI_ISL_601673, EPI_ISL_601685, EPI_ISL_601690 to 601691, EPI_ISL_601700, EPI_ISL_601715, EPI_ISL_601730, EPI_ISL_601733, EPI_ISL_601740, EPI_ISL_601747, EPI_ISL_601764, EPI_ISL_601768, EPI_ISL_601771, EPI_ISL_601778, EPI_ISL_601783, EPI_ISL_601785, EPI_ISL_602154, EPI_ISL_608348 to 608349, EPI_ISL_608353 to 608354, EPI_ISL_608357, EPI_ISL_608359, EPI_ISL_608368, EPI_ISL_608370, EPI_ISL_608373, EPI_ISL_608381 to 608382, EPI_ISL_608388 to 608389, EPI_ISL_608391 to 608398, EPI_ISL_608400, EPI_ISL_608402, EPI_ISL_608407 to 608408, EPI_ISL_608410 to 608412, EPI_ISL_608416 to 608417, EPI_ISL_608423, EPI_ISL_608426 to 608429, EPI_ISL_608432 to 608433, EPI_ISL_608436 to 608437, EPI_ISL_608442, EPI_ISL_608446 to 608447, EPI_ISL_608449 to 608451, EPI_ISL_608458 to 608459, EPI_ISL_608463 to 608464, EPI_ISL_608466 to 608467, EPI_ISL_608472, EPI_ISL_608475, EPI_ISL_608477 to 608478, EPI_ISL_608480, EPI_ISL_608484 to 608486, EPI_ISL_608490, EPI_ISL_608493 to 608495, EPI_ISL_608497 to 608498, EPI_ISL_608501, EPI_ISL_608506 to 608510, EPI_ISL_608514 to 608515, EPI_ISL_608521 to 608522, EPI_ISL_608524, EPI_ISL_608527, EPI_ISL_608529 to 608532, EPI_ISL_608535 to 608537, EPI_ISL_608540, EPI_ISL_608544 to 608545, EPI_ISL_608547, EPI_ISL_608550, EPI_ISL_608552, EPI_ISL_608557 to 608561, EPI_ISL_608565 to 608566, EPI_ISL_608568, EPI_ISL_608570 to 608572, EPI_ISL_608576 to 608578, EPI_ISL_608588, EPI_ISL_608597 to 608598, EPI_ISL_608602, EPI_ISL_608604, EPI_ISL_608607 to 608608, EPI_ISL_608611 to 608613, EPI_ISL_608615 to 608616, EPI_ISL_608618 to 608619, EPI_ISL_608624, EPI_ISL_608626 to 608629, EPI_ISL_608631 to 608632, EPI_ISL_608636 to 608638, EPI_ISL_608641 to 608645, EPI_ISL_608650, EPI_ISL_608653 to 608654, EPI_ISL_608656, EPI_ISL_608664, EPI_ISL_608666, EPI_ISL_608671, EPI_ISL_608673, EPI_ISL_608678 to 608679, EPI_ISL_608683, EPI_ISL_608688 to 608689, EPI_ISL_608693, EPI_ISL_608695 to 608696, EPI_ISL_608845, EPI_ISL_608852, EPI_ISL_608867 to 608868, EPI_ISL_608875 to 608876, EPI_ISL_608879, EPI_ISL_608892, EPI_ISL_608906, EPI_ISL_608908, EPI_ISL_608910, EPI_ISL_608912, EPI_ISL_608918, EPI_ISL_608923 to 608924, EPI_ISL_608927, EPI_ISL_608935, EPI_ISL_608939, EPI_ISL_608943, EPI_ISL_608947, EPI_ISL_608958 to 608960, EPI_ISL_608965, EPI_ISL_608968 to 608969, EPI_ISL_608974, EPI_ISL_608982, EPI_ISL_608987, EPI_ISL_608990, EPI_ISL_608996 to 608997, EPI_ISL_609003, EPI_ISL_609014, EPI_ISL_609023, EPI_ISL_609025, EPI_ISL_609033, EPI_ISL_609037, EPI_ISL_609045, EPI_ISL_609050, EPI_ISL_609054, EPI_ISL_609072, EPI_ISL_609077, EPI_ISL_609081, EPI_ISL_609086, EPI_ISL_609088, EPI_ISL_609091, EPI_ISL_609097, EPI_ISL_609099 to 609101, EPI_ISL_609111, EPI_ISL_609113 to 609114, EPI_ISL_609118, EPI_ISL_609122 to 609123, EPI_ISL_609129, EPI_ISL_609131, EPI_ISL_609142 to 609143, EPI_ISL_609156, EPI_ISL_609159 to 609169, EPI_ISL_609171 to 609177, EPI_ISL_609180 to 609181, EPI_ISL_609188, EPI_ISL_609193, EPI_ISL_609195 to 609196, EPI_ISL_609200 to 609201, EPI_ISL_609203 to 609205, EPI_ISL_609308 to 609314, EPI_ISL_609316 to 609324, EPI_ISL_609326 to 609334, EPI_ISL_609336 to 609342, EPI_ISL_609344 to 609354, EPI_ISL_609356 to 609377, EPI_ISL_609379 to 609380, EPI_ISL_609382 to 609392, EPI_ISL_609394 to 609405, EPI_ISL_609408 to 609418, EPI_ISL_609420 to 609424, EPI_ISL_609426 to 609447, EPI_ISL_609449, EPI_ISL_609451 to 609462, EPI_ISL_609464 to 609471, EPI_ISL_609473 to 609481, EPI_ISL_609484 to 609505, EPI_ISL_609509, EPI_ISL_609511, EPI_ISL_609513 to 609520, EPI_ISL_609522 to 609526, EPI_ISL_609528 to 609533, EPI_ISL_609535 to 609543, EPI_ISL_609546 to 609562, EPI_ISL_609564 to 609580, EPI_ISL_609582 to 609599, EPI_ISL_609601, EPI_ISL_609603 to 609623, EPI_ISL_609625 to 609642, EPI_ISL_609644 to 609655, EPI_ISL_609657, EPI_ISL_609659 to 609662, EPI_ISL_609665 to 609671, EPI_ISL_609673 to 609676, EPI_ISL_609678 to 609691, EPI_ISL_609693 to 609700, EPI_ISL_609702, EPI_ISL_609704 to 609732, EPI_ISL_609734 to 609738, EPI_ISL_609740 to 609743, EPI_ISL_609745, EPI_ISL_609747 to 609750, EPI_ISL_609752 to 609767, EPI_ISL_609769 to 609777, EPI_ISL_609779 to 609792, EPI_ISL_609794 to 609800, EPI_ISL_610629 to 610638, EPI_ISL_610640 to 610647, EPI_ISL_610649 to 610653, EPI_ISL_610655 to 610674, EPI_ISL_610676 to 610679, EPI_ISL_610681, EPI_ISL_610683 to 610685, EPI_ISL_610687 to 610689, EPI_ISL_610691 to 610693, EPI_ISL_610695 to 610696, EPI_ISL_610698, EPI_ISL_610700 to 610701, EPI_ISL_610703 to 610708, EPI_ISL_610710 to 610712, EPI_ISL_610714 to 610726, EPI_ISL_610728 to 610739, EPI_ISL_610741 to 610742, EPI_ISL_610744 to 610745, EPI_ISL_610747 to 610765, EPI_ISL_610767 to 610770, EPI_ISL_610772 to 610778, EPI_ISL_610781 to 610786, EPI_ISL_610788 to 610801, EPI_ISL_610803 to 610805, EPI_ISL_610807, EPI_ISL_610810 to 610813, EPI_ISL_610815 to 610818, EPI_ISL_610820, EPI_ISL_610822 to 610833, EPI_ISL_610835 to 610842, EPI_ISL_610844 to 610850, EPI_ISL_610852, EPI_ISL_610854 to 610857, EPI_ISL_610859 to 610860, EPI_ISL_610862 to 610867, EPI_ISL_610869 to 610875, EPI_ISL_610877 to 610884, EPI_ISL_610886 to 610891, EPI_ISL_610893 to 610898, EPI_ISL_610900 to 610912, EPI_ISL_610915 to 610917, EPI_ISL_610920 to 610921, EPI_ISL_623220, EPI_ISL_623247 to 623248, EPI_ISL_623256, EPI_ISL_623268, EPI_ISL_623273, EPI_ISL_623276, EPI_ISL_623281, EPI_ISL_623286, EPI_ISL_623288, EPI_ISL_623301, EPI_ISL_623308, EPI_ISL_623312, EPI_ISL_623321, EPI_ISL_623323, EPI_ISL_623330, EPI_ISL_623333 to 623334, EPI_ISL_623344 to 623347, EPI_ISL_623359 to 623360, EPI_ISL_623363 to 623364, EPI_ISL_623366, EPI_ISL_623370, EPI_ISL_623385 to 623386, EPI_ISL_623390, EPI_ISL_623392 to 623393, EPI_ISL_623397 to 623398, EPI_ISL_623404, EPI_ISL_623409, EPI_ISL_623411, EPI_ISL_623424 to 623427, EPI_ISL_623433, EPI_ISL_623437, EPI_ISL_623440, EPI_ISL_623444 to 623445, EPI_ISL_623448 to 623449, EPI_ISL_623451, EPI_ISL_623474, EPI_ISL_623482, EPI_ISL_623485, EPI_ISL_623488 to 623490, EPI_ISL_623494 to 623495, EPI_ISL_623520, EPI_ISL_623527, EPI_ISL_623531, EPI_ISL_623537, EPI_ISL_623539, EPI_ISL_623545, EPI_ISL_623547 to 623548, EPI_ISL_623550, EPI_ISL_623556 to 623557, EPI_ISL_623559, EPI_ISL_623568 to 623570, EPI_ISL_623572, EPI_ISL_623574 to 623576, EPI_ISL_623584, EPI_ISL_623587, EPI_ISL_623593, EPI_ISL_623595, EPI_ISL_623600, EPI_ISL_623605, EPI_ISL_623608 to 623609, EPI_ISL_623611 to 623612, EPI_ISL_623616 to 623617, EPI_ISL_623619, EPI_ISL_623626, EPI_ISL_623640 to 623641, EPI_ISL_623643 to 623645, EPI_ISL_623647, EPI_ISL_623650 to 623652, EPI_ISL_623654, EPI_ISL_623657 to 623658, EPI_ISL_623662 to 623663, EPI_ISL_623665 to 623667, EPI_ISL_623675, EPI_ISL_623679 to 623680, EPI_ISL_623683, EPI_ISL_623688, EPI_ISL_623690, EPI_ISL_623694, EPI_ISL_623702 to 623703, EPI_ISL_623712, EPI_ISL_623717, EPI_ISL_623721, EPI_ISL_623725 to 623726, EPI_ISL_623731, EPI_ISL_623733, EPI_ISL_623740, EPI_ISL_623742, EPI_ISL_623746, EPI_ISL_623749 to 623751, EPI_ISL_623754, EPI_ISL_623758 to 623759, EPI_ISL_623762 to 623764, EPI_ISL_623774, EPI_ISL_623785, EPI_ISL_623788, EPI_ISL_623790 to 623791, EPI_ISL_623793 to 623794, EPI_ISL_623797 to 623798, EPI_ISL_623800, EPI_ISL_623802, EPI_ISL_623806, EPI_ISL_623808, EPI_ISL_623811 to 623812, EPI_ISL_623815 to 623816, EPI_ISL_623823, EPI_ISL_623829, EPI_ISL_623831 to 623834, EPI_ISL_623838, EPI_ISL_623841, EPI_ISL_623844, EPI_ISL_623846 to 623847, EPI_ISL_623849, EPI_ISL_623854, EPI_ISL_623856 to 623857, EPI_ISL_625281 to 625288, EPI_ISL_625290 to 625296, EPI_ISL_625298 to 625303, EPI_ISL_625305 to 625311, EPI_ISL_625313 to 625314, EPI_ISL_625432, EPI_ISL_625437 to 625444, EPI_ISL_625446 to 625453, EPI_ISL_630972, EPI_ISL_630977, EPI_ISL_630979, EPI_ISL_630984, EPI_ISL_630988, EPI_ISL_630996 to 630997, EPI_ISL_631001, EPI_ISL_631003, EPI_ISL_631005, EPI_ISL_631008, EPI_ISL_631016, EPI_ISL_631025, EPI_ISL_631028, EPI_ISL_631038 to 631039, EPI_ISL_631045, EPI_ISL_631053, EPI_ISL_631055, EPI_ISL_631062 to 631063, EPI_ISL_631069, EPI_ISL_631078, EPI_ISL_631080, EPI_ISL_631094, EPI_ISL_631101, EPI_ISL_631105, EPI_ISL_631109, EPI_ISL_631117, EPI_ISL_631123, EPI_ISL_631129, EPI_ISL_631135, EPI_ISL_631137, EPI_ISL_631142, EPI_ISL_631144, EPI_ISL_631148 to 631149, EPI_ISL_631154 to 631155, EPI_ISL_631160, EPI_ISL_631164, EPI_ISL_631171, EPI_ISL_631174, EPI_ISL_631177, EPI_ISL_631180 to 631181, EPI_ISL_631185, EPI_ISL_631191, EPI_ISL_631194, EPI_ISL_631199, EPI_ISL_631204, EPI_ISL_631208, EPI_ISL_631219 to 631221, EPI_ISL_631224 to 631225, EPI_ISL_631235, EPI_ISL_631237, EPI_ISL_631245, EPI_ISL_631254, EPI_ISL_631257, EPI_ISL_631262, EPI_ISL_631266, EPI_ISL_631268, EPI_ISL_631270 to 631274, EPI_ISL_633100 to 633102, EPI_ISL_633104 to 633107, EPI_ISL_633109 to 633110, EPI_ISL_633116 to 633117, EPI_ISL_633120 to 633124, EPI_ISL_633126 to 633131, EPI_ISL_633135 to 633136, EPI_ISL_633140 to 633142, EPI_ISL_633147 to 633150, EPI_ISL_633153, EPI_ISL_633155 to 633157, EPI_ISL_633159, EPI_ISL_633161, EPI_ISL_633163 to 633164, EPI_ISL_633170, EPI_ISL_633173, EPI_ISL_633176 to 633177, EPI_ISL_633183, EPI_ISL_633186 to 633189, EPI_ISL_633192, EPI_ISL_633194, EPI_ISL_633197, EPI_ISL_633199 to 633200, EPI_ISL_633205, EPI_ISL_633208, EPI_ISL_633211 to 633212, EPI_ISL_633214 to 633217, EPI_ISL_633219 to 633220, EPI_ISL_633223 to 633225, EPI_ISL_633227, EPI_ISL_633229, EPI_ISL_633231, EPI_ISL_633234 to 633236, EPI_ISL_633239, EPI_ISL_633242, EPI_ISL_633245 to 633250, EPI_ISL_633252 to 633255, EPI_ISL_633257 to 633258, EPI_ISL_633260, EPI_ISL_633263 to 633267, EPI_ISL_633270, EPI_ISL_633273, EPI_ISL_633275, EPI_ISL_633277 to 633278, EPI_ISL_633281, EPI_ISL_633284, EPI_ISL_633286, EPI_ISL_633288, EPI_ISL_633291 to 633292, EPI_ISL_633294 to 633295, EPI_ISL_633298 to 633300, EPI_ISL_633302, EPI_ISL_633308, EPI_ISL_633311, EPI_ISL_633313 to 633315, EPI_ISL_633319, EPI_ISL_633321 to 633322, EPI_ISL_633324 to 633325, EPI_ISL_633329 to 633330, EPI_ISL_633333 to 633334, EPI_ISL_633336 to 633337, EPI_ISL_633340, EPI_ISL_633344 to 633347, EPI_ISL_633349, EPI_ISL_633356 to 633357, EPI_ISL_633359 to 633360, EPI_ISL_633363, EPI_ISL_633365 to 633366, EPI_ISL_633369 to 633370, EPI_ISL_633372 to 633375, EPI_ISL_633378, EPI_ISL_633380, EPI_ISL_633383, EPI_ISL_633385, EPI_ISL_633388 to 633390, EPI_ISL_633393, EPI_ISL_633395, EPI_ISL_633397 to 633399, EPI_ISL_633403, EPI_ISL_633405, EPI_ISL_633407 to 633408, EPI_ISL_634790 to 634816

see above	Lighthouse Lab in Cambridge	Wellcome Sanger Institute for the COVID-19 Genomics UK (COG-UK) consortium	Cordelia Langford; David K. Jackson; Dominic Kwiatkowski; Ewan Harrison; Ian Johnston; John Sillitoe on behalf of the Wellcome Sanger Institute COVID-19 Surveillance Team; John Sillitoe on behalf of the Wellcome Sanger Institute COVID-19 Surveillance Team (http://www.sanger.ac.uk/covid-team); Rob Howes; Roberto Amato; Sonia Goncalves; The Lighthouse Lab in Cambridge and Alex Alderton

EPI_ISL_641608 to 641611, EPI_ISL_641613 to 641623, EPI_ISL_641625 to 641627, EPI_ISL_641629 to 641639, EPI_ISL_641641 to 641653, EPI_ISL_641655 to 641660, EPI_ISL_641662 to 641663, EPI_ISL_641665 to 641671, EPI_ISL_641673 to 641687, EPI_ISL_641689 to 641737, EPI_ISL_641739 to 641741, EPI_ISL_641743 to 641756, EPI_ISL_641758 to 641760, EPI_ISL_641762 to 641765, EPI_ISL_641767 to 641791, EPI_ISL_641793 to 641796, EPI_ISL_641798 to 641800, EPI_ISL_641802 to 641821, EPI_ISL_641823 to 641826, EPI_ISL_641828 to 641839, EPI_ISL_641841 to 641846, EPI_ISL_641848 to 641849, EPI_ISL_641851 to 641868, EPI_ISL_641870 to 641880, EPI_ISL_641882 to 641939, EPI_ISL_641941 to 641949, EPI_ISL_641951 to 641952, EPI_ISL_641954, EPI_ISL_641956 to 641959, EPI_ISL_641961 to 641985, EPI_ISL_641988 to 641994, EPI_ISL_641996 to 642000, EPI_ISL_642002 to 642006, EPI_ISL_642009 to 642010, EPI_ISL_642012 to 642032, EPI_ISL_642034 to 642038, EPI_ISL_642040 to 642055, EPI_ISL_642057 to 642076, EPI_ISL_642078 to 642093, EPI_ISL_642095 to 642117, EPI_ISL_642119 to 642120, EPI_ISL_642122 to 642133, EPI_ISL_642135 to 642150, EPI_ISL_642152 to 642153, EPI_ISL_642155 to 642171, EPI_ISL_642173 to 642193, EPI_ISL_642195 to 642201, EPI_ISL_642203, EPI_ISL_642205 to 642209, EPI_ISL_642211 to 642212, EPI_ISL_642215 to 642216, EPI_ISL_642218, EPI_ISL_642220 to 642223, EPI_ISL_642225 to 642235, EPI_ISL_642237 to 642238, EPI_ISL_642240 to 642242, EPI_ISL_642244 to 642246, EPI_ISL_642248 to 642257, EPI_ISL_642259 to 642266, EPI_ISL_642268 to 642270, EPI_ISL_642272 to 642273, EPI_ISL_642275 to 642286, EPI_ISL_642288, EPI_ISL_642290 to 642296, EPI_ISL_642298 to 642299, EPI_ISL_642301 to 642316, EPI_ISL_642318, EPI_ISL_642324 to 642343, EPI_ISL_642345 to 642360, EPI_ISL_642362, EPI_ISL_642364 to 642371, EPI_ISL_642373 to 642377, EPI_ISL_642379 to 642381, EPI_ISL_642383 to 642385, EPI_ISL_642387 to 642399, EPI_ISL_642401 to 642407, EPI_ISL_642409 to 642410, EPI_ISL_642412 to 642418, EPI_ISL_642420, EPI_ISL_642422 to 642435, EPI_ISL_642437, EPI_ISL_642439 to 642442, EPI_ISL_642444 to 642449, EPI_ISL_642451 to 642452, EPI_ISL_642454 to 642457, EPI_ISL_642459 to 642470, EPI_ISL_642472 to 642478, EPI_ISL_642481 to 642482, EPI_ISL_642484 to 642486, EPI_ISL_642488 to 642492, EPI_ISL_642495 to 642498, EPI_ISL_642500 to 642504, EPI_ISL_642507 to 642542, EPI_ISL_642545 to 642548, EPI_ISL_642550 to 642572, EPI_ISL_642574 to 642576, EPI_ISL_642578 to 642581, EPI_ISL_642584 to 642591, EPI_ISL_642593 to 642601, EPI_ISL_642603 to 642605, EPI_ISL_642608 to 642624, EPI_ISL_642626, EPI_ISL_642628 to 642646, EPI_ISL_642648 to 642652, EPI_ISL_642654,

EPI_ISL_642656, EPI_ISL_642658 to 642661, EPI_ISL_642665 to 642672, EPI_ISL_642674 to 642681, EPI_ISL_642683 to 642693, EPI_ISL_642695 to 642697, EPI_ISL_642699 to 642703, EPI_ISL_642705 to 642727, EPI_ISL_642729 to 642733, EPI_ISL_642735 to 642750, EPI_ISL_642752 to 642760, EPI_ISL_643044 to 643055, EPI_ISL_643057 to 643063, EPI_ISL_643065 to 643074, EPI_ISL_643077, EPI_ISL_643079 to 643095, EPI_ISL_643097 to 643102, EPI_ISL_643104, EPI_ISL_643106 to 643112, EPI_ISL_643114 to 643119, EPI_ISL_643121 to 643127, EPI_ISL_643129 to 643137, EPI_ISL_643139 to 643140, EPI_ISL_643142 to 643148, EPI_ISL_643150 to 643179, EPI_ISL_643181 to 643212, EPI_ISL_643214 to 643227, EPI_ISL_643229 to 643230, EPI_ISL_643232 to 643233, EPI_ISL_643235, EPI_ISL_643237 to 643238, EPI_ISL_643241 to 643253, EPI_ISL_643256 to 643274, EPI_ISL_643276 to 643285, EPI_ISL_643288, EPI_ISL_643292 to 643301, EPI_ISL_643303 to 643312, EPI_ISL_643314, EPI_ISL_643992, EPI_ISL_643994 to 643995, EPI_ISL_643997 to 644002, EPI_ISL_644004 to 644005, EPI_ISL_644009 to 644014, EPI_ISL_644016 to 644021, EPI_ISL_644023 to 644028, EPI_ISL_644030 to 644032, EPI_ISL_644034 to 644041, EPI_ISL_644044 to 644051, EPI_ISL_644094, EPI_ISL_644101, EPI_ISL_644107, EPI_ISL_644112, EPI_ISL_646140 to 646147, EPI_ISL_646149, EPI_ISL_646151 to 646154, EPI_ISL_646158 to 646161, EPI_ISL_646163 to 646164, EPI_ISL_646167 to 646168, EPI_ISL_646171, EPI_ISL_646174 to 646179, EPI_ISL_646183 to 646184, EPI_ISL_646186 to 646188, EPI_ISL_646192, EPI_ISL_646194 to 646197, EPI_ISL_646200 to 646203, EPI_ISL_646205 to 646206, EPI_ISL_646209, EPI_ISL_646211, EPI_ISL_646213 to 646214, EPI_ISL_646216 to 646222, EPI_ISL_646225 to 646230, EPI_ISL_646233 to 646234, EPI_ISL_646236 to 646241, EPI_ISL_646243 to 646244, EPI_ISL_646246 to 646247, EPI_ISL_646250, EPI_ISL_646253 to 646260, EPI_ISL_646262 to 646267, EPI_ISL_646270, EPI_ISL_646272 to 646275, EPI_ISL_646280 to 646281, EPI_ISL_646284 to 646290, EPI_ISL_646292 to 646296, EPI_ISL_646298, EPI_ISL_646300 to 646302, EPI_ISL_646304, EPI_ISL_646306, EPI_ISL_646308, EPI_ISL_646310 to 646312, EPI_ISL_646315 to 646317, EPI_ISL_646319, EPI_ISL_646321 to 646322, EPI_ISL_646324 to 646326, EPI_ISL_646328 to 646333, EPI_ISL_646335 to 646341, EPI_ISL_646344, EPI_ISL_646348, EPI_ISL_646351 to 646353, EPI_ISL_646355, EPI_ISL_646357 to 646361, EPI_ISL_646364 to 646365, EPI_ISL_646368, EPI_ISL_646371 to 646372, EPI_ISL_646374, EPI_ISL_646378 to 646381, EPI_ISL_646384, EPI_ISL_646386 to 646387, EPI_ISL_646389 to 646392, EPI_ISL_646394 to 646396, EPI_ISL_646399 to 646401, EPI_ISL_646403 to 646411, EPI_ISL_646413 to 646417, EPI_ISL_646421 to 646425, EPI_ISL_646427, EPI_ISL_646433, EPI_ISL_646435 to 646440, EPI_ISL_646444 to 646447, EPI_ISL_646449 to 646450, EPI_ISL_646452 to 646453, EPI_ISL_646456 to 646462, EPI_ISL_646464 to 646465, EPI_ISL_646469 to 646475, EPI_ISL_646480 to 646484, EPI_ISL_646495 to 646496, EPI_ISL_646498 to 646499, EPI_ISL_646501, EPI_ISL_646503 to 646504, EPI_ISL_646507, EPI_ISL_646517, EPI_ISL_646519, EPI_ISL_646523 to 646524, EPI_ISL_646527, EPI_ISL_646530 to 646531, EPI_ISL_646536, EPI_ISL_646539, EPI_ISL_646547, EPI_ISL_646550, EPI_ISL_646553 to 646554, EPI_ISL_646556, EPI_ISL_646562, EPI_ISL_646565, EPI_ISL_646570, EPI_ISL_646574 to 646575, EPI_ISL_646578 to 646579, EPI_ISL_646582, EPI_ISL_646584, EPI_ISL_646588, EPI_ISL_646590, EPI_ISL_646593, EPI_ISL_646596, EPI_ISL_646598, EPI_ISL_646602, EPI_ISL_646604, EPI_ISL_646608 to 646609, EPI_ISL_646615, EPI_ISL_646617, EPI_ISL_646619 to 646620, EPI_ISL_646622, EPI_ISL_646624, EPI_ISL_646630, EPI_ISL_646632 to 646634, EPI_ISL_646636 to 646637, EPI_ISL_646641, EPI_ISL_646643, EPI_ISL_646645 to 646647, EPI_ISL_646649 to 646650, EPI_ISL_646656 to 646657, EPI_ISL_646659, EPI_ISL_646661, EPI_ISL_646663, EPI_ISL_646667, EPI_ISL_646669 to 646670, EPI_ISL_646672, EPI_ISL_646677, EPI_ISL_646680 to 646682, EPI_ISL_646685, EPI_ISL_646688, EPI_ISL_646690, EPI_ISL_646694, EPI_ISL_646700 to 646701, EPI_ISL_646703 to 646704, EPI_ISL_646714, EPI_ISL_646717, EPI_ISL_646720, EPI_ISL_646723, EPI_ISL_646725 to 646726, EPI_ISL_646728 to 646730, EPI_ISL_646732 to 646733, EPI_ISL_646735, EPI_ISL_646742, EPI_ISL_646745, EPI_ISL_646748, EPI_ISL_646754, EPI_ISL_646759, EPI_ISL_646764 to 646766, EPI_ISL_646772, EPI_ISL_646774 to 646775, EPI_ISL_646777 to 646779, EPI_ISL_646781, EPI_ISL_646783, EPI_ISL_646785 to 646786, EPI_ISL_646788, EPI_ISL_646792, EPI_ISL_646794, EPI_ISL_646798, EPI_ISL_646818, EPI_ISL_646825 to 646826, EPI_ISL_646837, EPI_ISL_646850, EPI_ISL_646853 to 646854, EPI_ISL_646868 to 646869, EPI_ISL_646872, EPI_ISL_646874, EPI_ISL_646877, EPI_ISL_646880, EPI_ISL_646885, EPI_ISL_646891 to 646892, EPI_ISL_646902, EPI_ISL_646905, EPI_ISL_646912, EPI_ISL_646915, EPI_ISL_646921, EPI_ISL_646929, EPI_ISL_646936 to 646937, EPI_ISL_646939, EPI_ISL_646948, EPI_ISL_646955, EPI_ISL_646960, EPI_ISL_646964, EPI_ISL_646974 to 646977, EPI_ISL_646983, EPI_ISL_646988, EPI_ISL_646992, EPI_ISL_646996, EPI_ISL_647001, EPI_ISL_647003, EPI_ISL_647006, EPI_ISL_647014, EPI_ISL_647023, EPI_ISL_647031 to 647032, EPI_ISL_647035, EPI_ISL_647045, EPI_ISL_647050, EPI_ISL_647059, EPI_ISL_647061, EPI_ISL_647067, EPI_ISL_647076, EPI_ISL_647087, EPI_ISL_647090, EPI_ISL_647097, EPI_ISL_647102, EPI_ISL_647105, EPI_ISL_647109 to 647110, EPI_ISL_647114 to 647115, EPI_ISL_647117, EPI_ISL_647797 to 647806, EPI_ISL_647854, EPI_ISL_647856 to 647862, EPI_ISL_647864, EPI_ISL_647866 to 647869, EPI_ISL_647871 to 647875, EPI_ISL_647877 to 647889, EPI_ISL_647891 to 647892, EPI_ISL_647894 to 647899, EPI_ISL_647902, EPI_ISL_647904 to 647905, EPI_ISL_647907, EPI_ISL_647910 to 647912, EPI_ISL_647914 to 647915, EPI_ISL_647917 to 647968, EPI_ISL_649494 to 649495, EPI_ISL_649497 to 649502, EPI_ISL_649504 to 649523, EPI_ISL_649525 to 649536, EPI_ISL_649538 to 649542, EPI_ISL_649544 to 649547, EPI_ISL_649556 to 649558, EPI_ISL_649560 to 649564, EPI_ISL_649568 to 649579, EPI_ISL_649581 to 649590, EPI_ISL_649592 to 649596, EPI_ISL_649598 to 649603, EPI_ISL_649605, EPI_ISL_649608, EPI_ISL_649610 to 649622, EPI_ISL_649624, EPI_ISL_649634 to 649648, EPI_ISL_649650 to 649659, EPI_ISL_649661 to 649663, EPI_ISL_649666 to 649673, EPI_ISL_649675 to 649699, EPI_ISL_649701 to 649709, EPI_ISL_649711, EPI_ISL_649713 to 649714, EPI_ISL_649717 to 649721, EPI_ISL_649723 to 649724, EPI_ISL_649727 to 649731, EPI_ISL_649733 to 649739, EPI_ISL_649741 to 649743, EPI_ISL_649745, EPI_ISL_649747 to 649749, EPI_ISL_649752 to 649755, EPI_ISL_649757 to 649765, EPI_ISL_649767 to 649783, EPI_ISL_654959 to 654999, EPI_ISL_655001 to 655009, EPI_ISL_655012 to 655021, EPI_ISL_655023 to 655029, EPI_ISL_655031 to 655032, EPI_ISL_655034 to 655035, EPI_ISL_655039 to 655043, EPI_ISL_655045, EPI_ISL_655047, EPI_ISL_655049, EPI_ISL_655051, EPI_ISL_655053 to 655057, EPI_ISL_655059 to 655062, EPI_ISL_655064 to 655067, EPI_ISL_655069 to 655070, EPI_ISL_655072 to 655073, EPI_ISL_655075 to 655076, EPI_ISL_655078 to 655085, EPI_ISL_655087 to 655088, EPI_ISL_655090 to 655097, EPI_ISL_655099, EPI_ISL_655101 to 655104, EPI_ISL_655106, EPI_ISL_655109 to 655110, EPI_ISL_655112 to 655114, EPI_ISL_655116 to 655123, EPI_ISL_655125 to 655127, EPI_ISL_655129 to 655130, EPI_ISL_655132 to 655140, EPI_ISL_655142 to 655158, EPI_ISL_655160, EPI_ISL_655162 to 655166, EPI_ISL_655169 to 655176, EPI_ISL_655178 to 655180, EPI_ISL_655182 to 655183, EPI_ISL_655185, EPI_ISL_655188 to 655194, EPI_ISL_655196 to 655211, EPI_ISL_655214, EPI_ISL_655216 to 655217, EPI_ISL_655219 to 655223, EPI_ISL_655226 to 655228, EPI_ISL_655230 to 655245, EPI_ISL_655247, EPI_ISL_655250 to 655259, EPI_ISL_655262, EPI_ISL_655264 to 655270, EPI_ISL_655272, EPI_ISL_655651, EPI_ISL_655660, EPI_ISL_655662, EPI_ISL_655672, EPI_ISL_655677, EPI_ISL_655694 to 655695, EPI_ISL_655699, EPI_ISL_655711, EPI_ISL_655727, EPI_ISL_655735, EPI_ISL_655737, EPI_ISL_655739, EPI_ISL_655744 to 655745, EPI_ISL_655756, EPI_ISL_655768 to 655769, EPI_ISL_655773, EPI_ISL_655779 to 655780, EPI_ISL_655783, EPI_ISL_655788, EPI_ISL_655798, EPI_ISL_655804, EPI_ISL_655806 to 655807, EPI_ISL_655809, EPI_ISL_655817, EPI_ISL_655819, EPI_ISL_655841, EPI_ISL_655847, EPI_ISL_655854, EPI_ISL_655861, EPI_ISL_655868, EPI_ISL_655874, EPI_ISL_655879, EPI_ISL_655883, EPI_ISL_655891, EPI_ISL_655907, EPI_ISL_655911 to 655912, EPI_ISL_655918, EPI_ISL_655920, EPI_ISL_655924, EPI_ISL_655929, EPI_ISL_655957, EPI_ISL_655959, EPI_ISL_655967, EPI_ISL_655971, EPI_ISL_655973, EPI_ISL_655975, EPI_ISL_657027 to 657057, EPI_ISL_657059 to 657067, EPI_ISL_657070, EPI_ISL_657072 to 657081, EPI_ISL_657083, EPI_ISL_657086 to 657092, EPI_ISL_657094 to 657100, EPI_ISL_657102 to 657114, EPI_ISL_657117 to 657148, EPI_ISL_657150 to 657155, EPI_ISL_657157 to 657190, EPI_ISL_657192 to 657201, EPI_ISL_657203 to 657209, EPI_ISL_657211 to 657213, EPI_ISL_657215, EPI_ISL_657217 to 657220, EPI_ISL_657222 to 657235, EPI_ISL_657237 to 657240, EPI_ISL_657242 to 657254, EPI_ISL_657256, EPI_ISL_657258 to 657260, EPI_ISL_657264 to 657293, EPI_ISL_657296 to 657330, EPI_ISL_657332 to 657340, EPI_ISL_657342 to 657343, EPI_ISL_657345 to 657354, EPI_ISL_658405 to 658408, EPI_ISL_658411, EPI_ISL_658413 to 658428, EPI_ISL_658430 to 658453, EPI_ISL_658456 to 658488, EPI_ISL_658491 to 658493, EPI_ISL_658495 to 658503, EPI_ISL_658508 to 658511, EPI_ISL_658513 to 658517, EPI_ISL_658519 to 658523, EPI_ISL_658525 to 658531, EPI_ISL_658533 to 658538, EPI_ISL_658540 to 658575, EPI_ISL_658577 to 658583, EPI_ISL_658585 to 658598, EPI_ISL_658600 to 658638, EPI_ISL_658640 to 658654, EPI_ISL_658656 to 658659, EPI_ISL_658662 to 658665, EPI_ISL_658667, EPI_ISL_658669 to 658680, EPI_ISL_658682 to 658685, EPI_ISL_658688 to 658689, EPI_ISL_658691 to 658715, EPI_ISL_658717, EPI_ISL_658719 to 658721, EPI_ISL_658723 to 658727, EPI_ISL_658731 to 658738, EPI_ISL_658740, EPI_ISL_659744, EPI_ISL_659746 to 659747, EPI_ISL_659751 to 659754, EPI_ISL_659756, EPI_ISL_659758 to 659763, EPI_ISL_659765 to 659767, EPI_ISL_659770 to 659771, EPI_ISL_659773 to 659776, EPI_ISL_659778 to 659779, EPI_ISL_659781 to 659784, EPI_ISL_659787, EPI_ISL_659789 to 659794, EPI_ISL_659796 to 659797, EPI_ISL_659799 to 659803, EPI_ISL_659805 to 659808, EPI_ISL_659810 to 659811, EPI_ISL_659813 to 659823, EPI_ISL_659825, EPI_ISL_659827 to 659833, EPI_ISL_659836 to 659838, EPI_ISL_659840, EPI_ISL_659842 to 659847, EPI_ISL_659849, EPI_ISL_659851 to 659859, EPI_ISL_659861, EPI_ISL_659863, EPI_ISL_659865 to 659869, EPI_ISL_659871 to 659884, EPI_ISL_659886 to 659887, EPI_ISL_659889 to 659900, EPI_ISL_659902, EPI_ISL_659907 to 659908, EPI_ISL_659910, EPI_ISL_659912 to 659913, EPI_ISL_659915, EPI_ISL_659917 to 659947, EPI_ISL_659949 to 659951, EPI_ISL_659953, EPI_ISL_659955 to 659959, EPI_ISL_659961 to 659967, EPI_ISL_659971 to 659972, EPI_ISL_659975, EPI_ISL_659977 to 659984, EPI_ISL_659986, EPI_ISL_659988 to 659989, EPI_ISL_659991, EPI_ISL_659993 to 659994, EPI_ISL_659996 to 659998, EPI_ISL_660000 to 660001, EPI_ISL_660005 to 660008, EPI_ISL_660010 to 660011, EPI_ISL_660013 to 660016, EPI_ISL_660019 to 660025, EPI_ISL_660027 to 660030, EPI_ISL_660032 to 660041, EPI_ISL_660043 to 660054, EPI_ISL_660056 to 660062, EPI_ISL_660064 to 660065, EPI_ISL_661314 to 661316, EPI_ISL_661319 to 661320, EPI_ISL_661322 to 661333, EPI_ISL_661335, EPI_ISL_661337 to 661344, EPI_ISL_661346 to 661349, EPI_ISL_661351 to 661352, EPI_ISL_661354 to 661356, EPI_ISL_661358 to 661361, EPI_ISL_661363 to 661366, EPI_ISL_661369 to 661376, EPI_ISL_661378, EPI_ISL_661380, EPI_ISL_661382 to 661385, EPI_ISL_661387 to 661394, EPI_ISL_661396 to 661401, EPI_ISL_661403 to 661413, EPI_ISL_661415 to 661425, EPI_ISL_661427 to 661432, EPI_ISL_661435 to 661441, EPI_ISL_661443 to 661446, EPI_ISL_661448 to 661452, EPI_ISL_661454 to 661457, EPI_ISL_661459 to 661460, EPI_ISL_661462 to 661464, EPI_ISL_661466, EPI_ISL_661468 to 661472, EPI_ISL_661475 to 661478, EPI_ISL_661480 to 661484, EPI_ISL_661486 to 661487, EPI_ISL_661489 to 661490, EPI_ISL_661492 to 661493, EPI_ISL_661496 to 661498, EPI_ISL_661500 to 661510, EPI_ISL_661512, EPI_ISL_661514, EPI_ISL_661517 to 661519, EPI_ISL_661521 to 661522, EPI_ISL_661524 to 661535, EPI_ISL_661537, EPI_ISL_661539 to 661542, EPI_ISL_661544 to 661545, EPI_ISL_661548 to 661550, EPI_ISL_661552, EPI_ISL_661554 to 661569, EPI_ISL_661571 to 661580, EPI_ISL_661584 to 661587, EPI_ISL_661591 to 661593, EPI_ISL_661595 to 661600, EPI_ISL_661602 to 661605, EPI_ISL_661607 to 661618, EPI_ISL_661620, EPI_ISL_661622, EPI_ISL_661624, EPI_ISL_661626 to 661631, EPI_ISL_661633 to 661635, EPI_ISL_661637, EPI_ISL_661642 to 661645, EPI_ISL_661648 to 661650, EPI_ISL_661655, EPI_ISL_661658 to 661659, EPI_ISL_661661, EPI_ISL_661663, EPI_ISL_661665 to 661666, EPI_ISL_661668 to 661671, EPI_ISL_661673 to 661674, EPI_ISL_661677 to 661678, EPI_ISL_661681, EPI_ISL_661683, EPI_ISL_661685 to 661686, EPI_ISL_661688 to 661690, EPI_ISL_661694, EPI_ISL_661698 to 661701, EPI_ISL_661703 to 661704, EPI_ISL_661706, EPI_ISL_661708 to 661709, EPI_ISL_661715 to 661716, EPI_ISL_661718 to 661720, EPI_ISL_661722 to 661725, EPI_ISL_661727, EPI_ISL_661730 to 661731, EPI_ISL_661733, EPI_ISL_661735 to 661738, EPI_ISL_661740, EPI_ISL_661744 to 661746, EPI_ISL_661748, EPI_ISL_661753 to 661758, EPI_ISL_661760, EPI_ISL_661762, EPI_ISL_661764 to 661768, EPI_ISL_661770 to 661773, EPI_ISL_661776 to 661777, EPI_ISL_661780, EPI_ISL_661782, EPI_ISL_661784, EPI_ISL_661786 to 661788, EPI_ISL_661790, EPI_ISL_661792, EPI_ISL_661795, EPI_ISL_661798, EPI_ISL_661800, EPI_ISL_661802 to 661804, EPI_ISL_661806 to 661810, EPI_ISL_661812 to 661813, EPI_ISL_661816, EPI_ISL_661818 to 661819, EPI_ISL_661823 to 661825, EPI_ISL_661827 to 661829, EPI_ISL_661831 to 661832, EPI_ISL_661835, EPI_ISL_661837, EPI_ISL_661839 to 661842, EPI_ISL_661846 to 661852, EPI_ISL_661855, EPI_ISL_661857, EPI_ISL_661859 to 661862, EPI_ISL_661865 to 661868, EPI_ISL_661870, EPI_ISL_661872, EPI_ISL_661875, EPI_ISL_661877 to 661880, EPI_ISL_661884 to 661885, EPI_ISL_661888 to 661891, EPI_ISL_661893 to 661894, EPI_ISL_661899, EPI_ISL_661901, EPI_ISL_661904 to 661914, EPI_ISL_661916, EPI_ISL_661919, EPI_ISL_661922 to 661924, EPI_ISL_661927, EPI_ISL_661929, EPI_ISL_661931 to 661940, EPI_ISL_661942, EPI_ISL_661945, EPI_ISL_661948 to 661950, EPI_ISL_661952, EPI_ISL_661955, EPI_ISL_661957, EPI_ISL_661959 to 661960, EPI_ISL_661963, EPI_ISL_661967, EPI_ISL_661971 to 661973, EPI_ISL_661975 to 661976, EPI_ISL_661979, EPI_ISL_661984, EPI_ISL_661987, EPI_ISL_661993 to 661994, EPI_ISL_661996 to 661997, EPI_ISL_662003, EPI_ISL_662005, EPI_ISL_662009, EPI_ISL_662011 to 662012, EPI_ISL_662016, EPI_ISL_662021, EPI_ISL_662024, EPI_ISL_662027 to 662028, EPI_ISL_662030, EPI_ISL_662035, EPI_ISL_662037, EPI_ISL_662039 to 662040, EPI_ISL_662042, EPI_ISL_662044, EPI_ISL_662052, EPI_ISL_662054, EPI_ISL_662059, EPI_ISL_662064, EPI_ISL_662066, EPI_ISL_662069, EPI_ISL_662072 to 662073, EPI_ISL_662075 to 662077, EPI_ISL_662082 to 662085, EPI_ISL_662087 to 662092, EPI_ISL_662094, EPI_ISL_662096, EPI_ISL_662102 to 662104, EPI_ISL_662109 to 662111, EPI_ISL_662117, EPI_ISL_662119, EPI_ISL_662121, EPI_ISL_662125 to 662128, EPI_ISL_662130, EPI_ISL_662134, EPI_ISL_662136, EPI_ISL_662139, EPI_ISL_662142, EPI_ISL_662144, EPI_ISL_662147, EPI_ISL_662149, EPI_ISL_662152 to 662155, EPI_ISL_662157 to 662158, EPI_ISL_662161, EPI_ISL_662168, EPI_ISL_662171 to 662172, EPI_ISL_662180, EPI_ISL_662188 to 662190, EPI_ISL_662195, EPI_ISL_662197, EPI_ISL_662199, EPI_ISL_662201, EPI_ISL_662204 to 662205, EPI_ISL_662210 to 662211, EPI_ISL_662216, EPI_ISL_662220 to 662222, EPI_ISL_662225 to 662226, EPI_ISL_662230, EPI_ISL_662232, EPI_ISL_662241, EPI_ISL_662243, EPI_ISL_662246, EPI_ISL_662250, EPI_ISL_662253, EPI_ISL_662256 to 662257, EPI_ISL_662261 to 662262, EPI_ISL_662264, EPI_ISL_662268, EPI_ISL_662271, EPI_ISL_662277, EPI_ISL_662280, EPI_ISL_662282 to 662284, EPI_ISL_662287 to 662289, EPI_ISL_662293 to 662294, EPI_ISL_662297 to 662298, EPI_ISL_662301 to 662302, EPI_ISL_662304, EPI_ISL_662306, EPI_ISL_662311, EPI_ISL_662313 to 662317, EPI_ISL_662323 to 662326, EPI_ISL_662328 to 662333, EPI_ISL_662335, EPI_ISL_662337 to 662341, EPI_ISL_662345, EPI_ISL_662347 to 662351, EPI_ISL_662353, EPI_ISL_662355, EPI_ISL_662357, EPI_ISL_662361 to 662362, EPI_ISL_662364, EPI_ISL_662366 to 662368, EPI_ISL_662370 to 662371, EPI_ISL_662376 to 662380, EPI_ISL_662383 to 662384, EPI_ISL_662386 to 662388, EPI_ISL_662392, EPI_ISL_662395 to 662396, EPI_ISL_662399, EPI_ISL_662401, EPI_ISL_662406 to 662409, EPI_ISL_662414, EPI_ISL_662416 to 662417, EPI_ISL_662420 to 662424, EPI_ISL_662428, EPI_ISL_662430, EPI_ISL_662432, EPI_ISL_662434 to 662443, EPI_ISL_662448 to 662451, EPI_ISL_662453 to 662456, EPI_ISL_662458 to 662459, EPI_ISL_662461, EPI_ISL_662465 to 662467, EPI_ISL_662469, EPI_ISL_662471, EPI_ISL_662473, EPI_ISL_662475 to 662476, EPI_ISL_662481 to 662482, EPI_ISL_662484, EPI_ISL_662490, EPI_ISL_662494 to 662495, EPI_ISL_662497 to 662498, EPI_ISL_662500 to 662501, EPI_ISL_662504 to 662506, EPI_ISL_662509 to 662510, EPI_ISL_662512 to 662513, EPI_ISL_662515, EPI_ISL_662517, EPI_ISL_662521 to 662523, EPI_ISL_662525, EPI_ISL_662527 to 662528, EPI_ISL_662530, EPI_ISL_662532, EPI_ISL_662534, EPI_ISL_662536 to 662542, EPI_ISL_662544, EPI_ISL_662546 to 662548, EPI_ISL_662550 to 662552, EPI_ISL_662554 to 662555, EPI_ISL_662557, EPI_ISL_662560 to 662563, EPI_ISL_662565 to 662574, EPI_ISL_662577, EPI_ISL_662582, EPI_ISL_662585, EPI_ISL_662587, EPI_ISL_662589, EPI_ISL_662591 to 662592, EPI_ISL_662595 to 662596, EPI_ISL_662598 to 662600, EPI_ISL_662603, EPI_ISL_662607, EPI_ISL_662611 to 662613, EPI_ISL_662615, EPI_ISL_662618 to 662620, EPI_ISL_662627, EPI_ISL_662655 to 662656, EPI_ISL_662660 to 662663, EPI_ISL_662668, EPI_ISL_662675 to 662677, EPI_ISL_662680 to 662682, EPI_ISL_662684, EPI_ISL_662688 to 662692, EPI_ISL_662695, EPI_ISL_662698 to 662699, EPI_ISL_662701 to 662707, EPI_ISL_662710 to 662711, EPI_ISL_662713, EPI_ISL_662715 to 662716, EPI_ISL_662720, EPI_ISL_662724, EPI_ISL_662726 to 662727, EPI_ISL_662729 to 662732, EPI_ISL_662734, EPI_ISL_662736 to 662737, EPI_ISL_662739, EPI_ISL_662746 to 662750, EPI_ISL_662752 to 662758, EPI_ISL_662760 to 662761, EPI_ISL_662764 to 662769, EPI_ISL_662771, EPI_ISL_662775 to 662780, EPI_ISL_662782 to 662785, EPI_ISL_662787, EPI_ISL_662789, EPI_ISL_662791 to 662792, EPI_ISL_662794 to 662797, EPI_ISL_662800, EPI_ISL_662802 to 662803, EPI_ISL_662805, EPI_ISL_662808 to 662809, EPI_ISL_662811, EPI_ISL_662813 to 662815, EPI_ISL_662818 to 662819, EPI_ISL_662823 to 662826, EPI_ISL_662829 to 662831, EPI_ISL_662833 to 662835, EPI_ISL_662837 to 662840, EPI_ISL_662843 to 662844, EPI_ISL_662846 to 662848, EPI_ISL_662850 to 662851, EPI_ISL_662853, EPI_ISL_662856 to 662857, EPI_ISL_662860 to 662868, EPI_ISL_662870 to 662873, EPI_ISL_662875 to 662876, EPI_ISL_662878 to 662884, EPI_ISL_662886 to 662895, EPI_ISL_662897 to 662932, EPI_ISL_662934 to 662938, EPI_ISL_662940 to 662951, EPI_ISL_662955 to 662957, EPI_ISL_662960 to 662961, EPI_ISL_662963 to 662978, EPI_ISL_662980 to 662983, EPI_ISL_662986 to 662999, EPI_ISL_663001 to 663002, EPI_ISL_663004 to 663016, EPI_ISL_663018 to 663027, EPI_ISL_663029, EPI_ISL_663031 to 663039, EPI_ISL_663041 to 663045, EPI_ISL_663047 to 663050, EPI_ISL_663052 to 663054, EPI_ISL_663056 to 663058, EPI_ISL_663060, EPI_ISL_663063 to 663066, EPI_ISL_663068 to 663072, EPI_ISL_663074 to 663075, EPI_ISL_663078 to 663099, EPI_ISL_663101 to 663104, EPI_ISL_663106, EPI_ISL_663109 to 663121, EPI_ISL_663123 to 663124, EPI_ISL_663126 to 663127, EPI_ISL_663129 to 663158, EPI_ISL_663160 to 663170, EPI_ISL_663172 to 663177, EPI_ISL_663180 to 663184, EPI_ISL_663186 to 663196, EPI_ISL_663198 to 663199, EPI_ISL_663201 to 663205, EPI_ISL_667814, EPI_ISL_667816, EPI_ISL_667824 to 667825, EPI_ISL_667828, EPI_ISL_667832, EPI_ISL_667836, EPI_ISL_667838, EPI_ISL_667843, EPI_ISL_667849, EPI_ISL_667851, EPI_ISL_667855, EPI_ISL_667857, EPI_ISL_667859 to 667860, EPI_ISL_667863, EPI_ISL_667866, EPI_ISL_667872 to 667873, EPI_ISL_667881, EPI_ISL_667883, EPI_ISL_667885, EPI_ISL_667887 to 667889, EPI_ISL_667891, EPI_ISL_667893, EPI_ISL_667895, EPI_ISL_667898, EPI_ISL_667900, EPI_ISL_667904, EPI_ISL_667907, EPI_ISL_667912, EPI_ISL_667915, EPI_ISL_667917 to 667919, EPI_ISL_667922, EPI_ISL_667924, EPI_ISL_667927 to 667929, EPI_ISL_667931, EPI_ISL_667938, EPI_ISL_667941, EPI_ISL_667947, EPI_ISL_667950, EPI_ISL_667952, EPI_ISL_667958 to 667960, EPI_ISL_667962 to 667963, EPI_ISL_667966 to 667967, EPI_ISL_667969 to 667970, EPI_ISL_667972 to 667973, EPI_ISL_667975, EPI_ISL_667977 to 667978, EPI_ISL_667983, EPI_ISL_667986, EPI_ISL_667989 to 667990, EPI_ISL_667992, EPI_ISL_667996, EPI_ISL_668001, EPI_ISL_668004 to 668006, EPI_ISL_668010, EPI_ISL_668013, EPI_ISL_668021 to 668022, EPI_ISL_668024, EPI_ISL_668028 to 668031, EPI_ISL_668033 to 668035, EPI_ISL_668044 to 668045, EPI_ISL_668049 to 668050, EPI_ISL_668052 to 668057, EPI_ISL_668060, EPI_ISL_668062 to 668064, EPI_ISL_668066 to 668067, EPI_ISL_668073 to 668075, EPI_ISL_668078, EPI_ISL_668082, EPI_ISL_668084, EPI_ISL_668093 to 668096, EPI_ISL_668098, EPI_ISL_668102, EPI_ISL_668105 to 668107, EPI_ISL_668113 to 668114, EPI_ISL_668116, EPI_ISL_668118 to 668121, EPI_ISL_668124, EPI_ISL_668129 to 668130, EPI_ISL_668132, EPI_ISL_668134 to 668135, EPI_ISL_668142, EPI_ISL_668144 to 668145, EPI_ISL_668150, EPI_ISL_668157, EPI_ISL_668159, EPI_ISL_668161 to 668162, EPI_ISL_668165, EPI_ISL_668167 to 668168, EPI_ISL_668173 to 668174, EPI_ISL_668176 to 668178, EPI_ISL_668181 to 668183, EPI_ISL_668186, EPI_ISL_668189, EPI_ISL_668193, EPI_ISL_668196 to 668199, EPI_ISL_668202 to 668203, EPI_ISL_668205 to 668206, EPI_ISL_668208 to 668209, EPI_ISL_668215 to 668218, EPI_ISL_668220, EPI_ISL_668223 to 668224, EPI_ISL_668226, EPI_ISL_668229 to 668231, EPI_ISL_668235 to 668236, EPI_ISL_668240 to 668243, EPI_ISL_668245, EPI_ISL_668248 to 668249, EPI_ISL_668253, EPI_ISL_668256 to 668260, EPI_ISL_668264, EPI_ISL_668266, EPI_ISL_668269, EPI_ISL_668271 to 668273, EPI_ISL_668276 to 668278, EPI_ISL_668282, EPI_ISL_668284, EPI_ISL_668286 to 668288, EPI_ISL_668290 to 668291, EPI_ISL_668300, EPI_ISL_668303, EPI_ISL_668307, EPI_ISL_668311, EPI_ISL_668320 to 668321, EPI_ISL_668323, EPI_ISL_668328, EPI_ISL_668330 to 668334, EPI_ISL_668338 to 668340, EPI_ISL_668344, EPI_ISL_668346, EPI_ISL_668348, EPI_ISL_668350, EPI_ISL_668352, EPI_ISL_668359 to 668360, EPI_ISL_668362, EPI_ISL_668364, EPI_ISL_668366 to 668367, EPI_ISL_668372, EPI_ISL_668380 to 668381, EPI_ISL_668383, EPI_ISL_668386 to 668387, EPI_ISL_672754, EPI_ISL_672758 to 672761, EPI_ISL_672764 to 672765, EPI_ISL_672767 to 672770, EPI_ISL_672773 to 672775, EPI_ISL_672777 to 672778, EPI_ISL_672780 to 672782, EPI_ISL_672786 to 672790, EPI_ISL_672793, EPI_ISL_672795 to 672799, EPI_ISL_672804 to 672805, EPI_ISL_672807, EPI_ISL_672810 to 672811, EPI_ISL_672813, EPI_ISL_672819, EPI_ISL_672823 to 672825, EPI_ISL_672828 to 672829, EPI_ISL_672834 to 672838, EPI_ISL_672840 to 672841, EPI_ISL_672844 to 672846, EPI_ISL_672848, EPI_ISL_672850 to 672851, EPI_ISL_672853 to 672861, EPI_ISL_672863 to 672864, EPI_ISL_672866 to 672870, EPI_ISL_672872, EPI_ISL_672874, EPI_ISL_672876 to 672879, EPI_ISL_672881 to 672882, EPI_ISL_672884, EPI_ISL_672886, EPI_ISL_672888 to 672889, EPI_ISL_672893 to 672895, EPI_ISL_672897, EPI_ISL_672899 to 672900, EPI_ISL_672902 to 672905, EPI_ISL_672907 to 672908, EPI_ISL_672911, EPI_ISL_674498 to 674499, EPI_ISL_674501, EPI_ISL_674514, EPI_ISL_674516, EPI_ISL_674522, EPI_ISL_674527 to 674528, EPI_ISL_674530, EPI_ISL_674543 to 674545, EPI_ISL_674550, EPI_ISL_674558, EPI_ISL_674563, EPI_ISL_674569, EPI_ISL_674585, EPI_ISL_674589 to 674590, EPI_ISL_674595, EPI_ISL_674603, EPI_ISL_674605, EPI_ISL_674609, EPI_ISL_674613, EPI_ISL_674625, EPI_ISL_674635, EPI_ISL_674652, EPI_ISL_674655, EPI_ISL_674657 to 674658, EPI_ISL_674661 to 674662, EPI_ISL_674667, EPI_ISL_674678, EPI_ISL_674685, EPI_ISL_674690 to 674692, EPI_ISL_674698, EPI_ISL_674707, EPI_ISL_674714, EPI_ISL_674716 to 674717, EPI_ISL_674723 to 674725, EPI_ISL_674736, EPI_ISL_674743 to 674747, EPI_ISL_674749, EPI_ISL_674751, EPI_ISL_674756, EPI_ISL_674762 to 674763, EPI_ISL_674772 to 674773, EPI_ISL_674779, EPI_ISL_674781, EPI_ISL_674783, EPI_ISL_674786, EPI_ISL_674791 to 674792, EPI_ISL_674794, EPI_ISL_674808 to 674809, EPI_ISL_676314 to 676317, EPI_ISL_676319 to 676323, EPI_ISL_676326, EPI_ISL_676328 to 676333, EPI_ISL_676335, EPI_ISL_676337 to 676346, EPI_ISL_676348 to 676350, EPI_ISL_676353 to 676357, EPI_ISL_676359 to 676360, EPI_ISL_676362 to 676366, EPI_ISL_676368 to 676372, EPI_ISL_676374 to 676382, EPI_ISL_676384 to 676406, EPI_ISL_676409 to 676413, EPI_ISL_676415 to 676430, EPI_ISL_676432, EPI_ISL_676434 to 676440, EPI_ISL_676442 to 676444, EPI_ISL_676446 to 676449, EPI_ISL_676451 to 676453, EPI_ISL_676455 to 676457, EPI_ISL_676459 to 676462, EPI_ISL_676464 to 676483, EPI_ISL_676485 to 676488, EPI_ISL_703262 to 703263, EPI_ISL_703270, EPI_ISL_703280, EPI_ISL_703285 to 703286, EPI_ISL_703290 to 703291, EPI_ISL_703296, EPI_ISL_703300, EPI_ISL_703304, EPI_ISL_703309, EPI_ISL_703320, EPI_ISL_703323, EPI_ISL_703325, EPI_ISL_703331, EPI_ISL_703337, EPI_ISL_703345, EPI_ISL_703349 to 703350, EPI_ISL_703352, EPI_ISL_703355, EPI_ISL_703370 to 703371, EPI_ISL_703378 to 703379, EPI_ISL_703381 to 703382,

EPI_ISL_703384, EPI_ISL_703389, EPI_ISL_703391, EPI_ISL_703393, EPI_ISL_703399, EPI_ISL_703401, EPI_ISL_703407 to 703408, EPI_ISL_703410 to 703411, EPI_ISL_703415, EPI_ISL_703422, EPI_ISL_703426, EPI_ISL_703428 to 703429, EPI_ISL_703431, EPI_ISL_703439, EPI_ISL_703442, EPI_ISL_703444 to
703445, EPI_ISL_703447, EPI_ISL_703453, EPI_ISL_703456, EPI_ISL_703458, EPI_ISL_703463, EPI_ISL_703472, EPI_ISL_703474, EPI_ISL_703482, EPI_ISL_703491, EPI_ISL_703496, EPI_ISL_703499, EPI_ISL_703501, EPI_ISL_703506, EPI_ISL_703510, EPI_ISL_703514 to 703515, EPI_ISL_703517, EPI_ISL_703522,
EPI_ISL_703525, EPI_ISL_703529, EPI_ISL_703534, EPI_ISL_703538, EPI_ISL_703541, EPI_ISL_703546, EPI_ISL_703553, EPI_ISL_703557, EPI_ISL_703560 to 703561, EPI_ISL_703578, EPI_ISL_703583 to 703584, EPI_ISL_703598, EPI_ISL_703600, EPI_ISL_703608, EPI_ISL_703612, EPI_ISL_703617, EPI_ISL_703626,
EPI_ISL_703631, EPI_ISL_703633 to 703634, EPI_ISL_703640 to 703641, EPI_ISL_703645, EPI_ISL_703650 to 703651, EPI_ISL_703657, EPI_ISL_703659, EPI_ISL_703661, EPI_ISL_703672 to 703673, EPI_ISL_703677, EPI_ISL_703685, EPI_ISL_703694, EPI_ISL_703700, EPI_ISL_703703, EPI_ISL_703786,
EPI_ISL_703790, EPI_ISL_703799, EPI_ISL_703801, EPI_ISL_703808, EPI_ISL_703816, EPI_ISL_703819 to 703820, EPI_ISL_703822, EPI_ISL_703824 to 703825, EPI_ISL_703827, EPI_ISL_703830, EPI_ISL_703835 to 703836, EPI_ISL_703838, EPI_ISL_703840 to 703841, EPI_ISL_703843, EPI_ISL_703849,
EPI_ISL_703851 to 703852, EPI_ISL_703854, EPI_ISL_703857, EPI_ISL_703861 to 703862, EPI_ISL_703864, EPI_ISL_703866, EPI_ISL_703869, EPI_ISL_703877 to 703878, EPI_ISL_703880, EPI_ISL_703882 to 703883, EPI_ISL_703885, EPI_ISL_703887, EPI_ISL_703889 to 703890, EPI_ISL_703893, EPI_ISL_703895,
EPI_ISL_703900 to 703901, EPI_ISL_703904, EPI_ISL_703906 to 703907, EPI_ISL_703909 to 703910, EPI_ISL_703912, EPI_ISL_703917, EPI_ISL_703919, EPI_ISL_703925, EPI_ISL_703928, EPI_ISL_703930 to 703931, EPI_ISL_703934, EPI_ISL_703936, EPI_ISL_703938 to 703939, EPI_ISL_703941, EPI_ISL_703943 to
703944, EPI_ISL_703949, EPI_ISL_703951, EPI_ISL_703956 to 703957, EPI_ISL_703959 to 703960, EPI_ISL_703962, EPI_ISL_703964 to 703965, EPI_ISL_703967, EPI_ISL_703972 to 703973, EPI_ISL_703975 to 703976, EPI_ISL_703978, EPI_ISL_703980 to 703981, EPI_ISL_703983 to 703984, EPI_ISL_703986,
EPI_ISL_703988, EPI_ISL_703991, EPI_ISL_703993 to 703994, EPI_ISL_703996 to 703997, EPI_ISL_703999 to 704000, EPI_ISL_704002, EPI_ISL_704004 to 704005, EPI_ISL_704007 to 704008, EPI_ISL_704010, EPI_ISL_704013, EPI_ISL_704015 to 704016, EPI_ISL_704018 to 704019, EPI_ISL_704023 to 704024,
EPI_ISL_704026 to 704027, EPI_ISL_704029, EPI_ISL_704031 to 704032, EPI_ISL_704034 to 704035, EPI_ISL_704037, EPI_ISL_704040, EPI_ISL_704042, EPI_ISL_704045, EPI_ISL_704047 to 704048, EPI_ISL_704051, EPI_ISL_704053, EPI_ISL_704055 to 704056, EPI_ISL_704058 to 704059, EPI_ISL_704061,
EPI_ISL_704063 to 704064, EPI_ISL_704066 to 704067, EPI_ISL_704071 to 704072, EPI_ISL_704077 to 704078, EPI_ISL_704080, EPI_ISL_704082 to 704083, EPI_ISL_704085, EPI_ISL_704088, EPI_ISL_704090 to 704091, EPI_ISL_704093 to 704094, EPI_ISL_704096, EPI_ISL_704098 to 704099, EPI_ISL_704103 to 704104,
EPI_ISL_704106 to 704107, EPI_ISL_704109 to 704110, EPI_ISL_704114, EPI_ISL_704116 to 704117, EPI_ISL_704121 to 704122, EPI_ISL_704124 to 704125, EPI_ISL_704127 to 704128, EPI_ISL_704130, EPI_ISL_704133, EPI_ISL_704135 to 704136, EPI_ISL_704138, EPI_ISL_704140, EPI_ISL_704143 to 704144,
EPI_ISL_704146, EPI_ISL_704151 to 704152, EPI_ISL_704154, EPI_ISL_704157, EPI_ISL_704159 to 704160, EPI_ISL_704162, EPI_ISL_704164 to 704165, EPI_ISL_704167, EPI_ISL_704169 to 704170, EPI_ISL_704172 to 704173, EPI_ISL_704177 to 704178, EPI_ISL_704180, EPI_ISL_704182 to 704183, EPI_ISL_704185 to
704186, EPI_ISL_704188 to 704189, EPI_ISL_704191 to 704192, EPI_ISL_704197, EPI_ISL_704199 to 704200, EPI_ISL_704202, EPI_ISL_704204 to 704205, EPI_ISL_704208, EPI_ISL_704210 to 704216, EPI_ISL_704220 to 704221, EPI_ISL_704223 to 704224, EPI_ISL_704226 to 704227, EPI_ISL_704229, EPI_ISL_704232,
EPI_ISL_704234 to 704235, EPI_ISL_704240, EPI_ISL_704242, EPI_ISL_704244 to 704245, EPI_ISL_704248 to 704249, EPI_ISL_704251, EPI_ISL_704253 to 704254, EPI_ISL_704261, EPI_ISL_704263 to 704264, EPI_ISL_704268, EPI_ISL_704272, EPI_ISL_704274 to 704275, EPI_ISL_704278 to 704279, EPI_ISL_704281,
EPI_ISL_704284, EPI_ISL_704287 to 704288, EPI_ISL_704290, EPI_ISL_704293, EPI_ISL_704295 to 704296, EPI_ISL_704298, EPI_ISL_704300, EPI_ISL_704303 to 704304, EPI_ISL_704306, EPI_ISL_704310, EPI_ISL_704313 to 704314, EPI_ISL_704316, EPI_ISL_704318 to 704319, EPI_ISL_704321, EPI_ISL_704323 to
704324, EPI_ISL_704326 to 704327, EPI_ISL_704330, EPI_ISL_704335, EPI_ISL_704337 to 704338, EPI_ISL_704340, EPI_ISL_704342 to 704343, EPI_ISL_704345 to 704346, EPI_ISL_704348 to 704349, EPI_ISL_704351 to 704352, EPI_ISL_704355, EPI_ISL_704357 to 704358, EPI_ISL_704360, EPI_ISL_704362 to 704363,
EPI_ISL_704365, EPI_ISL_704368, EPI_ISL_704370, EPI_ISL_704376, EPI_ISL_704380 to 704381, EPI_ISL_704383, EPI_ISL_704386, EPI_ISL_704388, EPI_ISL_704391, EPI_ISL_704394, EPI_ISL_704396, EPI_ISL_704399 to 704400, EPI_ISL_704402, EPI_ISL_704404 to 704405, EPI_ISL_704407, EPI_ISL_704412,
EPI_ISL_704415, EPI_ISL_704420 to 704421, EPI_ISL_704423 to 704424, EPI_ISL_704426, EPI_ISL_704428, EPI_ISL_704431, EPI_ISL_704434, EPI_ISL_704440, EPI_ISL_704442, EPI_ISL_704444 to 704445, EPI_ISL_704447 to 704448, EPI_ISL_704450 to 704451, EPI_ISL_704453, EPI_ISL_704455 to 704456,
EPI_ISL_704458, EPI_ISL_704461, EPI_ISL_704463 to 704464, EPI_ISL_704466, EPI_ISL_704468 to 704469, EPI_ISL_704471, EPI_ISL_704474, EPI_ISL_704476 to 704477, EPI_ISL_704479 to 704480, EPI_ISL_704482 to 704483, EPI_ISL_704485, EPI_ISL_704487 to 704488, EPI_ISL_704491, EPI_ISL_704493,
EPI_ISL_704495 to 704496, EPI_ISL_704498, EPI_ISL_704500 to 704501, EPI_ISL_704504, EPI_ISL_704506, EPI_ISL_704508, EPI_ISL_704511 to 704512, EPI_ISL_704514, EPI_ISL_704523, EPI_ISL_704525, EPI_ISL_704527, EPI_ISL_704530 to 704531, EPI_ISL_704533, EPI_ISL_704536, EPI_ISL_704538 to 704539,
EPI_ISL_704541 to 704542, EPI_ISL_704544, EPI_ISL_704547, EPI_ISL_704549 to 704550, EPI_ISL_704552, EPI_ISL_704556, EPI_ISL_704558, EPI_ISL_704560 to 704561, EPI_ISL_704565 to 704566, EPI_ISL_704568 to 704569, EPI_ISL_704571, EPI_ISL_704642, EPI_ISL_704644 to 704645, EPI_ISL_704647,
EPI_ISL_704649, EPI_ISL_704652, EPI_ISL_704655, EPI_ISL_704657 to 704658, EPI_ISL_704663, EPI_ISL_704665, EPI_ISL_704667, EPI_ISL_704670, EPI_ISL_704672 to 704673, EPI_ISL_704675, EPI_ISL_704678, EPI_ISL_704680 to 704681, EPI_ISL_704684, EPI_ISL_704686, EPI_ISL_708855, EPI_ISL_708884,
EPI_ISL_708890, EPI_ISL_708905 to 708906, EPI_ISL_708910, EPI_ISL_708922, EPI_ISL_708933, EPI_ISL_708953, EPI_ISL_708957, EPI_ISL_708985, EPI_ISL_709005, EPI_ISL_709009, EPI_ISL_709013, EPI_ISL_709022, EPI_ISL_709036, EPI_ISL_709040, EPI_ISL_709050, EPI_ISL_709056, EPI_ISL_709058,
EPI_ISL_709060, EPI_ISL_709077 to 709079, EPI_ISL_709082, EPI_ISL_709091, EPI_ISL_709107 to 709108, EPI_ISL_709119, EPI_ISL_709131 to 709133, EPI_ISL_709135, EPI_ISL_709139 to 709142, EPI_ISL_709144, EPI_ISL_709150 to 709151, EPI_ISL_709159 to 709161, EPI_ISL_709170 to 709171, EPI_ISL_709174,
EPI_ISL_709189 to 709191, EPI_ISL_709199, EPI_ISL_709201, EPI_ISL_709215, EPI_ISL_709231, EPI_ISL_709234, EPI_ISL_709236, EPI_ISL_709239, EPI_ISL_709242 to 709243, EPI_ISL_709248, EPI_ISL_709264, EPI_ISL_709274, EPI_ISL_709277, EPI_ISL_709283, EPI_ISL_709285, EPI_ISL_709288, EPI_ISL_709290 to
709292, EPI_ISL_709301, EPI_ISL_709311 to 709312, EPI_ISL_709320, EPI_ISL_709324, EPI_ISL_709327, EPI_ISL_709332, EPI_ISL_709334, EPI_ISL_709336, EPI_ISL_709343, EPI_ISL_709345, EPI_ISL_709353, EPI_ISL_709358 to 709360, EPI_ISL_709364 to 709365, EPI_ISL_709367, EPI_ISL_709373, EPI_ISL_709376,
EPI_ISL_709380, EPI_ISL_709383 to 709384, EPI_ISL_709392, EPI_ISL_709395 to 709396, EPI_ISL_709399, EPI_ISL_709403 to 709404, EPI_ISL_709406 to 709407, EPI_ISL_709409, EPI_ISL_709417, EPI_ISL_709419, EPI_ISL_709423 to 709424, EPI_ISL_709426, EPI_ISL_709436, EPI_ISL_709438 to 709441,
EPI_ISL_709443, EPI_ISL_709446, EPI_ISL_709450, EPI_ISL_709453 to 709454, EPI_ISL_709456 to 709457, EPI_ISL_709464 to 709466, EPI_ISL_709469, EPI_ISL_709472, EPI_ISL_709474, EPI_ISL_709483, EPI_ISL_709486 to 709487, EPI_ISL_709490, EPI_ISL_709494, EPI_ISL_709496 to 709498, EPI_ISL_709500,
EPI_ISL_709502 to 709503, EPI_ISL_709509 to 709510, EPI_ISL_709517 to 709518, EPI_ISL_709522, EPI_ISL_709524, EPI_ISL_709527, EPI_ISL_709530, EPI_ISL_709533, EPI_ISL_709537, EPI_ISL_709539, EPI_ISL_709541, EPI_ISL_709549, EPI_ISL_709555, EPI_ISL_709557 to 709558, EPI_ISL_709561,
EPI_ISL_709565, EPI_ISL_709800, EPI_ISL_709806, EPI_ISL_709809, EPI_ISL_709811 to 709812, EPI_ISL_709816, EPI_ISL_709818, EPI_ISL_709820, EPI_ISL_709826, EPI_ISL_709831 to 709832, EPI_ISL_709834 to 709835, EPI_ISL_709840, EPI_ISL_709845, EPI_ISL_709848 to 709849, EPI_ISL_709851,
EPI_ISL_709855, EPI_ISL_709857 to 709859, EPI_ISL_709861, EPI_ISL_709875, EPI_ISL_710615, EPI_ISL_710619, EPI_ISL_710621 to 710623, EPI_ISL_710627, EPI_ISL_710629, EPI_ISL_710633, EPI_ISL_710635, EPI_ISL_710637, EPI_ISL_710640 to 710644, EPI_ISL_710646, EPI_ISL_710650 to 710652,
EPI_ISL_710654 to 710655, EPI_ISL_710657 to 710660, EPI_ISL_710664, EPI_ISL_710666 to 710670, EPI_ISL_710672 to 710675, EPI_ISL_710677 to 710680, EPI_ISL_710683 to 710688, EPI_ISL_710690 to 710691, EPI_ISL_710696 to 710697, EPI_ISL_710748, EPI_ISL_710753 to 710755, EPI_ISL_710757 to 710766,
EPI_ISL_710768, EPI_ISL_710770 to 710772, EPI_ISL_710774 to 710790, EPI_ISL_710792 to 710800, EPI_ISL_710802 to 710805, EPI_ISL_710807 to 710815, EPI_ISL_710818 to 710820, EPI_ISL_710831 to 710832, EPI_ISL_710834, EPI_ISL_710838 to 710841, EPI_ISL_710843 to 710864, EPI_ISL_710867 to 710868,
EPI_ISL_710872, EPI_ISL_710877, EPI_ISL_710879 to 710886, EPI_ISL_710903 to 710906, EPI_ISL_710995 to 710999, EPI_ISL_711001 to 711007, EPI_ISL_711014, EPI_ISL_711049, EPI_ISL_711052 to 711054, EPI_ISL_719605 to 719613, EPI_ISL_719617 to 719626, EPI_ISL_719629 to 719631, EPI_ISL_719633 to 719636,
EPI_ISL_719640 to 719644, EPI_ISL_719646, EPI_ISL_719648, EPI_ISL_719650 to 719653, EPI_ISL_719655 to 719658, EPI_ISL_719660 to 719661, EPI_ISL_719663 to 719666, EPI_ISL_719668 to 719673, EPI_ISL_719677 to 719681, EPI_ISL_719683 to 719688, EPI_ISL_719691 to 719694, EPI_ISL_719698 to 719703,
EPI_ISL_719705, EPI_ISL_719707 to 719710, EPI_ISL_719712 to 719717, EPI_ISL_719720, EPI_ISL_719722 to 719740, EPI_ISL_719742, EPI_ISL_719744, EPI_ISL_719748 to 719750, EPI_ISL_719752 to 719753, EPI_ISL_719755 to 719756, EPI_ISL_719758 to 719761, EPI_ISL_719763 to 719764, EPI_ISL_719766,
EPI_ISL_719768 to 719775, EPI_ISL_719777, EPI_ISL_719780 to 719783, EPI_ISL_719786 to 719793, EPI_ISL_719796, EPI_ISL_719798, EPI_ISL_719800, EPI_ISL_719802 to 719815, EPI_ISL_719817 to 719819, EPI_ISL_719821 to 719823, EPI_ISL_719829, EPI_ISL_719834, EPI_ISL_719839, EPI_ISL_719841,
EPI_ISL_719846 to 719850, EPI_ISL_719852, EPI_ISL_719856 to 719858, EPI_ISL_719861 to 719862, EPI_ISL_719864 to 719868, EPI_ISL_719870 to 719873, EPI_ISL_719875 to 719878, EPI_ISL_719880 to 719881, EPI_ISL_719883 to 719885, EPI_ISL_719887, EPI_ISL_719889 to 719892, EPI_ISL_719894, EPI_ISL_719896,
EPI_ISL_719898, EPI_ISL_719900 to 719902, EPI_ISL_719904, EPI_ISL_719907 to 719918, EPI_ISL_719920 to 719925, EPI_ISL_719927 to 719931, EPI_ISL_719934, EPI_ISL_719938, EPI_ISL_719940 to 719941, EPI_ISL_719944, EPI_ISL_719946 to 719947, EPI_ISL_719951, EPI_ISL_719953, EPI_ISL_719956 to 719959,
EPI_ISL_719961, EPI_ISL_719963 to 719966, EPI_ISL_719969, EPI_ISL_719972, EPI_ISL_719974 to 719976, EPI_ISL_719978 to 719981, EPI_ISL_719983 to 719986, EPI_ISL_720668, EPI_ISL_720672, EPI_ISL_720704, EPI_ISL_720726, EPI_ISL_720728, EPI_ISL_720732 to 720733, EPI_ISL_720738, EPI_ISL_720740,
EPI_ISL_720752, EPI_ISL_720755, EPI_ISL_720761, EPI_ISL_720767, EPI_ISL_720776, EPI_ISL_720787, EPI_ISL_720792, EPI_ISL_720805, EPI_ISL_720811, EPI_ISL_720819, EPI_ISL_720831, EPI_ISL_720834, EPI_ISL_720841, EPI_ISL_720850, EPI_ISL_720870, EPI_ISL_720877, EPI_ISL_720889, EPI_ISL_720897,
EPI_ISL_720904, EPI_ISL_720909 to 720915, EPI_ISL_720917 to 720922, EPI_ISL_720924, EPI_ISL_720926 to 720927, EPI_ISL_720929 to 720934, EPI_ISL_720936 to 720946, EPI_ISL_720948 to 720950, EPI_ISL_720952 to 720953, EPI_ISL_720955, EPI_ISL_720957, EPI_ISL_720959 to 720967, EPI_ISL_720969 to 720972,
EPI_ISL_720974 to 720986, EPI_ISL_720988, EPI_ISL_720990 to 720991, EPI_ISL_720993 to 720996, EPI_ISL_720998, EPI_ISL_721000 to 721008, EPI_ISL_721010 to 721031, EPI_ISL_721033 to 721036, EPI_ISL_721039 to 721043, EPI_ISL_721045 to 721060, EPI_ISL_721062 to 721083, EPI_ISL_721085 to 721110,
EPI_ISL_721112 to 721115, EPI_ISL_721119 to 721134, EPI_ISL_721136 to 721142, EPI_ISL_721144 to 721155, EPI_ISL_721157, EPI_ISL_721159, EPI_ISL_721161 to 721166, EPI_ISL_721168 to 721169, EPI_ISL_721171 to 721172, EPI_ISL_721174 to 721184, EPI_ISL_721519, EPI_ISL_721521, EPI_ISL_721533 to 721538,
EPI_ISL_721540 to 721541, EPI_ISL_721543 to 721544, EPI_ISL_721548 to 721549, EPI_ISL_721551 to 721552, EPI_ISL_721554, EPI_ISL_721558 to 721561, EPI_ISL_721563 to 721567, EPI_ISL_728300, EPI_ISL_730656 to 730657, EPI_ISL_730659, EPI_ISL_730664, EPI_ISL_730673, EPI_ISL_730676, EPI_ISL_730678,
EPI_ISL_730697, EPI_ISL_730705, EPI_ISL_730710, EPI_ISL_730718, EPI_ISL_730723, EPI_ISL_730737, EPI_ISL_730759, EPI_ISL_730765, EPI_ISL_730777, EPI_ISL_730780, EPI_ISL_730785, EPI_ISL_730793, EPI_ISL_730795 to 730796, EPI_ISL_730802 to 730804, EPI_ISL_730808 to 730809, EPI_ISL_730813,
EPI_ISL_730815, EPI_ISL_730828, EPI_ISL_730831, EPI_ISL_730846, EPI_ISL_730849 to 730850, EPI_ISL_730858, EPI_ISL_730860, EPI_ISL_730862, EPI_ISL_730864, EPI_ISL_730867, EPI_ISL_730869, EPI_ISL_730875, EPI_ISL_730887, EPI_ISL_730894, EPI_ISL_730910 to 730911, EPI_ISL_730913, EPI_ISL_731256 to
731257, EPI_ISL_731259, EPI_ISL_731261 to 731262, EPI_ISL_731264, EPI_ISL_731267, EPI_ISL_731273, EPI_ISL_731280 to 731281, EPI_ISL_731284, EPI_ISL_731286 to 731287, EPI_ISL_731291, EPI_ISL_731295, EPI_ISL_731299, EPI_ISL_731305, EPI_ISL_731308 to 731309, EPI_ISL_731314, EPI_ISL_731319,
EPI_ISL_731321, EPI_ISL_731326, EPI_ISL_731333 to 731334, EPI_ISL_731341, EPI_ISL_731345 to 731346, EPI_ISL_731349, EPI_ISL_731353 to 731354, EPI_ISL_731367 to 731368, EPI_ISL_731378 to 731380, EPI_ISL_731382 to 731383, EPI_ISL_731386, EPI_ISL_731390, EPI_ISL_731392 to 731393, EPI_ISL_731398,
EPI_ISL_731400, EPI_ISL_731402 to 731404, EPI_ISL_731406, EPI_ISL_731411, EPI_ISL_731414, EPI_ISL_731417, EPI_ISL_731420 to 731421, EPI_ISL_731426, EPI_ISL_731431 to 731432, EPI_ISL_731435, EPI_ISL_731444, EPI_ISL_731447, EPI_ISL_731449, EPI_ISL_731452 to 731453, EPI_ISL_731458 to 731459,
EPI_ISL_731466, EPI_ISL_731477, EPI_ISL_731479, EPI_ISL_731484 to 731486, EPI_ISL_731488, EPI_ISL_731491, EPI_ISL_731493, EPI_ISL_731496, EPI_ISL_731499, EPI_ISL_731501, EPI_ISL_731507 to 731508, EPI_ISL_731517, EPI_ISL_731536, EPI_ISL_731544, EPI_ISL_731548, EPI_ISL_731550 to 731551,
EPI_ISL_731894, EPI_ISL_733916, EPI_ISL_733918 to 733920, EPI_ISL_733922 to 733932, EPI_ISL_733934 to 733935, EPI_ISL_733937, EPI_ISL_733939 to 733941, EPI_ISL_733943 to 733944, EPI_ISL_733946, EPI_ISL_733948, EPI_ISL_733950 to 733951, EPI_ISL_733953 to 733957, EPI_ISL_733959, EPI_ISL_733961 to
733962, EPI_ISL_733964, EPI_ISL_733967 to 733976, EPI_ISL_733978 to 733981, EPI_ISL_733983 to 733991, EPI_ISL_733996 to 734002, EPI_ISL_734005 to 734006, EPI_ISL_734009, EPI_ISL_734011 to 734012, EPI_ISL_734014 to 734015, EPI_ISL_734017 to 734025, EPI_ISL_734027, EPI_ISL_734029, EPI_ISL_734031 to
734040, EPI_ISL_734044 to 734047, EPI_ISL_734050, EPI_ISL_734052 to 734054, EPI_ISL_734056 to 734062, EPI_ISL_734065, EPI_ISL_734068, EPI_ISL_734070 to 734081, EPI_ISL_734083 to 734084, EPI_ISL_734086 to 734089, EPI_ISL_734094 to 734099, EPI_ISL_734101 to 734106, EPI_ISL_734108, EPI_ISL_734110,
EPI_ISL_734112, EPI_ISL_734114 to 734118, EPI_ISL_734120 to 734125, EPI_ISL_734127, EPI_ISL_734129 to 734131, EPI_ISL_734133 to 734142, EPI_ISL_734145 to 734147, EPI_ISL_734149, EPI_ISL_734151 to 734152, EPI_ISL_734154 to 734159, EPI_ISL_734161 to 734162, EPI_ISL_735861, EPI_ISL_735894,
EPI_ISL_735909, EPI_ISL_735920, EPI_ISL_735939, EPI_ISL_735952, EPI_ISL_735956, EPI_ISL_735961, EPI_ISL_735972, EPI_ISL_735978, EPI_ISL_735987, EPI_ISL_735993, EPI_ISL_735995, EPI_ISL_736003, EPI_ISL_736007, EPI_ISL_736015, EPI_ISL_736018, EPI_ISL_736024, EPI_ISL_736038, EPI_ISL_736041,
EPI_ISL_736057, EPI_ISL_736059, EPI_ISL_736066, EPI_ISL_736072, EPI_ISL_736090, EPI_ISL_736099, EPI_ISL_736101, EPI_ISL_736107, EPI_ISL_736130 to 736132, EPI_ISL_736162, EPI_ISL_736168, EPI_ISL_736182 to 736184, EPI_ISL_736209, EPI_ISL_736212, EPI_ISL_736221, EPI_ISL_736223, EPI_ISL_736231,
EPI_ISL_736235 to 736236, EPI_ISL_736238, EPI_ISL_736242 to 736243, EPI_ISL_736250, EPI_ISL_736255, EPI_ISL_736257, EPI_ISL_736261, EPI_ISL_736266, EPI_ISL_736275, EPI_ISL_736277, EPI_ISL_736290, EPI_ISL_736304, EPI_ISL_736309, EPI_ISL_736312 to 736313, EPI_ISL_736315 to 736316,
EPI_ISL_736321, EPI_ISL_736325, EPI_ISL_736330 to 736332, EPI_ISL_736334, EPI_ISL_736347, EPI_ISL_736352, EPI_ISL_736355, EPI_ISL_736367, EPI_ISL_736370 to 736371, EPI_ISL_736373, EPI_ISL_736376, EPI_ISL_736378, EPI_ISL_736388 to 736391, EPI_ISL_736394, EPI_ISL_736398, EPI_ISL_736404,
EPI_ISL_736414, EPI_ISL_736416, EPI_ISL_736423, EPI_ISL_736426, EPI_ISL_736431, EPI_ISL_736437 to 736438, EPI_ISL_736442, EPI_ISL_736444, EPI_ISL_736455 to 736456, EPI_ISL_736461, EPI_ISL_736464, EPI_ISL_736470 to 736471, EPI_ISL_736478, EPI_ISL_736488, EPI_ISL_736492, EPI_ISL_736532 to
736533, EPI_ISL_736567, EPI_ISL_736580, EPI_ISL_736582, EPI_ISL_736605, EPI_ISL_736624, EPI_ISL_736627 to 736628, EPI_ISL_736631, EPI_ISL_736646, EPI_ISL_736650, EPI_ISL_736657, EPI_ISL_736672, EPI_ISL_736678, EPI_ISL_736695, EPI_ISL_736700, EPI_ISL_736721, EPI_ISL_736745, EPI_ISL_736752,
EPI_ISL_736754, EPI_ISL_736774, EPI_ISL_756690, EPI_ISL_756695 to 756696, EPI_ISL_756702, EPI_ISL_756705 to 756706, EPI_ISL_756709, EPI_ISL_756716, EPI_ISL_756723, EPI_ISL_756729, EPI_ISL_756737, EPI_ISL_756744 to 756745, EPI_ISL_756748, EPI_ISL_756750, EPI_ISL_756761, EPI_ISL_756773,
EPI_ISL_756775, EPI_ISL_756780, EPI_ISL_756784, EPI_ISL_756795, EPI_ISL_756800, EPI_ISL_756803, EPI_ISL_756805, EPI_ISL_756824, EPI_ISL_756835, EPI_ISL_756840, EPI_ISL_756849 to 756850, EPI_ISL_756852, EPI_ISL_756861, EPI_ISL_756877 to 756879, EPI_ISL_756885 to 756886, EPI_ISL_756888 to
756889, EPI_ISL_756893 to 756895, EPI_ISL_756897, EPI_ISL_756900, EPI_ISL_756909 to 756910, EPI_ISL_756912, EPI_ISL_756920, EPI_ISL_756923, EPI_ISL_756931, EPI_ISL_756940, EPI_ISL_756947, EPI_ISL_756951, EPI_ISL_756953, EPI_ISL_756955, EPI_ISL_756959, EPI_ISL_756970 to 756971, EPI_ISL_756973,
EPI_ISL_756977, EPI_ISL_756982, EPI_ISL_756998, EPI_ISL_757005, EPI_ISL_757007 to 757009, EPI_ISL_757011 to 757012, EPI_ISL_757021, EPI_ISL_757027 to 757028, EPI_ISL_757032, EPI_ISL_757034 to 757038, EPI_ISL_757040 to 757053, EPI_ISL_757055, EPI_ISL_757057 to 757070, EPI_ISL_757073 to 757077,
EPI_ISL_757079 to 757083, EPI_ISL_757085, EPI_ISL_757087 to 757089, EPI_ISL_757091 to 757093, EPI_ISL_757096 to 757097, EPI_ISL_757099 to 757102, EPI_ISL_757104 to 757105, EPI_ISL_757107 to 757108, EPI_ISL_757111, EPI_ISL_757115 to 757118, EPI_ISL_757120 to 757182, EPI_ISL_757184, EPI_ISL_757186
to 757190, EPI_ISL_757192 to 757198, EPI_ISL_757200 to 757202, EPI_ISL_757204, EPI_ISL_757207 to 757209, EPI_ISL_757211 to 757214, EPI_ISL_757217 to 757252, EPI_ISL_757254 to 757256, EPI_ISL_757258 to 757284, EPI_ISL_760250 to 760258, EPI_ISL_760260, EPI_ISL_760262, EPI_ISL_760264, EPI_ISL_760269,
EPI_ISL_760271 to 760272, EPI_ISL_760274 to 760275, EPI_ISL_760277, EPI_ISL_760279 to 760280, EPI_ISL_760282 to 760283, EPI_ISL_760287, EPI_ISL_760289, EPI_ISL_760291, EPI_ISL_760294, EPI_ISL_760299 to 760300, EPI_ISL_760302 to 760304, EPI_ISL_760307 to 760309, EPI_ISL_760313 to 760317,
EPI_ISL_760319 to 760320, EPI_ISL_760322 to 760325, EPI_ISL_760328 to 760329, EPI_ISL_760331 to 760332, EPI_ISL_760334 to 760340, EPI_ISL_760342 to 760343, EPI_ISL_760345, EPI_ISL_760347 to 760349, EPI_ISL_760351 to 760353, EPI_ISL_760356 to 760357, EPI_ISL_760361 to 760362, EPI_ISL_760365 to
760368, EPI_ISL_760373 to 760376, EPI_ISL_760378 to 760380, EPI_ISL_760383, EPI_ISL_760393 to 760395, EPI_ISL_760401 to 760404, EPI_ISL_760406 to 760407, EPI_ISL_760409 to 760411, EPI_ISL_760413 to 760414, EPI_ISL_760417, EPI_ISL_760422 to 760423, EPI_ISL_760426 to 760429, EPI_ISL_760431,
EPI_ISL_760433, EPI_ISL_760435, EPI_ISL_760437 to 760440, EPI_ISL_760443 to 760444, EPI_ISL_760446 to 760447, EPI_ISL_760449, EPI_ISL_760452 to 760456, EPI_ISL_760458 to 760459, EPI_ISL_760462 to 760470, EPI_ISL_760472 to 760473, EPI_ISL_760476 to 760478, EPI_ISL_760480 to 760484, EPI_ISL_760487
to 760490, EPI_ISL_760492 to 760494, EPI_ISL_760496 to 760497, EPI_ISL_760499, EPI_ISL_760502 to 760507, EPI_ISL_760510 to 760511, EPI_ISL_760513 to 760515, EPI_ISL_760517 to 760520, EPI_ISL_760522 to 760523, EPI_ISL_760525 to 760527, EPI_ISL_760529, EPI_ISL_760532 to 760533, EPI_ISL_760536 to
760537, EPI_ISL_760539 to 760542, EPI_ISL_760547 to 760548, EPI_ISL_760553, EPI_ISL_760560, EPI_ISL_760563, EPI_ISL_760566, EPI_ISL_760569, EPI_ISL_760571, EPI_ISL_760575 to 760581, EPI_ISL_760583 to 760585, EPI_ISL_760590, EPI_ISL_760592, EPI_ISL_760594, EPI_ISL_760598, EPI_ISL_760602,
EPI_ISL_760604, EPI_ISL_760607, EPI_ISL_760610 to 760611, EPI_ISL_760614, EPI_ISL_760616, EPI_ISL_760618 to 760619, EPI_ISL_760621 to 760622, EPI_ISL_760626, EPI_ISL_760629, EPI_ISL_760632, EPI_ISL_760634 to 760636, EPI_ISL_760638, EPI_ISL_760641, EPI_ISL_760643, EPI_ISL_760645 to 760646,
EPI_ISL_760648, EPI_ISL_760651, EPI_ISL_760655, EPI_ISL_760657, EPI_ISL_760659, EPI_ISL_760662, EPI_ISL_760668 to 760669, EPI_ISL_760671, EPI_ISL_760673 to 760676, EPI_ISL_760681 to 760683, EPI_ISL_760686 to 760687, EPI_ISL_760689 to 760690, EPI_ISL_760692, EPI_ISL_760694 to 760696,
EPI_ISL_760700, EPI_ISL_760704, EPI_ISL_760706 to 760707, EPI_ISL_760712 to 760713, EPI_ISL_760716 to 760717, EPI_ISL_760720, EPI_ISL_760729 to 760730, EPI_ISL_760732, EPI_ISL_760735, EPI_ISL_760741 to 760744, EPI_ISL_760746, EPI_ISL_760749 to 760751, EPI_ISL_760754, EPI_ISL_760757 to 760758,
EPI_ISL_760760, EPI_ISL_760764 to 760768, EPI_ISL_760772 to 760774, EPI_ISL_760777, EPI_ISL_760779, EPI_ISL_760781 to 760782, EPI_ISL_760787, EPI_ISL_760791, EPI_ISL_760793, EPI_ISL_760797 to 760798, EPI_ISL_760800, EPI_ISL_760803, EPI_ISL_760805, EPI_ISL_760811, EPI_ISL_760815 to 760816,
EPI_ISL_760818 to 760820, EPI_ISL_760823 to 760824, EPI_ISL_760828 to 760829, EPI_ISL_760831, EPI_ISL_760834 to 760836, EPI_ISL_760840 to 760841, EPI_ISL_760843, EPI_ISL_760845, EPI_ISL_760847 to 760851, EPI_ISL_760854 to 760856, EPI_ISL_760866, EPI_ISL_760869 to 760870, EPI_ISL_760872,
EPI_ISL_760875, EPI_ISL_760877, EPI_ISL_760879 to 760880, EPI_ISL_760882, EPI_ISL_760885 to 760886, EPI_ISL_760893, EPI_ISL_760895, EPI_ISL_760899, EPI_ISL_760901 to 760902, EPI_ISL_760907, EPI_ISL_760909 to 760911, EPI_ISL_760913 to 760914, EPI_ISL_760919, EPI_ISL_760926 to 760927,
EPI_ISL_760930, EPI_ISL_760934 to 760935, EPI_ISL_760944, EPI_ISL_760946 to 760947, EPI_ISL_760958, EPI_ISL_760964 to 760966, EPI_ISL_760970, EPI_ISL_760972 to 760974, EPI_ISL_760981, EPI_ISL_760984, EPI_ISL_760986, EPI_ISL_760990 to 760993, EPI_ISL_760995 to 760999, EPI_ISL_761003 to 761007,
EPI_ISL_761010, EPI_ISL_761012, EPI_ISL_761014 to 761016, EPI_ISL_761018 to 761020, EPI_ISL_761024, EPI_ISL_761026, EPI_ISL_761028, EPI_ISL_761030 to 761031, EPI_ISL_761034 to 761035, EPI_ISL_761037 to 761038, EPI_ISL_761041, EPI_ISL_761043 to 761045, EPI_ISL_761047, EPI_ISL_761051,
EPI_ISL_761053, EPI_ISL_761055, EPI_ISL_761058, EPI_ISL_761062 to 761063, EPI_ISL_761067 to 761068, EPI_ISL_761070 to 761073, EPI_ISL_761076 to 761078, EPI_ISL_761094 to 761095, EPI_ISL_761113 to 761114, EPI_ISL_761116, EPI_ISL_761118, EPI_ISL_761120, EPI_ISL_761131, EPI_ISL_761143,
EPI_ISL_761145, EPI_ISL_761157, EPI_ISL_761176, EPI_ISL_761187, EPI_ISL_761191, EPI_ISL_761193 to 761194, EPI_ISL_761204, EPI_ISL_761207, EPI_ISL_761209, EPI_ISL_761225, EPI_ISL_761227, EPI_ISL_761229, EPI_ISL_761239, EPI_ISL_761241, EPI_ISL_761252, EPI_ISL_761255, EPI_ISL_761259,
EPI_ISL_761266, EPI_ISL_761276, EPI_ISL_761278, EPI_ISL_761283, EPI_ISL_761287, EPI_ISL_761292 to 761293, EPI_ISL_761296, EPI_ISL_761298, EPI_ISL_761302, EPI_ISL_761307, EPI_ISL_761313, EPI_ISL_761331, EPI_ISL_761338 to 761339, EPI_ISL_761348, EPI_ISL_761351, EPI_ISL_761353, EPI_ISL_761355,
EPI_ISL_761921, EPI_ISL_761923 to 761926, EPI_ISL_761928, EPI_ISL_761930 to 761933, EPI_ISL_761936, EPI_ISL_761938, EPI_ISL_761940 to 761945, EPI_ISL_761947 to 761951, EPI_ISL_761957 to 761960, EPI_ISL_761962 to 761966, EPI_ISL_761969, EPI_ISL_761972 to 761975, EPI_ISL_761978, EPI_ISL_761980,
EPI_ISL_761982 to 761984, EPI_ISL_761986 to 761993, EPI_ISL_761996, EPI_ISL_761998, EPI_ISL_762001, EPI_ISL_762003 to 762006, EPI_ISL_762010 to 762011, EPI_ISL_762013, EPI_ISL_762017, EPI_ISL_762020 to 762022, EPI_ISL_762031 to 762035, EPI_ISL_762039 to 762040, EPI_ISL_762044, EPI_ISL_762046 to

762049, EPI_ISL_762051 to 762053, EPI_ISL_762055 to 762056, EPI_ISL_762058, EPI_ISL_762062, EPI_ISL_762068, EPI_ISL_762070 to 762071, EPI_ISL_762076 to 762077, EPI_ISL_762080 to 762084, EPI_ISL_762086, EPI_ISL_762088, EPI_ISL_762092, EPI_ISL_762094 to 762095, EPI_ISL_762099 to 762102,
EPI_ISL_762105 to 762108, EPI_ISL_762110 to 762111, EPI_ISL_762115 to 762116, EPI_ISL_762119 to 762121, EPI_ISL_762123, EPI_ISL_762127, EPI_ISL_762129 to 762130, EPI_ISL_762132, EPI_ISL_762134, EPI_ISL_762141, EPI_ISL_762143 to 762145, EPI_ISL_762149 to 762151, EPI_ISL_762154, EPI_ISL_762156 to
762158, EPI_ISL_762162, EPI_ISL_762164 to 762165, EPI_ISL_762167 to 762172, EPI_ISL_762176 to 762177, EPI_ISL_762179, EPI_ISL_762182, EPI_ISL_762184, EPI_ISL_762188, EPI_ISL_762190 to 762191, EPI_ISL_762196 to 762197, EPI_ISL_762548, EPI_ISL_762555 to 762556, EPI_ISL_762560 to 762561,
EPI_ISL_762567, EPI_ISL_762574, EPI_ISL_762576, EPI_ISL_762583, EPI_ISL_762586, EPI_ISL_762589, EPI_ISL_762597, EPI_ISL_762602, EPI_ISL_762604, EPI_ISL_762614, EPI_ISL_762618, EPI_ISL_762622, EPI_ISL_762630 to 762631, EPI_ISL_762633, EPI_ISL_762639, EPI_ISL_762644, EPI_ISL_762646,
EPI_ISL_762648, EPI_ISL_762652 to 762654, EPI_ISL_762656 to 762657, EPI_ISL_762661 to 762663, EPI_ISL_762669, EPI_ISL_762676, EPI_ISL_762678, EPI_ISL_762690, EPI_ISL_762694, EPI_ISL_762701, EPI_ISL_762704, EPI_ISL_762708, EPI_ISL_762728, EPI_ISL_762731 to 762732, EPI_ISL_762734,
EPI_ISL_762739, EPI_ISL_762743 to 762744, EPI_ISL_762746, EPI_ISL_762749, EPI_ISL_762758 to 762761, EPI_ISL_762766, EPI_ISL_762770, EPI_ISL_762773, EPI_ISL_762775, EPI_ISL_762779, EPI_ISL_762785, EPI_ISL_762794, EPI_ISL_762801, EPI_ISL_762809, EPI_ISL_762813 to 762814, EPI_ISL_762816,
EPI_ISL_762824, EPI_ISL_762826, EPI_ISL_762837, EPI_ISL_762846, EPI_ISL_762850, EPI_ISL_762853 to 762854, EPI_ISL_762856, EPI_ISL_762861, EPI_ISL_762865 to 762866, EPI_ISL_762868, EPI_ISL_762870 to 762872, EPI_ISL_762876 to 762877, EPI_ISL_762881, EPI_ISL_762885, EPI_ISL_762888,
EPI_ISL_762893, EPI_ISL_762897 to 762898, EPI_ISL_762905, EPI_ISL_762907, EPI_ISL_762909, EPI_ISL_762918, EPI_ISL_762924 to 762925, EPI_ISL_762927 to 762930, EPI_ISL_762934, EPI_ISL_762936, EPI_ISL_762941, EPI_ISL_762950, EPI_ISL_762953 to 762954, EPI_ISL_762956, EPI_ISL_762959 to 762961,
EPI_ISL_762966, EPI_ISL_762980, EPI_ISL_762984 to 762987, EPI_ISL_767092, EPI_ISL_767096, EPI_ISL_767113 to 767114, EPI_ISL_767117 to 767118, EPI_ISL_769223 to 769224, EPI_ISL_769229, EPI_ISL_769233, EPI_ISL_769236, EPI_ISL_769243 to 769246, EPI_ISL_769249 to 769250, EPI_ISL_769252,
EPI_ISL_769254 to 769255, EPI_ISL_769259 to 769261, EPI_ISL_769267, EPI_ISL_769273, EPI_ISL_769275 to 769276, EPI_ISL_769281 to 769282, EPI_ISL_769284, EPI_ISL_769286, EPI_ISL_769288 to 769291, EPI_ISL_769294, EPI_ISL_769297, EPI_ISL_769306, EPI_ISL_769308, EPI_ISL_769310, EPI_ISL_769312,
EPI_ISL_769314, EPI_ISL_769316, EPI_ISL_769319 to 769323, EPI_ISL_769327, EPI_ISL_769330, EPI_ISL_769334, EPI_ISL_769341, EPI_ISL_769343, EPI_ISL_769345, EPI_ISL_769348 to 769349, EPI_ISL_769352 to 769353, EPI_ISL_769355, EPI_ISL_769360 to 769361, EPI_ISL_769365, EPI_ISL_769367,
EPI_ISL_769370, EPI_ISL_769374, EPI_ISL_769380 to 769381, EPI_ISL_769384 to 769385, EPI_ISL_769388 to 769389, EPI_ISL_769391 to 769392, EPI_ISL_769394, EPI_ISL_769399, EPI_ISL_769401 to 769402, EPI_ISL_769404 to 769406, EPI_ISL_769412 to 769413, EPI_ISL_769416, EPI_ISL_769418 to 769422,
EPI_ISL_769427 to 769428, EPI_ISL_769430 to 769431, EPI_ISL_769434, EPI_ISL_769440 to 769441, EPI_ISL_769445, EPI_ISL_769449 to 769450, EPI_ISL_769452, EPI_ISL_769454, EPI_ISL_769457 to 769460, EPI_ISL_769462, EPI_ISL_769466, EPI_ISL_769470 to 769471, EPI_ISL_769473 to 769476, EPI_ISL_769481 to
769482, EPI_ISL_769489, EPI_ISL_769497, EPI_ISL_769499, EPI_ISL_769501, EPI_ISL_769503 to 769504, EPI_ISL_769506, EPI_ISL_769511, EPI_ISL_769513, EPI_ISL_769517 to 769519, EPI_ISL_769522, EPI_ISL_769524 to 769525, EPI_ISL_769528, EPI_ISL_769530, EPI_ISL_769533, EPI_ISL_769535, EPI_ISL_769540,
EPI_ISL_769543, EPI_ISL_769551, EPI_ISL_769556, EPI_ISL_769567, EPI_ISL_769577, EPI_ISL_769583, EPI_ISL_769589, EPI_ISL_769594, EPI_ISL_769606 to 769607, EPI_ISL_769609, EPI_ISL_769611 to 769614, EPI_ISL_769617, EPI_ISL_769619, EPI_ISL_769625, EPI_ISL_769641, EPI_ISL_769652, EPI_ISL_769655,
EPI_ISL_769661 to 769662, EPI_ISL_769666 to 769667, EPI_ISL_769677, EPI_ISL_769687, EPI_ISL_769689, EPI_ISL_769691, EPI_ISL_769705, EPI_ISL_769711, EPI_ISL_769713 to 769714, EPI_ISL_769716, EPI_ISL_769720, EPI_ISL_769724, EPI_ISL_769735, EPI_ISL_769743, EPI_ISL_769751 to 769753,
EPI_ISL_769758 to 769759, EPI_ISL_769761, EPI_ISL_769768, EPI_ISL_769773, EPI_ISL_769787 to 769788, EPI_ISL_769794, EPI_ISL_769802, EPI_ISL_769806, EPI_ISL_769813, EPI_ISL_769818, EPI_ISL_769826, EPI_ISL_769834, EPI_ISL_769836 to 769837, EPI_ISL_769842, EPI_ISL_769850, EPI_ISL_769861 to
769862, EPI_ISL_777016, EPI_ISL_777019, EPI_ISL_777022 to 777023, EPI_ISL_777029, EPI_ISL_777031, EPI_ISL_777034, EPI_ISL_777055, EPI_ISL_777058, EPI_ISL_777061 to 777062, EPI_ISL_777065 to 777066, EPI_ISL_777068, EPI_ISL_777073, EPI_ISL_777095, EPI_ISL_777098, EPI_ISL_777100, EPI_ISL_777115,
EPI_ISL_777117 to 777118, EPI_ISL_777120 to 777122, EPI_ISL_777124, EPI_ISL_777130 to 777131, EPI_ISL_777140, EPI_ISL_777151, EPI_ISL_777160, EPI_ISL_777162, EPI_ISL_777164, EPI_ISL_777176, EPI_ISL_777180, EPI_ISL_777189, EPI_ISL_777205, EPI_ISL_777209, EPI_ISL_777222, EPI_ISL_777225,
EPI_ISL_777227, EPI_ISL_777230, EPI_ISL_777232 to 777233, EPI_ISL_777236, EPI_ISL_777238, EPI_ISL_777242, EPI_ISL_777246, EPI_ISL_777268, EPI_ISL_777283, EPI_ISL_777298, EPI_ISL_777300, EPI_ISL_777321, EPI_ISL_777337, EPI_ISL_777340 to 777341, EPI_ISL_777346, EPI_ISL_777353, EPI_ISL_777356,
EPI_ISL_777358, EPI_ISL_777361, EPI_ISL_777364, EPI_ISL_777372, EPI_ISL_777377, EPI_ISL_777382 to 777383, EPI_ISL_777385, EPI_ISL_777392 to 777393, EPI_ISL_777399, EPI_ISL_777402, EPI_ISL_777407, EPI_ISL_777410, EPI_ISL_777420, EPI_ISL_777423, EPI_ISL_777432, EPI_ISL_777443, EPI_ISL_777462,
EPI_ISL_777471, EPI_ISL_777487, EPI_ISL_777496, EPI_ISL_777498, EPI_ISL_777505 to 777506, EPI_ISL_777510, EPI_ISL_777514, EPI_ISL_777518, EPI_ISL_777526, EPI_ISL_777531, EPI_ISL_777533, EPI_ISL_777554, EPI_ISL_777557, EPI_ISL_777560, EPI_ISL_777562, EPI_ISL_777574, EPI_ISL_777583,
EPI_ISL_777610, EPI_ISL_777612, EPI_ISL_777628, EPI_ISL_777641, EPI_ISL_777646, EPI_ISL_777653, EPI_ISL_777658, EPI_ISL_777660, EPI_ISL_777662 to 777663, EPI_ISL_777667, EPI_ISL_777670, EPI_ISL_778003 to 778004, EPI_ISL_778008 to 778009, EPI_ISL_778024, EPI_ISL_778082, EPI_ISL_778093,
EPI_ISL_778106 to 778107, EPI_ISL_778131, EPI_ISL_778136, EPI_ISL_778229, EPI_ISL_778247, EPI_ISL_778250, EPI_ISL_778258, EPI_ISL_778286, EPI_ISL_778289, EPI_ISL_778299, EPI_ISL_778301 to 778303, EPI_ISL_778325, EPI_ISL_778334, EPI_ISL_778336 to 778337, EPI_ISL_778345, EPI_ISL_778352,
EPI_ISL_778359, EPI_ISL_778361 to 778362, EPI_ISL_778364, EPI_ISL_778373, EPI_ISL_778375 to 778376, EPI_ISL_778378, EPI_ISL_778397, EPI_ISL_778407, EPI_ISL_778411, EPI_ISL_778419 to 778420, EPI_ISL_778422, EPI_ISL_778424, EPI_ISL_778428, EPI_ISL_778451, EPI_ISL_778457, EPI_ISL_778461 to
778462, EPI_ISL_778496, EPI_ISL_778499, EPI_ISL_778506, EPI_ISL_778525, EPI_ISL_778530, EPI_ISL_778534, EPI_ISL_778540 to 778541, EPI_ISL_778551, EPI_ISL_778553, EPI_ISL_778568, EPI_ISL_778570, EPI_ISL_778577, EPI_ISL_778579, EPI_ISL_778617, EPI_ISL_778619 to 778620, EPI_ISL_778629,
EPI_ISL_778631, EPI_ISL_781910, EPI_ISL_781978, EPI_ISL_781990, EPI_ISL_782003 to 782004, EPI_ISL_782036, EPI_ISL_782039, EPI_ISL_782064, EPI_ISL_782133, EPI_ISL_782138, EPI_ISL_782146, EPI_ISL_782151 to 782155, EPI_ISL_782157 to 782159, EPI_ISL_782162, EPI_ISL_782166, EPI_ISL_782170,
EPI_ISL_782173 to 782178, EPI_ISL_782180 to 782181, EPI_ISL_782185, EPI_ISL_782187 to 782189, EPI_ISL_782193 to 782194, EPI_ISL_782198, EPI_ISL_782200, EPI_ISL_782205, EPI_ISL_782208 to 782209, EPI_ISL_782214 to 782215, EPI_ISL_782221, EPI_ISL_782227, EPI_ISL_782229 to 782230, EPI_ISL_782233 to
782234, EPI_ISL_782239, EPI_ISL_782241, EPI_ISL_782244, EPI_ISL_782247 to 782248, EPI_ISL_782253 to 782254, EPI_ISL_782261 to 782262, EPI_ISL_782265, EPI_ISL_782273, EPI_ISL_782277, EPI_ISL_782279, EPI_ISL_782281, EPI_ISL_782285 to 782286, EPI_ISL_782288, EPI_ISL_782293 to 782294,
EPI_ISL_782296, EPI_ISL_782299 to 782300, EPI_ISL_782302, EPI_ISL_782305, EPI_ISL_782310 to 782311, EPI_ISL_782316, EPI_ISL_782320 to 782322, EPI_ISL_782327, EPI_ISL_782331, EPI_ISL_782333 to 782334, EPI_ISL_782336 to 782338, EPI_ISL_782340, EPI_ISL_782343 to 782344, EPI_ISL_782346 to 782347,
EPI_ISL_782349, EPI_ISL_782353 to 782354, EPI_ISL_782356 to 782357, EPI_ISL_782359 to 782360, EPI_ISL_782365 to 782367, EPI_ISL_782373, EPI_ISL_782375 to 782376, EPI_ISL_782381, EPI_ISL_782383, EPI_ISL_782385, EPI_ISL_782387, EPI_ISL_782391 to 782392, EPI_ISL_782395, EPI_ISL_782397,
EPI_ISL_782400, EPI_ISL_782407 to 782408, EPI_ISL_782411, EPI_ISL_782414 to 782418, EPI_ISL_782422, EPI_ISL_782424, EPI_ISL_782427 to 782430, EPI_ISL_782432, EPI_ISL_782434, EPI_ISL_782436, EPI_ISL_782440 to 782441, EPI_ISL_782443, EPI_ISL_782445, EPI_ISL_782453, EPI_ISL_782455,
EPI_ISL_782457, EPI_ISL_782459 to 782460, EPI_ISL_782463, EPI_ISL_782470, EPI_ISL_782473, EPI_ISL_782477, EPI_ISL_782479 to 782480, EPI_ISL_782483, EPI_ISL_782486, EPI_ISL_782499, EPI_ISL_782503, EPI_ISL_782523, EPI_ISL_782533, EPI_ISL_782536, EPI_ISL_782544, EPI_ISL_782569, EPI_ISL_782576,
EPI_ISL_782584, EPI_ISL_782607, EPI_ISL_782609, EPI_ISL_782618, EPI_ISL_782623, EPI_ISL_782633, EPI_ISL_782639, EPI_ISL_782688, EPI_ISL_782704, EPI_ISL_782706, EPI_ISL_782709, EPI_ISL_782713, EPI_ISL_782715, EPI_ISL_782729, EPI_ISL_797716, EPI_ISL_797722, EPI_ISL_797724 to 797725,
EPI_ISL_797731, EPI_ISL_797733 to 797736, EPI_ISL_797738 to 797741, EPI_ISL_797744, EPI_ISL_797746 to 797751, EPI_ISL_797753 to 797755, EPI_ISL_797757 to 797759, EPI_ISL_797761 to 797763, EPI_ISL_797766 to 797770, EPI_ISL_797773 to 797778, EPI_ISL_797781, EPI_ISL_797785, EPI_ISL_797789 to 797790,
EPI_ISL_797792 to 797793, EPI_ISL_797795 to 797796, EPI_ISL_797801 to 797804, EPI_ISL_797808 to 797809, EPI_ISL_797815 to 797816, EPI_ISL_797819, EPI_ISL_797822 to 797824, EPI_ISL_797827, EPI_ISL_797829 to 797833, EPI_ISL_797835 to 797836, EPI_ISL_797842, EPI_ISL_797844 to 797846, EPI_ISL_797849
to 797850, EPI_ISL_797852 to 797855, EPI_ISL_797858 to 797859, EPI_ISL_797861, EPI_ISL_797863, EPI_ISL_797866, EPI_ISL_797869, EPI_ISL_797871, EPI_ISL_797873, EPI_ISL_797875, EPI_ISL_797878, EPI_ISL_797880, EPI_ISL_797882 to 797884, EPI_ISL_797887, EPI_ISL_797889, EPI_ISL_797891,
EPI_ISL_797895 to 797896, EPI_ISL_797899 to 797903, EPI_ISL_797905, EPI_ISL_797908, EPI_ISL_797910, EPI_ISL_797912 to 797913, EPI_ISL_797915 to 797916, EPI_ISL_797919 to 797921, EPI_ISL_797923, EPI_ISL_797925 to 797929, EPI_ISL_797931, EPI_ISL_797935, EPI_ISL_797937 to 797939, EPI_ISL_797941 to
797942, EPI_ISL_797944, EPI_ISL_797946 to 797947, EPI_ISL_797949 to 797950, EPI_ISL_797952 to 797955, EPI_ISL_797958, EPI_ISL_797960, EPI_ISL_797962 to 797964, EPI_ISL_797970 to 797973, EPI_ISL_797977, EPI_ISL_797980, EPI_ISL_797983, EPI_ISL_797985 to 797990, EPI_ISL_797993 to 797994,
EPI_ISL_798001, EPI_ISL_798004, EPI_ISL_798008, EPI_ISL_798010, EPI_ISL_798017, EPI_ISL_798036 to 798037, EPI_ISL_798039, EPI_ISL_798041, EPI_ISL_798043, EPI_ISL_798047, EPI_ISL_798051 to 798052, EPI_ISL_798056, EPI_ISL_798063, EPI_ISL_798071, EPI_ISL_798074 to 798075, EPI_ISL_798080 to
798082, EPI_ISL_798087, EPI_ISL_798091, EPI_ISL_798107, EPI_ISL_798116, EPI_ISL_798118, EPI_ISL_798120, EPI_ISL_798129, EPI_ISL_798141, EPI_ISL_798146, EPI_ISL_798150, EPI_ISL_798156, EPI_ISL_798171 to 798172, EPI_ISL_798179, EPI_ISL_798184, EPI_ISL_798190, EPI_ISL_798192, EPI_ISL_798195,
EPI_ISL_798207, EPI_ISL_798215, EPI_ISL_798223, EPI_ISL_798226 to 798228, EPI_ISL_798231, EPI_ISL_798238, EPI_ISL_798240, EPI_ISL_798247, EPI_ISL_798249, EPI_ISL_798259, EPI_ISL_798263 to 798265, EPI_ISL_798278 to 798279, EPI_ISL_798286 to 798287, EPI_ISL_798297, EPI_ISL_798302 to 798303,
EPI_ISL_798308, EPI_ISL_798313, EPI_ISL_798332, EPI_ISL_799006, EPI_ISL_799027, EPI_ISL_799042 to 799043, EPI_ISL_799046, EPI_ISL_799060, EPI_ISL_799064, EPI_ISL_799067, EPI_ISL_799071, EPI_ISL_799080, EPI_ISL_799100, EPI_ISL_799102, EPI_ISL_799107 to 799108, EPI_ISL_799126, EPI_ISL_799129 to
799130, EPI_ISL_799144, EPI_ISL_799150, EPI_ISL_799155, EPI_ISL_799164, EPI_ISL_799172, EPI_ISL_799197, EPI_ISL_799203, EPI_ISL_799216, EPI_ISL_799218, EPI_ISL_799230, EPI_ISL_799255, EPI_ISL_799262 to 799264, EPI_ISL_799267, EPI_ISL_799272, EPI_ISL_799289 to 799290, EPI_ISL_799294,
EPI_ISL_799297 to 799298, EPI_ISL_799300 to 799302, EPI_ISL_799304, EPI_ISL_799310, EPI_ISL_799312, EPI_ISL_799316, EPI_ISL_799318, EPI_ISL_799322, EPI_ISL_799326, EPI_ISL_799338, EPI_ISL_799341, EPI_ISL_799343, EPI_ISL_799345, EPI_ISL_799349, EPI_ISL_799355, EPI_ISL_799369, EPI_ISL_799371,
EPI_ISL_799374, EPI_ISL_799377, EPI_ISL_799381, EPI_ISL_799389, EPI_ISL_799393, EPI_ISL_799396, EPI_ISL_799398, EPI_ISL_799402, EPI_ISL_799408 to 799409, EPI_ISL_799417 to 799419, EPI_ISL_799422, EPI_ISL_799424, EPI_ISL_799428, EPI_ISL_799431, EPI_ISL_799435, EPI_ISL_799438, EPI_ISL_799444,
EPI_ISL_799449, EPI_ISL_799451 to 799452, EPI_ISL_799456 to 799459, EPI_ISL_799461, EPI_ISL_799464, EPI_ISL_799471, EPI_ISL_799474, EPI_ISL_799478, EPI_ISL_799480 to 799481, EPI_ISL_799483, EPI_ISL_799499, EPI_ISL_799503, EPI_ISL_799507, EPI_ISL_799513, EPI_ISL_799516, EPI_ISL_799518,
EPI_ISL_799524, EPI_ISL_799526 to 799527, EPI_ISL_799530, EPI_ISL_799532, EPI_ISL_799537, EPI_ISL_799539, EPI_ISL_799541 to 799542, EPI_ISL_799544, EPI_ISL_799549, EPI_ISL_799554, EPI_ISL_799556, EPI_ISL_799559 to 799560, EPI_ISL_799564, EPI_ISL_799566, EPI_ISL_799569, EPI_ISL_799571,
EPI_ISL_799574, EPI_ISL_799581, EPI_ISL_799584, EPI_ISL_799586, EPI_ISL_799592, EPI_ISL_799596, EPI_ISL_799599, EPI_ISL_799601, EPI_ISL_799615, EPI_ISL_799622, EPI_ISL_799634, EPI_ISL_799641, EPI_ISL_799649, EPI_ISL_799661, EPI_ISL_799664, EPI_ISL_799669, EPI_ISL_799676, EPI_ISL_799679,
EPI_ISL_799681 to 799682, EPI_ISL_799686, EPI_ISL_799713, EPI_ISL_799718, EPI_ISL_799722, EPI_ISL_799730 to 799731, EPI_ISL_799735, EPI_ISL_799739, EPI_ISL_799748, EPI_ISL_799761, EPI_ISL_799775 to 799776, EPI_ISL_799780, EPI_ISL_799783, EPI_ISL_799786, EPI_ISL_799791, EPI_ISL_799805,
EPI_ISL_799811, EPI_ISL_799819, EPI_ISL_799825, EPI_ISL_799830, EPI_ISL_799838, EPI_ISL_799843, EPI_ISL_799861, EPI_ISL_799864 to 799866, EPI_ISL_799869, EPI_ISL_799871, EPI_ISL_799875, EPI_ISL_799883, EPI_ISL_799888 to 799889, EPI_ISL_799892 to 799894, EPI_ISL_799901, EPI_ISL_799910,
EPI_ISL_799912, EPI_ISL_799916 to 799919, EPI_ISL_799937, EPI_ISL_799946, EPI_ISL_799951, EPI_ISL_799954, EPI_ISL_799965, EPI_ISL_799968 to 799969, EPI_ISL_799973, EPI_ISL_799976, EPI_ISL_799980, EPI_ISL_799986 to 799987, EPI_ISL_799992, EPI_ISL_799997, EPI_ISL_800000 to 800001,
EPI_ISL_800005, EPI_ISL_800010, EPI_ISL_800027, EPI_ISL_800029 to 800030, EPI_ISL_800032, EPI_ISL_800034, EPI_ISL_800038, EPI_ISL_800048, EPI_ISL_800052, EPI_ISL_800056, EPI_ISL_800068, EPI_ISL_800070, EPI_ISL_800079, EPI_ISL_800084, EPI_ISL_800106, EPI_ISL_800113, EPI_ISL_800120,
EPI_ISL_800122, EPI_ISL_800131, EPI_ISL_800138, EPI_ISL_800141 to 800142, EPI_ISL_800151, EPI_ISL_800164, EPI_ISL_800174 to 800176, EPI_ISL_800180, EPI_ISL_800183, EPI_ISL_800190 to 800191, EPI_ISL_800200 to 800201, EPI_ISL_800204, EPI_ISL_800217, EPI_ISL_800221, EPI_ISL_800234 to 800235,
EPI_ISL_800237, EPI_ISL_800245, EPI_ISL_800736, EPI_ISL_800745, EPI_ISL_800812, EPI_ISL_800827, EPI_ISL_801236 to 801237, EPI_ISL_801244, EPI_ISL_801252, EPI_ISL_801254, EPI_ISL_801256, EPI_ISL_801258, EPI_ISL_801260, EPI_ISL_801276 to 801277, EPI_ISL_801284 to 801285, EPI_ISL_801290,
EPI_ISL_801305, EPI_ISL_801323, EPI_ISL_801334, EPI_ISL_801337, EPI_ISL_801343, EPI_ISL_801354, EPI_ISL_811194 to 811196, EPI_ISL_811199 to 811200, EPI_ISL_811208 to 811210, EPI_ISL_811213, EPI_ISL_811216, EPI_ISL_811218 to 811221, EPI_ISL_811224 to 811225, EPI_ISL_811227, EPI_ISL_811230 to
811231, EPI_ISL_811235, EPI_ISL_811237, EPI_ISL_811239 to 811240, EPI_ISL_811243, EPI_ISL_811246, EPI_ISL_811249 to 811250, EPI_ISL_811252 to 811253, EPI_ISL_811256, EPI_ISL_811258 to 811259, EPI_ISL_811261, EPI_ISL_811263, EPI_ISL_811266, EPI_ISL_811271, EPI_ISL_811274, EPI_ISL_811276 to
811279, EPI_ISL_811281, EPI_ISL_811283, EPI_ISL_811286, EPI_ISL_811288, EPI_ISL_811290 to 811292, EPI_ISL_811294 to 811299, EPI_ISL_811301, EPI_ISL_811303, EPI_ISL_811305, EPI_ISL_811314, EPI_ISL_811316 to 811317, EPI_ISL_811319, EPI_ISL_811324, EPI_ISL_811327 to 811328, EPI_ISL_811330,
EPI_ISL_811337, EPI_ISL_811340, EPI_ISL_811346 to 811347, EPI_ISL_811352, EPI_ISL_811355 to 811356, EPI_ISL_811360 to 811361, EPI_ISL_811365, EPI_ISL_811371 to 811372, EPI_ISL_811374, EPI_ISL_811379, EPI_ISL_811384, EPI_ISL_811387, EPI_ISL_811391, EPI_ISL_811396, EPI_ISL_811398,
EPI_ISL_811401 to 811403, EPI_ISL_811405, EPI_ISL_811407, EPI_ISL_811411 to 811413, EPI_ISL_811415, EPI_ISL_811417, EPI_ISL_811419, EPI_ISL_811421, EPI_ISL_811423 to 811425, EPI_ISL_811427 to 811428, EPI_ISL_811437, EPI_ISL_811440 to 811442, EPI_ISL_811447 to 811450, EPI_ISL_820147,
EPI_ISL_820150, EPI_ISL_820152, EPI_ISL_820171, EPI_ISL_820176 to 820177, EPI_ISL_820184, EPI_ISL_820197, EPI_ISL_820212, EPI_ISL_820220, EPI_ISL_820260, EPI_ISL_820270, EPI_ISL_820272, EPI_ISL_820283, EPI_ISL_820312, EPI_ISL_820319, EPI_ISL_820387, EPI_ISL_820417, EPI_ISL_820421,
EPI_ISL_820448, EPI_ISL_820461, EPI_ISL_820465, EPI_ISL_820468, EPI_ISL_820477, EPI_ISL_820479, EPI_ISL_820489, EPI_ISL_820505, EPI_ISL_820512, EPI_ISL_820518, EPI_ISL_820547, EPI_ISL_820558, EPI_ISL_820577, EPI_ISL_820590, EPI_ISL_820613, EPI_ISL_820617, EPI_ISL_820620, EPI_ISL_820628,
EPI_ISL_820633 to 820634, EPI_ISL_820639, EPI_ISL_820645, EPI_ISL_821032, EPI_ISL_821105, EPI_ISL_821150, EPI_ISL_821198, EPI_ISL_821225, EPI_ISL_821227, EPI_ISL_821241, EPI_ISL_821245, EPI_ISL_821258, EPI_ISL_821621, EPI_ISL_821995, EPI_ISL_822001, EPI_ISL_822004, EPI_ISL_822006,
EPI_ISL_822011, EPI_ISL_822022 to 822023, EPI_ISL_822029, EPI_ISL_822040 to 822041, EPI_ISL_822048, EPI_ISL_822067, EPI_ISL_822075, EPI_ISL_822083, EPI_ISL_822099, EPI_ISL_822113, EPI_ISL_822119, EPI_ISL_822122, EPI_ISL_822130, EPI_ISL_822148, EPI_ISL_822153, EPI_ISL_822156, EPI_ISL_822158,
EPI_ISL_822165, EPI_ISL_822170, EPI_ISL_822178, EPI_ISL_822182, EPI_ISL_822237, EPI_ISL_822240, EPI_ISL_822256, EPI_ISL_822264, EPI_ISL_822275, EPI_ISL_822281, EPI_ISL_822288, EPI_ISL_822292, EPI_ISL_822295, EPI_ISL_822323, EPI_ISL_822327, EPI_ISL_822330, EPI_ISL_822333, EPI_ISL_822338,
EPI_ISL_822341, EPI_ISL_834554, EPI_ISL_834556 to 834564, EPI_ISL_834566 to 834567, EPI_ISL_834569 to 834570, EPI_ISL_834573 to 834575, EPI_ISL_834577 to 834578, EPI_ISL_834580, EPI_ISL_834585 to 834586, EPI_ISL_834588, EPI_ISL_834591 to 834592, EPI_ISL_834595, EPI_ISL_834597 to 834598,
EPI_ISL_834600 to 834601, EPI_ISL_834605, EPI_ISL_834607, EPI_ISL_834610 to 834612, EPI_ISL_834616, EPI_ISL_834618 to 834619, EPI_ISL_834621, EPI_ISL_834625, EPI_ISL_834627 to 834628, EPI_ISL_834631, EPI_ISL_834633, EPI_ISL_834635, EPI_ISL_834639, EPI_ISL_834643, EPI_ISL_834648 to 834652,
EPI_ISL_834654 to 834655, EPI_ISL_834659, EPI_ISL_834662 to 834663, EPI_ISL_834665, EPI_ISL_834671 to 834674, EPI_ISL_834677, EPI_ISL_834679 to 834680, EPI_ISL_834682, EPI_ISL_834685, EPI_ISL_834688 to 834690, EPI_ISL_834696 to 834700, EPI_ISL_834702 to 834706, EPI_ISL_834708 to 834709,
EPI_ISL_834711 to 834713, EPI_ISL_834715 to 834719, EPI_ISL_834721 to 834723, EPI_ISL_834725, EPI_ISL_834727 to 834728, EPI_ISL_834730 to 834732, EPI_ISL_834734 to 834736, EPI_ISL_834739, EPI_ISL_834742 to 834744, EPI_ISL_834746 to 834748, EPI_ISL_834750, EPI_ISL_834753 to 834754, EPI_ISL_834759
to 834760, EPI_ISL_834762 to 834764, EPI_ISL_834766 to 834768, EPI_ISL_834770, EPI_ISL_834773 to 834778, EPI_ISL_834783 to 834787, EPI_ISL_834796, EPI_ISL_835144, EPI_ISL_835146 to 835148, EPI_ISL_835150, EPI_ISL_835152, EPI_ISL_835158, EPI_ISL_835161 to 835164, EPI_ISL_835166, EPI_ISL_835168,
EPI_ISL_835171 to 835175, EPI_ISL_835177 to 835182, EPI_ISL_835184, EPI_ISL_835187 to 835193, EPI_ISL_835195, EPI_ISL_835197, EPI_ISL_835203, EPI_ISL_835206, EPI_ISL_835208 to 835213, EPI_ISL_835215 to 835216, EPI_ISL_835218, EPI_ISL_835220, EPI_ISL_835222, EPI_ISL_835224 to 835225,
EPI_ISL_835227 to 835230, EPI_ISL_835233 to 835234, EPI_ISL_835236, EPI_ISL_835239, EPI_ISL_835245, EPI_ISL_835248 to 835251, EPI_ISL_835253, EPI_ISL_835255 to 835256, EPI_ISL_835262 to 835267, EPI_ISL_835273 to 835283, EPI_ISL_835285 to 835286, EPI_ISL_835293, EPI_ISL_835295, EPI_ISL_835297,
EPI_ISL_835299 to 835301, EPI_ISL_835304, EPI_ISL_835306 to 835307, EPI_ISL_835310, EPI_ISL_835312, EPI_ISL_835314, EPI_ISL_835316 to 835318, EPI_ISL_835321 to 835322, EPI_ISL_835324, EPI_ISL_835328, EPI_ISL_835331 to 835332, EPI_ISL_835336, EPI_ISL_835338, EPI_ISL_835341, EPI_ISL_835345,
EPI_ISL_835348 to 835351, EPI_ISL_835354, EPI_ISL_835357 to 835358, EPI_ISL_835360 to 835361, EPI_ISL_835363, EPI_ISL_835366 to 835367, EPI_ISL_835370 to 835371, EPI_ISL_835374 to 835375, EPI_ISL_835377 to 835379, EPI_ISL_835382, EPI_ISL_835384 to 835388, EPI_ISL_835391 to 835392, EPI_ISL_835394
to 835396, EPI_ISL_835398 to 835402, EPI_ISL_835404, EPI_ISL_835406 to 835408, EPI_ISL_835410 to 835411, EPI_ISL_835413, EPI_ISL_835419, EPI_ISL_835421, EPI_ISL_835423 to 835424, EPI_ISL_835433, EPI_ISL_835439, EPI_ISL_835447 to 835448, EPI_ISL_835457, EPI_ISL_835464 to 835465, EPI_ISL_835470,
EPI_ISL_835475, EPI_ISL_835481, EPI_ISL_835486, EPI_ISL_835488, EPI_ISL_835493, EPI_ISL_835497, EPI_ISL_835499 to 835500, EPI_ISL_835502, EPI_ISL_835506 to 835507, EPI_ISL_835518, EPI_ISL_835531, EPI_ISL_835533, EPI_ISL_835535, EPI_ISL_835539 to 835541, EPI_ISL_835547, EPI_ISL_835553,
EPI_ISL_835573 to 835574, EPI_ISL_835576, EPI_ISL_835582, EPI_ISL_835589 to 835590, EPI_ISL_835592, EPI_ISL_835597, EPI_ISL_835600, EPI_ISL_835603, EPI_ISL_835610, EPI_ISL_835616, EPI_ISL_835618, EPI_ISL_835623, EPI_ISL_835643, EPI_ISL_835646 to 835647, EPI_ISL_835651, EPI_ISL_835659,
EPI_ISL_835664, EPI_ISL_835673, EPI_ISL_835677 to 835678, EPI_ISL_835683, EPI_ISL_835686 to 835688, EPI_ISL_835692, EPI_ISL_835695, EPI_ISL_835703, EPI_ISL_835707, EPI_ISL_836748 to 836750, EPI_ISL_836752 to 836753, EPI_ISL_836755 to 836756, EPI_ISL_836758 to 836759, EPI_ISL_836761 to 836762,
EPI_ISL_836764, EPI_ISL_836771, EPI_ISL_836773 to 836776, EPI_ISL_836778 to 836780, EPI_ISL_836783, EPI_ISL_836786, EPI_ISL_836791, EPI_ISL_836793 to 836794, EPI_ISL_836796, EPI_ISL_836798, EPI_ISL_836800 to 836801, EPI_ISL_836804 to 836805, EPI_ISL_836808 to 836809, EPI_ISL_836812,
EPI_ISL_836816, EPI_ISL_836818, EPI_ISL_836824, EPI_ISL_836828, EPI_ISL_836830 to 836833, EPI_ISL_836835, EPI_ISL_836838, EPI_ISL_836841, EPI_ISL_836845, EPI_ISL_836852, EPI_ISL_836854 to 836857, EPI_ISL_836860 to 836861, EPI_ISL_836865, EPI_ISL_836868, EPI_ISL_836871, EPI_ISL_836873,
EPI_ISL_836879, EPI_ISL_836882, EPI_ISL_836884 to 836886, EPI_ISL_836888 to 836889, EPI_ISL_836892, EPI_ISL_836894, EPI_ISL_836899, EPI_ISL_836904 to 836905, EPI_ISL_836911, EPI_ISL_836915, EPI_ISL_836918 to 836919, EPI_ISL_836924 to 836925, EPI_ISL_836927, EPI_ISL_836932 to 836934,
EPI_ISL_836938, EPI_ISL_836941 to 836945, EPI_ISL_836949 to 836951, EPI_ISL_836953, EPI_ISL_836956, EPI_ISL_836958, EPI_ISL_836960 to 836961, EPI_ISL_836963 to 836965, EPI_ISL_836968, EPI_ISL_836970, EPI_ISL_836973, EPI_ISL_846528, EPI_ISL_846530, EPI_ISL_846534, EPI_ISL_846539,
EPI_ISL_851077, EPI_ISL_851081 to 851082, EPI_ISL_851094 to 851095, EPI_ISL_851107, EPI_ISL_851116, EPI_ISL_851119, EPI_ISL_851124, EPI_ISL_851126, EPI_ISL_851130, EPI_ISL_851134 to 851135, EPI_ISL_851137, EPI_ISL_851146 to 851148, EPI_ISL_851167, EPI_ISL_851180, EPI_ISL_851182,
EPI_ISL_851190, EPI_ISL_851218, EPI_ISL_851241, EPI_ISL_851246, EPI_ISL_851252, EPI_ISL_851257 to 851259, EPI_ISL_851264, EPI_ISL_851270, EPI_ISL_851276, EPI_ISL_851296, EPI_ISL_851301, EPI_ISL_851306 to 851307, EPI_ISL_851325, EPI_ISL_851327, EPI_ISL_851329, EPI_ISL_851335, EPI_ISL_851346,
EPI_ISL_851358, EPI_ISL_851365, EPI_ISL_851380, EPI_ISL_851382 to 851385, EPI_ISL_851387 to 851388, EPI_ISL_851390 to 851392, EPI_ISL_851395 to 851399, EPI_ISL_851402, EPI_ISL_851404, EPI_ISL_851407, EPI_ISL_851410, EPI_ISL_851412 to 851413, EPI_ISL_851416 to 851417, EPI_ISL_851420,
EPI_ISL_851425, EPI_ISL_851427 to 851428, EPI_ISL_851430 to 851432, EPI_ISL_851435 to 851440, EPI_ISL_851442, EPI_ISL_851444 to 851445, EPI_ISL_851447, EPI_ISL_851450, EPI_ISL_851452 to 851456, EPI_ISL_851460, EPI_ISL_851462 to 851465, EPI_ISL_851467 to 851468, EPI_ISL_851473, EPI_ISL_851475 to
851476, EPI_ISL_851480 to 851482, EPI_ISL_851484, EPI_ISL_851486 to 851491, EPI_ISL_851494 to 851495, EPI_ISL_851498 to 851499, EPI_ISL_851503 to 851504, EPI_ISL_851506 to 851507, EPI_ISL_851510, EPI_ISL_851512, EPI_ISL_851515 to 851517, EPI_ISL_851520 to 851521, EPI_ISL_851524 to 851526,

EPI_ISL_851528 to 851529, EPI_ISL_851531 to 851533, EPI_ISL_851535 to 851536, EPI_ISL_851538, EPI_ISL_851541, EPI_ISL_851543, EPI_ISL_851547 to 851548, EPI_ISL_851551, EPI_ISL_851553 to 851554, EPI_ISL_851560, EPI_ISL_851562 to 851563, EPI_ISL_851568, EPI_ISL_851570, EPI_ISL_851573 to 851577,
EPI_ISL_851579 to 851581, EPI_ISL_851583 to 851586, EPI_ISL_851589 to 851592, EPI_ISL_851596 to 851597, EPI_ISL_851599 to 851602, EPI_ISL_851605 to 851606, EPI_ISL_851608 to 851612, EPI_ISL_851615 to 851617, EPI_ISL_851619 to 851622, EPI_ISL_851627, EPI_ISL_851629 to 851630, EPI_ISL_851632 to
851635, EPI_ISL_851637, EPI_ISL_851640 to 851641, EPI_ISL_851647 to 851648, EPI_ISL_851651, EPI_ISL_851653 to 851656, EPI_ISL_851658 to 851659, EPI_ISL_851661, EPI_ISL_851663 to 851665, EPI_ISL_851668, EPI_ISL_851670 to 851672, EPI_ISL_851674, EPI_ISL_851676, EPI_ISL_851686 to 851687,
EPI_ISL_851690, EPI_ISL_851693, EPI_ISL_851701, EPI_ISL_851714, EPI_ISL_851720, EPI_ISL_851725, EPI_ISL_851729, EPI_ISL_851738, EPI_ISL_851754, EPI_ISL_851757, EPI_ISL_851760, EPI_ISL_851763, EPI_ISL_851767, EPI_ISL_851770, EPI_ISL_851772, EPI_ISL_851774, EPI_ISL_851780 to 851781,
EPI_ISL_851785, EPI_ISL_851792, EPI_ISL_851794, EPI_ISL_851797 to 851799, EPI_ISL_851803, EPI_ISL_851805, EPI_ISL_851807, EPI_ISL_851829, EPI_ISL_851837, EPI_ISL_851840, EPI_ISL_851847, EPI_ISL_851851, EPI_ISL_851857, EPI_ISL_851863 to 851864, EPI_ISL_851866, EPI_ISL_851870 to 851871,
EPI_ISL_851876 to 851877, EPI_ISL_851885, EPI_ISL_851888, EPI_ISL_851891, EPI_ISL_851895, EPI_ISL_851898, EPI_ISL_851901, EPI_ISL_851905 to 851906, EPI_ISL_851920, EPI_ISL_851922, EPI_ISL_851934, EPI_ISL_851940, EPI_ISL_851950, EPI_ISL_851955 to 851956, EPI_ISL_852258, EPI_ISL_852262 to
852263, EPI_ISL_852265, EPI_ISL_852267, EPI_ISL_852269 to 852271, EPI_ISL_852273 to 852280, EPI_ISL_852283 to 852284, EPI_ISL_852287 to 852290, EPI_ISL_852292, EPI_ISL_852295 to 852300, EPI_ISL_852303 to 852306, EPI_ISL_852308 to 852309, EPI_ISL_852313 to 852314, EPI_ISL_852316, EPI_ISL_852319,
EPI_ISL_852321, EPI_ISL_852324 to 852325, EPI_ISL_852328 to 852329, EPI_ISL_852332 to 852337, EPI_ISL_852340 to 852346, EPI_ISL_852348, EPI_ISL_852350, EPI_ISL_852352 to 852354, EPI_ISL_852356 to 852363, EPI_ISL_852365, EPI_ISL_852371 to 852374, EPI_ISL_852376 to 852377, EPI_ISL_852379 to 852382,
EPI_ISL_852385 to 852388, EPI_ISL_852391 to 852397, EPI_ISL_852399 to 852400, EPI_ISL_852402, EPI_ISL_852406, EPI_ISL_852408, EPI_ISL_852410, EPI_ISL_852412 to 852413, EPI_ISL_852415, EPI_ISL_852417 to 852420, EPI_ISL_852424 to 852426, EPI_ISL_852428 to 852429, EPI_ISL_852432 to 852434,
EPI_ISL_852436, EPI_ISL_852439, EPI_ISL_852441 to 852442, EPI_ISL_852444 to 852445, EPI_ISL_852447 to 852450, EPI_ISL_852452, EPI_ISL_852454, EPI_ISL_852456 to 852457, EPI_ISL_852459, EPI_ISL_852461, EPI_ISL_852463, EPI_ISL_852465 to 852466, EPI_ISL_852469 to 852471, EPI_ISL_852474 to 852480,
EPI_ISL_852482 to 852484, EPI_ISL_852486 to 852487, EPI_ISL_852490 to 852496, EPI_ISL_852499, EPI_ISL_852501 to 852504, EPI_ISL_852511 to 852512, EPI_ISL_852514 to 852515, EPI_ISL_852517 to 852518, EPI_ISL_852520 to 852522, EPI_ISL_852525 to 852528, EPI_ISL_852531 to 852534, EPI_ISL_852536 to
852537, EPI_ISL_852539, EPI_ISL_852541, EPI_ISL_852544, EPI_ISL_852546, EPI_ISL_852548 to 852550, EPI_ISL_852553, EPI_ISL_852556, EPI_ISL_852560 to 852563, EPI_ISL_857870 to 857872, EPI_ISL_857876, EPI_ISL_857878, EPI_ISL_857885, EPI_ISL_857887 to 857889, EPI_ISL_857893 to 857894,
EPI_ISL_857896 to 857898, EPI_ISL_857900, EPI_ISL_857902, EPI_ISL_857904 to 857906, EPI_ISL_857908 to 857912, EPI_ISL_857915 to 857917, EPI_ISL_857919, EPI_ISL_857924 to 857927, EPI_ISL_857929, EPI_ISL_857933 to 857934, EPI_ISL_857936 to 857939, EPI_ISL_857943, EPI_ISL_857947 to 857950,
EPI_ISL_857952 to 857955, EPI_ISL_857957 to 857959, EPI_ISL_858011 to 858012, EPI_ISL_858015, EPI_ISL_858017, EPI_ISL_858020, EPI_ISL_858022 to 858026, EPI_ISL_858028 to 858029, EPI_ISL_858031 to 858032, EPI_ISL_858035 to 858036, EPI_ISL_858038, EPI_ISL_858041, EPI_ISL_858043 to 858044,
EPI_ISL_858048 to 858052, EPI_ISL_858054 to 858055, EPI_ISL_858057 to 858061, EPI_ISL_858158, EPI_ISL_858162, EPI_ISL_858164, EPI_ISL_858168, EPI_ISL_858172 to 858176, EPI_ISL_858178 to 858183, EPI_ISL_858185, EPI_ISL_858187 to 858188, EPI_ISL_858190 to 858194, EPI_ISL_858197 to 858199,
EPI_ISL_858201, EPI_ISL_858204 to 858206, EPI_ISL_858223, EPI_ISL_858225 to 858232, EPI_ISL_858234 to 858237, EPI_ISL_858242 to 858243, EPI_ISL_858247 to 858251, EPI_ISL_858253, EPI_ISL_858261 to 858264, EPI_ISL_858272, EPI_ISL_858274 to 858275, EPI_ISL_858281 to 858282, EPI_ISL_858284 to 858285,
EPI_ISL_858290 to 858291, EPI_ISL_858293 to 858295, EPI_ISL_858298, EPI_ISL_858300 to 858301, EPI_ISL_858303 to 858305, EPI_ISL_858313 to 858314, EPI_ISL_858316 to 858318, EPI_ISL_858320, EPI_ISL_858327, EPI_ISL_858330 to 858331, EPI_ISL_858333, EPI_ISL_858337 to 858339, EPI_ISL_858344 to 858347,
EPI_ISL_858349, EPI_ISL_858351 to 858353, EPI_ISL_858358 to 858360, EPI_ISL_858362 to 858363, EPI_ISL_858366 to 858367, EPI_ISL_858369, EPI_ISL_858373, EPI_ISL_858376, EPI_ISL_858379, EPI_ISL_858383, EPI_ISL_858385, EPI_ISL_858392, EPI_ISL_858394, EPI_ISL_858399, EPI_ISL_858406 to 858407,
EPI_ISL_858424, EPI_ISL_858428 to 858429, EPI_ISL_858431 to 858433, EPI_ISL_858435, EPI_ISL_858441, EPI_ISL_858443, EPI_ISL_858445, EPI_ISL_858447, EPI_ISL_858449 to 858450, EPI_ISL_858452 to 858455, EPI_ISL_858459 to 858460, EPI_ISL_858462, EPI_ISL_858466 to 858467, EPI_ISL_858469 to 858470,
EPI_ISL_858473 to 858475, EPI_ISL_858480 to 858481, EPI_ISL_858485 to 858488, EPI_ISL_858492 to 858495, EPI_ISL_858499 to 858500, EPI_ISL_858502, EPI_ISL_858505, EPI_ISL_858508, EPI_ISL_858512, EPI_ISL_858514 to 858515, EPI_ISL_858518, EPI_ISL_858520, EPI_ISL_858522, EPI_ISL_858525,
EPI_ISL_858528 to 858529, EPI_ISL_858532 to 858533, EPI_ISL_858535 to 858537, EPI_ISL_858542 to 858543, EPI_ISL_858549 to 858551, EPI_ISL_858554, EPI_ISL_858556, EPI_ISL_858561 to 858562, EPI_ISL_858564, EPI_ISL_858568, EPI_ISL_858570 to 858571, EPI_ISL_858583 to 858584, EPI_ISL_858588 to 858589,
EPI_ISL_858593 to 858595, EPI_ISL_858597, EPI_ISL_858599 to 858600, EPI_ISL_858602 to 858603, EPI_ISL_858605 to 858607, EPI_ISL_858609, EPI_ISL_858614, EPI_ISL_858617 to 858618, EPI_ISL_858622 to 858623, EPI_ISL_858626 to 858629, EPI_ISL_858631, EPI_ISL_858634 to 858635, EPI_ISL_858637,
EPI_ISL_858639, EPI_ISL_858642, EPI_ISL_858659 to 858660, EPI_ISL_858664, EPI_ISL_858712, EPI_ISL_858718, EPI_ISL_858721, EPI_ISL_858726 to 858728, EPI_ISL_858730 to 858731, EPI_ISL_858738, EPI_ISL_858741, EPI_ISL_858743 to 858744, EPI_ISL_858747, EPI_ISL_858751, EPI_ISL_858763,
EPI_ISL_858769, EPI_ISL_858771, EPI_ISL_858777, EPI_ISL_858781, EPI_ISL_858842 to 858843, EPI_ISL_858859 to 858860, EPI_ISL_858863, EPI_ISL_858865, EPI_ISL_858900, EPI_ISL_858903, EPI_ISL_858921, EPI_ISL_858927, EPI_ISL_858969, EPI_ISL_859033, EPI_ISL_859055, EPI_ISL_859062, EPI_ISL_859078,
EPI_ISL_859119, EPI_ISL_859130, EPI_ISL_859150, EPI_ISL_859159, EPI_ISL_859170, EPI_ISL_859176, EPI_ISL_859214, EPI_ISL_859301, EPI_ISL_859458, EPI_ISL_859464, EPI_ISL_859467 to 859468, EPI_ISL_859477, EPI_ISL_859499, EPI_ISL_859514, EPI_ISL_859520, EPI_ISL_863245, EPI_ISL_863248 to 863249,
EPI_ISL_863252 to 863253, EPI_ISL_863255, EPI_ISL_863257, EPI_ISL_863262 to 863263, EPI_ISL_863265 to 863267, EPI_ISL_863269, EPI_ISL_863277 to 863278, EPI_ISL_863283, EPI_ISL_863285 to 863286, EPI_ISL_863288, EPI_ISL_863291, EPI_ISL_863294 to 863296, EPI_ISL_863300 to 863301, EPI_ISL_863304 to
863305, EPI_ISL_863308 to 863309, EPI_ISL_863312, EPI_ISL_863315, EPI_ISL_863317, EPI_ISL_863321 to 863324, EPI_ISL_863327 to 863329, EPI_ISL_863331 to 863333, EPI_ISL_863335, EPI_ISL_863337, EPI_ISL_863339 to 863340, EPI_ISL_863344 to 863345, EPI_ISL_863347, EPI_ISL_863349 to 863352,
EPI_ISL_863354, EPI_ISL_863358, EPI_ISL_863361, EPI_ISL_863366, EPI_ISL_863368, EPI_ISL_863370, EPI_ISL_863372 to 863374, EPI_ISL_863380 to 863382, EPI_ISL_863384, EPI_ISL_863386, EPI_ISL_863388, EPI_ISL_863390 to 863392, EPI_ISL_863398 to 863400, EPI_ISL_863402, EPI_ISL_863405,
EPI_ISL_863409, EPI_ISL_863413, EPI_ISL_863415, EPI_ISL_863422, EPI_ISL_863425, EPI_ISL_863427 to 863428, EPI_ISL_863430 to 863431, EPI_ISL_863433, EPI_ISL_863436, EPI_ISL_863439, EPI_ISL_863441, EPI_ISL_863444 to 863445, EPI_ISL_863449, EPI_ISL_863451 to 863453, EPI_ISL_863459,
EPI_ISL_863462, EPI_ISL_863464, EPI_ISL_863466, EPI_ISL_863468 to 863469, EPI_ISL_863471, EPI_ISL_863473 to 863474, EPI_ISL_863481, EPI_ISL_863483, EPI_ISL_863485, EPI_ISL_863487 to 863489, EPI_ISL_863492, EPI_ISL_863496 to 863497, EPI_ISL_863499 to 863500, EPI_ISL_863502, EPI_ISL_863505,
EPI_ISL_863507, EPI_ISL_863509, EPI_ISL_863511 to 863513, EPI_ISL_863517, EPI_ISL_863521 to 863522, EPI_ISL_863528, EPI_ISL_863530, EPI_ISL_863532, EPI_ISL_863635, EPI_ISL_863639, EPI_ISL_863645, EPI_ISL_863721, EPI_ISL_863729, EPI_ISL_863746, EPI_ISL_863830, EPI_ISL_863841, EPI_ISL_864543,
EPI_ISL_874656, EPI_ISL_874676, EPI_ISL_874736 to 874737, EPI_ISL_874739, EPI_ISL_874798, EPI_ISL_874806, EPI_ISL_874808, EPI_ISL_874863, EPI_ISL_874896, EPI_ISL_874922, EPI_ISL_874965, EPI_ISL_891279, EPI_ISL_891281, EPI_ISL_891283, EPI_ISL_891286, EPI_ISL_891288, EPI_ISL_891297,
EPI_ISL_891330, EPI_ISL_891335, EPI_ISL_891338, EPI_ISL_891345, EPI_ISL_891353, EPI_ISL_891361, EPI_ISL_891375, EPI_ISL_891380, EPI_ISL_891391, EPI_ISL_891393, EPI_ISL_891396, EPI_ISL_891410, EPI_ISL_891415, EPI_ISL_891426, EPI_ISL_891430, EPI_ISL_891435, EPI_ISL_891445 to 891446,
EPI_ISL_891453, EPI_ISL_891456, EPI_ISL_891462, EPI_ISL_891465, EPI_ISL_891469, EPI_ISL_891487, EPI_ISL_891491, EPI_ISL_891495, EPI_ISL_891497, EPI_ISL_891502, EPI_ISL_891505, EPI_ISL_891509, EPI_ISL_891516, EPI_ISL_891532, EPI_ISL_891538 to 891539, EPI_ISL_891571, EPI_ISL_891585,
EPI_ISL_891587 to 891588, EPI_ISL_891603 to 891605, EPI_ISL_891609 to 891610, EPI_ISL_891612, EPI_ISL_891626, EPI_ISL_891628, EPI_ISL_891636, EPI_ISL_891639, EPI_ISL_891656, EPI_ISL_891658, EPI_ISL_891664, EPI_ISL_891685, EPI_ISL_891689, EPI_ISL_891694, EPI_ISL_891700, EPI_ISL_891705,
EPI_ISL_891711, EPI_ISL_891717, EPI_ISL_891721 to 891722, EPI_ISL_891727, EPI_ISL_891740 to 891741, EPI_ISL_891747, EPI_ISL_891749, EPI_ISL_891769, EPI_ISL_891772, EPI_ISL_891791, EPI_ISL_891793, EPI_ISL_891802, EPI_ISL_891809, EPI_ISL_891819, EPI_ISL_891834, EPI_ISL_891836, EPI_ISL_891846,
EPI_ISL_891852, EPI_ISL_891855, EPI_ISL_891869, EPI_ISL_891873, EPI_ISL_891875, EPI_ISL_891881, EPI_ISL_891884 to 891885, EPI_ISL_892211, EPI_ISL_907120, EPI_ISL_907123 to 907126, EPI_ISL_907128 to 907132, EPI_ISL_907134 to 907135, EPI_ISL_907140 to 907143, EPI_ISL_907145 to 907146,
EPI_ISL_907149 to 907151, EPI_ISL_907153 to 907154, EPI_ISL_907156, EPI_ISL_907158 to 907159, EPI_ISL_907162, EPI_ISL_907165 to 907166, EPI_ISL_907169 to 907172, EPI_ISL_907178, EPI_ISL_907181, EPI_ISL_907185, EPI_ISL_907189, EPI_ISL_907196, EPI_ISL_907201, EPI_ISL_907204, EPI_ISL_907207 to
907208, EPI_ISL_907213, EPI_ISL_907215 to 907218, EPI_ISL_907220, EPI_ISL_907226, EPI_ISL_907228, EPI_ISL_907230, EPI_ISL_907234 to 907237, EPI_ISL_907239 to 907240, EPI_ISL_907242, EPI_ISL_907244, EPI_ISL_907247, EPI_ISL_907249, EPI_ISL_907252 to 907253, EPI_ISL_907255, EPI_ISL_907257 to
907258, EPI_ISL_907261 to 907262, EPI_ISL_907266, EPI_ISL_907270, EPI_ISL_907272, EPI_ISL_907274, EPI_ISL_907277, EPI_ISL_907280, EPI_ISL_907282 to 907283, EPI_ISL_907285 to 907286, EPI_ISL_907289 to 907291, EPI_ISL_907293 to 907295, EPI_ISL_907297 to 907299, EPI_ISL_907301, EPI_ISL_907305 to
907307, EPI_ISL_907310, EPI_ISL_907316, EPI_ISL_907319 to 907320, EPI_ISL_907324, EPI_ISL_907330 to 907331, EPI_ISL_907334 to 907335, EPI_ISL_907339 to 907340, EPI_ISL_907342 to 907344, EPI_ISL_907356 to 907358, EPI_ISL_907360 to 907361, EPI_ISL_907365, EPI_ISL_907368, EPI_ISL_907370,
EPI_ISL_907379, EPI_ISL_907394 to 907395, EPI_ISL_907401, EPI_ISL_907405, EPI_ISL_907408, EPI_ISL_907418 to 907421, EPI_ISL_907425 to 907426, EPI_ISL_907430 to 907431, EPI_ISL_907433 to 907436, EPI_ISL_907454 to 907456, EPI_ISL_907465, EPI_ISL_907473, EPI_ISL_907475, EPI_ISL_907481,
EPI_ISL_907486, EPI_ISL_907488, EPI_ISL_907490, EPI_ISL_907495, EPI_ISL_907498, EPI_ISL_907501 to 907502, EPI_ISL_907506, EPI_ISL_907514 to 907515, EPI_ISL_907520, EPI_ISL_907525, EPI_ISL_907527, EPI_ISL_907530, EPI_ISL_907534 to 907535, EPI_ISL_907539, EPI_ISL_907541, EPI_ISL_907544 to
907545, EPI_ISL_907548, EPI_ISL_907558, EPI_ISL_907560, EPI_ISL_907562, EPI_ISL_907564 to 907566, EPI_ISL_907570, EPI_ISL_907572, EPI_ISL_907577, EPI_ISL_907581, EPI_ISL_907585 to 907586, EPI_ISL_907588, EPI_ISL_907591 to 907593, EPI_ISL_907595, EPI_ISL_907597, EPI_ISL_907599, EPI_ISL_907603
to 907604, EPI_ISL_907608 to 907609, EPI_ISL_907613 to 907614, EPI_ISL_907617, EPI_ISL_907619 to 907620, EPI_ISL_907624 to 907625, EPI_ISL_907627, EPI_ISL_907629, EPI_ISL_907632, EPI_ISL_907634 to 907635, EPI_ISL_907637, EPI_ISL_907639, EPI_ISL_907643, EPI_ISL_907646, EPI_ISL_907649 to 907650,
EPI_ISL_907655, EPI_ISL_907657, EPI_ISL_907660, EPI_ISL_907662 to 907663, EPI_ISL_907665 to 907666, EPI_ISL_907668 to 907671, EPI_ISL_907674, EPI_ISL_907678 to 907679, EPI_ISL_907682 to 907683, EPI_ISL_907685, EPI_ISL_907687 to 907688, EPI_ISL_907705 to 907707, EPI_ISL_907721 to 907722,
EPI_ISL_907747, EPI_ISL_907749, EPI_ISL_907756, EPI_ISL_907763, EPI_ISL_907765, EPI_ISL_907772 to 907773, EPI_ISL_907786, EPI_ISL_907788 to 907789, EPI_ISL_907794 to 907796, EPI_ISL_907800 to 907802, EPI_ISL_907809, EPI_ISL_907812 to 907814, EPI_ISL_907817, EPI_ISL_907821, EPI_ISL_907835,
EPI_ISL_907837, EPI_ISL_907860, EPI_ISL_907863, EPI_ISL_907872, EPI_ISL_907874 to 907875, EPI_ISL_907877, EPI_ISL_907882, EPI_ISL_907891, EPI_ISL_907894 to 907895, EPI_ISL_907898 to 907899, EPI_ISL_907903, EPI_ISL_907912, EPI_ISL_907914 to 907915, EPI_ISL_907921, EPI_ISL_907936,
EPI_ISL_907940, EPI_ISL_907946, EPI_ISL_907949, EPI_ISL_907953, EPI_ISL_907956, EPI_ISL_907960, EPI_ISL_907967, EPI_ISL_907972, EPI_ISL_907975 to 907976, EPI_ISL_907979, EPI_ISL_907981 to 907982, EPI_ISL_907987 to 907989, EPI_ISL_907994 to 907995, EPI_ISL_907998, EPI_ISL_908002 to 908003,
EPI_ISL_908016 to 908017, EPI_ISL_908022, EPI_ISL_908025 to 908026, EPI_ISL_908028, EPI_ISL_908051, EPI_ISL_908054, EPI_ISL_908061, EPI_ISL_908064, EPI_ISL_908072 to 908075, EPI_ISL_908081, EPI_ISL_908087, EPI_ISL_908094, EPI_ISL_908097, EPI_ISL_908104, EPI_ISL_908113, EPI_ISL_908116,
EPI_ISL_908122, EPI_ISL_908131, EPI_ISL_908151, EPI_ISL_908168, EPI_ISL_908171, EPI_ISL_908177, EPI_ISL_908179, EPI_ISL_908181, EPI_ISL_908187, EPI_ISL_908194, EPI_ISL_908200, EPI_ISL_908208, EPI_ISL_908214, EPI_ISL_908218, EPI_ISL_908229, EPI_ISL_908231, EPI_ISL_908237 to 908238,
EPI_ISL_908241, EPI_ISL_908243, EPI_ISL_908256, EPI_ISL_908260 to 908262, EPI_ISL_908265 to 908266, EPI_ISL_908289, EPI_ISL_908298, EPI_ISL_908302 to 908303, EPI_ISL_908307 to 908308, EPI_ISL_908323, EPI_ISL_908326, EPI_ISL_908337 to 908338, EPI_ISL_908340, EPI_ISL_908357, EPI_ISL_908366,
EPI_ISL_908387, EPI_ISL_908394, EPI_ISL_908403, EPI_ISL_908411 to 908412, EPI_ISL_908416, EPI_ISL_908421, EPI_ISL_908429, EPI_ISL_908431, EPI_ISL_908442 to 908444, EPI_ISL_908447, EPI_ISL_908451, EPI_ISL_908455, EPI_ISL_908470, EPI_ISL_908486, EPI_ISL_908489, EPI_ISL_908495 to 908496,
EPI_ISL_908507, EPI_ISL_908512, EPI_ISL_908516, EPI_ISL_908521 to 908522, EPI_ISL_908530, EPI_ISL_908538, EPI_ISL_908557, EPI_ISL_908564, EPI_ISL_908568, EPI_ISL_908573, EPI_ISL_908589, EPI_ISL_908958, EPI_ISL_908962, EPI_ISL_908965, EPI_ISL_908974, EPI_ISL_908982, EPI_ISL_908989,
EPI_ISL_908993, EPI_ISL_909002, EPI_ISL_909004 to 909005, EPI_ISL_909008, EPI_ISL_909010, EPI_ISL_909029 to 909030, EPI_ISL_909034, EPI_ISL_909038, EPI_ISL_909053, EPI_ISL_909056, EPI_ISL_909058, EPI_ISL_909063, EPI_ISL_909068 to 909069, EPI_ISL_909083, EPI_ISL_909085 to 909087,
EPI_ISL_909090, EPI_ISL_909094, EPI_ISL_909113 to 909114, EPI_ISL_909117 to 909118, EPI_ISL_909121, EPI_ISL_909150, EPI_ISL_909158, EPI_ISL_909162, EPI_ISL_909165, EPI_ISL_909169 to 909171, EPI_ISL_909176 to 909177, EPI_ISL_909184, EPI_ISL_909194, EPI_ISL_909206, EPI_ISL_909208,
EPI_ISL_909210 to 909211, EPI_ISL_909219, EPI_ISL_909227, EPI_ISL_909233, EPI_ISL_909235 to 909236, EPI_ISL_909255, EPI_ISL_909257, EPI_ISL_909262, EPI_ISL_909272, EPI_ISL_909283, EPI_ISL_909289, EPI_ISL_909303 to 909304, EPI_ISL_909306, EPI_ISL_909317, EPI_ISL_909325, EPI_ISL_909342,
EPI_ISL_909351, EPI_ISL_909353, EPI_ISL_909356, EPI_ISL_909374, EPI_ISL_909376, EPI_ISL_909382, EPI_ISL_909387, EPI_ISL_909390 to 909391, EPI_ISL_909393, EPI_ISL_909403, EPI_ISL_909405, EPI_ISL_909407, EPI_ISL_909422, EPI_ISL_909428 to 909429, EPI_ISL_909435, EPI_ISL_909438, EPI_ISL_909490,
EPI_ISL_909496, EPI_ISL_909508, EPI_ISL_909528, EPI_ISL_909532, EPI_ISL_909537 to 909538, EPI_ISL_909549, EPI_ISL_909554, EPI_ISL_909557, EPI_ISL_909570, EPI_ISL_909588 to 909590, EPI_ISL_912212, EPI_ISL_912219, EPI_ISL_916503, EPI_ISL_916505, EPI_ISL_916507, EPI_ISL_916509 to 916515,
EPI_ISL_916517 to 916523, EPI_ISL_916527, EPI_ISL_916530 to 916532, EPI_ISL_916534 to 916535, EPI_ISL_916538 to 916541, EPI_ISL_916544, EPI_ISL_916547 to 916548, EPI_ISL_916550, EPI_ISL_916552 to 916553, EPI_ISL_916557, EPI_ISL_916561, EPI_ISL_916564, EPI_ISL_916567 to 916569, EPI_ISL_916571 to
916574, EPI_ISL_916577, EPI_ISL_916580, EPI_ISL_916582 to 916583, EPI_ISL_916585, EPI_ISL_916588, EPI_ISL_916590 to 916591, EPI_ISL_916593 to 916598, EPI_ISL_916600 to 916603, EPI_ISL_916607 to 916610, EPI_ISL_916612, EPI_ISL_916616 to 916617, EPI_ISL_916619, EPI_ISL_916621, EPI_ISL_916625 to
916626, EPI_ISL_916633, EPI_ISL_916645, EPI_ISL_916648, EPI_ISL_916661 to 916662, EPI_ISL_916668, EPI_ISL_916688, EPI_ISL_916694, EPI_ISL_916696, EPI_ISL_916704, EPI_ISL_916711, EPI_ISL_916716, EPI_ISL_916728, EPI_ISL_916730, EPI_ISL_916736, EPI_ISL_916761, EPI_ISL_916800, EPI_ISL_916809 to
916810, EPI_ISL_916812, EPI_ISL_916815, EPI_ISL_916821, EPI_ISL_916825, EPI_ISL_916834, EPI_ISL_916855, EPI_ISL_916869, EPI_ISL_916882, EPI_ISL_916891, EPI_ISL_916898, EPI_ISL_916905, EPI_ISL_916907 to 916908, EPI_ISL_916911 to 916912, EPI_ISL_916916, EPI_ISL_916918, EPI_ISL_916946,
EPI_ISL_916948 to 916949, EPI_ISL_916951, EPI_ISL_916953 to 916954, EPI_ISL_916961, EPI_ISL_916967 to 916968, EPI_ISL_916976, EPI_ISL_916979, EPI_ISL_916981, EPI_ISL_916985, EPI_ISL_917002, EPI_ISL_917021, EPI_ISL_917025, EPI_ISL_917029, EPI_ISL_917032, EPI_ISL_917036, EPI_ISL_917038,
EPI_ISL_917047, EPI_ISL_917049, EPI_ISL_917054 to 917055, EPI_ISL_917063, EPI_ISL_917067, EPI_ISL_917071, EPI_ISL_917074, EPI_ISL_917076 to 917077, EPI_ISL_917079, EPI_ISL_917082 to 917083, EPI_ISL_917086, EPI_ISL_917088, EPI_ISL_917091, EPI_ISL_917093, EPI_ISL_917096, EPI_ISL_917099,
EPI_ISL_917105, EPI_ISL_917112, EPI_ISL_917120, EPI_ISL_917125, EPI_ISL_917139, EPI_ISL_917141, EPI_ISL_917143 to 917145, EPI_ISL_917148, EPI_ISL_917156, EPI_ISL_917159, EPI_ISL_917161, EPI_ISL_917163, EPI_ISL_917844, EPI_ISL_917847, EPI_ISL_917850 to 917851, EPI_ISL_917853 to 917864,
EPI_ISL_917866 to 917868, EPI_ISL_917873 to 917880, EPI_ISL_917882 to 917893, EPI_ISL_917896 to 917898, EPI_ISL_917900 to 917906, EPI_ISL_917909 to 917910, EPI_ISL_917912 to 917917, EPI_ISL_917920 to 917927, EPI_ISL_917934, EPI_ISL_917939, EPI_ISL_917942, EPI_ISL_917957 to 917959, EPI_ISL_917961,
EPI_ISL_917963, EPI_ISL_917965, EPI_ISL_917967 to 917968, EPI_ISL_917970 to 917973, EPI_ISL_917977 to 917982, EPI_ISL_917984, EPI_ISL_917986, EPI_ISL_917988, EPI_ISL_917992, EPI_ISL_917994 to 917999, EPI_ISL_918001 to 918002, EPI_ISL_918006, EPI_ISL_918010 to 918011, EPI_ISL_918013,
EPI_ISL_918015, EPI_ISL_918020 to 918021, EPI_ISL_918023, EPI_ISL_918025 to 918029, EPI_ISL_918032 to 918033, EPI_ISL_918036 to 918044, EPI_ISL_918046 to 918048, EPI_ISL_918051 to 918057, EPI_ISL_918059, EPI_ISL_918062 to 918067, EPI_ISL_918070 to 918071, EPI_ISL_918073 to 918075, EPI_ISL_918080,
EPI_ISL_918083 to 918087, EPI_ISL_918089, EPI_ISL_918091 to 918092, EPI_ISL_918096, EPI_ISL_918098 to 918099, EPI_ISL_918101 to 918103, EPI_ISL_918105 to 918107, EPI_ISL_918109, EPI_ISL_918111 to 918115, EPI_ISL_918118 to 918123, EPI_ISL_932252, EPI_ISL_932256, EPI_ISL_932265 to 932267,
EPI_ISL_932269, EPI_ISL_932271 to 932272, EPI_ISL_932275, EPI_ISL_932277, EPI_ISL_932281, EPI_ISL_932286, EPI_ISL_932293, EPI_ISL_932295 to 932296, EPI_ISL_932299, EPI_ISL_932301, EPI_ISL_932304, EPI_ISL_932308, EPI_ISL_932311 to 932312, EPI_ISL_932316 to 932318, EPI_ISL_932322 to 932323,
EPI_ISL_932326, EPI_ISL_932328, EPI_ISL_932330 to 932331, EPI_ISL_932335, EPI_ISL_932337, EPI_ISL_932341, EPI_ISL_932351 to 932353, EPI_ISL_932355, EPI_ISL_932359 to 932361, EPI_ISL_932370, EPI_ISL_932381, EPI_ISL_932397, EPI_ISL_932401, EPI_ISL_932404 to 932405, EPI_ISL_932409 to 932410,
EPI_ISL_932415, EPI_ISL_932418, EPI_ISL_932423 to 932424, EPI_ISL_932427, EPI_ISL_932431 to 932432, EPI_ISL_932438, EPI_ISL_932448 to 932449, EPI_ISL_932451 to 932452, EPI_ISL_932454, EPI_ISL_932459, EPI_ISL_932461, EPI_ISL_932466, EPI_ISL_932482, EPI_ISL_932487, EPI_ISL_932490,
EPI_ISL_932493 to 932494, EPI_ISL_932497, EPI_ISL_932499 to 932501, EPI_ISL_932518, EPI_ISL_932524, EPI_ISL_932531, EPI_ISL_932534, EPI_ISL_932536 to 932538, EPI_ISL_932540, EPI_ISL_932545, EPI_ISL_932558, EPI_ISL_932564, EPI_ISL_932568, EPI_ISL_932606, EPI_ISL_932711, EPI_ISL_932746,
EPI_ISL_932764, EPI_ISL_932831, EPI_ISL_932857 to 932858, EPI_ISL_932863, EPI_ISL_932865, EPI_ISL_932879, EPI_ISL_932884 to 932885, EPI_ISL_932894, EPI_ISL_932896 to 932897, EPI_ISL_932899, EPI_ISL_932903, EPI_ISL_932905, EPI_ISL_932908, EPI_ISL_932914, EPI_ISL_932917, EPI_ISL_932921,
EPI_ISL_932925, EPI_ISL_932929 to 932931, EPI_ISL_932936, EPI_ISL_932944, EPI_ISL_932948, EPI_ISL_932955, EPI_ISL_932958, EPI_ISL_932965 to 932967, EPI_ISL_932980, EPI_ISL_932986, EPI_ISL_932994, EPI_ISL_933000, EPI_ISL_933004, EPI_ISL_933006, EPI_ISL_933009, EPI_ISL_933011, EPI_ISL_933018,
EPI_ISL_933020 to 933021, EPI_ISL_933035 to 933037, EPI_ISL_933040, EPI_ISL_933042, EPI_ISL_933050 to 933051, EPI_ISL_933054, EPI_ISL_933056, EPI_ISL_933058, EPI_ISL_933067, EPI_ISL_933069, EPI_ISL_933073 to 933074, EPI_ISL_933079, EPI_ISL_933099, EPI_ISL_933109 to 933110, EPI_ISL_933493 to
933494, EPI_ISL_939284, EPI_ISL_939295, EPI_ISL_939297 to 939299, EPI_ISL_939303, EPI_ISL_939309 to 939312, EPI_ISL_939316, EPI_ISL_939322, EPI_ISL_939324 to 939326, EPI_ISL_939328, EPI_ISL_939330 to 939331, EPI_ISL_939333, EPI_ISL_939335 to 939336, EPI_ISL_939338, EPI_ISL_939341 to 939342,
EPI_ISL_939344 to 939345, EPI_ISL_939347, EPI_ISL_939349, EPI_ISL_939353, EPI_ISL_939360, EPI_ISL_939362 to 939363, EPI_ISL_939367, EPI_ISL_939374 to 939377, EPI_ISL_939379, EPI_ISL_939381, EPI_ISL_939383 to 939386, EPI_ISL_939392, EPI_ISL_939394, EPI_ISL_939400, EPI_ISL_939409 to 939410,
EPI_ISL_939417, EPI_ISL_939419, EPI_ISL_939427, EPI_ISL_939434 to 939437, EPI_ISL_939440 to 939442, EPI_ISL_939444 to 939445, EPI_ISL_939447 to 939448, EPI_ISL_939451 to 939452, EPI_ISL_939455, EPI_ISL_939463 to 939465, EPI_ISL_939467, EPI_ISL_939471, EPI_ISL_939474, EPI_ISL_939479 to 939480,
EPI_ISL_939482, EPI_ISL_939484, EPI_ISL_939486, EPI_ISL_939488 to 939489, EPI_ISL_939491, EPI_ISL_939496, EPI_ISL_939499, EPI_ISL_939502, EPI_ISL_939511, EPI_ISL_939515, EPI_ISL_939518, EPI_ISL_939527, EPI_ISL_939529 to 939531, EPI_ISL_939538 to 939540, EPI_ISL_939544 to 939545,
EPI_ISL_939549, EPI_ISL_939553, EPI_ISL_939558, EPI_ISL_939564, EPI_ISL_939569 to 939570, EPI_ISL_939575, EPI_ISL_939580, EPI_ISL_939583, EPI_ISL_939586, EPI_ISL_939590, EPI_ISL_939592, EPI_ISL_939602, EPI_ISL_944793, EPI_ISL_944795, EPI_ISL_944797, EPI_ISL_944800, EPI_ISL_944803,
EPI_ISL_944805 to 944813, EPI_ISL_944817, EPI_ISL_944819 to 944822, EPI_ISL_944825 to 944826, EPI_ISL_944828 to 944831, EPI_ISL_944834 to 944839, EPI_ISL_944842, EPI_ISL_944844 to 944854, EPI_ISL_944856 to 944860, EPI_ISL_944864 to 944865, EPI_ISL_944867, EPI_ISL_944869, EPI_ISL_944871 to 944872,
EPI_ISL_944876, EPI_ISL_944879 to 944880, EPI_ISL_944883, EPI_ISL_944885 to 944889, EPI_ISL_944891, EPI_ISL_944895, EPI_ISL_944897 to 944898, EPI_ISL_944900 to 944902, EPI_ISL_944904 to 944906, EPI_ISL_944908 to 944912, EPI_ISL_944915, EPI_ISL_944917 to 944923, EPI_ISL_944925 to 944930,
EPI_ISL_944932 to 944936, EPI_ISL_944939, EPI_ISL_944941, EPI_ISL_944943 to 944951, EPI_ISL_944953 to 944959, EPI_ISL_944961 to 944962, EPI_ISL_944964 to 944971, EPI_ISL_944973 to 944975, EPI_ISL_944978, EPI_ISL_944980 to 944982, EPI_ISL_944984 to 944986, EPI_ISL_944988 to 944990, EPI_ISL_944993
to 944995, EPI_ISL_944998 to 944999, EPI_ISL_945001 to 945005, EPI_ISL_945009, EPI_ISL_945011 to 945012, EPI_ISL_945014 to 945015, EPI_ISL_945017 to 945023, EPI_ISL_945025 to 945027, EPI_ISL_945029 to 945033, EPI_ISL_945035, EPI_ISL_945038, EPI_ISL_945040, EPI_ISL_945042, EPI_ISL_945044 to 945046,
EPI_ISL_945048, EPI_ISL_945065, EPI_ISL_945126, EPI_ISL_945136, EPI_ISL_945162, EPI_ISL_945209, EPI_ISL_945234, EPI_ISL_945718, EPI_ISL_945722, EPI_ISL_945726, EPI_ISL_945728 to 945730, EPI_ISL_945735 to 945737, EPI_ISL_945741 to 945744, EPI_ISL_945747, EPI_ISL_945755, EPI_ISL_945757,

EPI_ISL_945759, EPI_ISL_945761, EPI_ISL_945763, EPI_ISL_945768, EPI_ISL_945771 to 945772, EPI_ISL_945776 to 945777, EPI_ISL_945781, EPI_ISL_945788, EPI_ISL_945792, EPI_ISL_945795 to 945796, EPI_ISL_945800, EPI_ISL_945802, EPI_ISL_945804, EPI_ISL_945808 to 945809, EPI_ISL_945812 to 945813, EPI_ISL_945816 to 945817, EPI_ISL_945821, EPI_ISL_945824, EPI_ISL_945826, EPI_ISL_945828, EPI_ISL_945841, EPI_ISL_945843 to 945844, EPI_ISL_945846, EPI_ISL_945848 to 945851, EPI_ISL_945858, EPI_ISL_945860, EPI_ISL_945862 to 945863, EPI_ISL_945865, EPI_ISL_945867 to 945868, EPI_ISL_945870 to 945871, EPI_ISL_945874 to 945875, EPI_ISL_945877 to 945879, EPI_ISL_945882, EPI_ISL_945885, EPI_ISL_945897 to 945900, EPI_ISL_945904, EPI_ISL_945906 to 945910, EPI_ISL_945920 to 945921, EPI_ISL_945926, EPI_ISL_945933, EPI_ISL_945938 to 945940, EPI_ISL_945944, EPI_ISL_945950 to 945951, EPI_ISL_945954, EPI_ISL_945958, EPI_ISL_945961, EPI_ISL_945963, EPI_ISL_945965, EPI_ISL_945969, EPI_ISL_945971 to 945972, EPI_ISL_945980, EPI_ISL_945988, EPI_ISL_945990, EPI_ISL_945993 to 945994, EPI_ISL_945997 to 945998, EPI_ISL_946000, EPI_ISL_946002, EPI_ISL_946005 to 946006, EPI_ISL_946008 to 946010, EPI_ISL_946014, EPI_ISL_946023, EPI_ISL_946028, EPI_ISL_946032, EPI_ISL_946037, EPI_ISL_946041, EPI_ISL_946047 to 946049, EPI_ISL_946052, EPI_ISL_946055, EPI_ISL_946057 to 946058, EPI_ISL_946066 to 946068, EPI_ISL_946072, EPI_ISL_946086 to 946088, EPI_ISL_946090, EPI_ISL_946093, EPI_ISL_946096, EPI_ISL_946106, EPI_ISL_946110, EPI_ISL_946112 to 946113, EPI_ISL_946115, EPI_ISL_946117, EPI_ISL_946123 to 946124, EPI_ISL_946126 to 946127, EPI_ISL_946135, EPI_ISL_946145, EPI_ISL_946147, EPI_ISL_946150, EPI_ISL_946157, EPI_ISL_946160, EPI_ISL_946162, EPI_ISL_946169, EPI_ISL_946173 to 946174, EPI_ISL_946181 to 946182, EPI_ISL_946190, EPI_ISL_946192, EPI_ISL_946196, EPI_ISL_946206, EPI_ISL_946208, EPI_ISL_946210 to 946211, EPI_ISL_946213, EPI_ISL_946215, EPI_ISL_946218, EPI_ISL_946220, EPI_ISL_946224 to 946225, EPI_ISL_946227, EPI_ISL_946229, EPI_ISL_946232 to 946234, EPI_ISL_946243, EPI_ISL_946254, EPI_ISL_946259 to 946260, EPI_ISL_946264, EPI_ISL_946270 to 946271, EPI_ISL_946274, EPI_ISL_946281, EPI_ISL_946284 to 946286, EPI_ISL_946289 to 946290, EPI_ISL_946292, EPI_ISL_946298, EPI_ISL_946301 to 946304, EPI_ISL_946326, EPI_ISL_946328, EPI_ISL_946333, EPI_ISL_946335, EPI_ISL_946338 to 946339, EPI_ISL_946351, EPI_ISL_946356, EPI_ISL_946360 to 946361, EPI_ISL_946365 to 946367, EPI_ISL_946371, EPI_ISL_946373, EPI_ISL_946375, EPI_ISL_946378 to 946380, EPI_ISL_946385, EPI_ISL_946391, EPI_ISL_946395, EPI_ISL_946398, EPI_ISL_946400, EPI_ISL_946405, EPI_ISL_946407 to 946408, EPI_ISL_946410 to 946411, EPI_ISL_946419, EPI_ISL_947108, EPI_ISL_947118 to 947119, EPI_ISL_947126, EPI_ISL_947131, EPI_ISL_947134, EPI_ISL_947137, EPI_ISL_947141, EPI_ISL_947153, EPI_ISL_947165, EPI_ISL_947169, EPI_ISL_947179, EPI_ISL_947182, EPI_ISL_947186, EPI_ISL_947191, EPI_ISL_947197, EPI_ISL_947204, EPI_ISL_947214, EPI_ISL_947229 to 947230, EPI_ISL_947236, EPI_ISL_947239, EPI_ISL_947245, EPI_ISL_947263, EPI_ISL_947267, EPI_ISL_947269, EPI_ISL_947280, EPI_ISL_947283, EPI_ISL_947294 to 947295, EPI_ISL_947314, EPI_ISL_947316, EPI_ISL_947331 to 947332, EPI_ISL_947335, EPI_ISL_947338, EPI_ISL_947347, EPI_ISL_947350, EPI_ISL_947352, EPI_ISL_947357, EPI_ISL_947364 to 947365, EPI_ISL_947370, EPI_ISL_947377, EPI_ISL_947386, EPI_ISL_947391, EPI_ISL_947394, EPI_ISL_947396, EPI_ISL_947398, EPI_ISL_947404 to 947405, EPI_ISL_948100, EPI_ISL_948103, EPI_ISL_948109, EPI_ISL_948111, EPI_ISL_948113, EPI_ISL_948115, EPI_ISL_948119, EPI_ISL_948126 to 948127, EPI_ISL_948130, EPI_ISL_948140, EPI_ISL_948146, EPI_ISL_948154, EPI_ISL_948156 to 948158, EPI_ISL_948169 to 948171, EPI_ISL_948173, EPI_ISL_948175, EPI_ISL_948179, EPI_ISL_948196, EPI_ISL_948202, EPI_ISL_948206, EPI_ISL_948217 to 948218, EPI_ISL_948222, EPI_ISL_948225 to 948226, EPI_ISL_948232, EPI_ISL_948238, EPI_ISL_948243, EPI_ISL_948245, EPI_ISL_948248, EPI_ISL_948254, EPI_ISL_948256, EPI_ISL_948267 to 948268, EPI_ISL_948271 to 948272, EPI_ISL_948276, EPI_ISL_948285, EPI_ISL_948287, EPI_ISL_948290 to 948291, EPI_ISL_948293, EPI_ISL_948295, EPI_ISL_948299, EPI_ISL_948303, EPI_ISL_948307, EPI_ISL_948317 to 948320, EPI_ISL_948324, EPI_ISL_948329, EPI_ISL_948335, EPI_ISL_948343, EPI_ISL_948352, EPI_ISL_948354, EPI_ISL_948356 to 948358, EPI_ISL_948363, EPI_ISL_948365, EPI_ISL_948367, EPI_ISL_948370, EPI_ISL_948373, EPI_ISL_948375, EPI_ISL_948379, EPI_ISL_948384, EPI_ISL_948386, EPI_ISL_948394, EPI_ISL_948399, EPI_ISL_948401, EPI_ISL_948403 to 948405, EPI_ISL_948409, EPI_ISL_948411, EPI_ISL_948415 to 948418, EPI_ISL_948426, EPI_ISL_948429 to 948430, EPI_ISL_948432, EPI_ISL_948436, EPI_ISL_948439 to 948440, EPI_ISL_948443, EPI_ISL_948446 to 948448, EPI_ISL_948787 to 948793, EPI_ISL_948795 to 948799, EPI_ISL_948801, EPI_ISL_948804 to 948806, EPI_ISL_948809 to 948810, EPI_ISL_948812 to 948817, EPI_ISL_948819 to 948822, EPI_ISL_948824 to 948827, EPI_ISL_948829 to 948830, EPI_ISL_948832, EPI_ISL_948834 to 948839, EPI_ISL_948842 to 948845, EPI_ISL_948847 to 948848, EPI_ISL_948850 to 948854, EPI_ISL_948857 to 948861, EPI_ISL_948863 to 948869, EPI_ISL_948871, EPI_ISL_948873 to 948875, EPI_ISL_948877 to 948880, EPI_ISL_948883, EPI_ISL_948885 to 948886, EPI_ISL_948889 to 948891, EPI_ISL_948893 to 948897, EPI_ISL_948899 to 948908, EPI_ISL_948910 to 948917, EPI_ISL_948919, EPI_ISL_948921 to 948922, EPI_ISL_948924 to 948925, EPI_ISL_948928 to 948930, EPI_ISL_948932 to 948933, EPI_ISL_948935 to 948941, EPI_ISL_948946, EPI_ISL_948950 to 948951, EPI_ISL_948954 to 948955, EPI_ISL_948957 to 948958, EPI_ISL_948960 to 948965, EPI_ISL_948967 to 948972, EPI_ISL_948974, EPI_ISL_948976 to 948977, EPI_ISL_948979 to 948980, EPI_ISL_948983, EPI_ISL_948985, EPI_ISL_948993, EPI_ISL_948995, EPI_ISL_948998 to 948999, EPI_ISL_949002, EPI_ISL_949006, EPI_ISL_949009 to 949010, EPI_ISL_949014 to 949015, EPI_ISL_949017 to 949018, EPI_ISL_949020 to 949021, EPI_ISL_949023, EPI_ISL_949025 to 949027, EPI_ISL_949030 to 949031, EPI_ISL_949033 to 949034, EPI_ISL_949036 to 949037, EPI_ISL_949040, EPI_ISL_949043 to 949044, EPI_ISL_949047 to 949051, EPI_ISL_949053 to 949054, EPI_ISL_949056 to 949058, EPI_ISL_949061, EPI_ISL_949064, EPI_ISL_949066, EPI_ISL_957054, EPI_ISL_957061, EPI_ISL_957065, EPI_ISL_957067 to 957068, EPI_ISL_957071 to 957072, EPI_ISL_957075, EPI_ISL_957077, EPI_ISL_957080 to 957081, EPI_ISL_957083, EPI_ISL_957086, EPI_ISL_957089 to 957095, EPI_ISL_957097, EPI_ISL_957103, EPI_ISL_957105, EPI_ISL_957107, EPI_ISL_957110, EPI_ISL_957114 to 957115, EPI_ISL_957117, EPI_ISL_957121 to 957122, EPI_ISL_957124, EPI_ISL_957149 to 957151, EPI_ISL_957154, EPI_ISL_957157 to 957158, EPI_ISL_957160, EPI_ISL_957162, EPI_ISL_957166, EPI_ISL_957172, EPI_ISL_957177, EPI_ISL_957183, EPI_ISL_957187 to 957188, EPI_ISL_957190 to 957191, EPI_ISL_957194, EPI_ISL_957198, EPI_ISL_957200 to 957201, EPI_ISL_957205 to 957206, EPI_ISL_957208, EPI_ISL_957210 to 957212, EPI_ISL_957216, EPI_ISL_957219, EPI_ISL_957223 to 957225, EPI_ISL_957227, EPI_ISL_957229, EPI_ISL_957231, EPI_ISL_957237, EPI_ISL_957239, EPI_ISL_957242 to 957243, EPI_ISL_957248, EPI_ISL_957253 to 957257, EPI_ISL_957259, EPI_ISL_957263, EPI_ISL_957266, EPI_ISL_957270, EPI_ISL_957275 to 957276, EPI_ISL_957279, EPI_ISL_957283, EPI_ISL_957285 to 957287, EPI_ISL_957289, EPI_ISL_957291 to 957292, EPI_ISL_957294, EPI_ISL_957299, EPI_ISL_957304, EPI_ISL_957308, EPI_ISL_957310 to 957312, EPI_ISL_957314 to 957315, EPI_ISL_957322 to 957323, EPI_ISL_957330 to 957331, EPI_ISL_957334, EPI_ISL_957336 to 957337, EPI_ISL_957633, EPI_ISL_957637, EPI_ISL_957649, EPI_ISL_957651, EPI_ISL_957653, EPI_ISL_957663, EPI_ISL_957669, EPI_ISL_957673 to 957674, EPI_ISL_957678 to 957679, EPI_ISL_957681 to 957682, EPI_ISL_957694, EPI_ISL_957713, EPI_ISL_957715, EPI_ISL_957717 to 957718, EPI_ISL_957720, EPI_ISL_957725, EPI_ISL_957730, EPI_ISL_957732, EPI_ISL_957743, EPI_ISL_957749 to 957750, EPI_ISL_957756, EPI_ISL_957765, EPI_ISL_957768, EPI_ISL_957770, EPI_ISL_957773 to 957774, EPI_ISL_957778, EPI_ISL_957785, EPI_ISL_957793 to 957794, EPI_ISL_957796, EPI_ISL_957802, EPI_ISL_957814, EPI_ISL_957819, EPI_ISL_957836 to 957837, EPI_ISL_957839, EPI_ISL_957848, EPI_ISL_957851, EPI_ISL_957853 to 957854, EPI_ISL_957864 to 957865, EPI_ISL_957875, EPI_ISL_957879, EPI_ISL_957884, EPI_ISL_957889, EPI_ISL_957894, EPI_ISL_963326, EPI_ISL_963341, EPI_ISL_963344 to 963346, EPI_ISL_963348, EPI_ISL_963355 to 963358, EPI_ISL_963370 to 963371, EPI_ISL_963380, EPI_ISL_963386, EPI_ISL_963397, EPI_ISL_963419, EPI_ISL_963425 to 963426, EPI_ISL_963449, EPI_ISL_963452, EPI_ISL_963458, EPI_ISL_963475, EPI_ISL_963481, EPI_ISL_963495, EPI_ISL_963509, EPI_ISL_963520 to 963521, EPI_ISL_963529 to 963530, EPI_ISL_963538, EPI_ISL_963559 to 963560, EPI_ISL_963562, EPI_ISL_963570, EPI_ISL_963573, EPI_ISL_963576, EPI_ISL_963581, EPI_ISL_963584, EPI_ISL_963591, EPI_ISL_963594, EPI_ISL_963601, EPI_ISL_963618, EPI_ISL_963626, EPI_ISL_963633, EPI_ISL_963639, EPI_ISL_963641, EPI_ISL_964349, EPI_ISL_964351 to 964352, EPI_ISL_964355, EPI_ISL_964357 to 964359, EPI_ISL_964362, EPI_ISL_964364, EPI_ISL_964366, EPI_ISL_964368 to 964369, EPI_ISL_964371, EPI_ISL_964380, EPI_ISL_964382, EPI_ISL_964384, EPI_ISL_964387 to 964388, EPI_ISL_964393, EPI_ISL_964396 to 964397, EPI_ISL_964401, EPI_ISL_964404, EPI_ISL_964408 to 964409, EPI_ISL_964413, EPI_ISL_964415 to 964417, EPI_ISL_964421 to 964422, EPI_ISL_964424 to 964425, EPI_ISL_964427, EPI_ISL_964430, EPI_ISL_964433, EPI_ISL_964437, EPI_ISL_964439, EPI_ISL_964441 to 964442, EPI_ISL_964446, EPI_ISL_964452, EPI_ISL_964456 to 964458, EPI_ISL_964468 to 964469, EPI_ISL_964471 to 964473, EPI_ISL_964476, EPI_ISL_964480, EPI_ISL_964482 to 964484, EPI_ISL_964487 to 964489, EPI_ISL_964493 to 964494, EPI_ISL_964500 to 964501, EPI_ISL_964503 to 964504, EPI_ISL_964509, EPI_ISL_964511 to 964512, EPI_ISL_964516, EPI_ISL_964518, EPI_ISL_964520 to 964521, EPI_ISL_964523, EPI_ISL_964527 to 964528, EPI_ISL_964532, EPI_ISL_964537, EPI_ISL_964540 to 964541, EPI_ISL_969455, EPI_ISL_986104, EPI_ISL_986106, EPI_ISL_986109, EPI_ISL_986115, EPI_ISL_986117 to 986120, EPI_ISL_986125 to 986126, EPI_ISL_986128, EPI_ISL_986132 to 986134, EPI_ISL_986139, EPI_ISL_986144, EPI_ISL_986152, EPI_ISL_986154, EPI_ISL_986159, EPI_ISL_986165, EPI_ISL_986167, EPI_ISL_986169, EPI_ISL_986171 to 986172, EPI_ISL_986174, EPI_ISL_986180, EPI_ISL_986184 to 986185, EPI_ISL_986190 to 986191, EPI_ISL_986197, EPI_ISL_986199 to 986201, EPI_ISL_986204 to 986205, EPI_ISL_986208 to 986209, EPI_ISL_986212, EPI_ISL_986215, EPI_ISL_986217, EPI_ISL_986222, EPI_ISL_986229 to 986230, EPI_ISL_986234, EPI_ISL_986236, EPI_ISL_986238 to 986239, EPI_ISL_986241, EPI_ISL_986243 to 986244, EPI_ISL_986248 to 986250, EPI_ISL_986254, EPI_ISL_986257, EPI_ISL_986260, EPI_ISL_986262 to 986263, EPI_ISL_986265 to 986268, EPI_ISL_986270 to 986274, EPI_ISL_986276 to 986277, EPI_ISL_986279 to 986286, EPI_ISL_986288, EPI_ISL_986290 to 986293, EPI_ISL_986295, EPI_ISL_986297 to 986299, EPI_ISL_986301 to 986306, EPI_ISL_986308 to 986311, EPI_ISL_986314, EPI_ISL_986316 to 986317, EPI_ISL_986320 to 986324, EPI_ISL_986326 to 986327, EPI_ISL_986329 to 986334, EPI_ISL_986337 to 986341, EPI_ISL_986343 to 986348, EPI_ISL_986351, EPI_ISL_986353, EPI_ISL_986357 to 986361, EPI_ISL_986363 to 986364, EPI_ISL_986366 to 986370, EPI_ISL_986372 to 986375, EPI_ISL_986377 to 986382, EPI_ISL_986384 to 986387, EPI_ISL_986389 to 986396, EPI_ISL_986399 to 986400, EPI_ISL_986402 to 986423, EPI_ISL_986426 to 986436, EPI_ISL_986438, EPI_ISL_987311, EPI_ISL_987316 to 987317, EPI_ISL_987323 to 987324, EPI_ISL_987335, EPI_ISL_987337, EPI_ISL_987341, EPI_ISL_987347, EPI_ISL_987349 to 987350, EPI_ISL_987359, EPI_ISL_987362, EPI_ISL_987370, EPI_ISL_987373, EPI_ISL_987376, EPI_ISL_987385, EPI_ISL_987390, EPI_ISL_987394 to 987395, EPI_ISL_987404 to 987405, EPI_ISL_987422, EPI_ISL_987436 to 987437, EPI_ISL_987439 to 987440, EPI_ISL_987454, EPI_ISL_987461, EPI_ISL_987470, EPI_ISL_987483, EPI_ISL_987485, EPI_ISL_987491, EPI_ISL_987501, EPI_ISL_987503, EPI_ISL_987507, EPI_ISL_987511, EPI_ISL_987529, EPI_ISL_987535 to 987536, EPI_ISL_987549 to 987550, EPI_ISL_987552, EPI_ISL_987555 to 987556, EPI_ISL_987568, EPI_ISL_987570 to 987572, EPI_ISL_987574, EPI_ISL_987614, EPI_ISL_987616, EPI_ISL_987619, EPI_ISL_987621, EPI_ISL_989450 to 989453, EPI_ISL_989455 to 989456, EPI_ISL_989459 to 989463, EPI_ISL_989466 to 989474, EPI_ISL_989476 to 989481, EPI_ISL_989530 to 989533, EPI_ISL_989535 to 989536, EPI_ISL_989539 to 989540, EPI_ISL_989542 to 989543, EPI_ISL_989545 to 989555, EPI_ISL_989557 to 989564, EPI_ISL_989567, EPI_ISL_989569, EPI_ISL_989571 to 989577, EPI_ISL_989579 to 989581, EPI_ISL_989583, EPI_ISL_989585 to 989590, EPI_ISL_989592 to 989595, EPI_ISL_989597, EPI_ISL_989600, EPI_ISL_989602 to 989603, EPI_ISL_989605 to 989609, EPI_ISL_989611 to 989622, EPI_ISL_989624 to 989628, EPI_ISL_989631 to 989632, EPI_ISL_989635, EPI_ISL_989637 to 989651, EPI_ISL_989653, EPI_ISL_989655, EPI_ISL_989658 to 989660, EPI_ISL_989663 to 989664, EPI_ISL_989666 to 989668, EPI_ISL_989670 to 989676, EPI_ISL_989678 to 989688, EPI_ISL_989690 to 989692, EPI_ISL_989694 to 989695, EPI_ISL_989795 to 989811, EPI_ISL_989813 to 989819, EPI_ISL_989821 to 989825, EPI_ISL_989827 to 989830, EPI_ISL_989832 to 989833, EPI_ISL_989837 to 989838, EPI_ISL_989840 to 989842, EPI_ISL_989844 to 989845, EPI_ISL_989847, EPI_ISL_989852, EPI_ISL_989854 to 989855, EPI_ISL_989882 to 989887, EPI_ISL_989890 to 989892, EPI_ISL_989894, EPI_ISL_989896, EPI_ISL_989898 to 989902, EPI_ISL_989904 to 989908, EPI_ISL_989910 to 989912, EPI_ISL_989914 to 989918, EPI_ISL_989920, EPI_ISL_989922 to 989935, EPI_ISL_989937 to 989952, EPI_ISL_989954, EPI_ISL_989956, EPI_ISL_989958 to 989960, EPI_ISL_989962 to 989964, EPI_ISL_989967 to 989970, EPI_ISL_989972 to 989974, EPI_ISL_989976 to 989978, EPI_ISL_989981 to 989982, EPI_ISL_989986 to 989987, EPI_ISL_989996, EPI_ISL_990058, EPI_ISL_990060 to 990075, EPI_ISL_990078 to 990091, EPI_ISL_990093 to 990094, EPI_ISL_990096, EPI_ISL_990098, EPI_ISL_990100, EPI_ISL_990103 to 990108, EPI_ISL_990110 to 990119, EPI_ISL_990122 to 990125, EPI_ISL_990127 to 990134, EPI_ISL_990136 to 990137, EPI_ISL_990139 to 990140, EPI_ISL_991352 to 991353, EPI_ISL_991356, EPI_ISL_991361, EPI_ISL_991363, EPI_ISL_991365, EPI_ISL_991371, EPI_ISL_991373 to 991375, EPI_ISL_991384, EPI_ISL_991390, EPI_ISL_991393, EPI_ISL_991396, EPI_ISL_991398, EPI_ISL_991400 to 991402, EPI_ISL_991404 to 991408, EPI_ISL_991410, EPI_ISL_991413 to 991415, EPI_ISL_991420 to 991421, EPI_ISL_991423 to 991425, EPI_ISL_991427, EPI_ISL_991429 to 991430, EPI_ISL_991438 to 991442, EPI_ISL_991444 to 991445, EPI_ISL_991447, EPI_ISL_991449, EPI_ISL_991459, EPI_ISL_991463, EPI_ISL_991466, EPI_ISL_991468 to 991469, EPI_ISL_991471, EPI_ISL_991474, EPI_ISL_991477, EPI_ISL_991479, EPI_ISL_991481, EPI_ISL_991484 to 991485, EPI_ISL_991487, EPI_ISL_991495, EPI_ISL_991497, EPI_ISL_991501, EPI_ISL_991508 to 991510, EPI_ISL_991513 to 991514, EPI_ISL_991516, EPI_ISL_991519 to 991524, EPI_ISL_991529, EPI_ISL_991533, EPI_ISL_991535, EPI_ISL_991541 to 991542, EPI_ISL_991545, EPI_ISL_991550, EPI_ISL_991552, EPI_ISL_991559 to 991560, EPI_ISL_991562 to 991563, EPI_ISL_991904, EPI_ISL_992047, EPI_ISL_992049, EPI_ISL_992056, EPI_ISL_992060, EPI_ISL_992065, EPI_ISL_992070, EPI_ISL_992072, EPI_ISL_992075, EPI_ISL_992084, EPI_ISL_992086, EPI_ISL_992088, EPI_ISL_992093, EPI_ISL_992095 to 992096, EPI_ISL_992106 to 992107, EPI_ISL_992112 to 992113, EPI_ISL_992120, EPI_ISL_992123, EPI_ISL_992126, EPI_ISL_992132 to 992134, EPI_ISL_992137, EPI_ISL_992139, EPI_ISL_992141 to 992142, EPI_ISL_992145, EPI_ISL_992148, EPI_ISL_992150, EPI_ISL_992160, EPI_ISL_992162 to 992163, EPI_ISL_992165, EPI_ISL_992167 to 992170, EPI_ISL_992174, EPI_ISL_992176, EPI_ISL_992182, EPI_ISL_992185, EPI_ISL_992191 to 992195, EPI_ISL_992199 to 992202, EPI_ISL_992212, EPI_ISL_992216 to 992217, EPI_ISL_992222, EPI_ISL_992224, EPI_ISL_992228, EPI_ISL_992230 to 992233, EPI_ISL_992239, EPI_ISL_992242, EPI_ISL_992244 to 992245, EPI_ISL_992247 to 992249, EPI_ISL_992251, EPI_ISL_992254 to 992256, EPI_ISL_992266, EPI_ISL_992268, EPI_ISL_992270, EPI_ISL_992274, EPI_ISL_992278, EPI_ISL_992281, EPI_ISL_992290, EPI_ISL_992296 to 992300, EPI_ISL_992307 to 992308, EPI_ISL_992319 to 992320, EPI_ISL_992325 to 992326, EPI_ISL_992328, EPI_ISL_992339 to 992343, EPI_ISL_992347, EPI_ISL_994068 to 994069, EPI_ISL_994073, EPI_ISL_994075, EPI_ISL_994080, EPI_ISL_994082, EPI_ISL_994084 to 994085, EPI_ISL_994087, EPI_ISL_994091, EPI_ISL_994094, EPI_ISL_994101, EPI_ISL_994104, EPI_ISL_994106 to 994107, EPI_ISL_994110, EPI_ISL_994116 to 994117, EPI_ISL_994123 to 994124, EPI_ISL_994127, EPI_ISL_994129, EPI_ISL_994132, EPI_ISL_994134 to 994135, EPI_ISL_994137, EPI_ISL_994142, EPI_ISL_994144, EPI_ISL_994146 to 994148, EPI_ISL_994150, EPI_ISL_994152, EPI_ISL_994154, EPI_ISL_994156 to 994158, EPI_ISL_994161, EPI_ISL_994165 to 994166, EPI_ISL_994168, EPI_ISL_994170 to 994171, EPI_ISL_994174 to 994175, EPI_ISL_994177, EPI_ISL_994183 to 994184, EPI_ISL_994186, EPI_ISL_994188, EPI_ISL_994192 to 994193, EPI_ISL_994195 to 994197, EPI_ISL_994200 to 994201, EPI_ISL_994204, EPI_ISL_994206, EPI_ISL_994209 to 994211, EPI_ISL_994213 to 994217, EPI_ISL_994219, EPI_ISL_994222, EPI_ISL_994224, EPI_ISL_994227, EPI_ISL_994229 to 994230, EPI_ISL_994237, EPI_ISL_994243 to 994244, EPI_ISL_994247 to 994249, EPI_ISL_994251 to 994252, EPI_ISL_994255 to 994257, EPI_ISL_994260, EPI_ISL_994263 to 994267, EPI_ISL_994269 to 994270, EPI_ISL_994272, EPI_ISL_994275, EPI_ISL_994277, EPI_ISL_994279, EPI_ISL_994282 to 994286, EPI_ISL_994316, EPI_ISL_994326, EPI_ISL_994333, EPI_ISL_994336, EPI_ISL_994347, EPI_ISL_994353, EPI_ISL_994365, EPI_ISL_994367, EPI_ISL_994371, EPI_ISL_994377, EPI_ISL_994439, EPI_ISL_994443, EPI_ISL_994450, EPI_ISL_994486, EPI_ISL_994490, EPI_ISL_994502, EPI_ISL_994510, EPI_ISL_994518, EPI_ISL_994523, EPI_ISL_994528, EPI_ISL_994537, EPI_ISL_994542, EPI_ISL_994546, EPI_ISL_1005966, EPI_ISL_1005968, EPI_ISL_1005981, EPI_ISL_1005989, EPI_ISL_1005995, EPI_ISL_1006018, EPI_ISL_1006020, EPI_ISL_1006025, EPI_ISL_1006030, EPI_ISL_1006046 to 1006047, EPI_ISL_1006052, EPI_ISL_1006066, EPI_ISL_1006070, EPI_ISL_1006073, EPI_ISL_1006077, EPI_ISL_1006080, EPI_ISL_1006083, EPI_ISL_1006087, EPI_ISL_1006094, EPI_ISL_1006108, EPI_ISL_1006140, EPI_ISL_1006150, EPI_ISL_1006164, EPI_ISL_1006168 to 1006169, EPI_ISL_1007562, EPI_ISL_1007578, EPI_ISL_1012808, EPI_ISL_1012865, EPI_ISL_1019507 to 1019510, EPI_ISL_1019512 to 1019513, EPI_ISL_1019516, EPI_ISL_1019518 to 1019528, EPI_ISL_1019530 to 1019544, EPI_ISL_1019546 to 1019571, EPI_ISL_1019573 to 1019575, EPI_ISL_1019577 to 1019582, EPI_ISL_1019584 to 1019585, EPI_ISL_1019587 to 1019590, EPI_ISL_1019592 to 1019593, EPI_ISL_1019595, EPI_ISL_1019597 to 1019598, EPI_ISL_1019600 to 1019601, EPI_ISL_1019603, EPI_ISL_1019605 to 1019609, EPI_ISL_1019611 to 1019614, EPI_ISL_1019616 to 1019620, EPI_ISL_1019622 to 1019625, EPI_ISL_1019629 to 1019636, EPI_ISL_1019638 to 1019639, EPI_ISL_1019641, EPI_ISL_1019643, EPI_ISL_1019646, EPI_ISL_1019648, EPI_ISL_1019651, EPI_ISL_1019654, EPI_ISL_1019656 to 1019657, EPI_ISL_1019659, EPI_ISL_1019661, EPI_ISL_1019666 to 1019667, EPI_ISL_1019669 to 1019670, EPI_ISL_1019673, EPI_ISL_1019675, EPI_ISL_1019679 to 1019680, EPI_ISL_1019684 to 1019685, EPI_ISL_1019687, EPI_ISL_1019690, EPI_ISL_1019694 to 1019695, EPI_ISL_1019701, EPI_ISL_1019703 to 1019704, EPI_ISL_1019709, EPI_ISL_1019711, EPI_ISL_1019714, EPI_ISL_1019717, EPI_ISL_1019719, EPI_ISL_1019721, EPI_ISL_1019723, EPI_ISL_1019727 to 1019728, EPI_ISL_1019730, EPI_ISL_1019733, EPI_ISL_1019735 to 1019736, EPI_ISL_1019738, EPI_ISL_1019740 to 1019741, EPI_ISL_1019749, EPI_ISL_1019751 to 1019752, EPI_ISL_1019754 to 1019755, EPI_ISL_1019757, EPI_ISL_1019760 to 1019761, EPI_ISL_1019765 to 1019766, EPI_ISL_1019769, EPI_ISL_1019773 to 1019774, EPI_ISL_1019776 to 1019777, EPI_ISL_1019781 to 1019782, EPI_ISL_1019784, EPI_ISL_1019786, EPI_ISL_1019789, EPI_ISL_1019791, EPI_ISL_1019794, EPI_ISL_1019796 to 1019797, EPI_ISL_1019802, EPI_ISL_1019804, EPI_ISL_1019806, EPI_ISL_1019809, EPI_ISL_1019811, EPI_ISL_1019814, EPI_ISL_1019816 to 1019817, EPI_ISL_1019821, EPI_ISL_1019824, EPI_ISL_1019826, EPI_ISL_1019829, EPI_ISL_1019831 to 1019832, EPI_ISL_1019835, EPI_ISL_1019839, EPI_ISL_1019844 to 1019845, EPI_ISL_1019857, EPI_ISL_1019859 to 1019860, EPI_ISL_1019862, EPI_ISL_1019864, EPI_ISL_1019868, EPI_ISL_1019870 to 1019871, EPI_ISL_1019876, EPI_ISL_1019879, EPI_ISL_1019884, EPI_ISL_1019886 to 1019887, EPI_ISL_1019889, EPI_ISL_1019891 to 1019892, EPI_ISL_1019894 to 1019896, EPI_ISL_1019898 to 1019900, EPI_ISL_1019902, EPI_ISL_1019904 to 1019905, EPI_ISL_1019907, EPI_ISL_1019909 to 1019910, EPI_ISL_1019913 to 1019915, EPI_ISL_1019917, EPI_ISL_1019919, EPI_ISL_1019923 to 1019925, EPI_ISL_1019927 to 1019932, EPI_ISL_1019935, EPI_ISL_1019939 to 1019940, EPI_ISL_1019942 to 1019944, EPI_ISL_1019946 to 1019949, EPI_ISL_1019951, EPI_ISL_1019953, EPI_ISL_1019955 to 1019959, EPI_ISL_1019961, EPI_ISL_1019964, EPI_ISL_1019969 to 1019970, EPI_ISL_1019972 to 1019973, EPI_ISL_1019975, EPI_ISL_1019978, EPI_ISL_1019980 to 1019981, EPI_ISL_1019983 to 1019992, EPI_ISL_1019994 to 1019999, EPI_ISL_1020003 to 1020004, EPI_ISL_1020006 to 1020012, EPI_ISL_1020015

see above	Lighthouse Lab in Glasgow	Wellcome Sanger Institute for the COVID-19 Genomics UK (COG-UK) Consortium	Anna Dominiczak and Alex Alderton; Carol Clugston; Cordelia Langford; David Gray; David K. Jackson; Dominic Kwiatkowski; Ewan Harrison; Harper VanSteenhouse; Ian Johnston; Jeffrey Barrett; John Sillitoe on behalf of the Wellcome Sanger Institute COVID-19 Surveillance Team; John Sillitoe on behalf of the Wellcome Sanger Institute COVID-19 Surveillance Team (http://www.sanger.ac.uk/covid-team); Roberto Amato; Sonia Goncalves; Yumi Kasai

EPI_ISL_530352 to 530353, EPI_ISL_530358, EPI_ISL_530360, EPI_ISL_530362 to 530363, EPI_ISL_530374 to 530375, EPI_ISL_530378, EPI_ISL_530380, EPI_ISL_530382 to 530384, EPI_ISL_530387, EPI_ISL_530390, EPI_ISL_530394 to 530397, EPI_ISL_530399 to 530404, EPI_ISL_530406 to 530409, EPI_ISL_530411 to 530412, EPI_ISL_530414 to 530419, EPI_ISL_530421 to 530425, EPI_ISL_530427 to 530428, EPI_ISL_530430 to 530431, EPI_ISL_530433 to 530434, EPI_ISL_530436, EPI_ISL_530438 to 530439, EPI_ISL_530441 to 530445, EPI_ISL_530447, EPI_ISL_530449 to 530452, EPI_ISL_530454 to 530455, EPI_ISL_530457 to 530467, EPI_ISL_530470 to 530471, EPI_ISL_530473 to 530474, EPI_ISL_530476 to 530478, EPI_ISL_530483 to 530484, EPI_ISL_530486 to 530487, EPI_ISL_530490 to 530491, EPI_ISL_530493 to 530496, EPI_ISL_530498 to 530499, EPI_ISL_530501, EPI_ISL_530503 to 530508, EPI_ISL_530511, EPI_ISL_530513 to 530521, EPI_ISL_530530 to 530532, EPI_ISL_530534, EPI_ISL_530536 to 530538, EPI_ISL_530540 to 530541, EPI_ISL_530543 to 530552, EPI_ISL_530554 to 530557, EPI_ISL_530559 to 530561, EPI_ISL_530563 to 530568, EPI_ISL_530570 to 530576, EPI_ISL_530578, EPI_ISL_530580 to 530587, EPI_ISL_530589 to 530591, EPI_ISL_530593 to 530595, EPI_ISL_530597 to 530599, EPI_ISL_530601 to 530603, EPI_ISL_530606 to 530613, EPI_ISL_530615 to 530621, EPI_ISL_530623 to 530624, EPI_ISL_530626 to 530629, EPI_ISL_530631 to 530635, EPI_ISL_530637 to 530639, EPI_ISL_530641, EPI_ISL_530643 to 530644, EPI_ISL_530646, EPI_ISL_530648, EPI_ISL_530650, EPI_ISL_530652 to 530654, EPI_ISL_530656, EPI_ISL_530660 to 530662, EPI_ISL_530664 to 530665, EPI_ISL_530667 to 530672, EPI_ISL_530674, EPI_ISL_530677 to 530680, EPI_ISL_530683 to 530685, EPI_ISL_530687 to 530688, EPI_ISL_530690 to 530694, EPI_ISL_530696 to 530707, EPI_ISL_530709 to 530713, EPI_ISL_530715 to 530721, EPI_ISL_530723 to 530727, EPI_ISL_530729 to 530734, EPI_ISL_530736 to 530738, EPI_ISL_530741 to 530742, EPI_ISL_530745, EPI_ISL_530747 to 530753, EPI_ISL_530755 to 530756, EPI_ISL_530758 to 530760, EPI_ISL_530763,

EPI_ISL_530765, EPI_ISL_530770, EPI_ISL_530772 to 530780, EPI_ISL_530782 to 530789, EPI_ISL_530791 to 530794, EPI_ISL_530796 to 530798, EPI_ISL_530801 to 530825, EPI_ISL_530827 to 530835, EPI_ISL_530837 to 530844, EPI_ISL_530848 to 530852, EPI_ISL_530854, EPI_ISL_530857 to 530861, EPI_ISL_530863 to 530867, EPI_ISL_530869 to 530870, EPI_ISL_530872, EPI_ISL_530876 to 530888, EPI_ISL_530890 to 530897, EPI_ISL_530900 to 530903, EPI_ISL_530905, EPI_ISL_530908 to 530917, EPI_ISL_530919 to 530920, EPI_ISL_530922, EPI_ISL_530924, EPI_ISL_530926 to 530928, EPI_ISL_530930, EPI_ISL_530932 to 530935, EPI_ISL_530937 to 530942, EPI_ISL_530944 to 530952, EPI_ISL_530954 to 530957, EPI_ISL_530960, EPI_ISL_530964, EPI_ISL_530966 to 530970, EPI_ISL_530974, EPI_ISL_530977 to 530978, EPI_ISL_530980 to 530982, EPI_ISL_530984, EPI_ISL_530988 to 530989, EPI_ISL_530991 to 530995, EPI_ISL_530998 to 531001, EPI_ISL_531003, EPI_ISL_531005, EPI_ISL_531007, EPI_ISL_531009 to 531011, EPI_ISL_531013 to 531016, EPI_ISL_531018, EPI_ISL_531021 to 531024, EPI_ISL_531026 to 531028, EPI_ISL_531030, EPI_ISL_531034 to 531037, EPI_ISL_531039, EPI_ISL_531041 to 531044, EPI_ISL_531046 to 531047, EPI_ISL_531050, EPI_ISL_531053, EPI_ISL_531055, EPI_ISL_531058 to 531064, EPI_ISL_531067 to 531078, EPI_ISL_531080 to 531082, EPI_ISL_531084 to 531086, EPI_ISL_531088, EPI_ISL_531094 to 531095, EPI_ISL_531097 to 531098, EPI_ISL_531101 to 531102, EPI_ISL_531105 to 531109, EPI_ISL_531111 to 531113, EPI_ISL_531117, EPI_ISL_531119 to 531120, EPI_ISL_531122, EPI_ISL_531124 to 531125, EPI_ISL_531127 to 531129, EPI_ISL_531133 to 531141, EPI_ISL_531145 to 531146, EPI_ISL_531150 to 531152, EPI_ISL_531158, EPI_ISL_531161, EPI_ISL_531163 to 531165, EPI_ISL_531167 to 531171, EPI_ISL_531173, EPI_ISL_531176 to 531181, EPI_ISL_531183 to 531186, EPI_ISL_531192 to 531197, EPI_ISL_531199 to 531201, EPI_ISL_531203, EPI_ISL_531206, EPI_ISL_531209 to 531210, EPI_ISL_531213, EPI_ISL_531215 to 531220, EPI_ISL_531222, EPI_ISL_531226 to 531227, EPI_ISL_531229 to 531230, EPI_ISL_531232 to 531233, EPI_ISL_531235 to 531237, EPI_ISL_531239 to 531242, EPI_ISL_531244 to 531245, EPI_ISL_531247, EPI_ISL_531250 to 531251, EPI_ISL_531253 to 531265, EPI_ISL_531267 to 531278, EPI_ISL_531280 to 531288, EPI_ISL_531290, EPI_ISL_531292 to 531293, EPI_ISL_531295 to 531301, EPI_ISL_531303 to 531320, EPI_ISL_531322 to 531327, EPI_ISL_531329, EPI_ISL_531331 to 531332, EPI_ISL_531334, EPI_ISL_531338 to 531351, EPI_ISL_531353 to 531354, EPI_ISL_531356 to 531360, EPI_ISL_531362 to 531364, EPI_ISL_531366 to 531373, EPI_ISL_531375 to 531378, EPI_ISL_531380, EPI_ISL_531383, EPI_ISL_531385, EPI_ISL_531387, EPI_ISL_531389 to 531390, EPI_ISL_531392 to 531394, EPI_ISL_531396 to 531404, EPI_ISL_531406 to 531411, EPI_ISL_531413 to 531415, EPI_ISL_531417 to 531419, EPI_ISL_531424 to 531429, EPI_ISL_531431, EPI_ISL_531433 to 531437, EPI_ISL_531439 to 531441, EPI_ISL_531443 to 531444, EPI_ISL_531446 to 531459, EPI_ISL_531461 to 531463, EPI_ISL_531465 to 531469, EPI_ISL_531471 to 531472, EPI_ISL_531475, EPI_ISL_531479, EPI_ISL_531482, EPI_ISL_531486 to 531487, EPI_ISL_531489 to 531491, EPI_ISL_531494, EPI_ISL_531496 to 531499, EPI_ISL_531501 to 531503, EPI_ISL_531505 to 531522, EPI_ISL_531525 to 531526, EPI_ISL_531528 to 531535, EPI_ISL_531537 to 531545, EPI_ISL_531550 to 531555, EPI_ISL_531558 to 531562, EPI_ISL_531568, EPI_ISL_531571, EPI_ISL_531573 to 531574, EPI_ISL_531576, EPI_ISL_531578, EPI_ISL_531580 to 531582, EPI_ISL_531584, EPI_ISL_531586 to 531588, EPI_ISL_531592 to 531594, EPI_ISL_531596 to 531597, EPI_ISL_531600 to 531606, EPI_ISL_531608, EPI_ISL_531610 to 531614, EPI_ISL_531616, EPI_ISL_531619, EPI_ISL_531621 to 531624, EPI_ISL_531627 to 531630, EPI_ISL_531632 to 531633, EPI_ISL_531635 to 531636, EPI_ISL_531642 to 531645, EPI_ISL_531647, EPI_ISL_531650 to 531652, EPI_ISL_531654, EPI_ISL_531656, EPI_ISL_531658, EPI_ISL_531661 to 531667, EPI_ISL_531669, EPI_ISL_531671, EPI_ISL_531673, EPI_ISL_531675 to 531676, EPI_ISL_531679 to 531680, EPI_ISL_531682 to 531685, EPI_ISL_531692 to 531701, EPI_ISL_531704 to 531705, EPI_ISL_531707 to 531713, EPI_ISL_531715 to 531716, EPI_ISL_531718 to 531720, EPI_ISL_531722 to 531725, EPI_ISL_531728, EPI_ISL_531731, EPI_ISL_531733, EPI_ISL_531739, EPI_ISL_531741, EPI_ISL_531744 to 531746, EPI_ISL_531748, EPI_ISL_531750 to 531752, EPI_ISL_531756 to 531757, EPI_ISL_531759 to 531764, EPI_ISL_531766 to 531767, EPI_ISL_531769 to 531774, EPI_ISL_531776 to 531778, EPI_ISL_531780 to 531783, EPI_ISL_531785 to 531787, EPI_ISL_531790 to 531791, EPI_ISL_531793 to 531800, EPI_ISL_531803 to 531804, EPI_ISL_531806 to 531811, EPI_ISL_531816, EPI_ISL_531818 to 531820, EPI_ISL_531822, EPI_ISL_531825, EPI_ISL_531827, EPI_ISL_531829 to 531831, EPI_ISL_531833 to 531834, EPI_ISL_531836 to 531841, EPI_ISL_531843 to 531846, EPI_ISL_531848 to 531849, EPI_ISL_531852 to 531854, EPI_ISL_531859 to 531861, EPI_ISL_531864, EPI_ISL_531866 to 531871, EPI_ISL_531874 to 531876, EPI_ISL_531879, EPI_ISL_531882 to 531883, EPI_ISL_531885 to 531886, EPI_ISL_531888, EPI_ISL_531891, EPI_ISL_531893, EPI_ISL_531895, EPI_ISL_531897 to 531901, EPI_ISL_531903, EPI_ISL_531906 to 531910, EPI_ISL_531916 to 531917, EPI_ISL_531920 to 531924, EPI_ISL_531926 to 531927, EPI_ISL_531930 to 531931, EPI_ISL_531934, EPI_ISL_531937 to 531940, EPI_ISL_531942, EPI_ISL_531944, EPI_ISL_531947 to 531950, EPI_ISL_531952 to 531953, EPI_ISL_531955 to 531962, EPI_ISL_531964 to 531972, EPI_ISL_531974 to 531976, EPI_ISL_531980 to 531981, EPI_ISL_531983, EPI_ISL_531985 to 531986, EPI_ISL_531988 to 531989, EPI_ISL_531991, EPI_ISL_531993 to 531994, EPI_ISL_531996 to 531999, EPI_ISL_532001, EPI_ISL_532006, EPI_ISL_532008 to 532011, EPI_ISL_532014 to 532015, EPI_ISL_532017, EPI_ISL_532019 to 532023, EPI_ISL_532025 to 532031, EPI_ISL_532033 to 532037, EPI_ISL_532039 to 532047, EPI_ISL_532050, EPI_ISL_532052, EPI_ISL_532054, EPI_ISL_532056, EPI_ISL_532058 to 532060, EPI_ISL_532063 to 532069, EPI_ISL_532072 to 532075, EPI_ISL_532079, EPI_ISL_532082 to 532084, EPI_ISL_532086 to 532092, EPI_ISL_532095 to 532097, EPI_ISL_532099, EPI_ISL_532101, EPI_ISL_532103 to 532104, EPI_ISL_532106 to 532107, EPI_ISL_532109 to 532117, EPI_ISL_532119 to 532122, EPI_ISL_532124, EPI_ISL_532126 to 532128, EPI_ISL_532132 to 532137, EPI_ISL_532140 to 532141, EPI_ISL_532143 to 532144, EPI_ISL_532146 to 532148, EPI_ISL_532150 to 532152, EPI_ISL_532156 to 532160, EPI_ISL_532162, EPI_ISL_532164 to 532166, EPI_ISL_532170 to 532173, EPI_ISL_532176, EPI_ISL_532180 to 532181, EPI_ISL_532183 to 532185, EPI_ISL_532188, EPI_ISL_532190 to 532191, EPI_ISL_532193, EPI_ISL_532198 to 532199, EPI_ISL_532201, EPI_ISL_532204 to 532206, EPI_ISL_532209, EPI_ISL_532212, EPI_ISL_532216 to 532217, EPI_ISL_532220, EPI_ISL_532222 to 532224, EPI_ISL_532226 to 532228, EPI_ISL_532230 to 532231, EPI_ISL_532233 to 532234, EPI_ISL_532236 to 532239, EPI_ISL_532243, EPI_ISL_532245, EPI_ISL_532247 to 532248, EPI_ISL_532251 to 532257, EPI_ISL_532260 to 532262, EPI_ISL_532265 to 532270, EPI_ISL_532273, EPI_ISL_532279 to 532280, EPI_ISL_532282, EPI_ISL_532284 to 532287, EPI_ISL_532289 to 532290, EPI_ISL_532295 to 532300, EPI_ISL_532302 to 532303, EPI_ISL_532305, EPI_ISL_532308 to 532310, EPI_ISL_532312 to 532314, EPI_ISL_532318 to 532320, EPI_ISL_532322 to 532326, EPI_ISL_532329 to 532331, EPI_ISL_532333, EPI_ISL_532336 to 532337, EPI_ISL_532339, EPI_ISL_532341, EPI_ISL_532343 to 532344, EPI_ISL_532348, EPI_ISL_532350, EPI_ISL_532352, EPI_ISL_532354, EPI_ISL_532357, EPI_ISL_532359 to 532360, EPI_ISL_532362, EPI_ISL_532366 to 532369, EPI_ISL_532371 to 532375, EPI_ISL_532377 to 532378, EPI_ISL_532380 to 532383, EPI_ISL_532386 to 532387, EPI_ISL_532389, EPI_ISL_532391 to 532393, EPI_ISL_532395 to 532398, EPI_ISL_532400 to 532401, EPI_ISL_532403, EPI_ISL_532406 to 532413, EPI_ISL_532415 to 532421, EPI_ISL_532424 to 532426, EPI_ISL_532428 to 532429, EPI_ISL_532433, EPI_ISL_532436 to 532437, EPI_ISL_532439, EPI_ISL_532442, EPI_ISL_532447, EPI_ISL_532449 to 532451, EPI_ISL_532453, EPI_ISL_532455, EPI_ISL_532457 to 532459, EPI_ISL_532461 to 532462, EPI_ISL_532465 to 532468, EPI_ISL_532470 to 532471, EPI_ISL_532474, EPI_ISL_532476 to 532478, EPI_ISL_532481 to 532483, EPI_ISL_532485, EPI_ISL_532487 to 532489, EPI_ISL_532491, EPI_ISL_532494 to 532505, EPI_ISL_532507 to 532509, EPI_ISL_532513, EPI_ISL_532516 to 532517, EPI_ISL_532520 to 532522, EPI_ISL_532524 to 532530, EPI_ISL_532532 to 532537, EPI_ISL_532539, EPI_ISL_532541 to 532545, EPI_ISL_532548, EPI_ISL_532550, EPI_ISL_532553 to 532559, EPI_ISL_532561 to 532562, EPI_ISL_532565, EPI_ISL_532567, EPI_ISL_532569 to 532571, EPI_ISL_532573 to 532579, EPI_ISL_532581, EPI_ISL_532584 to 532586, EPI_ISL_532588 to 532593, EPI_ISL_532595, EPI_ISL_532597, EPI_ISL_532599 to 532605, EPI_ISL_532607 to 532610, EPI_ISL_532613 to 532619, EPI_ISL_532622 to 532624, EPI_ISL_532627 to 532632, EPI_ISL_532634 to 532635, EPI_ISL_532637, EPI_ISL_532641 to 532644, EPI_ISL_532647, EPI_ISL_532649 to 532652, EPI_ISL_532654, EPI_ISL_532656 to 532657, EPI_ISL_532660, EPI_ISL_532662 to 532663, EPI_ISL_532665 to 532668, EPI_ISL_532671, EPI_ISL_532674 to 532682, EPI_ISL_532685 to 532688, EPI_ISL_532690 to 532696, EPI_ISL_532698 to 532704, EPI_ISL_532706, EPI_ISL_532708, EPI_ISL_532711 to 532714, EPI_ISL_532716 to 532719, EPI_ISL_532721 to 532722, EPI_ISL_532724 to 532727, EPI_ISL_532729 to 532732, EPI_ISL_532734 to 532737, EPI_ISL_532740 to 532743, EPI_ISL_532745, EPI_ISL_532747 to 532749, EPI_ISL_532751 to 532752, EPI_ISL_532754, EPI_ISL_532756 to 532758, EPI_ISL_532760 to 532764, EPI_ISL_532766, EPI_ISL_532768 to 532769, EPI_ISL_532771 to 532780, EPI_ISL_532783, EPI_ISL_532785, EPI_ISL_532787 to 532788, EPI_ISL_532790, EPI_ISL_532792 to 532800, EPI_ISL_532802 to 532810, EPI_ISL_532812 to 532813, EPI_ISL_532815 to 532817, EPI_ISL_532819 to 532820, EPI_ISL_532822 to 532823, EPI_ISL_532825 to 532834, EPI_ISL_532836 to 532837, EPI_ISL_532840, EPI_ISL_532842, EPI_ISL_532845, EPI_ISL_532847 to 532849, EPI_ISL_532853 to 532856, EPI_ISL_532860 to 532861, EPI_ISL_532863, EPI_ISL_532866 to 532872, EPI_ISL_532877, EPI_ISL_532879, EPI_ISL_532882 to 532884, EPI_ISL_532887 to 532890, EPI_ISL_532892 to 532893, EPI_ISL_532895, EPI_ISL_532897 to 532901, EPI_ISL_532903 to 532904, EPI_ISL_532907 to 532908, EPI_ISL_532911 to 532912, EPI_ISL_532917 to 532918, EPI_ISL_532922 to 532924, EPI_ISL_532927 to 532928, EPI_ISL_532930 to 532931, EPI_ISL_532934 to 532935, EPI_ISL_532938 to 532939, EPI_ISL_532941 to 532942, EPI_ISL_532944 to 532945, EPI_ISL_532947, EPI_ISL_532949 to 532950, EPI_ISL_532952 to 532961, EPI_ISL_532963 to 532968, EPI_ISL_532970 to 532975, EPI_ISL_532977, EPI_ISL_532979 to 532982, EPI_ISL_532984 to 532987, EPI_ISL_532989, EPI_ISL_532991, EPI_ISL_532993, EPI_ISL_532995 to 532999, EPI_ISL_533001 to 533005, EPI_ISL_533007 to 533011, EPI_ISL_533013 to 533015, EPI_ISL_533018 to 533023, EPI_ISL_533025 to 533026, EPI_ISL_533028, EPI_ISL_533031 to 533045, EPI_ISL_533047 to 533048, EPI_ISL_533050 to 533053, EPI_ISL_533056 to 533058, EPI_ISL_533060 to 533065, EPI_ISL_533067 to 533071, EPI_ISL_533073 to 533078, EPI_ISL_533080 to 533083, EPI_ISL_533085, EPI_ISL_533088, EPI_ISL_533090, EPI_ISL_533092 to 533093, EPI_ISL_533095 to 533097, EPI_ISL_533099 to 533106, EPI_ISL_533108 to 533116, EPI_ISL_533118, EPI_ISL_533120 to 533129, EPI_ISL_533131 to 533132, EPI_ISL_533134, EPI_ISL_533137, EPI_ISL_533142 to 533143, EPI_ISL_533145 to 533146, EPI_ISL_533148 to 533153, EPI_ISL_533155 to 533165, EPI_ISL_533167 to 533174, EPI_ISL_533176 to 533179, EPI_ISL_533181 to 533187, EPI_ISL_533189 to 533190, EPI_ISL_533192 to 533198, EPI_ISL_533200 to 533203, EPI_ISL_533205 to 533206, EPI_ISL_533208 to 533221, EPI_ISL_533223 to 533227, EPI_ISL_533233 to 533235, EPI_ISL_533238 to 533240, EPI_ISL_533243 to 533249, EPI_ISL_533253 to 533255, EPI_ISL_533257 to 533261, EPI_ISL_533263 to 533266, EPI_ISL_533268 to 533269, EPI_ISL_533273 to 533275, EPI_ISL_533277 to 533282, EPI_ISL_533284, EPI_ISL_533286 to 533288, EPI_ISL_533290 to 533291, EPI_ISL_533293 to 533297, EPI_ISL_533299 to 533306, EPI_ISL_533308 to 533310, EPI_ISL_533313 to 533316, EPI_ISL_533318 to 533326, EPI_ISL_533329, EPI_ISL_533331 to 533333, EPI_ISL_533335 to 533339, EPI_ISL_533341 to 533342, EPI_ISL_533344 to 533345, EPI_ISL_533347 to 533349, EPI_ISL_533352 to 533353, EPI_ISL_533356 to 533357, EPI_ISL_533360 to 533364, EPI_ISL_533366, EPI_ISL_533368 to 533372, EPI_ISL_533374 to 533375, EPI_ISL_533377 to 533386, EPI_ISL_533388 to 533393, EPI_ISL_533395, EPI_ISL_533397 to 533398, EPI_ISL_533400 to 533402, EPI_ISL_533404 to 533405, EPI_ISL_533407 to 533410, EPI_ISL_533412 to 533418, EPI_ISL_533420, EPI_ISL_533422, EPI_ISL_533424 to 533426, EPI_ISL_533428 to 533431, EPI_ISL_533433, EPI_ISL_536819 to 536822, EPI_ISL_536825 to 536831, EPI_ISL_536833, EPI_ISL_536835, EPI_ISL_536837 to 536843, EPI_ISL_536845, EPI_ISL_536847 to 536849, EPI_ISL_536851 to 536855, EPI_ISL_536857, EPI_ISL_536860 to 536866, EPI_ISL_536868 to 536877, EPI_ISL_536879 to 536882, EPI_ISL_536884 to 536888, EPI_ISL_536890 to 536899, EPI_ISL_536904 to 536915, EPI_ISL_536917 to 536929, EPI_ISL_536931 to 536935, EPI_ISL_536937 to 536940, EPI_ISL_536943 to 536945, EPI_ISL_536947 to 536950, EPI_ISL_536952 to 536954, EPI_ISL_536958 to 536964, EPI_ISL_536966 to 536991, EPI_ISL_536993 to 536994, EPI_ISL_536996 to 537009, EPI_ISL_537011 to 537016, EPI_ISL_537018 to 537019, EPI_ISL_537021 to 537022, EPI_ISL_537024 to 537034, EPI_ISL_537036 to 537039, EPI_ISL_537042 to 537044, EPI_ISL_537048 to 537053, EPI_ISL_537055 to 537060, EPI_ISL_537062, EPI_ISL_537064 to 537073, EPI_ISL_537075 to 537076, EPI_ISL_537078 to 537113, EPI_ISL_537115 to 537121, EPI_ISL_537123 to 537124, EPI_ISL_537126 to 537134, EPI_ISL_537136 to 537146, EPI_ISL_537148 to 537151, EPI_ISL_537153 to 537154, EPI_ISL_537156 to 537177, EPI_ISL_537179 to 537182, EPI_ISL_537184 to 537185, EPI_ISL_537188 to 537193, EPI_ISL_537195 to 537196, EPI_ISL_537198 to 537202, EPI_ISL_537204 to 537205, EPI_ISL_539897 to 539898, EPI_ISL_539900 to 539905, EPI_ISL_539907 to 539919, EPI_ISL_539921 to 539939, EPI_ISL_539941 to 539960, EPI_ISL_539964 to 539967, EPI_ISL_539969 to 539981, EPI_ISL_539983 to 539988, EPI_ISL_539990 to 540002, EPI_ISL_540004 to 540014, EPI_ISL_540016 to 540019, EPI_ISL_540022 to 540023, EPI_ISL_540025 to 540029, EPI_ISL_540031 to 540048, EPI_ISL_540050 to 540052, EPI_ISL_540054 to 540058, EPI_ISL_540060 to 540072, EPI_ISL_540074 to 540086, EPI_ISL_540088 to 540098, EPI_ISL_540101 to 540110, EPI_ISL_540112 to 540113, EPI_ISL_540115 to 540132, EPI_ISL_540134 to 540152, EPI_ISL_540154 to 540155, EPI_ISL_540157, EPI_ISL_540159 to 540160, EPI_ISL_540162 to 540164, EPI_ISL_540166 to 540183, EPI_ISL_540185 to 540189, EPI_ISL_540191 to 540228, EPI_ISL_540230 to 540231, EPI_ISL_540233 to 540236, EPI_ISL_540239 to 540240, EPI_ISL_540242 to 540246, EPI_ISL_540248 to 540249, EPI_ISL_540251 to 540254, EPI_ISL_540256 to 540272, EPI_ISL_540274, EPI_ISL_540276 to 540277, EPI_ISL_540279 to 540280, EPI_ISL_540282 to 540283, EPI_ISL_540285 to 540286, EPI_ISL_540288 to 540294, EPI_ISL_540296 to 540304, EPI_ISL_540306 to 540308, EPI_ISL_540310 to 540312, EPI_ISL_540314 to 540320, EPI_ISL_540322 to 540326, EPI_ISL_540328, EPI_ISL_540330 to 540331, EPI_ISL_540335 to 540350, EPI_ISL_540352 to 540370, EPI_ISL_540372 to 540373, EPI_ISL_540376 to 540387, EPI_ISL_540389 to 540399, EPI_ISL_540401 to 540411, EPI_ISL_540413 to 540414, EPI_ISL_540416, EPI_ISL_540419 to 540420, EPI_ISL_541784 to 541791, EPI_ISL_541793, EPI_ISL_541795 to 541797, EPI_ISL_541800 to 541805, EPI_ISL_541807 to 541816, EPI_ISL_541818 to 541821, EPI_ISL_541823 to 541842, EPI_ISL_541844, EPI_ISL_541846, EPI_ISL_549547 to 549549, EPI_ISL_549551 to 549563, EPI_ISL_549566 to 549571, EPI_ISL_549573 to 549579, EPI_ISL_549581 to 549583, EPI_ISL_549585 to 549589, EPI_ISL_549591, EPI_ISL_549593, EPI_ISL_549595 to 549599, EPI_ISL_549601 to 549603, EPI_ISL_549605 to 549614, EPI_ISL_549616 to 549636, EPI_ISL_549638 to 549649, EPI_ISL_549651 to 549676, EPI_ISL_549678 to 549682, EPI_ISL_549684, EPI_ISL_549686 to 549694, EPI_ISL_549696 to 549705, EPI_ISL_549707 to 549708, EPI_ISL_549710 to 549715, EPI_ISL_549717 to 549718, EPI_ISL_549720, EPI_ISL_549722 to 549725, EPI_ISL_549727 to 549740, EPI_ISL_549742, EPI_ISL_549745, EPI_ISL_549747 to 549769, EPI_ISL_549773 to 549774, EPI_ISL_549776 to 549778, EPI_ISL_549780 to 549783, EPI_ISL_549785 to 549788, EPI_ISL_549790 to 549803, EPI_ISL_549805 to 549806, EPI_ISL_553732, EPI_ISL_566998 to 566999, EPI_ISL_567001 to 567023, EPI_ISL_567025 to 567026, EPI_ISL_567028 to 567031, EPI_ISL_567033, EPI_ISL_567035 to 567036, EPI_ISL_567038 to 567042, EPI_ISL_567044 to 567047, EPI_ISL_567049 to 567058, EPI_ISL_567060 to 567070, EPI_ISL_567072 to 567076, EPI_ISL_567078 to 567083, EPI_ISL_567085 to 567088, EPI_ISL_567090 to 567091, EPI_ISL_567093 to 567100, EPI_ISL_567103 to 567109, EPI_ISL_567111 to 567112, EPI_ISL_567115 to 567125, EPI_ISL_567127, EPI_ISL_567129, EPI_ISL_567131 to 567133, EPI_ISL_567135, EPI_ISL_567137, EPI_ISL_567140 to 567148, EPI_ISL_567150 to 567157, EPI_ISL_567159 to 567162, EPI_ISL_567164 to 567169, EPI_ISL_567171 to 567183, EPI_ISL_567185 to 567201, EPI_ISL_567203 to 567212, EPI_ISL_567215 to 567216, EPI_ISL_567218 to 567220, EPI_ISL_567222 to 567223, EPI_ISL_567225 to 567226, EPI_ISL_567228 to 567230, EPI_ISL_567232 to 567233, EPI_ISL_567235 to 567240, EPI_ISL_567242 to 567243, EPI_ISL_567245 to 567259, EPI_ISL_567261 to 567262, EPI_ISL_567264, EPI_ISL_567266 to 567277, EPI_ISL_567279, EPI_ISL_567281 to 567288, EPI_ISL_567290 to 567294, EPI_ISL_567297, EPI_ISL_567299 to 567304, EPI_ISL_567306 to 567317, EPI_ISL_567320, EPI_ISL_567322 to 567325, EPI_ISL_567327, EPI_ISL_567329 to 567341, EPI_ISL_567345 to 567350, EPI_ISL_567352 to 567353, EPI_ISL_567355 to 567370, EPI_ISL_567372 to 567374, EPI_ISL_567376 to 567384, EPI_ISL_567386, EPI_ISL_567388 to 567389, EPI_ISL_567391 to 567394, EPI_ISL_567396 to 567431, EPI_ISL_567433 to 567443, EPI_ISL_567445 to 567446, EPI_ISL_567448 to 567455, EPI_ISL_567457 to 567463, EPI_ISL_567465 to 567468, EPI_ISL_567470 to 567475, EPI_ISL_567477 to 567486, EPI_ISL_567488 to 567490, EPI_ISL_567492 to 567503, EPI_ISL_567505 to 567515, EPI_ISL_567517 to 567518, EPI_ISL_567520 to 567526, EPI_ISL_567528 to 567532, EPI_ISL_568223 to 568279, EPI_ISL_568281 to 568288, EPI_ISL_568291 to 568299, EPI_ISL_568302, EPI_ISL_568304 to 568314, EPI_ISL_568316, EPI_ISL_568318 to 568324, EPI_ISL_568326 to 568343, EPI_ISL_568345 to 568355, EPI_ISL_568357 to 568363, EPI_ISL_568366 to 568384, EPI_ISL_568388 to 568391, EPI_ISL_568393 to 568394, EPI_ISL_568401 to 568408, EPI_ISL_568411 to 568414, EPI_ISL_568416 to 568418, EPI_ISL_568420 to 568421, EPI_ISL_568423 to 568427, EPI_ISL_568429 to 568434, EPI_ISL_568437 to 568451, EPI_ISL_568455 to 568457, EPI_ISL_568459, EPI_ISL_568462 to 568464, EPI_ISL_575338 to 575346, EPI_ISL_575348 to 575366, EPI_ISL_575368 to 575388, EPI_ISL_575390 to 575398, EPI_ISL_575996 to 575997, EPI_ISL_575999 to 576000, EPI_ISL_576002, EPI_ISL_576006 to 576017, EPI_ISL_576019 to 576025, EPI_ISL_576027 to 576043, EPI_ISL_576045 to 576049, EPI_ISL_576051 to 576063, EPI_ISL_576065 to 576066, EPI_ISL_576069, EPI_ISL_576071 to 576080, EPI_ISL_576082 to 576087, EPI_ISL_576089 to 576093, EPI_ISL_579759 to 579771, EPI_ISL_579773 to 579793, EPI_ISL_579795 to 579796, EPI_ISL_579798 to 579807, EPI_ISL_579809 to 579834, EPI_ISL_579836 to 579842, EPI_ISL_579844 to 579865, EPI_ISL_579867 to 579906, EPI_ISL_579908 to 579917, EPI_ISL_580858 to 580860, EPI_ISL_580862 to 580863, EPI_ISL_580865 to 580869, EPI_ISL_580871 to 580872, EPI_ISL_580874, EPI_ISL_580878 to 580881, EPI_ISL_580883 to 580884, EPI_ISL_580886 to 580889, EPI_ISL_580891, EPI_ISL_580893 to 580894, EPI_ISL_580899, EPI_ISL_580901, EPI_ISL_580905 to 580908, EPI_ISL_580911 to 580912, EPI_ISL_580914, EPI_ISL_580916, EPI_ISL_580919 to 580920, EPI_ISL_580922 to 580927, EPI_ISL_580930 to 580934, EPI_ISL_580936 to 580939, EPI_ISL_580941 to 580947, EPI_ISL_580949, EPI_ISL_580952 to 580955, EPI_ISL_580957 to 580958, EPI_ISL_580960, EPI_ISL_580962 to 580975, EPI_ISL_580977, EPI_ISL_580981 to 580989, EPI_ISL_580992, EPI_ISL_580994, EPI_ISL_580997, EPI_ISL_580999, EPI_ISL_581001 to 581003, EPI_ISL_581005 to 581007, EPI_ISL_581010 to 581011, EPI_ISL_581013 to 581017, EPI_ISL_581019 to 581022, EPI_ISL_581024, EPI_ISL_581027, EPI_ISL_581030 to 581031, EPI_ISL_581033 to 581034, EPI_ISL_581036 to 581037, EPI_ISL_581042 to 581044, EPI_ISL_581047 to 581053, EPI_ISL_581055, EPI_ISL_581057, EPI_ISL_581059 to 581063, EPI_ISL_581068, EPI_ISL_581070 to 581073, EPI_ISL_581075, EPI_ISL_581077, EPI_ISL_581079 to 581083, EPI_ISL_581085, EPI_ISL_581087, EPI_ISL_581089 to 581091, EPI_ISL_581094 to 581096, EPI_ISL_581098, EPI_ISL_581100 to 581102, EPI_ISL_581104 to 581105, EPI_ISL_581108 to 581110, EPI_ISL_581112, EPI_ISL_581127, EPI_ISL_581132 to 581133, EPI_ISL_581141, EPI_ISL_581146, EPI_ISL_581154, EPI_ISL_581166, EPI_ISL_581174, EPI_ISL_581182 to 581183, EPI_ISL_581189, EPI_ISL_581193 to 581194, EPI_ISL_581196, EPI_ISL_581214, EPI_ISL_581217, EPI_ISL_581223, EPI_ISL_581226, EPI_ISL_581229 to 581230, EPI_ISL_581245, EPI_ISL_581247, EPI_ISL_581262, EPI_ISL_581267, EPI_ISL_581285, EPI_ISL_581291, EPI_ISL_581310, EPI_ISL_581324, EPI_ISL_581329, EPI_ISL_581334, EPI_ISL_581337, EPI_ISL_581341, EPI_ISL_581344, EPI_ISL_581346, EPI_ISL_581354, EPI_ISL_581358, EPI_ISL_581377, EPI_ISL_581381, EPI_ISL_581384, EPI_ISL_581387, EPI_ISL_581392, EPI_ISL_581395, EPI_ISL_581402 to 581403, EPI_ISL_581405, EPI_ISL_581407 to 581408, EPI_ISL_581410, EPI_ISL_581418 to 581420, EPI_ISL_581437, EPI_ISL_581439, EPI_ISL_581441, EPI_ISL_581443, EPI_ISL_587304 to 587306, EPI_ISL_587308 to 587315, EPI_ISL_587317, EPI_ISL_587319 to 587335, EPI_ISL_587337 to 587346, EPI_ISL_587348 to 587362, EPI_ISL_587364 to 587379, EPI_ISL_587381, EPI_ISL_587384 to 587385, EPI_ISL_587387 to 587389, EPI_ISL_587391 to 587400, EPI_ISL_587402 to 587409, EPI_ISL_587411, EPI_ISL_587413 to 587417, EPI_ISL_587419 to 587425, EPI_ISL_587427 to 587428, EPI_ISL_587430 to 587440, EPI_ISL_587442 to 587443, EPI_ISL_587445 to 587446, EPI_ISL_587449 to 587450, EPI_ISL_587452 to 587456, EPI_ISL_587458 to 587460, EPI_ISL_587462 to 587463, EPI_ISL_587465, EPI_ISL_587467 to 587471, EPI_ISL_587473 to 587476, EPI_ISL_587478 to 587481, EPI_ISL_587483 to 587485, EPI_ISL_587487 to 587494, EPI_ISL_587496 to 587497, EPI_ISL_587500 to 587506, EPI_ISL_587508 to 587514, EPI_ISL_587516 to 587524, EPI_ISL_587526 to 587530, EPI_ISL_587532 to 587533, EPI_ISL_587535 to 587536, EPI_ISL_587538 to 587543, EPI_ISL_587545 to 587549, EPI_ISL_587551 to 587589, EPI_ISL_587591 to 587592, EPI_ISL_587595 to 587606, EPI_ISL_587608 to 587629, EPI_ISL_587631 to 587638, EPI_ISL_587640 to 587653, EPI_ISL_587655 to 587665, EPI_ISL_587667 to 587674, EPI_ISL_587676 to 587681, EPI_ISL_587683 to 587691, EPI_ISL_587693 to 587706, EPI_ISL_587709 to 587710, EPI_ISL_588060, EPI_ISL_588062 to 588066, EPI_ISL_588068, EPI_ISL_588070 to 588074, EPI_ISL_588076 to 588086, EPI_ISL_588088 to 588111, EPI_ISL_588113, EPI_ISL_588115 to 588117, EPI_ISL_588119 to 588124, EPI_ISL_588126 to 588127, EPI_ISL_588129 to 588130, EPI_ISL_588132 to 588136, EPI_ISL_588138 to 588143, EPI_ISL_588145 to 588174, EPI_ISL_588176 to 588197, EPI_ISL_588199 to 588206, EPI_ISL_588208 to 588210, EPI_ISL_588212 to 588227, EPI_ISL_588229 to 588235, EPI_ISL_588237 to 588244, EPI_ISL_588247 to 588248, EPI_ISL_588250 to 588251, EPI_ISL_588253 to 588263, EPI_ISL_588265 to 588279, EPI_ISL_588282 to 588284, EPI_ISL_588286 to 588297, EPI_ISL_588299, EPI_ISL_588301 to 588304, EPI_ISL_588306 to 588320, EPI_ISL_588322 to 588346, EPI_ISL_588348, EPI_ISL_588350 to 588359, EPI_ISL_588361 to 588372, EPI_ISL_588374 to 588379, EPI_ISL_588381 to 588389, EPI_ISL_588391 to 588395, EPI_ISL_588397 to 588400, EPI_ISL_588402 to 588418, EPI_ISL_588420 to 588423, EPI_ISL_588425 to 588437, EPI_ISL_588439 to 588454, EPI_ISL_588456 to 588469, EPI_ISL_588472 to 588479, EPI_ISL_588481 to 588493, EPI_ISL_588497 to 588522, EPI_ISL_588524 to 588540, EPI_ISL_588542 to 588553, EPI_ISL_588555 to 588571, EPI_ISL_588573 to 588574, EPI_ISL_588576 to 588580, EPI_ISL_588582, EPI_ISL_588584, EPI_ISL_588586 to 588600, EPI_ISL_588602 to 588617, EPI_ISL_588619 to 588654, EPI_ISL_588656, EPI_ISL_588658 to 588659, EPI_ISL_588661 to 588662, EPI_ISL_588664 to 588672, EPI_ISL_588675 to 588694, EPI_ISL_588696, EPI_ISL_588698 to 588713, EPI_ISL_588716, EPI_ISL_588718 to 588723, EPI_ISL_588725 to 588727, EPI_ISL_588730 to 588782, EPI_ISL_588784 to 588791, EPI_ISL_588793 to 588810, EPI_ISL_588812 to 588820, EPI_ISL_588822 to 588823, EPI_ISL_588825 to 588843, EPI_ISL_588846 to 588866, EPI_ISL_588868 to 588881, EPI_ISL_588883 to 588911, EPI_ISL_588913 to 588918, EPI_ISL_588920 to 588926, EPI_ISL_588928 to 588939, EPI_ISL_588941 to 588957, EPI_ISL_588959 to 588961, EPI_ISL_588963, EPI_ISL_588965 to 588987, EPI_ISL_588989 to 588991, EPI_ISL_588993 to 588994, EPI_ISL_588996 to 589011, EPI_ISL_589013 to 589014, EPI_ISL_589016, EPI_ISL_589022 to 589023, EPI_ISL_589027, EPI_ISL_589031 to 589033, EPI_ISL_589035 to 589036, EPI_ISL_589042, EPI_ISL_589044 to 589045, EPI_ISL_589047 to 589049, EPI_ISL_589053 to 589055, EPI_ISL_589058, EPI_ISL_589061, EPI_ISL_589064 to 589066, EPI_ISL_589068 to 589069, EPI_ISL_589072 to 589073, EPI_ISL_589076, EPI_ISL_589078, EPI_ISL_589082, EPI_ISL_589084, EPI_ISL_589087 to 589089, EPI_ISL_589092 to 589094, EPI_ISL_589096,

EPI_ISL_589100, EPI_ISL_589102, EPI_ISL_589105 to 589107, EPI_ISL_589112 to 589113, EPI_ISL_589119 to 589120, EPI_ISL_589124 to 589126, EPI_ISL_589128 to 589129, EPI_ISL_589131 to 589132, EPI_ISL_589136, EPI_ISL_589138 to 589142, EPI_ISL_589144 to 589146, EPI_ISL_589148, EPI_ISL_589150 to 589152, EPI_ISL_589154 to 589156, EPI_ISL_589158, EPI_ISL_589162 to 589165, EPI_ISL_589175, EPI_ISL_589177 to 589178, EPI_ISL_589180 to 589181, EPI_ISL_589186, EPI_ISL_589188 to 589192, EPI_ISL_589194, EPI_ISL_589197, EPI_ISL_589201 to 589204, EPI_ISL_589206 to 589207, EPI_ISL_589209 to 589210, EPI_ISL_589213, EPI_ISL_589215 to 589216, EPI_ISL_589219 to 589220, EPI_ISL_589222 to 589223, EPI_ISL_589232, EPI_ISL_589236, EPI_ISL_589238 to 589241, EPI_ISL_589244 to 589246, EPI_ISL_589249 to 589250, EPI_ISL_589252, EPI_ISL_589254 to 589255, EPI_ISL_589257 to 589258, EPI_ISL_589263, EPI_ISL_589774 to 589777, EPI_ISL_589788, EPI_ISL_589790, EPI_ISL_589804 to 589834, EPI_ISL_589836, EPI_ISL_589839 to 589845, EPI_ISL_589847 to 589849, EPI_ISL_589852 to 589856, EPI_ISL_589859 to 589871, EPI_ISL_589873 to 589876, EPI_ISL_589878 to 589890, EPI_ISL_589893 to 589894, EPI_ISL_589897 to 589903, EPI_ISL_589905, EPI_ISL_589907 to 589910, EPI_ISL_589912, EPI_ISL_589914 to 589916, EPI_ISL_589918 to 589929, EPI_ISL_589931 to 589933, EPI_ISL_589935, EPI_ISL_589937 to 589940, EPI_ISL_589942 to 589945, EPI_ISL_589947, EPI_ISL_589949 to 589962, EPI_ISL_589965 to 589967, EPI_ISL_589969 to 589973, EPI_ISL_589975 to 589993, EPI_ISL_589995 to 589999, EPI_ISL_590001 to 590007, EPI_ISL_590009 to 590013, EPI_ISL_590016 to 590018, EPI_ISL_590021, EPI_ISL_590023 to 590034, EPI_ISL_590037 to 590039, EPI_ISL_590041, EPI_ISL_590043 to 590045, EPI_ISL_590048 to 590062, EPI_ISL_590064 to 590067, EPI_ISL_590069 to 590074, EPI_ISL_590076 to 590078, EPI_ISL_590080 to 590105, EPI_ISL_590107 to 590118, EPI_ISL_590120 to 590125, EPI_ISL_590127 to 590142, EPI_ISL_590144 to 590166, EPI_ISL_590168 to 590171, EPI_ISL_590176 to 590180, EPI_ISL_590182 to 590185, EPI_ISL_590187 to 590192, EPI_ISL_590195 to 590197, EPI_ISL_590199, EPI_ISL_590201 to 590205, EPI_ISL_590207 to 590209, EPI_ISL_590211 to 590212, EPI_ISL_590216 to 590219, EPI_ISL_590221 to 590223, EPI_ISL_590227, EPI_ISL_590229 to 590233, EPI_ISL_590237 to 590238, EPI_ISL_590240 to 590245, EPI_ISL_590247 to 590249, EPI_ISL_590251 to 590254, EPI_ISL_590256, EPI_ISL_590258, EPI_ISL_590262, EPI_ISL_590264, EPI_ISL_590266 to 590271, EPI_ISL_590273, EPI_ISL_590278 to 590281, EPI_ISL_590285, EPI_ISL_590289 to 590294, EPI_ISL_590297 to 590299, EPI_ISL_590301 to 590305, EPI_ISL_590310 to 590311, EPI_ISL_590313, EPI_ISL_590315 to 590318, EPI_ISL_590320, EPI_ISL_590322 to 590325, EPI_ISL_590330 to 590334, EPI_ISL_590336, EPI_ISL_590338 to 590345, EPI_ISL_590348 to 590350, EPI_ISL_590353 to 590354, EPI_ISL_590356, EPI_ISL_590358, EPI_ISL_590360, EPI_ISL_590362 to 590363, EPI_ISL_590365, EPI_ISL_590368, EPI_ISL_590370 to 590372, EPI_ISL_590374 to 590378, EPI_ISL_590381 to 590382, EPI_ISL_590384 to 590385, EPI_ISL_590387, EPI_ISL_590391, EPI_ISL_590393 to 590396, EPI_ISL_590400 to 590401, EPI_ISL_590404, EPI_ISL_590406 to 590418, EPI_ISL_590420 to 590421, EPI_ISL_590424 to 590428, EPI_ISL_590430 to 590432, EPI_ISL_590434 to 590437, EPI_ISL_590439 to 590440, EPI_ISL_590443 to 590445, EPI_ISL_590447, EPI_ISL_590449 to 590451, EPI_ISL_590454 to 590461, EPI_ISL_590463 to 590480, EPI_ISL_590482 to 590492, EPI_ISL_590494 to 590496, EPI_ISL_590498 to 590506, EPI_ISL_590508 to 590512, EPI_ISL_590514 to 590522, EPI_ISL_590524 to 590525, EPI_ISL_590527 to 590560, EPI_ISL_590562, EPI_ISL_590565 to 590569, EPI_ISL_590571 to 590575, EPI_ISL_590577 to 590592, EPI_ISL_590594, EPI_ISL_590596 to 590614, EPI_ISL_590616 to 590627, EPI_ISL_590629 to 590631, EPI_ISL_590633 to 590643, EPI_ISL_590645 to 590652, EPI_ISL_590654 to 590658, EPI_ISL_590660 to 590663, EPI_ISL_590665 to 590679, EPI_ISL_590681 to 590688, EPI_ISL_597234 to 597236, EPI_ISL_597240, EPI_ISL_597242, EPI_ISL_597244, EPI_ISL_597249, EPI_ISL_597252, EPI_ISL_597260 to 597261, EPI_ISL_597263, EPI_ISL_597267 to 597268, EPI_ISL_597272, EPI_ISL_597275 to 597276, EPI_ISL_597278, EPI_ISL_597280 to 597281, EPI_ISL_597288, EPI_ISL_597290 to 597292, EPI_ISL_597296, EPI_ISL_597298 to 597299, EPI_ISL_597302 to 597303, EPI_ISL_597311 to 597312, EPI_ISL_597314 to 597315, EPI_ISL_597318, EPI_ISL_597322, EPI_ISL_597326, EPI_ISL_597328 to 597330, EPI_ISL_597332 to 597334, EPI_ISL_597336, EPI_ISL_597338 to 597339, EPI_ISL_597347 to 597348, EPI_ISL_597351 to 597353, EPI_ISL_597355 to 597357, EPI_ISL_597359 to 597360, EPI_ISL_597362 to 597364, EPI_ISL_597369 to 597370, EPI_ISL_597376, EPI_ISL_597379, EPI_ISL_597382 to 597386, EPI_ISL_597389, EPI_ISL_597391, EPI_ISL_597393 to 597395, EPI_ISL_597400, EPI_ISL_597404, EPI_ISL_597406 to 597411, EPI_ISL_597413, EPI_ISL_597415 to 597417, EPI_ISL_597419 to 597423, EPI_ISL_597430 to 597431, EPI_ISL_597434, EPI_ISL_597436, EPI_ISL_597439 to 597440, EPI_ISL_597442 to 597444, EPI_ISL_597447 to 597448, EPI_ISL_597452, EPI_ISL_597454 to 597456, EPI_ISL_597471, EPI_ISL_597474 to 597475, EPI_ISL_597481, EPI_ISL_597484 to 597485, EPI_ISL_597491 to 597493, EPI_ISL_597496, EPI_ISL_597498 to 597501, EPI_ISL_597503, EPI_ISL_597506, EPI_ISL_597509, EPI_ISL_597511, EPI_ISL_597513 to 597516, EPI_ISL_597518, EPI_ISL_597521, EPI_ISL_598831 to 598847, EPI_ISL_598849 to 598874, EPI_ISL_598876 to 598889, EPI_ISL_598891 to 598918, EPI_ISL_598920 to 598931, EPI_ISL_598933 to 598935, EPI_ISL_598937 to 598944, EPI_ISL_598946 to 598958, EPI_ISL_598960 to 598969, EPI_ISL_598971 to 598983, EPI_ISL_598985 to 598989, EPI_ISL_598991 to 599009, EPI_ISL_599011 to 599042, EPI_ISL_599044 to 599048, EPI_ISL_599050 to 599074, EPI_ISL_599076, EPI_ISL_599078 to 599100, EPI_ISL_599103 to 599105, EPI_ISL_599107 to 599113, EPI_ISL_599115 to 599118, EPI_ISL_599120 to 599131, EPI_ISL_599133 to 599164, EPI_ISL_599167, EPI_ISL_599169 to 599182, EPI_ISL_599184 to 599193, EPI_ISL_599195 to 599200, EPI_ISL_599202 to 599204, EPI_ISL_599206 to 599220, EPI_ISL_599222 to 599227, EPI_ISL_599229 to 599252, EPI_ISL_599254 to 599264, EPI_ISL_599266 to 599269, EPI_ISL_599271 to 599273, EPI_ISL_599275 to 599280, EPI_ISL_599282 to 599313, EPI_ISL_599315 to 599319, EPI_ISL_599322 to 599344, EPI_ISL_599346, EPI_ISL_599348, EPI_ISL_599350 to 599359, EPI_ISL_599361, EPI_ISL_599363 to 599371, EPI_ISL_599373 to 599385, EPI_ISL_599387 to 599394, EPI_ISL_599396 to 599401, EPI_ISL_599403 to 599411, EPI_ISL_599414 to 599421, EPI_ISL_599423 to 599433, EPI_ISL_599435 to 599462, EPI_ISL_599464 to 599473, EPI_ISL_599475 to 599477, EPI_ISL_599479 to 599482, EPI_ISL_599485, EPI_ISL_599487 to 599489, EPI_ISL_599491 to 599501, EPI_ISL_599503 to 599506, EPI_ISL_599508 to 599514, EPI_ISL_599517 to 599523, EPI_ISL_599525 to 599526, EPI_ISL_599528 to 599535, EPI_ISL_599537, EPI_ISL_599539 to 599552, EPI_ISL_599555, EPI_ISL_599557, EPI_ISL_599561 to 599563, EPI_ISL_599565 to 599575, EPI_ISL_599578 to 599586, EPI_ISL_599588 to 599589, EPI_ISL_599591, EPI_ISL_599593 to 599594, EPI_ISL_599596 to 599598, EPI_ISL_599600 to 599602, EPI_ISL_599605 to 599608, EPI_ISL_599610 to 599615, EPI_ISL_599617 to 599626, EPI_ISL_599629 to 599639, EPI_ISL_599642 to 599647, EPI_ISL_599649 to 599662, EPI_ISL_599664 to 599666, EPI_ISL_599668 to 599680, EPI_ISL_599682 to 599698, EPI_ISL_599701 to 599729, EPI_ISL_599732 to 599759, EPI_ISL_599761 to 599762, EPI_ISL_599764, EPI_ISL_599766 to 599776, EPI_ISL_599778 to 599782, EPI_ISL_599785 to 599787, EPI_ISL_599789 to 599802, EPI_ISL_599804 to 599810, EPI_ISL_599813, EPI_ISL_599815, EPI_ISL_599817, EPI_ISL_600128 to 600138, EPI_ISL_600140 to 600168, EPI_ISL_600170 to 600171, EPI_ISL_600173 to 600201, EPI_ISL_600203 to 600205, EPI_ISL_600207 to 600210, EPI_ISL_600212 to 600220, EPI_ISL_600222 to 600232, EPI_ISL_600234, EPI_ISL_600236, EPI_ISL_600238 to 600251, EPI_ISL_600254 to 600259, EPI_ISL_600261 to 600262, EPI_ISL_600264 to 600272, EPI_ISL_600274 to 600296, EPI_ISL_600298 to 600301, EPI_ISL_600303 to 600305, EPI_ISL_600307, EPI_ISL_600309 to 600340, EPI_ISL_600342 to 600345, EPI_ISL_600347 to 600368, EPI_ISL_600371 to 600373, EPI_ISL_600375 to 600376, EPI_ISL_600378 to 600382, EPI_ISL_600384 to 600385, EPI_ISL_600389 to 600392, EPI_ISL_600394, EPI_ISL_600396 to 600404, EPI_ISL_600406 to 600413, EPI_ISL_600415 to 600427, EPI_ISL_600447 to 600448, EPI_ISL_600450, EPI_ISL_600452 to 600453, EPI_ISL_600455, EPI_ISL_600457 to 600458, EPI_ISL_600460, EPI_ISL_600462, EPI_ISL_600464 to 600465, EPI_ISL_600467, EPI_ISL_600469 to 600470, EPI_ISL_600472, EPI_ISL_600474 to 600475, EPI_ISL_600477, EPI_ISL_600479, EPI_ISL_600481 to 600483, EPI_ISL_600485 to 600486, EPI_ISL_600488, EPI_ISL_600490 to 600491, EPI_ISL_600493 to 600494, EPI_ISL_600496 to 600497, EPI_ISL_600499, EPI_ISL_600501, EPI_ISL_600503 to 600504, EPI_ISL_600506, EPI_ISL_600509, EPI_ISL_600511, EPI_ISL_600513 to 600514, EPI_ISL_600516, EPI_ISL_600518, EPI_ISL_600520, EPI_ISL_600539 to 600540, EPI_ISL_600542, EPI_ISL_600544 to 600545, EPI_ISL_600547 to 600548, EPI_ISL_600550 to 600551, EPI_ISL_600553, EPI_ISL_600555 to 600556, EPI_ISL_600558, EPI_ISL_600560 to 600561, EPI_ISL_600563, EPI_ISL_600565 to 600566, EPI_ISL_600568 to 600569, EPI_ISL_600571 to 600592, EPI_ISL_600594 to 600602, EPI_ISL_600604, EPI_ISL_600606 to 600634, EPI_ISL_600636 to 600642, EPI_ISL_600644 to 600687, EPI_ISL_600689 to 600702, EPI_ISL_600704 to 600712, EPI_ISL_600714 to 600718, EPI_ISL_600720 to 600721, EPI_ISL_600723 to 600738, EPI_ISL_600741 to 600758, EPI_ISL_600760 to 600783, EPI_ISL_600785 to 600807, EPI_ISL_600810 to 600817, EPI_ISL_600819 to 600823, EPI_ISL_600825 to 600850, EPI_ISL_600852 to 600863, EPI_ISL_600865 to 600876, EPI_ISL_600878 to 600881, EPI_ISL_600883 to 600884, EPI_ISL_600886 to 600891, EPI_ISL_600893 to 600900, EPI_ISL_600902, EPI_ISL_600907 to 600911, EPI_ISL_600914, EPI_ISL_600916 to 600920, EPI_ISL_600922, EPI_ISL_600924, EPI_ISL_600932 to 600955, EPI_ISL_600957 to 600966, EPI_ISL_600969 to 600973, EPI_ISL_600975 to 600980, EPI_ISL_600982 to 600985, EPI_ISL_600987 to 601001, EPI_ISL_601003 to 601021, EPI_ISL_601024 to 601027, EPI_ISL_601029 to 601036, EPI_ISL_601038 to 601046, EPI_ISL_601048 to 601060, EPI_ISL_601062 to 601065, EPI_ISL_601067 to 601070, EPI_ISL_601072 to 601096, EPI_ISL_601098, EPI_ISL_601100 to 601109, EPI_ISL_601111 to 601149, EPI_ISL_601151 to 601191, EPI_ISL_601193 to 601213, EPI_ISL_601215 to 601223, EPI_ISL_601226 to 601233, EPI_ISL_601236 to 601238, EPI_ISL_601244, EPI_ISL_601246, EPI_ISL_601250 to 601254, EPI_ISL_601257, EPI_ISL_601259 to 601261, EPI_ISL_601265 to 601266, EPI_ISL_601269, EPI_ISL_601271, EPI_ISL_601274, EPI_ISL_601276, EPI_ISL_601279, EPI_ISL_601284 to 601286, EPI_ISL_601288, EPI_ISL_601295 to 601296, EPI_ISL_601300, EPI_ISL_601308 to 601309, EPI_ISL_601315, EPI_ISL_601319, EPI_ISL_601323, EPI_ISL_601331 to 601336, EPI_ISL_601339, EPI_ISL_601341 to 601342, EPI_ISL_601344, EPI_ISL_601347 to 601348, EPI_ISL_601351 to 601353, EPI_ISL_601355 to 601358, EPI_ISL_601362, EPI_ISL_601364, EPI_ISL_601372, EPI_ISL_601374, EPI_ISL_601380, EPI_ISL_601384, EPI_ISL_601386 to 601387, EPI_ISL_601389, EPI_ISL_601391, EPI_ISL_601393, EPI_ISL_601400 to 601403, EPI_ISL_601405, EPI_ISL_601407 to 601408, EPI_ISL_601411, EPI_ISL_601413 to 601414, EPI_ISL_601416, EPI_ISL_601421, EPI_ISL_601423 to 601424, EPI_ISL_601426, EPI_ISL_601429 to 601430, EPI_ISL_601432 to 601433, EPI_ISL_601435 to 601436, EPI_ISL_601445 to 601446, EPI_ISL_601453 to 601454, EPI_ISL_601459 to 601462, EPI_ISL_601466 to 601467, EPI_ISL_601469, EPI_ISL_601471, EPI_ISL_601477, EPI_ISL_601479, EPI_ISL_601481 to 601483, EPI_ISL_601486, EPI_ISL_601488 to 601489, EPI_ISL_601787 to 601796, EPI_ISL_601798 to 601801, EPI_ISL_601804, EPI_ISL_601806 to 601807, EPI_ISL_601809 to 601810, EPI_ISL_601812 to 601817, EPI_ISL_601820 to 601833, EPI_ISL_601835, EPI_ISL_601837 to 601840, EPI_ISL_601842 to 601845, EPI_ISL_601847 to 601855, EPI_ISL_601858 to 601863, EPI_ISL_601865 to 601880, EPI_ISL_601882 to 601923, EPI_ISL_601925 to 601927, EPI_ISL_601930 to 601945, EPI_ISL_601947 to 601949, EPI_ISL_601951 to 601970, EPI_ISL_601972, EPI_ISL_601974 to 601976, EPI_ISL_601978 to 601992, EPI_ISL_601994 to 601995, EPI_ISL_601997 to 601999, EPI_ISL_602001 to 602002, EPI_ISL_602004 to 602005, EPI_ISL_602007 to 602014, EPI_ISL_602016 to 602025, EPI_ISL_602030 to 602031, EPI_ISL_602033 to 602076, EPI_ISL_602078 to 602080, EPI_ISL_602084 to 602089, EPI_ISL_602093 to 602101, EPI_ISL_602104 to 602105, EPI_ISL_602107 to 602108, EPI_ISL_602110 to 602115, EPI_ISL_602117 to 602121, EPI_ISL_602123 to 602127, EPI_ISL_602129 to 602130, EPI_ISL_602132 to 602134, EPI_ISL_602136 to 602144, EPI_ISL_602156, EPI_ISL_609178 to 609179, EPI_ISL_609182, EPI_ISL_609184 to 609186, EPI_ISL_609190 to 609191, EPI_ISL_609194, EPI_ISL_609197 to 609199, EPI_ISL_609202, EPI_ISL_609206, EPI_ISL_611287 to 611297, EPI_ISL_611299 to 611302, EPI_ISL_611305 to 611310, EPI_ISL_611312, EPI_ISL_611314 to 611315, EPI_ISL_611317, EPI_ISL_611320 to 611325, EPI_ISL_611327 to 611345, EPI_ISL_611348 to 611361, EPI_ISL_611363 to 611368, EPI_ISL_611370 to 611375, EPI_ISL_611377, EPI_ISL_611379 to 611387, EPI_ISL_611389 to 611398, EPI_ISL_611400 to 611432, EPI_ISL_611434 to 611442, EPI_ISL_611444 to 611445, EPI_ISL_611447 to 611448, EPI_ISL_611450 to 611453, EPI_ISL_611455, EPI_ISL_611457 to 611458, EPI_ISL_611460 to 611462, EPI_ISL_611464 to 611466, EPI_ISL_611469 to 611475, EPI_ISL_611477 to 611484, EPI_ISL_623863, EPI_ISL_623865, EPI_ISL_623868, EPI_ISL_623873, EPI_ISL_623876, EPI_ISL_623883 to 623884, EPI_ISL_623886, EPI_ISL_623893, EPI_ISL_623895, EPI_ISL_623903, EPI_ISL_623907 to 623908, EPI_ISL_623911, EPI_ISL_623916 to 623917, EPI_ISL_623921, EPI_ISL_623924, EPI_ISL_623930, EPI_ISL_623933 to 623934, EPI_ISL_623942, EPI_ISL_623944, EPI_ISL_623949, EPI_ISL_623954, EPI_ISL_623956, EPI_ISL_623959, EPI_ISL_623963, EPI_ISL_623975 to 623977, EPI_ISL_623986, EPI_ISL_623990 to 623991, EPI_ISL_623994 to 623995, EPI_ISL_624010, EPI_ISL_624018 to 624019, EPI_ISL_624022, EPI_ISL_624027 to 624028, EPI_ISL_624032, EPI_ISL_624034, EPI_ISL_624043, EPI_ISL_624049, EPI_ISL_624067, EPI_ISL_624073, EPI_ISL_624077, EPI_ISL_624092, EPI_ISL_624105, EPI_ISL_624112, EPI_ISL_624114, EPI_ISL_624120, EPI_ISL_624122, EPI_ISL_624132, EPI_ISL_624140, EPI_ISL_624145, EPI_ISL_624147 to 624148, EPI_ISL_624164 to 624165, EPI_ISL_624176, EPI_ISL_624181, EPI_ISL_624188 to 624189, EPI_ISL_625317, EPI_ISL_625319 to 625325, EPI_ISL_625327, EPI_ISL_625329 to 625330, EPI_ISL_625332 to 625333, EPI_ISL_625335 to 625357, EPI_ISL_625359, EPI_ISL_625361 to 625364, EPI_ISL_625366 to 625375, EPI_ISL_625377 to 625395, EPI_ISL_629755 to 629782, EPI_ISL_629785 to 629787, EPI_ISL_629789 to 629806, EPI_ISL_629808 to 629813, EPI_ISL_629815 to 629818, EPI_ISL_629820 to 629822, EPI_ISL_629824 to 629832, EPI_ISL_629834 to 629838, EPI_ISL_629840 to 629848, EPI_ISL_629850 to 629859, EPI_ISL_629861 to 629865, EPI_ISL_629867 to 629876, EPI_ISL_629878 to 629880, EPI_ISL_629884 to 629886, EPI_ISL_629889, EPI_ISL_629891 to 629901, EPI_ISL_629903 to 629906, EPI_ISL_629908, EPI_ISL_629910 to 629911, EPI_ISL_629913, EPI_ISL_629915 to 629931, EPI_ISL_629933 to 629939, EPI_ISL_629941, EPI_ISL_629943 to 629952, EPI_ISL_629954 to 629969, EPI_ISL_629971 to 629973, EPI_ISL_629975 to 629976, EPI_ISL_629979 to 629986, EPI_ISL_629988 to 629993, EPI_ISL_629995 to 629999, EPI_ISL_630001 to 630006, EPI_ISL_630702 to 630705, EPI_ISL_630718 to 630719, EPI_ISL_630725, EPI_ISL_630733, EPI_ISL_630761, EPI_ISL_630764, EPI_ISL_630775, EPI_ISL_630778, EPI_ISL_630782, EPI_ISL_630787, EPI_ISL_630804, EPI_ISL_630813, EPI_ISL_630815, EPI_ISL_630824, EPI_ISL_630827, EPI_ISL_630833, EPI_ISL_630851, EPI_ISL_630853, EPI_ISL_630859, EPI_ISL_630864, EPI_ISL_630871 to 630873, EPI_ISL_630875, EPI_ISL_630883, EPI_ISL_630888, EPI_ISL_630900, EPI_ISL_630915, EPI_ISL_630923, EPI_ISL_630926, EPI_ISL_630931, EPI_ISL_630933, EPI_ISL_630941, EPI_ISL_630948 to 630949, EPI_ISL_630955, EPI_ISL_630959, EPI_ISL_630965, EPI_ISL_633103, EPI_ISL_633111 to 633113, EPI_ISL_633115, EPI_ISL_633119, EPI_ISL_633125, EPI_ISL_633132, EPI_ISL_633137 to 633139, EPI_ISL_633143 to 633146, EPI_ISL_633151 to 633152, EPI_ISL_633154, EPI_ISL_633158, EPI_ISL_633160, EPI_ISL_633162, EPI_ISL_633166 to 633169, EPI_ISL_633171, EPI_ISL_633174 to 633175, EPI_ISL_633178 to 633182, EPI_ISL_633184 to 633185, EPI_ISL_633190 to 633191, EPI_ISL_633193, EPI_ISL_633196, EPI_ISL_633202 to 633204, EPI_ISL_633206 to 633207, EPI_ISL_633209 to 633210, EPI_ISL_633213, EPI_ISL_633222, EPI_ISL_633226, EPI_ISL_633228, EPI_ISL_633230, EPI_ISL_633232 to 633233, EPI_ISL_633237, EPI_ISL_633240 to 633241, EPI_ISL_633243 to 633244, EPI_ISL_633251, EPI_ISL_633256, EPI_ISL_633261, EPI_ISL_633268 to 633269, EPI_ISL_633271 to 633272, EPI_ISL_633274, EPI_ISL_633276, EPI_ISL_633279 to 633280, EPI_ISL_633283, EPI_ISL_633285, EPI_ISL_633287, EPI_ISL_633289, EPI_ISL_633293, EPI_ISL_633296, EPI_ISL_633301, EPI_ISL_633303 to 633307, EPI_ISL_633309 to 633310, EPI_ISL_633312, EPI_ISL_633316 to 633318, EPI_ISL_633320, EPI_ISL_633323, EPI_ISL_633326 to 633328, EPI_ISL_633332, EPI_ISL_633335, EPI_ISL_633338 to 633339, EPI_ISL_633342 to 633343, EPI_ISL_633348, EPI_ISL_633351 to 633355, EPI_ISL_633358, EPI_ISL_633362, EPI_ISL_633364, EPI_ISL_633367, EPI_ISL_633371, EPI_ISL_633376 to 633377, EPI_ISL_633379, EPI_ISL_633381 to 633382, EPI_ISL_633384, EPI_ISL_633386 to 633387, EPI_ISL_633391 to 633392, EPI_ISL_633394, EPI_ISL_633396, EPI_ISL_633400 to 633402, EPI_ISL_633404, EPI_ISL_633406, EPI_ISL_633409 to 633420, EPI_ISL_633422 to 633431, EPI_ISL_633433 to 633442, EPI_ISL_633444 to 633462, EPI_ISL_633464, EPI_ISL_633466 to 633473, EPI_ISL_633475 to 633488, EPI_ISL_633490 to 633491, EPI_ISL_633493 to 633497, EPI_ISL_633499 to 633539, EPI_ISL_633541 to 633566, EPI_ISL_633570 to 633576, EPI_ISL_633578 to 633584, EPI_ISL_633586 to 633588, EPI_ISL_633591 to 633614, EPI_ISL_633616 to 633641, EPI_ISL_633643 to 633645, EPI_ISL_633647 to 633652, EPI_ISL_633654 to 633692, EPI_ISL_633694 to 633695, EPI_ISL_633698 to 633699, EPI_ISL_633701 to 633705, EPI_ISL_633707, EPI_ISL_633709 to 633729, EPI_ISL_633731 to 633750, EPI_ISL_633752 to 633758, EPI_ISL_633761 to 633776, EPI_ISL_633778 to 633780, EPI_ISL_633783 to 633785, EPI_ISL_633787, EPI_ISL_633789 to 633793, EPI_ISL_633795 to 633798, EPI_ISL_633801 to 633805, EPI_ISL_633807 to 633808, EPI_ISL_633810 to 633812, EPI_ISL_633816 to 633820, EPI_ISL_633822 to 633824, EPI_ISL_633826 to 633827, EPI_ISL_633829 to 633830, EPI_ISL_633832, EPI_ISL_633834, EPI_ISL_633836 to 633844, EPI_ISL_633846 to 633848, EPI_ISL_633850, EPI_ISL_633852 to 633859, EPI_ISL_633861 to 633871, EPI_ISL_633873 to 633876, EPI_ISL_633879 to 633885, EPI_ISL_633888, EPI_ISL_633890 to 633892, EPI_ISL_633894 to 633899, EPI_ISL_633901 to 633902, EPI_ISL_633904, EPI_ISL_633906, EPI_ISL_633908 to 633909, EPI_ISL_633911 to 633913, EPI_ISL_633915, EPI_ISL_633917 to 633921, EPI_ISL_633924 to 633936, EPI_ISL_633938 to 633942, EPI_ISL_633944 to 633971, EPI_ISL_633973 to 633987, EPI_ISL_633989 to 634017, EPI_ISL_634019 to 634040, EPI_ISL_634042, EPI_ISL_634044 to 634052, EPI_ISL_634054 to 634062, EPI_ISL_634064 to 634071, EPI_ISL_634073 to 634091, EPI_ISL_634093, EPI_ISL_634095 to 634097, EPI_ISL_634099 to 634106, EPI_ISL_634110 to 634112, EPI_ISL_634114 to 634122, EPI_ISL_634124 to 634126, EPI_ISL_634128 to 634139, EPI_ISL_634141 to 634147, EPI_ISL_634150 to 634155, EPI_ISL_634157 to 634163, EPI_ISL_634165 to 634176, EPI_ISL_634179 to 634186, EPI_ISL_634188 to 634193, EPI_ISL_634195 to 634200, EPI_ISL_634203 to 634220, EPI_ISL_634222 to 634223, EPI_ISL_634226 to 634229, EPI_ISL_634231 to 634237, EPI_ISL_634239 to 634241, EPI_ISL_634243 to 634250, EPI_ISL_634252, EPI_ISL_634520 to 634533, EPI_ISL_634535 to 634536, EPI_ISL_634538 to 634539, EPI_ISL_634541 to 634576, EPI_ISL_634578 to 634589, EPI_ISL_634591 to 634603, EPI_ISL_634605 to 634612, EPI_ISL_634614 to 634636, EPI_ISL_634638 to 634664, EPI_ISL_634666 to 634677, EPI_ISL_634679 to 634703, EPI_ISL_634705 to 634706, EPI_ISL_634708 to 634712, EPI_ISL_634714 to 634718, EPI_ISL_634720 to 634723, EPI_ISL_634725 to 634733, EPI_ISL_634735 to 634736, EPI_ISL_634738 to 634742, EPI_ISL_634744 to 634753

see above	Lighthouse Lab in Glasgow	Wellcome Sanger Institute for the COVID-19 Genomics UK (COG-UK) consortium	Anna Dominiczak and Alex Alderton; Carol Clugston; Cordelia Langford; David Gray; David K. Jackson; Dominic Kwiatkowski; Ewan Harrison; Harper VanSteenhouse; Ian Johnston; John Sillitoe; John Sillitoe on behalf of the Wellcome Sanger Institute COVID-19 Surveillance Team; John Sillitoe on behalf of the Wellcome Sanger Institute COVID-19 Surveillance Team (http://www.sanger.ac.uk/covid-team); Roberto Amato; Sonia Goncalves; Yumi Kasai

EPI_ISL_540824 to 540836, EPI_ISL_540839 to 540844, EPI_ISL_540846 to 540847, EPI_ISL_540849, EPI_ISL_540851 to 540853, EPI_ISL_540855 to 540859, EPI_ISL_540861, EPI_ISL_540863 to 540864, EPI_ISL_540866, EPI_ISL_540870, EPI_ISL_559947 to 559950, EPI_ISL_559952, EPI_ISL_559954 to 559956, EPI_ISL_559958, EPI_ISL_559960, EPI_ISL_559962, EPI_ISL_559964, EPI_ISL_559966 to 559970, EPI_ISL_573769, EPI_ISL_585297, EPI_ISL_585299 to 585300, EPI_ISL_585303 to 585307, EPI_ISL_585310 to 585313, EPI_ISL_585315 to 585326, EPI_ISL_585328 to 585330, EPI_ISL_585332 to 585341, EPI_ISL_585343, EPI_ISL_585347, EPI_ISL_585349, EPI_ISL_585351 to 585354, EPI_ISL_585356 to 585357, EPI_ISL_585359, EPI_ISL_585361 to 585365, EPI_ISL_585367 to 585378, EPI_ISL_585381, EPI_ISL_585385, EPI_ISL_585387, EPI_ISL_585389, EPI_ISL_585391 to 585394, EPI_ISL_585396 to 585397, EPI_ISL_585400, EPI_ISL_585402 to 585407, EPI_ISL_585410 to 585414, EPI_ISL_585417 to 585420, EPI_ISL_651424, EPI_ISL_651790 to 651810, EPI_ISL_651886, EPI_ISL_865004, EPI_ISL_865008, EPI_ISL_865068

see above	Lighthouse Lab in Glasgow / MRC-University of Glasgow Centre for Virus Research	COVID-19 Genomics UK (COG-UK) Consortium	Alasdair MacLean; Alice Broos; Ana da Silva Filipe; Anna Dominiczak; Antonia Ho; Carol Clugston; Daniel Mair; David Gray; David L Robertson; Elihu Aranday-Cortes; Emma Thomson; Harper VanSteenhouse; James Shepherd; Jenna Nichols; Joseph Hughes; Kathy Li; Kathy Smollett; Kyriaki Nomikou; Lily Tong; Marc Niebel; Matthew Holden; Natasha Jesudason; Natasha Johnson; Patawee Asamaphan; Rajiv Shah; Richard Orton; Rory Gunson; Sarah McDonald; Sharif Shaaban; Sreenu Vattipally; Stephen Carmichael; Yumi Kasai

EPI_ISL_642762 to 642778, EPI_ISL_642780 to 642793, EPI_ISL_642795 to 642806, EPI_ISL_642808 to 642812, EPI_ISL_642814 to 642819, EPI_ISL_642821 to 642825, EPI_ISL_642827 to 642832, EPI_ISL_642834 to 642835, EPI_ISL_642837 to 642851, EPI_ISL_642854 to 642867, EPI_ISL_642869, EPI_ISL_642871 to 642874, EPI_ISL_642876 to 642880, EPI_ISL_642883 to 642899, EPI_ISL_642901 to 642902, EPI_ISL_642904 to 642911, EPI_ISL_642913 to 642928, EPI_ISL_642930 to 642934, EPI_ISL_642937, EPI_ISL_642939, EPI_ISL_642942, EPI_ISL_642945 to 643005, EPI_ISL_643007, EPI_ISL_643009 to 643019, EPI_ISL_643021 to 643028, EPI_ISL_643030 to 643043, EPI_ISL_643984, EPI_ISL_643986 to 643989, EPI_ISL_643991, EPI_ISL_644052 to 644059, EPI_ISL_644061, EPI_ISL_644063 to 644064, EPI_ISL_644066 to 644069, EPI_ISL_644071 to 644079, EPI_ISL_644081 to 644085, EPI_ISL_644087 to 644091, EPI_ISL_644120, EPI_ISL_645221, EPI_ISL_645226, EPI_ISL_645241, EPI_ISL_645244, EPI_ISL_645263 to 645264, EPI_ISL_645285, EPI_ISL_645300, EPI_ISL_645302, EPI_ISL_645308, EPI_ISL_645319, EPI_ISL_645324, EPI_ISL_645327, EPI_ISL_645329, EPI_ISL_645354, EPI_ISL_645360, EPI_ISL_645367, EPI_ISL_645369, EPI_ISL_645374,

EPI_ISL_645383, EPI_ISL_645391, EPI_ISL_645393, EPI_ISL_645395, EPI_ISL_645399, EPI_ISL_645405 to 645406, EPI_ISL_645421, EPI_ISL_645424, EPI_ISL_645441, EPI_ISL_645453, EPI_ISL_645477, EPI_ISL_645489, EPI_ISL_645513, EPI_ISL_645517, EPI_ISL_647807 to 647810, EPI_ISL_647812 to 647818, EPI_ISL_647820 to 647824, EPI_ISL_647826 to 647841, EPI_ISL_647843 to 647846, EPI_ISL_647849 to 647853, EPI_ISL_649192, EPI_ISL_649205, EPI_ISL_649255, EPI_ISL_649283, EPI_ISL_649320, EPI_ISL_649362, EPI_ISL_649386, EPI_ISL_649405, EPI_ISL_649424, EPI_ISL_649479, EPI_ISL_649548 to 649555, EPI_ISL_656685, EPI_ISL_656705, EPI_ISL_656728, EPI_ISL_656731 to 656733, EPI_ISL_656744 to 656746, EPI_ISL_656757, EPI_ISL_656774, EPI_ISL_656777 to 656779, EPI_ISL_656788 to 656789, EPI_ISL_656799, EPI_ISL_656807 to 656808, EPI_ISL_656810 to 656811, EPI_ISL_656824, EPI_ISL_656827, EPI_ISL_656829, EPI_ISL_656834 to 656835, EPI_ISL_656838, EPI_ISL_656840 to 656841, EPI_ISL_656846, EPI_ISL_656848, EPI_ISL_656860, EPI_ISL_656862, EPI_ISL_656865, EPI_ISL_656867, EPI_ISL_656869, EPI_ISL_656871, EPI_ISL_656877, EPI_ISL_656879, EPI_ISL_656887, EPI_ISL_656892 to 656893, EPI_ISL_656906, EPI_ISL_656910, EPI_ISL_656913, EPI_ISL_656922, EPI_ISL_656924, EPI_ISL_656930, EPI_ISL_656932, EPI_ISL_656935 to 656936, EPI_ISL_656942, EPI_ISL_656945, EPI_ISL_656947, EPI_ISL_656950, EPI_ISL_656955, EPI_ISL_656957 to 656958, EPI_ISL_656961 to 656962, EPI_ISL_656965 to 656966, EPI_ISL_656969, EPI_ISL_656973 to 656974, EPI_ISL_656976, EPI_ISL_656979 to 656980, EPI_ISL_656982, EPI_ISL_656987, EPI_ISL_656991, EPI_ISL_656997, EPI_ISL_657003 to 657004, EPI_ISL_657008, EPI_ISL_657011, EPI_ISL_657014, EPI_ISL_657016 to 657017, EPI_ISL_657021, EPI_ISL_657023, EPI_ISL_657026, EPI_ISL_657359 to 657361, EPI_ISL_657365, EPI_ISL_657367, EPI_ISL_657370, EPI_ISL_657373, EPI_ISL_657375, EPI_ISL_657377, EPI_ISL_657381, EPI_ISL_657385 to 657386, EPI_ISL_657390, EPI_ISL_657392 to 657393, EPI_ISL_657396, EPI_ISL_657398, EPI_ISL_657401, EPI_ISL_657405 to 657406, EPI_ISL_657408, EPI_ISL_657410 to 657411, EPI_ISL_657413 to 657414, EPI_ISL_657416 to 657417, EPI_ISL_657419, EPI_ISL_657424 to 657425, EPI_ISL_657428 to 657430, EPI_ISL_657438 to 657441, EPI_ISL_657443, EPI_ISL_657446, EPI_ISL_657451, EPI_ISL_657453, EPI_ISL_657457, EPI_ISL_657459, EPI_ISL_657464, EPI_ISL_657472 to 657473, EPI_ISL_657479, EPI_ISL_657483 to 657484, EPI_ISL_657487 to 657488, EPI_ISL_657490 to 657491, EPI_ISL_657493, EPI_ISL_657497, EPI_ISL_657500, EPI_ISL_657503, EPI_ISL_657505, EPI_ISL_657509 to 657510, EPI_ISL_657512 to 657513, EPI_ISL_657515, EPI_ISL_657518 to 657519, EPI_ISL_657526 to 657527, EPI_ISL_657531 to 657533, EPI_ISL_657535 to 657536, EPI_ISL_657542 to 657544, EPI_ISL_657547, EPI_ISL_657550, EPI_ISL_657553 to 657558, EPI_ISL_657563 to 657564, EPI_ISL_657567 to 657568, EPI_ISL_657570 to 657571, EPI_ISL_657574 to 657575, EPI_ISL_657577, EPI_ISL_657581, EPI_ISL_657583 to 657585, EPI_ISL_657587, EPI_ISL_657589, EPI_ISL_657591, EPI_ISL_657593, EPI_ISL_657596, EPI_ISL_657599, EPI_ISL_657602, EPI_ISL_657605 to 657606, EPI_ISL_657608 to 657611, EPI_ISL_657614 to 657615, EPI_ISL_657625, EPI_ISL_657627, EPI_ISL_657629, EPI_ISL_657631 to 657632, EPI_ISL_657635 to 657636, EPI_ISL_657639 to 657640, EPI_ISL_657642, EPI_ISL_657644, EPI_ISL_657647 to 657648, EPI_ISL_657650 to 657651, EPI_ISL_657657, EPI_ISL_657662 to 657663, EPI_ISL_657665 to 657666, EPI_ISL_657676, EPI_ISL_657680 to 657682, EPI_ISL_657688, EPI_ISL_657694, EPI_ISL_657696, EPI_ISL_657700, EPI_ISL_657703, EPI_ISL_657705 to 657706, EPI_ISL_657709 to 657712, EPI_ISL_657714 to 657718, EPI_ISL_657722, EPI_ISL_657727, EPI_ISL_657729 to 657730, EPI_ISL_657732 to 657735, EPI_ISL_657738, EPI_ISL_657740 to 657743, EPI_ISL_657745, EPI_ISL_657751 to 657753, EPI_ISL_657755, EPI_ISL_657757 to 657758, EPI_ISL_657768, EPI_ISL_657770, EPI_ISL_657772 to 657777, EPI_ISL_657779, EPI_ISL_657781, EPI_ISL_657783, EPI_ISL_657785, EPI_ISL_657788 to 657789, EPI_ISL_657791 to 657792, EPI_ISL_657794, EPI_ISL_657796 to 657797, EPI_ISL_657808, EPI_ISL_657814, EPI_ISL_657819, EPI_ISL_657822 to 657823, EPI_ISL_657827, EPI_ISL_657829 to 657830, EPI_ISL_657832, EPI_ISL_657835 to 657836, EPI_ISL_657843, EPI_ISL_657850, EPI_ISL_657854, EPI_ISL_657858 to 657859, EPI_ISL_657862, EPI_ISL_657865, EPI_ISL_657870, EPI_ISL_657872, EPI_ISL_657874, EPI_ISL_657879, EPI_ISL_657886 to 657887, EPI_ISL_657889, EPI_ISL_657892, EPI_ISL_657894, EPI_ISL_657898, EPI_ISL_657901, EPI_ISL_657906, EPI_ISL_657910, EPI_ISL_657913, EPI_ISL_657915 to 657916, EPI_ISL_657918 to 657919, EPI_ISL_657921 to 657923, EPI_ISL_657925, EPI_ISL_657927 to 657930, EPI_ISL_657932, EPI_ISL_657938, EPI_ISL_657941, EPI_ISL_657948, EPI_ISL_657950, EPI_ISL_657954, EPI_ISL_657956, EPI_ISL_657958, EPI_ISL_657961, EPI_ISL_657963, EPI_ISL_657965, EPI_ISL_657970 to 657973, EPI_ISL_657975, EPI_ISL_657977, EPI_ISL_657980 to 657982, EPI_ISL_657984 to 657985, EPI_ISL_657988, EPI_ISL_657993, EPI_ISL_657995 to 657996, EPI_ISL_657999, EPI_ISL_658002 to 658006, EPI_ISL_658009, EPI_ISL_658015 to 658018, EPI_ISL_658021 to 658023, EPI_ISL_658025 to 658027, EPI_ISL_658035, EPI_ISL_658038 to 658040, EPI_ISL_658042, EPI_ISL_658044, EPI_ISL_658047, EPI_ISL_658049 to 658051, EPI_ISL_658053, EPI_ISL_658057 to 658061, EPI_ISL_658065 to 658067, EPI_ISL_658070, EPI_ISL_658079, EPI_ISL_658081 to 658082, EPI_ISL_658087 to 658088, EPI_ISL_658090, EPI_ISL_658094, EPI_ISL_658102 to 658103, EPI_ISL_658105 to 658109, EPI_ISL_658111, EPI_ISL_658113, EPI_ISL_658117 to 658118, EPI_ISL_658121, EPI_ISL_658123, EPI_ISL_658126 to 658127, EPI_ISL_658129 to 658130, EPI_ISL_658132, EPI_ISL_658135 to 658137, EPI_ISL_658139 to 658141, EPI_ISL_658143, EPI_ISL_658145 to 658147, EPI_ISL_658150 to 658152, EPI_ISL_658154, EPI_ISL_658168 to 658170, EPI_ISL_658172 to 658174, EPI_ISL_658176, EPI_ISL_658180, EPI_ISL_658182 to 658185, EPI_ISL_658193, EPI_ISL_658204, EPI_ISL_658210 to 658211, EPI_ISL_658213, EPI_ISL_658216, EPI_ISL_658219, EPI_ISL_658222 to 658223, EPI_ISL_658225, EPI_ISL_658229 to 658230, EPI_ISL_658233, EPI_ISL_658235, EPI_ISL_658237 to 658238, EPI_ISL_658241, EPI_ISL_658243 to 658245, EPI_ISL_658247 to 658248, EPI_ISL_658251, EPI_ISL_658254 to 658255, EPI_ISL_658257, EPI_ISL_658261 to 658262, EPI_ISL_658264 to 658265, EPI_ISL_658268, EPI_ISL_658273, EPI_ISL_658277, EPI_ISL_658280 to 658284, EPI_ISL_658288, EPI_ISL_658290 to 658291, EPI_ISL_658293 to 658298, EPI_ISL_658300 to 658302, EPI_ISL_658306, EPI_ISL_658308 to 658309, EPI_ISL_658315, EPI_ISL_658318, EPI_ISL_658322, EPI_ISL_658324 to 658325, EPI_ISL_658328 to 658329, EPI_ISL_658333, EPI_ISL_658336, EPI_ISL_658338 to 658339, EPI_ISL_658342, EPI_ISL_658344, EPI_ISL_658346 to 658347, EPI_ISL_658349, EPI_ISL_658354, EPI_ISL_658357 to 658359, EPI_ISL_658365, EPI_ISL_658369, EPI_ISL_658372 to 658375, EPI_ISL_658377 to 658379, EPI_ISL_658384 to 658386, EPI_ISL_658389, EPI_ISL_658391 to 658393, EPI_ISL_658395, EPI_ISL_658399, EPI_ISL_658403, EPI_ISL_658741 to 658743, EPI_ISL_658746 to 658747, EPI_ISL_658751, EPI_ISL_658754 to 658755, EPI_ISL_658757 to 658758, EPI_ISL_658761, EPI_ISL_658765, EPI_ISL_658770, EPI_ISL_658772 to 658774, EPI_ISL_658776 to 658780, EPI_ISL_658782 to 658786, EPI_ISL_658789, EPI_ISL_658792 to 658793, EPI_ISL_658796 to 658799, EPI_ISL_658801 to 658803, EPI_ISL_658806, EPI_ISL_658808, EPI_ISL_658810 to 658815, EPI_ISL_658817 to 658823, EPI_ISL_658825, EPI_ISL_658827 to 658828, EPI_ISL_658830 to 658832, EPI_ISL_658834 to 658835, EPI_ISL_658839, EPI_ISL_658841 to 658843, EPI_ISL_658845, EPI_ISL_658847, EPI_ISL_658849 to 658851, EPI_ISL_658853 to 658855, EPI_ISL_658857 to 658858, EPI_ISL_658860 to 658862, EPI_ISL_658867, EPI_ISL_658869, EPI_ISL_658871 to 658872, EPI_ISL_658874, EPI_ISL_658876, EPI_ISL_658879, EPI_ISL_658882, EPI_ISL_658884, EPI_ISL_658887, EPI_ISL_658889 to 658890, EPI_ISL_658892, EPI_ISL_658894 to 658895, EPI_ISL_658897 to 658898, EPI_ISL_658902, EPI_ISL_658908, EPI_ISL_658910 to 658911, EPI_ISL_658915, EPI_ISL_658918, EPI_ISL_658920, EPI_ISL_658922, EPI_ISL_658924, EPI_ISL_658926 to 658929, EPI_ISL_658932, EPI_ISL_658934 to 658935, EPI_ISL_658937, EPI_ISL_658939, EPI_ISL_658941 to 658942, EPI_ISL_658944, EPI_ISL_658946 to 658947, EPI_ISL_658950 to 658953, EPI_ISL_658955 to 658957, EPI_ISL_658962, EPI_ISL_658965 to 658968, EPI_ISL_658972 to 658975, EPI_ISL_658982 to 658985, EPI_ISL_658987 to 658993, EPI_ISL_658997, EPI_ISL_658999, EPI_ISL_659001 to 659002, EPI_ISL_659004, EPI_ISL_659006, EPI_ISL_659008 to 659010, EPI_ISL_659012, EPI_ISL_659014, EPI_ISL_659020, EPI_ISL_659023 to 659024, EPI_ISL_659026 to 659027, EPI_ISL_659029 to 659034, EPI_ISL_659036 to 659039, EPI_ISL_659042 to 659044, EPI_ISL_659047, EPI_ISL_659049, EPI_ISL_659051, EPI_ISL_659055 to 659056, EPI_ISL_659058, EPI_ISL_659060 to 659066, EPI_ISL_659068 to 659069, EPI_ISL_659074 to 659075, EPI_ISL_659078, EPI_ISL_659081, EPI_ISL_659085 to 659087, EPI_ISL_659089, EPI_ISL_659092, EPI_ISL_659095 to 659096, EPI_ISL_662633 to 662634, EPI_ISL_662636 to 662638, EPI_ISL_662640, EPI_ISL_662643 to 662646, EPI_ISL_673577, EPI_ISL_673594, EPI_ISL_673599, EPI_ISL_673601, EPI_ISL_673603, EPI_ISL_673621, EPI_ISL_673633, EPI_ISL_673642, EPI_ISL_673646, EPI_ISL_673655, EPI_ISL_673657, EPI_ISL_673659, EPI_ISL_673663, EPI_ISL_673673, EPI_ISL_673688, EPI_ISL_673696, EPI_ISL_673702, EPI_ISL_673708 to 673709, EPI_ISL_673715, EPI_ISL_673726, EPI_ISL_673729 to 673730, EPI_ISL_673733, EPI_ISL_673745, EPI_ISL_673750, EPI_ISL_673760, EPI_ISL_673765, EPI_ISL_673767, EPI_ISL_673785, EPI_ISL_673787, EPI_ISL_673790, EPI_ISL_673796, EPI_ISL_673798, EPI_ISL_673801, EPI_ISL_673803, EPI_ISL_673812 to 673814, EPI_ISL_673821, EPI_ISL_673828 to 673829, EPI_ISL_673832 to 673835, EPI_ISL_673848, EPI_ISL_673856 to 673857, EPI_ISL_673859, EPI_ISL_673865, EPI_ISL_673867, EPI_ISL_673871, EPI_ISL_673873 to 673877, EPI_ISL_673880 to 673881, EPI_ISL_673884 to 673887, EPI_ISL_673893 to 673899, EPI_ISL_673901 to 673903, EPI_ISL_673905 to 673914, EPI_ISL_673916 to 673917, EPI_ISL_673919 to 673925, EPI_ISL_673927, EPI_ISL_673929 to 673932, EPI_ISL_673934 to 673939, EPI_ISL_673941, EPI_ISL_673943 to 673946, EPI_ISL_673949, EPI_ISL_673951 to 673955, EPI_ISL_673957 to 673959, EPI_ISL_673961, EPI_ISL_673963 to 673970, EPI_ISL_673972 to 673979, EPI_ISL_673981 to 673986, EPI_ISL_673988 to 673989, EPI_ISL_673991, EPI_ISL_673993 to 673997, EPI_ISL_674000 to 674004, EPI_ISL_674007 to 674014, EPI_ISL_674016, EPI_ISL_674020 to 674028, EPI_ISL_674030 to 674036, EPI_ISL_674039 to 674049, EPI_ISL_674051 to 674052, EPI_ISL_674054 to 674061, EPI_ISL_674063 to 674071, EPI_ISL_674073 to 674077, EPI_ISL_674079 to 674081, EPI_ISL_674085, EPI_ISL_674088 to 674091, EPI_ISL_674093 to 674107, EPI_ISL_674109 to 674111, EPI_ISL_674113 to 674115, EPI_ISL_674117, EPI_ISL_674119 to 674124, EPI_ISL_674126 to 674127, EPI_ISL_674129 to 674132, EPI_ISL_674134 to 674135, EPI_ISL_674137 to 674143, EPI_ISL_674145, EPI_ISL_674147 to 674154, EPI_ISL_674167, EPI_ISL_674173, EPI_ISL_674178, EPI_ISL_674183 to 674185, EPI_ISL_674187, EPI_ISL_674189, EPI_ISL_674191, EPI_ISL_674196, EPI_ISL_674198, EPI_ISL_674200, EPI_ISL_674202, EPI_ISL_674205, EPI_ISL_674209 to 674210, EPI_ISL_674212 to 674214, EPI_ISL_674216, EPI_ISL_674219 to 674221, EPI_ISL_674225, EPI_ISL_674230, EPI_ISL_674232, EPI_ISL_674236, EPI_ISL_674238 to 674239, EPI_ISL_674244, EPI_ISL_674246 to 674248, EPI_ISL_674250 to 674252, EPI_ISL_674257 to 674259, EPI_ISL_674263, EPI_ISL_674265, EPI_ISL_674269, EPI_ISL_674274 to 674275, EPI_ISL_674279 to 674280, EPI_ISL_674282, EPI_ISL_674289 to 674291, EPI_ISL_674293, EPI_ISL_674298, EPI_ISL_674302 to 674304, EPI_ISL_674306, EPI_ISL_674309, EPI_ISL_674314 to 674316, EPI_ISL_674320 to 674321, EPI_ISL_674325 to 674326, EPI_ISL_674330 to 674331, EPI_ISL_674333, EPI_ISL_674335 to 674336, EPI_ISL_674342, EPI_ISL_674344, EPI_ISL_674349, EPI_ISL_674355 to 674356, EPI_ISL_674358 to 674359, EPI_ISL_674363, EPI_ISL_674365, EPI_ISL_674371, EPI_ISL_674373, EPI_ISL_674378 to 674379, EPI_ISL_674381 to 674382, EPI_ISL_674384, EPI_ISL_674386, EPI_ISL_674388, EPI_ISL_674398, EPI_ISL_674401, EPI_ISL_674403, EPI_ISL_674405, EPI_ISL_674410, EPI_ISL_674414 to 674415, EPI_ISL_674417, EPI_ISL_674422 to 674423, EPI_ISL_674426 to 674427, EPI_ISL_674430, EPI_ISL_674433, EPI_ISL_674437, EPI_ISL_674440, EPI_ISL_674443 to 674444, EPI_ISL_674448, EPI_ISL_674450, EPI_ISL_674453, EPI_ISL_674457 to 674458, EPI_ISL_674460, EPI_ISL_674464, EPI_ISL_674468, EPI_ISL_674473, EPI_ISL_674475, EPI_ISL_674480 to 674483, EPI_ISL_674491 to 674492, EPI_ISL_674494, EPI_ISL_674496 to 674497, EPI_ISL_674503, EPI_ISL_674505, EPI_ISL_674507 to 674513, EPI_ISL_674515, EPI_ISL_674517, EPI_ISL_674519, EPI_ISL_674521, EPI_ISL_674523 to 674526, EPI_ISL_674529, EPI_ISL_674531 to 674536, EPI_ISL_674538 to 674542, EPI_ISL_674547 to 674549, EPI_ISL_674553 to 674556, EPI_ISL_674560 to 674562, EPI_ISL_674564, EPI_ISL_674566 to 674568, EPI_ISL_674570, EPI_ISL_674572 to 674575, EPI_ISL_674577 to 674578, EPI_ISL_674580 to 674582, EPI_ISL_674586 to 674587, EPI_ISL_674591, EPI_ISL_674593 to 674594, EPI_ISL_674596 to 674602, EPI_ISL_674604, EPI_ISL_674606, EPI_ISL_674608, EPI_ISL_674610 to 674611, EPI_ISL_674614 to 674615, EPI_ISL_674617 to 674624, EPI_ISL_674626 to 674628, EPI_ISL_674631 to 674632, EPI_ISL_674634, EPI_ISL_674636, EPI_ISL_674638 to 674644, EPI_ISL_674646 to 674651, EPI_ISL_674654, EPI_ISL_674656, EPI_ISL_674660, EPI_ISL_674663 to 674664, EPI_ISL_674666, EPI_ISL_674668 to 674669, EPI_ISL_674673, EPI_ISL_674675 to 674676, EPI_ISL_674679 to 674684, EPI_ISL_674688 to 674689, EPI_ISL_674693 to 674695, EPI_ISL_674697, EPI_ISL_674699 to 674702, EPI_ISL_674704 to 674706, EPI_ISL_674708 to 674710, EPI_ISL_674712, EPI_ISL_674719 to 674721, EPI_ISL_674728 to 674735, EPI_ISL_674737 to 674739, EPI_ISL_674742, EPI_ISL_674748, EPI_ISL_674750, EPI_ISL_674753 to 674755, EPI_ISL_674757, EPI_ISL_674761, EPI_ISL_674764 to 674769, EPI_ISL_674771, EPI_ISL_674774, EPI_ISL_674777 to 674778, EPI_ISL_674784 to 674785, EPI_ISL_674787 to 674790, EPI_ISL_674793, EPI_ISL_674796 to 674800, EPI_ISL_674803, EPI_ISL_674805, EPI_ISL_674810 to 674822, EPI_ISL_674824 to 674825, EPI_ISL_674827, EPI_ISL_674829 to 674833, EPI_ISL_674835 to 674836, EPI_ISL_674839, EPI_ISL_674841 to 674842, EPI_ISL_674844, EPI_ISL_674848, EPI_ISL_674853, EPI_ISL_674855 to 674858, EPI_ISL_674860 to 674861, EPI_ISL_674863 to 674865, EPI_ISL_674869 to 674870, EPI_ISL_674874 to 674875, EPI_ISL_674877 to 674881, EPI_ISL_674884 to 674887, EPI_ISL_674889 to 674890, EPI_ISL_674892, EPI_ISL_674894 to 674895, EPI_ISL_674897 to 674898, EPI_ISL_674901, EPI_ISL_674904 to 674905, EPI_ISL_674907, EPI_ISL_674911, EPI_ISL_674914 to 674916, EPI_ISL_674918, EPI_ISL_674921, EPI_ISL_674928 to 674932, EPI_ISL_674934 to 674935, EPI_ISL_674937 to 674938, EPI_ISL_674943, EPI_ISL_674945, EPI_ISL_674947 to 674948, EPI_ISL_674954, EPI_ISL_674956, EPI_ISL_674958, EPI_ISL_674960 to 674961, EPI_ISL_674963 to 674971, EPI_ISL_674974 to 674976, EPI_ISL_674978 to 674982, EPI_ISL_674984 to 674985, EPI_ISL_674987, EPI_ISL_674989, EPI_ISL_674991, EPI_ISL_674993 to 674994, EPI_ISL_674996 to 674997, EPI_ISL_674999 to 675000, EPI_ISL_675002 to 675003, EPI_ISL_675005, EPI_ISL_675007, EPI_ISL_675010, EPI_ISL_675013 to 675015, EPI_ISL_675017, EPI_ISL_675020, EPI_ISL_675022 to 675023, EPI_ISL_675025, EPI_ISL_675028 to 675032, EPI_ISL_675034 to 675037, EPI_ISL_675041, EPI_ISL_675043, EPI_ISL_675045, EPI_ISL_675047 to 675052, EPI_ISL_675054 to 675055, EPI_ISL_675057, EPI_ISL_675060 to 675063, EPI_ISL_675065 to 675070, EPI_ISL_675072, EPI_ISL_675074, EPI_ISL_675076 to 675079, EPI_ISL_675083 to 675086, EPI_ISL_675088 to 675090, EPI_ISL_675092 to 675093, EPI_ISL_675095, EPI_ISL_675097, EPI_ISL_675099 to 675101, EPI_ISL_675103, EPI_ISL_675105 to 675106, EPI_ISL_675108 to 675109, EPI_ISL_675111, EPI_ISL_675113 to 675116, EPI_ISL_675119, EPI_ISL_675121 to 675122, EPI_ISL_675124 to 675125, EPI_ISL_675127, EPI_ISL_675131 to 675132, EPI_ISL_675134, EPI_ISL_675145, EPI_ISL_675153, EPI_ISL_675160, EPI_ISL_675165, EPI_ISL_675170, EPI_ISL_675178 to 675179, EPI_ISL_675183, EPI_ISL_675185, EPI_ISL_675210, EPI_ISL_675212, EPI_ISL_675217, EPI_ISL_675219, EPI_ISL_675224, EPI_ISL_675228, EPI_ISL_675238, EPI_ISL_675241, EPI_ISL_675243 to 675244, EPI_ISL_675260, EPI_ISL_675264, EPI_ISL_675275 to 675277, EPI_ISL_675282, EPI_ISL_675284 to 675285, EPI_ISL_675292, EPI_ISL_675301, EPI_ISL_675305 to 675308, EPI_ISL_675311, EPI_ISL_675313, EPI_ISL_675317 to 675318, EPI_ISL_675320, EPI_ISL_675326, EPI_ISL_675331 to 675332, EPI_ISL_675340, EPI_ISL_675357, EPI_ISL_675364, EPI_ISL_675368, EPI_ISL_675371, EPI_ISL_675389, EPI_ISL_675397, EPI_ISL_675399, EPI_ISL_675404, EPI_ISL_675417, EPI_ISL_675420, EPI_ISL_675423 to 675424, EPI_ISL_675432, EPI_ISL_675436, EPI_ISL_675439, EPI_ISL_675443 to 675444, EPI_ISL_675446, EPI_ISL_675462, EPI_ISL_675467, EPI_ISL_675472, EPI_ISL_675475, EPI_ISL_675480, EPI_ISL_675486, EPI_ISL_675495 to 675496, EPI_ISL_675499 to 675500, EPI_ISL_675503 to 675505, EPI_ISL_675507, EPI_ISL_675513, EPI_ISL_675518, EPI_ISL_675533, EPI_ISL_675535, EPI_ISL_675538, EPI_ISL_675542, EPI_ISL_675547, EPI_ISL_675550, EPI_ISL_675556, EPI_ISL_675564 to 675565, EPI_ISL_675576, EPI_ISL_675578, EPI_ISL_675601 to 675602, EPI_ISL_675621, EPI_ISL_675631, EPI_ISL_675634, EPI_ISL_675644, EPI_ISL_675652, EPI_ISL_675660, EPI_ISL_675663, EPI_ISL_675667, EPI_ISL_675676, EPI_ISL_675682, EPI_ISL_675693 to 675694, EPI_ISL_675698, EPI_ISL_675711 to 675713, EPI_ISL_675717, EPI_ISL_675722 to 675726, EPI_ISL_675729, EPI_ISL_675731 to 675732, EPI_ISL_675734, EPI_ISL_675737, EPI_ISL_675741, EPI_ISL_675743, EPI_ISL_675747, EPI_ISL_675749, EPI_ISL_675752 to 675755, EPI_ISL_675762 to 675763, EPI_ISL_675765, EPI_ISL_675772, EPI_ISL_675781, EPI_ISL_675783 to 675784, EPI_ISL_675786 to 675787, EPI_ISL_675790 to 675792, EPI_ISL_675795, EPI_ISL_675804 to 675805, EPI_ISL_675807 to 675809, EPI_ISL_675811, EPI_ISL_675814 to 675816, EPI_ISL_675819, EPI_ISL_675821, EPI_ISL_675824, EPI_ISL_675828, EPI_ISL_675834 to 675835, EPI_ISL_675837 to 675838, EPI_ISL_675840, EPI_ISL_675843 to 675844, EPI_ISL_675849 to 675851, EPI_ISL_675853 to 675854, EPI_ISL_675857, EPI_ISL_675859, EPI_ISL_675862 to 675863, EPI_ISL_675865 to 675867, EPI_ISL_675871 to 675874, EPI_ISL_675876 to 675877, EPI_ISL_675879, EPI_ISL_675881, EPI_ISL_675884, EPI_ISL_675886, EPI_ISL_675888 to 675889, EPI_ISL_675891, EPI_ISL_675893, EPI_ISL_675898 to 675900, EPI_ISL_675904 to 675905, EPI_ISL_675907, EPI_ISL_675909 to 675912, EPI_ISL_675914 to 675915, EPI_ISL_675918 to 675919, EPI_ISL_675921 to 675922, EPI_ISL_675926 to 675927, EPI_ISL_675931, EPI_ISL_675933, EPI_ISL_675935 to 675936, EPI_ISL_675938, EPI_ISL_675940, EPI_ISL_675943, EPI_ISL_675946 to 675947, EPI_ISL_675949 to 675950, EPI_ISL_675953, EPI_ISL_675957 to 675961, EPI_ISL_675963 to 675964, EPI_ISL_675971, EPI_ISL_675975, EPI_ISL_675981, EPI_ISL_675983 to 675985, EPI_ISL_675990 to 675991, EPI_ISL_675996 to 675999, EPI_ISL_676001, EPI_ISL_676003 to 676005, EPI_ISL_676007 to 676009, EPI_ISL_676011, EPI_ISL_676013 to 676019, EPI_ISL_676021 to 676025, EPI_ISL_676027 to 676029, EPI_ISL_676031, EPI_ISL_676033, EPI_ISL_676035, EPI_ISL_676037 to 676038, EPI_ISL_676040 to 676044, EPI_ISL_676046 to 676053, EPI_ISL_676056 to 676063, EPI_ISL_676067 to 676071, EPI_ISL_676073, EPI_ISL_676075 to 676077, EPI_ISL_676079, EPI_ISL_676081 to 676083, EPI_ISL_676085 to 676092, EPI_ISL_676094, EPI_ISL_676096 to 676097, EPI_ISL_676099 to 676104, EPI_ISL_676106 to 676111, EPI_ISL_676113, EPI_ISL_676115 to 676119, EPI_ISL_676121 to 676122, EPI_ISL_676124 to 676127, EPI_ISL_676129 to 676135, EPI_ISL_676137 to 676139, EPI_ISL_676141 to 676142, EPI_ISL_676147 to 676150, EPI_ISL_676152, EPI_ISL_676154, EPI_ISL_676156 to 676159, EPI_ISL_676161 to 676169, EPI_ISL_676171, EPI_ISL_676173 to 676174, EPI_ISL_676176, EPI_ISL_676178, EPI_ISL_676180 to 676182, EPI_ISL_676186 to 676193, EPI_ISL_676195 to 676196, EPI_ISL_676198 to 676201, EPI_ISL_676203 to 676204, EPI_ISL_676206 to 676210, EPI_ISL_676212 to 676217, EPI_ISL_676219, EPI_ISL_676221, EPI_ISL_676223, EPI_ISL_676226 to 676229, EPI_ISL_676231 to 676258, EPI_ISL_676260, EPI_ISL_676262, EPI_ISL_676266 to 676269, EPI_ISL_676271 to 676276, EPI_ISL_676280 to 676281, EPI_ISL_676283 to 676288, EPI_ISL_676290 to 676300, EPI_ISL_676302 to 676303, EPI_ISL_676306, EPI_ISL_676308, EPI_ISL_676310 to 676311, EPI_ISL_681325 to 681338, EPI_ISL_681341 to 681344, EPI_ISL_681346, EPI_ISL_681348 to 681366, EPI_ISL_681369 to 681376, EPI_ISL_681378 to 681380, EPI_ISL_681382 to 681384, EPI_ISL_681386 to 681387, EPI_ISL_681389, EPI_ISL_681391 to 681409, EPI_ISL_681411 to 681412, EPI_ISL_681414 to 681422, EPI_ISL_681426 to 681434, EPI_ISL_681437 to 681438, EPI_ISL_681440, EPI_ISL_681442 to 681444, EPI_ISL_681447 to 681452, EPI_ISL_681454 to 681457, EPI_ISL_681460 to 681462, EPI_ISL_681467, EPI_ISL_681469 to 681471, EPI_ISL_681474 to 681476, EPI_ISL_681478 to 681481, EPI_ISL_681484 to 681485, EPI_ISL_681487 to 681490, EPI_ISL_681492 to 681504, EPI_ISL_681506 to 681512, EPI_ISL_681515 to 681519, EPI_ISL_681521 to 681522, EPI_ISL_681524, EPI_ISL_681526 to 681527, EPI_ISL_681530 to 681532, EPI_ISL_681534 to 681538, EPI_ISL_681540 to 681542, EPI_ISL_681545 to 681546, EPI_ISL_681548 to 681557, EPI_ISL_681559 to 681560, EPI_ISL_681562, EPI_ISL_681566, EPI_ISL_681569 to 681580, EPI_ISL_681582 to 681584, EPI_ISL_681586 to 681587, EPI_ISL_681589 to 681594, EPI_ISL_681597 to 681598, EPI_ISL_681600 to 681605, EPI_ISL_681607 to 681608, EPI_ISL_681610 to 681611, EPI_ISL_681613 to 681618, EPI_ISL_681620 to 681628, EPI_ISL_681630 to 681633, EPI_ISL_681635 to 681636, EPI_ISL_681638 to 681642, EPI_ISL_681644, EPI_ISL_681646, EPI_ISL_681648, EPI_ISL_681650 to 681652, EPI_ISL_681654 to 681655, EPI_ISL_681660, EPI_ISL_681666 to 681668, EPI_ISL_681670, EPI_ISL_705191 to 705192, EPI_ISL_705196, EPI_ISL_705199 to 705200, EPI_ISL_705202, EPI_ISL_708847 to 708848, EPI_ISL_708850, EPI_ISL_708857, EPI_ISL_708862, EPI_ISL_708871, EPI_ISL_708879, EPI_ISL_708889, EPI_ISL_708896 to 708898, EPI_ISL_708900, EPI_ISL_708912 to 708913, EPI_ISL_708916, EPI_ISL_708920, EPI_ISL_708951, EPI_ISL_708979, EPI_ISL_708983 to 708984, EPI_ISL_708986, EPI_ISL_709569 to 709570, EPI_ISL_709572 to 709573, EPI_ISL_709575 to 709579, EPI_ISL_709582 to 709585, EPI_ISL_709588 to 709593, EPI_ISL_709596 to 709597, EPI_ISL_709605, EPI_ISL_709608 to 709609, EPI_ISL_709614 to 709615, EPI_ISL_709618 to 709619, EPI_ISL_709623, EPI_ISL_709625, EPI_ISL_709629, EPI_ISL_709631 to 709632, EPI_ISL_709637, EPI_ISL_709640, EPI_ISL_709644, EPI_ISL_709646 to 709648, EPI_ISL_709650, EPI_ISL_709652, EPI_ISL_709654 to 709656, EPI_ISL_709658 to 709659, EPI_ISL_709662, EPI_ISL_709666, EPI_ISL_709669, EPI_ISL_709673 to 709676, EPI_ISL_709678 to 709680, EPI_ISL_709684, EPI_ISL_709687, EPI_ISL_709689, EPI_ISL_709692, EPI_ISL_709696 to 709697, EPI_ISL_709700 to 709701, EPI_ISL_709703 to 709705, EPI_ISL_709710 to 709711, EPI_ISL_709713, EPI_ISL_709716, EPI_ISL_709719, EPI_ISL_709721, EPI_ISL_709723, EPI_ISL_709725, EPI_ISL_709727, EPI_ISL_709731, EPI_ISL_709733 to 709735, EPI_ISL_709738, EPI_ISL_709740 to 709743, EPI_ISL_709747, EPI_ISL_709749 to 709751, EPI_ISL_709754, EPI_ISL_709757, EPI_ISL_709760 to 709762, EPI_ISL_709764, EPI_ISL_709766, EPI_ISL_709768 to 709772, EPI_ISL_709774, EPI_ISL_709777, EPI_ISL_709780 to 709781, EPI_ISL_709784 to 709786, EPI_ISL_709788 to 709790, EPI_ISL_709792, EPI_ISL_709794 to 709795, EPI_ISL_709798, EPI_ISL_709802, EPI_ISL_709815, EPI_ISL_709819, EPI_ISL_709823 to 709824, EPI_ISL_709828, EPI_ISL_709830, EPI_ISL_709833, EPI_ISL_709838 to 709839, EPI_ISL_709841, EPI_ISL_709852 to 709853, EPI_ISL_709856, EPI_ISL_709860, EPI_ISL_709863, EPI_ISL_709866, EPI_ISL_709868, EPI_ISL_709871 to 709872, EPI_ISL_709879, EPI_ISL_709881 to 709888, EPI_ISL_709890 to 709891, EPI_ISL_709893, EPI_ISL_709895 to 709896, EPI_ISL_709898, EPI_ISL_709900 to 709913, EPI_ISL_709920 to 709925, EPI_ISL_709927 to 709928, EPI_ISL_709931 to 709933, EPI_ISL_709935 to 709936, EPI_ISL_709938 to 709945, EPI_ISL_710907, EPI_ISL_719991, EPI_ISL_719995 to 719998, EPI_ISL_720002, EPI_ISL_720006, EPI_ISL_720008 to 720009, EPI_ISL_720011, EPI_ISL_720013, EPI_ISL_720017, EPI_ISL_720019 to 720021, EPI_ISL_720023 to 720025, EPI_ISL_720030, EPI_ISL_720033 to 720034, EPI_ISL_720036, EPI_ISL_720038 to 720039, EPI_ISL_720042 to 720044, EPI_ISL_720046 to 720050, EPI_ISL_720053 to 720054, EPI_ISL_720056, EPI_ISL_720058, EPI_ISL_720060, EPI_ISL_720063 to 720064, EPI_ISL_720067, EPI_ISL_720069 to 720070, EPI_ISL_720072 to 720075, EPI_ISL_720077 to 720079, EPI_ISL_720084, EPI_ISL_720086 to 720087, EPI_ISL_720089 to 720091, EPI_ISL_720093 to 720094, EPI_ISL_720098 to 720099, EPI_ISL_720102 to 720105, EPI_ISL_720113 to 720128, EPI_ISL_720130 to 720132, EPI_ISL_720134 to 720138, EPI_ISL_720140 to 720141, EPI_ISL_720144 to 720145, EPI_ISL_720148 to 720150, EPI_ISL_720152 to 720155, EPI_ISL_720157 to 720159, EPI_ISL_720161 to 720162, EPI_ISL_720164 to 720169, EPI_ISL_720171, EPI_ISL_720174 to 720175, EPI_ISL_720177, EPI_ISL_720181, EPI_ISL_720184 to 720186, EPI_ISL_720188 to 720189, EPI_ISL_720191 to 720192,

EPI_ISL_720194 to 720195, EPI_ISL_720197 to 720198, EPI_ISL_720200 to 720202, EPI_ISL_720204 to 720208, EPI_ISL_720210 to 720211, EPI_ISL_720214 to 720215, EPI_ISL_720217 to 720219, EPI_ISL_720221 to 720223, EPI_ISL_720230, EPI_ISL_720233 to 720236, EPI_ISL_720238 to 720244, EPI_ISL_720247 to 720248, EPI_ISL_720251, EPI_ISL_720253 to 720256, EPI_ISL_720258, EPI_ISL_720262 to 720264, EPI_ISL_720266, EPI_ISL_720274 to 720279, EPI_ISL_720285 to 720286, EPI_ISL_720289, EPI_ISL_720295 to 720298, EPI_ISL_720300, EPI_ISL_720305 to 720306, EPI_ISL_720308 to 720309, EPI_ISL_720314 to 720317, EPI_ISL_720320 to 720325, EPI_ISL_720327, EPI_ISL_720330, EPI_ISL_720332, EPI_ISL_720334, EPI_ISL_720336 to 720344, EPI_ISL_720347 to 720350, EPI_ISL_720352 to 720353, EPI_ISL_720355 to 720363, EPI_ISL_720365 to 720366, EPI_ISL_720368, EPI_ISL_720371 to 720381, EPI_ISL_720383 to 720384, EPI_ISL_720386 to 720387, EPI_ISL_720389 to 720390, EPI_ISL_720392 to 720393, EPI_ISL_720395 to 720401, EPI_ISL_720404 to 720407, EPI_ISL_720409 to 720413, EPI_ISL_720418, EPI_ISL_720421, EPI_ISL_720423 to 720436, EPI_ISL_720438, EPI_ISL_720440 to 720444, EPI_ISL_720447 to 720449, EPI_ISL_720451, EPI_ISL_720454 to 720460, EPI_ISL_720463 to 720465, EPI_ISL_720468, EPI_ISL_720470 to 720477, EPI_ISL_720479, EPI_ISL_720481 to 720487, EPI_ISL_720489 to 720493, EPI_ISL_720495 to 720496, EPI_ISL_720498 to 720501, EPI_ISL_720504, EPI_ISL_720506 to 720512, EPI_ISL_720514 to 720515, EPI_ISL_720517 to 720525, EPI_ISL_720527 to 720528, EPI_ISL_720530 to 720538, EPI_ISL_720540, EPI_ISL_720542, EPI_ISL_720545 to 720550, EPI_ISL_720552 to 720558, EPI_ISL_720560 to 720562, EPI_ISL_720564 to 720565, EPI_ISL_720567 to 720575, EPI_ISL_720577 to 720579, EPI_ISL_720581 to 720587, EPI_ISL_720589 to 720596, EPI_ISL_720598 to 720600, EPI_ISL_720603 to 720607, EPI_ISL_720609 to 720611, EPI_ISL_720615 to 720616, EPI_ISL_720618 to 720628, EPI_ISL_720630, EPI_ISL_720632, EPI_ISL_720635 to 720637, EPI_ISL_720639 to 720640, EPI_ISL_720642 to 720646, EPI_ISL_720648, EPI_ISL_720650 to 720651, EPI_ISL_720653 to 720658, EPI_ISL_720660 to 720663, EPI_ISL_720665 to 720667, EPI_ISL_720671, EPI_ISL_720673, EPI_ISL_720679, EPI_ISL_720681, EPI_ISL_720687, EPI_ISL_720691, EPI_ISL_720696, EPI_ISL_720698, EPI_ISL_720701, EPI_ISL_720706 to 720707, EPI_ISL_720710, EPI_ISL_720712, EPI_ISL_720714, EPI_ISL_720717, EPI_ISL_720719, EPI_ISL_720723 to 720725, EPI_ISL_720730, EPI_ISL_720736, EPI_ISL_720742, EPI_ISL_720746 to 720748, EPI_ISL_720751, EPI_ISL_720753, EPI_ISL_720760, EPI_ISL_720768 to 720769, EPI_ISL_720771 to 720772, EPI_ISL_720774 to 720775, EPI_ISL_720778, EPI_ISL_720781 to 720785, EPI_ISL_720789, EPI_ISL_720791, EPI_ISL_720794 to 720795, EPI_ISL_720797, EPI_ISL_720799, EPI_ISL_720804, EPI_ISL_720808 to 720809, EPI_ISL_720812, EPI_ISL_720817, EPI_ISL_720820, EPI_ISL_720822, EPI_ISL_720824, EPI_ISL_720829 to 720830, EPI_ISL_720832, EPI_ISL_720835, EPI_ISL_720838 to 720840, EPI_ISL_720843, EPI_ISL_720854 to 720855, EPI_ISL_720859 to 720862, EPI_ISL_720865, EPI_ISL_720868, EPI_ISL_720871, EPI_ISL_720874, EPI_ISL_720876, EPI_ISL_720878 to 720879, EPI_ISL_720882, EPI_ISL_720884, EPI_ISL_720890, EPI_ISL_720892, EPI_ISL_720895 to 720896, EPI_ISL_720899, EPI_ISL_720902, EPI_ISL_720906, EPI_ISL_720908, EPI_ISL_721526, EPI_ISL_721529, EPI_ISL_731251, EPI_ISL_731892, EPI_ISL_733607, EPI_ISL_733617, EPI_ISL_733628, EPI_ISL_733630, EPI_ISL_733644, EPI_ISL_733648, EPI_ISL_733652, EPI_ISL_733657, EPI_ISL_733664, EPI_ISL_733666, EPI_ISL_733669, EPI_ISL_733687, EPI_ISL_733711, EPI_ISL_733735, EPI_ISL_733739, EPI_ISL_733746, EPI_ISL_733749, EPI_ISL_733757, EPI_ISL_733760, EPI_ISL_733764, EPI_ISL_733768, EPI_ISL_733771, EPI_ISL_733796, EPI_ISL_733798, EPI_ISL_733801, EPI_ISL_733811, EPI_ISL_733813, EPI_ISL_733820, EPI_ISL_733825, EPI_ISL_733828 to 733829, EPI_ISL_733832, EPI_ISL_733838, EPI_ISL_733851, EPI_ISL_733855, EPI_ISL_733861, EPI_ISL_733872, EPI_ISL_733889, EPI_ISL_733900, EPI_ISL_733902 to 733904, EPI_ISL_733914, EPI_ISL_735512, EPI_ISL_735515 to 735516, EPI_ISL_735520 to 735521, EPI_ISL_735528, EPI_ISL_735534 to 735535, EPI_ISL_735537, EPI_ISL_735542 to 735543, EPI_ISL_735545, EPI_ISL_735548 to 735549, EPI_ISL_735551, EPI_ISL_735554 to 735555, EPI_ISL_735560, EPI_ISL_735563, EPI_ISL_735565, EPI_ISL_735567, EPI_ISL_735572, EPI_ISL_735574, EPI_ISL_735576 to 735577, EPI_ISL_735582 to 735583, EPI_ISL_735589 to 735592, EPI_ISL_735598 to 735599, EPI_ISL_735602, EPI_ISL_735609, EPI_ISL_735615, EPI_ISL_735620, EPI_ISL_735622, EPI_ISL_735626, EPI_ISL_735630, EPI_ISL_735633 to 735634, EPI_ISL_735640, EPI_ISL_735642, EPI_ISL_735644 to 735646, EPI_ISL_735649, EPI_ISL_735653, EPI_ISL_735656 to 735657, EPI_ISL_735675 to 735676, EPI_ISL_735688, EPI_ISL_735693, EPI_ISL_735696, EPI_ISL_735701, EPI_ISL_735711, EPI_ISL_735713, EPI_ISL_735715, EPI_ISL_735718, EPI_ISL_735721 to 735722, EPI_ISL_735725 to 735726, EPI_ISL_735729, EPI_ISL_735732, EPI_ISL_735736 to 735737, EPI_ISL_735741 to 735743, EPI_ISL_735745, EPI_ISL_735753 to 735754, EPI_ISL_735756, EPI_ISL_735758, EPI_ISL_735764, EPI_ISL_735769, EPI_ISL_735777, EPI_ISL_735781, EPI_ISL_735790 to 735791, EPI_ISL_735793, EPI_ISL_735799, EPI_ISL_735802, EPI_ISL_735804, EPI_ISL_735806 to 735807, EPI_ISL_735812, EPI_ISL_735814, EPI_ISL_735817, EPI_ISL_735833, EPI_ISL_735836 to 735838, EPI_ISL_735844, EPI_ISL_735846 to 735847, EPI_ISL_735855, EPI_ISL_735858, EPI_ISL_736219, EPI_ISL_736230, EPI_ISL_736232, EPI_ISL_736249, EPI_ISL_736251, EPI_ISL_736262, EPI_ISL_736265, EPI_ISL_736267, EPI_ISL_736279, EPI_ISL_736286, EPI_ISL_736299, EPI_ISL_736308, EPI_ISL_736314, EPI_ISL_736317, EPI_ISL_736329, EPI_ISL_736333, EPI_ISL_736342, EPI_ISL_736374, EPI_ISL_736385, EPI_ISL_736387, EPI_ISL_736403, EPI_ISL_736410, EPI_ISL_736420 to 736421, EPI_ISL_736433, EPI_ISL_736435 to 736436, EPI_ISL_736449, EPI_ISL_736452, EPI_ISL_736463, EPI_ISL_736467, EPI_ISL_736497, EPI_ISL_736502, EPI_ISL_736504, EPI_ISL_736523, EPI_ISL_736527, EPI_ISL_736542, EPI_ISL_736550 to 736552, EPI_ISL_736565, EPI_ISL_736590, EPI_ISL_736597, EPI_ISL_736610, EPI_ISL_736617, EPI_ISL_736637, EPI_ISL_736675, EPI_ISL_736682, EPI_ISL_736693, EPI_ISL_736703, EPI_ISL_736713, EPI_ISL_736716, EPI_ISL_736744, EPI_ISL_756377, EPI_ISL_756691 to 756694, EPI_ISL_756698 to 756701, EPI_ISL_756703 to 756704, EPI_ISL_756707 to 756708, EPI_ISL_756710, EPI_ISL_756712 to 756715, EPI_ISL_756718 to 756719, EPI_ISL_756721 to 756722, EPI_ISL_756724 to 756728, EPI_ISL_756730 to 756735, EPI_ISL_756738 to 756743, EPI_ISL_756746 to 756747, EPI_ISL_756749, EPI_ISL_756751 to 756760, EPI_ISL_756762 to 756771, EPI_ISL_756774, EPI_ISL_756777 to 756778, EPI_ISL_756781 to 756783, EPI_ISL_756785 to 756788, EPI_ISL_756790 to 756792, EPI_ISL_756794, EPI_ISL_756796 to 756799, EPI_ISL_756801 to 756802, EPI_ISL_756804, EPI_ISL_756806 to 756819, EPI_ISL_756822, EPI_ISL_756825 to 756834, EPI_ISL_756836 to 756839, EPI_ISL_756841 to 756848, EPI_ISL_756851, EPI_ISL_756853, EPI_ISL_756855 to 756858, EPI_ISL_756860, EPI_ISL_756862 to 756866, EPI_ISL_756868 to 756876, EPI_ISL_756880, EPI_ISL_756882 to 756884, EPI_ISL_756890 to 756892, EPI_ISL_756896, EPI_ISL_756898 to 756899, EPI_ISL_756901 to 756905, EPI_ISL_756908, EPI_ISL_756911, EPI_ISL_756913 to 756917, EPI_ISL_756919, EPI_ISL_756921 to 756922, EPI_ISL_756924 to 756927, EPI_ISL_756929 to 756930, EPI_ISL_756932, EPI_ISL_756934, EPI_ISL_756936 to 756938, EPI_ISL_756941 to 756942, EPI_ISL_756944 to 756946, EPI_ISL_756948 to 756950, EPI_ISL_756956 to 756958, EPI_ISL_756961, EPI_ISL_756963 to 756968, EPI_ISL_756972, EPI_ISL_756975 to 756976, EPI_ISL_756978 to 756981, EPI_ISL_756983 to 756988, EPI_ISL_756990 to 756994, EPI_ISL_756996 to 756997, EPI_ISL_757000 to 757004, EPI_ISL_757006, EPI_ISL_757010, EPI_ISL_757013 to 757016, EPI_ISL_757019, EPI_ISL_757023 to 757026, EPI_ISL_757029 to 757030, EPI_ISL_760698, EPI_ISL_760801, EPI_ISL_760809 to 760810, EPI_ISL_760842, EPI_ISL_760844, EPI_ISL_760863, EPI_ISL_760873, EPI_ISL_760896, EPI_ISL_760905, EPI_ISL_760943, EPI_ISL_760954, EPI_ISL_760959, EPI_ISL_760961, EPI_ISL_760963, EPI_ISL_760983, EPI_ISL_760987, EPI_ISL_761002, EPI_ISL_761023, EPI_ISL_761039, EPI_ISL_761050, EPI_ISL_761079, EPI_ISL_761357 to 761366, EPI_ISL_761368 to 761371, EPI_ISL_761373 to 761380, EPI_ISL_761382 to 761389, EPI_ISL_761391 to 761397, EPI_ISL_761399 to 761425, EPI_ISL_761427 to 761447, EPI_ISL_761449 to 761458, EPI_ISL_761460 to 761471, EPI_ISL_761473 to 761480, EPI_ISL_761482 to 761484, EPI_ISL_761486 to 761489, EPI_ISL_761492 to 761504, EPI_ISL_761506 to 761515, EPI_ISL_761517 to 761535, EPI_ISL_761537 to 761538, EPI_ISL_761540 to 761551, EPI_ISL_761553 to 761556, EPI_ISL_761558 to 761600, EPI_ISL_761602 to 761603, EPI_ISL_761605, EPI_ISL_761607 to 761627, EPI_ISL_761629 to 761632, EPI_ISL_761634 to 761640, EPI_ISL_761642 to 761647, EPI_ISL_761649 to 761652, EPI_ISL_761655, EPI_ISL_761657 to 761664, EPI_ISL_762199 to 762219, EPI_ISL_762221 to 762228, EPI_ISL_762230 to 762239, EPI_ISL_762241 to 762279, EPI_ISL_762281 to 762284, EPI_ISL_762286 to 762301, EPI_ISL_762303 to 762307, EPI_ISL_762309, EPI_ISL_762311 to 762343, EPI_ISL_762345 to 762361, EPI_ISL_762365 to 762367, EPI_ISL_762369 to 762380, EPI_ISL_762382 to 762386, EPI_ISL_762388 to 762419, EPI_ISL_762421 to 762424, EPI_ISL_762427, EPI_ISL_762429 to 762431, EPI_ISL_762433 to 762445, EPI_ISL_762447 to 762448, EPI_ISL_762450 to 762457, EPI_ISL_762459 to 762465, EPI_ISL_762467 to 762496, EPI_ISL_762498, EPI_ISL_762501 to 762505, EPI_ISL_762507 to 762518, EPI_ISL_762520 to 762545, EPI_ISL_762864, EPI_ISL_762867, EPI_ISL_762880, EPI_ISL_762884, EPI_ISL_762891, EPI_ISL_762896, EPI_ISL_762903, EPI_ISL_762908, EPI_ISL_762912, EPI_ISL_762914, EPI_ISL_762937, EPI_ISL_762945, EPI_ISL_762967 to 762968, EPI_ISL_762976, EPI_ISL_762978, EPI_ISL_762981, EPI_ISL_767090, EPI_ISL_767098 to 767101, EPI_ISL_767107 to 767109, EPI_ISL_767111 to 767112, EPI_ISL_767134, EPI_ISL_767239, EPI_ISL_768846 to 768850, EPI_ISL_768852 to 768856, EPI_ISL_768858, EPI_ISL_768860 to 768867, EPI_ISL_768869 to 768884, EPI_ISL_768886 to 768897, EPI_ISL_769226 to 769228, EPI_ISL_769230 to 769232, EPI_ISL_769235, EPI_ISL_769237 to 769239, EPI_ISL_769242, EPI_ISL_769247 to 769248, EPI_ISL_769251, EPI_ISL_769253, EPI_ISL_769256, EPI_ISL_769258, EPI_ISL_769264 to 769265, EPI_ISL_769268, EPI_ISL_769272, EPI_ISL_769277, EPI_ISL_769279, EPI_ISL_769283, EPI_ISL_769285, EPI_ISL_769287, EPI_ISL_769293, EPI_ISL_769295 to 769296, EPI_ISL_769299 to 769305, EPI_ISL_769307, EPI_ISL_769309, EPI_ISL_769311, EPI_ISL_769313, EPI_ISL_769315, EPI_ISL_769317 to 769318, EPI_ISL_769324, EPI_ISL_769326, EPI_ISL_769331 to 769332, EPI_ISL_769336 to 769340, EPI_ISL_769342, EPI_ISL_769344, EPI_ISL_769346 to 769347, EPI_ISL_769350 to 769351, EPI_ISL_769354, EPI_ISL_769356 to 769359, EPI_ISL_769362 to 769364, EPI_ISL_769366, EPI_ISL_769368 to 769369, EPI_ISL_769371 to 769373, EPI_ISL_769375 to 769379, EPI_ISL_769382, EPI_ISL_769393, EPI_ISL_769395, EPI_ISL_769397 to 769398, EPI_ISL_769400, EPI_ISL_769403, EPI_ISL_769407, EPI_ISL_769409 to 769411, EPI_ISL_769415, EPI_ISL_769423 to 769424, EPI_ISL_769426, EPI_ISL_769429, EPI_ISL_769432, EPI_ISL_769436 to 769439, EPI_ISL_769443 to 769444, EPI_ISL_769447 to 769448, EPI_ISL_769451, EPI_ISL_769453, EPI_ISL_769455 to 769456, EPI_ISL_769461, EPI_ISL_769463 to 769464, EPI_ISL_769467, EPI_ISL_769472, EPI_ISL_769477 to 769480, EPI_ISL_769483 to 769484, EPI_ISL_769486, EPI_ISL_769490 to 769493, EPI_ISL_769495, EPI_ISL_769498, EPI_ISL_769507 to 769510, EPI_ISL_769514, EPI_ISL_769516, EPI_ISL_769520 to 769521, EPI_ISL_769523, EPI_ISL_769527, EPI_ISL_769529, EPI_ISL_769531, EPI_ISL_769534, EPI_ISL_769536 to 769538, EPI_ISL_769544 to 769550, EPI_ISL_769552, EPI_ISL_769554 to 769555, EPI_ISL_769557 to 769559, EPI_ISL_769561 to 769566, EPI_ISL_769569 to 769576, EPI_ISL_769578 to 769580, EPI_ISL_769582, EPI_ISL_769584 to 769588, EPI_ISL_769590 to 769593, EPI_ISL_769595 to 769605, EPI_ISL_769608, EPI_ISL_769610, EPI_ISL_769615 to 769616, EPI_ISL_769618, EPI_ISL_769620 to 769624, EPI_ISL_769626 to 769640, EPI_ISL_769642 to 769643, EPI_ISL_769645 to 769647, EPI_ISL_769649 to 769651, EPI_ISL_769653 to 769654, EPI_ISL_769656 to 769660, EPI_ISL_769663 to 769665, EPI_ISL_769668 to 769676, EPI_ISL_769678 to 769686, EPI_ISL_769688, EPI_ISL_769690, EPI_ISL_769692 to 769694, EPI_ISL_769697 to 769702, EPI_ISL_769704, EPI_ISL_769706 to 769710, EPI_ISL_769712, EPI_ISL_769715, EPI_ISL_769717 to 769719, EPI_ISL_769721 to 769723, EPI_ISL_769725 to 769734, EPI_ISL_769736 to 769740, EPI_ISL_769742, EPI_ISL_769744 to 769750, EPI_ISL_769754 to 769755, EPI_ISL_769757, EPI_ISL_769760, EPI_ISL_769762 to 769763, EPI_ISL_769765 to 769767, EPI_ISL_769770 to 769772, EPI_ISL_769774 to 769786, EPI_ISL_769789 to 769790, EPI_ISL_769792 to 769793, EPI_ISL_769795 to 769801, EPI_ISL_769803 to 769805, EPI_ISL_769807 to 769812, EPI_ISL_769814, EPI_ISL_769816 to 769817, EPI_ISL_769819 to 769825, EPI_ISL_769827 to 769830, EPI_ISL_769832 to 769833, EPI_ISL_769835, EPI_ISL_769838 to 769839, EPI_ISL_769841, EPI_ISL_769843 to 769849, EPI_ISL_769851 to 769860, EPI_ISL_777064, EPI_ISL_777084, EPI_ISL_777102, EPI_ISL_777104, EPI_ISL_777245, EPI_ISL_778298, EPI_ISL_778300, EPI_ISL_778307, EPI_ISL_778310, EPI_ISL_778313 to 778314, EPI_ISL_778318, EPI_ISL_778320 to 778321, EPI_ISL_778326, EPI_ISL_778329, EPI_ISL_778338 to 778339, EPI_ISL_778341, EPI_ISL_778344, EPI_ISL_778351, EPI_ISL_778363, EPI_ISL_778366 to 778367, EPI_ISL_778379, EPI_ISL_778381, EPI_ISL_778387 to 778389, EPI_ISL_778391, EPI_ISL_778394 to 778395, EPI_ISL_778402, EPI_ISL_778405, EPI_ISL_778409, EPI_ISL_778413, EPI_ISL_778431, EPI_ISL_778434 to 778436, EPI_ISL_778439 to 778443, EPI_ISL_778446, EPI_ISL_778449, EPI_ISL_778456, EPI_ISL_778460, EPI_ISL_778464, EPI_ISL_778466 to 778470, EPI_ISL_778475, EPI_ISL_778477, EPI_ISL_778480 to 778481, EPI_ISL_778483, EPI_ISL_778485 to 778486, EPI_ISL_778489, EPI_ISL_778492, EPI_ISL_778495, EPI_ISL_778501 to 778503, EPI_ISL_778505, EPI_ISL_778508 to 778509, EPI_ISL_778511, EPI_ISL_778513 to 778515, EPI_ISL_778518, EPI_ISL_778520 to 778521, EPI_ISL_778526, EPI_ISL_778532, EPI_ISL_778542, EPI_ISL_778544 to 778545, EPI_ISL_778549, EPI_ISL_778552, EPI_ISL_778554 to 778555, EPI_ISL_778557, EPI_ISL_778559, EPI_ISL_778561, EPI_ISL_778563, EPI_ISL_778566 to 778567, EPI_ISL_778573 to 778575, EPI_ISL_778581 to 778582, EPI_ISL_778585, EPI_ISL_778587, EPI_ISL_778589, EPI_ISL_778591, EPI_ISL_778596 to 778597, EPI_ISL_778604, EPI_ISL_778606, EPI_ISL_778609 to 778610, EPI_ISL_778613, EPI_ISL_778622, EPI_ISL_778624 to 778626, EPI_ISL_778632 to 778634, EPI_ISL_778639 to 778640, EPI_ISL_778642, EPI_ISL_781178 to 781180, EPI_ISL_781182, EPI_ISL_781184, EPI_ISL_781193, EPI_ISL_781196 to 781198, EPI_ISL_781208, EPI_ISL_781211 to 781213, EPI_ISL_781215, EPI_ISL_781217 to 781218, EPI_ISL_781222, EPI_ISL_781227 to 781229, EPI_ISL_781231 to 781232, EPI_ISL_781236, EPI_ISL_781239 to 781240, EPI_ISL_781243, EPI_ISL_781246 to 781247, EPI_ISL_781252, EPI_ISL_781254 to 781255, EPI_ISL_781257, EPI_ISL_781265 to 781268, EPI_ISL_781270 to 781272, EPI_ISL_781274, EPI_ISL_781277, EPI_ISL_781284, EPI_ISL_781286, EPI_ISL_781290, EPI_ISL_781293, EPI_ISL_781296, EPI_ISL_781300, EPI_ISL_781302 to 781303, EPI_ISL_781305, EPI_ISL_781309, EPI_ISL_781311 to 781312, EPI_ISL_781314, EPI_ISL_781316 to 781317, EPI_ISL_781320, EPI_ISL_781322, EPI_ISL_781326, EPI_ISL_781329, EPI_ISL_781335, EPI_ISL_781338 to 781339, EPI_ISL_781343, EPI_ISL_781346 to 781347, EPI_ISL_781350 to 781351, EPI_ISL_781353, EPI_ISL_781357, EPI_ISL_781361, EPI_ISL_781369, EPI_ISL_781373, EPI_ISL_781380 to 781384, EPI_ISL_781386, EPI_ISL_781388, EPI_ISL_781390 to 781391, EPI_ISL_781393 to 781394, EPI_ISL_781399, EPI_ISL_781404, EPI_ISL_781410, EPI_ISL_781414 to 781422, EPI_ISL_781425, EPI_ISL_781428 to 781430, EPI_ISL_781449 to 781450, EPI_ISL_781453 to 781454, EPI_ISL_781460 to 781462, EPI_ISL_781467, EPI_ISL_781470, EPI_ISL_781476 to 781477, EPI_ISL_781479, EPI_ISL_781482, EPI_ISL_781484 to 781486, EPI_ISL_781491, EPI_ISL_781496, EPI_ISL_781501, EPI_ISL_781507, EPI_ISL_781509, EPI_ISL_781511, EPI_ISL_781522, EPI_ISL_781527, EPI_ISL_781529, EPI_ISL_781536 to 781537, EPI_ISL_781541, EPI_ISL_781544, EPI_ISL_781547 to 781548, EPI_ISL_781554 to 781556, EPI_ISL_781567, EPI_ISL_781578 to 781579, EPI_ISL_781597 to 781599, EPI_ISL_781605, EPI_ISL_781609, EPI_ISL_781611, EPI_ISL_781615, EPI_ISL_781619, EPI_ISL_781621, EPI_ISL_781623 to 781624, EPI_ISL_781627, EPI_ISL_781636, EPI_ISL_781651 to 781652, EPI_ISL_781663, EPI_ISL_781665, EPI_ISL_781669, EPI_ISL_781674, EPI_ISL_781677, EPI_ISL_781681, EPI_ISL_781684, EPI_ISL_781695, EPI_ISL_781699, EPI_ISL_781701, EPI_ISL_781724 to 781725, EPI_ISL_781730, EPI_ISL_781732, EPI_ISL_781735, EPI_ISL_781742 to 781743, EPI_ISL_781746 to 781747, EPI_ISL_781763, EPI_ISL_781767, EPI_ISL_781773, EPI_ISL_781777, EPI_ISL_781783 to 781785, EPI_ISL_781807, EPI_ISL_781832, EPI_ISL_781834, EPI_ISL_781837, EPI_ISL_781839 to 781841, EPI_ISL_781851, EPI_ISL_781858, EPI_ISL_781862, EPI_ISL_781876, EPI_ISL_781885, EPI_ISL_781897 to 781898, EPI_ISL_781903, EPI_ISL_781907, EPI_ISL_781909, EPI_ISL_781914, EPI_ISL_781920 to 781921, EPI_ISL_781925, EPI_ISL_781938, EPI_ISL_781940 to 781942, EPI_ISL_781944, EPI_ISL_781946, EPI_ISL_781948, EPI_ISL_781952, EPI_ISL_781955, EPI_ISL_781968, EPI_ISL_781973, EPI_ISL_781977, EPI_ISL_781979, EPI_ISL_781984, EPI_ISL_781987, EPI_ISL_781994, EPI_ISL_781998 to 782000, EPI_ISL_782009, EPI_ISL_782015, EPI_ISL_782021, EPI_ISL_782028, EPI_ISL_782030 to 782032, EPI_ISL_782035, EPI_ISL_782037, EPI_ISL_782040, EPI_ISL_782042, EPI_ISL_782044, EPI_ISL_782046, EPI_ISL_782049, EPI_ISL_782051 to 782052, EPI_ISL_782054 to 782055, EPI_ISL_782058, EPI_ISL_782061, EPI_ISL_782073, EPI_ISL_782077, EPI_ISL_782080 to 782081, EPI_ISL_782087 to 782088, EPI_ISL_782092, EPI_ISL_782094, EPI_ISL_782098, EPI_ISL_782103, EPI_ISL_782105 to 782106, EPI_ISL_782113, EPI_ISL_782125, EPI_ISL_782129 to 782131, EPI_ISL_782134, EPI_ISL_782136, EPI_ISL_782143, EPI_ISL_782736, EPI_ISL_782740, EPI_ISL_782742 to 782743, EPI_ISL_782751, EPI_ISL_782767, EPI_ISL_782770, EPI_ISL_782772, EPI_ISL_782778 to 782779, EPI_ISL_782789 to 782790, EPI_ISL_782798, EPI_ISL_782811, EPI_ISL_782819, EPI_ISL_782821, EPI_ISL_782823, EPI_ISL_782828, EPI_ISL_782831, EPI_ISL_782858 to 782859, EPI_ISL_782861, EPI_ISL_782865, EPI_ISL_782874 to 782876, EPI_ISL_782879, EPI_ISL_782891, EPI_ISL_782895 to 782896, EPI_ISL_782915 to 782916, EPI_ISL_782922, EPI_ISL_782934, EPI_ISL_782943 to 782944, EPI_ISL_782949, EPI_ISL_782953, EPI_ISL_782955, EPI_ISL_782957, EPI_ISL_782961, EPI_ISL_782965, EPI_ISL_782972, EPI_ISL_782980 to 782981, EPI_ISL_782986, EPI_ISL_782992, EPI_ISL_783001, EPI_ISL_783003 to 783005, EPI_ISL_783011, EPI_ISL_783017, EPI_ISL_783029, EPI_ISL_783035, EPI_ISL_783048, EPI_ISL_783050, EPI_ISL_783053, EPI_ISL_783061, EPI_ISL_783072, EPI_ISL_783077, EPI_ISL_783093, EPI_ISL_783103, EPI_ISL_783108, EPI_ISL_783121 to 783122, EPI_ISL_783131, EPI_ISL_783135, EPI_ISL_783142, EPI_ISL_783152, EPI_ISL_783156, EPI_ISL_783162, EPI_ISL_783171, EPI_ISL_783175, EPI_ISL_783179, EPI_ISL_783186, EPI_ISL_783189, EPI_ISL_783191, EPI_ISL_783212, EPI_ISL_783232, EPI_ISL_783246, EPI_ISL_783253, EPI_ISL_783256, EPI_ISL_783261, EPI_ISL_783287, EPI_ISL_783289, EPI_ISL_783301, EPI_ISL_783314, EPI_ISL_783317, EPI_ISL_783332 to 783333, EPI_ISL_783354, EPI_ISL_783356, EPI_ISL_783361, EPI_ISL_783363, EPI_ISL_783366, EPI_ISL_797118, EPI_ISL_797120 to 797128, EPI_ISL_797130 to 797134, EPI_ISL_797139, EPI_ISL_797141 to 797142, EPI_ISL_797145, EPI_ISL_797147 to 797148, EPI_ISL_797150, EPI_ISL_797154 to 797155, EPI_ISL_797157 to 797158, EPI_ISL_797160 to 797163, EPI_ISL_797167 to 797175, EPI_ISL_797177, EPI_ISL_797180 to 797184, EPI_ISL_797186 to 797192, EPI_ISL_797196 to 797197, EPI_ISL_797199 to 797206, EPI_ISL_797209 to 797210, EPI_ISL_797212 to 797214, EPI_ISL_797218 to 797222, EPI_ISL_797225 to 797226, EPI_ISL_797228, EPI_ISL_797230 to 797232, EPI_ISL_797234 to 797236, EPI_ISL_797238 to 797240, EPI_ISL_797242 to 797251, EPI_ISL_797254 to 797263, EPI_ISL_797267 to 797280, EPI_ISL_797284 to 797285, EPI_ISL_797287 to 797298, EPI_ISL_797300, EPI_ISL_797303 to 797304, EPI_ISL_797306, EPI_ISL_797308, EPI_ISL_797310, EPI_ISL_797313 to 797318, EPI_ISL_797321 to 797322, EPI_ISL_797324, EPI_ISL_797326, EPI_ISL_797328 to 797331, EPI_ISL_797333 to 797336, EPI_ISL_797339 to 797343, EPI_ISL_797345, EPI_ISL_797348, EPI_ISL_797351 to 797352, EPI_ISL_797355 to 797359, EPI_ISL_797361, EPI_ISL_797363, EPI_ISL_797365, EPI_ISL_797368 to 797372, EPI_ISL_797376, EPI_ISL_797378, EPI_ISL_797380, EPI_ISL_797384 to 797386, EPI_ISL_797388 to 797393, EPI_ISL_797395 to 797396, EPI_ISL_798335, EPI_ISL_798338 to 798339, EPI_ISL_798342, EPI_ISL_798344, EPI_ISL_798347 to 798348, EPI_ISL_798351, EPI_ISL_798353, EPI_ISL_798355, EPI_ISL_798357 to 798359, EPI_ISL_798361, EPI_ISL_798363 to 798364, EPI_ISL_798366, EPI_ISL_798371, EPI_ISL_798373 to 798377, EPI_ISL_798380 to 798383, EPI_ISL_798385, EPI_ISL_798390 to 798393, EPI_ISL_798395, EPI_ISL_798397 to 798398, EPI_ISL_798400, EPI_ISL_798402 to 798403, EPI_ISL_798406 to 798408, EPI_ISL_798410 to 798411, EPI_ISL_798414 to 798415, EPI_ISL_798421 to 798422, EPI_ISL_798425 to 798427, EPI_ISL_798429, EPI_ISL_798432, EPI_ISL_798434 to 798435, EPI_ISL_798437, EPI_ISL_798440 to 798441, EPI_ISL_798443, EPI_ISL_798447 to 798449, EPI_ISL_798451 to 798454, EPI_ISL_798456, EPI_ISL_798458 to 798460, EPI_ISL_798463 to 798464, EPI_ISL_798466, EPI_ISL_798470, EPI_ISL_798473 to 798474, EPI_ISL_798476 to 798477, EPI_ISL_798480, EPI_ISL_798482, EPI_ISL_798484 to 798486, EPI_ISL_798488, EPI_ISL_798490, EPI_ISL_798494 to 798497, EPI_ISL_798499, EPI_ISL_798505 to 798506, EPI_ISL_798511 to 798512, EPI_ISL_798514 to 798515, EPI_ISL_798518, EPI_ISL_798522 to 798523, EPI_ISL_798525 to 798528, EPI_ISL_798530, EPI_ISL_798532, EPI_ISL_798536 to 798537, EPI_ISL_798540, EPI_ISL_798543, EPI_ISL_798547, EPI_ISL_798553, EPI_ISL_798556 to 798557, EPI_ISL_798562, EPI_ISL_798565 to 798572, EPI_ISL_798576 to 798578, EPI_ISL_798581, EPI_ISL_798583 to 798587, EPI_ISL_798592, EPI_ISL_798594, EPI_ISL_798597, EPI_ISL_798599, EPI_ISL_798605 to 798606, EPI_ISL_798608, EPI_ISL_798610 to 798611, EPI_ISL_798613, EPI_ISL_798617, EPI_ISL_798619 to 798620, EPI_ISL_798623, EPI_ISL_798625, EPI_ISL_798628 to 798630, EPI_ISL_798636, EPI_ISL_798638, EPI_ISL_798644 to 798647, EPI_ISL_798650 to 798651, EPI_ISL_798653 to 798654, EPI_ISL_798656 to 798657, EPI_ISL_798660, EPI_ISL_798662 to 798663, EPI_ISL_798666, EPI_ISL_798670, EPI_ISL_798672, EPI_ISL_798675, EPI_ISL_798691, EPI_ISL_798693, EPI_ISL_798696, EPI_ISL_798701 to 798702, EPI_ISL_798707, EPI_ISL_798712, EPI_ISL_798716, EPI_ISL_798720 to 798721, EPI_ISL_798724 to 798725, EPI_ISL_798738 to 798740, EPI_ISL_798755, EPI_ISL_798764, EPI_ISL_798767, EPI_ISL_798769 to 798770, EPI_ISL_798774, EPI_ISL_798777, EPI_ISL_798781, EPI_ISL_798801, EPI_ISL_798803, EPI_ISL_798808 to 798809, EPI_ISL_798821 to 798823, EPI_ISL_798830, EPI_ISL_798832, EPI_ISL_798837 to 798838, EPI_ISL_798851, EPI_ISL_798855 to 798856, EPI_ISL_798863 to 798864, EPI_ISL_798870, EPI_ISL_798874, EPI_ISL_798882, EPI_ISL_798889, EPI_ISL_798892, EPI_ISL_798910 to 798911, EPI_ISL_798919, EPI_ISL_798922, EPI_ISL_798925, EPI_ISL_798930, EPI_ISL_798933, EPI_ISL_798936, EPI_ISL_798942, EPI_ISL_798948, EPI_ISL_798955, EPI_ISL_798966 to 798967, EPI_ISL_798970, EPI_ISL_798973, EPI_ISL_798975, EPI_ISL_798977, EPI_ISL_798981, EPI_ISL_798983, EPI_ISL_798987, EPI_ISL_798994 to 798996, EPI_ISL_799296, EPI_ISL_799303, EPI_ISL_799305 to 799306, EPI_ISL_799308, EPI_ISL_799311, EPI_ISL_799314, EPI_ISL_799317, EPI_ISL_799319 to 799320, EPI_ISL_799323, EPI_ISL_799325, EPI_ISL_799327, EPI_ISL_799333, EPI_ISL_799346 to 799347, EPI_ISL_799350 to 799353, EPI_ISL_799356 to 799357, EPI_ISL_799362 to 799364, EPI_ISL_799366, EPI_ISL_799372 to 799373, EPI_ISL_799375 to 799376, EPI_ISL_799380, EPI_ISL_799384, EPI_ISL_799386 to 799388, EPI_ISL_799390, EPI_ISL_799392, EPI_ISL_799394 to 799395, EPI_ISL_799397, EPI_ISL_799400, EPI_ISL_799403 to 799404, EPI_ISL_799410 to 799413, EPI_ISL_799415, EPI_ISL_799420, EPI_ISL_799430, EPI_ISL_799432, EPI_ISL_799434, EPI_ISL_799436 to 799437, EPI_ISL_799439 to 799441, EPI_ISL_799443, EPI_ISL_799445, EPI_ISL_799447 to 799448, EPI_ISL_799454, EPI_ISL_799465 to 799470, EPI_ISL_799476, EPI_ISL_799479, EPI_ISL_799482, EPI_ISL_799485, EPI_ISL_799488, EPI_ISL_799490, EPI_ISL_799500, EPI_ISL_799502, EPI_ISL_799506, EPI_ISL_799509, EPI_ISL_799514 to 799515, EPI_ISL_799519, EPI_ISL_799522, EPI_ISL_799529, EPI_ISL_799534, EPI_ISL_799536, EPI_ISL_799538, EPI_ISL_799540, EPI_ISL_799545 to 799547, EPI_ISL_799550 to 799551, EPI_ISL_799555, EPI_ISL_799563, EPI_ISL_799567, EPI_ISL_799570, EPI_ISL_799572, EPI_ISL_799575, EPI_ISL_799577, EPI_ISL_799579 to 799580, EPI_ISL_799582, EPI_ISL_799588, EPI_ISL_799590, EPI_ISL_799595, EPI_ISL_799598, EPI_ISL_799600, EPI_ISL_799603 to 799604, EPI_ISL_799608, EPI_ISL_799614, EPI_ISL_799617 to 799618, EPI_ISL_799620 to 799621, EPI_ISL_799623, EPI_ISL_799625, EPI_ISL_799627 to 799628, EPI_ISL_799633,

EPI_ISL_799635, EPI_ISL_799642, EPI_ISL_799645 to 799647, EPI_ISL_799651, EPI_ISL_799653, EPI_ISL_799666 to 799668, EPI_ISL_799672, EPI_ISL_799675, EPI_ISL_799680, EPI_ISL_799685, EPI_ISL_799688, EPI_ISL_799690, EPI_ISL_799694 to 799698, EPI_ISL_799700 to 799701, EPI_ISL_799703 to 799705, EPI_ISL_799710, EPI_ISL_799712, EPI_ISL_799714, EPI_ISL_799717, EPI_ISL_799719 to 799721, EPI_ISL_799723, EPI_ISL_799728, EPI_ISL_799733, EPI_ISL_799736, EPI_ISL_799742, EPI_ISL_799745 to 799746, EPI_ISL_799753, EPI_ISL_799755, EPI_ISL_799764, EPI_ISL_799770 to 799772, EPI_ISL_799774, EPI_ISL_799778, EPI_ISL_799782, EPI_ISL_799785, EPI_ISL_799793 to 799794, EPI_ISL_799798, EPI_ISL_799800, EPI_ISL_799802, EPI_ISL_799806, EPI_ISL_799810, EPI_ISL_799813, EPI_ISL_799815 to 799817, EPI_ISL_799824, EPI_ISL_799826, EPI_ISL_799828 to 799829, EPI_ISL_799833 to 799836, EPI_ISL_799842, EPI_ISL_799848 to 799849, EPI_ISL_799852, EPI_ISL_799854, EPI_ISL_799858, EPI_ISL_799862 to 799863, EPI_ISL_799867, EPI_ISL_799870, EPI_ISL_799872 to 799874, EPI_ISL_799878, EPI_ISL_799885, EPI_ISL_799895 to 799896, EPI_ISL_799900, EPI_ISL_799904 to 799905, EPI_ISL_799913, EPI_ISL_799921, EPI_ISL_799926 to 799927, EPI_ISL_799931, EPI_ISL_800584, EPI_ISL_800657, EPI_ISL_800701, EPI_ISL_800778, EPI_ISL_800814, EPI_ISL_800834, EPI_ISL_800846, EPI_ISL_800863, EPI_ISL_800868, EPI_ISL_801205, EPI_ISL_801217, EPI_ISL_811205, EPI_ISL_811207, EPI_ISL_811211 to 811212, EPI_ISL_811214 to 811215, EPI_ISL_811217, EPI_ISL_811236, EPI_ISL_811241, EPI_ISL_811245, EPI_ISL_811247, EPI_ISL_811251, EPI_ISL_811255, EPI_ISL_811262, EPI_ISL_811269, EPI_ISL_811275, EPI_ISL_811280, EPI_ISL_811285, EPI_ISL_811293, EPI_ISL_811302, EPI_ISL_811307, EPI_ISL_811310 to 811312, EPI_ISL_811321, EPI_ISL_811329, EPI_ISL_811332, EPI_ISL_811334 to 811336, EPI_ISL_811339, EPI_ISL_811342, EPI_ISL_811344, EPI_ISL_811350, EPI_ISL_811353, EPI_ISL_811358 to 811359, EPI_ISL_811362, EPI_ISL_811367, EPI_ISL_811369, EPI_ISL_811373, EPI_ISL_811380 to 811383, EPI_ISL_811385, EPI_ISL_811394 to 811395, EPI_ISL_811397, EPI_ISL_811400, EPI_ISL_811404, EPI_ISL_811406, EPI_ISL_811410, EPI_ISL_811414, EPI_ISL_811420, EPI_ISL_811429 to 811430, EPI_ISL_811433, EPI_ISL_811436, EPI_ISL_811444, EPI_ISL_811455, EPI_ISL_811469, EPI_ISL_811472, EPI_ISL_811478, EPI_ISL_811482, EPI_ISL_811487, EPI_ISL_811498, EPI_ISL_811500, EPI_ISL_811502, EPI_ISL_811516, EPI_ISL_811519, EPI_ISL_811524 to 811525, EPI_ISL_811530, EPI_ISL_811538, EPI_ISL_811541, EPI_ISL_811545, EPI_ISL_811548, EPI_ISL_811559, EPI_ISL_811563, EPI_ISL_811565, EPI_ISL_811569, EPI_ISL_811573, EPI_ISL_811580, EPI_ISL_811584, EPI_ISL_811591, EPI_ISL_811594, EPI_ISL_811603, EPI_ISL_811606, EPI_ISL_811610, EPI_ISL_811617 to 811618, EPI_ISL_811620 to 811622, EPI_ISL_811625, EPI_ISL_811627, EPI_ISL_811634, EPI_ISL_811645, EPI_ISL_811650 to 811651, EPI_ISL_811657, EPI_ISL_811660 to 811661, EPI_ISL_811664, EPI_ISL_811666, EPI_ISL_811669, EPI_ISL_811672 to 811673, EPI_ISL_811677 to 811678, EPI_ISL_811682, EPI_ISL_811688, EPI_ISL_811698, EPI_ISL_811701 to 811702, EPI_ISL_811708, EPI_ISL_811720, EPI_ISL_811727, EPI_ISL_811736, EPI_ISL_811740 to 811741, EPI_ISL_811750, EPI_ISL_811753 to 811754, EPI_ISL_811756, EPI_ISL_811759, EPI_ISL_811762, EPI_ISL_811767, EPI_ISL_811776, EPI_ISL_811785, EPI_ISL_811787, EPI_ISL_811807 to 811808, EPI_ISL_811810 to 811813, EPI_ISL_811817 to 811818, EPI_ISL_811820 to 811824, EPI_ISL_811826 to 811833, EPI_ISL_811835 to 811840, EPI_ISL_811843 to 811844, EPI_ISL_811846 to 811848, EPI_ISL_811851 to 811854, EPI_ISL_811857, EPI_ISL_811861, EPI_ISL_811864, EPI_ISL_811866 to 811868, EPI_ISL_811871, EPI_ISL_811875 to 811884, EPI_ISL_811887, EPI_ISL_811890, EPI_ISL_811892, EPI_ISL_811897 to 811901, EPI_ISL_811903 to 811905, EPI_ISL_811908 to 811916, EPI_ISL_811918 to 811921, EPI_ISL_811923 to 811940, EPI_ISL_811945, EPI_ISL_811947 to 811948, EPI_ISL_811950 to 811956, EPI_ISL_811960 to 811976, EPI_ISL_811979, EPI_ISL_811982 to 811983, EPI_ISL_811985 to 811989, EPI_ISL_811992 to 811997, EPI_ISL_811999 to 812001, EPI_ISL_812003 to 812012, EPI_ISL_812014 to 812016, EPI_ISL_812019 to 812025, EPI_ISL_812027, EPI_ISL_812029 to 812032, EPI_ISL_812034 to 812036, EPI_ISL_812039 to 812045, EPI_ISL_812047 to 812054, EPI_ISL_812056 to 812057, EPI_ISL_812059, EPI_ISL_812062 to 812063, EPI_ISL_812065, EPI_ISL_812067 to 812071, EPI_ISL_812074 to 812079, EPI_ISL_812082, EPI_ISL_812084 to 812086, EPI_ISL_812088 to 812089, EPI_ISL_812091, EPI_ISL_812093 to 812099, EPI_ISL_812101 to 812102, EPI_ISL_812104 to 812113, EPI_ISL_820649, EPI_ISL_820655 to 820656, EPI_ISL_820658 to 820659, EPI_ISL_820661 to 820664, EPI_ISL_820666 to 820671, EPI_ISL_820674 to 820676, EPI_ISL_820679 to 820685, EPI_ISL_820689 to 820691, EPI_ISL_820696 to 820702, EPI_ISL_820706 to 820707, EPI_ISL_820709 to 820721, EPI_ISL_820724 to 820732, EPI_ISL_820734 to 820735, EPI_ISL_820737 to 820739, EPI_ISL_820741 to 820746, EPI_ISL_820749 to 820751, EPI_ISL_820753, EPI_ISL_820755 to 820758, EPI_ISL_820760 to 820763, EPI_ISL_820765 to 820767, EPI_ISL_820769 to 820781, EPI_ISL_820783 to 820795, EPI_ISL_820797 to 820798, EPI_ISL_820800 to 820801, EPI_ISL_820803 to 820814, EPI_ISL_820816 to 820819, EPI_ISL_820821 to 820822, EPI_ISL_820824 to 820827, EPI_ISL_820829 to 820832, EPI_ISL_820834 to 820838, EPI_ISL_820840 to 820845, EPI_ISL_820847 to 820850, EPI_ISL_820853 to 820856, EPI_ISL_820858 to 820861, EPI_ISL_820863, EPI_ISL_820866 to 820867, EPI_ISL_820870 to 820871, EPI_ISL_820873 to 820875, EPI_ISL_820878 to 820884, EPI_ISL_820886, EPI_ISL_820888, EPI_ISL_820890 to 820892, EPI_ISL_820894, EPI_ISL_820899 to 820902, EPI_ISL_820904 to 820907, EPI_ISL_820909 to 820913, EPI_ISL_820915 to 820920, EPI_ISL_820922 to 820927, EPI_ISL_820929 to 820934, EPI_ISL_820936 to 820944, EPI_ISL_820946 to 820947, EPI_ISL_820950 to 820951, EPI_ISL_820953, EPI_ISL_820955, EPI_ISL_820957, EPI_ISL_820959 to 820974, EPI_ISL_820976 to 821001, EPI_ISL_821274 to 821275, EPI_ISL_821277, EPI_ISL_821280 to 821282, EPI_ISL_821291, EPI_ISL_821293, EPI_ISL_821296 to 821297, EPI_ISL_821299 to 821300, EPI_ISL_821302, EPI_ISL_821304 to 821307, EPI_ISL_821312 to 821313, EPI_ISL_821315, EPI_ISL_821317, EPI_ISL_821319, EPI_ISL_821321 to 821322, EPI_ISL_821325 to 821326, EPI_ISL_821328, EPI_ISL_821330 to 821332, EPI_ISL_821334, EPI_ISL_821337, EPI_ISL_821340 to 821341, EPI_ISL_821344 to 821345, EPI_ISL_821349, EPI_ISL_821352, EPI_ISL_821355 to 821359, EPI_ISL_821361 to 821362, EPI_ISL_821364, EPI_ISL_821369, EPI_ISL_821373, EPI_ISL_821377, EPI_ISL_821379 to 821380, EPI_ISL_821385, EPI_ISL_821387 to 821388, EPI_ISL_821391, EPI_ISL_821395 to 821397, EPI_ISL_821400, EPI_ISL_821402, EPI_ISL_821405, EPI_ISL_821407, EPI_ISL_821409, EPI_ISL_821417, EPI_ISL_821420, EPI_ISL_821422, EPI_ISL_821424, EPI_ISL_821428 to 821429, EPI_ISL_821431 to 821432, EPI_ISL_821434, EPI_ISL_821438, EPI_ISL_821443, EPI_ISL_821445, EPI_ISL_821448 to 821450, EPI_ISL_821452, EPI_ISL_821455, EPI_ISL_821457, EPI_ISL_821460 to 821462, EPI_ISL_821464, EPI_ISL_821467 to 821468, EPI_ISL_821471 to 821472, EPI_ISL_821474 to 821475, EPI_ISL_821477 to 821478, EPI_ISL_821480 to 821481, EPI_ISL_821486, EPI_ISL_821489, EPI_ISL_821494, EPI_ISL_821498 to 821499, EPI_ISL_821501, EPI_ISL_821505, EPI_ISL_821507 to 821509, EPI_ISL_821512, EPI_ISL_821515, EPI_ISL_821517, EPI_ISL_821521 to 821522, EPI_ISL_821524, EPI_ISL_821527, EPI_ISL_821530 to 821532, EPI_ISL_821535 to 821537, EPI_ISL_821539 to 821542, EPI_ISL_821545, EPI_ISL_821560 to 821562, EPI_ISL_821569, EPI_ISL_821571, EPI_ISL_821573 to 821574, EPI_ISL_821578, EPI_ISL_821580 to 821582, EPI_ISL_821587, EPI_ISL_821589, EPI_ISL_821591, EPI_ISL_821595, EPI_ISL_821597 to 821598, EPI_ISL_821601, EPI_ISL_821603, EPI_ISL_821609 to 821612, EPI_ISL_821619, EPI_ISL_821625, EPI_ISL_821629, EPI_ISL_821631 to 821638, EPI_ISL_821640 to 821642, EPI_ISL_821645, EPI_ISL_821647 to 821648, EPI_ISL_821650 to 821653, EPI_ISL_821655 to 821658, EPI_ISL_822305, EPI_ISL_826466, EPI_ISL_833590, EPI_ISL_833593, EPI_ISL_833596 to 833597, EPI_ISL_833604, EPI_ISL_833611 to 833612, EPI_ISL_833614 to 833615, EPI_ISL_833617 to 833621, EPI_ISL_833629, EPI_ISL_833632, EPI_ISL_833634, EPI_ISL_833643, EPI_ISL_833647 to 833648, EPI_ISL_833658, EPI_ISL_833667, EPI_ISL_833676, EPI_ISL_833678, EPI_ISL_833685, EPI_ISL_833693, EPI_ISL_833700, EPI_ISL_833712, EPI_ISL_833716, EPI_ISL_833721, EPI_ISL_833725 to 833726, EPI_ISL_833736, EPI_ISL_833743, EPI_ISL_833755 to 833756, EPI_ISL_833759, EPI_ISL_833766 to 833768, EPI_ISL_833780, EPI_ISL_833791, EPI_ISL_833795, EPI_ISL_833801, EPI_ISL_833803 to 833804, EPI_ISL_833809, EPI_ISL_833814, EPI_ISL_833817 to 833818, EPI_ISL_833823, EPI_ISL_833830, EPI_ISL_833834 to 833835, EPI_ISL_833845 to 833846, EPI_ISL_833848, EPI_ISL_833856, EPI_ISL_833865, EPI_ISL_833868, EPI_ISL_833872, EPI_ISL_833879, EPI_ISL_833881, EPI_ISL_833884, EPI_ISL_833889 to 833890, EPI_ISL_833894 to 833895, EPI_ISL_833901, EPI_ISL_833908, EPI_ISL_833916, EPI_ISL_833924, EPI_ISL_833930 to 833931, EPI_ISL_835710 to 835722, EPI_ISL_835724 to 835727, EPI_ISL_835729 to 835730, EPI_ISL_835733 to 835735, EPI_ISL_835737 to 835739, EPI_ISL_835742 to 835745, EPI_ISL_835747, EPI_ISL_835749 to 835758, EPI_ISL_835760 to 835769, EPI_ISL_835771 to 835772, EPI_ISL_835774, EPI_ISL_835778, EPI_ISL_835781, EPI_ISL_835783 to 835797, EPI_ISL_835801 to 835805, EPI_ISL_835808, EPI_ISL_835810 to 835816, EPI_ISL_835818 to 835837, EPI_ISL_835839 to 835844, EPI_ISL_835846, EPI_ISL_835848 to 835850, EPI_ISL_835853 to 835858, EPI_ISL_835861 to 835864, EPI_ISL_835868 to 835873, EPI_ISL_835877 to 835879, EPI_ISL_835881 to 835885, EPI_ISL_835887 to 835888, EPI_ISL_835891 to 835894, EPI_ISL_835896 to 835901, EPI_ISL_835903 to 835904, EPI_ISL_835906 to 835907, EPI_ISL_835910, EPI_ISL_835913, EPI_ISL_835915 to 835917, EPI_ISL_835919 to 835921, EPI_ISL_835923, EPI_ISL_835925 to 835926, EPI_ISL_835928 to 835933, EPI_ISL_835936, EPI_ISL_835938 to 835941, EPI_ISL_835943, EPI_ISL_835945 to 835950, EPI_ISL_835953 to 835958, EPI_ISL_835960, EPI_ISL_835964 to 835968, EPI_ISL_835971 to 835972, EPI_ISL_835975 to 835982, EPI_ISL_835984 to 835985, EPI_ISL_835987 to 835993, EPI_ISL_835995 to 836001, EPI_ISL_836003 to 836005, EPI_ISL_836007 to 836018, EPI_ISL_836022, EPI_ISL_836024 to 836025, EPI_ISL_836027 to 836036, EPI_ISL_836039 to 836040, EPI_ISL_836042, EPI_ISL_836044 to 836049, EPI_ISL_836051, EPI_ISL_836053 to 836054, EPI_ISL_836056 to 836060, EPI_ISL_836064 to 836070, EPI_ISL_836072 to 836074, EPI_ISL_836076 to 836079, EPI_ISL_836081 to 836086, EPI_ISL_836088 to 836095, EPI_ISL_836097 to 836108, EPI_ISL_836110, EPI_ISL_836113 to 836114, EPI_ISL_836116 to 836118, EPI_ISL_836120 to 836128, EPI_ISL_836131, EPI_ISL_836133, EPI_ISL_836135 to 836136, EPI_ISL_836140 to 836141, EPI_ISL_836144 to 836146, EPI_ISL_836150 to 836151, EPI_ISL_836153 to 836154, EPI_ISL_836156 to 836160, EPI_ISL_836163 to 836166, EPI_ISL_836168 to 836171, EPI_ISL_836173, EPI_ISL_836175 to 836176, EPI_ISL_836178 to 836180, EPI_ISL_836183 to 836184, EPI_ISL_836186 to 836191, EPI_ISL_836193 to 836195, EPI_ISL_836200, EPI_ISL_836202 to 836206, EPI_ISL_836208 to 836210, EPI_ISL_836212 to 836220, EPI_ISL_836223 to 836224, EPI_ISL_836226 to 836227, EPI_ISL_836230, EPI_ISL_836232 to 836233, EPI_ISL_836235 to 836236, EPI_ISL_836238, EPI_ISL_836240, EPI_ISL_836242, EPI_ISL_836244 to 836250, EPI_ISL_836252 to 836253, EPI_ISL_836255 to 836256, EPI_ISL_836258 to 836261, EPI_ISL_836263, EPI_ISL_836265 to 836271, EPI_ISL_836273 to 836274, EPI_ISL_836276, EPI_ISL_836278 to 836280, EPI_ISL_836282, EPI_ISL_836284, EPI_ISL_836288, EPI_ISL_836292, EPI_ISL_836294 to 836295, EPI_ISL_836297 to 836299, EPI_ISL_836302 to 836304, EPI_ISL_836306, EPI_ISL_836308 to 836310, EPI_ISL_836312 to 836314, EPI_ISL_836316 to 836319, EPI_ISL_836321 to 836324, EPI_ISL_836326 to 836329, EPI_ISL_836331, EPI_ISL_836333, EPI_ISL_836335, EPI_ISL_836338 to 836342, EPI_ISL_836344 to 836347, EPI_ISL_836350 to 836352, EPI_ISL_836355 to 836359, EPI_ISL_836363 to 836374, EPI_ISL_836377, EPI_ISL_836379, EPI_ISL_836383 to 836384, EPI_ISL_836386 to 836389, EPI_ISL_836392 to 836393, EPI_ISL_836396, EPI_ISL_836400 to 836404, EPI_ISL_836406, EPI_ISL_836408 to 836412, EPI_ISL_836414, EPI_ISL_836416 to 836420, EPI_ISL_851957 to 851958, EPI_ISL_851960 to 851961, EPI_ISL_851963, EPI_ISL_851965 to 851969, EPI_ISL_851971 to 851972, EPI_ISL_851974 to 851976, EPI_ISL_851978 to 851984, EPI_ISL_851986, EPI_ISL_851990 to 851994, EPI_ISL_851996 to 852000, EPI_ISL_852002 to 852003, EPI_ISL_852007 to 852024, EPI_ISL_852026 to 852029, EPI_ISL_852032 to 852035, EPI_ISL_852037, EPI_ISL_852040 to 852041, EPI_ISL_852043, EPI_ISL_852045, EPI_ISL_852048 to 852053, EPI_ISL_852055, EPI_ISL_852057 to 852063, EPI_ISL_852065 to 852066, EPI_ISL_852069 to 852070, EPI_ISL_852072 to 852073, EPI_ISL_852076, EPI_ISL_852078, EPI_ISL_852080, EPI_ISL_852082, EPI_ISL_852086 to 852087, EPI_ISL_852089 to 852090, EPI_ISL_852092 to 852094, EPI_ISL_852097 to 852099, EPI_ISL_852101 to 852103, EPI_ISL_852105 to 852106, EPI_ISL_852108 to 852115, EPI_ISL_852118 to 852122, EPI_ISL_852124 to 852126, EPI_ISL_852128 to 852140, EPI_ISL_852142, EPI_ISL_852144 to 852152, EPI_ISL_852155 to 852160, EPI_ISL_852163, EPI_ISL_852166 to 852168, EPI_ISL_852170, EPI_ISL_852172 to 852175, EPI_ISL_852177 to 852178, EPI_ISL_852180 to 852182, EPI_ISL_852185 to 852187, EPI_ISL_852189, EPI_ISL_852192 to 852193, EPI_ISL_852195 to 852199, EPI_ISL_852201 to 852204, EPI_ISL_852211, EPI_ISL_852213, EPI_ISL_852215, EPI_ISL_852217 to 852218, EPI_ISL_852220, EPI_ISL_852222 to 852224, EPI_ISL_852226 to 852229, EPI_ISL_852233 to 852236, EPI_ISL_852238 to 852255, EPI_ISL_858268, EPI_ISL_858803, EPI_ISL_863531, EPI_ISL_863533 to 863537, EPI_ISL_863540, EPI_ISL_863542 to 863544, EPI_ISL_863546 to 863556, EPI_ISL_863559 to 863575, EPI_ISL_863577 to 863578, EPI_ISL_863581 to 863585, EPI_ISL_863587 to 863589, EPI_ISL_863591 to 863598, EPI_ISL_863601 to 863606, EPI_ISL_863608 to 863610, EPI_ISL_863612 to 863617, EPI_ISL_863619 to 863620, EPI_ISL_863622, EPI_ISL_863624 to 863626, EPI_ISL_863629 to 863630, EPI_ISL_863634, EPI_ISL_863636 to 863637, EPI_ISL_863641 to 863644, EPI_ISL_863646 to 863648, EPI_ISL_863650, EPI_ISL_863652, EPI_ISL_863654 to 863658, EPI_ISL_863660 to 863662, EPI_ISL_863664 to 863665, EPI_ISL_863667, EPI_ISL_863669 to 863682, EPI_ISL_863684, EPI_ISL_863687 to 863695, EPI_ISL_863697, EPI_ISL_863699 to 863700, EPI_ISL_863702 to 863706, EPI_ISL_863708 to 863714, EPI_ISL_863718 to 863720, EPI_ISL_863722 to 863724, EPI_ISL_863726 to 863728, EPI_ISL_863730 to 863731, EPI_ISL_863734, EPI_ISL_863736 to 863738, EPI_ISL_863740 to 863741, EPI_ISL_863744 to 863745, EPI_ISL_863747 to 863758, EPI_ISL_863760 to 863761, EPI_ISL_863763 to 863789, EPI_ISL_863792 to 863797, EPI_ISL_863799 to 863800, EPI_ISL_863802, EPI_ISL_863805 to 863807, EPI_ISL_863809 to 863810, EPI_ISL_863813 to 863814, EPI_ISL_863816 to 863829, EPI_ISL_863831 to 863833, EPI_ISL_863836 to 863840, EPI_ISL_863842 to 863844, EPI_ISL_863846 to 863852, EPI_ISL_863854 to 863855, EPI_ISL_863858 to 863860, EPI_ISL_863862 to 863866, EPI_ISL_863868, EPI_ISL_863899, EPI_ISL_863913, EPI_ISL_863921 to 863922, EPI_ISL_863956, EPI_ISL_863969, EPI_ISL_864005, EPI_ISL_864009, EPI_ISL_864026, EPI_ISL_864036, EPI_ISL_864043, EPI_ISL_864098, EPI_ISL_864165, EPI_ISL_864169, EPI_ISL_864173, EPI_ISL_864175 to 864176, EPI_ISL_864191, EPI_ISL_864204 to 864209, EPI_ISL_864211 to 864212, EPI_ISL_864214, EPI_ISL_864216 to 864220, EPI_ISL_864224 to 864225, EPI_ISL_864227 to 864239, EPI_ISL_864241 to 864249, EPI_ISL_864252 to 864265, EPI_ISL_864269 to 864277, EPI_ISL_864279, EPI_ISL_864281 to 864283, EPI_ISL_864285 to 864288, EPI_ISL_864290 to 864292, EPI_ISL_864294, EPI_ISL_864296, EPI_ISL_864298 to 864299, EPI_ISL_864302 to 864306, EPI_ISL_864309 to 864317, EPI_ISL_864320 to 864327, EPI_ISL_864329 to 864333, EPI_ISL_864336 to 864340, EPI_ISL_864343 to 864345, EPI_ISL_864349 to 864352, EPI_ISL_864354, EPI_ISL_864359 to 864367, EPI_ISL_864370, EPI_ISL_864372 to 864381, EPI_ISL_864383 to 864388, EPI_ISL_864390 to 864394, EPI_ISL_864398, EPI_ISL_864401, EPI_ISL_864403, EPI_ISL_864405 to 864418, EPI_ISL_864420 to 864422, EPI_ISL_864424 to 864426, EPI_ISL_864428 to 864430, EPI_ISL_864434 to 864436, EPI_ISL_864438 to 864440, EPI_ISL_864442 to 864443, EPI_ISL_864445 to 864448, EPI_ISL_864450 to 864451, EPI_ISL_864453 to 864457, EPI_ISL_864460 to 864473, EPI_ISL_864475 to 864476, EPI_ISL_864479 to 864481, EPI_ISL_864483 to 864485, EPI_ISL_864487 to 864490, EPI_ISL_864492 to 864496, EPI_ISL_864498 to 864499, EPI_ISL_864501 to 864502, EPI_ISL_864507 to 864510, EPI_ISL_864512 to 864515, EPI_ISL_864517 to 864519, EPI_ISL_864521 to 864524, EPI_ISL_864526 to 864528, EPI_ISL_864530 to 864531, EPI_ISL_864533 to 864535, EPI_ISL_864538, EPI_ISL_864540 to 864542, EPI_ISL_873279 to 873281, EPI_ISL_873284, EPI_ISL_873287, EPI_ISL_873289 to 873290, EPI_ISL_873293 to 873294, EPI_ISL_873296, EPI_ISL_873298, EPI_ISL_873300 to 873301, EPI_ISL_873305, EPI_ISL_873307 to 873309, EPI_ISL_873311, EPI_ISL_873314 to 873315, EPI_ISL_873317, EPI_ISL_873321, EPI_ISL_873323, EPI_ISL_873327 to 873332, EPI_ISL_873335 to 873336, EPI_ISL_873345 to 873346, EPI_ISL_873349, EPI_ISL_873351 to 873352, EPI_ISL_873355 to 873356, EPI_ISL_873360 to 873361, EPI_ISL_873363, EPI_ISL_873366 to 873367, EPI_ISL_873372 to 873373, EPI_ISL_873376 to 873379, EPI_ISL_873383 to 873387, EPI_ISL_873389 to 873398, EPI_ISL_873400 to 873402, EPI_ISL_873404 to 873407, EPI_ISL_873409 to 873411, EPI_ISL_873413, EPI_ISL_873420 to 873421, EPI_ISL_873423 to 873425, EPI_ISL_873427, EPI_ISL_873430, EPI_ISL_873434 to 873441, EPI_ISL_873444 to 873450, EPI_ISL_873453, EPI_ISL_873455, EPI_ISL_873460 to 873461, EPI_ISL_873463, EPI_ISL_873466 to 873467, EPI_ISL_873470 to 873472, EPI_ISL_873474 to 873475, EPI_ISL_873478 to 873480, EPI_ISL_873483 to 873487, EPI_ISL_873493, EPI_ISL_873496, EPI_ISL_873498, EPI_ISL_873502 to 873506, EPI_ISL_873508, EPI_ISL_873511 to 873512, EPI_ISL_873514 to 873515, EPI_ISL_873517, EPI_ISL_873520, EPI_ISL_873522 to 873523, EPI_ISL_873525 to 873526, EPI_ISL_873528 to 873531, EPI_ISL_873533 to 873536, EPI_ISL_873538 to 873541, EPI_ISL_873544 to 873550, EPI_ISL_873553, EPI_ISL_873556, EPI_ISL_873559 to 873560, EPI_ISL_873562 to 873565, EPI_ISL_873567 to 873570, EPI_ISL_873572, EPI_ISL_873575, EPI_ISL_873577 to 873581, EPI_ISL_873583, EPI_ISL_873585 to 873589, EPI_ISL_873591 to 873592, EPI_ISL_873594, EPI_ISL_873596 to 873601, EPI_ISL_873603 to 873604, EPI_ISL_873606 to 873610, EPI_ISL_873612, EPI_ISL_873615 to 873616, EPI_ISL_873618, EPI_ISL_873620, EPI_ISL_873622 to 873623, EPI_ISL_873626 to 873628, EPI_ISL_873631, EPI_ISL_873641, EPI_ISL_873645, EPI_ISL_873652, EPI_ISL_873654, EPI_ISL_873656 to 873657, EPI_ISL_873660, EPI_ISL_873669, EPI_ISL_873672, EPI_ISL_873679, EPI_ISL_873684, EPI_ISL_873716, EPI_ISL_873725 to 873726, EPI_ISL_873729 to 873730, EPI_ISL_873734, EPI_ISL_873741, EPI_ISL_873743 to 873744, EPI_ISL_873748 to 873749, EPI_ISL_873755, EPI_ISL_873769, EPI_ISL_873783, EPI_ISL_873786, EPI_ISL_873794, EPI_ISL_873797, EPI_ISL_873806, EPI_ISL_873809, EPI_ISL_873812, EPI_ISL_873817, EPI_ISL_873822, EPI_ISL_873827, EPI_ISL_873829, EPI_ISL_873833 to 873834, EPI_ISL_873840, EPI_ISL_873842, EPI_ISL_873847, EPI_ISL_873853, EPI_ISL_873857, EPI_ISL_873859 to 873860, EPI_ISL_873862, EPI_ISL_873865, EPI_ISL_873870, EPI_ISL_873873, EPI_ISL_873875, EPI_ISL_873884, EPI_ISL_873886 to 873887, EPI_ISL_873900, EPI_ISL_873904, EPI_ISL_873910, EPI_ISL_873913, EPI_ISL_873916, EPI_ISL_873921, EPI_ISL_873923 to 873926, EPI_ISL_873928, EPI_ISL_873934, EPI_ISL_873937, EPI_ISL_873942, EPI_ISL_873947 to 873948, EPI_ISL_873950, EPI_ISL_873954, EPI_ISL_873956, EPI_ISL_873960, EPI_ISL_874990 to 874992, EPI_ISL_874994 to 874996, EPI_ISL_874998 to 875002, EPI_ISL_875004 to 875006, EPI_ISL_875008 to 875019, EPI_ISL_875021 to 875024, EPI_ISL_875026 to 875031, EPI_ISL_875033 to 875036, EPI_ISL_875038 to 875039, EPI_ISL_875041, EPI_ISL_875043, EPI_ISL_875045, EPI_ISL_875047, EPI_ISL_875049, EPI_ISL_875051 to 875052, EPI_ISL_875055, EPI_ISL_875057 to 875061, EPI_ISL_875063 to 875080, EPI_ISL_875082 to 875097, EPI_ISL_875100 to 875102, EPI_ISL_875105 to 875111, EPI_ISL_875113 to 875128, EPI_ISL_875130 to 875146, EPI_ISL_875148 to 875161, EPI_ISL_875164 to 875171, EPI_ISL_875173 to 875197, EPI_ISL_875199 to 875202, EPI_ISL_875204, EPI_ISL_875206 to 875209, EPI_ISL_875211 to 875218, EPI_ISL_875220 to 875225, EPI_ISL_875227 to 875231, EPI_ISL_875233, EPI_ISL_875235 to 875241, EPI_ISL_875243, EPI_ISL_875246 to 875255, EPI_ISL_875257 to 875263, EPI_ISL_875267, EPI_ISL_875269, EPI_ISL_875271 to 875274, EPI_ISL_875276 to 875281, EPI_ISL_875283, EPI_ISL_875285 to 875290, EPI_ISL_875292 to 875306, EPI_ISL_875308 to 875310, EPI_ISL_875313 to 875316, EPI_ISL_875319, EPI_ISL_875321 to 875326, EPI_ISL_875328 to 875332, EPI_ISL_875334 to 875341, EPI_ISL_878759, EPI_ISL_878769, EPI_ISL_878772, EPI_ISL_878782, EPI_ISL_878792 to 878793, EPI_ISL_878800, EPI_ISL_878806 to 878807, EPI_ISL_878814, EPI_ISL_878824, EPI_ISL_878833, EPI_ISL_878842, EPI_ISL_878846, EPI_ISL_878848, EPI_ISL_878851 to 878852, EPI_ISL_878858, EPI_ISL_878860, EPI_ISL_878879 to 878880, EPI_ISL_878882, EPI_ISL_878884, EPI_ISL_878892, EPI_ISL_878896, EPI_ISL_878902, EPI_ISL_878906, EPI_ISL_878918 to 878919, EPI_ISL_878934, EPI_ISL_878941, EPI_ISL_878943, EPI_ISL_878951 to 878952, EPI_ISL_878967, EPI_ISL_878971 to 878972, EPI_ISL_878982, EPI_ISL_878990, EPI_ISL_879001, EPI_ISL_879009, EPI_ISL_879013 to 879014, EPI_ISL_879019, EPI_ISL_879021, EPI_ISL_879023, EPI_ISL_879025, EPI_ISL_879028, EPI_ISL_879032, EPI_ISL_879034, EPI_ISL_879037 to 879038, EPI_ISL_879042 to 879044, EPI_ISL_879049, EPI_ISL_879052, EPI_ISL_879055, EPI_ISL_879057, EPI_ISL_879063, EPI_ISL_879065, EPI_ISL_879086 to 879087, EPI_ISL_879089, EPI_ISL_879092 to 879093, EPI_ISL_879096, EPI_ISL_879378, EPI_ISL_879388, EPI_ISL_879395 to 879397, EPI_ISL_879404, EPI_ISL_879409, EPI_ISL_879412 to 879413, EPI_ISL_879424 to 879425, EPI_ISL_879429, EPI_ISL_879442, EPI_ISL_879448 to 879450, EPI_ISL_879457, EPI_ISL_879459, EPI_ISL_879461, EPI_ISL_879464, EPI_ISL_879467, EPI_ISL_879471, EPI_ISL_879477, EPI_ISL_879479, EPI_ISL_879495, EPI_ISL_879502, EPI_ISL_879514, EPI_ISL_879526, EPI_ISL_879538, EPI_ISL_879540, EPI_ISL_879552, EPI_ISL_879554, EPI_ISL_879560 to 879561, EPI_ISL_879571 to 879572, EPI_ISL_879575 to 879576, EPI_ISL_879581, EPI_ISL_879586, EPI_ISL_879591 to 879592, EPI_ISL_879597, EPI_ISL_879600 to 879601, EPI_ISL_879608, EPI_ISL_879611, EPI_ISL_879613, EPI_ISL_879618, EPI_ISL_879621, EPI_ISL_879623, EPI_ISL_879628, EPI_ISL_879642 to 879643, EPI_ISL_879653 to 879654, EPI_ISL_879663 to 879664, EPI_ISL_879675, EPI_ISL_879678, EPI_ISL_879681, EPI_ISL_879686, EPI_ISL_879689, EPI_ISL_879703, EPI_ISL_879707, EPI_ISL_879710 to 879713, EPI_ISL_879715, EPI_ISL_879717, EPI_ISL_880584, EPI_ISL_880586 to 880594, EPI_ISL_880596 to 880597, EPI_ISL_880599 to 880600, EPI_ISL_880602, EPI_ISL_880605 to 880606, EPI_ISL_880608 to 880613, EPI_ISL_880615 to 880617, EPI_ISL_880619, EPI_ISL_880622 to 880623, EPI_ISL_880625 to 880626, EPI_ISL_880628 to 880631, EPI_ISL_880633, EPI_ISL_880635 to 880636, EPI_ISL_880638 to 880642, EPI_ISL_880645, EPI_ISL_880648 to 880663, EPI_ISL_880665 to 880666, EPI_ISL_880668, EPI_ISL_880670 to 880671, EPI_ISL_880673 to 880674, EPI_ISL_880676 to 880681, EPI_ISL_880683 to 880687, EPI_ISL_880689, EPI_ISL_880691, EPI_ISL_880693 to 880694, EPI_ISL_880696, EPI_ISL_880698, EPI_ISL_880701 to 880709, EPI_ISL_880711 to 880713, EPI_ISL_880716, EPI_ISL_880718 to 880724, EPI_ISL_880726 to 880727, EPI_ISL_880730 to 880732, EPI_ISL_880736 to 880737, EPI_ISL_880740 to 880741, EPI_ISL_880743 to 880744, EPI_ISL_880746, EPI_ISL_880748 to 880750, EPI_ISL_880753 to 880754, EPI_ISL_880756 to 880757, EPI_ISL_880760, EPI_ISL_880762 to 880778, EPI_ISL_880780, EPI_ISL_880782 to 880785, EPI_ISL_880787, EPI_ISL_880789 to 880792, EPI_ISL_880794 to 880795, EPI_ISL_880798 to 880799, EPI_ISL_880801 to 880803, EPI_ISL_880805, EPI_ISL_880807 to 880809, EPI_ISL_880811 to 880813, EPI_ISL_880816 to 880818, EPI_ISL_880820 to 880824, EPI_ISL_880826 to 880827, EPI_ISL_880829 to 880833, EPI_ISL_880836 to 880837, EPI_ISL_880839, EPI_ISL_880841 to 880844, EPI_ISL_880847 to 880857, EPI_ISL_880859 to 880862, EPI_ISL_880864 to 880866, EPI_ISL_880868 to 880870, EPI_ISL_880872, EPI_ISL_880874, EPI_ISL_880876 to 880878, EPI_ISL_880880 to 880881, EPI_ISL_880885 to 880887, EPI_ISL_880889 to 880890, EPI_ISL_880893, EPI_ISL_880895 to 880896, EPI_ISL_880901 to 880902, EPI_ISL_880904 to 880909, EPI_ISL_880911 to 880913, EPI_ISL_880915, EPI_ISL_880918, EPI_ISL_880921 to 880935, EPI_ISL_881618, EPI_ISL_882610, EPI_ISL_908936 to 908937, EPI_ISL_908939 to 908941, EPI_ISL_908943, EPI_ISL_908947 to 908948, EPI_ISL_908950, EPI_ISL_908954, EPI_ISL_908956, EPI_ISL_908960, EPI_ISL_908964, EPI_ISL_908966, EPI_ISL_908969 to 908973, EPI_ISL_908976, EPI_ISL_908980 to 908981, EPI_ISL_908984, EPI_ISL_908987 to 908988, EPI_ISL_908995 to 908997, EPI_ISL_909000 to 909001, EPI_ISL_909003, EPI_ISL_909012 to 909014, EPI_ISL_909016, EPI_ISL_909018, EPI_ISL_909020, EPI_ISL_909022 to 909023, EPI_ISL_909025 to 909027, EPI_ISL_909032 to 909033, EPI_ISL_909037, EPI_ISL_909039, EPI_ISL_909041 to 909045, EPI_ISL_909051, EPI_ISL_909054 to 909055, EPI_ISL_909059, EPI_ISL_909062,

EPI_ISL_909064 to 909065, EPI_ISL_909070, EPI_ISL_909076 to 909078, EPI_ISL_909082, EPI_ISL_909084, EPI_ISL_909091, EPI_ISL_909098, EPI_ISL_909104, EPI_ISL_909108, EPI_ISL_909110, EPI_ISL_909112, EPI_ISL_909123 to 909124, EPI_ISL_909126 to 909127, EPI_ISL_909129, EPI_ISL_909132, EPI_ISL_909135 to 909137, EPI_ISL_909139 to 909142, EPI_ISL_909146 to 909147, EPI_ISL_909149, EPI_ISL_909151 to 909154, EPI_ISL_909159 to 909161, EPI_ISL_909163 to 909164, EPI_ISL_909166 to 909168, EPI_ISL_909172, EPI_ISL_909175, EPI_ISL_909179, EPI_ISL_909181, EPI_ISL_909183, EPI_ISL_909185 to 909186, EPI_ISL_909188, EPI_ISL_909190, EPI_ISL_909195 to 909196, EPI_ISL_909198 to 909199, EPI_ISL_909201, EPI_ISL_909205, EPI_ISL_909207, EPI_ISL_909209, EPI_ISL_909213 to 909214, EPI_ISL_909216, EPI_ISL_909221 to 909222, EPI_ISL_909224, EPI_ISL_909226, EPI_ISL_909229, EPI_ISL_909232, EPI_ISL_909241, EPI_ISL_909244 to 909250, EPI_ISL_909252 to 909253, EPI_ISL_909256, EPI_ISL_909258 to 909259, EPI_ISL_909261, EPI_ISL_909264 to 909268, EPI_ISL_909271, EPI_ISL_909274, EPI_ISL_909276 to 909277, EPI_ISL_909282, EPI_ISL_909284, EPI_ISL_909286 to 909288, EPI_ISL_909290 to 909291, EPI_ISL_909299 to 909301, EPI_ISL_909305, EPI_ISL_909307 to 909308, EPI_ISL_909310, EPI_ISL_909313, EPI_ISL_909315, EPI_ISL_909319 to 909320, EPI_ISL_909323, EPI_ISL_909329 to 909332, EPI_ISL_909335 to 909336, EPI_ISL_909338, EPI_ISL_909347, EPI_ISL_909350, EPI_ISL_909354 to 909355, EPI_ISL_909357, EPI_ISL_909360, EPI_ISL_909362 to 909363, EPI_ISL_909365, EPI_ISL_909377, EPI_ISL_909383, EPI_ISL_909386, EPI_ISL_909395 to 909396, EPI_ISL_909398 to 909399, EPI_ISL_909402, EPI_ISL_909404, EPI_ISL_909411 to 909413, EPI_ISL_909415, EPI_ISL_909418 to 909420, EPI_ISL_909427, EPI_ISL_909430, EPI_ISL_909432 to 909434, EPI_ISL_909436, EPI_ISL_909441, EPI_ISL_909444 to 909445, EPI_ISL_909448 to 909449, EPI_ISL_909451 to 909452, EPI_ISL_909454 to 909455, EPI_ISL_909458 to 909459, EPI_ISL_909461 to 909464, EPI_ISL_909469 to 909470, EPI_ISL_909473 to 909474, EPI_ISL_909477, EPI_ISL_909479 to 909480, EPI_ISL_909483, EPI_ISL_909485 to 909486, EPI_ISL_909489, EPI_ISL_909491 to 909493, EPI_ISL_909495, EPI_ISL_909499, EPI_ISL_909501 to 909502, EPI_ISL_909505 to 909507, EPI_ISL_909510 to 909511, EPI_ISL_909515, EPI_ISL_909519 to 909521, EPI_ISL_909523, EPI_ISL_909527, EPI_ISL_909530, EPI_ISL_909533, EPI_ISL_909535, EPI_ISL_909540 to 909543, EPI_ISL_909547, EPI_ISL_909551, EPI_ISL_909556, EPI_ISL_909558 to 909560, EPI_ISL_909562 to 909563, EPI_ISL_909565 to 909567, EPI_ISL_909576, EPI_ISL_909579, EPI_ISL_909584, EPI_ISL_909587, EPI_ISL_909591 to 909592, EPI_ISL_912224 to 912225, EPI_ISL_932253, EPI_ISL_932258 to 932260, EPI_ISL_932262 to 932263, EPI_ISL_932278, EPI_ISL_932283, EPI_ISL_932288, EPI_ISL_932294, EPI_ISL_932298, EPI_ISL_932306 to 932307, EPI_ISL_932320, EPI_ISL_932324, EPI_ISL_932327, EPI_ISL_932332, EPI_ISL_932339 to 932340, EPI_ISL_932346, EPI_ISL_932350, EPI_ISL_932354, EPI_ISL_932363 to 932366, EPI_ISL_932368, EPI_ISL_932372, EPI_ISL_932374 to 932375, EPI_ISL_932377, EPI_ISL_932380, EPI_ISL_932383, EPI_ISL_932385, EPI_ISL_932387, EPI_ISL_932395 to 932396, EPI_ISL_932399, EPI_ISL_932402, EPI_ISL_932406, EPI_ISL_932411, EPI_ISL_932413, EPI_ISL_932417, EPI_ISL_932419, EPI_ISL_932426, EPI_ISL_932428 to 932429, EPI_ISL_932434, EPI_ISL_932437, EPI_ISL_932442, EPI_ISL_932456, EPI_ISL_932469, EPI_ISL_932472, EPI_ISL_932474, EPI_ISL_932476 to 932478, EPI_ISL_932483 to 932485, EPI_ISL_932491 to 932492, EPI_ISL_932495 to 932496, EPI_ISL_932504 to 932505, EPI_ISL_932507, EPI_ISL_932509, EPI_ISL_932514, EPI_ISL_932516, EPI_ISL_932519 to 932521, EPI_ISL_932528 to 932529, EPI_ISL_932542, EPI_ISL_932544, EPI_ISL_932546, EPI_ISL_932548 to 932551, EPI_ISL_932553, EPI_ISL_932555, EPI_ISL_932559, EPI_ISL_932561, EPI_ISL_932563, EPI_ISL_932565, EPI_ISL_932570 to 932573, EPI_ISL_932575, EPI_ISL_932577 to 932580, EPI_ISL_932582 to 932586, EPI_ISL_932590 to 932592, EPI_ISL_932594 to 932595, EPI_ISL_932597, EPI_ISL_932600 to 932602, EPI_ISL_932605, EPI_ISL_932608 to 932609, EPI_ISL_932612, EPI_ISL_932616, EPI_ISL_932618, EPI_ISL_932623 to 932634, EPI_ISL_932636 to 932637, EPI_ISL_932639, EPI_ISL_932641 to 932642, EPI_ISL_932649 to 932650, EPI_ISL_932653, EPI_ISL_932657, EPI_ISL_932659, EPI_ISL_932662 to 932663, EPI_ISL_932672, EPI_ISL_932675, EPI_ISL_932679, EPI_ISL_932681, EPI_ISL_932683 to 932685, EPI_ISL_932692, EPI_ISL_932694 to 932695, EPI_ISL_932697 to 932700, EPI_ISL_932702, EPI_ISL_932707 to 932708, EPI_ISL_932713, EPI_ISL_932716, EPI_ISL_932719 to 932720, EPI_ISL_932723 to 932724, EPI_ISL_932726 to 932727, EPI_ISL_932729 to 932731, EPI_ISL_932733, EPI_ISL_932735 to 932736, EPI_ISL_932741, EPI_ISL_932744, EPI_ISL_932747 to 932748, EPI_ISL_932750, EPI_ISL_932752, EPI_ISL_932757, EPI_ISL_932759, EPI_ISL_932762, EPI_ISL_932765 to 932767, EPI_ISL_932769, EPI_ISL_932773 to 932775, EPI_ISL_932777, EPI_ISL_932780, EPI_ISL_932783 to 932784, EPI_ISL_932786, EPI_ISL_932792 to 932793, EPI_ISL_932795, EPI_ISL_932797 to 932799, EPI_ISL_932801 to 932804, EPI_ISL_932806, EPI_ISL_932809 to 932811, EPI_ISL_932813, EPI_ISL_932815, EPI_ISL_932821, EPI_ISL_932823 to 932824, EPI_ISL_932828, EPI_ISL_932833, EPI_ISL_932841, EPI_ISL_932845 to 932849, EPI_ISL_932851 to 932852, EPI_ISL_932854, EPI_ISL_932869, EPI_ISL_932872, EPI_ISL_932875, EPI_ISL_932880, EPI_ISL_932886 to 932888, EPI_ISL_932892, EPI_ISL_932898, EPI_ISL_932904, EPI_ISL_932909 to 932910, EPI_ISL_932913, EPI_ISL_932916, EPI_ISL_932926, EPI_ISL_932933 to 932934, EPI_ISL_932945 to 932946, EPI_ISL_932953 to 932954, EPI_ISL_932959 to 932963, EPI_ISL_932971, EPI_ISL_932974, EPI_ISL_932977 to 932978, EPI_ISL_932985, EPI_ISL_932989, EPI_ISL_932992, EPI_ISL_932998 to 932999, EPI_ISL_933008, EPI_ISL_933012, EPI_ISL_933014, EPI_ISL_933016, EPI_ISL_933019, EPI_ISL_933022 to 933023, EPI_ISL_933027, EPI_ISL_933032, EPI_ISL_933034, EPI_ISL_933039, EPI_ISL_933043, EPI_ISL_933046 to 933048, EPI_ISL_933060 to 933061, EPI_ISL_933063, EPI_ISL_933071, EPI_ISL_933075 to 933078, EPI_ISL_933085, EPI_ISL_933088, EPI_ISL_933091, EPI_ISL_933101, EPI_ISL_933107 to 933108, EPI_ISL_933426 to 933428, EPI_ISL_933430 to 933438, EPI_ISL_933441 to 933444, EPI_ISL_933446, EPI_ISL_933449 to 933455, EPI_ISL_933457, EPI_ISL_933461 to 933462, EPI_ISL_933465 to 933466, EPI_ISL_933469 to 933470, EPI_ISL_933473 to 933477, EPI_ISL_933482, EPI_ISL_933487 to 933489, EPI_ISL_933491, EPI_ISL_933496, EPI_ISL_933498, EPI_ISL_933503, EPI_ISL_933507 to 933509, EPI_ISL_937887, EPI_ISL_937889 to 937891, EPI_ISL_937894 to 937898, EPI_ISL_937901 to 937905, EPI_ISL_937907, EPI_ISL_937910 to 937911, EPI_ISL_937913 to 937917, EPI_ISL_937921, EPI_ISL_937923 to 937924, EPI_ISL_937927 to 937931, EPI_ISL_937933 to 937935, EPI_ISL_937938 to 937939, EPI_ISL_937941, EPI_ISL_937943 to 937948, EPI_ISL_937954, EPI_ISL_937956 to 937959, EPI_ISL_937961 to 937962, EPI_ISL_937964 to 937970, EPI_ISL_937972 to 937976, EPI_ISL_937979, EPI_ISL_937981, EPI_ISL_937983 to 937986, EPI_ISL_937988, EPI_ISL_937991 to 937992, EPI_ISL_937994 to 937996, EPI_ISL_938000 to 938005, EPI_ISL_938007, EPI_ISL_938009 to 938013, EPI_ISL_938015 to 938016, EPI_ISL_938018 to 938021, EPI_ISL_938023 to 938031, EPI_ISL_938034 to 938035, EPI_ISL_938037, EPI_ISL_938040 to 938043, EPI_ISL_938045 to 938049, EPI_ISL_938052 to 938059, EPI_ISL_938061, EPI_ISL_938063 to 938066, EPI_ISL_938069, EPI_ISL_938071 to 938074, EPI_ISL_938076, EPI_ISL_938078 to 938080, EPI_ISL_938082 to 938086, EPI_ISL_938088 to 938095, EPI_ISL_938097 to 938099, EPI_ISL_938101 to 938102, EPI_ISL_938104 to 938108, EPI_ISL_938111, EPI_ISL_938116, EPI_ISL_938119 to 938124, EPI_ISL_938128 to 938135, EPI_ISL_938137 to 938138, EPI_ISL_938140 to 938142, EPI_ISL_938144 to 938151, EPI_ISL_938153, EPI_ISL_938155, EPI_ISL_938158 to 938167, EPI_ISL_938170 to 938180, EPI_ISL_938182 to 938186, EPI_ISL_938189 to 938196, EPI_ISL_938200 to 938204, EPI_ISL_938206 to 938210, EPI_ISL_938212 to 938214, EPI_ISL_938216 to 938222, EPI_ISL_938225 to 938228, EPI_ISL_938230 to 938233, EPI_ISL_938580, EPI_ISL_938582, EPI_ISL_938584 to 938587, EPI_ISL_938589, EPI_ISL_938591 to 938602, EPI_ISL_938604 to 938605, EPI_ISL_938607, EPI_ISL_938609, EPI_ISL_938611 to 938618, EPI_ISL_938620 to 938624, EPI_ISL_938626 to 938635, EPI_ISL_938637 to 938642, EPI_ISL_938644 to 938652, EPI_ISL_938654 to 938664, EPI_ISL_938666, EPI_ISL_938668 to 938671, EPI_ISL_938673 to 938684, EPI_ISL_938686 to 938689, EPI_ISL_938691 to 938692, EPI_ISL_938694, EPI_ISL_938696 to 938698, EPI_ISL_938701 to 938705, EPI_ISL_938709 to 938711, EPI_ISL_938713 to 938725, EPI_ISL_938727 to 938733, EPI_ISL_938736, EPI_ISL_938738, EPI_ISL_938740 to 938741, EPI_ISL_938743 to 938746, EPI_ISL_938749 to 938750, EPI_ISL_938752 to 938756, EPI_ISL_938758 to 938761, EPI_ISL_938763 to 938764, EPI_ISL_938766 to 938767, EPI_ISL_938769 to 938771, EPI_ISL_938773 to 938776, EPI_ISL_938779 to 938780, EPI_ISL_938783 to 938786, EPI_ISL_938788 to 938789, EPI_ISL_938791 to 938801, EPI_ISL_938803 to 938828, EPI_ISL_938830, EPI_ISL_938832 to 938833, EPI_ISL_938836, EPI_ISL_938838 to 938840, EPI_ISL_938842 to 938845, EPI_ISL_938847 to 938856, EPI_ISL_938858 to 938872, EPI_ISL_938875 to 938877, EPI_ISL_938879 to 938894, EPI_ISL_938896 to 938901, EPI_ISL_938903 to 938905, EPI_ISL_938907 to 938909, EPI_ISL_938911 to 938912, EPI_ISL_938914 to 938925, EPI_ISL_938928 to 938929, EPI_ISL_938931 to 938933, EPI_ISL_938945 to 938947, EPI_ISL_938949 to 938950, EPI_ISL_938952 to 938956, EPI_ISL_938958 to 938960, EPI_ISL_938962 to 938973, EPI_ISL_938975, EPI_ISL_938977 to 938987, EPI_ISL_938989 to 938990, EPI_ISL_938992, EPI_ISL_938994 to 938996, EPI_ISL_938998 to 939011, EPI_ISL_939013 to 939014, EPI_ISL_939017 to 939022, EPI_ISL_939024 to 939033, EPI_ISL_939035, EPI_ISL_939037, EPI_ISL_939039 to 939040, EPI_ISL_939042 to 939043, EPI_ISL_939046 to 939049, EPI_ISL_939053, EPI_ISL_939055 to 939057, EPI_ISL_939059 to 939074, EPI_ISL_939076 to 939083, EPI_ISL_939085 to 939092, EPI_ISL_939094 to 939095, EPI_ISL_939097 to 939102, EPI_ISL_939104, EPI_ISL_939106 to 939126, EPI_ISL_939128 to 939131, EPI_ISL_939134 to 939137, EPI_ISL_939139 to 939143, EPI_ISL_939145 to 939146, EPI_ISL_939148 to 939164, EPI_ISL_939167, EPI_ISL_939169 to 939182, EPI_ISL_939184 to 939187, EPI_ISL_939189 to 939194, EPI_ISL_939196 to 939207, EPI_ISL_939209 to 939215, EPI_ISL_939217 to 939227, EPI_ISL_939229 to 939231, EPI_ISL_939233 to 939239, EPI_ISL_939241 to 939246, EPI_ISL_939248 to 939250, EPI_ISL_939252, EPI_ISL_939255, EPI_ISL_939258, EPI_ISL_939261 to 939263, EPI_ISL_939265, EPI_ISL_939267 to 939270, EPI_ISL_939272 to 939274, EPI_ISL_939276 to 939280, EPI_ISL_939286, EPI_ISL_939290 to 939294, EPI_ISL_939296, EPI_ISL_939301 to 939302, EPI_ISL_939307, EPI_ISL_939313, EPI_ISL_939315, EPI_ISL_939318 to 939319, EPI_ISL_939321, EPI_ISL_939329, EPI_ISL_939334, EPI_ISL_939337, EPI_ISL_939339, EPI_ISL_939343, EPI_ISL_939346, EPI_ISL_939348, EPI_ISL_939350 to 939352, EPI_ISL_939355 to 939357, EPI_ISL_939359, EPI_ISL_939365, EPI_ISL_939369 to 939370, EPI_ISL_939372, EPI_ISL_939378, EPI_ISL_939380, EPI_ISL_939388 to 939389, EPI_ISL_939391, EPI_ISL_939393, EPI_ISL_939395 to 939399, EPI_ISL_939401 to 939404, EPI_ISL_939406, EPI_ISL_939413, EPI_ISL_939415 to 939416, EPI_ISL_939420, EPI_ISL_939422, EPI_ISL_939429, EPI_ISL_939431 to 939432, EPI_ISL_939438 to 939439, EPI_ISL_939443, EPI_ISL_939450, EPI_ISL_939458 to 939461, EPI_ISL_939466, EPI_ISL_939468 to 939469, EPI_ISL_939472 to 939473, EPI_ISL_939477 to 939478, EPI_ISL_939481, EPI_ISL_939485, EPI_ISL_939487, EPI_ISL_939492 to 939494, EPI_ISL_939497 to 939498, EPI_ISL_939500, EPI_ISL_939504 to 939508, EPI_ISL_939510, EPI_ISL_939514, EPI_ISL_939519 to 939522, EPI_ISL_939524, EPI_ISL_939526, EPI_ISL_939528, EPI_ISL_939532 to 939533, EPI_ISL_939535 to 939537, EPI_ISL_939542, EPI_ISL_939548, EPI_ISL_939551 to 939552, EPI_ISL_939554, EPI_ISL_939556, EPI_ISL_939561 to 939563, EPI_ISL_939567, EPI_ISL_939571 to 939574, EPI_ISL_939576 to 939579, EPI_ISL_939581 to 939582, EPI_ISL_939585, EPI_ISL_939588, EPI_ISL_939594 to 939595, EPI_ISL_939598, EPI_ISL_939600, EPI_ISL_939603, EPI_ISL_939605 to 939606, EPI_ISL_945050, EPI_ISL_945057 to 945060, EPI_ISL_945062, EPI_ISL_945064, EPI_ISL_945069, EPI_ISL_945071, EPI_ISL_945074, EPI_ISL_945081 to 945082, EPI_ISL_945091 to 945092, EPI_ISL_945094, EPI_ISL_945101, EPI_ISL_945104 to 945105, EPI_ISL_945108, EPI_ISL_945110 to 945111, EPI_ISL_945118, EPI_ISL_945120 to 945121, EPI_ISL_945127 to 945129, EPI_ISL_945132 to 945134, EPI_ISL_945137 to 945138, EPI_ISL_945140 to 945141, EPI_ISL_945149 to 945150, EPI_ISL_945153 to 945154, EPI_ISL_945161, EPI_ISL_945164 to 945165, EPI_ISL_945167 to 945168, EPI_ISL_945171 to 945172, EPI_ISL_945174 to 945175, EPI_ISL_945178 to 945179, EPI_ISL_945182, EPI_ISL_945185, EPI_ISL_945187, EPI_ISL_945193, EPI_ISL_945196, EPI_ISL_945198, EPI_ISL_945200, EPI_ISL_945205 to 945206, EPI_ISL_945210 to 945211, EPI_ISL_945213 to 945216, EPI_ISL_945218 to 945220, EPI_ISL_945225, EPI_ISL_945227, EPI_ISL_945229 to 945230, EPI_ISL_945242, EPI_ISL_945245 to 945246, EPI_ISL_945253, EPI_ISL_945259, EPI_ISL_945262, EPI_ISL_945265, EPI_ISL_945267, EPI_ISL_945269, EPI_ISL_945272, EPI_ISL_945278, EPI_ISL_945280 to 945281, EPI_ISL_945283, EPI_ISL_945286, EPI_ISL_945290 to 945291, EPI_ISL_945293 to 945295, EPI_ISL_945298, EPI_ISL_945300, EPI_ISL_945302, EPI_ISL_945308, EPI_ISL_945313 to 945314, EPI_ISL_945317 to 945318, EPI_ISL_945327 to 945331, EPI_ISL_945333, EPI_ISL_945336, EPI_ISL_945338 to 945340, EPI_ISL_945349, EPI_ISL_945354 to 945355, EPI_ISL_945360, EPI_ISL_945362, EPI_ISL_945368 to 945369, EPI_ISL_945371, EPI_ISL_945373, EPI_ISL_945715, EPI_ISL_945719, EPI_ISL_945721, EPI_ISL_945723 to 945724, EPI_ISL_945727, EPI_ISL_945731, EPI_ISL_945734, EPI_ISL_945739 to 945740, EPI_ISL_945745, EPI_ISL_945748, EPI_ISL_945751, EPI_ISL_945754, EPI_ISL_945756, EPI_ISL_945764, EPI_ISL_945767, EPI_ISL_945769 to 945770, EPI_ISL_945773, EPI_ISL_945775, EPI_ISL_945778 to 945779, EPI_ISL_945784 to 945787, EPI_ISL_945789 to 945790, EPI_ISL_945793 to 945794, EPI_ISL_945801, EPI_ISL_945805, EPI_ISL_945810 to 945811, EPI_ISL_945814, EPI_ISL_945819 to 945820, EPI_ISL_945823, EPI_ISL_945829 to 945830, EPI_ISL_945833, EPI_ISL_945838, EPI_ISL_945840, EPI_ISL_945842, EPI_ISL_945845, EPI_ISL_945847, EPI_ISL_945852, EPI_ISL_945856, EPI_ISL_945864, EPI_ISL_945866, EPI_ISL_945880 to 945881, EPI_ISL_945883, EPI_ISL_945886 to 945887, EPI_ISL_945890 to 945894, EPI_ISL_945896, EPI_ISL_945902, EPI_ISL_945905, EPI_ISL_945912 to 945913, EPI_ISL_945915, EPI_ISL_945918 to 945919, EPI_ISL_945922, EPI_ISL_945924 to 945925, EPI_ISL_945927 to 945930, EPI_ISL_945936 to 945937, EPI_ISL_945941 to 945943, EPI_ISL_945946 to 945949, EPI_ISL_945952, EPI_ISL_945957, EPI_ISL_945959, EPI_ISL_945964, EPI_ISL_945968, EPI_ISL_945974 to 945975, EPI_ISL_945978, EPI_ISL_945982, EPI_ISL_945985 to 945987, EPI_ISL_945989, EPI_ISL_945991 to 945992, EPI_ISL_945995, EPI_ISL_946001, EPI_ISL_946004, EPI_ISL_946012 to 946013, EPI_ISL_946015 to 946017, EPI_ISL_946019, EPI_ISL_946022, EPI_ISL_946024, EPI_ISL_946027, EPI_ISL_946031, EPI_ISL_946033 to 946034, EPI_ISL_946036, EPI_ISL_946038 to 946040, EPI_ISL_946042 to 946046, EPI_ISL_946051, EPI_ISL_946053, EPI_ISL_946056, EPI_ISL_946060 to 946061, EPI_ISL_946063 to 946065, EPI_ISL_946074, EPI_ISL_946077, EPI_ISL_946079, EPI_ISL_946081 to 946083, EPI_ISL_946085, EPI_ISL_946091, EPI_ISL_946098 to 946100, EPI_ISL_946102 to 946105, EPI_ISL_946107 to 946108, EPI_ISL_946111, EPI_ISL_946114, EPI_ISL_946116, EPI_ISL_946118, EPI_ISL_946120, EPI_ISL_946122, EPI_ISL_946125, EPI_ISL_946128, EPI_ISL_946130 to 946134, EPI_ISL_946136, EPI_ISL_946138, EPI_ISL_946140 to 946144, EPI_ISL_946148, EPI_ISL_946151, EPI_ISL_946153 to 946156, EPI_ISL_946158 to 946159, EPI_ISL_946161, EPI_ISL_946163 to 946168, EPI_ISL_946170 to 946172, EPI_ISL_946179, EPI_ISL_946184 to 946185, EPI_ISL_946187, EPI_ISL_946189, EPI_ISL_946191, EPI_ISL_946193, EPI_ISL_946195, EPI_ISL_946197, EPI_ISL_946200, EPI_ISL_946203, EPI_ISL_946205, EPI_ISL_946209, EPI_ISL_946212, EPI_ISL_946216 to 946217, EPI_ISL_946223, EPI_ISL_946226, EPI_ISL_946228, EPI_ISL_946230, EPI_ISL_946235 to 946242, EPI_ISL_946244 to 946249, EPI_ISL_946251 to 946252, EPI_ISL_946255 to 946258, EPI_ISL_946261 to 946263, EPI_ISL_946265 to 946269, EPI_ISL_946273, EPI_ISL_946275 to 946278, EPI_ISL_946280, EPI_ISL_946282 to 946283, EPI_ISL_946291, EPI_ISL_946294, EPI_ISL_946296 to 946297, EPI_ISL_946299, EPI_ISL_946305, EPI_ISL_946307, EPI_ISL_946309 to 946311, EPI_ISL_946313, EPI_ISL_946315 to 946317, EPI_ISL_946320 to 946323, EPI_ISL_946325, EPI_ISL_946327, EPI_ISL_946329 to 946330, EPI_ISL_946332, EPI_ISL_946334, EPI_ISL_946336, EPI_ISL_946341 to 946343, EPI_ISL_946345 to 946347, EPI_ISL_946350, EPI_ISL_946352 to 946355, EPI_ISL_946358 to 946359, EPI_ISL_946362 to 946364, EPI_ISL_946368 to 946370, EPI_ISL_946372, EPI_ISL_946376, EPI_ISL_946383 to 946384, EPI_ISL_946386, EPI_ISL_946388, EPI_ISL_946392 to 946393, EPI_ISL_946396, EPI_ISL_946399, EPI_ISL_946401 to 946402, EPI_ISL_946404, EPI_ISL_946406, EPI_ISL_946409, EPI_ISL_946414 to 946418, EPI_ISL_946420, EPI_ISL_947109 to 947115, EPI_ISL_947117, EPI_ISL_947122, EPI_ISL_947124, EPI_ISL_947128, EPI_ISL_947130, EPI_ISL_947132 to 947133, EPI_ISL_947136, EPI_ISL_947140, EPI_ISL_947142 to 947145, EPI_ISL_947149, EPI_ISL_947151 to 947152, EPI_ISL_947154 to 947162, EPI_ISL_947164, EPI_ISL_947166 to 947168, EPI_ISL_947170, EPI_ISL_947173 to 947178, EPI_ISL_947180, EPI_ISL_947184 to 947185, EPI_ISL_947188 to 947189, EPI_ISL_947192 to 947195, EPI_ISL_947198, EPI_ISL_947200 to 947201, EPI_ISL_947203, EPI_ISL_947205 to 947207, EPI_ISL_947209 to 947210, EPI_ISL_947215 to 947216, EPI_ISL_947218 to 947220, EPI_ISL_947222 to 947224, EPI_ISL_947226 to 947227, EPI_ISL_947231 to 947232, EPI_ISL_947234 to 947235, EPI_ISL_947240, EPI_ISL_947242 to 947244, EPI_ISL_947246, EPI_ISL_947248, EPI_ISL_947251, EPI_ISL_947254, EPI_ISL_947256, EPI_ISL_947258 to 947259, EPI_ISL_947261, EPI_ISL_947264, EPI_ISL_947266, EPI_ISL_947274, EPI_ISL_947276 to 947277, EPI_ISL_947279, EPI_ISL_947282, EPI_ISL_947286, EPI_ISL_947288 to 947289, EPI_ISL_947291 to 947292, EPI_ISL_947299 to 947300, EPI_ISL_947302 to 947303, EPI_ISL_947306, EPI_ISL_947310 to 947311, EPI_ISL_947313, EPI_ISL_947318, EPI_ISL_947321, EPI_ISL_947333 to 947334, EPI_ISL_947336 to 947337, EPI_ISL_947339, EPI_ISL_947342, EPI_ISL_947345 to 947346, EPI_ISL_947348 to 947349, EPI_ISL_947351, EPI_ISL_947355, EPI_ISL_947358, EPI_ISL_947366 to 947368, EPI_ISL_947371 to 947372, EPI_ISL_947374 to 947376, EPI_ISL_947378 to 947379, EPI_ISL_947381, EPI_ISL_947384 to 947385, EPI_ISL_947387, EPI_ISL_947390, EPI_ISL_947392 to 947393, EPI_ISL_947395, EPI_ISL_947400 to 947401, EPI_ISL_947403, EPI_ISL_948327, EPI_ISL_956765 to 956782, EPI_ISL_956784, EPI_ISL_956787 to 956796, EPI_ISL_956798 to 956803, EPI_ISL_956805 to 956808, EPI_ISL_956811 to 956835, EPI_ISL_956837 to 956857, EPI_ISL_956859 to 956861, EPI_ISL_956863 to 956874, EPI_ISL_956876 to 956879, EPI_ISL_956881 to 956887, EPI_ISL_956889 to 956898, EPI_ISL_956900 to 956910, EPI_ISL_956912 to 956917, EPI_ISL_956919 to 956927, EPI_ISL_956929 to 956933, EPI_ISL_956935 to 956938, EPI_ISL_956940 to 956948, EPI_ISL_956950, EPI_ISL_956952 to 956960, EPI_ISL_956963 to 956964, EPI_ISL_956966, EPI_ISL_956968 to 956980, EPI_ISL_956982 to 956997, EPI_ISL_957000 to 957006, EPI_ISL_957009 to 957023, EPI_ISL_957025 to 957030, EPI_ISL_957032 to 957045, EPI_ISL_957047 to 957051, EPI_ISL_957056, EPI_ISL_957058 to 957059, EPI_ISL_957062 to 957064, EPI_ISL_957066, EPI_ISL_957069 to 957070, EPI_ISL_957074, EPI_ISL_957076, EPI_ISL_957085, EPI_ISL_957087 to 957088, EPI_ISL_957098, EPI_ISL_957106, EPI_ISL_957108 to 957109, EPI_ISL_957111 to 957113, EPI_ISL_957116, EPI_ISL_957119 to 957120, EPI_ISL_957123, EPI_ISL_957125 to 957126, EPI_ISL_957128 to 957129, EPI_ISL_957131 to 957132, EPI_ISL_957134 to 957135, EPI_ISL_957137 to 957139, EPI_ISL_957142 to 957148, EPI_ISL_957152 to 957153, EPI_ISL_957159, EPI_ISL_957161, EPI_ISL_957163, EPI_ISL_957167, EPI_ISL_957169, EPI_ISL_957173 to 957174, EPI_ISL_957179, EPI_ISL_957181 to 957182, EPI_ISL_957185 to 957186, EPI_ISL_957189, EPI_ISL_957192 to 957193, EPI_ISL_957195 to 957197, EPI_ISL_957202 to 957204, EPI_ISL_957209, EPI_ISL_957213, EPI_ISL_957217 to 957218, EPI_ISL_957220 to 957222, EPI_ISL_957226, EPI_ISL_957228, EPI_ISL_957230, EPI_ISL_957233 to 957236, EPI_ISL_957238, EPI_ISL_957240 to 957241, EPI_ISL_957244, EPI_ISL_957249, EPI_ISL_957251 to 957252, EPI_ISL_957261, EPI_ISL_957265, EPI_ISL_957267, EPI_ISL_957269, EPI_ISL_957273, EPI_ISL_957277 to 957278, EPI_ISL_957280 to 957282, EPI_ISL_957284, EPI_ISL_957288, EPI_ISL_957290, EPI_ISL_957293, EPI_ISL_957295 to 957296, EPI_ISL_957298, EPI_ISL_957300, EPI_ISL_957303, EPI_ISL_957307, EPI_ISL_957309, EPI_ISL_957313, EPI_ISL_957316 to 957318, EPI_ISL_957321, EPI_ISL_957324 to 957326, EPI_ISL_957329, EPI_ISL_957333, EPI_ISL_957335, EPI_ISL_957338 to 957354, EPI_ISL_957356 to 957359, EPI_ISL_957361 to 957370, EPI_ISL_957372 to 957377, EPI_ISL_957379 to 957385, EPI_ISL_957387 to 957390, EPI_ISL_957392 to 957398, EPI_ISL_957402 to 957403, EPI_ISL_957405 to 957406, EPI_ISL_957408 to 957410, EPI_ISL_957412, EPI_ISL_957414 to 957416, EPI_ISL_957418 to 957419, EPI_ISL_957421 to 957422, EPI_ISL_957424 to 957431, EPI_ISL_957433 to 957452, EPI_ISL_957454 to 957457, EPI_ISL_957460 to 957473, EPI_ISL_957475 to 957477, EPI_ISL_957481 to 957492, EPI_ISL_957494 to 957509, EPI_ISL_957511 to 957515, EPI_ISL_957517 to 957522, EPI_ISL_957524 to 957525, EPI_ISL_957527 to 957535, EPI_ISL_957538 to 957561, EPI_ISL_957563 to 957564, EPI_ISL_957566 to 957575, EPI_ISL_957577 to 957585, EPI_ISL_957587 to 957596, EPI_ISL_957599 to 957606, EPI_ISL_957608 to 957611, EPI_ISL_957614, EPI_ISL_957616, EPI_ISL_957619 to 957621, EPI_ISL_957623 to 957624, EPI_ISL_957628 to 957632, EPI_ISL_957635, EPI_ISL_957641, EPI_ISL_957643, EPI_ISL_957645, EPI_ISL_957650, EPI_ISL_957652, EPI_ISL_957654 to 957655, EPI_ISL_957659 to 957660, EPI_ISL_957662, EPI_ISL_957667 to 957668, EPI_ISL_957671 to 957672, EPI_ISL_957675, EPI_ISL_957677, EPI_ISL_957683 to 957687, EPI_ISL_957690 to 957692, EPI_ISL_957695, EPI_ISL_957698 to 957699, EPI_ISL_957701, EPI_ISL_957705, EPI_ISL_957707, EPI_ISL_957710 to 957712, EPI_ISL_957722 to 957724, EPI_ISL_957726 to 957727, EPI_ISL_957741, EPI_ISL_957744 to 957745, EPI_ISL_957747, EPI_ISL_957759 to 957762, EPI_ISL_957766 to 957767, EPI_ISL_957769, EPI_ISL_957771 to 957772, EPI_ISL_957775, EPI_ISL_957777, EPI_ISL_957780, EPI_ISL_957786 to 957787, EPI_ISL_957795, EPI_ISL_957797, EPI_ISL_957799 to 957801, EPI_ISL_957804, EPI_ISL_957810, EPI_ISL_957813, EPI_ISL_957816, EPI_ISL_957822 to 957823, EPI_ISL_957826 to 957830, EPI_ISL_957832, EPI_ISL_957835, EPI_ISL_957838, EPI_ISL_957841, EPI_ISL_957843 to 957844, EPI_ISL_957849, EPI_ISL_957856, EPI_ISL_957858 to 957859, EPI_ISL_957862 to 957863, EPI_ISL_957866 to 957867, EPI_ISL_957872, EPI_ISL_957877 to 957878, EPI_ISL_957885 to 957886, EPI_ISL_957888, EPI_ISL_957892 to 957893, EPI_ISL_957897, EPI_ISL_957899 to 957901, EPI_ISL_957904, EPI_ISL_957907, EPI_ISL_957909 to 957921, EPI_ISL_957923 to 957927, EPI_ISL_957930, EPI_ISL_957932 to 957946, EPI_ISL_957948 to 957961, EPI_ISL_957964 to 957965, EPI_ISL_957967 to 957972, EPI_ISL_957974 to 957988, EPI_ISL_957990 to 958009, EPI_ISL_958011 to 958016, EPI_ISL_958018, EPI_ISL_958020 to 958031, EPI_ISL_958033, EPI_ISL_958035 to 958046, EPI_ISL_958048 to 958051, EPI_ISL_958055 to 958063, EPI_ISL_958065 to 958074, EPI_ISL_958076 to 958079, EPI_ISL_958081 to 958090, EPI_ISL_958092, EPI_ISL_958094 to 958095, EPI_ISL_958097 to 958099, EPI_ISL_958101, EPI_ISL_958103, EPI_ISL_958105 to 958108, EPI_ISL_958110 to 958118, EPI_ISL_958120 to 958122, EPI_ISL_958124, EPI_ISL_958126 to 958139, EPI_ISL_958141 to 958156, EPI_ISL_958158 to 958165, EPI_ISL_958168 to 958173, EPI_ISL_958175 to 958180, EPI_ISL_958182 to 958185, EPI_ISL_958188 to 958196, EPI_ISL_958198 to 958200, EPI_ISL_958202 to 958205, EPI_ISL_958207 to 958213, EPI_ISL_958215, EPI_ISL_958217 to 958223, EPI_ISL_958225, EPI_ISL_958227 to 958248, EPI_ISL_958250, EPI_ISL_958252 to 958253, EPI_ISL_958255 to 958258, EPI_ISL_958260 to 958276, EPI_ISL_958278 to 958287, EPI_ISL_958289 to 958296, EPI_ISL_958298 to 958307, EPI_ISL_958309 to 958331, EPI_ISL_958333 to 958334, EPI_ISL_958336 to 958341, EPI_ISL_958343 to 958346, EPI_ISL_958348 to 958361, EPI_ISL_958363, EPI_ISL_958365 to 958379, EPI_ISL_958381 to 958394, EPI_ISL_958396 to 958398, EPI_ISL_958400 to 958402, EPI_ISL_958404 to 958412, EPI_ISL_958414, EPI_ISL_958416, EPI_ISL_958418 to 958420, EPI_ISL_958422 to 958445, EPI_ISL_958447 to 958462, EPI_ISL_958464 to 958465, EPI_ISL_958467 to 958474, EPI_ISL_958476 to 958484, EPI_ISL_958486 to 958488, EPI_ISL_958490 to 958499, EPI_ISL_958501 to 958508, EPI_ISL_958510 to 958514, EPI_ISL_958517 to 958522, EPI_ISL_958524 to 958539, EPI_ISL_958541, EPI_ISL_958543 to 958553, EPI_ISL_958556 to 958565, EPI_ISL_958567 to 958569, EPI_ISL_958571, EPI_ISL_958573 to 958576, EPI_ISL_958579 to 958580, EPI_ISL_958582 to 958583, EPI_ISL_958586 to 958595, EPI_ISL_958597 to 958599, EPI_ISL_958602 to 958613, EPI_ISL_958615 to 958616,

EPI_ISL_958619 to 958624, EPI_ISL_958626, EPI_ISL_958628 to 958633, EPI_ISL_958637 to 958644, EPI_ISL_958646, EPI_ISL_958648 to 958652, EPI_ISL_958654 to 958655, EPI_ISL_958657 to 958658, EPI_ISL_958660 to 958665, EPI_ISL_958667 to 958671, EPI_ISL_958674 to 958676, EPI_ISL_958678 to 958693, EPI_ISL_958695 to 958705, EPI_ISL_958707 to 958713, EPI_ISL_958715 to 958720, EPI_ISL_958722 to 958728, EPI_ISL_958730 to 958740, EPI_ISL_958742 to 958745, EPI_ISL_958747, EPI_ISL_958749, EPI_ISL_958752, EPI_ISL_958754 to 958761, EPI_ISL_958763, EPI_ISL_958766 to 958777, EPI_ISL_958780 to 958781, EPI_ISL_958783 to 958784, EPI_ISL_958786 to 958794, EPI_ISL_958796 to 958807, EPI_ISL_958811 to 958812, EPI_ISL_958814 to 958824, EPI_ISL_958826 to 958828, EPI_ISL_958830 to 958844, EPI_ISL_958846, EPI_ISL_958848 to 958851, EPI_ISL_958853, EPI_ISL_958858 to 958861, EPI_ISL_958863 to 958868, EPI_ISL_958870, EPI_ISL_958872 to 958873, EPI_ISL_958875, EPI_ISL_958877 to 958878, EPI_ISL_958880, EPI_ISL_958882, EPI_ISL_958886 to 958892, EPI_ISL_958894 to 958896, EPI_ISL_958898 to 958901, EPI_ISL_958903 to 958908, EPI_ISL_958912 to 958916, EPI_ISL_959271 to 959273, EPI_ISL_963324 to 963325, EPI_ISL_963327 to 963329, EPI_ISL_963331 to 963340, EPI_ISL_963342 to 963343, EPI_ISL_963347, EPI_ISL_963350 to 963354, EPI_ISL_963359 to 963362, EPI_ISL_963364, EPI_ISL_963366 to 963369, EPI_ISL_963372 to 963375, EPI_ISL_963377 to 963378, EPI_ISL_963382, EPI_ISL_963384 to 963385, EPI_ISL_963387 to 963388, EPI_ISL_963390 to 963394, EPI_ISL_963396, EPI_ISL_963398 to 963405, EPI_ISL_963407 to 963413, EPI_ISL_963415 to 963418, EPI_ISL_963420, EPI_ISL_963422 to 963424, EPI_ISL_963428 to 963430, EPI_ISL_963432, EPI_ISL_963435 to 963436, EPI_ISL_963438 to 963448, EPI_ISL_963450 to 963451, EPI_ISL_963453 to 963457, EPI_ISL_963460 to 963472, EPI_ISL_963474, EPI_ISL_963476, EPI_ISL_963478 to 963480, EPI_ISL_963482 to 963484, EPI_ISL_963486 to 963488, EPI_ISL_963492 to 963494, EPI_ISL_963497 to 963503, EPI_ISL_963505 to 963507, EPI_ISL_963510, EPI_ISL_963512 to 963513, EPI_ISL_963516 to 963518, EPI_ISL_963522 to 963528, EPI_ISL_963531 to 963537, EPI_ISL_963539 to 963549, EPI_ISL_963551 to 963553, EPI_ISL_963555 to 963558, EPI_ISL_963561, EPI_ISL_963565 to 963569, EPI_ISL_963571 to 963572, EPI_ISL_963579 to 963580, EPI_ISL_963583, EPI_ISL_963585, EPI_ISL_963587 to 963590, EPI_ISL_963592 to 963593, EPI_ISL_963595 to 963598, EPI_ISL_963600, EPI_ISL_963602, EPI_ISL_963604 to 963606, EPI_ISL_963608 to 963610, EPI_ISL_963612, EPI_ISL_963614 to 963615, EPI_ISL_963617, EPI_ISL_963621 to 963622, EPI_ISL_963624 to 963625, EPI_ISL_963627 to 963632, EPI_ISL_963636 to 963638, EPI_ISL_963640, EPI_ISL_963642, EPI_ISL_963644 to 963648, EPI_ISL_964346, EPI_ISL_964350, EPI_ISL_964353 to 964354, EPI_ISL_964363, EPI_ISL_964365, EPI_ISL_964370, EPI_ISL_964373 to 964374, EPI_ISL_964379, EPI_ISL_964381, EPI_ISL_964383, EPI_ISL_964385 to 964386, EPI_ISL_964389, EPI_ISL_964392, EPI_ISL_964395, EPI_ISL_964398, EPI_ISL_964400, EPI_ISL_964402 to 964403, EPI_ISL_964406, EPI_ISL_964412, EPI_ISL_964414, EPI_ISL_964419, EPI_ISL_964423, EPI_ISL_964426, EPI_ISL_964428, EPI_ISL_964431 to 964432, EPI_ISL_964435, EPI_ISL_964438, EPI_ISL_964443 to 964444, EPI_ISL_964447 to 964448, EPI_ISL_964453, EPI_ISL_964455, EPI_ISL_964461, EPI_ISL_964466 to 964467, EPI_ISL_964478 to 964479, EPI_ISL_964481, EPI_ISL_964486, EPI_ISL_964491 to 964492, EPI_ISL_964495 to 964499, EPI_ISL_964505 to 964507, EPI_ISL_964510, EPI_ISL_964513, EPI_ISL_964515, EPI_ISL_964517, EPI_ISL_964519, EPI_ISL_964522, EPI_ISL_964524, EPI_ISL_964529 to 964531, EPI_ISL_964534 to 964536, EPI_ISL_964538, EPI_ISL_970587, EPI_ISL_970591, EPI_ISL_970593, EPI_ISL_970595, EPI_ISL_970597, EPI_ISL_970599, EPI_ISL_970601 to 970602, EPI_ISL_970605, EPI_ISL_970607, EPI_ISL_970609, EPI_ISL_970612, EPI_ISL_970614, EPI_ISL_970618 to 970619, EPI_ISL_970621, EPI_ISL_970623, EPI_ISL_970628, EPI_ISL_970630, EPI_ISL_970632 to 970633, EPI_ISL_970637, EPI_ISL_970639, EPI_ISL_970641, EPI_ISL_970643, EPI_ISL_970645, EPI_ISL_970648, EPI_ISL_970650, EPI_ISL_970652, EPI_ISL_970654, EPI_ISL_970656, EPI_ISL_970658, EPI_ISL_970660 to 970661, EPI_ISL_970664, EPI_ISL_970666, EPI_ISL_970668, EPI_ISL_970670, EPI_ISL_970673, EPI_ISL_970675, EPI_ISL_970677, EPI_ISL_970680 to 970681, EPI_ISL_970683, EPI_ISL_970685, EPI_ISL_970687, EPI_ISL_970689 to 970690, EPI_ISL_970692, EPI_ISL_970694, EPI_ISL_970697, EPI_ISL_970699, EPI_ISL_970701, EPI_ISL_970704, EPI_ISL_970706, EPI_ISL_970708 to 970709, EPI_ISL_970711, EPI_ISL_970713, EPI_ISL_970715, EPI_ISL_970717, EPI_ISL_970720, EPI_ISL_970722, EPI_ISL_970724, EPI_ISL_970726 to 970727, EPI_ISL_970729, EPI_ISL_970732, EPI_ISL_970734, EPI_ISL_970736, EPI_ISL_970738 to 970739, EPI_ISL_970741, EPI_ISL_970746 to 970747, EPI_ISL_970749, EPI_ISL_970751, EPI_ISL_970753, EPI_ISL_970755, EPI_ISL_970757 to 970758, EPI_ISL_970760, EPI_ISL_970762, EPI_ISL_970764, EPI_ISL_970770, EPI_ISL_970772, EPI_ISL_970774 to 970775, EPI_ISL_970777, EPI_ISL_970779, EPI_ISL_970781 to 970782, EPI_ISL_970784, EPI_ISL_970786, EPI_ISL_970788, EPI_ISL_970790, EPI_ISL_970794, EPI_ISL_970796 to 970797, EPI_ISL_970800, EPI_ISL_970802 to 970803, EPI_ISL_970805, EPI_ISL_970807 to 970808, EPI_ISL_970826, EPI_ISL_970845, EPI_ISL_970860, EPI_ISL_970875, EPI_ISL_970899, EPI_ISL_970925, EPI_ISL_970944, EPI_ISL_970968, EPI_ISL_970986, EPI_ISL_971008, EPI_ISL_971023, EPI_ISL_971037, EPI_ISL_971039, EPI_ISL_971042, EPI_ISL_971044, EPI_ISL_971050, EPI_ISL_971055, EPI_ISL_971059 to 971060, EPI_ISL_971063, EPI_ISL_971066, EPI_ISL_971069, EPI_ISL_971074, EPI_ISL_971076, EPI_ISL_971079, EPI_ISL_971083, EPI_ISL_971087, EPI_ISL_971090, EPI_ISL_971093, EPI_ISL_971096, EPI_ISL_971099, EPI_ISL_971102, EPI_ISL_971105, EPI_ISL_971108 to 971109, EPI_ISL_971117, EPI_ISL_971119, EPI_ISL_971123, EPI_ISL_971125, EPI_ISL_971128, EPI_ISL_971130, EPI_ISL_971134 to 971135, EPI_ISL_971142, EPI_ISL_971146, EPI_ISL_971149, EPI_ISL_971153, EPI_ISL_971156, EPI_ISL_971161, EPI_ISL_971164 to 971165, EPI_ISL_971168, EPI_ISL_971171, EPI_ISL_971174, EPI_ISL_971176, EPI_ISL_971180, EPI_ISL_971185, EPI_ISL_971190 to 971191, EPI_ISL_971197, EPI_ISL_971200, EPI_ISL_971204, EPI_ISL_971206, EPI_ISL_971209, EPI_ISL_971211, EPI_ISL_971215, EPI_ISL_971218, EPI_ISL_971221, EPI_ISL_971228, EPI_ISL_971231, EPI_ISL_971234, EPI_ISL_971236, EPI_ISL_971239, EPI_ISL_971242, EPI_ISL_971246, EPI_ISL_971248, EPI_ISL_971251, EPI_ISL_971254 to 971255, EPI_ISL_971258, EPI_ISL_971261, EPI_ISL_971264, EPI_ISL_971273, EPI_ISL_971277, EPI_ISL_971284, EPI_ISL_971286, EPI_ISL_971289, EPI_ISL_971297, EPI_ISL_971301, EPI_ISL_971304, EPI_ISL_971307, EPI_ISL_971310, EPI_ISL_971316, EPI_ISL_971320, EPI_ISL_971324, EPI_ISL_971327, EPI_ISL_971330, EPI_ISL_971333, EPI_ISL_971336, EPI_ISL_971339, EPI_ISL_971343, EPI_ISL_971347, EPI_ISL_971350, EPI_ISL_971352, EPI_ISL_971357, EPI_ISL_971360, EPI_ISL_971364 to 971365, EPI_ISL_971370, EPI_ISL_971373, EPI_ISL_971376, EPI_ISL_971378, EPI_ISL_971382, EPI_ISL_971385, EPI_ISL_971394, EPI_ISL_971400, EPI_ISL_971402, EPI_ISL_971405, EPI_ISL_971407, EPI_ISL_971410, EPI_ISL_971413, EPI_ISL_971417, EPI_ISL_971423, EPI_ISL_971428, EPI_ISL_979974, EPI_ISL_979976 to 979982, EPI_ISL_979984 to 979985, EPI_ISL_979987 to 979991, EPI_ISL_979993 to 979998, EPI_ISL_980000 to 980005, EPI_ISL_980007 to 980023, EPI_ISL_980025, EPI_ISL_980027 to 980029, EPI_ISL_980032 to 980039, EPI_ISL_980041 to 980046, EPI_ISL_980048 to 980052, EPI_ISL_980054 to 980056, EPI_ISL_980058 to 980061, EPI_ISL_980063 to 980094, EPI_ISL_980096, EPI_ISL_980098 to 980099, EPI_ISL_980101 to 980102, EPI_ISL_980104, EPI_ISL_980106 to 980108, EPI_ISL_980110 to 980121, EPI_ISL_980123 to 980135, EPI_ISL_980137 to 980139, EPI_ISL_980141 to 980148, EPI_ISL_980151 to 980154, EPI_ISL_980156, EPI_ISL_980158 to 980162, EPI_ISL_980164 to 980167, EPI_ISL_980169 to 980175, EPI_ISL_980179 to 980180, EPI_ISL_980182 to 980199, EPI_ISL_980201 to 980233, EPI_ISL_980551 to 980554, EPI_ISL_980556 to 980558, EPI_ISL_980560 to 980567, EPI_ISL_980569 to 980573, EPI_ISL_980576 to 980582, EPI_ISL_980586 to 980590, EPI_ISL_980592 to 980602, EPI_ISL_980604 to 980605, EPI_ISL_980607 to 980616, EPI_ISL_980618 to 980625, EPI_ISL_980627 to 980628, EPI_ISL_980630, EPI_ISL_980632 to 980648, EPI_ISL_980650 to 980656, EPI_ISL_980659 to 980660, EPI_ISL_980662, EPI_ISL_980664 to 980676, EPI_ISL_980679 to 980685, EPI_ISL_980687 to 980689, EPI_ISL_980691 to 980712, EPI_ISL_980714 to 980715, EPI_ISL_980717 to 980718, EPI_ISL_980720 to 980721, EPI_ISL_980725 to 980728, EPI_ISL_980730 to 980732, EPI_ISL_980735 to 980736, EPI_ISL_980738, EPI_ISL_980740 to 980747, EPI_ISL_980749 to 980751, EPI_ISL_980753 to 980756, EPI_ISL_980758 to 980771, EPI_ISL_980773, EPI_ISL_980775 to 980788, EPI_ISL_980790 to 980791, EPI_ISL_980794 to 980806, EPI_ISL_985946 to 985964, EPI_ISL_985967 to 985970, EPI_ISL_985972 to 985975, EPI_ISL_985978 to 985981, EPI_ISL_985983, EPI_ISL_985985, EPI_ISL_985987 to 985995, EPI_ISL_985997 to 986002, EPI_ISL_986004 to 986014, EPI_ISL_986016 to 986025, EPI_ISL_986027 to 986037, EPI_ISL_986039 to 986054, EPI_ISL_986056 to 986058, EPI_ISL_986061 to 986062, EPI_ISL_986064 to 986072, EPI_ISL_986074 to 986075, EPI_ISL_986077 to 986078, EPI_ISL_986080 to 986087, EPI_ISL_986089, EPI_ISL_986091 to 986097, EPI_ISL_986099 to 986103, EPI_ISL_986105, EPI_ISL_986107 to 986108, EPI_ISL_986110, EPI_ISL_986113, EPI_ISL_986121, EPI_ISL_986123 to 986124, EPI_ISL_986127, EPI_ISL_986130 to 986131, EPI_ISL_986135 to 986138, EPI_ISL_986140 to 986143, EPI_ISL_986145 to 986146, EPI_ISL_986148 to 986151, EPI_ISL_986155, EPI_ISL_986158, EPI_ISL_986161, EPI_ISL_986164, EPI_ISL_986166, EPI_ISL_986168, EPI_ISL_986170, EPI_ISL_986173, EPI_ISL_986176 to 986177, EPI_ISL_986179, EPI_ISL_986186 to 986188, EPI_ISL_986192 to 986196, EPI_ISL_986198, EPI_ISL_986202 to 986203, EPI_ISL_986206 to 986207, EPI_ISL_986210, EPI_ISL_986214, EPI_ISL_986216, EPI_ISL_986219 to 986221, EPI_ISL_986223 to 986228, EPI_ISL_986231 to 986233, EPI_ISL_986235, EPI_ISL_986237, EPI_ISL_986240, EPI_ISL_986242, EPI_ISL_986245 to 986247, EPI_ISL_986251 to 986253, EPI_ISL_986256, EPI_ISL_986258 to 986259, EPI_ISL_986264, EPI_ISL_986269, EPI_ISL_987284 to 987286, EPI_ISL_987298, EPI_ISL_987305, EPI_ISL_987329, EPI_ISL_987331, EPI_ISL_987334, EPI_ISL_987338, EPI_ISL_987342 to 987343, EPI_ISL_987346, EPI_ISL_987351 to 987353, EPI_ISL_987356, EPI_ISL_987358, EPI_ISL_987364, EPI_ISL_987366, EPI_ISL_987371, EPI_ISL_987377, EPI_ISL_987396, EPI_ISL_987403, EPI_ISL_987434, EPI_ISL_987442, EPI_ISL_987447, EPI_ISL_987459, EPI_ISL_987466, EPI_ISL_987473, EPI_ISL_987487, EPI_ISL_987492, EPI_ISL_987495, EPI_ISL_987504, EPI_ISL_987515 to 987517, EPI_ISL_987522, EPI_ISL_987528, EPI_ISL_987531, EPI_ISL_987533, EPI_ISL_987537 to 987539, EPI_ISL_987543, EPI_ISL_987548, EPI_ISL_987551, EPI_ISL_987558, EPI_ISL_987563 to 987565, EPI_ISL_987569, EPI_ISL_987580, EPI_ISL_987582, EPI_ISL_987585 to 987586, EPI_ISL_987588, EPI_ISL_987592 to 987593, EPI_ISL_987601, EPI_ISL_987608, EPI_ISL_987618, EPI_ISL_987626, EPI_ISL_987630, EPI_ISL_987632, EPI_ISL_987634 to 987636, EPI_ISL_988205, EPI_ISL_988207, EPI_ISL_988209, EPI_ISL_988217 to 988218, EPI_ISL_988222 to 988225, EPI_ISL_988229, EPI_ISL_988235 to 988236, EPI_ISL_988238 to 988239, EPI_ISL_988241 to 988243, EPI_ISL_988248 to 988250, EPI_ISL_988254, EPI_ISL_988256, EPI_ISL_988261 to 988263, EPI_ISL_988270 to 988271, EPI_ISL_988276, EPI_ISL_988286, EPI_ISL_988290, EPI_ISL_988296, EPI_ISL_988299, EPI_ISL_988305 to 988310, EPI_ISL_988312 to 988314, EPI_ISL_988317, EPI_ISL_988321, EPI_ISL_988325, EPI_ISL_988329, EPI_ISL_988332, EPI_ISL_988335, EPI_ISL_988337, EPI_ISL_988347, EPI_ISL_988351, EPI_ISL_988357 to 988358, EPI_ISL_988362 to 988364, EPI_ISL_988370, EPI_ISL_988377 to 988378, EPI_ISL_988384 to 988385, EPI_ISL_988389, EPI_ISL_988391, EPI_ISL_988395, EPI_ISL_988398 to 988399, EPI_ISL_988403 to 988404, EPI_ISL_988417 to 988418, EPI_ISL_988423, EPI_ISL_988426 to 988427, EPI_ISL_988430, EPI_ISL_988434, EPI_ISL_988436, EPI_ISL_988446, EPI_ISL_988448 to 988449, EPI_ISL_988452 to 988453, EPI_ISL_988459 to 988460, EPI_ISL_988463, EPI_ISL_988468, EPI_ISL_988480, EPI_ISL_988483 to 988501, EPI_ISL_988503 to 988521, EPI_ISL_988524 to 988526, EPI_ISL_988528 to 988531, EPI_ISL_988533 to 988539, EPI_ISL_988541 to 988545, EPI_ISL_988548 to 988552, EPI_ISL_988554 to 988559, EPI_ISL_988561 to 988562, EPI_ISL_988564, EPI_ISL_988567 to 988575, EPI_ISL_988578 to 988580, EPI_ISL_988582, EPI_ISL_988584 to 988589, EPI_ISL_988592 to 988599, EPI_ISL_988601 to 988603, EPI_ISL_988605 to 988610, EPI_ISL_988612 to 988618, EPI_ISL_988620, EPI_ISL_988622 to 988623, EPI_ISL_988625 to 988634, EPI_ISL_988638 to 988640, EPI_ISL_988642 to 988645, EPI_ISL_988647 to 988651, EPI_ISL_988653, EPI_ISL_988655, EPI_ISL_988657, EPI_ISL_988660 to 988666, EPI_ISL_988668, EPI_ISL_988670, EPI_ISL_988672 to 988675, EPI_ISL_988678 to 988684, EPI_ISL_989362, EPI_ISL_989831, EPI_ISL_991351, EPI_ISL_991358 to 991359, EPI_ISL_991367 to 991368, EPI_ISL_991377, EPI_ISL_991379, EPI_ISL_991381 to 991383, EPI_ISL_991385 to 991387, EPI_ISL_991395, EPI_ISL_991397, EPI_ISL_991399, EPI_ISL_991403, EPI_ISL_991409, EPI_ISL_991417, EPI_ISL_991419, EPI_ISL_991422, EPI_ISL_991428, EPI_ISL_991432, EPI_ISL_991434 to 991435, EPI_ISL_991437, EPI_ISL_991443, EPI_ISL_991446, EPI_ISL_991450 to 991451, EPI_ISL_991453, EPI_ISL_991455, EPI_ISL_991458, EPI_ISL_991460, EPI_ISL_991464 to 991465, EPI_ISL_991467, EPI_ISL_991470, EPI_ISL_991476, EPI_ISL_991478, EPI_ISL_991482, EPI_ISL_991486, EPI_ISL_991489, EPI_ISL_991491, EPI_ISL_991493 to 991494, EPI_ISL_991496, EPI_ISL_991498 to 991499, EPI_ISL_991502 to 991503, EPI_ISL_991506, EPI_ISL_991515, EPI_ISL_991517, EPI_ISL_991525, EPI_ISL_991527 to 991528, EPI_ISL_991530 to 991532, EPI_ISL_991534, EPI_ISL_991543 to 991544, EPI_ISL_991546, EPI_ISL_991548, EPI_ISL_991551, EPI_ISL_991553, EPI_ISL_991556, EPI_ISL_991558, EPI_ISL_991561, EPI_ISL_993083, EPI_ISL_993086, EPI_ISL_993088 to 993089, EPI_ISL_993092 to 993093, EPI_ISL_993099, EPI_ISL_993101 to 993104, EPI_ISL_993107, EPI_ISL_993112, EPI_ISL_993117 to 993118, EPI_ISL_993120 to 993121, EPI_ISL_993124 to 993125, EPI_ISL_993128 to 993132, EPI_ISL_993134 to 993136, EPI_ISL_993138, EPI_ISL_993141, EPI_ISL_993143 to 993145, EPI_ISL_993147 to 993148, EPI_ISL_993150, EPI_ISL_993156, EPI_ISL_993158 to 993160, EPI_ISL_993163 to 993164, EPI_ISL_993166 to 993167, EPI_ISL_993169, EPI_ISL_993172, EPI_ISL_993177 to 993183, EPI_ISL_993185, EPI_ISL_993188 to 993193, EPI_ISL_993195, EPI_ISL_993197, EPI_ISL_993200, EPI_ISL_993203, EPI_ISL_993207, EPI_ISL_993210, EPI_ISL_993212, EPI_ISL_993215, EPI_ISL_993218 to 993219, EPI_ISL_993223, EPI_ISL_993226, EPI_ISL_993228, EPI_ISL_993230, EPI_ISL_993232 to 993233, EPI_ISL_993235 to 993236, EPI_ISL_993238, EPI_ISL_993240, EPI_ISL_993246 to 993247, EPI_ISL_993249 to 993254, EPI_ISL_993259 to 993262, EPI_ISL_993264 to 993265, EPI_ISL_993267, EPI_ISL_993270, EPI_ISL_993272 to 993281, EPI_ISL_993283, EPI_ISL_993285 to 993290, EPI_ISL_993293, EPI_ISL_993295 to 993296, EPI_ISL_993298, EPI_ISL_993300 to 993301, EPI_ISL_993303, EPI_ISL_993305, EPI_ISL_993308, EPI_ISL_993310, EPI_ISL_993312, EPI_ISL_993314 to 993315, EPI_ISL_993318 to 993320, EPI_ISL_993323 to 993324, EPI_ISL_993332, EPI_ISL_993335 to 993336, EPI_ISL_993339 to 993340, EPI_ISL_993342 to 993346, EPI_ISL_993349 to 993352, EPI_ISL_993354 to 993355, EPI_ISL_993357 to 993360, EPI_ISL_993363, EPI_ISL_993365 to 993367, EPI_ISL_993372 to 993374, EPI_ISL_993380, EPI_ISL_993382, EPI_ISL_993384, EPI_ISL_993388, EPI_ISL_993392, EPI_ISL_993394 to 993396, EPI_ISL_993413 to 993414, EPI_ISL_993416, EPI_ISL_993419, EPI_ISL_993421 to 993423, EPI_ISL_993426, EPI_ISL_993428 to 993429, EPI_ISL_993432 to 993434, EPI_ISL_993436, EPI_ISL_993438, EPI_ISL_993441 to 993447, EPI_ISL_993450 to 993452, EPI_ISL_993454, EPI_ISL_993456, EPI_ISL_993458 to 993459, EPI_ISL_993461, EPI_ISL_993463 to 993464, EPI_ISL_993467 to 993468, EPI_ISL_993470 to 993472, EPI_ISL_993474 to 993476, EPI_ISL_993478 to 993480, EPI_ISL_993483 to 993484, EPI_ISL_993486, EPI_ISL_993489, EPI_ISL_993491, EPI_ISL_993494 to 993495, EPI_ISL_993498 to 993499, EPI_ISL_993502 to 993503, EPI_ISL_993509 to 993512, EPI_ISL_993514 to 993518, EPI_ISL_993520 to 993521, EPI_ISL_993524 to 993527, EPI_ISL_993529 to 993530, EPI_ISL_993533 to 993537, EPI_ISL_993539 to 993541, EPI_ISL_993545, EPI_ISL_993547 to 993548, EPI_ISL_993551 to 993553, EPI_ISL_993556 to 993559, EPI_ISL_993561, EPI_ISL_993563 to 993567, EPI_ISL_993569, EPI_ISL_993572, EPI_ISL_993574 to 993580, EPI_ISL_993585 to 993586, EPI_ISL_993588, EPI_ISL_993590 to 993592, EPI_ISL_993594, EPI_ISL_993597, EPI_ISL_993603 to 993604, EPI_ISL_993606 to 993608, EPI_ISL_993610 to 993611, EPI_ISL_993614 to 993615, EPI_ISL_993618, EPI_ISL_993622 to 993627, EPI_ISL_993629, EPI_ISL_993635 to 993637, EPI_ISL_993642 to 993643, EPI_ISL_993645, EPI_ISL_993647, EPI_ISL_993650 to 993653, EPI_ISL_993657 to 993659, EPI_ISL_993662 to 993668, EPI_ISL_993672 to 993675, EPI_ISL_993677 to 993679, EPI_ISL_993681, EPI_ISL_993685 to 993687, EPI_ISL_993689, EPI_ISL_993691, EPI_ISL_993693 to 993696, EPI_ISL_993699 to 993703, EPI_ISL_993705, EPI_ISL_993707, EPI_ISL_993710 to 993712, EPI_ISL_993714 to 993716, EPI_ISL_993719, EPI_ISL_993723 to 993726, EPI_ISL_993728 to 993732, EPI_ISL_993734 to 993735, EPI_ISL_993748, EPI_ISL_993751 to 993753, EPI_ISL_993755, EPI_ISL_993759 to 993760, EPI_ISL_993763, EPI_ISL_993765, EPI_ISL_993767, EPI_ISL_993769, EPI_ISL_993772 to 993773, EPI_ISL_993775, EPI_ISL_993777, EPI_ISL_993781, EPI_ISL_993783, EPI_ISL_993785 to 993790, EPI_ISL_993796, EPI_ISL_993799, EPI_ISL_993801, EPI_ISL_993806, EPI_ISL_993812, EPI_ISL_993820, EPI_ISL_993822, EPI_ISL_993826, EPI_ISL_993828, EPI_ISL_993831, EPI_ISL_993834 to 993835, EPI_ISL_993839, EPI_ISL_993841 to 993842, EPI_ISL_993844 to 993845, EPI_ISL_993848, EPI_ISL_993851 to 993852, EPI_ISL_993857 to 993858, EPI_ISL_993860 to 993861, EPI_ISL_993875, EPI_ISL_993877, EPI_ISL_993880, EPI_ISL_993882 to 993884, EPI_ISL_993886, EPI_ISL_993888 to 993889, EPI_ISL_993896 to 993899, EPI_ISL_993901 to 993902, EPI_ISL_993904 to 993905, EPI_ISL_993908, EPI_ISL_993910, EPI_ISL_993913, EPI_ISL_993917 to 993918, EPI_ISL_993924, EPI_ISL_993928, EPI_ISL_993931 to 993936, EPI_ISL_993938 to 993941, EPI_ISL_993943 to 993944, EPI_ISL_993947 to 993948, EPI_ISL_993950, EPI_ISL_993952, EPI_ISL_993959, EPI_ISL_993964 to 993965, EPI_ISL_993970 to 993971, EPI_ISL_993974, EPI_ISL_993978, EPI_ISL_993981, EPI_ISL_993985 to 993986, EPI_ISL_993988 to 993991, EPI_ISL_993998 to 993999, EPI_ISL_994002, EPI_ISL_994005, EPI_ISL_994012 to 994013, EPI_ISL_994016 to 994017, EPI_ISL_994021, EPI_ISL_994023, EPI_ISL_994026, EPI_ISL_994034, EPI_ISL_994036 to 994037, EPI_ISL_994039, EPI_ISL_994043, EPI_ISL_994047, EPI_ISL_994049, EPI_ISL_994051, EPI_ISL_994056 to 994057, EPI_ISL_994062 to 994063, EPI_ISL_994065, EPI_ISL_994652, EPI_ISL_1005718, EPI_ISL_1005727, EPI_ISL_1005731, EPI_ISL_1005741 to 1005743, EPI_ISL_1005746, EPI_ISL_1005753, EPI_ISL_1005760, EPI_ISL_1005765, EPI_ISL_1005774, EPI_ISL_1005778, EPI_ISL_1005785, EPI_ISL_1005799, EPI_ISL_1005804, EPI_ISL_1005807, EPI_ISL_1005809, EPI_ISL_1005817, EPI_ISL_1005831, EPI_ISL_1005836, EPI_ISL_1005838, EPI_ISL_1005841, EPI_ISL_1005862, EPI_ISL_1005873 to 1005874, EPI_ISL_1005883, EPI_ISL_1005885, EPI_ISL_1005889, EPI_ISL_1005892, EPI_ISL_1005903, EPI_ISL_1005911 to 1005912, EPI_ISL_1005914, EPI_ISL_1005927, EPI_ISL_1005935, EPI_ISL_1005940, EPI_ISL_1005951 to 1005952, EPI_ISL_1005957 to 1005958, EPI_ISL_1005961 to 1005963, EPI_ISL_1005969 to 1005979, EPI_ISL_1005982 to 1005985, EPI_ISL_1005987 to 1005988, EPI_ISL_1005990 to 1005994, EPI_ISL_1005999, EPI_ISL_1006001 to 1006003, EPI_ISL_1006005, EPI_ISL_1006007 to 1006008, EPI_ISL_1006010 to 1006014, EPI_ISL_1006016 to 1006017, EPI_ISL_1006019, EPI_ISL_1006021 to 1006024, EPI_ISL_1006026, EPI_ISL_1006028 to 1006029, EPI_ISL_1006031 to 1006034, EPI_ISL_1006036, EPI_ISL_1006038 to 1006041, EPI_ISL_1006043 to 1006045, EPI_ISL_1006048 to 1006051, EPI_ISL_1006053 to 1006056, EPI_ISL_1006060 to 1006061, EPI_ISL_1006063, EPI_ISL_1006065, EPI_ISL_1006067 to 1006069, EPI_ISL_1006074 to 1006076, EPI_ISL_1006078 to 1006079, EPI_ISL_1006081 to 1006082, EPI_ISL_1006085 to 1006086, EPI_ISL_1006088 to 1006089, EPI_ISL_1006091, EPI_ISL_1006093, EPI_ISL_1006095, EPI_ISL_1006097, EPI_ISL_1006099 to 1006101, EPI_ISL_1006103, EPI_ISL_1006105, EPI_ISL_1006107, EPI_ISL_1006110 to 1006111, EPI_ISL_1006113, EPI_ISL_1006115 to 1006116, EPI_ISL_1006118, EPI_ISL_1006120 to 1006127, EPI_ISL_1006130 to 1006133, EPI_ISL_1006135, EPI_ISL_1006137 to 1006138, EPI_ISL_1006141 to 1006149, EPI_ISL_1006151 to 1006155, EPI_ISL_1006157 to 1006158, EPI_ISL_1006160 to 1006162, EPI_ISL_1006165 to 1006166, EPI_ISL_1006170 to 1006171, EPI_ISL_1006173 to 1006177, EPI_ISL_1006179 to 1006185, EPI_ISL_1006341 to 1006342, EPI_ISL_1006344 to 1006356, EPI_ISL_1006358 to 1006361, EPI_ISL_1006363 to 1006371, EPI_ISL_1006373 to 1006385, EPI_ISL_1006387, EPI_ISL_1006389 to 1006391, EPI_ISL_1006393 to 1006395, EPI_ISL_1006399 to 1006407, EPI_ISL_1006409 to 1006412, EPI_ISL_1006414 to 1006424, EPI_ISL_1006426 to 1006428, EPI_ISL_1006430 to 1006431, EPI_ISL_1006433 to 1006438, EPI_ISL_1006440 to 1006445, EPI_ISL_1006447 to 1006449, EPI_ISL_1006451 to 1006452, EPI_ISL_1006454 to 1006455, EPI_ISL_1006457 to 1006465, EPI_ISL_1006467, EPI_ISL_1006469, EPI_ISL_1006472 to 1006479, EPI_ISL_1006481 to 1006489, EPI_ISL_1006491 to 1006511, EPI_ISL_1006513 to 1006515, EPI_ISL_1006517 to 1006523, EPI_ISL_1006525 to 1006532, EPI_ISL_1006534 to 1006539, EPI_ISL_1006544, EPI_ISL_1006546 to 1006547, EPI_ISL_1006551, EPI_ISL_1006553, EPI_ISL_1006555 to 1006556, EPI_ISL_1006559 to 1006560, EPI_ISL_1006566, EPI_ISL_1006568, EPI_ISL_1006570, EPI_ISL_1006573 to 1006574, EPI_ISL_1006576, EPI_ISL_1006579 to 1006580, EPI_ISL_1006582, EPI_ISL_1006584, EPI_ISL_1006586, EPI_ISL_1006589 to 1006591, EPI_ISL_1006594, EPI_ISL_1006601 to 1006602, EPI_ISL_1006604 to 1006607, EPI_ISL_1006609, EPI_ISL_1006613 to 1006614, EPI_ISL_1006620 to 1006621, EPI_ISL_1006624, EPI_ISL_1006626, EPI_ISL_1006630, EPI_ISL_1006632, EPI_ISL_1006634, EPI_ISL_1006636 to 1006637, EPI_ISL_1006639, EPI_ISL_1006645 to 1006646, EPI_ISL_1006648 to 1006651, EPI_ISL_1006656, EPI_ISL_1006658 to 1006666, EPI_ISL_1006668 to 1006687, EPI_ISL_1006689 to 1006707, EPI_ISL_1006709 to 1006717, EPI_ISL_1006719 to 1006721, EPI_ISL_1006723 to 1006735, EPI_ISL_1006737 to 1006740, EPI_ISL_1006742 to 1006745, EPI_ISL_1006747 to 1006760, EPI_ISL_1006762 to 1006769, EPI_ISL_1006771 to 1006773, EPI_ISL_1006775 to 1006793, EPI_ISL_1006796, EPI_ISL_1006798 to 1006800, EPI_ISL_1006802 to 1006803, EPI_ISL_1006805 to 1006806, EPI_ISL_1006808 to 1006818, EPI_ISL_1006821 to 1006834, EPI_ISL_1006836 to 1006840, EPI_ISL_1006842 to 1006844, EPI_ISL_1006846, EPI_ISL_1006848 to 1006852, EPI_ISL_1006854 to 1006856, EPI_ISL_1006858 to 1006863, EPI_ISL_1006941 to 1006947, EPI_ISL_1006949, EPI_ISL_1006951, EPI_ISL_1006953 to 1006954, EPI_ISL_1006956 to 1006960, EPI_ISL_1006962 to 1006984, EPI_ISL_1006986 to 1006994, EPI_ISL_1006996 to 1007006, EPI_ISL_1007008, EPI_ISL_1007010 to 1007021, EPI_ISL_1007023 to 1007026, EPI_ISL_1007028 to 1007032, EPI_ISL_1007034 to 1007041, EPI_ISL_1007044, EPI_ISL_1007046 to 1007048, EPI_ISL_1007050 to 1007054, EPI_ISL_1007056 to 1007058, EPI_ISL_1007060 to 1007061, EPI_ISL_1007065 to 1007068, EPI_ISL_1007070 to 1007073, EPI_ISL_1007075 to 1007077, EPI_ISL_1007079 to 1007104, EPI_ISL_1007106 to 1007119, EPI_ISL_1007121 to 1007127, EPI_ISL_1007129 to 1007141, EPI_ISL_1007143 to 1007158, EPI_ISL_1007160, EPI_ISL_1007162 to 1007174, EPI_ISL_1007176 to 1007180, EPI_ISL_1007526 to 1007527, EPI_ISL_1007575, EPI_ISL_1012403 to 1012405, EPI_ISL_1012408, EPI_ISL_1012410 to 1012413, EPI_ISL_1012415, EPI_ISL_1012417 to 1012418, EPI_ISL_1012420 to 1012422, EPI_ISL_1012425, EPI_ISL_1012427, EPI_ISL_1012429, EPI_ISL_1012432 to 1012434, EPI_ISL_1012436, EPI_ISL_1012440 to 1012447, EPI_ISL_1012451 to 1012455, EPI_ISL_1012457, EPI_ISL_1012459 to 1012467, EPI_ISL_1012470 to 1012479, EPI_ISL_1012483, EPI_ISL_1012485 to 1012492, EPI_ISL_1012494 to 1012495, EPI_ISL_1012497, EPI_ISL_1012500, EPI_ISL_1012502 to 1012508, EPI_ISL_1012511 to 1012512, EPI_ISL_1012515 to 1012520, EPI_ISL_1012522, EPI_ISL_1012524 to 1012530, EPI_ISL_1012533 to 1012536, EPI_ISL_1012538, EPI_ISL_1012540, EPI_ISL_1012542, EPI_ISL_1012544, EPI_ISL_1012546 to 1012548, EPI_ISL_1012608, EPI_ISL_1012669 to 1012671, EPI_ISL_1012862, EPI_ISL_1019409 to 1019416, EPI_ISL_1019418 to 1019423, EPI_ISL_1019425 to 1019426, EPI_ISL_1019428 to 1019430, EPI_ISL_1019432 to 1019433, EPI_ISL_1019435 to 1019440, EPI_ISL_1019442 to 1019445, EPI_ISL_1019447 to 1019458, EPI_ISL_1019460 to 1019465, EPI_ISL_1019467, EPI_ISL_1019469 to 1019470, EPI_ISL_1019472 to 1019480, EPI_ISL_1019586, EPI_ISL_1020016, EPI_ISL_1033566 to 1033570, EPI_ISL_1033572 to 1033577, EPI_ISL_1033579 to 1033585, EPI_ISL_1033587 to 1033592, EPI_ISL_1033594 to 1033597, EPI_ISL_1033599 to 1033600, EPI_ISL_1033602 to 1033603, EPI_ISL_1033605 to 1033608, EPI_ISL_1033611 to 1033615,

EPI_ISL_1033618, EPI_ISL_1033622 to 1033630, EPI_ISL_1033632 to 1033640, EPI_ISL_1033642 to 1033643, EPI_ISL_1033645 to 1033647, EPI_ISL_1033650 to 1033659, EPI_ISL_1033661 to 1033662, EPI_ISL_1033664, EPI_ISL_1033666, EPI_ISL_1033668, EPI_ISL_1033671 to 1033672, EPI_ISL_1033674 to 1033698, EPI_ISL_1033700, EPI_ISL_1033702 to 1033703, EPI_ISL_1033705 to 1033722, EPI_ISL_1033726 to 1033730, EPI_ISL_1033732, EPI_ISL_1033734 to 1033735, EPI_ISL_1033737 to 1033744, EPI_ISL_1033747 to 1033749, EPI_ISL_1033751, EPI_ISL_1033753 to 1033754, EPI_ISL_1033756 to 1033762, EPI_ISL_1033765, EPI_ISL_1033768 to 1033771, EPI_ISL_1033774 to 1033779, EPI_ISL_1033781 to 1033788, EPI_ISL_1033790, EPI_ISL_1033792, EPI_ISL_1033794 to 1033796, EPI_ISL_1033798 to 1033800, EPI_ISL_1033803 to 1033805, EPI_ISL_1033808 to 1033822, EPI_ISL_1033824 to 1033828, EPI_ISL_1033830, EPI_ISL_1033833 to 1033835, EPI_ISL_1033837 to 1033844, EPI_ISL_1033846 to 1033849, EPI_ISL_1033851 to 1033852, EPI_ISL_1033854, EPI_ISL_1033858 to 1033861, EPI_ISL_1033864, EPI_ISL_1033866 to 1033867, EPI_ISL_1033869, EPI_ISL_1033871 to 1033877, EPI_ISL_1033879, EPI_ISL_1033881 to 1033883, EPI_ISL_1033885 to 1033887, EPI_ISL_1033890 to 1033892, EPI_ISL_1033896 to 1033897, EPI_ISL_1033899 to 1033902, EPI_ISL_1033905 to 1033906, EPI_ISL_1033908, EPI_ISL_1033910 to 1033915, EPI_ISL_1033917, EPI_ISL_1033919 to 1033927, EPI_ISL_1033929 to 1033932, EPI_ISL_1033934 to 1033938, EPI_ISL_1033940 to 1033959, EPI_ISL_1033961 to 1033965, EPI_ISL_1033967 to 1033972, EPI_ISL_1033975 to 1033976, EPI_ISL_1033978 to 1033983, EPI_ISL_1033985, EPI_ISL_1033987 to 1033988, EPI_ISL_1033990 to 1034001, EPI_ISL_1034003 to 1034028, EPI_ISL_1034031 to 1034039, EPI_ISL_1034041 to 1034046, EPI_ISL_1034049 to 1034104, EPI_ISL_1034107 to 1034112, EPI_ISL_1034114, EPI_ISL_1034116 to 1034131, EPI_ISL_1034133 to 1034139

see above	Lighthouse Lab in Milton Keynes	Wellcome Sanger Institute for the COVID-19 Genomics UK (COG-UK) Consortium	Cordelia Langford; David K. Jackson; Dominic Kwiatkowski; Ewan Harrison; Ian Johnston; Jeffrey Barrett; John Sillitoe on behalf of the Wellcome Sanger Institute COVID-19 Surveillance Team; John Sillitoe on behalf of the Wellcome Sanger Institute COVID-19 Surveillance Team (http://www.sanger.ac.uk/covid-team); Roberto Amato; Sonia Goncalves; The Lighthouse Lab in Milton Keynes and Alex Alderton

EPI_ISL_549808 to 549823, EPI_ISL_549825 to 549826, EPI_ISL_549828, EPI_ISL_549830 to 549831, EPI_ISL_549833 to 549835, EPI_ISL_549837 to 549860, EPI_ISL_549862 to 549872, EPI_ISL_549874 to 549883, EPI_ISL_549886 to 549901, EPI_ISL_549903 to 549907, EPI_ISL_549909 to 549918, EPI_ISL_549921 to 549926, EPI_ISL_549928 to 549945, EPI_ISL_549947 to 549952, EPI_ISL_549954 to 549964, EPI_ISL_549966 to 549971, EPI_ISL_549973 to 549975, EPI_ISL_549977 to 549980, EPI_ISL_549982 to 549983, EPI_ISL_549985 to 549986, EPI_ISL_549988 to 549994, EPI_ISL_549996, EPI_ISL_549998 to 550009, EPI_ISL_550011 to 550027, EPI_ISL_550030 to 550068, EPI_ISL_550070 to 550071, EPI_ISL_550073 to 550075, EPI_ISL_550077 to 550086, EPI_ISL_550088 to 550095, EPI_ISL_550097 to 550120, EPI_ISL_550122 to 550124, EPI_ISL_550126, EPI_ISL_550129 to 550131, EPI_ISL_550133 to 550139, EPI_ISL_550141 to 550143, EPI_ISL_550145 to 550150, EPI_ISL_550152 to 550153, EPI_ISL_550155 to 550167, EPI_ISL_550169 to 550170, EPI_ISL_550172 to 550178, EPI_ISL_550180 to 550191, EPI_ISL_550193 to 550201, EPI_ISL_550203 to 550208, EPI_ISL_550211 to 550225, EPI_ISL_550227 to 550229, EPI_ISL_550231 to 550236, EPI_ISL_550238 to 550241, EPI_ISL_550243 to 550260, EPI_ISL_550262 to 550270, EPI_ISL_550272 to 550276, EPI_ISL_550278 to 550283, EPI_ISL_550285 to 550287, EPI_ISL_550289 to 550295, EPI_ISL_550297 to 550299, EPI_ISL_550301 to 550311, EPI_ISL_550313 to 550321, EPI_ISL_550323 to 550324, EPI_ISL_550326 to 550331, EPI_ISL_550337, EPI_ISL_550339 to 550366, EPI_ISL_550369, EPI_ISL_550372 to 550373, EPI_ISL_550376 to 550383, EPI_ISL_550385 to 550392, EPI_ISL_550394, EPI_ISL_550396 to 550410, EPI_ISL_550412, EPI_ISL_550414 to 550415, EPI_ISL_550417 to 550423, EPI_ISL_550428, EPI_ISL_550430 to 550432, EPI_ISL_550434 to 550435, EPI_ISL_550437 to 550440, EPI_ISL_550442 to 550443, EPI_ISL_550445 to 550454, EPI_ISL_550456 to 550466, EPI_ISL_550468 to 550473, EPI_ISL_550475, EPI_ISL_550478 to 550481, EPI_ISL_550483, EPI_ISL_550486 to 550488, EPI_ISL_550490 to 550492, EPI_ISL_550496, EPI_ISL_550498, EPI_ISL_550500, EPI_ISL_550503 to 550511, EPI_ISL_550514 to 550516, EPI_ISL_550518 to 550519, EPI_ISL_550521 to 550525, EPI_ISL_550528 to 550536, EPI_ISL_550538 to 550545, EPI_ISL_550547 to 550558, EPI_ISL_550560 to 550561, EPI_ISL_550563 to 550564, EPI_ISL_550566 to 550580, EPI_ISL_550582 to 550589, EPI_ISL_550591 to 550592, EPI_ISL_550594 to 550598, EPI_ISL_550600 to 550622, EPI_ISL_550625 to 550638, EPI_ISL_550640, EPI_ISL_550642 to 550654, EPI_ISL_550656, EPI_ISL_550658 to 550659, EPI_ISL_550661 to 550668, EPI_ISL_550670 to 550676, EPI_ISL_550678 to 550699, EPI_ISL_550701 to 550709, EPI_ISL_550711 to 550720, EPI_ISL_550722 to 550738, EPI_ISL_550740 to 550747, EPI_ISL_550749 to 550755, EPI_ISL_550757 to 550780, EPI_ISL_550782 to 550837, EPI_ISL_550839, EPI_ISL_550841 to 550848, EPI_ISL_550850 to 550869, EPI_ISL_550871 to 550888, EPI_ISL_550890, EPI_ISL_550892 to 550908, EPI_ISL_550910 to 550928, EPI_ISL_550930 to 550932, EPI_ISL_550934, EPI_ISL_550937 to 550951, EPI_ISL_550953 to 550969, EPI_ISL_550971 to 550982, EPI_ISL_550984, EPI_ISL_550986 to 551000, EPI_ISL_551002, EPI_ISL_551004 to 551006, EPI_ISL_551008 to 551012, EPI_ISL_551014 to 551018, EPI_ISL_551021 to 551041, EPI_ISL_551043 to 551046, EPI_ISL_551048 to 551050, EPI_ISL_551052 to 551057, EPI_ISL_551059 to 551062, EPI_ISL_551064 to 551071, EPI_ISL_551073 to 551078, EPI_ISL_551080 to 551090, EPI_ISL_551093 to 551104, EPI_ISL_551107, EPI_ISL_551109 to 551113, EPI_ISL_551115 to 551122, EPI_ISL_551124, EPI_ISL_551126 to 551133, EPI_ISL_551135 to 551136, EPI_ISL_551138 to 551158, EPI_ISL_551161 to 551176, EPI_ISL_551178 to 551180, EPI_ISL_551182 to 551190, EPI_ISL_551192 to 551194, EPI_ISL_551196 to 551199, EPI_ISL_551202, EPI_ISL_551205 to 551210, EPI_ISL_551212 to 551214, EPI_ISL_551216 to 551217, EPI_ISL_551220 to 551225, EPI_ISL_551227 to 551234, EPI_ISL_551236 to 551248, EPI_ISL_551250 to 551253, EPI_ISL_551255, EPI_ISL_551257 to 551274, EPI_ISL_551277 to 551281, EPI_ISL_551284, EPI_ISL_551578 to 551579, EPI_ISL_551581 to 551585, EPI_ISL_551587 to 551592, EPI_ISL_551594 to 551596, EPI_ISL_551598, EPI_ISL_551600 to 551601, EPI_ISL_551603 to 551607, EPI_ISL_551611 to 551618, EPI_ISL_551620 to 551627, EPI_ISL_551629, EPI_ISL_551632 to 551634, EPI_ISL_551636 to 551642, EPI_ISL_551644 to 551646, EPI_ISL_551648 to 551651, EPI_ISL_551653 to 551658, EPI_ISL_551661 to 551662, EPI_ISL_551664 to 551665, EPI_ISL_551667, EPI_ISL_551670 to 551672, EPI_ISL_551674 to 551678, EPI_ISL_551680, EPI_ISL_551683 to 551685, EPI_ISL_551687 to 551692, EPI_ISL_551695 to 551697, EPI_ISL_551699 to 551705, EPI_ISL_551707 to 551713, EPI_ISL_551715 to 551718, EPI_ISL_551720 to 551724, EPI_ISL_551726, EPI_ISL_551728 to 551734, EPI_ISL_551737 to 551739, EPI_ISL_551741, EPI_ISL_551744 to 551745, EPI_ISL_551747 to 551748, EPI_ISL_551750, EPI_ISL_551752 to 551754, EPI_ISL_551758, EPI_ISL_551765 to 551766, EPI_ISL_551773 to 551774, EPI_ISL_551777, EPI_ISL_551781 to 551782, EPI_ISL_551786, EPI_ISL_551790 to 551794, EPI_ISL_551797, EPI_ISL_551800, EPI_ISL_551802, EPI_ISL_551809, EPI_ISL_551813 to 551814, EPI_ISL_551817, EPI_ISL_551820, EPI_ISL_551828 to 551831, EPI_ISL_551833, EPI_ISL_551923 to 551924, EPI_ISL_551926 to 551931, EPI_ISL_551934 to 551936, EPI_ISL_551938 to 551940, EPI_ISL_551942 to 551950, EPI_ISL_551952, EPI_ISL_551954 to 551960, EPI_ISL_551962 to 551963, EPI_ISL_551965 to 551966, EPI_ISL_551968 to 551970, EPI_ISL_551972 to 551976, EPI_ISL_551978 to 551979, EPI_ISL_551981 to 551982, EPI_ISL_551984 to 551987, EPI_ISL_551989, EPI_ISL_551991 to 551992, EPI_ISL_551995 to 551996, EPI_ISL_551998 to 552000, EPI_ISL_552002 to 552004, EPI_ISL_552007, EPI_ISL_552011 to 552012, EPI_ISL_552016, EPI_ISL_552018 to 552021, EPI_ISL_552023 to 552024, EPI_ISL_552026 to 552036, EPI_ISL_552038 to 552043, EPI_ISL_552045, EPI_ISL_552047 to 552050, EPI_ISL_552052, EPI_ISL_552054 to 552063, EPI_ISL_552066 to 552072, EPI_ISL_552075 to 552082, EPI_ISL_552084 to 552087, EPI_ISL_552089 to 552090, EPI_ISL_552092 to 552093, EPI_ISL_552096 to 552097, EPI_ISL_552099 to 552104, EPI_ISL_552107 to 552123, EPI_ISL_552127 to 552147, EPI_ISL_552149 to 552150, EPI_ISL_552152 to 552156, EPI_ISL_552158 to 552159, EPI_ISL_552161 to 552165, EPI_ISL_552167 to 552169, EPI_ISL_552171 to 552172, EPI_ISL_552174 to 552179, EPI_ISL_552181 to 552183, EPI_ISL_552186, EPI_ISL_552188 to 552189, EPI_ISL_552191 to 552198, EPI_ISL_552200 to 552201, EPI_ISL_552203 to 552206, EPI_ISL_552208, EPI_ISL_552210 to 552215, EPI_ISL_552217 to 552226, EPI_ISL_552228 to 552232, EPI_ISL_552234 to 552238, EPI_ISL_552241 to 552245, EPI_ISL_552247 to 552249, EPI_ISL_552251 to 552257, EPI_ISL_552260 to 552261, EPI_ISL_552263 to 552268, EPI_ISL_552270 to 552273, EPI_ISL_552275, EPI_ISL_552277 to 552285, EPI_ISL_552287 to 552291, EPI_ISL_552293, EPI_ISL_552296, EPI_ISL_552298 to 552303, EPI_ISL_552305, EPI_ISL_552307, EPI_ISL_552309 to 552313, EPI_ISL_552315 to 552318, EPI_ISL_552320 to 552330, EPI_ISL_552332, EPI_ISL_552334 to 552341, EPI_ISL_552343 to 552358, EPI_ISL_552361 to 552363, EPI_ISL_552365, EPI_ISL_552367 to 552373, EPI_ISL_552375 to 552376, EPI_ISL_552378, EPI_ISL_552380 to 552387, EPI_ISL_552389, EPI_ISL_552393 to 552398, EPI_ISL_552400 to 552402, EPI_ISL_552405 to 552412, EPI_ISL_552414, EPI_ISL_552416 to 552423, EPI_ISL_552426 to 552433, EPI_ISL_552436 to 552445, EPI_ISL_552448, EPI_ISL_552450 to 552456, EPI_ISL_552459 to 552462, EPI_ISL_552465, EPI_ISL_552467, EPI_ISL_552469 to 552472, EPI_ISL_552474 to 552475, EPI_ISL_552477 to 552478, EPI_ISL_552480, EPI_ISL_552483 to 552484, EPI_ISL_552487, EPI_ISL_552490 to 552491, EPI_ISL_552493 to 552505, EPI_ISL_552509 to 552511, EPI_ISL_552513, EPI_ISL_552517 to 552518, EPI_ISL_552520, EPI_ISL_552522, EPI_ISL_552524 to 552525, EPI_ISL_552528 to 552529, EPI_ISL_552531, EPI_ISL_552535 to 552536, EPI_ISL_552538, EPI_ISL_552541 to 552542, EPI_ISL_552544 to 552545, EPI_ISL_552548 to 552551, EPI_ISL_552553 to 552555, EPI_ISL_552557 to 552558, EPI_ISL_552561, EPI_ISL_552563, EPI_ISL_552567 to 552569, EPI_ISL_552571, EPI_ISL_552581 to 552582, EPI_ISL_552584 to 552586, EPI_ISL_552588 to 552589, EPI_ISL_552594, EPI_ISL_552597, EPI_ISL_552599, EPI_ISL_552601 to 552604, EPI_ISL_552606, EPI_ISL_552609 to 552613, EPI_ISL_552616, EPI_ISL_552619, EPI_ISL_552621, EPI_ISL_552623 to 552628, EPI_ISL_552630, EPI_ISL_552632 to 552635, EPI_ISL_552637 to 552638, EPI_ISL_552640 to 552641, EPI_ISL_552643, EPI_ISL_552647, EPI_ISL_552650 to 552652, EPI_ISL_552656, EPI_ISL_552658, EPI_ISL_552660 to 552664, EPI_ISL_552667 to 552668, EPI_ISL_552672 to 552676, EPI_ISL_552680, EPI_ISL_552682 to 552685, EPI_ISL_552688, EPI_ISL_552690 to 552698, EPI_ISL_552700 to 552702, EPI_ISL_552704 to 552705, EPI_ISL_552708 to 552710, EPI_ISL_552714 to 552715, EPI_ISL_552718, EPI_ISL_552725, EPI_ISL_552727, EPI_ISL_552733, EPI_ISL_552736, EPI_ISL_552738, EPI_ISL_552740, EPI_ISL_552747, EPI_ISL_552752 to 552753, EPI_ISL_552756, EPI_ISL_552759, EPI_ISL_552763, EPI_ISL_552765, EPI_ISL_552767, EPI_ISL_552769, EPI_ISL_552772, EPI_ISL_552781, EPI_ISL_552784, EPI_ISL_552788 to 552791, EPI_ISL_552795, EPI_ISL_552799, EPI_ISL_552802, EPI_ISL_552809 to 552811, EPI_ISL_552814, EPI_ISL_552823, EPI_ISL_552842, EPI_ISL_552852, EPI_ISL_552867, EPI_ISL_552874 to 552875, EPI_ISL_552879, EPI_ISL_552905, EPI_ISL_552910, EPI_ISL_552936, EPI_ISL_552938 to 552939, EPI_ISL_552947 to 552950, EPI_ISL_552953, EPI_ISL_552979, EPI_ISL_552987, EPI_ISL_552990, EPI_ISL_552995, EPI_ISL_552997, EPI_ISL_553008 to 553012, EPI_ISL_553014 to 553021, EPI_ISL_553023 to 553024, EPI_ISL_553026 to 553029, EPI_ISL_553031 to 553036, EPI_ISL_553040 to 553043, EPI_ISL_553046 to 553049, EPI_ISL_553051 to 553053, EPI_ISL_553056 to 553057, EPI_ISL_553059, EPI_ISL_553061 to 553067, EPI_ISL_553072 to 553077, EPI_ISL_553079 to 553081, EPI_ISL_553083, EPI_ISL_553086 to 553089, EPI_ISL_553093 to 553095, EPI_ISL_553097 to 553099, EPI_ISL_553101 to 553104, EPI_ISL_553106 to 553107, EPI_ISL_553111 to 553121, EPI_ISL_553123 to 553127, EPI_ISL_553130 to 553135, EPI_ISL_553137 to 553143, EPI_ISL_553145 to 553147, EPI_ISL_553151 to 553156, EPI_ISL_553158 to 553159, EPI_ISL_553161 to 553169, EPI_ISL_553171 to 553174, EPI_ISL_553176 to 553181, EPI_ISL_553184 to 553185, EPI_ISL_553188 to 553192, EPI_ISL_553194 to 553198, EPI_ISL_553200 to 553201, EPI_ISL_553203 to 553205, EPI_ISL_553207 to 553214, EPI_ISL_553216 to 553230, EPI_ISL_553232 to 553233, EPI_ISL_553236 to 553238, EPI_ISL_553243 to 553245, EPI_ISL_553249, EPI_ISL_553253 to 553268, EPI_ISL_553270, EPI_ISL_553275 to 553276, EPI_ISL_553295, EPI_ISL_553323, EPI_ISL_553879 to 553880, EPI_ISL_553885, EPI_ISL_553889, EPI_ISL_553893 to 553894, EPI_ISL_553896 to 553898, EPI_ISL_553905, EPI_ISL_553907, EPI_ISL_553929, EPI_ISL_553934, EPI_ISL_553939, EPI_ISL_553952, EPI_ISL_553954 to 553957, EPI_ISL_553972, EPI_ISL_553983, EPI_ISL_553987, EPI_ISL_553989, EPI_ISL_553993 to 553994, EPI_ISL_554009, EPI_ISL_554015, EPI_ISL_554024 to 554026, EPI_ISL_554028, EPI_ISL_554042, EPI_ISL_554048, EPI_ISL_554054, EPI_ISL_554057 to 554058, EPI_ISL_554065, EPI_ISL_554080, EPI_ISL_554097, EPI_ISL_554099 to 554101, EPI_ISL_554118, EPI_ISL_554121, EPI_ISL_554123 to 554125, EPI_ISL_554127 to 554128, EPI_ISL_554132 to 554134, EPI_ISL_554137 to 554139, EPI_ISL_554142 to 554144, EPI_ISL_554146 to 554148, EPI_ISL_554150, EPI_ISL_554152 to 554154, EPI_ISL_554159 to 554160, EPI_ISL_554163, EPI_ISL_554166, EPI_ISL_554168 to 554169, EPI_ISL_554173, EPI_ISL_554178 to 554182, EPI_ISL_554184 to 554185, EPI_ISL_554191, EPI_ISL_554200 to 554201, EPI_ISL_554204 to 554205, EPI_ISL_554208, EPI_ISL_554211, EPI_ISL_554214, EPI_ISL_554217 to 554218, EPI_ISL_554221, EPI_ISL_554223 to 554224, EPI_ISL_554226, EPI_ISL_554228, EPI_ISL_554231, EPI_ISL_554237, EPI_ISL_554239 to 554240, EPI_ISL_554242 to 554247, EPI_ISL_554249, EPI_ISL_554253 to 554256, EPI_ISL_554258, EPI_ISL_554261 to 554262, EPI_ISL_554264, EPI_ISL_554267, EPI_ISL_554269, EPI_ISL_554271, EPI_ISL_554274, EPI_ISL_554276, EPI_ISL_554284 to 554287, EPI_ISL_554289 to 554291, EPI_ISL_554294, EPI_ISL_554297, EPI_ISL_554300, EPI_ISL_554302, EPI_ISL_554304, EPI_ISL_554306, EPI_ISL_554308, EPI_ISL_554310, EPI_ISL_554312 to 554315, EPI_ISL_554317, EPI_ISL_554319, EPI_ISL_554619, EPI_ISL_554622, EPI_ISL_554628 to 554629, EPI_ISL_554633, EPI_ISL_554635 to 554637, EPI_ISL_554639, EPI_ISL_554641, EPI_ISL_554643 to 554644, EPI_ISL_554647 to 554648, EPI_ISL_554658 to 554660, EPI_ISL_554665, EPI_ISL_554668, EPI_ISL_554670 to 554675, EPI_ISL_554678 to 554679, EPI_ISL_554682 to 554685, EPI_ISL_554687, EPI_ISL_554690 to 554691, EPI_ISL_554695, EPI_ISL_554698, EPI_ISL_554700 to 554701, EPI_ISL_554705 to 554707, EPI_ISL_554712 to 554715, EPI_ISL_554717 to 554724, EPI_ISL_554729, EPI_ISL_554731, EPI_ISL_554734, EPI_ISL_554737 to 554740, EPI_ISL_554744 to 554747, EPI_ISL_554749, EPI_ISL_554751 to 554753, EPI_ISL_554760, EPI_ISL_554762, EPI_ISL_554765 to 554766, EPI_ISL_554768 to 554770, EPI_ISL_554773 to 554775, EPI_ISL_554783 to 554791, EPI_ISL_554793, EPI_ISL_554801, EPI_ISL_554805 to 554806, EPI_ISL_554814, EPI_ISL_554817 to 554819, EPI_ISL_554821 to 554822, EPI_ISL_554824 to 554826, EPI_ISL_554828 to 554829, EPI_ISL_554831, EPI_ISL_554835, EPI_ISL_554838 to 554839, EPI_ISL_554842 to 554844, EPI_ISL_554851 to 554852, EPI_ISL_554854 to 554855, EPI_ISL_554857, EPI_ISL_554860, EPI_ISL_555558, EPI_ISL_555616, EPI_ISL_556075 to 556077, EPI_ISL_556079, EPI_ISL_556083, EPI_ISL_556087, EPI_ISL_556090, EPI_ISL_556092 to 556093, EPI_ISL_556095, EPI_ISL_556097, EPI_ISL_556102, EPI_ISL_556104, EPI_ISL_556109, EPI_ISL_556112, EPI_ISL_556116, EPI_ISL_556126, EPI_ISL_556138 to 556139, EPI_ISL_556146, EPI_ISL_556148, EPI_ISL_556163, EPI_ISL_556166, EPI_ISL_556170, EPI_ISL_556172, EPI_ISL_556176, EPI_ISL_556187, EPI_ISL_556195, EPI_ISL_556197 to 556199, EPI_ISL_556204, EPI_ISL_556208, EPI_ISL_556211, EPI_ISL_556214, EPI_ISL_556223, EPI_ISL_556232 to 556233, EPI_ISL_556235, EPI_ISL_556237, EPI_ISL_556244, EPI_ISL_556248, EPI_ISL_556251, EPI_ISL_556253, EPI_ISL_556261, EPI_ISL_556263, EPI_ISL_556266, EPI_ISL_556275 to 556276, EPI_ISL_556285, EPI_ISL_556289, EPI_ISL_556293 to 556294, EPI_ISL_556298, EPI_ISL_556300, EPI_ISL_556304, EPI_ISL_557167 to 557168, EPI_ISL_557170 to 557175, EPI_ISL_557177 to 557179, EPI_ISL_557181 to 557185, EPI_ISL_557187 to 557188, EPI_ISL_557190 to 557191, EPI_ISL_557194 to 557198, EPI_ISL_557202 to 557206, EPI_ISL_557208 to 557212, EPI_ISL_557214 to 557224, EPI_ISL_557226, EPI_ISL_557228, EPI_ISL_557230 to 557231, EPI_ISL_557233 to 557236, EPI_ISL_557239 to 557242, EPI_ISL_557244, EPI_ISL_557246 to 557248, EPI_ISL_557251 to 557253, EPI_ISL_557256 to 557261, EPI_ISL_557263 to 557264, EPI_ISL_557266 to 557268, EPI_ISL_557270, EPI_ISL_557273, EPI_ISL_557275 to 557282, EPI_ISL_557285 to 557286, EPI_ISL_557288 to 557293, EPI_ISL_557296 to 557298, EPI_ISL_557300, EPI_ISL_557303, EPI_ISL_557305, EPI_ISL_557307, EPI_ISL_557309 to 557312, EPI_ISL_557314 to 557319, EPI_ISL_557321 to 557323, EPI_ISL_557326 to 557329, EPI_ISL_557331 to 557334, EPI_ISL_557336 to 557339, EPI_ISL_557341 to 557344, EPI_ISL_557346 to 557350, EPI_ISL_557352, EPI_ISL_557357, EPI_ISL_557360, EPI_ISL_557364 to 557365, EPI_ISL_557367, EPI_ISL_557369, EPI_ISL_557372, EPI_ISL_557375, EPI_ISL_557377, EPI_ISL_557384 to 557387, EPI_ISL_557389, EPI_ISL_557391, EPI_ISL_557393 to 557397, EPI_ISL_557400 to 557401, EPI_ISL_557407, EPI_ISL_557410, EPI_ISL_557413, EPI_ISL_557416, EPI_ISL_557418, EPI_ISL_557421, EPI_ISL_557426 to 557427, EPI_ISL_557429, EPI_ISL_557431 to 557432, EPI_ISL_557435, EPI_ISL_557437, EPI_ISL_557441 to 557443, EPI_ISL_557445, EPI_ISL_557450 to 557451, EPI_ISL_557455 to 557461, EPI_ISL_557463 to 557465, EPI_ISL_557467, EPI_ISL_557470, EPI_ISL_557473, EPI_ISL_557479, EPI_ISL_557483, EPI_ISL_557486, EPI_ISL_557489 to 557490, EPI_ISL_557492, EPI_ISL_557495 to 557496, EPI_ISL_557498, EPI_ISL_557505 to 557506, EPI_ISL_557508, EPI_ISL_557510, EPI_ISL_557512 to 557515, EPI_ISL_557517, EPI_ISL_557519 to 557521, EPI_ISL_557525, EPI_ISL_557529, EPI_ISL_557532, EPI_ISL_557534 to 557536, EPI_ISL_557538, EPI_ISL_557540, EPI_ISL_557542, EPI_ISL_557545 to 557547, EPI_ISL_557550, EPI_ISL_557552, EPI_ISL_557740, EPI_ISL_557742, EPI_ISL_557748, EPI_ISL_557753, EPI_ISL_557757, EPI_ISL_557765 to 557766, EPI_ISL_557781, EPI_ISL_557784, EPI_ISL_557789, EPI_ISL_557793, EPI_ISL_557795 to 557796, EPI_ISL_557800, EPI_ISL_557802, EPI_ISL_557812, EPI_ISL_557821 to 557824, EPI_ISL_557827, EPI_ISL_557829 to 557830, EPI_ISL_557838, EPI_ISL_557845, EPI_ISL_557847 to 557849, EPI_ISL_557853 to 557854, EPI_ISL_557858, EPI_ISL_557866 to 557867, EPI_ISL_557869 to 557870, EPI_ISL_557876 to 557879, EPI_ISL_557890, EPI_ISL_557893 to 557895, EPI_ISL_557905, EPI_ISL_557909, EPI_ISL_557911, EPI_ISL_557919, EPI_ISL_557925 to 557927, EPI_ISL_557938, EPI_ISL_557940 to 557942, EPI_ISL_557944, EPI_ISL_557947, EPI_ISL_557949, EPI_ISL_557953 to 557954, EPI_ISL_557956, EPI_ISL_557959, EPI_ISL_557964 to 557965, EPI_ISL_557972, EPI_ISL_557974, EPI_ISL_557977, EPI_ISL_557979, EPI_ISL_557984, EPI_ISL_557989, EPI_ISL_558021, EPI_ISL_558026, EPI_ISL_558028, EPI_ISL_558037, EPI_ISL_558041, EPI_ISL_558058, EPI_ISL_558068, EPI_ISL_558096, EPI_ISL_558155, EPI_ISL_558195, EPI_ISL_558234, EPI_ISL_558239, EPI_ISL_558241 to 558243, EPI_ISL_558245 to 558247, EPI_ISL_558251 to 558255, EPI_ISL_558257 to 558258, EPI_ISL_558262, EPI_ISL_558264 to 558266, EPI_ISL_558272 to 558273, EPI_ISL_558278 to 558279, EPI_ISL_558281, EPI_ISL_558284 to 558287, EPI_ISL_558290, EPI_ISL_558294 to 558304, EPI_ISL_558306, EPI_ISL_558309, EPI_ISL_558313 to 558315, EPI_ISL_558317 to 558320, EPI_ISL_558322 to 558323, EPI_ISL_558325 to 558327, EPI_ISL_558331, EPI_ISL_558333 to 558334, EPI_ISL_558336 to 558337, EPI_ISL_558340 to 558342, EPI_ISL_558344, EPI_ISL_558347, EPI_ISL_558351 to 558353, EPI_ISL_558357 to 558361, EPI_ISL_558363, EPI_ISL_558365, EPI_ISL_558367, EPI_ISL_558369, EPI_ISL_558372, EPI_ISL_558374 to 558376, EPI_ISL_558378, EPI_ISL_558380 to 558386, EPI_ISL_558388, EPI_ISL_558390 to 558391, EPI_ISL_558393, EPI_ISL_558397, EPI_ISL_558399 to 558401, EPI_ISL_558638, EPI_ISL_558731, EPI_ISL_558914, EPI_ISL_558920, EPI_ISL_558923, EPI_ISL_558925, EPI_ISL_558928, EPI_ISL_558932, EPI_ISL_558934, EPI_ISL_558951, EPI_ISL_558959, EPI_ISL_558965, EPI_ISL_558970, EPI_ISL_558973, EPI_ISL_558980 to 558981, EPI_ISL_559000, EPI_ISL_559005, EPI_ISL_559009, EPI_ISL_559011, EPI_ISL_559028, EPI_ISL_559043, EPI_ISL_559047, EPI_ISL_559049, EPI_ISL_559057, EPI_ISL_559061, EPI_ISL_559063, EPI_ISL_559071, EPI_ISL_559080 to 559081, EPI_ISL_559084, EPI_ISL_559088, EPI_ISL_559100, EPI_ISL_559132, EPI_ISL_559136, EPI_ISL_559149, EPI_ISL_559154, EPI_ISL_559157, EPI_ISL_559163 to 559164, EPI_ISL_559167, EPI_ISL_559174, EPI_ISL_559187, EPI_ISL_559194 to 559195, EPI_ISL_559201, EPI_ISL_559218, EPI_ISL_559223, EPI_ISL_559236, EPI_ISL_559241, EPI_ISL_559247, EPI_ISL_559253, EPI_ISL_559256, EPI_ISL_559259, EPI_ISL_559263, EPI_ISL_559277 to 559278, EPI_ISL_559283 to 559284, EPI_ISL_559290, EPI_ISL_559300, EPI_ISL_559312, EPI_ISL_559314, EPI_ISL_559319 to 559320, EPI_ISL_559326, EPI_ISL_559331, EPI_ISL_559335, EPI_ISL_559352, EPI_ISL_559354 to 559355, EPI_ISL_559368, EPI_ISL_559662 to 559669, EPI_ISL_559671 to 559672, EPI_ISL_559674 to 559692, EPI_ISL_559694 to 559699, EPI_ISL_559701 to 559705, EPI_ISL_559707 to 559708, EPI_ISL_559710 to 559714, EPI_ISL_559716, EPI_ISL_559718 to 559723, EPI_ISL_559725 to 559732, EPI_ISL_559735 to 559739, EPI_ISL_559741 to 559747, EPI_ISL_559749 to 559751, EPI_ISL_566421, EPI_ISL_566424 to 566425, EPI_ISL_566430 to 566434, EPI_ISL_566436 to 566441, EPI_ISL_566443, EPI_ISL_566446 to 566452, EPI_ISL_566454, EPI_ISL_566458 to 566463, EPI_ISL_566465 to 566466, EPI_ISL_566468 to 566472, EPI_ISL_566475, EPI_ISL_566477 to 566478, EPI_ISL_566480 to 566485, EPI_ISL_566487 to 566491, EPI_ISL_566496 to 566499, EPI_ISL_566503, EPI_ISL_566506 to 566507, EPI_ISL_566509 to 566512, EPI_ISL_566514 to 566517, EPI_ISL_566520 to 566523, EPI_ISL_566525 to 566527, EPI_ISL_566530 to 566533, EPI_ISL_566535 to 566536, EPI_ISL_566539 to 566542, EPI_ISL_566545 to 566553, EPI_ISL_566555 to 566562, EPI_ISL_566564 to 566566, EPI_ISL_566568 to 566574, EPI_ISL_566577 to 566585, EPI_ISL_566590 to 566609, EPI_ISL_566612 to 566618, EPI_ISL_566620 to 566624, EPI_ISL_566626 to 566630, EPI_ISL_566632 to 566634, EPI_ISL_566636, EPI_ISL_566639 to 566646, EPI_ISL_566649 to 566658, EPI_ISL_566660, EPI_ISL_566663 to 566665, EPI_ISL_566668 to 566671, EPI_ISL_566673, EPI_ISL_566675 to 566693, EPI_ISL_566695 to 566702, EPI_ISL_566704, EPI_ISL_566706, EPI_ISL_566708 to 566723, EPI_ISL_566725 to 566732, EPI_ISL_566734 to 566745, EPI_ISL_566748 to 566754, EPI_ISL_566756 to 566785, EPI_ISL_566787, EPI_ISL_566789 to 566790, EPI_ISL_566792 to 566794, EPI_ISL_566796 to 566798, EPI_ISL_566801 to 566804, EPI_ISL_566806 to 566810, EPI_ISL_566812 to 566814, EPI_ISL_566816 to 566822, EPI_ISL_566824 to 566837, EPI_ISL_566839 to 566840, EPI_ISL_566842 to 566860, EPI_ISL_566862, EPI_ISL_566864 to 566865, EPI_ISL_566867 to 566872, EPI_ISL_566874 to 566875, EPI_ISL_566877, EPI_ISL_566879 to 566896, EPI_ISL_566898 to 566903, EPI_ISL_566906 to 566921, EPI_ISL_566923 to 566924, EPI_ISL_566926, EPI_ISL_566928 to 566931, EPI_ISL_566933 to 566935, EPI_ISL_566937 to 566950, EPI_ISL_566952 to 566963, EPI_ISL_566965 to 566969, EPI_ISL_566971 to 566972, EPI_ISL_566974 to 566985, EPI_ISL_566987 to 566989, EPI_ISL_566991 to 566997, EPI_ISL_568289, EPI_ISL_568364 to 568365, EPI_ISL_568386 to 568387, EPI_ISL_568470 to 568471, EPI_ISL_568476, EPI_ISL_575367, EPI_ISL_575762 to 575766, EPI_ISL_575768 to 575777, EPI_ISL_575779 to 575787, EPI_ISL_575789 to 575795, EPI_ISL_575797 to 575800, EPI_ISL_575802 to 575804, EPI_ISL_575806 to 575807, EPI_ISL_575809 to 575811, EPI_ISL_575813 to 575820, EPI_ISL_575823 to 575836, EPI_ISL_575838 to 575843, EPI_ISL_575846 to 575849, EPI_ISL_575851 to 575855, EPI_ISL_575857 to 575869, EPI_ISL_575872 to 575874, EPI_ISL_575876, EPI_ISL_575878 to

575882, EPI_ISL_575884 to 575892, EPI_ISL_575894, EPI_ISL_575896 to 575901, EPI_ISL_575904 to 575911, EPI_ISL_575913 to 575916, EPI_ISL_575918 to 575924, EPI_ISL_575926 to 575927, EPI_ISL_575929 to 575930, EPI_ISL_575932, EPI_ISL_575934 to 575956, EPI_ISL_575958 to 575959, EPI_ISL_575961 to 575965, EPI_ISL_575967 to 575968, EPI_ISL_575970 to 575973, EPI_ISL_575975 to 575978, EPI_ISL_575980 to 575981, EPI_ISL_575984 to 575993, EPI_ISL_576094, EPI_ISL_580631, EPI_ISL_580634, EPI_ISL_580637 to 580639, EPI_ISL_580641 to 580642, EPI_ISL_580644, EPI_ISL_580647, EPI_ISL_580649, EPI_ISL_580652 to 580653, EPI_ISL_580656 to 580658, EPI_ISL_580661 to 580662, EPI_ISL_580665 to 580673, EPI_ISL_580683, EPI_ISL_580685, EPI_ISL_580687 to 580688, EPI_ISL_580694, EPI_ISL_580698, EPI_ISL_580700 to 580702, EPI_ISL_580704, EPI_ISL_580707, EPI_ISL_580709, EPI_ISL_580711 to 580713, EPI_ISL_580716 to 580719, EPI_ISL_580726, EPI_ISL_580728 to 580729, EPI_ISL_580731 to 580733, EPI_ISL_580735 to 580736, EPI_ISL_580741 to 580750, EPI_ISL_580752 to 580753, EPI_ISL_580755, EPI_ISL_580757, EPI_ISL_580760, EPI_ISL_580762, EPI_ISL_580766 to 580768, EPI_ISL_580770, EPI_ISL_580773, EPI_ISL_580775 to 580779, EPI_ISL_580781 to 580782, EPI_ISL_580786, EPI_ISL_580794 to 580797, EPI_ISL_580800 to 580804, EPI_ISL_580806 to 580810, EPI_ISL_580812, EPI_ISL_580814 to 580815, EPI_ISL_580817 to 580820, EPI_ISL_580826 to 580827, EPI_ISL_580829 to 580832, EPI_ISL_580836 to 580837, EPI_ISL_580839, EPI_ISL_580841 to 580845, EPI_ISL_580847 to 580848, EPI_ISL_580850, EPI_ISL_580852, EPI_ISL_580854, EPI_ISL_580856 to 580857, EPI_ISL_580861, EPI_ISL_580864, EPI_ISL_580873, EPI_ISL_580875, EPI_ISL_580877, EPI_ISL_580882, EPI_ISL_580885, EPI_ISL_580892, EPI_ISL_580896 to 580898, EPI_ISL_580900, EPI_ISL_580902 to 580904, EPI_ISL_580910, EPI_ISL_580913, EPI_ISL_580915, EPI_ISL_580917 to 580918, EPI_ISL_580921, EPI_ISL_580929, EPI_ISL_580935, EPI_ISL_580940, EPI_ISL_580948, EPI_ISL_580950 to 580951, EPI_ISL_580961, EPI_ISL_580976, EPI_ISL_580979 to 580980, EPI_ISL_580990 to 580991, EPI_ISL_580993, EPI_ISL_580995, EPI_ISL_580998, EPI_ISL_581000, EPI_ISL_581004, EPI_ISL_581008 to 581009, EPI_ISL_581018, EPI_ISL_581023, EPI_ISL_581025 to 581026, EPI_ISL_581028 to 581029, EPI_ISL_581032, EPI_ISL_581035, EPI_ISL_581038, EPI_ISL_581040 to 581041, EPI_ISL_581046, EPI_ISL_581054, EPI_ISL_581056, EPI_ISL_581058, EPI_ISL_581064 to 581065, EPI_ISL_581067, EPI_ISL_581069, EPI_ISL_581074, EPI_ISL_581078, EPI_ISL_581084, EPI_ISL_581086, EPI_ISL_581093, EPI_ISL_581097, EPI_ISL_581099, EPI_ISL_581103, EPI_ISL_581106 to 581107, EPI_ISL_581111, EPI_ISL_581113 to 581116, EPI_ISL_581118 to 581124, EPI_ISL_581126, EPI_ISL_581128 to 581129, EPI_ISL_581131, EPI_ISL_581134, EPI_ISL_581136 to 581140, EPI_ISL_581142 to 581145, EPI_ISL_581147 to 581148, EPI_ISL_581151 to 581153, EPI_ISL_581155 to 581165, EPI_ISL_581167 to 581173, EPI_ISL_581175 to 581181, EPI_ISL_581184 to 581188, EPI_ISL_581190, EPI_ISL_581192, EPI_ISL_581195, EPI_ISL_581197 to 581203, EPI_ISL_581205 to 581213, EPI_ISL_581215 to 581216, EPI_ISL_581218 to 581222, EPI_ISL_581224 to 581225, EPI_ISL_581227 to 581228, EPI_ISL_581231 to 581241, EPI_ISL_581243 to 581244, EPI_ISL_581246, EPI_ISL_581248 to 581260, EPI_ISL_581263 to 581266, EPI_ISL_581268 to 581270, EPI_ISL_581272 to 581284, EPI_ISL_581287 to 581290, EPI_ISL_581292 to 581306, EPI_ISL_581308, EPI_ISL_581311 to 581322, EPI_ISL_581325 to 581328, EPI_ISL_581330 to 581333, EPI_ISL_581335 to 581336, EPI_ISL_581338 to 581340, EPI_ISL_581342 to 581343, EPI_ISL_581345, EPI_ISL_581347 to 581349, EPI_ISL_581352 to 581353, EPI_ISL_581355 to 581357, EPI_ISL_581360 to 581365, EPI_ISL_581368 to 581374, EPI_ISL_581376, EPI_ISL_581385 to 581386, EPI_ISL_581389 to 581390, EPI_ISL_581397 to 581399, EPI_ISL_581401, EPI_ISL_581421 to 581422, EPI_ISL_581428, EPI_ISL_581434, EPI_ISL_581438, EPI_ISL_581447, EPI_ISL_589017 to 589019, EPI_ISL_589024, EPI_ISL_589026, EPI_ISL_589028 to 589030, EPI_ISL_589034, EPI_ISL_589039 to 589041, EPI_ISL_589043, EPI_ISL_589051, EPI_ISL_589057, EPI_ISL_589063, EPI_ISL_589067, EPI_ISL_589070, EPI_ISL_589074 to 589075, EPI_ISL_589077, EPI_ISL_589080 to 589081, EPI_ISL_589086, EPI_ISL_589090 to 589091, EPI_ISL_589095, EPI_ISL_589097 to 589099, EPI_ISL_589101, EPI_ISL_589103, EPI_ISL_589110 to 589111, EPI_ISL_589114 to 589115, EPI_ISL_589117 to 589118, EPI_ISL_589121, EPI_ISL_589123, EPI_ISL_589133, EPI_ISL_589147, EPI_ISL_589153, EPI_ISL_589157, EPI_ISL_589160 to 589161, EPI_ISL_589166 to 589174, EPI_ISL_589176, EPI_ISL_589179, EPI_ISL_589183, EPI_ISL_589185, EPI_ISL_589195 to 589196, EPI_ISL_589199 to 589200, EPI_ISL_589205, EPI_ISL_589211 to 589212, EPI_ISL_589221, EPI_ISL_589224, EPI_ISL_589226 to 589227, EPI_ISL_589231, EPI_ISL_589233, EPI_ISL_589235, EPI_ISL_589237, EPI_ISL_589242 to 589243, EPI_ISL_589247, EPI_ISL_589251, EPI_ISL_589256, EPI_ISL_589259 to 589261, EPI_ISL_589265 to 589266, EPI_ISL_589297, EPI_ISL_589304, EPI_ISL_589335, EPI_ISL_589355, EPI_ISL_589377, EPI_ISL_589407, EPI_ISL_589417, EPI_ISL_589430, EPI_ISL_589440, EPI_ISL_589493, EPI_ISL_589519 to 589521, EPI_ISL_589573 to 589589, EPI_ISL_589591 to 589594, EPI_ISL_589596 to 589607, EPI_ISL_589609 to 589616, EPI_ISL_589618, EPI_ISL_589620 to 589621, EPI_ISL_589623 to 589635, EPI_ISL_589637 to 589638, EPI_ISL_589640 to 589643, EPI_ISL_589645 to 589649, EPI_ISL_589651 to 589662, EPI_ISL_589664, EPI_ISL_589666 to 589675, EPI_ISL_589679 to 589681, EPI_ISL_589684 to 589685, EPI_ISL_589687 to 589694, EPI_ISL_589696 to 589700, EPI_ISL_589702 to 589703, EPI_ISL_589705 to 589706, EPI_ISL_589708 to 589709, EPI_ISL_589711 to 589712, EPI_ISL_589714 to 589721, EPI_ISL_589723 to 589732, EPI_ISL_589734 to 589736, EPI_ISL_589738 to 589755, EPI_ISL_589757, EPI_ISL_589759 to 589761, EPI_ISL_589763 to 589764, EPI_ISL_589766, EPI_ISL_589770 to 589773, EPI_ISL_589787, EPI_ISL_589789, EPI_ISL_589791 to 589792, EPI_ISL_589794 to 589801, EPI_ISL_590172, EPI_ISL_590174 to 590175, EPI_ISL_598251 to 598268, EPI_ISL_598270 to 598282, EPI_ISL_598284, EPI_ISL_598286 to 598288, EPI_ISL_598290 to 598304, EPI_ISL_598306 to 598308, EPI_ISL_598310 to 598321, EPI_ISL_598323 to 598327, EPI_ISL_598329, EPI_ISL_598332 to 598341, EPI_ISL_598343 to 598344, EPI_ISL_598346 to 598349, EPI_ISL_598351 to 598360, EPI_ISL_598362 to 598366, EPI_ISL_598369 to 598371, EPI_ISL_598373 to 598377, EPI_ISL_598379 to 598383, EPI_ISL_598385 to 598387, EPI_ISL_598390, EPI_ISL_598392 to 598407, EPI_ISL_598409 to 598412, EPI_ISL_598414 to 598423, EPI_ISL_598425 to 598426, EPI_ISL_598428 to 598432, EPI_ISL_598434, EPI_ISL_598437 to 598451, EPI_ISL_598453 to 598457, EPI_ISL_598459 to 598461, EPI_ISL_598463 to 598476, EPI_ISL_598478 to 598479, EPI_ISL_598481 to 598488, EPI_ISL_598490 to 598505, EPI_ISL_598507 to 598513, EPI_ISL_598515 to 598523, EPI_ISL_598525 to 598529, EPI_ISL_598531, EPI_ISL_598533 to 598534, EPI_ISL_598536 to 598537, EPI_ISL_598539 to 598544, EPI_ISL_598546 to 598559, EPI_ISL_598562 to 598563, EPI_ISL_598566 to 598567, EPI_ISL_598569 to 598571, EPI_ISL_598573, EPI_ISL_598575 to 598587, EPI_ISL_598590 to 598596, EPI_ISL_598598 to 598602, EPI_ISL_598605, EPI_ISL_598607 to 598614, EPI_ISL_598616 to 598619, EPI_ISL_598621 to 598624, EPI_ISL_598626 to 598632, EPI_ISL_598634, EPI_ISL_598636 to 598637, EPI_ISL_598639 to 598649, EPI_ISL_598651 to 598655, EPI_ISL_598657, EPI_ISL_598659 to 598666, EPI_ISL_598668 to 598685, EPI_ISL_598687 to 598690, EPI_ISL_598692 to 598700, EPI_ISL_598702, EPI_ISL_598704 to 598731, EPI_ISL_598733 to 598743, EPI_ISL_598747 to 598750, EPI_ISL_598752 to 598758, EPI_ISL_598760 to 598761, EPI_ISL_598763 to 598764, EPI_ISL_598767, EPI_ISL_598769 to 598776, EPI_ISL_598778 to 598792, EPI_ISL_598794 to 598803, EPI_ISL_598805 to 598807, EPI_ISL_598809 to 598813, EPI_ISL_598816, EPI_ISL_598818 to 598820, EPI_ISL_598822 to 598823, EPI_ISL_599818 to 599820, EPI_ISL_599822 to 599831, EPI_ISL_599833, EPI_ISL_599835 to 599844, EPI_ISL_599846 to 599866, EPI_ISL_599868 to 599885, EPI_ISL_599887, EPI_ISL_599889 to 599903, EPI_ISL_599905 to 599912, EPI_ISL_599914 to 599925, EPI_ISL_599927 to 599947, EPI_ISL_599949, EPI_ISL_599951 to 599955, EPI_ISL_599957 to 599959, EPI_ISL_599961 to 599962, EPI_ISL_599964 to 599972, EPI_ISL_599974 to 599990, EPI_ISL_599992 to 599994, EPI_ISL_599996 to 600006, EPI_ISL_600008 to 600032, EPI_ISL_600034 to 600046, EPI_ISL_600048 to 600052, EPI_ISL_600054 to 600055, EPI_ISL_600057 to 600082, EPI_ISL_600084 to 600090, EPI_ISL_600094 to 600106, EPI_ISL_600108 to 600122, EPI_ISL_600124 to 600127, EPI_ISL_600892, EPI_ISL_600901, EPI_ISL_600912 to 600913, EPI_ISL_600915, EPI_ISL_600925 to 600931, EPI_ISL_601224, EPI_ISL_601234 to 601235, EPI_ISL_601239 to 601240, EPI_ISL_601242 to 601243, EPI_ISL_601248 to 601249, EPI_ISL_601256, EPI_ISL_601258, EPI_ISL_601263 to 601264, EPI_ISL_601267 to 601268, EPI_ISL_601270, EPI_ISL_601272 to 601273, EPI_ISL_601275, EPI_ISL_601277, EPI_ISL_601280 to 601283, EPI_ISL_601287, EPI_ISL_601289, EPI_ISL_601291 to 601294, EPI_ISL_601298 to 601299, EPI_ISL_601302 to 601307, EPI_ISL_601310 to 601313, EPI_ISL_601316 to 601317, EPI_ISL_601320 to 601322, EPI_ISL_601324 to 601327, EPI_ISL_601329 to 601330, EPI_ISL_601337 to 601338, EPI_ISL_601340, EPI_ISL_601343, EPI_ISL_601345 to 601346, EPI_ISL_601349 to 601350, EPI_ISL_601354, EPI_ISL_601359 to 601360, EPI_ISL_601363, EPI_ISL_601365, EPI_ISL_601367 to 601371, EPI_ISL_601373, EPI_ISL_601376, EPI_ISL_601378 to 601379, EPI_ISL_601381 to 601383, EPI_ISL_601385, EPI_ISL_601388, EPI_ISL_601390, EPI_ISL_601392, EPI_ISL_601394, EPI_ISL_601396 to 601398, EPI_ISL_601404, EPI_ISL_601406, EPI_ISL_601409 to 601410, EPI_ISL_601412, EPI_ISL_601415, EPI_ISL_601417 to 601420, EPI_ISL_601427 to 601428, EPI_ISL_601431, EPI_ISL_601434, EPI_ISL_601437 to 601442, EPI_ISL_601444, EPI_ISL_601447 to 601452, EPI_ISL_601455 to 601458, EPI_ISL_601463 to 601465, EPI_ISL_601468, EPI_ISL_601470, EPI_ISL_601472 to 601476, EPI_ISL_601478, EPI_ISL_601485, EPI_ISL_601487, EPI_ISL_601490 to 601492, EPI_ISL_601494, EPI_ISL_601496 to 601498, EPI_ISL_601500, EPI_ISL_601502 to 601505, EPI_ISL_601508 to 601514, EPI_ISL_601516 to 601521, EPI_ISL_601523 to 601524, EPI_ISL_601526 to 601527, EPI_ISL_601529, EPI_ISL_601531 to 601535, EPI_ISL_601537 to 601540, EPI_ISL_601542 to 601543, EPI_ISL_601545 to 601546, EPI_ISL_601551 to 601554, EPI_ISL_601556 to 601557, EPI_ISL_601559 to 601566, EPI_ISL_601568, EPI_ISL_601570, EPI_ISL_601572, EPI_ISL_601574 to 601577, EPI_ISL_601581 to 601583, EPI_ISL_601586 to 601587, EPI_ISL_601592 to 601593, EPI_ISL_601596, EPI_ISL_601598 to 601599, EPI_ISL_601601 to 601603, EPI_ISL_601605, EPI_ISL_601607 to 601610, EPI_ISL_601612 to 601614, EPI_ISL_601620 to 601621, EPI_ISL_601623, EPI_ISL_601625 to 601626, EPI_ISL_601629 to 601630, EPI_ISL_601632, EPI_ISL_601635, EPI_ISL_601637 to 601638, EPI_ISL_601640, EPI_ISL_601642 to 601647, EPI_ISL_601649, EPI_ISL_601651, EPI_ISL_601653 to 601656, EPI_ISL_601658 to 601662, EPI_ISL_601665, EPI_ISL_601669 to 601670, EPI_ISL_601672, EPI_ISL_601674 to 601679, EPI_ISL_601681, EPI_ISL_601683 to 601684, EPI_ISL_601686 to 601689, EPI_ISL_601692 to 601697, EPI_ISL_601699, EPI_ISL_601701 to 601703, EPI_ISL_601705 to 601708, EPI_ISL_601710 to 601714, EPI_ISL_601716 to 601720, EPI_ISL_601722 to 601727, EPI_ISL_601729, EPI_ISL_601732, EPI_ISL_601734 to 601735, EPI_ISL_601737 to 601739, EPI_ISL_601741 to 601746, EPI_ISL_601749 to 601750, EPI_ISL_601752 to 601754, EPI_ISL_601757 to 601763, EPI_ISL_601767, EPI_ISL_601769 to 601770, EPI_ISL_601772, EPI_ISL_601774 to 601777, EPI_ISL_601779 to 601782, EPI_ISL_601784, EPI_ISL_601786, EPI_ISL_602027, EPI_ISL_602029, EPI_ISL_602145, EPI_ISL_602147 to 602149, EPI_ISL_602151 to 602153, EPI_ISL_606296 to 606299, EPI_ISL_606301 to 606307, EPI_ISL_606309 to 606310, EPI_ISL_606312 to 606313, EPI_ISL_606315, EPI_ISL_606317 to 606318, EPI_ISL_606320, EPI_ISL_606322 to 606332, EPI_ISL_606334 to 606352, EPI_ISL_606354 to 606359, EPI_ISL_606361 to 606369, EPI_ISL_606371 to 606374, EPI_ISL_606376 to 606378, EPI_ISL_606380, EPI_ISL_606382 to 606401, EPI_ISL_606403, EPI_ISL_606405 to 606407, EPI_ISL_606409 to 606414, EPI_ISL_606417 to 606421, EPI_ISL_606423, EPI_ISL_606426, EPI_ISL_606428 to 606451, EPI_ISL_606453 to 606458, EPI_ISL_606460 to 606461, EPI_ISL_606463, EPI_ISL_606465 to 606484, EPI_ISL_606487, EPI_ISL_606489 to 606507, EPI_ISL_606509 to 606511, EPI_ISL_606513 to 606515, EPI_ISL_606517 to 606522, EPI_ISL_606525 to 606542, EPI_ISL_606544 to 606546, EPI_ISL_606548, EPI_ISL_606550 to 606564, EPI_ISL_606566 to 606572, EPI_ISL_606574 to 606587, EPI_ISL_606589 to 606597, EPI_ISL_606599 to 606607, EPI_ISL_606609 to 606616, EPI_ISL_606618 to 606621, EPI_ISL_606623, EPI_ISL_606625 to 606627, EPI_ISL_606629 to 606632, EPI_ISL_606635 to 606645, EPI_ISL_606647 to 606653, EPI_ISL_606656 to 606670, EPI_ISL_606672 to 606680, EPI_ISL_606682 to 606691, EPI_ISL_606693, EPI_ISL_606695 to 606699, EPI_ISL_606703 to 606725, EPI_ISL_606727, EPI_ISL_606729, EPI_ISL_606731, EPI_ISL_606734 to 606739, EPI_ISL_606741, EPI_ISL_606743 to 606744, EPI_ISL_606746, EPI_ISL_606748, EPI_ISL_606750 to 606753, EPI_ISL_606755 to 606763, EPI_ISL_606765 to 606773, EPI_ISL_606775 to 606785, EPI_ISL_606787 to 606790, EPI_ISL_606792 to 606793, EPI_ISL_606796, EPI_ISL_606798 to 606801, EPI_ISL_606803 to 606805, EPI_ISL_606807 to 606820, EPI_ISL_606822 to 606828, EPI_ISL_606830 to 606832, EPI_ISL_606834 to 606843, EPI_ISL_606846 to 606860, EPI_ISL_606864 to 606865, EPI_ISL_606869 to 606874, EPI_ISL_606876 to 606877, EPI_ISL_606881 to 606886, EPI_ISL_606888 to 606891, EPI_ISL_606893, EPI_ISL_606895 to 606896, EPI_ISL_606898 to 606900, EPI_ISL_606902 to 606903, EPI_ISL_606906 to 606908, EPI_ISL_606910 to 606915, EPI_ISL_606917 to 606921, EPI_ISL_606923, EPI_ISL_607292 to 607302, EPI_ISL_607304 to 607307, EPI_ISL_607309 to 607311, EPI_ISL_607313 to 607315, EPI_ISL_607317 to 607321, EPI_ISL_607324 to 607325, EPI_ISL_607328, EPI_ISL_607330 to 607333, EPI_ISL_607335 to 607337, EPI_ISL_607339 to 607342, EPI_ISL_607344, EPI_ISL_607346 to 607352, EPI_ISL_607354 to 607356, EPI_ISL_607358 to 607372, EPI_ISL_607374, EPI_ISL_607376 to 607396, EPI_ISL_607398 to 607407, EPI_ISL_607411 to 607415, EPI_ISL_607417, EPI_ISL_607419 to 607422, EPI_ISL_607424 to 607425, EPI_ISL_607427, EPI_ISL_607429 to 607432, EPI_ISL_607434 to 607447, EPI_ISL_607449 to 607454, EPI_ISL_607456 to 607459, EPI_ISL_607462 to 607464, EPI_ISL_607466 to 607475, EPI_ISL_607477 to 607494, EPI_ISL_607497 to 607504, EPI_ISL_607507 to 607515, EPI_ISL_607517 to 607518, EPI_ISL_607520 to 607538, EPI_ISL_607540 to 607546, EPI_ISL_607548 to 607549, EPI_ISL_607551 to 607561, EPI_ISL_607563 to 607565, EPI_ISL_607567 to 607571, EPI_ISL_607573 to 607574, EPI_ISL_607576 to 607578, EPI_ISL_607580 to 607582, EPI_ISL_607584 to 607585, EPI_ISL_607588 to 607611, EPI_ISL_607613, EPI_ISL_607615 to 607622, EPI_ISL_608697 to 608703, EPI_ISL_608705 to 608711, EPI_ISL_608713 to 608714, EPI_ISL_608716 to 608719, EPI_ISL_608721 to 608723, EPI_ISL_608725 to 608726, EPI_ISL_608732 to 608735, EPI_ISL_608737 to 608740, EPI_ISL_608742 to 608747, EPI_ISL_608749 to 608751, EPI_ISL_608753, EPI_ISL_608755 to 608758, EPI_ISL_608760 to 608767, EPI_ISL_608771 to 608773, EPI_ISL_608775 to 608780, EPI_ISL_608782 to 608784, EPI_ISL_608786, EPI_ISL_608788 to 608795, EPI_ISL_608797, EPI_ISL_608800 to 608808, EPI_ISL_608810 to 608818, EPI_ISL_608820 to 608823, EPI_ISL_608825 to 608826, EPI_ISL_608828, EPI_ISL_608830 to 608831, EPI_ISL_608833, EPI_ISL_608837 to 608839, EPI_ISL_608841 to 608842, EPI_ISL_608844, EPI_ISL_608846, EPI_ISL_608848, EPI_ISL_608850, EPI_ISL_608854, EPI_ISL_608857, EPI_ISL_608859, EPI_ISL_608861, EPI_ISL_608871, EPI_ISL_608882, EPI_ISL_608893, EPI_ISL_608901, EPI_ISL_608905, EPI_ISL_608940, EPI_ISL_608951, EPI_ISL_608954, EPI_ISL_608977 to 608978, EPI_ISL_608986, EPI_ISL_609001, EPI_ISL_609006, EPI_ISL_609008, EPI_ISL_609017, EPI_ISL_609029 to 609030, EPI_ISL_609036, EPI_ISL_609038, EPI_ISL_609042, EPI_ISL_609046, EPI_ISL_609049, EPI_ISL_609058 to 609059, EPI_ISL_609062 to 609063, EPI_ISL_609066, EPI_ISL_609071, EPI_ISL_609076, EPI_ISL_609084, EPI_ISL_609108, EPI_ISL_609110, EPI_ISL_609115 to 609117, EPI_ISL_609126, EPI_ISL_609128, EPI_ISL_609136 to 609139, EPI_ISL_609149, EPI_ISL_609220 to 609223, EPI_ISL_609225 to 609226, EPI_ISL_609229, EPI_ISL_609231 to 609236, EPI_ISL_609238 to 609239, EPI_ISL_609241 to 609257, EPI_ISL_609259, EPI_ISL_609261 to 609266, EPI_ISL_609268 to 609284, EPI_ISL_609288, EPI_ISL_609290, EPI_ISL_609292 to 609296, EPI_ISL_609298 to 609301, EPI_ISL_609303 to 609307, EPI_ISL_623211 to 623213, EPI_ISL_623215 to 623219, EPI_ISL_623221, EPI_ISL_623223, EPI_ISL_623225 to 623233, EPI_ISL_623236 to 623237, EPI_ISL_623239 to 623244, EPI_ISL_623246, EPI_ISL_623249 to 623251, EPI_ISL_623253 to 623255, EPI_ISL_623258, EPI_ISL_623261, EPI_ISL_623263, EPI_ISL_623265, EPI_ISL_623269 to 623271, EPI_ISL_623274 to 623275, EPI_ISL_623277, EPI_ISL_623279 to 623280, EPI_ISL_623282 to 623285, EPI_ISL_623287, EPI_ISL_623289 to 623294, EPI_ISL_623296 to 623297, EPI_ISL_623299 to 623300, EPI_ISL_623302 to 623307, EPI_ISL_623309 to 623310, EPI_ISL_623314 to 623320, EPI_ISL_623322, EPI_ISL_623324, EPI_ISL_623327 to 623329, EPI_ISL_623331 to 623332, EPI_ISL_623335 to 623336, EPI_ISL_623338 to 623342, EPI_ISL_623350 to 623358, EPI_ISL_623361 to 623362, EPI_ISL_623365, EPI_ISL_623367 to 623369, EPI_ISL_623372 to 623373, EPI_ISL_623375 to 623384, EPI_ISL_623389, EPI_ISL_623394 to 623396, EPI_ISL_623400 to 623403, EPI_ISL_623405 to 623408, EPI_ISL_623410, EPI_ISL_623412 to 623413, EPI_ISL_623415 to 623416, EPI_ISL_623419 to 623420, EPI_ISL_623422 to 623423, EPI_ISL_623429 to 623432, EPI_ISL_623434 to 623436, EPI_ISL_623439, EPI_ISL_623442 to 623443, EPI_ISL_623446 to 623447, EPI_ISL_623452, EPI_ISL_623454 to 623455, EPI_ISL_623457, EPI_ISL_623459 to 623462, EPI_ISL_623464 to 623467, EPI_ISL_623469 to 623473, EPI_ISL_623478 to 623481, EPI_ISL_623483, EPI_ISL_623487, EPI_ISL_623491 to 623493, EPI_ISL_623496, EPI_ISL_623499 to 623505, EPI_ISL_623507, EPI_ISL_623509 to 623514, EPI_ISL_623516 to 623517, EPI_ISL_623519, EPI_ISL_623521, EPI_ISL_623523 to 623525, EPI_ISL_623529, EPI_ISL_623534 to 623536, EPI_ISL_623540 to 623542, EPI_ISL_623549, EPI_ISL_623552 to 623553, EPI_ISL_623561, EPI_ISL_623563 to 623566, EPI_ISL_623573, EPI_ISL_623577, EPI_ISL_623579 to 623580, EPI_ISL_623582 to 623583, EPI_ISL_623586, EPI_ISL_623588, EPI_ISL_623591, EPI_ISL_623596, EPI_ISL_623598 to 623599, EPI_ISL_623601, EPI_ISL_623603 to 623604, EPI_ISL_623606 to 623607, EPI_ISL_623610, EPI_ISL_623614 to 623615, EPI_ISL_623620 to 623623, EPI_ISL_623625, EPI_ISL_623627 to 623629, EPI_ISL_623631 to 623632, EPI_ISL_623634, EPI_ISL_623636 to 623638, EPI_ISL_623642, EPI_ISL_623646, EPI_ISL_623648 to 623649, EPI_ISL_623656, EPI_ISL_623659 to 623660, EPI_ISL_623668 to 623673, EPI_ISL_623676, EPI_ISL_623681 to 623682, EPI_ISL_623684, EPI_ISL_623686 to 623687, EPI_ISL_623689, EPI_ISL_623691 to 623693, EPI_ISL_623695, EPI_ISL_623697 to 623699, EPI_ISL_623704 to 623709, EPI_ISL_623711, EPI_ISL_623714 to 623715, EPI_ISL_623718, EPI_ISL_623720, EPI_ISL_623722 to 623723, EPI_ISL_623727 to 623729, EPI_ISL_623734, EPI_ISL_623737, EPI_ISL_623739, EPI_ISL_623744 to 623745, EPI_ISL_623748, EPI_ISL_623752 to 623753, EPI_ISL_623755 to 623757, EPI_ISL_623760 to 623761, EPI_ISL_623766 to 623770, EPI_ISL_623773, EPI_ISL_623777 to 623778, EPI_ISL_623780, EPI_ISL_623782 to 623784, EPI_ISL_623786 to 623787, EPI_ISL_623789, EPI_ISL_623792, EPI_ISL_623795, EPI_ISL_623803 to 623805, EPI_ISL_623807, EPI_ISL_623813 to 623814, EPI_ISL_623817 to 623821, EPI_ISL_623824 to 623826, EPI_ISL_623835 to 623837, EPI_ISL_623842, EPI_ISL_623845, EPI_ISL_623851 to 623853, EPI_ISL_625406 to 625407, EPI_ISL_625409 to 625421, EPI_ISL_625424, EPI_ISL_625426, EPI_ISL_625430, EPI_ISL_629109 to 629119, EPI_ISL_629121 to 629136, EPI_ISL_629139 to 629176, EPI_ISL_629178 to 629198, EPI_ISL_629200 to 629208, EPI_ISL_629210 to 629216, EPI_ISL_629218, EPI_ISL_629220 to 629228, EPI_ISL_629230 to 629240, EPI_ISL_629242 to 629244, EPI_ISL_629246 to 629251, EPI_ISL_629253 to 629271, EPI_ISL_629273 to 629275, EPI_ISL_629278 to 629304, EPI_ISL_629306 to 629308, EPI_ISL_629310, EPI_ISL_629312 to 629319, EPI_ISL_629321 to 629322, EPI_ISL_629324 to 629330, EPI_ISL_629332 to 629336, EPI_ISL_629338 to 629346, EPI_ISL_629348 to 629362, EPI_ISL_629364 to 629397, EPI_ISL_629399 to 629402, EPI_ISL_629405 to 629407, EPI_ISL_629410 to 629427, EPI_ISL_629429 to 629437, EPI_ISL_629439, EPI_ISL_629441 to 629445, EPI_ISL_629447 to 629449, EPI_ISL_629451 to 629453, EPI_ISL_629455 to 629458, EPI_ISL_629460 to 629461, EPI_ISL_629463 to 629465, EPI_ISL_629467 to 629475, EPI_ISL_629477, EPI_ISL_629480 to 629483, EPI_ISL_629485 to 629489, EPI_ISL_629491 to 629494, EPI_ISL_629496 to 629498, EPI_ISL_629500 to 629502, EPI_ISL_629504 to 629508, EPI_ISL_629510 to 629511, EPI_ISL_629513 to 629515, EPI_ISL_629517 to 629519, EPI_ISL_629521 to 629525, EPI_ISL_629527, EPI_ISL_629529 to 629531, EPI_ISL_629535 to 629543, EPI_ISL_629545, EPI_ISL_629547, EPI_ISL_629550 to 629552, EPI_ISL_629555 to 629556, EPI_ISL_629558, EPI_ISL_629560 to 629561, EPI_ISL_629564 to 629566, EPI_ISL_629569, EPI_ISL_629571, EPI_ISL_629574 to 629579, EPI_ISL_629582, EPI_ISL_629584 to 629589, EPI_ISL_629594 to 629596, EPI_ISL_629598, EPI_ISL_629601, EPI_ISL_629603 to 629606, EPI_ISL_629609 to 629619, EPI_ISL_629621 to 629631, EPI_ISL_629634 to 629637, EPI_ISL_629639 to 629642, EPI_ISL_629644, EPI_ISL_629646 to 629653, EPI_ISL_629655 to 629661, EPI_ISL_629664, EPI_ISL_629667, EPI_ISL_629669 to 629671, EPI_ISL_629673 to 629677, EPI_ISL_629679 to 629682, EPI_ISL_629684 to 629687, EPI_ISL_629689, EPI_ISL_629691, EPI_ISL_629693 to 629698, EPI_ISL_629700, EPI_ISL_629704 to 629705, EPI_ISL_629707 to 629709, EPI_ISL_629712 to 629719, EPI_ISL_629721 to 629722, EPI_ISL_629724, EPI_ISL_629726, EPI_ISL_629730 to 629732, EPI_ISL_629734, EPI_ISL_629737 to 629738, EPI_ISL_629740, EPI_ISL_629743 to 629746, EPI_ISL_629748 to 629752, EPI_ISL_630007 to 630009, EPI_ISL_630011 to 630013, EPI_ISL_630015 to 630018, EPI_ISL_630020 to 630021, EPI_ISL_630023 to 630025, EPI_ISL_630027 to 630035, EPI_ISL_630038 to 630047, EPI_ISL_630049 to 630050, EPI_ISL_630052 to 630085, EPI_ISL_630087 to 630090, EPI_ISL_630092 to 630094, EPI_ISL_630096 to 630108, EPI_ISL_630111 to 630113, EPI_ISL_630115 to 630128, EPI_ISL_630130 to 630146, EPI_ISL_630148 to 630150, EPI_ISL_630152 to 630161, EPI_ISL_630163 to 630166, EPI_ISL_630168 to 630169, EPI_ISL_630171 to 630178, EPI_ISL_630180 to 630186, EPI_ISL_630188, EPI_ISL_630190, EPI_ISL_630192 to 630194, EPI_ISL_630196 to 630204, EPI_ISL_630206 to 630209, EPI_ISL_630211 to 630218, EPI_ISL_630220 to 630223, EPI_ISL_630225 to 630231, EPI_ISL_630233 to 630234, EPI_ISL_630236 to 630237, EPI_ISL_630240 to 630243, EPI_ISL_630246, EPI_ISL_630248, EPI_ISL_630250 to 630251, EPI_ISL_630253, EPI_ISL_630256 to 630260, EPI_ISL_630262 to 630265, EPI_ISL_630267 to 630275, EPI_ISL_630277 to 630286, EPI_ISL_630288 to 630297, EPI_ISL_630299 to 630300, EPI_ISL_630302 to 630318, EPI_ISL_630320 to 630331, EPI_ISL_630333 to 630342, EPI_ISL_630344, EPI_ISL_630346 to 630347, EPI_ISL_630973 to 630974, EPI_ISL_630978, EPI_ISL_630981, EPI_ISL_630986, EPI_ISL_630990 to 630991, EPI_ISL_630993, EPI_ISL_630995, EPI_ISL_630998, EPI_ISL_631000, EPI_ISL_631002, EPI_ISL_631006 to 631007, EPI_ISL_631009 to 631010, EPI_ISL_631013, EPI_ISL_631015, EPI_ISL_631019 to 631022, EPI_ISL_631024, EPI_ISL_631026, EPI_ISL_631029 to 631030, EPI_ISL_631032, EPI_ISL_631034, EPI_ISL_631036 to 631037, EPI_ISL_631040, EPI_ISL_631042 to 631043, EPI_ISL_631047 to 631049, EPI_ISL_631051 to 631052, EPI_ISL_631058, EPI_ISL_631061, EPI_ISL_631064 to 631067, EPI_ISL_631070 to 631073, EPI_ISL_631075 to 631077, EPI_ISL_631079, EPI_ISL_631082 to 631083, EPI_ISL_631085, EPI_ISL_631087 to 631088, EPI_ISL_631091 to 631092, EPI_ISL_631095, EPI_ISL_631098, EPI_ISL_631100, EPI_ISL_631103 to 631104, EPI_ISL_631106, EPI_ISL_631108, EPI_ISL_631110, EPI_ISL_631114 to 631115, EPI_ISL_631119 to 631122, EPI_ISL_631124 to 631126, EPI_ISL_631130, EPI_ISL_631134, EPI_ISL_631136, EPI_ISL_631138 to 631141, EPI_ISL_631143, EPI_ISL_631145 to 631146, EPI_ISL_631151 to 631153, EPI_ISL_631156, EPI_ISL_631161 to 631162, EPI_ISL_631166 to 631170, EPI_ISL_631172, EPI_ISL_631176, EPI_ISL_631178 to 631179, EPI_ISL_631182 to 631183, EPI_ISL_631186 to 631188, EPI_ISL_631193, EPI_ISL_631196 to 631198, EPI_ISL_631200 to 631201, EPI_ISL_631205 to 631207, EPI_ISL_631209 to 631211, EPI_ISL_631213 to 631214, EPI_ISL_631216, EPI_ISL_631218, EPI_ISL_631223, EPI_ISL_631226 to 631227, EPI_ISL_631229 to 631230, EPI_ISL_631232 to 631234, EPI_ISL_631236, EPI_ISL_631238, EPI_ISL_631240, EPI_ISL_631242 to 631244, EPI_ISL_631246 to 631249, EPI_ISL_631251 to 631253, EPI_ISL_631255 to 631256, EPI_ISL_631258 to 631261, EPI_ISL_631264, EPI_ISL_631267, EPI_ISL_631275, EPI_ISL_634256, EPI_ISL_634258 to 634259, EPI_ISL_634261 to 634265, EPI_ISL_634267 to 634278, EPI_ISL_634280 to 634300, EPI_ISL_634303, EPI_ISL_634305 to 634310, EPI_ISL_634312 to 634317, EPI_ISL_634319, EPI_ISL_634321 to 634325, EPI_ISL_634327 to 634332, EPI_ISL_634334 to 634336, EPI_ISL_634340, EPI_ISL_634342 to 634352, EPI_ISL_634354 to 634362, EPI_ISL_634364 to 634365, EPI_ISL_634367 to 634375, EPI_ISL_634378 to 634383, EPI_ISL_634386 to 634390,

EPI_ISL_634392 to 634394, EPI_ISL_634396 to 634405, EPI_ISL_634407, EPI_ISL_634409 to 634427, EPI_ISL_634429 to 634444, EPI_ISL_634446, EPI_ISL_634448 to 634453, EPI_ISL_634455 to 634459, EPI_ISL_634461, EPI_ISL_634463 to 634464, EPI_ISL_634466 to 634474, EPI_ISL_634476 to 634479, EPI_ISL_634481 to 634482, EPI_ISL_634484, EPI_ISL_634486 to 634491, EPI_ISL_634494 to 634495, EPI_ISL_634497 to 634498, EPI_ISL_634500 to 634509, EPI_ISL_634511 to 634516, EPI_ISL_634518 to 634519			
see above	Lighthouse Lab in Milton Keynes	Wellcome Sanger Institute for the COVID-19 Genomics UK (COG-UK) consortium	Cordelia Langford; David K. Jackson; Dominic Kwiatkowski; Ewan Harrison; Ian Johnston; John Sillitoe on behalf of the Wellcome Sanger Institute COVID-19 Surveillance Team; John Sillitoe on behalf of the Wellcome Sanger Institute COVID-19 Surveillance Team (http://www.sanger.ac.uk/covid-team); Roberto Amato; Sonia Goncalves; The Lighthouse Lab in Alderley Park and Alex Alderton; The Lighthouse Lab in Milton Keynes and Alex Alderton
EPI_ISL_730621, EPI_ISL_730624 to 730625, EPI_ISL_730630, EPI_ISL_730635, EPI_ISL_751191 to 751192, EPI_ISL_766586	Limmattal Hospital	Institute of Medical Virology, University of Zurich	Alexandra Trkola; Annette Audigé; Cyril Shah; Gabriela Ziltener; Jon Huder; Jürg Böni; Kevin Steiner; Maria Grünberg; Maryam Zaheri; Michael Huber; Riccarda Capaul; Roberto Buonomano; Stefan Schmutz; Verena Kufner
EPI_ISL_631689 to 631690	Lincoln Medical Center	New York City Public Health Laboratory	Jade Wang; et al.
EPI_ISL_484354 to 484355, EPI_ISL_484357 to 484358, EPI_ISL_484360 to 484363, EPI_ISL_484366, EPI_ISL_484368 to 484372, EPI_ISL_484392 to 484394, EPI_ISL_484396, EPI_ISL_484399 to 484402, EPI_ISL_484406, EPI_ISL_490563, EPI_ISL_490566 to 490571, EPI_ISL_490573 to 490577, EPI_ISL_490579 to 490580, EPI_ISL_493549 to 493551, EPI_ISL_493553 to 493556, EPI_ISL_493558, EPI_ISL_493560, EPI_ISL_493564, EPI_ISL_493567 to 493568, EPI_ISL_493570 to 493572, EPI_ISL_493574, EPI_ISL_493576, EPI_ISL_493578, EPI_ISL_493580 to 493581, EPI_ISL_493584, EPI_ISL_493590 to 493591, EPI_ISL_493603 to 493604, EPI_ISL_549517 to 549522, EPI_ISL_572938, EPI_ISL_572943 to 572944, EPI_ISL_573437 to 573449, EPI_ISL_679946, EPI_ISL_679951 to 679954, EPI_ISL_679957 to 679958, EPI_ISL_679960, EPI_ISL_679962, EPI_ISL_679965, EPI_ISL_679967 to 679968, EPI_ISL_725541, EPI_ISL_725544 to 725546, EPI_ISL_725548 to 725550, EPI_ISL_725552 to 725553, EPI_ISL_725555 to 725556, EPI_ISL_725558 to 725560, EPI_ISL_725563 to 725564, EPI_ISL_725568 to 725571, EPI_ISL_725573 to 725574, EPI_ISL_725577, EPI_ISL_725581, EPI_ISL_725583, EPI_ISL_725586 to 725589, EPI_ISL_725592, EPI_ISL_725594, EPI_ISL_725604, EPI_ISL_840093, EPI_ISL_840123, EPI_ISL_840128 to 840129, EPI_ISL_840131 to 840132, EPI_ISL_840136, EPI_ISL_866999, EPI_ISL_867022, EPI_ISL_922041, EPI_ISL_922051, EPI_ISL_922064, EPI_ISL_922070, EPI_ISL_922081, EPI_ISL_922084, EPI_ISL_922091, EPI_ISL_922106, EPI_ISL_922112 to 922113, EPI_ISL_922116, EPI_ISL_922136, EPI_ISL_922139, EPI_ISL_922143, EPI_ISL_922151, EPI_ISL_922160, EPI_ISL_922170, EPI_ISL_950716, EPI_ISL_950732, EPI_ISL_950735, EPI_ISL_950738, EPI_ISL_950741, EPI_ISL_950745, EPI_ISL_950749, EPI_ISL_950754, EPI_ISL_998936, EPI_ISL_998938, EPI_ISL_998954, EPI_ISL_998956, EPI_ISL_998960, EPI_ISL_998962 to 998964, EPI_ISL_998974, EPI_ISL_998977			
see above	Lincolnshire Hospitals and DeepSeq Nottingham	COVID-19 Genomics UK (COG-UK) Consortium	Christopher Moore; Fei Sang; Joeseph Chappell; Johnny Debebe; Jonathan Ball; Matthew Carlisle; Matthew Loose; Nadine Holmes; Nichola Duckworth; Patrick McClure; Sarah Walsh; Tim Sloan; Victoria Wright
EPI_ISL_648200 to 648203	Linkoping	The Public Health Agency of Sweden	Anna Risberg; Anna-Malin Linde; Karin Tegmark-Wisell; Maria Lind Karlberg; Mattias Haukland; Mia Brytting; Olov Svartstrom; Oskar Karlsson Lindsjo; Petra Edquist; Reza Advani; Sandra Broddesson
EPI_ISL_1008713	Lister Laboratorio de Referencia S.A. de C.V.	Instituto de diagnóstico y Referencia Epidemiologicos (INDRE) Departamento de Virología	Abril Rodriguez-Maldonado; Claudia Wong-Arambula; Ernesto Ramirez-Gonzalez.; Fabiola Garces-Ayala; Gisela Barrera-Badillo; Irma Lopez-Martinez; Lucia Hernandez-Rivas; Natividad Cruz-Ortiz; Tatiana Nunez-Garcia
EPI_ISL_541847 to 541877	Lithuanian University of Health Sciences Hospital, Department of Laboratory Medicine	Lithuanian University of Health Sciences, Laboratory of Molecular Cardiology	Arnoldas Pautienius; Astra Vitkauskiene; Dovydas Gecys; Kamile Tamusauskaite; Laura Pareckaite; Lukas Zemaitis; Vaiva Lesauskaite
EPI_ISL_536398, EPI_ISL_603082 to 603118, EPI_ISL_603120, EPI_ISL_636604, EPI_ISL_636839 to 636861, EPI_ISL_636863 to 636870, EPI_ISL_636872 to 636882, EPI_ISL_636884 to 636906, EPI_ISL_770498 to 770532			
see above	Lithuanian University of Health Sciences Hospital, Department of Laboratory Medicine	Lithuanian University of Health Sciences, Molecular cardiology lab.	Arnoldas Pautienius; Astra Vitkauskiene; Dovydas Gecys; Ingrida Olendrait; Kamile Tamusauskaite; Laura Pareckaite; Lukas Zemaitis; Vaiva Lesauskaite
EPI_ISL_439672 to 439673, EPI_ISL_439677, EPI_ISL_439681 to 439684, EPI_ISL_439687, EPI_ISL_439697, EPI_ISL_439704, EPI_ISL_439706 to 439710, EPI_ISL_439712, EPI_ISL_439714 to 439715, EPI_ISL_439718 to 439722, EPI_ISL_439724 to 439725, EPI_ISL_439728, EPI_ISL_439730 to 439731, EPI_ISL_439733, EPI_ISL_439737, EPI_ISL_439744, EPI_ISL_439747 to 439748, EPI_ISL_439751 to 439754, EPI_ISL_439756 to 439758, EPI_ISL_439762 to 439763, EPI_ISL_439765, EPI_ISL_439767, EPI_ISL_439770, EPI_ISL_439775, EPI_ISL_439777, EPI_ISL_439780, EPI_ISL_439782, EPI_ISL_439784 to 439785, EPI_ISL_439787 to 439788, EPI_ISL_439790 to 439791, EPI_ISL_439793, EPI_ISL_439795 to 439796, EPI_ISL_439798 to 439799, EPI_ISL_439804, EPI_ISL_439806 to 439808, EPI_ISL_439810, EPI_ISL_439812, EPI_ISL_439815, EPI_ISL_439817, EPI_ISL_439820 to 439821, EPI_ISL_439824 to 439825, EPI_ISL_439827, EPI_ISL_439832, EPI_ISL_439834, EPI_ISL_439836 to 439837, EPI_ISL_439848 to 439849, EPI_ISL_439855, EPI_ISL_439857, EPI_ISL_439861, EPI_ISL_440853, EPI_ISL_440863 to 440864, EPI_ISL_440866 to 440867, EPI_ISL_440873, EPI_ISL_440875 to 440876, EPI_ISL_440878, EPI_ISL_440881, EPI_ISL_440883 to 440884, EPI_ISL_440886 to 440887, EPI_ISL_440892, EPI_ISL_440894 to 440895, EPI_ISL_440897 to 440898, EPI_ISL_440900, EPI_ISL_440903 to 440905, EPI_ISL_440910, EPI_ISL_440912, EPI_ISL_440917, EPI_ISL_440922 to 440925, EPI_ISL_440927, EPI_ISL_440930, EPI_ISL_440935 to 440936, EPI_ISL_440939, EPI_ISL_440946, EPI_ISL_440948 to 440949, EPI_ISL_449605, EPI_ISL_449608, EPI_ISL_449611, EPI_ISL_449614, EPI_ISL_449623, EPI_ISL_453255, EPI_ISL_453327, EPI_ISL_453433, EPI_ISL_453435, EPI_ISL_453437, EPI_ISL_453441 to 453442, EPI_ISL_453446, EPI_ISL_453452 to 453453, EPI_ISL_471993 to 472001, EPI_ISL_472003 to 472016, EPI_ISL_472018 to 472022, EPI_ISL_472057, EPI_ISL_472090 to 472093, EPI_ISL_472096 to 472100, EPI_ISL_472102, EPI_ISL_472104 to 472107, EPI_ISL_472109, EPI_ISL_472111, EPI_ISL_472113 to 472116, EPI_ISL_472118 to 472119, EPI_ISL_472121 to 472122, EPI_ISL_472124, EPI_ISL_472126 to 472127, EPI_ISL_472129, EPI_ISL_472131 to 472135, EPI_ISL_478392, EPI_ISL_478394 to 478396, EPI_ISL_484254, EPI_ISL_484256, EPI_ISL_484262 to 484263, EPI_ISL_490336 to 490339, EPI_ISL_490343, EPI_ISL_490345 to 490348, EPI_ISL_490354, EPI_ISL_490359, EPI_ISL_490364 to 490365, EPI_ISL_490370 to 490371, EPI_ISL_490373 to 490374, EPI_ISL_490377 to 490380, EPI_ISL_490382, EPI_ISL_490398, EPI_ISL_490408 to 490410, EPI_ISL_490412 to 490415, EPI_ISL_490417 to 490420, EPI_ISL_499461 to 499469, EPI_ISL_499640 to 499649, EPI_ISL_499651, EPI_ISL_499653 to 499776, EPI_ISL_499811 to 499824, EPI_ISL_499984 to 500156, EPI_ISL_512342, EPI_ISL_512344, EPI_ISL_517001 to 517008, EPI_ISL_517010 to 517018, EPI_ISL_517020 to 517025, EPI_ISL_517027, EPI_ISL_517029, EPI_ISL_517031, EPI_ISL_517033 to 517034, EPI_ISL_517036 to 517037, EPI_ISL_517039 to 517041, EPI_ISL_517044 to 517045, EPI_ISL_517047 to 517052, EPI_ISL_517055, EPI_ISL_517057 to 517061, EPI_ISL_517063 to 517064, EPI_ISL_517066, EPI_ISL_517068, EPI_ISL_517070 to 517085, EPI_ISL_517087, EPI_ISL_517093, EPI_ISL_517095, EPI_ISL_517108, EPI_ISL_517116, EPI_ISL_517121, EPI_ISL_517123, EPI_ISL_517129, EPI_ISL_517139, EPI_ISL_517144, EPI_ISL_517146, EPI_ISL_517151, EPI_ISL_517155, EPI_ISL_517159 to 517160, EPI_ISL_517165 to 517166, EPI_ISL_517170 to 517178, EPI_ISL_517180, EPI_ISL_517182 to 517193, EPI_ISL_517195 to 517196, EPI_ISL_517198 to 517201, EPI_ISL_517203 to 517227, EPI_ISL_517231 to 517238, EPI_ISL_517240 to 517244, EPI_ISL_517246 to 517273, EPI_ISL_517275 to 517276, EPI_ISL_517278 to 517279, EPI_ISL_517281 to 517282, EPI_ISL_517284, EPI_ISL_517286 to 517289, EPI_ISL_517291 to 517294, EPI_ISL_517296 to 517297, EPI_ISL_517300 to 517320, EPI_ISL_517322 to 517341, EPI_ISL_517343 to 517371, EPI_ISL_517373, EPI_ISL_517380, EPI_ISL_517385, EPI_ISL_517396 to 517397, EPI_ISL_517399, EPI_ISL_517410, EPI_ISL_517416, EPI_ISL_517422, EPI_ISL_517432 to 517433, EPI_ISL_517438, EPI_ISL_517444, EPI_ISL_517448 to 517449, EPI_ISL_517452, EPI_ISL_517464 to 517466, EPI_ISL_517469 to 517473, EPI_ISL_517475 to 517477, EPI_ISL_517479 to 517498, EPI_ISL_517500 to 517509, EPI_ISL_526368 to 526391, EPI_ISL_526393 to 526394, EPI_ISL_534732, EPI_ISL_534748 to 534755, EPI_ISL_540587 to 540588, EPI_ISL_540590 to 540593, EPI_ISL_540603 to 540605, EPI_ISL_540610, EPI_ISL_540613 to 540614, EPI_ISL_540617 to 540620, EPI_ISL_540622 to 540625, EPI_ISL_540629, EPI_ISL_572710, EPI_ISL_573236 to 573246, EPI_ISL_584607 to 584608, EPI_ISL_584611 to 584613, EPI_ISL_584615, EPI_ISL_584617 to 584620, EPI_ISL_612190 to 612191, EPI_ISL_612401 to 612449, EPI_ISL_627445 to 627449, EPI_ISL_638449 to 638453, EPI_ISL_639002, EPI_ISL_651095, EPI_ISL_651429 to 651430, EPI_ISL_652073 to 652102, EPI_ISL_665113 to 665142, EPI_ISL_666022, EPI_ISL_679391, EPI_ISL_704903, EPI_ISL_704905, EPI_ISL_705423 to 705424, EPI_ISL_705471, EPI_ISL_705806 to 705892, EPI_ISL_705894 to 705941, EPI_ISL_724356 to 724357, EPI_ISL_724361, EPI_ISL_724364 to 724370, EPI_ISL_724372, EPI_ISL_724378, EPI_ISL_724380, EPI_ISL_724382 to 724384, EPI_ISL_724387 to 724389, EPI_ISL_724394, EPI_ISL_724410, EPI_ISL_724412 to 724413, EPI_ISL_724415, EPI_ISL_724418, EPI_ISL_724427, EPI_ISL_724430 to 724431, EPI_ISL_724434, EPI_ISL_724436, EPI_ISL_724438, EPI_ISL_724442 to 724444, EPI_ISL_724446, EPI_ISL_724448 to 724449, EPI_ISL_724451 to 724452, EPI_ISL_724454, EPI_ISL_724458, EPI_ISL_724460, EPI_ISL_724463 to 724465, EPI_ISL_724467 to 724468, EPI_ISL_724472, EPI_ISL_724474, EPI_ISL_724479, EPI_ISL_724481 to 724483, EPI_ISL_724487, EPI_ISL_724489 to 724490, EPI_ISL_724496, EPI_ISL_724499, EPI_ISL_724501 to 724503, EPI_ISL_724507, EPI_ISL_724510, EPI_ISL_724514, EPI_ISL_724516 to 724517, EPI_ISL_724519, EPI_ISL_724522, EPI_ISL_813826, EPI_ISL_838459, EPI_ISL_838464, EPI_ISL_838531, EPI_ISL_838534, EPI_ISL_838564, EPI_ISL_838586, EPI_ISL_838590, EPI_ISL_838594, EPI_ISL_838600 to 838602, EPI_ISL_838604, EPI_ISL_838608, EPI_ISL_838611, EPI_ISL_838621, EPI_ISL_838624, EPI_ISL_838644, EPI_ISL_838656, EPI_ISL_838658, EPI_ISL_865179, EPI_ISL_865183, EPI_ISL_865193, EPI_ISL_865218, EPI_ISL_865223, EPI_ISL_865238, EPI_ISL_865246, EPI_ISL_865248, EPI_ISL_865252, EPI_ISL_865259, EPI_ISL_865289 to 865290, EPI_ISL_865294, EPI_ISL_865296, EPI_ISL_865298, EPI_ISL_865300, EPI_ISL_865311, EPI_ISL_865337, EPI_ISL_865347, EPI_ISL_865349, EPI_ISL_865377, EPI_ISL_865385, EPI_ISL_865387, EPI_ISL_865398, EPI_ISL_865431, EPI_ISL_865470, EPI_ISL_919460, EPI_ISL_919512, EPI_ISL_919515, EPI_ISL_919519, EPI_ISL_919526, EPI_ISL_919531, EPI_ISL_919539, EPI_ISL_919549, EPI_ISL_919554, EPI_ISL_919568, EPI_ISL_919579, EPI_ISL_919587, EPI_ISL_919624, EPI_ISL_919626, EPI_ISL_919714, EPI_ISL_949656 to 949658, EPI_ISL_949661, EPI_ISL_949664, EPI_ISL_949666, EPI_ISL_949668, EPI_ISL_949671, EPI_ISL_949673, EPI_ISL_949676, EPI_ISL_949680, EPI_ISL_997401, EPI_ISL_997429, EPI_ISL_997437, EPI_ISL_997454, EPI_ISL_997461, EPI_ISL_997463, EPI_ISL_997466, EPI_ISL_997512 to 997514, EPI_ISL_997517, EPI_ISL_997524, EPI_ISL_997557, EPI_ISL_997562			
see above	Liverpool Clinical Laboratories	COVID-19 Genomics UK (COG-UK) Consortium	A Alrezaihi; Alessandro Gerada; Alistair Darby; Angela Cowell; Anita Lucaci; Anu Chawla; Cassie Olateju; Catherine Hartley; Charlotte Nelson; Ecaterina Vamos; Elaine O'Toole; Elaine O'ÄôToole; Eleanor G Bentley; Ghada T Shawli; Isabel García-Dorival; Isabel Garca-Dorival; James Johnson; James P Stewart; Jenifer Manson; Joanne Watts; Jones Benjamin; Jordan J Clark; Julian Hiscox; L Luu; Lucille Rainbow; M Almsaud; Margaret Hughes; Mark Whitehead; Matthew Gemmell; Miren Iturriza-Gomara; Muhannad Alruwaili; N.P Randle; Neil Swainston; PKF Gilmore; Parul Sharma; Rebekah Penrice-Randal; Rebekah Penrice-Randal¬†; Richard Eccles; Richard Gregory; Sam Haldenby; Steve Paterson; Stuart D Armstrong; Trevor Ian Robinson; Ximeng Han
EPI_ISL_632050	Long Island Jewish Medical Center	New York City Public Health Laboratory	Jade Wang; et al.
EPI_ISL_708182 to 708183, EPI_ISL_710099 to 710104, EPI_ISL_710106 to 710109, EPI_ISL_710112 to 710113, EPI_ISL_710116, EPI_ISL_710430 to 710432, EPI_ISL_737236 to 737241, EPI_ISL_737243 to 737249, EPI_ISL_737251 to 737256, EPI_ISL_737258 to 737260, EPI_ISL_754130 to 754132, EPI_ISL_765998 to 766007, EPI_ISL_766009 to 766014, EPI_ISL_962857			
see above	Los Angeles County PHL	Los Angeles County PHL	P. Hemarajata et al.
EPI_ISL_694046, EPI_ISL_707693 to 707695, EPI_ISL_707885 to 707886, EPI_ISL_707888, EPI_ISL_707903 to 707927, EPI_ISL_707929 to 707932, EPI_ISL_708505, EPI_ISL_708507, EPI_ISL_708724 to 708727, EPI_ISL_708729 to 708730, EPI_ISL_708732 to 708734, EPI_ISL_735374 to 735383			
see above	Los Angeles County Public Health Laboratory	Los Angeles County Public Health Laboratory	P. Hemarajata et al.; Peera Hemarajata et al.
EPI_ISL_436044	Louisiana Office of Public Health Laboratories	Pathogen Discovery, Respiratory Viruses Branch, Division of Viral Diseases, Centers for Disease Control and Prevention	Anna Uehara; Bettina Bankamp; Clinton R. Paden; Haibin Wang; Jing Zhang; Krista Queen; Suxiang Tong; Yan Li; Ying Tao; Zachary Weiner
EPI_ISL_1008209	Ludwig Boltzmann Institut für Experimentelle und Klinische Traumatologie, Lorenz Böhler UKH	Bergthaler laboratory, CeMM Research Center for Molecular Medicine of the Austrian Academy of Sciences	Andreas Bergthaler; Anna Schedl; Bekir Erguner; Benedikt Agerer; Christoph Bock; Jan Laine; Lukas Endler; Maelle Le Moing; Martin Senekowitsch; Michael Schuster; Thomas Penz
EPI_ISL_434658	Lundens VC	The Public Health Agency of Sweden	Anna Risberg; Anna-Malin Linde; Karin Tegmark-Wisell; Maria Lind Karlberg; Marita Dagner; Mia Brytting; Olov Svartstrom; Oskar Karlsson Lindsjo; Theresa Enkirch
EPI_ISL_470790 to 470800, EPI_ISL_477277 to 477290, EPI_ISL_481241, EPI_ISL_509686, EPI_ISL_514629 to 514635, EPI_ISL_515267 to 515268, EPI_ISL_530163 to 530168, EPI_ISL_560798 to 560805, EPI_ISL_644944, EPI_ISL_683678 to 683688, EPI_ISL_708504, EPI_ISL_802573, EPI_ISL_911531, EPI_ISL_982347			
see above	M Health Fairview	Minnesota Department of Health, Public Health Laboratory	Alexandra Lorentz; Jacob Garfin; Kelly Pung; Matt Plumb; and Xiong Wang
EPI_ISL_443183, EPI_ISL_443253 to 443256, EPI_ISL_445077	M Health Fairview	University of Minnesota Genomics Center	Andrew Nelson; Benjamin Auch; Daryl M. Gohl; Jerry Daniel; John Garbe; Patrick Grady; Ray Watson; Sophia Yohe; and Kenneth B. Beckman

EPI_ISL_514637, EPI_ISL_514643, EPI_ISL_514650, EPI_ISL_514652, EPI_ISL_514657	M Health Fairview St. Joseph's Hospital	Minnesota Department of Health, Public Health Laboratory	Jacob Garfin; Matt Plumb; and Xiong Wang
EPI_ISL_451149 to 451153, EPI_ISL_451666	M.P Shah Government Medocal college Jamnagar	Gujarat Biotechnology Research Centre	; A M Kadri; Afzal Ansari; Akanksha Verma; Amit Kanani; Ankit Hinsu; Apurvasinh Puvar; Bhavesh Modi; Binita Aring; Chaitanya Joshi; Dinesh Kumar; Fenil Patel; Gaurishankar Shrimali; Janvi Raval; Komal Patel; Labdhi Pandya; Madhvi Joshi; Maharshi Pandya; Monika Gandhi; Neha Rajpara; Nidhi Patel; Nitin Savaliya; Pinal Trivedi; Pragya Sharma; Pritesh Sabara; Priti Pandita; R D Dixit; Raghawendra Kumar; Ramesh Pandit; Snehal Bagatharia; Tejas Shah; Zarna Patel; Zuber Saiyed
EPI_ISL_424843 to 424847, EPI_ISL_424908 to 424920	MA State Public Health Laboratory	Pathogen Discovery, Respiratory Viruses Branch, Division of Viral Diseases, Centers for Disease Control and Prevention	Alison S. Laufer Halpin; Anna Uehara; Christopher A. Elkins; Clinton R. Paden; Haibin Wang; Jing Zhang; Krista Queen; Mary S. Keckler; Rachel Marine; Suxiang Tong; Yan Li; Ying Tao
EPI_ISL_751559, EPI_ISL_751577, EPI_ISL_751604, EPI_ISL_751606 to 751607, EPI_ISL_751625, EPI_ISL_751628 to 751629, EPI_ISL_751700, EPI_ISL_751702 to 751703, EPI_ISL_751725, EPI_ISL_751771			
see above	MD DOH Laboratories Administration	Genomics and Discovery, Respiratory Viruses Branch, Division of Viral Diseases, Centers for Disease Control and Prevention	Anna Montmayeur; Anna Uehara; Clinton R. Paden; Haibin Wang; Jing Zhang; Justin Lee; Krista Queen; Mili Sheth; Peter W. Cook; Rachel Marine; Suxiang Tong; Yan Li; Ying Tao
EPI_ISL_424869, EPI_ISL_426425, EPI_ISL_447843	MD DOH Laboratories Administration	Pathogen Discovery, Respiratory Viruses Branch, Division of Viral Diseases, Centers for Disease Control and Prevention	Alison S. Laufer Halpin; Anna Uehara; Christopher A. Elkins; Clinton R. Paden; Haibin Wang; Jasmine Padilla; Jing Zhang; Justin Lee; Krista Queen; Mary S. Keckler; Rachel Marine; Suxiang Tong; Yan Li; Ying Tao
EPI_ISL_524873, EPI_ISL_524875 to 524882, EPI_ISL_524884 to 524901, EPI_ISL_534699 to 534706, EPI_ISL_534708 to 534711, EPI_ISL_534713, EPI_ISL_534716, EPI_ISL_591022 to 591032, EPI_ISL_591034 to 591036, EPI_ISL_602258 to 602277, EPI_ISL_633083 to 633090, EPI_ISL_633092 to 633099, EPI_ISL_648271 to 648281, EPI_ISL_648283 to 648285, EPI_ISL_648287 to 648302, EPI_ISL_660071 to 660105, EPI_ISL_942352			
see above	MD PHL	MD PHL	Maryland Department of Health Laboratories Administration
EPI_ISL_736927, EPI_ISL_736995	MDS	KRISP, KZN Research Innovation and Sequencing Platform	ChimukangaraB; Giandhari J; Khan S; Lessells R; Mdlalose K; Pillay S; Tegally H; Wilkinson E; York D; de Oliveira T
EPI_ISL_594134, EPI_ISL_594144, EPI_ISL_594147, EPI_ISL_603040, EPI_ISL_603047 to 603048	MDU-PHL, The Peter Doherty Institute for Infection and Immunity	MDU-PHL, The Peter Doherty Institute for Infection and Immunity	Caly, L.; Druce, J.; M.B.; M.L.; N.L.; Sait; Sait, M.; Schultz; Seemann, T.; Sherry; Sherry, N.
EPI_ISL_445311, EPI_ISL_445328	MEGASALUD S.A.	Instituto de Salud Publica de Chile	Alejandra Acevedo; Andrés E Castillo; Bárbara Parra; Carolina Tambley; Gabriel Leal; Jaime Lagos; Jorge Fernandez; Loredana Arata; Patricia Bustos; Paz Tapia; Rodrigo Fasce; Winston Andrade
EPI_ISL_445263, EPI_ISL_445358	MEGASALUD SPA.	Instituto de Salud Publica de Chile	Alejandra Acevedo; Andrés E Castillo; Bárbara Parra; Carolina Tambley; Gabriel Leal; Jaime Lagos; Jorge Fernandez; Loredana Arata; Patricia Bustos; Paz Tapia; Rodrigo Fasce; Winston Andrade
EPI_ISL_767596 to 767597, EPI_ISL_767629 to 767647, EPI_ISL_767655 to 767664, EPI_ISL_767681 to 767715	MEMORIAL SLOAN KETTERING CANCER CENTER	Wadsworth Center, New York State Department.of Health	Alexis Russel; Daryl M. Lamson; Erasmus Schneider; Erica Lasek-Nesselquist; John Kelly; Jonathan Plitnick; Kirsten St. George; Matthew Shudt; Melissa A Leisner; Navjot Singh; Sara Griesemer
EPI_ISL_982872 to 982889, EPI_ISL_982905 to 982907	MEPHI Aix Marseille University (AMU)	MEPHI Aix Marseille University (AMU)	Anthony LEVASSEUR
EPI_ISL_568877 to 568899, EPI_ISL_568901 to 568912, EPI_ISL_568914 to 568922, EPI_ISL_568924 to 568936, EPI_ISL_568939 to 568942, EPI_ISL_568944 to 568945, EPI_ISL_568947 to 568956, EPI_ISL_568958 to 568984, EPI_ISL_568986, EPI_ISL_568992 to 568998, EPI_ISL_569000, EPI_ISL_569002 to 569006, EPI_ISL_569008 to 569029, EPI_ISL_569031 to 569038, EPI_ISL_569041, EPI_ISL_569043 to 569051, EPI_ISL_569053 to 569058, EPI_ISL_569060 to 569064, EPI_ISL_569067 to 569068, EPI_ISL_569070 to 569082, EPI_ISL_569084 to 569094, EPI_ISL_569096 to 569111, EPI_ISL_569113 to 569117, EPI_ISL_569119 to 569121, EPI_ISL_569123 to 569130, EPI_ISL_569132 to 569147, EPI_ISL_569149 to 569151, EPI_ISL_569155 to 569162, EPI_ISL_569164 to 569166, EPI_ISL_569168 to 569193, EPI_ISL_569195 to 569205, EPI_ISL_569207 to 569208, EPI_ISL_569210 to 569211, EPI_ISL_569214, EPI_ISL_569216 to 569217, EPI_ISL_569219 to 569223, EPI_ISL_569225 to 569241, EPI_ISL_569243 to 569244, EPI_ISL_569246 to 569259, EPI_ISL_569261 to 569282, EPI_ISL_569284 to 569285, EPI_ISL_569288, EPI_ISL_569290 to 569305, EPI_ISL_569307 to 569318, EPI_ISL_569321, EPI_ISL_569323 to 569341, EPI_ISL_569343 to 569348, EPI_ISL_569350 to 569375, EPI_ISL_569377 to 569384, EPI_ISL_569386 to 569395, EPI_ISL_569397 to 569408, EPI_ISL_569410 to 569429, EPI_ISL_569431 to 569438, EPI_ISL_569440 to 569441, EPI_ISL_569443 to 569451, EPI_ISL_569456 to 569479, EPI_ISL_569481, EPI_ISL_569483 to 569485, EPI_ISL_569489 to 569538, EPI_ISL_569540 to 569578, EPI_ISL_569582 to 569590, EPI_ISL_569592 to 569595, EPI_ISL_569597 to 569608, EPI_ISL_644392 to 644397, EPI_ISL_644399 to 644401, EPI_ISL_644403 to 644564, EPI_ISL_896158, EPI_ISL_982232, EPI_ISL_1020349 to 1020372, EPI_ISL_1020388			
see above	MEPHI, Aix Marseille University	MEPHI, Aix Marseille University	Anthony LEVASSEUR
EPI_ISL_856708 to 856710	MFD Veselbas centrs "Dziedniecba"	Latvian Biomedical Research and Study Centre	Ivars Silamielis; Iveta Bere; Jnis Kloviš; Jnis Pjalkovskis; Kaspars Megnis; Marija Donaka; Monta Ustinova; Uga Dumpis; Vita Rovte; ikita Zrelovs
EPI_ISL_413571	MHC Brabant Zuidoost	Erasmus Medical Center	Anne van der Linden; Annemiek van der Eijk; Aura Timen; Bas Oude Munnink; Claudia Schapendonk; Corien Swaan; Corine GeurtsvanKessel; David Nieuwenhuijse; Irina Chestakova; Jeroen van Kampen; Jolanda Voermans; Madelief Mollers; Manon Haverkate; Marion Koopmans; Mark Pronk; Mart Stein; Pascal Lexmond; Reina Sikkema; Richard Molenkamp; Sandra Kengne Kamga Mobou; on behalf of the Dutch national COVID-19 response team.
EPI_ISL_413568	MHC Drente	Erasmus Medical Center	Anne van der Linden; Annemiek van der Eijk; Aura Timen; Bas Oude Munnink; Claudia Schapendonk; Corien Swaan; Corine GeurtsvanKessel; David Nieuwenhuijse; Irina Chestakova; Jeroen van Kampen; Jolanda Voermans; Madelief Mollers; Manon Haverkate; Marion Koopmans; Mark Pronk; Mart Stein; Pascal Lexmond; Reina Sikkema; Richard Molenkamp; Sandra Kengne Kamga Mobou; on behalf of the Dutch national COVID-19 response team.
EPI_ISL_413591	MHC Flevoland	Erasmus Medical Center	Anne van der Linden; Annemiek van der Eijk; Aura Timen; Bas Oude Munnink; Claudia Schapendonk; Corien Swaan; Corine GeurtsvanKessel; David Nieuwenhuijse; Irina Chestakova; Jeroen van Kampen; Jolanda Voermans; Madelief Mollers; Manon Haverkate; Marion Koopmans; Mark Pronk; Mart Stein; Pascal Lexmond; Reina Sikkema; Richard Molenkamp; Sandra Kengne Kamga Mobou; on behalf of the Dutch national COVID-19 response team.
EPI_ISL_413566, EPI_ISL_413577	MHC Gooi & Vechtstreek	Erasmus Medical Center	Anne van der Linden; Annemiek van der Eijk; Aura Timen; Bas Oude Munnink; Claudia Schapendonk; Corien Swaan; Corine GeurtsvanKessel; David Nieuwenhuijse; Irina Chestakova; Jeroen van Kampen; Jolanda Voermans; Madelief Mollers; Manon Haverkate; Marion Koopmans; Mark Pronk; Mart Stein; Pascal Lexmond; Reina Sikkema; Richard Molenkamp; Sandra Kengne Kamga Mobou; on behalf of the Dutch national COVID-19 response team.
EPI_ISL_413579	MHC Haaglanden	Erasmus Medical Center	Anne van der Linden; Annemiek van der Eijk; Aura Timen; Bas Oude Munnink; Claudia Schapendonk; Corien Swaan; Corine GeurtsvanKessel; David Nieuwenhuijse; Irina Chestakova; Jeroen van Kampen; Jolanda Voermans; Madelief Mollers; Manon Haverkate; Marion Koopmans; Mark Pronk; Mart Stein; Pascal Lexmond; Reina Sikkema; Richard Molenkamp; Sandra Kengne Kamga Mobou; on behalf of the Dutch national COVID-19 response team.
EPI_ISL_413580	MHC Hart voor Brabant	Erasmus Medical Center	Anne van der Linden; Annemiek van der Eijk; Aura Timen; Bas Oude Munnink; Claudia Schapendonk; Corien Swaan; Corine GeurtsvanKessel; David Nieuwenhuijse; Irina Chestakova; Jeroen van Kampen; Jolanda Voermans; Madelief Mollers; Manon Haverkate; Marion Koopmans; Mark Pronk; Mart Stein; Pascal Lexmond; Reina Sikkema; Richard Molenkamp; Sandra Kengne Kamga Mobou; on behalf of the Dutch national COVID-19 response team.
EPI_ISL_413572	MHC Kennemerland	Erasmus Medical Center	Anne van der Linden; Annemiek van der Eijk; Aura Timen; Bas Oude Munnink; Claudia Schapendonk; Corien Swaan; Corine GeurtsvanKessel; David Nieuwenhuijse; Irina Chestakova; Jeroen van Kampen; Jolanda Voermans; Madelief Mollers; Manon Haverkate; Marion Koopmans; Mark Pronk; Mart Stein; Pascal Lexmond; Reina Sikkema; Richard Molenkamp; Sandra Kengne Kamga Mobou; on behalf of the Dutch national COVID-19 response team.
EPI_ISL_413583	MHC Rotterdam-Rijnmond	Erasmus Medical Center	Anne van der Linden; Annemiek van der Eijk; Aura Timen; Bas Oude Munnink; Claudia Schapendonk; Corien Swaan; Corine GeurtsvanKessel; David Nieuwenhuijse; Irina Chestakova; Jeroen van Kampen; Jolanda Voermans; Madelief Mollers; Manon Haverkate; Marion Koopmans; Mark Pronk; Mart Stein; Pascal Lexmond; Reina Sikkema; Richard Molenkamp; Sandra Kengne Kamga Mobou; on behalf of the Dutch national COVID-19 response team.
EPI_ISL_413588 to 413590	MHC Utrecht	Erasmus Medical Center	Anne van der Linden; Annemiek van der Eijk; Aura Timen; Bas Oude Munnink; Claudia Schapendonk; Corien Swaan; Corine GeurtsvanKessel; David Nieuwenhuijse; Irina Chestakova; Jeroen van Kampen; Jolanda Voermans; Madelief Mollers; Manon Haverkate; Marion Koopmans; Mark Pronk; Mart Stein; Pascal Lexmond; Reina Sikkema; Richard Molenkamp; Sandra Kengne Kamga Mobou; on behalf of the Dutch national COVID-19 response team.
EPI_ISL_413564, EPI_ISL_413574	MHC West-Brabant	Erasmus Medical Center	Anne van der Linden; Annemiek van der Eijk; Aura Timen; Bas Oude Munnink; Claudia Schapendonk; Corien Swaan; Corine GeurtsvanKessel; David Nieuwenhuijse; Irina Chestakova; Jeroen van Kampen; Jolanda Voermans; Madelief Mollers; Manon Haverkate; Marion Koopmans; Mark Pronk; Mart Stein; Pascal Lexmond; Reina Sikkema; Richard Molenkamp; Sandra Kengne Kamga Mobou; on behalf of the Dutch national COVID-19 response team.
EPI_ISL_751601, EPI_ISL_751622, EPI_ISL_751769	MI - Michigan Department of Health and Human Services - Bureau of Laboratories	Genomics and Discovery, Respiratory Viruses Branch, Division of Viral Diseases, Centers for Disease Control and Prevention	Anna Montmayeur; Anna Uehara; Clinton R. Paden; Haibin Wang; Jing Zhang; Justin Lee; Krista Queen; Mili Sheth; Peter W. Cook; Rachel Marine; Suxiang Tong; Yan Li; Ying Tao
EPI_ISL_486394, EPI_ISL_486398 to 486399,	MIMS	Department of Neurovirology, National Institute of Mental	Anita Desai; Chitra Pattabiraman; Harsha PK; Manjunatha Venkataswamy; Pramada Prasad; Ravi Vasanthapuram; Risha Rasheed; Shafeeq S Hameed;

EPI_ISL_515968 to 515973		Health and Neuroscience (NIMHANS)	Vijayalakshmi Reddy
EPI_ISL_710592 to 710593	MLF Care AB	The Public Health Agency of Sweden	Department of Microbiology; The Public Health Agency of Sweden
EPI_ISL_452108, EPI_ISL_452113, EPI_ISL_452133, EPI_ISL_594463	MN Department of Health	Pathogen Discovery, Respiratory Viruses Branch, Division of Viral Diseases, Centers for Disease Control and Prevention	Alison S. Laufer Halpin; Anna Montmayeur; Anna Uehara; Christopher A. Elkins; Clinton Paden; Clinton R. Paden; Haibin Wang; Jing Zhang; Julu Bhatnagar; Krista Queen; Mary S. Keckler; Rachel Marine; Suxiang Tong; Yan Li; Ying Tao; Zachary Weiner
EPI_ISL_751573, EPI_ISL_751576, EPI_ISL_751641, EPI_ISL_751653 to 751655, EPI_ISL_751660, EPI_ISL_751716, EPI_ISL_751733, EPI_ISL_751739			
see above	MN PHL Division, Minnesota Department of Health	Genomics and Discovery, Respiratory Viruses Branch, Division of Viral Diseases, Centers for Disease Control and Prevention	Anna Montmayeur; Anna Uehara; Clinton R. Paden; Haibin Wang; Jing Zhang; Justin Lee; Krista Queen; Mili Sheth; Peter W. Cook; Rachel Marine; Suxiang Tong; Yan Li; Ying Tao
EPI_ISL_426426 to 426427, EPI_ISL_527667 to 527736	MN PHL Division, Minnesota Department of Health	Pathogen Discovery, Respiratory Viruses Branch, Division of Viral Diseases, Centers for Disease Control and Prevention	Alison S. Laufer Halpin; Anna Montmayeur; Anna Uehara; Brian Lynch; Christopher A. Elkins; Clinton R. Paden; Haibin Wang; Jing Zhang; Krista Queen; Mary S. Keckler; Rachel Marine; Suxiang Tong; Yan Li; Ying Tao
EPI_ISL_751634 to 751635, EPI_ISL_751656 to 751657, EPI_ISL_751715, EPI_ISL_751737, EPI_ISL_751740, EPI_ISL_751774	MO State Public Health Laboratory	Genomics and Discovery, Respiratory Viruses Branch, Division of Viral Diseases, Centers for Disease Control and Prevention	Anna Montmayeur; Anna Uehara; Clinton R. Paden; Haibin Wang; Jing Zhang; Justin Lee; Krista Queen; Mili Sheth; Peter W. Cook; Rachel Marine; Suxiang Tong; Yan Li; Ying Tao
EPI_ISL_424870	MO State Public Health Laboratory	Pathogen Discovery, Respiratory Viruses Branch, Division of Viral Diseases, Centers for Disease Control and Prevention	Alison S. Laufer Halpin; Anna Uehara; Christopher A. Elkins; Clinton R. Paden; Haibin Wang; Jing Zhang; Krista Queen; Mary S. Keckler; Rachel Marine; Suxiang Tong; Yan Li; Ying Tao
EPI_ISL_903383	MOH - Jaber Al-Ahmad Hospital (Innovation Research Laboratory)	MOH - Jaber Al-Ahmad Hospital (Innovation Research Laboratory)	Mohammad Alghounaim; Salman Al-Sabah
EPI_ISL_1016175 to 1016176, EPI_ISL_1016198, EPI_ISL_1016205	MONTEFIORE MEDICAL CENTER LABORATORIES	Wadsworth Center, New York State Department of Health	Alexis Russel; Daryl M. Lamson; Erasmus Schneider; Erica Lasek-Nesselquist; John Kelly; Jonathan Plitnick; Kirsten St. George; Matthew Shudt; Melissa A Leisner; Navjot Singh
EPI_ISL_765490 to 765491, EPI_ISL_765530 to 765566	MONTEFIORE MEDICAL CENTER LABORATORIES	Wadsworth Center, New York State Department.of Health	Alexis Russel; Daryl M. Lamson; Erasmus Schneider; Erica Lasek-Nesselquist; John Kelly; Jonathan Plitnick; Kirsten St. George; Matthew Shudt; Melissa A Leisner; Navjot Singh; Sara Griesemer
EPI_ISL_428856	MRCG at LSHTM Genomics Lab	MRCG at LSHTM Genomics lab	Sesay et al
EPI_ISL_428857, EPI_ISL_471158, EPI_ISL_471163 to 471164, EPI_ISL_471167, EPI_ISL_471171, EPI_ISL_560992, EPI_ISL_560994 to 560995, EPI_ISL_560997, EPI_ISL_561000 to 561001, EPI_ISL_561003 to 561004, EPI_ISL_561006, EPI_ISL_561011 to 561013, EPI_ISL_561021 to 561022, EPI_ISL_561034, EPI_ISL_561038, EPI_ISL_561040 to 561044, EPI_ISL_561046 to 561049, EPI_ISL_561051 to 561056, EPI_ISL_561058 to 561071, EPI_ISL_561073 to 561077, EPI_ISL_561079 to 561091, EPI_ISL_561093 to 561100, EPI_ISL_561102, EPI_ISL_561105, EPI_ISL_561107 to 561108, EPI_ISL_561110 to 561125, EPI_ISL_561127, EPI_ISL_561129 to 561146, EPI_ISL_561148, EPI_ISL_561150 to 561151, EPI_ISL_561153 to 561160, EPI_ISL_561162 to 561174, EPI_ISL_561176 to 561182, EPI_ISL_561184 to 561185, EPI_ISL_561187 to 561191, EPI_ISL_561193 to 561196, EPI_ISL_561198 to 561206, EPI_ISL_561212 to 561213, EPI_ISL_561215, EPI_ISL_561218, EPI_ISL_561222 to 561223, EPI_ISL_561229 to 561230, EPI_ISL_561232, EPI_ISL_561236 to 561245, EPI_ISL_561247 to 561249, EPI_ISL_561251, EPI_ISL_561257, EPI_ISL_561260, EPI_ISL_561263, EPI_ISL_561268, EPI_ISL_561270, EPI_ISL_561273, EPI_ISL_561277, EPI_ISL_561279, EPI_ISL_561282 to 561285, EPI_ISL_561287 to 561300, EPI_ISL_561303 to 561311, EPI_ISL_561313 to 561315, EPI_ISL_561324, EPI_ISL_561327, EPI_ISL_561330, EPI_ISL_561332 to 561333, EPI_ISL_915421			
see above	MRCG at LSHTM Genomics lab	MRCG at LSHTM Genomics lab	Abdoulie Kanteh; Abdul Karim sesay; Bakary Sanyang; Jarra Manneh; Mariama Kujabi; Sesay et al
EPI_ISL_428855	MRCG at LSHTM Geomics lab	MRCG at LSHTM Genomics lab	Sesay et al
EPI_ISL_751661, EPI_ISL_751679, EPI_ISL_751683, EPI_ISL_751776	MS Public Health Laboratory	Genomics and Discovery, Respiratory Viruses Branch, Division of Viral Diseases, Centers for Disease Control and Prevention	Anna Montmayeur; Anna Uehara; Clinton R. Paden; Haibin Wang; Jing Zhang; Justin Lee; Krista Queen; Mili Sheth; Peter W. Cook; Rachel Marine; Suxiang Tong; Yan Li; Ying Tao
EPI_ISL_648004 to 648034, EPI_ISL_648037 to 648042	MS Public Health Laboratory	Pathogen Discovery, Respiratory Viruses Branch, Division of Viral Diseases, Centers for Disease Control and Prevention	Anna Montmayeur; Anna Uehara; Brian Lynch; Clinton R. Paden; Haibin Wang; Jing Zhang; Krista Queen; Rachel Marine; Suxiang Tong; Yan Li; Ying Tao
EPI_ISL_414476, EPI_ISL_415151, EPI_ISL_421348 to 421355, EPI_ISL_421357 to 421360, EPI_ISL_421363 to 421367, EPI_ISL_421369 to 421371, EPI_ISL_421375 to 421376, EPI_ISL_421378 to 421379, EPI_ISL_421383 to 421391, EPI_ISL_421394 to 421417, EPI_ISL_421419 to 421435, EPI_ISL_421593 to 421600, EPI_ISL_421602 to 421608, EPI_ISL_421611 to 421617, EPI_ISL_421619 to 421621, EPI_ISL_421623 to 421624, EPI_ISL_421626 to 421635, EPI_ISL_422488 to 422492, EPI_ISL_422494 to 422511, EPI_ISL_422513 to 422517, EPI_ISL_422519 to 422525, EPI_ISL_422527 to 422537, EPI_ISL_422539 to 422544, EPI_ISL_422546 to 422562, EPI_ISL_450004 to 450056, EPI_ISL_450058 to 450070, EPI_ISL_450072, EPI_ISL_450074 to 450082, EPI_ISL_450086 to 450087, EPI_ISL_450089 to 450118, EPI_ISL_450120 to 450121, EPI_ISL_450123 to 450130, EPI_ISL_450132 to 450170, EPI_ISL_802168, EPI_ISL_882805			
see above	MSHS Clinical Microbiology Laboratories	MSHS Pathogen Surveillance Program	Adolfo Garcia-Sarstre; Adolfo Garcia-Sastre; Adolfo García-Sastre; Adriana van de Guchte; Ajay Obla; Alberto Paniz-Mondolfi; Alberto Paniz-mondolfi; Ana S. Gonzalez-Reiche; Ana Silvia Gonzalez-Reiche; Ana Sylvia Gonzalez-Reiche; Andrew Kasarskis; Angela Amoako; Ashley S. Salimbangon; Betsaida Salom Melo; Bremy Albuquerque; Bremy Alburquerque; Brianne Ciferri; Charles Gleason; Deena R. Altman; Denise Jurczyszak; Elena Hirsch; Emilia Mia Sordillo; Emilia Sordillo; Emily Ferreri; Florian Krammer; Gintaras Deikus; Giulio Kleiner; Gopi Patel; Hala Alshammary; Harm van Bakel; Irina Oussenko; Jayeeta Dutta; Jose Polanco; Juan Soto; Judith Aberg; Katherine Beach; Kathryn Twyman; Kayla Russo; Komal Srivastava; Levy Sominsky; Lisa Miorin; Mahmoud Awawda; Marta Luksza; Matthew Hernandez; Matthew M. Hernandez; Melissa Gitman; Melissa Smith; Michael D. Nowak; Mitchell J. Sullivan; Mitchell Sullivan; Nancy Francoeur; Rachel Chernet; Randy Albrecht; Robert Sebra; Sarah Schaefer; Shelcie Fabre; Shwetha Hara Sridhar; Shwetha Sridhar Hara; Viviana Simon; Wen-chun Liu; Ying-Chih Wang; Zenab Khan
EPI_ISL_751561, EPI_ISL_751633, EPI_ISL_751640, EPI_ISL_751710 to 751711, EPI_ISL_751713, EPI_ISL_751738, EPI_ISL_751775			
see above	MT Public Health Laboratory	Genomics and Discovery, Respiratory Viruses Branch, Division of Viral Diseases, Centers for Disease Control and Prevention	Anna Montmayeur; Anna Uehara; Clinton R. Paden; Haibin Wang; Jing Zhang; Justin Lee; Krista Queen; Mili Sheth; Peter W. Cook; Rachel Marine; Suxiang Tong; Yan Li; Ying Tao
EPI_ISL_445309, EPI_ISL_445355	MUTUAL DE SEGURIDAD C.CH.C.	Instituto de Salud Publica de Chile	Alejandra Acevedo; Andrés E Castillo; Bárbara Parra; Carolina Tambley; Gabriel Leal; Jaime Lagos; Jorge Fernandez; Loredana Arata; Patricia Bustos; Paz Tapia; Rodrigo Fasce; Winston Andrade
EPI_ISL_631277 to 631303, EPI_ISL_632311	MVZ DIAMEDIS Diagnostische Medizin Sennestadt GmbH	Bielefeld University	Alexander Sczyrba; Christiane Scherer; David Brandt; Jörn Kalinowski; Levin-Joe Klages; Marina Simunovic; Markus Haak; Svenja Vinke; Tobias Busche
EPI_ISL_640219 to 640229, EPI_ISL_640231 to 640233, EPI_ISL_640235 to 640236, EPI_ISL_640238 to 640248, EPI_ISL_640251 to 640264, EPI_ISL_640268, EPI_ISL_640271 to 640272			
see above	MVZ Laborärzte Singen	MVZ Laborärzte Singen	Folker Wenzel; Frithjof Blessing; Jonas Schmidt; Sandro Berghaus
EPI_ISL_933568, EPI_ISL_934416	Macedonian Academy of Sciences and Arts	Research Center for Genetic Engineering and Biotechnology "Georgi D. Efremov" , Macedonian Academy of Sciences and Arts	RCGEB - MASA
EPI_ISL_648545 to 648554, EPI_ISL_672098 to 672102, EPI_ISL_672394 to 672422, EPI_ISL_672502 to 672530, EPI_ISL_738707 to 738708, EPI_ISL_738916, EPI_ISL_738964, EPI_ISL_739061, EPI_ISL_739133, EPI_ISL_739172, EPI_ISL_739197, EPI_ISL_739390, EPI_ISL_739393, EPI_ISL_739429, EPI_ISL_739539, EPI_ISL_739567			
see above	Madera County Department of Public Health	Chan-Zuckerberg Biohub	CZB Cliahub Consortium
EPI_ISL_508423 to 508440	Mahatma Gandhi Institute of Medical Sciences	National Institute of Biomedical Genomics	Arindam Maitra; Deepashri Maraskolhe; Rahul Narang; Saumitra Das; Vijayshri Deotale
EPI_ISL_511924, EPI_ISL_511926 to 511929, EPI_ISL_522437	Mahatma Gandhi Institute of Medical Sciences	National Institute of Biomedical Genomics - DBT's PAN-INDIA 1000 SARS--CoV-2 RNA Genome Sequencing Consortium	Arindam Maitra; Deepashri Maraskolhe; Rahul Narang; Saumitra Das; Vijayshri Deotale
EPI_ISL_614294, EPI_ISL_631528, EPI_ISL_631538, EPI_ISL_631546, EPI_ISL_631796, EPI_ISL_631863, EPI_ISL_631881, EPI_ISL_631915, EPI_ISL_631993			
see above	Maimonides Medical Center	New York City Public Health Laboratory	Jade Wang; et al.
EPI_ISL_513414 to 513416, EPI_ISL_513418 to 513444, EPI_ISL_513447 to 513486, EPI_ISL_513488 to 513510, EPI_ISL_966820	Maine HETL	Tewhey Lab, The Jackson Laboratory	Barter, M.; Dewey, H.; H. and Tewhey, R.; Iosue, F.; Lynch, R.; Matluk, N.; Munger
EPI_ISL_755306 to 755309, EPI_ISL_755311 to 755317, EPI_ISL_755319, EPI_ISL_755321, EPI_ISL_755323 to 755327, EPI_ISL_755329 to 755335, EPI_ISL_755337 to 755349, EPI_ISL_755352 to 755353, EPI_ISL_755355 to 755362, EPI_ISL_755365 to 755384, EPI_ISL_755386 to 755392, EPI_ISL_755394 to 755408, EPI_ISL_755410 to 755412, EPI_ISL_755414, EPI_ISL_755416, EPI_ISL_755418 to 755419, EPI_ISL_755421 to 755425, EPI_ISL_755427 to 755434, EPI_ISL_755436 to 755437, EPI_ISL_755439 to 755441, EPI_ISL_755443 to 755444, EPI_ISL_755446, EPI_ISL_755448 to 755453, EPI_ISL_755457 to 755458, EPI_ISL_755462 to 755463, EPI_ISL_755465 to 755481, EPI_ISL_755483 to 755488, EPI_ISL_755490 to 755500, EPI_ISL_755502 to 755503, EPI_ISL_755507 to 755514, EPI_ISL_755517 to 755520, EPI_ISL_755522 to 755541, EPI_ISL_755543 to 755549, EPI_ISL_755551, EPI_ISL_755553, EPI_ISL_755556 to 755567			

see above	Maine Health and Environmental Testing Laboratory	Tewhey Lab, The Jackson Laboratory	Barter, M.; Dewey, H.; H. and Tewhey, R.; Iosue, F.; Lynch, R.; Matluk, N.; Munger
EPI_ISL_428348	Maison de Santé du Val d'Ormois	National Reference Center for Viruses of Respiratory Infections, Institut Pasteur, Paris	Angela Brisebarre; Etienne Simon-Lorière; Flora Donati; Marion Barbet; Maud Vanpeene; Mélanie Albert; Méline Bizard; Sylvie Behillil; Sylvie van der Werf; Vincent Enouf
EPI_ISL_1027629 to 1027636, EPI_ISL_1027658 to 1027666	Mako Medical Laboratories	Mako Medical Laboratories	"Lauren Moon; Matthew Tugwell"
EPI_ISL_528738 to 528744, EPI_ISL_568873 to 568875, EPI_ISL_582124	Malaysia Genome Institute	Malaysia Genome Institute	Azrin Ahmad; Enizza Kasim; Gan Han Ming; Irni Suhayu Sapian; Mohd Faizal Abu Bakar; Mohd Noor Mat Isa; Nor Azfa Johari; Nurhezreen Md Iqbal; Shahrul Hisham Zainal Ariffin; Shamsidar Sopie; Siti Noraini Othman; Tan Ju Lin; Yusuf Muhammad Noor
EPI_ISL_640074	Mamre CDC wc MRC	NHLS/UCT	Arash Iranzadeh; Bruna Galvao; Carolyn Williamson; Deelan Doolabh; Diana Hardie; Innocent Mudau; Kruger Marais; Lynn Tyers; Marvin Hsiao; Stephen Korsman
EPI_ISL_583198	Marin County Public Health Department	Chan-Zuckerberg Biohub	CZB Cliahub Consortium
EPI_ISL_1005683	Martigues Hospital	Cerba	Benazra M; Haim-Boukobza S; Lecorche E; Negre V; Olivi M; Roquebert B; Trombert-Paolantoni S; Vidal B
EPI_ISL_1005682	Martigues hospital	Cerba	Benazra M; Haim-Boukobza S; Lecorche E; Negre V; Olivi M; Roquebert B; Trombert-Paolantoni S; Vidal B
EPI_ISL_955230	Mary Eliza Mahoney HC	NJ Public Health and Environmental Laboratories	Byeong Jeong; Dana Woell; Lindsey Bodnar; Shiv Verma
EPI_ISL_481265 to 481282, EPI_ISL_486912 to 486917, EPI_ISL_509444 to 509491, EPI_ISL_522824 to 522834, EPI_ISL_522836 to 522840, EPI_ISL_522842 to 522846, EPI_ISL_522848 to 522854, EPI_ISL_522869 to 522871			
see above	Maryland Department of Health	Maryland Department of Health	Keller, E.; Maryland Department of Health Laboratories Administration
EPI_ISL_454431, EPI_ISL_454435	Maryland Department of Health Laboratories Administration	Maryland Department of Health Laboratories Administration	MDH Laboratories Administration
EPI_ISL_529167 to 529168	Maryland Department of Health Laboratory	Maryland Department of Health Laboratory	Eric Keller; Maryland Department of Health Laboratories Administration
EPI_ISL_538265 to 538274, EPI_ISL_560806 to 560832, EPI_ISL_629036 to 629080, EPI_ISL_666771 to 666813, EPI_ISL_682060 to 682078, EPI_ISL_710090 to 710098, EPI_ISL_955107 to 955108, EPI_ISL_955110 to 955111, EPI_ISL_955118 to 955119, EPI_ISL_1015566 to 1015568, EPI_ISL_1015575, EPI_ISL_1015609 to 1015610, EPI_ISL_1015612 to 1015614, EPI_ISL_1015627, EPI_ISL_1038809, EPI_ISL_1038823, EPI_ISL_1038829, EPI_ISL_1038837			
see above	Maryland Public Health Laboratory	Maryland Public Health Laboratory	Maryland Department of Health Laboratories Administration
EPI_ISL_931488, EPI_ISL_931491 to 931493	Maryland Public Health Laboratory (MD PHL)	Maryland Public Health Laboratory (MD PHL)	Maryland Department of Health Laboratories Administration
EPI_ISL_700544	Masincedane Clinic wc MAS	NHLS/UCT	Arash Iranzadeh; Bruna Galvao; Carolyn Williamson; Deelan Doolabh; Diana Hardie; Innocent Mudau; Kruger Marais; Lynn Tyers; Marvin Hsiao; Stephen Korsman
EPI_ISL_676995 to 677025, EPI_ISL_677049 to 677076, EPI_ISL_677111 to 677129, EPI_ISL_734447 to 734481	Masonic Medical Research Institute	Wadsworth Center, New York State Department.of Health	Alexis Russel; Daryl M. Lamson; Erasmus Schneider; Erica Lasek-Nesselquist; John Kelly; Jonathan Plitnick; Kirsten St. George; Nathan Tucker; Navjot Singh; Ryan D. Pfeiffer; Sara Griesemer
EPI_ISL_409067	Massachusetts Department of Public Health	Pathogen Discovery, Respiratory Viruses Branch, Division of Viral Diseases, Centers for Dieases Control and Prevention	Anna Uehara; Brett L. Whitaker; Brian Lynch; Clinton R. Paden; Janna' R. Murray; Jing Zhang; Krista Queen; Lijuan Wang; Senthil Kumar K. Sakthivel; Shifaq Kamili; Stephen Lindstrom; Susan I. Gerber; Suxiang Tong; Xiaoyan Lu; Yan Li; Ying Tao
EPI_ISL_460097 to 460099, EPI_ISL_460101 to 460112, EPI_ISL_460114 to 460126, EPI_ISL_460128 to 460135, EPI_ISL_460137 to 460150, EPI_ISL_460153 to 460166, EPI_ISL_460168 to 460191, EPI_ISL_460195 to 460198, EPI_ISL_460201 to 460210, EPI_ISL_460212 to 460217, EPI_ISL_460220 to 460221, EPI_ISL_460223 to 460227, EPI_ISL_460229 to 460235, EPI_ISL_460237 to 460246, EPI_ISL_460248 to 460250, EPI_ISL_460252 to 460258, EPI_ISL_460261, EPI_ISL_460263 to 460286, EPI_ISL_460288 to 460302, EPI_ISL_460304 to 460317, EPI_ISL_460319, EPI_ISL_460321 to 460349, EPI_ISL_460351 to 460354, EPI_ISL_460356 to 460362, EPI_ISL_460364 to 460366, EPI_ISL_460368 to 460373, EPI_ISL_460376 to 460386, EPI_ISL_460388 to 460392, EPI_ISL_460394, EPI_ISL_460396 to 460400, EPI_ISL_460402 to 460404, EPI_ISL_460406 to 460410, EPI_ISL_460412 to 460415, EPI_ISL_460417 to 460422, EPI_ISL_460425 to 460432, EPI_ISL_460434 to 460437, EPI_ISL_460440 to 460448, EPI_ISL_460450 to 460451, EPI_ISL_460453 to 460472, EPI_ISL_765613 to 765631, EPI_ISL_765633 to 765634, EPI_ISL_765636 to 765718, EPI_ISL_765720 to 765730, EPI_ISL_765732 to 765752, EPI_ISL_765754 to 765769, EPI_ISL_765771 to 765783, EPI_ISL_765785 to 765798, EPI_ISL_765800 to 765803, EPI_ISL_765805 to 765817, EPI_ISL_765824 to 765840, EPI_ISL_765842 to 765858, EPI_ISL_765860 to 765887, EPI_ISL_765889, EPI_ISL_765892, EPI_ISL_765986			
see above	Massachusetts General Hospital	Infectious Disease Program, Broad Institute of Harvard and MIT	A.E.; Adams, G.; Anahtar, M.; B.L.; B.W.; Bauer, M.; Birren; Branda, J.; Carter, A.; Cerrato, F.; Chaluvadi, S.; Chapman; Cusick, C.; D.J.; DeRuff, K.; E. and Sabeti; Flowers, K.; Gallagher, G.; Gladden-Young, A.; Gnirke, A.; Harris, J.; J.E.; K.J.; LaRocque, R.; Lagerborg, K.; Lemieux; Lin; Loreth, C.; MacInnis; Neumann, A.; Normandin, E.; P.C.; Park; Pierce, V.; Reilly, S.; Rosenberg; Rosenberg, E.; Rudy, M.; Ryan, E.; S.B.; Sabeti; Shaw, B.; Siddle; Slater, D.; Smole, S.; Tomkins-Tinch, C.; Turbett, S.
EPI_ISL_692769 to 692774, EPI_ISL_692776, EPI_ISL_692778, EPI_ISL_692780, EPI_ISL_692782, EPI_ISL_692785, EPI_ISL_692787 to 692789, EPI_ISL_692792, EPI_ISL_692794 to 692796, EPI_ISL_692801, EPI_ISL_692809 to 692817, EPI_ISL_692819 to 692820, EPI_ISL_692822, EPI_ISL_692831 to 692832, EPI_ISL_692835 to 692839, EPI_ISL_692841 to 692842, EPI_ISL_692846 to 692848, EPI_ISL_692850 to 692851, EPI_ISL_692853, EPI_ISL_692855, EPI_ISL_692858, EPI_ISL_692860, EPI_ISL_692865, EPI_ISL_692867, EPI_ISL_692869 to 692871, EPI_ISL_692873 to 692874, EPI_ISL_692876, EPI_ISL_692878, EPI_ISL_692880, EPI_ISL_692886, EPI_ISL_692888, EPI_ISL_692890 to 692895, EPI_ISL_692897, EPI_ISL_692899 to 692901, EPI_ISL_692903, EPI_ISL_692909 to 692913, EPI_ISL_692915, EPI_ISL_692918 to 692919, EPI_ISL_692921 to 692924, EPI_ISL_692926 to 692931, EPI_ISL_692933, EPI_ISL_692935, EPI_ISL_692937, EPI_ISL_692939 to 692947, EPI_ISL_692949 to 692950, EPI_ISL_692952 to 692953, EPI_ISL_692956 to 692957, EPI_ISL_692961 to 692966, EPI_ISL_692968, EPI_ISL_692970, EPI_ISL_692972, EPI_ISL_692974 to 692979, EPI_ISL_692981, EPI_ISL_692984 to 692986, EPI_ISL_692989, EPI_ISL_692992, EPI_ISL_692996, EPI_ISL_693000, EPI_ISL_693003 to 693004, EPI_ISL_693006, EPI_ISL_693008 to 693009, EPI_ISL_693013, EPI_ISL_693016, EPI_ISL_693018 to 693019, EPI_ISL_693021, EPI_ISL_693023, EPI_ISL_693028, EPI_ISL_693030, EPI_ISL_693032, EPI_ISL_693036 to 693037, EPI_ISL_693039, EPI_ISL_693043, EPI_ISL_693045 to 693049, EPI_ISL_693051 to 693052, EPI_ISL_693058, EPI_ISL_693060, EPI_ISL_693062 to 693067, EPI_ISL_693069 to 693072, EPI_ISL_693074 to 693078, EPI_ISL_693080, EPI_ISL_693082 to 693084, EPI_ISL_693090 to 693091, EPI_ISL_693098 to 693099, EPI_ISL_693102 to 693103, EPI_ISL_693105 to 693107, EPI_ISL_693109 to 693110, EPI_ISL_693113 to 693115, EPI_ISL_693120 to 693121, EPI_ISL_693124 to 693128, EPI_ISL_693130 to 693131, EPI_ISL_693134, EPI_ISL_693137, EPI_ISL_693143 to 693147, EPI_ISL_693152, EPI_ISL_693158, EPI_ISL_693162, EPI_ISL_693167, EPI_ISL_693176 to 693179, EPI_ISL_693182, EPI_ISL_693188 to 693190, EPI_ISL_876163, EPI_ISL_913411, EPI_ISL_1009713, EPI_ISL_1009719 to 1009720, EPI_ISL_1009733, EPI_ISL_1009748			
see above	Massachusetts State Public Health Laboratory	Massachusetts State Public Health Laboratory	Andrew Lang; Glen Gallagher; Sandra Smole; Timelia Fink
EPI_ISL_418799, EPI_ISL_418801, EPI_ISL_420878 to 420879	Mater Pathology	Public Health Virology Laboratory	Alyssa Pyke; Amanda De Jong; Andrew Van Den Hurk; Bixing Huang; Carmel Taylor; David Warrilow; Doris Genge; Elisabeth Gamez; Glen Hewitson; Ian Maxwell Mackay; Inga Sultana; Jamie McMahon; Jean Barcelon; Judy Northill; Mitchell Finger; Natalie Simpson; Neelima Nair; Peter Burtonclay; Peter Moore; Sarah Wheatley; Sean Moody; Sonja Hall-Mendelin; Timothy Gardam; and Frederick Moore
EPI_ISL_508415 to 508422, EPI_ISL_653833 to 653858	Maulana Azad Medical College	National Institute of Biomedical Genomics	Arindam Maitra; Oves Siddiqui; Saumitra Das; Sonal Saxena; Vikas Manchanda
EPI_ISL_420898 to 420903, EPI_ISL_420905 to 420907, EPI_ISL_420912	Max von Pettenkofer Institute, Virology, National Reference Center for Retroviruses, LMU Munich	Laboratory for Functional Genome Analysis, Dept. Genomics, Gene Center of the LMU Munich	Alexander Graf; Ashok Varadharajan; Helmut Blum; Max Muenchhoff; Oliver Keppler; Stefan Krebs
EPI_ISL_437204 to 437225, EPI_ISL_437227 to 437239, EPI_ISL_437241 to 437292, EPI_ISL_437294 to 437297, EPI_ISL_437359, EPI_ISL_451937, EPI_ISL_451940 to 451941, EPI_ISL_451944 to 451945, EPI_ISL_452103 to 452104, EPI_ISL_466875, EPI_ISL_466877 to 466885, EPI_ISL_466888 to 466889, EPI_ISL_466891 to 466893, EPI_ISL_466895 to 466897, EPI_ISL_466899 to 466907, EPI_ISL_466909 to 466911, EPI_ISL_466913, EPI_ISL_466916 to 466925			
see above	Max von Pettenkofer Institute, Virology, National Reference Center for Retroviruses, LMU München	Laboratory for Functional Genome Analysis, Dept. Genomics, Gene Center of the LMU Munich	Alexander Graf; Helmut Blum; Max Muenchhoff; Oliver Keppler; Stefan Krebs
EPI_ISL_547870 to 547876	Maximum Containment Laboratory, National Institute of Virology	Maximum Containment Laboratory, National Institute of Virology	Abraham, P.; D.A.; Nyayanit; P.D.; Shete-aich, A.; Yadav
EPI_ISL_477272 to 477276, EPI_ISL_477291 to 477312, EPI_ISL_481242, EPI_ISL_482967 to 482987, EPI_ISL_495597 to 495609, EPI_ISL_496918, EPI_ISL_507957 to 507961, EPI_ISL_514619 to 514628, EPI_ISL_514636, EPI_ISL_514638 to 514640, EPI_ISL_514644, EPI_ISL_514653 to 514656, EPI_ISL_515263 to 515266, EPI_ISL_518856 to 518900, EPI_ISL_521153, EPI_ISL_527630 to 527631, EPI_ISL_539842 to 539849, EPI_ISL_560792 to 560797, EPI_ISL_603010 to 603011, EPI_ISL_644936 to 644940, EPI_ISL_654698, EPI_ISL_683695, EPI_ISL_683714 to 683728, EPI_ISL_708483, EPI_ISL_723141 to 723145			
see above	Mayo Clinic & Mayo Clinic Laboratories	Minnesota Department of Health, Public Health Laboratory	Alexandra Lorentz; Jacob Garfin; Kelly Pung; Matt Plumb; and Xiong Wang
EPI_ISL_490978, EPI_ISL_490988, EPI_ISL_490997 to 491000, EPI_ISL_491004	Mayo Clinic Laboratories	UW Virology Lab	Alexander Greninger; Amin Addetia; Hong Xie; Keith R Jerome; Lasata Shrestha; Meei-Li Huang; Pavitra Roychoudhury; Truong Nguyen; Victoria M Rachleff
EPI_ISL_500503 to 500531, EPI_ISL_500533 to 500538, EPI_ISL_501072 to 501082, EPI_ISL_501165	Mayo Clinic Laboratories	University of Washington Virology Lab	Alexander Greninger; Amin Addetia; Hong Xie; Keith R Jerome; Lasata Shrestha; Meei-Li Huang; Pavitra Roychoudhury; Truong Nguyen; Victoria M Rachleff
EPI_ISL_569620	McCrossan Boys Ranch	South Dakota Public Health Laboratory	Jacob Garfin; Matt Plumb; Xiong Wang; and Chris Carlson
EPI_ISL_456377 to 456378	MedLab Central Ltd	Institute of Environmental Science and Research (ESR)	Anja Werno; Antje van der Linden; Arlo Upton; Chris Mansell; David Hammer; Dragana Drinkovic; Erasmus Smit; Gary McAuliffe; Hana Sofia Andersson; James Ussher; Jill Sherwood; Joep de Ligt; Josh Freeman; Julia Howard; Juliet Elvy; Lauren Jelly; Mary DeAlmeida; Matt Blakiston; Matt Storey; Matthew Rogers; Max Bloomfield; Michael Addidle; Michelle Balm; Sally Roberts; Sarah Jefferies; Sharmini Muttaiyah; Susan Morpeth; Susan Taylor; Timothy

			Blackmore; Vani Sathyendran; Veronica Playle; Virginia Hope; Xiaoyun Ren
EPI_ISL_475745 to 475749, EPI_ISL_475751 to 475753, EPI_ISL_482759 to 482774, EPI_ISL_483035 to 483036, EPI_ISL_483038	Medical Ain Shams Research Institute (MASRI), Ain Shams University	Medical Ain Shams Research Institute (MASRI), Ain Shams University	Ahmad Moustafa; Ashraf Omar; Aya Mohamed; Fatma Ebied; Ghada Ismael; Hala Hafez; Hesham Elghazaly; Hoda Abd Elsatar; Mahmoud Elmeitini; Mahmoud Elmeteini; Manal Hamdy Elsaid; Osama Mansour; Reham Mamdouh; Samia Abdo; Sara Elnakeep; Sara Hassan Agwa; Shaimaa Moustafa
EPI_ISL_476832	Medical Biology Department, Kocaeli University	Medical Genetics Department, Kocaeli University	Akhan S; Akpinar G; Canturk NZ.; Cine N; Eren-Keskin S; Ilgazli A; Karadenizli A; Kasap M; Savli H; Sayan M; Sunnetci-Akkoyunlu D
EPI_ISL_495535 to 495542, EPI_ISL_515801 to 515833, EPI_ISL_515835 to 515878, EPI_ISL_515880 to 515893	Medical Disagnostics Services (MDS)	KRISP, KZN Research Innovation and Sequencing Platform	Chimukangara B; ChimukangaraB; Giandhari J; Khan S; Lessells R; Mdlalose K; Pillay S; Tegally H; Wilkinson E; York D; de Oliveira T
EPI_ISL_1040930, EPI_ISL_1040936, EPI_ISL_1040987, EPI_ISL_1040990, EPI_ISL_1041003, EPI_ISL_1041009	Medical Laboratory Bruss	Laboratory of Recombinant Vaccines	Boguslaw Szewczyk; Krystyna Bienkowska-Szewczyk; Lukasz Rabalski; Maciej Grzybek; Maciej Kosinski; Pawel Pisarek
EPI_ISL_493381 to 493382, EPI_ISL_549039 to 549041, EPI_ISL_549059, EPI_ISL_549070, EPI_ISL_549081, EPI_ISL_549085 to 549086, EPI_ISL_549164, EPI_ISL_549166, EPI_ISL_590879 to 590880, EPI_ISL_590893, EPI_ISL_590929, EPI_ISL_590932 to 590933, EPI_ISL_590945, EPI_ISL_635099, EPI_ISL_635101, EPI_ISL_635134, EPI_ISL_635159, EPI_ISL_635169, EPI_ISL_635192 to 635193, EPI_ISL_668400, EPI_ISL_708041, EPI_ISL_708115 to 708117, EPI_ISL_708136 to 708139, EPI_ISL_708196, EPI_ISL_708199, EPI_ISL_759977 to 759980, EPI_ISL_759983, EPI_ISL_1013494, EPI_ISL_1040144			
see above	Medical Microbiology Unit, Department for Laboratory Medicine, Drammen Hospital, Vestre Viken Health Trust,	Norwegian Institute of Public Health, Department of Virology	Engebretsen Serina Beate Atiya R Ali; Garcia Llorente Ignacio; Hilde Elshaug; Hilde Synnøve Vollan; Hilde Vollan; Kamilla Heddeland Instefjord; Karoline Bragstad; Kathrine Stene-Johansen; Marie Paulsen Madsen; Olav Hungnes; Rasmus Riis Kopperud
EPI_ISL_482745 to 482746	Medical Microbiology, Leiden University Medical Center	Medical Microbiology, Leiden University Medical Center	Dalebout; E.J.; J.C.; J.C. and Sidorov, I.; J.J. and Sidorov, I.; N.S.; Ogando; Snijder; T.J.; Zevenhoven; de Vries
EPI_ISL_707692, EPI_ISL_707696, EPI_ISL_708652	Medical Research Center, Faculty of Medicine, Syarif Hidayatullah State Islamic University Jakarta	Medical Research Center, Faculty of Medicine, Syarif Hidayatullah State Islamic University Jakarta	Chris Adhiyanto; Dennis Nurjadi; Endah Wulandari; Erike A Suwarsono; Erike Suwarsono; Ferania Mela; Flori R Sari; Hari Hendarto; Laifa Hendarmin; Rini Puspitaningrum; Zeti Harriyati
EPI_ISL_591522 to 591523, EPI_ISL_591526 to 591530	Medicina Norte U Chile - Servicio Medico Legal	Center for Mathematical Modeling and Center for Genome Regulation. Santiago, Chile	Allende ML; Ferres M.; Gaete A; Gaggero A; González M; Maass A; Palma R; Travisany D; Urra C; Valiente F; Varas M
EPI_ISL_435281	Medistra Hospital Jakarta	Eijkman Institute for Molecular Biology, Ministry of Research and Technology/National Agency for Research and Innovation	Amin Soebandrio; David H Muljono; Edison Johar; Frilasita A Yudhaputri; Hidayat Trimarsanto; Khin Saw Myint; Safarina G Malik
EPI_ISL_581478 to 581485	Medizinische Klinik Innere Medizin I, Universitätsklinikum Tübingen	NGS Competence Center Tübingen, Institut für Medizinische Mikrobiologie und Hygiene, Universitätsklinikum Tübingen	Angel Angelov
EPI_ISL_451534, EPI_ISL_451553 to 451555, EPI_ISL_451558, EPI_ISL_451567, EPI_ISL_451583, EPI_ISL_451597, EPI_ISL_451610, EPI_ISL_513341, EPI_ISL_526192, EPI_ISL_591492, EPI_ISL_593654, EPI_ISL_767882 to 767883			
see above	Medlab Pathology	NSW Health Pathology - Institute of Clinical Pathology and Medical Research; Westmead Hospital; University of Sydney	CIDM-PH et al.
EPI_ISL_536411, EPI_ISL_536793	Medtimes Molecular Laboratory	Medtimes Molecular Laboratory	Eric Chan; Isaac Chow; Jacqueline Tam; Winsome Wong
EPI_ISL_583427 to 583428, EPI_ISL_583431 to 583433, EPI_ISL_583437, EPI_ISL_583439 to 583440, EPI_ISL_583443, EPI_ISL_583445 to 583446, EPI_ISL_583448 to 583449, EPI_ISL_583456 to 583457, EPI_ISL_583460 to 583462, EPI_ISL_583465 to 583466, EPI_ISL_583468 to 583470, EPI_ISL_583472, EPI_ISL_583475 to 583476, EPI_ISL_583478 to 583480			
see above	Memorial Sloan Kettering Cancer Center	van Bakel Laboratory, Genetics and Genomics Sciences, Icahn School of Medicine at Mount Sinai	Adolfo García-Sastre; Adriana van de Guchte; Ajay Obla; Ana S. Gonzalez-Reiche; Genovefa Papanicolaou; Gunjan Shah; Harm van Bakel; Jayeeta Dutta; Judith Aberg; Kent Sepkowitz; Miguel-Angel Perales; Ngolela Esther Babady; Sadaf Aslam; Teresa Aydillo; Tobias Hohl; Zenab Khan; and Mini Kamboj
EPI_ISL_631832	Metro Urgicare	New York City Public Health Laboratory	Jade Wang; et al.
EPI_ISL_436800 to 436804, EPI_ISL_436807 to 436808, EPI_ISL_436812, EPI_ISL_436814 to 436815, EPI_ISL_436818 to 436825, EPI_ISL_436827, EPI_ISL_436829 to 436831, EPI_ISL_436833 to 436834, EPI_ISL_436836, EPI_ISL_436838, EPI_ISL_436841 to 436843, EPI_ISL_436846 to 436848, EPI_ISL_436850, EPI_ISL_436852 to 436857, EPI_ISL_436859, EPI_ISL_436861, EPI_ISL_436863 to 436865, EPI_ISL_436867 to 436870, EPI_ISL_436874 to 436880, EPI_ISL_436884 to 436887, EPI_ISL_436889, EPI_ISL_437099 to 437101, EPI_ISL_437107, EPI_ISL_437109, EPI_ISL_437111, EPI_ISL_437113, EPI_ISL_437115, EPI_ISL_437117, EPI_ISL_437120, EPI_ISL_437123, EPI_ISL_437128, EPI_ISL_437131 to 437132, EPI_ISL_437135, EPI_ISL_437137 to 437140, EPI_ISL_437142, EPI_ISL_437145 to 437147, EPI_ISL_437150, EPI_ISL_437152 to 437155, EPI_ISL_437157, EPI_ISL_437162 to 437164, EPI_ISL_437166, EPI_ISL_437170, EPI_ISL_437174, EPI_ISL_437176, EPI_ISL_437180, EPI_ISL_447057, EPI_ISL_447059, EPI_ISL_447063 to 447064, EPI_ISL_447068 to 447069, EPI_ISL_447071 to 447072, EPI_ISL_447077, EPI_ISL_447081 to 447083, EPI_ISL_447088 to 447096, EPI_ISL_447098 to 447099, EPI_ISL_447101 to 447102, EPI_ISL_447104 to 447107, EPI_ISL_447111, EPI_ISL_447113, EPI_ISL_447115 to 447118, EPI_ISL_447163, EPI_ISL_447165, EPI_ISL_447167 to 447168, EPI_ISL_447172 to 447175, EPI_ISL_447177 to 447179, EPI_ISL_447183 to 447184, EPI_ISL_447189 to 447191, EPI_ISL_447193 to 447194, EPI_ISL_447196 to 447197, EPI_ISL_447200 to 447201, EPI_ISL_447204 to 447207, EPI_ISL_447209 to 447212, EPI_ISL_447215 to 447216, EPI_ISL_447218 to 447221, EPI_ISL_447224, EPI_ISL_447228 to 447229, EPI_ISL_450572, EPI_ISL_450574, EPI_ISL_450576 to 450577, EPI_ISL_450580 to 450581, EPI_ISL_450585, EPI_ISL_450588, EPI_ISL_450592, EPI_ISL_450601, EPI_ISL_450604, EPI_ISL_450606 to 450607, EPI_ISL_450610, EPI_ISL_450612, EPI_ISL_450614, EPI_ISL_450616, EPI_ISL_450618, EPI_ISL_450620, EPI_ISL_450623, EPI_ISL_450631, EPI_ISL_450635 to 450636, EPI_ISL_452262, EPI_ISL_452264, EPI_ISL_452266, EPI_ISL_452270, EPI_ISL_452272, EPI_ISL_452274, EPI_ISL_452278 to 452285, EPI_ISL_452287 to 452292, EPI_ISL_452294 to 452295, EPI_ISL_452297 to 452298, EPI_ISL_452300 to 452303, EPI_ISL_452305 to 452306, EPI_ISL_452309 to 452311, EPI_ISL_452316 to 452326, EPI_ISL_459994 to 459999, EPI_ISL_460003 to 460004, EPI_ISL_460008 to 460009, EPI_ISL_460012, EPI_ISL_460015 to 460018, EPI_ISL_460020 to 460021, EPI_ISL_460023, EPI_ISL_460025 to 460026, EPI_ISL_460028, EPI_ISL_460031, EPI_ISL_460033 to 460034, EPI_ISL_460040, EPI_ISL_460042, EPI_ISL_460556 to 460560, EPI_ISL_460569, EPI_ISL_460571 to 460574, EPI_ISL_460581 to 460588, EPI_ISL_460599 to 460602, EPI_ISL_462673 to 462675, EPI_ISL_462678 to 462681, EPI_ISL_462683 to 462701, EPI_ISL_462706 to 462708, EPI_ISL_462719 to 462720, EPI_ISL_462725, EPI_ISL_462731 to 462734, EPI_ISL_462736, EPI_ISL_471679, EPI_ISL_471685 to 471687, EPI_ISL_471689, EPI_ISL_471691 to 471693, EPI_ISL_471695 to 471696, EPI_ISL_471701 to 471702, EPI_ISL_471704, EPI_ISL_471707 to 471709, EPI_ISL_471712 to 471717, EPI_ISL_471720 to 471722, EPI_ISL_471724 to 471725, EPI_ISL_471728 to 471729, EPI_ISL_471731 to 471732, EPI_ISL_471739 to 471740, EPI_ISL_471742 to 471743, EPI_ISL_471745, EPI_ISL_471749 to 471750, EPI_ISL_471754, EPI_ISL_471767 to 471772, EPI_ISL_471787 to 471797, EPI_ISL_471807 to 471810, EPI_ISL_471812 to 471815, EPI_ISL_471817 to 471823, EPI_ISL_471825 to 471826, EPI_ISL_471830 to 471835, EPI_ISL_471837 to 471840, EPI_ISL_471842 to 471844, EPI_ISL_471847 to 471848, EPI_ISL_471850 to 471859, EPI_ISL_471871 to 471891, EPI_ISL_471896 to 471910, EPI_ISL_476027 to 476030, EPI_ISL_476032 to 476034, EPI_ISL_476038 to 476046, EPI_ISL_476048 to 476051, EPI_ISL_476053 to 476056, EPI_ISL_476058 to 476060, EPI_ISL_476062 to 476064, EPI_ISL_507431, EPI_ISL_507434 to 507435, EPI_ISL_507437 to 507438, EPI_ISL_507441 to 507442, EPI_ISL_507444 to 507446, EPI_ISL_507459 to 507460, EPI_ISL_507462, EPI_ISL_507464 to 507471, EPI_ISL_507528, EPI_ISL_507602 to 507623, EPI_ISL_507682, EPI_ISL_507684 to 507686, EPI_ISL_507688 to 507720, EPI_ISL_507739 to 507740, EPI_ISL_507743 to 507748, EPI_ISL_507760 to 507761, EPI_ISL_507763 to 507764, EPI_ISL_507777 to 507789, EPI_ISL_507870 to 507871, EPI_ISL_507874, EPI_ISL_507879, EPI_ISL_507881 to 507917, EPI_ISL_507919 to 507928, EPI_ISL_507930 to 507933, EPI_ISL_516267 to 516270, EPI_ISL_516272 to 516275, EPI_ISL_516277 to 516278, EPI_ISL_516281 to 516283, EPI_ISL_516285, EPI_ISL_516287, EPI_ISL_516294 to 516297, EPI_ISL_516302, EPI_ISL_516306, EPI_ISL_516308, EPI_ISL_516311, EPI_ISL_516314, EPI_ISL_516316 to 516317, EPI_ISL_516321 to 516322, EPI_ISL_516324, EPI_ISL_516329 to 516332, EPI_ISL_516334 to 516335, EPI_ISL_516348, EPI_ISL_516351, EPI_ISL_516359 to 516363, EPI_ISL_516365, EPI_ISL_516367, EPI_ISL_516371 to 516373, EPI_ISL_516376, EPI_ISL_516380, EPI_ISL_516412, EPI_ISL_529818, EPI_ISL_529830, EPI_ISL_529832, EPI_ISL_529850, EPI_ISL_529874, EPI_ISL_529888, EPI_ISL_565837 to 565838, EPI_ISL_565843, EPI_ISL_565846, EPI_ISL_565849, EPI_ISL_565853, EPI_ISL_565855, EPI_ISL_565873, EPI_ISL_565881, EPI_ISL_565884, EPI_ISL_565949, EPI_ISL_565951, EPI_ISL_565953 to 565989, EPI_ISL_566019 to 566035, EPI_ISL_576208 to 576221, EPI_ISL_577565 to 577596, EPI_ISL_583506 to 583508, EPI_ISL_583511, EPI_ISL_583515, EPI_ISL_583517 to 583519, EPI_ISL_583521 to 583523, EPI_ISL_583525, EPI_ISL_583530 to 583532, EPI_ISL_614166, EPI_ISL_614168 to 614180, EPI_ISL_614199, EPI_ISL_614201, EPI_ISL_614203 to 614246, EPI_ISL_644364 to 644374, EPI_ISL_666930 to 666963, EPI_ISL_708204 to 708205, EPI_ISL_708207 to 708231, EPI_ISL_708241 to 708273, EPI_ISL_708283 to 708305, EPI_ISL_708354 to 708358, EPI_ISL_708360 to 708378, EPI_ISL_708563, EPI_ISL_708568 to 708606, EPI_ISL_737153 to 737198, EPI_ISL_737301 to 737305, EPI_ISL_888851, EPI_ISL_1008825, EPI_ISL_1008863			
see above	Michigan Department of Health and Human Services, Bureau of Laboratories	Michigan Department of Health and Human Services, Bureau of Laboratories	Blankenship HM; Riner D; Soehnlen MK
EPI_ISL_454353	Microbial Genome Sequencing Center; Microbial Genomic Epidemiology Laboratory	Microbial Genomic Epidemiology Laboratory, University of Pittsburgh	Chinelo Ezeonwuku; Dan Snyder; Jane W. Marsh; Kady D. Waggle; Lee H. Harrison; Marissa P. Griffith; Mustapha M. Mustapha; Stephanie L. Mitchell; Vatsala R. Srinivasa; Vaughn S. Cooper
EPI_ISL_480333	Microbial Genomics Laboratory, Institut Pasteur de Montevideo	Microbial Genomics Laboratory, Institut Pasteur de Montevideo	Cecilia Salazar; Gonzalo Moratorio; Gregorio Iraola; Ignacio Ferrés; Marianoel Pereira; Pilar Moreno
EPI_ISL_875558, EPI_ISL_977160	Microbiologia e Virologia	Istituto Zooprofilattico Sperimentale delle Venezie	Adelaide Milani; Alessia Schivo; Alice Fusaro; Ambra Pastori; Annalisa Salviato; Antonia Ricci; Bianca Zecchin; Calogero Terregino; Erika Giorgia Quaranta; Isabella Monne
EPI_ISL_480560 to 480561, EPI_ISL_480616, EPI_ISL_480687, EPI_ISL_480746, EPI_ISL_480748 to 480750, EPI_ISL_480752 to 480763, EPI_ISL_480767 to 480777, EPI_ISL_518063 to 518080, EPI_ISL_518093 to 518094, EPI_ISL_518096 to 518097, EPI_ISL_518231 to 518232, EPI_ISL_518234 to 518236, EPI_ISL_518238, EPI_ISL_518241 to 518242, EPI_ISL_518250 to 518278, EPI_ISL_518282 to 518285, EPI_ISL_518287 to 518290, EPI_ISL_518292 to 518302, EPI_ISL_518304 to 518309, EPI_ISL_518311 to 518314, EPI_ISL_518316, EPI_ISL_518318 to 518319, EPI_ISL_518321 to 518326, EPI_ISL_518328 to 518337, EPI_ISL_518339 to 518347, EPI_ISL_518349 to 518365, EPI_ISL_518367 to 518371, EPI_ISL_518374 to 518376, EPI_ISL_518378, EPI_ISL_518380 to 518384, EPI_ISL_518386 to 518387, EPI_ISL_518402 to 518407, EPI_ISL_518410 to 518412, EPI_ISL_518415 to 518416, EPI_ISL_518422, EPI_ISL_518472 to 518477, EPI_ISL_518479 to 518480, EPI_ISL_518482 to 518488, EPI_ISL_518490 to 518511, EPI_ISL_518514 to 518520, EPI_ISL_518522 to 518531, EPI_ISL_518533 to 518542, EPI_ISL_518544 to 518565, EPI_ISL_518567 to 518574, EPI_ISL_518576 to 518582, EPI_ISL_518584, EPI_ISL_518586 to 518592, EPI_ISL_518594 to 518597, EPI_ISL_518599 to 518647, EPI_ISL_518649, EPI_ISL_518652 to 518660, EPI_ISL_518662, EPI_ISL_518664 to 518667, EPI_ISL_518669 to 518709, EPI_ISL_518711 to 518722, EPI_ISL_518724 to 518763, EPI_ISL_518765 to 518771, EPI_ISL_518773 to 518792, EPI_ISL_518795 to 518796, EPI_ISL_518901 to 518915, EPI_ISL_518917, EPI_ISL_518919 to 518934, EPI_ISL_518937 to 518941, EPI_ISL_518943 to 518952, EPI_ISL_518954 to 518969, EPI_ISL_518971 to 518973, EPI_ISL_518975, EPI_ISL_518977 to 518980, EPI_ISL_519052 to 519111, EPI_ISL_519113 to 519128, EPI_ISL_519130 to 519161, EPI_ISL_519163 to 519164, EPI_ISL_519166 to 519174, EPI_ISL_519176 to 519185, EPI_ISL_519187, EPI_ISL_519189 to 519191, EPI_ISL_519193 to 519196, EPI_ISL_519198 to 519201, EPI_ISL_519203 to 519217, EPI_ISL_519219 to 519226, EPI_ISL_519228 to 519229, EPI_ISL_519231 to 519255, EPI_ISL_519329 to 519330, EPI_ISL_519333 to 519334, EPI_ISL_519336 to 519350, EPI_ISL_519352 to 519355, EPI_ISL_519357 to 519358, EPI_ISL_519360 to 519369, EPI_ISL_519371 to 519384, EPI_ISL_519386 to 519387, EPI_ISL_519389 to 519390, EPI_ISL_519392 to 519403, EPI_ISL_519405 to 519406, EPI_ISL_519408 to 519410, EPI_ISL_519416, EPI_ISL_519418, EPI_ISL_519422 to 519424, EPI_ISL_519426, EPI_ISL_519428, EPI_ISL_519433, EPI_ISL_519436, EPI_ISL_519438 to 519440, EPI_ISL_519442 to 519443, EPI_ISL_519446 to 519447, EPI_ISL_519449, EPI_ISL_519452 to 519454, EPI_ISL_519458 to 519459, EPI_ISL_519465, EPI_ISL_519470 to 519474, EPI_ISL_519476, EPI_ISL_519478, EPI_ISL_519481 to 519482, EPI_ISL_519484 to 519485, EPI_ISL_519503 to 519508, EPI_ISL_519518, EPI_ISL_519522 to 519549, EPI_ISL_519551 to 519575, EPI_ISL_519577 to 519623, EPI_ISL_519625, EPI_ISL_519627 to 519629, EPI_ISL_519631 to 519643, EPI_ISL_519645 to 519672, EPI_ISL_519674 to 519689, EPI_ISL_519691 to 519755, EPI_ISL_519757 to 519781, EPI_ISL_519783 to 519784, EPI_ISL_519786 to 519816, EPI_ISL_519818 to 519878, EPI_ISL_519880 to 519894, EPI_ISL_519896 to 519924, EPI_ISL_519926 to 519947, EPI_ISL_519951 to 519969, EPI_ISL_519971 to 520050, EPI_ISL_520052 to 520063, EPI_ISL_520065 to 520074, EPI_ISL_520076 to 520078, EPI_ISL_520080 to 520119, EPI_ISL_520121 to 520180, EPI_ISL_520215 to 520230, EPI_ISL_520233 to 520235, EPI_ISL_520237 to 520238, EPI_ISL_520240 to 520241, EPI_ISL_520243 to 520248, EPI_ISL_520421, EPI_ISL_520449, EPI_ISL_520507 to 520513, EPI_ISL_520516 to 520526, EPI_ISL_520528 to 520552, EPI_ISL_520554 to 520594, EPI_ISL_520596 to 520603, EPI_ISL_520606 to 520643, EPI_ISL_520645 to 520654, EPI_ISL_520656 to 520662, EPI_ISL_520765 to 520774, EPI_ISL_520776 to 520790, EPI_ISL_520844 to 520863, EPI_ISL_520929 to 520947,			

EPI_ISL_520949 to 520952, EPI_ISL_520954 to 520962, EPI_ISL_520964 to 521074, EPI_ISL_521076 to 521089, EPI_ISL_521091 to 521101, EPI_ISL_521154, EPI_ISL_521156 to 521163, EPI_ISL_521165 to 521168, EPI_ISL_521170 to 521204, EPI_ISL_521206 to 521208, EPI_ISL_521210 to 521215, EPI_ISL_521217 to 521222, EPI_ISL_521227 to 521231, EPI_ISL_521234 to 521269, EPI_ISL_521278 to 521285, EPI_ISL_521287 to 521314, EPI_ISL_521450, EPI_ISL_521452 to 521461, EPI_ISL_521562 to 521571, EPI_ISL_521573 to 521594, EPI_ISL_521596 to 521597, EPI_ISL_521599 to 521604, EPI_ISL_521606 to 521619, EPI_ISL_521622, EPI_ISL_521624 to 521681, EPI_ISL_521683 to 521727, EPI_ISL_521729 to 521734, EPI_ISL_521736 to 521758, EPI_ISL_521760 to 521774, EPI_ISL_521776 to 521793, EPI_ISL_521795 to 521824, EPI_ISL_521826 to 521839, EPI_ISL_521887, EPI_ISL_521906 to 521907, EPI_ISL_521909 to 521910, EPI_ISL_521927 to 521944, EPI_ISL_521972 to 521974, EPI_ISL_521989, EPI_ISL_521991 to 522054, EPI_ISL_522111 to 522118, EPI_ISL_522188 to 522189, EPI_ISL_522192 to 522231, EPI_ISL_522243 to 522245, EPI_ISL_522247, EPI_ISL_522249 to 522253, EPI_ISL_562168 to 562174, EPI_ISL_562591 to 562592, EPI_ISL_562594, EPI_ISL_562596 to 562627, EPI_ISL_562629 to 562639, EPI_ISL_562641 to 562644, EPI_ISL_562647 to 562657, EPI_ISL_562660 to 562668, EPI_ISL_562670, EPI_ISL_562672 to 562688, EPI_ISL_562691 to 562695, EPI_ISL_562698, EPI_ISL_562701 to 562704, EPI_ISL_562706 to 562708, EPI_ISL_562728 to 562753, EPI_ISL_562761 to 562790, EPI_ISL_562793 to 562802, EPI_ISL_562810 to 562837, EPI_ISL_562846 to 562909, EPI_ISL_562914 to 562923, EPI_ISL_562925 to 562928, EPI_ISL_562930 to 562935, EPI_ISL_562937 to 562945, EPI_ISL_562947 to 562950, EPI_ISL_562952 to 562960, EPI_ISL_562962 to 562999, EPI_ISL_563001 to 563130, EPI_ISL_563134 to 563135, EPI_ISL_563145 to 563406, EPI_ISL_563424 to 563496, EPI_ISL_563500 to 563550, EPI_ISL_563552 to 563608, EPI_ISL_563610 to 563720, EPI_ISL_563724 to 563789, EPI_ISL_563791, EPI_ISL_563793 to 563807, EPI_ISL_563809 to 563810, EPI_ISL_563812 to 563816, EPI_ISL_563818 to 563819, EPI_ISL_563821 to 563831, EPI_ISL_563833 to 563839, EPI_ISL_563841 to 563865, EPI_ISL_563867 to 563879, EPI_ISL_563881 to 563924, EPI_ISL_563926 to 563937, EPI_ISL_563939 to 563972, EPI_ISL_563976, EPI_ISL_563979 to 563986, EPI_ISL_563988 to 563996, EPI_ISL_564050 to 564054, EPI_ISL_564111 to 564179, EPI_ISL_564255 to 564269, EPI_ISL_564271 to 564317, EPI_ISL_564351, EPI_ISL_564398 to 564403, EPI_ISL_564405 to 564543, EPI_ISL_564545, EPI_ISL_564565 to 565135, EPI_ISL_565223 to 565381, EPI_ISL_565383 to 565384, EPI_ISL_565386 to 565393, EPI_ISL_565397 to 565398, EPI_ISL_565400, EPI_ISL_565405 to 565408, EPI_ISL_565410 to 565414, EPI_ISL_565416 to 565458, EPI_ISL_565460, EPI_ISL_565462, EPI_ISL_565468, EPI_ISL_565470 to 565508, EPI_ISL_565510 to 565729, EPI_ISL_565743 to 565829, EPI_ISL_591718, EPI_ISL_591793 to 591798, EPI_ISL_591801 to 591806, EPI_ISL_591811, EPI_ISL_591819 to 591821, EPI_ISL_591826 to 591848, EPI_ISL_591853, EPI_ISL_591905 to 591906, EPI_ISL_591910 to 591911, EPI_ISL_591913 to 591916, EPI_ISL_591918, EPI_ISL_591920 to 591924, EPI_ISL_591926 to 591927, EPI_ISL_591929 to 591931, EPI_ISL_591933 to 591939, EPI_ISL_591961 to 591967, EPI_ISL_591969 to 592135, EPI_ISL_592196 to 592484, EPI_ISL_592487, EPI_ISL_592510, EPI_ISL_592517 to 592530, EPI_ISL_592704, EPI_ISL_592819, EPI_ISL_592822 to 592823, EPI_ISL_592825 to 592827, EPI_ISL_592829 to 592835, EPI_ISL_592838 to 592856, EPI_ISL_592866 to 592877, EPI_ISL_592882 to 592957, EPI_ISL_592959 to 592968, EPI_ISL_592986 to 593002, EPI_ISL_593008 to 593032, EPI_ISL_593034, EPI_ISL_593036 to 593047, EPI_ISL_593049 to 593124, EPI_ISL_593129 to 593214, EPI_ISL_593217 to 593255, EPI_ISL_593257 to 593410, EPI_ISL_593413 to 593475, EPI_ISL_640303 to 640305, EPI_ISL_640308, EPI_ISL_640310, EPI_ISL_640312 to 640318, EPI_ISL_640320 to 640322, EPI_ISL_640324 to 640325, EPI_ISL_640327 to 640329, EPI_ISL_640331, EPI_ISL_640333 to 640336, EPI_ISL_640338 to 640341, EPI_ISL_640344 to 640345, EPI_ISL_640347 to 640348, EPI_ISL_640351 to 640355, EPI_ISL_640358 to 640364, EPI_ISL_640367 to 640369, EPI_ISL_640371 to 640373, EPI_ISL_640376 to 640380, EPI_ISL_640382 to 640385, EPI_ISL_640388, EPI_ISL_640390, EPI_ISL_640392, EPI_ISL_640394, EPI_ISL_640396 to 640398, EPI_ISL_640400 to 640408, EPI_ISL_640410 to 640413, EPI_ISL_640415 to 640417, EPI_ISL_640419 to 640425, EPI_ISL_640427, EPI_ISL_640432 to 640437, EPI_ISL_640439 to 640442, EPI_ISL_640444 to 640446, EPI_ISL_640448 to 640452, EPI_ISL_640454 to 640457, EPI_ISL_640459 to 640470, EPI_ISL_640472 to 640473, EPI_ISL_640475, EPI_ISL_640478, EPI_ISL_640480, EPI_ISL_640482, EPI_ISL_640485 to 640487, EPI_ISL_640490, EPI_ISL_640492, EPI_ISL_640494 to 640508, EPI_ISL_640510 to 640511, EPI_ISL_640513 to 640514, EPI_ISL_640516 to 640518, EPI_ISL_640520 to 640521, EPI_ISL_640523 to 640524, EPI_ISL_640528 to 640531, EPI_ISL_640533, EPI_ISL_640535 to 640536, EPI_ISL_640541, EPI_ISL_640544 to 640550, EPI_ISL_640552 to 640555, EPI_ISL_640557 to 640561, EPI_ISL_640565 to 640567, EPI_ISL_640569 to 640582, EPI_ISL_640584, EPI_ISL_640586 to 640587, EPI_ISL_640589, EPI_ISL_640591 to 640598, EPI_ISL_640600, EPI_ISL_640603 to 640604, EPI_ISL_640610 to 640612, EPI_ISL_640614 to 640624, EPI_ISL_640628 to 640629, EPI_ISL_640631 to 640633, EPI_ISL_640635 to 640638, EPI_ISL_640642 to 640643, EPI_ISL_640646 to 640649, EPI_ISL_640651 to 640653, EPI_ISL_640655 to 640657, EPI_ISL_640659 to 640663, EPI_ISL_640665 to 640668, EPI_ISL_640670 to 640673, EPI_ISL_640675, EPI_ISL_640677 to 640679, EPI_ISL_640684 to 640695, EPI_ISL_640697 to 640699, EPI_ISL_640703 to 640704, EPI_ISL_640706 to 640711, EPI_ISL_640713 to 640715, EPI_ISL_640717 to 640719, EPI_ISL_640721, EPI_ISL_640723 to 640731, EPI_ISL_640733 to 640737, EPI_ISL_640743, EPI_ISL_640745 to 640747, EPI_ISL_640749 to 640750, EPI_ISL_640753 to 640754, EPI_ISL_640756 to 640762, EPI_ISL_640765 to 640767, EPI_ISL_640770, EPI_ISL_640773, EPI_ISL_640776 to 640778, EPI_ISL_640780 to 640782, EPI_ISL_640785 to 640786, EPI_ISL_640788 to 640790, EPI_ISL_640792 to 640794, EPI_ISL_640796, EPI_ISL_640798 to 640802, EPI_ISL_640805 to 640813, EPI_ISL_640816 to 640817, EPI_ISL_640819 to 640826, EPI_ISL_640829 to 640835, EPI_ISL_640837 to 640847, EPI_ISL_640849 to 640854, EPI_ISL_640856, EPI_ISL_640859, EPI_ISL_640862, EPI_ISL_640864, EPI_ISL_640866 to 640869, EPI_ISL_640872, EPI_ISL_640874 to 640897, EPI_ISL_640899, EPI_ISL_640901, EPI_ISL_640903 to 640909, EPI_ISL_640911, EPI_ISL_640913 to 640919, EPI_ISL_640921 to 640926, EPI_ISL_640928, EPI_ISL_640931 to 640932, EPI_ISL_640934 to 640936, EPI_ISL_640938, EPI_ISL_640940 to 640941, EPI_ISL_640943 to 640945, EPI_ISL_640947 to 640948, EPI_ISL_640950 to 640957, EPI_ISL_640959 to 640964, EPI_ISL_640966, EPI_ISL_640968 to 640973, EPI_ISL_640975 to 640977, EPI_ISL_640979 to 640980, EPI_ISL_640983 to 640984, EPI_ISL_640987 to 640989, EPI_ISL_640991 to 640997, EPI_ISL_640999 to 641001, EPI_ISL_641003 to 641005, EPI_ISL_641008 to 641011, EPI_ISL_641013 to 641014, EPI_ISL_641018, EPI_ISL_641020 to 641022, EPI_ISL_641024 to 641030, EPI_ISL_641032, EPI_ISL_641034 to 641036, EPI_ISL_641038, EPI_ISL_641040 to 641043, EPI_ISL_641045 to 641046, EPI_ISL_641048, EPI_ISL_641050 to 641051, EPI_ISL_641053 to 641054, EPI_ISL_641056, EPI_ISL_641058, EPI_ISL_641060 to 641061, EPI_ISL_641063 to 641066, EPI_ISL_641071, EPI_ISL_641073 to 641074, EPI_ISL_641077 to 641078, EPI_ISL_641081 to 641085, EPI_ISL_641089, EPI_ISL_641093 to 641096, EPI_ISL_641099 to 641101, EPI_ISL_641103 to 641105, EPI_ISL_641107 to 641109, EPI_ISL_641111 to 641115, EPI_ISL_641117 to 641118, EPI_ISL_641120, EPI_ISL_641122 to 641124, EPI_ISL_641128 to 641138, EPI_ISL_641140 to 641142, EPI_ISL_641146 to 641162, EPI_ISL_641164, EPI_ISL_641167 to 641168, EPI_ISL_641170 to 641179, EPI_ISL_641181, EPI_ISL_641183, EPI_ISL_641185 to 641187, EPI_ISL_641189 to 641190, EPI_ISL_641192 to 641201, EPI_ISL_641208 to 641209, EPI_ISL_641211 to 641212, EPI_ISL_641214, EPI_ISL_641216 to 641217, EPI_ISL_641219, EPI_ISL_641221 to 641226, EPI_ISL_641228 to 641230, EPI_ISL_641233 to 641235, EPI_ISL_641238 to 641245, EPI_ISL_641248 to 641250, EPI_ISL_641252 to 641257, EPI_ISL_641259, EPI_ISL_641261 to 641264, EPI_ISL_641266 to 641268, EPI_ISL_641270 to 641272, EPI_ISL_641274 to 641276, EPI_ISL_641278, EPI_ISL_641281 to 641287, EPI_ISL_641289 to 641291, EPI_ISL_641293 to 641303, EPI_ISL_641307 to 641308, EPI_ISL_663437 to 663440, EPI_ISL_663529 to 663543, EPI_ISL_663546 to 664019, EPI_ISL_779625, EPI_ISL_854750, EPI_ISL_854754 to 854755, EPI_ISL_877570, EPI_ISL_877574, EPI_ISL_877580 to 877581, EPI_ISL_962827, EPI_ISL_979357, EPI_ISL_979360 to 979361, EPI_ISL_1033145 to 1033152, EPI_ISL_1033156 to 1033157			
see above	Microbiological Diagnostic Unit - Public Health Laboratory (MDU-PHL)	MDU-PHL	M. B.; M.L.; N.L.; Sait; Sait, M.; Schultz; Schultz M.; Schultz M. B.; Schultz M.B.; Seemann T.; Seemann, T.; Sherry; Sherry, N.
EPI_ISL_518651	Microbiological Diagnostic Unit - Public Health Laboratory (MDU-PHL)	Microbiological Diagnostic Unit - Public Health Laboratory (MDU-PHL)	Sait, M.; Schultz M.; Seemann T.; Sherry, N.
EPI_ISL_649149 to 649151	Microbiological Diagnostic Unit - Public Health Laboratory (MDU-PHL), The Peter Doherty Institute for Infection and Immunity	Microbiological Diagnostic Unit - Public Health Laboratory (MDU-PHL), The Peter Doherty Institute for Infection and Immunity	Caly, L.; Druce, J.; M.B.; M.L.; N.L.; Sait; Schultz; Seemann, T.; Sherry
EPI_ISL_419714 to 419723, EPI_ISL_419725 to 419732, EPI_ISL_419823 to 419825, EPI_ISL_419827 to 419830, EPI_ISL_419980, EPI_ISL_419999 to 420003, EPI_ISL_420006 to 420017, EPI_ISL_426906 to 426907, EPI_ISL_426910, EPI_ISL_426914, EPI_ISL_426917 to 426922, EPI_ISL_427054 to 427058, EPI_ISL_427060 to 427063, EPI_ISL_427065 to 427066, EPI_ISL_427068 to 427074, EPI_ISL_427076, EPI_ISL_427078, EPI_ISL_427133, EPI_ISL_427148 to 427149, EPI_ISL_430471 to 430472, EPI_ISL_430639 to 430640, EPI_ISL_430643, EPI_ISL_430645 to 430647, EPI_ISL_430649, EPI_ISL_430651, EPI_ISL_430653 to 430655, EPI_ISL_430658 to 430659, EPI_ISL_430661, EPI_ISL_430663, EPI_ISL_430669 to 430670, EPI_ISL_430673 to 430674, EPI_ISL_430676 to 430684, EPI_ISL_430686 to 430687, EPI_ISL_430716			
see above	Microbiological Diagnostic Unit Public Health Laboratory	Microbiological Diagnostic Unit Public Health Laboratory	Sait, M.; Schultz M.; Seemann T.; Sherry, N.
EPI_ISL_456478 to 456479, EPI_ISL_456595	Microbiological Diagnostic Unit Public Health Laboratory	Microbiological Diagnostic Unit Public Health Laboratory, The Peter Doherty Institute for Infection and Immunity	Sait, M.; Schultz M.; Seemann T.; Sherry, N.
EPI_ISL_447899	Microbiology	Microbiology	Ahmed; Hasanuzzaman, M.; Islam; M. and Saha; M.S.; M.S.I.; Malaker, R.; Rahman, H.; S.K.; Saha, S.; Sajib; Z.B.
EPI_ISL_513925	Microbiology & Bioinformatics and Biostatistics, Kohat University of Science and Technology (Pakistan) & Shanghai Jiao Tong University (China)	Microbiology & Bioinformatics and Biostatistics, Kohat University of Science and Technology (Pakistan) & Shanghai Jiao Tong University (China)	A.S.; Ali, S.; D.Q.; Khan; M.T.; Muhammad; N. and Wei; T.A.
EPI_ISL_524450	Microbiology & Immunology, University of North Carolina	Microbiology & Immunology, University of North Carolina	A.B.; A.G.; Bailey; C.P.; Caro-Vegas; Dittmer, D.; Eason; J.T.; Juarez, A.; L.J.; Landis; M.B.; McNamara; Miller; Moorad, R.; Pluta; R.P.; Seltzer; T.A.; Thompson, C.; Vahrson, W.; Villamor, F.
EPI_ISL_528788, EPI_ISL_528790 to 528791, EPI_ISL_528793, EPI_ISL_528796, EPI_ISL_528799, EPI_ISL_528801 to 528803, EPI_ISL_528805 to 528808, EPI_ISL_541756, EPI_ISL_541767, EPI_ISL_541773			
see above	Microbiology Department, Barking Havering and Redbridge University Hospitals NHS trust	Wellcome Sanger Institute for the COVID-19 Genomics UK (COG-UK) consortium	Amy Ash; Cherian Koshy and Alex Alderton; Cordelia Langford; David K. Jackson; Dominic Kwiatkowski; Ewan Harrison; Fatima Ali; Ian Johnston; John Sillitoe on behalf of the Wellcome Sanger Institute COVID-19 Surveillance Team; John Sillitoe on behalf of the Wellcome Sanger Institute COVID-19 Surveillance Team (http://www.sanger.ac.uk/covid-team); Roberto Amato; Sonia Goncalves
EPI_ISL_482069, EPI_ISL_482071 to 482074, EPI_ISL_482076, EPI_ISL_482078 to 482079, EPI_ISL_482081, EPI_ISL_482084 to 482086, EPI_ISL_482088 to 482090, EPI_ISL_482092 to 482095, EPI_ISL_482099 to 482100, EPI_ISL_482103 to 482104, EPI_ISL_482106, EPI_ISL_482108 to 482109, EPI_ISL_482111 to 482114, EPI_ISL_482116 to 482118, EPI_ISL_488837 to 488839, EPI_ISL_488842 to 488844, EPI_ISL_488848, EPI_ISL_488850 to 488852, EPI_ISL_488857 to 488860, EPI_ISL_488862, EPI_ISL_488865 to 488871, EPI_ISL_488873			
see above	Microbiology Department, Hereford County Hospital	Wellcome Sanger Institute for the COVID-19 Genomics UK (COG-UK) consortium	Alison Johnson; Cordelia Langford; David K. Jackson; Dominic Kwiatkowski; Ewan Harrison; Fenella Halstead; Ian Johnston; Jane Thomas; John Sillitoe on behalf of the Wellcome Sanger Institute COVID-19 Surveillance Team (http://www.sanger.ac.uk/covid-team); Roberto Amato; Samantha Lamb and Alex Alderton; Sonia Goncalves; Venkat Sivaprakasam; Wendy Hogsden
EPI_ISL_833183, EPI_ISL_848199	Microbiology Department. Complexo Hospitalario Universitario de Vigo	Microbiology Department. Complexo Hospitalario Universitario de Vigo	Complexo Hospitalario Universitario de Vigo (CHUVI). EPICOVIGAL.; Complexo Hospitalario Universitario de Vigo. EPICOVIGAL.; Microbiology Department
EPI_ISL_833184	Microbiology Department. Hospital Clinico Universitario de Santiago	Microbiology Department. Complexo Hospitalario Universitario de Vigo	Microbiology Departments from Complexo Hospitalario Universitario de Vigo and Hospital Clinico Universitario de Santiago. EPICOVIGAL.
EPI_ISL_462991	Microbiology Division	Microbiology Division	Flores, H.
EPI_ISL_529080 to 529101, EPI_ISL_529103, EPI_ISL_529105 to 529108	Microbiology Division, SC DHEC	Microbiology Division, SC DHEC	Flores, H.
EPI_ISL_447281 to 447297, EPI_ISL_447299 to 447303, EPI_ISL_447305 to 447310	Microbiology Division, Barzilai University Medical Center	Stern Lab	Stern Lab
EPI_ISL_538390 to 538393, EPI_ISL_538395 to 538397, EPI_ISL_538399 to 538402, EPI_ISL_538404, EPI_ISL_538406 to 538407, EPI_ISL_538410 to 538413, EPI_ISL_538415, EPI_ISL_538417 to 538418, EPI_ISL_538420 to 538423, EPI_ISL_538425 to 538428, EPI_ISL_538430 to 538434, EPI_ISL_539879, EPI_ISL_541663 to 541670, EPI_ISL_541672 to 541675, EPI_ISL_541677 to 541679, EPI_ISL_574543 to 574547, EPI_ISL_574550 to 574555, EPI_ISL_574557 to 574571, EPI_ISL_574573 to 574575			
see above	Microbiology Division, South Carolina Department of Health and Environmental Control	Microbiology Division, South Carolina Department of Health and Environmental Control	Flores, H.
EPI_ISL_981946 to 981966, EPI_ISL_981968 to 981974	Microbiology Service, Hospital Universitario Clinico San Cecilio, Granada	Microbiology Service, Hospital Universitario Clinico San Cecilio, Granada	Adolfo de Salazar; Ana Fuentes; Federico García; Laura Viñuela; Natalia Chueca
EPI_ISL_468339 to 468340	Microbiology Service, University Hospital of A Coruna-Biomedical Research Institute	Genomes & Disease, Center for Research in Molecular Medicine and Chronic Diseases, University of Santiago de Compostela	Ana Pequeno-Valtierra; Cristina Gomez; Daniel Garcia-Souto; G Bou; Javier Temes; Jorge Arca; Jorge Rodriguez-Castro; Jose M. C. Tubio; Juan A. Vallejo; Kelly Conde; M Poza; Martin Santamarina; Soraya Rumbo

EPI_ISL_455312 to 455313	Microbiology Unit, Department of Pathology & Laboratory Medicine, IIUM Medical Centre	SEA Microbiome Unit, Faculty of Industrial Sciences & Technology, Universiti Malaysia Pahang	Ahmad Hafiz Bin Zulkifly; Ahmad Hafiz Zulkifly; Ahmad Mahfuz Gazali; Hajar Fauzan Ahmad; IIUM Medical Centre Covid19 Taskforce; Mohd Fazli Farida Asras; Mohd Nazli Bin Kamarulzaman; Muhammad Adam Lee Abdullah; Norhidayah Binti Kamarudin; Norhidayah Kamarudin; UMP Covid19 Team
EPI_ISL_418267, EPI_ISL_418269	Microbiology and Immunology department, Pasteur institute in Ho Chi Minh city	Microbiology and Immunology department, Pasteur institute in Ho Chi Minh city	A.H.; C.Q.; Cao; D.T.; Dao; H.Q.; H.T.; H.T.T.; Hoang; Huynh; K.C.; L.T.; L.T. and Nguyen; L.T.K.; Le; Luong; M.H.; N.P.H.; N.T.; Nguyen; Pham; Phan; Phung; Q.C.; Q.D.; S.N.; T.M.; T.N.A.; T.T.; T.T.N.; T.V.; T.V. and Phan; Tran; Tran; T.; Vo; Vu
EPI_ISL_509424 to 509429	Microbiology and Immunology, University of South Alabama	Microbiology and Immunology, University of South Alabama	Borchert; Fouty, B.; G.M.; Houserova, D.; J.O.; R.A.; R.R.; Rayner; Roberts; Wood
EPI_ISL_738147, EPI_ISL_738194, EPI_ISL_738243, EPI_ISL_825156, EPI_ISL_911524 to 911525, EPI_ISL_983097 to 983098	Microbiology and Virology Unit, Florence Careggi University Hospital	Microbiology and Virology Unit, Florence Careggi University Hospital	Alberto Antonelli; Emanuele Gori; Fabio Morecchiato; Gian Maria Rossolini; Ilaria Baccani; Marco Coppi; Noemi Aiezza; Simona Pollini; Vincenzo Di Pilato
EPI_ISL_447260, EPI_ISL_447263 to 447265, EPI_ISL_447267 to 447269, EPI_ISL_447271 to 447275, EPI_ISL_447277, EPI_ISL_447279 to 447280	Microbiology laboratory, Assuta Ashdod University-Affiliated Hospital	Stern Lab	Stern Lab
EPI_ISL_613459 to 613462	Microbiology, Koc University	Microbiology, Koc University	Can, F.; Dogan, O.; Kuskucu; M.A.; Nurtop, E.; Ozer, B.
EPI_ISL_547433 to 547444, EPI_ISL_613570, EPI_ISL_613636 to 613705, EPI_ISL_613958, EPI_ISL_614982 to 614994, EPI_ISL_614997 to 615003, EPI_ISL_615005 to 615021, EPI_ISL_615023 to 615035, EPI_ISL_615037 to 615042, EPI_ISL_615044 to 615049, EPI_ISL_615051, EPI_ISL_615053 to 615059, EPI_ISL_615061 to 615062, EPI_ISL_632797 to 632813, EPI_ISL_632815 to 632822, EPI_ISL_637190 to 637196, EPI_ISL_637198, EPI_ISL_637202 to 637203, EPI_ISL_637206, EPI_ISL_637210 to 637212, EPI_ISL_637217 to 637219, EPI_ISL_637222, EPI_ISL_637224 to 637227, EPI_ISL_637231, EPI_ISL_671270 to 671271, EPI_ISL_671273 to 671278, EPI_ISL_671280 to 671282, EPI_ISL_671285 to 671287, EPI_ISL_671289 to 671294, EPI_ISL_671296 to 671297, EPI_ISL_671299 to 671306, EPI_ISL_671308 to 671310, EPI_ISL_671312 to 671317, EPI_ISL_671319 to 671325, EPI_ISL_671327, EPI_ISL_671329, EPI_ISL_671332			
see above	Microbiology, Department of Pathology, St. Bernard's Hospital, Gibraltar Health Authority	Respiratory Virus Unit, Microbiology Services Colindale, Public Health England	Charlotte Gillborn-Jones (Gibraltar); Dr Nicholas Cortes (Gibraltar); PHE Covid Sequencing Team
EPI_ISL_709959 to 710033, EPI_ISL_710535, EPI_ISL_766318 to 766394, EPI_ISL_769872	Microbiology, Department of Pathology, St. Bernard's Hospital, Gibraltar Health Authority	Respiratory Virus Unit, National Infection Service, Public Health England	Charlotte Gillborn-Jones (Gibraltar); Dr Nicholas Cortes (Gibraltar); PHE Covid Sequencing Team
EPI_ISL_667759, EPI_ISL_667762, EPI_ISL_667771	Microbiology, Infectious Diseases and Immunology, Centre de Recherche du Centre Hospitalier de l'Universite de Montreal	Microbiology, Infectious Diseases and Immunology, Centre de Recherche du Centre Hospitalier de l'Universite de Montreal	Benoit, P.; Coutlee, F.; Gagnon, S.; Grandjean-Lapierre, S.; Hardy, I.; Kaufmann, D.; Point, F.; Tremblay, C.
EPI_ISL_513585, EPI_ISL_513587 to 513588	Microbiology, Pathology, Kettering General Hospital	Wellcome Sanger Institute for the COVID-19 Genomics UK (COG-UK) consortium	Anita Kenyon; Cordelia Langford; David K. Jackson; Dominic Kwiatkowski; Ewan Harrison; Ian Johnston; John Sillitoe on behalf of the Wellcome Sanger Institute COVID-19 Surveillance Team (http://www.sanger.ac.uk/covid-team); Roberto Amato; Sahar El-dirdiri; Sonia Goncalves; Thomas Davis and Alex Alderton
EPI_ISL_434571	Microbiology, The University of Hong Kong	Microbiology, The University of Hong Kong	Chan; J.F.W. and Yuen; K.-Y.
EPI_ISL_475717 to 475721	Microbiology, University Hospital Donostia	Microbiology, University Hospital Donostia	Cilla, G.; J.M.; Marimon; Montes, M.; Pineiro, L.
EPI_ISL_486646, EPI_ISL_486650 to 486653, EPI_ISL_486655, EPI_ISL_486657 to 486660, EPI_ISL_486662 to 486665	Microbiology, Virology and Biemergency Laboratory-ASST FBF Sacco	Microbiology, Virology and Biemergency Laboratory-ASST FBF Sacco	Comandatore F; Mancon A; Micheli V; Rimoldi SG; Romeri F
EPI_ISL_456213 to 456214, EPI_ISL_456255 to 456258, EPI_ISL_456376, EPI_ISL_456385 to 456386, EPI_ISL_456403, EPI_ISL_548002, EPI_ISL_548004 to 548006, EPI_ISL_548008 to 548011, EPI_ISL_548081 to 548082, EPI_ISL_548123 to 548124, EPI_ISL_548127, EPI_ISL_548132, EPI_ISL_548143, EPI_ISL_579100, EPI_ISL_579102 to 579104, EPI_ISL_579129 to 579132, EPI_ISL_579228 to 579230, EPI_ISL_579294 to 579310, EPI_ISL_579313 to 579314, EPI_ISL_579404, EPI_ISL_622787, EPI_ISL_622792, EPI_ISL_622802, EPI_ISL_622817 to 622820, EPI_ISL_622831 to 622832, EPI_ISL_637086, EPI_ISL_649121 to 649122, EPI_ISL_661265, EPI_ISL_682279 to 682282, EPI_ISL_682287 to 682290, EPI_ISL_707806 to 707807, EPI_ISL_732968 to 732970, EPI_ISL_755624, EPI_ISL_755634 to 755635, EPI_ISL_794624 to 794625, EPI_ISL_877215, EPI_ISL_877217, EPI_ISL_1016855, EPI_ISL_1016857, EPI_ISL_1016863, EPI_ISL_1016865, EPI_ISL_1016872, EPI_ISL_1016874 to 1016876			
see above	Middlemore Hospital	Institute of Environmental Science and Research (ESR)	Anja Werno; Antje van der Linden; Arlo Upton; Chris Mansell; David Hammer; Dragana Drinkovic; Erasmus Smit; Gary McAuliffe; Hana Sofia Andersson; Hermes Perez; James Ussher; Jill Sherwood; Jing Wang; Joep de Ligt; Josh Freeman; Julia Howard; Juliet Elvy; Lauren Jelly; Mary DeAlmeida; Matt Blakiston; Matt Storey; Matthew Rogers; Max Bloomfield; Michael Addidle; Michelle Balm; Muhammad Faisal; Nikki Freed; Olin Silander; Sally Roberts; Sarah Jefferies; Sharmini Muttaiyah; Susan Morpeth; Susan Taylor; Timothy Blackmore; Vani Sathyendran; Veronica Playle; Virginia Hope; Xiaoyun Ren
EPI_ISL_661291 to 661292	Mikrobiologen	The Public Health Agency of Sweden	Department of Microbiology; The Public Health Agency of Sweden
EPI_ISL_678273 to 678288	Mikrobiologie, RARI	Mikrobiologie, RARI	A.V.; Badanin; D.V.; E.A.; Fedorov; Guseva; Krasnov; Kutyrev; N.A.; N.P.; Naryshkina; Portenko; S.A.; Sharapova; Shcherbakova; Sosedova; V.V.; Y.M.
EPI_ISL_735413	Militello Centro de Diagnosticos e Biopesquisa Clinica	Instituto Adolfo Lutz, Interdiciplinary Procedures Center, Strategic Laboratory	Claudia Regina Gonçalves; Claudio Tavares Sacchi; Erica Valessa Ramos Gomes; Karoline Rodrigues Campos
EPI_ISL_718132 to 718146, EPI_ISL_718148 to 718166, EPI_ISL_718168 to 718173, EPI_ISL_718175, EPI_ISL_718177, EPI_ISL_718179 to 718181, EPI_ISL_718183 to 718184, EPI_ISL_718186 to 718190, EPI_ISL_718193 to 718196, EPI_ISL_718199 to 718202, EPI_ISL_718206 to 718215, EPI_ISL_718217, EPI_ISL_718220 to 718221			
see above	Ministry of Health Hospitals	Institute of Health and Community Medicine	Chan Chia Jui; Chua Hock Hin; David Perera; Ooi Mong How; Tonnii Sia Loong Loong; Wong Jyn Shan; Wong Kiing Aik
EPI_ISL_428712 to 428723, EPI_ISL_429861 to 429873, EPI_ISL_437304 to 437335, EPI_ISL_811139 to 811140, EPI_ISL_811142, EPI_ISL_812763 to 812764, EPI_ISL_812773, EPI_ISL_894246 to 894276, EPI_ISL_935073 to 935078, EPI_ISL_935080 to 935088, EPI_ISL_935102, EPI_ISL_939627, EPI_ISL_940634 to 940642, EPI_ISL_940646 to 940647, EPI_ISL_940649 to 940655, EPI_ISL_940658 to 940659, EPI_ISL_940661 to 940667, EPI_ISL_940670 to 940675, EPI_ISL_940677 to 940682, EPI_ISL_983410 to 983420, EPI_ISL_983422 to 983432, EPI_ISL_1008616, EPI_ISL_1008618 to 1008620, EPI_ISL_1008623, EPI_ISL_1008628, EPI_ISL_1008630 to 1008632, EPI_ISL_1008634 to 1008646, EPI_ISL_1020421 to 1020461, EPI_ISL_1020464 to 1020473, EPI_ISL_1020479 to 1020483, EPI_ISL_1034233 to 1034235, EPI_ISL_1034238 to 1034243, EPI_ISL_1034245 to 1034247, EPI_ISL_1034249, EPI_ISL_1034251 to 1034259			
see above	Ministry of Health Turkey	Ministry of Health Turkey	Ayse Basak Altas; Aye Baak Alta; Fatma Bayrakdar; Gulay Korukluoglu; Gülay Korukluolu; Selçuk Klç; Suleyman Yalcin; Süleyman Yalcin; Süleyman Yalçn; Tülin Demir; Yasemin Cosgun; Yasemin Cogun
EPI_ISL_427408, EPI_ISL_427416 to 427418	Ministry of Public Health (MoPH)	Biomedical Research Center (BRC)	Abdullatif Al-Khal; Ajaeb D. M. H. Al-Nabet; Asmaa A. Al-Thani.; Einas A. E. Al-Kuwari; Fatiha M. Benslimane; Hadi M. Yassine; Hamad E. Al-Romaihi; Heba A. Al-Khatib; Mohammed Al-Thani; Muna A. S. Al-Maslamani; Nourah B. M. Younes; Peter V. Coyle; Salih Al-Marri; Sonia Boughattas
EPI_ISL_634857	Minneapolis VA Health Care System	Minnesota Department of Health, Public Health Laboratory	Alexandra Lorentz; Jacob Garfin; Matt Plumb; and Xiong Wang
EPI_ISL_414589 to 414590, EPI_ISL_417191 to 417192, EPI_ISL_417194, EPI_ISL_417196, EPI_ISL_417469 to 417480, EPI_ISL_417492 to 417503, EPI_ISL_419388, EPI_ISL_419390 to 419397, EPI_ISL_421683 to 421692, EPI_ISL_427273 to 427287, EPI_ISL_437361 to 437386, EPI_ISL_450748 to 450780, EPI_ISL_460045 to 460073, EPI_ISL_460075 to 460078, EPI_ISL_462845 to 462862, EPI_ISL_462864 to 462895, EPI_ISL_462897 to 462911, EPI_ISL_470747 to 470789, EPI_ISL_476706 to 476766, EPI_ISL_482946 to 482966, EPI_ISL_482988 to 483017, EPI_ISL_495610 to 495628, EPI_ISL_496916 to 496917, EPI_ISL_496919 to 496930, EPI_ISL_507934 to 507956, EPI_ISL_507973 to 507983, EPI_ISL_514616 to 514618, EPI_ISL_514658 to 514672, EPI_ISL_515262, EPI_ISL_515269, EPI_ISL_518855, EPI_ISL_527573 to 527629, EPI_ISL_530172 to 530213, EPI_ISL_530215 to 530224, EPI_ISL_539824 to 539841, EPI_ISL_560743 to 560791, EPI_ISL_576228 to 576256, EPI_ISL_590753 to 590759, EPI_ISL_590761 to 590822, EPI_ISL_590837, EPI_ISL_602956 to 602999, EPI_ISL_603006 to 603009, EPI_ISL_603015, EPI_ISL_603018 to 603020, EPI_ISL_634838 to 634856, EPI_ISL_634858 to 634863, EPI_ISL_634866 to 634875, EPI_ISL_654695, EPI_ISL_654700 to 654729, EPI_ISL_683729 to 683750, EPI_ISL_708460 to 708482, EPI_ISL_708485 to 708503, EPI_ISL_723118 to 723140, EPI_ISL_723146			
see above	Minnesota Department of Health, Public Health Laboratory	Minnesota Department of Health, Public Health Laboratory	Alexandra Lorentz; Jacob Garfin; Jacob Garfin and Xiong Wang; Jake Garfin and Xiong Wang; Matt Plumb; Xiong Wang; and Xiong Wang
EPI_ISL_900507 to 900509	Mirialis	CNR Virus des Infections Respiratoires - France SUD	Antonin Bal; Bruno Lina; Gregory Destras; Gwendolyne Burfin; Hadrien Règue; Laurence Josset; Martine Valette; Quentin Semanas
EPI_ISL_640025	Mitchells Plain CHC wc MHC	NHLS/UCT	Arash Iranzadeh; Bruna Galvao; Carolyn Williamson; Deelan Doolabh; Diana Hardie; Innocent Mudau; Kruger Marais; Lynn Tyers; Marvin Hsiao; Stephen Korsman
EPI_ISL_640020, EPI_ISL_640028, EPI_ISL_640065, EPI_ISL_700489, EPI_ISL_700572, EPI_ISL_700597	Mitchells Plain Hospital wc MPH	NHLS/UCT	Arash Iranzadeh; Bruna Galvao; Carolyn Williamson; Deelan Doolabh; Diana Hardie; Innocent Mudau; Kruger Marais; Lynn Tyers; Marvin Hsiao; Stephen Korsman
EPI_ISL_960095, EPI_ISL_960098, EPI_ISL_960100	Mitchells Plain Hospital wc MPH	National Health Laboratory Service/UCT	Arash Iranzadeh; Bruna Galvao; Carolyn Williamson; Deelan Doolabh; Diana Hardie; Innocent Mudau; Kruger Marais; Lynn Tyers; Marvin Hsiao; Stephen Korsman
EPI_ISL_458079	Mitra Keluarga Hospital Kenjeran	Institute of Tropical Disease, Universitas Airlangga	Aldise M Nastri; Anastasia W Jefuna; Gatot Soegiarto; Jezzy R Dewantari; Kazufumi Shimizu; Krisnoadi Rahardjo; Laksmi Wulandari; Maria I Lusida; Mitsuhiro Nishimura; Resti Yudhawati; Retno A Setyoningrum; Rima R Prasetya; Soetjipto; Yasuko Mori; Yohko K Shimizu
EPI_ISL_759964	Mitra Keluarga Hospital Waru	Institute of Tropical Disease, Universitas Airlangga	Aldise M Nastri; Christina Dian Anggraeni; Gatot Soegiarto; Jezzy R Dewantari; Kazufumi Shimizu; Krisnoadi Rahardjo; Laksmi Wulandari; Maria I Lusida; Resti Yudhawati; Rima R Prasetya; Soetjipto; Yasuko Mori

EPI_ISL_437192	Mitra Keluarga Kelapa Gading Hospital	Eijkman Institute for Molecular Biology, Ministry of Research and Technology/National Agency for Research and Innovation	Amin Soebandrio; David H Muljono; Edison Johar; Frilasita A Yudhaputri; Hidayat Trimarsanto; Khin Saw Myint; Safarina G Malik
EPI_ISL_435119	Mohammed Bin Rashid University of Medicine and Health Sciences	Al Jalila Children's Hospital	Abdulmajeed Alkhaja; Abiola Catherine Senok; Ahmad Abou Tayoun; Alawi Alsheikh-Ali; Divinlal Harilal; Hamda Khansaheb; Hanan Al Suwaidi; Mohammed Uddin; Norbert Nowotny; Qutayba Hamid; Rabih Halwani; Rifat Hamoudi; Rupa Murthy Varghese; Sathishkumar Ramaswamy; Tom Loney; Zulfa Omar Deesi
EPI_ISL_435120 to 435143, EPI_ISL_463740, EPI_ISL_469276 to 469281, EPI_ISL_520663 to 520664, EPI_ISL_520666 to 520698, EPI_ISL_520700 to 520744, EPI_ISL_523952			
see above	Mohammed Bin Rashid University of Medicine and Health Sciences	Al Jalila Genomics Center	Abdulmajeed Alkhaja; Abiola Catherine Senok; Ahmad Abou Tayoun; Alawi Alsheikh-Ali; Divinlal Harilal; Hamda Khansaheb; Hanan Al Suwaidi; Mohammed Uddin; Norbert Nowotny; Qutayba Hamid; Rabih Halwani; Rifat Hamoudi; Rupa Murthy Varghese; Sathishkumar Ramaswamy; Tom Loney; Zulfa Omar Deesi
EPI_ISL_660259, EPI_ISL_660261 to 660263	Molecualr Diagnostic Services (MDS)	KRISP, KZN Research Innovation and Sequencing Platform	Giandhari J; Khan S; Lessells R; Mdlalose K; Pillay S; Tegally H; Wilkinson E; York D; de Oliveira T
EPI_ISL_849763	Molecular Biology, IZS Sicilia	Molecular Biology, IZS Sicilia	Di Gaudio, F.; Reale, S.; S. and Vitale, F.; Scibetta; Seidita, G.
EPI_ISL_421573 to 421576	Molecular Diagnostic Services	KRISP, KZN Research Innovation and Sequencing Platform	Chimukangara B; Deforche K; Giandhari J; Lessells R; Ngcapu S; Pillay S; Samsunder N; Tegally H; Wilkinson E; de Oliveira T
EPI_ISL_467431, EPI_ISL_467444 to 467446, EPI_ISL_467475 to 467486, EPI_ISL_467488 to 467491, EPI_ISL_467494 to 467504, EPI_ISL_467506, EPI_ISL_482702, EPI_ISL_482704 to 482705, EPI_ISL_482708 to 482709, EPI_ISL_482716 to 482723, EPI_ISL_482852 to 482853, EPI_ISL_482862, EPI_ISL_482867, EPI_ISL_487329 to 487330, EPI_ISL_487332, EPI_ISL_487334, EPI_ISL_487336 to 487340			
see above	Molecular Diagnostics Services (MDS)	KRISP, KZN Research Innovation and Sequencing Platform	Chimukangara B; Giandhari J; Khan S; Lessells R; Mdlalose K; Pillay S; Tegally H; Wilkinson E; York D; de Oliveira T
EPI_ISL_462995 to 463000	Molecular Genetics	Molecular Genetics	Coto, E.; Gomez, J.
EPI_ISL_444455, EPI_ISL_444519	Molecular Infectious Disease	Molecular Infectious Disease	Anderson; B.P.; Gerasimova, A.; Kagan; R.M. and Owen, R.; Rosenthal; S.H.
EPI_ISL_681673 to 681675, EPI_ISL_681681 to 681684, EPI_ISL_681689 to 681690, EPI_ISL_681692, EPI_ISL_681695 to 681698, EPI_ISL_681700 to 681701	Molecular Medicine Laboratory, University of Magallanes	Centro Asistencial Docente y de Investigacion, Universidad de Magallanes	Diego Alvarez; Hermy Alvarez; Jacqueline Aldridge; Jorge González; Marcelo Navarrete; Marco Montes de Oca; Roberto Uribe-Paredes
EPI_ISL_451306 to 451309, EPI_ISL_460079 to 460095	Molecular Virology Unit, Fondazione IRCCS Policlinico San Matteo , Pavia	Laboratory of Virology, INMI Lazzaro Spallanzani IRCCS	Antonino Di Caro; Antonio Piralla; Barbara Bartolini; Cesare E.M. Gruber; Fausto Baldanti; Maria R. Capobianchi; Martina Rueca
EPI_ISL_510535	Molecular Virology, Instituto Carlos Chagas / Fiocruz Paraná	Universidade Federal do Parana (UFPR)	Duarte dos Santos, C.; Raboni, S.; Suzukawa, A.; Tscha, M.; Zanluca, C.
EPI_ISL_460096, EPI_ISL_460604 to 460605, EPI_ISL_462149 to 462150, EPI_ISL_467774 to 467775, EPI_ISL_470539, EPI_ISL_479620 to 479624, EPI_ISL_486815 to 486829, EPI_ISL_534337 to 534349, EPI_ISL_610227 to 610244, EPI_ISL_653922 to 653932, EPI_ISL_660435 to 660438, EPI_ISL_717965 to 717974, EPI_ISL_732659 to 732676, EPI_ISL_745223 to 745224, EPI_ISL_745233 to 745259, EPI_ISL_754196 to 754203, EPI_ISL_754205 to 754214			
see above	Molecular diagnostic laboratory of Federal Budget Institution of Science "Central Research Institute of Epidemiology" of The Federal Service on Customers' Rights Protection and Human Well-being Surveillance	Group of Genomics and Postgenomic Technologies of Central Research Institute of Epidemilology	Akimkin VG; Bukharina A.Y.; Bukharina AY; Bulanenko VP; Dudorova A.V.; Dudorova AV; Kaptelova VV; Korneenko EV; Saenko SS; Samoilov AE; Shipulina OY; Sizova TV; Speranskaya AS; Tivanova EV; Valdokhina AV
EPI_ISL_614347 to 614349, EPI_ISL_614351 to 614368, EPI_ISL_614371 to 614372, EPI_ISL_614375 to 614377, EPI_ISL_614379 to 614381, EPI_ISL_614384, EPI_ISL_614386 to 614388, EPI_ISL_614391, EPI_ISL_614393, EPI_ISL_681829, EPI_ISL_681832 to 681836, EPI_ISL_681840 to 681841			
see above	Molecular diagnostic unit for viral haemorrhagic fevers and emerging viruses, Bouaké CHU Laboratory	Project group Epidemology of Highly Pathogenic Microorganisms, Robert Koch-Institute	Adjaratou Traoré; Bamba Fatoumata Touré; Chantal Akoua-Koffi; Coulibaly Mbegnan; Diané Bamourou; Essia Belarbi; Etilé Anoh; Fabian Leendertz; Grit Schubert; Kra Ouffoué; Monemo Pacome; Safiatou Karidioula; Soundélé Maïté
EPI_ISL_759860 to 759865	Molecular, ProgenaBiome	Molecular, ProgenaBiome	A.J.; Papoutsis
EPI_ISL_631513, EPI_ISL_631560 to 631561, EPI_ISL_631982, EPI_ISL_632111	Montefiore Medical Center	New York City Public Health Laboratory	Jade Wang; et al.
EPI_ISL_467945	Montefiore Medical Center, Dept. of Pathology, Clinical Virology	Albert Einstein College of Medicine, Dept. of Microbiology & Immunology, Chandran lab	Amanda Mengotto; Amy S. Fox; Ariel S. Wirchnianski; Catalina Florez; D. Yitzchak Goldstein; Denise Haslwanter; Duncan Kimmel; Ethan Laudermilch; J. Maximilian Fels; Johanna P. Daily; John M. Greally; Karin A. Skalina; Kartik Chandran; Libusha Kelly; M. Eugenia Dieterle; Michael B. Prystowsky; Robert H. Bortz III; Rohit K. Jangra; Ryan Forster; Saad Khan; Shahina B. Maqbool; Wendy A. Szymczak
EPI_ISL_739101, EPI_ISL_739329, EPI_ISL_739336, EPI_ISL_739364, EPI_ISL_739372, EPI_ISL_739546	Monterey County Public Health Lab	Chan-Zuckerberg Biohub	CZB Cliahub Consortium
EPI_ISL_426581, EPI_ISL_426883 to 426897	Motol University Hospital	Institute of Applied Biotechnologies a.s.	Adam Novotný; Jan Geryk; Kateina Kvapilová; Martin Kašný; Milan Macek; Pavel Devínek; Petr Brož; Petr Klempt; Petr Kvapil
EPI_ISL_631512, EPI_ISL_631525, EPI_ISL_631532, EPI_ISL_631534, EPI_ISL_631537, EPI_ISL_631549, EPI_ISL_631764, EPI_ISL_631785 to 631786, EPI_ISL_631830, EPI_ISL_631914, EPI_ISL_631983			
see above	Mount Sinai Hospital	New York City Public Health Laboratory	Jade Wang; et al.
EPI_ISL_632101 to 632102	Mount Sinai Queens	New York City Public Health Laboratory	Jade Wang; et al.
EPI_ISL_631880	Mount Sinai West	New York City Public Health Laboratory	Jade Wang; et al.
EPI_ISL_700479, EPI_ISL_1040701, EPI_ISL_1040712	Mowbray Maternity Hospital wc MMH	NHLS/UCT	Arash Iranzadeh; Bruna Galvao; Carolyn Williamson; Deelan Doolabh; Diana Hardie; Innocent Mudau; Kruger Marais; Lynn Tyers; Marvin Hsiao; Stephen Korsman
EPI_ISL_960104	Mowbray Maternity Hospital wc MMH	National Health Laboratory Service/UCT	Arash Iranzadeh; Bruna Galvao; Carolyn Williamson; Deelan Doolabh; Diana Hardie; Innocent Mudau; Kruger Marais; Lynn Tyers; Marvin Hsiao; Stephen Korsman
EPI_ISL_730622, EPI_ISL_730626 to 730629, EPI_ISL_730631 to 730633, EPI_ISL_730637 to 730639	Männedorf Hospital	Institute of Medical Virology, University of Zurich	Alexandra Trkola; Gabriela Ziltener; Jürg Böni; Maryam Zaheri; Michael Huber; Stefan Schmutz; Verena Kufner
EPI_ISL_490205 to 490208	München Klinik Schwabing	MGZ Medical Genetics Center	Dieter A. Wolf; Elke Holinski-Feder
EPI_ISL_510810 to 510816	NA	The Public Health Agency of Sweden	Anna Risberg; Anna-Malin Linde; Karin Tegmark-Wisell; Maria Lind Karlberg; Mattias Haukland; Mia Brytting; Olov Svartstrom; Oskar Karlsson Lindsjo; Petra Edquist; Reza Advani; Sandra Broddesson
EPI_ISL_424871 to 424873, EPI_ISL_426428, EPI_ISL_452124, EPI_ISL_452126, EPI_ISL_955298	NC State Laboratory of Public Health	Pathogen Discovery, Respiratory Viruses Branch, Division of Viral Diseases, Centers for Disease Control and Prevention	Alison S. Laufer Halpin; Anna Montmayeur; Anna Uehara; Christopher A. Elkins; Clinton R. Paden; Haibin Wang; Jing Zhang; Krista Queen; Mary S. Keckler; Peter Cook; Rachel Marine; Suxiang Tong; Yan Li; Ying Tao; Zachary Weiner
EPI_ISL_528683	NCDC Institute of Genomics and Integrative Biology	NCDC Institute of Genomics and Integrative Biology	Ajit Shewale; Akshay Kanakan; Anurag Agrawal; Azka Khan; Bharathram Uppili; Mahesh S. Dhar; Mohammed Faruq; Partha Rakshit; Pooja Sharma; RadhaKrishnan VS; Rajesh Pandey; Robin Marwal; Sandhya Kabra; Simmi Tiwari; Sujeet Singh; Tushar Nale; Vivekanand A
EPI_ISL_581506 to 581507	NCDC/IGIB	NCDC/IGIB	Ajit Shewale; Akshay Kanakan; Anurag Agrawal; Azka Khan; Bharathram Uppili; Mahesh S. Dhar; Mohammed Faruq; Nishu Tyagi; Partha Rakshit; Pooja Sharma; RadhaKrishnan VS; Rajesh Pandey; Robin Marwal; Sandhya Kabra; Simmi Tiwari; Sujeet Singh; Tushar Nale; Vivekanand A
EPI_ISL_751583, EPI_ISL_751585, EPI_ISL_751608, EPI_ISL_751638, EPI_ISL_751681, EPI_ISL_751732, EPI_ISL_751734			
see above	NE Public Health Laboratory	Genomics and Discovery, Respiratory Viruses Branch, Division of Viral Diseases, Centers for Disease Control and Prevention	Anna Montmayeur; Anna Uehara; Clinton R. Paden; Haibin Wang; Jing Zhang; Justin Lee; Krista Queen; Mili Sheth; Peter W. Cook; Rachel Marine; Suxiang Tong; Yan Li; Ying Tao
EPI_ISL_424874 to 424875	NE Public Health Laboratory	Pathogen Discovery, Respiratory Viruses Branch, Division of	Alison S. Laufer Halpin; Anna Uehara; Christopher A. Elkins; Clinton R. Paden; Haibin Wang; Jing Zhang; Krista Queen; Mary S. Keckler; Rachel Marine;

		Viral Diseases, Centers for Disease Control and Prevention	Suxiang Tong; Yan Li; Ying Tao
EPI_ISL_605882 to 605907, EPI_ISL_605909 to 605923	NGS Lab, DNA SOLUTION LTD.	NGS Lab, DNA SOLUTION LTD.	Chowdhury, M.; H.U.; Haider; Hasan; Hosen; K.N.; Khaleque, A.; Khan; Khan, M.; M.B.; M.F.A.; M.H.; M.I.; M.N.I.; Polol; Rabbi; Rahman, M.; Razu; Sufian, A.
EPI_ISL_420791 to 420792	NH Department of Health and Human Services Public Health Labs	Pathogen Discovery, Respiratory Viruses Branch, Division of Viral Diseases, Centers for Disease Control and Prevention	Alison S. Laufer Halpin; Anne Uehara; Christopher A. Elkins; Clinton R. Paden; Haibin Wang; Jasmine Padilla; Jing Zhang; Justin Lee; Krista Queen; Mary S. Keckler; Rachel Marine; Suxiang Tong; Yan Li; Ying Tao
EPI_ISL_751558, EPI_ISL_751582, EPI_ISL_751584, EPI_ISL_751587, EPI_ISL_751642, EPI_ISL_751678, EPI_ISL_751712, EPI_ISL_751735			
see above	NH Dept. of Health and Human Services Public Health Labs	Genomics and Discovery, Respiratory Viruses Branch, Division of Viral Diseases, Centers for Disease Control and Prevention	Anna Montmayeur; Anna Uehara; Clinton R. Paden; Haibin Wang; Jing Zhang; Justin Lee; Krista Queen; Mili Sheth; Peter W. Cook; Rachel Marine; Suxiang Tong; Yan Li; Ying Tao
EPI_ISL_424876 to 424877	NH Dept. of Health and Human Services Public Health Labs	Pathogen Discovery, Respiratory Viruses Branch, Division of Viral Diseases, Centers for Disease Control and Prevention	Alison S. Laufer Halpin; Anna Uehara; Christopher A. Elkins; Clinton R. Paden; Haibin Wang; Jing Zhang; Krista Queen; Mary S. Keckler; Rachel Marine; Suxiang Tong; Yan Li; Ying Tao
EPI_ISL_411950, EPI_ISL_411952 to 411953	NHC Key laboratory of Enteric Pathogenic Microbiology, Institute of Pathogenic Microbiology	Jiangsu Provincial Center for Disease Control & Prevention	Baoli Zhu; Bin Wu; Fengcai Zhu; Kangchen Zhao; Lunbiao Cui; Ming Wu; Tao Wu; Xiaojuan Zhu; Yin Chen; Yiyue Ge
EPI_ISL_825044 to 825045, EPI_ISL_825050 to 825053, EPI_ISL_825059 to 825060	NHL Municipal Medical College, Ahmedbad	Gujarat Biotechnology Research Centre	Afzal Ansari; Apurvasinh Puvar; Atit Shah; Bhavin Prajapati; Bimal Chauhan; Chaitanya Joshi; Dinesh Kumar; Janvi Raval; Jayshri Pethani; Labdhi Pandya; Madhvi Joshi; Monila Patel; NM Shaikh; Nikha Trivedi; Nitin Savaliya; Ramesh Pandit; Tanmay Mehta; Zarna Patel; Zuber Saiyed
EPI_ISL_682323 to 682331, EPI_ISL_682333 to 682335, EPI_ISL_682337 to 682338, EPI_ISL_682341 to 682342, EPI_ISL_682345, EPI_ISL_682347, EPI_ISL_682350 to 682351, EPI_ISL_684047			
see above	NHLS Universitas Academic	UFS Virology	D Goedhals; MM Nyaga; MT Mogotsi; P Nthiga; PA Bester; T de Oliveira
EPI_ISL_467437, EPI_ISL_467441 to 467443, EPI_ISL_467493, EPI_ISL_467507 to 467509, EPI_ISL_467511 to 467515, EPI_ISL_467517 to 467524, EPI_ISL_482710 to 482712, EPI_ISL_482726, EPI_ISL_487277, EPI_ISL_487280 to 487281, EPI_ISL_487288, EPI_ISL_487295, EPI_ISL_487297, EPI_ISL_487304, EPI_ISL_487308, EPI_ISL_487311 to 487314, EPI_ISL_487316, EPI_ISL_487318 to 487322, EPI_ISL_487324 to 487325, EPI_ISL_487328, EPI_ISL_495520, EPI_ISL_495527 to 495530, EPI_ISL_495534, EPI_ISL_495543 to 495544, EPI_ISL_495546, EPI_ISL_495549 to 495550, EPI_ISL_495557 to 495559, EPI_ISL_495561 to 495562, EPI_ISL_498055, EPI_ISL_498060 to 498062, EPI_ISL_498064 to 498067, EPI_ISL_498069 to 498075, EPI_ISL_498077 to 498079, EPI_ISL_498081 to 498082, EPI_ISL_498084 to 498126, EPI_ISL_509223 to 509226, EPI_ISL_509228 to 509258, EPI_ISL_509260 to 509267, EPI_ISL_509269 to 509271, EPI_ISL_509274 to 509276, EPI_ISL_509279, EPI_ISL_509281, EPI_ISL_509283 to 509290, EPI_ISL_509292 to 509297, EPI_ISL_509299 to 509358, EPI_ISL_509360 to 509369, EPI_ISL_515568 to 515570, EPI_ISL_515573 to 515575, EPI_ISL_515579 to 515580, EPI_ISL_515582, EPI_ISL_515584 to 515586, EPI_ISL_515589, EPI_ISL_515592 to 515594, EPI_ISL_515596 to 515597, EPI_ISL_515600 to 515604, EPI_ISL_515606 to 515607, EPI_ISL_515610, EPI_ISL_515612 to 515613, EPI_ISL_515615 to 515616, EPI_ISL_515619, EPI_ISL_515622 to 515623, EPI_ISL_515627 to 515628, EPI_ISL_515631, EPI_ISL_515633 to 515635, EPI_ISL_515639 to 515645, EPI_ISL_515647, EPI_ISL_515649 to 515662, EPI_ISL_515665 to 515666, EPI_ISL_515668 to 515669, EPI_ISL_515671 to 515672, EPI_ISL_515674 to 515676, EPI_ISL_515678 to 515694, EPI_ISL_515696 to 515712, EPI_ISL_515714 to 515716, EPI_ISL_515718 to 515725, EPI_ISL_515728 to 515734, EPI_ISL_515736 to 515741, EPI_ISL_515743 to 515745, EPI_ISL_515747 to 515749, EPI_ISL_515751 to 515754, EPI_ISL_515756 to 515760, EPI_ISL_515762 to 515764, EPI_ISL_515767, EPI_ISL_515769, EPI_ISL_515771, EPI_ISL_515773, EPI_ISL_515775 to 515778, EPI_ISL_515780 to 515784, EPI_ISL_515787, EPI_ISL_515789 to 515792, EPI_ISL_515794, EPI_ISL_515796 to 515797, EPI_ISL_515799, EPI_ISL_518034 to 518035, EPI_ISL_518037 to 518052, EPI_ISL_529720 to 529721, EPI_ISL_529723 to 529724, EPI_ISL_529726, EPI_ISL_529728 to 529745, EPI_ISL_529747 to 529753, EPI_ISL_529755 to 529767, EPI_ISL_529769 to 529780, EPI_ISL_529782 to 529794, EPI_ISL_529796 to 529802, EPI_ISL_535390, EPI_ISL_535392 to 535400, EPI_ISL_535403 to 535406, EPI_ISL_535408, EPI_ISL_535410, EPI_ISL_535412 to 535418, EPI_ISL_535420 to 535422, EPI_ISL_535424 to 535428, EPI_ISL_535430 to 535436, EPI_ISL_535438 to 535452, EPI_ISL_535454 to 535473, EPI_ISL_535475 to 535481, EPI_ISL_535483 to 535485, EPI_ISL_535487 to 535488, EPI_ISL_535496 to 535516, EPI_ISL_535518 to 535520, EPI_ISL_535522 to 535523, EPI_ISL_535526 to 535527, EPI_ISL_535529 to 535534, EPI_ISL_535536 to 535537, EPI_ISL_535539 to 535547, EPI_ISL_535551 to 535572, EPI_ISL_602632 to 602642, EPI_ISL_602644 to 602654, EPI_ISL_602656 to 602698, EPI_ISL_602700 to 602701, EPI_ISL_602703 to 602733, EPI_ISL_602735 to 602772, EPI_ISL_602775 to 602791, EPI_ISL_602793, EPI_ISL_602795 to 602804, EPI_ISL_602806 to 602815, EPI_ISL_602817 to 602818, EPI_ISL_602820 to 602822, EPI_ISL_602824 to 602826, EPI_ISL_602828, EPI_ISL_602831 to 602845, EPI_ISL_602848 to 602851, EPI_ISL_602853 to 602855, EPI_ISL_602858 to 602867, EPI_ISL_602869 to 602873, EPI_ISL_602875 to 602881, EPI_ISL_602883 to 602884, EPI_ISL_602887, EPI_ISL_602890, EPI_ISL_602892, EPI_ISL_602894 to 602896, EPI_ISL_602899 to 602900, EPI_ISL_602902, EPI_ISL_602904, EPI_ISL_602906 to 602908, EPI_ISL_602911 to 602913, EPI_ISL_602916 to 602917, EPI_ISL_602919 to 602920, EPI_ISL_602922 to 602924, EPI_ISL_602926, EPI_ISL_602928 to 602930, EPI_ISL_605785 to 605788, EPI_ISL_605790, EPI_ISL_660159 to 660166, EPI_ISL_660168 to 660169, EPI_ISL_660171 to 660176, EPI_ISL_660181, EPI_ISL_660185, EPI_ISL_660189, EPI_ISL_660191 to 660220, EPI_ISL_660223 to 660225, EPI_ISL_660227, EPI_ISL_660234 to 660246, EPI_ISL_660248 to 660255, EPI_ISL_660605 to 660606, EPI_ISL_660609, EPI_ISL_660613, EPI_ISL_660618 to 660628, EPI_ISL_660630 to 660636, EPI_ISL_660638 to 660645, EPI_ISL_660649 to 660652, EPI_ISL_660654 to 660655, EPI_ISL_660657, EPI_ISL_660661 to 660663, EPI_ISL_678598 to 678603, EPI_ISL_678605 to 678614, EPI_ISL_678616 to 678617, EPI_ISL_678619 to 678620, EPI_ISL_678622 to 678624, EPI_ISL_678626, EPI_ISL_678631, EPI_ISL_678637, EPI_ISL_736926, EPI_ISL_736984 to 736994, EPI_ISL_825109, EPI_ISL_825129 to 825130			
see above	NHLS-IALCH	KRISP, KZN Research Innovation and Sequencing Platform	Cele S; Chimukangara B; ChimukangaraB; Gazy I; Giandhari J; Karim F; Khan S; Lessells R; Mdlalose K; Pillay S; Sigal A; Tegally H; Wilkinson E; York D; de Oliveira T
EPI_ISL_860554 to 860571, EPI_ISL_860573 to 860575, EPI_ISL_860577 to 860585, EPI_ISL_860587 to 860588, EPI_ISL_860590 to 860594, EPI_ISL_860596 to 860612, EPI_ISL_860614 to 860630			
see above	NHLS-IALCH	KRISP, KZn Research Innovation and Sequencing Platform	Giandhari J; Khan S; Lessells R; Mdlalose K; Pillay S; Tegally H; Wilkinson E; York D; de Oliveira T
EPI_ISL_736928 to 736929	NHLS-UCT	KRISP, KZN Research Innovation and Sequencing Platform	Arash Iranzadeh; Bruna Galvao; Carolyn Williamson; Deelan Doolabh; Diana Hardie; Emanuel James San; Houriiyah Tegally; Innocent Mudau; Jennifer Giandhari; Kruger Marais; Lynn Tyers; Marvin Hsiao; Stephen Korsman; Sureshnee Pillay; Tulio de Oliveira
EPI_ISL_632242, EPI_ISL_632247 to 632249	NHSGGC West of Scotland Specialist Virology Centre	MRC-University of Glasgow Centre for Virus Research	Alasdair MacLean; Alex Alderton; Ana da Silva Filipe; Antonia Ho; Cordelia Langford; Daniel Mair; David K. Jackson; David L Robertson; Dominic Kwiatkowski; Emma Thomson; Ewan Harrison; Ian Johnston; James Shepherd; Jenna Nichols; John Sillitoe on behalf of the Wellcome Sanger Institute COVID-19 Surveillance Team (http://www.sanger.ac.uk/covid-team); Joseph Hughes; Kathy Li; Kathy Smollett; Kyriaki Nomikou; Lily Tong; Natasha Jesudason; Natasha Johnson; Rajiv Shah; Richard Orton; Roberto Amato; Rory Gunson; Sonia Goncalves; Sreenu Vattipally; Stephen Carmichael
EPI_ISL_459096 to 459100, EPI_ISL_459102 to 459107, EPI_ISL_459109, EPI_ISL_459111 to 459116, EPI_ISL_459118 to 459122, EPI_ISL_459124 to 459125, EPI_ISL_459127, EPI_ISL_459130 to 459134, EPI_ISL_459136 to 459138, EPI_ISL_459140 to 459142, EPI_ISL_459145 to 459147, EPI_ISL_459149, EPI_ISL_459151, EPI_ISL_459153 to 459158, EPI_ISL_459161, EPI_ISL_459164 to 459165, EPI_ISL_459506, EPI_ISL_459508 to 459510, EPI_ISL_459513 to 459514, EPI_ISL_459517 to 459522, EPI_ISL_459524 to 459530, EPI_ISL_459532 to 459536, EPI_ISL_459538 to 459539, EPI_ISL_459542, EPI_ISL_459544, EPI_ISL_459546 to 459548, EPI_ISL_459550, EPI_ISL_459552, EPI_ISL_459555 to 459563, EPI_ISL_459565 to 459566, EPI_ISL_459568 to 459569, EPI_ISL_459572, EPI_ISL_459574 to 459575, EPI_ISL_459577 to 459578, EPI_ISL_459580 to 459581, EPI_ISL_459583 to 459584, EPI_ISL_459586 to 459587, EPI_ISL_459589 to 459591, EPI_ISL_459593 to 459598, EPI_ISL_459600, EPI_ISL_459602 to 459603, EPI_ISL_459605 to 459610, EPI_ISL_459613 to 459614, EPI_ISL_459616 to 459617, EPI_ISL_459619, EPI_ISL_459621, EPI_ISL_459623 to 459626, EPI_ISL_459628 to 459632, EPI_ISL_459635 to 459638, EPI_ISL_459640 to 459641, EPI_ISL_459643 to 459649, EPI_ISL_459651 to 459652, EPI_ISL_459654, EPI_ISL_459656 to 459657, EPI_ISL_459659 to 459662, EPI_ISL_459665 to 459668, EPI_ISL_459670, EPI_ISL_459672, EPI_ISL_459678 to 459682, EPI_ISL_459684, EPI_ISL_459686, EPI_ISL_459688 to 459697, EPI_ISL_459699 to 459705, EPI_ISL_459707 to 459714, EPI_ISL_459716, EPI_ISL_459718, EPI_ISL_459720 to 459721, EPI_ISL_459724, EPI_ISL_469930 to 469935, EPI_ISL_469940 to 469941, EPI_ISL_469943 to 469950, EPI_ISL_469952, EPI_ISL_469954, EPI_ISL_469956 to 469957, EPI_ISL_469959 to 469969, EPI_ISL_469971 to 469974, EPI_ISL_469976 to 469978, EPI_ISL_469980 to 469986, EPI_ISL_469988 to 469991, EPI_ISL_469993 to 469994, EPI_ISL_469997, EPI_ISL_469999 to 470001, EPI_ISL_470005 to 470007, EPI_ISL_470009 to 470010, EPI_ISL_489380, EPI_ISL_489391 to 489392, EPI_ISL_489396, EPI_ISL_489401, EPI_ISL_489407 to 489408, EPI_ISL_489418, EPI_ISL_489429, EPI_ISL_489442, EPI_ISL_489449, EPI_ISL_489455, EPI_ISL_489470, EPI_ISL_489480, EPI_ISL_489489, EPI_ISL_489536 to 489537, EPI_ISL_489543, EPI_ISL_489547, EPI_ISL_489578 to 489580, EPI_ISL_489584 to 489588, EPI_ISL_489590 to 489591, EPI_ISL_489593 to 489604, EPI_ISL_489606, EPI_ISL_489608, EPI_ISL_489612 to 489613, EPI_ISL_489615 to 489617, EPI_ISL_489619 to 489623, EPI_ISL_489625 to 489628, EPI_ISL_489631 to 489633, EPI_ISL_489635, EPI_ISL_489637 to 489643, EPI_ISL_489647 to 489651, EPI_ISL_489654, EPI_ISL_489656 to 489658, EPI_ISL_489660 to 489661, EPI_ISL_489663 to 489664, EPI_ISL_489666 to 489670, EPI_ISL_489672, EPI_ISL_489674, EPI_ISL_489676, EPI_ISL_489678 to 489680, EPI_ISL_489682 to 489685, EPI_ISL_489689 to 489690, EPI_ISL_489692 to 489694, EPI_ISL_489697 to 489699, EPI_ISL_489701, EPI_ISL_489703 to 489706, EPI_ISL_501633 to 501635, EPI_ISL_524494, EPI_ISL_524500, EPI_ISL_524513 to 524514, EPI_ISL_524516, EPI_ISL_524518 to 524520, EPI_ISL_524526 to 524527, EPI_ISL_524534 to 524536, EPI_ISL_524539, EPI_ISL_524545, EPI_ISL_524550, EPI_ISL_524552, EPI_ISL_524556, EPI_ISL_524562 to 524563, EPI_ISL_524566, EPI_ISL_524572, EPI_ISL_530361, EPI_ISL_530366, EPI_ISL_530386, EPI_ISL_530410, EPI_ISL_530429, EPI_ISL_530468, EPI_ISL_530480, EPI_ISL_530488 to 530489, EPI_ISL_530523, EPI_ISL_530528, EPI_ISL_530533, EPI_ISL_530542, EPI_ISL_530553, EPI_ISL_530562, EPI_ISL_530596, EPI_ISL_530659, EPI_ISL_530673, EPI_ISL_530682, EPI_ISL_530708, EPI_ISL_530722, EPI_ISL_530743, EPI_ISL_530754, EPI_ISL_530764, EPI_ISL_530766 to 530768, EPI_ISL_530826, EPI_ISL_530836, EPI_ISL_530845, EPI_ISL_530862, EPI_ISL_530871, EPI_ISL_530906, EPI_ISL_530923, EPI_ISL_530929, EPI_ISL_530953, EPI_ISL_530959, EPI_ISL_530961 to 530962, EPI_ISL_530973, EPI_ISL_530976, EPI_ISL_530979, EPI_ISL_530983, EPI_ISL_530985 to 530986, EPI_ISL_530997, EPI_ISL_531002, EPI_ISL_531004, EPI_ISL_531006, EPI_ISL_531017, EPI_ISL_531020, EPI_ISL_531032 to 531033, EPI_ISL_531038, EPI_ISL_531040, EPI_ISL_531045, EPI_ISL_531048 to 531049, EPI_ISL_531052, EPI_ISL_531054, EPI_ISL_531057, EPI_ISL_531065, EPI_ISL_531079, EPI_ISL_531087, EPI_ISL_531091, EPI_ISL_531096, EPI_ISL_531099 to 531100, EPI_ISL_531103 to 531104, EPI_ISL_531116, EPI_ISL_531118, EPI_ISL_531123, EPI_ISL_531126, EPI_ISL_531130, EPI_ISL_531142, EPI_ISL_531147, EPI_ISL_531154 to 531157, EPI_ISL_531166, EPI_ISL_531174 to 531175, EPI_ISL_531189, EPI_ISL_531198, EPI_ISL_531207, EPI_ISL_531212, EPI_ISL_531214, EPI_ISL_531223, EPI_ISL_531249, EPI_ISL_531266, EPI_ISL_531321, EPI_ISL_531330, EPI_ISL_531365, EPI_ISL_531374, EPI_ISL_531382, EPI_ISL_531412, EPI_ISL_531416, EPI_ISL_531420, EPI_ISL_531422, EPI_ISL_531432, EPI_ISL_531442, EPI_ISL_531464, EPI_ISL_531470, EPI_ISL_531473, EPI_ISL_531480, EPI_ISL_531483 to 531485, EPI_ISL_531492, EPI_ISL_531495, EPI_ISL_531500, EPI_ISL_531504, EPI_ISL_531524, EPI_ISL_531546, EPI_ISL_531548, EPI_ISL_531557, EPI_ISL_531563 to 531565, EPI_ISL_531569 to 531570, EPI_ISL_531575, EPI_ISL_531577, EPI_ISL_531583, EPI_ISL_531590, EPI_ISL_531595, EPI_ISL_531598 to 531599, EPI_ISL_531607, EPI_ISL_531617, EPI_ISL_531625, EPI_ISL_531631, EPI_ISL_531634, EPI_ISL_531638 to 531639, EPI_ISL_531649, EPI_ISL_531660, EPI_ISL_531668, EPI_ISL_531672, EPI_ISL_531677 to 531678, EPI_ISL_531689 to 531691, EPI_ISL_531702 to 531703, EPI_ISL_531714, EPI_ISL_531730, EPI_ISL_531732, EPI_ISL_531743, EPI_ISL_531754 to 531755, EPI_ISL_531768, EPI_ISL_531801 to 531802, EPI_ISL_531805, EPI_ISL_531814 to 531815, EPI_ISL_531821, EPI_ISL_531824, EPI_ISL_531832, EPI_ISL_531842, EPI_ISL_531856 to 531858, EPI_ISL_531862 to 531863, EPI_ISL_531865, EPI_ISL_531873, EPI_ISL_531877, EPI_ISL_531880 to 531881, EPI_ISL_531884, EPI_ISL_531887, EPI_ISL_531889 to 531890, EPI_ISL_531892, EPI_ISL_531894, EPI_ISL_531905, EPI_ISL_531912 to 531913, EPI_ISL_531918 to 531919, EPI_ISL_531925, EPI_ISL_531929, EPI_ISL_531932 to 531933, EPI_ISL_531935 to 531936, EPI_ISL_531943, EPI_ISL_531945, EPI_ISL_531951, EPI_ISL_531954, EPI_ISL_531963, EPI_ISL_531973, EPI_ISL_531978 to 531979, EPI_ISL_531982, EPI_ISL_531984, EPI_ISL_531990, EPI_ISL_531995, EPI_ISL_532003 to 532005, EPI_ISL_532018, EPI_ISL_532024, EPI_ISL_532038, EPI_ISL_532049, EPI_ISL_532053, EPI_ISL_532055, EPI_ISL_532057, EPI_ISL_532062, EPI_ISL_532070, EPI_ISL_532076 to 532078, EPI_ISL_532080 to 532081, EPI_ISL_532093 to 532094, EPI_ISL_532098, EPI_ISL_532102, EPI_ISL_532105, EPI_ISL_532118, EPI_ISL_532123, EPI_ISL_532125, EPI_ISL_532129 to 532131, EPI_ISL_532138, EPI_ISL_532178, EPI_ISL_532192, EPI_ISL_532195 to 532196, EPI_ISL_532200, EPI_ISL_532202 to 532203, EPI_ISL_532210, EPI_ISL_532214, EPI_ISL_532241, EPI_ISL_532258, EPI_ISL_532263, EPI_ISL_532271 to 532272, EPI_ISL_532275 to 532276, EPI_ISL_532278, EPI_ISL_532283, EPI_ISL_532288, EPI_ISL_532293, EPI_ISL_532301, EPI_ISL_532311, EPI_ISL_532316 to 532317, EPI_ISL_532321, EPI_ISL_532328, EPI_ISL_532334 to 532335, EPI_ISL_532338, EPI_ISL_532340, EPI_ISL_532347, EPI_ISL_532349, EPI_ISL_532351, EPI_ISL_532353, EPI_ISL_532355 to 532356, EPI_ISL_532363 to 532364, EPI_ISL_532384, EPI_ISL_532388, EPI_ISL_532390, EPI_ISL_532394, EPI_ISL_532399, EPI_ISL_532402, EPI_ISL_532404, EPI_ISL_532414, EPI_ISL_532430, EPI_ISL_532432, EPI_ISL_532440, EPI_ISL_532444, EPI_ISL_532446, EPI_ISL_532452, EPI_ISL_532454, EPI_ISL_532456, EPI_ISL_532463 to 532464, EPI_ISL_532472 to 532473, EPI_ISL_532480, EPI_ISL_532484, EPI_ISL_532492 to 532493, EPI_ISL_532506, EPI_ISL_532510, EPI_ISL_532512, EPI_ISL_532514 to 532515, EPI_ISL_532518, EPI_ISL_532523, EPI_ISL_532531, EPI_ISL_532538, EPI_ISL_532540, EPI_ISL_532563 to 532564, EPI_ISL_532572, EPI_ISL_532582, EPI_ISL_532587, EPI_ISL_532594, EPI_ISL_532598, EPI_ISL_532620 to 532621, EPI_ISL_532625 to 532626, EPI_ISL_532648, EPI_ISL_532670, EPI_ISL_532697, EPI_ISL_532715, EPI_ISL_532733, EPI_ISL_532738, EPI_ISL_532744, EPI_ISL_532746, EPI_ISL_532750, EPI_ISL_532753, EPI_ISL_532755, EPI_ISL_532767, EPI_ISL_532784, EPI_ISL_532786, EPI_ISL_532791, EPI_ISL_532811, EPI_ISL_532838, EPI_ISL_532865, EPI_ISL_532874, EPI_ISL_532876, EPI_ISL_532878, EPI_ISL_532885, EPI_ISL_532891, EPI_ISL_532894, EPI_ISL_532896, EPI_ISL_532905 to 532906, EPI_ISL_532915, EPI_ISL_532932 to 532933, EPI_ISL_532962, EPI_ISL_532988, EPI_ISL_533016, EPI_ISL_533027, EPI_ISL_533072, EPI_ISL_533228, EPI_ISL_533231 to 533232, EPI_ISL_533236 to 533237, EPI_ISL_533241, EPI_ISL_533252, EPI_ISL_533256, EPI_ISL_533262, EPI_ISL_533271, EPI_ISL_533283, EPI_ISL_533311, EPI_ISL_533343, EPI_ISL_533346, EPI_ISL_533350 to 533351, EPI_ISL_533376, EPI_ISL_533394, EPI_ISL_533406, EPI_ISL_533419, EPI_ISL_533421, EPI_ISL_534365 to 534369, EPI_ISL_534371 to 534378, EPI_ISL_534380, EPI_ISL_534382 to 534387, EPI_ISL_534389 to 534394, EPI_ISL_534396 to 534399, EPI_ISL_534401 to 534402, EPI_ISL_534404 to 534415, EPI_ISL_534418 to 534431, EPI_ISL_534433 to 534447, EPI_ISL_534449, EPI_ISL_534452 to 534453, EPI_ISL_534455 to 534459, EPI_ISL_534461 to 534463, EPI_ISL_534465, EPI_ISL_534467 to 534474, EPI_ISL_534476 to 534478, EPI_ISL_534480 to 534484, EPI_ISL_534486 to 534491, EPI_ISL_534493, EPI_ISL_534495 to 534503, EPI_ISL_534505 to 534509, EPI_ISL_534511, EPI_ISL_534515 to 534517, EPI_ISL_534520 to 534522, EPI_ISL_534524, EPI_ISL_534526 to 534531, EPI_ISL_534534, EPI_ISL_534536 to 534539, EPI_ISL_534541 to 534542, EPI_ISL_534544 to 534546, EPI_ISL_534548 to 534549, EPI_ISL_534551 to 534560, EPI_ISL_534562 to 534565, EPI_ISL_534567 to 534568, EPI_ISL_534570, EPI_ISL_534572 to 534599, EPI_ISL_534601 to 534603, EPI_ISL_534605 to 534611, EPI_ISL_534613 to 534617, EPI_ISL_534619 to 534621, EPI_ISL_534623 to 534625, EPI_ISL_534627 to 534633, EPI_ISL_534636 to 534643, EPI_ISL_534645 to 534648, EPI_ISL_534650 to 534653, EPI_ISL_534656 to 534659, EPI_ISL_534661 to 534681, EPI_ISL_534683 to 534688, EPI_ISL_534690			
see above	NHSGGC West of Scotland Specialist Virology Centre /	Wellcome Sanger Institute for the COVID-19 Genomics UK	Alasdair MacLean; Alice Broos; Ana da Silva Filipe; Antonia Ho; Cordelia Langford; Daniel Mair; David K. Jackson; David L Robertson; Dominic

	MRC-University of Glasgow Centre for Virus Research	(COG-UK) consortium	Kwiatkowski; Elihu Aranday-Cortes; Emma Thomson and Alex Alderton; Ewan Harrison; Ian Johnston; James Shepherd; Jenna Nichols; John Sillitoe; John Sillitoe on behalf of the Wellcome Sanger Institute COVID-19 Surveillance Team (http://www.sanger.ac.uk/covid-team); Joseph Hughes; Kathy Li; Kathy Smollett; Kirstyn Brunker; Kyriaki Nomikou; Lily Tong; Marc Niebel; Natasha Jesudason; Natasha Johnson; Patawee Asamaphan; Rajiv Shah; Richard Orton; Roberto Amato; Rory Gunson; Sarah McDonald; Sonia Goncalves; Sreenu Vattipally; Stephen Carmichael; Yasmin Parr
EPI_ISL_418241 to 418242, EPI_ISL_420037, EPI_ISL_766861 to 766869, EPI_ISL_766871 to 766875	NIC Viral Respiratory Unit - Institut Pasteur of Algeria	National Reference Center for Viruses of Respiratory Infections, Institut Pasteur, Paris	Angela Brisebarre; Etienne Simon-Lorière; Fawzi Derrar; Flora Donati; Marion Barbet; Maud Vanpeene; Mélanie Albert; Méline Bizard; Sylvie Behillil; Sylvie van der Werf; Vincent Enouf
EPI_ISL_452192 to 452217, EPI_ISL_454521 to 454522, EPI_ISL_454524 to 454534, EPI_ISL_454536 to 454537, EPI_ISL_454540, EPI_ISL_454542 to 454544, EPI_ISL_454546 to 454547, EPI_ISL_454549, EPI_ISL_454551 to 454552, EPI_ISL_454556 to 454558, EPI_ISL_454560 to 454570, EPI_ISL_479493 to 479524, EPI_ISL_479526 to 479548, EPI_ISL_479550 to 479560, EPI_ISL_479562 to 479568, EPI_ISL_479570 to 479572, EPI_ISL_479657, EPI_ISL_577640, EPI_ISL_577643 to 577645, EPI_ISL_577647 to 577649, EPI_ISL_577651 to 577652, EPI_ISL_577655, EPI_ISL_577658 to 577665, EPI_ISL_577670, EPI_ISL_577672, EPI_ISL_577676 to 577686, EPI_ISL_577688 to 577693, EPI_ISL_577695, EPI_ISL_577698 to 577702, EPI_ISL_577704 to 577705, EPI_ISL_577707, EPI_ISL_577709 to 577715, EPI_ISL_577717 to 577720, EPI_ISL_577722 to 577723, EPI_ISL_577725 to 577733, EPI_ISL_582241, EPI_ISL_1034155, EPI_ISL_1034157 to 1034159, EPI_ISL_1034162 to 1034163, EPI_ISL_1034166 to 1034168, EPI_ISL_1034171, EPI_ISL_1034173, EPI_ISL_1034175, EPI_ISL_1034177, EPI_ISL_1034180 to 1034181, EPI_ISL_1034184 to 1034186, EPI_ISL_1034188 to 1034189			
see above	NIV Influenza	NIV Influenza	Potdar V
EPI_ISL_450321 to 450325	NIV Pune	CSIR-Centre for Cellular and Molecular Biology	A. Awhale; A. Gondhalikar; A. Jagtap; Archana Bharadwaj Siva; B. Minhas S. Bharadwaj; Deepak Kumar; Devi Prasad Vijayashankara; Dhiviya Vedagiri; Disha Nanda; Divya Das; Divya Gupta; Dr ML Choudhary; Dr Priya Abraham; Dr V A Potdar; G. Aditya Kumar; Gangumala Srinivas Reddy; H Kaushal Y. Gurav; H. Kengle; Jotin Gogoi; K. Patel; Karthik Bharadwaj Tallapaka; Koushick Sivakumar; Krishnan Harinivas Harshan; Lamuk Zaveri; M. Kakade; Manish; Manish Bhattacharjee; N Srivastava; Namami Gaur; P Shinde; P. Malsane; Payel Mukherjee; Pooja Ramesh Gupta; Priya Singh; Purushotham Vodnala; R. Gunjikar; Rajan Kumar Jha; Rakesh K Mishra; Ravi Prasad Mukku; Renu Sudhakar; S. Bhorekar; S. Digraskar; S. Hundekar; S. Jadhav; S. Salve; S. Tomar; Sakshi Shambhavi; Santosh Kumar Kuncha; Shagufta Khan; Shraddha Vijay Lahoti; Sofia Banu; Somesh Gorde; Sujoy Deb; Swati Bayyana; Tulasi Nagabandi; U. Saha; V Malik; V. Awtade; V. Vipat; Vishal Sah; Yogesh Balakartik; Zeba Rizvi
EPI_ISL_751659, EPI_ISL_751662, EPI_ISL_751680, EPI_ISL_751709, EPI_ISL_751773	NJ Public Health and Environmental Laboratories	Genomics and Discovery, Respiratory Viruses Branch, Division of Viral Diseases, Centers for Disease Control and Prevention	Anna Montmayeur; Anna Uehara; Clinton R. Paden; Haibin Wang; Jing Zhang; Justin Lee; Krista Queen; Mili Sheth; Peter W. Cook; Rachel Marine; Suxiang Tong; Yan Li; Ying Tao
EPI_ISL_424878, EPI_ISL_424901, EPI_ISL_452116 to 452117	NJ Public Health and Environmental Laboratories	Pathogen Discovery, Respiratory Viruses Branch, Division of Viral Diseases, Centers for Disease Control and Prevention	Alison S. Laufer Halpin; Anna Montmayeur; Anna Uehara; Christopher A. Elkins; Clinton R. Paden; Haibin Wang; Jing Zhang; Krista Queen; Mary S. Keckler; Rachel Marine; Suxiang Tong; Yan Li; Ying Tao; Zachary Weiner
EPI_ISL_452109, EPI_ISL_594462	NM Department of Health	Pathogen Discovery, Respiratory Viruses Branch, Division of Viral Diseases, Centers for Disease Control and Prevention	Alison S. Laufer Halpin; Anna Montmayeur; Anna Uehara; Christopher A. Elkins; Clinton Paden; Clinton R. Paden; Haibin Wang; Jing Zhang; Julu Bhatnagar; Krista Queen; Mary S. Keckler; Rachel Marine; Suxiang Tong; Yan Li; Ying Tao; Zachary Weiner
EPI_ISL_830572, EPI_ISL_883427 to 883428	NORTHWELL HEALTH LABORATORIES	Wadsworth Center, New York State Department of Health	Alexis Russel; Daryl M. Lamson; Erasmus Schneider; Erica Lasek-Nesselquist; John Kelly; Jonathan Plitnick; Kirsten St. George; Matthew Shudt; Melissa A Leisner; Navjot Singh
EPI_ISL_416742 to 416743	NRL for Influenza, Centrum Epidemiology and Microbiology of National Institute of Public Health, Czech Republic	Charite Universitaetsmedizin Berlin, Institute of Virology	Akexander Nagy; Barbara Muehlemann; Christian Drosten; Dusan Trnka; Helena Jirincova; Jaromira Vecerova; Jorn Beheim-Schwarzbach; Julia Schneider; Ludmila Novakova; Talitha Veith; Terry Jones; Victor M Corman
EPI_ISL_514440, EPI_ISL_514580, EPI_ISL_514613, EPI_ISL_514615	NSTU COVID-19 Diagnostic Center	NSU Genome Research Institute (NGRI), North South University	Abdus Sadique; Aura Rahman; Dr. Muhammad Maqsud Hossain; Hasan Mahmud Reza; Jahidul Alam; Md Aminul Islam; Prof; Prof. Abdul Khaleque; Prof. Firoz Ahmed; Prof. Kazi Nadim Hasan; Prof. Md. Didar-Ul-Alam; Tahrima Huq; Tamanna Afroze
EPI_ISL_458579 to 458581, EPI_ISL_458583 to 458584, EPI_ISL_458588 to 458589, EPI_ISL_458591, EPI_ISL_458593, EPI_ISL_458597, EPI_ISL_458600 to 458607, EPI_ISL_458609 to 458626, EPI_ISL_458628, EPI_ISL_458631 to 458633, EPI_ISL_458635 to 458638, EPI_ISL_458640 to 458654, EPI_ISL_458656 to 458657, EPI_ISL_458660 to 458662, EPI_ISL_458664, EPI_ISL_458667 to 458672, EPI_ISL_458674 to 458679, EPI_ISL_458681, EPI_ISL_458684, EPI_ISL_458686 to 458691, EPI_ISL_458693 to 458694, EPI_ISL_458696 to 458697, EPI_ISL_458699 to 458703, EPI_ISL_458706 to 458711, EPI_ISL_458713 to 458715, EPI_ISL_458717 to 458718, EPI_ISL_469302 to 469303, EPI_ISL_469307, EPI_ISL_469309, EPI_ISL_469312 to 469316, EPI_ISL_469319, EPI_ISL_469324 to 469325, EPI_ISL_469328 to 469330, EPI_ISL_469335 to 469336, EPI_ISL_469340, EPI_ISL_469344, EPI_ISL_469915, EPI_ISL_488003 to 488005, EPI_ISL_488007 to 488009, EPI_ISL_488011 to 488014, EPI_ISL_488018, EPI_ISL_488020 to 488023, EPI_ISL_488025 to 488026, EPI_ISL_488028, EPI_ISL_488030, EPI_ISL_488036, EPI_ISL_488038 to 488040, EPI_ISL_488042, EPI_ISL_488044, EPI_ISL_488046 to 488049, EPI_ISL_488051, EPI_ISL_488053, EPI_ISL_488055 to 488058, EPI_ISL_488060 to 488061, EPI_ISL_488063 to 488071, EPI_ISL_488075 to 488078, EPI_ISL_488080 to 488081, EPI_ISL_488084 to 488085, EPI_ISL_488087 to 488093, EPI_ISL_488095 to 488096, EPI_ISL_488099, EPI_ISL_488101 to 488108, EPI_ISL_488110 to 488111, EPI_ISL_488113 to 488120, EPI_ISL_488123 to 488124, EPI_ISL_488126 to 488134, EPI_ISL_488136 to 488139, EPI_ISL_488141 to 488142, EPI_ISL_488144 to 488147, EPI_ISL_488150 to 488154, EPI_ISL_488156 to 488164, EPI_ISL_488166, EPI_ISL_488168, EPI_ISL_488171 to 488172, EPI_ISL_488174, EPI_ISL_488176, EPI_ISL_488179 to 488184, EPI_ISL_488186, EPI_ISL_488456 to 488459, EPI_ISL_488461 to 488491, EPI_ISL_488494 to 488498, EPI_ISL_488501 to 488541, EPI_ISL_488543 to 488545, EPI_ISL_488547 to 488552, EPI_ISL_488554 to 488561, EPI_ISL_488563 to 488596, EPI_ISL_488598 to 488622, EPI_ISL_488624 to 488652, EPI_ISL_488654 to 488769, EPI_ISL_488771 to 488780, EPI_ISL_488782 to 488832, EPI_ISL_488834 to 488836, EPI_ISL_489065, EPI_ISL_489067 to 489068, EPI_ISL_489070, EPI_ISL_489072 to 489089, EPI_ISL_489091 to 489094, EPI_ISL_489097, EPI_ISL_489099 to 489110, EPI_ISL_489113 to 489114, EPI_ISL_489117, EPI_ISL_489119, EPI_ISL_489121 to 489129, EPI_ISL_489131 to 489133, EPI_ISL_489135, EPI_ISL_489137 to 489144, EPI_ISL_489146, EPI_ISL_489149 to 489151, EPI_ISL_489153 to 489155, EPI_ISL_492207 to 492209, EPI_ISL_492450 to 492456, EPI_ISL_492458 to 492466, EPI_ISL_492469 to 492485, EPI_ISL_492487 to 492493, EPI_ISL_492916, EPI_ISL_492918 to 492920, EPI_ISL_492922 to 492928, EPI_ISL_492930, EPI_ISL_492932, EPI_ISL_492934 to 492939, EPI_ISL_492942 to 492949, EPI_ISL_492951 to 492956, EPI_ISL_492958 to 492959, EPI_ISL_492962 to 492966, EPI_ISL_492968 to 492970, EPI_ISL_492973 to 492977			
see above	NU-OMICS DNA Sequencing research facility, Northumbria University	Wellcome Sanger Institute for the COVID-19 Genomics UK (COG-UK) consortium	Andrew Nelson; Brendan Payne; Chris Duncan; Clive Graham; Cordelia Langford; Darren Smith and Alex Alderton; David K. Jackson; Debra Padgett; Dominic Kwiatkowski; Edward Barton; Emma Swindells; Ewan Harrison; Garren Scott; Gary Black; Gary Eltringham; Greg Young; Ian Johnston; Jane Greenaway; Jennifer Collins; John Allan; John Sillitoe on behalf of the Wellcome Sanger Institute COVID-19 Surveillance Team (http://www.sanger.ac.uk/covid-team); Joshua Loh; Lynn Dover; Matthew Bashton; Paul Baker; Roberto Amato; Sarah Essex; Sheia Waugh; Shirelle Burton-Fanning; Sonia Goncalves; Steve Ligget; Wen Yew; Yusri Taha
EPI_ISL_632313	NU-sjukvården	Clinical Microbiology, Sahlgrenska University Hospital,	Hedvig Engström Jakobsson; Johan Ringlander; Josefin Olausson; Magnus Lindh
EPI_ISL_632312	NU-sjukvården	Clinical microbiology, Sahlgrenska University Hospital	Hedvig Engström Jakobsson; Johan Ringlander; Josefin Olausson; Magnus Lindh
EPI_ISL_751556 to 751557, EPI_ISL_751636 to 751637, EPI_ISL_751639, EPI_ISL_751708, EPI_ISL_751714, EPI_ISL_751736, EPI_ISL_751778			
see above	NV State Public Health Laboratory	Genomics and Discovery, Respiratory Viruses Branch, Division of Viral Diseases, Centers for Disease Control and Prevention	Anna Montmayeur; Anna Uehara; Clinton R. Paden; Haibin Wang; Jing Zhang; Justin Lee; Krista Queen; Mili Sheth; Peter W. Cook; Rachel Marine; Suxiang Tong; Yan Li; Ying Tao
EPI_ISL_426429	NV State Public Health Laboratory	Pathogen Discovery, Respiratory Viruses Branch, Division of Viral Diseases, Centers for Disease Control and Prevention	Alison S. Laufer Halpin; Anna Uehara; Christopher A. Elkins; Clinton R. Paden; Haibin Wang; Jing Zhang; Krista Queen; Mary S. Keckler; Rachel Marine; Suxiang Tong; Yan Li; Ying Tao
EPI_ISL_424879	NV-Southern Nevada Public Health Laboratory	Pathogen Discovery, Respiratory Viruses Branch, Division of Viral Diseases, Centers for Disease Control and Prevention	Alison S. Laufer Halpin; Anna Uehara; Christopher A. Elkins; Clinton R. Paden; Haibin Wang; Jing Zhang; Krista Queen; Mary S. Keckler; Rachel Marine; Suxiang Tong; Yan Li; Ying Tao
EPI_ISL_631541, EPI_ISL_631572, EPI_ISL_631862, EPI_ISL_632039, EPI_ISL_632062, EPI_ISL_632074, EPI_ISL_632963			
see above	NYC Department Of Health And Mental Hygiene	New York City Public Health Laboratory	Jade Wang; et al.
EPI_ISL_420793, EPI_ISL_424906, EPI_ISL_436047 to 436082, EPI_ISL_480784 to 480785	NYC Department of Health and Mental Hygiene	Pathogen Discovery, Respiratory Viruses Branch, Division of Viral Diseases, Centers for Disease Control and Prevention	Alison S. Laufer Halpin; Anna Uehara; Anne Uehara; Bettina Bankamp; Christine Mahl; Christopher A. Elkins; Christy Harrison; Clinton R. Paden; Haibin Wang; Jasmine Padilla; Jennifer Rakeman; Jing Zhang; Justin Lee; Krista Queen; Mary S. Keckler; Rachel Marine; Sally Slavinski; Suxiang Tong; Yan Li; Ying Tao; Zachary Weiner
EPI_ISL_631974	NYC HH Coney Island Hospital	New York City Public Health Laboratory	Jade Wang; et al.
EPI_ISL_631564	NYC HH Elmhurst Hospital Medical Center	New York City Public Health Laboratory	Jade Wang; et al.
EPI_ISL_631556	NYC HH Jacobi Medical Center	New York City Public Health Laboratory	Jade Wang; et al.
EPI_ISL_631520, EPI_ISL_631557, EPI_ISL_631577, EPI_ISL_631584, EPI_ISL_631587, EPI_ISL_631603, EPI_ISL_631607, EPI_ISL_631686, EPI_ISL_631693, EPI_ISL_631752 to 631754, EPI_ISL_631827, EPI_ISL_631953, EPI_ISL_632026, EPI_ISL_632072, EPI_ISL_632116, EPI_ISL_632131, EPI_ISL_632146 to 632147, EPI_ISL_632177, EPI_ISL_632186 to 632187, EPI_ISL_632192 to 632193, EPI_ISL_632235			
see above	NYC HH Lincoln Medical And Mental Health Center	New York City Public Health Laboratory	Jade Wang; et al.
EPI_ISL_632060	NYC HH Queens Hospital Center	New York City Public Health Laboratory	Jade Wang; et al.
EPI_ISL_594457	NYS Department of Health	Pathogen Discovery, Respiratory Viruses Branch, Division of Viral Diseases, Centers for Disease Control and Prevention	Anna Uehara; Clinton Paden; Haibin Wang; Jing Zhang; Julu Bhatnagar; Krista Queen; Suxiang Tong; Yan Li; Ying Tao
EPI_ISL_751560, EPI_ISL_751580 to 751581, EPI_ISL_751658, EPI_ISL_751682,	NYSDOH Wadsworth Center, Virology Lab	Genomics and Discovery, Respiratory Viruses Branch, Division of Viral Diseases, Centers for Disease Control and	Anna Montmayeur; Anna Uehara; Clinton R. Paden; Haibin Wang; Jing Zhang; Justin Lee; Krista Queen; Mili Sheth; Peter W. Cook; Rachel Marine; Suxiang Tong; Yan Li; Ying Tao

EPI_ISL_751717		Prevention	
EPI_ISL_416830 to 416832, EPI_ISL_418192 to 418193, EPI_ISL_418196 to 418200, EPI_ISL_418203 to 418204, EPI_ISL_418972 to 418980	NYU Langone Health	Department of Pathology and Medicine, New York University School of Medicine	Adriana Heguy; Amy Rapkiewicz; Antonio Serrano; Christian Marier; Dacia Dimartino; Emily Huang; Gael Westby; George Jour; Guomiao Shen; Jared Pinnell; John Cadley; John Chen; Margaret Black; Maria Aguero-Rosenfeld; Marie Samanovic-Golden; Mark J. Mulligan; Mark Mulligan; Matija Snuderl; Matt Maurano; Matthew T. Maurano; Megan Hogan; Nick Vulpescu; Paolo Cotzia; Paul Zappile; Peter Meyn; Sitharam Ramaswami; Xiaojun Feng; Yutong Zhang
EPI_ISL_419696 to 419700, EPI_ISL_419702 to 419705, EPI_ISL_420296 to 420302, EPI_ISL_420307 to 420309, EPI_ISL_420570 to 420573, EPI_ISL_420575 to 420582, EPI_ISL_420585 to 420591, EPI_ISL_421577 to 421592, EPI_ISL_421706 to 421707, EPI_ISL_421709 to 421712, EPI_ISL_421714 to 421715, EPI_ISL_421717 to 421733, EPI_ISL_424929 to 424968, EPI_ISL_426617 to 426626, EPI_ISL_427469 to 427482, EPI_ISL_427484 to 427485, EPI_ISL_427487 to 427496, EPI_ISL_427498, EPI_ISL_427502 to 427505, EPI_ISL_427507 to 427516, EPI_ISL_427518 to 427521, EPI_ISL_427524, EPI_ISL_427627 to 427641, EPI_ISL_428757 to 428770, EPI_ISL_428772 to 428793, EPI_ISL_428795, EPI_ISL_428797 to 428799, EPI_ISL_428801, EPI_ISL_428804 to 428805, EPI_ISL_430323 to 430434, EPI_ISL_435475 to 435549, EPI_ISL_444613, EPI_ISL_444615 to 444648, EPI_ISL_444650 to 444671, EPI_ISL_444673, EPI_ISL_444675, EPI_ISL_444677 to 444688, EPI_ISL_444690 to 444708, EPI_ISL_444710 to 444777, EPI_ISL_444780 to 444782, EPI_ISL_444784, EPI_ISL_444786 to 444791, EPI_ISL_450393 to 450394, EPI_ISL_450396 to 450402, EPI_ISL_451401 to 451402, EPI_ISL_451404 to 451424, EPI_ISL_451426 to 451432, EPI_ISL_451434 to 451472, EPI_ISL_451474 to 451479, EPI_ISL_451665, EPI_ISL_456004, EPI_ISL_456006 to 456008, EPI_ISL_456010 to 456047, EPI_ISL_456049 to 456056, EPI_ISL_456058, EPI_ISL_456060 to 456063, EPI_ISL_456065 to 456066, EPI_ISL_456107 to 456109, EPI_ISL_456111 to 456113, EPI_ISL_458017 to 458021, EPI_ISL_467377 to 467381, EPI_ISL_467384 to 467385, EPI_ISL_467387, EPI_ISL_467391, EPI_ISL_467394 to 467396, EPI_ISL_467398 to 467400, EPI_ISL_467405, EPI_ISL_467409, EPI_ISL_467411 to 467414, EPI_ISL_467416 to 467418, EPI_ISL_467420 to 467422, EPI_ISL_1016013, EPI_ISL_1016017			
see above	NYU Langone Health	Departments of Pathology and Medicine, New York University School of Medicine	Adriana Heguy; Alireza Khodadadi-Jamayran; Amy Rapkiewicz; Andre M. Ribeiro-dos-Santos; Andrew Lytle; Antonio Serrano; Brendan Belovarac; Christian Marier; Dacia Dimartino; Emily Guzman; Emily Huang; Gael Westby; George Jour; Guiqing Wang; Guomiao Shen; Iman Osman; Jared Pinnell; John Cadley; John Chen; Lawrence H. Lin; Ludovic Boytard; Margaret Black; Maria Aguero-Rosenfeld; Marie Samanovic-Golden; Mark J. Mulligan; Matija Snuderl; Matthew T. Maurano; Megan Hogan; Nick Vulpescu; Paolo Cotzia; Paul Zappile; Peter Meyn; Raquel Ordonez Ciriza; Raven Luther; Sitharam Ramaswami; Tatyana Gindin; Theodore Vougiouklakis; Vanessa Raabe; Xiaojun Feng; Yutong Zhang
EPI_ISL_631515, EPI_ISL_631523, EPI_ISL_631526 to 631527, EPI_ISL_631531, EPI_ISL_631558 to 631559, EPI_ISL_632043 to 632044, EPI_ISL_632067, EPI_ISL_632076, EPI_ISL_632079			
see above	NYU Langone Health	New York City Public Health Laboratory	Jade Wang; et al.
EPI_ISL_625559 to 625576, EPI_ISL_625578 to 625592	NaN	Chan-Zuckerberg Biohub	CZB Cliahub Consortium
EPI_ISL_745188	Nababeep Hospital	National Health Laboratory Service (NHLS), Tygerberg	Bronwyn Kleinhans; Eduan Wilkindon; Gert van Zyl; Houriiyah Tegally; Kayla Delaney; Susan Engelbrecht; Tulio de Oliveira; Wolfgang Preiser
EPI_ISL_479997 to 480001	Nagano Environmental Conservation Research Institute	Pathogen Genomics Center, National Institute of Infectious Diseases	Hajime Kamiya; Kentaro Itokawa; Makoto Kuroda; Masanori Hashino; Motoi Suzuki; Naoko Shimodaira; Rina Tanaka; Tsuyoshi Sekizuka
EPI_ISL_480003	Nagasaki Prefectural Institute for Environmental Research and Public Health	Pathogen Genomics Center, National Institute of Infectious Diseases	Fumiaki Matsumoto; Hajime Kamiya; Kentaro Itokawa; Makoto Kuroda; Masanori Hashino; Motoi Suzuki; Rina Tanaka; Tsuyoshi Sekizuka
EPI_ISL_480042 to 480064	Nagoya City Public Health Research Institute	Pathogen Genomics Center, National Institute of Infectious Diseases	Hajime Kamiya; Kentaro Itokawa; Makoto Kuroda; Masanori Hashino; Motoi Suzuki; Rina Tanaka; Shinichiro Shibata; Takuya Miki; Tsuyoshi Sekizuka
EPI_ISL_738754, EPI_ISL_738777, EPI_ISL_738820, EPI_ISL_738836, EPI_ISL_738870, EPI_ISL_738897, EPI_ISL_738911, EPI_ISL_738952, EPI_ISL_738959, EPI_ISL_739169, EPI_ISL_739181, EPI_ISL_739223, EPI_ISL_739251, EPI_ISL_739301, EPI_ISL_739315, EPI_ISL_739325, EPI_ISL_739339, EPI_ISL_739469, EPI_ISL_739471, EPI_ISL_739474, EPI_ISL_739514, EPI_ISL_739557, EPI_ISL_739628, EPI_ISL_739635, EPI_ISL_739645			
see above	Napa-Solano-Yolo- Marin County (NSYM) Public Health Laboratories	Chan-Zuckerberg Biohub	CZB Cliahub Consortium
EPI_ISL_434660, EPI_ISL_434674, EPI_ISL_445229, EPI_ISL_445236, EPI_ISL_450817, EPI_ISL_450823, EPI_ISL_450825, EPI_ISL_452229 to 452230, EPI_ISL_452235, EPI_ISL_452239			
see above	Narhalsan Backa vardcentral	The Public Health Agency of Sweden	Anna Risberg; Anna-Malin Linde; Karin Tegmark-Wisell; Maria Lind Karlberg; Mats Olsson; Mia Brytting; Olov Svartstrom; Oskar Karlsson Lindsjo; Theresa Enkirch
EPI_ISL_539885, EPI_ISL_582840, EPI_ISL_710589 to 710591, EPI_ISL_732655 to 732656	Narhalsan Fjallbacka VC	The Public Health Agency of Sweden	Anna Risberg; Anna-Malin Linde; Department of Microbiology; Karin Tegmark-Wisell; Maria Lind Karlberg; Mattias Haukland; Mia Brytting; Olov Svartstrom; Oskar Karlsson Lindsjo; Petra Edquist; Reza Advani; Sandra Broddesson; The Public Health Agency of Sweden; Theresa Enkirch
EPI_ISL_455107	Narhalsan Mellerud	The Public Health Agency of Sweden	Anna Risberg; Anna-Malin Linde; Karin Tegmark-Wisell; Maria Lind Karlberg; Maria Nykvist; Mia Brytting; Olov Svartstrom; Oskar Karlsson Lindsjo; Theresa Enkirch
EPI_ISL_434670, EPI_ISL_445237, EPI_ISL_450815, EPI_ISL_450820	Narhalsan Molnlycke, Barn och ungdomsmedicin	The Public Health Agency of Sweden	Anna Risberg; Anna-Malin Linde; Karin Tegmark-Wisell; Maria Lind Karlberg; Mats Reimer; Mia Brytting; Olov Svartstrom; Oskar Karlsson Lindsjo; Theresa Enkirch
EPI_ISL_475537	Narhalsan Oden VC	The Public Health Agency of Sweden	Anna Risberg; Anna-Malin Linde; Karin Tegmark-Wisell; Maria Lind Karlberg; Mattias Haukland; Mia Brytting; Olov Svartstrom; Oskar Karlsson Lindsjo; Reza Advani; Sandra Broddesson
EPI_ISL_445224, EPI_ISL_450830, EPI_ISL_469069, EPI_ISL_539882	Narhalsan Olskroken VC	The Public Health Agency of Sweden	Anna Risberg; Anna-Malin Linde; Karin Tegmark-Wisell; Mahin Ghoroghi; Maria Lind Karlberg; Mattias Haukland; Mia Brytting; Olov Svartstrom; Oskar Karlsson Lindsjo; Petra Edquist; Reza Advani; Sandra Broddesson; Shamam Muradrasoli; Theresa Enkirch
EPI_ISL_434671, EPI_ISL_450829, EPI_ISL_452238, EPI_ISL_469058, EPI_ISL_469060 to 469061, EPI_ISL_475524, EPI_ISL_475539			
see above	Narhalsan Sjobo vardcentral	The Public Health Agency of Sweden	Anna Risberg; Anna-Malin Linde; Karin Tegmark-Wisell; Lovisa Hjerten; Maria Lind Karlberg; Mattias Haukland; Mia Brytting; Olov Svartstrom; Oskar Karlsson Lindsjo; Petra Edquist; Reza Advani; Sandra Broddesson; Shamam Muradrasoli; Theresa Enkirch
EPI_ISL_523960, EPI_ISL_523962, EPI_ISL_523964, EPI_ISL_523966, EPI_ISL_523968	National Agency for Public Health, Republic of Moldova	Charite Universitatsmedizin Berlin, Institute of Virology	Ala Halacu; Barbara Muhlemann; Barbara Mühlemann; Christian Drosten; Elizabeta Jancheska; Golubinka Bosevska; Joern Beheim-Schwarzbach; Jorn Beheim-Schwarzbach; Julia Schneider; Maja Kuzmanovska; Mariana Apostol; Talitha Veith; Terry Jones; Victor M Corman
EPI_ISL_683449	National Center of Disease Control and Prevention of the Republic of Armenia	Charite Universitatsmedizin Berlin, Institute of Virology	Barbara Muehlemann; Christian Drosten; Julia Schneider; Jörn Beheim-Schwarzbach; National Center of Disease Control and Prevention of the Republic of Armenia Terry Jones; Talitha Veith; Victor M Corman
EPI_ISL_683450 to 683451	National Center of Disease Control and Prevention of the Republic of Armenia	Charité Universitätsmedizin Berlin, Institute of Virology	Barbara Muehlemann; Christian Drosten; Julia Schneider; Jörn Beheim-Schwarzbach; National Center of Disease Control and Prevention of the Republic of Armenia Terry Jones; Talitha Veith; Victor M Corman
EPI_ISL_454572	National Center of Expertise	National Center for Expertise, Kazakhstan National Center for Biotechnology, Kazakhstan	; Abdaliyev Askar; Akhmetollayev Ilyas; Amirgazin Asylulan; Aushakhmetova Zabira; Kalendar Ruslan; Lutsay Viktoriya; Rakhmetova Akbota; Ramankulov Yerlan; Shevtsov Alexandr
EPI_ISL_454571	National Center of Expertise	National Center for Expertise, National Center for Biotechnology, Kazakhstan	; Abdaliyev Askar; Akhmetollayev Ilyas; Amirgazin Asylulan; Aushakhmetova Zabira; Kalendar Ruslan; Lutsay Viktoriya; Rakhmetova Akbota; Ramankulov Yerlan; Shevtsov Alexandr
EPI_ISL_496524 to 496526, EPI_ISL_496531 to 496532, EPI_ISL_496535, EPI_ISL_496555 to 496587, EPI_ISL_721646 to 721651, EPI_ISL_721653 to 721657, EPI_ISL_721659 to 721667, EPI_ISL_722177 to 722186			
see above	National Centre For Cell Science	National Centre For Cell Science	Arvind Sahu; DBT's PAN-INDIA 1000 SARS-CoV2 RNA genome sequencing consortium; Dhiraj Paul; Girdhari Lal; Janesh Kumar; Kavita Bala Anand; Kunal Jani; Maharashtra COVID-19 Study Group; Manoj Kumar Bhat; Murlidhar Tambe; Radha Chauhan; Rajesh Karyakarte; Rajiv Mohan Gupta; Santosh Karade; Shelinder Pal Singh Shergill; Sourav Sen; Suvarna Joshi; Vasudevan Seshadri; Yogesh S Shouche
EPI_ISL_436413 to 436415, EPI_ISL_436417 to 436422, EPI_ISL_436424 to 436426, EPI_ISL_436428 to 436437, EPI_ISL_436440, EPI_ISL_436444 to 436445, EPI_ISL_436447 to 436463, EPI_ISL_482498, EPI_ISL_482501, EPI_ISL_482503, EPI_ISL_482509, EPI_ISL_482511 to 482513, EPI_ISL_482515, EPI_ISL_482531, EPI_ISL_482537, EPI_ISL_482545 to 482547, EPI_ISL_482552, EPI_ISL_482555 to 482556, EPI_ISL_482560, EPI_ISL_482563, EPI_ISL_482574, EPI_ISL_482587, EPI_ISL_482590 to 482591, EPI_ISL_482611 to 482614, EPI_ISL_482616, EPI_ISL_482620, EPI_ISL_482628 to 482631, EPI_ISL_482634 to 482635, EPI_ISL_482637, EPI_ISL_482640 to 482643, EPI_ISL_482650 to 482651, EPI_ISL_482655 to 482656, EPI_ISL_482660 to 482661, EPI_ISL_482663 to 482665, EPI_ISL_482669, EPI_ISL_636740, EPI_ISL_636742 to 636743, EPI_ISL_636746 to 636748, EPI_ISL_636750, EPI_ISL_636752 to 636757, EPI_ISL_636759 to 636762, EPI_ISL_636766, EPI_ISL_636770, EPI_ISL_636774, EPI_ISL_636776 to 636777, EPI_ISL_636780 to 636781, EPI_ISL_636787 to 636788, EPI_ISL_636795, EPI_ISL_636801 to 636802, EPI_ISL_636804 to 636805, EPI_ISL_636809, EPI_ISL_636811, EPI_ISL_636813, EPI_ISL_636815 to 636824, EPI_ISL_636826, EPI_ISL_636828 to 636831			
see above	National Centre for Disease control (NCDC)	NCDC/CSIR-IGIB	Aarti Tewari; Ajit Shewale; Anurag Agrawal#; Anurag Agrawal*; Aparna Swaminathan; Asangla Kamai; Bharathram Uppili; Bharathram Uppili2*; Bibhash Nandi; Debasis Dash; Dhirendra Kumar; Hema Gogia; Hemlata Lall; Himanshu Vashisht; Ishtaq Ahmed; Mahesh S Dhar; Mahesh S. Dhar1*; Manish Kumar; Manju Bala; Meena Datta; Mitali Mukerji; Mohammed Faruq; Mohammed Faruq#; Nidhi Saini; Nishu Tyagi; Partha Rakshit#; Partha Rakshit*;

			Pooja Sharma; Pooja Sharma2*; Poonam Gupta; Pramod Kumar#; Prateek Singh; Preeti Madan; Priyanka Singh; RadhaKrishnan VS; Radhakrishanan VS; Rajesh Pandey#; Robin Marwal; Robin Marwal1*; Sandhya Kabra; Saruchi Wadhwa; Satyabrata Bag; Shaista Khan; Simmi Tiwari; Simrita Singh; Sujeet Singh; Tushar Nale; Uma Sharma; Varun Jaiswal; Vivekanand A
EPI_ISL_435060 to 435072, EPI_ISL_435074, EPI_ISL_435078, EPI_ISL_435080 to 435088, EPI_ISL_435090 to 435106, EPI_ISL_435108 to 435112	National Centre for Disease control (NCDC), CSIR-Institute of Genomics and Integrative Biology (CSIR-IGIB)	NCDC/CSIR-IGIB	Aarti Tewari; Anurag Agrawal; Bharathram Uppili; Bibhash Nandi; Debasis Dash; Dhirendra Kumar; Hema Gogia; Hemlata Lall; Himanshu Vashisht; Mahesh Dhar; Manju Bala; Meena Datta; Mitali Mukerji; Mohammed Faruq; Nidhi Saini; Nishu Tyagi; Partha Rakshit; Pooja Sharma; Poonam Gupta; Pramod Kumar; Prateek Singh; Preeti Madan; Priyanka Singh; Rajesh Pandey; Sandhya Kabra; Saruchi Wadhwa; Satyabrata Bag; Simrita Singh; Sujeet Singh; Uma Sharma; Varun Jaiswal; Vivekanand A
EPI_ISL_407988, EPI_ISL_410535, EPI_ISL_414378 to 414380, EPI_ISL_420099 to 420111	National Centre for Infectious Diseases	Programme in Emerging Infectious Diseases, Duke-NUS Medical School	Barnaby E Young; Danielle E Anderson; David CB Lye; Gavin JD Smith; Jayanthi Jayakumar; Linfa Wang; Louisa Sun; Martin Linster; Yan Zhuang; Yee Sin Leo; Yvonne CF Su
EPI_ISL_466839, EPI_ISL_466841, EPI_ISL_466850, EPI_ISL_466853, EPI_ISL_466855, EPI_ISL_466857, EPI_ISL_466860, EPI_ISL_466863 to 466864, EPI_ISL_466866 to 466867, EPI_ISL_466870 to 466872, EPI_ISL_528548 to 528549, EPI_ISL_528555 to 528556, EPI_ISL_528560, EPI_ISL_528562, EPI_ISL_528576, EPI_ISL_528581, EPI_ISL_528586			
see above	National Genomics Core-Center for DNA Fingerprinting and Diagnostics	National Genomics Core- Center for DNA Fingerprinting and Diagnostics (NGC-CDFD)- DBT's PAN-INDIA-1000 Genome consortium	ASHWIN DALAL; Abhijeeth Singh Thakur; Ajay Kumar Chaudhary; Akash Chinchole; Amrita Bhattacherjee; Arjita Jaiswal; Arpita Singh; Ashwin Dalal; Bala Pratyusha; Bathula Siddardha; Bharath Kumar; Bhavani Sontam; Binod Bihari Pradhan; Brahmaji Sontyana; C Bala Maddeleti; C. Arun Kumar; Ch Venkateshwar Goud; Chandra Shekhar Singh; Chandra Shekar P; DEBASHIS MITRA; DIVYA VASHISHT; Debashis Mitra; Debashish Mitra; Devanshi Gupta; Devinder Singh Negi; Dhanraj Adey; Divya Vashisht; G Shashikanth; Heena Shah; J. Mallikarjun; Jamal Md Nurul Jain; Jenige Aravindh Kumar; K. Viswakalyan; Kaisar Ahmad Lone; Kandali Sreethi Sreenivasulu Reddy; Kausika Kumar Malik; Lavanya Banda; M Sri Lalitha; MURALI DHARAN BASHYAM; Mohammad Mudassir; Mugdha Singh; Murali Dharan Bashyam; Muthulakshmi; N. Sudheer; Nalini Raghunathan; Nanci Rani K; Neeraj Kumar; Nimmala Naresh; Niteen Pathak; Parveen Kumar; Pradipta Hore; R HARINARAYANAN; R Harinarayanan; R Lakshmi Vaishna; R. Angalena; R. Manorama; RASHNA BHANDARI; Rahul Baroi; Rajeshree Sanyal; Raju Kumar; Rashna Bhandari; Reelina Basu; Romila Moirangthem; S Vasantha Rani; SPR Prasad; Sandip Patra; Sanjana Sarkar; Sayantan Goswami; Shaffiqu T S; Shaik Nasar Vali; Shalini Aricthota; Shivani Yadav; Shruti Dasgupta; Shubhra Ganguli; Sobhan Babu; Srinivas G; Suchitra Upreti; Sunke Vijayakumar; Surya Chodisetty; Surya Vamshi; Swathi Chodisetty; T. Navaneetha; Usha Rani Dutta; V Naga Sailaja; Vani Singh; Vinay Donipadi; Vineesha Oddi; Yogesh Patidar
EPI_ISL_560386	National Health Laboratory	Botswana Institute for Technology Research and innovation	Dineo Emang Tshiamo. Gape Nyepetsi; Kefentse Arnold Tumedi; Madisa Mine; Maitshwarelo Ignatius Matsheka; Thongbotho Mphoyakgosi
EPI_ISL_464112 to 464114, EPI_ISL_464118 to 464119, EPI_ISL_464121, EPI_ISL_464123, EPI_ISL_464126 to 464133, EPI_ISL_464137 to 464138, EPI_ISL_464145, EPI_ISL_464153, EPI_ISL_464155, EPI_ISL_464157			
see above	National Health Laboratory Service (NHLS), Tygerberg	Division of Medical Virology, Stellenbosch University and National Health Laboratory Service (NHLS)	Bronwyn Kleinhans; Eduan Wilkindon; Gert van Zyl; Houriiyah Tegally; Kayla Delaney; Susan Engelbrecht; Tulio de Oliveira; Wolfgang Preiser
EPI_ISL_634978 to 634979, EPI_ISL_634981, EPI_ISL_634983 to 634987, EPI_ISL_634989 to 634990, EPI_ISL_634993 to 635019, EPI_ISL_635021 to 635022, EPI_ISL_635024 to 635041, EPI_ISL_635043, EPI_ISL_635045 to 635058			
see above	National Health Laboratory Service - Inkosi Albert Luthuli Central Hospital (NHLS-IALCH)	KRISP, KZN Research Innovation and Sequencing Platform	Giandhari J; Khan S; Lessells R; Mdlalose K; Pillay S; Tegally H; Wilkinson E; York D; de Oliveira T
EPI_ISL_944129 to 944177	National Health Laboratory Service, South Africa	KRISP, KZN Research Innovation and Sequencing Platform	Emmanuel SJ; Giandhari J; Khan S; Laguda-Akingba O; Lessells R; Mdlalose K; Pillay S; Tegally H; Wilkinson E; York D; de Oliveira T
EPI_ISL_456596 to 456597, EPI_ISL_456600, EPI_ISL_456606 to 456608, EPI_ISL_456611	National Health Laboratory, Timor-Leste	Microbiological Diagnostic Unit Public Health Laboratory, The Peter Doherty Institute for Infection and Immunity	Antonia da Costa, E.; Baird, R.; Barreto, I.; Caly, L.; Canisia, D.; Dakh, F.; Dolores de Jesus da Costa, M.; Douglas, N.; Francis, J.; Freeman, K.; Jayanti Pereira Tilman, A.; Marr, I.; Sait, M.; Salles de Sousa, A.; Schultz, M.; Seemann, T.; Sherry, N.; Soares da Silva, E.; Wapling, J.; Ximenes, J.
EPI_ISL_435303, EPI_ISL_435305, EPI_ISL_435308, EPI_ISL_435310 to 435317, EPI_ISL_455694 to 455712, EPI_ISL_455714, EPI_ISL_455718, EPI_ISL_511891 to 511898			
see above	National Hospital of Tropical Diseases	Oxford University Clinical Research Unit, Hanoi, Vietnam	H. Rogier van Doorn; H.Rogier van Doorn; Le Nguyen Minh Hoa; Nguyen Thi Hong Thuong; Nguyen Thi Ngoc Diep; Nguyen Thi Tam; Nguyen Thu Trang; Pham Ngoc Thach; Van Dinh Trang; Vu Thi Ngoc Bich; on behalf of the OUCRU COVID-19 research group
EPI_ISL_416028, EPI_ISL_416031 to 416032, EPI_ISL_416035 to 416036, EPI_ISL_515525	National Influenza Center - Instituto Adolfo Lutz	Instituto Adolfo Lutz, Interdiciplinary Procedures Center, Strategic Laboratory	Adriana Bugno; Adriano Abbud; Carlos Henrique Camargo; Claudia Regina Gonçalves; Claudio Tavares Sacchi; Daniela Bernardes Borges da Silva; Erica Valessa Ramos Gomes; Fabiana Cristina Pereira dos Santos; Maria do Carmo Sampaio Tavares Timenetsky; Simone Guadagnucci Morillo; Terezinha Maria de Paiva
EPI_ISL_408668	National Influenza Center - National Institute of Hygiene and Epidemiology (NIHE)	National Influenza Center - National Institute of Hygiene and Epidemiology (NIHE)	; Hoang Vu Mai Phuong; Le Thi Quynh Mai; Le Thi Thanh; Nguyen Le Khanh Hang; Nguyen Phuong Anh; Nguyen Vu Son; Pham Thi Hien; Tran Thu Huong; Ung Thi Hong Trang; Vuong Duc Cuong
EPI_ISL_486887 to 486889	National Influenza Center, Bahrain	National Influenza Center, Bahrain	AlWasti, H.; Altaif, Z.; Shehab, F.; Zaed, A.
EPI_ISL_420543, EPI_ISL_420545, EPI_ISL_420547, EPI_ISL_420549, EPI_ISL_420551, EPI_ISL_420553, EPI_ISL_420555, EPI_ISL_421662 to 421672, EPI_ISL_424361 to 424365, EPI_ISL_426179			
see above	National Influenza Center, Indian Council of Medical Research - National Institute of Virology	Indian Council of Medical Research-National Institute of Virology, Microbial Containment Complex	Amita Jain; Anita M. Shete; Anita Shete-Aich; Atanu Basu; Bharati Malhotra; Bharti Malhotra; Deepika Chaudhary; Dimpal A. Nyayanit; Gajanan Sapkal; Gururaj Deshpande; Hitesh Dighe; Lalit Dar; M Choudhary; Manohar. L. Chaudhary; Padinjaremattathil Thankappan Ullas; Pragya D. Yadav; Pragya D. Yadav. Savita Patil; Pranita Gawande; Prasad Sarkale; Priya Abraham; Sarah Cherian; Savita Patil; Sreelekshmy Mohandas; Triparna Majumdar; Varsha Potdar
EPI_ISL_416427 to 416431, EPI_ISL_660541, EPI_ISL_759869 to 759891, EPI_ISL_766029, EPI_ISL_977145 to 977152	National Influenza Center, National Institute of Hygiene and Epidemiology (NIHE)	National Influenza Center, National Institute of Hygiene and Epidemiology (NIHE)	Dang Duc Anh; Futoshi Hasebe; Hoang Vu Mai Phuong; Kouichi Morita; Le Quynh Mai; Le Thi Thanh; Meng Ling Moi; Nguyen Le Khanh Hang; Nguyen Phuong Anh; Nguyen Vu Son; Pham Hong Quynh Anh; Pham Thi Hien; Taichiro Takemura; Takeshi Nabeshima; Tran Thu Huong; Ung Thi Hong Trang; Vuong Duc Cuong
EPI_ISL_660542	National Influenza Center, National Institute of Hygiene and Epidemiology (NIHE)	National Institute of Hygiene and Epidemiology (NIHE)	Dang Duc Anh; Futoshi Hasebe; Hoang Vu Mai Phuong; Kouichi Morita; Le Quynh Mai; Le Thi Thanh; Meng Ling Moi; Nguyen Le Khanh Hang; Nguyen Phuong Anh; Nguyen Vu Son; Pham Hong Quynh Anh; Pham Thi Hien; Taichiro Takemura; Takeshi Nabeshima; Tran Thu Huong; Ung Thi Hong Trang; Vuong Duc Cuong
EPI_ISL_959284	National Influenza Center, Virology Department	National Influenza Center	A Nejati; F Ajaminejad and T Mokhtari Azad; J Yavarian; K Sadeghi; N Ghavvami; NZ Shafiei Jandaghi; V Salimi
EPI_ISL_467778 to 467781	National Influenza Centre Romania	Charite Universitatsmedizin Berlin, Institute of Virology	Barbara Muehlemann; Christian Drosten; Jorn Beheim-Schwarzbach; Julia Schneider; L. Ustea; M. Lazar; N. Paraschiv; Talitha Veith; Terry Jones; Victor M Corman
EPI_ISL_410301	National Influenza Centre, National Public Health Laboratory, Kathmandu, Nepal	The University of Hong Kong	Alfonso J. Rodriguez-Morales; Anup Bastola; Basu Dev Pandey; Bibek Kumar Lal; Daniel Chu; Haogao Gu; Leo Poon; Malik Peiris; Ranjit Sah; Runa Jha
EPI_ISL_596386	National Institute for Allergy and Infectious Diseases Integrated Research Facility - Frederick (NIAID IRF- Frederick), National Institutes of Health (NIH)	National Institute for Allergy and Infectious Diseases Integrated Research Facility - Frederick (NIAID IRF-Frederick), National Institutes of Health (NIH)	B. and Palacios, G.; Beitzel; J.R.; Kocher, G.; Kugelman
EPI_ISL_402125	National Institute for Communicable Disease Control and Prevention (ICDC) Chinese Center for Disease Control and Prevention (China CDC)	National Institute for Communicable Disease Control and Prevention (ICDC) Chinese Center for Disease Control and Prevention (China CDC)	Chen; Dai; F.-H.; Hu, Y.; J.-H.; J.-J.; J.-L. and Zhu; Liu, Y.; Pei; Q.-M.; She; Song; T.-Y.; Tao; Tian; Wang; Wang, W.; Wu, F.; Xu, L.; Y.-L.; Y.-M.; Y.-Y.; Y.-Z.; Yu, B.; Z.-G.; Z.-W.; Zhang; Zhao, S.; Zheng
EPI_ISL_417186, EPI_ISL_430297, EPI_ISL_514355, EPI_ISL_514360, EPI_ISL_514367, EPI_ISL_514375, EPI_ISL_514382, EPI_ISL_514424, EPI_ISL_515156, EPI_ISL_515166, EPI_ISL_515172, EPI_ISL_622922, EPI_ISL_622934 to 622941			
see above	National Institute for Communicable Diseases of the National Health Laboratory Service	National Institute for Communicable Diseases of the National Health Laboratory Service	A; Allam M; Bhiman JN; Ismail A; Khumalo Z; Kwenda S; Mnyameni F; Mohale T; Mtshali P; Subramoney K; van Heusden P; von Gottberg
EPI_ISL_710542 to 710546, EPI_ISL_718262 to 718265, EPI_ISL_721624 to 721628, EPI_ISL_728279 to 728287, EPI_ISL_735510, EPI_ISL_746826 to 746835, EPI_ISL_765567 to 765573, EPI_ISL_803898			
see above	National Institute for Infectious Diseases, INMI, "L. Spallanzani" IRCCS	National Institute for Infectious Diseases, INMI, "L. Spallanzani" IRCCS	A Di Caro; A. Di Caro; B Bartolini; B. Bartolini; C.E.M Gruber; E Giombini; E. Giombini; F Messina; F. Carletti; F. Messina; G Parisi; M Rueca; M. Rueca; M.R Capobianchi; MR Capobianchi; O Butera
EPI_ISL_469254 to 469255	National Institute for Viral Disease Control and Prevention, China CDC	Institute of Viral Disease Control and Prevention, China CDC	Chun Huang; Dayan Wang; George F. Gao; George Fu Gao; Guizhen Wu; Jun Han; Li Zhao; Lijuan Chen; Peihua NiuBaoying Huang; Roujian Lu; Shiwen Wang; Weifeng Shi; Wenbo Xu; Wenjie Tan; Wenling Wang; William J. Liu; Xiang ZhaoLijuan Chen; Yanhai Wang; Yao MengZhixiao ChenYuchao Wu; Yong Zhang; Yubai Bi

EPI_ISL_408480 to 408482, EPI_ISL_408484 to 408486, EPI_ISL_408488	National Institute for Viral Disease Control and Prevention, China CDC	National Institute for Viral Disease Control & Prevention, CCDC	Baoying Huang; Fei Ye; George F. Gao; Guizhen Wu; Hengqin Wang; Huiping Yang; Jianan Xu; Jianxiong Li; Kangchen Zhao; Li Zhao; Peihua Niu; Quanyi Wang; Roujian Lu; Sheng Ye; Shenjiao Wang; Ti Liu; Wenbo Xu; Wenjie Tan; Wenjie TanXiaoqing FuXiang ZhaoWenling Wang Peihua NiuRoujian Lu; Wenling Wang; Xiang Zhao; Yang Pan; Yanhong SunBaoying HuangLi ZhaoFei YeWenbo XuGeorge F. GaoGuizhen Wu; Yong Shi; Zhaoguo Wang
EPI_ISL_402119 to 402121, EPI_ISL_469256, EPI_ISL_498691 to 498694, EPI_ISL_575330, EPI_ISL_591270 to 591280	National Institute for Viral Disease Control and Prevention, China CDC	National Institute for Viral Disease Control and Prevention, China CDC	Cao Chen; Chun Huang; Dayan Wang; Dong Xia; Faxian ZhanWeifeng ShiBaoying HuangJun LiuLi ZhaoYao MengFei YeNa Zhu; Geogia Fu Gao; George F. Gao; George F.Gao; Guizhen Wu; Haijun Du; Hong Wang; Huilai Ma; Ji Wang; Jingdong Song; Juan Song; Jun Han; Kang Xiao; LingLing Mao; Qinqin Song; Rongbao Gao; Ruqin Gao; Shiwen Wang; Shuai Pang; Weifeng Shi; Weimin Zhou; Weimin ZhouPeihua NiuPeipei LiuFaxian ZhanWeifeng ShiBaoying HuangJun LiuLi ZhaoYao MengXiaozhou HeFei YeNa ZhuYang LiJing ChenWenbo XuGeorge F. GaoGuizhen Wu; WenQing YaoWenbo Xu; Wenbo Xu; Wenjie TanXiang ZhaoWenling WangXuejun MaYongzhong JiangRoujian Lu; Wenjie TanXiang ZhaoWenling WangXuejun MaYongzhong JiangRoujian LuJi WangWeimin ZhouPeihua NiuPeipei LiuFaxian ZhanWeifeng ShiBaoying HuangJun LiuLi ZhaoYao MengXiaozhou HeFei YeNa ZhuYang LiJing ChenWenbo XuGeorge F. GaoGuizhen Wu; Wenjie TanXuejun MaXiang ZhaoWenling WangYongzhong JiangRoujian LuJi WangPeihua Niu; William J. Liu; Xiang Zhao; Xiang ZhaoLijuan Chen; Xiaozhou HePeipei Liu; Xuancheng Lu; Yang LiJing ChenWenbo XuGeorge F. GaoGuizhen Wu; Yang Song; Yanhai Wang; Yao Meng; Yao MengZhixiao ChenYuchao Wu; Yong Zhang; Yong ZhangBo ZhijianJianqun Zhang; Yuan He; Yuchao Wu; Zhaoguo Wang; ZhiQiang Xia; Zhixiao Chen
EPI_ISL_434534	National Institute for Viral Disease Control and Prevention, China CDC	National Institute for Viral Disease Control and Prevention, China CDC, Yunnan Provincial CDC	Baoying Huang; Fei Ye; Guizhen Wu; Huijuan Wang; Li Zhao; Peihua Niu; Roujian Lu; Wenjie Tan; Wenling Wang
EPI_ISL_458133	National Institute of Biotechnology	Bioinformatics Division, National Institute of Biotechnology	Arittra Bhattacharjee; Asif Rashid; Chaman Ara Keya; Keshob Chandra Das; Md. Hadisur Rahman; Md. Moniruzzaman; Md. Nazrul Islam; Md. Ruhul Amin; Md. Salim Khan; Md. Salimullah; Mohammad Uzzal Hossain
EPI_ISL_447904	National Institute of Biotechnology	National Institute of Biotechnology	Arittra Bhattacharjee; Asif Rashid; Chaman Ara Keya; Irfan Ahmed; Keshob Chandra Das; Md. Hadisur Rahman; Md. Moniruzzaman; Md. Nazrul Islam; Md. Ruhul Amin; Md. Salimullah; Mohammad Uzzal Hossain; Tahia Anan Rahman
EPI_ISL_708839 to 708840	National Institute of Blood Diseases (NIBD), Molecular Biology Lab	Genomics Lab NIBD	Aneeta Shahni; Arshi Naz; Gul Sufaida; Samina Naz Mukry; Sayed Ali Raza; Shariq Ahmed; Tahir Sultan Shamsi
EPI_ISL_538504 to 538505, EPI_ISL_538508 to 538509, EPI_ISL_574433, EPI_ISL_576386 to 576387, EPI_ISL_747233 to 747234, EPI_ISL_756376, EPI_ISL_756401, EPI_ISL_757287			
see above	National Institute of Health Research and Development	National Institute of Health Research and Development	A; AA; Adam; Agustiningsih; D; F; Febriyani; Febriyanti; HA; HD; Hariastuti; Herman; Herna; IL; Ikawati; Indalao; Indalau; Indrasari; J; K; KD; KNA; Kipuw; Kurniawati; M; Monika; Muna; Mursinah; N; ND; NI; NK; NL; Nikmah; Nugraha; Nurhadi; Paisal; Pangesti; Pawestri; Puspa; Puspandari; Puspandary; R; Ramadhany; Rizki; Rukminiati; Setiawaty; Setiawaty, V.; Soekarso; Subangkit; Susanti; Susilarini; T; UA; V; Vivi; Wibowo; Wulandari; Y
EPI_ISL_489991, EPI_ISL_515472 to 515473, EPI_ISL_515475 to 515477	National Institute of Health, Department of Medical Sciences, Ministry of Public Health, Thailand	National Institute of Health, Department of Medical Sciences, Ministry of Public Health, Thailand	Chittaganpitch; Malinee; Malinee Chittaganpitch; Okada; Parnmen; Phuygun; Pilailuk; Pilailuk Okada; Siripaporn; Siripaporn Phuygun; Sittiporn; Sittiporn Parnmen; Sunthareeya; Sunthareeya Waicharoen; Thanadachakul; Thanutsapa; Thanutsapa Thanadachakul; Waicharoen; Warawan; Warawan Wongboot; Wongboot
EPI_ISL_450197, EPI_ISL_455584, EPI_ISL_455589 to 455593, EPI_ISL_455624, EPI_ISL_515287 to 515291, EPI_ISL_515463, EPI_ISL_515470			
see above	National Institute of Health. Department of medical Sciences, Ministry of Public Health, Thailand	National Institute of Health. Department of medical Sciences, Ministry of Public Health, Thailand	Chittaganpitch; Malinee; Okada; Parnmen; Phuygun; Pilailuk; Siripaporn; Sittiporn; Sunthareeya; Thanadachakul; Thanutsapa; Waicharoen; Warawan; Wongboot
EPI_ISL_479756 to 479758	National Institute of Hygiene and Epidemiology (NIHE)	National Key Laboratory of Gene Technology, Institute of Biotechnology (IBT)	Chu Hoang Ha; Dang Duc Anh; Futoshi Hasebe; Ho Thi Thuong; Hoang Ha; Hoang Vu Mai Phuong; Le Quynh Mai; Le Thi Thanh; Le Tung Lam; Nguyen Hong Trang; Nguyen Le Khanh Hang; Nguyen Phuong Anh; Nguyen Vu Son; Pham Thi Hien; Taichiro Takemura; Tran Huyen Linh; Tran Thu Huong; Truong Nam Hai; Ung Thi Hong Trang; Vuong Duc Cuong
EPI_ISL_794744, EPI_ISL_862835 to 862836, EPI_ISL_875346, EPI_ISL_885152 to 885154, EPI_ISL_979544, EPI_ISL_979799, EPI_ISL_979801 to 979804, EPI_ISL_979893, EPI_ISL_979959 to 979961, EPI_ISL_979963 to 979969			
see above	National Institute of Infectious Diseases-Prof. Dr. Matei Bals Molecular Diagnostics Laboratory	National Institute of Infectious Diseases-Prof. Dr. Matei Bals Molecular Diagnostics Laboratory	Andreea Tudor; Corina Casangiu; Dan Otelea; Leontina Banica; Marius Surleac; Petre Milu; Simona Paraschiv
EPI_ISL_480414 to 480421, EPI_ISL_480424 to 480427, EPI_ISL_480439 to 480444	National Institute of Laboratory Medicine and Referral Center	Bangladesh Council of Scientific and Industrial Research	A. K. M. Shamsuzzaman; Abu Sayeed Mohammad Mahmud; Asish Kumar Ghosh; Barna Goswami; Eshrar Osman; Iffat Jahan; Mahmuda Yeasmin; Md. Ahasan Habib; Md. Maruf Ahmed Molla; Md. Murshed Hasan Sarkar; Md. Saddam Hossain; Md. Salim Khan; Mohammad Samir Uzzaman; Salek Ahmed Sajib; Shahina Akter; Shahjahan Siddike; Sheikh Md. Selim Al Din; Tanjina Akhter Banu; Tasnim Nafisa; Utpal Chandra Ray
EPI_ISL_455420, EPI_ISL_455458 to 455459, EPI_ISL_462090 to 462098, EPI_ISL_464159 to 464166, EPI_ISL_465163 to 465164, EPI_ISL_466626 to 466630, EPI_ISL_466636 to 466639, EPI_ISL_466644 to 466645, EPI_ISL_466649 to 466650, EPI_ISL_466686 to 466691, EPI_ISL_466693 to 466694, EPI_ISL_469286, EPI_ISL_469297 to 469300, EPI_ISL_475083 to 475084, EPI_ISL_475165 to 475173, EPI_ISL_475238, EPI_ISL_475754 to 475761, EPI_ISL_480445 to 480450, EPI_ISL_482485 to 482489, EPI_ISL_482700 to 482701, EPI_ISL_483622 to 483624, EPI_ISL_483626 to 483636, EPI_ISL_483641 to 483646, EPI_ISL_483686 to 483687, EPI_ISL_483689 to 483695, EPI_ISL_483699 to 483700, EPI_ISL_483703, EPI_ISL_483705, EPI_ISL_483707, EPI_ISL_483710, EPI_ISL_487362 to 487364, EPI_ISL_487366 to 487367, EPI_ISL_487371 to 487373, EPI_ISL_487375 to 487376, EPI_ISL_487378, EPI_ISL_487380, EPI_ISL_487382 to 487386, EPI_ISL_487392 to 487396, EPI_ISL_490109 to 490111, EPI_ISL_490113 to 490114, EPI_ISL_490144, EPI_ISL_490164 to 490165, EPI_ISL_490167 to 490168, EPI_ISL_498227 to 498228, EPI_ISL_498253 to 498254, EPI_ISL_498267 to 498268, EPI_ISL_498273 to 498274, EPI_ISL_498417, EPI_ISL_498419, EPI_ISL_498466 to 498467, EPI_ISL_498549 to 498550, EPI_ISL_498783 to 498797, EPI_ISL_498800 to 498802, EPI_ISL_498804 to 498806, EPI_ISL_498808, EPI_ISL_498811, EPI_ISL_498814 to 498818, EPI_ISL_498830, EPI_ISL_498892, EPI_ISL_498932 to 498933, EPI_ISL_503958, EPI_ISL_504040, EPI_ISL_504137, EPI_ISL_504176 to 504184, EPI_ISL_514129 to 514130, EPI_ISL_514228 to 514229, EPI_ISL_514231 to 514252, EPI_ISL_603221 to 603225, EPI_ISL_603238 to 603247, EPI_ISL_603249, EPI_ISL_943561			
see above	National Institute of Laboratory Medicine and Referral Center	Genomic Research Lab, BCSIR	; A. K. M. Shamsuzzaman; Abu Sayeed Mohammad Mahmud; Abu Sayeed Mohammad Mahmud; Arifa Akram; Asish Kumar Ghos; Asish Kumar Ghosh; Barna Goswami; Bayzid Bin Monir; Eshrar Osman; Iffat Jahan; Mahmuda Yeasmin; MahmudaYeasmin; Md. Ahasan Habib; Md. Ahashan Habib; Md. Maruf Ahmed Molla; Md. Masum Hossain Arif; Md. Murshed Hasan Sarkar; Md. Murshed Hasan Sarker; Md. Saddam Hossain; Md. Salim Khan; Mohammad Samir Uzzaman; Monira Parveen; Salek Ahmed Sajib; Shahina Akhter; Shahina Akter; Shahjahan Siddike; Sheikh Md. Selim Al Din; Tanjina Akhtar Banu; Tanjina Akhter Banu; Tanjina Akhter Banu; Tarannum Taznin; Tasnim Nafisa; Tasnim Nafisa; Utpal Chandra Ray
EPI_ISL_539776, EPI_ISL_539778 to 539782	National Institute of Public Health (Czech Republic)	State Veterinary Institute Prague	A; D; H; Jirincova; L; Nagy; Novakova; Trnka; Vecerova, J.
EPI_ISL_905748, EPI_ISL_905751, EPI_ISL_905756, EPI_ISL_905758, EPI_ISL_905761	National Institute of Public Health - National Institute of Hygiene	National Institute of Public Health - National Institute of Hygiene	Wokowicz Tomasz; Zacharczuk Katarzyna
EPI_ISL_825149	National Institute of Virology Bangalore Unit (NIV BU)	Department of Neurovirology, National Institute of Mental Health and Neurosciences (NIMHANS)	Anita S Desai; Chitra Pattabiraman; Pramada Prasad; V Ravi
EPI_ISL_541681 to 541685, EPI_ISL_541687 to 541702, EPI_ISL_541704 to 541708, EPI_ISL_541710 to 541711, EPI_ISL_541713 to 541721, EPI_ISL_541723 to 541731, EPI_ISL_541733 to 541748, EPI_ISL_541751			
see above	National Institute of Virology, NIV Influenza	National Institute of Virology, NIV Influenza	Potdar V
EPI_ISL_434555, EPI_ISL_434558	National Institutes of Health, University of the Philippines Manila	Philippine Genome Center	Alessandra C. Sanchez; Benedict A. Maralit; Bernard Demot; Carlo M. Lapid; Christina Tan; El King D. Morado; Eva Maria Cutiongco-de la Paz; Francis A. Tablizo; Haifa L.Gaza; Jan Michael C. Yap; Jarvin E. Nipales; Jo-Hannah S. Llames; John Mark Velasco; Joshua Gregor A. Dizon; Joy Ann Petronio-Santos; Julius Aaron Mejia; Kris P. Punayan; Kristianne Arielle D. Gabriel; Maribell Dollete; Marissa M. Alejandria; Raul V. Destura; Shana F. Genavia; Shebna Rose D. Fabilloren; Shiela Mae M. Araiza; Sonia Salamat; and Cynthia P. Saloma
EPI_ISL_1033077, EPI_ISL_1033091	National Laboratory for Health, Environment and Food	CISLD (Clinical Institute of Special Laboratory Diagnostics), University Children's Hospital, University Medical Center Ljubljana	Ana Grom; Barbara Jenko Bizjan; Jernej Kova; Katarina Kozmos; Marko Pokorn; Maruša Debeljak; Robert Šket; Tadej Battelino; Tine Tesovnik
EPI_ISL_449799, EPI_ISL_892244	National Laboratory for Health, Environment and Food	National Laboratory for Health, Environment and Food	Aleksander Mahnic; Blazun Vosner H.; Duh D.; Hedzet S.; Janezic S.; Mahnic A.; Maja Rupnik; Rupnik M.; Sandra Janezic; Zavrsnik J.
EPI_ISL_512597 to 512600, EPI_ISL_512603 to 512612, EPI_ISL_512614 to 512618, EPI_ISL_512620 to 512626, EPI_ISL_512628 to 512629, EPI_ISL_512631 to 512632, EPI_ISL_512634, EPI_ISL_512636, EPI_ISL_512638, EPI_ISL_512640 to 512643, EPI_ISL_512810			
see above	National Laboratory for Influenza/Virology reference laboratory, Public Health Center of the Ministry of Health of Ukraine	Respiratory Virus Unit, Microbiology Services Colindale, Public Health England	Dr. Iryna Demchyshyna; PHE Covid Sequencing Team
EPI_ISL_483637, EPI_ISL_671414 to 671418	National Laboratory of Virology, Szentágothai Research Centre	National Laboratory of Virology, Szentágothai Research Centre	Balázs Somogyi; Brigitta; Endre Gábor Tóth; Ferenc Jakab; Gábor Kemenesi

EPI_ISL_644786 to 644821, EPI_ISL_647978	National Microbiology Reference Laboratory	Quadram Institute Bioscience	Agnes Juru; Alexander Goredema; Ana-Victoria Gutierrez; Andrew J. Page; Andrew Tarupiwa; Barbra Murwira; Beuty Makamure; Charles Nyagupe; David Baker; Gaetan Thilliez; Gemma Kay; Gibson Mhlanga; Hlanai Gumbo; Isaac Phiri; Justin O'Grady; Leonardo de Oliveira Martins; Muchaneta Mugabe; Portia Manangazira; Robert Kingsley; Sekesai Zinyowera; Tapfumanei Mashe; Tatenda Takawira; Thanh Le Viet
EPI_ISL_738086	National Public Health Laboratory	Department of Medical Microbiology, Hospital Pengajar Universiti Putra Malaysia	Hui-Yee Chee; Jia-Yong Lam; Muhammad MI; Narcisse Joseph; Nor Zahrin Hasran; Syafinaz Amin-Nordin; Yien-Ping Wong
EPI_ISL_457827 to 457829, EPI_ISL_457833, EPI_ISL_457843	National Public Health Laboratory	KEMRI-Wellcome Trust Research Programme/KEMRI-CGMR-C Kilifi	Githinji G. et al 2020
EPI_ISL_416829, EPI_ISL_416866, EPI_ISL_416885 to 416886, EPI_ISL_416907	National Public Health Laboratory	Malaysia Genome Institute	Azrin Ahmad; Enizza Kasim; Hani Mat Hussin; Irni Suhayu Sapian; Mohd Faizal Abu Bakar; Mohd Noor Mat Isa; Noorliza Mohamad Noordin; Nor Azfa Johari; Norazimah Tajudin; Nurhezreen Md Iqbal; Rehan Shuhada Abu Bakar; Selvanesan Sengol; Shahrul Hisham Zainal Ariffin; Shamsidar Sopie; Siti Noraini Othman; W Afiza W Mohd Arifin; Yu Kie Chem; Yusuf Muhammad Noor
EPI_ISL_410719	National Public Health Laboratory	National Public Health Laboratory	Cui L; Lin RTP; Mak TM; Octavia S
EPI_ISL_410716	National Public Health Laboratory, National Centre for Infectious Diseases	National Centre for Infectious Diseases, National Centre for Infectious Diseases	Cui L; Lin RTP; Mak TM; Octavia S
EPI_ISL_410713 to 410715, EPI_ISL_418992 to 419001, EPI_ISL_422428 to 422435, EPI_ISL_428822 to 428850, EPI_ISL_435678 to 435700, EPI_ISL_443188 to 443193, EPI_ISL_443195, EPI_ISL_443197 to 443249, EPI_ISL_462276 to 462288, EPI_ISL_462290 to 462293, EPI_ISL_462295 to 462303, EPI_ISL_462305 to 462326, EPI_ISL_462328, EPI_ISL_462338, EPI_ISL_462340 to 462361, EPI_ISL_462369 to 462433, EPI_ISL_469081 to 469096, EPI_ISL_469098 to 469121, EPI_ISL_469124 to 469155, EPI_ISL_469274, EPI_ISL_475937 to 475951, EPI_ISL_475953 to 475955, EPI_ISL_475957 to 475960, EPI_ISL_475962 to 475963, EPI_ISL_475966 to 475973, EPI_ISL_475979, EPI_ISL_475981, EPI_ISL_475983, EPI_ISL_475985 to 475986, EPI_ISL_475990 to 475991, EPI_ISL_475993, EPI_ISL_475995, EPI_ISL_475997, EPI_ISL_479574 to 479578, EPI_ISL_479580 to 479603, EPI_ISL_483580 to 483592, EPI_ISL_483596 to 483621, EPI_ISL_490049, EPI_ISL_490052 to 490054, EPI_ISL_490057 to 490058, EPI_ISL_490060 to 490079, EPI_ISL_493390 to 493392, EPI_ISL_493395, EPI_ISL_493401, EPI_ISL_493408 to 493425, EPI_ISL_498564 to 498571, EPI_ISL_498581 to 498595, EPI_ISL_498601 to 498605, EPI_ISL_498608 to 498611, EPI_ISL_498613, EPI_ISL_498615 to 498616, EPI_ISL_498618, EPI_ISL_512822 to 512830, EPI_ISL_512832 to 512842, EPI_ISL_516807 to 516826, EPI_ISL_527359 to 527368, EPI_ISL_527370 to 527377, EPI_ISL_527379, EPI_ISL_536412 to 536450, EPI_ISL_548972 to 548974, EPI_ISL_548977 to 549005, EPI_ISL_574493 to 574540, EPI_ISL_596456 to 596483, EPI_ISL_596485 to 596496, EPI_ISL_605817 to 605824, EPI_ISL_626628 to 626635, EPI_ISL_626639 to 626643, EPI_ISL_645115 to 645118, EPI_ISL_645120 to 645132, EPI_ISL_654810 to 654817, EPI_ISL_693307 to 693331, EPI_ISL_728179 to 728188, EPI_ISL_728190 to 728196, EPI_ISL_728198 to 728199, EPI_ISL_754084 to 754108, EPI_ISL_768618 to 768630, EPI_ISL_803959 to 803964, EPI_ISL_825082 to 825083, EPI_ISL_833373 to 833375, EPI_ISL_857469 to 857471, EPI_ISL_862816 to 862823, EPI_ISL_891231 to 891235, EPI_ISL_906833 to 906835, EPI_ISL_937515 to 937516, EPI_ISL_953385 to 953388, EPI_ISL_981007 to 981010, EPI_ISL_981012, EPI_ISL_995294 to 995295, EPI_ISL_1034260 to 1034262			
see above	National Public Health Laboratory, National Centre for Infectious Diseases	National Public Health Laboratory, National Centre for Infectious Diseases	Chavatte JM; Chavatte Jean-Marc; Cui L; Cui Lin; Lin Cui; Lin RTP; Lin Raymond Tzer Pin; Mak TM; Mak Tze Minn; Octavia S; Octavia Sophie; Raymond Tzer Pin Lin; Sophie Octavia; Tze Minn Mak; Zhenyang Zhou; Zhou Z
EPI_ISL_416741, EPI_ISL_450496 to 450497	National Public Health Surveillance Laboratory, Vilnius, Lithuania	Charite Universitaetsmedizin Berlin, Institute of Virology	Ana Steponkiene; Barbara Muehlemann; Christian Drosten; Jorn Beheim-Schwarzbach; Jorn Beheim-Schwarzbach; Julia Schneider; Talitha Veith; Terry Jones; Victor M Corman
EPI_ISL_480224, EPI_ISL_480297 to 480310	National Reference Laboratory "Influenza and acute respiratory diseases"	NRL-HIV	Ivailo Alexiev; Ivan Ivanov; Ivva Philipova
EPI_ISL_833578	National Reference Laboratory for COVID-19, Pasteur Institute of Iran	National Reference Laboratory for COVID-19, Pasteur Institute of Iran	Ahmad Ghasemi; Farideh Niknam; Hessam Nemati; Kayhan Azadmanesh; Mahsa Tavakoli; Maryam Rostamtabar; Marzieh Sadjadi; Mohamad Sadegh Shams Nosrati; Mohammad Hassan Pouriayevali; Mohammad Mehdi Mortazavipour; Mostafa Salehi-Vaziri; Parastoo Yekta; Sahar Khakifirouz; Sana Eybpoosh; Sanam Azad-Manjiri; Sepideh Gerdooei; Setareh Kashanian; Tahereh Mohammadi; Tahmineh Jalali; Zabihollah Shoja; Zahra Ahmadi; Zahra Fereydouni; Zeynab VeisiZadeh
EPI_ISL_737201	National Reference Laboratory, Nigeria Centre for Disease Control.	National Reference Laboratory, Nigeria Centre for Disease Control, Gaduwa,Abuja, Nigeria	Akinpelu Afolabi; Anthony Ahumibe; Catherine Okoi; Celestina Obiekea; Chimaobi Chukwu; Dr Adesuyi Omoare; Dr Chikwe Ihekweazu; Dr Chinwe Ochu; Dr Ndodo Nnaemeka; Dr Sikiru Badaru; Grace Esebanmen; Kingsley Njoku; Nwando Mba; Olusola Akanbi; Shirlee Wohl; oluwaseyi Ajegbe
EPI_ISL_507008	National Veterinary Institute	National Veterinary Institute	Siamak Zohari
EPI_ISL_435674 to 435677, EPI_ISL_443187	National Virology Reference Laboratory	National Public Health Laboratory, National Centre for Infectious Diseases	Chavatte Jean-Marc; Cui Lin; Lin Raymond Tzer Pin; Mak Tze Minn; Octavia Sophie; Taib Surita; Zaini Zainun
EPI_ISL_666696 to 666710	National Virus Reference Lab (NVRL)	Irish Coronavirus Sequencing Consortium-Teagasc Grange	Aljandro Abner Garcia Leon; Calum Walsh; Fiona Crispie; Matthew McCabe; Michael Carr; Paul Cotter
EPI_ISL_605058, EPI_ISL_605060 to 605067, EPI_ISL_605069 to 605070, EPI_ISL_605072 to 605076, EPI_ISL_605078 to 605080	National Virus Reference Laboratory	Irish Coronavirus Sequencing Consortium - Helixworks	Conor Crosbie; Nimesh Pinnamaneni; Sachin Chalapati
EPI_ISL_671337 to 671400, EPI_ISL_671402 to 671403, EPI_ISL_681893 to 681928, EPI_ISL_682057, EPI_ISL_763350, EPI_ISL_763352, EPI_ISL_767724, EPI_ISL_767728 to 767838, EPI_ISL_768744, EPI_ISL_768746 to 768750, EPI_ISL_791292			
see above	National Virus Reference Laboratory	Irish Coronavirus Sequencing Consortium - Teagasc Moorepark	Alejandro Abner Garcia Leon; Calm Walsh; Calum Walsh; Fiona Crispie; Genuity Ireland; John Kenny; Matthew McCabe; Paddy Mallon; Paul Cotter
EPI_ISL_708526 to 708527	National Virus Reference Laboratory	Irish Coronavirus Sequencing Consortium - Teagasc Oakpark	Ewen Mullins; Matthew McCabe; Michele Della Bartola
EPI_ISL_500573 to 500578, EPI_ISL_500580 to 500589, EPI_ISL_500591, EPI_ISL_500593 to 500595, EPI_ISL_501259, EPI_ISL_501268, EPI_ISL_501273 to 501274, EPI_ISL_512087 to 512089, EPI_ISL_512091 to 512094, EPI_ISL_512097 to 512101, EPI_ISL_512103 to 512106, EPI_ISL_525352 to 525353, EPI_ISL_525356 to 525362, EPI_ISL_525364 to 525376, EPI_ISL_525382 to 525388, EPI_ISL_525390, EPI_ISL_525394, EPI_ISL_525396 to 525418, EPI_ISL_528440 to 528447, EPI_ISL_528449 to 528452, EPI_ISL_528456 to 528457, EPI_ISL_528459 to 528464, EPI_ISL_528467 to 528468, EPI_ISL_528471 to 528472, EPI_ISL_528475, EPI_ISL_528477, EPI_ISL_528479 to 528484, EPI_ISL_578241, EPI_ISL_578246 to 578334, EPI_ISL_596889 to 596891, EPI_ISL_596893 to 596915, EPI_ISL_596917 to 596925, EPI_ISL_596927, EPI_ISL_639825 to 639827, EPI_ISL_639829 to 639831, EPI_ISL_639833 to 639836, EPI_ISL_639838 to 639848, EPI_ISL_639850 to 639852, EPI_ISL_639854 to 639856, EPI_ISL_639859 to 639904, EPI_ISL_639906 to 639908, EPI_ISL_671864 to 671885, EPI_ISL_671888 to 671894, EPI_ISL_671899 to 671916, EPI_ISL_671918 to 671931, EPI_ISL_671934 to 671935, EPI_ISL_671937 to 671939, EPI_ISL_732458 to 732523, EPI_ISL_752511 to 752536, EPI_ISL_752538 to 752555, EPI_ISL_752597, EPI_ISL_789063 to 789082, EPI_ISL_789087, EPI_ISL_789089 to 789091, EPI_ISL_789093, EPI_ISL_789095 to 789096, EPI_ISL_789098 to 789101, EPI_ISL_837375 to 837394, EPI_ISL_875364 to 875366, EPI_ISL_875392 to 875400, EPI_ISL_875413 to 875430, EPI_ISL_875450, EPI_ISL_875453 to 875455, EPI_ISL_875464 to 875478, EPI_ISL_875502 to 875506, EPI_ISL_875686, EPI_ISL_888830 to 888834, EPI_ISL_909833 to 909848, EPI_ISL_909850 to 909893, EPI_ISL_909909, EPI_ISL_911245, EPI_ISL_959702, EPI_ISL_959722 to 959725, EPI_ISL_959738, EPI_ISL_959745 to 959746, EPI_ISL_959750 to 959764, EPI_ISL_959767 to 959770, EPI_ISL_959777 to 959796, EPI_ISL_959811 to 959832, EPI_ISL_959850, EPI_ISL_1001171 to 1001221			
see above	National Virus Reference Laboratory	National Virus Reference Laboratory	Aditi Chaturvedi; Cillian F De Gascun; Dana Alalwan; Daniel Hare; Gabriel Gonzalez; Jonathan Dean; Michael Carr; Suzie Coughlan; Zoe Yandle
EPI_ISL_493149 to 493159, EPI_ISL_493161, EPI_ISL_493163 to 493186, EPI_ISL_493188 to 493189	National Virus Resource Center, Chinese Academy of Sciences, Wuhan 430071, China	Computational Virology Group, Center for Bacteria and Viruses Resources and Bioinformation, Wuhan Institute of Virology, Chinese Academy of SciencesWuhan 430071, China	Di Liu; Hongping Wei; Jianjun Chen; Jin Xiong; Yi Huang; Yi Yan
EPI_ISL_444994 to 445000	Naval Health Research Center	Naval Medical Research Center Biological Defense Research Directorate	Adrian Paskey; Chris Myers; Dessiree Pena-Gomez; Ewell Hollis; Kimberly Bishop-Lilly; Kyle Long; Logan Voegtly; Melinda Balansay-Ames; Nathaniel Christy; Regina Cer; Roger Pan
EPI_ISL_491907 to 491931, EPI_ISL_491942 to 491943	Naval Infectious Diseases Diagnostic Laboratory	Naval Medical Research Center Biological Defense Research Directorate	Adrian Paskey; Dessiree Pena-Gomez; Francisco Malgon Bautista; Hua Wei Chen; Kimberly Bishop-Lilly; Kyle Long; Lindsay Glang; Logan Voegtly; Mark Simons; Megan Schilling; Regina Cer; Victor Sugiharto
EPI_ISL_514128	Navy and Marine Corps Public Health Center	Centers for Disease Control and Prevention	Anna Montmayeur; Anna Uehara; Clinton R. Paden; Haibin Wang; Jing Zhang; Krista Queen; Rachel Marine; Suxiang Tong; Yan Li; Ying Tao
EPI_ISL_514062 to 514075, EPI_ISL_514077 to 514092	Navy and Marine Corps Public Health Center	Pathogen Discovery, Respiratory Viruses Branch, Division of Viral Diseases, Centers for Disease Control and Prevention	Anna Montmayeur; Anna Uehara; Clinton R. Paden; Haibin Wang; Jing Zhang; Krista Queen; Rachel Marine; Suxiang Tong; Yan Li; Ying Tao
EPI_ISL_466652 to 466653, EPI_ISL_466655, EPI_ISL_466658, EPI_ISL_466661, EPI_ISL_466665 to 466672, EPI_ISL_466675, EPI_ISL_466677 to 466678, EPI_ISL_466680, EPI_ISL_475176, EPI_ISL_475178, EPI_ISL_475180, EPI_ISL_475184 to 475192, EPI_ISL_475194 to 475198, EPI_ISL_475200 to 475201, EPI_ISL_475203 to 475205, EPI_ISL_475208 to 475211, EPI_ISL_475213 to 475217, EPI_ISL_475219 to 475222, EPI_ISL_475225, EPI_ISL_475227 to 475229, EPI_ISL_475231 to 475233, EPI_ISL_475236 to 475237, EPI_ISL_732820 to 732826			
see above	Nebraska Public Health Laboratory	UNMC COVID-19 Response Team	UNMC COVID-19 Response Team
EPI_ISL_766938	Nevada State Health Lab	Nevada State Health Lab	Andrew Gorzalski
EPI_ISL_514673, EPI_ISL_515293 to 515297, EPI_ISL_515299 to 515309, EPI_ISL_515311 to 515322, EPI_ISL_515324 to 515325, EPI_ISL_515327 to 515334, EPI_ISL_515336 to 515342, EPI_ISL_515344 to 515359, EPI_ISL_515361 to 515379, EPI_ISL_515381 to 515382, EPI_ISL_515384 to 515394, EPI_ISL_515396 to 515399, EPI_ISL_515401, EPI_ISL_515403 to 515407, EPI_ISL_515409 to 515434, EPI_ISL_515436 to 515457, EPI_ISL_515459 to 515462			
see above	Nevada State Public Health Laboratory	Nevada State Public Health Laboratory	Andrew Gorzalski; Chris Laverdure; Cyprian Rosetto; Cyprian Rossetto; David Jackson; Heather Kerwin; Joel R. Sevinsky; Natalie Crawford; Paul Hartley; Richard Tillet; Richard Tillett; Stephanie Van Hooser; Subhash C. Verma; and Mark Pandori
EPI_ISL_700442, EPI_ISL_700444	New Horizon Clinic wc NZC	NHLS/UCT	Arash Iranzadeh; Bruna Galvao; Carolyn Williamson; Deelan Doolabh; Diana Hardie; Houriiyah Tegally; Innocent Mudau; Kruger Marais; Lynn Tyers; Marvin Hsiao; Stephen Korsman

EPI_ISL_960116	New Horizon Clinic wc NZC	National Health Laboratory Service/UCT	Arash Iranzadeh; Bruna Galvao; Carolyn Williamson; Deelan Doolabh; Diana Hardie; Innocent Mudau; Kruger Marais; Lynn Tyers; Marvin Hsiao; Stephen Korsman
EPI_ISL_696468	New Horizon Clinic wc NZC & NHLS/UCT	KRISP, KZN Research Innovation and Sequencing Platform	Arash Iranzadeh; Bruna Galvao; Carolyn Williamson; Deelan Doolabh; Diana Hardie; Emanuel James San; Houriiyah Tegally; Innocent Mudau; Jennifer Giandhari; Kruger Marais; Lynn Tyers; Marvin Hsiao; Stephen Korsman; Sureshnee Pillay; Tulio de Oliveira
EPI_ISL_510882 to 510888, EPI_ISL_535264 to 535266, EPI_ISL_535268 to 535274, EPI_ISL_535276, EPI_ISL_535278 to 535288, EPI_ISL_535291 to 535293, EPI_ISL_535295, EPI_ISL_535297 to 535303, EPI_ISL_542014, EPI_ISL_542016 to 542027, EPI_ISL_542029 to 542033, EPI_ISL_542035, EPI_ISL_542038 to 542063, EPI_ISL_542065 to 542074, EPI_ISL_542077 to 542080, EPI_ISL_542082 to 542084, EPI_ISL_542086 to 542095, EPI_ISL_542097, EPI_ISL_569632 to 569663, EPI_ISL_574260 to 574287, EPI_ISL_574291, EPI_ISL_602162 to 602189, EPI_ISL_602191 to 602197, EPI_ISL_602199 to 602207, EPI_ISL_632823 to 632828, EPI_ISL_632831 to 632832, EPI_ISL_632834, EPI_ISL_632836 to 632837, EPI_ISL_632839, EPI_ISL_632841, EPI_ISL_632843 to 632845, EPI_ISL_644587 to 644609, EPI_ISL_732704 to 732708, EPI_ISL_732710 to 732712, EPI_ISL_732714 to 732716, EPI_ISL_732718 to 732740, EPI_ISL_732742 to 732744, EPI_ISL_732746 to 732747, EPI_ISL_751804 to 751808, EPI_ISL_751810 to 751846, EPI_ISL_766895 to 766896, EPI_ISL_766899 to 766923, EPI_ISL_767051 to 767088, EPI_ISL_824554, EPI_ISL_831909, EPI_ISL_1038969 to 1038970			
see above	New Mexico Department of Health Scientific Laboratory	New Mexico Department of Health Scientific Laboratory	Anastacia Griego-Fisher; D'Eldra Malone; D'eldra Malone; Ellie Johnson; Jennifer Benoit
EPI_ISL_467529, EPI_ISL_467536, EPI_ISL_467545, EPI_ISL_467551 to 467552, EPI_ISL_467563 to 467564, EPI_ISL_467572, EPI_ISL_467577, EPI_ISL_467583, EPI_ISL_467590 to 467591, EPI_ISL_467594, EPI_ISL_467596, EPI_ISL_467598 to 467600, EPI_ISL_467604, EPI_ISL_467608, EPI_ISL_467610 to 467612, EPI_ISL_467624, EPI_ISL_467630, EPI_ISL_467632 to 467634, EPI_ISL_467644, EPI_ISL_467651 to 467654, EPI_ISL_467661, EPI_ISL_467663, EPI_ISL_485872, EPI_ISL_507986 to 507987, EPI_ISL_507991, EPI_ISL_507995, EPI_ISL_508002, EPI_ISL_508013 to 508014, EPI_ISL_508018, EPI_ISL_508026, EPI_ISL_508029, EPI_ISL_508057, EPI_ISL_508063, EPI_ISL_508066, EPI_ISL_508091, EPI_ISL_508093, EPI_ISL_508097, EPI_ISL_508099 to 508101, EPI_ISL_610268 to 610281			
see above	New Mexico Department of Health Scientific Laboratory Division	Center for Global Health, University of New Mexico Health Sciences Center	Darrell Dinwiddie; Daryl Domman; Joseph Hicks; Kurt Schwalm; Michael Edwards; Twila Kunde
EPI_ISL_596447 to 596448, EPI_ISL_602208 to 602209, EPI_ISL_602278 to 602281	New Mexico Department of Health Scientific Laboratory Division	Los Alamos National Laboratory Bioscience Division	Alina Deshpande; Cheryl Gleasner; Chien-Chi Lo; Joseph Hicks; Kim McMurry; Michael Edwards; Migun Shakya; Patrick Chain; Twila Kunde
EPI_ISL_1010703, EPI_ISL_1010712 to 1010713, EPI_ISL_1033027	New South Wales Health Pathology Royal Prince Alfred Hospital	Microbiology RPAH	Au, J.; Bull, R.; Deveson, I.; Foster, C.; Rawlinson, W.; Ruiz Silva, M.; Van Hal, S.
EPI_ISL_631514, EPI_ISL_632055 to 632056	New York Community Hospital	New York City Public Health Laboratory	Jade Wang; et al.
EPI_ISL_1016265	New York Presbyterian Hospital	Wadsworth Center, New York State Department of Health	Alexis Russel; Daryl M. Lamson; Erasmus Schneider; Erica Lasek-Nesselquist; John Kelly; Jonathan Plitnick; Kirsten St. George; Matthew Shudt; Melissa A Leisner; Navjot Singh
EPI_ISL_631509, EPI_ISL_631533, EPI_ISL_631536, EPI_ISL_631539 to 631540, EPI_ISL_631542, EPI_ISL_632038, EPI_ISL_632041 to 632042, EPI_ISL_632045, EPI_ISL_632047			
see above	New York Presbyterian Lower Manhattan	New York City Public Health Laboratory	Jade Wang; et al.
EPI_ISL_631530, EPI_ISL_631547, EPI_ISL_631568 to 631569, EPI_ISL_631861, EPI_ISL_632040, EPI_ISL_632049, EPI_ISL_632052 to 632053, EPI_ISL_632063 to 632064, EPI_ISL_632070 to 632071			
see above	New York Presbyterian Queens	New York City Public Health Laboratory	Jade Wang; et al.
EPI_ISL_631517 to 631519, EPI_ISL_631544 to 631545, EPI_ISL_631548, EPI_ISL_631566 to 631567, EPI_ISL_632046, EPI_ISL_632048, EPI_ISL_632054			
see above	New York Presbyterian-Brooklyn Methodist Hospital	New York City Public Health Laboratory	Jade Wang; et al.
EPI_ISL_631510 to 631511, EPI_ISL_631860	New York Presbyterian/ Weill Cornell Medical Center	New York City Public Health Laboratory	Jade Wang; et al.
EPI_ISL_631502, EPI_ISL_632057	New York-Presbyterian-Columbia University Medical Center	New York City Public Health Laboratory	Jade Wang; et al.
EPI_ISL_427526 to 427618	NewYork-Presbyterian & Mason Lab	Mason Lab	Alon Shaiber; Arryn Craney; Benjamin Young; Cem Meydan; Chandrima Bhattacharya; Christopher E. Mason; Christopher Mozsary; Craig D. Westover; Daniel J. Butler; David Danko; Dmitry Meleshko; Dong Xu; Ebrahim Afshinnekoo; Fritz J. Sedlazeck; Hanna Rennert; Iman Hajirasouliha; Jenny Xiang; Joel Rosiene; John Sipley; Jonathan Foox; Justyna Gawrys; Krista Ryon; Lars F. Westblade; Lin Cong; Marcin Imielinski; Maria Sierra; Massimo Loda; Matthew MacKay; Melissa Cushing; Mirella Salvatore; Nikolay A. Ivanov; Phyllis Ruggiero; Priya Velu; Shawn Levy
EPI_ISL_516934, EPI_ISL_516938	Nicolae Testemitanu State University of Medicine and Pharmacy	International Centre for Genetic Engineering and Biotechnology (ICGEB) and ARGO Open Lab Platform for Genome Sequencing	Dal Monego S; Licastro D; Marcello A; Rajasekharan S; Ulinici M
EPI_ISL_455422, EPI_ISL_455426	Nigeria Centre for Disease Control	African Centre of Excellence for Genomics of Infectious Diseases (ACEGID), Redeemer's University, Ede, Osun State, Nigeria	Ajogbasile F.V.; Folarin O.A.; Happi C.T.; Ihekweazu C.; Kayode A.; Oguzie J.; Olawoye I.; Olumade T.; Oluniyi P.E.; Uwanibe J.
EPI_ISL_872625, EPI_ISL_906278 to 906279, EPI_ISL_906281, EPI_ISL_941284 to 941286, EPI_ISL_941293, EPI_ISL_941297	Nigeria Centre for Disease Control (NCDC)	African Centre of Excellence for Genomics of Infectious Diseases (ACEGID), Redeemer's University	Oluniyi P.E. et al
EPI_ISL_455362, EPI_ISL_455412 to 455413, EPI_ISL_455419, EPI_ISL_455423 to 455424, EPI_ISL_455429, EPI_ISL_455431, EPI_ISL_487091, EPI_ISL_487099, EPI_ISL_487101 to 487103, EPI_ISL_487105 to 487110, EPI_ISL_487112, EPI_ISL_527873 to 527874, EPI_ISL_527876, EPI_ISL_527878 to 527884, EPI_ISL_527887 to 527893, EPI_ISL_527898, EPI_ISL_527901, EPI_ISL_527903 to 527905, EPI_ISL_527910 to 527912, EPI_ISL_527914 to 527915, EPI_ISL_729920, EPI_ISL_729923 to 729926, EPI_ISL_729928 to 729932, EPI_ISL_729935, EPI_ISL_729937 to 729948, EPI_ISL_729950 to 729957, EPI_ISL_729959 to 729962, EPI_ISL_729964, EPI_ISL_729966 to 729970, EPI_ISL_729972 to 729980, EPI_ISL_729982, EPI_ISL_729985 to 729989, EPI_ISL_729992 to 729995, EPI_ISL_729999, EPI_ISL_730004, EPI_ISL_730006 to 730007, EPI_ISL_730009 to 730010, EPI_ISL_730012 to 730013, EPI_ISL_730015, EPI_ISL_730017, EPI_ISL_730019 to 730021, EPI_ISL_730024, EPI_ISL_730028 to 730029, EPI_ISL_730031 to 730036, EPI_ISL_730038 to 730039, EPI_ISL_730042, EPI_ISL_730044, EPI_ISL_730048			
see above	Nigeria Centre for Disease Control (NCDC)	African Centre of Excellence for Genomics of Infectious Diseases (ACEGID), Redeemer's University, Ede, Osun State, Nigeria	Ajogbasile F.V.; Folarin O.A.; Happi C.T.; Ihekweazu C.; Kayode A.; Oguzie J.; Olawoye I.; Olumade T.; Oluniyi P.E.; Oluniyi P.E. et al; Uwanibe J.
EPI_ISL_487113	Nigeria Centre for Disease Control (NCDC)	Redeemer's University, ACEGID	Ajogbasile F.V.; Folarin O.A.; Happi C.T.; Ihekweazu C.; Kayode A.; Oguzie J.; Olawoye I.; Olumade T.; Oluniyi P.E.; Uwanibe J.
EPI_ISL_977549 to 977559	Nigeria Centre of Disease Control (NCDC)	African Centre of Excellence for Genomics of Infectious Diseases (ACEGID), Redeemer's University	Olawoye I. B. et al
EPI_ISL_462992	Nigerian Institute of Medical Research	Nigerian Institute of Medical Research	A.P.; Abosede, O.; Adegbola, R.; Adesegun, A.; Ahmed, R.; Amoo; C.K.; F.A.; Ige; J.O.; Liboro, G.; O.B.; O.S.; Odewale, E.; Okwuraiwe; Omilabu; Onwuamah; Oyefolu, A.; S. and Audu, R.; Saibu; Salako, B.; Salu; Sokei, J.
EPI_ISL_479913 to 479924	Niigata City Public Health Research Institute	Pathogen Genomics Center, National Institute of Infectious Diseases	Hajime Kamiya; Kentaro Itokawa; Makoto Kuroda; Masanori Hashino; Motoi Suzuki; Rina Tanaka; Tsuyoshi Sekizuka; Yurie Takahashi
EPI_ISL_479869, EPI_ISL_479902, EPI_ISL_685297 to 685310, EPI_ISL_687699 to 687717, EPI_ISL_692051 to 692052	Niigata Prefectural Institute of Public Health and Environmental Sciences	Pathogen Genomics Center, National Institute of Infectious Diseases	Hajime Kamiya; Kentaro Itokawa; Makoto Kuroda; Masanori Hashino; Motoi Suzuki; Reiko Arai; Rina Tanaka; Tsuyoshi Sekizuka
EPI_ISL_420151	Nordland Hospital - Bodo, Laboratory Department, Molecular Biology Unit	Norwegian Institute of Public Health, Department of Virology	Hilde Elshaug; Kamilla Heddeland Instefjord; Karoline Bragstad; Kathrine Stene-Johansen; Olav Hungnes
EPI_ISL_635142, EPI_ISL_635145, EPI_ISL_635182, EPI_ISL_668398, EPI_ISL_668419, EPI_ISL_668422 to 668423, EPI_ISL_668429 to 668432, EPI_ISL_668434, EPI_ISL_708097 to 708105, EPI_ISL_964956 to 964957			
see above	Nordland Hospital - Bodo, Laboratory Department, Molecular Biology Unit	Norwegian Institute of Public Health, Department of Virology	Atiya R Ali; Hilde Elshaug; Hilde Vollan; Ignacio Garcia Llorente; Kamilla Heddeland Instefjord; Karoline Bragstad; Kathrine Stene-Johansen; Marie Paulsen Madsen; Olav Hungnes; Rasmus Riis Kopperud; Serina B Engebretsen
EPI_ISL_534239 to 534243	Norra Alvsborgs lanssjukhus	The Public Health Agency of Sweden	Anna Risberg; Anna-Malin Linde; Karin Tegmark-Wisell; Maria Lind Karlberg; Mattias Haukland; Mia Brytting; Olov Svartstrom; Oskar Karlsson Lindsjo; Petra Edquist; Reza Advani; Sandra Broddesson
EPI_ISL_456294, EPI_ISL_456395 to 456397, EPI_ISL_548030, EPI_ISL_548045, EPI_ISL_548047 to 548048, EPI_ISL_548057, EPI_ISL_548067, EPI_ISL_548075, EPI_ISL_548100, EPI_ISL_579406 to 579407, EPI_ISL_1016866			
see above	North Shore Hospital	Institute of Environmental Science and Research (ESR)	Anja Werno; Antje van der Linden; Arlo Upton; Chris Mansell; David Hammer; Dragana Drinkovic; Erasmus Smit; Gary McAuliffe; Hana Sofia Andersson; Hermes Perez; James Ussher; Jill Sherwood; Jing Wang; Joep de Ligt; Josh Freeman; Julia Howard; Juliet Elvy; Lauren Jelly; Mary DeAlmeida; Matt Blakiston; Matt Storey; Matthew Rogers; Max Bloomfield; Michael Addidle; Michelle Balm; Muhammad Faisal; Nikki Freed; Olin Silander; Sally Roberts; Sarah Jefferies; Sharmini Muttaiyah; Susan Morpeth; Susan Taylor; Timothy Blackmore; Vani Sathyendran; Veronica Playle; Virginia Hope; Xiaoyun Ren

EPI_ISL_765819 to 765823	North Shore Medical Center	Infectious Disease Program, Broad Institute of Harvard and MIT	A.E.; Adams, G.; Anahtar, M.; B.L.; B.W.; Bauer, M.; Birren; Branda, J.; Carter, A.; Cerrato, F.; Chaluvadi, S.; Chapman; Cusick, C.; D.J.; DeRuff, K.; E. and Sabeti; Flowers, K.; Gallagher, G.; Gladden-Young, A.; Gnirke, A.; Harris, J.; J.E.; K.J.; LaRocque, R.; Lagerborg, K.; Lemieux; Lin; Loreth, C.; MacInnis; Neumann, A.; Normandin, E.; P.C.; Park; Pierce, V.; Reilly, S.; Rosenberg; Rudy, M.; Ryan, E.; S.B.; Shaw, B.; Siddle; Slater, D.; Smole, S.; Tomkins-Tinch, C.; Turbett, S.
EPI_ISL_482138	North West London Pathology, Imperial College Healthcare NHS Trust	Wellcome Sanger Institute for the COVID-19 Genomics UK (COG-UK) Consortium	Aileen Rowan; Alison Holmes; Anjna Badhan; Carolina Herrera and Alex Alderton; Cordelia Langford; David K. Jackson; David Muir; Dominic Kwiatkowski; Ewan Harrison; Frankie Bolt; Graham Taylor; Ian Johnston; James Price; John Sillitoe on behalf of the Wellcome Sanger Institute COVID-19 Surveillance Team; Ling Li; Paul Randell; Roberto Amato; Sonia Goncalves
EPI_ISL_516831 to 516836, EPI_ISL_516841 to 516844, EPI_ISL_516846, EPI_ISL_516848, EPI_ISL_516852, EPI_ISL_516857 to 516858, EPI_ISL_516863 to 516866, EPI_ISL_516868, EPI_ISL_516870, EPI_ISL_516873 to 516874, EPI_ISL_516876 to 516878, EPI_ISL_516880, EPI_ISL_516883, EPI_ISL_524619 to 524625, EPI_ISL_524628 to 524634, EPI_ISL_524636, EPI_ISL_524638, EPI_ISL_524640 to 524643, EPI_ISL_524645 to 524649, EPI_ISL_524651 to 524654, EPI_ISL_524656, EPI_ISL_524658 to 524659, EPI_ISL_524663, EPI_ISL_524665, EPI_ISL_524668 to 524671, EPI_ISL_524673 to 524674, EPI_ISL_524676, EPI_ISL_524680, EPI_ISL_524682 to 524684, EPI_ISL_524686, EPI_ISL_524688 to 524694, EPI_ISL_524696, EPI_ISL_524698 to 524700, EPI_ISL_524702 to 524712			
see above	North West London Pathology, Imperial College Healthcare NHS Trust	Wellcome Sanger Institute for the COVID-19 Genomics UK (COG-UK) consortium	Aileen Rowan; Alison Holmes; Anjna Badhan; Carolina Herrera and Alex Alderton; Cordelia Langford; David K. Jackson; David Muir; Dominic Kwiatkowski; Ewan Harrison; Frankie Bolt; Graham Taylor; Ian Johnston; James Price; John Sillitoe on behalf of the Wellcome Sanger Institute COVID-19 Surveillance Team (http://www.sanger.ac.uk/covid-team); Ling Li; Paul Randell; Roberto Amato; Sonia Goncalves
EPI_ISL_453489, EPI_ISL_453492, EPI_ISL_453499, EPI_ISL_453507, EPI_ISL_453509, EPI_ISL_453534, EPI_ISL_453538, EPI_ISL_453542, EPI_ISL_453544, EPI_ISL_457303, EPI_ISL_457305, EPI_ISL_457311 to 457312, EPI_ISL_457316, EPI_ISL_457319 to 457320, EPI_ISL_457325, EPI_ISL_461791 to 461793, EPI_ISL_461795, EPI_ISL_461797, EPI_ISL_461801, EPI_ISL_472228, EPI_ISL_472249, EPI_ISL_472255 to 472256, EPI_ISL_478513, EPI_ISL_478515 to 478517, EPI_ISL_478519 to 478522, EPI_ISL_478524 to 478525, EPI_ISL_478527 to 478532, EPI_ISL_478536 to 478537, EPI_ISL_478539, EPI_ISL_478541, EPI_ISL_478543 to 478544, EPI_ISL_478546 to 478551, EPI_ISL_478553, EPI_ISL_478555 to 478556, EPI_ISL_478558 to 478559, EPI_ISL_478561, EPI_ISL_478564 to 478569, EPI_ISL_478571, EPI_ISL_478573, EPI_ISL_478575 to 478588, EPI_ISL_478591 to 478594, EPI_ISL_478596, EPI_ISL_478608, EPI_ISL_478614, EPI_ISL_478616, EPI_ISL_478619, EPI_ISL_478621 to 478624, EPI_ISL_478626 to 478628, EPI_ISL_478630, EPI_ISL_478632, EPI_ISL_478636 to 478638, EPI_ISL_478645, EPI_ISL_478648, EPI_ISL_478652, EPI_ISL_478655, EPI_ISL_478657 to 478658, EPI_ISL_478661 to 478662, EPI_ISL_484264 to 484265, EPI_ISL_484267, EPI_ISL_484269 to 484271, EPI_ISL_484273 to 484277, EPI_ISL_484279 to 484280, EPI_ISL_484282 to 484283, EPI_ISL_484288 to 484290, EPI_ISL_484292 to 484293, EPI_ISL_484295, EPI_ISL_484300, EPI_ISL_484302, EPI_ISL_484304 to 484307, EPI_ISL_484309 to 484310, EPI_ISL_484312 to 484313, EPI_ISL_484315 to 484318, EPI_ISL_484320 to 484327, EPI_ISL_490444, EPI_ISL_490449, EPI_ISL_490451 to 490453, EPI_ISL_490461, EPI_ISL_490467, EPI_ISL_490469, EPI_ISL_490472 to 490474, EPI_ISL_490478 to 490479, EPI_ISL_490481 to 490482, EPI_ISL_490485, EPI_ISL_490487, EPI_ISL_490492, EPI_ISL_493452 to 493456, EPI_ISL_493458 to 493461, EPI_ISL_493463 to 493466, EPI_ISL_493468 to 493470, EPI_ISL_493472, EPI_ISL_493476 to 493478, EPI_ISL_493480 to 493482, EPI_ISL_493484 to 493493, EPI_ISL_493496 to 493502, EPI_ISL_493504 to 493506, EPI_ISL_493508 to 493513, EPI_ISL_493516 to 493519, EPI_ISL_493521 to 493523, EPI_ISL_493525 to 493527, EPI_ISL_493529 to 493530, EPI_ISL_493532 to 493537, EPI_ISL_493539 to 493540, EPI_ISL_499777 to 499807, EPI_ISL_507110 to 507114, EPI_ISL_507116 to 507118, EPI_ISL_507120 to 507121, EPI_ISL_507123 to 507127, EPI_ISL_507129, EPI_ISL_512348 to 512351, EPI_ISL_512353 to 512354, EPI_ISL_512356, EPI_ISL_512358 to 512361, EPI_ISL_572712 to 572713, EPI_ISL_572729, EPI_ISL_572942, EPI_ISL_572950, EPI_ISL_573266 to 573413, EPI_ISL_573760 to 573761, EPI_ISL_573799, EPI_ISL_573871 to 573872, EPI_ISL_576986 to 576989, EPI_ISL_576991 to 577004, EPI_ISL_577006 to 577007, EPI_ISL_577010 to 577014, EPI_ISL_577016 to 577017, EPI_ISL_577019 to 577036, EPI_ISL_577038, EPI_ISL_584681 to 584691, EPI_ISL_584693 to 584694, EPI_ISL_584696 to 584699, EPI_ISL_584701 to 584702, EPI_ISL_584704 to 584705, EPI_ISL_584707, EPI_ISL_584713, EPI_ISL_584847 to 584848, EPI_ISL_584858 to 584862, EPI_ISL_584865 to 584866, EPI_ISL_584869 to 584873, EPI_ISL_584875 to 584877, EPI_ISL_584879 to 584881, EPI_ISL_585504, EPI_ISL_585506 to 585511, EPI_ISL_585515 to 585517, EPI_ISL_585519, EPI_ISL_585524, EPI_ISL_585529, EPI_ISL_585531, EPI_ISL_594987 to 594995, EPI_ISL_594997 to 595000, EPI_ISL_595002 to 595003, EPI_ISL_595005 to 595006, EPI_ISL_595009 to 595012, EPI_ISL_595014, EPI_ISL_595016 to 595017, EPI_ISL_595021, EPI_ISL_595023, EPI_ISL_595025, EPI_ISL_595031, EPI_ISL_595033, EPI_ISL_595035, EPI_ISL_595037 to 595038, EPI_ISL_595041 to 595045, EPI_ISL_612134, EPI_ISL_612523 to 612545, EPI_ISL_612549 to 612570, EPI_ISL_638555 to 638580, EPI_ISL_652111 to 652116, EPI_ISL_652337 to 652347, EPI_ISL_664856, EPI_ISL_665651, EPI_ISL_665787, EPI_ISL_665868 to 665875, EPI_ISL_665964 to 665970, EPI_ISL_665983 to 666021, EPI_ISL_666084 to 666106, EPI_ISL_666591 to 666592, EPI_ISL_705486, EPI_ISL_706276 to 706286, EPI_ISL_706288 to 706314, EPI_ISL_706316 to 706339, EPI_ISL_706341 to 706354, EPI_ISL_723558 to 723559, EPI_ISL_723561 to 723563, EPI_ISL_723565 to 723569, EPI_ISL_723571 to 723572, EPI_ISL_723574, EPI_ISL_723576, EPI_ISL_723578 to 723579, EPI_ISL_723581 to 723587, EPI_ISL_723589 to 723590, EPI_ISL_723593 to 723594, EPI_ISL_723596 to 723597, EPI_ISL_723600 to 723601, EPI_ISL_723603 to 723607, EPI_ISL_723610, EPI_ISL_723612, EPI_ISL_723615, EPI_ISL_723618 to 723622, EPI_ISL_723624, EPI_ISL_723626, EPI_ISL_723629 to 723630, EPI_ISL_724067, EPI_ISL_724073 to 724076, EPI_ISL_724078 to 724079, EPI_ISL_724083, EPI_ISL_724086, EPI_ISL_724088 to 724091, EPI_ISL_724094, EPI_ISL_724097, EPI_ISL_724100, EPI_ISL_724102 to 724105, EPI_ISL_724107 to 724108, EPI_ISL_724112, EPI_ISL_724118 to 724120, EPI_ISL_724123, EPI_ISL_724856 to 724857, EPI_ISL_724859 to 724860, EPI_ISL_724863 to 724864, EPI_ISL_724868 to 724869, EPI_ISL_724872 to 724874, EPI_ISL_724876, EPI_ISL_724880, EPI_ISL_724882 to 724883, EPI_ISL_724887 to 724892, EPI_ISL_724894 to 724896, EPI_ISL_724910, EPI_ISL_724915, EPI_ISL_724918, EPI_ISL_724924 to 724926, EPI_ISL_724928, EPI_ISL_724932, EPI_ISL_724936, EPI_ISL_724938 to 724942, EPI_ISL_724949 to 724951, EPI_ISL_724954 to 724955, EPI_ISL_724957 to 724959, EPI_ISL_724967, EPI_ISL_725004 to 725009, EPI_ISL_725011 to 725012, EPI_ISL_725014 to 725016, EPI_ISL_725020 to 725023, EPI_ISL_725025 to 725027, EPI_ISL_741421, EPI_ISL_741423, EPI_ISL_741426 to 741430, EPI_ISL_741432 to 741433, EPI_ISL_741436, EPI_ISL_741438, EPI_ISL_741440 to 741445, EPI_ISL_741447 to 741448, EPI_ISL_741451 to 741452, EPI_ISL_839511, EPI_ISL_839523, EPI_ISL_839525 to 839526, EPI_ISL_839548, EPI_ISL_839556, EPI_ISL_839558, EPI_ISL_839569, EPI_ISL_839587, EPI_ISL_839688, EPI_ISL_839699, EPI_ISL_839704, EPI_ISL_839710, EPI_ISL_839713, EPI_ISL_866425 to 866426, EPI_ISL_866428 to 866429, EPI_ISL_866431, EPI_ISL_866435, EPI_ISL_866457 to 866459, EPI_ISL_866461, EPI_ISL_866534, EPI_ISL_921147 to 921148, EPI_ISL_921156, EPI_ISL_921176, EPI_ISL_921178, EPI_ISL_921182, EPI_ISL_921214 to 921215, EPI_ISL_921225, EPI_ISL_921231, EPI_ISL_921235, EPI_ISL_921239, EPI_ISL_921241 to 921244, EPI_ISL_921246, EPI_ISL_921253 to 921254, EPI_ISL_921260 to 921261, EPI_ISL_921263, EPI_ISL_921267 to 921268, EPI_ISL_921270, EPI_ISL_921280 to 921282, EPI_ISL_921286, EPI_ISL_921288 to 921289, EPI_ISL_921296, EPI_ISL_921304 to 921305, EPI_ISL_921312 to 921314, EPI_ISL_921319, EPI_ISL_921326, EPI_ISL_921332, EPI_ISL_921334 to 921335, EPI_ISL_921337, EPI_ISL_921340 to 921341, EPI_ISL_921362, EPI_ISL_921364, EPI_ISL_921366, EPI_ISL_921370, EPI_ISL_921372, EPI_ISL_921377 to 921378, EPI_ISL_921384, EPI_ISL_921390 to 921392, EPI_ISL_921397, EPI_ISL_921402, EPI_ISL_921405 to 921406, EPI_ISL_921410, EPI_ISL_921413, EPI_ISL_921419, EPI_ISL_921425 to 921426, EPI_ISL_921433 to 921434, EPI_ISL_921446, EPI_ISL_921457, EPI_ISL_921463, EPI_ISL_921465 to 921466, EPI_ISL_921473, EPI_ISL_921480, EPI_ISL_921485, EPI_ISL_921495 to 921496, EPI_ISL_921507, EPI_ISL_921509, EPI_ISL_921517, EPI_ISL_921546, EPI_ISL_921553 to 921554, EPI_ISL_921562, EPI_ISL_921566, EPI_ISL_921568, EPI_ISL_921571 to 921572, EPI_ISL_921574 to 921578, EPI_ISL_921590, EPI_ISL_921592 to 921595, EPI_ISL_921607 to 921609, EPI_ISL_921611, EPI_ISL_921614, EPI_ISL_921618, EPI_ISL_921625, EPI_ISL_921627, EPI_ISL_921632, EPI_ISL_921636, EPI_ISL_921640, EPI_ISL_921644, EPI_ISL_921651, EPI_ISL_921653, EPI_ISL_921657 to 921658, EPI_ISL_921660, EPI_ISL_921667 to 921668, EPI_ISL_921710, EPI_ISL_921718, EPI_ISL_921720, EPI_ISL_921728, EPI_ISL_921731, EPI_ISL_921736, EPI_ISL_921738, EPI_ISL_950233, EPI_ISL_950235 to 950236, EPI_ISL_950251, EPI_ISL_950256 to 950257, EPI_ISL_950260, EPI_ISL_950262, EPI_ISL_950264, EPI_ISL_950266, EPI_ISL_950278, EPI_ISL_950280, EPI_ISL_950282, EPI_ISL_950287, EPI_ISL_950291 to 950293, EPI_ISL_950295 to 950296, EPI_ISL_950300 to 950305, EPI_ISL_950307 to 950308, EPI_ISL_950310, EPI_ISL_950314, EPI_ISL_950316 to 950324, EPI_ISL_950326 to 950329, EPI_ISL_950331 to 950336, EPI_ISL_950355, EPI_ISL_950361, EPI_ISL_950363 to 950364, EPI_ISL_950369 to 950370, EPI_ISL_950373, EPI_ISL_950375, EPI_ISL_950381 to 950382, EPI_ISL_950385, EPI_ISL_950387, EPI_ISL_950389 to 950390, EPI_ISL_950392 to 950393, EPI_ISL_950404 to 950406, EPI_ISL_950411, EPI_ISL_950414, EPI_ISL_950436, EPI_ISL_950439, EPI_ISL_950441, EPI_ISL_950445, EPI_ISL_950447 to 950450, EPI_ISL_950454, EPI_ISL_950460 to 950461, EPI_ISL_950464, EPI_ISL_950466, EPI_ISL_950468 to 950470, EPI_ISL_950473 to 950475, EPI_ISL_950478 to 950484, EPI_ISL_950486 to 950488, EPI_ISL_950492, EPI_ISL_950494, EPI_ISL_950497 to 950498, EPI_ISL_950502, EPI_ISL_950506, EPI_ISL_950510, EPI_ISL_950512, EPI_ISL_950514, EPI_ISL_950517 to 950521, EPI_ISL_950523, EPI_ISL_950525 to 950527, EPI_ISL_950529, EPI_ISL_950531, EPI_ISL_950536 to 950539, EPI_ISL_950541 to 950543, EPI_ISL_998314, EPI_ISL_998316 to 998318, EPI_ISL_998325, EPI_ISL_998327, EPI_ISL_998333, EPI_ISL_998335, EPI_ISL_998337, EPI_ISL_998341 to 998344, EPI_ISL_998347 to 998351, EPI_ISL_998363 to 998370, EPI_ISL_998375, EPI_ISL_998377, EPI_ISL_998380 to 998389, EPI_ISL_998391, EPI_ISL_998393, EPI_ISL_998395, EPI_ISL_998399 to 998402, EPI_ISL_998405, EPI_ISL_998407, EPI_ISL_998409 to 998410, EPI_ISL_998413, EPI_ISL_998434, EPI_ISL_998436 to 998439, EPI_ISL_998441 to 998466, EPI_ISL_998470, EPI_ISL_998473 to 998475, EPI_ISL_998477 to 998479, EPI_ISL_998482 to 998483, EPI_ISL_998485 to 998489, EPI_ISL_998491 to 998492, EPI_ISL_998494 to 998495, EPI_ISL_998497, EPI_ISL_998501 to 998503, EPI_ISL_998517 to 998520, EPI_ISL_998526, EPI_ISL_998529, EPI_ISL_998534, EPI_ISL_998537, EPI_ISL_998539, EPI_ISL_998542, EPI_ISL_998555, EPI_ISL_998559, EPI_ISL_998563, EPI_ISL_998565, EPI_ISL_998567 to 998569, EPI_ISL_998573 to 998575, EPI_ISL_998577 to 998578, EPI_ISL_998581 to 998583, EPI_ISL_998586, EPI_ISL_998596 to 998599, EPI_ISL_998602, EPI_ISL_998605 to 998607, EPI_ISL_998609 to 998610, EPI_ISL_998612, EPI_ISL_998614 to 998615, EPI_ISL_998617, EPI_ISL_998620 to 998621, EPI_ISL_998624, EPI_ISL_998628, EPI_ISL_998639, EPI_ISL_998642, EPI_ISL_998645, EPI_ISL_998659, EPI_ISL_998671, EPI_ISL_998686, EPI_ISL_998691, EPI_ISL_998711 to 998712, EPI_ISL_998714 to 998715, EPI_ISL_998722, EPI_ISL_998724, EPI_ISL_998727 to 998735			
see above	Northumbria University / South Tees Hospitals NHS Foundation Trust / North Cumbria Integrated Care NHS Foundation Trust / North Tees and Hartlepool NHS Foundation Trust / Newcastle Hospitals NHS Foundation Trust	COVID-19 Genomics UK (COG-UK) Consortium	Andrew Nelson; Brendan Payne; Clive Graham; Darren L Smith; Debra Padgett; Edward Barton; Emma Swindells; Garren Scott; Gary Black; Gary Eltringham; Giles S Holt; Greg R Young; Jane Greenaway; Jennifer Collins; John Allan; Joshua Loh; Lynn Dover; Matthew Bashton; Mohammad A Tariq; Paul Baker; Sarah Essex; Steve Liggett; Wen C Yew; Yusri Taha
EPI_ISL_631505	Northwell Health-GoHealth Urgent Care 10025	New York City Public Health Laboratory	Jade Wang; et al.
EPI_ISL_832203 to 832204, EPI_ISL_1034278 to 1034279	Northwest Pathology	UW Virology Lab	Alexander Greninger; Hong Xie; Keith R Jerome; Lasata Shrestha; Meei-Li Huang; Michelle Lin; Pavitra Roychoudhury; Shah A Mohamed Bakhash
EPI_ISL_444521 to 444525, EPI_ISL_444527 to 444528, EPI_ISL_444530 to 444535, EPI_ISL_444537 to 444538, EPI_ISL_444540, EPI_ISL_444544 to 444547, EPI_ISL_444549 to 444550, EPI_ISL_444552 to 444553, EPI_ISL_444555 to 444556, EPI_ISL_444558, EPI_ISL_444560 to 444562, EPI_ISL_444564, EPI_ISL_444566 to 444581, EPI_ISL_444583, EPI_ISL_444585 to 444601, EPI_ISL_444603 to 444609, EPI_ISL_626359 to 626360, EPI_ISL_626364 to 626366, EPI_ISL_626368, EPI_ISL_626370 to 626379, EPI_ISL_626381 to 626410, EPI_ISL_626415, EPI_ISL_626431, EPI_ISL_626438 to 626439, EPI_ISL_626442 to 626443, EPI_ISL_626445 to 626453, EPI_ISL_626457 to 626461, EPI_ISL_626464 to 626470, EPI_ISL_626472 to 626473, EPI_ISL_626476, EPI_ISL_626478, EPI_ISL_626480, EPI_ISL_626485 to 626489, EPI_ISL_626492 to 626495, EPI_ISL_626497 to 626498, EPI_ISL_626502 to 626503, EPI_ISL_626505 to 626509, EPI_ISL_626511, EPI_ISL_626514 to 626515, EPI_ISL_626521 to 626522, EPI_ISL_626524 to 626527, EPI_ISL_626529, EPI_ISL_626532, EPI_ISL_626536, EPI_ISL_626538 to 626540, EPI_ISL_626542, EPI_ISL_825014			
see above	Northwestern Memorial Hospital	Ozer Lab	Alan R. Hauser; Chad J. Achenbach; Chao Qi; Egon A. Ozer; Hannah H. Nam; Judd F. Hultquist; Lacy M. Simons; Lawrence J. Jennings; Michael G. Ison; Ramon Lorenzo-Redondo; Scott C. Roberts
EPI_ISL_635066 to 635067, EPI_ISL_635074 to 635079, EPI_ISL_635178, EPI_ISL_635187 to 635188, EPI_ISL_668410 to 668411, EPI_ISL_668435 to 668437, EPI_ISL_708028 to 708029, EPI_ISL_708040, EPI_ISL_708049 to 708051, EPI_ISL_708140 to 708141, EPI_ISL_708169 to 708170, EPI_ISL_708180 to 708181, EPI_ISL_759981, EPI_ISL_759989, EPI_ISL_796654 to 796657, EPI_ISL_796659 to 796662, EPI_ISL_860209, EPI_ISL_962909, EPI_ISL_1013466, EPI_ISL_1013469			
see above	Norwegian Institute of Public Health, Department of Virology	Norwegian Institute of Public Health, Department of Virology	Atiya R Ali; Engebretsen Serina Beate Atiya R Ali; Garcia Llorente Ignacio; Hilde Elshaug; Hilde Vollan; Ignacio Garcia Llorente; Kamilla Heddeland Instefjord; Karoline Bragstad; Kathrine Stene-Johansen; Marie Paulsen Madsen; Olav Hungnes; Rasmus Riis Kopperud; Serina B Engebretsen
EPI_ISL_534326	Notre Dame Intermedica Saude AS	Instituto Adolfo Lutz, Interdiciplinary Procedures Center, Strategic Laboratory	Claudia Regina Gonçalves; Claudio Tavares Sacchi; Erica Valessa Ramos Gomes
EPI_ISL_900521 to 900523	Novabio	CNR Virus des Infections Respiratoires - France SUD	Antonin Bal; Bruno Lina; Gregory Destras; Gwendolyne Burfin; Hadrien Règue; Laurence Josset; Martine Valette; Quentin Semanas; Sylvie Larrat
EPI_ISL_735438	Nuclei Acid Testing - Rwanda National Reference Laboratory	GIGA Medical Genomics	Bouchra Boujemla; Esperence Umumararungu; Jacob Souopgui; Keith Durkin; Léon Mutesa; Maria Artesi; Marie-Pierre Hayette; Patrick Tuyisenge; Robert Rutayisire; Sabin Nsanzimana; Swaibu Gatare; Sébastien Bontems; Vincent Bours; Yvan Butera
EPI_ISL_735436 to 735437, EPI_ISL_735444, EPI_ISL_735448	Nucleic Acid Testing - Rwanda National Reference Laboratory	GIGA Medical Genomics	Bouchra Boujemla; Esperence Umumararungu; Jacob Souopgui; Keith Durkin; Léon Mutesa; Maria Artesi; Marie-Pierre Hayette; Patrick Tuyisenge; Robert Rutayisire; Sabin Nsanzimana; Swaibu Gatare; Sébastien Bontems; Vincent Bours; Yvan Butera
EPI_ISL_700471 to 700472, EPI_ISL_700486, EPI_ISL_700511, EPI_ISL_700523, EPI_ISL_700580	Nyanga CDC wc NGC	NHLS/UCT	Arash Iranzadeh; Bruna Galvao; Carolyn Williamson; Deelan Doolabh; Diana Hardie; Innocent Mudau; Kruger Marais; Lynn Tyers; Marvin Hsiao; Stephen Korsman
EPI_ISL_512846	O.I.J. MORGUE JUDICIAL	Inciensa, Instituto Costarricense de Investigación y Enseñanza en Nutrición y Salud	Adriana Godinez & Melany Calderon; Claudio Soto-Garita; Estela Cordero; Francisco Duarte; Hebleen Porras

EPI_ISL_631570, EPI_ISL_631591, EPI_ISL_631594, EPI_ISL_631598, EPI_ISL_631605, EPI_ISL_631617 to 631619, EPI_ISL_631685, EPI_ISL_631687, EPI_ISL_631694 to 631695, EPI_ISL_631758 to 631763, EPI_ISL_631797, EPI_ISL_631840 to 631841, EPI_ISL_631843, EPI_ISL_631845, EPI_ISL_631898, EPI_ISL_631932, EPI_ISL_631934, EPI_ISL_631936 to 631938, EPI_ISL_631956, EPI_ISL_632006, EPI_ISL_632073, EPI_ISL_632175 to 632176, EPI_ISL_632178, EPI_ISL_632183, EPI_ISL_632202, EPI_ISL_632238, EPI_ISL_632945 to 632946, EPI_ISL_883325, EPI_ISL_966397			
see above	OCME Office Of Chief Medical Examiner	New York City Public Health Laboratory	Jade Wang; et al.
EPI_ISL_751586, EPI_ISL_751609 to 751610, EPI_ISL_751613, EPI_ISL_751616, EPI_ISL_751741, EPI_ISL_751777, EPI_ISL_751788			
see above	OH Department of Health Laboratory	Genomics and Discovery, Respiratory Viruses Branch, Division of Viral Diseases, Centers for Disease Control and Prevention	Anna Montmayeur; Anna Uehara; Clinton R. Paden; Haibin Wang; Jing Zhang; Justin Lee; Krista Queen; Mili Sheth; Peter W. Cook; Rachel Marine; Suxiang Tong; Yan Li; Ying Tao
EPI_ISL_424880, EPI_ISL_426430 to 426431	OH Department of Health Laboratory	Pathogen Discovery, Respiratory Viruses Branch, Division of Viral Diseases, Centers for Disease Control and Prevention	Alison S. Laufer Halpin; Anna Uehara; Christopher A. Elkins; Clinton R. Paden; Haibin Wang; Jing Zhang; Krista Queen; Mary S. Keckler; Rachel Marine; Suxiang Tong; Yan Li; Ying Tao
EPI_ISL_525803, EPI_ISL_525808 to 525809, EPI_ISL_525813, EPI_ISL_525815 to 525818, EPI_ISL_525823, EPI_ISL_525826, EPI_ISL_525828 to 525832, EPI_ISL_525842 to 525843, EPI_ISL_525845 to 525846, EPI_ISL_525848, EPI_ISL_525852, EPI_ISL_525854, EPI_ISL_525863 to 525864, EPI_ISL_525867, EPI_ISL_525869, EPI_ISL_525873, EPI_ISL_525877, EPI_ISL_526087 to 526089, EPI_ISL_526092 to 526093, EPI_ISL_526097			
see above	OHSU Lab Services Molecular Microbiology Lab	Ginkgo Bioworks Clinical Laboratory	Alec J. Hirsch; Alex Plocik; Andrew C. Adey; Becky Schilling; Benjamin N. Bimber; Birgitte Simen; Brendan L. O'Connell; Brian J. O'Roak; Daniel N. Streblow; Guang Fan; James McGann; Jim Griffin; Keith Robison; Malaika Mckenzie-Bennett; Michelle Spencer; Rebecca Littlefield; Ruth V. Nichols; William B. Messer
EPI_ISL_437521, EPI_ISL_437523 to 437525, EPI_ISL_437528 to 437529, EPI_ISL_437531 to 437532, EPI_ISL_437535, EPI_ISL_468081 to 468084, EPI_ISL_468089, EPI_ISL_468095, EPI_ISL_468103, EPI_ISL_468105, EPI_ISL_468109 to 468110, EPI_ISL_468112 to 468113, EPI_ISL_468117, EPI_ISL_468120, EPI_ISL_468123, EPI_ISL_468125 to 468126, EPI_ISL_509059 to 509062, EPI_ISL_509064 to 509067, EPI_ISL_509069 to 509076, EPI_ISL_509079 to 509095, EPI_ISL_509097, EPI_ISL_509099, EPI_ISL_509101 to 509104, EPI_ISL_509106 to 509151, EPI_ISL_509153 to 509157, EPI_ISL_509159 to 509171, EPI_ISL_509174 to 509176, EPI_ISL_509181 to 509189, EPI_ISL_509191 to 509195, EPI_ISL_509197 to 509199, EPI_ISL_509201 to 509206, EPI_ISL_509208 to 509214, EPI_ISL_525802, EPI_ISL_525804 to 525807, EPI_ISL_525825, EPI_ISL_525827, EPI_ISL_525833, EPI_ISL_525841, EPI_ISL_525847, EPI_ISL_525849, EPI_ISL_525851, EPI_ISL_525856 to 525858, EPI_ISL_525860, EPI_ISL_525862, EPI_ISL_525868, EPI_ISL_525870, EPI_ISL_525874, EPI_ISL_525876, EPI_ISL_525878, EPI_ISL_525880 to 525881, EPI_ISL_525883, EPI_ISL_525885, EPI_ISL_525891 to 525925, EPI_ISL_525927 to 525945, EPI_ISL_525947 to 525967, EPI_ISL_525969 to 525977, EPI_ISL_525979 to 526022, EPI_ISL_526024 to 526041, EPI_ISL_526043 to 526069, EPI_ISL_526071 to 526076, EPI_ISL_526078 to 526080, EPI_ISL_526082 to 526086, EPI_ISL_526091, EPI_ISL_526094 to 526096, EPI_ISL_526098 to 526114, EPI_ISL_526551, EPI_ISL_667079, EPI_ISL_667085 to 667096, EPI_ISL_667098 to 667102, EPI_ISL_667104 to 667107, EPI_ISL_667109 to 667110, EPI_ISL_667112 to 667114, EPI_ISL_667116 to 667117, EPI_ISL_667119, EPI_ISL_667121 to 667140, EPI_ISL_667142 to 667147, EPI_ISL_667149 to 667154, EPI_ISL_667156 to 667182, EPI_ISL_667184 to 667185, EPI_ISL_667187 to 667197, EPI_ISL_667199 to 667203, EPI_ISL_667205, EPI_ISL_667207 to 667210, EPI_ISL_667212 to 667213, EPI_ISL_667216 to 667220, EPI_ISL_667222 to 667226, EPI_ISL_667229 to 667235, EPI_ISL_667237, EPI_ISL_667239 to 667245, EPI_ISL_667247 to 667248, EPI_ISL_667251 to 667258, EPI_ISL_667260 to 667271, EPI_ISL_667273 to 667278, EPI_ISL_667280, EPI_ISL_667282 to 667283, EPI_ISL_667285 to 667286, EPI_ISL_667289, EPI_ISL_667291 to 667294, EPI_ISL_667296 to 667299, EPI_ISL_667301, EPI_ISL_667304 to 667305, EPI_ISL_667307 to 667311, EPI_ISL_667313, EPI_ISL_667315 to 667317, EPI_ISL_667319 to 667325, EPI_ISL_667327 to 667332, EPI_ISL_667334 to 667335, EPI_ISL_667338, EPI_ISL_667341 to 667343, EPI_ISL_667345, EPI_ISL_667347 to 667348, EPI_ISL_667350 to 667352, EPI_ISL_667354 to 667359, EPI_ISL_667361, EPI_ISL_667363 to 667369, EPI_ISL_667371 to 667376, EPI_ISL_667378, EPI_ISL_667380, EPI_ISL_667383 to 667384, EPI_ISL_667390, EPI_ISL_667393, EPI_ISL_667395 to 667397, EPI_ISL_667400 to 667403, EPI_ISL_667405 to 667410, EPI_ISL_667413 to 667415, EPI_ISL_667417, EPI_ISL_667419 to 667420, EPI_ISL_667422 to 667424, EPI_ISL_667426 to 667427, EPI_ISL_667429 to 667431, EPI_ISL_667435 to 667442, EPI_ISL_667444 to 667452, EPI_ISL_667454, EPI_ISL_667457 to 667460, EPI_ISL_667462 to 667463, EPI_ISL_667465 to 667468, EPI_ISL_667470 to 667475, EPI_ISL_667478 to 667480, EPI_ISL_667483 to 667485, EPI_ISL_667488, EPI_ISL_667491 to 667493, EPI_ISL_667498, EPI_ISL_667500 to 667501, EPI_ISL_667505 to 667516, EPI_ISL_667519 to 667549, EPI_ISL_667551 to 667553, EPI_ISL_862034, EPI_ISL_906045			
see above	OHSU Lab Services Molecular Microbiology Lab	Oregon SARS-CoV-2 Genome Sequencing Center	Alec J. Hirsch; Andrew C. Adey; Benjamin N. Bimber; Brendan L. O'Connell; Brian J. O'Roak; Daniel N. Streblow; Donna Hansel; Guang Fan; Kayla Carter; Qin; Ruth V. Nichols; Sally B. Grindstaff; Sally Grindstaff; William B. Messer; Xuan; Xuan Qin
EPI_ISL_955307 to 955308	OK Public Health Laboratory, Oklahoma State DOH	Pathogen Discovery, Respiratory Viruses Branch, Division of Viral Diseases, Centers for Disease Control and Prevention	Anna Uehara; Clinton R. Paden; Haibin Wang; Jing Zhang; Krista Queen; Peter Cook; Suxiang Tong; Yan Li; Ying Tao
EPI_ISL_457705	OMAN-NIC	Department of Microbiology and Immunology- SQUH	Abdulla Balkhair; Ahlam Al-Amri; Aisha Al-Amri; Aisha Al-Busaidi; Amina Al Jardani; Fahad Zadjali; Fatma BaAlawi; Hamida AL Barwani; Hanan Al-kindi; Intisar Al-Shukri; Khulood Al-Mammary; Mohammed Al-Tobi; Samiha Al Kharusi; Samira Al-Maruqi; Zeyana AL-Dahmani
EPI_ISL_751615, EPI_ISL_751689, EPI_ISL_751693	OR State PHL-Virology/Immunology Section	Genomics and Discovery, Respiratory Viruses Branch, Division of Viral Diseases, Centers for Disease Control and Prevention	Anna Montmayeur; Anna Uehara; Clinton R. Paden; Haibin Wang; Jing Zhang; Justin Lee; Krista Queen; Mili Sheth; Peter W. Cook; Rachel Marine; Suxiang Tong; Yan Li; Ying Tao
EPI_ISL_419558	OR State PHL-Virology/Immunology Section	Pathogen Discovery, Respiratory Viruses Branch, Division of Viral Diseases, Centers for Disease Control and Prevention	Anna Uehara; Clinton R. Paden; Haibin Wang; Jasmine Padilla; Jing Zhang; Justin Lee; Krista Queen; Suxiang Tong; Yan Li; Ying Tao
EPI_ISL_534316	OS Mun Santana Lauro Ribas Braga	Instituto Adolfo Lutz, Interdiciplinary Procedures Center, Strategic Laboratory	Claudia Regina Gonçalves; Claudio Tavares Sacchi; Erica Valessa Ramos Gomes
EPI_ISL_833230	OSPEDALE S.S. ANNUNZIATA CHIETI - CLINICA MEDICA (MEDICINA GENERALE 1)	Istituto Zooprofilattico Sperimentale dell'Abruzzo e Molise "G.Caporale"	Ancora M; Calistri P; Cammà C; Curini V; Di Domenico M; Di Pasquale A; Lorusso A; Mangone I; Marcacci M; Puglia I; Rinaldi A; Savini G.
EPI_ISL_498171 to 498192	OUCRU	OUCRU	Dinh Nguyen Huy Man; Guy Thwaites; Lam Anh Nguyet; Lam Minh Yen; Le Manh Hung; Le Nguyen Truc Nhu; Nghiem My Ngoc; Ngo Ngoc Quang Minh; Nguyen Tan Binh; Nguyen Thanh Dung; Nguyen Thanh Phong; Nguyen Thanh Truong; Nguyen Thi Han Ny; Nguyen Thi Thu Hong; Nguyen Tri Dung; Nguyen Van Vinh Chau; Tang Chi Thuong; Tran Chanh Xuan; Tran Nguyen Hoang Tu; Tran Tan Thanh; Tran Tinh Hien; Vo Thanh Lam; and Le Van Tan; for OUCRU COVID-19 research group*
EPI_ISL_450738 to 450745	OUCRU/HTD	OUCRU/HTD	Dinh Nguyen Huy Man; Guy Thwaites; Lam Anh Nguyet; Lam Minh Yen; Le Manh Hung; Le Nguyen Truc Nhu; Nghiem My Ngoc; Ngo Ngoc Quang Minh; Nguyen Tan Binh; Nguyen Thanh Dung; Nguyen Thanh Phong; Nguyen Thanh Truong; Nguyen Thi Han Ny; Nguyen Thi Thu Hong; Nguyen Tri Dung; Nguyen Van Vinh Chau; Tang Chi Thuong; Tran Chanh Xuan; Tran Nguyen Hoang Tu; Tran Tan Thanh; Tran Tinh Hien; Vo Thanh Lam; and Le Van Tan; for OUCRU COVID-19 research group*
EPI_ISL_767405	Ochsner Health	BioInfo Experts, LLC	Amy Feehan; David J. Nolan; David Moraga Amador; Julia-Garcia-Diaz; Luke Caruso; Lydia Von Borstel; Rebecca Rose; Sissy Cross; Susanna L. Lamers; Tong Yang; Wayra Navia; Xiao Hui Zhou
EPI_ISL_426627 to 426628	Ochsner Health	BioInfoExperts, LLC	Amy Feehan; David Moraga Amador; David Nolan; Julia-Garcia-Diaz; Luke Caruso; Lydia Von Borstel; Rebecca Rose; Sissy Cross; Susanna Lamers; Tong Yang; Wayra Navia; Xiao Hui Zhou
EPI_ISL_436939, EPI_ISL_436942 to 436950, EPI_ISL_436952 to 436954, EPI_ISL_436956 to 436961, EPI_ISL_455609 to 455623, EPI_ISL_463277 to 463300, EPI_ISL_468701 to 468703, EPI_ISL_468705 to 468717			
see above	Ochsner Health	Bioinfoexperts, LLC	Amy Feehan; David J. Nolan; David Moraga Amador; Julia-Garcia-Diaz; Luke Caruso; Lydia Von Borstel; Rebecca Rose; Sissy Cross; Susanna L. Lamers; Tong Yang; Wayra Navia; Xiao Hui Zhou
EPI_ISL_763076	Ohio Department Of Health	Ohio Department of Health	Caitlin McDonnell; Eric Brandt; Glen McGillivary; Heather Blankenship; Jennifer Holmes; Kelsey Florek; Keoni Omura; Kirtana Ramadugu; Quanta Brown; and Tammy Bannerman
EPI_ISL_763321	Ohio Department of Health Laboratory	Ohio Depart of Health Laboratory	Caitlin McDonnell; Eric Brandt; Glen McGillivary; Heather Blankenship; Jennifer Holmes; Kelsey Florek; Keoni Omura; Kirtana Ramadugu; Quanta Brown; and Tammy Bannerman
EPI_ISL_765209, EPI_ISL_765222, EPI_ISL_962182	Ohio Department of Health Laboratory	Ohio Department of Health Laboratory	Brent Lee; Caitiln McDonnell; Caitlin McDonnell; Eric Brandt; Erica Leasure; Glen McGillivary; Heather Blankenship; Holmes; Jade Mowery; Jennifer; Kelsey Florek; Keoni Omura; Kirtana Ramadugu; Quanta Brown; Quanta Brown and Tammy Bannerman; Stephanie Mccracken; Tyler Payne; and Tammy Bannerman
EPI_ISL_763319	Ohio Department of Health Laboratory	Ohio Department of Health laboratory	Caitlin McDonnell; Eric Brandt; Glen McGillivary; Heather Blankenship; Jennifer Holmes; Kelsey Florek; Keoni Omura; Kirtana Ramadugu; Quanta Brown; and Tammy Bannerman
EPI_ISL_479979 to 479984, EPI_ISL_480109 to 480119	Oita Prefectural Institute of Public Health and Environmental Science	Pathogen Genomics Center, National Institute of Infectious Diseases	Hajime Kamiya; Kentaro Itokawa; Makoto Kuroda; Mari Sasaki; Masanori Hashino; Motoi Suzuki; Rina Tanaka; Tsuyoshi Sekizuka
EPI_ISL_535361 to 535364, EPI_ISL_812346, EPI_ISL_861423 to 861424	Oklahoma Animal Disease Diagnostic Laboratory	Oklahoma Animal Disease Diagnostic Laboratory	Akhilesh Ramachandran; Anil Kaul; Girish Patil; Jeremiah Saliki; Jerry Malayer; John C Ritchey; Sai Narayanan; Sunil More; Teluguakula Narasaraju
EPI_ISL_475762	Oklahoma State Department of Health	França Lab	Caio Martinelle B. de França; Graham Wiley; Samuel T. Dunn; and Matthew J. Miller.
EPI_ISL_455111	Olof Norrby	The Public Health Agency of Sweden	Anna Risberg; Anna-Malin Linde; Bla Kustens halsocentral; Karin Tegmark-Wisell; Maria Lind Karlberg; Mia Brytting; Olov Svartstrom; Oskar Karlsson Lindsjo; Theresa Enkirch
EPI_ISL_458116 to 458124	Oman National Influenza Centre	Department of Microbiology and Immunology-SQUH	Abdulla Balkhair; Ahlam Al-Amri; Aisha Al-Amri; Aisha Al-Busaidi; Amina Al Jardani; Fahad Zadjali; Fatma BaAlawi; Hamida AL Barwani; Hanan Al-kindi;

			Intisar Al-Shukri; Khulood Al-Mammary; Mohammed Al-Tobi; Samiha Al Kharusi; Samira Al-Maruqi; Zeyana AL-Dahmani
EPI_ISL_457703, EPI_ISL_457707	Oman-NIC	Department of Microbiology and Immunology- SQUH	Abdulla Balkhair; Ahlam Al-Amri; Aisha Al-Amri; Aisha Al-Busaidi; Amina Al Jardani; Fahad Zadjali; Fatma BaAlawi; Hamida AL Barwani; Hanan Al-kindi; Intisar Al-Shukri; Khulood Al-Mammary; Mohammed Al-Tobi; Samiha Al Kharusi; Samira Al-Maruqi; Zeyana AL-Dahmani
EPI_ISL_457981, EPI_ISL_491968 to 491970, EPI_ISL_491973 to 491974, EPI_ISL_491976, EPI_ISL_491978 to 491986, EPI_ISL_491988 to 491992, EPI_ISL_491994 to 492003, EPI_ISL_492005 to 492012, EPI_ISL_492014, EPI_ISL_492016 to 492017, EPI_ISL_492019 to 492026			
see above	Oman-NIC	Department of Microbiology and Immunology-SQUH	Abdulla Balkhair; Ahlam Al-Amri; Aisha Al-Amri; Aisha Al-Busaidi; Amina Al Jardani; Fahad Zadjali; Fatma BaAlawi; Hamida AL Barwani; Hanan Al-kindi; Intisar Al-Shukri; Khulood Al-Mammary; Mohammed Al-Tobi; Samiha Al Kharusi; Samira Al-Maruqi; Zeyana AL-Dahmani
EPI_ISL_457702	Oman-NIC	Microbiology laboratory- Sultan Qaboos University Hospital	Abdulla Balkhair; Ahlam Al-Amri; Aisha Al-Amri; Aisha Al-Busaidi; Amina Al Jardani; Fahad Zadjali; Fatma BaAlawi; Hamida AL Barwani; Hanan Al-kindi; Intisar Al-Shukri; Khulood Al-Mammary; Mohammed Al-Tobi; Samiha Al Kharusi; Samira Al-Maruqi; Zeyana AL-Dahmani
EPI_ISL_457701, EPI_ISL_457704, EPI_ISL_457706, EPI_ISL_457937 to 457939, EPI_ISL_457974 to 457980, EPI_ISL_457985 to 457998	Oman-NIC	Oman-NIC	Abdulla Balkhair; Ahlam Al-Amri; Aisha Al-Amri; Aisha Al-Busaidi; Amina Al Jardani; Fahad Zadjali; Fatma BaAlawi; Hamida AL Barwani; Hanan Al-kindi; Intisar Al-Shukri; Khulood Al-Mammary; Mohammed Al-Tobi; Samiha Al Kharusi; Samira Al-Maruqi; Zeyana AL-Dahmani
EPI_ISL_491116, EPI_ISL_491121 to 491171, EPI_ISL_525423 to 525429, EPI_ISL_525466	Oman-National Influenza Center	Biotechnology & OMICs Laboratory	Abdul Latif Khan; Adil Al-Wahaibi; Adil Khan; Ahlam Al-Amri; Ahmed Al-Harrasi; Ahmed Al-Rawahi; Aisha Al-Amri; Aisha Al-Busaidi; Amina Al-Jardani; Hanan Al-Kindi; Intisar Al-Shukri; Sajjad Asaf; Samiha Al-Kharusi; Samira Al-Mahruqi; Seif Al-Abri; Seif Al-Abri.
EPI_ISL_518821 to 518829, EPI_ISL_518831, EPI_ISL_518833 to 518845, EPI_ISL_518847, EPI_ISL_518849 to 518854	Oman-National Influenza Center	Biotechnology & OMICs Laboratory, Natural & Medical Sciences Research Center, University of Nizwa	Abdul Latif Khan; Adil Al-Wahaibi; Adil Khan; Ahlam Al-Amri; Ahmed Al-Harrasi; Ahmed Al-Rawahi; Aisha Al-Amri; Aisha Al-Busaidi; Amina Al-Jardani; Hanan Al-Kindi; Intisar Al-Shukri; Sajjad Asaf; Samiha Al-Kharusi; Samira Al-Mahruqi; Seif Al-Abri; Seif Al-Abri.
EPI_ISL_492065	Oman-National Influenza Center	Department of Microbiology and Immunology-SQUH Department of Microbiology and Immunology, Sultan Qaboos University Hopsital, P.O 35, Postal code 123	Abdulla Balkhair; Amina Al Jardani; Azza Al-Rashdi; Fahad Zadjali; Fatma BaAlawi; Hamida AL Barwani; Hanan Al-kindi; Intisar Al-Shukri; Khulood Al-Mammary; Samiha Al Kharusi; Samira Al-Maruqi; Zeyana AL-Dahmani
EPI_ISL_766569	Oman-National Influenza Center	Oman-National Influenza Center	Aisha Al-Busaidi; Amina Al-Jardani; Hamida Al-Barwani; Hanan Al-Kindi; Intisar Al-Shukri; Laila Al-Balushi; Samiha Al-Kharusi; Samira Al-Mahruqi
EPI_ISL_962502	Omics Sciences Laboratory	Omics Sciences Laboratory	Darlyn Amaya; Derly Andrade Molina; Emily Sulay Saltos Montalvo; Gabriel Morey León; Juan Carlos Fernández Cadena; Katheryn Sacheri Viteri; Paula Juliana Gavilanes Jarrín; Rubén Armas González
EPI_ISL_569735 to 569857, EPI_ISL_629031 to 629034, EPI_ISL_639912 to 639918, EPI_ISL_639920 to 639947, EPI_ISL_733484 to 733485, EPI_ISL_733489 to 733490, EPI_ISL_733493			
see above	Omsk Research Institute of Natural Focal Infections	WHO National Influenza Centre Russian Federation	Aleksei Vasilenko; Andrey Komissarov; Anna Ivanova; Artem Fadeev; Daria Danilenko; Daria Nashatyreva; Dmitry Bazhenov; Dmitry Lioznov; Ekaterina Gradoboeva; Ekaterina Savkina; Elena Nabieva; Elena Poleshchuk; Georgii Bazykin; Ksenia Safina; Kseniya Komissarova; Valery Yakimenko
EPI_ISL_434661 to 434662, EPI_ISL_434664, EPI_ISL_434673, EPI_ISL_475528, EPI_ISL_475533 to 475535, EPI_ISL_475538, EPI_ISL_539883 to 539884, EPI_ISL_710587 to 710588			
see above	Omtanken Grimmered	The Public Health Agency of Sweden	Anna Risberg; Anna-Malin Linde; Bernd Sengpiel; Department of Microbiology; Karin Tegmark-Wisell; Maria Lind Karlberg; Mattias Haukland; Mia Brytting; Olov Svartstrom; Oskar Karlsson Lindsjo; Reza Advani; Sandra Broddesson; The Public Health Agency of Sweden; Theresa Enkirch
EPI_ISL_468592 to 468603, EPI_ISL_468605, EPI_ISL_468607 to 468608, EPI_ISL_468611 to 468612, EPI_ISL_468614, EPI_ISL_513707 to 513721, EPI_ISL_513723 to 513729, EPI_ISL_513734 to 513735, EPI_ISL_513737 to 513758, EPI_ISL_513760 to 513762, EPI_ISL_513766 to 513772, EPI_ISL_582976 to 582996, EPI_ISL_582998 to 583004, EPI_ISL_583006 to 583008, EPI_ISL_583011 to 583026, EPI_ISL_583028 to 583029, EPI_ISL_583032 to 583033, EPI_ISL_583035 to 583036, EPI_ISL_583038 to 583044, EPI_ISL_625627 to 625630, EPI_ISL_625636, EPI_ISL_625641 to 625645, EPI_ISL_625647 to 625652, EPI_ISL_625654 to 625657, EPI_ISL_648496 to 648500, EPI_ISL_648503, EPI_ISL_648505 to 648506, EPI_ISL_648508 to 648516, EPI_ISL_648518 to 648520, EPI_ISL_648522 to 648527, EPI_ISL_648529 to 648530, EPI_ISL_672360 to 672374, EPI_ISL_672477 to 672487, EPI_ISL_738709, EPI_ISL_738722, EPI_ISL_738729, EPI_ISL_738752, EPI_ISL_738755, EPI_ISL_738757 to 738758, EPI_ISL_738761 to 738762, EPI_ISL_738782, EPI_ISL_738784, EPI_ISL_738787, EPI_ISL_738790, EPI_ISL_738799, EPI_ISL_738808, EPI_ISL_738811, EPI_ISL_738815 to 738816, EPI_ISL_738818, EPI_ISL_738823 to 738824, EPI_ISL_738826, EPI_ISL_738829 to 738830, EPI_ISL_738840 to 738841, EPI_ISL_738845, EPI_ISL_738850, EPI_ISL_738856 to 738857, EPI_ISL_738860, EPI_ISL_738862 to 738863, EPI_ISL_738866, EPI_ISL_738869, EPI_ISL_738871 to 738874, EPI_ISL_738878, EPI_ISL_738880, EPI_ISL_738882, EPI_ISL_738884 to 738885, EPI_ISL_738888, EPI_ISL_738893, EPI_ISL_738899 to 738900, EPI_ISL_738905, EPI_ISL_738907 to 738908, EPI_ISL_738921, EPI_ISL_738928, EPI_ISL_738930, EPI_ISL_738935, EPI_ISL_738937, EPI_ISL_738939, EPI_ISL_738944, EPI_ISL_738946, EPI_ISL_738949, EPI_ISL_738954, EPI_ISL_738966, EPI_ISL_738968, EPI_ISL_738970, EPI_ISL_738973, EPI_ISL_738975, EPI_ISL_738978, EPI_ISL_738981, EPI_ISL_738989, EPI_ISL_738991, EPI_ISL_738993, EPI_ISL_738995, EPI_ISL_739002, EPI_ISL_739013, EPI_ISL_739016, EPI_ISL_739028, EPI_ISL_739034, EPI_ISL_739042, EPI_ISL_739044, EPI_ISL_739048, EPI_ISL_739055, EPI_ISL_739063, EPI_ISL_739069, EPI_ISL_739071 to 739072, EPI_ISL_739078, EPI_ISL_739081, EPI_ISL_739089, EPI_ISL_739092, EPI_ISL_739094, EPI_ISL_739097, EPI_ISL_739099, EPI_ISL_739105, EPI_ISL_739108, EPI_ISL_739116, EPI_ISL_739125, EPI_ISL_739132, EPI_ISL_739139, EPI_ISL_739142 to 739143, EPI_ISL_739151, EPI_ISL_739154, EPI_ISL_739156, EPI_ISL_739162, EPI_ISL_739175 to 739176, EPI_ISL_739182, EPI_ISL_739185, EPI_ISL_739191, EPI_ISL_739199, EPI_ISL_739205, EPI_ISL_739211, EPI_ISL_739216, EPI_ISL_739222, EPI_ISL_739227, EPI_ISL_739230, EPI_ISL_739238 to 739239, EPI_ISL_739245, EPI_ISL_739255, EPI_ISL_739258 to 739259, EPI_ISL_739265 to 739267, EPI_ISL_739271, EPI_ISL_739276, EPI_ISL_739279, EPI_ISL_739289, EPI_ISL_739291, EPI_ISL_739296, EPI_ISL_739304, EPI_ISL_739314, EPI_ISL_739322, EPI_ISL_739331, EPI_ISL_739334, EPI_ISL_739341 to 739342, EPI_ISL_739345, EPI_ISL_739347, EPI_ISL_739350, EPI_ISL_739353, EPI_ISL_739359, EPI_ISL_739362, EPI_ISL_739369, EPI_ISL_739373, EPI_ISL_739375, EPI_ISL_739379, EPI_ISL_739384, EPI_ISL_739386, EPI_ISL_739396 to 739397, EPI_ISL_739401, EPI_ISL_739410, EPI_ISL_739412, EPI_ISL_739420 to 739421, EPI_ISL_739423, EPI_ISL_739428, EPI_ISL_739432, EPI_ISL_739437 to 739438, EPI_ISL_739441, EPI_ISL_739445, EPI_ISL_739450, EPI_ISL_739455, EPI_ISL_739460, EPI_ISL_739462, EPI_ISL_739464 to 739465, EPI_ISL_739477, EPI_ISL_739482, EPI_ISL_739486, EPI_ISL_739488, EPI_ISL_739490, EPI_ISL_739493 to 739494, EPI_ISL_739496 to 739497, EPI_ISL_739500, EPI_ISL_739513, EPI_ISL_739517, EPI_ISL_739524, EPI_ISL_739529, EPI_ISL_739532, EPI_ISL_739534, EPI_ISL_739540, EPI_ISL_739545, EPI_ISL_739547, EPI_ISL_739556, EPI_ISL_739559, EPI_ISL_739566, EPI_ISL_739568, EPI_ISL_739570, EPI_ISL_739573, EPI_ISL_739577, EPI_ISL_739581 to 739582, EPI_ISL_739586, EPI_ISL_739591 to 739592, EPI_ISL_739595, EPI_ISL_739599, EPI_ISL_739607, EPI_ISL_739609, EPI_ISL_739614, EPI_ISL_739620, EPI_ISL_739625, EPI_ISL_739627, EPI_ISL_739636 to 739637, EPI_ISL_739641, EPI_ISL_739646, EPI_ISL_739654, EPI_ISL_739658			
see above	Orange County Public Health Lab	Chan-Zuckerberg Biohub	CZB Cliahub Consortium
EPI_ISL_486121 to 486132, EPI_ISL_486134, EPI_ISL_486137 to 486139, EPI_ISL_486141 to 486151, EPI_ISL_486153 to 486168, EPI_ISL_486171 to 486174, EPI_ISL_486176 to 486189, EPI_ISL_486191 to 486196, EPI_ISL_486198, EPI_ISL_486202, EPI_ISL_486204 to 486206, EPI_ISL_486208 to 486218, EPI_ISL_486220 to 486222, EPI_ISL_486224, EPI_ISL_486226, EPI_ISL_486228 to 486244, EPI_ISL_486247, EPI_ISL_486249 to 486253, EPI_ISL_486255 to 486272, EPI_ISL_486274 to 486276, EPI_ISL_486278 to 486279, EPI_ISL_513789 to 513806, EPI_ISL_513814 to 513817, EPI_ISL_513820, EPI_ISL_513822, EPI_ISL_513824, EPI_ISL_513826, EPI_ISL_513828 to 513830, EPI_ISL_513832 to 513833, EPI_ISL_513835 to 513839, EPI_ISL_548270 to 548276, EPI_ISL_548283, EPI_ISL_548285, EPI_ISL_548289, EPI_ISL_548296, EPI_ISL_548298, EPI_ISL_548300 to 548301, EPI_ISL_548305 to 548306, EPI_ISL_548308 to 548309, EPI_ISL_548312 to 548313, EPI_ISL_548315, EPI_ISL_548317, EPI_ISL_548323, EPI_ISL_548326, EPI_ISL_548328, EPI_ISL_548332, EPI_ISL_548334, EPI_ISL_548373, EPI_ISL_548375, EPI_ISL_548377 to 548378, EPI_ISL_548381, EPI_ISL_548386 to 548387, EPI_ISL_548404, EPI_ISL_548408, EPI_ISL_548431, EPI_ISL_548439, EPI_ISL_548444, EPI_ISL_548460, EPI_ISL_548463 to 548464, EPI_ISL_548484 to 548485, EPI_ISL_548488, EPI_ISL_548580 to 548581, EPI_ISL_548585 to 548586, EPI_ISL_548590 to 548592, EPI_ISL_548594, EPI_ISL_548601, EPI_ISL_548608 to 548609, EPI_ISL_548611, EPI_ISL_548613 to 548615, EPI_ISL_548620, EPI_ISL_548623, EPI_ISL_548625, EPI_ISL_548627 to 548628, EPI_ISL_548634 to 548635, EPI_ISL_548637 to 548638, EPI_ISL_548641, EPI_ISL_548646, EPI_ISL_548648, EPI_ISL_548651, EPI_ISL_548653 to 548654, EPI_ISL_548660, EPI_ISL_548662, EPI_ISL_548665 to 548668, EPI_ISL_548674 to 548675, EPI_ISL_548677			
see above	Orange County Public Health Laboratory	Chan-Zuckerberg Biohub	CZB Cliahub Consortium
EPI_ISL_648207 to 648208	Orebro	The Public Health Agency of Sweden	Anna Risberg; Anna-Malin Linde; Karin Tegmark-Wisell; Maria Lind Karlberg; Mattias Haukland; Mia Brytting; Olov Svartstrom; Oskar Karlsson Lindsjo; Petra Edquist; Reza Advani; Sandra Broddesson
EPI_ISL_455840 to 455841, EPI_ISL_475128 to 475135, EPI_ISL_475139, EPI_ISL_510817, EPI_ISL_560972, EPI_ISL_582785 to 582789, EPI_ISL_582791 to 582793, EPI_ISL_582841, EPI_ISL_615079, EPI_ISL_615111 to 615114, EPI_ISL_615120, EPI_ISL_648204 to 648206, EPI_ISL_654943, EPI_ISL_660378 to 660383, EPI_ISL_660422 to 660426, EPI_ISL_661282, EPI_ISL_676493, EPI_ISL_676513			
see above	Orebro klinisk mikrobiologi	The Public Health Agency of Sweden	Anna Risberg; Anna-Malin Linde; Department of Microbiology; Karin Tegmark-Wisell; Maria Lind Karlberg; Mattias Haukland; Mia Brytting; Olov Svartstrom; Oskar Karlsson Lindsjo; Petra Edquist; Reza Advani; Sandra Broddesson; Shamam Muradrasoli; The Public Health Agency of Sweden
EPI_ISL_420794	Oregon State Public Health- Virology section	Pathogen Discovery, Respiratory Viruses Branch, Division of Viral Diseases, Centers for Disease Control and Prevention	Alison S. Laufer Halpin; Anne Uehara; Christopher A. Elkins; Clinton R. Paden; Haibin Wang; Jasmine Padilla; Jing Zhang; Justin Lee; Krista Queen; Mary S. Keckler; Rachel Marine; Suxiang Tong; Yan Li; Ying Tao
EPI_ISL_475511	Orestadsklinikens VC	The Public Health Agency of Sweden	Anna Risberg; Anna-Malin Linde; Karin Tegmark-Wisell; Maria Lind Karlberg; Mattias Haukland; Mia Brytting; Olov Svartstrom; Oskar Karlsson Lindsjo; Reza Advani; Sandra Broddesson
EPI_ISL_900511	Oriade	CNR Virus des Infections Respiratoires - France SUD	Antonin Bal; Bruno Lina; Gregory Destras; Gwendolyne Burfin; Hadrien Règue; Laurence Josset; Martine Valette; Quentin Semanas; Sylvie Larrat
EPI_ISL_840374 to 840375, EPI_ISL_840377 to 840378, EPI_ISL_840380, EPI_ISL_840395, EPI_ISL_840404, EPI_ISL_840411, EPI_ISL_840422, EPI_ISL_840426, EPI_ISL_840428, EPI_ISL_840430 to 840433, EPI_ISL_840443 to 840444, EPI_ISL_840454 to 840455, EPI_ISL_840460, EPI_ISL_840462 to 840464, EPI_ISL_840466, EPI_ISL_840473, EPI_ISL_840479 to 840480, EPI_ISL_840497, EPI_ISL_840520, EPI_ISL_840522, EPI_ISL_840529, EPI_ISL_840533, EPI_ISL_840543, EPI_ISL_840559, EPI_ISL_840561 to 840562, EPI_ISL_840565, EPI_ISL_840572, EPI_ISL_840575 to 840576, EPI_ISL_840585, EPI_ISL_840611, EPI_ISL_840615, EPI_ISL_840617, EPI_ISL_840620, EPI_ISL_840627, EPI_ISL_840629 to 840630, EPI_ISL_840632, EPI_ISL_840636, EPI_ISL_840643, EPI_ISL_840649, EPI_ISL_840674, EPI_ISL_840677, EPI_ISL_840680, EPI_ISL_840687, EPI_ISL_840701, EPI_ISL_840737 to 840738, EPI_ISL_840744 to 840745, EPI_ISL_840749, EPI_ISL_840751, EPI_ISL_840812, EPI_ISL_841321, EPI_ISL_841330, EPI_ISL_841339 to 841340, EPI_ISL_841347 to 841349, EPI_ISL_841355, EPI_ISL_841368, EPI_ISL_841397, EPI_ISL_841400, EPI_ISL_841403, EPI_ISL_841405, EPI_ISL_841416, EPI_ISL_841422 to 841423, EPI_ISL_841429, EPI_ISL_841431, EPI_ISL_841435, EPI_ISL_841440, EPI_ISL_841448, EPI_ISL_841451, EPI_ISL_841453, EPI_ISL_841456, EPI_ISL_841473, EPI_ISL_841475 to 841476, EPI_ISL_841481, EPI_ISL_841492, EPI_ISL_841496, EPI_ISL_841508 to 841511, EPI_ISL_841515 to 841518, EPI_ISL_841521 to 841522, EPI_ISL_841524, EPI_ISL_841526, EPI_ISL_841528, EPI_ISL_841533 to 841535, EPI_ISL_841541 to 841542, EPI_ISL_841546, EPI_ISL_841580, EPI_ISL_841611, EPI_ISL_841631, EPI_ISL_841659, EPI_ISL_841661, EPI_ISL_841669, EPI_ISL_841679, EPI_ISL_841684 to 841688, EPI_ISL_841690, EPI_ISL_841694, EPI_ISL_841702, EPI_ISL_841704 to 841706, EPI_ISL_841710 to 841715, EPI_ISL_841718, EPI_ISL_841724, EPI_ISL_841727, EPI_ISL_841743 to 841744, EPI_ISL_841747 to 841748, EPI_ISL_841752, EPI_ISL_867193, EPI_ISL_867204, EPI_ISL_867212, EPI_ISL_867214 to 867215, EPI_ISL_867225, EPI_ISL_867237 to 867239, EPI_ISL_867244, EPI_ISL_867248 to 867249, EPI_ISL_867251 to 867252, EPI_ISL_867254, EPI_ISL_867257, EPI_ISL_867260 to 867263, EPI_ISL_867268, EPI_ISL_867270, EPI_ISL_867305 to 867312, EPI_ISL_867320, EPI_ISL_867327, EPI_ISL_867332, EPI_ISL_867335, EPI_ISL_867342, EPI_ISL_867344, EPI_ISL_867353, EPI_ISL_867362, EPI_ISL_867364 to 867368, EPI_ISL_867381 to 867382, EPI_ISL_867384, EPI_ISL_867386, EPI_ISL_867392 to 867400, EPI_ISL_867403 to 867404, EPI_ISL_867415, EPI_ISL_867417, EPI_ISL_867426, EPI_ISL_867430, EPI_ISL_867442, EPI_ISL_867456, EPI_ISL_867460, EPI_ISL_867462 to 867463, EPI_ISL_867465 to 867466, EPI_ISL_867470, EPI_ISL_867485, EPI_ISL_867492, EPI_ISL_867494, EPI_ISL_867497, EPI_ISL_867506, EPI_ISL_867509 to 867510, EPI_ISL_867518, EPI_ISL_867550, EPI_ISL_867552 to 867553, EPI_ISL_867573, EPI_ISL_867584, EPI_ISL_867588, EPI_ISL_867591, EPI_ISL_867599, EPI_ISL_867610, EPI_ISL_867621, EPI_ISL_867623, EPI_ISL_867628, EPI_ISL_867637, EPI_ISL_867650, EPI_ISL_867653 to 867655, EPI_ISL_867661, EPI_ISL_867663 to 867665, EPI_ISL_867667 to 867668, EPI_ISL_867670 to 867674, EPI_ISL_867676, EPI_ISL_867681 to 867682, EPI_ISL_867684, EPI_ISL_867688, EPI_ISL_867692, EPI_ISL_867716, EPI_ISL_867719, EPI_ISL_867721 to 867722, EPI_ISL_867726, EPI_ISL_867759, EPI_ISL_867762, EPI_ISL_867764, EPI_ISL_867768, EPI_ISL_867771, EPI_ISL_867776, EPI_ISL_867779, EPI_ISL_867795 to 867796, EPI_ISL_867812, EPI_ISL_867815 to			

867816, EPI_ISL_867821, EPI_ISL_867828, EPI_ISL_867832, EPI_ISL_867841 to 867843, EPI_ISL_867847, EPI_ISL_867853, EPI_ISL_867861, EPI_ISL_867863, EPI_ISL_867875, EPI_ISL_867880 to 867881, EPI_ISL_867900, EPI_ISL_867906, EPI_ISL_867912, EPI_ISL_867915, EPI_ISL_867923, EPI_ISL_951496 to 951497, EPI_ISL_951499 to 951501, EPI_ISL_951503, EPI_ISL_951506, EPI_ISL_951510 to 951511, EPI_ISL_951514 to 951515, EPI_ISL_951517 to 951519, EPI_ISL_951521 to 951523, EPI_ISL_951527, EPI_ISL_951529, EPI_ISL_951531, EPI_ISL_951534 to 951535, EPI_ISL_951538, EPI_ISL_951541 to 951542, EPI_ISL_951545, EPI_ISL_951550, EPI_ISL_951553 to 951554, EPI_ISL_951556, EPI_ISL_951558 to 951560, EPI_ISL_951567, EPI_ISL_951571 to 951573, EPI_ISL_951576 to 951581, EPI_ISL_951583, EPI_ISL_951585, EPI_ISL_951592, EPI_ISL_951603 to 951605, EPI_ISL_951607, EPI_ISL_951613 to 951614, EPI_ISL_951624, EPI_ISL_951634 to 951635, EPI_ISL_951637, EPI_ISL_951646, EPI_ISL_951651 to 951653, EPI_ISL_951660, EPI_ISL_951664, EPI_ISL_951670, EPI_ISL_951677, EPI_ISL_951679 to 951680, EPI_ISL_951684 to 951688, EPI_ISL_951691, EPI_ISL_951694, EPI_ISL_951698, EPI_ISL_951700 to 951705, EPI_ISL_951708 to 951709, EPI_ISL_951711, EPI_ISL_951713, EPI_ISL_951718, EPI_ISL_951720, EPI_ISL_951730 to 951732, EPI_ISL_951735, EPI_ISL_951738, EPI_ISL_951742, EPI_ISL_951746 to 951747, EPI_ISL_951749, EPI_ISL_951752, EPI_ISL_951757, EPI_ISL_951762, EPI_ISL_951767, EPI_ISL_951772, EPI_ISL_951777, EPI_ISL_951786, EPI_ISL_951802, EPI_ISL_951821, EPI_ISL_951825, EPI_ISL_951829, EPI_ISL_951831 to 951834, EPI_ISL_951842 to 951843, EPI_ISL_951845, EPI_ISL_951847 to 951853, EPI_ISL_951855, EPI_ISL_951859, EPI_ISL_951878, EPI_ISL_951882, EPI_ISL_951887, EPI_ISL_951894, EPI_ISL_951896, EPI_ISL_951901 to 951902, EPI_ISL_951904 to 951905, EPI_ISL_951909 to 951910, EPI_ISL_951913, EPI_ISL_951916 to 951919, EPI_ISL_951921, EPI_ISL_951923 to 951924, EPI_ISL_951927 to 951928, EPI_ISL_951930 to 951933, EPI_ISL_951935, EPI_ISL_951937, EPI_ISL_951939 to 951940, EPI_ISL_951943, EPI_ISL_951945, EPI_ISL_951947 to 951949, EPI_ISL_951951, EPI_ISL_951953 to 951958, EPI_ISL_951960 to 951961, EPI_ISL_951963, EPI_ISL_951966, EPI_ISL_951975, EPI_ISL_951977, EPI_ISL_951983, EPI_ISL_951985 to 951987, EPI_ISL_951998 to 952000, EPI_ISL_952006, EPI_ISL_952010, EPI_ISL_952014, EPI_ISL_952018, EPI_ISL_952022, EPI_ISL_952036, EPI_ISL_952040, EPI_ISL_952044, EPI_ISL_952047 to 952050, EPI_ISL_952053, EPI_ISL_952058, EPI_ISL_952060 to 952067, EPI_ISL_952069 to 952072, EPI_ISL_952074, EPI_ISL_952076 to 952077, EPI_ISL_952079, EPI_ISL_952082 to 952083, EPI_ISL_952087, EPI_ISL_952089 to 952090, EPI_ISL_952092, EPI_ISL_952095, EPI_ISL_952097, EPI_ISL_952100, EPI_ISL_952102, EPI_ISL_952108 to 952114, EPI_ISL_952120, EPI_ISL_952124, EPI_ISL_952132 to 952134, EPI_ISL_952136, EPI_ISL_952139 to 952141, EPI_ISL_952143 to 952151, EPI_ISL_952156 to 952157, EPI_ISL_952159, EPI_ISL_952161 to 952162, EPI_ISL_952165 to 952166, EPI_ISL_952171 to 952172, EPI_ISL_952177, EPI_ISL_952180, EPI_ISL_952187, EPI_ISL_952190, EPI_ISL_952194 to 952196, EPI_ISL_952199, EPI_ISL_952201 to 952205, EPI_ISL_952214, EPI_ISL_952216 to 952217, EPI_ISL_952224, EPI_ISL_952226, EPI_ISL_952231, EPI_ISL_952234, EPI_ISL_952238 to 952239, EPI_ISL_952246, EPI_ISL_952248, EPI_ISL_952251 to 952253, EPI_ISL_952255 to 952256, EPI_ISL_952258 to 952259, EPI_ISL_952263 to 952264, EPI_ISL_952266, EPI_ISL_952269 to 952272, EPI_ISL_952274 to 952276, EPI_ISL_952278, EPI_ISL_952282, EPI_ISL_952286 to 952294, EPI_ISL_952297, EPI_ISL_952303 to 952306, EPI_ISL_952308 to 952309, EPI_ISL_952311 to 952313, EPI_ISL_952315 to 952316, EPI_ISL_952321, EPI_ISL_952324 to 952331, EPI_ISL_952334 to 952335, EPI_ISL_952337 to 952343, EPI_ISL_952345 to 952346, EPI_ISL_952349 to 952351, EPI_ISL_952353 to 952356, EPI_ISL_952358 to 952360, EPI_ISL_952362 to 952364, EPI_ISL_952372 to 952376, EPI_ISL_999516 to 999521, EPI_ISL_999523, EPI_ISL_999529 to 999530, EPI_ISL_999532 to 999533, EPI_ISL_999537 to 999538, EPI_ISL_999540 to 999545, EPI_ISL_999547 to 999550, EPI_ISL_999553 to 999554, EPI_ISL_999557, EPI_ISL_999560, EPI_ISL_999566, EPI_ISL_999569, EPI_ISL_999571 to 999572, EPI_ISL_999574, EPI_ISL_999582 to 999583, EPI_ISL_999585 to 999586, EPI_ISL_999588 to 999589, EPI_ISL_999595 to 999599, EPI_ISL_999601 to 999602, EPI_ISL_999605 to 999607, EPI_ISL_999609, EPI_ISL_999612 to 999614, EPI_ISL_999618, EPI_ISL_999620 to 999621, EPI_ISL_999623, EPI_ISL_999625 to 999628, EPI_ISL_999630, EPI_ISL_999633, EPI_ISL_999652, EPI_ISL_999664, EPI_ISL_999669, EPI_ISL_999687, EPI_ISL_999694 to 999695, EPI_ISL_999702, EPI_ISL_999717, EPI_ISL_999741, EPI_ISL_999747, EPI_ISL_999756 to 999757, EPI_ISL_999764 to 999765, EPI_ISL_999767, EPI_ISL_999770, EPI_ISL_999781, EPI_ISL_999783, EPI_ISL_999825, EPI_ISL_999830, EPI_ISL_999832 to 999833, EPI_ISL_999848, EPI_ISL_999852 to 999853, EPI_ISL_999858 to 999859, EPI_ISL_999870, EPI_ISL_999879, EPI_ISL_999881, EPI_ISL_999894, EPI_ISL_999900, EPI_ISL_999905 to 999908, EPI_ISL_999910, EPI_ISL_999916 to 999917, EPI_ISL_999921, EPI_ISL_999923, EPI_ISL_999931, EPI_ISL_999935 to 999936, EPI_ISL_999943			
see above	Originating lab: Wales Specialist Virology Centre Sequencing lab: Pathogen Genomics Unit	Public Health Wales Microbiology Cardiff Wales Specialist Virology Centre	Alec Birchley; Alexander Adams; Amy Gaskin; Angela Marchbank; Bree Gatica-Wilcox; Catherine Moore; Jason Coombes; Joanne Watkins; Joel Southgate; Johnathan Evans; Laura Gifford; Lauren Gilbert; Lee Graham; Malorie Perry; Matthew Bull; Nicole Pacchiarini; Sally Corden; Sara Kumziene-Summerhayes; Sara Rey; Sarah Taylor; Simon Cottrell; Sophie Jones; Tom Connor
EPI_ISL_455100, EPI_ISL_475519, EPI_ISL_710585 to 710586	Orsa VC	The Public Health Agency of Sweden	Anna Risberg; Anna-Karin Lundqvist; Anna-Malin Linde; Department of Microbiology; Karin Tegmark-Wisell; Maria Lind Karlberg; Mattias Haukland; Mia Brytting; Olov Svartstrom; Oskar Karlsson Lindsjo; Reza Advani; Sandra Broddesson; The Public Health Agency of Sweden; Theresa Enkirch
EPI_ISL_479944 to 479958, EPI_ISL_479966	Osaka Institute of Public Health	Pathogen Genomics Center, National Institute of Infectious Diseases	Hajime Kamiya; Kazushi Motomura; Kentaro Itokawa; Makoto Kuroda; Masanori Hashino; Motoi Suzuki; Rina Tanaka; Saeko Morikawa; Satoshi Hiroi; Tsuyoshi Sekizuka
EPI_ISL_417483	Oslo University Hospital, Department of Medical Microbiology	Norwegian Institute of Public Health	Hilde Elshaug; Kamilla Heddeland Instefjord; Karoline Bragstad; Kathrine Stene-Johansen; Olav Hungnes
EPI_ISL_417484, EPI_ISL_417488, EPI_ISL_420135, EPI_ISL_420150, EPI_ISL_471177, EPI_ISL_493352 to 493355, EPI_ISL_493385, EPI_ISL_493387, EPI_ISL_493389, EPI_ISL_549029 to 549035, EPI_ISL_590878, EPI_ISL_590900, EPI_ISL_590908 to 590910, EPI_ISL_590913 to 590914, EPI_ISL_590916, EPI_ISL_590918, EPI_ISL_590923, EPI_ISL_590927 to 590928, EPI_ISL_590930, EPI_ISL_590934 to 590935, EPI_ISL_590978 to 590980, EPI_ISL_591011, EPI_ISL_591020, EPI_ISL_635085, EPI_ISL_635095, EPI_ISL_635119, EPI_ISL_635129, EPI_ISL_635155, EPI_ISL_635172, EPI_ISL_635197, EPI_ISL_668401 to 668402, EPI_ISL_708033 to 708034, EPI_ISL_708052 to 708054, EPI_ISL_708096, EPI_ISL_708118 to 708121, EPI_ISL_708171 to 708175, EPI_ISL_759982, EPI_ISL_759990, EPI_ISL_964281, EPI_ISL_964292, EPI_ISL_964295, EPI_ISL_964955, EPI_ISL_964998 to 964999, EPI_ISL_1013507			
see above	Oslo University Hospital, Department of Medical Microbiology	Norwegian Institute of Public Health, Department of Virology	Atiya R Ali; Engebretsen Serina Beate Atiya R Ali; Garcia Llorente Ignacio; Hilde Elshaug; Hilde Synnøve Vollan; Hilde Vollan; Ignacio Garcia Llorente; Kamilla Heddeland Instefjord; Karoline Bragstad; Kathrine Stene-Johansen; Marie Paulsen Madsen; Olav Hungnes; Rasmus Riis Kopperud; Serina B Engebretsen
EPI_ISL_458066 to 458069, EPI_ISL_495274 to 495297, EPI_ISL_644715 to 644734	Osmania Medical College	CSIR-Centre for Cellular and Molecular Biology	Archana Bharadwaj Siva; B Himasri; Blessy B John; Dhiviya Vedagiri; Divya Gupta; Divya Tej Sowpati; Dr.P.Shashikala Reddy; Dr.S.Pavani; Dr.Satyaprasad; Dr.V.Sudha Rani; Karthik Bharadwaj Tallapaka; Krishnan Harinivas Harshan; Lamuk Zaveri; M Soujanya Reddy; Mahboob Khan; Namami Gaur; Nikhil Hajirnis; Onkar Kulkarni; Payel Mukherjee; Pratheusa Maccha; Priya Singh; Purushotham Vodnala; Rakesh K Mishra; Sakshi Shambhavi; Santosh Kumar Kuncha; Shagufta Khan; Shashikala Reddy; Sofia Banu; Tulasi Nagabandi; Vishal Sah; Viswagithe S L
EPI_ISL_447902	Osmania University	Osmania University	Amit; An and Kumar, K.; Bosinger; Kalyani, P.; Nagamani, K.; Pankaj Singh, D.; Radhakrishna, M.; Raja Rao, M.; Ravikumar, P.; S.E. and Rama, A.; Sunitha, P.; Thrilok Chander, B.; U.A.
EPI_ISL_528922, EPI_ISL_528924, EPI_ISL_528926 to 528928, EPI_ISL_529018, EPI_ISL_529026	Ospedale "Giuseppe Mazzini"-Teramo	Istitituto Zooprofilattico Sperimentale dell'Abruzzo e Molise "G.Caporale"	Ancora M; Cammà C; Curini V; Di Domenico M; Di Pasquale A; Lorusso A; Mangone I; Marcacci M; Puglia I; Rinaldi A; Savini G.
EPI_ISL_529014 to 529015, EPI_ISL_529022	Ospedale "Ss. Annunziata"	Istitituto Zooprofilattico Sperimentale dell'Abruzzo e Molise "G.Caporale"	Ancora M; Cammà C; Curini V; Di Domenico M; Di Pasquale A; Lorusso A; Mangone I; Marcacci M; Puglia I; Rinaldi A; Savini G.
EPI_ISL_420564	Ospedale Civile Castel Di Sangro	Istituto Zooprofilattico Sperimentale dell'Abruzzo e Molise "G.Caporale"	Ancora M; Cammà C; Curini V; Di Domenico M; Di Pasquale A; Lorusso A; Mangone I; Marcacci M; Puglia I; Rinaldi A; Savini G
EPI_ISL_418260, EPI_ISL_420563, EPI_ISL_429228, EPI_ISL_429231 to 429232, EPI_ISL_429235	Ospedale Civile Giuseppe Mazzini	Istituto Zooprofilattico Sperimentale dell'Abruzzo e Molise "G. Caporale"	Ancora M; Camma C; Cammà C; Curini V; Di Domenico M; Di Pasquale A; Lorusso A; Mangone I; Marcacci M; Puglia I; Rinaldi A; Savini G; Savini G.
EPI_ISL_420565, EPI_ISL_420568 to 420569, EPI_ISL_420583, EPI_ISL_435145, EPI_ISL_436723	Ospedale Civile Giuseppe Mazzini	Istituto Zooprofilattico Sperimentale dell'Abruzzo e Molise "G.Caporale"	Ancora M; Cammà C; Curini V; Di Domenico M; Di Pasquale A; Lorusso A; Mangone I; Marcacci M; Puglia I; Rinaldi A; Savini G
EPI_ISL_418257	Ospedale Civile Giuseppe Mazzini, Teramo	Istituto Zooprofilattico Sperimentale dell'Abruzzo e Molise "G.Caporale"	Ancora M; Cammà C; Curini V; Di Domenico M; Di Pasquale A; Lorusso A; Mangone I; Marcacci M; Puglia I; Rinaldi A; Savini G.
EPI_ISL_528990	Ospedale Civile Maria SS. dello Splendore	Istitituto Zooprofilattico Sperimentale dell'Abruzzo e Molise "G.Caporale"	Ancora M; Cammà C; Curini V; Di Domenico M; Di Pasquale A; Lorusso A; Mangone I; Marcacci M; Puglia I; Rinaldi A; Savini G.
EPI_ISL_429236	Ospedale Civile S. Liberatore di Atri	Istituto Zooprofilattico Sperimentale dell'Abruzzo e Molise "G. Caporale"	Ancora M; Camma C; Curini V; Di Domenico M; Di Pasquale A; Lorusso A; Mangone I; Marcacci M; Puglia I; Rinaldi A; Savini G
EPI_ISL_436719 to 436722, EPI_ISL_436724, EPI_ISL_436731 to 436732	Ospedale Civile S. Liberatore di Atri	Istituto Zooprofilattico Sperimentale dell'Abruzzo e Molise "G.Caporale"	Ancora M; Cammà C; Curini V; Di Domenico M; Di Pasquale A; Lorusso A; Mangone I; Marcacci M; Puglia I; Rinaldi A; Savini G
EPI_ISL_528919, EPI_ISL_528929, EPI_ISL_528993, EPI_ISL_529007, EPI_ISL_529009, EPI_ISL_529020 to 529021	Ospedale Civile S. Liberatore-Atri	Istitituto Zooprofilattico Sperimentale dell'Abruzzo e Molise "G.Caporale"	Ancora M; Cammà C; Curini V; Di Domenico M; Di Pasquale A; Lorusso A; Mangone I; Marcacci M; Puglia I; Rinaldi A; Savini G.
EPI_ISL_918410	Ospedale Di Venere - Carbonara	Istituto Zooprofilattico Sperimentale della Puglia e della Basilicata	Bianco A.; Capozzi L.; Cipolletta D.; Del Sambro L.; Galante D.; Manzulli V; Pace L.; Parisi A.; Rondinone V.; Simone D.
EPI_ISL_747465 to 747470, EPI_ISL_747484 to 747485	Ospedale Mater Salutis	Istituto Zooprofilattico Sperimentale delle Venezie	Adelaide Milani; Alessia Schivo; Alice Fusaro; Ambra Pastori; Annalisa Salviato; Antonia Ricci; Bianca Zecchin; Calogero Terregino; Erika Giorgia Quaranta; Isabella Monne
EPI_ISL_420566 to 420567, EPI_ISL_436718	Ospedale Regionale San Salvatore	Istituto Zooprofilattico Sperimentale dell'Abruzzo e Molise "G.Caporale"	Ancora M; Cammà C; Curini V; Di Domenico M; Di Pasquale A; Lorusso A; Mangone I; Marcacci M; Puglia I; Rinaldi A; Savini G
EPI_ISL_528925	Ospedale Regionale San Salvatore-L'Aquila	Istitituto Zooprofilattico Sperimentale dell'Abruzzo e Molise "G.Caporale"	Ancora M; Cammà C; Curini V; Di Domenico M; Di Pasquale A; Lorusso A; Mangone I; Marcacci M; Puglia I; Rinaldi A; Savini G.
EPI_ISL_435148, EPI_ISL_435150 to 435151	Ospedale SS Annunziata	Istituto Zooprofilattico Sperimentale dell'Abruzzo e Molise "G.Caporale"	Ancora M; Cammà C; Curini V; Di Domenico M; Di Pasquale A; Lorusso A; Mangone I; Marcacci M; Puglia I; Rinaldi A; Savini G
EPI_ISL_961093	Ospedale SS Annunziata	Istituto Zooprofilattico Sperimentale dell'Abruzzo e Molise "G. Caporale"	Ancora M; Calistri P; Cammà C; Curini V; Di Domenico M; Di Pasquale A; Lorusso A; Mangone I; Marcacci M; Puglia I; Rinaldi A; Savini G; Scialabba S
EPI_ISL_833265 to 833269	Ospedale SS Annunziata Chieti	Istituto Zooprofilattico Sperimentale dell'Abruzzo e Molise "G.	Ancora M; Calistri P; Cammà C; Curini V; Di Domenico M; Di Pasquale A; Lorusso A; Mangone I; Marcacci M; Puglia I; Rinaldi A; Savini G

		Caporale"	
EPI_ISL_528991, EPI_ISL_529016	Ospedale SS Annunziata-Sulmona	Istitituto Zooprofilattico Sperimentale dell'Abruzzo e Molise "G.Caporale"	Ancora M; Cammà C; Curini V; Di Domenico M; Di Pasquale A; Lorusso A; Mangone I; Marcacci M; Puglia I; Rinaldi A; Savini G.
EPI_ISL_747459 to 747464, EPI_ISL_747482 to 747483	Ospedale San Bonifacio	Istituto Zooprofilattico Sperimentale delle Venezie	Adelaide Milani; Alessia Schivo; Alice Fusaro; Ambra Pastori; Annalisa Salviato; Antonia Ricci; Bianca Zecchin; Calogero Terregino; Erika Giorgia Quaranta; Isabella Monne
EPI_ISL_941336 to 941337	Ospedale San Camillo De Lellis di Rieti	INMI Lazzaro Spallanzani IRCCS	Antonino Di Caro; Assunta De Luca; Barbara Bartolini; Cesare E.M. Gruber; Emanuela Giombini; Francesco Messina; Luca Casertano; Maria R. Capobianchi; Martina Rueca; Ornella Butera; Stefano Venarubea
EPI_ISL_794750, EPI_ISL_918408 to 918409, EPI_ISL_1008664, EPI_ISL_1008700	Ospedale Santa Caterina Novella	Istituto Zooprofilattico Sperimentale della Puglia e della Basilicata	Bianco A.; Capozzi L.; Chironna M.; Cipolletta D.; Del Sambro L.; Galante D.; Loconsole D.; Manzulli V; Pace L.; Parisi A.; Ridolfi D.; Rondinone V.; Sallustio A.; Simone D.
EPI_ISL_794747, EPI_ISL_794751	Ospedale Vito Fazzi	Istituto Zooprofilattico Sperimentale della Puglia e della Basilicata	Bianco A.; Capozzi L.; Cipolletta D.; Del Sambro L.; Galante D.; Manzulli V; Pace L.; Parisi A.; Rondinone V.
EPI_ISL_418256	Ospedale "San Liberatore" di Atri	Istituto Zooprofilattico Sperimentale dell'Abruzzo e Molise "G. Caporale"	Ancora M; Cammà C; Curini V; Di Domenico M; Di Pasquale A; Lorusso A; Mangone I; Marcacci M; Puglia I; Rinaldi A; Savini G.
EPI_ISL_475124, EPI_ISL_476145 to 476147, EPI_ISL_548248	Ostersund klinisk mikrobiologi	The Public Health Agency of Sweden	Anna Risberg; Anna-Malin Linde; Karin Tegmark-Wisell; Maria Lind Karlberg; Mattias Haukland; Mia Brytting; Olov Svartstrom; Oskar Karlsson Lindsjo; Petra Edquist; Reza Advani; Sandra Broddesson; Shamam Muradrasoli
EPI_ISL_549042 to 549047, EPI_ISL_549092 to 549111, EPI_ISL_549113 to 549118, EPI_ISL_549126 to 549131, EPI_ISL_549133 to 549142, EPI_ISL_549144 to 549153, EPI_ISL_549155 to 549163, EPI_ISL_590894 to 590897, EPI_ISL_590921 to 590922, EPI_ISL_590931, EPI_ISL_590948, EPI_ISL_590986 to 591006, EPI_ISL_591009, EPI_ISL_591015 to 591017, EPI_ISL_635107 to 635109, EPI_ISL_635116, EPI_ISL_635149 to 635152, EPI_ISL_635164, EPI_ISL_635174 to 635175, EPI_ISL_635185, EPI_ISL_668433, EPI_ISL_668438 to 668440, EPI_ISL_708032, EPI_ISL_708110 to 708114, EPI_ISL_964952			
see above	Ostfold Hospital Trust - Kalnes, Centre for Laboratory Medicine, Section for gene technology and infection serology	Norwegian Institute of Public Health, Department of Virology	Atiya R Ali; Hilde Elshaug; Hilde Synnøve Vollan; Hilde Vollan; Ignacio Garcia Llorente; Kamilla Heddeland Instefjord; Karoline Bragstad; Kathrine Stene-Johansen; Marie Paulsen Madsen; Olav Hungnes; Rasmus Riis Kopperud; Serina B Engebretsen
EPI_ISL_480190 to 480195	Ota Health Center Welfare Section	Pathogen Genomics Center, National Institute of Infectious Diseases	Chika Takahashi; Hajime Kamiya; Kentaro Itokawa; Makoto Kuroda; Masanori Hashino; Motoi Suzuki; Rina Tanaka; Tsuyoshi Sekizuka
EPI_ISL_640017, EPI_ISL_640023, EPI_ISL_640077, EPI_ISL_700447, EPI_ISL_700575, EPI_ISL_700594	Oudtshoorn Hospital wc OUD	NHLS/UCT	Arash Iranzadeh; Bruna Galvao; Carolyn Williamson; Deelan Doolabh; Diana Hardie; Innocent Mudau; Kruger Marais; Lynn Tyers; Marvin Hsiao; Stephen Korsman
EPI_ISL_1035903, EPI_ISL_1035923, EPI_ISL_1035931 to 1035951	Outre Mer CH de Mayotte	National Reference Center for Viruses of Respiratory Infections, Institut Pasteur, Paris	Angela Brisebarre; Camille Capel; Combe Patrice; Etienne Simon-Lorière; Marion Barbet; Maud Vanpeene; Méline Bizard; Sylvie Behillil; Sylvie van der Werf; Vincent Enouf
EPI_ISL_912606, EPI_ISL_995333 to 995335, EPI_ISL_995337, EPI_ISL_995339, EPI_ISL_995344, EPI_ISL_995346, EPI_ISL_995349, EPI_ISL_995354 to 995355, EPI_ISL_995359, EPI_ISL_995361, EPI_ISL_995364, EPI_ISL_995367, EPI_ISL_995371 to 995372, EPI_ISL_995374 to 995376, EPI_ISL_995379, EPI_ISL_995382, EPI_ISL_995384 to 995386, EPI_ISL_995389 to 995390, EPI_ISL_995392 to 995401, EPI_ISL_995403 to 995404, EPI_ISL_995406, EPI_ISL_995409, EPI_ISL_995412, EPI_ISL_995416, EPI_ISL_995420, EPI_ISL_995423 to 995424, EPI_ISL_995428 to 995437, EPI_ISL_995439, EPI_ISL_995441 to 995446			
see above	Outre mer	National Reference Center for Viruses of Respiratory Infections, Institut Pasteur, Paris	Angela Brisebarre; Camille Capel; Combe Patrice; Etienne Simon-Lorière; Marion Barbet; Maud Vanpeene; Méline Bizard; Sylvie Behillil; Sylvie van der Werf; Vincent Enouf
EPI_ISL_448449 to 448451, EPI_ISL_448453 to 448463, EPI_ISL_448465, EPI_ISL_448467 to 448473, EPI_ISL_448476 to 448483, EPI_ISL_448485 to 448488, EPI_ISL_448490 to 448508, EPI_ISL_448510 to 448522, EPI_ISL_448525 to 448527, EPI_ISL_448529 to 448538, EPI_ISL_448540 to 448542, EPI_ISL_448545 to 448553, EPI_ISL_448555 to 448557, EPI_ISL_448559, EPI_ISL_448561, EPI_ISL_448563 to 448568, EPI_ISL_448570 to 448573, EPI_ISL_448575, EPI_ISL_448577, EPI_ISL_448579 to 448580, EPI_ISL_448582, EPI_ISL_448584 to 448585, EPI_ISL_448587 to 448588, EPI_ISL_448590 to 448614, EPI_ISL_448616 to 448640, EPI_ISL_448643 to 448646, EPI_ISL_448650 to 448652, EPI_ISL_448654 to 448664, EPI_ISL_448666 to 448676, EPI_ISL_448678 to 448682, EPI_ISL_448684 to 448692, EPI_ISL_448694 to 448720, EPI_ISL_448722 to 448740, EPI_ISL_448743 to 448747, EPI_ISL_448749 to 448752, EPI_ISL_448754 to 448760, EPI_ISL_448762 to 448797, EPI_ISL_448799 to 448804, EPI_ISL_448806 to 448812, EPI_ISL_448814, EPI_ISL_448816 to 448818, EPI_ISL_448820 to 448822, EPI_ISL_448824 to 448825, EPI_ISL_478729 to 478740, EPI_ISL_478742 to 478748, EPI_ISL_478752 to 478765, EPI_ISL_478768 to 478769, EPI_ISL_478771 to 478784, EPI_ISL_478786 to 478787, EPI_ISL_478790 to 478799, EPI_ISL_478801 to 478857, EPI_ISL_478859 to 478866, EPI_ISL_478868 to 478870, EPI_ISL_478872 to 478883, EPI_ISL_478885 to 478889, EPI_ISL_478891 to 478896, EPI_ISL_478898 to 478899, EPI_ISL_478902 to 478903, EPI_ISL_478905 to 478911, EPI_ISL_478913 to 478914, EPI_ISL_478917 to 478929, EPI_ISL_478931 to 478934, EPI_ISL_478936 to 478937, EPI_ISL_478939 to 478941, EPI_ISL_478943, EPI_ISL_478945, EPI_ISL_478948 to 478950, EPI_ISL_478952, EPI_ISL_478954 to 478957, EPI_ISL_478959 to 478963, EPI_ISL_478965, EPI_ISL_478967 to 478977, EPI_ISL_478979 to 478999, EPI_ISL_479001, EPI_ISL_479003, EPI_ISL_479005 to 479007, EPI_ISL_479011 to 479020, EPI_ISL_479022 to 479027, EPI_ISL_479030 to 479033, EPI_ISL_479035 to 479038, EPI_ISL_479040 to 479048, EPI_ISL_479050 to 479052, EPI_ISL_479054 to 479064, EPI_ISL_479066 to 479074, EPI_ISL_479076 to 479077, EPI_ISL_479079 to 479086, EPI_ISL_479088 to 479108, EPI_ISL_479110 to 479111, EPI_ISL_479113 to 479117, EPI_ISL_479119 to 479124, EPI_ISL_479126 to 479127, EPI_ISL_479130 to 479138, EPI_ISL_479140 to 479144, EPI_ISL_479146 to 479148, EPI_ISL_479150, EPI_ISL_479152 to 479158, EPI_ISL_479160, EPI_ISL_479162, EPI_ISL_479165 to 479174, EPI_ISL_534763 to 534772, EPI_ISL_534774 to 534787, EPI_ISL_534789 to 534793, EPI_ISL_534795 to 534811, EPI_ISL_534813 to 534817, EPI_ISL_534819 to 534823, EPI_ISL_534825 to 534831, EPI_ISL_534833 to 534863, EPI_ISL_534865 to 534868, EPI_ISL_534870 to 534876, EPI_ISL_534878 to 534907, EPI_ISL_534909 to 534915, EPI_ISL_534917 to 534918, EPI_ISL_534920 to 534922, EPI_ISL_534924 to 534935, EPI_ISL_534938 to 534939, EPI_ISL_534941 to 534945, EPI_ISL_534947 to 534948, EPI_ISL_534950, EPI_ISL_534952 to 534955, EPI_ISL_534958 to 534961, EPI_ISL_534963 to 534964, EPI_ISL_534966 to 534967, EPI_ISL_534969 to 534973, EPI_ISL_534975 to 534976, EPI_ISL_534978, EPI_ISL_534980 to 534990, EPI_ISL_534993 to 534998, EPI_ISL_535000 to 535015, EPI_ISL_535017 to 535023, EPI_ISL_549329, EPI_ISL_549333, EPI_ISL_549342, EPI_ISL_549345 to 549348, EPI_ISL_549396 to 549459, EPI_ISL_549523 to 549546, EPI_ISL_559753 to 559784, EPI_ISL_559786 to 559794, EPI_ISL_559796 to 559797, EPI_ISL_559799 to 559824, EPI_ISL_559826 to 559832, EPI_ISL_559834 to 559839, EPI_ISL_559841 to 559845, EPI_ISL_559847 to 559879, EPI_ISL_559881 to 559885, EPI_ISL_559887 to 559897, EPI_ISL_559984 to 559987, EPI_ISL_559989 to 559997, EPI_ISL_559999 to 560053, EPI_ISL_560069 to 560082, EPI_ISL_560305 to 560306, EPI_ISL_572714, EPI_ISL_572723 to 572724, EPI_ISL_572726 to 572728, EPI_ISL_572846, EPI_ISL_572849 to 572850, EPI_ISL_572857 to 572861, EPI_ISL_572869 to 572870, EPI_ISL_572873, EPI_ISL_572947, EPI_ISL_572958, EPI_ISL_572960, EPI_ISL_572962 to 573084, EPI_ISL_573154 to 573235, EPI_ISL_573566 to 573651, EPI_ISL_573759, EPI_ISL_573811 to 573855, EPI_ISL_573866 to 573869, EPI_ISL_574257, EPI_ISL_576569 to 576585, EPI_ISL_576587, EPI_ISL_576589 to 576599, EPI_ISL_576601 to 576610, EPI_ISL_576613 to 576619, EPI_ISL_576621 to 576624, EPI_ISL_576626, EPI_ISL_576628, EPI_ISL_576630 to 576643, EPI_ISL_576646 to 576671, EPI_ISL_576674 to 576676, EPI_ISL_576680 to 576694, EPI_ISL_576696 to 576701, EPI_ISL_576703, EPI_ISL_576705 to 576722, EPI_ISL_576724 to 576725, EPI_ISL_576727 to 576748, EPI_ISL_576750 to 576752, EPI_ISL_576754 to 576755, EPI_ISL_576757 to 576766, EPI_ISL_576768 to 576790, EPI_ISL_576792 to 576808, EPI_ISL_576810 to 576816, EPI_ISL_576818 to 576851, EPI_ISL_576853 to 576855, EPI_ISL_576857 to 576859, EPI_ISL_576861, EPI_ISL_576863 to 576865, EPI_ISL_576867 to 576872, EPI_ISL_576874 to 576876, EPI_ISL_576920 to 576928, EPI_ISL_576930 to 576944, EPI_ISL_576946 to 576956, EPI_ISL_576958 to 576960, EPI_ISL_577068 to 577078, EPI_ISL_577080 to 577095, EPI_ISL_577383 to 577384, EPI_ISL_577401 to 577411, EPI_ISL_577413 to 577416, EPI_ISL_584680, EPI_ISL_594468 to 594470, EPI_ISL_594472, EPI_ISL_594474 to 594475, EPI_ISL_594477 to 594483, EPI_ISL_594617 to 594620, EPI_ISL_594622 to 594636, EPI_ISL_594638 to 594644, EPI_ISL_594890 to 594893, EPI_ISL_594895 to 594914, EPI_ISL_594916 to 594922, EPI_ISL_595326 to 595328, EPI_ISL_595617 to 595660, EPI_ISL_595662 to 595670, EPI_ISL_595672 to 595689, EPI_ISL_595691 to 595727, EPI_ISL_595729, EPI_ISL_595731 to 595747, EPI_ISL_595749 to 595751, EPI_ISL_595753 to 595755, EPI_ISL_595757 to 595798, EPI_ISL_627079, EPI_ISL_627085, EPI_ISL_627250 to 627251, EPI_ISL_627255 to 627262, EPI_ISL_627292, EPI_ISL_628334 to 628368, EPI_ISL_628414 to 628420, EPI_ISL_628422 to 628700, EPI_ISL_637855, EPI_ISL_637858, EPI_ISL_637861, EPI_ISL_637867, EPI_ISL_638037, EPI_ISL_638073 to 638078, EPI_ISL_638086 to 638089, EPI_ISL_638170 to 638171, EPI_ISL_638173, EPI_ISL_638175, EPI_ISL_638207, EPI_ISL_638459 to 638531, EPI_ISL_638736 to 638876, EPI_ISL_638894, EPI_ISL_638944 to 639001, EPI_ISL_639627 to 639630, EPI_ISL_651086 to 651088, EPI_ISL_651103 to 651106, EPI_ISL_651417, EPI_ISL_651421, EPI_ISL_651431 to 651433, EPI_ISL_651469 to 651483, EPI_ISL_651525, EPI_ISL_651529, EPI_ISL_652117 to 652150, EPI_ISL_652711 to 653026, EPI_ISL_679629 to 679640, EPI_ISL_679642 to 679650, EPI_ISL_679652, EPI_ISL_679654 to 679660, EPI_ISL_679662 to 679663, EPI_ISL_679665 to 679687, EPI_ISL_679689 to 679696, EPI_ISL_679698 to 679722, EPI_ISL_679724 to 679747, EPI_ISL_679750 to 679760, EPI_ISL_679762, EPI_ISL_679766, EPI_ISL_679769 to 679770, EPI_ISL_679772 to 679773, EPI_ISL_679777, EPI_ISL_679779 to 679783, EPI_ISL_679785 to 679786, EPI_ISL_679788 to 679815, EPI_ISL_679817, EPI_ISL_679819 to 679826, EPI_ISL_679828 to 679840, EPI_ISL_679842 to 679851, EPI_ISL_680579, EPI_ISL_704594, EPI_ISL_704812, EPI_ISL_704815, EPI_ISL_704817, EPI_ISL_704845, EPI_ISL_704861, EPI_ISL_704864, EPI_ISL_704866, EPI_ISL_704869, EPI_ISL_704872, EPI_ISL_704874, EPI_ISL_704877, EPI_ISL_704879, EPI_ISL_704882, EPI_ISL_704885, EPI_ISL_705417, EPI_ISL_705426 to 705430, EPI_ISL_705443 to 705444, EPI_ISL_705473 to 705485, EPI_ISL_705489, EPI_ISL_705972 to 706193, EPI_ISL_706195 to 706275, EPI_ISL_706891 to 706974, EPI_ISL_723631 to 723649, EPI_ISL_723651 to 723655, EPI_ISL_723657 to 723667, EPI_ISL_723669 to 723672, EPI_ISL_723674 to 723675, EPI_ISL_723677 to 723695, EPI_ISL_723697 to 723710, EPI_ISL_723712 to 723723, EPI_ISL_723725 to 723751, EPI_ISL_723753 to 723766, EPI_ISL_723768 to 723770, EPI_ISL_723772 to 723793, EPI_ISL_723795 to 723841, EPI_ISL_723843 to 723845, EPI_ISL_723847 to 723850, EPI_ISL_723950 to 723954, EPI_ISL_723956, EPI_ISL_723958 to 723961, EPI_ISL_723965 to 723973, EPI_ISL_723975 to 723977, EPI_ISL_723980 to 724006, EPI_ISL_724011 to 724021, EPI_ISL_724023 to 724026, EPI_ISL_724028 to 724032, EPI_ISL_724035 to 724036, EPI_ISL_724039 to 724045, EPI_ISL_724049 to 724050, EPI_ISL_724053 to 724062, EPI_ISL_724778, EPI_ISL_724780 to 724788, EPI_ISL_724790 to 724796, EPI_ISL_724799 to 724803, EPI_ISL_724805, EPI_ISL_724808 to 724814, EPI_ISL_724816, EPI_ISL_724820 to 724822, EPI_ISL_724824 to 724826, EPI_ISL_724830, EPI_ISL_724832 to 724835, EPI_ISL_727807 to 727837, EPI_ISL_727839 to 727856, EPI_ISL_727858 to 727860, EPI_ISL_740897 to 740904, EPI_ISL_740906 to 740929, EPI_ISL_740931 to 740937, EPI_ISL_740939 to 740941, EPI_ISL_740943 to 740952, EPI_ISL_741018 to 741021, EPI_ISL_741024 to 741028, EPI_ISL_741030 to 741036, EPI_ISL_741038, EPI_ISL_741040 to 741041, EPI_ISL_741043, EPI_ISL_741047, EPI_ISL_741051 to 741057, EPI_ISL_741059 to 741060, EPI_ISL_741062 to 741066, EPI_ISL_741068 to 741070, EPI_ISL_741073 to 741082, EPI_ISL_741084 to 741092, EPI_ISL_741094, EPI_ISL_741096 to 741103, EPI_ISL_741105 to 741108, EPI_ISL_741110 to 741114, EPI_ISL_741117 to 741125, EPI_ISL_741127 to 741128, EPI_ISL_741131 to 741134, EPI_ISL_741136 to 741145, EPI_ISL_741147 to 741156, EPI_ISL_741158 to 741159, EPI_ISL_741161 to 741164, EPI_ISL_741166, EPI_ISL_741169 to 741171, EPI_ISL_741173, EPI_ISL_741175 to 741181, EPI_ISL_741183 to 741185, EPI_ISL_741349 to 741356, EPI_ISL_741362 to 741378, EPI_ISL_741380 to 741399, EPI_ISL_741401 to 741417, EPI_ISL_741419 to 741420, EPI_ISL_741760 to 741771, EPI_ISL_741773 to 741784, EPI_ISL_741786 to 741804, EPI_ISL_741806 to 741811, EPI_ISL_741813 to 741833, EPI_ISL_741835 to 741836, EPI_ISL_741838, EPI_ISL_741840 to 741865, EPI_ISL_741867 to 741897, EPI_ISL_741899 to 741917, EPI_ISL_741919 to 741931, EPI_ISL_741933 to 741935, EPI_ISL_742247 to 742259, EPI_ISL_763838, EPI_ISL_763841, EPI_ISL_763844 to 763846, EPI_ISL_763859 to 763863, EPI_ISL_763928 to 763974, EPI_ISL_764077 to 764079, EPI_ISL_764085 to 764093, EPI_ISL_764117, EPI_ISL_764243, EPI_ISL_764525 to 764572, EPI_ISL_814466 to 814467, EPI_ISL_819578, EPI_ISL_819585 to 819587, EPI_ISL_819590 to 819593, EPI_ISL_819613 to 819646, EPI_ISL_819675 to 819685, EPI_ISL_819689 to 819706, EPI_ISL_819708 to 819709, EPI_ISL_819712 to 819718, EPI_ISL_819759 to 819762, EPI_ISL_819764 to 819794, EPI_ISL_819822 to 819827, EPI_ISL_819852 to 819863, EPI_ISL_839369, EPI_ISL_839381 to 839382, EPI_ISL_839384, EPI_ISL_839445, EPI_ISL_840222 to 840223, EPI_ISL_840249, EPI_ISL_840252 to 840253, EPI_ISL_840257, EPI_ISL_840259 to 840260, EPI_ISL_840263 to 840267, EPI_ISL_840270, EPI_ISL_840274, EPI_ISL_840276, EPI_ISL_840281 to 840283, EPI_ISL_840288 to 840291, EPI_ISL_840294 to 840295, EPI_ISL_840299 to 840304, EPI_ISL_840306 to 840307, EPI_ISL_840310 to 840313, EPI_ISL_840315, EPI_ISL_840320, EPI_ISL_840322, EPI_ISL_840325, EPI_ISL_840327, EPI_ISL_840330 to 840333, EPI_ISL_840336, EPI_ISL_840339, EPI_ISL_840341 to 840342, EPI_ISL_840344, EPI_ISL_840348, EPI_ISL_840350 to 840351, EPI_ISL_840353, EPI_ISL_840357 to 840358, EPI_ISL_840360 to 840363, EPI_ISL_840365 to 840366, EPI_ISL_867037 to 867040, EPI_ISL_867045 to 867046, EPI_ISL_867048 to 867051, EPI_ISL_867055 to 867058, EPI_ISL_867061, EPI_ISL_867063 to 867069, EPI_ISL_867071 to 867074, EPI_ISL_867077 to 867081, EPI_ISL_867084, EPI_ISL_867089 to 867097, EPI_ISL_867099 to 867100, EPI_ISL_867106 to 867108, EPI_ISL_867114, EPI_ISL_867118 to 867119, EPI_ISL_867127, EPI_ISL_867129, EPI_ISL_867131 to 867134, EPI_ISL_867137 to 867138, EPI_ISL_867140 to 867141, EPI_ISL_867144 to 867145, EPI_ISL_867147 to 867148, EPI_ISL_867155, EPI_ISL_867163 to 867167, EPI_ISL_867169 to 867170, EPI_ISL_867173 to 867175, EPI_ISL_867177 to 867179, EPI_ISL_867182, EPI_ISL_867185, EPI_ISL_922172 to 922173, EPI_ISL_922177 to 922178, EPI_ISL_922180, EPI_ISL_922182 to 922185, EPI_ISL_922190, EPI_ISL_922192, EPI_ISL_922197 to 922201, EPI_ISL_922203, EPI_ISL_922206 to 922209, EPI_ISL_922211 to 922214, EPI_ISL_922216 to 922220, EPI_ISL_922223 to 922226, EPI_ISL_922229 to 922234, EPI_ISL_922238 to 922242, EPI_ISL_922244, EPI_ISL_922246, EPI_ISL_922249, EPI_ISL_922255, EPI_ISL_922258 to 922264, EPI_ISL_922267 to 922269, EPI_ISL_922272 to 922274, EPI_ISL_922276 to 922277, EPI_ISL_922279, EPI_ISL_922281 to 922293, EPI_ISL_922296 to 922297, EPI_ISL_922299, EPI_ISL_922302, EPI_ISL_922304, EPI_ISL_922307 to 922309, EPI_ISL_922311, EPI_ISL_922313, EPI_ISL_922317, EPI_ISL_922321, EPI_ISL_922323 to 922330, EPI_ISL_922333 to 922336, EPI_ISL_922338 to 922340, EPI_ISL_922342 to 922343, EPI_ISL_922345 to 922349, EPI_ISL_950764 to 950767, EPI_ISL_950772 to 950778, EPI_ISL_950780, EPI_ISL_950783, EPI_ISL_950785, EPI_ISL_950789 to 950791, EPI_ISL_950793 to 950796, EPI_ISL_950798 to 950801, EPI_ISL_950805 to 950811, EPI_ISL_950814, EPI_ISL_950820, EPI_ISL_950825 to 950826, EPI_ISL_950831 to 950834, EPI_ISL_950836 to 950838, EPI_ISL_950840, EPI_ISL_950842 to 950843, EPI_ISL_950846, EPI_ISL_950848, EPI_ISL_950850, EPI_ISL_950853 to 950857, EPI_ISL_950861 to 950862, EPI_ISL_950868 to 950869, EPI_ISL_950874 to 950877, EPI_ISL_950879 to 950881, EPI_ISL_950884 to 950887, EPI_ISL_950889 to 950893, EPI_ISL_950897 to 950898, EPI_ISL_950904, EPI_ISL_950908, EPI_ISL_950910 to 950912, EPI_ISL_950914, EPI_ISL_950917 to 950918, EPI_ISL_950924 to 950929, EPI_ISL_950931, EPI_ISL_950933 to 950936, EPI_ISL_950938 to 950941, EPI_ISL_950943, EPI_ISL_950946, EPI_ISL_950948 to 950950, EPI_ISL_950952 to 950955, EPI_ISL_950958 to 950959, EPI_ISL_950964 to 950966, EPI_ISL_950968, EPI_ISL_950970 to 950971, EPI_ISL_950974, EPI_ISL_950976, EPI_ISL_950979 to 950983, EPI_ISL_950987, EPI_ISL_950991 to 950997, EPI_ISL_950999, EPI_ISL_951002 to 951006, EPI_ISL_951009, EPI_ISL_951011 to 951016, EPI_ISL_951020 to 951021, EPI_ISL_951024 to 951029, EPI_ISL_951032 to 951034, EPI_ISL_951036 to 951038, EPI_ISL_951043 to 951046, EPI_ISL_951053, EPI_ISL_951056 to 951059, EPI_ISL_951061, EPI_ISL_951064, EPI_ISL_951066, EPI_ISL_951068 to 951070, EPI_ISL_951073 to 951075, EPI_ISL_951079 to 951085, EPI_ISL_951087 to 951088, EPI_ISL_951090, EPI_ISL_951093 to 951094, EPI_ISL_951098, EPI_ISL_951102, EPI_ISL_951104 to 951105, EPI_ISL_951110 to 951112, EPI_ISL_951114 to 951118, EPI_ISL_951120, EPI_ISL_951122, EPI_ISL_951125 to 951126, EPI_ISL_951131 to 951132, EPI_ISL_951134, EPI_ISL_951136 to 951139, EPI_ISL_951141, EPI_ISL_951144 to 951145, EPI_ISL_951147, EPI_ISL_951149, EPI_ISL_951152 to 951155, EPI_ISL_951159 to 951160, EPI_ISL_951162 to 951163, EPI_ISL_951165 to 951166, EPI_ISL_951169, EPI_ISL_951171 to 951173, EPI_ISL_951176, EPI_ISL_951178, EPI_ISL_951181, EPI_ISL_951184 to 951189, EPI_ISL_951191 to 951192, EPI_ISL_951196, EPI_ISL_951204 to 951207, EPI_ISL_951211, EPI_ISL_951213 to 951214, EPI_ISL_951218, EPI_ISL_951223 to 951225, EPI_ISL_951227 to 951230, EPI_ISL_951233 to 951235, EPI_ISL_951237 to 951238, EPI_ISL_951241, EPI_ISL_951245 to 951246, EPI_ISL_951249,			

EPI_ISL_951252, EPI_ISL_951254, EPI_ISL_951258, EPI_ISL_951264, EPI_ISL_951267, EPI_ISL_951270 to 951274, EPI_ISL_951276 to 951277, EPI_ISL_951281 to 951283, EPI_ISL_951287 to 951291, EPI_ISL_951293 to 951294, EPI_ISL_951297, EPI_ISL_951299 to 951301, EPI_ISL_951304, EPI_ISL_951307 to 951309, EPI_ISL_951311, EPI_ISL_951313, EPI_ISL_951316 to 951317, EPI_ISL_951319 to 951320, EPI_ISL_951323 to 951324, EPI_ISL_951326 to 951329, EPI_ISL_951331, EPI_ISL_951333 to 951336, EPI_ISL_951338 to 951344, EPI_ISL_951346 to 951347, EPI_ISL_951349, EPI_ISL_951351 to 951353, EPI_ISL_951356, EPI_ISL_951359 to 951362, EPI_ISL_951364, EPI_ISL_951366 to 951368, EPI_ISL_951370 to 951373, EPI_ISL_951376, EPI_ISL_951378 to 951384, EPI_ISL_951386 to 951389, EPI_ISL_951391, EPI_ISL_951395 to 951398, EPI_ISL_951400 to 951402, EPI_ISL_951404 to 951409, EPI_ISL_951412 to 951417, EPI_ISL_951421, EPI_ISL_951423 to 951429, EPI_ISL_951435 to 951438, EPI_ISL_951442, EPI_ISL_951444 to 951447, EPI_ISL_951451 to 951453, EPI_ISL_951456 to 951459, EPI_ISL_951461 to 951462, EPI_ISL_951464, EPI_ISL_951466, EPI_ISL_951468 to 951471, EPI_ISL_951475 to 951482, EPI_ISL_951484 to 951485, EPI_ISL_951487, EPI_ISL_951489, EPI_ISL_998986 to 998988, EPI_ISL_998992, EPI_ISL_998994, EPI_ISL_998996 to 998997, EPI_ISL_999000 to 999002, EPI_ISL_999004 to 999007, EPI_ISL_999009 to 999010, EPI_ISL_999013 to 999014, EPI_ISL_999018 to 999021, EPI_ISL_999023, EPI_ISL_999025, EPI_ISL_999029, EPI_ISL_999031, EPI_ISL_999033 to 999041, EPI_ISL_999044, EPI_ISL_999048 to 999051, EPI_ISL_999054 to 999067, EPI_ISL_999069, EPI_ISL_999071, EPI_ISL_999077 to 999078, EPI_ISL_999081, EPI_ISL_999083 to 999089, EPI_ISL_999093 to 999095, EPI_ISL_999099, EPI_ISL_999101 to 999106, EPI_ISL_999108 to 999109, EPI_ISL_999111 to 999112, EPI_ISL_999116, EPI_ISL_999119 to 999129, EPI_ISL_999132 to 999133, EPI_ISL_999135 to 999139, EPI_ISL_999141 to 999146, EPI_ISL_999148, EPI_ISL_999151 to 999154, EPI_ISL_999157 to 999163, EPI_ISL_999165, EPI_ISL_999168 to 999170, EPI_ISL_999173 to 999175, EPI_ISL_999177 to 999180, EPI_ISL_999182 to 999184, EPI_ISL_999187 to 999192, EPI_ISL_999196 to 999198, EPI_ISL_999200, EPI_ISL_999203 to 999205, EPI_ISL_999207 to 999208, EPI_ISL_999210 to 999213, EPI_ISL_999215 to 999217, EPI_ISL_999219, EPI_ISL_999222 to 999230, EPI_ISL_999232 to 999236, EPI_ISL_999238 to 999239, EPI_ISL_999241 to 999243, EPI_ISL_999246, EPI_ISL_999248 to 999249, EPI_ISL_999251 to 999254, EPI_ISL_999256 to 999259, EPI_ISL_999262 to 999263, EPI_ISL_999265, EPI_ISL_999267 to 999268, EPI_ISL_999274, EPI_ISL_999276, EPI_ISL_999281 to 999284, EPI_ISL_999287, EPI_ISL_999289 to 999293, EPI_ISL_999296 to 999301, EPI_ISL_999303, EPI_ISL_999307, EPI_ISL_999310 to 999311, EPI_ISL_999314, EPI_ISL_999317 to 999318, EPI_ISL_999320, EPI_ISL_999322, EPI_ISL_999324 to 999332, EPI_ISL_999335, EPI_ISL_999337, EPI_ISL_999339 to 999340, EPI_ISL_999342 to 999352, EPI_ISL_999355, EPI_ISL_999360, EPI_ISL_999362, EPI_ISL_999364 to 999365, EPI_ISL_999368 to 999374, EPI_ISL_999378 to 999379, EPI_ISL_999381 to 999386, EPI_ISL_999388, EPI_ISL_999390 to 999391, EPI_ISL_999393 to 999397, EPI_ISL_999399, EPI_ISL_999401 to 999402, EPI_ISL_999404 to 999408, EPI_ISL_999410 to 999414, EPI_ISL_999419 to 999420, EPI_ISL_999425 to 999426, EPI_ISL_999428, EPI_ISL_999431 to 999434, EPI_ISL_999437 to 999438, EPI_ISL_999440, EPI_ISL_999443 to 999447, EPI_ISL_999449, EPI_ISL_999452 to 999456, EPI_ISL_999458, EPI_ISL_999463, EPI_ISL_999465 to 999476, EPI_ISL_999478 to 999479, EPI_ISL_999482 to 999491, EPI_ISL_999493 to 999494, EPI_ISL_999496 to 999497, EPI_ISL_999499 to 999503, EPI_ISL_999505, EPI_ISL_999507, EPI_ISL_999509 to 999514			
see above	Oxford Viromics, NDM, University of Oxford; Oxford University Hospitals; Basingstoke and North Hampshire Hospital	COVID-19 Genomics UK (COG-UK) Consortium	Alex Mobbs; Amy Trebes; Anita Justice; Catrin Moore; Christophe Fraser; David Bonsall; David Buck; Emma Wise; George Macintyre; Jessica Lynch; John Todd; Mariateresa de Cesare; Matilde Mori; Monique Andersson; Nathan Moore; Nick Cortes; Robert Shaw; Stephen Kidd; Tanya Golubchik; Timothy Peto
EPI_ISL_965280 to 965300	P.O.CARDARELLI	Istituto Zooprofilattico Sperimentale dell'Abruzzo e Molise "G. Caporale"	Ancora M; Calistri P; Cammà C; Curini V; Di Domenico M; Di Pasquale A; Lorusso A; Mangone I; Marcacci M; Niro G; Puglia I; Rinaldi A; Savini G; Scialabba S; Scutellà M
EPI_ISL_424881 to 424886, EPI_ISL_426432 to 426434, EPI_ISL_447844, EPI_ISL_450802 to 450803, EPI_ISL_576170 to 576171	PA Department of Health, Bureau of Laboratories	Pathogen Discovery, Respiratory Viruses Branch, Division of Viral Diseases, Centers for Disease Control and Prevention	Alison S. Laufer Halpin; Anna Montmayeur; Anna Uehara; Bettina Bankamp; Brian Lynch; Christopher A. Elkins; Clinton R. Paden; Haibin Wang; Jasmine Padilla; Jing Zhang; Justin Lee; Krista Queen; Mary S. Keckler; Peter Cook; Rachel Marine; Suxiang Tong; Yan Li; Ying Tao; Zachary Weiner
EPI_ISL_767877, EPI_ISL_767897	PALMS	NSW Health Pathology - Institute of Clinical Pathology and Medical Research; Westmead Hospital; University of Sydney	CIDM-PH et al.
EPI_ISL_482028	PHE South West Regional Laboratory, National Infection Service	Wellcome Sanger Institute for the COVID-19 Genomics UK (COG-UK) Consortium	Barry Vipond; Cordelia Langford; David K. Jackson; Dominic Kwiatkowski; Dr Peter Muir; Ewan Harrison; Hannah Pymont; Ian Johnston; John Sillitoe on behalf of the Wellcome Sanger Institute COVID-19 Surveillance Team; Rich Hopes; Roberto Amato; Sonia Goncalves; Stephanie Hutchings; and Alex Alderton
EPI_ISL_439957, EPI_ISL_439959, EPI_ISL_439964 to 439967, EPI_ISL_439969, EPI_ISL_439971 to 439974, EPI_ISL_439978 to 439979, EPI_ISL_439981 to 439986, EPI_ISL_439988, EPI_ISL_439992, EPI_ISL_439998, EPI_ISL_440001 to 440002, EPI_ISL_440005 to 440006, EPI_ISL_440008, EPI_ISL_440013, EPI_ISL_440018, EPI_ISL_440020 to 440023, EPI_ISL_440027, EPI_ISL_440029, EPI_ISL_440032 to 440033, EPI_ISL_440037 to 440039, EPI_ISL_440042, EPI_ISL_440050, EPI_ISL_440052, EPI_ISL_440054, EPI_ISL_440056, EPI_ISL_440063, EPI_ISL_440065, EPI_ISL_440068, EPI_ISL_440070 to 440071, EPI_ISL_440081, EPI_ISL_440087, EPI_ISL_440091 to 440095, EPI_ISL_440098, EPI_ISL_440100 to 440103, EPI_ISL_440105 to 440106, EPI_ISL_440114 to 440116, EPI_ISL_440123 to 440124, EPI_ISL_440126 to 440128, EPI_ISL_440135, EPI_ISL_440139, EPI_ISL_440141, EPI_ISL_440149, EPI_ISL_440156, EPI_ISL_440158, EPI_ISL_440162, EPI_ISL_440167 to 440168, EPI_ISL_440178, EPI_ISL_440187, EPI_ISL_440189, EPI_ISL_440194, EPI_ISL_440196, EPI_ISL_440198, EPI_ISL_440201, EPI_ISL_440206, EPI_ISL_440214, EPI_ISL_440218 to 440219, EPI_ISL_440222, EPI_ISL_440224 to 440225, EPI_ISL_440232 to 440233, EPI_ISL_440236, EPI_ISL_440241, EPI_ISL_440243, EPI_ISL_440248, EPI_ISL_440250, EPI_ISL_440255 to 440256, EPI_ISL_440269, EPI_ISL_440272, EPI_ISL_440275, EPI_ISL_440286, EPI_ISL_440291, EPI_ISL_440298 to 440299, EPI_ISL_440624, EPI_ISL_440626, EPI_ISL_440628, EPI_ISL_440632, EPI_ISL_440634 to 440636, EPI_ISL_440639 to 440643, EPI_ISL_440645 to 440647, EPI_ISL_440649 to 440650, EPI_ISL_440653, EPI_ISL_440655 to 440660, EPI_ISL_440662 to 440667, EPI_ISL_440670 to 440671, EPI_ISL_440673 to 440674, EPI_ISL_440676, EPI_ISL_440679, EPI_ISL_440681 to 440685, EPI_ISL_440688 to 440692, EPI_ISL_440694 to 440695, EPI_ISL_440697 to 440705, EPI_ISL_440707, EPI_ISL_440710 to 440712, EPI_ISL_440715 to 440719, EPI_ISL_440721, EPI_ISL_440723 to 440724, EPI_ISL_440726, EPI_ISL_440728 to 440730, EPI_ISL_440732 to 440742, EPI_ISL_440744 to 440747, EPI_ISL_440751 to 440755, EPI_ISL_440757 to 440759, EPI_ISL_440761 to 440768, EPI_ISL_440770, EPI_ISL_440773 to 440777, EPI_ISL_440779, EPI_ISL_440781, EPI_ISL_440783 to 440785, EPI_ISL_440787 to 440791, EPI_ISL_440793 to 440796, EPI_ISL_440799 to 440804, EPI_ISL_440806 to 440809, EPI_ISL_442624 to 442630, EPI_ISL_442632 to 442634, EPI_ISL_442636 to 442641, EPI_ISL_442643 to 442650, EPI_ISL_442652 to 442658, EPI_ISL_442660, EPI_ISL_442662 to 442665, EPI_ISL_442667 to 442676, EPI_ISL_442679 to 442686, EPI_ISL_442688 to 442692, EPI_ISL_442694 to 442697, EPI_ISL_442699 to 442702, EPI_ISL_442704 to 442714, EPI_ISL_442718 to 442720, EPI_ISL_442722, EPI_ISL_442724 to 442732, EPI_ISL_442734 to 442737, EPI_ISL_442739 to 442745, EPI_ISL_442747 to 442749, EPI_ISL_442751 to 442765, EPI_ISL_442767 to 442775, EPI_ISL_442777 to 442797, EPI_ISL_442799 to 442802, EPI_ISL_442804 to 442805, EPI_ISL_442807 to 442810, EPI_ISL_443686 to 443687, EPI_ISL_443690 to 443692, EPI_ISL_443697 to 443699, EPI_ISL_443701 to 443702, EPI_ISL_443704, EPI_ISL_443706 to 443707, EPI_ISL_443709 to 443711, EPI_ISL_443714 to 443720, EPI_ISL_443726, EPI_ISL_443729 to 443730, EPI_ISL_443733, EPI_ISL_443738 to 443749, EPI_ISL_443751 to 443752, EPI_ISL_443757 to 443760, EPI_ISL_443763 to 443765, EPI_ISL_443768 to 443770, EPI_ISL_443772 to 443778, EPI_ISL_443780 to 443784, EPI_ISL_443786 to 443791, EPI_ISL_443793, EPI_ISL_443795 to 443798, EPI_ISL_443800 to 443805, EPI_ISL_443807 to 443809, EPI_ISL_443811 to 443813, EPI_ISL_443815 to 443817, EPI_ISL_443819 to 443821, EPI_ISL_443825 to 443828, EPI_ISL_443830 to 443836, EPI_ISL_443838 to 443844, EPI_ISL_443846 to 443847, EPI_ISL_443851 to 443860, EPI_ISL_443862, EPI_ISL_443864 to 443867, EPI_ISL_443869 to 443873, EPI_ISL_443876 to 443877, EPI_ISL_443879 to 443885, EPI_ISL_443888, EPI_ISL_443891 to 443899, EPI_ISL_443901 to 443902, EPI_ISL_443904, EPI_ISL_443906 to 443907, EPI_ISL_443909 to 443912, EPI_ISL_443914 to 443916, EPI_ISL_443918 to 443919, EPI_ISL_443922, EPI_ISL_443924 to 443925, EPI_ISL_443927 to 443929, EPI_ISL_443933, EPI_ISL_443935 to 443936, EPI_ISL_443938 to 443959, EPI_ISL_443961 to 443964, EPI_ISL_443967 to 443973, EPI_ISL_443975 to 443976, EPI_ISL_443978, EPI_ISL_443980 to 443983, EPI_ISL_443985 to 443992, EPI_ISL_443996 to 444003, EPI_ISL_444006 to 444007, EPI_ISL_444009 to 444013, EPI_ISL_444015 to 444016, EPI_ISL_444018 to 444021, EPI_ISL_458300 to 458306, EPI_ISL_458308, EPI_ISL_458311, EPI_ISL_458313, EPI_ISL_458316 to 458317, EPI_ISL_458319 to 458321, EPI_ISL_458323 to 458324, EPI_ISL_458329 to 458330, EPI_ISL_458332 to 458334, EPI_ISL_458337 to 458339, EPI_ISL_458341, EPI_ISL_458343 to 458345, EPI_ISL_458348 to 458355, EPI_ISL_458357 to 458363, EPI_ISL_458365, EPI_ISL_458367 to 458368, EPI_ISL_458370 to 458372, EPI_ISL_458375, EPI_ISL_458377, EPI_ISL_458379, EPI_ISL_458381 to 458382, EPI_ISL_458384 to 458385, EPI_ISL_458391, EPI_ISL_458394 to 458397, EPI_ISL_458400, EPI_ISL_458403, EPI_ISL_458405 to 458407, EPI_ISL_458410, EPI_ISL_458412 to 458416, EPI_ISL_458419 to 458423, EPI_ISL_458425 to 458427, EPI_ISL_458430 to 458431, EPI_ISL_458434 to 458443, EPI_ISL_458445 to 458449, EPI_ISL_458451 to 458453, EPI_ISL_458455 to 458457, EPI_ISL_458459 to 458462, EPI_ISL_458465 to 458466, EPI_ISL_458468 to 458472, EPI_ISL_458474, EPI_ISL_458476, EPI_ISL_458479 to 458480, EPI_ISL_458483 to 458486, EPI_ISL_458489 to 458490, EPI_ISL_458492, EPI_ISL_458495 to 458497, EPI_ISL_458500 to 458503, EPI_ISL_458507, EPI_ISL_458509 to 458513, EPI_ISL_458515, EPI_ISL_458721 to 458723, EPI_ISL_458725 to 458728, EPI_ISL_458732 to 458733, EPI_ISL_458736, EPI_ISL_458739, EPI_ISL_458743, EPI_ISL_458745 to 458746, EPI_ISL_458748 to 458752, EPI_ISL_458755 to 458775, EPI_ISL_458777 to 458779, EPI_ISL_458781 to 458785, EPI_ISL_458787, EPI_ISL_458791 to 458794, EPI_ISL_458796 to 458799, EPI_ISL_458801, EPI_ISL_458803 to 458804, EPI_ISL_458808, EPI_ISL_458810 to 458811, EPI_ISL_458814 to 458817, EPI_ISL_458819 to 458822, EPI_ISL_458824 to 458827, EPI_ISL_458829 to 458833, EPI_ISL_458835 to 458836, EPI_ISL_458838 to 458840, EPI_ISL_458842, EPI_ISL_458844, EPI_ISL_458847 to 458848, EPI_ISL_458851, EPI_ISL_458854, EPI_ISL_458856, EPI_ISL_458858 to 458859, EPI_ISL_458861 to 458864, EPI_ISL_458866, EPI_ISL_458868, EPI_ISL_458871, EPI_ISL_458877 to 458880, EPI_ISL_458883, EPI_ISL_458885 to 458886, EPI_ISL_458888, EPI_ISL_458892, EPI_ISL_458894, EPI_ISL_458899, EPI_ISL_458903 to 458904, EPI_ISL_458908 to 458909, EPI_ISL_458911 to 458915, EPI_ISL_458917 to 458919, EPI_ISL_458922 to 458928, EPI_ISL_458930 to 458932, EPI_ISL_458934, EPI_ISL_458937 to 458939, EPI_ISL_458941, EPI_ISL_458943, EPI_ISL_458945 to 458947, EPI_ISL_458949 to 458953, EPI_ISL_458955 to 458957, EPI_ISL_458960 to 458967, EPI_ISL_458969, EPI_ISL_458971 to 458972, EPI_ISL_458974 to 458979, EPI_ISL_458981 to 458987, EPI_ISL_458989 to 458992, EPI_ISL_458994 to 458995, EPI_ISL_458998, EPI_ISL_459001 to 459002, EPI_ISL_459004 to 459008, EPI_ISL_459010, EPI_ISL_459012, EPI_ISL_459014 to 459017, EPI_ISL_459020 to 459022, EPI_ISL_459024 to 459030, EPI_ISL_459033 to 459036, EPI_ISL_459038, EPI_ISL_459040 to 459042, EPI_ISL_459046 to 459048, EPI_ISL_459050 to 459053, EPI_ISL_459055, EPI_ISL_459057 to 459061, EPI_ISL_459064 to 459066, EPI_ISL_459068 to 459071, EPI_ISL_459076 to 459083, EPI_ISL_459085 to 459086, EPI_ISL_459090 to 459091, EPI_ISL_459093, EPI_ISL_459725 to 459726, EPI_ISL_467695, EPI_ISL_467699, EPI_ISL_467708, EPI_ISL_467713, EPI_ISL_467718, EPI_ISL_469345 to 469350, EPI_ISL_469353 to 469362, EPI_ISL_469364 to 469366, EPI_ISL_469368 to 469370, EPI_ISL_469372 to 469373, EPI_ISL_469375 to 469377, EPI_ISL_469379 to 469392, EPI_ISL_469394 to 469398, EPI_ISL_469400 to 469410, EPI_ISL_469412 to 469424, EPI_ISL_469427 to 469428, EPI_ISL_469430 to 469432, EPI_ISL_469434 to 469440, EPI_ISL_469442 to 469446, EPI_ISL_469448 to 469449, EPI_ISL_469451 to 469459, EPI_ISL_469462 to 469466, EPI_ISL_469468, EPI_ISL_469470 to 469472, EPI_ISL_469474 to 469481, EPI_ISL_469483 to 469486, EPI_ISL_469488 to 469490, EPI_ISL_469492 to 469495, EPI_ISL_469497 to 469500, EPI_ISL_469502 to 469510, EPI_ISL_469513, EPI_ISL_469515 to 469521, EPI_ISL_469523 to 469526, EPI_ISL_469529, EPI_ISL_469531 to 469532, EPI_ISL_469535 to 469539, EPI_ISL_469543 to 469544, EPI_ISL_469546 to 469551, EPI_ISL_469553, EPI_ISL_469556 to 469560, EPI_ISL_469562, EPI_ISL_469564, EPI_ISL_469566, EPI_ISL_469568, EPI_ISL_469570 to 469571, EPI_ISL_469573 to 469574, EPI_ISL_469577, EPI_ISL_469579 to 469584, EPI_ISL_469586, EPI_ISL_469588, EPI_ISL_469591, EPI_ISL_469600, EPI_ISL_469603 to 469605, EPI_ISL_469607 to 469610, EPI_ISL_469612 to 469616, EPI_ISL_469621, EPI_ISL_469624 to 469626, EPI_ISL_469629 to 469630, EPI_ISL_469632, EPI_ISL_469634, EPI_ISL_469636 to 469639, EPI_ISL_469641, EPI_ISL_469643 to 469645, EPI_ISL_469649, EPI_ISL_469651 to 469652, EPI_ISL_469654, EPI_ISL_469657, EPI_ISL_469659, EPI_ISL_469661, EPI_ISL_469663 to 469668, EPI_ISL_469670, EPI_ISL_469674 to 469677, EPI_ISL_469679 to 469684, EPI_ISL_469686 to 469687, EPI_ISL_469691 to 469694, EPI_ISL_469696 to 469697, EPI_ISL_469700 to 469701, EPI_ISL_469704 to 469706, EPI_ISL_469708 to 469715, EPI_ISL_469720 to 469721, EPI_ISL_469723 to 469728, EPI_ISL_469730 to 469731, EPI_ISL_469733, EPI_ISL_469735, EPI_ISL_469737 to 469747, EPI_ISL_469749 to 469750, EPI_ISL_469752 to 469753, EPI_ISL_469755, EPI_ISL_469758, EPI_ISL_469760, EPI_ISL_469763, EPI_ISL_469765 to 469768, EPI_ISL_469771 to 469775, EPI_ISL_469777, EPI_ISL_469779, EPI_ISL_469781 to 469782, EPI_ISL_469785 to 469789, EPI_ISL_469792 to 469793, EPI_ISL_469798 to 469799, EPI_ISL_469801 to 469804, EPI_ISL_469809 to 469811, EPI_ISL_469814, EPI_ISL_469818 to 469820, EPI_ISL_469822, EPI_ISL_469827, EPI_ISL_469829, EPI_ISL_469831, EPI_ISL_469833, EPI_ISL_469837, EPI_ISL_469840 to 469841, EPI_ISL_469914, EPI_ISL_470356 to 470357, EPI_ISL_470529 to 470530, EPI_ISL_481768 to 481777, EPI_ISL_481779, EPI_ISL_481781 to 481789, EPI_ISL_481791 to 481794, EPI_ISL_481797, EPI_ISL_481799 to 481801, EPI_ISL_481803, EPI_ISL_481806 to 481808, EPI_ISL_481810 to 481813, EPI_ISL_481815, EPI_ISL_481817 to 481826, EPI_ISL_481828, EPI_ISL_481830 to 481831, EPI_ISL_481833 to 481834, EPI_ISL_481836 to 481837, EPI_ISL_481839 to 481840, EPI_ISL_481842 to 481843, EPI_ISL_481845 to 481848, EPI_ISL_481850 to 481859, EPI_ISL_481861 to 481863, EPI_ISL_481865 to 481871, EPI_ISL_481873 to 481874, EPI_ISL_481876 to 481879, EPI_ISL_481882 to 481884, EPI_ISL_481886 to 481888, EPI_ISL_481890 to 481891, EPI_ISL_481893 to 481895, EPI_ISL_481898 to 481907, EPI_ISL_481909 to 481915, EPI_ISL_481917 to 481922, EPI_ISL_481924 to 481927, EPI_ISL_481929 to 481936, EPI_ISL_481938 to 481943, EPI_ISL_481945 to 481952, EPI_ISL_481954, EPI_ISL_481957 to 481958, EPI_ISL_481960 to 481962, EPI_ISL_481964, EPI_ISL_481966, EPI_ISL_481968 to 481969, EPI_ISL_481971 to 481977, EPI_ISL_481984, EPI_ISL_481991, EPI_ISL_481995 to 481996, EPI_ISL_481998 to 482005, EPI_ISL_482007, EPI_ISL_482009 to 482011, EPI_ISL_482013, EPI_ISL_482015, EPI_ISL_482017 to 482018, EPI_ISL_482020 to 482021, EPI_ISL_482025 to 482027, EPI_ISL_482030, EPI_ISL_487524, EPI_ISL_487526 to 487528, EPI_ISL_487530 to 487533, EPI_ISL_487535 to 487536, EPI_ISL_487538 to 487539, EPI_ISL_487541 to 487545, EPI_ISL_487547, EPI_ISL_487549, EPI_ISL_487551 to 487555, EPI_ISL_487560 to 487561, EPI_ISL_487563, EPI_ISL_487565 to 487567, EPI_ISL_487570 to 487571, EPI_ISL_487573 to 487577, EPI_ISL_487579 to 487580, EPI_ISL_487583, EPI_ISL_487586 to 487588, EPI_ISL_487590 to 487592, EPI_ISL_487594, EPI_ISL_487596, EPI_ISL_487598 to 487599, EPI_ISL_487601 to 487602, EPI_ISL_487604 to 487605, EPI_ISL_487608, EPI_ISL_487610 to 487613, EPI_ISL_487617 to 487620, EPI_ISL_487622 to 487624, EPI_ISL_487629, EPI_ISL_487634, EPI_ISL_488187 to 488194, EPI_ISL_488196 to 488197, EPI_ISL_488199 to 488203, EPI_ISL_488205 to 488210, EPI_ISL_488213, EPI_ISL_488216 to 488228, EPI_ISL_488230 to 488232, EPI_ISL_488234, EPI_ISL_488236 to 488241, EPI_ISL_488243 to 488257, EPI_ISL_488259, EPI_ISL_488261 to 488269, EPI_ISL_488271 to 488277, EPI_ISL_488280, EPI_ISL_488283 to 488287, EPI_ISL_488290 to 488293, EPI_ISL_488295 to 488312, EPI_ISL_488314 to 488319, EPI_ISL_488321 to 488328, EPI_ISL_488330 to 488331, EPI_ISL_488337 to 488339, EPI_ISL_488341, EPI_ISL_488343, EPI_ISL_488345 to 488349, EPI_ISL_488351 to 488365, EPI_ISL_488367, EPI_ISL_488369, EPI_ISL_488371 to 488374, EPI_ISL_488376 to 488386, EPI_ISL_488388 to 488390, EPI_ISL_488392, EPI_ISL_488395, EPI_ISL_488398, EPI_ISL_488400 to 488407, EPI_ISL_488410, EPI_ISL_488412 to 488413, EPI_ISL_488415 to 488423, EPI_ISL_488425 to 488427, EPI_ISL_488429 to 488440, EPI_ISL_488442 to 488453, EPI_ISL_488455, EPI_ISL_488877, EPI_ISL_489120, EPI_ISL_492185 to 492187, EPI_ISL_492197, EPI_ISL_492203, EPI_ISL_492211, EPI_ISL_492213 to 492215, EPI_ISL_492217, EPI_ISL_492219 to 492220, EPI_ISL_492222 to 492223, EPI_ISL_492225 to 492226, EPI_ISL_492229 to 492230, EPI_ISL_492232 to 492233, EPI_ISL_492235, EPI_ISL_492238 to 492240, EPI_ISL_492242 to 492244, EPI_ISL_492246, EPI_ISL_492249 to 492250, EPI_ISL_492252 to 492254, EPI_ISL_492256, EPI_ISL_492258, EPI_ISL_492261 to 492266, EPI_ISL_492268, EPI_ISL_492270 to 492271, EPI_ISL_492274, EPI_ISL_492277 to 492279, EPI_ISL_492281, EPI_ISL_492283 to 492287, EPI_ISL_492289 to 492292, EPI_ISL_492294, EPI_ISL_492298 to 492302, EPI_ISL_492306 to 492309, EPI_ISL_492313 to 492315, EPI_ISL_492318 to 492322, EPI_ISL_492325, EPI_ISL_492329, EPI_ISL_492331 to 492332, EPI_ISL_492334 to 492339, EPI_ISL_492341, EPI_ISL_492344, EPI_ISL_492346 to 492350, EPI_ISL_492352 to 492353, EPI_ISL_492355, EPI_ISL_492359, EPI_ISL_492361, EPI_ISL_492363 to 492364, EPI_ISL_492366 to 492367, EPI_ISL_492370 to 492373, EPI_ISL_492377, EPI_ISL_492381, EPI_ISL_492383, EPI_ISL_492386, EPI_ISL_492388, EPI_ISL_492390, EPI_ISL_492392 to 492393, EPI_ISL_492395 to 492401, EPI_ISL_492403 to 492407, EPI_ISL_492410 to 492413, EPI_ISL_492415 to 492418, EPI_ISL_492420, EPI_ISL_492423 to 492427, EPI_ISL_492429 to 492430, EPI_ISL_492433 to 492434, EPI_ISL_492437, EPI_ISL_492439 to 492442, EPI_ISL_492444 to 492445, EPI_ISL_492447 to 492449, EPI_ISL_492505 to 492508, EPI_ISL_492510 to 492511, EPI_ISL_492513, EPI_ISL_492515 to 492523, EPI_ISL_492525, EPI_ISL_492527 to 492541, EPI_ISL_492543 to 492545, EPI_ISL_492547 to 492554, EPI_ISL_492556 to 492562, EPI_ISL_492564 to 492567, EPI_ISL_492569 to 492584, EPI_ISL_492586 to 492587, EPI_ISL_492589, EPI_ISL_492591 to 492592, EPI_ISL_492595, EPI_ISL_492597 to 492602, EPI_ISL_492604 to 492630, EPI_ISL_492632 to 492645, EPI_ISL_492647 to 492649, EPI_ISL_492651 to 492667, EPI_ISL_492669 to 492671, EPI_ISL_492673 to 492703, EPI_ISL_492705 to 492717, EPI_ISL_492719 to 492720, EPI_ISL_492722 to 492723, EPI_ISL_492725 to 492740, EPI_ISL_492742, EPI_ISL_492744 to 492745, EPI_ISL_492764 to 492772, EPI_ISL_492774 to 492778, EPI_ISL_492780, EPI_ISL_492782 to 492785, EPI_ISL_492787 to 492792, EPI_ISL_492794 to 492801, EPI_ISL_492803 to 492804, EPI_ISL_492806 to 492811, EPI_ISL_492814 to 492819, EPI_ISL_492821 to 492823, EPI_ISL_492825 to 492835, EPI_ISL_495097 to 495104, EPI_ISL_495107 to 495110, EPI_ISL_495112 to 495114, EPI_ISL_495116 to 495119, EPI_ISL_501556, EPI_ISL_501559 to 501561, EPI_ISL_501563 to 501568, EPI_ISL_501570, EPI_ISL_501572 to 501575, EPI_ISL_501578, EPI_ISL_501580 to 501581, EPI_ISL_501583, EPI_ISL_501586, EPI_ISL_501588 to 501593, EPI_ISL_501595 to 501599, EPI_ISL_501601, EPI_ISL_501606 to 501607, EPI_ISL_501609, EPI_ISL_501611, EPI_ISL_511955 to 511958, EPI_ISL_511960, EPI_ISL_511964, EPI_ISL_511966 to 511968, EPI_ISL_511971 to 511974, EPI_ISL_511976, EPI_ISL_511978 to 511981, EPI_ISL_513584, EPI_ISL_524486 to 524492, EPI_ISL_539894, EPI_ISL_539896			
see above	PHE South West Regional Laboratory, National Infection Service	Wellcome Sanger Institute for the COVID-19 Genomics UK (COG-UK) consortium	Alex Alderton; Barry Vipond; Cordelia Langford; David K. Jackson; Dominic Kwiatkowski; Dr Peter Muir; Ewan Harrison; Hannah Pymont; Ian Johnston; John Sillitoe on behalf of the Wellcome Sanger Institute COVID-19 Surveillance Team; John Sillitoe on behalf of the Wellcome Sanger Institute COVID-19 Surveillance Team (http://www.sanger.ac.uk/covid-team); Rich Hopes; Roberto Amato; Sonia Goncalves; Stephanie Hutchings; and Alex Alderton

EPI_ISL_678257	PHI Institute of Nephrology - Struga	Research Center for Genetic Engineering and Biotechnology "Georgi D. Efremov" , Macedonian Academy of Sciences and Arts	RCGEB - MASA
EPI_ISL_968212	PHV-FSS	PHV-FSS	Son Nguyen et al
EPI_ISL_733570	POK OI HOSPITAL	Hong Kong Department of Health	Alan K.L. Tsang; Dominic N.C. Tsang; Edman T.K. Lam; Peter C.W. Yip; Rickjason C.W. Chan
EPI_ISL_445356	PONTIFICIA U. CATOLICA SERV. LABORATORIO	Instituto de Salud Publica de Chile	Alejandra Acevedo; Andrés E Castillo; Bárbara Parra; Carolina Tambley; Gabriel Leal; Jaime Lagos; Jorge Fernandez; Loredana Arata; Patricia Bustos; Paz Tapia; Rodrigo Fasce; Winston Andrade
EPI_ISL_447845	PR - Biological and Chemical Emergencies Lab Office of Public Health Preparedness and Response	Pathogen Discovery, Respiratory Viruses Branch, Division of Viral Diseases, Centers for Disease Control and Prevention	Alison S. Laufer Halpin; Anna Uehara; Christopher A. Elkins; Clinton R. Paden; Haibin Wang; Jasmine Padilla; Jing Zhang; Justin Lee; Krista Queen; Mary S. Keckler; Suxiang Tong; Yan Li; Ying Tao
EPI_ISL_1036205	PRES TERR CASOLI	Istituto Zooprofilattico Sperimentale dell'Abruzzo e Molise "G. Caporale"	Ancora M; Calistri P; Cammà C; Curini V; Di Domenico M; Di Pasquale A; Lorusso A; Mangone I; Marcacci M; Puglia I; Rinaldi A; Savini G; Scialabba S
EPI_ISL_534322	PS Mun Julio Tupy	Instituto Adolfo Lutz, Interdiciplinary Procedures Center, Strategic Laboratory	Claudia Regina Gonçalves; Claudio Tavares Sacchi; Erica Valessa Ramos Gomes
EPI_ISL_523973	PS Municipal Dona Maria Antonieta Ferreira de Barros	Instituto Adolfo Lutz, Interdiciplinary Procedures Center, Strategic Laboratory	Claudia Regina Gonçalves; Claudio Tavares Sacchi; Erica Valessa Ramos Gomes
EPI_ISL_547570	PS Municipal Dr Augusto Gomes de Mattos	Instituto Adolfo Lutz, Interdiciplinary Procedures Center, Strategic Laboratory	Claudia Regina Gonçalves; Claudio Tavares Sacchi; Erica Valessa Ramos Gomes; Karoline Rodrigues Campos
EPI_ISL_515523 to 515524, EPI_ISL_515566, EPI_ISL_524466, EPI_ISL_527866	PS Municipal Dr Lauro Ribas Braga	Instituto Adolfo Lutz, Interdiciplinary Procedures Center, Strategic Laboratory	01246-1301; 355 - Brazil; Av. Dr. Arnaldo; Cerqueira Cesar; Claudia Regina Gonçalves; Claudio Tavares Sacchi; Erica Valessa Ramos Gomes; São Paulo - SP
EPI_ISL_524465	PS Municipal Dr. Caetano Virgílio Neto	Instituto Adolfo Lutz, Interdiciplinary Procedures Center, Strategic Laboratory	Claudia Regina Gonçalves; Claudio Tavares Sacchi; Erica Valessa Ramos Gomes
EPI_ISL_534321	PS e Maternidade Nair Fonseca Leitao Arantes	Instituto Adolfo Lutz, Interdiciplinary Procedures Center, Strategic Laboratory	Claudia Regina Gonçalves; Claudio Tavares Sacchi; Erica Valessa Ramos Gomes
EPI_ISL_700435, EPI_ISL_700439, EPI_ISL_700478, EPI_ISL_700551, EPI_ISL_700586	Pacaltsdorp Clinic wc PAC	NHLS/UCT	Arash Iranzadeh; Bruna Galvao; Carolyn Williamson; Deelan Doolabh; Diana Hardie; Houriiyah Tegally; Innocent Mudau; Kruger Marais; Lynn Tyers; Marvin Hsiao; Stephen Korsman
EPI_ISL_696459, EPI_ISL_696470, EPI_ISL_696474	Pacaltsdorp Clinic wc PAC & NHLS/UCT	KRISP, KZN Research Innovation and Sequencing Platform	Arash Iranzadeh; Bruna Galvao; Carolyn Williamson; Deelan Doolabh; Diana Hardie; Emanuel James San; Houriiyah Tegally; Innocent Mudau; Jennifer Giandhari; Kruger Marais; Lynn Tyers; Marvin Hsiao; Stephen Korsman; Sureshnee Pillay; Tulio de Oliveira
EPI_ISL_596500 to 596504, EPI_ISL_596506 to 596518, EPI_ISL_596520 to 596521, EPI_ISL_596523, EPI_ISL_596525 to 596545, EPI_ISL_596547 to 596567	Palestinian Ministry of Health	Molecular Genetics Lab	Damien Richard; Dana Najjar; Francois Balloux; Hisham Darwish; Husam Sallam; Issa Shtayeh; Lucy van Dorp; Mahmoud Ruzayqat; Nouar Qutob; Osama Najjar; Zaidoun Salah
EPI_ISL_955905	Pamela Youde Nethersole Eastern Hospital	Hong Kong Department of Health	Alan K.L. Tsang; Dominic N.C. Tsang; Edman T.K. Lam; Peter C.W. Yip; Rickjason C.W. Chan
EPI_ISL_707936 to 707939, EPI_ISL_708184 to 708186, EPI_ISL_708188 to 708190, EPI_ISL_708192 to 708195	Pamukkale University Hospital	Pamukkale University Department of Medical Genetics	Onur TOKGUN et al.
EPI_ISL_984880, EPI_ISL_984921, EPI_ISL_994831, EPI_ISL_994836, EPI_ISL_994875, EPI_ISL_994882, EPI_ISL_994889, EPI_ISL_994897, EPI_ISL_994902, EPI_ISL_994908, EPI_ISL_994910, EPI_ISL_994913 to 994914, EPI_ISL_994923, EPI_ISL_994926 to 994927, EPI_ISL_994948, EPI_ISL_994950, EPI_ISL_994962, EPI_ISL_994964 to 994965, EPI_ISL_994982, EPI_ISL_995009, EPI_ISL_995028, EPI_ISL_995038, EPI_ISL_995044, EPI_ISL_995047, EPI_ISL_995051, EPI_ISL_995070, EPI_ISL_995096, EPI_ISL_995098, EPI_ISL_995100 to 995101, EPI_ISL_995106, EPI_ISL_995117, EPI_ISL_995130, EPI_ISL_995137, EPI_ISL_995149, EPI_ISL_995153, EPI_ISL_995169, EPI_ISL_1040480			
see above	Pandemic Response Lab - NYC	Pandemic Response Lab, R&D	Cybill del Castillo; Haiping Hao; Henry Lee; Jon Laurent; Melissa Hopkins; Michael Hammerling; Pradeep Bugga; William Ward
EPI_ISL_450785 to 450791, EPI_ISL_455015 to 455016, EPI_ISL_461481 to 461482, EPI_ISL_483829 to 483849	Pandit Deendayal Upadhyay Government Medical College, Rajkot	Gujarat Biotechnology Research Centre	A M Kadri; Afzal Ansari; Amit Kanani; Anjali Rajwar; Ankit Hinsu; Apurvasinh Puvar; Armi Chaudhari; Arti Trivedi; Bhavesh Modi; Bhavya Jindal; Camellia Chakraborty; Chaitanya Joshi; Dinesh Kumar; Gauravi Dhruv; Gaurishankar Shrimali; Harsh Bakshi; Janvi Raval; Komal Patel; Labdhi Pandya; Madhvi Joshi; Maharshi Pandya; Manish Pattani; Monika Gandhi; Neelam Nathani; Neha Rajpara; Nidhi Patel; Nikha Trivedi; Nitin Savaliya; Pinal Trivedi; Pooja P Doshi; Prakash Modi; Pritesh Sabara; R D Dixit; Raghawendra Kumar; Ramesh Pandit; Sejul Antala; Sharmistha Majumdar; Siddhant Kumar; Snehal Bagatharia; Tejas Shah; Umang Mishra; Zarna Patel; Zuber Saiyed
EPI_ISL_455605	Panyananthaphikkhu Chonprathan Medical Center	National Institute of Health. Department of medical Sciences, Ministry of Public Health, Thailand	Chittaganpitch; Malinee; Okada; Parnmen; Phuygun; Pilailuk; Siripaporn; Sittiporn; Sunthareeya; Thanadachakul; Thanutsapa; Waicharoen; Warawan; Wongboot
EPI_ISL_434701	Panyanunthaphikkhu Chonprathan Medical Center (PCMC)	National Institute of Health. Department of medical Sciences, Ministry of Public Health, Thailand	Chittaganpitch; Malinee; Okada; Parnmen; Phuygun; Pilailuk; Siripaporn; Sittiporn; Sunthareeya; Thanadachakul; Thanutsapa; Waicharoen; Warawan; Wongboot
EPI_ISL_421502 to 421503	Parc des Dames	National Reference Center for Viruses of Respiratory Infections, Institut Pasteur, Paris	Angela Brisebarre; Etienne Simon-Lorière; Flora Donati; Marion Barbet; Maud Vanpeene; Mélanie Albert; Méline Bizard; Sylvie Behillil; Sylvie van der Werf; Vincent Enouf
EPI_ISL_448222	Pasteur Insitute Ho Chi Minh City	National Key Laboratory of Gene Technology, Institute of Biotechnology, Vietnam Academy of Science and Technology	Cao Minh Thang; Chu Hoang Ha; Ho Thi Thuong; Hoang Ha; Huynh Thi Kim Loan; Le Tung Lam; Luong Chan Quang; Luu Thuy Tien; Nguyen Hong Trang; Nguyen Thi Ngoc Thao; Nguyen Trung Hieu; Nguyen Vu Thuong; Pham Duy Quang; Phan Trong Lan; Tran Huyen Linh; Truong Nam Hai
EPI_ISL_654866 to 654870, EPI_ISL_654872 to 654879, EPI_ISL_654881 to 654891, EPI_ISL_760247	Pasteur Institute in Ho Chi Minh city	Department of Microbiology and Immunology - Pasteur Institute in Ho Chi Minh city	Anh Hoang Nguyen; Cao Minh Thng; Hang Thi Thu Pham; Hieu Minh Le; Hieu Trung Nguyen; Hoàng Kim Loan; Hoàng Minh; Hoàng Nh ào; Hoàng Quc Cng; Hoàng Thùy Linh; Hunh Kim Mai; Hunh Phng Tho; Hunh Th Kim Loan; Hà Bích Ngc; Khu Vn Ngha; La Hoàng Huy; Lan Trong Phan; Loan Thi Kim Huynh; Long Thanh Nguyen; Lê Hoàng Chng; Lê Ngc Tú; Lê Th Kim Trang; Lê Xuân Huy; Lng Chn Quang; Manh Huy Dao; Nguyn Bo Triu; Nguyn Hoàng Anh; Nguyn Hoàng Quân; Nguyn Hoàng V; Nguyn Ngc Anh Tun; Nguyn Thanh Long; Nguyn Thu Ngc; Nguyn Th Ngc Tho; Nguyn Th Phng Thúy; Nguyn Th Thanh Thng; Nguyn Th Thanh Tho; Nguyn Trung Hiu; Nguyn Trng Toàn; Nguyn Vit Thinh; Nguyn V Thng; Nhung Pham Hong Vu; Phan Trng Lân; Phm Duy Quang; Phm Th Nhung; Phm Th Thu Hng; Quang Chan Luong; Quang Duy Pham; Thang Minh Cao; Thao Thi Ngoc Nguyen; Thuong Vu Nguyen; Trng Công Hiu; Trng Thùy Dung; Trn Th Hng Kim; Trn Tôn; Trnh Hoàng Long; Viên ng Khánh Linh; Võ Th Trang ài; V Phm Hng Nhung; ào Huy Mnh; ng Thanh Giang; Thái Hùng
EPI_ISL_442523	Pasteur Institute of Iran	Kawsar Human Genetic Research Company	Elmira Khateri; Maraym Firoozi; Maryam Abbasalipour Bashash; Mohammad Ali Khosravi; Razieh Zeinali and Fahimeh Hoseini; Sanaz Mostafavi Jabbari; Sirous Zeinali; Sormeh Pourtavakoli
EPI_ISL_660143 to 660146, EPI_ISL_660148, EPI_ISL_660150, EPI_ISL_660152, EPI_ISL_660154, EPI_ISL_660157 to 660158	PathCare	National Health Laboratory Service (NHLS), Tygerberg	Bronwyn Kleinhans; Davis M-A; Draper C; Eduan Wilkindon; Gert van Zyl; Houriiyah Tegally; Hsiao M; Kayla Delaney; Siegfried N; Susan Engelbrecht; Tulio de Oliveira; Williamson C; Wolfgang Preiser
EPI_ISL_456163, EPI_ISL_456171, EPI_ISL_456194 to 456195, EPI_ISL_456215, EPI_ISL_456221 to 456224, EPI_ISL_582018 to 582020, EPI_ISL_622804 to 622806, EPI_ISL_637088, EPI_ISL_755626			
see above	PathLab Bay of Plenty	Institute of Environmental Science and Research (ESR)	Anja Werno; Antje van der Linden; Arlo Upton; Chris Mansell; David Hammer; Dragana Drinkovic; Erasmus Smit; Gary McAuliffe; Hana Sofia Andersson; Hermes Perez; James Ussher; Jill Sherwood; Jing Wang; Joep de Ligt; Josh Freeman; Julia Howard; Juliet Elvy; Lauren Jelly; Mary DeAlmeida; Matt Blakiston; Matt Storey; Matthew Rogers; Max Bloomfield; Michael Addidle; Michelle Balm; Muhammad Faisal; Nikki Freed; Olin Silander; Sally Roberts; Sarah Jefferies; Sharmini Muttaiyah; Susan Morpeth; Susan Taylor; Timothy Blackmore; Vani Sathyendran; Veronica Playle; Virginia Hope; Xiaoyun Ren
EPI_ISL_420456, EPI_ISL_470830 to 470831, EPI_ISL_470833, EPI_ISL_470835, EPI_ISL_470839 to 470841, EPI_ISL_470843, EPI_ISL_470845 to 470849, EPI_ISL_470851, EPI_ISL_470853 to 470856, EPI_ISL_470858, EPI_ISL_470861 to 470865, EPI_ISL_470867 to 470869, EPI_ISL_470872 to 470873			
see above	PathWest Laboratory Medicine WA	PathWest Laboratory Medicine WA	Avram Levy; Chisha Sikazwe; David Smith and David Speers; Jurissa Lang

EPI_ISL_512712 to 512717, EPI_ISL_512719 to 512720, EPI_ISL_512722 to 512725, EPI_ISL_512727 to 512728, EPI_ISL_512730, EPI_ISL_512732, EPI_ISL_512734 to 512735, EPI_ISL_512737 to 512739, EPI_ISL_512741 to 512744, EPI_ISL_512747 to 512750, EPI_ISL_512752, EPI_ISL_512754 to 512765, EPI_ISL_594167, EPI_ISL_594170 to 594171, EPI_ISL_594174 to 594175, EPI_ISL_594179, EPI_ISL_596677 to 596713, EPI_ISL_596819 to 596862, EPI_ISL_605825 to 605836, EPI_ISL_672619, EPI_ISL_672621, EPI_ISL_672623, EPI_ISL_672625 to 672626, EPI_ISL_672630 to 672631, EPI_ISL_672633 to 672635, EPI_ISL_672637, EPI_ISL_672642, EPI_ISL_672644, EPI_ISL_672647 to 672648, EPI_ISL_672650, EPI_ISL_672652 to 672653, EPI_ISL_708738 to 708739, EPI_ISL_708789 to 708790, EPI_ISL_708793, EPI_ISL_794675, EPI_ISL_810967, EPI_ISL_857486 to 857487, EPI_ISL_933788, EPI_ISL_933799			
see above	PathWest Laboratory Medicine WA	PathWest Laboratory Medicine WA Microbial Surveillance Unit	PathWest Laboratory Medicine WA Microbial Surveillance Unit
EPI_ISL_582022 to 582023	Pathlab Lakes	Institute of Environmental Science and Research (ESR)	Anja Werno; Antje van der Linden; Arlo Upton; Chris Mansell; David Hammer; Dragana Drinkovic; Erasmus Smit; Gary McAuliffe; Hana Sofia Andersson; Hermes Perez; James Ussher; Jill Sherwood; Jing Wang; Joep de Ligt; Josh Freeman; Julia Howard; Juliet Elvy; Lauren Jelly; Mary DeAlmeida; Matt Blakiston; Matt Storey; Matthew Rogers; Max Bloomfield; Michael Addidle; Michelle Balm; Muhammad Faisal; Nikki Freed; Olin Silander; Sally Roberts; Sarah Jefferies; Sharmini Muttaiyah; Susan Morpeth; Susan Taylor; Timothy Blackmore; Vani Sathyendran; Veronica Playle; Virginia Hope; Xiaoyun Ren
EPI_ISL_480208, EPI_ISL_591347 to 591432, EPI_ISL_591434 to 591483, EPI_ISL_591535 to 591539, EPI_ISL_667559 to 667564, EPI_ISL_667566 to 667592, EPI_ISL_667594 to 667617, EPI_ISL_667619 to 667620, EPI_ISL_667622 to 667633, EPI_ISL_667635 to 667728, EPI_ISL_667730 to 667733, EPI_ISL_667735 to 667757, EPI_ISL_684110 to 684111, EPI_ISL_684113 to 684116, EPI_ISL_684118 to 684126, EPI_ISL_684128 to 684130, EPI_ISL_684132 to 684133, EPI_ISL_684139, EPI_ISL_684142 to 684143, EPI_ISL_684145 to 684146, EPI_ISL_684150 to 684153, EPI_ISL_684156 to 684158, EPI_ISL_684162 to 684164, EPI_ISL_684166 to 684168, EPI_ISL_684170 to 684219, EPI_ISL_684289 to 684324, EPI_ISL_684339 to 684363, EPI_ISL_684365 to 684379, EPI_ISL_684381 to 684407, EPI_ISL_684484 to 684505, EPI_ISL_684523 to 684527, EPI_ISL_684529 to 684543, EPI_ISL_684545 to 684555, EPI_ISL_684558 to 684565, EPI_ISL_684578 to 684614, EPI_ISL_684693 to 684717, EPI_ISL_684736 to 684797, EPI_ISL_684857 to 684880, EPI_ISL_685133 to 685219, EPI_ISL_685238, EPI_ISL_685240 to 685278, EPI_ISL_685282 to 685296, EPI_ISL_685311 to 685397, EPI_ISL_685400 to 685406, EPI_ISL_685505 to 685583, EPI_ISL_685585 to 685605, EPI_ISL_685617 to 685665, EPI_ISL_685695 to 685917, EPI_ISL_685925 to 685962, EPI_ISL_685988 to 686082, EPI_ISL_686109 to 686189, EPI_ISL_686191 to 686265, EPI_ISL_686267 to 686427, EPI_ISL_686429 to 686520, EPI_ISL_686612 to 686658, EPI_ISL_686845 to 687042, EPI_ISL_687079 to 687166, EPI_ISL_687172 to 687174, EPI_ISL_687177, EPI_ISL_687179, EPI_ISL_687181 to 687182, EPI_ISL_687184 to 687185, EPI_ISL_687188 to 687532, EPI_ISL_687561 to 687570, EPI_ISL_687572 to 687611, EPI_ISL_687614, EPI_ISL_687616 to 687618, EPI_ISL_687624 to 687698, EPI_ISL_687718 to 688283, EPI_ISL_688402 to 688582, EPI_ISL_688605 to 688611, EPI_ISL_688613 to 688614, EPI_ISL_688616 to 688618, EPI_ISL_688624 to 688625, EPI_ISL_688635 to 688699, EPI_ISL_688710 to 688896, EPI_ISL_688969 to 689046, EPI_ISL_689089 to 689459, EPI_ISL_689497 to 689611, EPI_ISL_689624 to 689639, EPI_ISL_689641 to 689645, EPI_ISL_689649 to 689685, EPI_ISL_689698 to 689758, EPI_ISL_689826 to 690010, EPI_ISL_690029 to 690209, EPI_ISL_690254 to 690431, EPI_ISL_690527 to 690611, EPI_ISL_690614 to 690617, EPI_ISL_690620 to 690625, EPI_ISL_690632 to 690633, EPI_ISL_690635, EPI_ISL_690684 to 690791, EPI_ISL_690845 to 690901, EPI_ISL_690909 to 690981, EPI_ISL_690987 to 691283, EPI_ISL_691289 to 691363, EPI_ISL_691379 to 691508, EPI_ISL_691546 to 691549, EPI_ISL_691552 to 691611, EPI_ISL_691744, EPI_ISL_691748, EPI_ISL_691752, EPI_ISL_691759 to 691763, EPI_ISL_691767 to 692046, EPI_ISL_692059 to 692093, EPI_ISL_692098 to 692118, EPI_ISL_692129 to 692161, EPI_ISL_692164 to 692218, EPI_ISL_692230 to 692283, EPI_ISL_692328 to 692441, EPI_ISL_692498 to 692652, EPI_ISL_692680 to 692732, EPI_ISL_721568 to 721569, EPI_ISL_721575, EPI_ISL_721577 to 721623, EPI_ISL_736892 to 736924, EPI_ISL_768634 to 768641, EPI_ISL_768643 to 768675, EPI_ISL_768677 to 768689, EPI_ISL_768691 to 768716, EPI_ISL_768718 to 768724, EPI_ISL_779211, EPI_ISL_779237 to 779238, EPI_ISL_779663, EPI_ISL_779686, EPI_ISL_825387 to 825388, EPI_ISL_825391 to 825392, EPI_ISL_833370, EPI_ISL_860109, EPI_ISL_884864, EPI_ISL_892701 to 892703, EPI_ISL_893239 to 893240, EPI_ISL_918133			
see above	Pathogen Genomics Center, National Institute of Infectious Diseases	Pathogen Genomics Center, National Institute of Infectious Diseases	Hajime Kamiya; Kentaro Itokawa; Makoto Kuroda; Masanori Hashino; Motoi Suzuki; Rina Tanaka; Ryohei Nomoto; Tomoe Shimada; Tsuyoshi Sekizuka
EPI_ISL_636972, EPI_ISL_678247	Pathogen Genomics Lab King Abdullah University of Science and Technology (KAUST)	Pathogen Genomics Lab King Abdullah University of Science and Technology (KAUST)	Abbas Al Mutair; Afrah Alsomali; Amanda Ooi; Amit Kumar Subudhi; Anwar Hashem; Arnab Pain; Asim Khogeer; Awad Al-Omari; Fadwa Alofi; Fathia Ben Rached; Kahled Algithami; Luke Esau; Muhammad Shuaib; Naif Almontashiri; Olga Douvropoulou; Raeece Naeem; Rahul P Salunke; Raushan Nugmanova; Samer Salih; Sara Mfarrej; Sharif Hala
EPI_ISL_437459 to 437466, EPI_ISL_437468 to 437475, EPI_ISL_437477, EPI_ISL_437479, EPI_ISL_437481 to 437497, EPI_ISL_437691 to 437762, EPI_ISL_512874 to 512896, EPI_ISL_512898 to 512909, EPI_ISL_512911 to 512924, EPI_ISL_512926 to 512939, EPI_ISL_512941 to 512957, EPI_ISL_512959, EPI_ISL_512963 to 512964, EPI_ISL_512966 to 512967, EPI_ISL_512969 to 512971, EPI_ISL_512973 to 512977, EPI_ISL_512979 to 512984, EPI_ISL_512986 to 513006, EPI_ISL_513008 to 513010, EPI_ISL_513012 to 513029, EPI_ISL_513032 to 513043, EPI_ISL_513045 to 513067, EPI_ISL_513069 to 513083, EPI_ISL_513085 to 513087, EPI_ISL_513089 to 513109, EPI_ISL_513111 to 513129, EPI_ISL_513131 to 513139, EPI_ISL_513141 to 513154, EPI_ISL_513156 to 513164, EPI_ISL_513166 to 513212, EPI_ISL_513214 to 513219, EPI_ISL_513221 to 513222, EPI_ISL_513224, EPI_ISL_513226, EPI_ISL_513228 to 513231, EPI_ISL_513233 to 513236, EPI_ISL_513238, EPI_ISL_513240 to 513254, EPI_ISL_513257 to 513264, EPI_ISL_636964 to 636967, EPI_ISL_677922 to 677944, EPI_ISL_677968 to 677969, EPI_ISL_677972 to 677975, EPI_ISL_677977 to 678038, EPI_ISL_678070, EPI_ISL_678073 to 678146, EPI_ISL_678152 to 678159, EPI_ISL_678189 to 678226, EPI_ISL_678230 to 678231, EPI_ISL_678233, EPI_ISL_678237, EPI_ISL_678239, EPI_ISL_678241, EPI_ISL_678246, EPI_ISL_751223 to 751238			
see above	Pathogen Genomics Lab King Abdullah University of Science and Technology(KAUST)	Pathogen Genomics Lab King Abdullah University of Science and Technology(KAUST)	Abbas Al Mutair; Abdulaziz Alahmadi; Afrah Alsomali; Ahmad Bakur Mahmoud; Amanda; Amanda Ooi; Amit Kumar Subudhi; Amit Subudhi; Anwar Hashem; Arnab Pain; Asim Khogeer; Awad A-Omari; Awad Al-Omari; Fadwa Alofi; Fathia Ben Rached; Fathia Ben-Rached; Fathia Rached; Jumana Taha; Kahled Algithami; Khaled Algithami; Luke; Luke Esau; Muhammad Shuaib; Naif Almontashiri; Nashwa Al-khotani; Olga Douvropoulou; Raeece Naeem; Rahul P Salunke; Rahul Salunke; Raushan Nugmanova; Samer Salih; Sara Mfarrej; Sharif Hala
EPI_ISL_470897 to 470899	Pathogenic Microorganisms Variability Laboratory	Pathogenic Microorganisms Variability Laboratory	Alexander Gintsburg; Alexey Shchetinin; Andrey Botikov; Elena Shidlovskaya; Maria Nikiforova; Nadezhda Kuznetsova; Vladimir Gushchin
EPI_ISL_566077 to 566083, EPI_ISL_596318 to 596352, EPI_ISL_733156, EPI_ISL_733158, EPI_ISL_733160, EPI_ISL_733163, EPI_ISL_733167 to 733168, EPI_ISL_733170, EPI_ISL_733174 to 733176, EPI_ISL_733178 to 733182, EPI_ISL_733189 to 733192, EPI_ISL_733194, EPI_ISL_733196 to 733200, EPI_ISL_733203 to 733208, EPI_ISL_733210 to 733211, EPI_ISL_733215, EPI_ISL_733220, EPI_ISL_733222			
see above	Pathogenic Microorganisms Variability Laboratory	WHO National Influenza Centre Russian Federation	Alexander Gintsburg; Alexey Shchetinin; Andrey Komissarov; Andrey Pochtovyy; Anna Ignatieva; Anna Ivanova; Artem Fadeev; Artem Tkachuk; Daria Danilenko; Dmitry Bazhenov; Dmitry Lioznov; Ekaterina Milashenko; Elena Burtseva; Elena Nabieva; Elena Shidlovskaya; Elena Vokalova; Elizaveta Divisenko; Evgeniya Mukasheva; Evgeny Usachev; Evgeny Usachev; Georgii Bazykin; Kirill Krasnoslobotsev; Ksenia Safina; Kseniya Komissarova; Ludmila Kolobukhina; Maria Nikiforova; Maxim Rubalsky; Nadezhda Kuznetsova; Oleg Rubalsky; Olga Burgasova; Sergey Alkhovsky; Svetlana Smetanina; Svetlana Trushakova; Tatyana Vishnevskaya; Valeria Bacalin; Vladimir Gushchin
EPI_ISL_583619 to 583627, EPI_ISL_583629	Pathologie-Labor Dr. Obrist- Dr. Brunhuber	Bergthaler laboratory, CeMM Research Center for Molecular Medicine of the Austrian Academy of Sciences	Adi Steinrigl; Alexander Lercher; Alexandra Popa; Andreas Bergthaler; Benedikt Agerer; Christian Paar; Christoph Bock; Daniela Schmid; Dorothee von Laer; Elisabeth Puchhammer-Stoeckl; Franz Allerberger; Gernot Walder; Gregor Hörmann; Guenter Weiss; Gunther Vogl; Henrique Colaco; Jakob-Wendelin Genger; Jan Laine; Judith Aberle; Kinga Rigler-Hohenwarter; Lukas Endler; Manfred Nairz; Mark Smyth; Martin Senekowitsch; Michael Schuster; Peter Hufnagl; Peter Obrist; Rainer Gattringer; Sabine Sussitz-Rack; Stephan Aberle; Thomas Penz; Wegene Borena
EPI_ISL_421636	Pathology North	Public Health Virology Laboratory	Alyssa Pyke; Amanda De Jong; Andrew Van Den Hurk; Bixing Huang; Carmel Taylor; David Warrilow; Doris Genge; Elisabeth Gamez; Glen Hewitson; Ian Maxwell Mackay; Inga Sultana; Jamie McMahon; Jean Barcelon; Judy Northill; Mitchell Finger; Natalie Simpson; Neelima Nair; Peter Burtonclay; Peter Moore; Sarah Wheatley; Sean Moody; Sonja Hall-Mendelin; Timothy Gardam; and Frederick Moore
EPI_ISL_745096	Pathology North - Hunter - NSW Health Pathology	CIDM-PH, Westmead Hospital	CIDM-PH et al.
EPI_ISL_513335, EPI_ISL_513360, EPI_ISL_513368, EPI_ISL_526152, EPI_ISL_593655 to 593658, EPI_ISL_740891, EPI_ISL_767920			
see above	Pathology North - Hunter - NSW Health Pathology	NSW Health Pathology - Institute of Clinical Pathology and Medical Research; Westmead Hospital; University of Sydney	CIDM-PH et al.
EPI_ISL_451487 to 451488, EPI_ISL_451552, EPI_ISL_451604, EPI_ISL_513386 to 513388	Pathology North - NSW Health Pathology	NSW Health Pathology - Institute of Clinical Pathology and Medical Research; Westmead Hospital; University of Sydney	CIDM-PH et al.
EPI_ISL_710129	Pathology North - Royal North Shore Hospital - NSW Health Pathology	CIDM-PH et al.	CIDM-PH et al.
EPI_ISL_745097 to 745107	Pathology North - Royal North Shore Hospital - NSW Health Pathology	CIDM-PH, Westmead Hospital	CIDM-PH et al.
EPI_ISL_478673 to 478674, EPI_ISL_513375 to 513376, EPI_ISL_526128, EPI_ISL_526130, EPI_ISL_526132 to 526133, EPI_ISL_526154 to 526155, EPI_ISL_526170, EPI_ISL_544969, EPI_ISL_544976, EPI_ISL_544982, EPI_ISL_544991, EPI_ISL_545008, EPI_ISL_591493, EPI_ISL_593659, EPI_ISL_708799, EPI_ISL_740866, EPI_ISL_740875 to 740878, EPI_ISL_740883 to 740889, EPI_ISL_767868, EPI_ISL_767870 to 767871			
see above	Pathology North - Royal North Shore Hospital - NSW Health Pathology	NSW Health Pathology - Institute of Clinical Pathology and Medical Research; Westmead Hospital; University of Sydney	CIDM-PH et al.
EPI_ISL_451587, EPI_ISL_451589, EPI_ISL_451600, EPI_ISL_490043	Pathology North Hunter- NSW Health Pathology	NSW Health Pathology - Institute of Clinical Pathology and Medical Research; Westmead Hospital; University of Sydney	CIDM-PH et al.
EPI_ISL_407894, EPI_ISL_407896, EPI_ISL_410717 to 410718, EPI_ISL_414414, EPI_ISL_417031, EPI_ISL_418802 to 418804, EPI_ISL_418807 to 418808, EPI_ISL_444611, EPI_ISL_444793			
see above	Pathology Queensland	Public Health Virology Laboratory	Alyssa Pyke; Amanda De Jong; Andrew Van Den Hurk; Ben Huang; Bixing Huang; Carmel Taylor; David Warrilow; Doris Genge; Elisabeth Gamez; Glen Hewitson; Ian Maxwell Mackay; Inga Sultana; Jamie McMahon; Jean Barcelon; Judy Northill; Mitchell Finger; Natalie Simpson; Neelima Nair; Peter Burtonclay; Peter Moore; Sarah Wheatley; Sean Moody; Sonja Hall-Mendelin; Timothy Gardam; and Frederick Moore; and Frederick Moore.
EPI_ISL_447595	Pathology Queensland, Sunshine Coast University Hospital	Public Health Virology Laboratory	Alyssa Pyke; Amanda De Jong; Andrew Van Den Hurk; Bixing Huang; Carmel Taylor; David Warrilow; Doris Genge; Elisabeth Gamez; Glen Hewitson; Ian Maxwell Mackay; Inga Sultana; Jamie McMahon; Jean Barcelon; Judy Northill; Mitchell Finger; Natalie Simpson; Neelima Nair; Peter Burtonclay; Peter Moore; Sarah Wheatley; Sean Moody; Sonja Hall-Mendelin; Timothy Gardam; and Frederick Moore
EPI_ISL_451530 to 451531, EPI_ISL_451533, EPI_ISL_451536, EPI_ISL_451542 to 451544, EPI_ISL_451549, EPI_ISL_451559 to 451565, EPI_ISL_451578, EPI_ISL_451581, EPI_ISL_451603, EPI_ISL_451608, EPI_ISL_451642 to 451643, EPI_ISL_455037, EPI_ISL_455071, EPI_ISL_455094			
see above	Pathology Sydney South West - NSW Health Pathology	NSW Health Pathology - Institute of Clinical Pathology and	CIDM-PH et al.

		Medical Research; Westmead Hospital; University of Sydney	
EPI_ISL_451490 to 451516, EPI_ISL_451522 to 451529, EPI_ISL_451532, EPI_ISL_451535, EPI_ISL_451537 to 451539, EPI_ISL_451541, EPI_ISL_451546 to 451547, EPI_ISL_451550 to 451551, EPI_ISL_451556 to 451557, EPI_ISL_451566, EPI_ISL_451568 to 451571, EPI_ISL_451573, EPI_ISL_451575 to 451577, EPI_ISL_451579 to 451580, EPI_ISL_451582, EPI_ISL_451584, EPI_ISL_451586, EPI_ISL_451606 to 451607, EPI_ISL_451613 to 451630, EPI_ISL_451637 to 451640, EPI_ISL_455028 to 455035, EPI_ISL_455038 to 455040, EPI_ISL_455044 to 455047, EPI_ISL_455049, EPI_ISL_455055 to 455062, EPI_ISL_455064, EPI_ISL_455066 to 455067, EPI_ISL_455069 to 455070, EPI_ISL_455095, EPI_ISL_490036, EPI_ISL_490038 to 490039, EPI_ISL_490044, EPI_ISL_490046, EPI_ISL_498748 to 498750, EPI_ISL_498761 to 498762, EPI_ISL_498766 to 498767, EPI_ISL_498776 to 498777, EPI_ISL_498779 to 498780, EPI_ISL_513321 to 513322, EPI_ISL_513324 to 513326, EPI_ISL_513332, EPI_ISL_513338 to 513339, EPI_ISL_513342, EPI_ISL_513345 to 513346, EPI_ISL_513350 to 513351, EPI_ISL_513358 to 513359, EPI_ISL_513369 to 513371, EPI_ISL_513378, EPI_ISL_513380, EPI_ISL_513390 to 513395, EPI_ISL_526115 to 526118, EPI_ISL_526138 to 526141, EPI_ISL_526150 to 526151, EPI_ISL_526156, EPI_ISL_526161 to 526165, EPI_ISL_526199, EPI_ISL_526689, EPI_ISL_544955, EPI_ISL_544986, EPI_ISL_544989 to 544990, EPI_ISL_545006, EPI_ISL_545029, EPI_ISL_545032 to 545033, EPI_ISL_545038, EPI_ISL_545046 to 545047, EPI_ISL_545050, EPI_ISL_591494 to 591501, EPI_ISL_593660 to 593662, EPI_ISL_593664 to 593665, EPI_ISL_593667 to 593682, EPI_ISL_593770, EPI_ISL_667777, EPI_ISL_740890, EPI_ISL_767898, EPI_ISL_767933 to 767937			
see above	Pathology West - NSW Health Pathology	NSW Health Pathology - Institute of Clinical Pathology and Medical Research; Westmead Hospital; University of Sydney	CIDM-PH et al.
EPI_ISL_681320 to 681321	Pathology and Laboratory Medicine, UW-Madison	Pathology and Laboratory Medicine, UW-Madison	Baczenas; Baker, D.; Braun, K.; J.J.; Moreno, G.
EPI_ISL_960111	Pelican Park CDC wc PAX	National Health Laboratory Service/UCT	Arash Iranzadeh; Bruna Galvao; Carolyn Williamson; Deelan Doolabh; Diana Hardie; Innocent Mudau; Kruger Marais; Lynn Tyers; Marvin Hsiao; Stephen Korsman
EPI_ISL_455585, EPI_ISL_455608	Phramongkutklao Hospital	National Institute of Health. Department of medical Sciences, Ministry of Public Health, Thailand	Chittaganpitch; Malinee; Okada; Parnmen; Phuygun; Pilailuk; Siripaporn; Sittiporn; Sunthareeya; Thanadachakul; Thanutsapa; Waicharoen; Warawan; Wongboot
EPI_ISL_468066	Physiology, Istanbul Medeniyet University	Physiology, Istanbul Medeniyet University	B. and Kara, A.; Caykara, B.; Cevher Keskin; H.H.; Pence; Pence, S.; Tekin, S.; Yiyit, N.
EPI_ISL_514750	Pirogov Russian National Research Medical University	Pirogov Russian National Research Medical University, Research and Development	Blagodatskikh; K.A.
EPI_ISL_443308	Plaisance	National Reference Center for Viruses of Respiratory Infections, Institut Pasteur, Paris	Angela Brisebarre; Etienne Simon-Lorière; Flora Donati; Marion Barbet; Maud Vanpeene; Mélanie Albert; Méline Bizard; Sylvie Behillil; Sylvie van der Werf; Vincent Enouf
EPI_ISL_984623 to 984627, EPI_ISL_1000038, EPI_ISL_1000040, EPI_ISL_1000042, EPI_ISL_1000044 to 1000048, EPI_ISL_1000050	Plateforme BIS Namur	GIGA Medical Genomics	Bouchra Boujemla; Cécile Meex; Keith Durkin; Maria Artesi; Marie-Pierre Hayette; Pierrette Melin; Raphaël Boreux; Sébastien Bontems; Vincent Bours
EPI_ISL_940843, EPI_ISL_940847, EPI_ISL_940849, EPI_ISL_940892	Platform BIS UZA/UAntwerpen	UAntwerp, Laboratory of Medical Microbiology	Basil Britto Xavier; Christine Lammens; Herman Goossens; Jasmine Coppens; Marie Le Mercier; Veerle Matheeussen
EPI_ISL_857042, EPI_ISL_857054 to 857055, EPI_ISL_887588, EPI_ISL_887590, EPI_ISL_888590, EPI_ISL_888592, EPI_ISL_888595, EPI_ISL_888623, EPI_ISL_888626, EPI_ISL_888818			
see above	Platform BIS UZA/UAntwerpen, University Hospital Antwerp, Edegem, Belgium	UAntwerp, Laboratory of Medical Microbiology, Campus Drie Eiken S6.26, Universiteitsplein 1, 2610, Wilrijk, Antwerp, Belgium	Basil Britto Xavier; Christine Lammens; Herman Goossens; Jasmine Coppens; Veerle Matheeussen
EPI_ISL_522547 to 522548	Platforme CYROI	UMR PIMIT Université de La Réunion	Camille Lebarbenchon; David Wilkinson; Patrick Mavingui
EPI_ISL_700430, EPI_ISL_700434, EPI_ISL_700500, EPI_ISL_700518, EPI_ISL_700528, EPI_ISL_700560	Plettenberg Bay Clinic wc PLC	NHLS/UCT	Arash Iranzadeh; Bruna Galvao; Carolyn Williamson; Deelan Doolabh; Diana Hardie; Houriiyah Tegally; Innocent Mudau; Kruger Marais; Lynn Tyers; Marvin Hsiao; Stephen Korsman
EPI_ISL_539850 to 539851	Pok Oi Hospital	Hong Kong Department of Health	Alan K.L. Tsang; Dominic N.C. Tsang; Edman T.K. Lam; Peter C.W. Yip; Rickjason C.W. Chan
EPI_ISL_891212	Population Medicine and Diagnostic Sciences, Cornell University	Population Medicine and Diagnostic Sciences, Cornell University	Cardia Caserta, L.; D.G.; E. and Diel; Mitchel; P.K.; Plachorzyk
EPI_ISL_745187	Port Nolloth Hospital	National Health Laboratory Service (NHLS), Tygerberg	Bronwyn Kleinhans; Eduan Wilkindon; Gert van Zyl; Houriiyah Tegally; Kayla Delaney; Susan Engelbrecht; Tulio de Oliveira; Wolfgang Preiser
EPI_ISL_735433	Posto de Atendimento Saude Cidade Pasc Cajati	Instituto Adolfo Lutz, Interdiciplinary Procedures Center, Strategic Laboratory	Claudia Regina Gonçalves; Claudio Tavares Sacchi; Erica Valessa Ramos Gomes; Karoline Rodrigues Campos
EPI_ISL_434699, EPI_ISL_434703, EPI_ISL_434705 to 434706, EPI_ISL_455606 to 455607	Praram 9 Hospital	National Institute of Health. Department of medical Sciences, Ministry of Public Health, Thailand	Chittaganpitch; Malinee; Okada; Parnmen; Phuygun; Pilailuk; Siripaporn; Sittiporn; Sunthareeya; Thanadachakul; Thanutsapa; Waicharoen; Warawan; Wongboot
EPI_ISL_759959	Premier Hospital Surabaya	Institute of Tropical Disease, Universitas Airlangga	Aldise M Nastri; Gatot Soegiarto; Hartono Tanto; Jezzy R Dewantari; Kazufumi Shimizu; Krisnoadi Rahardjo; Laksmi Wulandari; Maria I Lusida; Resti Yudhawati; Rima R Prasetya; Soetjipto; Yasuko Mori
EPI_ISL_418255	Presidio Ospedaliero "S. Spirito" - PESCARA	Istituto Zooprofilattico Sperimentale dell'Abruzzo e Molise "G. Caporale"	Cammà C; Di Pasquale A; Lorusso A; Mangone I; Marcacci M; Monaco F; Puglia I; Rinaldi A; Savini G
EPI_ISL_529013	Presidio Ospedaliero "S.Filippo e Nicola"-Avezzano	Istitituto Zooprofilattico Sperimentale dell'Abruzzo e Molise "G.Caporale"	Ancora M; Cammà C; Curini V; Di Domenico M; Di Pasquale A; Lorusso A; Mangone I; Marcacci M; Puglia I; Rinaldi A; Savini G.
EPI_ISL_528920 to 528921	Presidio Ospedaliero "Santo Spirito"-Pescara	Istitituto Zooprofilattico Sperimentale dell'Abruzzo e Molise "G.Caporale"	Ancora M; Cammà C; Curini V; Di Domenico M; Di Pasquale A; Lorusso A; Mangone I; Marcacci M; Puglia I; Rinaldi A; Savini G.
EPI_ISL_429226 to 429227	Presidio Ospedaliero Santo Spirito	Istituto Zooprofilattico Sperimentale dell'Abruzzo e Molise "G. Caporale"	Ancora M; Camma C; Curini V; Di Domenico M; Di Pasquale A; Lorusso A; Mangone I; Marcacci M; Puglia I; Rinaldi A; Savini G
EPI_ISL_1008678, EPI_ISL_1008703	Presidio di Brindisi Di Summa - Perrino	Istituto Zooprofilattico Sperimentale della Puglia e della Basilicata	Bianco A.; Capozzi L.; Chironna M.; Del Sambro L.; Loconsole D.; Parisi A.; Ridolfi D.; Sallustio A.; Simone D.
EPI_ISL_418258 to 418259	Presidio ospedaliero "Santo Spirito"	Istituto Zooprofilattico Sperimentale dell'Abruzzo e Molise "G. Caporale"	Ancora M; Cammà C; Curini V; Di Domenico M; Di Pasquale A; Lorusso A; Mangone I; Marcacci M; Puglia I; Rinaldi A; Savini G.
EPI_ISL_759955	Primasatya Husada Citra Hospital	Institute of Tropical Disease, Universitas Airlangga	Aldise M Nastri; Gatot Soegiarto; Jezzy R Dewantari; Kazufumi Shimizu; Krisnoadi Rahardjo; Laksmi Wulandari; Maria I Lusida; Pudji Djanuartono; Resti Yudhawati; Rima R Prasetya; Soetjipto; Yasuko Mori
EPI_ISL_436097	Prince Charles Hospital	Public Health Virology Laboratory, Forensics and Scientific Services, Queensland Health	Alyssa Pyke; Amanda De Jong; Andrew Van Den Hurk; Bixing Huang; Carmel Taylor; David Warrilow; Doris Genge; Elisabeth Gamez; Glen Hewitson; Ian Maxwell Mackay; Inga Sultana and Frederick Moore; Jamie McMahon; Jean Barcelon; Judy Northill; Lisa Leckie; Mitchell Finger; Natalie Simpson; Neelima Nair; Peter Burtonclay; Peter Moore; Sarah Wheatley; Sean Moody; Sonja Hall-Mendelin
EPI_ISL_417064, EPI_ISL_539813	Prince of Wales Hospital	Hong Kong Department of Health	Alan K.L. Tsang; Dominic N.C. Tsang; Edman T.K. Lam; Peter C.W. Yip; Rickjason C.W. Chan
EPI_ISL_539806 to 539808	Princess Margaret Hospital	Hong Kong Department of Health	Alan K.L. Tsang; Dominic N.C. Tsang; Edman T.K. Lam; Peter C.W. Yip; Rickjason C.W. Chan
EPI_ISL_770533 to 770549	Private lab	Lithuanian University of Health Sciences, Molecular cardiology lab.	Arnoldas Pautienius; Astra Vitkauskiene; Dovydas Gecys; Ingrida Olendrait; Kamile Tamusauskaite; Laura Pareckaite; Lukas Zemaitis; Vaiva Lesauskaite
EPI_ISL_514432	Prof. Massimo Zollo CEINGE TASK-FORCE COVID19 - Regione Campania	Prof. Massimo Zollo CEINGE TASK-FORCE COVID19 - Regione Campania	Angelo Boccia; Claudia Tiberio; Dae young Kong; Fatemeh asadzadeh; Giorgia Borriello; Giovanna Fusco; Giovanni Paolella; Giuseppe Castaldo; Hong Yeoul Kim and Massimo Zollo; Jae Ho Jung; Jae Myun Lee; Kyong Seop Yun; Laura Marrone; Lorenzo Chiariotti; Luigi Atripaldi; Marika Comegna; Martina Bianchi; Maurizio Viscardi; Rino Cerino; Roberto Siciliano; Sergio Brandi; Stefano Pascarella; Veronica Ferrucci
EPI_ISL_513513 to 513515, EPI_ISL_513518 to 513522, EPI_ISL_513524 to 513527, EPI_ISL_513529 to 513551, EPI_ISL_513553, EPI_ISL_513556 to 513564, EPI_ISL_513567 to 513568, EPI_ISL_513571 to 513583			
see above	Programa de Oncovirologia, Instituto Nacional de Câncer	Programa de Oncovirologia, Instituto Nacional de Câncer	Andreia C. de Melo; Brunna M. Alves; Claudia Cicala; James Arthos; João P.B. Viola; Juliana D. Siqueira; Livia R. Goes; Marcelo A. Soares
EPI_ISL_574479 to 574492	Programme in Emerging Infectious Diseases, Duke-NUS	National Public Health Laboratory, National Centre for	Adrian Eng Zheng Kang; Danielle E Anderson; Lin Cui; Raymond Tzer Pin Lin; Sophie Octavia; Tze Minn Mak; Zhenyang Zhou

	Medical School	Infectious Diseases	
EPI_ISL_527858	Pronto Atendimento Sancta Maggiore Jardim Paulista	Instituto Adolfo Lutz, Interdiciplinary Procedures Center, Strategic Laboratory	Claudia Regina Gonçalves; Claudio Tavares Sacchi; Erica Valessa Ramos Gomes
EPI_ISL_603021, EPI_ISL_693204	Pronto Socorro Dr. Conrado Cesarino Nuvolini	Instituto Adolfo Lutz, Interdiciplinary Procedures Center, Strategic Laboratory	Claudia Regina Gonçalves; Claudio Tavares Sacchi; Erica Valessa Ramos Gomes; Karoline Rodrigues Campos
EPI_ISL_471556	Pronto Socorro Jose Ibrahin	Instituto Adolfo Lutz, Interdiciplinary Procedures Center, Strategic Laboratory	Claudia Regina Gonçalves; Claudio Tavares Sacchi; Erica Valessa Ramos Gomes
EPI_ISL_527867	Pronto Socorro Municipal - Balneario São José	Instituto Adolfo Lutz, Interdiciplinary Procedures Center, Strategic Laboratory	Claudia Regina Gonçalves; Claudio Tavares Sacchi; Erica Valessa Ramos Gomes
EPI_ISL_523961	Pronto Socorro Municipal 21 de Junho	Instituto Adolfo Lutz, Interdiciplinary Procedures Center, Strategic Laboratory	Claudia Regina Gonçalves; Claudio Tavares Sacchi; Erica Valessa Ramos Gomes
EPI_ISL_515529	Pronto Socorro Municipal Julio Tupy	Instituto Adolfo Lutz, Interdiciplinary Procedures Center, Strategic Laboratory	Claudia Regina Gonçalves; Claudio Tavares Sacchi; Erica Valessa Ramos Gomes
EPI_ISL_693202	Pronto Socorro Municipal Prof. Joao Catarin Mezomo	Instituto Adolfo Lutz, Interdiciplinary Procedures Center, Strategic Laboratory	Claudia Regina Gonçalves; Claudio Tavares Sacchi; Erica Valessa Ramos Gomes; Karoline Rodrigues Campos
EPI_ISL_515554, EPI_ISL_523958 to 523959	Pronto Socorro Municipal de Perus	Instituto Adolfo Lutz, Interdiciplinary Procedures Center, Strategic Laboratory	Claudia Regina Gonçalves; Claudio Tavares Sacchi; Erica Valessa Ramos Gomes
EPI_ISL_693231	Pronto Socorro Municipal de Santa Branca	Instituto Adolfo Lutz, Interdiciplinary Procedures Center, Strategic Laboratory	Claudia Regina Gonçalves; Claudio Tavares Sacchi; Erica Valessa Ramos Gomes; Karoline Rodrigues Campos
EPI_ISL_693210	Pronto-Socorro Dr. Osmar Mesquita	Instituto Adolfo Lutz, Interdiciplinary Procedures Center, Strategic Laboratory	Claudia Regina Gonçalves; Claudio Tavares Sacchi; Erica Valessa Ramos Gomes; Karoline Rodrigues Campos
EPI_ISL_404895	Providence Regional Medical Center	Division of Viral Diseases, Centers for Disease Control and Prevention	C.R.; Gerber; Li, Y.; Lindstrom, S.; Lu, X.; Paden; Queen, K.; S.I.; Tao, Y.; Tong, S.; Zhang, J.
EPI_ISL_482292 to 482336, EPI_ISL_482338 to 482362, EPI_ISL_482364 to 482390, EPI_ISL_482392 to 482467, EPI_ISL_486830 to 486831	Providence St. Joseph Health Molecular Genomics Laboratory	Providence St. Joseph Health Molecular Genomics Laboratory	Alexa K Dowdell; Brian D Piening; Carlo B Bifulco; Fred L Robinson; Mary Campbell
EPI_ISL_538513	Provincial Health Laboratory Bekasi West Java	National Institute of Health Research and Development	AA; HA; HD; Ikawati; KD; KNA; Nugraha; Paisal; Pangesti; Pawestri; Puspa; Setiawaty, V.; Soekarso; Subangkit; T
EPI_ISL_632058 to 632059	Proximedical Urgent Care	New York City Public Health Laboratory	Jade Wang; et al.
EPI_ISL_572329	Public Health Authority of the Slovak Republic, Bratislava	Faculty of Natural Sciences, Comenius University in Bratislava	Boris Klempa; Broa Brejová; Dominika Friová; Edita Staroová; Elena Tichá; Jozef Nosek; Juraj Kopáek; Kristína Boršová; Martina Liková; Martina Neboháová; Monika Sláviková; Sabina Fumaová Havlíková; Tomáš Vina; Viktória Hodorová; Viktória abanová; ubomíra Lukáiková
EPI_ISL_516988 to 516989	Public Health Authority of the Slovak Republic, Department of Medical Microbiology	Charite Universitatsmedizin Berlin, Institute of Virology	Barbara Muehlemann; Christian Drosten; Julia Schneider; Jörn Beheim-Schwarzbach; Mgr. Edita Staronova; Talitha Veith; Terry Jones; Victor M Corman
EPI_ISL_636973	Public Health Lab	Public Health Lab	Alwasti; H
EPI_ISL_429815, EPI_ISL_469224 to 469240, EPI_ISL_482479	Public Health Laboratory	National Microbiology Laboratory	Adel Malek; Anna Majer; Elsie Grudeski; Gary Van Domselaar; George Zahariadis; Grace Seo; Jennifer Tanner; Kristyn Burak; Laura Gilbert; Matthew Gilmour; Morag Graham; Natalie Knox; Nathalie Bastien; Philip Mabon; Rhiannon Huzarewich; Robert Needle; Russell Mandes; Shari Tyson; Timothy Booth; Yan Li; Yang Yu
EPI_ISL_613463 to 613525, EPI_ISL_613527 to 613540	Public Health Laboratory - Infectious Disease Lab, Minnesota Department of Health Infectious Disease Laboratory Submission Group	Minnesota Department of Health, Public Health Laboratory	Garfin, J.; Lorentz, A.; Plumb, M.; Wang, X.
EPI_ISL_425177	Public Health Ontario	Public Health Agency of Canada - National Microbiology Laboratory	Amrit S. Boese; Anders Leung; Darwyn Kobasa; Jonathan Audet; Joshua Quick; Morag Graham; Natalie Knox; Nikesh Tailor; Shari Tyson
EPI_ISL_418322 to 418383	Public Health Ontario Laboratories	Public Health Ontario Laboratories	Aimin Li; Alireza Eshaghi; Christine Frantz; Jonathan B Gubbay; Samir N Patel; Sandeep Nagra; Vanessa G Allen
EPI_ISL_413015	Public Health Ontario Laboratory	National Microbiology Laboratory	Adrian Zetner; Anna Majer; Darryl Falzarano; Erika Landry; Gerdts Volker; Grace Seo; Guillaume Poliquin; Jocelyne Lew; Jonathan Gubbay; Matthew Gilmour; Morag Graham; Natalie Knox; Nathalie Bastien; Philip Mabon; Rob Kozak; Samira Mubareka; Shari Tyson; Stephanie Booth; Timothy Booth; Tom Graefenhan; Yan Li
EPI_ISL_413014	Public Health Ontario Laboratory	Ontario Agency for Health Protection and Promotion (OAHPP)	Aimin Li; Alireza Eshaghi; Christine Frantz; Jonathan B Gubbay; Samir N Patel; Sandeep Nagra; Vanessa G Allen
EPI_ISL_548687, EPI_ISL_548690 to 548705, EPI_ISL_548708 to 548709, EPI_ISL_548711 to 548714, EPI_ISL_548716 to 548718, EPI_ISL_548720 to 548724, EPI_ISL_548726 to 548735, EPI_ISL_548737 to 548738, EPI_ISL_548740 to 548747, EPI_ISL_548750 to 548755, EPI_ISL_548759 to 548796, EPI_ISL_548798 to 548862, EPI_ISL_548864 to 548866, EPI_ISL_548868 to 548879, EPI_ISL_548881, EPI_ISL_548884, EPI_ISL_548886 to 548896, EPI_ISL_548898 to 548906, EPI_ISL_548908 to 548935, EPI_ISL_548937 to 548941, EPI_ISL_636907 to 636925, EPI_ISL_636927 to 636947, EPI_ISL_636949 to 636959, EPI_ISL_671496 to 671603, EPI_ISL_671605 to 671606, EPI_ISL_966036 to 966043, EPI_ISL_966046 to 966058			
see above	Public Health Ontario Laboratory	Public Health Ontario Laboratory	Aimin Li; Alireza Eshaghi; Andre Villegas; Ashleigh Sullivan; Christine Frantz; Dean Maxwell; Esha Joshi; Heather Rilkoff; Jared Simpson; Jennifer L Guthrie; Jonathan B Gubbay; Karthikeyan Sivaraman; Lawrence Heisler; Matthew Watson; Michael CY Li; Michael Laszloffy; Nahuel Fittipaldi; Philip Banh; Richard de Borja; Samir N Patel; Sandeep Nagra; Sandra Zittermann; Sarah Teatero; Vanessa G Allen; Yao Chen; Yogi Sundaravadanam
EPI_ISL_576131 to 576144, EPI_ISL_593597 to 593616, EPI_ISL_593618 to 593636, EPI_ISL_593638 to 593645, EPI_ISL_639741 to 639749, EPI_ISL_639751 to 639820, EPI_ISL_641309 to 641310, EPI_ISL_693250 to 693297, EPI_ISL_849749 to 849751, EPI_ISL_849753, EPI_ISL_849755, EPI_ISL_849758			
see above	Public Health Virology Laboratory, Forensic and Scientific Services (PHV-FSS)	Public Health Virology Laboratory, Forensic and Scientific Services (PHV-FSS)	Son Nguyen et al
EPI_ISL_985235	Public Health Virology-Forensic and Scientific Services	Public Health Virology-Forensic and Scientific Services	Son Nguyen et al
EPI_ISL_944644, EPI_ISL_944737, EPI_ISL_944739, EPI_ISL_944742, EPI_ISL_944746 to 944747, EPI_ISL_962180, EPI_ISL_968081			
see above	Public Health Virology-Forensic and Scientific Services (PHV-FSS)	Public Health Virology-Forensic and Scientific Services (PHV-FSS)	Alyssa T. Pyke et al.; Son Nguyen et al
EPI_ISL_512785, EPI_ISL_512787, EPI_ISL_512789 to 512790, EPI_ISL_512795, EPI_ISL_512797 to 512803, EPI_ISL_512805 to 512809, EPI_ISL_513310			
see above	Public Health, United States Air Force School of Aerospace Medicine	Public Health, United States Air Force School of Aerospace Medicine	A.C.; A.K.; A.W.; B.C.; C.R.; Chapleau; Connors; E.A.; Fries; J.R.; Javorina; Lambert; Macias; Meyer; Purves; R.R.; R.R. and Starr; S.M.; Starr
EPI_ISL_513312	Public Health, United States Air Force School of Aerospace Medicine	Public Health, United States Air Force School of Aerospace Medicine	A.C.; A.K.; A.W.; B.C.; C.R.; Chapleau; Connors; E.A.; Fries; J.R.; Javorina; Lambert; Macias; Meyer; Purves; R.R. and Starr; S.M.
EPI_ISL_434541, EPI_ISL_434543, EPI_ISL_434545 to 434549, EPI_ISL_434551	Puerto Rico Department of Health	Centers for Disease Control and Prevention, Dengue Branch	Betzabel Flores; Chaney Kalinich; Fabiola Cruz; Gilberto A. Santiago; Glenda Gonzalez; Jessica I. Falcon; Jorge L. Munoz-Jordan; Joseph Fauver; Keyla Charriez; Nathan Grubaugh
EPI_ISL_437189	Pusat Pertamina Hospital	Eijkman Institute for Molecular Biology, Ministry of Research and Technology/National Agency for Research and Innovation	Amin Soebandrio; David H Muljono; Edison Johar; Frilasita A Yudhaputri; Hidayat Trimarsanto; Khin Saw Myint; Safarina G Malik
EPI_ISL_632936	Puskesmas Mlati 1 Sleman	Genetics Working Group (Pokja Genetik) Faculty of Medicine,	Afiahayati; Bambang Sigit Riyanto; Dwi AA Nugrahaningsih; Edwin W. Daniwijaya; Eggi Arguni; Eko Budiono; Endah Supriyati; Ernawati; Gunadi; Hendra

		Public Health and Nursing Universitas Gadjah Mada (FK-KMK UGM); Disease Investigation Center Wates Ministry of Agriculture Indonesia; Department of Microbiology FK-KMK UGM; Laboratorium Diagnostik Yayasan Tahija World Mosquito Program (WMP) Yogyakarta Center for Tropical Medicine FK-KMK UGM; Integrated Research Center FK-KMK UGM; Department of Computer Science and Electronics FMIPA UGM; RSUP Dr. Sardjito	Wibawa; Heni Retnowulan; Ika Trisnawati; Ira Puspitawati; Kristy Iskandar; Ludhang P. Rizki; Marcellus; Mohamad S. Hakim; Munawar Gani; Nungki Anggorowati; Nur Imma Fatimah Harahap; Nur Rahmi Ananda; Osman Sianipar; Riat El Khair; Satria Maulana; Siswanto; Sumardi; Titik Nuryastuti; Tri Wibawa; Umi Solekhah Intansari; Untung Wirawan; William Widitjiarso; Yunika Puspadewi; Elizabeth Henny Herningtiyas
EPI_ISL_766037	Puskesmas Palmerah	Eijkman Institute for Molecular Biology, Ministry of Research and Technology/National Agency for Research and Innovation	Amin Soebandrio; David H Muljono; Edison Johar; Frilasita A Yudhaputri; Hidayat Trimarsanto; Khin Saw Myint; Safarina G Malik
EPI_ISL_574610	Puskesmas Tambora	Eijkman Institute for Molecular Biology, Ministry of Research and Technology/National Agency for Research and Innovation	Amin Soebandrio; David H Muljono; Edison Johar; Frilasita A Yudhaputri; Herawati Sudoyo; Hidayat Trimarsanto; Iskandar A Adnan; Khin Saw Myint; Safarina G Malik; Willy Agustine

EPI_ISL_579515 to 579517, EPI_ISL_579530, EPI_ISL_579534, EPI_ISL_579537 to 579577, EPI_ISL_579579 to 579583, EPI_ISL_579585 to 579593, EPI_ISL_579597 to 579598, EPI_ISL_579600, EPI_ISL_579604 to 579605, EPI_ISL_579607, EPI_ISL_579613, EPI_ISL_579616, EPI_ISL_579619, EPI_ISL_579636, EPI_ISL_579638, EPI_ISL_579640, EPI_ISL_579649, EPI_ISL_579664, EPI_ISL_579669, EPI_ISL_579679, EPI_ISL_579696, EPI_ISL_960224 to 960225

see above	QEII Health Sciences Centre	National Microbiology Laboratory (NML)	Anna Majer; Anneliese Landgraff; CanCOGeN's metadata curation team; Dan Gaston; Darian Hole; Elsie Grudeski; Gary Van Domselaar; Grace Seo; Janice Pettipas; Jason LeBlanc; Jennifer Tanner; Kirsten Biggar; Madison Chapel; Morag Graham; Natalie Knox; Nathalie Bastien; Philip Mabon; Public Health Agency of Canada CanCOGeN team; Public Health Agency of Canada's CanCOGeN team; Rhiannon Huzarewich; Russell Mandes; Shari Tyson; Timothy Booth; Todd Hatchette; Yan Li
EPI_ISL_444612	QML Pathology	Public Health Virology Laboratory	Alyssa Pyke; Amanda De Jong; Andrew Van Den Hurk; Bixing Huang; Carmel Taylor; David Warrilow; Doris Genge; Elisabeth Gamez; Glen Hewitson; Ian Maxwell Mackay; Inga Sultana; Jamie McMahon; Jean Barcelon; Judy Northill; Mitchell Finger; Natalie Simpson; Neelima Nair; Peter Burtonclay; Peter Moore; Sarah Wheatley; Sean Moody; Sonja Hall-Mendelin; Timothy Gardam; and Frederick Moore
EPI_ISL_613457	QUARANTINE CAMP	Hong Kong Department of Health	Alan K.L. Tsang; Dominic N.C. Tsang; Edman T.K. Lam; Peter C.W. Yip; Rickjason C.W. Chan
EPI_ISL_518819	Qadr Hospital, Tangerang, Banten	Biosafety Level-3 Laboratory, Indonesian Institute of Sciences (LIPI)	Ade Andriani; Ahmad Fathoni; Andri Wardiana; Anggia Prasetyoputri; Anik Budhi Dharmayanthi; Ario Betha Juanssilfero; Asep M Ridwanuloh; Isa Nuryana; Puspita Lisdiyanti; Ratih Asmana Ningrum; Rifki Sadikin; Syam Budi Iryanto; Wien Kusharyoto

EPI_ISL_448223 to 448230, EPI_ISL_448232 to 448251, EPI_ISL_448253 to 448256, EPI_ISL_448258 to 448274, EPI_ISL_448276 to 448283, EPI_ISL_448285 to 448297, EPI_ISL_448299 to 448301, EPI_ISL_448304 to 448309, EPI_ISL_448311 to 448317, EPI_ISL_448320 to 448332, EPI_ISL_448335 to 448336, EPI_ISL_448338 to 448342, EPI_ISL_448344 to 448346, EPI_ISL_448348, EPI_ISL_448350 to 448352, EPI_ISL_448355, EPI_ISL_448357 to 448366, EPI_ISL_448369 to 448372, EPI_ISL_448374 to 448377, EPI_ISL_448379 to 448380, EPI_ISL_448383 to 448385, EPI_ISL_448387 to 448388, EPI_ISL_448979 to 448988, EPI_ISL_448991 to 448996, EPI_ISL_448998 to 448999, EPI_ISL_449001 to 449013, EPI_ISL_449015, EPI_ISL_449017, EPI_ISL_449019 to 449032, EPI_ISL_449034 to 449051, EPI_ISL_449053 to 449057, EPI_ISL_449059 to 449063, EPI_ISL_449065 to 449066, EPI_ISL_449068 to 449075, EPI_ISL_449077 to 449087, EPI_ISL_449089 to 449097, EPI_ISL_449099 to 449107, EPI_ISL_449109 to 449112, EPI_ISL_449115 to 449119, EPI_ISL_449121 to 449122, EPI_ISL_449124 to 449133, EPI_ISL_449135 to 449146, EPI_ISL_449148 to 449175, EPI_ISL_453545 to 453548, EPI_ISL_453550 to 453556, EPI_ISL_453558 to 453561, EPI_ISL_453563, EPI_ISL_453565 to 453567, EPI_ISL_453569 to 453575, EPI_ISL_453577, EPI_ISL_453579 to 453580, EPI_ISL_453582 to 453588, EPI_ISL_453591 to 453601, EPI_ISL_453603 to 453613, EPI_ISL_457326, EPI_ISL_457328 to 457342, EPI_ISL_457344 to 457347, EPI_ISL_457350 to 457358, EPI_ISL_457360 to 457362, EPI_ISL_457365 to 457371, EPI_ISL_457373 to 457375, EPI_ISL_457377 to 457383, EPI_ISL_457385 to 457392, EPI_ISL_457394 to 457395, EPI_ISL_457397, EPI_ISL_457401 to 457403, EPI_ISL_457405 to 457409, EPI_ISL_457411 to 457420, EPI_ISL_457423, EPI_ISL_457425 to 457427, EPI_ISL_457430 to 457431, EPI_ISL_457433 to 457434, EPI_ISL_457436 to 457438, EPI_ISL_457440 to 457442, EPI_ISL_457445 to 457449, EPI_ISL_457451 to 457452, EPI_ISL_457455 to 457465, EPI_ISL_457467 to 457480, EPI_ISL_457482 to 457501, EPI_ISL_457503 to 457507, EPI_ISL_457509 to 457518, EPI_ISL_457520 to 457521, EPI_ISL_457523 to 457525, EPI_ISL_457527 to 457535, EPI_ISL_457538 to 457548, EPI_ISL_457550 to 457553, EPI_ISL_457555 to 457556, EPI_ISL_457558 to 457563, EPI_ISL_457565 to 457572, EPI_ISL_461805 to 461806, EPI_ISL_461809 to 461814, EPI_ISL_461816 to 461817, EPI_ISL_461820 to 461823, EPI_ISL_461825, EPI_ISL_461827 to 461829, EPI_ISL_461836, EPI_ISL_461839, EPI_ISL_461844, EPI_ISL_461850, EPI_ISL_461857 to 461858, EPI_ISL_461860 to 461868, EPI_ISL_461870 to 461872, EPI_ISL_461876, EPI_ISL_461879 to 461881, EPI_ISL_461883 to 461885, EPI_ISL_461887 to 461888, EPI_ISL_461890, EPI_ISL_472291, EPI_ISL_472296, EPI_ISL_472300 to 472303, EPI_ISL_472305 to 472307, EPI_ISL_472310 to 472311, EPI_ISL_472313 to 472314, EPI_ISL_472316 to 472317, EPI_ISL_472320, EPI_ISL_472322, EPI_ISL_472326, EPI_ISL_472328, EPI_ISL_472330, EPI_ISL_472334, EPI_ISL_472336, EPI_ISL_472342 to 472343, EPI_ISL_472347, EPI_ISL_472349, EPI_ISL_472354, EPI_ISL_472356, EPI_ISL_472361, EPI_ISL_472363, EPI_ISL_472365 to 472368, EPI_ISL_472371, EPI_ISL_472375 to 472376, EPI_ISL_472381, EPI_ISL_478719 to 478720, EPI_ISL_490498, EPI_ISL_490502, EPI_ISL_490504 to 490507, EPI_ISL_490509, EPI_ISL_490511, EPI_ISL_490514 to 490515, EPI_ISL_490519 to 490520, EPI_ISL_490524, EPI_ISL_490526, EPI_ISL_490528 to 490530, EPI_ISL_490532 to 490533, EPI_ISL_490537 to 490538, EPI_ISL_490540 to 490541, EPI_ISL_490543, EPI_ISL_490549 to 490551, EPI_ISL_490556 to 490558, EPI_ISL_493542 to 493544, EPI_ISL_529238, EPI_ISL_529240 to 529250, EPI_ISL_529557 to 529589, EPI_ISL_540691 to 540695, EPI_ISL_540698, EPI_ISL_549336, EPI_ISL_549344, EPI_ISL_549349 to 549351, EPI_ISL_549477 to 549490, EPI_ISL_572711, EPI_ISL_572717 to 572720, EPI_ISL_572847, EPI_ISL_572939 to 572941, EPI_ISL_572954, EPI_ISL_573085 to 573131, EPI_ISL_573414 to 573417, EPI_ISL_577099, EPI_ISL_577102 to 577103, EPI_ISL_577107, EPI_ISL_577109, EPI_ISL_584165 to 584166, EPI_ISL_584717, EPI_ISL_584729, EPI_ISL_584737 to 584738, EPI_ISL_584746, EPI_ISL_584915, EPI_ISL_584939, EPI_ISL_584945, EPI_ISL_584947, EPI_ISL_584968, EPI_ISL_585591, EPI_ISL_585603, EPI_ISL_585610, EPI_ISL_595115 to 595127, EPI_ISL_595129 to 595135, EPI_ISL_595140 to 595143, EPI_ISL_595145 to 595152, EPI_ISL_595155, EPI_ISL_595157, EPI_ISL_595159, EPI_ISL_595161 to 595162, EPI_ISL_595169, EPI_ISL_595172, EPI_ISL_595177, EPI_ISL_595179, EPI_ISL_595184, EPI_ISL_595190, EPI_ISL_595201, EPI_ISL_595203, EPI_ISL_595205 to 595206, EPI_ISL_595210, EPI_ISL_595212 to 595213, EPI_ISL_595219, EPI_ISL_595222, EPI_ISL_595224, EPI_ISL_595227 to 595229, EPI_ISL_595231, EPI_ISL_595843 to 595845, EPI_ISL_595848, EPI_ISL_595852, EPI_ISL_595854 to 595856, EPI_ISL_595858, EPI_ISL_595862 to 595864, EPI_ISL_595866 to 595867, EPI_ISL_595871 to 595872, EPI_ISL_595875 to 595880, EPI_ISL_595882, EPI_ISL_595884, EPI_ISL_595886, EPI_ISL_595888 to 595897, EPI_ISL_595899 to 595900, EPI_ISL_595902 to 595906, EPI_ISL_595908 to 595914, EPI_ISL_595916 to 595918, EPI_ISL_595920, EPI_ISL_595922, EPI_ISL_595926 to 595927, EPI_ISL_595929 to 595931, EPI_ISL_595935 to 595938, EPI_ISL_595940 to 595943, EPI_ISL_595945, EPI_ISL_595947 to 595948, EPI_ISL_595950 to 595951, EPI_ISL_595954, EPI_ISL_595956 to 595957, EPI_ISL_595960, EPI_ISL_595962 to 595963, EPI_ISL_595965, EPI_ISL_595967 to 595971, EPI_ISL_595974, EPI_ISL_595976 to 595983, EPI_ISL_595987 to 595990, EPI_ISL_595992 to 595993, EPI_ISL_595995, EPI_ISL_595997 to 595999, EPI_ISL_596001 to 596011, EPI_ISL_596014, EPI_ISL_596016 to 596018, EPI_ISL_596020 to 596024, EPI_ISL_596026 to 596027, EPI_ISL_596031, EPI_ISL_596033 to 596036, EPI_ISL_596038 to 596039, EPI_ISL_596041, EPI_ISL_596045 to 596051, EPI_ISL_596053, EPI_ISL_596056 to 596059, EPI_ISL_596063 to 596065, EPI_ISL_596067 to 596085, EPI_ISL_596087 to 596093, EPI_ISL_596095 to 596096, EPI_ISL_596099 to 596108, EPI_ISL_596110 to 596112, EPI_ISL_596114 to 596138, EPI_ISL_596140 to 596143, EPI_ISL_596145 to 596147, EPI_ISL_596149 to 596150, EPI_ISL_596152 to 596157, EPI_ISL_596159 to 596172, EPI_ISL_596174, EPI_ISL_596176 to 596185, EPI_ISL_596188 to 596189, EPI_ISL_627071 to 627077, EPI_ISL_627080 to 627081, EPI_ISL_627083 to 627084, EPI_ISL_627213 to 627214, EPI_ISL_627300, EPI_ISL_627570 to 627665, EPI_ISL_637857, EPI_ISL_637864 to 637866, EPI_ISL_638581 to 638583, EPI_ISL_651089 to 651094, EPI_ISL_651100 to 651102, EPI_ISL_651418 to 651419, EPI_ISL_651434, EPI_ISL_651438, EPI_ISL_651484, EPI_ISL_651526, EPI_ISL_652151 to 652156, EPI_ISL_652216 to 652285, EPI_ISL_652348 to 652363, EPI_ISL_653027 to 653058, EPI_ISL_664741, EPI_ISL_664743, EPI_ISL_664747, EPI_ISL_664764, EPI_ISL_664857 to 664940, EPI_ISL_665788, EPI_ISL_665850, EPI_ISL_665865 to 665867, EPI_ISL_665876 to 665897, EPI_ISL_665957 to 665963, EPI_ISL_678917, EPI_ISL_678919, EPI_ISL_704842, EPI_ISL_704847, EPI_ISL_704850, EPI_ISL_704853, EPI_ISL_704855, EPI_ISL_704858, EPI_ISL_704887, EPI_ISL_704890, EPI_ISL_704892, EPI_ISL_704908, EPI_ISL_704911, EPI_ISL_704914, EPI_ISL_704916, EPI_ISL_704921, EPI_ISL_704923, EPI_ISL_704926, EPI_ISL_704929, EPI_ISL_705422, EPI_ISL_705431 to 705432, EPI_ISL_705567 to 705612, EPI_ISL_706356 to 706382, EPI_ISL_706384 to 706411, EPI_ISL_706413 to 706419, EPI_ISL_723673, EPI_ISL_724125, EPI_ISL_724127 to 724131, EPI_ISL_724133, EPI_ISL_724135, EPI_ISL_724137 to 724138, EPI_ISL_724142, EPI_ISL_724144, EPI_ISL_724148 to 724150, EPI_ISL_724152, EPI_ISL_724154 to 724155, EPI_ISL_724157, EPI_ISL_724159, EPI_ISL_724161 to 724162, EPI_ISL_724168, EPI_ISL_724172, EPI_ISL_724174, EPI_ISL_724178 to 724179, EPI_ISL_724184 to 724185, EPI_ISL_724187 to 724188, EPI_ISL_724191, EPI_ISL_724193, EPI_ISL_724195, EPI_ISL_724197, EPI_ISL_724201 to 724209, EPI_ISL_725029, EPI_ISL_725031, EPI_ISL_725033, EPI_ISL_725036, EPI_ISL_725038 to 725042, EPI_ISL_725045 to 725047, EPI_ISL_725049 to 725051, EPI_ISL_725054 to 725060, EPI_ISL_725062 to 725064, EPI_ISL_725066, EPI_ISL_725068 to 725071, EPI_ISL_725073 to 725074, EPI_ISL_725077 to 725078, EPI_ISL_725080, EPI_ISL_725082, EPI_ISL_725085, EPI_ISL_725088, EPI_ISL_725093, EPI_ISL_725095 to 725096, EPI_ISL_725098, EPI_ISL_725101, EPI_ISL_725103, EPI_ISL_725107 to 725108, EPI_ISL_725110 to 725111, EPI_ISL_725113, EPI_ISL_725115 to 725117, EPI_ISL_725119 to 725120, EPI_ISL_725122 to 725123, EPI_ISL_725125, EPI_ISL_725128 to 725131, EPI_ISL_725133 to 725134, EPI_ISL_725137, EPI_ISL_725139 to 725141, EPI_ISL_725143, EPI_ISL_725145 to 725148, EPI_ISL_725150 to 725151, EPI_ISL_725155 to 725156, EPI_ISL_725160 to 725163, EPI_ISL_725166 to 725167, EPI_ISL_725172, EPI_ISL_725174 to 725175, EPI_ISL_725177 to 725180, EPI_ISL_725184, EPI_ISL_725186 to 725196, EPI_ISL_725205, EPI_ISL_725211, EPI_ISL_725216, EPI_ISL_725220, EPI_ISL_725223 to 725224, EPI_ISL_725226 to 725228, EPI_ISL_725230, EPI_ISL_725232 to 725233, EPI_ISL_725236 to 725241, EPI_ISL_725246, EPI_ISL_725248 to 725250, EPI_ISL_725252 to 725253, EPI_ISL_725255 to 725256, EPI_ISL_725260 to 725261, EPI_ISL_725264, EPI_ISL_725266 to 725270, EPI_ISL_725273 to 725274, EPI_ISL_725276, EPI_ISL_725278 to 725279, EPI_ISL_725281 to 725282, EPI_ISL_725285, EPI_ISL_725288 to 725289, EPI_ISL_725291 to 725298, EPI_ISL_725300 to 725301, EPI_ISL_725303, EPI_ISL_725306 to 725309, EPI_ISL_725311, EPI_ISL_725315, EPI_ISL_725318 to 725319, EPI_ISL_725323 to 725324, EPI_ISL_725326 to 725329, EPI_ISL_725331 to 725332, EPI_ISL_725335 to 725336, EPI_ISL_725338 to 725340, EPI_ISL_725342 to 725344, EPI_ISL_725348 to 725349, EPI_ISL_725354, EPI_ISL_725359, EPI_ISL_725362 to 725363, EPI_ISL_725365, EPI_ISL_725368, EPI_ISL_725371, EPI_ISL_725374, EPI_ISL_725376 to 725377, EPI_ISL_725379, EPI_ISL_725381, EPI_ISL_725384, EPI_ISL_725386, EPI_ISL_725389, EPI_ISL_725391, EPI_ISL_725393, EPI_ISL_725396, EPI_ISL_725398 to 725401, EPI_ISL_725403, EPI_ISL_725405 to 725406, EPI_ISL_725408 to 725410, EPI_ISL_725412 to 725416, EPI_ISL_725424, EPI_ISL_725428 to 725431, EPI_ISL_725434 to 725437, EPI_ISL_725439 to 725441, EPI_ISL_725443, EPI_ISL_725449 to 725450, EPI_ISL_725455, EPI_ISL_725460, EPI_ISL_725463 to 725464, EPI_ISL_725466 to 725467, EPI_ISL_725470 to 725473, EPI_ISL_725477, EPI_ISL_725481 to 725484, EPI_ISL_725486, EPI_ISL_725489 to 725490, EPI_ISL_725495, EPI_ISL_725497 to 725498, EPI_ISL_725501 to 725502, EPI_ISL_725505, EPI_ISL_725507, EPI_ISL_725510 to 725511,
[truncated: 565,153 more chars]
