# Supplementary material for: Highly accurate whole-genome imputation of SARS-CoV-2 from partial or low-quality sequences
Source: Gigascience. 2021 Dec 2;10(12):giab078. doi: 10.1093/gigascience/giab078 (PMC8643610; doi:10.1093/gigascience/giab078)
Supplement: giab078_Response_to_Reviewer_Comments_Original_Submission [file giab078_response_to_reviewer_comments_original_submission.pdf]

Author's Response To Reviewer Comments

Close

Editor COMMENT
==============
As the reviewers were split on their advice we got some additional input from our Editorial Board. They agreed that this method could be pretty useful, but the paper needs more work to make this rationale and advantages more clear. To do this they felt it would be good to make sure you clearly clarify lineages and perhaps regions where it doesn't work so well. Following the suggestion of referee 2 it would also be good if you could include indels too.
In addition, please register any new software application in the bio.tools and SciCrunch.org databases to receive RRID (Research Resource Identification Initiative ID) and biotoolsID identifiers, and include these in your manuscript. This will facilitate tracking, reproducibility and re-use of your tool.
RESPONSE
=========
We are very happy to see that one of the referees and the editorial board found the tool useful (we are actually using it a lot). We have addressed all the comments of the referees (including indels in the imputation as well) and, following your request, we have registered the application in the bio.tools and SciCrunch.org databases. We have received the following identifiers: biotools:impusars and RRID:SCR_021707, respectively. These identifiers have been added to the manuscript.

Reviewer #1:

COMMENT
=========
The authors have introduced an imputation pipeline that integrated softwares of minimac 3, minimac 4 and PANGOLIN to impute the variant of the missing region of the SARS-CoV-2 sequencing data. The accuracy of the imputation for genotyping assay kits is around 0.9. The idea is interesting and may be helpful in a few limited scenario. However, given the high mutation rate of the SARS-CoV-2 and for most of the studies that can generate high quality SARS-CoV-2 (reference-based) genome assembly, I don't think the method will be widely used in the SARS-CoV-2 studies. In addition, it lacks a bit genuine creativity in terms of mathematics behind the method. I think the author's study may be more suitable for a journal like bioinformatics.
RESPONSE
=========
We are coordinating the COVID-19 circuit of genomic surveillance in Andalusia (https://www.clinbioinfosspa.es/COVID_circuit/), the third largest region in Europe with a population similar to countries like Switzerland or Austria and, contrarily to the impression of the referee, this application has received an enthusiastic acceptance from our users. What is original here is the application of the concept of imputation. We are not intending to improve imputation methods, but to use them in a field in which they have scarcely been used. It is true that imputation was unnecessary in conventional virology studies, but we are dealing now with a completely different scenario, of massive sequencing in which a number of errors are inevitable and can be corrected by imputation. And this scenario is here to stay, now with COVID and with other viruses in the future, because viral genome sequencing has demonstrated its usefulness in viral epidemiology.

Reviewer #2:
In their manuscript, Ortuno et al. develop a procedure for imputing missing genotypes of SARS-CoV-2. Missing genotypes can arise from fragmented whole genome assemblies, targeted sequencing (e.g. spike protein), or incomplete genotype panels. I really like this idea and thought the paper was conducted quite carefully. I was impressed by the high level of precision across all experiments. I have a few minor comments, questions, and suggestions below:

Major comments:

COMMENT

=========
My understanding is that only SNPs are imputed by the program. Is this correct? If this is the case, can the authors comment on the frequency of other types of variants in the SARS-CoV-2 genome? How common are small indels, large indels, or rearrangements?
RESPONSE
=========
The reviewer is right, impuSARS was originally implemented and validated only for SNPs. The main reason was the complexity of building a reference considering both SNPs and indels, along with the higher relevance of SNPs to determine different lineages. Nevertheless, given the increasing importance of small indels in recent lineages like Delta, we have made available a new reference including both SNPs and small indels and expanding the number of sequences and lineages. As mentioned in the manuscript, the reference will be periodically updated to contain not only new emerging lineages but also other types of mutations not considered before.
As the reviewer suggests, we have included a supplementary figure (Fig. S4) in the manuscript to show the frequency of each type of variant, thus justifying the creation of the new reference. As shown in that figure, SNPs are the predominant type of mutations in SARS-CoV-2 with an average of >33 SNPs per sequence. Otherwise, only an average of 3.12 insertions are found per sequence whereas deletions are almost zero (0.04). Also, those indels are commonly short, in the range between 3 to 12 nucleotides.

COMMENT
=========
Can the authors include code for building their reference panel? This would enable the same pipeline to be applied to updated SARS-CoV-2 references or to other kinds of viruses entirely. For example, metagenomic DNA sequencing often yields partial viral genomes, and it would be great to use this same pipeline to impute these genomes (where sufficient references exist).
RESPONSE
=========
This is a good suggestion. The newly built docker for impuSARS now includes an additional command, named impuSARS_reference, to allow users building their own reference panel. The main input for this new functionality will be a FASTA file with all sequences adequately aligned. Additionally, users should also provide the reference sequence in FASTA format, the character chosen for missing regions (if any) and the name and path for the output reference. Detailed instructions of usage are added to the tool repository. Additionally, the main script impuSARS has been also updated with new optional arguments to allow users introducing their own reference panels for customized imputations. The manuscript has been updated to describe this new functionality.

COMMENT
=========
I noticed that several of the PANGOLIN lineages seem especially hard to impute. Can the authors comment on why this might be the case? Regarding the PANGOLIN lineages, how to these correspond to specific variants of interest (e.g. delta variant)? Is this information provided to users? A visual could really help here showing the phylogenetic relationships between PANGOLIN lineages and how they relate to variants of interest.
RESPONSE
=========
Thank you very much for bringing this interesting point. As the reviewer mentions, some lineages could result in less precise imputations as Figure 5 depicts. Essentially, the imputation efficiency depends on how representative is region captured for each specific lineage. That is, if the captured regions do not include variations that are specific for that lineage, impuSARS is not able to differentiate between different lineages and may misclassify lineages after imputation. That is the case, for example, for the lineage B.1.1.119 when capturing regions from the genotyping assay kit DeepChek®-8-plex CoV-2 or B.1 lineage when only capturing spike protein regions. Nevertheless, these lineages are less interesting for prospective imputation purposes because they belong to a very initial phase of the coronavirus pandemics and are very close in the phylogenetic tree. In other words, they do not provide important alterations to make them clearly distinguishable or to consider them variants of interest/concern (VOC/VOI). Moreover, most of them are extinct nowadays. On the other hand, the manuscript has strongly validated that important lineages classified as VOC/VOI were accurately imputed and lineages correctly obtained. The manuscript has been updated to discuss this behavior and discuss the reasons in detail.
Regarding the new VOC/VOI names, we would like to clarify that these nomenclatures were not incorporated yet by the time this manuscript was written. Indeed, latest PANGOLIN versions now specify the exact correspondence between its lineages and standard VOI identifiers. For example, B.1.1.7 (Alpha

variant) or B.1.351 (Beta variant). Therefore, the lineages obtained by impuSARS will also include such information and will be provided to users. Nevertheless, for the sake of clarity, we have updated any reference to VOCs in the manuscript by their current nomenclature (instead of using British, South African, Brazilian, etc.) Table 3 provides a visual grasp on the distribution of mutations across variants.

COMMENT
=========
The authors indicate that missing regions of partial genome assemblies must be indicated by Ns. This seems like an artificial constraint that may be a pain point for users. Can the authors modify their program to detect missing regions from FASTA files and automatically fill these regions with Ns prior to imputation?
RESPONSE
=========
Again, the referee pointed to another interesting point. Most bioinformatic pipelines for SARS-CoV-2 sequencing use Ns to indicate a region that cannot be captured or coverage/quality is not enough to determine nucleotides in that region. In fact, that is the case for all sequences used as reference panel from GISAID. And typically, FASTA files use this convention as well, to distinguish unresolved sequence stretches from real gaps in the sequence. As the reviewer suggests, this situation can be different for those cases where a capture kit is designed for a specific sequence region. There, only the captured sequence is provided and missing regions are not filled with any character (just missing). In that case, users do not need to manually fill the sequence since impuSARS already aligns the captured sequence with the viral reference, thus identifying those missing regions and imputing them. Therefore, no input preparation is needed from users. Moreover, since impuSARS now imputes indels as well, potential conflicts between missing bases or insertions/deletions would be solved in the imputation process.
We have clarified in the manuscript that both options Ns or just the absence of regions are valid for our tool.

Minor comments:

COMMENT
=========
For the installation options, please provide an alternative to docker. Would it be feasible to add an installation option using conda?
RESPONSE
=========
We completely agree with the referee that an installation with conda could simplify the execution, having encapsulated all external tools in one package. Regrettably, impuSARS includes dependencies to tools like Minimac that are unavailable in bioconda, probably because it lacks standard installation procedures. This dependency makes it harder to create an all-in conda package, since Minimac should be installed separately to satisfy dependencies. Another solution would be to adapt Minimac to conda but this would need more effort, especially in terms of reimplementation licences and the non-standard installation procedure, among other things. For those reasons, we chose docker as a first option, where we could encapsulate tools from different sources/languages into a reproducible and distributable environment. Nevertheless, we will keep exploring a way to make Minimac available in conda and, therefore, integrate the entire impuSARS tool as an all-in conda package in the future.

COMMENT
=========
In their methods, could the authors clearly define true positives, true negatives, false positives, and false negatives in the context of their validation experiments? Related to this point, I noticed that the precision is consistently high in the validation experiments, but recall can be quite low. I assume this means that the program will not impute a genotype where there is insufficient evidence, leaving it as a "N". In this case, users should have high confidence in all imputed genotypes. Is this correct?
RESPONSE
=========
We have incorporated in the manuscript a brief explanation about how true positives, false positives, true negatives and false negatives are obtained together with the prediction metrics. We hope that now the manuscript results clearer in this respect.
Regarding the second part, recall is consistently lower mainly due to private and rare mutations. That is, each sequence includes a set of private mutations or vary low frequency mutations which are hard or impossible to impute because there is not enough representation. This is a universal problem for any imputation tool or strategy. These mutations are then frequently assigned to reference making the

number of false positives increased, thus reducing the recall. However, these mutations are not informative for lineage classification and their imputation is less relevant for our classification purpose. We have added a comment clarifying this point.

COMMENT
=========
All the figures in the manuscript were of low resolution and difficult to read.
RESPONSE
=========
This is surprising, because the figures we uploaded were at high resolution. It might be the PDF built by the submission system that includes the figures at the end of the manuscript and probably compress them. The figures independently submitted are high resolution. I do not know how is the referee's interface but probably it allows accessing to the original figures.

COMMENT
=========
The authors should use a consistent tense (present or past) throughout the manuscript. In some places future tense was even used: "Once we have validated the robustness of our imputation against different missing regions scenarios, the validation will focus on the imputation of variants"
RESPONSE
=========
We apologize for this, which is the result of a multi-author document writing. We have reviewed carefully the text to make the tenses consistent. Thanks for calling out attention on this.

Close
