# Supplementary material for: Highly accurate whole-genome imputation of SARS-CoV-2 from partial or low-quality sequences
Source: Gigascience. 2021 Dec 2;10(12):giab078. doi: 10.1093/gigascience/giab078 (PMC8643610; doi:10.1093/gigascience/giab078)
Supplement: giab078_Response_to_Reviewer_Comments_Revision_1 [file giab078_response_to_reviewer_comments_revision_1.pdf]

Author's Response To Reviewer Comments

Close

Editor comments:
Please check all the references are up-to-date (e.g. the preprint in Ref 4 is now out https://www.nature.com/articles/s41586-021-03677-y), and software is cited according to the software citation guidelines (https://doi.org/10.12688/f1000research.26932.2).
RESPONSE
=========
We have checked the references and updated the preprints that have been published during the review period. We also have cited the software according to the guidelines provided.

Reviewer reports:
Reviewer #2: My only remaining comment is regarding the installation option. It would be helpful if the authors could provide an alternative installation path other than docker. The authors write that one of the dependencies was not available for a complete conda installation - this is OK - the authors can still provide a route to install all other packages using conda and indicate that the remaining dependency needs to be installed manually.
RESPONSE
=========
We have added the option of conda installation in the way the referee suggested.

Close
