# Supplementary material for: Highly accurate whole-genome imputation of SARS-CoV-2 from partial or low-quality sequences
Source: Gigascience. 2021 Dec 2;10(12):giab078. doi: 10.1093/gigascience/giab078 (PMC8643610; doi:10.1093/gigascience/giab078)
Supplement: giab078_Reviewer_2_Report_Original_Submission — Stephen Nayfach -- 9/1/2021 Reviewed [file giab078_reviewer_2_report_original_submission.pdf]

Reviewer Report

Title: Highly accurate whole genome imputation of SARS-CoV-2 from partial or low-quality sequences

Version: Original Submission **Date:** 9/1/2021

Reviewer name: Stephen Nayfach

Reviewer Comments to Author:

In their manuscript, Ortuno et al. develop a procedure for imputing missing genotypes of SARS-CoV-2. Missing genotypes can arise from fragmented whole genome assemblies, targeted sequencing (e.g. spike protein), or incomplete genotype panels. I really like this idea and thought the paper was conducted quite carefully. I was impressed by the high level of precision across all experiments. I have a few minor comments, questions, and suggestions below:

Major comments:

My understanding is that only SNPs are imputed by the program. Is this correct? If this is the case, can the authors comment on the frequency of other types of variants in the SARS-CoV-2 genome? How common are small indels, large indels, or rearrangements?

Can the authors include code for building their reference panel? This would enable the same pipeline to be applied to updated SARS-CoV-2 references or to other kinds of viruses entirely. For example, metagenomic DNA sequencing often yields partial viral genomes, and it would be great to use this same pipeline to impute these genomes (where sufficient references exist).

I noticed that several of the PANGOLIN lineages seem especially hard to impute. Can the authors comment on why this might be the case? Regarding the PANGOLIN lineages, how to these correspond to specific variants of interest (e.g. delta variant)? Is this information provided to users? A visual could really help here showing the phylogenetic relationships between PANGOLIN lineages and how they relate to variants of interest.

The authors indicate that missing regions of partial genome assemblies must be indicated by Ns. This seems like an artificial constraint that may be a pain point for users. Can the authors modify their program to detect missing regions from FASTA files and automatically fill these regions with Ns prior to imputation?

Minor comments:

For the installation options, please provide an alternative to docker. Would it be feasible to add an installation option using conda?

In their methods, could the authors clearly define true positives, true negatives, false positives, and false negatives in the context of their validation experiments? Related to this point, I noticed that the precision is consistently high in the validation experiments, but recall can be quite low. I assume this means that the program will not impute a genotype where there is insufficient evidence, leaving it as a "N". In this case, users should have high confidence in all imputed genotypes. Is this correct?

All the figures in the manuscript were of low resolution and difficult to read.

The authors should use a consistent tense (present or past) throughout the manuscript. In some places future tense was even used: "Once we have validated the robustness of our imputation against different

missing regions scenarios, the validation will focus on the imputation of variants"

Level of Interest

Please indicate how interesting you found the manuscript: Choose an item.

Quality of Written English

Please indicate the quality of language in the manuscript: Choose an item.

Declaration of Competing Interests

Please complete a declaration of competing interests, considering the following questions:

- Have you in the past five years received reimbursements, fees, funding, or salary from an organisation that may in any way gain or lose financially from the publication of this manuscript, either now or in the future?
- Do you hold any stocks or shares in an organisation that may in any way gain or lose financially from the publication of this manuscript, either now or in the future?
- Do you hold or are you currently applying for any patents relating to the content of the manuscript?
- Have you received reimbursements, fees, funding, or salary from an organization that holds or has applied for patents relating to the content of the manuscript?
- Do you have any other financial competing interests?
- Do you have any non-financial competing interests in relation to this paper?

If you can answer no to all of the above, write 'I declare that I have no competing interests' below. If your reply is yes to any, please give details below.

I declare that I have no competing interests

I agree to the open peer review policy of the journal. I understand that my name will be included on my report to the authors and, if the manuscript is accepted for publication, my named report including any attachments I upload will be posted on the website along with the authors' responses. I agree for my report to be made available under an Open Access Creative Commons CC-BY license (http://creativecommons.org/licenses/by/4.0/). I understand that any comments which I do not wish to be included in my named report can be included as confidential comments to the editors, which will not be published.

Choose an item.

To further support our reviewers, we have joined with Publons, where you can gain additional credit to further highlight your hard work (see: https://publons.com/journal/530/gigascience). On publication of this paper, your review will be automatically added to Publons, you can then choose whether or not to claim your Publons credit. I understand this statement.

Yes Choose an item.
